# Characterization dataset for pre- and post-irradiated shrimp waste chitosan

**DOI:** 10.1016/j.dib.2020.106081

**Published:** 2020-07-25

**Authors:** Siddhartha Pati, Paramananda Jena, Salwa Shahimi, Bryan Raveen Nelson, Diptikanta Acharya, Bishnu Prasad Dash, Anil Chatterji

**Affiliations:** a Department of Biosciences & Biotechnology, Fakir Mohan University, Balasore-756089, Odisha, India; b Institute of Tropical Biodiversity and Sustainable Development, University Malaysia Terengganu, 21030 Kuala Nerus, Terengganu, Malaysia; c Research Division, Association for Biodiversity Conservation and Research (ABC), Devine Colony, 756001 Balasore, Odisha, India; d School of Marine and Environmental Sciences, Universiti Malaysia Terengganu, 21030 Kuala Nerus, Terengganu, Malaysia; e Department of Biotechnology, GIET University, Gunupur, Odisha,765022 India; f Centre of Excellence (CoE) for Bioresource Management and Energy Conservation Material Development, Fakir Mohan University, Balasore-756089, Odisha, India; g Aquamarina Research Foundation, Dona Paula, Goa-403004, India

**Keywords:** Industrial waste, Biopolymer, Radiation, Composition, Functional group, Spectroscopy

## Abstract

This dataset presents morphological features, elemental composition and functional groups of different pre- and post-gamma (γ)-irradiated chitosan (10kGy & 20kGy) prepared from shrimp waste. The γ-irradiated chitosan was characterized using Fourier transfer infrared (FTIR) spectroscopy and X-ray diffraction (XRD) analyses. Thermogravimetry/differential thermal analysis (TG/DTA) were performed using Perkin Elmer Pyris Diamond DSC with a heating rate of 10 °C/minute and dynamic synthetic atmospheric air set at flow rate of 100 ml/minute. We observed γ-irradiated chitosan to have shorter polymer size, small pores and compacted structure with active alkyl and hydroxyl groups when compared to non-irradiated chitosan. Our data provides baseline understanding for structure of shrimp chitosan after ^60^Co exposure which means, the biopolymer becomes more stable and is considered suitable for vast food industry applications.

**Specifications table**

**Table:** 

**Subject**	Materials Science
**Specific subject area**	Biomaterials
**Type of data**	Figure and Graph
**How data were acquired**	Experimental, Scanning Electron Microscopy (SEM) micrographs captured using (Carl ZEISS SMT, Germany), Thermogravimetric-differential thermal analysis Pyris Diamond TG/DTA, Perkin Elmer Instrument, USA), Fourier transfer infrared (FTIR) (Thermo Nicolet™ 6700; Thermo FisherScientificUSA), X-ray diffraction(XRD, PAN AlyticalX'pert Pro-powder diffractometer, UK), X-ray and Graphing data analysis (OriginPro, Version 2019b. OriginLab Co. USA)
**Data format**	Raw and analyzed
**Parameters for data collection**	Characterization of γ-irradiation shrimp waste chitosan through FTIR, XRD, TG/DTA and SEM to monitor the changes in structural, thermal and morphological performance.
**Description of data collection**	γ-irradiation (10 & 20 kGy) was administered to chitosan. FTIR characterizes chemical changes within chitosan whereas X-ray Diffraction (XRD) characterizes effects towards chitosan composition. Thermal gravimetric analysis (TGA) is used to evaluate the thermal stability of pre- and post- irradiated chitosan. Scanning electron microscopy enables observation of chitosan morphology.
**Data source location**	Balasore, Odisha, India
**Data accessibility**	With the article

**Value of the data**

• The data informs about high-performance biopolymers produced from crustacean waste.
• Biowaste data from this article assist manufacturing plants to develop stable biopolymers that are usable for various applications.
• Researchers can use surface features, elemental composition and functional group data to compare with their findings on pre- and post-irradiated chitosan.
• This data article benefits researchers by providing baseline information on chemical composition, stability and molecular weight changes of chitosan (biopolymers) afterγ-irradiation.

## 1 Data description

**Fig. 1:**
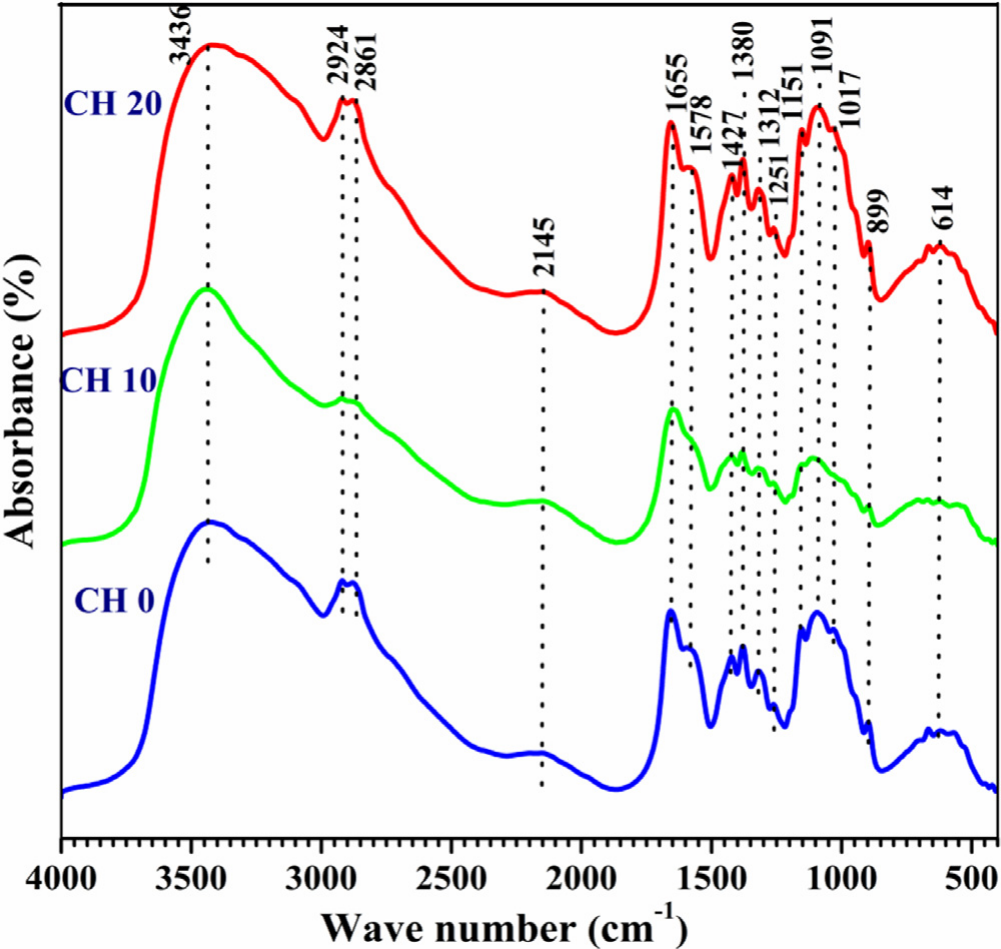
Spectra of non-irradiated chitosan (CH0), 10 kGy gamma-irradiated chitosan(CH10) and 20 kGy gamma-irradiated chitosan(CH20) in Fourier transfer infrared analysis.

**Fig. 2:**
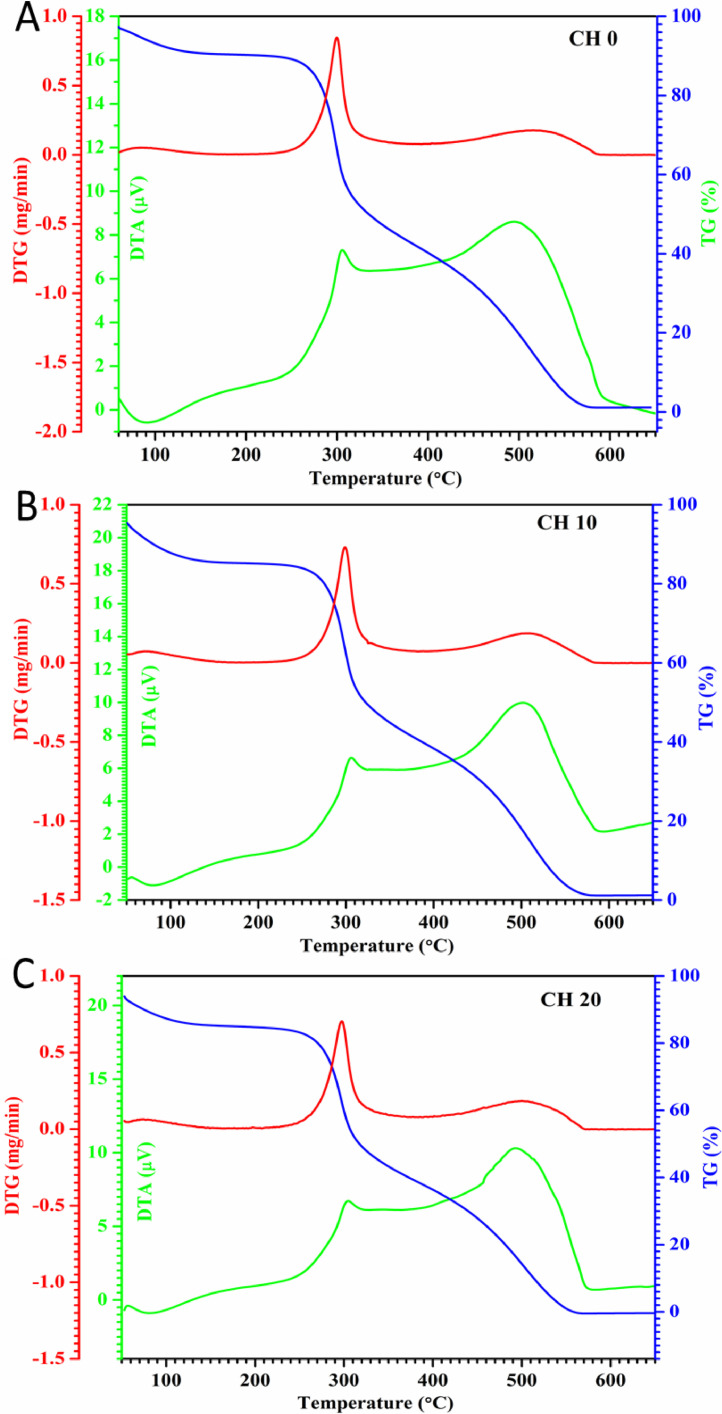
Thermogravimetric-differential thermal analysis results for non-irradiated chitosan (A, CH0), 10 kGy gamma-irradiated chitosan(B, CH10) and 20 kGy gamma-irradiated chitosan(C, CH20).

**Fig. 3:**
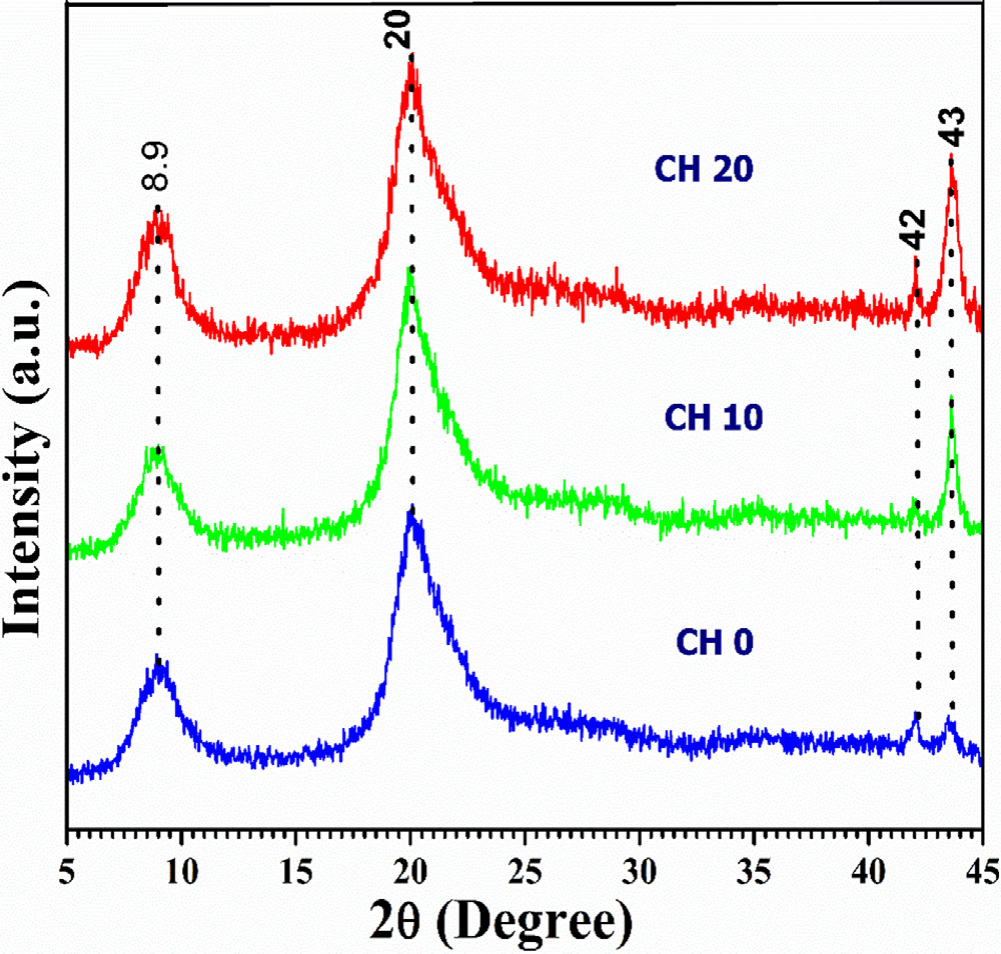
Results for non-irradiated chitosan (CH0), 10 kGy gamma-irradiated chitosan(CH10) and 20 kGy gamma-irradiated chitosan(CH20) after X-raydiffraction.

**Fig. 4:**
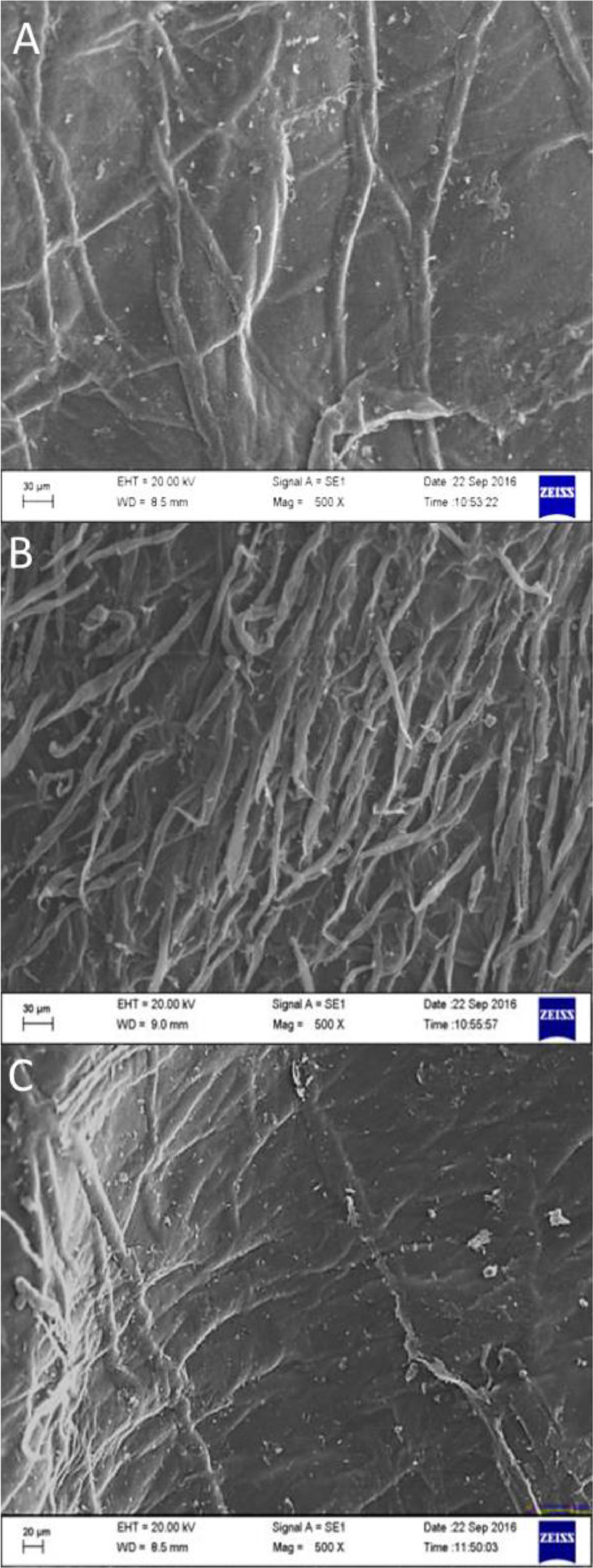
Micrographs of non-irradiated chitosan(A, CH0), 10 kGy gamma-irradiated chitosan(B, CH10) and 20 kGy gamma-irradiated chitosan(C, CH20) under 500X magnification using Scanning Electron Microscopy.

As long-chain polymeric saccharide, chitosan has some free groups, such as amino, alkyl and hydroxyl which may contain radicals. The provision γ-irradiation allows crosslinking between radicals to develop new functional groups. In fact, FTIR (Fig. 1) readings such as 3000∼3800 cm^−1^ (OH, NH2), 2850 cm^−1^ (-CH stretching), 1645 cm^−1^ (-CONH-, amide I), 1550 cm^−1^ (-NH, amide II), 1570 cm^−1^ (C—N stretching), 1300 cm^−1^ (amide III bond), and 1103 cm^−1^ (C—O stretching vibration) coincide with aforementioned functional groups. Comparatively, FTIR readings of 3070–3750 cm^−1^ for non-irradiated and γ-irradiated (10 & 20 kGy) chitosan (control group)coincide with OH and NH_2_ functional groups which means, the test chitosan is very pure. Separately, thermogravimetry analysis (Fig. 2) indicates that moisture evaporation causes samples to lose their weight at 100–150 °C. Being hydrophilic, chitosan will decompose their pyranose ring and b-glycosidic-linkages situated in-between glycosamine and N-acetyl glucosamine moieties. We also examined the effects of sterilization radiation to modify chitosan characteristics through X-ray diffraction (Fig. 3). Attaining intensities at 6.6°, 8.5°, 15.1°, 19.8°, 20.0°, 24.0° and 24.9 are indicators that γ-irradiated chitosan possesses similar polymeric characteristics with non-irradiated chitosan. In addition, scanning electron microscopy examination(Fig. 4) revealed that the morphology of γ-irradiated chitosan is slightly smooth, shorter and having decreased pore sizes than non-irradiated chitosan since it appears longer and having a variety of pore sizes.

## 2 Experimental design, materials and methods

Shrimp waste (shells) from food processing company in Bahabalpur, Balasore were washed with distilled water before crushed using mortar and pestle. Crushed samples were stored (duplicates) in polyethylene bags at ambient temperature (28±2 °C) for 24 h so that partial autolysis facilitates chemical extraction of chitosan. Shrimp chitosan was isolated using four processes namely, Demineralization, Deproteinization Decoloration and Deacetylation [1,2]. Samples were placed in vials, sealed under vacuum, and γ -irradiated at ambient temperature (fix dose rate 10 kGy/h; 10, and 20 kGy doses) using ^60^Co as the radiation source. Un-irradiated chitosan and chitosan irradiated at 10 kGy and 20 kGy will be referred to as CH0, CH10 and CHI20, respectively. All the chitosan samples were characterized for surface morphology along with Fourier transform infrared (FTIR) spectroscopy, spectroscopy, X-ray diffraction, and TGA studies.

FTIR spectra of all three chitosan samples were obtained by using the FTIR spectrometer (Thermo Nicolet™ 6700; Thermo Fisher Scientific, USA) at a resolution of 2 cm^−1^ and wavelengths from 4000 to 500 cm^−1^. For FTIR analysis the samples were previously ground and mixed thoroughly with potassium bromide, an infrared transparent matrix, at 1:100 (sample: KBr) ratio, respectively. Another batch comprising of γ-irradiated and non-irradiated samples were heated at 10  °C/min to 700  °C in oxygen rich air (21%) followed by thermogravimetric analysis (TGA) and derivative thermogravimetry (DTG) analysis using a thermogravimetric analyzer (Pyris Diamond TG-DTA, PerkinElmer Instrument, USA). Another batch of samples were exposed to CuKα radiation (λ = 1.5406 nm), 45 kV, 30 mA, division slit 0.25° with Co as anode and graphite monochromator. Before and after γ-irradiation, chitosan samples were analyzed using Panalytical Diffractometer (X'Pert Pro, USA) at 0.025° (2θ) angle and 52°–45° range with 1.25 s scan time for 1 hour. Lastly, all chitosan samples are fixed onto aluminum stubs using conductive tape before platinum coating using the sputter coater (POLARON-SC7620, Carbon Accessory, and Model-CA76). Micrograph comparisons are made between γ-irradiated and non-irradiated using Scanning Electron Microscopy (Carl ZEISS SMT, Germany) at 500X magnifications.

## Declaration of Competing Interest

The authors declare that they have no known competing financial interests or personal relationships which have, or could be perceived to have, influenced the work reported in this article.

## Appendix Supplementary materials

Image, application 1

Image, application 2
