# Supplementary material for: Characterization dataset for pre- and post-irradiated shrimp waste chitosan
Source: Data Brief. 2020 Jul 25;32:106081. doi: 10.1016/j.dib.2020.106081 (PMC7397402; doi:10.1016/j.dib.2020.106081)
Supplement: Supplementary file 1 [file mmc1.zip › FTIR-CH10.docx]

| 3.99E+02 | 0.00E+00 |
| --- | --- |
| 4.01E+02 | 1.32E-03 |
| 4.03E+02 | 3.03E-03 |
| 4.05E+02 | 3.87E-03 |
| 4.07E+02 | 5.42E-03 |
| 4.09E+02 | 5.82E-03 |
| 4.11E+02 | 5.17E-03 |
| 4.13E+02 | 4.54E-03 |
| 4.15E+02 | 4.27E-03 |
| 4.17E+02 | 3.64E-03 |
| 4.18E+02 | 3.14E-03 |
| 4.20E+02 | 2.96E-03 |
| 4.22E+02 | 3.02E-03 |
| 4.24E+02 | 3.61E-03 |
| 4.26E+02 | 4.38E-03 |
| 4.28E+02 | 5.24E-03 |
| 4.30E+02 | 5.84E-03 |
| 4.32E+02 | 6.28E-03 |
| 4.34E+02 | 6.79E-03 |
| 4.36E+02 | 7.00E-03 |
| 4.38E+02 | 7.10E-03 |
| 4.40E+02 | 7.02E-03 |
| 4.42E+02 | 6.88E-03 |
| 4.44E+02 | 6.69E-03 |
| 4.45E+02 | 6.55E-03 |
| 4.47E+02 | 6.64E-03 |
| 4.49E+02 | 6.95E-03 |
| 4.51E+02 | 7.27E-03 |
| 4.53E+02 | 7.53E-03 |
| 4.55E+02 | 7.88E-03 |
| 4.57E+02 | 8.37E-03 |
| 4.59E+02 | 8.81E-03 |
| 4.61E+02 | 9.17E-03 |
| 4.63E+02 | 9.67E-03 |
| 4.65E+02 | 1.03E-02 |
| 4.67E+02 | 1.10E-02 |
| 4.69E+02 | 1.19E-02 |
| 4.71E+02 | 1.30E-02 |
| 4.72E+02 | 1.42E-02 |
| 4.74E+02 | 1.55E-02 |
| 4.76E+02 | 1.70E-02 |
| 4.78E+02 | 1.83E-02 |
| 4.80E+02 | 1.94E-02 |
| 4.82E+02 | 2.02E-02 |
| 4.84E+02 | 2.08E-02 |
| 4.86E+02 | 2.13E-02 |
| 4.88E+02 | 2.18E-02 |
| 4.90E+02 | 2.23E-02 |
| 4.92E+02 | 2.31E-02 |
| 4.94E+02 | 2.37E-02 |
| 4.96E+02 | 2.43E-02 |
| 4.98E+02 | 2.52E-02 |
| 4.99E+02 | 2.63E-02 |
| 5.01E+02 | 2.76E-02 |
| 5.03E+02 | 2.89E-02 |
| 5.05E+02 | 3.01E-02 |
| 5.07E+02 | 3.11E-02 |
| 5.09E+02 | 3.23E-02 |
| 5.11E+02 | 3.36E-02 |
| 5.13E+02 | 3.50E-02 |
| 5.15E+02 | 3.62E-02 |
| 5.17E+02 | 3.74E-02 |
| 5.19E+02 | 3.87E-02 |
| 5.21E+02 | 3.97E-02 |
| 5.23E+02 | 4.06E-02 |
| 5.25E+02 | 4.14E-02 |
| 5.26E+02 | 4.19E-02 |
| 5.28E+02 | 4.23E-02 |
| 5.30E+02 | 4.25E-02 |
| 5.32E+02 | 4.27E-02 |
| 5.34E+02 | 4.26E-02 |
| 5.36E+02 | 4.27E-02 |
| 5.38E+02 | 4.27E-02 |
| 5.40E+02 | 4.27E-02 |
| 5.42E+02 | 4.29E-02 |
| 5.44E+02 | 4.33E-02 |
| 5.46E+02 | 4.37E-02 |
| 5.48E+02 | 4.41E-02 |
| 5.50E+02 | 4.42E-02 |
| 5.52E+02 | 4.44E-02 |
| 5.53E+02 | 4.44E-02 |
| 5.55E+02 | 4.44E-02 |
| 5.57E+02 | 4.45E-02 |
| 5.59E+02 | 4.44E-02 |
| 5.61E+02 | 4.44E-02 |
| 5.63E+02 | 4.43E-02 |
| 5.65E+02 | 4.41E-02 |
| 5.67E+02 | 4.40E-02 |
| 5.69E+02 | 4.39E-02 |
| 5.71E+02 | 4.38E-02 |
| 5.73E+02 | 4.36E-02 |
| 5.75E+02 | 4.34E-02 |
| 5.77E+02 | 4.31E-02 |
| 5.79E+02 | 4.30E-02 |
| 5.80E+02 | 4.29E-02 |
| 5.82E+02 | 4.28E-02 |
| 5.84E+02 | 4.28E-02 |
| 5.86E+02 | 4.28E-02 |
| 5.88E+02 | 4.28E-02 |
| 5.90E+02 | 4.29E-02 |
| 5.92E+02 | 4.32E-02 |
| 5.94E+02 | 4.35E-02 |
| 5.96E+02 | 4.39E-02 |
| 5.98E+02 | 4.44E-02 |
| 6.00E+02 | 4.47E-02 |
| 6.02E+02 | 4.51E-02 |
| 6.04E+02 | 4.55E-02 |
| 6.06E+02 | 4.59E-02 |
| 6.07E+02 | 4.63E-02 |
| 6.09E+02 | 4.67E-02 |
| 6.11E+02 | 4.70E-02 |
| 6.13E+02 | 4.73E-02 |
| 6.15E+02 | 4.75E-02 |
| 6.17E+02 | 4.77E-02 |
| 6.19E+02 | 4.79E-02 |
| 6.21E+02 | 4.81E-02 |
| 6.23E+02 | 4.82E-02 |
| 6.25E+02 | 4.83E-02 |
| 6.27E+02 | 4.82E-02 |
| 6.29E+02 | 4.80E-02 |
| 6.31E+02 | 4.78E-02 |
| 6.33E+02 | 4.76E-02 |
| 6.34E+02 | 4.74E-02 |
| 6.36E+02 | 4.72E-02 |
| 6.38E+02 | 4.70E-02 |
| 6.40E+02 | 4.67E-02 |
| 6.42E+02 | 4.64E-02 |
| 6.44E+02 | 4.63E-02 |
| 6.46E+02 | 4.61E-02 |
| 6.48E+02 | 4.61E-02 |
| 6.50E+02 | 4.61E-02 |
| 6.52E+02 | 4.64E-02 |
| 6.54E+02 | 4.68E-02 |
| 6.56E+02 | 4.72E-02 |
| 6.58E+02 | 4.76E-02 |
| 6.60E+02 | 4.81E-02 |
| 6.61E+02 | 4.86E-02 |
| 6.63E+02 | 4.91E-02 |
| 6.65E+02 | 4.95E-02 |
| 6.67E+02 | 4.98E-02 |
| 6.69E+02 | 4.98E-02 |
| 6.71E+02 | 4.96E-02 |
| 6.73E+02 | 4.94E-02 |
| 6.75E+02 | 4.91E-02 |
| 6.77E+02 | 4.88E-02 |
| 6.79E+02 | 4.85E-02 |
| 6.81E+02 | 4.82E-02 |
| 6.83E+02 | 4.79E-02 |
| 6.85E+02 | 4.76E-02 |
| 6.87E+02 | 4.75E-02 |
| 6.88E+02 | 4.76E-02 |
| 6.90E+02 | 4.78E-02 |
| 6.92E+02 | 4.80E-02 |
| 6.94E+02 | 4.81E-02 |
| 6.96E+02 | 4.83E-02 |
| 6.98E+02 | 4.86E-02 |
| 7.00E+02 | 4.90E-02 |
| 7.02E+02 | 4.93E-02 |
| 7.04E+02 | 4.95E-02 |
| 7.06E+02 | 4.95E-02 |
| 7.08E+02 | 4.94E-02 |
| 7.10E+02 | 4.93E-02 |
| 7.12E+02 | 4.92E-02 |
| 7.14E+02 | 4.89E-02 |
| 7.15E+02 | 4.86E-02 |
| 7.17E+02 | 4.83E-02 |
| 7.19E+02 | 4.79E-02 |
| 7.21E+02 | 4.76E-02 |
| 7.23E+02 | 4.73E-02 |
| 7.25E+02 | 4.69E-02 |
| 7.27E+02 | 4.66E-02 |
| 7.29E+02 | 4.63E-02 |
| 7.31E+02 | 4.60E-02 |
| 7.33E+02 | 4.58E-02 |
| 7.35E+02 | 4.55E-02 |
| 7.37E+02 | 4.54E-02 |
| 7.39E+02 | 4.51E-02 |
| 7.41E+02 | 4.48E-02 |
| 7.42E+02 | 4.45E-02 |
| 7.44E+02 | 4.42E-02 |
| 7.46E+02 | 4.41E-02 |
| 7.48E+02 | 4.38E-02 |
| 7.50E+02 | 4.35E-02 |
| 7.52E+02 | 4.31E-02 |
| 7.54E+02 | 4.26E-02 |
| 7.56E+02 | 4.22E-02 |
| 7.58E+02 | 4.18E-02 |
| 7.60E+02 | 4.14E-02 |
| 7.62E+02 | 4.10E-02 |
| 7.64E+02 | 4.06E-02 |
| 7.66E+02 | 4.02E-02 |
| 7.68E+02 | 3.98E-02 |
| 7.69E+02 | 3.94E-02 |
| 7.71E+02 | 3.90E-02 |
| 7.73E+02 | 3.86E-02 |
| 7.75E+02 | 3.84E-02 |
| 7.77E+02 | 3.80E-02 |
| 7.79E+02 | 3.76E-02 |
| 7.81E+02 | 3.72E-02 |
| 7.83E+02 | 3.67E-02 |
| 7.85E+02 | 3.62E-02 |
| 7.87E+02 | 3.57E-02 |
| 7.89E+02 | 3.53E-02 |
| 7.91E+02 | 3.48E-02 |
| 7.93E+02 | 3.43E-02 |
| 7.95E+02 | 3.37E-02 |
| 7.96E+02 | 3.32E-02 |
| 7.98E+02 | 3.26E-02 |
| 8.00E+02 | 3.21E-02 |
| 8.02E+02 | 3.16E-02 |
| 8.04E+02 | 3.12E-02 |
| 8.06E+02 | 3.09E-02 |
| 8.08E+02 | 3.05E-02 |
| 8.10E+02 | 3.02E-02 |
| 8.12E+02 | 2.98E-02 |
| 8.14E+02 | 2.95E-02 |
| 8.16E+02 | 2.92E-02 |
| 8.18E+02 | 2.88E-02 |
| 8.20E+02 | 2.84E-02 |
| 8.22E+02 | 2.79E-02 |
| 8.23E+02 | 2.75E-02 |
| 8.25E+02 | 2.70E-02 |
| 8.27E+02 | 2.64E-02 |
| 8.29E+02 | 2.60E-02 |
| 8.31E+02 | 2.57E-02 |
| 8.33E+02 | 2.53E-02 |
| 8.35E+02 | 2.49E-02 |
| 8.37E+02 | 2.46E-02 |
| 8.39E+02 | 2.43E-02 |
| 8.41E+02 | 2.39E-02 |
| 8.43E+02 | 2.36E-02 |
| 8.45E+02 | 2.33E-02 |
| 8.47E+02 | 2.30E-02 |
| 8.49E+02 | 2.28E-02 |
| 8.50E+02 | 2.26E-02 |
| 8.52E+02 | 2.24E-02 |
| 8.54E+02 | 2.23E-02 |
| 8.56E+02 | 2.22E-02 |
| 8.58E+02 | 2.21E-02 |
| 8.60E+02 | 2.20E-02 |
| 8.62E+02 | 2.21E-02 |
| 8.64E+02 | 2.22E-02 |
| 8.66E+02 | 2.24E-02 |
| 8.68E+02 | 2.27E-02 |
| 8.70E+02 | 2.31E-02 |
| 8.72E+02 | 2.36E-02 |
| 8.74E+02 | 2.45E-02 |
| 8.76E+02 | 2.55E-02 |
| 8.77E+02 | 2.68E-02 |
| 8.79E+02 | 2.83E-02 |
| 8.81E+02 | 3.00E-02 |
| 8.83E+02 | 3.19E-02 |
| 8.85E+02 | 3.39E-02 |
| 8.87E+02 | 3.59E-02 |
| 8.89E+02 | 3.79E-02 |
| 8.91E+02 | 3.96E-02 |
| 8.93E+02 | 4.09E-02 |
| 8.95E+02 | 4.19E-02 |
| 8.97E+02 | 4.24E-02 |
| 8.99E+02 | 4.25E-02 |
| 9.01E+02 | 4.22E-02 |
| 9.03E+02 | 4.16E-02 |
| 9.04E+02 | 4.07E-02 |
| 9.06E+02 | 3.98E-02 |
| 9.08E+02 | 3.88E-02 |
| 9.10E+02 | 3.80E-02 |
| 9.12E+02 | 3.74E-02 |
| 9.14E+02 | 3.72E-02 |
| 9.16E+02 | 3.72E-02 |
| 9.18E+02 | 3.76E-02 |
| 9.20E+02 | 3.84E-02 |
| 9.22E+02 | 3.93E-02 |
| 9.24E+02 | 4.05E-02 |
| 9.26E+02 | 4.18E-02 |
| 9.28E+02 | 4.32E-02 |
| 9.30E+02 | 4.46E-02 |
| 9.31E+02 | 4.60E-02 |
| 9.33E+02 | 4.73E-02 |
| 9.35E+02 | 4.86E-02 |
| 9.37E+02 | 4.99E-02 |
| 9.39E+02 | 5.11E-02 |
| 9.41E+02 | 5.21E-02 |
| 9.43E+02 | 5.29E-02 |
| 9.45E+02 | 5.37E-02 |
| 9.47E+02 | 5.43E-02 |
| 9.49E+02 | 5.47E-02 |
| 9.51E+02 | 5.50E-02 |
| 9.53E+02 | 5.52E-02 |
| 9.55E+02 | 5.54E-02 |
| 9.57E+02 | 5.57E-02 |
| 9.58E+02 | 5.59E-02 |
| 9.60E+02 | 5.63E-02 |
| 9.62E+02 | 5.68E-02 |
| 9.64E+02 | 5.74E-02 |
| 9.66E+02 | 5.81E-02 |
| 9.68E+02 | 5.89E-02 |
| 9.70E+02 | 5.99E-02 |
| 9.72E+02 | 6.09E-02 |
| 9.74E+02 | 6.20E-02 |
| 9.76E+02 | 6.31E-02 |
| 9.78E+02 | 6.42E-02 |
| 9.80E+02 | 6.53E-02 |
| 9.82E+02 | 6.63E-02 |
| 9.84E+02 | 6.72E-02 |
| 9.85E+02 | 6.80E-02 |
| 9.87E+02 | 6.87E-02 |
| 9.89E+02 | 6.92E-02 |
| 9.91E+02 | 6.97E-02 |
| 9.93E+02 | 6.99E-02 |
| 9.95E+02 | 7.01E-02 |
| 9.97E+02 | 7.03E-02 |
| 9.99E+02 | 7.04E-02 |
| 1.00E+03 | 7.06E-02 |
| 1.00E+03 | 7.08E-02 |
| 1.00E+03 | 7.11E-02 |
| 1.01E+03 | 7.14E-02 |
| 1.01E+03 | 7.17E-02 |
| 1.01E+03 | 7.21E-02 |
| 1.01E+03 | 7.25E-02 |
| 1.01E+03 | 7.29E-02 |
| 1.02E+03 | 7.33E-02 |
| 1.02E+03 | 7.37E-02 |
| 1.02E+03 | 7.42E-02 |
| 1.02E+03 | 7.47E-02 |
| 1.02E+03 | 7.52E-02 |
| 1.03E+03 | 7.56E-02 |
| 1.03E+03 | 7.60E-02 |
| 1.03E+03 | 7.64E-02 |
| 1.03E+03 | 7.67E-02 |
| 1.03E+03 | 7.70E-02 |
| 1.04E+03 | 7.73E-02 |
| 1.04E+03 | 7.76E-02 |
| 1.04E+03 | 7.78E-02 |
| 1.04E+03 | 7.81E-02 |
| 1.04E+03 | 7.84E-02 |
| 1.05E+03 | 7.88E-02 |
| 1.05E+03 | 7.93E-02 |
| 1.05E+03 | 7.99E-02 |
| 1.05E+03 | 8.05E-02 |
| 1.05E+03 | 8.12E-02 |
| 1.05E+03 | 8.19E-02 |
| 1.06E+03 | 8.27E-02 |
| 1.06E+03 | 8.35E-02 |
| 1.06E+03 | 8.43E-02 |
| 1.06E+03 | 8.50E-02 |
| 1.06E+03 | 8.58E-02 |
| 1.07E+03 | 8.66E-02 |
| 1.07E+03 | 8.73E-02 |
| 1.07E+03 | 8.80E-02 |
| 1.07E+03 | 8.87E-02 |
| 1.07E+03 | 8.93E-02 |
| 1.08E+03 | 8.99E-02 |
| 1.08E+03 | 9.04E-02 |
| 1.08E+03 | 9.10E-02 |
| 1.08E+03 | 9.15E-02 |
| 1.08E+03 | 9.19E-02 |
| 1.09E+03 | 9.23E-02 |
| 1.09E+03 | 9.26E-02 |
| 1.09E+03 | 9.29E-02 |
| 1.09E+03 | 9.33E-02 |
| 1.09E+03 | 9.36E-02 |
| 1.10E+03 | 9.39E-02 |
| 1.10E+03 | 9.41E-02 |
| 1.10E+03 | 9.43E-02 |
| 1.10E+03 | 9.45E-02 |
| 1.10E+03 | 9.47E-02 |
| 1.11E+03 | 9.49E-02 |
| 1.11E+03 | 9.50E-02 |
| 1.11E+03 | 9.51E-02 |
| 1.11E+03 | 9.52E-02 |
| 1.11E+03 | 9.51E-02 |
| 1.11E+03 | 9.51E-02 |
| 1.12E+03 | 9.49E-02 |
| 1.12E+03 | 9.47E-02 |
| 1.12E+03 | 9.43E-02 |
| 1.12E+03 | 9.38E-02 |
| 1.12E+03 | 9.31E-02 |
| 1.13E+03 | 9.23E-02 |
| 1.13E+03 | 9.14E-02 |
| 1.13E+03 | 9.06E-02 |
| 1.13E+03 | 8.97E-02 |
| 1.13E+03 | 8.90E-02 |
| 1.14E+03 | 8.83E-02 |
| 1.14E+03 | 8.79E-02 |
| 1.14E+03 | 8.78E-02 |
| 1.14E+03 | 8.78E-02 |
| 1.14E+03 | 8.79E-02 |
| 1.15E+03 | 8.81E-02 |
| 1.15E+03 | 8.82E-02 |
| 1.15E+03 | 8.83E-02 |
| 1.15E+03 | 8.83E-02 |
| 1.15E+03 | 8.81E-02 |
| 1.16E+03 | 8.77E-02 |
| 1.16E+03 | 8.71E-02 |
| 1.16E+03 | 8.63E-02 |
| 1.16E+03 | 8.51E-02 |
| 1.16E+03 | 8.37E-02 |
| 1.16E+03 | 8.20E-02 |
| 1.17E+03 | 8.00E-02 |
| 1.17E+03 | 7.78E-02 |
| 1.17E+03 | 7.53E-02 |
| 1.17E+03 | 7.26E-02 |
| 1.17E+03 | 6.97E-02 |
| 1.18E+03 | 6.69E-02 |
| 1.18E+03 | 6.40E-02 |
| 1.18E+03 | 6.14E-02 |
| 1.18E+03 | 5.90E-02 |
| 1.18E+03 | 5.71E-02 |
| 1.19E+03 | 5.55E-02 |
| 1.19E+03 | 5.44E-02 |
| 1.19E+03 | 5.37E-02 |
| 1.19E+03 | 5.35E-02 |
| 1.19E+03 | 5.36E-02 |
| 1.20E+03 | 5.38E-02 |
| 1.20E+03 | 5.39E-02 |
| 1.20E+03 | 5.38E-02 |
| 1.20E+03 | 5.33E-02 |
| 1.20E+03 | 5.26E-02 |
| 1.21E+03 | 5.16E-02 |
| 1.21E+03 | 5.05E-02 |
| 1.21E+03 | 4.94E-02 |
| 1.21E+03 | 4.84E-02 |
| 1.21E+03 | 4.76E-02 |
| 1.21E+03 | 4.72E-02 |
| 1.22E+03 | 4.71E-02 |
| 1.22E+03 | 4.74E-02 |
| 1.22E+03 | 4.80E-02 |
| 1.22E+03 | 4.87E-02 |
| 1.22E+03 | 4.96E-02 |
| 1.23E+03 | 5.05E-02 |
| 1.23E+03 | 5.14E-02 |
| 1.23E+03 | 5.23E-02 |
| 1.23E+03 | 5.31E-02 |
| 1.23E+03 | 5.40E-02 |
| 1.24E+03 | 5.50E-02 |
| 1.24E+03 | 5.60E-02 |
| 1.24E+03 | 5.70E-02 |
| 1.24E+03 | 5.81E-02 |
| 1.24E+03 | 5.92E-02 |
| 1.25E+03 | 6.04E-02 |
| 1.25E+03 | 6.17E-02 |
| 1.25E+03 | 6.29E-02 |
| 1.25E+03 | 6.41E-02 |
| 1.25E+03 | 6.53E-02 |
| 1.26E+03 | 6.62E-02 |
| 1.26E+03 | 6.71E-02 |
| 1.26E+03 | 6.77E-02 |
| 1.26E+03 | 6.81E-02 |
| 1.26E+03 | 6.83E-02 |
| 1.27E+03 | 6.81E-02 |
| 1.27E+03 | 6.78E-02 |
| 1.27E+03 | 6.73E-02 |
| 1.27E+03 | 6.68E-02 |
| 1.27E+03 | 6.64E-02 |
| 1.27E+03 | 6.62E-02 |
| 1.28E+03 | 6.61E-02 |
| 1.28E+03 | 6.64E-02 |
| 1.28E+03 | 6.71E-02 |
| 1.28E+03 | 6.80E-02 |
| 1.28E+03 | 6.93E-02 |
| 1.29E+03 | 7.08E-02 |
| 1.29E+03 | 7.25E-02 |
| 1.29E+03 | 7.41E-02 |
| 1.29E+03 | 7.58E-02 |
| 1.29E+03 | 7.74E-02 |
| 1.30E+03 | 7.88E-02 |
| 1.30E+03 | 8.00E-02 |
| 1.30E+03 | 8.10E-02 |
| 1.30E+03 | 8.17E-02 |
| 1.30E+03 | 8.22E-02 |
| 1.31E+03 | 8.26E-02 |
| 1.31E+03 | 8.29E-02 |
| 1.31E+03 | 8.31E-02 |
| 1.31E+03 | 8.33E-02 |
| 1.31E+03 | 8.36E-02 |
| 1.32E+03 | 8.38E-02 |
| 1.32E+03 | 8.41E-02 |
| 1.32E+03 | 8.42E-02 |
| 1.32E+03 | 8.42E-02 |
| 1.32E+03 | 8.42E-02 |
| 1.32E+03 | 8.40E-02 |
| 1.33E+03 | 8.35E-02 |
| 1.33E+03 | 8.27E-02 |
| 1.33E+03 | 8.19E-02 |
| 1.33E+03 | 8.10E-02 |
| 1.33E+03 | 8.01E-02 |
| 1.34E+03 | 7.94E-02 |
| 1.34E+03 | 7.88E-02 |
| 1.34E+03 | 7.85E-02 |
| 1.34E+03 | 7.82E-02 |
| 1.34E+03 | 7.81E-02 |
| 1.35E+03 | 7.83E-02 |
| 1.35E+03 | 7.87E-02 |
| 1.35E+03 | 7.91E-02 |
| 1.35E+03 | 7.94E-02 |
| 1.35E+03 | 7.98E-02 |
| 1.36E+03 | 8.03E-02 |
| 1.36E+03 | 8.12E-02 |
| 1.36E+03 | 8.25E-02 |
| 1.36E+03 | 8.38E-02 |
| 1.36E+03 | 8.53E-02 |
| 1.37E+03 | 8.69E-02 |
| 1.37E+03 | 8.86E-02 |
| 1.37E+03 | 9.03E-02 |
| 1.37E+03 | 9.22E-02 |
| 1.37E+03 | 9.46E-02 |
| 1.37E+03 | 9.69E-02 |
| 1.38E+03 | 9.87E-02 |
| 1.38E+03 | 1.00E-01 |
| 1.38E+03 | 1.01E-01 |
| 1.38E+03 | 1.01E-01 |
| 1.38E+03 | 1.01E-01 |
| 1.39E+03 | 1.00E-01 |
| 1.39E+03 | 9.82E-02 |
| 1.39E+03 | 9.62E-02 |
| 1.39E+03 | 9.40E-02 |
| 1.39E+03 | 9.16E-02 |
| 1.40E+03 | 8.97E-02 |
| 1.40E+03 | 8.85E-02 |
| 1.40E+03 | 8.81E-02 |
| 1.40E+03 | 8.83E-02 |
| 1.40E+03 | 8.89E-02 |
| 1.41E+03 | 8.95E-02 |
| 1.41E+03 | 9.05E-02 |
| 1.41E+03 | 9.18E-02 |
| 1.41E+03 | 9.30E-02 |
| 1.41E+03 | 9.40E-02 |
| 1.42E+03 | 9.51E-02 |
| 1.42E+03 | 9.60E-02 |
| 1.42E+03 | 9.64E-02 |
| 1.42E+03 | 9.66E-02 |
| 1.42E+03 | 9.66E-02 |
| 1.43E+03 | 9.64E-02 |
| 1.43E+03 | 9.62E-02 |
| 1.43E+03 | 9.59E-02 |
| 1.43E+03 | 9.54E-02 |
| 1.43E+03 | 9.47E-02 |
| 1.43E+03 | 9.43E-02 |
| 1.44E+03 | 9.38E-02 |
| 1.44E+03 | 9.32E-02 |
| 1.44E+03 | 9.25E-02 |
| 1.44E+03 | 9.18E-02 |
| 1.44E+03 | 9.09E-02 |
| 1.45E+03 | 9.01E-02 |
| 1.45E+03 | 8.93E-02 |
| 1.45E+03 | 8.85E-02 |
| 1.45E+03 | 8.79E-02 |
| 1.45E+03 | 8.74E-02 |
| 1.46E+03 | 8.68E-02 |
| 1.46E+03 | 8.64E-02 |
| 1.46E+03 | 8.56E-02 |
| 1.46E+03 | 8.48E-02 |
| 1.46E+03 | 8.38E-02 |
| 1.47E+03 | 8.27E-02 |
| 1.47E+03 | 8.13E-02 |
| 1.47E+03 | 7.97E-02 |
| 1.47E+03 | 7.81E-02 |
| 1.47E+03 | 7.67E-02 |
| 1.48E+03 | 7.51E-02 |
| 1.48E+03 | 7.33E-02 |
| 1.48E+03 | 7.14E-02 |
| 1.48E+03 | 6.97E-02 |
| 1.48E+03 | 6.83E-02 |
| 1.48E+03 | 6.72E-02 |
| 1.49E+03 | 6.60E-02 |
| 1.49E+03 | 6.52E-02 |
| 1.49E+03 | 6.43E-02 |
| 1.49E+03 | 6.40E-02 |
| 1.49E+03 | 6.32E-02 |
| 1.50E+03 | 6.28E-02 |
| 1.50E+03 | 6.24E-02 |
| 1.50E+03 | 6.23E-02 |
| 1.50E+03 | 6.22E-02 |
| 1.50E+03 | 6.17E-02 |
| 1.51E+03 | 6.14E-02 |
| 1.51E+03 | 6.14E-02 |
| 1.51E+03 | 6.17E-02 |
| 1.51E+03 | 6.26E-02 |
| 1.51E+03 | 6.36E-02 |
| 1.52E+03 | 6.50E-02 |
| 1.52E+03 | 6.67E-02 |
| 1.52E+03 | 6.89E-02 |
| 1.52E+03 | 7.04E-02 |
| 1.52E+03 | 7.25E-02 |
| 1.53E+03 | 7.48E-02 |
| 1.53E+03 | 7.69E-02 |
| 1.53E+03 | 7.92E-02 |
| 1.53E+03 | 8.12E-02 |
| 1.53E+03 | 8.32E-02 |
| 1.54E+03 | 8.54E-02 |
| 1.54E+03 | 8.76E-02 |
| 1.54E+03 | 8.96E-02 |
| 1.54E+03 | 9.15E-02 |
| 1.54E+03 | 9.34E-02 |
| 1.54E+03 | 9.55E-02 |
| 1.55E+03 | 9.63E-02 |
| 1.55E+03 | 9.77E-02 |
| 1.55E+03 | 9.96E-02 |
| 1.55E+03 | 1.01E-01 |
| 1.55E+03 | 1.03E-01 |
| 1.56E+03 | 1.04E-01 |
| 1.56E+03 | 1.05E-01 |
| 1.56E+03 | 1.06E-01 |
| 1.56E+03 | 1.07E-01 |
| 1.56E+03 | 1.08E-01 |
| 1.57E+03 | 1.09E-01 |
| 1.57E+03 | 1.10E-01 |
| 1.57E+03 | 1.11E-01 |
| 1.57E+03 | 1.12E-01 |
| 1.57E+03 | 1.12E-01 |
| 1.58E+03 | 1.13E-01 |
| 1.58E+03 | 1.15E-01 |
| 1.58E+03 | 1.15E-01 |
| 1.58E+03 | 1.16E-01 |
| 1.58E+03 | 1.16E-01 |
| 1.59E+03 | 1.17E-01 |
| 1.59E+03 | 1.17E-01 |
| 1.59E+03 | 1.18E-01 |
| 1.59E+03 | 1.18E-01 |
| 1.59E+03 | 1.19E-01 |
| 1.59E+03 | 1.20E-01 |
| 1.60E+03 | 1.20E-01 |
| 1.60E+03 | 1.21E-01 |
| 1.60E+03 | 1.22E-01 |
| 1.60E+03 | 1.22E-01 |
| 1.60E+03 | 1.23E-01 |
| 1.61E+03 | 1.24E-01 |
| 1.61E+03 | 1.26E-01 |
| 1.61E+03 | 1.27E-01 |
| 1.61E+03 | 1.29E-01 |
| 1.61E+03 | 1.30E-01 |
| 1.62E+03 | 1.32E-01 |
| 1.62E+03 | 1.34E-01 |
| 1.62E+03 | 1.36E-01 |
| 1.62E+03 | 1.38E-01 |
| 1.62E+03 | 1.39E-01 |
| 1.63E+03 | 1.41E-01 |
| 1.63E+03 | 1.42E-01 |
| 1.63E+03 | 1.43E-01 |
| 1.63E+03 | 1.45E-01 |
| 1.63E+03 | 1.46E-01 |
| 1.64E+03 | 1.47E-01 |
| 1.64E+03 | 1.47E-01 |
| 1.64E+03 | 1.47E-01 |
| 1.64E+03 | 1.47E-01 |
| 1.64E+03 | 1.47E-01 |
| 1.64E+03 | 1.48E-01 |
| 1.65E+03 | 1.48E-01 |
| 1.65E+03 | 1.48E-01 |
| 1.65E+03 | 1.48E-01 |
| 1.65E+03 | 1.47E-01 |
| 1.65E+03 | 1.47E-01 |
| 1.66E+03 | 1.46E-01 |
| 1.66E+03 | 1.45E-01 |
| 1.66E+03 | 1.44E-01 |
| 1.66E+03 | 1.42E-01 |
| 1.66E+03 | 1.40E-01 |
| 1.67E+03 | 1.38E-01 |
| 1.67E+03 | 1.36E-01 |
| 1.67E+03 | 1.34E-01 |
| 1.67E+03 | 1.32E-01 |
| 1.67E+03 | 1.30E-01 |
| 1.68E+03 | 1.27E-01 |
| 1.68E+03 | 1.24E-01 |
| 1.68E+03 | 1.21E-01 |
| 1.68E+03 | 1.17E-01 |
| 1.68E+03 | 1.13E-01 |
| 1.69E+03 | 1.09E-01 |
| 1.69E+03 | 1.05E-01 |
| 1.69E+03 | 1.02E-01 |
| 1.69E+03 | 9.80E-02 |
| 1.69E+03 | 9.42E-02 |
| 1.70E+03 | 9.01E-02 |
| 1.70E+03 | 8.61E-02 |
| 1.70E+03 | 8.22E-02 |
| 1.70E+03 | 7.86E-02 |
| 1.70E+03 | 7.48E-02 |
| 1.70E+03 | 7.10E-02 |
| 1.71E+03 | 6.76E-02 |
| 1.71E+03 | 6.44E-02 |
| 1.71E+03 | 6.16E-02 |
| 1.71E+03 | 5.88E-02 |
| 1.71E+03 | 5.66E-02 |
| 1.72E+03 | 5.46E-02 |
| 1.72E+03 | 5.23E-02 |
| 1.72E+03 | 4.99E-02 |
| 1.72E+03 | 4.77E-02 |
| 1.72E+03 | 4.59E-02 |
| 1.73E+03 | 4.41E-02 |
| 1.73E+03 | 4.24E-02 |
| 1.73E+03 | 4.06E-02 |
| 1.73E+03 | 3.90E-02 |
| 1.73E+03 | 3.74E-02 |
| 1.74E+03 | 3.57E-02 |
| 1.74E+03 | 3.39E-02 |
| 1.74E+03 | 3.21E-02 |
| 1.74E+03 | 3.06E-02 |
| 1.74E+03 | 2.92E-02 |
| 1.75E+03 | 2.76E-02 |
| 1.75E+03 | 2.63E-02 |
| 1.75E+03 | 2.53E-02 |
| 1.75E+03 | 2.45E-02 |
| 1.75E+03 | 2.37E-02 |
| 1.75E+03 | 2.30E-02 |
| 1.76E+03 | 2.23E-02 |
| 1.76E+03 | 2.15E-02 |
| 1.76E+03 | 2.07E-02 |
| 1.76E+03 | 1.97E-02 |
| 1.76E+03 | 1.87E-02 |
| 1.77E+03 | 1.79E-02 |
| 1.77E+03 | 1.72E-02 |
| 1.77E+03 | 1.64E-02 |
| 1.77E+03 | 1.56E-02 |
| 1.77E+03 | 1.49E-02 |
| 1.78E+03 | 1.43E-02 |
| 1.78E+03 | 1.37E-02 |
| 1.78E+03 | 1.32E-02 |
| 1.78E+03 | 1.27E-02 |
| 1.78E+03 | 1.21E-02 |
| 1.79E+03 | 1.16E-02 |
| 1.79E+03 | 1.11E-02 |
| 1.79E+03 | 1.05E-02 |
| 1.79E+03 | 9.98E-03 |
| 1.79E+03 | 9.50E-03 |
| 1.80E+03 | 9.01E-03 |
| 1.80E+03 | 8.56E-03 |
| 1.80E+03 | 8.13E-03 |
| 1.80E+03 | 7.77E-03 |
| 1.80E+03 | 7.53E-03 |
| 1.81E+03 | 7.29E-03 |
| 1.81E+03 | 7.04E-03 |
| 1.81E+03 | 6.85E-03 |
| 1.81E+03 | 6.68E-03 |
| 1.81E+03 | 6.52E-03 |
| 1.81E+03 | 6.35E-03 |
| 1.82E+03 | 6.06E-03 |
| 1.82E+03 | 5.72E-03 |
| 1.82E+03 | 5.43E-03 |
| 1.82E+03 | 5.19E-03 |
| 1.82E+03 | 4.91E-03 |
| 1.83E+03 | 4.61E-03 |
| 1.83E+03 | 4.40E-03 |
| 1.83E+03 | 4.26E-03 |
| 1.83E+03 | 4.15E-03 |
| 1.83E+03 | 4.09E-03 |
| 1.84E+03 | 4.04E-03 |
| 1.84E+03 | 4.03E-03 |
| 1.84E+03 | 4.00E-03 |
| 1.84E+03 | 3.98E-03 |
| 1.84E+03 | 3.88E-03 |
| 1.85E+03 | 3.77E-03 |
| 1.85E+03 | 3.72E-03 |
| 1.85E+03 | 3.72E-03 |
| 1.85E+03 | 3.75E-03 |
| 1.85E+03 | 3.83E-03 |
| 1.86E+03 | 3.81E-03 |
| 1.86E+03 | 3.64E-03 |
| 1.86E+03 | 3.43E-03 |
| 1.86E+03 | 3.26E-03 |
| 1.86E+03 | 3.12E-03 |
| 1.86E+03 | 3.08E-03 |
| 1.87E+03 | 3.09E-03 |
| 1.87E+03 | 3.17E-03 |
| 1.87E+03 | 3.32E-03 |
| 1.87E+03 | 3.50E-03 |
| 1.87E+03 | 3.75E-03 |
| 1.88E+03 | 4.03E-03 |
| 1.88E+03 | 4.26E-03 |
| 1.88E+03 | 4.48E-03 |
| 1.88E+03 | 4.51E-03 |
| 1.88E+03 | 4.44E-03 |
| 1.89E+03 | 4.45E-03 |
| 1.89E+03 | 4.56E-03 |
| 1.89E+03 | 4.70E-03 |
| 1.89E+03 | 4.90E-03 |
| 1.89E+03 | 5.13E-03 |
| 1.90E+03 | 5.38E-03 |
| 1.90E+03 | 5.60E-03 |
| 1.90E+03 | 5.84E-03 |
| 1.90E+03 | 6.11E-03 |
| 1.90E+03 | 6.36E-03 |
| 1.91E+03 | 6.56E-03 |
| 1.91E+03 | 6.81E-03 |
| 1.91E+03 | 7.02E-03 |
| 1.91E+03 | 7.15E-03 |
| 1.91E+03 | 7.32E-03 |
| 1.91E+03 | 7.56E-03 |
| 1.92E+03 | 7.85E-03 |
| 1.92E+03 | 8.18E-03 |
| 1.92E+03 | 8.52E-03 |
| 1.92E+03 | 8.85E-03 |
| 1.92E+03 | 9.17E-03 |
| 1.93E+03 | 9.58E-03 |
| 1.93E+03 | 1.01E-02 |
| 1.93E+03 | 1.05E-02 |
| 1.93E+03 | 1.08E-02 |
| 1.93E+03 | 1.11E-02 |
| 1.94E+03 | 1.14E-02 |
| 1.94E+03 | 1.17E-02 |
| 1.94E+03 | 1.20E-02 |
| 1.94E+03 | 1.24E-02 |
| 1.94E+03 | 1.28E-02 |
| 1.95E+03 | 1.33E-02 |
| 1.95E+03 | 1.38E-02 |
| 1.95E+03 | 1.44E-02 |
| 1.95E+03 | 1.50E-02 |
| 1.95E+03 | 1.56E-02 |
| 1.96E+03 | 1.61E-02 |
| 1.96E+03 | 1.65E-02 |
| 1.96E+03 | 1.68E-02 |
| 1.96E+03 | 1.72E-02 |
| 1.96E+03 | 1.75E-02 |
| 1.97E+03 | 1.79E-02 |
| 1.97E+03 | 1.82E-02 |
| 1.97E+03 | 1.85E-02 |
| 1.97E+03 | 1.88E-02 |
| 1.97E+03 | 1.92E-02 |
| 1.97E+03 | 1.96E-02 |
| 1.98E+03 | 2.00E-02 |
| 1.98E+03 | 2.04E-02 |
| 1.98E+03 | 2.06E-02 |
| 1.98E+03 | 2.08E-02 |
| 1.98E+03 | 2.10E-02 |
| 1.99E+03 | 2.12E-02 |
| 1.99E+03 | 2.15E-02 |
| 1.99E+03 | 2.17E-02 |
| 1.99E+03 | 2.20E-02 |
| 1.99E+03 | 2.23E-02 |
| 2.00E+03 | 2.27E-02 |
| 2.00E+03 | 2.30E-02 |
| 2.00E+03 | 2.34E-02 |
| 2.00E+03 | 2.38E-02 |
| 2.00E+03 | 2.42E-02 |
| 2.01E+03 | 2.45E-02 |
| 2.01E+03 | 2.48E-02 |
| 2.01E+03 | 2.52E-02 |
| 2.01E+03 | 2.56E-02 |
| 2.01E+03 | 2.60E-02 |
| 2.02E+03 | 2.64E-02 |
| 2.02E+03 | 2.68E-02 |
| 2.02E+03 | 2.73E-02 |
| 2.02E+03 | 2.78E-02 |
| 2.02E+03 | 2.83E-02 |
| 2.02E+03 | 2.88E-02 |
| 2.03E+03 | 2.93E-02 |
| 2.03E+03 | 2.97E-02 |
| 2.03E+03 | 3.02E-02 |
| 2.03E+03 | 3.06E-02 |
| 2.03E+03 | 3.10E-02 |
| 2.04E+03 | 3.14E-02 |
| 2.04E+03 | 3.19E-02 |
| 2.04E+03 | 3.23E-02 |
| 2.04E+03 | 3.27E-02 |
| 2.04E+03 | 3.31E-02 |
| 2.05E+03 | 3.35E-02 |
| 2.05E+03 | 3.40E-02 |
| 2.05E+03 | 3.44E-02 |
| 2.05E+03 | 3.48E-02 |
| 2.05E+03 | 3.51E-02 |
| 2.06E+03 | 3.54E-02 |
| 2.06E+03 | 3.58E-02 |
| 2.06E+03 | 3.61E-02 |
| 2.06E+03 | 3.64E-02 |
| 2.06E+03 | 3.67E-02 |
| 2.07E+03 | 3.70E-02 |
| 2.07E+03 | 3.73E-02 |
| 2.07E+03 | 3.76E-02 |
| 2.07E+03 | 3.79E-02 |
| 2.07E+03 | 3.82E-02 |
| 2.08E+03 | 3.85E-02 |
| 2.08E+03 | 3.88E-02 |
| 2.08E+03 | 3.90E-02 |
| 2.08E+03 | 3.94E-02 |
| 2.08E+03 | 3.97E-02 |
| 2.08E+03 | 4.01E-02 |
| 2.09E+03 | 4.04E-02 |
| 2.09E+03 | 4.08E-02 |
| 2.09E+03 | 4.12E-02 |
| 2.09E+03 | 4.15E-02 |
| 2.09E+03 | 4.19E-02 |
| 2.10E+03 | 4.23E-02 |
| 2.10E+03 | 4.27E-02 |
| 2.10E+03 | 4.31E-02 |
| 2.10E+03 | 4.34E-02 |
| 2.10E+03 | 4.37E-02 |
| 2.11E+03 | 4.41E-02 |
| 2.11E+03 | 4.44E-02 |
| 2.11E+03 | 4.47E-02 |
| 2.11E+03 | 4.50E-02 |
| 2.11E+03 | 4.53E-02 |
| 2.12E+03 | 4.56E-02 |
| 2.12E+03 | 4.59E-02 |
| 2.12E+03 | 4.61E-02 |
| 2.12E+03 | 4.63E-02 |
| 2.12E+03 | 4.65E-02 |
| 2.13E+03 | 4.67E-02 |
| 2.13E+03 | 4.69E-02 |
| 2.13E+03 | 4.71E-02 |
| 2.13E+03 | 4.73E-02 |
| 2.13E+03 | 4.74E-02 |
| 2.13E+03 | 4.76E-02 |
| 2.14E+03 | 4.77E-02 |
| 2.14E+03 | 4.79E-02 |
| 2.14E+03 | 4.80E-02 |
| 2.14E+03 | 4.81E-02 |
| 2.14E+03 | 4.82E-02 |
| 2.15E+03 | 4.83E-02 |
| 2.15E+03 | 4.83E-02 |
| 2.15E+03 | 4.83E-02 |
| 2.15E+03 | 4.84E-02 |
| 2.15E+03 | 4.83E-02 |
| 2.16E+03 | 4.83E-02 |
| 2.16E+03 | 4.83E-02 |
| 2.16E+03 | 4.82E-02 |
| 2.16E+03 | 4.82E-02 |
| 2.16E+03 | 4.81E-02 |
| 2.17E+03 | 4.80E-02 |
| 2.17E+03 | 4.80E-02 |
| 2.17E+03 | 4.79E-02 |
| 2.17E+03 | 4.79E-02 |
| 2.17E+03 | 4.78E-02 |
| 2.18E+03 | 4.78E-02 |
| 2.18E+03 | 4.78E-02 |
| 2.18E+03 | 4.78E-02 |
| 2.18E+03 | 4.78E-02 |
| 2.18E+03 | 4.78E-02 |
| 2.18E+03 | 4.78E-02 |
| 2.19E+03 | 4.79E-02 |
| 2.19E+03 | 4.79E-02 |
| 2.19E+03 | 4.79E-02 |
| 2.19E+03 | 4.79E-02 |
| 2.19E+03 | 4.79E-02 |
| 2.20E+03 | 4.79E-02 |
| 2.20E+03 | 4.79E-02 |
| 2.20E+03 | 4.78E-02 |
| 2.20E+03 | 4.78E-02 |
| 2.20E+03 | 4.77E-02 |
| 2.21E+03 | 4.77E-02 |
| 2.21E+03 | 4.76E-02 |
| 2.21E+03 | 4.75E-02 |
| 2.21E+03 | 4.75E-02 |
| 2.21E+03 | 4.74E-02 |
| 2.22E+03 | 4.73E-02 |
| 2.22E+03 | 4.72E-02 |
| 2.22E+03 | 4.72E-02 |
| 2.22E+03 | 4.71E-02 |
| 2.22E+03 | 4.70E-02 |
| 2.23E+03 | 4.69E-02 |
| 2.23E+03 | 4.69E-02 |
| 2.23E+03 | 4.68E-02 |
| 2.23E+03 | 4.67E-02 |
| 2.23E+03 | 4.66E-02 |
| 2.24E+03 | 4.66E-02 |
| 2.24E+03 | 4.65E-02 |
| 2.24E+03 | 4.64E-02 |
| 2.24E+03 | 4.64E-02 |
| 2.24E+03 | 4.63E-02 |
| 2.24E+03 | 4.63E-02 |
| 2.25E+03 | 4.62E-02 |
| 2.25E+03 | 4.62E-02 |
| 2.25E+03 | 4.61E-02 |
| 2.25E+03 | 4.60E-02 |
| 2.25E+03 | 4.59E-02 |
| 2.26E+03 | 4.58E-02 |
| 2.26E+03 | 4.57E-02 |
| 2.26E+03 | 4.56E-02 |
| 2.26E+03 | 4.55E-02 |
| 2.26E+03 | 4.54E-02 |
| 2.27E+03 | 4.53E-02 |
| 2.27E+03 | 4.52E-02 |
| 2.27E+03 | 4.51E-02 |
| 2.27E+03 | 4.50E-02 |
| 2.27E+03 | 4.49E-02 |
| 2.28E+03 | 4.48E-02 |
| 2.28E+03 | 4.47E-02 |
| 2.28E+03 | 4.47E-02 |
| 2.28E+03 | 4.46E-02 |
| 2.28E+03 | 4.45E-02 |
| 2.29E+03 | 4.45E-02 |
| 2.29E+03 | 4.45E-02 |
| 2.29E+03 | 4.45E-02 |
| 2.29E+03 | 4.44E-02 |
| 2.29E+03 | 4.44E-02 |
| 2.29E+03 | 4.44E-02 |
| 2.30E+03 | 4.44E-02 |
| 2.30E+03 | 4.44E-02 |
| 2.30E+03 | 4.44E-02 |
| 2.30E+03 | 4.45E-02 |
| 2.30E+03 | 4.45E-02 |
| 2.31E+03 | 4.45E-02 |
| 2.31E+03 | 4.46E-02 |
| 2.31E+03 | 4.47E-02 |
| 2.31E+03 | 4.47E-02 |
| 2.31E+03 | 4.48E-02 |
| 2.32E+03 | 4.49E-02 |
| 2.32E+03 | 4.49E-02 |
| 2.32E+03 | 4.50E-02 |
| 2.32E+03 | 4.51E-02 |
| 2.32E+03 | 4.51E-02 |
| 2.33E+03 | 4.52E-02 |
| 2.33E+03 | 4.52E-02 |
| 2.33E+03 | 4.53E-02 |
| 2.33E+03 | 4.54E-02 |
| 2.33E+03 | 4.54E-02 |
| 2.34E+03 | 4.55E-02 |
| 2.34E+03 | 4.56E-02 |
| 2.34E+03 | 4.57E-02 |
| 2.34E+03 | 4.59E-02 |
| 2.34E+03 | 4.60E-02 |
| 2.35E+03 | 4.61E-02 |
| 2.35E+03 | 4.62E-02 |
| 2.35E+03 | 4.63E-02 |
| 2.35E+03 | 4.63E-02 |
| 2.35E+03 | 4.64E-02 |
| 2.35E+03 | 4.65E-02 |
| 2.36E+03 | 4.66E-02 |
| 2.36E+03 | 4.67E-02 |
| 2.36E+03 | 4.68E-02 |
| 2.36E+03 | 4.70E-02 |
| 2.36E+03 | 4.71E-02 |
| 2.37E+03 | 4.72E-02 |
| 2.37E+03 | 4.73E-02 |
| 2.37E+03 | 4.74E-02 |
| 2.37E+03 | 4.75E-02 |
| 2.37E+03 | 4.75E-02 |
| 2.38E+03 | 4.75E-02 |
| 2.38E+03 | 4.75E-02 |
| 2.38E+03 | 4.76E-02 |
| 2.38E+03 | 4.76E-02 |
| 2.38E+03 | 4.77E-02 |
| 2.39E+03 | 4.77E-02 |
| 2.39E+03 | 4.78E-02 |
| 2.39E+03 | 4.79E-02 |
| 2.39E+03 | 4.80E-02 |
| 2.39E+03 | 4.81E-02 |
| 2.40E+03 | 4.83E-02 |
| 2.40E+03 | 4.85E-02 |
| 2.40E+03 | 4.86E-02 |
| 2.40E+03 | 4.88E-02 |
| 2.40E+03 | 4.90E-02 |
| 2.40E+03 | 4.92E-02 |
| 2.41E+03 | 4.94E-02 |
| 2.41E+03 | 4.96E-02 |
| 2.41E+03 | 4.98E-02 |
| 2.41E+03 | 4.99E-02 |
| 2.41E+03 | 5.01E-02 |
| 2.42E+03 | 5.03E-02 |
| 2.42E+03 | 5.05E-02 |
| 2.42E+03 | 5.07E-02 |
| 2.42E+03 | 5.09E-02 |
| 2.42E+03 | 5.12E-02 |
| 2.43E+03 | 5.14E-02 |
| 2.43E+03 | 5.16E-02 |
| 2.43E+03 | 5.19E-02 |
| 2.43E+03 | 5.22E-02 |
| 2.43E+03 | 5.24E-02 |
| 2.44E+03 | 5.27E-02 |
| 2.44E+03 | 5.30E-02 |
| 2.44E+03 | 5.33E-02 |
| 2.44E+03 | 5.37E-02 |
| 2.44E+03 | 5.40E-02 |
| 2.45E+03 | 5.44E-02 |
| 2.45E+03 | 5.47E-02 |
| 2.45E+03 | 5.51E-02 |
| 2.45E+03 | 5.54E-02 |
| 2.45E+03 | 5.58E-02 |
| 2.45E+03 | 5.62E-02 |
| 2.46E+03 | 5.65E-02 |
| 2.46E+03 | 5.69E-02 |
| 2.46E+03 | 5.73E-02 |
| 2.46E+03 | 5.76E-02 |
| 2.46E+03 | 5.80E-02 |
| 2.47E+03 | 5.84E-02 |
| 2.47E+03 | 5.87E-02 |
| 2.47E+03 | 5.91E-02 |
| 2.47E+03 | 5.95E-02 |
| 2.47E+03 | 5.99E-02 |
| 2.48E+03 | 6.03E-02 |
| 2.48E+03 | 6.07E-02 |
| 2.48E+03 | 6.11E-02 |
| 2.48E+03 | 6.16E-02 |
| 2.48E+03 | 6.20E-02 |
| 2.49E+03 | 6.24E-02 |
| 2.49E+03 | 6.29E-02 |
| 2.49E+03 | 6.33E-02 |
| 2.49E+03 | 6.37E-02 |
| 2.49E+03 | 6.41E-02 |
| 2.50E+03 | 6.46E-02 |
| 2.50E+03 | 6.50E-02 |
| 2.50E+03 | 6.54E-02 |
| 2.50E+03 | 6.58E-02 |
| 2.50E+03 | 6.62E-02 |
| 2.51E+03 | 6.67E-02 |
| 2.51E+03 | 6.71E-02 |
| 2.51E+03 | 6.74E-02 |
| 2.51E+03 | 6.79E-02 |
| 2.51E+03 | 6.83E-02 |
| 2.51E+03 | 6.87E-02 |
| 2.52E+03 | 6.91E-02 |
| 2.52E+03 | 6.95E-02 |
| 2.52E+03 | 6.99E-02 |
| 2.52E+03 | 7.03E-02 |
| 2.52E+03 | 7.07E-02 |
| 2.53E+03 | 7.12E-02 |
| 2.53E+03 | 7.16E-02 |
| 2.53E+03 | 7.20E-02 |
| 2.53E+03 | 7.23E-02 |
| 2.53E+03 | 7.27E-02 |
| 2.54E+03 | 7.31E-02 |
| 2.54E+03 | 7.35E-02 |
| 2.54E+03 | 7.39E-02 |
| 2.54E+03 | 7.43E-02 |
| 2.54E+03 | 7.47E-02 |
| 2.55E+03 | 7.51E-02 |
| 2.55E+03 | 7.55E-02 |
| 2.55E+03 | 7.60E-02 |
| 2.55E+03 | 7.64E-02 |
| 2.55E+03 | 7.69E-02 |
| 2.56E+03 | 7.73E-02 |
| 2.56E+03 | 7.77E-02 |
| 2.56E+03 | 7.82E-02 |
| 2.56E+03 | 7.86E-02 |
| 2.56E+03 | 7.91E-02 |
| 2.56E+03 | 7.95E-02 |
| 2.57E+03 | 7.99E-02 |
| 2.57E+03 | 8.04E-02 |
| 2.57E+03 | 8.08E-02 |
| 2.57E+03 | 8.12E-02 |
| 2.57E+03 | 8.16E-02 |
| 2.58E+03 | 8.21E-02 |
| 2.58E+03 | 8.25E-02 |
| 2.58E+03 | 8.29E-02 |
| 2.58E+03 | 8.33E-02 |
| 2.58E+03 | 8.38E-02 |
| 2.59E+03 | 8.42E-02 |
| 2.59E+03 | 8.46E-02 |
| 2.59E+03 | 8.51E-02 |
| 2.59E+03 | 8.55E-02 |
| 2.59E+03 | 8.60E-02 |
| 2.60E+03 | 8.64E-02 |
| 2.60E+03 | 8.68E-02 |
| 2.60E+03 | 8.72E-02 |
| 2.60E+03 | 8.77E-02 |
| 2.60E+03 | 8.81E-02 |
| 2.61E+03 | 8.85E-02 |
| 2.61E+03 | 8.89E-02 |
| 2.61E+03 | 8.93E-02 |
| 2.61E+03 | 8.97E-02 |
| 2.61E+03 | 9.01E-02 |
| 2.62E+03 | 9.05E-02 |
| 2.62E+03 | 9.09E-02 |
| 2.62E+03 | 9.14E-02 |
| 2.62E+03 | 9.18E-02 |
| 2.62E+03 | 9.23E-02 |
| 2.62E+03 | 9.28E-02 |
| 2.63E+03 | 9.33E-02 |
| 2.63E+03 | 9.38E-02 |
| 2.63E+03 | 9.43E-02 |
| 2.63E+03 | 9.48E-02 |
| 2.63E+03 | 9.54E-02 |
| 2.64E+03 | 9.60E-02 |
| 2.64E+03 | 9.66E-02 |
| 2.64E+03 | 9.72E-02 |
| 2.64E+03 | 9.78E-02 |
| 2.64E+03 | 9.84E-02 |
| 2.65E+03 | 9.90E-02 |
| 2.65E+03 | 9.96E-02 |
| 2.65E+03 | 1.00E-01 |
| 2.65E+03 | 1.01E-01 |
| 2.65E+03 | 1.01E-01 |
| 2.66E+03 | 1.02E-01 |
| 2.66E+03 | 1.02E-01 |
| 2.66E+03 | 1.03E-01 |
| 2.66E+03 | 1.04E-01 |
| 2.66E+03 | 1.04E-01 |
| 2.67E+03 | 1.05E-01 |
| 2.67E+03 | 1.05E-01 |
| 2.67E+03 | 1.06E-01 |
| 2.67E+03 | 1.06E-01 |
| 2.67E+03 | 1.07E-01 |
| 2.67E+03 | 1.08E-01 |
| 2.68E+03 | 1.08E-01 |
| 2.68E+03 | 1.09E-01 |
| 2.68E+03 | 1.09E-01 |
| 2.68E+03 | 1.10E-01 |
| 2.68E+03 | 1.10E-01 |
| 2.69E+03 | 1.11E-01 |
| 2.69E+03 | 1.11E-01 |
| 2.69E+03 | 1.12E-01 |
| 2.69E+03 | 1.12E-01 |
| 2.69E+03 | 1.13E-01 |
| 2.70E+03 | 1.13E-01 |
| 2.70E+03 | 1.13E-01 |
| 2.70E+03 | 1.14E-01 |
| 2.70E+03 | 1.14E-01 |
| 2.70E+03 | 1.15E-01 |
| 2.71E+03 | 1.15E-01 |
| 2.71E+03 | 1.16E-01 |
| 2.71E+03 | 1.16E-01 |
| 2.71E+03 | 1.17E-01 |
| 2.71E+03 | 1.17E-01 |
| 2.72E+03 | 1.17E-01 |
| 2.72E+03 | 1.18E-01 |
| 2.72E+03 | 1.18E-01 |
| 2.72E+03 | 1.19E-01 |
| 2.72E+03 | 1.19E-01 |
| 2.72E+03 | 1.19E-01 |
| 2.73E+03 | 1.20E-01 |
| 2.73E+03 | 1.20E-01 |
| 2.73E+03 | 1.20E-01 |
| 2.73E+03 | 1.21E-01 |
| 2.73E+03 | 1.21E-01 |
| 2.74E+03 | 1.21E-01 |
| 2.74E+03 | 1.22E-01 |
| 2.74E+03 | 1.22E-01 |
| 2.74E+03 | 1.22E-01 |
| 2.74E+03 | 1.22E-01 |
| 2.75E+03 | 1.23E-01 |
| 2.75E+03 | 1.23E-01 |
| 2.75E+03 | 1.23E-01 |
| 2.75E+03 | 1.24E-01 |
| 2.75E+03 | 1.24E-01 |
| 2.76E+03 | 1.24E-01 |
| 2.76E+03 | 1.25E-01 |
| 2.76E+03 | 1.25E-01 |
| 2.76E+03 | 1.26E-01 |
| 2.76E+03 | 1.26E-01 |
| 2.77E+03 | 1.27E-01 |
| 2.77E+03 | 1.27E-01 |
| 2.77E+03 | 1.27E-01 |
| 2.77E+03 | 1.28E-01 |
| 2.77E+03 | 1.28E-01 |
| 2.78E+03 | 1.29E-01 |
| 2.78E+03 | 1.29E-01 |
| 2.78E+03 | 1.29E-01 |
| 2.78E+03 | 1.30E-01 |
| 2.78E+03 | 1.30E-01 |
| 2.78E+03 | 1.31E-01 |
| 2.79E+03 | 1.31E-01 |
| 2.79E+03 | 1.32E-01 |
| 2.79E+03 | 1.32E-01 |
| 2.79E+03 | 1.33E-01 |
| 2.79E+03 | 1.34E-01 |
| 2.80E+03 | 1.34E-01 |
| 2.80E+03 | 1.35E-01 |
| 2.80E+03 | 1.35E-01 |
| 2.80E+03 | 1.36E-01 |
| 2.80E+03 | 1.36E-01 |
| 2.81E+03 | 1.37E-01 |
| 2.81E+03 | 1.37E-01 |
| 2.81E+03 | 1.38E-01 |
| 2.81E+03 | 1.38E-01 |
| 2.81E+03 | 1.39E-01 |
| 2.82E+03 | 1.39E-01 |
| 2.82E+03 | 1.40E-01 |
| 2.82E+03 | 1.40E-01 |
| 2.82E+03 | 1.41E-01 |
| 2.82E+03 | 1.41E-01 |
| 2.83E+03 | 1.42E-01 |
| 2.83E+03 | 1.42E-01 |
| 2.83E+03 | 1.43E-01 |
| 2.83E+03 | 1.43E-01 |
| 2.83E+03 | 1.44E-01 |
| 2.83E+03 | 1.45E-01 |
| 2.84E+03 | 1.45E-01 |
| 2.84E+03 | 1.46E-01 |
| 2.84E+03 | 1.47E-01 |
| 2.84E+03 | 1.48E-01 |
| 2.84E+03 | 1.49E-01 |
| 2.85E+03 | 1.50E-01 |
| 2.85E+03 | 1.51E-01 |
| 2.85E+03 | 1.51E-01 |
| 2.85E+03 | 1.52E-01 |
| 2.85E+03 | 1.53E-01 |
| 2.86E+03 | 1.53E-01 |
| 2.86E+03 | 1.54E-01 |
| 2.86E+03 | 1.54E-01 |
| 2.86E+03 | 1.54E-01 |
| 2.86E+03 | 1.55E-01 |
| 2.87E+03 | 1.55E-01 |
| 2.87E+03 | 1.55E-01 |
| 2.87E+03 | 1.55E-01 |
| 2.87E+03 | 1.56E-01 |
| 2.87E+03 | 1.56E-01 |
| 2.88E+03 | 1.56E-01 |
| 2.88E+03 | 1.56E-01 |
| 2.88E+03 | 1.56E-01 |
| 2.88E+03 | 1.56E-01 |
| 2.88E+03 | 1.57E-01 |
| 2.88E+03 | 1.57E-01 |
| 2.89E+03 | 1.57E-01 |
| 2.89E+03 | 1.57E-01 |
| 2.89E+03 | 1.57E-01 |
| 2.89E+03 | 1.57E-01 |
| 2.89E+03 | 1.57E-01 |
| 2.90E+03 | 1.57E-01 |
| 2.90E+03 | 1.57E-01 |
| 2.90E+03 | 1.57E-01 |
| 2.90E+03 | 1.57E-01 |
| 2.90E+03 | 1.57E-01 |
| 2.91E+03 | 1.57E-01 |
| 2.91E+03 | 1.58E-01 |
| 2.91E+03 | 1.58E-01 |
| 2.91E+03 | 1.58E-01 |
| 2.91E+03 | 1.59E-01 |
| 2.92E+03 | 1.59E-01 |
| 2.92E+03 | 1.59E-01 |
| 2.92E+03 | 1.60E-01 |
| 2.92E+03 | 1.60E-01 |
| 2.92E+03 | 1.60E-01 |
| 2.93E+03 | 1.60E-01 |
| 2.93E+03 | 1.59E-01 |
| 2.93E+03 | 1.59E-01 |
| 2.93E+03 | 1.59E-01 |
| 2.93E+03 | 1.58E-01 |
| 2.94E+03 | 1.58E-01 |
| 2.94E+03 | 1.57E-01 |
| 2.94E+03 | 1.57E-01 |
| 2.94E+03 | 1.56E-01 |
| 2.94E+03 | 1.56E-01 |
| 2.94E+03 | 1.56E-01 |
| 2.95E+03 | 1.55E-01 |
| 2.95E+03 | 1.55E-01 |
| 2.95E+03 | 1.55E-01 |
| 2.95E+03 | 1.55E-01 |
| 2.95E+03 | 1.55E-01 |
| 2.96E+03 | 1.54E-01 |
| 2.96E+03 | 1.54E-01 |
| 2.96E+03 | 1.54E-01 |
| 2.96E+03 | 1.54E-01 |
| 2.96E+03 | 1.54E-01 |
| 2.97E+03 | 1.53E-01 |
| 2.97E+03 | 1.53E-01 |
| 2.97E+03 | 1.53E-01 |
| 2.97E+03 | 1.53E-01 |
| 2.97E+03 | 1.52E-01 |
| 2.98E+03 | 1.52E-01 |
| 2.98E+03 | 1.52E-01 |
| 2.98E+03 | 1.52E-01 |
| 2.98E+03 | 1.52E-01 |
| 2.98E+03 | 1.52E-01 |
| 2.99E+03 | 1.52E-01 |
| 2.99E+03 | 1.52E-01 |
| 2.99E+03 | 1.52E-01 |
| 2.99E+03 | 1.52E-01 |
| 2.99E+03 | 1.52E-01 |
| 2.99E+03 | 1.52E-01 |
| 3.00E+03 | 1.52E-01 |
| 3.00E+03 | 1.52E-01 |
| 3.00E+03 | 1.53E-01 |
| 3.00E+03 | 1.53E-01 |
| 3.00E+03 | 1.53E-01 |
| 3.01E+03 | 1.53E-01 |
| 3.01E+03 | 1.54E-01 |
| 3.01E+03 | 1.54E-01 |
| 3.01E+03 | 1.54E-01 |
| 3.01E+03 | 1.54E-01 |
| 3.02E+03 | 1.55E-01 |
| 3.02E+03 | 1.55E-01 |
| 3.02E+03 | 1.55E-01 |
| 3.02E+03 | 1.56E-01 |
| 3.02E+03 | 1.56E-01 |
| 3.03E+03 | 1.56E-01 |
| 3.03E+03 | 1.57E-01 |
| 3.03E+03 | 1.57E-01 |
| 3.03E+03 | 1.58E-01 |
| 3.03E+03 | 1.58E-01 |
| 3.04E+03 | 1.58E-01 |
| 3.04E+03 | 1.59E-01 |
| 3.04E+03 | 1.59E-01 |
| 3.04E+03 | 1.59E-01 |
| 3.04E+03 | 1.60E-01 |
| 3.05E+03 | 1.60E-01 |
| 3.05E+03 | 1.61E-01 |
| 3.05E+03 | 1.61E-01 |
| 3.05E+03 | 1.61E-01 |
| 3.05E+03 | 1.62E-01 |
| 3.05E+03 | 1.62E-01 |
| 3.06E+03 | 1.63E-01 |
| 3.06E+03 | 1.63E-01 |
| 3.06E+03 | 1.63E-01 |
| 3.06E+03 | 1.64E-01 |
| 3.06E+03 | 1.64E-01 |
| 3.07E+03 | 1.65E-01 |
| 3.07E+03 | 1.65E-01 |
| 3.07E+03 | 1.66E-01 |
| 3.07E+03 | 1.66E-01 |
| 3.07E+03 | 1.66E-01 |
| 3.08E+03 | 1.67E-01 |
| 3.08E+03 | 1.67E-01 |
| 3.08E+03 | 1.68E-01 |
| 3.08E+03 | 1.68E-01 |
| 3.08E+03 | 1.68E-01 |
| 3.09E+03 | 1.69E-01 |
| 3.09E+03 | 1.69E-01 |
| 3.09E+03 | 1.70E-01 |
| 3.09E+03 | 1.70E-01 |
| 3.09E+03 | 1.70E-01 |
| 3.10E+03 | 1.71E-01 |
| 3.10E+03 | 1.71E-01 |
| 3.10E+03 | 1.72E-01 |
| 3.10E+03 | 1.72E-01 |
| 3.10E+03 | 1.72E-01 |
| 3.10E+03 | 1.73E-01 |
| 3.11E+03 | 1.73E-01 |
| 3.11E+03 | 1.73E-01 |
| 3.11E+03 | 1.74E-01 |
| 3.11E+03 | 1.74E-01 |
| 3.11E+03 | 1.74E-01 |
| 3.12E+03 | 1.75E-01 |
| 3.12E+03 | 1.75E-01 |
| 3.12E+03 | 1.76E-01 |
| 3.12E+03 | 1.76E-01 |
| 3.12E+03 | 1.77E-01 |
| 3.13E+03 | 1.77E-01 |
| 3.13E+03 | 1.78E-01 |
| 3.13E+03 | 1.78E-01 |
| 3.13E+03 | 1.79E-01 |
| 3.13E+03 | 1.79E-01 |
| 3.14E+03 | 1.80E-01 |
| 3.14E+03 | 1.80E-01 |
| 3.14E+03 | 1.81E-01 |
| 3.14E+03 | 1.81E-01 |
| 3.14E+03 | 1.82E-01 |
| 3.15E+03 | 1.82E-01 |
| 3.15E+03 | 1.83E-01 |
| 3.15E+03 | 1.83E-01 |
| 3.15E+03 | 1.84E-01 |
| 3.15E+03 | 1.84E-01 |
| 3.15E+03 | 1.85E-01 |
| 3.16E+03 | 1.86E-01 |
| 3.16E+03 | 1.86E-01 |
| 3.16E+03 | 1.87E-01 |
| 3.16E+03 | 1.87E-01 |
| 3.16E+03 | 1.88E-01 |
| 3.17E+03 | 1.89E-01 |
| 3.17E+03 | 1.89E-01 |
| 3.17E+03 | 1.90E-01 |
| 3.17E+03 | 1.90E-01 |
| 3.17E+03 | 1.91E-01 |
| 3.18E+03 | 1.92E-01 |
| 3.18E+03 | 1.92E-01 |
| 3.18E+03 | 1.93E-01 |
| 3.18E+03 | 1.94E-01 |
| 3.18E+03 | 1.94E-01 |
| 3.19E+03 | 1.95E-01 |
| 3.19E+03 | 1.96E-01 |
| 3.19E+03 | 1.96E-01 |
| 3.19E+03 | 1.97E-01 |
| 3.19E+03 | 1.98E-01 |
| 3.20E+03 | 1.98E-01 |
| 3.20E+03 | 1.99E-01 |
| 3.20E+03 | 2.00E-01 |
| 3.20E+03 | 2.00E-01 |
| 3.20E+03 | 2.01E-01 |
| 3.21E+03 | 2.02E-01 |
| 3.21E+03 | 2.02E-01 |
| 3.21E+03 | 2.03E-01 |
| 3.21E+03 | 2.04E-01 |
| 3.21E+03 | 2.04E-01 |
| 3.21E+03 | 2.05E-01 |
| 3.22E+03 | 2.06E-01 |
| 3.22E+03 | 2.06E-01 |
| 3.22E+03 | 2.07E-01 |
| 3.22E+03 | 2.08E-01 |
| 3.22E+03 | 2.08E-01 |
| 3.23E+03 | 2.09E-01 |
| 3.23E+03 | 2.10E-01 |
| 3.23E+03 | 2.10E-01 |
| 3.23E+03 | 2.11E-01 |
| 3.23E+03 | 2.12E-01 |
| 3.24E+03 | 2.12E-01 |
| 3.24E+03 | 2.13E-01 |
| 3.24E+03 | 2.13E-01 |
| 3.24E+03 | 2.14E-01 |
| 3.24E+03 | 2.15E-01 |
| 3.25E+03 | 2.15E-01 |
| 3.25E+03 | 2.16E-01 |
| 3.25E+03 | 2.16E-01 |
| 3.25E+03 | 2.17E-01 |
| 3.25E+03 | 2.17E-01 |
| 3.26E+03 | 2.18E-01 |
| 3.26E+03 | 2.18E-01 |
| 3.26E+03 | 2.19E-01 |
| 3.26E+03 | 2.19E-01 |
| 3.26E+03 | 2.20E-01 |
| 3.26E+03 | 2.20E-01 |
| 3.27E+03 | 2.21E-01 |
| 3.27E+03 | 2.21E-01 |
| 3.27E+03 | 2.22E-01 |
| 3.27E+03 | 2.22E-01 |
| 3.27E+03 | 2.23E-01 |
| 3.28E+03 | 2.23E-01 |
| 3.28E+03 | 2.24E-01 |
| 3.28E+03 | 2.24E-01 |
| 3.28E+03 | 2.25E-01 |
| 3.28E+03 | 2.26E-01 |
| 3.29E+03 | 2.26E-01 |
| 3.29E+03 | 2.27E-01 |
| 3.29E+03 | 2.27E-01 |
| 3.29E+03 | 2.28E-01 |
| 3.29E+03 | 2.28E-01 |
| 3.30E+03 | 2.29E-01 |
| 3.30E+03 | 2.30E-01 |
| 3.30E+03 | 2.30E-01 |
| 3.30E+03 | 2.31E-01 |
| 3.30E+03 | 2.31E-01 |
| 3.31E+03 | 2.32E-01 |
| 3.31E+03 | 2.33E-01 |
| 3.31E+03 | 2.33E-01 |
| 3.31E+03 | 2.34E-01 |
| 3.31E+03 | 2.35E-01 |
| 3.32E+03 | 2.35E-01 |
| 3.32E+03 | 2.36E-01 |
| 3.32E+03 | 2.37E-01 |
| 3.32E+03 | 2.38E-01 |
| 3.32E+03 | 2.38E-01 |
| 3.32E+03 | 2.39E-01 |
| 3.33E+03 | 2.40E-01 |
| 3.33E+03 | 2.41E-01 |
| 3.33E+03 | 2.42E-01 |
| 3.33E+03 | 2.43E-01 |
| 3.33E+03 | 2.44E-01 |
| 3.34E+03 | 2.45E-01 |
| 3.34E+03 | 2.45E-01 |
| 3.34E+03 | 2.46E-01 |
| 3.34E+03 | 2.47E-01 |
| 3.34E+03 | 2.48E-01 |
| 3.35E+03 | 2.49E-01 |
| 3.35E+03 | 2.50E-01 |
| 3.35E+03 | 2.51E-01 |
| 3.35E+03 | 2.52E-01 |
| 3.35E+03 | 2.53E-01 |
| 3.36E+03 | 2.54E-01 |
| 3.36E+03 | 2.55E-01 |
| 3.36E+03 | 2.56E-01 |
| 3.36E+03 | 2.57E-01 |
| 3.36E+03 | 2.58E-01 |
| 3.37E+03 | 2.58E-01 |
| 3.37E+03 | 2.59E-01 |
| 3.37E+03 | 2.60E-01 |
| 3.37E+03 | 2.61E-01 |
| 3.37E+03 | 2.62E-01 |
| 3.37E+03 | 2.63E-01 |
| 3.38E+03 | 2.63E-01 |
| 3.38E+03 | 2.64E-01 |
| 3.38E+03 | 2.65E-01 |
| 3.38E+03 | 2.66E-01 |
| 3.38E+03 | 2.67E-01 |
| 3.39E+03 | 2.67E-01 |
| 3.39E+03 | 2.68E-01 |
| 3.39E+03 | 2.69E-01 |
| 3.39E+03 | 2.69E-01 |
| 3.39E+03 | 2.70E-01 |
| 3.40E+03 | 2.71E-01 |
| 3.40E+03 | 2.71E-01 |
| 3.40E+03 | 2.72E-01 |
| 3.40E+03 | 2.72E-01 |
| 3.40E+03 | 2.73E-01 |
| 3.41E+03 | 2.74E-01 |
| 3.41E+03 | 2.74E-01 |
| 3.41E+03 | 2.74E-01 |
| 3.41E+03 | 2.75E-01 |
| 3.41E+03 | 2.75E-01 |
| 3.42E+03 | 2.76E-01 |
| 3.42E+03 | 2.76E-01 |
| 3.42E+03 | 2.76E-01 |
| 3.42E+03 | 2.77E-01 |
| 3.42E+03 | 2.77E-01 |
| 3.42E+03 | 2.77E-01 |
| 3.43E+03 | 2.78E-01 |
| 3.43E+03 | 2.78E-01 |
| 3.43E+03 | 2.78E-01 |
| 3.43E+03 | 2.78E-01 |
| 3.43E+03 | 2.78E-01 |
| 3.44E+03 | 2.78E-01 |
| 3.44E+03 | 2.78E-01 |
| 3.44E+03 | 2.78E-01 |
| 3.44E+03 | 2.78E-01 |
| 3.44E+03 | 2.78E-01 |
| 3.45E+03 | 2.78E-01 |
| 3.45E+03 | 2.78E-01 |
| 3.45E+03 | 2.78E-01 |
| 3.45E+03 | 2.78E-01 |
| 3.45E+03 | 2.78E-01 |
| 3.46E+03 | 2.77E-01 |
| 3.46E+03 | 2.77E-01 |
| 3.46E+03 | 2.77E-01 |
| 3.46E+03 | 2.77E-01 |
| 3.46E+03 | 2.76E-01 |
| 3.47E+03 | 2.76E-01 |
| 3.47E+03 | 2.75E-01 |
| 3.47E+03 | 2.75E-01 |
| 3.47E+03 | 2.74E-01 |
| 3.47E+03 | 2.74E-01 |
| 3.48E+03 | 2.73E-01 |
| 3.48E+03 | 2.73E-01 |
| 3.48E+03 | 2.72E-01 |
| 3.48E+03 | 2.72E-01 |
| 3.48E+03 | 2.71E-01 |
| 3.48E+03 | 2.70E-01 |
| 3.49E+03 | 2.70E-01 |
| 3.49E+03 | 2.69E-01 |
| 3.49E+03 | 2.68E-01 |
| 3.49E+03 | 2.67E-01 |
| 3.49E+03 | 2.67E-01 |
| 3.50E+03 | 2.66E-01 |
| 3.50E+03 | 2.65E-01 |
| 3.50E+03 | 2.64E-01 |
| 3.50E+03 | 2.63E-01 |
| 3.50E+03 | 2.62E-01 |
| 3.51E+03 | 2.62E-01 |
| 3.51E+03 | 2.61E-01 |
| 3.51E+03 | 2.60E-01 |
| 3.51E+03 | 2.58E-01 |
| 3.51E+03 | 2.57E-01 |
| 3.52E+03 | 2.56E-01 |
| 3.52E+03 | 2.55E-01 |
| 3.52E+03 | 2.54E-01 |
| 3.52E+03 | 2.53E-01 |
| 3.52E+03 | 2.52E-01 |
| 3.53E+03 | 2.51E-01 |
| 3.53E+03 | 2.50E-01 |
| 3.53E+03 | 2.49E-01 |
| 3.53E+03 | 2.48E-01 |
| 3.53E+03 | 2.46E-01 |
| 3.53E+03 | 2.45E-01 |
| 3.54E+03 | 2.44E-01 |
| 3.54E+03 | 2.42E-01 |
| 3.54E+03 | 2.41E-01 |
| 3.54E+03 | 2.40E-01 |
| 3.54E+03 | 2.38E-01 |
| 3.55E+03 | 2.37E-01 |
| 3.55E+03 | 2.36E-01 |
| 3.55E+03 | 2.35E-01 |
| 3.55E+03 | 2.33E-01 |
| 3.55E+03 | 2.32E-01 |
| 3.56E+03 | 2.30E-01 |
| 3.56E+03 | 2.29E-01 |
| 3.56E+03 | 2.27E-01 |
| 3.56E+03 | 2.26E-01 |
| 3.56E+03 | 2.25E-01 |
| 3.57E+03 | 2.23E-01 |
| 3.57E+03 | 2.22E-01 |
| 3.57E+03 | 2.21E-01 |
| 3.57E+03 | 2.19E-01 |
| 3.57E+03 | 2.18E-01 |
| 3.58E+03 | 2.16E-01 |
| 3.58E+03 | 2.15E-01 |
| 3.58E+03 | 2.13E-01 |
| 3.58E+03 | 2.11E-01 |
| 3.58E+03 | 2.09E-01 |
| 3.59E+03 | 2.08E-01 |
| 3.59E+03 | 2.06E-01 |
| 3.59E+03 | 2.05E-01 |
| 3.59E+03 | 2.03E-01 |
| 3.59E+03 | 2.02E-01 |
| 3.59E+03 | 2.00E-01 |
| 3.60E+03 | 1.98E-01 |
| 3.60E+03 | 1.96E-01 |
| 3.60E+03 | 1.94E-01 |
| 3.60E+03 | 1.92E-01 |
| 3.60E+03 | 1.90E-01 |
| 3.61E+03 | 1.88E-01 |
| 3.61E+03 | 1.85E-01 |
| 3.61E+03 | 1.83E-01 |
| 3.61E+03 | 1.81E-01 |
| 3.61E+03 | 1.79E-01 |
| 3.62E+03 | 1.77E-01 |
| 3.62E+03 | 1.75E-01 |
| 3.62E+03 | 1.73E-01 |
| 3.62E+03 | 1.70E-01 |
| 3.62E+03 | 1.68E-01 |
| 3.63E+03 | 1.66E-01 |
| 3.63E+03 | 1.63E-01 |
| 3.63E+03 | 1.60E-01 |
| 3.63E+03 | 1.57E-01 |
| 3.63E+03 | 1.54E-01 |
| 3.64E+03 | 1.51E-01 |
| 3.64E+03 | 1.47E-01 |
| 3.64E+03 | 1.44E-01 |
| 3.64E+03 | 1.40E-01 |
| 3.64E+03 | 1.37E-01 |
| 3.64E+03 | 1.33E-01 |
| 3.65E+03 | 1.30E-01 |
| 3.65E+03 | 1.26E-01 |
| 3.65E+03 | 1.23E-01 |
| 3.65E+03 | 1.19E-01 |
| 3.65E+03 | 1.16E-01 |
| 3.66E+03 | 1.12E-01 |
| 3.66E+03 | 1.08E-01 |
| 3.66E+03 | 1.05E-01 |
| 3.66E+03 | 1.01E-01 |
| 3.66E+03 | 9.67E-02 |
| 3.67E+03 | 9.31E-02 |
| 3.67E+03 | 8.97E-02 |
| 3.67E+03 | 8.63E-02 |
| 3.67E+03 | 8.30E-02 |
| 3.67E+03 | 7.99E-02 |
| 3.68E+03 | 7.68E-02 |
| 3.68E+03 | 7.39E-02 |
| 3.68E+03 | 7.12E-02 |
| 3.68E+03 | 6.87E-02 |
| 3.68E+03 | 6.63E-02 |
| 3.69E+03 | 6.41E-02 |
| 3.69E+03 | 6.20E-02 |
| 3.69E+03 | 5.99E-02 |
| 3.69E+03 | 5.81E-02 |
| 3.69E+03 | 5.63E-02 |
| 3.69E+03 | 5.46E-02 |
| 3.70E+03 | 5.29E-02 |
| 3.70E+03 | 5.11E-02 |
| 3.70E+03 | 4.94E-02 |
| 3.70E+03 | 4.75E-02 |
| 3.70E+03 | 4.57E-02 |
| 3.71E+03 | 4.40E-02 |
| 3.71E+03 | 4.24E-02 |
| 3.71E+03 | 4.08E-02 |
| 3.71E+03 | 3.94E-02 |
| 3.71E+03 | 3.82E-02 |
| 3.72E+03 | 3.71E-02 |
| 3.72E+03 | 3.63E-02 |
| 3.72E+03 | 3.57E-02 |
| 3.72E+03 | 3.50E-02 |
| 3.72E+03 | 3.44E-02 |
| 3.73E+03 | 3.37E-02 |
| 3.73E+03 | 3.30E-02 |
| 3.73E+03 | 3.22E-02 |
| 3.73E+03 | 3.16E-02 |
| 3.73E+03 | 3.07E-02 |
| 3.74E+03 | 2.98E-02 |
| 3.74E+03 | 2.88E-02 |
| 3.74E+03 | 2.80E-02 |
| 3.74E+03 | 2.72E-02 |
| 3.74E+03 | 2.65E-02 |
| 3.75E+03 | 2.58E-02 |
| 3.75E+03 | 2.52E-02 |
| 3.75E+03 | 2.45E-02 |
| 3.75E+03 | 2.39E-02 |
| 3.75E+03 | 2.32E-02 |
| 3.75E+03 | 2.26E-02 |
| 3.76E+03 | 2.21E-02 |
| 3.76E+03 | 2.17E-02 |
| 3.76E+03 | 2.10E-02 |
| 3.76E+03 | 2.05E-02 |
| 3.76E+03 | 2.00E-02 |
| 3.77E+03 | 1.97E-02 |
| 3.77E+03 | 1.92E-02 |
| 3.77E+03 | 1.88E-02 |
| 3.77E+03 | 1.84E-02 |
| 3.77E+03 | 1.79E-02 |
| 3.78E+03 | 1.75E-02 |
| 3.78E+03 | 1.72E-02 |
| 3.78E+03 | 1.68E-02 |
| 3.78E+03 | 1.64E-02 |
| 3.78E+03 | 1.61E-02 |
| 3.79E+03 | 1.57E-02 |
| 3.79E+03 | 1.54E-02 |
| 3.79E+03 | 1.51E-02 |
| 3.79E+03 | 1.47E-02 |
| 3.79E+03 | 1.43E-02 |
| 3.80E+03 | 1.41E-02 |
| 3.80E+03 | 1.37E-02 |
| 3.80E+03 | 1.34E-02 |
| 3.80E+03 | 1.31E-02 |
| 3.80E+03 | 1.29E-02 |
| 3.80E+03 | 1.26E-02 |
| 3.81E+03 | 1.23E-02 |
| 3.81E+03 | 1.21E-02 |
| 3.81E+03 | 1.18E-02 |
| 3.81E+03 | 1.15E-02 |
| 3.81E+03 | 1.12E-02 |
| 3.82E+03 | 1.09E-02 |
| 3.82E+03 | 1.07E-02 |
| 3.82E+03 | 1.06E-02 |
| 3.82E+03 | 1.03E-02 |
| 3.82E+03 | 1.01E-02 |
| 3.83E+03 | 9.87E-03 |
| 3.83E+03 | 9.73E-03 |
| 3.83E+03 | 9.60E-03 |
| 3.83E+03 | 9.40E-03 |
| 3.83E+03 | 9.25E-03 |
| 3.84E+03 | 9.06E-03 |
| 3.84E+03 | 8.80E-03 |
| 3.84E+03 | 8.63E-03 |
| 3.84E+03 | 8.63E-03 |
| 3.84E+03 | 8.57E-03 |
| 3.85E+03 | 8.45E-03 |
| 3.85E+03 | 8.35E-03 |
| 3.85E+03 | 8.22E-03 |
| 3.85E+03 | 8.14E-03 |
| 3.85E+03 | 7.98E-03 |
| 3.86E+03 | 7.90E-03 |
| 3.86E+03 | 7.81E-03 |
| 3.86E+03 | 7.73E-03 |
| 3.86E+03 | 7.62E-03 |
| 3.86E+03 | 7.45E-03 |
| 3.86E+03 | 7.32E-03 |
| 3.87E+03 | 7.36E-03 |
| 3.87E+03 | 7.35E-03 |
| 3.87E+03 | 7.13E-03 |
| 3.87E+03 | 7.00E-03 |
| 3.87E+03 | 6.92E-03 |
| 3.88E+03 | 6.79E-03 |
| 3.88E+03 | 6.71E-03 |
| 3.88E+03 | 6.61E-03 |
| 3.88E+03 | 6.56E-03 |
| 3.88E+03 | 6.50E-03 |
| 3.89E+03 | 6.46E-03 |
| 3.89E+03 | 6.40E-03 |
| 3.89E+03 | 6.35E-03 |
| 3.89E+03 | 6.31E-03 |
| 3.89E+03 | 6.27E-03 |
| 3.90E+03 | 6.12E-03 |
| 3.90E+03 | 6.05E-03 |
| 3.90E+03 | 6.05E-03 |
| 3.90E+03 | 6.00E-03 |
| 3.90E+03 | 5.96E-03 |
| 3.91E+03 | 5.98E-03 |
| 3.91E+03 | 5.96E-03 |
| 3.91E+03 | 5.94E-03 |
| 3.91E+03 | 5.92E-03 |
| 3.91E+03 | 5.90E-03 |
| 3.91E+03 | 5.85E-03 |
| 3.92E+03 | 5.80E-03 |
| 3.92E+03 | 5.71E-03 |
| 3.92E+03 | 5.62E-03 |
| 3.92E+03 | 5.57E-03 |
| 3.92E+03 | 5.55E-03 |
| 3.93E+03 | 5.52E-03 |
| 3.93E+03 | 5.51E-03 |
| 3.93E+03 | 5.46E-03 |
| 3.93E+03 | 5.40E-03 |
| 3.93E+03 | 5.33E-03 |
| 3.94E+03 | 5.27E-03 |
| 3.94E+03 | 5.19E-03 |
| 3.94E+03 | 5.11E-03 |
| 3.94E+03 | 5.00E-03 |
| 3.94E+03 | 4.92E-03 |
| 3.95E+03 | 4.85E-03 |
| 3.95E+03 | 4.78E-03 |
| 3.95E+03 | 4.73E-03 |
| 3.95E+03 | 4.71E-03 |
| 3.95E+03 | 4.68E-03 |
| 3.96E+03 | 4.64E-03 |
| 3.96E+03 | 4.55E-03 |
| 3.96E+03 | 4.48E-03 |
| 3.96E+03 | 4.41E-03 |
| 3.96E+03 | 4.31E-03 |
| 3.96E+03 | 4.19E-03 |
| 3.97E+03 | 4.04E-03 |
| 3.97E+03 | 3.90E-03 |
| 3.97E+03 | 3.76E-03 |
| 3.97E+03 | 3.59E-03 |
| 3.97E+03 | 3.44E-03 |
| 3.98E+03 | 3.30E-03 |
| 3.98E+03 | 3.15E-03 |
| 3.98E+03 | 2.98E-03 |
| 3.98E+03 | 2.80E-03 |
| 3.98E+03 | 2.63E-03 |
| 3.99E+03 | 2.45E-03 |
| 3.99E+03 | 2.27E-03 |
| 3.99E+03 | 2.08E-03 |
| 3.99E+03 | 1.87E-03 |
| 3.99E+03 | 1.69E-03 |
| 4.00E+03 | 1.52E-03 |
| 4.00E+03 | 1.43E-03 |
| 4.00E+03 | 1.38E-03 |
| 4.00E+03 | 0.00E+00 |
