# Supplementary material for: Characterization dataset for pre- and post-irradiated shrimp waste chitosan
Source: Data Brief. 2020 Jul 25;32:106081. doi: 10.1016/j.dib.2020.106081 (PMC7397402; doi:10.1016/j.dib.2020.106081)
Supplement: Supplementary file 1 [file mmc1.zip › TGA-CH0.docx]

| Module | | TG/DTA | | | |  | | | |  | | |  | |  | |  | |  |
| --- | --- | --- | --- | --- | --- | --- | --- | --- | --- | --- | --- | --- | --- | --- | --- | --- | --- | --- | --- |
| Channel | | 1 | | | |  | | | |  | | |  | |  | |  | |  |
| Data Name | | S-Ch-1,(30-03-2017) | | | | | | | |  | | |  | |  | |  | |  |
| Measurement Time | | 3/30/2017 12:51:33 PM | | | | | | | | | | |  | |  | |  | |  |
| Sample Name | | S-Ch-1 | | | |  | | | |  | | |  | |  | |  | |  |
| Sample Weight | | 5.355 | | | | mg | | | |  | | |  | |  | |  | |  |
| Reference Name | | Alumina | | | |  | | | |  | | |  | |  | |  | |  |
| Reference Weight | | 5.5 | | | | mg | | | |  | | |  | |  | |  | |  |
| Temperature Program | | | | | | Cel | | | | Cel | | | Cel/min | | min | | s | | Gas1 |
|  | | 1 | | | | 50 | | | | 650 | | | 10 | | 0 | | 0.5 | | Off |
|  | | Temperature Program Mode | | | | Lamp | | | |  | | |  | |  | |  | |  |
|  |  | |  |  | | |  | |  | | |
|  | | | | |  | | |  | | |  | | |  | |  | |
|  | | | | |  | | |  | | |  | | |  | |  | |
|  | | | | |  | | |  | | |  | | |  | |  | |
|  | | | | | | | |  | | |  | | |  | |  | |
|  | | | | | | | | | | |  | | |  | |  | |
|  | | | | |  | | |  | | |  | | |  | |  | |
|  | |  | | | |  | | | |  | | |  | |  | |  | |  |
|  | |  | | | |  | | | |  | | |  | |  | |  | |  |
| Time | | Temp. | | | | DTA | | | | TG | | | DTG | |  | |  | |  |
| min | | Cel | | | | uV | | | | ug | | | ug/min | |  | |  | |  |
| 0.008333 | | 57.36802 | | | | 0.552556 | | | | 5352.484 | | | 17.19531 | |  | |  | |  |
| 0.016667 | | 57.37029 | | | | 0.553386 | | | | 5352.336 | | | 17.19531 | |  | |  | |  |
| 0.025 | | 57.37029 | | | | 0.554041 | | | | 5352.211 | | | 17.04688 | |  | |  | |  |
| 0.033333 | | 57.37257 | | | | 0.554692 | | | | 5352.039 | | | 17.14844 | |  | |  | |  |
| 0.041667 | | 57.37143 | | | | 0.555121 | | | | 5351.938 | | | 17.0625 | |  | |  | |  |
| 0.05 | | 57.37711 | | | | 0.555772 | | | | 5351.797 | | | 17.13281 | |  | |  | |  |
| 0.058333 | | 57.37711 | | | | 0.556125 | | | | 5351.641 | | | 17.29688 | |  | |  | |  |
| 0.066667 | | 57.38052 | | | | 0.556781 | | | | 5351.492 | | | 17.21094 | |  | |  | |  |
| 0.075 | | 57.38166 | | | | 0.557463 | | | | 5351.359 | | | 17.25781 | |  | |  | |  |
| 0.083333 | | 57.38393 | | | | 0.55822 | | | | 5351.203 | | | 17.36719 | |  | |  | |  |
| 0.091667 | | 57.38734 | | | | 0.558967 | | | | 5351.078 | | | 17.35156 | |  | |  | |  |
| 0.1 | | 57.38734 | | | | 0.55972 | | | | 5350.914 | | | 17.28906 | |  | |  | |  |
| 0.108333 | | 57.39189 | | | | 0.560697 | | | | 5350.75 | | | 17.375 | |  | |  | |  |
| 0.116667 | | 57.39302 | | | | 0.561544 | | | | 5350.602 | | | 17.32813 | |  | |  | |  |
| 0.125 | | 57.39189 | | | | 0.562237 | | | | 5350.461 | | | 17.15625 | |  | |  | |  |
| 0.133333 | | 57.39189 | | | | 0.563128 | | | | 5350.297 | | | 16.99219 | |  | |  | |  |
| 0.141667 | | 57.39302 | | | | 0.563987 | | | | 5350.148 | | | 16.84375 | |  | |  | |  |
| 0.15 | | 57.3953 | | | | 0.564966 | | | | 5350.008 | | | 16.64844 | |  | |  | |  |
| 0.158333 | | 57.39643 | | | | 0.56579 | | | | 5349.852 | | | 16.5625 | |  | |  | |  |
| 0.166667 | | 57.39757 | | | | 0.566566 | | | | 5349.703 | | | 16.625 | |  | |  | |  |
| 0.175 | | 57.39643 | | | | 0.5673 | | | | 5349.578 | | | 16.50781 | |  | |  | |  |
| 0.183333 | | 57.39643 | | | | 0.567977 | | | | 5349.469 | | | 16.57031 | |  | |  | |  |
| 0.191667 | | 57.39416 | | | | 0.568499 | | | | 5349.352 | | | 16.51563 | |  | |  | |  |
| 0.2 | | 57.39757 | | | | 0.569303 | | | | 5349.227 | | | 16.42969 | |  | |  | |  |
| 0.208333 | | 57.40211 | | | | 0.570145 | | | | 5349.109 | | | 16.64063 | |  | |  | |  |
| 0.216667 | | 57.40325 | | | | 0.570797 | | | | 5348.961 | | | 17.09375 | |  | |  | |  |
| 0.225 | | 57.40894 | | | | 0.571828 | | | | 5348.844 | | | 17.13281 | |  | |  | |  |
| 0.233333 | | 57.4078 | | | | 0.572401 | | | | 5348.695 | | | 17.10938 | |  | |  | |  |
| 0.241667 | | 57.4078 | | | | 0.572995 | | | | 5348.563 | | | 17.14063 | |  | |  | |  |
| 0.25 | | 57.41121 | | | | 0.573816 | | | | 5348.43 | | | 16.95313 | |  | |  | |  |
| 0.258333 | | 57.40894 | | | | 0.574241 | | | | 5348.273 | | | 16.84375 | |  | |  | |  |
| 0.266667 | | 57.41121 | | | | 0.575218 | | | | 5348.063 | | | 16.71094 | |  | |  | |  |
| 0.275 | | 57.41575 | | | | 0.576484 | | | | 5347.914 | | | 16.50781 | |  | |  | |  |
| 0.283333 | | 57.41803 | | | | 0.57747 | | | | 5347.773 | | | 16.15625 | |  | |  | |  |
| 0.291667 | | 57.42371 | | | | 0.578911 | | | | 5347.625 | | | 16.02344 | |  | |  | |  |
| 0.3 | | 57.42598 | | | | 0.580052 | | | | 5347.508 | | | 15.49219 | |  | |  | |  |
| 0.308333 | | 57.42485 | | | | 0.580766 | | | | 5347.391 | | | 15.32031 | |  | |  | |  |
| 0.316667 | | 57.42826 | | | | 0.581876 | | | | 5347.273 | | | 15.57031 | |  | |  | |  |
| 0.325 | | 57.43167 | | | | 0.583024 | | | | 5347.172 | | | 15.6875 | |  | |  | |  |
| 0.333333 | | 57.4328 | | | | 0.584044 | | | | 5347.07 | | | 15.61719 | |  | |  | |  |
| 0.341667 | | 57.44076 | | | | 0.585597 | | | | 5346.961 | | | 15.85156 | |  | |  | |  |
| 0.35 | | 57.44303 | | | | 0.586686 | | | | 5346.898 | | | 15.84375 | |  | |  | |  |
| 0.358333 | | 57.4453 | | | | 0.587669 | | | | 5346.797 | | | 16.16406 | |  | |  | |  |
| 0.366667 | | 57.44985 | | | | 0.588699 | | | | 5346.633 | | | 16.28906 | |  | |  | |  |
| 0.375 | | 57.44871 | | | | 0.589176 | | | | 5346.484 | | | 16.59375 | |  | |  | |  |
| 0.383333 | | 57.4544 | | | | 0.59002 | | | | 5346.359 | | | 16.73438 | |  | |  | |  |
| 0.391667 | | 57.45894 | | | | 0.590938 | | | | 5346.195 | | | 16.82813 | |  | |  | |  |
| 0.4 | | 57.46349 | | | | 0.591953 | | | | 5346.063 | | | 16.69531 | |  | |  | |  |
| 0.408333 | | 57.46576 | | | | 0.592782 | | | | 5345.875 | | | 16.48438 | |  | |  | |  |
| 0.416667 | | 57.47031 | | | | 0.59363 | | | | 5345.727 | | | 16.44531 | |  | |  | |  |
| 0.425 | | 57.47485 | | | | 0.594439 | | | | 5345.547 | | | 16.34375 | |  | |  | |  |
| 0.433333 | | 57.48054 | | | | 0.595169 | | | | 5345.391 | | | 16.35156 | |  | |  | |  |
| 0.441667 | | 57.48508 | | | | 0.595892 | | | | 5345.234 | | | 16.08594 | |  | |  | |  |
| 0.45 | | 57.48963 | | | | 0.596879 | | | | 5345.117 | | | 15.97656 | |  | |  | |  |
| 0.458333 | | 57.49417 | | | | 0.597973 | | | | 5345 | | | 16.02344 | |  | |  | |  |
| 0.466667 | | 57.50099 | | | | 0.599224 | | | | 5344.867 | | | 16.125 | |  | |  | |  |
| 0.475 | | 57.50895 | | | | 0.600546 | | | | 5344.742 | | | 16.14844 | |  | |  | |  |
| 0.483333 | | 57.51577 | | | | 0.601579 | | | | 5344.609 | | | 16.02344 | |  | |  | |  |
| 0.491667 | | 57.52713 | | | | 0.603043 | | | | 5344.5 | | | 16.04688 | |  | |  | |  |
| 0.5 | | 57.53509 | | | | 0.604361 | | | | 5344.375 | | | 16.21094 | |  | |  | |  |
| 0.508333 | | 57.53622 | | | | 0.605142 | | | | 5344.242 | | | 16.46094 | |  | |  | |  |
| 0.516667 | | 57.53622 | | | | 0.605762 | | | | 5344.094 | | | 16.42969 | |  | |  | |  |
| 0.525 | | 57.53963 | | | | 0.606421 | | | | 5343.961 | | | 16.40625 | |  | |  | |  |
| 0.533333 | | 57.54646 | | | | 0.607299 | | | | 5343.844 | | | 16.27344 | |  | |  | |  |
| 0.541667 | | 57.55555 | | | | 0.608283 | | | | 5343.703 | | | 16.07813 | |  | |  | |  |
| 0.55 | | 57.56009 | | | | 0.608773 | | | | 5343.547 | | | 15.91406 | |  | |  | |  |
| 0.558333 | | 57.56464 | | | | 0.609164 | | | | 5343.375 | | | 15.94531 | |  | |  | |  |
| 0.566667 | | 57.57259 | | | | 0.609781 | | | | 5343.242 | | | 15.85938 | |  | |  | |  |
| 0.575 | | 57.57487 | | | | 0.610191 | | | | 5343.125 | | | 15.73438 | |  | |  | |  |
| 0.583333 | | 57.58055 | | | | 0.610801 | | | | 5342.992 | | | 15.72656 | |  | |  | |  |
| 0.591667 | | 57.59192 | | | | 0.61185 | | | | 5342.883 | | | 15.36719 | |  | |  | |  |
| 0.6 | | 57.6101 | | | | 0.613254 | | | | 5342.773 | | | 15.48438 | |  | |  | |  |
| 0.608333 | | 57.62146 | | | | 0.614346 | | | | 5342.641 | | | 15.59375 | |  | |  | |  |
| 0.616667 | | 57.62828 | | | | 0.615149 | | | | 5342.508 | | | 15.64063 | |  | |  | |  |
| 0.625 | | 57.63737 | | | | 0.615988 | | | | 5342.391 | | | 15.73438 | |  | |  | |  |
| 0.633333 | | 57.64533 | | | | 0.616962 | | | | 5342.266 | | | 15.60938 | |  | |  | |  |
| 0.641667 | | 57.65556 | | | | 0.618239 | | | | 5342.18 | | | 15.51563 | |  | |  | |  |
| 0.65 | | 57.66238 | | | | 0.619379 | | | | 5342.039 | | | 15.46875 | |  | |  | |  |
| 0.658333 | | 57.67261 | | | | 0.620376 | | | | 5341.898 | | | 15.5625 | |  | |  | |  |
| 0.666667 | | 57.6817 | | | | 0.621258 | | | | 5341.758 | | | 15.42188 | |  | |  | |  |
| 0.675 | | 57.68966 | | | | 0.621952 | | | | 5341.617 | | | 15.49219 | |  | |  | |  |
| 0.683333 | | 57.6942 | | | | 0.622522 | | | | 5341.508 | | | 15.21094 | |  | |  | |  |
| 0.691667 | | 57.70557 | | | | 0.623423 | | | | 5341.391 | | | 15.20313 | |  | |  | |  |
| 0.7 | | 57.71693 | | | | 0.624078 | | | | 5341.273 | | | 14.85156 | |  | |  | |  |
| 0.708333 | | 57.7283 | | | | 0.62486 | | | | 5341.141 | | | 14.88281 | |  | |  | |  |
| 0.716667 | | 57.73966 | | | | 0.625479 | | | | 5341.023 | | | 14.98438 | |  | |  | |  |
| 0.725 | | 57.74875 | | | | 0.625753 | | | | 5340.891 | | | 14.82813 | |  | |  | |  |
| 0.733333 | | 57.75898 | | | | 0.626112 | | | | 5340.797 | | | 14.71094 | |  | |  | |  |
| 0.741667 | | 57.77262 | | | | 0.626756 | | | | 5340.672 | | | 14.61719 | |  | |  | |  |
| 0.75 | | 57.78285 | | | | 0.626909 | | | | 5340.594 | | | 14.5 | |  | |  | |  |
| 0.758333 | | 57.79649 | | | | 0.627123 | | | | 5340.461 | | | 14.47656 | |  | |  | |  |
| 0.766667 | | 57.80899 | | | | 0.626956 | | | | 5340.313 | | | 14.57031 | |  | |  | |  |
| 0.775 | | 57.82035 | | | | 0.62679 | | | | 5340.203 | | | 14.40625 | |  | |  | |  |
| 0.783333 | | 57.83058 | | | | 0.626706 | | | | 5340.094 | | | 14.375 | |  | |  | |  |
| 0.791667 | | 57.84195 | | | | 0.626728 | | | | 5339.977 | | | 14.23438 | |  | |  | |  |
| 0.8 | | 57.85218 | | | | 0.626876 | | | | 5339.883 | | | 13.96875 | |  | |  | |  |
| 0.808333 | | 57.86695 | | | | 0.627265 | | | | 5339.766 | | | 14.02344 | |  | |  | |  |
| 0.816667 | | 57.88173 | | | | 0.627718 | | | | 5339.625 | | | 14.09375 | |  | |  | |  |
| 0.825 | | 57.89082 | | | | 0.628012 | | | | 5339.531 | | | 14.16406 | |  | |  | |  |
| 0.833333 | | 57.90446 | | | | 0.628644 | | | | 5339.414 | | | 14.28906 | |  | |  | |  |
| 0.841667 | | 57.91809 | | | | 0.629039 | | | | 5339.313 | | | 14.28125 | |  | |  | |  |
| 0.85 | | 57.92946 | | | | 0.62911 | | | | 5339.227 | | | 14.38281 | |  | |  | |  |
| 0.858333 | | 57.93855 | | | | 0.628978 | | | | 5339.109 | | | 14.35156 | |  | |  | |  |
| 0.866667 | | 57.95219 | | | | 0.629046 | | | | 5338.977 | | | 14.39844 | |  | |  | |  |
| 0.875 | | 57.96355 | | | | 0.628851 | | | | 5338.844 | | | 14.35938 | |  | |  | |  |
| 0.883333 | | 57.97947 | | | | 0.628751 | | | | 5338.711 | | | 14.48438 | |  | |  | |  |
| 0.891667 | | 57.99424 | | | | 0.628564 | | | | 5338.586 | | | 14.58594 | |  | |  | |  |
| 0.9 | | 58.01015 | | | | 0.628715 | | | | 5338.453 | | | 14.57031 | |  | |  | |  |
| 0.908333 | | 58.02947 | | | | 0.629091 | | | | 5338.328 | | | 14.54688 | |  | |  | |  |
| 0.916667 | | 58.04425 | | | | 0.629103 | | | | 5338.195 | | | 14.42188 | |  | |  | |  |
| 0.925 | | 58.05789 | | | | 0.629139 | | | | 5338.086 | | | 14.34375 | |  | |  | |  |
| 0.933333 | | 58.07607 | | | | 0.629484 | | | | 5337.938 | | | 14.33594 | |  | |  | |  |
| 0.941667 | | 58.09653 | | | | 0.62983 | | | | 5337.805 | | | 14.4375 | |  | |  | |  |
| 0.95 | | 58.11357 | | | | 0.629925 | | | | 5337.688 | | | 14.46875 | |  | |  | |  |
| 0.958333 | | 58.1329 | | | | 0.630269 | | | | 5337.578 | | | 14.60156 | |  | |  | |  |
| 0.966667 | | 58.14881 | | | | 0.630416 | | | | 5337.469 | | | 14.67969 | |  | |  | |  |
| 0.975 | | 58.16358 | | | | 0.630125 | | | | 5337.359 | | | 14.84375 | |  | |  | |  |
| 0.983333 | | 58.17836 | | | | 0.629752 | | | | 5337.242 | | | 14.85156 | |  | |  | |  |
| 0.991667 | | 58.19313 | | | | 0.629295 | | | | 5337.117 | | | 15.10156 | |  | |  | |  |
| 1 | | 58.21018 | | | | 0.628917 | | | | 5336.977 | | | 15.28125 | |  | |  | |  |
| 1.008333 | | 58.2295 | | | | 0.628475 | | | | 5336.844 | | | 15.30469 | |  | |  | |  |
| 1.016667 | | 58.24655 | | | | 0.6281 | | | | 5336.695 | | | 15.45313 | |  | |  | |  |
| 1.025 | | 58.26473 | | | | 0.627864 | | | | 5336.563 | | | 15.70313 | |  | |  | |  |
| 1.033333 | | 58.28379 | | | | 0.627541 | | | | 5336.43 | | | 15.5625 | |  | |  | |  |
| 1.041667 | | 58.29914 | | | | 0.626895 | | | | 5336.281 | | | 15.58594 | |  | |  | |  |
| 1.05 | | 58.31889 | | | | 0.626475 | | | | 5336.109 | | | 15.53906 | |  | |  | |  |
| 1.058333 | | 58.33753 | | | | 0.626134 | | | | 5335.984 | | | 15.5 | |  | |  | |  |
| 1.066667 | | 58.35618 | | | | 0.625793 | | | | 5335.836 | | | 15.52344 | |  | |  | |  |
| 1.075 | | 58.37811 | | | | 0.625503 | | | | 5335.68 | | | 15.61719 | |  | |  | |  |
| 1.083333 | | 58.40224 | | | | 0.625028 | | | | 5335.563 | | | 15.36719 | |  | |  | |  |
| 1.091667 | | 58.42089 | | | | 0.624188 | | | | 5335.43 | | | 15.29688 | |  | |  | |  |
| 1.1 | | 58.44063 | | | | 0.623358 | | | | 5335.32 | | | 15.38281 | |  | |  | |  |
| 1.108333 | | 58.46147 | | | | 0.622708 | | | | 5335.195 | | | 15.28125 | |  | |  | |  |
| 1.116667 | | 58.48121 | | | | 0.622289 | | | | 5335.055 | | | 15.35156 | |  | |  | |  |
| 1.125 | | 58.50205 | | | | 0.621938 | | | | 5334.914 | | | 15.46094 | |  | |  | |  |
| 1.133333 | | 58.5196 | | | | 0.620779 | | | | 5334.82 | | | 15.57813 | |  | |  | |  |
| 1.141667 | | 58.54044 | | | | 0.619692 | | | | 5334.703 | | | 15.35156 | |  | |  | |  |
| 1.15 | | 58.56237 | | | | 0.618951 | | | | 5334.57 | | | 15.39063 | |  | |  | |  |
| 1.158333 | | 58.57883 | | | | 0.618262 | | | | 5334.461 | | | 15.28906 | |  | |  | |  |
| 1.166667 | | 58.59528 | | | | 0.617543 | | | | 5334.32 | | | 15.21094 | |  | |  | |  |
| 1.175 | | 58.61393 | | | | 0.616747 | | | | 5334.18 | | | 15.33594 | |  | |  | |  |
| 1.183333 | | 58.63586 | | | | 0.615742 | | | | 5334.039 | | | 15.35156 | |  | |  | |  |
| 1.191667 | | 58.6567 | | | | 0.614623 | | | | 5333.945 | | | 15.21094 | |  | |  | |  |
| 1.2 | | 58.67535 | | | | 0.613421 | | | | 5333.813 | | | 15.23438 | |  | |  | |  |
| 1.208333 | | 58.69838 | | | | 0.612557 | | | | 5333.711 | | | 15.00781 | |  | |  | |  |
| 1.216667 | | 58.72361 | | | | 0.611994 | | | | 5333.586 | | | 15.02344 | |  | |  | |  |
| 1.225 | | 58.74664 | | | | 0.610989 | | | | 5333.445 | | | 15.22656 | |  | |  | |  |
| 1.233333 | | 58.76967 | | | | 0.610055 | | | | 5333.313 | | | 15.16406 | |  | |  | |  |
| 1.241667 | | 58.79819 | | | | 0.609438 | | | | 5333.203 | | | 15.41406 | |  | |  | |  |
| 1.25 | | 58.82451 | | | | 0.609162 | | | | 5333.078 | | | 15.35938 | |  | |  | |  |
| 1.258333 | | 58.85413 | | | | 0.608679 | | | | 5332.977 | | | 15.51563 | |  | |  | |  |
| 1.266667 | | 58.87935 | | | | 0.607955 | | | | 5332.844 | | | 15.45313 | |  | |  | |  |
| 1.275 | | 58.90129 | | | | 0.607194 | | | | 5332.695 | | | 15.54688 | |  | |  | |  |
| 1.283333 | | 58.9298 | | | | 0.606832 | | | | 5332.57 | | | 15.80469 | |  | |  | |  |
| 1.291667 | | 58.95284 | | | | 0.605918 | | | | 5332.414 | | | 16.03125 | |  | |  | |  |
| 1.3 | | 58.97806 | | | | 0.604763 | | | | 5332.289 | | | 16.13281 | |  | |  | |  |
| 1.308333 | | 59.00109 | | | | 0.603332 | | | | 5332.141 | | | 16.17969 | |  | |  | |  |
| 1.316667 | | 59.02523 | | | | 0.601826 | | | | 5332.023 | | | 16.20313 | |  | |  | |  |
| 1.325 | | 59.04826 | | | | 0.600463 | | | | 5331.875 | | | 16.14844 | |  | |  | |  |
| 1.333333 | | 59.07239 | | | | 0.599021 | | | | 5331.719 | | | 16.17969 | |  | |  | |  |
| 1.341667 | | 59.09652 | | | | 0.597455 | | | | 5331.547 | | | 16.19531 | |  | |  | |  |
| 1.35 | | 59.12613 | | | | 0.59624 | | | | 5331.398 | | | 16.25 | |  | |  | |  |
| 1.358333 | | 59.15575 | | | | 0.595337 | | | | 5331.266 | | | 16.19531 | |  | |  | |  |
| 1.366667 | | 59.17987 | | | | 0.593917 | | | | 5331.125 | | | 16.15625 | |  | |  | |  |
| 1.375 | | 59.20729 | | | | 0.59243 | | | | 5330.992 | | | 16.00781 | |  | |  | |  |
| 1.383333 | | 59.23033 | | | | 0.590783 | | | | 5330.852 | | | 16.00781 | |  | |  | |  |
| 1.391667 | | 59.25775 | | | | 0.589516 | | | | 5330.719 | | | 16.15625 | |  | |  | |  |
| 1.4 | | 59.28517 | | | | 0.588353 | | | | 5330.57 | | | 16.375 | |  | |  | |  |
| 1.408333 | | 59.31478 | | | | 0.586795 | | | | 5330.445 | | | 16.25781 | |  | |  | |  |
| 1.416667 | | 59.3411 | | | | 0.585118 | | | | 5330.305 | | | 16.53906 | |  | |  | |  |
| 1.425 | | 59.36962 | | | | 0.583462 | | | | 5330.195 | | | 16.6875 | |  | |  | |  |
| 1.433333 | | 59.39265 | | | | 0.581672 | | | | 5330.063 | | | 16.69531 | |  | |  | |  |
| 1.441667 | | 59.41678 | | | | 0.579624 | | | | 5329.906 | | | 16.82031 | |  | |  | |  |
| 1.45 | | 59.4453 | | | | 0.57749 | | | | 5329.742 | | | 16.83594 | |  | |  | |  |
| 1.458333 | | 59.47601 | | | | 0.575687 | | | | 5329.617 | | | 16.92188 | |  | |  | |  |
| 1.466667 | | 59.51111 | | | | 0.574447 | | | | 5329.445 | | | 17.02344 | |  | |  | |  |
| 1.475 | | 59.54292 | | | | 0.572948 | | | | 5329.289 | | | 17.1875 | |  | |  | |  |
| 1.483333 | | 59.57363 | | | | 0.571203 | | | | 5329.148 | | | 17.11719 | |  | |  | |  |
| 1.491667 | | 59.59995 | | | | 0.569538 | | | | 5328.992 | | | 17.375 | |  | |  | |  |
| 1.5 | | 59.62956 | | | | 0.568123 | | | | 5328.852 | | | 17.46094 | |  | |  | |  |
| 1.508333 | | 59.66027 | | | | 0.566478 | | | | 5328.703 | | | 17.51563 | |  | |  | |  |
| 1.516667 | | 59.68989 | | | | 0.564733 | | | | 5328.547 | | | 17.63281 | |  | |  | |  |
| 1.525 | | 59.72389 | | | | 0.563609 | | | | 5328.383 | | | 17.59375 | |  | |  | |  |
| 1.533333 | | 59.7557 | | | | 0.562399 | | | | 5328.242 | | | 17.95313 | |  | |  | |  |
| 1.541667 | | 59.7864 | | | | 0.56041 | | | | 5328.078 | | | 18.0625 | |  | |  | |  |
| 1.55 | | 59.81712 | | | | 0.558513 | | | | 5327.922 | | | 17.9375 | |  | |  | |  |
| 1.558333 | | 59.85002 | | | | 0.556973 | | | | 5327.773 | | | 17.92969 | |  | |  | |  |
| 1.566667 | | 59.88512 | | | | 0.555467 | | | | 5327.609 | | | 17.8125 | |  | |  | |  |
| 1.575 | | 59.91802 | | | | 0.553118 | | | | 5327.461 | | | 17.75 | |  | |  | |  |
| 1.583333 | | 59.95312 | | | | 0.55116 | | | | 5327.273 | | | 17.88281 | |  | |  | |  |
| 1.591667 | | 59.98383 | | | | 0.549274 | | | | 5327.109 | | | 17.77344 | |  | |  | |  |
| 1.6 | | 60.01892 | | | | 0.547018 | | | | 5326.977 | | | 17.83594 | |  | |  | |  |
| 1.608333 | | 60.05183 | | | | 0.544927 | | | | 5326.836 | | | 17.98438 | |  | |  | |  |
| 1.616667 | | 60.08693 | | | | 0.542927 | | | | 5326.703 | | | 17.90625 | |  | |  | |  |
| 1.625 | | 60.12202 | | | | 0.54064 | | | | 5326.563 | | | 18 | |  | |  | |  |
| 1.633333 | | 60.15274 | | | | 0.538332 | | | | 5326.406 | | | 18.16406 | |  | |  | |  |
| 1.641667 | | 60.18125 | | | | 0.536221 | | | | 5326.266 | | | 18.14063 | |  | |  | |  |
| 1.65 | | 60.20977 | | | | 0.534105 | | | | 5326.117 | | | 18.44531 | |  | |  | |  |
| 1.658333 | | 60.24487 | | | | 0.531807 | | | | 5325.945 | | | 18.78906 | |  | |  | |  |
| 1.666667 | | 60.27777 | | | | 0.529887 | | | | 5325.805 | | | 18.83594 | |  | |  | |  |
| 1.675 | | 60.31068 | | | | 0.528153 | | | | 5325.641 | | | 19.03906 | |  | |  | |  |
| 1.683333 | | 60.34358 | | | | 0.52566 | | | | 5325.469 | | | 19.125 | |  | |  | |  |
| 1.691667 | | 60.37539 | | | | 0.523171 | | | | 5325.32 | | | 19.19531 | |  | |  | |  |
| 1.7 | | 60.40719 | | | | 0.520915 | | | | 5325.117 | | | 19.23438 | |  | |  | |  |
| 1.708333 | | 60.439 | | | | 0.518672 | | | | 5324.922 | | | 19.14844 | |  | |  | |  |
| 1.716667 | | 60.47629 | | | | 0.5161 | | | | 5324.758 | | | 19.17188 | |  | |  | |  |
| 1.725 | | 60.51578 | | | | 0.514214 | | | | 5324.57 | | | 19.38281 | |  | |  | |  |
| 1.733333 | | 60.55307 | | | | 0.511814 | | | | 5324.406 | | | 19.03906 | |  | |  | |  |
| 1.741667 | | 60.58707 | | | | 0.50882 | | | | 5324.234 | | | 19.03906 | |  | |  | |  |
| 1.75 | | 60.62655 | | | | 0.506652 | | | | 5324.07 | | | 18.94531 | |  | |  | |  |
| 1.758333 | | 60.66055 | | | | 0.504153 | | | | 5323.93 | | | 19 | |  | |  | |  |
| 1.766667 | | 60.69675 | | | | 0.501254 | | | | 5323.766 | | | 19.09375 | |  | |  | |  |
| 1.775 | | 60.73404 | | | | 0.499425 | | | | 5323.586 | | | 19.08594 | |  | |  | |  |
| 1.783333 | | 60.77023 | | | | 0.497383 | | | | 5323.469 | | | 19.21094 | |  | |  | |  |
| 1.791667 | | 60.80533 | | | | 0.49444 | | | | 5323.32 | | | 19.39063 | |  | |  | |  |
| 1.8 | | 60.83824 | | | | 0.491965 | | | | 5323.172 | | | 19.40625 | |  | |  | |  |
| 1.808333 | | 60.87114 | | | | 0.48927 | | | | 5323.016 | | | 19.6875 | |  | |  | |  |
| 1.816667 | | 60.90843 | | | | 0.486461 | | | | 5322.836 | | | 20.16406 | |  | |  | |  |
| 1.825 | | 60.94243 | | | | 0.483622 | | | | 5322.68 | | | 20.27344 | |  | |  | |  |
| 1.833333 | | 60.97863 | | | | 0.481115 | | | | 5322.508 | | | 20.51563 | |  | |  | |  |
| 1.841667 | | 61.01701 | | | | 0.478427 | | | | 5322.32 | | | 20.64844 | |  | |  | |  |
| 1.85 | | 61.0554 | | | | 0.475644 | | | | 5322.156 | | | 20.75781 | |  | |  | |  |
| 1.858333 | | 61.0916 | | | | 0.473026 | | | | 5321.953 | | | 20.78125 | |  | |  | |  |
| 1.866667 | | 61.12779 | | | | 0.470123 | | | | 5321.727 | | | 20.86719 | |  | |  | |  |
| 1.875 | | 61.16618 | | | | 0.466955 | | | | 5321.539 | | | 21.125 | |  | |  | |  |
| 1.883333 | | 61.20567 | | | | 0.4648 | | | | 5321.328 | | | 21.41406 | |  | |  | |  |
| 1.891667 | | 61.24295 | | | | 0.462372 | | | | 5321.141 | | | 21.19531 | |  | |  | |  |
| 1.9 | | 61.28025 | | | | 0.45956 | | | | 5320.961 | | | 21.375 | |  | |  | |  |
| 1.908333 | | 61.32302 | | | | 0.457422 | | | | 5320.781 | | | 21.42188 | |  | |  | |  |
| 1.916667 | | 61.35812 | | | | 0.454409 | | | | 5320.594 | | | 21.625 | |  | |  | |  |
| 1.925 | | 61.3998 | | | | 0.451604 | | | | 5320.383 | | | 21.64063 | |  | |  | |  |
| 1.933333 | | 61.43709 | | | | 0.44905 | | | | 5320.164 | | | 21.625 | |  | |  | |  |
| 1.941667 | | 61.48096 | | | | 0.446366 | | | | 5320.016 | | | 21.75781 | |  | |  | |  |
| 1.95 | | 61.52264 | | | | 0.443428 | | | | 5319.82 | | | 21.85156 | |  | |  | |  |
| 1.958333 | | 61.56541 | | | | 0.440855 | | | | 5319.648 | | | 21.82813 | |  | |  | |  |
| 1.966667 | | 61.6038 | | | | 0.437182 | | | | 5319.438 | | | 21.75 | |  | |  | |  |
| 1.975 | | 61.64658 | | | | 0.433621 | | | | 5319.266 | | | 21.89063 | |  | |  | |  |
| 1.983333 | | 61.68716 | | | | 0.430458 | | | | 5319.086 | | | 21.94531 | |  | |  | |  |
| 1.991667 | | 61.72774 | | | | 0.427383 | | | | 5318.883 | | | 21.92188 | |  | |  | |  |
| 2 | | 61.76942 | | | | 0.425139 | | | | 5318.695 | | | 21.67969 | |  | |  | |  |
| 2.008333 | | 61.81219 | | | | 0.422492 | | | | 5318.523 | | | 21.8125 | |  | |  | |  |
| 2.016667 | | 61.85716 | | | | 0.41952 | | | | 5318.359 | | | 21.84375 | |  | |  | |  |
| 2.025 | | 61.89665 | | | | 0.417396 | | | | 5318.164 | | | 21.91406 | |  | |  | |  |
| 2.033333 | | 61.93833 | | | | 0.414783 | | | | 5317.961 | | | 21.9375 | |  | |  | |  |
| 2.041667 | | 61.97781 | | | | 0.411733 | | | | 5317.789 | | | 21.95313 | |  | |  | |  |
| 2.05 | | 62.01839 | | | | 0.409396 | | | | 5317.641 | | | 22.125 | |  | |  | |  |
| 2.058333 | | 62.05898 | | | | 0.406845 | | | | 5317.438 | | | 22.21094 | |  | |  | |  |
| 2.066667 | | 62.10065 | | | | 0.404106 | | | | 5317.25 | | | 22.24219 | |  | |  | |  |
| 2.075 | | 62.14453 | | | | 0.401772 | | | | 5317.063 | | | 22.51563 | |  | |  | |  |
| 2.083333 | | 62.19278 | | | | 0.399061 | | | | 5316.875 | | | 22.72656 | |  | |  | |  |
| 2.091667 | | 62.23446 | | | | 0.396219 | | | | 5316.695 | | | 22.86719 | |  | |  | |  |
| 2.1 | | 62.27614 | | | | 0.392921 | | | | 5316.492 | | | 23.17188 | |  | |  | |  |
| 2.108333 | | 62.31782 | | | | 0.389228 | | | | 5316.297 | | | 23.33594 | |  | |  | |  |
| 2.116667 | | 62.36279 | | | | 0.38651 | | | | 5316.102 | | | 23.55469 | |  | |  | |  |
| 2.125 | | 62.40995 | | | | 0.383446 | | | | 5315.875 | | | 23.73438 | |  | |  | |  |
| 2.133333 | | 62.45602 | | | | 0.380304 | | | | 5315.664 | | | 24.11719 | |  | |  | |  |
| 2.141667 | | 62.50208 | | | | 0.377452 | | | | 5315.461 | | | 24.24219 | |  | |  | |  |
| 2.15 | | 62.54815 | | | | 0.374068 | | | | 5315.234 | | | 24.36719 | |  | |  | |  |
| 2.158333 | | 62.59092 | | | | 0.371313 | | | | 5315.023 | | | 24.52344 | |  | |  | |  |
| 2.166667 | | 62.6326 | | | | 0.367961 | | | | 5314.797 | | | 24.82813 | |  | |  | |  |
| 2.175 | | 62.67867 | | | | 0.365031 | | | | 5314.57 | | | 25.11719 | |  | |  | |  |
| 2.183333 | | 62.72583 | | | | 0.363099 | | | | 5314.328 | | | 25.33594 | |  | |  | |  |
| 2.191667 | | 62.77518 | | | | 0.360498 | | | | 5314.102 | | | 25.70313 | |  | |  | |  |
| 2.2 | | 62.82016 | | | | 0.357575 | | | | 5313.883 | | | 26.01563 | |  | |  | |  |
| 2.208333 | | 62.86293 | | | | 0.354046 | | | | 5313.656 | | | 26.21875 | |  | |  | |  |
| 2.216667 | | 62.909 | | | | 0.350817 | | | | 5313.414 | | | 26.44531 | |  | |  | |  |
| 2.225 | | 62.95506 | | | | 0.347644 | | | | 5313.164 | | | 26.58594 | |  | |  | |  |
| 2.233333 | | 62.99893 | | | | 0.343678 | | | | 5312.914 | | | 26.63281 | |  | |  | |  |
| 2.241667 | | 63.0439 | | | | 0.340138 | | | | 5312.648 | | | 26.85938 | |  | |  | |  |
| 2.25 | | 63.08997 | | | | 0.336474 | | | | 5312.391 | | | 27.07813 | |  | |  | |  |
| 2.258333 | | 63.13274 | | | | 0.332043 | | | | 5312.148 | | | 27.125 | |  | |  | |  |
| 2.266667 | | 63.17661 | | | | 0.328509 | | | | 5311.906 | | | 27.1875 | |  | |  | |  |
| 2.275 | | 63.22378 | | | | 0.32492 | | | | 5311.664 | | | 27.49219 | |  | |  | |  |
| 2.283333 | | 63.27313 | | | | 0.322173 | | | | 5311.43 | | | 27.49219 | |  | |  | |  |
| 2.291667 | | 63.32139 | | | | 0.319595 | | | | 5311.195 | | | 27.60938 | |  | |  | |  |
| 2.3 | | 63.37075 | | | | 0.3164 | | | | 5310.938 | | | 27.8125 | |  | |  | |  |
| 2.308333 | | 63.4212 | | | | 0.314071 | | | | 5310.711 | | | 27.70313 | |  | |  | |  |
| 2.316667 | | 63.47165 | | | | 0.311071 | | | | 5310.469 | | | 27.71875 | |  | |  | |  |
| 2.325 | | 63.52101 | | | | 0.308542 | | | | 5310.211 | | | 27.72656 | |  | |  | |  |
| 2.333333 | | 63.56927 | | | | 0.305432 | | | | 5309.984 | | | 27.71094 | |  | |  | |  |
| 2.341667 | | 63.61863 | | | | 0.302351 | | | | 5309.734 | | | 27.74219 | |  | |  | |  |
| 2.35 | | 63.66908 | | | | 0.298974 | | | | 5309.484 | | | 27.85156 | |  | |  | |  |
| 2.358333 | | 63.71953 | | | | 0.295111 | | | | 5309.273 | | | 27.51563 | |  | |  | |  |
| 2.366667 | | 63.76889 | | | | 0.292439 | | | | 5309.055 | | | 27.44531 | |  | |  | |  |
| 2.375 | | 63.81934 | | | | 0.288698 | | | | 5308.82 | | | 27.40625 | |  | |  | |  |
| 2.383333 | | 63.8676 | | | | 0.285189 | | | | 5308.602 | | | 27.28125 | |  | |  | |  |
| 2.391667 | | 63.91695 | | | | 0.282088 | | | | 5308.367 | | | 27.63281 | |  | |  | |  |
| 2.4 | | 63.96631 | | | | 0.278818 | | | | 5308.133 | | | 27.75 | |  | |  | |  |
| 2.408333 | | 64.01567 | | | | 0.276101 | | | | 5307.953 | | | 27.94531 | |  | |  | |  |
| 2.416667 | | 64.06503 | | | | 0.272601 | | | | 5307.734 | | | 27.82031 | |  | |  | |  |
| 2.425 | | 64.11767 | | | | 0.270037 | | | | 5307.523 | | | 28.05469 | |  | |  | |  |
| 2.433333 | | 64.16812 | | | | 0.266666 | | | | 5307.32 | | | 28.1875 | |  | |  | |  |
| 2.441667 | | 64.212 | | | | 0.263364 | | | | 5307.047 | | | 28.46094 | |  | |  | |  |
| 2.45 | | 64.26135 | | | | 0.259981 | | | | 5306.797 | | | 28.69531 | |  | |  | |  |
| 2.458333 | | 64.31071 | | | | 0.256142 | | | | 5306.547 | | | 28.80469 | |  | |  | |  |
| 2.466667 | | 64.36335 | | | | 0.253307 | | | | 5306.328 | | | 28.85156 | |  | |  | |  |
| 2.475 | | 64.4138 | | | | 0.249507 | | | | 5306.063 | | | 28.79688 | |  | |  | |  |
| 2.483333 | | 64.46426 | | | | 0.246563 | | | | 5305.82 | | | 28.97656 | |  | |  | |  |
| 2.491667 | | 64.51361 | | | | 0.242788 | | | | 5305.539 | | | 29.11719 | |  | |  | |  |
| 2.5 | | 64.56406 | | | | 0.239098 | | | | 5305.281 | | | 29.30469 | |  | |  | |  |
| 2.508333 | | 64.61452 | | | | 0.235612 | | | | 5305.023 | | | 29.36719 | |  | |  | |  |
| 2.516667 | | 64.66278 | | | | 0.231983 | | | | 5304.773 | | | 29.49219 | |  | |  | |  |
| 2.525 | | 64.71872 | | | | 0.22955 | | | | 5304.539 | | | 29.45313 | |  | |  | |  |
| 2.533333 | | 64.76917 | | | | 0.225921 | | | | 5304.273 | | | 29.64063 | |  | |  | |  |
| 2.541667 | | 64.82072 | | | | 0.223322 | | | | 5304.016 | | | 29.71875 | |  | |  | |  |
| 2.55 | | 64.87007 | | | | 0.219342 | | | | 5303.75 | | | 29.83594 | |  | |  | |  |
| 2.558333 | | 64.91833 | | | | 0.215292 | | | | 5303.484 | | | 30.08594 | |  | |  | |  |
| 2.566667 | | 64.97098 | | | | 0.211687 | | | | 5303.234 | | | 30.39063 | |  | |  | |  |
| 2.575 | | 65.02363 | | | | 0.20778 | | | | 5302.992 | | | 30.33594 | |  | |  | |  |
| 2.583333 | | 65.07957 | | | | 0.20489 | | | | 5302.719 | | | 30.69531 | |  | |  | |  |
| 2.591667 | | 65.13221 | | | | 0.201541 | | | | 5302.461 | | | 30.64063 | |  | |  | |  |
| 2.6 | | 65.18925 | | | | 0.198871 | | | | 5302.188 | | | 30.71875 | |  | |  | |  |
| 2.608333 | | 65.24079 | | | | 0.194869 | | | | 5301.906 | | | 30.875 | |  | |  | |  |
| 2.616667 | | 65.29563 | | | | 0.191813 | | | | 5301.609 | | | 30.8125 | |  | |  | |  |
| 2.625 | | 65.34718 | | | | 0.187652 | | | | 5301.359 | | | 30.9375 | |  | |  | |  |
| 2.633333 | | 65.40202 | | | | 0.184678 | | | | 5301.063 | | | 31.1875 | |  | |  | |  |
| 2.641667 | | 65.45905 | | | | 0.18113 | | | | 5300.813 | | | 31.22656 | |  | |  | |  |
| 2.65 | | 65.5128 | | | | 0.178182 | | | | 5300.547 | | | 31.08594 | |  | |  | |  |
| 2.658333 | | 65.56654 | | | | 0.174713 | | | | 5300.273 | | | 31.28906 | |  | |  | |  |
| 2.666667 | | 65.6181 | | | | 0.171354 | | | | 5300.023 | | | 31.23438 | |  | |  | |  |
| 2.675 | | 65.67294 | | | | 0.168178 | | | | 5299.75 | | | 31.41406 | |  | |  | |  |
| 2.683333 | | 65.72668 | | | | 0.16486 | | | | 5299.461 | | | 31.50781 | |  | |  | |  |
| 2.691667 | | 65.78152 | | | | 0.16175 | | | | 5299.195 | | | 31.5625 | |  | |  | |  |
| 2.7 | | 65.83636 | | | | 0.158162 | | | | 5298.953 | | | 31.8125 | |  | |  | |  |
| 2.708333 | | 65.8923 | | | | 0.154818 | | | | 5298.68 | | | 31.9375 | |  | |  | |  |
| 2.716667 | | 65.94823 | | | | 0.150688 | | | | 5298.422 | | | 31.94531 | |  | |  | |  |
| 2.725 | | 66.00745 | | | | 0.147835 | | | | 5298.141 | | | 31.94531 | |  | |  | |  |
| 2.733333 | | 66.06339 | | | | 0.1438 | | | | 5297.867 | | | 32.17969 | |  | |  | |  |
| 2.741667 | | 66.12043 | | | | 0.140792 | | | | 5297.594 | | | 32.28125 | |  | |  | |  |
| 2.75 | | 66.17527 | | | | 0.136847 | | | | 5297.305 | | | 32.45313 | |  | |  | |  |
| 2.758333 | | 66.22681 | | | | 0.133673 | | | | 5297.031 | | | 32.69531 | |  | |  | |  |
| 2.766667 | | 66.28385 | | | | 0.130258 | | | | 5296.773 | | | 32.875 | |  | |  | |  |
| 2.775 | | 66.33979 | | | | 0.127499 | | | | 5296.508 | | | 32.84375 | |  | |  | |  |
| 2.783333 | | 66.39353 | | | | 0.124221 | | | | 5296.219 | | | 32.85156 | |  | |  | |  |
| 2.791667 | | 66.45056 | | | | 0.12128 | | | | 5295.93 | | | 32.82813 | |  | |  | |  |
| 2.8 | | 66.5087 | | | | 0.11817 | | | | 5295.641 | | | 33 | |  | |  | |  |
| 2.808333 | | 66.56244 | | | | 0.114662 | | | | 5295.328 | | | 33.21094 | |  | |  | |  |
| 2.816667 | | 66.62057 | | | | 0.11104 | | | | 5295.039 | | | 33.27344 | |  | |  | |  |
| 2.825 | | 66.6787 | | | | 0.107253 | | | | 5294.773 | | | 33.39844 | |  | |  | |  |
| 2.833333 | | 66.73683 | | | | 0.103543 | | | | 5294.5 | | | 33.54688 | |  | |  | |  |
| 2.841667 | | 66.80045 | | | | 0.099441 | | | | 5294.219 | | | 33.50781 | |  | |  | |  |
| 2.85 | | 66.85309 | | | | 0.095683 | | | | 5293.938 | | | 33.78125 | |  | |  | |  |
| 2.858333 | | 66.90793 | | | | 0.09195 | | | | 5293.625 | | | 34.03906 | |  | |  | |  |
| 2.866667 | | 66.96606 | | | | 0.089459 | | | | 5293.344 | | | 34.35156 | |  | |  | |  |
| 2.875 | | 67.02748 | | | | 0.086509 | | | | 5293.055 | | | 34.57031 | |  | |  | |  |
| 2.883333 | | 67.08232 | | | | 0.083089 | | | | 5292.75 | | | 34.69531 | |  | |  | |  |
| 2.891667 | | 67.13935 | | | | 0.079406 | | | | 5292.484 | | | 34.80469 | |  | |  | |  |
| 2.9 | | 67.19639 | | | | 0.076483 | | | | 5292.164 | | | 35 | |  | |  | |  |
| 2.908333 | | 67.25342 | | | | 0.072742 | | | | 5291.844 | | | 35.28906 | |  | |  | |  |
| 2.916667 | | 67.31594 | | | | 0.069807 | | | | 5291.523 | | | 35.30469 | |  | |  | |  |
| 2.925 | | 67.37078 | | | | 0.065583 | | | | 5291.203 | | | 35.39844 | |  | |  | |  |
| 2.933333 | | 67.43111 | | | | 0.062483 | | | | 5290.898 | | | 35.28125 | |  | |  | |  |
| 2.941667 | | 67.49142 | | | | 0.059113 | | | | 5290.602 | | | 35.41406 | |  | |  | |  |
| 2.95 | | 67.55175 | | | | 0.056375 | | | | 5290.273 | | | 35.50781 | |  | |  | |  |
| 2.958333 | | 67.60769 | | | | 0.053116 | | | | 5289.945 | | | 35.51563 | |  | |  | |  |
| 2.966667 | | 67.66692 | | | | 0.050185 | | | | 5289.648 | | | 35.625 | |  | |  | |  |
| 2.975 | | 67.72395 | | | | 0.04646 | | | | 5289.352 | | | 35.75 | |  | |  | |  |
| 2.983333 | | 67.78427 | | | | 0.043027 | | | | 5289.07 | | | 35.84375 | |  | |  | |  |
| 2.991667 | | 67.84241 | | | | 0.038868 | | | | 5288.758 | | | 35.625 | |  | |  | |  |
| 3 | | 67.89615 | | | | 0.03495 | | | | 5288.453 | | | 35.65625 | |  | |  | |  |
| 3.008333 | | 67.95647 | | | | 0.030766 | | | | 5288.156 | | | 35.77344 | |  | |  | |  |
| 3.016667 | | 68.02009 | | | | 0.027565 | | | | 5287.852 | | | 36.07813 | |  | |  | |  |
| 3.025 | | 68.0815 | | | | 0.023377 | | | | 5287.547 | | | 36.13281 | |  | |  | |  |
| 3.033333 | | 68.14073 | | | | 0.019904 | | | | 5287.234 | | | 36.23438 | |  | |  | |  |
| 3.041667 | | 68.20216 | | | | 0.015948 | | | | 5286.969 | | | 36.26563 | |  | |  | |  |
| 3.05 | | 68.26357 | | | | 0.012738 | | | | 5286.664 | | | 36.34375 | |  | |  | |  |
| 3.058333 | | 68.32719 | | | | 0.009125 | | | | 5286.352 | | | 36.4375 | |  | |  | |  |
| 3.066667 | | 68.38641 | | | | 0.0059 | | | | 5286.023 | | | 36.64063 | |  | |  | |  |
| 3.075 | | 68.44784 | | | | 0.002721 | | | | 5285.711 | | | 36.83594 | |  | |  | |  |
| 3.083333 | | 68.51365 | | | | 0.00045 | | | | 5285.398 | | | 36.875 | |  | |  | |  |
| 3.091667 | | 68.57616 | | | | -0.00192 | | | | 5285.094 | | | 37.01563 | |  | |  | |  |
| 3.1 | | 68.63429 | | | | -0.00476 | | | | 5284.781 | | | 37.14844 | |  | |  | |  |
| 3.108333 | | 68.69572 | | | | -0.00706 | | | | 5284.469 | | | 37.13281 | |  | |  | |  |
| 3.116667 | | 68.75494 | | | | -0.01044 | | | | 5284.133 | | | 37.20313 | |  | |  | |  |
| 3.125 | | 68.81526 | | | | -0.01293 | | | | 5283.82 | | | 37.64063 | |  | |  | |  |
| 3.133333 | | 68.87669 | | | | -0.01648 | | | | 5283.5 | | | 37.79688 | |  | |  | |  |
| 3.141667 | | 68.93481 | | | | -0.01957 | | | | 5283.18 | | | 38.01563 | |  | |  | |  |
| 3.15 | | 68.99624 | | | | -0.02346 | | | | 5282.852 | | | 37.9375 | |  | |  | |  |
| 3.158333 | | 69.06095 | | | | -0.02643 | | | | 5282.539 | | | 38.28125 | |  | |  | |  |
| 3.166667 | | 69.12127 | | | | -0.03032 | | | | 5282.219 | | | 38.32031 | |  | |  | |  |
| 3.175 | | 69.18159 | | | | -0.03377 | | | | 5281.859 | | | 38.84375 | |  | |  | |  |
| 3.183333 | | 69.24412 | | | | -0.03806 | | | | 5281.516 | | | 38.83594 | |  | |  | |  |
| 3.191667 | | 69.30663 | | | | -0.04135 | | | | 5281.18 | | | 39.09375 | |  | |  | |  |
| 3.2 | | 69.37025 | | | | -0.04578 | | | | 5280.867 | | | 39.46094 | |  | |  | |  |
| 3.208333 | | 69.43276 | | | | -0.04904 | | | | 5280.508 | | | 39.375 | |  | |  | |  |
| 3.216667 | | 69.49529 | | | | -0.0535 | | | | 5280.188 | | | 39.64063 | |  | |  | |  |
| 3.225 | | 69.5589 | | | | -0.05691 | | | | 5279.789 | | | 39.85156 | |  | |  | |  |
| 3.233333 | | 69.61922 | | | | -0.06093 | | | | 5279.477 | | | 40.07031 | |  | |  | |  |
| 3.241667 | | 69.68064 | | | | -0.06366 | | | | 5279.109 | | | 40 | |  | |  | |  |
| 3.25 | | 69.73987 | | | | -0.06737 | | | | 5278.742 | | | 40.25 | |  | |  | |  |
| 3.258333 | | 69.80019 | | | | -0.07009 | | | | 5278.414 | | | 39.83594 | |  | |  | |  |
| 3.266667 | | 69.86271 | | | | -0.07398 | | | | 5278.055 | | | 40.15625 | |  | |  | |  |
| 3.275 | | 69.92194 | | | | -0.07691 | | | | 5277.695 | | | 40.33594 | |  | |  | |  |
| 3.283333 | | 69.98665 | | | | -0.0801 | | | | 5277.336 | | | 40.40625 | |  | |  | |  |
| 3.291667 | | 70.04916 | | | | -0.08282 | | | | 5277.016 | | | 40.51563 | |  | |  | |  |
| 3.3 | | 70.11059 | | | | -0.0866 | | | | 5276.641 | | | 40.57813 | |  | |  | |  |
| 3.308333 | | 70.1742 | | | | -0.08986 | | | | 5276.367 | | | 40.67188 | |  | |  | |  |
| 3.316667 | | 70.2411 | | | | -0.09308 | | | | 5275.992 | | | 40.71094 | |  | |  | |  |
| 3.325 | | 70.30536 | | | | -0.09653 | | | | 5275.633 | | | 40.84375 | |  | |  | |  |
| 3.333333 | | 70.36878 | | | | -0.10002 | | | | 5275.297 | | | 40.83594 | |  | |  | |  |
| 3.341667 | | 70.43115 | | | | -0.10385 | | | | 5274.953 | | | 40.90625 | |  | |  | |  |
| 3.35 | | 70.49141 | | | | -0.10691 | | | | 5274.602 | | | 40.82813 | |  | |  | |  |
| 3.358333 | | 70.55589 | | | | -0.1103 | | | | 5274.258 | | | 40.82031 | |  | |  | |  |
| 3.366667 | | 70.61508 | | | | -0.1135 | | | | 5273.914 | | | 40.86719 | |  | |  | |  |
| 3.375 | | 70.67745 | | | | -0.11728 | | | | 5273.563 | | | 40.85156 | |  | |  | |  |
| 3.383333 | | 70.74405 | | | | -0.1198 | | | | 5273.234 | | | 40.76563 | |  | |  | |  |
| 3.391667 | | 70.80642 | | | | -0.12316 | | | | 5272.883 | | | 40.99219 | |  | |  | |  |
| 3.4 | | 70.86878 | | | | -0.12603 | | | | 5272.555 | | | 40.71875 | |  | |  | |  |
| 3.408333 | | 70.92587 | | | | -0.12981 | | | | 5272.219 | | | 40.84375 | |  | |  | |  |
| 3.416667 | | 70.9893 | | | | -0.13234 | | | | 5271.875 | | | 40.92188 | |  | |  | |  |
| 3.425 | | 71.05695 | | | | -0.13576 | | | | 5271.539 | | | 41.08594 | |  | |  | |  |
| 3.433333 | | 71.12248 | | | | -0.13875 | | | | 5271.211 | | | 41.26563 | |  | |  | |  |
| 3.441667 | | 71.18274 | | | | -0.14229 | | | | 5270.852 | | | 41.25781 | |  | |  | |  |
| 3.45 | | 71.24934 | | | | -0.14537 | | | | 5270.547 | | | 41.30469 | |  | |  | |  |
| 3.458333 | | 71.31276 | | | | -0.14868 | | | | 5270.195 | | | 41.46875 | |  | |  | |  |
| 3.466667 | | 71.37513 | | | | -0.1529 | | | | 5269.844 | | | 41.74219 | |  | |  | |  |
| 3.475 | | 71.44173 | | | | -0.15598 | | | | 5269.477 | | | 41.78125 | |  | |  | |  |
| 3.483333 | | 71.50409 | | | | -0.15978 | | | | 5269.109 | | | 41.96094 | |  | |  | |  |
| 3.491667 | | 71.5728 | | | | -0.1621 | | | | 5268.773 | | | 42.17969 | |  | |  | |  |
| 3.5 | | 71.63623 | | | | -0.16549 | | | | 5268.422 | | | 42.35156 | |  | |  | |  |
| 3.508333 | | 71.69648 | | | | -0.16795 | | | | 5268.055 | | | 42.48438 | |  | |  | |  |
| 3.516667 | | 71.76202 | | | | -0.17031 | | | | 5267.672 | | | 42.71094 | |  | |  | |  |
| 3.525 | | 71.82967 | | | | -0.17279 | | | | 5267.32 | | | 42.91406 | |  | |  | |  |
| 3.533333 | | 71.89204 | | | | -0.17577 | | | | 5266.945 | | | 43.07031 | |  | |  | |  |
| 3.541667 | | 71.95969 | | | | -0.17907 | | | | 5266.563 | | | 43.21094 | |  | |  | |  |
| 3.55 | | 72.02418 | | | | -0.18182 | | | | 5266.188 | | | 43.22656 | |  | |  | |  |
| 3.558333 | | 72.08655 | | | | -0.18566 | | | | 5265.813 | | | 43.26563 | |  | |  | |  |
| 3.566667 | | 72.15314 | | | | -0.18866 | | | | 5265.438 | | | 43.50781 | |  | |  | |  |
| 3.575 | | 72.21762 | | | | -0.19193 | | | | 5265.039 | | | 43.625 | |  | |  | |  |
| 3.583333 | | 72.28422 | | | | -0.19404 | | | | 5264.664 | | | 43.71875 | |  | |  | |  |
| 3.591667 | | 72.35399 | | | | -0.1959 | | | | 5264.281 | | | 43.54688 | |  | |  | |  |
| 3.6 | | 72.41741 | | | | -0.19858 | | | | 5263.914 | | | 43.6875 | |  | |  | |  |
| 3.608333 | | 72.48401 | | | | -0.20049 | | | | 5263.555 | | | 43.63281 | |  | |  | |  |
| 3.616667 | | 72.54849 | | | | -0.20364 | | | | 5263.164 | | | 43.71094 | |  | |  | |  |
| 3.625 | | 72.61298 | | | | -0.20642 | | | | 5262.781 | | | 43.83594 | |  | |  | |  |
| 3.633333 | | 72.67851 | | | | -0.21041 | | | | 5262.406 | | | 43.85938 | |  | |  | |  |
| 3.641667 | | 72.74828 | | | | -0.21374 | | | | 5262.07 | | | 44.10938 | |  | |  | |  |
| 3.65 | | 72.81382 | | | | -0.21739 | | | | 5261.688 | | | 44.32813 | |  | |  | |  |
| 3.658333 | | 72.88464 | | | | -0.21994 | | | | 5261.336 | | | 44.35156 | |  | |  | |  |
| 3.666667 | | 72.95124 | | | | -0.2223 | | | | 5260.961 | | | 44.60156 | |  | |  | |  |
| 3.675 | | 73.01466 | | | | -0.22544 | | | | 5260.578 | | | 44.92188 | |  | |  | |  |
| 3.683333 | | 73.08232 | | | | -0.22796 | | | | 5260.219 | | | 44.85938 | |  | |  | |  |
| 3.691667 | | 73.14892 | | | | -0.2312 | | | | 5259.813 | | | 45.03125 | |  | |  | |  |
| 3.7 | | 73.21551 | | | | -0.23423 | | | | 5259.414 | | | 45.13281 | |  | |  | |  |
| 3.708333 | | 73.28105 | | | | -0.23797 | | | | 5259.031 | | | 45.29688 | |  | |  | |  |
| 3.716667 | | 73.34659 | | | | -0.24127 | | | | 5258.641 | | | 45.32031 | |  | |  | |  |
| 3.725 | | 73.41107 | | | | -0.24459 | | | | 5258.227 | | | 45.33594 | |  | |  | |  |
| 3.733333 | | 73.47661 | | | | -0.24772 | | | | 5257.859 | | | 45.26563 | |  | |  | |  |
| 3.741667 | | 73.54004 | | | | -0.25065 | | | | 5257.469 | | | 45.25781 | |  | |  | |  |
| 3.75 | | 73.60875 | | | | -0.25377 | | | | 5257.086 | | | 45.41406 | |  | |  | |  |
| 3.758333 | | 73.6764 | | | | -0.25615 | | | | 5256.688 | | | 45.54688 | |  | |  | |  |
| 3.766667 | | 73.743 | | | | -0.25922 | | | | 5256.305 | | | 45.6875 | |  | |  | |  |
| 3.775 | | 73.80853 | | | | -0.26166 | | | | 5255.938 | | | 45.85938 | |  | |  | |  |
| 3.783333 | | 73.87724 | | | | -0.26436 | | | | 5255.57 | | | 46.03125 | |  | |  | |  |
| 3.791667 | | 73.94173 | | | | -0.26715 | | | | 5255.203 | | | 46.13281 | |  | |  | |  |
| 3.8 | | 74.00727 | | | | -0.26978 | | | | 5254.805 | | | 46.35938 | |  | |  | |  |
| 3.808333 | | 74.07175 | | | | -0.27308 | | | | 5254.414 | | | 46.60938 | |  | |  | |  |
| 3.816667 | | 74.14152 | | | | -0.27541 | | | | 5254.016 | | | 46.53125 | |  | |  | |  |
| 3.825 | | 74.20917 | | | | -0.27805 | | | | 5253.609 | | | 46.77344 | |  | |  | |  |
| 3.833333 | | 74.26942 | | | | -0.28087 | | | | 5253.203 | | | 47.00781 | |  | |  | |  |
| 3.841667 | | 74.33813 | | | | -0.28323 | | | | 5252.813 | | | 46.97656 | |  | |  | |  |
| 3.85 | | 74.40578 | | | | -0.28611 | | | | 5252.391 | | | 47.03906 | |  | |  | |  |
| 3.858333 | | 74.47449 | | | | -0.28822 | | | | 5251.977 | | | 47.03906 | |  | |  | |  |
| 3.866667 | | 74.54533 | | | | -0.2908 | | | | 5251.602 | | | 47.17188 | |  | |  | |  |
| 3.875 | | 74.61404 | | | | -0.2927 | | | | 5251.18 | | | 47.29688 | |  | |  | |  |
| 3.883333 | | 74.6838 | | | | -0.2953 | | | | 5250.758 | | | 47.28125 | |  | |  | |  |
| 3.891667 | | 74.75357 | | | | -0.29792 | | | | 5250.359 | | | 47.33594 | |  | |  | |  |
| 3.9 | | 74.82017 | | | | -0.30058 | | | | 5249.969 | | | 47.57813 | |  | |  | |  |
| 3.908333 | | 74.88888 | | | | -0.3038 | | | | 5249.578 | | | 47.48438 | |  | |  | |  |
| 3.916667 | | 74.96075 | | | | -0.30598 | | | | 5249.164 | | | 47.30469 | |  | |  | |  |
| 3.925 | | 75.02735 | | | | -0.30922 | | | | 5248.758 | | | 47.28125 | |  | |  | |  |
| 3.933333 | | 75.09395 | | | | -0.31179 | | | | 5248.367 | | | 47.30469 | |  | |  | |  |
| 3.941667 | | 75.16055 | | | | -0.31458 | | | | 5247.961 | | | 47.49219 | |  | |  | |  |
| 3.95 | | 75.22714 | | | | -0.31801 | | | | 5247.539 | | | 47.38281 | |  | |  | |  |
| 3.958333 | | 75.30219 | | | | -0.32043 | | | | 5247.156 | | | 47.39063 | |  | |  | |  |
| 3.966667 | | 75.37196 | | | | -0.32361 | | | | 5246.797 | | | 47.30469 | |  | |  | |  |
| 3.975 | | 75.43961 | | | | -0.32628 | | | | 5246.406 | | | 47.35938 | |  | |  | |  |
| 3.983333 | | 75.50938 | | | | -0.32862 | | | | 5246.008 | | | 47.57813 | |  | |  | |  |
| 3.991667 | | 75.57809 | | | | -0.33095 | | | | 5245.594 | | | 47.57813 | |  | |  | |  |
| 4 | | 75.64786 | | | | -0.33259 | | | | 5245.211 | | | 47.67969 | |  | |  | |  |
| 4.008333 | | 75.7208 | | | | -0.33504 | | | | 5244.82 | | | 47.78906 | |  | |  | |  |
| 4.016667 | | 75.79691 | | | | -0.33606 | | | | 5244.438 | | | 47.88281 | |  | |  | |  |
| 4.025 | | 75.86985 | | | | -0.33774 | | | | 5244.047 | | | 47.92969 | |  | |  | |  |
| 4.033333 | | 75.9375 | | | | -0.34058 | | | | 5243.625 | | | 48.09375 | |  | |  | |  |
| 4.041667 | | 76.00516 | | | | -0.3426 | | | | 5243.219 | | | 48.32813 | |  | |  | |  |
| 4.05 | | 76.07492 | | | | -0.34544 | | | | 5242.813 | | | 48.46094 | |  | |  | |  |
| 4.058333 | | 76.14892 | | | | -0.34701 | | | | 5242.398 | | | 48.34375 | |  | |  | |  |
| 4.066667 | | 76.21869 | | | | -0.35006 | | | | 5241.984 | | | 48.27344 | |  | |  | |  |
| 4.075 | | 76.2874 | | | | -0.35232 | | | | 5241.57 | | | 48.3125 | |  | |  | |  |
| 4.083333 | | 76.35716 | | | | -0.35455 | | | | 5241.156 | | | 48.28906 | |  | |  | |  |
| 4.091667 | | 76.42482 | | | | -0.35781 | | | | 5240.719 | | | 48.42188 | |  | |  | |  |
| 4.1 | | 76.49353 | | | | -0.35995 | | | | 5240.305 | | | 48.35938 | |  | |  | |  |
| 4.108333 | | 76.56118 | | | | -0.36313 | | | | 5239.922 | | | 48.35938 | |  | |  | |  |
| 4.116667 | | 76.63306 | | | | -0.3649 | | | | 5239.531 | | | 48.30469 | |  | |  | |  |
| 4.125 | | 76.70283 | | | | -0.3675 | | | | 5239.125 | | | 48.08594 | |  | |  | |  |
| 4.133333 | | 76.77471 | | | | -0.36916 | | | | 5238.727 | | | 48.24219 | |  | |  | |  |
| 4.141667 | | 76.84554 | | | | -0.37096 | | | | 5238.313 | | | 48.49219 | |  | |  | |  |
| 4.15 | | 76.91531 | | | | -0.37328 | | | | 5237.906 | | | 48.60156 | |  | |  | |  |
| 4.158333 | | 76.98613 | | | | -0.37519 | | | | 5237.508 | | | 48.71875 | |  | |  | |  |
| 4.166667 | | 77.06013 | | | | -0.37781 | | | | 5237.117 | | | 48.95313 | |  | |  | |  |
| 4.175 | | 77.13095 | | | | -0.38034 | | | | 5236.734 | | | 49.125 | |  | |  | |  |
| 4.183333 | | 77.20071 | | | | -0.38216 | | | | 5236.328 | | | 49.22656 | |  | |  | |  |
| 4.191667 | | 77.27155 | | | | -0.38429 | | | | 5235.891 | | | 49.10938 | |  | |  | |  |
| 4.2 | | 77.3466 | | | | -0.38596 | | | | 5235.469 | | | 49.34375 | |  | |  | |  |
| 4.208333 | | 77.41742 | | | | -0.38776 | | | | 5235.039 | | | 49.36719 | |  | |  | |  |
| 4.216667 | | 77.48824 | | | | -0.3895 | | | | 5234.602 | | | 49.44531 | |  | |  | |  |
| 4.225 | | 77.55695 | | | | -0.39122 | | | | 5234.164 | | | 49.53125 | |  | |  | |  |
| 4.233333 | | 77.63095 | | | | -0.39326 | | | | 5233.742 | | | 49.40625 | |  | |  | |  |
| 4.241667 | | 77.706 | | | | -0.39456 | | | | 5233.352 | | | 49.27344 | |  | |  | |  |
| 4.25 | | 77.77471 | | | | -0.39673 | | | | 5232.914 | | | 49.17969 | |  | |  | |  |
| 4.258333 | | 77.84766 | | | | -0.39877 | | | | 5232.5 | | | 49.07031 | |  | |  | |  |
| 4.266667 | | 77.92059 | | | | -0.40019 | | | | 5232.07 | | | 49.14063 | |  | |  | |  |
| 4.275 | | 77.99353 | | | | -0.40294 | | | | 5231.648 | | | 49.3125 | |  | |  | |  |
| 4.283333 | | 78.06224 | | | | -0.40525 | | | | 5231.258 | | | 49.32031 | |  | |  | |  |
| 4.291667 | | 78.13306 | | | | -0.40806 | | | | 5230.859 | | | 49.53906 | |  | |  | |  |
| 4.3 | | 78.20284 | | | | -0.41084 | | | | 5230.469 | | | 49.5625 | |  | |  | |  |
| 4.308333 | | 78.27471 | | | | -0.41345 | | | | 5230.078 | | | 49.53906 | |  | |  | |  |
| 4.316667 | | 78.34448 | | | | -0.41653 | | | | 5229.664 | | | 49.84375 | |  | |  | |  |
| 4.325 | | 78.41637 | | | | -0.41793 | | | | 5229.227 | | | 49.85156 | |  | |  | |  |
| 4.333333 | | 78.4893 | | | | -0.42005 | | | | 5228.82 | | | 50.02344 | |  | |  | |  |
| 4.341667 | | 78.56013 | | | | -0.42178 | | | | 5228.367 | | | 49.96875 | |  | |  | |  |
| 4.35 | | 78.63095 | | | | -0.42324 | | | | 5227.961 | | | 49.89063 | |  | |  | |  |
| 4.358333 | | 78.706 | | | | -0.42465 | | | | 5227.547 | | | 49.99219 | |  | |  | |  |
| 4.366667 | | 78.77471 | | | | -0.4267 | | | | 5227.102 | | | 50 | |  | |  | |  |
| 4.375 | | 78.84766 | | | | -0.42945 | | | | 5226.688 | | | 49.89063 | |  | |  | |  |
| 4.383333 | | 78.92165 | | | | -0.43195 | | | | 5226.242 | | | 49.92969 | |  | |  | |  |
| 4.391667 | | 78.99882 | | | | -0.43281 | | | | 5225.828 | | | 50.05469 | |  | |  | |  |
| 4.4 | | 79.07387 | | | | -0.43576 | | | | 5225.43 | | | 49.97656 | |  | |  | |  |
| 4.408333 | | 79.14469 | | | | -0.43726 | | | | 5225.008 | | | 50.00781 | |  | |  | |  |
| 4.416667 | | 79.2208 | | | | -0.43893 | | | | 5224.586 | | | 49.88281 | |  | |  | |  |
| 4.425 | | 79.2948 | | | | -0.44158 | | | | 5224.188 | | | 49.97656 | |  | |  | |  |
| 4.433333 | | 79.3688 | | | | -0.44348 | | | | 5223.758 | | | 50.14844 | |  | |  | |  |
| 4.441667 | | 79.43751 | | | | -0.44606 | | | | 5223.32 | | | 50.05469 | |  | |  | |  |
| 4.45 | | 79.51678 | | | | -0.44654 | | | | 5222.922 | | | 49.90625 | |  | |  | |  |
| 4.458333 | | 79.5929 | | | | -0.44771 | | | | 5222.5 | | | 50.21094 | |  | |  | |  |
| 4.466667 | | 79.66689 | | | | -0.44903 | | | | 5222.102 | | | 50.13281 | |  | |  | |  |
| 4.475 | | 79.73772 | | | | -0.44992 | | | | 5221.672 | | | 49.96094 | |  | |  | |  |
| 4.483333 | | 79.81065 | | | | -0.45148 | | | | 5221.234 | | | 50.14844 | |  | |  | |  |
| 4.491667 | | 79.88571 | | | | -0.45306 | | | | 5220.836 | | | 50.22656 | |  | |  | |  |
| 4.5 | | 79.95547 | | | | -0.45487 | | | | 5220.438 | | | 50.4375 | |  | |  | |  |
| 4.508333 | | 80.03053 | | | | -0.45724 | | | | 5219.984 | | | 50.42969 | |  | |  | |  |
| 4.516667 | | 80.10241 | | | | -0.45903 | | | | 5219.578 | | | 50.35156 | |  | |  | |  |
| 4.525 | | 80.18063 | | | | -0.46102 | | | | 5219.188 | | | 50.39063 | |  | |  | |  |
| 4.533333 | | 80.25463 | | | | -0.46319 | | | | 5218.75 | | | 50.49219 | |  | |  | |  |
| 4.541667 | | 80.32969 | | | | -0.46485 | | | | 5218.305 | | | 50.42969 | |  | |  | |  |
| 4.55 | | 80.40685 | | | | -0.46648 | | | | 5217.859 | | | 50.41406 | |  | |  | |  |
| 4.558333 | | 80.48085 | | | | -0.46764 | | | | 5217.438 | | | 50.29688 | |  | |  | |  |
| 4.566667 | | 80.5559 | | | | -0.46891 | | | | 5217.031 | | | 50.21094 | |  | |  | |  |
| 4.575 | | 80.63095 | | | | -0.47102 | | | | 5216.602 | | | 50 | |  | |  | |  |
| 4.583333 | | 80.70812 | | | | -0.47271 | | | | 5216.164 | | | 50.02344 | |  | |  | |  |
| 4.591667 | | 80.77895 | | | | -0.47583 | | | | 5215.758 | | | 50.14063 | |  | |  | |  |
| 4.6 | | 80.85506 | | | | -0.47746 | | | | 5215.344 | | | 50.33594 | |  | |  | |  |
| 4.608333 | | 80.92694 | | | | -0.47897 | | | | 5214.938 | | | 50.46875 | |  | |  | |  |
| 4.616667 | | 81.00093 | | | | -0.48078 | | | | 5214.531 | | | 50.26563 | |  | |  | |  |
| 4.625 | | 81.07281 | | | | -0.48156 | | | | 5214.141 | | | 50.21094 | |  | |  | |  |
| 4.633333 | | 81.1447 | | | | -0.48265 | | | | 5213.727 | | | 50.39844 | |  | |  | |  |
| 4.641667 | | 81.2187 | | | | -0.48423 | | | | 5213.289 | | | 50.54688 | |  | |  | |  |
| 4.65 | | 81.29269 | | | | -0.48535 | | | | 5212.852 | | | 50.75781 | |  | |  | |  |
| 4.658333 | | 81.36986 | | | | -0.48741 | | | | 5212.414 | | | 50.94531 | |  | |  | |  |
| 4.666667 | | 81.44491 | | | | -0.48925 | | | | 5212.023 | | | 51.07813 | |  | |  | |  |
| 4.675 | | 81.52313 | | | | -0.49052 | | | | 5211.617 | | | 51 | |  | |  | |  |
| 4.683333 | | 81.59924 | | | | -0.49237 | | | | 5211.164 | | | 51.07031 | |  | |  | |  |
| 4.691667 | | 81.67853 | | | | -0.49358 | | | | 5210.719 | | | 51.21094 | |  | |  | |  |
| 4.7 | | 81.7504 | | | | -0.49557 | | | | 5210.273 | | | 51.57031 | |  | |  | |  |
| 4.708333 | | 81.8244 | | | | -0.49679 | | | | 5209.82 | | | 51.63281 | |  | |  | |  |
| 4.716667 | | 81.8999 | | | | -0.49794 | | | | 5209.367 | | | 51.57813 | |  | |  | |  |
| 4.725 | | 81.97514 | | | | -0.50043 | | | | 5208.953 | | | 51.71875 | |  | |  | |  |
| 4.733333 | | 82.05037 | | | | -0.50177 | | | | 5208.516 | | | 51.72656 | |  | |  | |  |
| 4.741667 | | 82.12148 | | | | -0.50277 | | | | 5208.078 | | | 51.60938 | |  | |  | |  |
| 4.75 | | 82.19363 | | | | -0.505 | | | | 5207.594 | | | 51.39063 | |  | |  | |  |
| 4.758333 | | 82.26577 | | | | -0.5059 | | | | 5207.164 | | | 51.50781 | |  | |  | |  |
| 4.766667 | | 82.33688 | | | | -0.50685 | | | | 5206.734 | | | 51.34375 | |  | |  | |  |
| 4.775 | | 82.40387 | | | | -0.50773 | | | | 5206.289 | | | 51.25781 | |  | |  | |  |
| 4.783333 | | 82.48116 | | | | -0.50865 | | | | 5205.859 | | | 51.22656 | |  | |  | |  |
| 4.791667 | | 82.5564 | | | | -0.51081 | | | | 5205.445 | | | 51.45313 | |  | |  | |  |
| 4.8 | | 82.62957 | | | | -0.51172 | | | | 5205.039 | | | 51.39844 | |  | |  | |  |
| 4.808333 | | 82.70377 | | | | -0.51327 | | | | 5204.609 | | | 51.35938 | |  | |  | |  |
| 4.816667 | | 82.77798 | | | | -0.51455 | | | | 5204.203 | | | 51.25781 | |  | |  | |  |
| 4.825 | | 82.85115 | | | | -0.51552 | | | | 5203.789 | | | 51.47656 | |  | |  | |  |
| 4.833333 | | 82.92639 | | | | -0.51676 | | | | 5203.367 | | | 51.5 | |  | |  | |  |
| 4.841667 | | 83.00163 | | | | -0.51754 | | | | 5202.914 | | | 51.54688 | |  | |  | |  |
| 4.85 | | 83.07686 | | | | -0.51902 | | | | 5202.492 | | | 51.74219 | |  | |  | |  |
| 4.858333 | | 83.15415 | | | | -0.52043 | | | | 5202.063 | | | 51.75781 | |  | |  | |  |
| 4.866667 | | 83.23042 | | | | -0.52131 | | | | 5201.648 | | | 51.48438 | |  | |  | |  |
| 4.875 | | 83.30668 | | | | -0.52282 | | | | 5201.203 | | | 51.35938 | |  | |  | |  |
| 4.883333 | | 83.38088 | | | | -0.52367 | | | | 5200.773 | | | 51.35156 | |  | |  | |  |
| 4.891667 | | 83.45406 | | | | -0.52431 | | | | 5200.328 | | | 51.21094 | |  | |  | |  |
| 4.9 | | 83.53033 | | | | -0.5252 | | | | 5199.867 | | | 51.20313 | |  | |  | |  |
| 4.908333 | | 83.60453 | | | | -0.52613 | | | | 5199.438 | | | 51.125 | |  | |  | |  |
| 4.916667 | | 83.67873 | | | | -0.52825 | | | | 5199.047 | | | 51.17969 | |  | |  | |  |
| 4.925 | | 83.75397 | | | | -0.53004 | | | | 5198.633 | | | 51.13281 | |  | |  | |  |
| 4.933333 | | 83.83332 | | | | -0.53066 | | | | 5198.211 | | | 50.94531 | |  | |  | |  |
| 4.941667 | | 83.91268 | | | | -0.53233 | | | | 5197.805 | | | 50.96094 | |  | |  | |  |
| 4.95 | | 83.98379 | | | | -0.53409 | | | | 5197.383 | | | 51.32031 | |  | |  | |  |
| 4.958333 | | 84.06006 | | | | -0.53404 | | | | 5196.969 | | | 51.33594 | |  | |  | |  |
| 4.966667 | | 84.13941 | | | | -0.53478 | | | | 5196.531 | | | 51.4375 | |  | |  | |  |
| 4.975 | | 84.21774 | | | | -0.53594 | | | | 5196.117 | | | 51.61719 | |  | |  | |  |
| 4.983333 | | 84.29091 | | | | -0.53717 | | | | 5195.719 | | | 51.63281 | |  | |  | |  |
| 4.991667 | | 84.36924 | | | | -0.53871 | | | | 5195.289 | | | 51.84375 | |  | |  | |  |
| 5 | | 84.44859 | | | | -0.53982 | | | | 5194.813 | | | 51.71094 | |  | |  | |  |
| 5.008333 | | 84.52486 | | | | -0.54173 | | | | 5194.391 | | | 51.53906 | |  | |  | |  |
| 5.016667 | | 84.597 | | | | -0.5433 | | | | 5193.945 | | | 51.57813 | |  | |  | |  |
| 5.025 | | 84.67223 | | | | -0.5435 | | | | 5193.492 | | | 51.625 | |  | |  | |  |
| 5.033333 | | 84.75262 | | | | -0.54433 | | | | 5193.055 | | | 51.58594 | |  | |  | |  |
| 5.041667 | | 84.83404 | | | | -0.54422 | | | | 5192.594 | | | 51.49219 | |  | |  | |  |
| 5.05 | | 84.90824 | | | | -0.54381 | | | | 5192.18 | | | 51.41406 | |  | |  | |  |
| 5.058333 | | 84.98348 | | | | -0.54531 | | | | 5191.766 | | | 51.39844 | |  | |  | |  |
| 5.066667 | | 85.06284 | | | | -0.54688 | | | | 5191.328 | | | 51.42188 | |  | |  | |  |
| 5.075 | | 85.14013 | | | | -0.54777 | | | | 5190.891 | | | 51.22656 | |  | |  | |  |
| 5.083333 | | 85.21743 | | | | -0.54949 | | | | 5190.469 | | | 51.27344 | |  | |  | |  |
| 5.091667 | | 85.29575 | | | | -0.55046 | | | | 5190.055 | | | 51.5 | |  | |  | |  |
| 5.1 | | 85.37408 | | | | -0.55078 | | | | 5189.625 | | | 51.625 | |  | |  | |  |
| 5.108333 | | 85.44931 | | | | -0.55129 | | | | 5189.203 | | | 51.67188 | |  | |  | |  |
| 5.116667 | | 85.52351 | | | | -0.5512 | | | | 5188.773 | | | 51.67969 | |  | |  | |  |
| 5.125 | | 85.59463 | | | | -0.55253 | | | | 5188.367 | | | 51.8125 | |  | |  | |  |
| 5.133333 | | 85.66986 | | | | -0.55398 | | | | 5187.938 | | | 51.97656 | |  | |  | |  |
| 5.141667 | | 85.74612 | | | | -0.55475 | | | | 5187.477 | | | 51.83594 | |  | |  | |  |
| 5.15 | | 85.82033 | | | | -0.55629 | | | | 5187.023 | | | 51.92188 | |  | |  | |  |
| 5.158333 | | 85.89454 | | | | -0.55705 | | | | 5186.594 | | | 52.07031 | |  | |  | |  |
| 5.166667 | | 85.9677 | | | | -0.55819 | | | | 5186.156 | | | 52.07031 | |  | |  | |  |
| 5.175 | | 86.04295 | | | | -0.5592 | | | | 5185.719 | | | 51.91406 | |  | |  | |  |
| 5.183333 | | 86.11921 | | | | -0.55852 | | | | 5185.266 | | | 51.71875 | |  | |  | |  |
| 5.191667 | | 86.19547 | | | | -0.55904 | | | | 5184.844 | | | 51.67969 | |  | |  | |  |
| 5.2 | | 86.27277 | | | | -0.55953 | | | | 5184.398 | | | 51.48438 | |  | |  | |  |
| 5.208333 | | 86.34903 | | | | -0.55961 | | | | 5183.953 | | | 51.53906 | |  | |  | |  |
| 5.216667 | | 86.42942 | | | | -0.56037 | | | | 5183.516 | | | 51.28125 | |  | |  | |  |
| 5.225 | | 86.50671 | | | | -0.56147 | | | | 5183.109 | | | 51.32031 | |  | |  | |  |
| 5.233333 | | 86.58607 | | | | -0.56168 | | | | 5182.703 | | | 51.25 | |  | |  | |  |
| 5.241667 | | 86.66543 | | | | -0.56206 | | | | 5182.281 | | | 51.21094 | |  | |  | |  |
| 5.25 | | 86.74375 | | | | -0.56225 | | | | 5181.867 | | | 51.22656 | |  | |  | |  |
| 5.258333 | | 86.82105 | | | | -0.56216 | | | | 5181.43 | | | 51.23438 | |  | |  | |  |
| 5.266667 | | 86.89938 | | | | -0.56269 | | | | 5181.039 | | | 51.125 | |  | |  | |  |
| 5.275 | | 86.9777 | | | | -0.563 | | | | 5180.609 | | | 51.20313 | |  | |  | |  |
| 5.283333 | | 87.05809 | | | | -0.5636 | | | | 5180.188 | | | 51.07031 | |  | |  | |  |
| 5.291667 | | 87.13538 | | | | -0.5651 | | | | 5179.773 | | | 51.00781 | |  | |  | |  |
| 5.3 | | 87.21165 | | | | -0.5651 | | | | 5179.344 | | | 50.96875 | |  | |  | |  |
| 5.308333 | | 87.28895 | | | | -0.56581 | | | | 5178.922 | | | 50.75 | |  | |  | |  |
| 5.316667 | | 87.36521 | | | | -0.567 | | | | 5178.508 | | | 50.85938 | |  | |  | |  |
| 5.325 | | 87.44147 | | | | -0.56732 | | | | 5178.078 | | | 50.70313 | |  | |  | |  |
| 5.333333 | | 87.51568 | | | | -0.56812 | | | | 5177.672 | | | 50.54688 | |  | |  | |  |
| 5.341667 | | 87.59504 | | | | -0.56849 | | | | 5177.25 | | | 50.54688 | |  | |  | |  |
| 5.35 | | 87.67027 | | | | -0.56936 | | | | 5176.828 | | | 50.66406 | |  | |  | |  |
| 5.358333 | | 87.74654 | | | | -0.57073 | | | | 5176.422 | | | 50.75 | |  | |  | |  |
| 5.366667 | | 87.82074 | | | | -0.57078 | | | | 5176 | | | 50.78125 | |  | |  | |  |
| 5.375 | | 87.89906 | | | | -0.57137 | | | | 5175.586 | | | 50.66406 | |  | |  | |  |
| 5.383333 | | 87.97842 | | | | -0.57191 | | | | 5175.188 | | | 50.71875 | |  | |  | |  |
| 5.391667 | | 88.05675 | | | | -0.57177 | | | | 5174.766 | | | 50.94531 | |  | |  | |  |
| 5.4 | | 88.13301 | | | | -0.57214 | | | | 5174.32 | | | 50.83594 | |  | |  | |  |
| 5.408333 | | 88.21443 | | | | -0.57291 | | | | 5173.891 | | | 51.05469 | |  | |  | |  |
| 5.416667 | | 88.29379 | | | | -0.57297 | | | | 5173.453 | | | 50.98438 | |  | |  | |  |
| 5.425 | | 88.37212 | | | | -0.57398 | | | | 5173.055 | | | 51.07813 | |  | |  | |  |
| 5.433333 | | 88.45044 | | | | -0.57423 | | | | 5172.617 | | | 51.14844 | |  | |  | |  |
| 5.441667 | | 88.52979 | | | | -0.57392 | | | | 5172.164 | | | 50.9375 | |  | |  | |  |
| 5.45 | | 88.60606 | | | | -0.57441 | | | | 5171.758 | | | 50.70313 | |  | |  | |  |
| 5.458333 | | 88.68439 | | | | -0.57412 | | | | 5171.297 | | | 50.73438 | |  | |  | |  |
| 5.466667 | | 88.76271 | | | | -0.57386 | | | | 5170.883 | | | 50.58594 | |  | |  | |  |
| 5.475 | | 88.8431 | | | | -0.57411 | | | | 5170.445 | | | 50.45313 | |  | |  | |  |
| 5.483333 | | 88.92245 | | | | -0.57405 | | | | 5170.016 | | | 50.3125 | |  | |  | |  |
| 5.491667 | | 88.99873 | | | | -0.57393 | | | | 5169.617 | | | 50.24219 | |  | |  | |  |
| 5.5 | | 89.07808 | | | | -0.57448 | | | | 5169.219 | | | 50.53125 | |  | |  | |  |
| 5.508333 | | 89.15537 | | | | -0.57407 | | | | 5168.789 | | | 50.57813 | |  | |  | |  |
| 5.516667 | | 89.23679 | | | | -0.57384 | | | | 5168.391 | | | 50.50781 | |  | |  | |  |
| 5.525 | | 89.31615 | | | | -0.57458 | | | | 5167.992 | | | 50.61719 | |  | |  | |  |
| 5.533333 | | 89.39963 | | | | -0.57482 | | | | 5167.578 | | | 50.78906 | |  | |  | |  |
| 5.541667 | | 89.47692 | | | | -0.57558 | | | | 5167.172 | | | 50.92969 | |  | |  | |  |
| 5.55 | | 89.55628 | | | | -0.57663 | | | | 5166.711 | | | 51.20313 | |  | |  | |  |
| 5.558333 | | 89.63461 | | | | -0.5771 | | | | 5166.273 | | | 51.28906 | |  | |  | |  |
| 5.566667 | | 89.715 | | | | -0.57761 | | | | 5165.867 | | | 51.63281 | |  | |  | |  |
| 5.575 | | 89.79435 | | | | -0.57773 | | | | 5165.422 | | | 51.46875 | |  | |  | |  |
| 5.583333 | | 89.87577 | | | | -0.57693 | | | | 5164.977 | | | 51.25781 | |  | |  | |  |
| 5.591667 | | 89.95718 | | | | -0.57756 | | | | 5164.539 | | | 51.19531 | |  | |  | |  |
| 5.6 | | 90.03654 | | | | -0.57771 | | | | 5164.07 | | | 51.10938 | |  | |  | |  |
| 5.608333 | | 90.12106 | | | | -0.57709 | | | | 5163.625 | | | 51.13281 | |  | |  | |  |
| 5.616667 | | 90.19732 | | | | -0.57841 | | | | 5163.156 | | | 51.17969 | |  | |  | |  |
| 5.625 | | 90.27873 | | | | -0.57853 | | | | 5162.75 | | | 51.08594 | |  | |  | |  |
| 5.633333 | | 90.35809 | | | | -0.57799 | | | | 5162.352 | | | 50.95313 | |  | |  | |  |
| 5.641667 | | 90.43848 | | | | -0.57841 | | | | 5161.938 | | | 50.83594 | |  | |  | |  |
| 5.65 | | 90.52093 | | | | -0.57885 | | | | 5161.516 | | | 50.71094 | |  | |  | |  |
| 5.658333 | | 90.60235 | | | | -0.57891 | | | | 5161.086 | | | 51.03906 | |  | |  | |  |
| 5.666667 | | 90.68376 | | | | -0.57962 | | | | 5160.664 | | | 51.22656 | |  | |  | |  |
| 5.675 | | 90.76209 | | | | -0.58002 | | | | 5160.25 | | | 51.125 | |  | |  | |  |
| 5.683333 | | 90.83939 | | | | -0.58091 | | | | 5159.852 | | | 51.35156 | |  | |  | |  |
| 5.691667 | | 90.91771 | | | | -0.58127 | | | | 5159.445 | | | 51.33594 | |  | |  | |  |
| 5.7 | | 91.00222 | | | | -0.58087 | | | | 5159.039 | | | 51.17969 | |  | |  | |  |
| 5.708333 | | 91.0857 | | | | -0.58112 | | | | 5158.57 | | | 51.33594 | |  | |  | |  |
| 5.716667 | | 91.16608 | | | | -0.58198 | | | | 5158.109 | | | 51.21875 | |  | |  | |  |
| 5.725 | | 91.24545 | | | | -0.58145 | | | | 5157.695 | | | 50.98438 | |  | |  | |  |
| 5.733333 | | 91.32274 | | | | -0.58158 | | | | 5157.25 | | | 50.84375 | |  | |  | |  |
| 5.741667 | | 91.40107 | | | | -0.58213 | | | | 5156.82 | | | 50.625 | |  | |  | |  |
| 5.75 | | 91.48042 | | | | -0.58135 | | | | 5156.414 | | | 50.30469 | |  | |  | |  |
| 5.758333 | | 91.56184 | | | | -0.58083 | | | | 5155.969 | | | 50.21875 | |  | |  | |  |
| 5.766667 | | 91.64429 | | | | -0.58098 | | | | 5155.555 | | | 50.10156 | |  | |  | |  |
| 5.775 | | 91.72159 | | | | -0.58002 | | | | 5155.164 | | | 49.91406 | |  | |  | |  |
| 5.783333 | | 91.80094 | | | | -0.57989 | | | | 5154.758 | | | 49.74219 | |  | |  | |  |
| 5.791667 | | 91.88442 | | | | -0.57912 | | | | 5154.359 | | | 49.55469 | |  | |  | |  |
| 5.8 | | 91.96584 | | | | -0.57774 | | | | 5153.984 | | | 49.55469 | |  | |  | |  |
| 5.808333 | | 92.0452 | | | | -0.57759 | | | | 5153.578 | | | 49.46875 | |  | |  | |  |
| 5.816667 | | 92.12661 | | | | -0.57727 | | | | 5153.18 | | | 49.44531 | |  | |  | |  |
| 5.825 | | 92.20803 | | | | -0.57634 | | | | 5152.781 | | | 49.34375 | |  | |  | |  |
| 5.833333 | | 92.28945 | | | | -0.57643 | | | | 5152.398 | | | 49.27344 | |  | |  | |  |
| 5.841667 | | 92.37189 | | | | -0.57625 | | | | 5152.008 | | | 49.1875 | |  | |  | |  |
| 5.85 | | 92.45332 | | | | -0.57591 | | | | 5151.602 | | | 49.25 | |  | |  | |  |
| 5.858333 | | 92.53577 | | | | -0.5767 | | | | 5151.188 | | | 48.97656 | |  | |  | |  |
| 5.866667 | | 92.61924 | | | | -0.57637 | | | | 5150.781 | | | 49.11719 | |  | |  | |  |
| 5.875 | | 92.69448 | | | | -0.57624 | | | | 5150.391 | | | 49.0625 | |  | |  | |  |
| 5.883333 | | 92.7728 | | | | -0.57736 | | | | 5150 | | | 48.94531 | |  | |  | |  |
| 5.891667 | | 92.85319 | | | | -0.57634 | | | | 5149.586 | | | 48.86719 | |  | |  | |  |
| 5.9 | | 92.93461 | | | | -0.57554 | | | | 5149.172 | | | 48.90625 | |  | |  | |  |
| 5.908333 | | 93.01396 | | | | -0.57548 | | | | 5148.797 | | | 48.89063 | |  | |  | |  |
| 5.916667 | | 93.09332 | | | | -0.575 | | | | 5148.367 | | | 48.96094 | |  | |  | |  |
| 5.925 | | 93.17245 | | | | -0.57479 | | | | 5147.961 | | | 48.90625 | |  | |  | |  |
| 5.933333 | | 93.25008 | | | | -0.57471 | | | | 5147.563 | | | 48.82031 | |  | |  | |  |
| 5.941667 | | 93.32772 | | | | -0.57378 | | | | 5147.164 | | | 49.00781 | |  | |  | |  |
| 5.95 | | 93.40337 | | | | -0.5735 | | | | 5146.742 | | | 48.95313 | |  | |  | |  |
| 5.958333 | | 93.48399 | | | | -0.57273 | | | | 5146.336 | | | 48.96094 | |  | |  | |  |
| 5.966667 | | 93.56361 | | | | -0.57132 | | | | 5145.906 | | | 49.19531 | |  | |  | |  |
| 5.975 | | 93.64523 | | | | -0.57084 | | | | 5145.508 | | | 49.29688 | |  | |  | |  |
| 5.983333 | | 93.72186 | | | | -0.57145 | | | | 5145.117 | | | 49.1875 | |  | |  | |  |
| 5.991667 | | 93.80049 | | | | -0.57075 | | | | 5144.672 | | | 49.28906 | |  | |  | |  |
| 6 | | 93.88012 | | | | -0.57088 | | | | 5144.273 | | | 49.28125 | |  | |  | |  |
| 6.008333 | | 93.95975 | | | | -0.5708 | | | | 5143.867 | | | 49.33594 | |  | |  | |  |
| 6.016667 | | 94.03838 | | | | -0.57027 | | | | 5143.422 | | | 49.36719 | |  | |  | |  |
| 6.025 | | 94.118 | | | | -0.57009 | | | | 5142.992 | | | 49.60938 | |  | |  | |  |
| 6.033333 | | 94.19663 | | | | -0.56951 | | | | 5142.594 | | | 49.63281 | |  | |  | |  |
| 6.041667 | | 94.27427 | | | | -0.56872 | | | | 5142.18 | | | 49.84375 | |  | |  | |  |
| 6.05 | | 94.35389 | | | | -0.56854 | | | | 5141.766 | | | 49.76563 | |  | |  | |  |
| 6.058333 | | 94.43153 | | | | -0.56762 | | | | 5141.344 | | | 49.97656 | |  | |  | |  |
| 6.066667 | | 94.51115 | | | | -0.56638 | | | | 5140.914 | | | 50.14063 | |  | |  | |  |
| 6.075 | | 94.59277 | | | | -0.56615 | | | | 5140.477 | | | 50.25781 | |  | |  | |  |
| 6.083333 | | 94.67339 | | | | -0.56565 | | | | 5140.055 | | | 50.28906 | |  | |  | |  |
| 6.091667 | | 94.75301 | | | | -0.56536 | | | | 5139.602 | | | 50.29688 | |  | |  | |  |
| 6.1 | | 94.83264 | | | | -0.56567 | | | | 5139.203 | | | 50.45313 | |  | |  | |  |
| 6.108333 | | 94.90928 | | | | -0.56495 | | | | 5138.75 | | | 50.26563 | |  | |  | |  |
| 6.116667 | | 94.9899 | | | | -0.56423 | | | | 5138.32 | | | 50.41406 | |  | |  | |  |
| 6.125 | | 95.06953 | | | | -0.56416 | | | | 5137.875 | | | 50.21875 | |  | |  | |  |
| 6.133333 | | 95.14716 | | | | -0.56307 | | | | 5137.453 | | | 50.39063 | |  | |  | |  |
| 6.141667 | | 95.22579 | | | | -0.56193 | | | | 5137.039 | | | 50.26563 | |  | |  | |  |
| 6.15 | | 95.3084 | | | | -0.56212 | | | | 5136.602 | | | 50.45313 | |  | |  | |  |
| 6.158333 | | 95.39101 | | | | -0.5614 | | | | 5136.211 | | | 50.375 | |  | |  | |  |
| 6.166667 | | 95.46964 | | | | -0.56053 | | | | 5135.773 | | | 50.28125 | |  | |  | |  |
| 6.175 | | 95.54728 | | | | -0.56079 | | | | 5135.375 | | | 50.1875 | |  | |  | |  |
| 6.183333 | | 95.6279 | | | | -0.56016 | | | | 5134.945 | | | 49.89063 | |  | |  | |  |
| 6.191667 | | 95.71051 | | | | -0.55877 | | | | 5134.547 | | | 49.69531 | |  | |  | |  |
| 6.2 | | 95.79113 | | | | -0.55881 | | | | 5134.094 | | | 49.35938 | |  | |  | |  |
| 6.208333 | | 95.87374 | | | | -0.55753 | | | | 5133.695 | | | 49.40625 | |  | |  | |  |
| 6.216667 | | 95.95436 | | | | -0.55643 | | | | 5133.281 | | | 49.05469 | |  | |  | |  |
| 6.225 | | 96.03797 | | | | -0.55631 | | | | 5132.883 | | | 48.92188 | |  | |  | |  |
| 6.233333 | | 96.1166 | | | | -0.55604 | | | | 5132.516 | | | 48.58594 | |  | |  | |  |
| 6.241667 | | 96.19225 | | | | -0.55585 | | | | 5132.133 | | | 48.39063 | |  | |  | |  |
| 6.25 | | 96.27287 | | | | -0.55577 | | | | 5131.758 | | | 48.27344 | |  | |  | |  |
| 6.258333 | | 96.35548 | | | | -0.55478 | | | | 5131.352 | | | 48.35938 | |  | |  | |  |
| 6.266667 | | 96.43809 | | | | -0.55351 | | | | 5130.992 | | | 48.46875 | |  | |  | |  |
| 6.275 | | 96.52269 | | | | -0.55326 | | | | 5130.602 | | | 48.45313 | |  | |  | |  |
| 6.283333 | | 96.60431 | | | | -0.55304 | | | | 5130.25 | | | 48.48438 | |  | |  | |  |
| 6.291667 | | 96.68393 | | | | -0.55247 | | | | 5129.867 | | | 48.35938 | |  | |  | |  |
| 6.3 | | 96.76555 | | | | -0.55237 | | | | 5129.477 | | | 48.35938 | |  | |  | |  |
| 6.308333 | | 96.84816 | | | | -0.55157 | | | | 5129.063 | | | 48.24219 | |  | |  | |  |
| 6.316667 | | 96.93176 | | | | -0.55034 | | | | 5128.648 | | | 48.10156 | |  | |  | |  |
| 6.325 | | 97.01139 | | | | -0.54997 | | | | 5128.242 | | | 47.9375 | |  | |  | |  |
| 6.333333 | | 97.09301 | | | | -0.54942 | | | | 5127.828 | | | 47.92969 | |  | |  | |  |
| 6.341667 | | 97.17363 | | | | -0.54866 | | | | 5127.445 | | | 47.55469 | |  | |  | |  |
| 6.35 | | 97.25027 | | | | -0.54854 | | | | 5127.039 | | | 47.66406 | |  | |  | |  |
| 6.358333 | | 97.32292 | | | | -0.54796 | | | | 5126.664 | | | 47.76563 | |  | |  | |  |
| 6.366667 | | 97.40056 | | | | -0.54758 | | | | 5126.266 | | | 47.53906 | |  | |  | |  |
| 6.375 | | 97.48218 | | | | -0.54698 | | | | 5125.883 | | | 47.5625 | |  | |  | |  |
| 6.383333 | | 97.56479 | | | | -0.54535 | | | | 5125.492 | | | 47.57031 | |  | |  | |  |
| 6.391667 | | 97.64441 | | | | -0.54537 | | | | 5125.148 | | | 47.49219 | |  | |  | |  |
| 6.4 | | 97.72105 | | | | -0.5456 | | | | 5124.727 | | | 47.4375 | |  | |  | |  |
| 6.408333 | | 97.80466 | | | | -0.5443 | | | | 5124.313 | | | 47.15625 | |  | |  | |  |
| 6.416667 | | 97.88528 | | | | -0.54411 | | | | 5123.938 | | | 47.03125 | |  | |  | |  |
| 6.425 | | 97.96291 | | | | -0.54461 | | | | 5123.539 | | | 47.03125 | |  | |  | |  |
| 6.433333 | | 98.04552 | | | | -0.54366 | | | | 5123.141 | | | 46.78125 | |  | |  | |  |
| 6.441667 | | 98.13113 | | | | -0.54314 | | | | 5122.75 | | | 46.46875 | |  | |  | |  |
| 6.45 | | 98.21771 | | | | -0.54226 | | | | 5122.359 | | | 46.54688 | |  | |  | |  |
| 6.458333 | | 98.29933 | | | | -0.54046 | | | | 5122 | | | 46.42969 | |  | |  | |  |
| 6.466667 | | 98.37796 | | | | -0.53982 | | | | 5121.617 | | | 46.32813 | |  | |  | |  |
| 6.475 | | 98.45759 | | | | -0.5395 | | | | 5121.227 | | | 46.32813 | |  | |  | |  |
| 6.483333 | | 98.54119 | | | | -0.53824 | | | | 5120.859 | | | 46.11719 | |  | |  | |  |
| 6.491667 | | 98.6248 | | | | -0.53766 | | | | 5120.516 | | | 46.34375 | |  | |  | |  |
| 6.5 | | 98.70641 | | | | -0.53797 | | | | 5120.109 | | | 46.19531 | |  | |  | |  |
| 6.508333 | | 98.79301 | | | | -0.5367 | | | | 5119.742 | | | 46.07031 | |  | |  | |  |
| 6.516667 | | 98.87463 | | | | -0.53613 | | | | 5119.359 | | | 46.21094 | |  | |  | |  |
| 6.525 | | 98.95724 | | | | -0.53639 | | | | 5118.977 | | | 46.28125 | |  | |  | |  |
| 6.533333 | | 99.03487 | | | | -0.53528 | | | | 5118.609 | | | 46.19531 | |  | |  | |  |
| 6.541667 | | 99.11549 | | | | -0.53433 | | | | 5118.195 | | | 46.46094 | |  | |  | |  |
| 6.55 | | 99.1991 | | | | -0.53386 | | | | 5117.828 | | | 46.49219 | |  | |  | |  |
| 6.558333 | | 99.27972 | | | | -0.53254 | | | | 5117.453 | | | 46.40625 | |  | |  | |  |
| 6.566667 | | 99.36034 | | | | -0.53101 | | | | 5117.047 | | | 46.47656 | |  | |  | |  |
| 6.575 | | 99.44196 | | | | -0.5306 | | | | 5116.648 | | | 46.42969 | |  | |  | |  |
| 6.583333 | | 99.52058 | | | | -0.52983 | | | | 5116.281 | | | 46.50781 | |  | |  | |  |
| 6.591667 | | 99.59723 | | | | -0.52948 | | | | 5115.852 | | | 46.30469 | |  | |  | |  |
| 6.6 | | 99.67884 | | | | -0.5295 | | | | 5115.461 | | | 46.15625 | |  | |  | |  |
| 6.608333 | | 99.76145 | | | | -0.52834 | | | | 5115.086 | | | 46.17969 | |  | |  | |  |
| 6.616667 | | 99.84406 | | | | -0.52761 | | | | 5114.688 | | | 46.07031 | |  | |  | |  |
| 6.625 | | 99.92369 | | | | -0.52728 | | | | 5114.313 | | | 45.66406 | |  | |  | |  |
| 6.633333 | | 100.0053 | | | | -0.52599 | | | | 5113.914 | | | 45.53125 | |  | |  | |  |
| 6.641667 | | 100.0839 | | | | -0.52576 | | | | 5113.555 | | | 45.80469 | |  | |  | |  |
| 6.65 | | 100.1656 | | | | -0.52548 | | | | 5113.195 | | | 45.67969 | |  | |  | |  |
| 6.658333 | | 100.2442 | | | | -0.52442 | | | | 5112.797 | | | 45.67188 | |  | |  | |  |
| 6.666667 | | 100.3268 | | | | -0.52343 | | | | 5112.43 | | | 45.5 | |  | |  | |  |
| 6.675 | | 100.4104 | | | | -0.52266 | | | | 5112.102 | | | 45.60156 | |  | |  | |  |
| 6.683333 | | 100.494 | | | | -0.52085 | | | | 5111.734 | | | 45.64063 | |  | |  | |  |
| 6.691667 | | 100.5716 | | | | -0.52034 | | | | 5111.32 | | | 45.53125 | |  | |  | |  |
| 6.7 | | 100.6513 | | | | -0.51981 | | | | 5110.953 | | | 45.58594 | |  | |  | |  |
| 6.708333 | | 100.7349 | | | | -0.5186 | | | | 5110.57 | | | 45.45313 | |  | |  | |  |
| 6.716667 | | 100.8195 | | | | -0.51774 | | | | 5110.219 | | | 45.375 | |  | |  | |  |
| 6.725 | | 100.9041 | | | | -0.51634 | | | | 5109.82 | | | 45.16406 | |  | |  | |  |
| 6.733333 | | 100.9867 | | | | -0.51458 | | | | 5109.438 | | | 45.24219 | |  | |  | |  |
| 6.741667 | | 101.0683 | | | | -0.51374 | | | | 5109.07 | | | 45.32031 | |  | |  | |  |
| 6.75 | | 101.1529 | | | | -0.51309 | | | | 5108.688 | | | 45.39063 | |  | |  | |  |
| 6.758333 | | 101.2405 | | | | -0.51172 | | | | 5108.32 | | | 45.40625 | |  | |  | |  |
| 6.766667 | | 101.3201 | | | | -0.51127 | | | | 5107.953 | | | 45.375 | |  | |  | |  |
| 6.775 | | 101.4037 | | | | -0.51073 | | | | 5107.602 | | | 45.25 | |  | |  | |  |
| 6.783333 | | 101.4863 | | | | -0.50914 | | | | 5107.203 | | | 45.20313 | |  | |  | |  |
| 6.791667 | | 101.5699 | | | | -0.50784 | | | | 5106.82 | | | 45.14844 | |  | |  | |  |
| 6.8 | | 101.6506 | | | | -0.50753 | | | | 5106.43 | | | 44.94531 | |  | |  | |  |
| 6.808333 | | 101.7352 | | | | -0.50623 | | | | 5106.055 | | | 44.96094 | |  | |  | |  |
| 6.816667 | | 101.8178 | | | | -0.50505 | | | | 5105.68 | | | 44.76563 | |  | |  | |  |
| 6.825 | | 101.9034 | | | | -0.50456 | | | | 5105.32 | | | 44.57031 | |  | |  | |  |
| 6.833333 | | 101.984 | | | | -0.50339 | | | | 5104.945 | | | 44.61719 | |  | |  | |  |
| 6.841667 | | 102.0616 | | | | -0.5023 | | | | 5104.586 | | | 44.53125 | |  | |  | |  |
| 6.85 | | 102.1452 | | | | -0.50114 | | | | 5104.227 | | | 44.39063 | |  | |  | |  |
| 6.858333 | | 102.2298 | | | | -0.49996 | | | | 5103.859 | | | 44.47656 | |  | |  | |  |
| 6.866667 | | 102.3154 | | | | -0.49979 | | | | 5103.508 | | | 44.21094 | |  | |  | |  |
| 6.875 | | 102.3921 | | | | -0.49937 | | | | 5103.164 | | | 44.30469 | |  | |  | |  |
| 6.883333 | | 102.4727 | | | | -0.49814 | | | | 5102.781 | | | 44.29688 | |  | |  | |  |
| 6.891667 | | 102.5543 | | | | -0.4974 | | | | 5102.43 | | | 44.25781 | |  | |  | |  |
| 6.9 | | 102.6369 | | | | -0.4962 | | | | 5102.07 | | | 44.30469 | |  | |  | |  |
| 6.908333 | | 102.7205 | | | | -0.49369 | | | | 5101.695 | | | 44.13281 | |  | |  | |  |
| 6.916667 | | 102.8041 | | | | -0.49253 | | | | 5101.352 | | | 44.14063 | |  | |  | |  |
| 6.925 | | 102.8877 | | | | -0.49191 | | | | 5100.961 | | | 44.1875 | |  | |  | |  |
| 6.933333 | | 102.9704 | | | | -0.49066 | | | | 5100.602 | | | 44.05469 | |  | |  | |  |
| 6.941667 | | 103.054 | | | | -0.48871 | | | | 5100.234 | | | 43.98438 | |  | |  | |  |
| 6.95 | | 103.1366 | | | | -0.48795 | | | | 5099.859 | | | 43.94531 | |  | |  | |  |
| 6.958333 | | 103.2222 | | | | -0.48639 | | | | 5099.508 | | | 43.60938 | |  | |  | |  |
| 6.966667 | | 103.3038 | | | | -0.485 | | | | 5099.133 | | | 43.55469 | |  | |  | |  |
| 6.975 | | 103.3884 | | | | -0.48425 | | | | 5098.758 | | | 43.58594 | |  | |  | |  |
| 6.983333 | | 103.472 | | | | -0.48295 | | | | 5098.406 | | | 43.30469 | |  | |  | |  |
| 6.991667 | | 103.5496 | | | | -0.48235 | | | | 5098.055 | | | 43.21875 | |  | |  | |  |
| 7 | | 103.6332 | | | | -0.48153 | | | | 5097.688 | | | 43.125 | |  | |  | |  |
| 7.008333 | | 103.7159 | | | | -0.47962 | | | | 5097.367 | | | 43.07031 | |  | |  | |  |
| 7.016667 | | 103.7985 | | | | -0.4789 | | | | 5097.016 | | | 43.13281 | |  | |  | |  |
| 7.025 | | 103.8781 | | | | -0.47847 | | | | 5096.656 | | | 42.95313 | |  | |  | |  |
| 7.033333 | | 103.9587 | | | | -0.47712 | | | | 5096.328 | | | 42.92969 | |  | |  | |  |
| 7.041667 | | 104.0413 | | | | -0.47689 | | | | 5095.977 | | | 42.73438 | |  | |  | |  |
| 7.05 | | 104.1263 | | | | -0.47608 | | | | 5095.633 | | | 42.88281 | |  | |  | |  |
| 7.058333 | | 104.2078 | | | | -0.47409 | | | | 5095.273 | | | 42.58594 | |  | |  | |  |
| 7.066667 | | 104.2894 | | | | -0.47291 | | | | 5094.906 | | | 42.45313 | |  | |  | |  |
| 7.075 | | 104.3749 | | | | -0.47091 | | | | 5094.57 | | | 42.54688 | |  | |  | |  |
| 7.083333 | | 104.4594 | | | | -0.46836 | | | | 5094.219 | | | 42.33594 | |  | |  | |  |
| 7.091667 | | 104.543 | | | | -0.46694 | | | | 5093.883 | | | 42.23438 | |  | |  | |  |
| 7.1 | | 104.6295 | | | | -0.4662 | | | | 5093.5 | | | 42.13281 | |  | |  | |  |
| 7.108333 | | 104.7179 | | | | -0.46441 | | | | 5093.188 | | | 42.26563 | |  | |  | |  |
| 7.116667 | | 104.8025 | | | | -0.46295 | | | | 5092.844 | | | 42.23438 | |  | |  | |  |
| 7.125 | | 104.888 | | | | -0.46188 | | | | 5092.484 | | | 42.35156 | |  | |  | |  |
| 7.133333 | | 104.9705 | | | | -0.45998 | | | | 5092.156 | | | 42.25781 | |  | |  | |  |
| 7.141667 | | 105.0501 | | | | -0.4582 | | | | 5091.813 | | | 42.375 | |  | |  | |  |
| 7.15 | | 105.1347 | | | | -0.45716 | | | | 5091.469 | | | 42.25781 | |  | |  | |  |
| 7.158333 | | 105.2202 | | | | -0.45582 | | | | 5091.102 | | | 42.10938 | |  | |  | |  |
| 7.166667 | | 105.3047 | | | | -0.45451 | | | | 5090.75 | | | 41.98438 | |  | |  | |  |
| 7.175 | | 105.3892 | | | | -0.45367 | | | | 5090.383 | | | 42.0625 | |  | |  | |  |
| 7.183333 | | 105.4728 | | | | -0.45271 | | | | 5090.031 | | | 41.85156 | |  | |  | |  |
| 7.191667 | | 105.5544 | | | | -0.45177 | | | | 5089.664 | | | 41.75781 | |  | |  | |  |
| 7.2 | | 105.6359 | | | | -0.45137 | | | | 5089.328 | | | 41.72656 | |  | |  | |  |
| 7.208333 | | 105.7205 | | | | -0.45013 | | | | 5088.992 | | | 41.73438 | |  | |  | |  |
| 7.216667 | | 105.807 | | | | -0.44826 | | | | 5088.656 | | | 41.5625 | |  | |  | |  |
| 7.225 | | 105.8964 | | | | -0.44625 | | | | 5088.297 | | | 41.42969 | |  | |  | |  |
| 7.233333 | | 105.9819 | | | | -0.44481 | | | | 5087.977 | | | 41.59375 | |  | |  | |  |
| 7.241667 | | 106.0664 | | | | -0.44274 | | | | 5087.633 | | | 41.55469 | |  | |  | |  |
| 7.25 | | 106.1637 | | | | -0.44124 | | | | 5087.234 | | | 41.5 | |  | |  | |  |
| 7.258333 | | 106.2286 | | | | -0.44126 | | | | 5086.953 | | | 41.25 | |  | |  | |  |
| 7.266667 | | 106.3112 | | | | -0.44022 | | | | 5086.625 | | | 41.1875 | |  | |  | |  |
| 7.275 | | 106.3927 | | | | -0.43976 | | | | 5086.289 | | | 41.03906 | |  | |  | |  |
| 7.283333 | | 106.4763 | | | | -0.43867 | | | | 5085.922 | | | 40.9375 | |  | |  | |  |
| 7.291667 | | 106.5736 | | | | -0.43682 | | | | 5085.516 | | | 40.67969 | |  | |  | |  |
| 7.3 | | 106.6552 | | | | -0.43511 | | | | 5085.164 | | | 40.46094 | |  | |  | |  |
| 7.308333 | | 106.7181 | | | | -0.4344 | | | | 5084.922 | | | 40.34375 | |  | |  | |  |
| 7.316667 | | 106.7987 | | | | -0.4322 | | | | 5084.586 | | | 40.24219 | |  | |  | |  |
| 7.325 | | 106.8802 | | | | -0.43115 | | | | 5084.258 | | | 39.96094 | |  | |  | |  |
| 7.333333 | | 106.9628 | | | | -0.42983 | | | | 5083.93 | | | 39.61719 | |  | |  | |  |
| 7.341667 | | 107.0473 | | | | -0.42821 | | | | 5083.625 | | | 39.47656 | |  | |  | |  |
| 7.35 | | 107.1299 | | | | -0.42727 | | | | 5083.313 | | | 39.5625 | |  | |  | |  |
| 7.358333 | | 107.2282 | | | | -0.42575 | | | | 5082.93 | | | 39.59375 | |  | |  | |  |
| 7.366667 | | 107.296 | | | | -0.42367 | | | | 5082.68 | | | 39.8125 | |  | |  | |  |
| 7.375 | | 107.3795 | | | | -0.42251 | | | | 5082.383 | | | 39.9375 | |  | |  | |  |
| 7.383333 | | 107.465 | | | | -0.42079 | | | | 5082.102 | | | 39.94531 | |  | |  | |  |
| 7.391667 | | 107.5506 | | | | -0.41923 | | | | 5081.781 | | | 39.96875 | |  | |  | |  |
| 7.4 | | 107.6518 | | | | -0.41758 | | | | 5081.383 | | | 39.85938 | |  | |  | |  |
| 7.408333 | | 107.7344 | | | | -0.41751 | | | | 5081.039 | | | 40.17188 | |  | |  | |  |
| 7.416667 | | 107.8022 | | | | -0.41615 | | | | 5080.742 | | | 40.50781 | |  | |  | |  |
| 7.425 | | 107.8847 | | | | -0.41569 | | | | 5080.398 | | | 40.42188 | |  | |  | |  |
| 7.433333 | | 107.9683 | | | | -0.41444 | | | | 5080.055 | | | 40.5625 | |  | |  | |  |
| 7.441667 | | 108.0518 | | | | -0.41326 | | | | 5079.719 | | | 40.52344 | |  | |  | |  |
| 7.45 | | 108.1383 | | | | -0.41179 | | | | 5079.398 | | | 40.375 | |  | |  | |  |
| 7.458333 | | 108.2396 | | | | -0.41013 | | | | 5078.953 | | | 40.14063 | |  | |  | |  |
| 7.466667 | | 108.3054 | | | | -0.40877 | | | | 5078.641 | | | 40.19531 | |  | |  | |  |
| 7.475 | | 108.4066 | | | | -0.40694 | | | | 5078.227 | | | 40.26563 | |  | |  | |  |
| 7.483333 | | 108.4912 | | | | -0.40603 | | | | 5077.867 | | | 40.25 | |  | |  | |  |
| 7.491667 | | 108.559 | | | | -0.40458 | | | | 5077.617 | | | 40.24219 | |  | |  | |  |
| 7.5 | | 108.6632 | | | | -0.40206 | | | | 5077.242 | | | 40 | |  | |  | |  |
| 7.508333 | | 108.732 | | | | -0.40113 | | | | 5076.977 | | | 40.20313 | |  | |  | |  |
| 7.516667 | | 108.8185 | | | | -0.39902 | | | | 5076.648 | | | 39.89844 | |  | |  | |  |
| 7.525 | | 108.9217 | | | | -0.39646 | | | | 5076.234 | | | 39.77344 | |  | |  | |  |
| 7.533333 | | 109.0052 | | | | -0.39591 | | | | 5075.891 | | | 39.86719 | |  | |  | |  |
| 7.541667 | | 109.0711 | | | | -0.39467 | | | | 5075.625 | | | 39.75781 | |  | |  | |  |
| 7.55 | | 109.1566 | | | | -0.39344 | | | | 5075.313 | | | 39.64063 | |  | |  | |  |
| 7.558333 | | 109.2411 | | | | -0.39218 | | | | 5074.953 | | | 39.85938 | |  | |  | |  |
| 7.566667 | | 109.3453 | | | | -0.39019 | | | | 5074.609 | | | 39.67188 | |  | |  | |  |
| 7.575 | | 109.4298 | | | | -0.3885 | | | | 5074.281 | | | 39.58594 | |  | |  | |  |
| 7.583333 | | 109.4966 | | | | -0.38774 | | | | 5074.008 | | | 39.375 | |  | |  | |  |
| 7.591667 | | 109.5802 | | | | -0.38578 | | | | 5073.695 | | | 39.125 | |  | |  | |  |
| 7.6 | | 109.6795 | | | | -0.38366 | | | | 5073.289 | | | 38.97656 | |  | |  | |  |
| 7.608333 | | 109.7433 | | | | -0.38364 | | | | 5073.016 | | | 39 | |  | |  | |  |
| 7.616667 | | 109.8465 | | | | -0.38131 | | | | 5072.641 | | | 38.83594 | |  | |  | |  |
| 7.625 | | 109.9163 | | | | -0.37934 | | | | 5072.391 | | | 38.82031 | |  | |  | |  |
| 7.633333 | | 110.0028 | | | | -0.378 | | | | 5072.086 | | | 38.58594 | |  | |  | |  |
| 7.641667 | | 110.1021 | | | | -0.37657 | | | | 5071.734 | | | 38.28125 | |  | |  | |  |
| 7.65 | | 110.167 | | | | -0.375 | | | | 5071.469 | | | 38.23438 | |  | |  | |  |
| 7.658333 | | 110.2643 | | | | -0.37348 | | | | 5071.109 | | | 38.03125 | |  | |  | |  |
| 7.666667 | | 110.3311 | | | | -0.37217 | | | | 5070.859 | | | 37.95313 | |  | |  | |  |
| 7.675 | | 110.4323 | | | | -0.37094 | | | | 5070.477 | | | 37.92188 | |  | |  | |  |
| 7.683333 | | 110.5188 | | | | -0.37059 | | | | 5070.195 | | | 37.79688 | |  | |  | |  |
| 7.691667 | | 110.5876 | | | | -0.36969 | | | | 5069.969 | | | 37.64063 | |  | |  | |  |
| 7.7 | | 110.6692 | | | | -0.3686 | | | | 5069.656 | | | 37.46094 | |  | |  | |  |
| 7.708333 | | 110.7675 | | | | -0.36766 | | | | 5069.313 | | | 37.59375 | |  | |  | |  |
| 7.716667 | | 110.8343 | | | | -0.36582 | | | | 5069.07 | | | 37.5625 | |  | |  | |  |
| 7.725 | | 110.9365 | | | | -0.36287 | | | | 5068.695 | | | 37.52344 | |  | |  | |  |
| 7.733333 | | 111.0044 | | | | -0.36191 | | | | 5068.469 | | | 37.51563 | |  | |  | |  |
| 7.741667 | | 111.0909 | | | | -0.35999 | | | | 5068.172 | | | 37.38281 | |  | |  | |  |
| 7.75 | | 111.1921 | | | | -0.35752 | | | | 5067.82 | | | 37.63281 | |  | |  | |  |
| 7.758333 | | 111.2766 | | | | -0.35624 | | | | 5067.484 | | | 37.49219 | |  | |  | |  |
| 7.766667 | | 111.3582 | | | | -0.3544 | | | | 5067.164 | | | 37.58594 | |  | |  | |  |
| 7.775 | | 111.426 | | | | -0.35203 | | | | 5066.922 | | | 37.64063 | |  | |  | |  |
| 7.783333 | | 111.5312 | | | | -0.35013 | | | | 5066.555 | | | 37.58594 | |  | |  | |  |
| 7.791667 | | 111.601 | | | | -0.34903 | | | | 5066.313 | | | 37.34375 | |  | |  | |  |
| 7.8 | | 111.6845 | | | | -0.34717 | | | | 5065.969 | | | 37.20313 | |  | |  | |  |
| 7.808333 | | 111.7838 | | | | -0.34617 | | | | 5065.594 | | | 37.05469 | |  | |  | |  |
| 7.816667 | | 111.8526 | | | | -0.34521 | | | | 5065.313 | | | 36.94531 | |  | |  | |  |
| 7.825 | | 111.9381 | | | | -0.34328 | | | | 5065 | | | 36.89063 | |  | |  | |  |
| 7.833333 | | 112.0403 | | | | -0.34175 | | | | 5064.625 | | | 36.60938 | |  | |  | |  |
| 7.841667 | | 112.1101 | | | | -0.34054 | | | | 5064.414 | | | 36.5625 | |  | |  | |  |
| 7.85 | | 112.2123 | | | | -0.33831 | | | | 5064.063 | | | 36.42969 | |  | |  | |  |
| 7.858333 | | 112.2841 | | | | -0.33713 | | | | 5063.828 | | | 36.26563 | |  | |  | |  |
| 7.866667 | | 112.3715 | | | | -0.3353 | | | | 5063.531 | | | 36.25781 | |  | |  | |  |
| 7.875 | | 112.4728 | | | | -0.33294 | | | | 5063.18 | | | 36.11719 | |  | |  | |  |
| 7.883333 | | 112.5416 | | | | -0.33156 | | | | 5062.953 | | | 35.88281 | |  | |  | |  |
| 7.891667 | | 112.63 | | | | -0.33033 | | | | 5062.656 | | | 35.78906 | |  | |  | |  |
| 7.9 | | 112.7185 | | | | -0.32773 | | | | 5062.367 | | | 35.84375 | |  | |  | |  |
| 7.908333 | | 112.8227 | | | | -0.32572 | | | | 5062.031 | | | 35.69531 | |  | |  | |  |
| 7.916667 | | 112.8915 | | | | -0.32473 | | | | 5061.789 | | | 35.60938 | |  | |  | |  |
| 7.925 | | 112.977 | | | | -0.32224 | | | | 5061.508 | | | 35.69531 | |  | |  | |  |
| 7.933333 | | 113.0831 | | | | -0.31989 | | | | 5061.164 | | | 35.60938 | |  | |  | |  |
| 7.941667 | | 113.148 | | | | -0.31977 | | | | 5060.945 | | | 35.73438 | |  | |  | |  |
| 7.95 | | 113.2276 | | | | -0.31792 | | | | 5060.641 | | | 35.55469 | |  | |  | |  |
| 7.958333 | | 113.3259 | | | | -0.31603 | | | | 5060.289 | | | 35.5 | |  | |  | |  |
| 7.966667 | | 113.3947 | | | | -0.31525 | | | | 5060.055 | | | 35.66406 | |  | |  | |  |
| 7.975 | | 113.4802 | | | | -0.31298 | | | | 5059.758 | | | 35.92188 | |  | |  | |  |
| 7.983333 | | 113.5647 | | | | -0.31155 | | | | 5059.477 | | | 35.96094 | |  | |  | |  |
| 7.991667 | | 113.665 | | | | -0.31016 | | | | 5059.102 | | | 36.08594 | |  | |  | |  |
| 8 | | 113.7515 | | | | -0.30827 | | | | 5058.813 | | | 35.96875 | |  | |  | |  |
| 8.008333 | | 113.8203 | | | | -0.30653 | | | | 5058.578 | | | 35.875 | |  | |  | |  |
| 8.016667 | | 113.9058 | | | | -0.30509 | | | | 5058.273 | | | 35.94531 | |  | |  | |  |
| 8.025 | | 113.9962 | | | | -0.30212 | | | | 5057.93 | | | 35.73438 | |  | |  | |  |
| 8.033333 | | 114.0866 | | | | -0.30011 | | | | 5057.625 | | | 35.875 | |  | |  | |  |
| 8.041667 | | 114.1751 | | | | -0.29895 | | | | 5057.32 | | | 35.86719 | |  | |  | |  |
| 8.05 | | 114.2793 | | | | -0.29714 | | | | 5056.969 | | | 35.80469 | |  | |  | |  |
| 8.058333 | | 114.3481 | | | | -0.29535 | | | | 5056.734 | | | 35.44531 | |  | |  | |  |
| 8.066667 | | 114.4306 | | | | -0.2947 | | | | 5056.43 | | | 35.32813 | |  | |  | |  |
| 8.075 | | 114.5338 | | | | -0.29234 | | | | 5056.094 | | | 35.13281 | |  | |  | |  |
| 8.083333 | | 114.6184 | | | | -0.29028 | | | | 5055.789 | | | 34.92969 | |  | |  | |  |
| 8.091667 | | 114.7049 | | | | -0.28844 | | | | 5055.477 | | | 34.78125 | |  | |  | |  |
| 8.1 | | 114.7923 | | | | -0.28729 | | | | 5055.195 | | | 34.64063 | |  | |  | |  |
| 8.108333 | | 114.8728 | | | | -0.28504 | | | | 5054.953 | | | 34.67969 | |  | |  | |  |
| 8.116667 | | 114.9581 | | | | -0.2827 | | | | 5054.664 | | | 34.50781 | |  | |  | |  |
| 8.125 | | 115.0415 | | | | -0.2813 | | | | 5054.406 | | | 34.23438 | |  | |  | |  |
| 8.133333 | | 115.1259 | | | | -0.27933 | | | | 5054.133 | | | 34.10938 | |  | |  | |  |
| 8.141667 | | 115.2094 | | | | -0.27757 | | | | 5053.859 | | | 34.125 | |  | |  | |  |
| 8.15 | | 115.2975 | | | | -0.27635 | | | | 5053.586 | | | 33.76563 | |  | |  | |  |
| 8.158333 | | 115.3838 | | | | -0.27427 | | | | 5053.289 | | | 33.55469 | |  | |  | |  |
| 8.166667 | | 115.4511 | | | | -0.27252 | | | | 5053.078 | | | 33.70313 | |  | |  | |  |
| 8.175 | | 115.5308 | | | | -0.27109 | | | | 5052.82 | | | 33.54688 | |  | |  | |  |
| 8.183333 | | 115.6142 | | | | -0.26907 | | | | 5052.547 | | | 33.375 | |  | |  | |  |
| 8.191667 | | 115.7147 | | | | -0.26673 | | | | 5052.219 | | | 33.32813 | |  | |  | |  |
| 8.2 | | 115.7953 | | | | -0.2657 | | | | 5051.977 | | | 33.34375 | |  | |  | |  |
| 8.208333 | | 115.8607 | | | | -0.26432 | | | | 5051.773 | | | 33.49219 | |  | |  | |  |
| 8.216667 | | 115.9593 | | | | -0.26177 | | | | 5051.43 | | | 33.23438 | |  | |  | |  |
| 8.225 | | 116.0247 | | | | -0.26058 | | | | 5051.227 | | | 33.32031 | |  | |  | |  |
| 8.233333 | | 116.1224 | | | | -0.25783 | | | | 5050.914 | | | 33.35938 | |  | |  | |  |
| 8.241667 | | 116.1878 | | | | -0.25589 | | | | 5050.695 | | | 33.55469 | |  | |  | |  |
| 8.25 | | 116.2703 | | | | -0.2546 | | | | 5050.414 | | | 33.375 | |  | |  | |  |
| 8.258333 | | 116.3585 | | | | -0.25278 | | | | 5050.125 | | | 33.53906 | |  | |  | |  |
| 8.266667 | | 116.4419 | | | | -0.25074 | | | | 5049.875 | | | 33.54688 | |  | |  | |  |
| 8.275 | | 116.5253 | | | | -0.24995 | | | | 5049.594 | | | 33.39063 | |  | |  | |  |
| 8.283333 | | 116.6078 | | | | -0.24866 | | | | 5049.305 | | | 33.21094 | |  | |  | |  |
| 8.291667 | | 116.7074 | | | | -0.24606 | | | | 5048.938 | | | 33.07813 | |  | |  | |  |
| 8.3 | | 116.7756 | | | | -0.24458 | | | | 5048.734 | | | 33.05469 | |  | |  | |  |
| 8.308333 | | 116.8591 | | | | -0.24267 | | | | 5048.43 | | | 33.13281 | |  | |  | |  |
| 8.316667 | | 116.9558 | | | | -0.23988 | | | | 5048.109 | | | 33.05469 | |  | |  | |  |
| 8.325 | | 117.0212 | | | | -0.23825 | | | | 5047.891 | | | 32.89844 | |  | |  | |  |
| 8.333333 | | 117.1046 | | | | -0.23702 | | | | 5047.648 | | | 32.86719 | |  | |  | |  |
| 8.341667 | | 117.1871 | | | | -0.23481 | | | | 5047.383 | | | 32.67188 | |  | |  | |  |
| 8.35 | | 117.2867 | | | | -0.23299 | | | | 5047.055 | | | 32.57813 | |  | |  | |  |
| 8.358333 | | 117.3692 | | | | -0.23167 | | | | 5046.766 | | | 32.46094 | |  | |  | |  |
| 8.366667 | | 117.4554 | | | | -0.22903 | | | | 5046.492 | | | 32.30469 | |  | |  | |  |
| 8.375 | | 117.5398 | | | | -0.22712 | | | | 5046.227 | | | 32.23438 | |  | |  | |  |
| 8.383333 | | 117.6261 | | | | -0.22571 | | | | 5045.977 | | | 32.03125 | |  | |  | |  |
| 8.391667 | | 117.6972 | | | | -0.22385 | | | | 5045.773 | | | 31.66406 | |  | |  | |  |
| 8.4 | | 117.7835 | | | | -0.22287 | | | | 5045.508 | | | 31.45313 | |  | |  | |  |
| 8.408333 | | 117.8868 | | | | -0.22125 | | | | 5045.188 | | | 31.39063 | |  | |  | |  |
| 8.416667 | | 117.9693 | | | | -0.21991 | | | | 5044.938 | | | 31.1875 | |  | |  | |  |
| 8.425 | | 118.0385 | | | | -0.21831 | | | | 5044.727 | | | 31.14063 | |  | |  | |  |
| 8.433333 | | 118.1409 | | | | -0.21725 | | | | 5044.438 | | | 30.97656 | |  | |  | |  |
| 8.441667 | | 118.2244 | | | | -0.2166 | | | | 5044.227 | | | 30.78125 | |  | |  | |  |
| 8.45 | | 118.2926 | | | | -0.21521 | | | | 5044.039 | | | 30.91406 | |  | |  | |  |
| 8.458333 | | 118.3789 | | | | -0.21397 | | | | 5043.781 | | | 30.82813 | |  | |  | |  |
| 8.466667 | | 118.4623 | | | | -0.21128 | | | | 5043.547 | | | 30.80469 | |  | |  | |  |
| 8.475 | | 118.542 | | | | -0.20856 | | | | 5043.297 | | | 30.92188 | |  | |  | |  |
| 8.483333 | | 118.6311 | | | | -0.20525 | | | | 5043.063 | | | 31.05469 | |  | |  | |  |
| 8.491667 | | 118.7126 | | | | -0.2027 | | | | 5042.82 | | | 30.72656 | |  | |  | |  |
| 8.5 | | 118.797 | | | | -0.20073 | | | | 5042.555 | | | 30.73438 | |  | |  | |  |
| 8.508333 | | 118.8786 | | | | -0.20003 | | | | 5042.305 | | | 30.71094 | |  | |  | |  |
| 8.516667 | | 118.9791 | | | | -0.19862 | | | | 5041.992 | | | 30.64844 | |  | |  | |  |
| 8.525 | | 119.0663 | | | | -0.197 | | | | 5041.719 | | | 30.78906 | |  | |  | |  |
| 8.533333 | | 119.1317 | | | | -0.19623 | | | | 5041.5 | | | 30.57813 | |  | |  | |  |
| 8.541667 | | 119.2142 | | | | -0.19407 | | | | 5041.281 | | | 30.47656 | |  | |  | |  |
| 8.55 | | 119.3147 | | | | -0.19131 | | | | 5040.961 | | | 30.19531 | |  | |  | |  |
| 8.558333 | | 119.382 | | | | -0.19025 | | | | 5040.773 | | | 30.28125 | |  | |  | |  |
| 8.566667 | | 119.4607 | | | | -0.18829 | | | | 5040.523 | | | 30.20313 | |  | |  | |  |
| 8.575 | | 119.564 | | | | -0.18569 | | | | 5040.195 | | | 30.16406 | |  | |  | |  |
| 8.583333 | | 119.6314 | | | | -0.18484 | | | | 5040.023 | | | 30.25 | |  | |  | |  |
| 8.591667 | | 119.7347 | | | | -0.18331 | | | | 5039.75 | | | 30.30469 | |  | |  | |  |
| 8.6 | | 119.821 | | | | -0.1812 | | | | 5039.508 | | | 30.28125 | |  | |  | |  |
| 8.608333 | | 119.8883 | | | | -0.17988 | | | | 5039.297 | | | 30.14063 | |  | |  | |  |
| 8.616667 | | 119.9879 | | | | -0.17854 | | | | 5039.008 | | | 30.05469 | |  | |  | |  |
| 8.625 | | 120.0703 | | | | -0.17647 | | | | 5038.75 | | | 30.25781 | |  | |  | |  |
| 8.633333 | | 120.1585 | | | | -0.17417 | | | | 5038.484 | | | 30.1875 | |  | |  | |  |
| 8.641667 | | 120.2287 | | | | -0.17292 | | | | 5038.273 | | | 30.22656 | |  | |  | |  |
| 8.65 | | 120.3178 | | | | -0.16999 | | | | 5038.023 | | | 30.07031 | |  | |  | |  |
| 8.658333 | | 120.4041 | | | | -0.16789 | | | | 5037.781 | | | 30.11719 | |  | |  | |  |
| 8.666667 | | 120.4885 | | | | -0.16676 | | | | 5037.539 | | | 30.03125 | |  | |  | |  |
| 8.675 | | 120.5709 | | | | -0.16453 | | | | 5037.266 | | | 29.92969 | |  | |  | |  |
| 8.683333 | | 120.6534 | | | | -0.16296 | | | | 5037.016 | | | 29.89844 | |  | |  | |  |
| 8.691667 | | 120.7397 | | | | -0.1622 | | | | 5036.758 | | | 29.99219 | |  | |  | |  |
| 8.7 | | 120.8269 | | | | -0.15978 | | | | 5036.523 | | | 29.92969 | |  | |  | |  |
| 8.708333 | | 120.9151 | | | | -0.15759 | | | | 5036.266 | | | 29.78125 | |  | |  | |  |
| 8.716667 | | 120.9976 | | | | -0.15686 | | | | 5036.023 | | | 29.94531 | |  | |  | |  |
| 8.725 | | 121.0953 | | | | -0.15395 | | | | 5035.734 | | | 29.67969 | |  | |  | |  |
| 8.733333 | | 121.1787 | | | | -0.1517 | | | | 5035.484 | | | 29.64063 | |  | |  | |  |
| 8.741667 | | 121.246 | | | | -0.15111 | | | | 5035.273 | | | 29.35156 | |  | |  | |  |
| 8.75 | | 121.3512 | | | | -0.14941 | | | | 5034.977 | | | 29.33594 | |  | |  | |  |
| 8.758333 | | 121.4214 | | | | -0.14742 | | | | 5034.797 | | | 29.25 | |  | |  | |  |
| 8.766667 | | 121.5048 | | | | -0.14717 | | | | 5034.531 | | | 28.96094 | |  | |  | |  |
| 8.775 | | 121.5911 | | | | -0.14555 | | | | 5034.32 | | | 29.07813 | |  | |  | |  |
| 8.783333 | | 121.6746 | | | | -0.14312 | | | | 5034.078 | | | 28.79688 | |  | |  | |  |
| 8.791667 | | 121.7751 | | | | -0.14088 | | | | 5033.805 | | | 28.625 | |  | |  | |  |
| 8.8 | | 121.8433 | | | | -0.13944 | | | | 5033.641 | | | 28.33594 | |  | |  | |  |
| 8.808333 | | 121.9305 | | | | -0.13647 | | | | 5033.406 | | | 28.21875 | |  | |  | |  |
| 8.816667 | | 122.0149 | | | | -0.13504 | | | | 5033.203 | | | 28.07031 | |  | |  | |  |
| 8.825 | | 122.1183 | | | | -0.13331 | | | | 5032.906 | | | 27.97656 | |  | |  | |  |
| 8.833333 | | 122.1875 | | | | -0.13142 | | | | 5032.75 | | | 28.07031 | |  | |  | |  |
| 8.841667 | | 122.269 | | | | -0.1308 | | | | 5032.531 | | | 27.90625 | |  | |  | |  |
| 8.85 | | 122.3544 | | | | -0.12966 | | | | 5032.336 | | | 27.71875 | |  | |  | |  |
| 8.858333 | | 122.4567 | | | | -0.12723 | | | | 5032.063 | | | 27.22656 | |  | |  | |  |
| 8.866667 | | 122.5279 | | | | -0.12555 | | | | 5031.898 | | | 27.30469 | |  | |  | |  |
| 8.875 | | 122.6151 | | | | -0.12375 | | | | 5031.664 | | | 27.09375 | |  | |  | |  |
| 8.883333 | | 122.7184 | | | | -0.12106 | | | | 5031.367 | | | 26.89063 | |  | |  | |  |
| 8.891667 | | 122.8 | | | | -0.1187 | | | | 5031.164 | | | 26.78906 | |  | |  | |  |
| 8.9 | | 122.8872 | | | | -0.11681 | | | | 5030.977 | | | 26.64844 | |  | |  | |  |
| 8.908333 | | 122.9716 | | | | -0.11557 | | | | 5030.789 | | | 26.48438 | |  | |  | |  |
| 8.916667 | | 123.055 | | | | -0.11377 | | | | 5030.555 | | | 26.22656 | |  | |  | |  |
| 8.925 | | 123.1413 | | | | -0.11268 | | | | 5030.352 | | | 26.3125 | |  | |  | |  |
| 8.933333 | | 123.2276 | | | | -0.11163 | | | | 5030.156 | | | 26.41406 | |  | |  | |  |
| 8.941667 | | 123.3101 | | | | -0.11002 | | | | 5029.945 | | | 26.27344 | |  | |  | |  |
| 8.95 | | 123.3925 | | | | -0.10823 | | | | 5029.742 | | | 26.09375 | |  | |  | |  |
| 8.958333 | | 123.476 | | | | -0.10625 | | | | 5029.539 | | | 26.24219 | |  | |  | |  |
| 8.966667 | | 123.5594 | | | | -0.10395 | | | | 5029.359 | | | 26.17969 | |  | |  | |  |
| 8.975 | | 123.6447 | | | | -0.10244 | | | | 5029.117 | | | 26.09375 | |  | |  | |  |
| 8.983333 | | 123.7282 | | | | -0.10079 | | | | 5028.883 | | | 26.13281 | |  | |  | |  |
| 8.991667 | | 123.8154 | | | | -0.09812 | | | | 5028.672 | | | 26.00781 | |  | |  | |  |
| 9 | | 123.8998 | | | | -0.09703 | | | | 5028.477 | | | 26.02344 | |  | |  | |  |
| 9.008333 | | 123.9908 | | | | -0.09496 | | | | 5028.242 | | | 25.89063 | |  | |  | |  |
| 9.016667 | | 124.0771 | | | | -0.09222 | | | | 5028.023 | | | 25.72656 | |  | |  | |  |
| 9.025 | | 124.1624 | | | | -0.0912 | | | | 5027.813 | | | 25.78906 | |  | |  | |  |
| 9.033333 | | 124.2468 | | | | -0.09024 | | | | 5027.586 | | | 25.72656 | |  | |  | |  |
| 9.041667 | | 124.3359 | | | | -0.08837 | | | | 5027.383 | | | 25.6875 | |  | |  | |  |
| 9.05 | | 124.425 | | | | -0.08582 | | | | 5027.156 | | | 25.89063 | |  | |  | |  |
| 9.058333 | | 124.5132 | | | | -0.08387 | | | | 5026.961 | | | 26 | |  | |  | |  |
| 9.066667 | | 124.5985 | | | | -0.08167 | | | | 5026.766 | | | 25.92188 | |  | |  | |  |
| 9.075 | | 124.6829 | | | | -0.0793 | | | | 5026.539 | | | 25.94531 | |  | |  | |  |
| 9.083333 | | 124.7702 | | | | -0.07795 | | | | 5026.328 | | | 25.92188 | |  | |  | |  |
| 9.091667 | | 124.8507 | | | | -0.07618 | | | | 5026.109 | | | 25.69531 | |  | |  | |  |
| 9.1 | | 124.9351 | | | | -0.07453 | | | | 5025.859 | | | 25.66406 | |  | |  | |  |
| 9.108333 | | 125.0224 | | | | -0.07291 | | | | 5025.625 | | | 25.625 | |  | |  | |  |
| 9.116667 | | 125.1096 | | | | -0.07051 | | | | 5025.422 | | | 25.47656 | |  | |  | |  |
| 9.125 | | 125.193 | | | | -0.0679 | | | | 5025.195 | | | 25.46094 | |  | |  | |  |
| 9.133333 | | 125.2749 | | | | -0.06659 | | | | 5024.984 | | | 25.17969 | |  | |  | |  |
| 9.141667 | | 125.3573 | | | | -0.06506 | | | | 5024.797 | | | 24.95313 | |  | |  | |  |
| 9.15 | | 125.4454 | | | | -0.06334 | | | | 5024.578 | | | 24.95313 | |  | |  | |  |
| 9.158333 | | 125.5306 | | | | -0.06228 | | | | 5024.383 | | | 24.90625 | |  | |  | |  |
| 9.166667 | | 125.6149 | | | | -0.06025 | | | | 5024.18 | | | 24.82031 | |  | |  | |  |
| 9.175 | | 125.6992 | | | | -0.05773 | | | | 5023.977 | | | 24.71875 | |  | |  | |  |
| 9.183333 | | 125.7891 | | | | -0.05528 | | | | 5023.805 | | | 24.72656 | |  | |  | |  |
| 9.191667 | | 125.8771 | | | | -0.05259 | | | | 5023.633 | | | 24.64844 | |  | |  | |  |
| 9.2 | | 125.9642 | | | | -0.04939 | | | | 5023.422 | | | 24.69531 | |  | |  | |  |
| 9.208333 | | 126.0475 | | | | -0.04814 | | | | 5023.219 | | | 24.54688 | |  | |  | |  |
| 9.216667 | | 126.1346 | | | | -0.04665 | | | | 5023.023 | | | 24.4375 | |  | |  | |  |
| 9.225 | | 126.2208 | | | | -0.04458 | | | | 5022.828 | | | 24.5625 | |  | |  | |  |
| 9.233333 | | 126.3032 | | | | -0.04323 | | | | 5022.625 | | | 24.58594 | |  | |  | |  |
| 9.241667 | | 126.3922 | | | | -0.04148 | | | | 5022.422 | | | 24.60938 | |  | |  | |  |
| 9.25 | | 126.4783 | | | | -0.03893 | | | | 5022.211 | | | 24.67969 | |  | |  | |  |
| 9.258333 | | 126.5655 | | | | -0.03685 | | | | 5022.039 | | | 24.72656 | |  | |  | |  |
| 9.266667 | | 126.646 | | | | -0.03635 | | | | 5021.836 | | | 24.59375 | |  | |  | |  |
| 9.275 | | 126.7284 | | | | -0.0341 | | | | 5021.609 | | | 24.5 | |  | |  | |  |
| 9.283333 | | 126.8136 | | | | -0.0326 | | | | 5021.406 | | | 24.35156 | |  | |  | |  |
| 9.291667 | | 126.9007 | | | | -0.03148 | | | | 5021.211 | | | 24.39063 | |  | |  | |  |
| 9.3 | | 126.9878 | | | | -0.0291 | | | | 5020.984 | | | 24.4375 | |  | |  | |  |
| 9.308333 | | 127.074 | | | | -0.02659 | | | | 5020.773 | | | 24.17969 | |  | |  | |  |
| 9.316667 | | 127.163 | | | | -0.02505 | | | | 5020.586 | | | 24.10938 | |  | |  | |  |
| 9.325 | | 127.2463 | | | | -0.02296 | | | | 5020.383 | | | 24.07813 | |  | |  | |  |
| 9.333333 | | 127.3315 | | | | -0.02058 | | | | 5020.203 | | | 23.53125 | |  | |  | |  |
| 9.341667 | | 127.4186 | | | | -0.01899 | | | | 5019.992 | | | 23.63281 | |  | |  | |  |
| 9.35 | | 127.5067 | | | | -0.01696 | | | | 5019.789 | | | 23.66406 | |  | |  | |  |
| 9.358333 | | 127.5947 | | | | -0.01476 | | | | 5019.617 | | | 23.44531 | |  | |  | |  |
| 9.366667 | | 127.6809 | | | | -0.01367 | | | | 5019.43 | | | 23.55469 | |  | |  | |  |
| 9.375 | | 127.767 | | | | -0.01179 | | | | 5019.234 | | | 23.5 | |  | |  | |  |
| 9.383333 | | 127.8494 | | | | -0.00942 | | | | 5019.102 | | | 23.28125 | |  | |  | |  |
| 9.391667 | | 127.9365 | | | | -0.00717 | | | | 5018.891 | | | 23.25781 | |  | |  | |  |
| 9.4 | | 128.0208 | | | | -0.00579 | | | | 5018.688 | | | 23.25781 | |  | |  | |  |
| 9.408333 | | 128.107 | | | | -0.00346 | | | | 5018.516 | | | 23.15625 | |  | |  | |  |
| 9.416667 | | 128.1941 | | | | -0.0011 | | | | 5018.313 | | | 23.25781 | |  | |  | |  |
| 9.425 | | 128.2784 | | | | 0.000426 | | | | 5018.117 | | | 23.16406 | |  | |  | |  |
| 9.433333 | | 128.3598 | | | | 0.002506 | | | | 5017.953 | | | 22.97656 | |  | |  | |  |
| 9.441667 | | 128.4441 | | | | 0.005035 | | | | 5017.758 | | | 23.03125 | |  | |  | |  |
| 9.45 | | 128.5266 | | | | 0.006021 | | | | 5017.555 | | | 23 | |  | |  | |  |
| 9.458333 | | 128.6155 | | | | 0.007951 | | | | 5017.375 | | | 22.89844 | |  | |  | |  |
| 9.466667 | | 128.7026 | | | | 0.010566 | | | | 5017.156 | | | 22.82813 | |  | |  | |  |
| 9.475 | | 128.7878 | | | | 0.011123 | | | | 5016.977 | | | 22.86719 | |  | |  | |  |
| 9.483333 | | 128.8759 | | | | 0.013152 | | | | 5016.813 | | | 22.85156 | |  | |  | |  |
| 9.491667 | | 128.962 | | | | 0.01633 | | | | 5016.617 | | | 22.99219 | |  | |  | |  |
| 9.5 | | 129.0482 | | | | 0.01802 | | | | 5016.43 | | | 22.80469 | |  | |  | |  |
| 9.508333 | | 129.1344 | | | | 0.020326 | | | | 5016.25 | | | 22.75 | |  | |  | |  |
| 9.516667 | | 129.2233 | | | | 0.023842 | | | | 5016.063 | | | 22.72656 | |  | |  | |  |
| 9.525 | | 129.3095 | | | | 0.025931 | | | | 5015.867 | | | 22.63281 | |  | |  | |  |
| 9.533333 | | 129.3956 | | | | 0.028104 | | | | 5015.688 | | | 22.54688 | |  | |  | |  |
| 9.541667 | | 129.4799 | | | | 0.030593 | | | | 5015.469 | | | 22.55469 | |  | |  | |  |
| 9.55 | | 129.567 | | | | 0.032031 | | | | 5015.297 | | | 22.41406 | |  | |  | |  |
| 9.558333 | | 129.6532 | | | | 0.033625 | | | | 5015.125 | | | 22.32031 | |  | |  | |  |
| 9.566667 | | 129.7375 | | | | 0.036145 | | | | 5014.938 | | | 22.02344 | |  | |  | |  |
| 9.575 | | 129.8218 | | | | 0.036921 | | | | 5014.758 | | | 21.875 | |  | |  | |  |
| 9.583333 | | 129.9023 | | | | 0.038053 | | | | 5014.578 | | | 21.73438 | |  | |  | |  |
| 9.591667 | | 129.9857 | | | | 0.040091 | | | | 5014.391 | | | 21.5 | |  | |  | |  |
| 9.6 | | 130.0718 | | | | 0.0405 | | | | 5014.219 | | | 21.36719 | |  | |  | |  |
| 9.608333 | | 130.1552 | | | | 0.041843 | | | | 5014.047 | | | 21.23438 | |  | |  | |  |
| 9.616667 | | 130.2432 | | | | 0.044532 | | | | 5013.906 | | | 21.14063 | |  | |  | |  |
| 9.625 | | 130.3275 | | | | 0.045448 | | | | 5013.742 | | | 20.92188 | |  | |  | |  |
| 9.633333 | | 130.4109 | | | | 0.046994 | | | | 5013.578 | | | 20.6875 | |  | |  | |  |
| 9.641667 | | 130.4942 | | | | 0.049925 | | | | 5013.422 | | | 20.59375 | |  | |  | |  |
| 9.65 | | 130.5794 | | | | 0.05145 | | | | 5013.266 | | | 20.64844 | |  | |  | |  |
| 9.658333 | | 130.6675 | | | | 0.053449 | | | | 5013.094 | | | 20.42188 | |  | |  | |  |
| 9.666667 | | 130.7555 | | | | 0.056176 | | | | 5012.93 | | | 20.35156 | |  | |  | |  |
| 9.675 | | 130.8473 | | | | 0.058389 | | | | 5012.781 | | | 20.34375 | |  | |  | |  |
| 9.683333 | | 130.9334 | | | | 0.060442 | | | | 5012.648 | | | 20.35156 | |  | |  | |  |
| 9.691667 | | 131.0215 | | | | 0.063465 | | | | 5012.484 | | | 20.17969 | |  | |  | |  |
| 9.7 | | 131.1067 | | | | 0.065381 | | | | 5012.297 | | | 20.00781 | |  | |  | |  |
| 9.708333 | | 131.1947 | | | | 0.067257 | | | | 5012.164 | | | 19.97656 | |  | |  | |  |
| 9.716667 | | 131.2818 | | | | 0.069994 | | | | 5012 | | | 20.03125 | |  | |  | |  |
| 9.725 | | 131.3699 | | | | 0.072009 | | | | 5011.82 | | | 20.0625 | |  | |  | |  |
| 9.733333 | | 131.457 | | | | 0.073619 | | | | 5011.656 | | | 19.86719 | |  | |  | |  |
| 9.741667 | | 131.5422 | | | | 0.075436 | | | | 5011.508 | | | 19.76563 | |  | |  | |  |
| 9.75 | | 131.6321 | | | | 0.077632 | | | | 5011.359 | | | 19.50781 | |  | |  | |  |
| 9.758333 | | 131.7154 | | | | 0.078577 | | | | 5011.195 | | | 19.28906 | |  | |  | |  |
| 9.766667 | | 131.8016 | | | | 0.080549 | | | | 5011.023 | | | 19.17969 | |  | |  | |  |
| 9.775 | | 131.8878 | | | | 0.083197 | | | | 5010.852 | | | 19.17188 | |  | |  | |  |
| 9.783333 | | 131.9749 | | | | 0.084986 | | | | 5010.711 | | | 19.00781 | |  | |  | |  |
| 9.791667 | | 132.0601 | | | | 0.086557 | | | | 5010.555 | | | 18.91406 | |  | |  | |  |
| 9.8 | | 132.1491 | | | | 0.089221 | | | | 5010.43 | | | 18.86719 | |  | |  | |  |
| 9.808333 | | 132.238 | | | | 0.090399 | | | | 5010.289 | | | 18.78906 | |  | |  | |  |
| 9.816667 | | 132.3261 | | | | 0.091797 | | | | 5010.141 | | | 18.79688 | |  | |  | |  |
| 9.825 | | 132.4132 | | | | 0.094363 | | | | 5009.977 | | | 18.8125 | |  | |  | |  |
| 9.833333 | | 132.5003 | | | | 0.095825 | | | | 5009.844 | | | 19.03125 | |  | |  | |  |
| 9.841667 | | 132.5892 | | | | 0.097193 | | | | 5009.688 | | | 18.88281 | |  | |  | |  |
| 9.85 | | 132.6763 | | | | 0.100275 | | | | 5009.539 | | | 18.79688 | |  | |  | |  |
| 9.858333 | | 132.7634 | | | | 0.102089 | | | | 5009.391 | | | 18.69531 | |  | |  | |  |
| 9.866667 | | 132.8477 | | | | 0.10282 | | | | 5009.227 | | | 18.95313 | |  | |  | |  |
| 9.875 | | 132.9357 | | | | 0.105771 | | | | 5009.07 | | | 18.96875 | |  | |  | |  |
| 9.883333 | | 133.021 | | | | 0.107468 | | | | 5008.883 | | | 18.82031 | |  | |  | |  |
| 9.891667 | | 133.109 | | | | 0.108721 | | | | 5008.727 | | | 18.77344 | |  | |  | |  |
| 9.9 | | 133.1933 | | | | 0.111735 | | | | 5008.578 | | | 18.82031 | |  | |  | |  |
| 9.908333 | | 133.2794 | | | | 0.114391 | | | | 5008.438 | | | 18.92188 | |  | |  | |  |
| 9.916667 | | 133.3647 | | | | 0.115884 | | | | 5008.242 | | | 18.89063 | |  | |  | |  |
| 9.925 | | 133.4499 | | | | 0.118762 | | | | 5008.086 | | | 18.89063 | |  | |  | |  |
| 9.933333 | | 133.5379 | | | | 0.120845 | | | | 5007.938 | | | 19.16406 | |  | |  | |  |
| 9.941667 | | 133.625 | | | | 0.121732 | | | | 5007.781 | | | 19.09375 | |  | |  | |  |
| 9.95 | | 133.714 | | | | 0.124291 | | | | 5007.617 | | | 19.02344 | |  | |  | |  |
| 9.958333 | | 133.7983 | | | | 0.126448 | | | | 5007.453 | | | 18.91406 | |  | |  | |  |
| 9.966667 | | 133.8873 | | | | 0.127331 | | | | 5007.297 | | | 18.98438 | |  | |  | |  |
| 9.975 | | 133.9744 | | | | 0.129225 | | | | 5007.141 | | | 18.98438 | |  | |  | |  |
| 9.983333 | | 134.0633 | | | | 0.131585 | | | | 5006.938 | | | 19.01563 | |  | |  | |  |
| 9.991667 | | 134.1542 | | | | 0.132931 | | | | 5006.789 | | | 18.86719 | |  | |  | |  |
| 10 | | 134.2422 | | | | 0.134567 | | | | 5006.633 | | | 18.72656 | |  | |  | |  |
| 10.00833 | | 134.3312 | | | | 0.136326 | | | | 5006.5 | | | 18.78906 | |  | |  | |  |
| 10.01667 | | 134.4202 | | | | 0.137666 | | | | 5006.32 | | | 18.60156 | |  | |  | |  |
| 10.025 | | 134.5101 | | | | 0.139052 | | | | 5006.172 | | | 18.65625 | |  | |  | |  |
| 10.03333 | | 134.5962 | | | | 0.140853 | | | | 5006.008 | | | 18.39844 | |  | |  | |  |
| 10.04167 | | 134.6871 | | | | 0.141875 | | | | 5005.875 | | | 18.63281 | |  | |  | |  |
| 10.05 | | 134.7742 | | | | 0.144012 | | | | 5005.727 | | | 18.36719 | |  | |  | |  |
| 10.05833 | | 134.8613 | | | | 0.146305 | | | | 5005.57 | | | 18.23438 | |  | |  | |  |
| 10.06667 | | 134.9502 | | | | 0.147776 | | | | 5005.438 | | | 18.20313 | |  | |  | |  |
| 10.075 | | 135.0364 | | | | 0.149203 | | | | 5005.281 | | | 18.15625 | |  | |  | |  |
| 10.08333 | | 135.1216 | | | | 0.151593 | | | | 5005.141 | | | 17.98438 | |  | |  | |  |
| 10.09167 | | 135.2087 | | | | 0.153659 | | | | 5004.969 | | | 17.90625 | |  | |  | |  |
| 10.1 | | 135.2958 | | | | 0.155609 | | | | 5004.852 | | | 17.83594 | |  | |  | |  |
| 10.10833 | | 135.381 | | | | 0.158435 | | | | 5004.711 | | | 17.85938 | |  | |  | |  |
| 10.11667 | | 135.4709 | | | | 0.161087 | | | | 5004.57 | | | 17.78906 | |  | |  | |  |
| 10.125 | | 135.5562 | | | | 0.16215 | | | | 5004.422 | | | 17.57813 | |  | |  | |  |
| 10.13333 | | 135.6423 | | | | 0.164032 | | | | 5004.289 | | | 17.5625 | |  | |  | |  |
| 10.14167 | | 135.7301 | | | | 0.165838 | | | | 5004.156 | | | 17.53125 | |  | |  | |  |
| 10.15 | | 135.8161 | | | | 0.166381 | | | | 5004.023 | | | 17.3125 | |  | |  | |  |
| 10.15833 | | 135.9022 | | | | 0.167913 | | | | 5003.875 | | | 17.22656 | |  | |  | |  |
| 10.16667 | | 135.991 | | | | 0.170493 | | | | 5003.727 | | | 17.21875 | |  | |  | |  |
| 10.175 | | 136.077 | | | | 0.171596 | | | | 5003.609 | | | 17.14063 | |  | |  | |  |
| 10.18333 | | 136.1658 | | | | 0.173769 | | | | 5003.469 | | | 17.11719 | |  | |  | |  |
| 10.19167 | | 136.2555 | | | | 0.176616 | | | | 5003.336 | | | 17.17188 | |  | |  | |  |
| 10.2 | | 136.3415 | | | | 0.177899 | | | | 5003.211 | | | 16.99219 | |  | |  | |  |
| 10.20833 | | 136.4285 | | | | 0.179827 | | | | 5003.086 | | | 17.0625 | |  | |  | |  |
| 10.21667 | | 136.5154 | | | | 0.183004 | | | | 5002.938 | | | 17.14063 | |  | |  | |  |
| 10.225 | | 136.5996 | | | | 0.184357 | | | | 5002.805 | | | 17.05469 | |  | |  | |  |
| 10.23333 | | 136.6856 | | | | 0.186303 | | | | 5002.656 | | | 16.91406 | |  | |  | |  |
| 10.24167 | | 136.7707 | | | | 0.189211 | | | | 5002.516 | | | 16.96875 | |  | |  | |  |
| 10.25 | | 136.8586 | | | | 0.19125 | | | | 5002.383 | | | 17 | |  | |  | |  |
| 10.25833 | | 136.9474 | | | | 0.19276 | | | | 5002.242 | | | 17.0625 | |  | |  | |  |
| 10.26667 | | 137.0315 | | | | 0.194891 | | | | 5002.078 | | | 16.96094 | |  | |  | |  |
| 10.275 | | 137.1166 | | | | 0.196496 | | | | 5001.945 | | | 16.8125 | |  | |  | |  |
| 10.28333 | | 137.2027 | | | | 0.197882 | | | | 5001.82 | | | 16.61719 | |  | |  | |  |
| 10.29167 | | 137.2877 | | | | 0.200445 | | | | 5001.672 | | | 16.41406 | |  | |  | |  |
| 10.3 | | 137.3747 | | | | 0.202164 | | | | 5001.531 | | | 16.32813 | |  | |  | |  |
| 10.30833 | | 137.4626 | | | | 0.203897 | | | | 5001.375 | | | 16.44531 | |  | |  | |  |
| 10.31667 | | 137.5467 | | | | 0.206405 | | | | 5001.242 | | | 16.57031 | |  | |  | |  |
| 10.325 | | 137.6337 | | | | 0.208582 | | | | 5001.125 | | | 16.58594 | |  | |  | |  |
| 10.33333 | | 137.7197 | | | | 0.209421 | | | | 5001 | | | 16.25 | |  | |  | |  |
| 10.34167 | | 137.8029 | | | | 0.211565 | | | | 5000.891 | | | 16.21875 | |  | |  | |  |
| 10.35 | | 137.889 | | | | 0.213308 | | | | 5000.766 | | | 16.24219 | |  | |  | |  |
| 10.35833 | | 137.975 | | | | 0.214386 | | | | 5000.609 | | | 16.1875 | |  | |  | |  |
| 10.36667 | | 138.0638 | | | | 0.216627 | | | | 5000.453 | | | 16.28906 | |  | |  | |  |
| 10.375 | | 138.1526 | | | | 0.21853 | | | | 5000.313 | | | 16.55469 | |  | |  | |  |
| 10.38333 | | 138.2395 | | | | 0.220032 | | | | 5000.219 | | | 16.38281 | |  | |  | |  |
| 10.39167 | | 138.3255 | | | | 0.222065 | | | | 5000.086 | | | 16.11719 | |  | |  | |  |
| 10.4 | | 138.4162 | | | | 0.224351 | | | | 4999.945 | | | 15.95313 | |  | |  | |  |
| 10.40833 | | 138.505 | | | | 0.226098 | | | | 4999.813 | | | 15.98438 | |  | |  | |  |
| 10.41667 | | 138.5891 | | | | 0.228065 | | | | 4999.672 | | | 16.11719 | |  | |  | |  |
| 10.425 | | 138.6761 | | | | 0.230944 | | | | 4999.492 | | | 15.92188 | |  | |  | |  |
| 10.43333 | | 138.7658 | | | | 0.232735 | | | | 4999.375 | | | 15.75781 | |  | |  | |  |
| 10.44167 | | 138.8528 | | | | 0.234262 | | | | 4999.266 | | | 15.8125 | |  | |  | |  |
| 10.45 | | 138.9406 | | | | 0.236996 | | | | 4999.164 | | | 15.54688 | |  | |  | |  |
| 10.45833 | | 139.0294 | | | | 0.238985 | | | | 4999.031 | | | 15.45313 | |  | |  | |  |
| 10.46667 | | 139.1191 | | | | 0.240607 | | | | 4998.875 | | | 15.51563 | |  | |  | |  |
| 10.475 | | 139.2061 | | | | 0.243603 | | | | 4998.766 | | | 15.70313 | |  | |  | |  |
| 10.48333 | | 139.293 | | | | 0.245532 | | | | 4998.664 | | | 15.53906 | |  | |  | |  |
| 10.49167 | | 139.379 | | | | 0.246688 | | | | 4998.516 | | | 15.36719 | |  | |  | |  |
| 10.5 | | 139.4725 | | | | 0.249285 | | | | 4998.422 | | | 15.15625 | |  | |  | |  |
| 10.50833 | | 139.5622 | | | | 0.250879 | | | | 4998.313 | | | 15.01563 | |  | |  | |  |
| 10.51667 | | 139.6482 | | | | 0.251881 | | | | 4998.172 | | | 15 | |  | |  | |  |
| 10.525 | | 139.7379 | | | | 0.254469 | | | | 4998.016 | | | 14.82813 | |  | |  | |  |
| 10.53333 | | 139.8239 | | | | 0.256619 | | | | 4997.898 | | | 14.85938 | |  | |  | |  |
| 10.54167 | | 139.9118 | | | | 0.258336 | | | | 4997.797 | | | 14.5625 | |  | |  | |  |
| 10.55 | | 139.9978 | | | | 0.2615 | | | | 4997.703 | | | 14.55469 | |  | |  | |  |
| 10.55833 | | 140.0866 | | | | 0.264231 | | | | 4997.602 | | | 14.375 | |  | |  | |  |
| 10.56667 | | 140.1745 | | | | 0.266039 | | | | 4997.477 | | | 14.3125 | |  | |  | |  |
| 10.575 | | 140.2624 | | | | 0.26871 | | | | 4997.375 | | | 14.38281 | |  | |  | |  |
| 10.58333 | | 140.3503 | | | | 0.270696 | | | | 4997.234 | | | 14.54688 | |  | |  | |  |
| 10.59167 | | 140.439 | | | | 0.272238 | | | | 4997.156 | | | 14.71875 | |  | |  | |  |
| 10.6 | | 140.5297 | | | | 0.273373 | | | | 4997.039 | | | 14.52344 | |  | |  | |  |
| 10.60833 | | 140.6148 | | | | 0.275537 | | | | 4996.945 | | | 14.50781 | |  | |  | |  |
| 10.61667 | | 140.7017 | | | | 0.277318 | | | | 4996.82 | | | 14.375 | |  | |  | |  |
| 10.625 | | 140.7878 | | | | 0.279164 | | | | 4996.688 | | | 14.5625 | |  | |  | |  |
| 10.63333 | | 140.8747 | | | | 0.281575 | | | | 4996.547 | | | 14.44531 | |  | |  | |  |
| 10.64167 | | 140.9616 | | | | 0.283318 | | | | 4996.406 | | | 14.55469 | |  | |  | |  |
| 10.65 | | 141.0495 | | | | 0.285152 | | | | 4996.305 | | | 14.35938 | |  | |  | |  |
| 10.65833 | | 141.1392 | | | | 0.288086 | | | | 4996.18 | | | 14.10938 | |  | |  | |  |
| 10.66667 | | 141.2271 | | | | 0.290225 | | | | 4996.078 | | | 14.08594 | |  | |  | |  |
| 10.675 | | 141.3159 | | | | 0.291821 | | | | 4995.93 | | | 13.94531 | |  | |  | |  |
| 10.68333 | | 141.4001 | | | | 0.293782 | | | | 4995.828 | | | 14.09375 | |  | |  | |  |
| 10.69167 | | 141.4916 | | | | 0.295456 | | | | 4995.695 | | | 14.20313 | |  | |  | |  |
| 10.7 | | 141.5804 | | | | 0.296425 | | | | 4995.594 | | | 14.01563 | |  | |  | |  |
| 10.70833 | | 141.6711 | | | | 0.298562 | | | | 4995.508 | | | 14.03125 | |  | |  | |  |
| 10.71667 | | 141.7599 | | | | 0.301498 | | | | 4995.391 | | | 13.98438 | |  | |  | |  |
| 10.725 | | 141.8477 | | | | 0.303936 | | | | 4995.289 | | | 13.60156 | |  | |  | |  |
| 10.73333 | | 141.9356 | | | | 0.306386 | | | | 4995.156 | | | 13.67188 | |  | |  | |  |
| 10.74167 | | 142.0235 | | | | 0.30949 | | | | 4995.023 | | | 13.69531 | |  | |  | |  |
| 10.75 | | 142.1067 | | | | 0.31157 | | | | 4994.922 | | | 13.55469 | |  | |  | |  |
| 10.75833 | | 142.1937 | | | | 0.313861 | | | | 4994.805 | | | 13.55469 | |  | |  | |  |
| 10.76667 | | 142.2834 | | | | 0.316629 | | | | 4994.695 | | | 13.32031 | |  | |  | |  |
| 10.775 | | 142.3703 | | | | 0.318296 | | | | 4994.625 | | | 13.02344 | |  | |  | |  |
| 10.78333 | | 142.4591 | | | | 0.319129 | | | | 4994.5 | | | 13.16406 | |  | |  | |  |
| 10.79167 | | 142.5442 | | | | 0.320549 | | | | 4994.383 | | | 12.96875 | |  | |  | |  |
| 10.8 | | 142.6312 | | | | 0.321197 | | | | 4994.297 | | | 13.03906 | |  | |  | |  |
| 10.80833 | | 142.7181 | | | | 0.322106 | | | | 4994.18 | | | 12.9375 | |  | |  | |  |
| 10.81667 | | 142.8051 | | | | 0.324637 | | | | 4994.094 | | | 12.88281 | |  | |  | |  |
| 10.825 | | 142.8902 | | | | 0.326211 | | | | 4994.023 | | | 12.83594 | |  | |  | |  |
| 10.83333 | | 142.9799 | | | | 0.327876 | | | | 4993.898 | | | 12.96094 | |  | |  | |  |
| 10.84167 | | 143.0668 | | | | 0.330409 | | | | 4993.813 | | | 12.86719 | |  | |  | |  |
| 10.85 | | 143.1519 | | | | 0.331609 | | | | 4993.695 | | | 12.99219 | |  | |  | |  |
| 10.85833 | | 143.237 | | | | 0.332626 | | | | 4993.602 | | | 13.01563 | |  | |  | |  |
| 10.86667 | | 143.323 | | | | 0.334662 | | | | 4993.492 | | | 12.86719 | |  | |  | |  |
| 10.875 | | 143.4118 | | | | 0.33636 | | | | 4993.391 | | | 12.97656 | |  | |  | |  |
| 10.88333 | | 143.5006 | | | | 0.337957 | | | | 4993.266 | | | 12.99219 | |  | |  | |  |
| 10.89167 | | 143.5894 | | | | 0.339299 | | | | 4993.172 | | | 12.96875 | |  | |  | |  |
| 10.9 | | 143.6791 | | | | 0.340819 | | | | 4993.047 | | | 13.1875 | |  | |  | |  |
| 10.90833 | | 143.7689 | | | | 0.341961 | | | | 4992.938 | | | 13.01563 | |  | |  | |  |
| 10.91667 | | 143.854 | | | | 0.343744 | | | | 4992.844 | | | 13.01563 | |  | |  | |  |
| 10.925 | | 143.9427 | | | | 0.345407 | | | | 4992.727 | | | 13.05469 | |  | |  | |  |
| 10.93333 | | 144.0325 | | | | 0.346577 | | | | 4992.609 | | | 12.78906 | |  | |  | |  |
| 10.94167 | | 144.1231 | | | | 0.348993 | | | | 4992.508 | | | 12.8125 | |  | |  | |  |
| 10.95 | | 144.2138 | | | | 0.351482 | | | | 4992.367 | | | 12.53125 | |  | |  | |  |
| 10.95833 | | 144.3044 | | | | 0.352685 | | | | 4992.281 | | | 12.42188 | |  | |  | |  |
| 10.96667 | | 144.3932 | | | | 0.354298 | | | | 4992.18 | | | 12.22656 | |  | |  | |  |
| 10.975 | | 144.4801 | | | | 0.356457 | | | | 4992.063 | | | 12.27344 | |  | |  | |  |
| 10.98333 | | 144.5671 | | | | 0.357264 | | | | 4991.984 | | | 12.10156 | |  | |  | |  |
| 10.99167 | | 144.6559 | | | | 0.358043 | | | | 4991.867 | | | 12.10156 | |  | |  | |  |
| 11 | | 144.7484 | | | | 0.360871 | | | | 4991.797 | | | 11.86719 | |  | |  | |  |
| 11.00833 | | 144.8362 | | | | 0.361827 | | | | 4991.711 | | | 11.71094 | |  | |  | |  |
| 11.01667 | | 144.9269 | | | | 0.363288 | | | | 4991.641 | | | 11.92969 | |  | |  | |  |
| 11.025 | | 145.0185 | | | | 0.366421 | | | | 4991.523 | | | 11.84375 | |  | |  | |  |
| 11.03333 | | 145.1054 | | | | 0.368148 | | | | 4991.453 | | | 11.92188 | |  | |  | |  |
| 11.04167 | | 145.1914 | | | | 0.368711 | | | | 4991.344 | | | 11.875 | |  | |  | |  |
| 11.05 | | 145.2793 | | | | 0.371183 | | | | 4991.273 | | | 12.03125 | |  | |  | |  |
| 11.05833 | | 145.3672 | | | | 0.372829 | | | | 4991.203 | | | 11.92969 | |  | |  | |  |
| 11.06667 | | 145.456 | | | | 0.373575 | | | | 4991.07 | | | 11.74219 | |  | |  | |  |
| 11.075 | | 145.5438 | | | | 0.375927 | | | | 4990.977 | | | 11.60938 | |  | |  | |  |
| 11.08333 | | 145.6323 | | | | 0.377807 | | | | 4990.867 | | | 11.41406 | |  | |  | |  |
| 11.09167 | | 145.7189 | | | | 0.37857 | | | | 4990.766 | | | 11.32813 | |  | |  | |  |
| 11.1 | | 145.8037 | | | | 0.381591 | | | | 4990.641 | | | 11.10938 | |  | |  | |  |
| 11.10833 | | 145.8866 | | | | 0.383677 | | | | 4990.563 | | | 10.99219 | |  | |  | |  |
| 11.11667 | | 145.9714 | | | | 0.384167 | | | | 4990.492 | | | 10.97656 | |  | |  | |  |
| 11.125 | | 146.0562 | | | | 0.385942 | | | | 4990.414 | | | 10.97656 | |  | |  | |  |
| 11.13333 | | 146.1418 | | | | 0.387811 | | | | 4990.344 | | | 10.86719 | |  | |  | |  |
| 11.14167 | | 146.2293 | | | | 0.388467 | | | | 4990.258 | | | 10.89844 | |  | |  | |  |
| 11.15 | | 146.3159 | | | | 0.390287 | | | | 4990.195 | | | 10.89063 | |  | |  | |  |
| 11.15833 | | 146.4025 | | | | 0.39246 | | | | 4990.117 | | | 11.23438 | |  | |  | |  |
| 11.16667 | | 146.4881 | | | | 0.393457 | | | | 4990.031 | | | 11.51563 | |  | |  | |  |
| 11.175 | | 146.572 | | | | 0.39561 | | | | 4989.938 | | | 11.46875 | |  | |  | |  |
| 11.18333 | | 146.6586 | | | | 0.397615 | | | | 4989.859 | | | 11.35156 | |  | |  | |  |
| 11.19167 | | 146.7451 | | | | 0.39806 | | | | 4989.766 | | | 11.46875 | |  | |  | |  |
| 11.2 | | 146.8353 | | | | 0.400725 | | | | 4989.68 | | | 11.35156 | |  | |  | |  |
| 11.20833 | | 146.9228 | | | | 0.402853 | | | | 4989.531 | | | 11.28906 | |  | |  | |  |
| 11.21667 | | 147.0103 | | | | 0.403867 | | | | 4989.391 | | | 11.36719 | |  | |  | |  |
| 11.225 | | 147.0995 | | | | 0.405392 | | | | 4989.305 | | | 11.21875 | |  | |  | |  |
| 11.23333 | | 147.187 | | | | 0.406995 | | | | 4989.219 | | | 11.34375 | |  | |  | |  |
| 11.24167 | | 147.2745 | | | | 0.407502 | | | | 4989.109 | | | 11.02344 | |  | |  | |  |
| 11.25 | | 147.3602 | | | | 0.409165 | | | | 4989.023 | | | 10.88281 | |  | |  | |  |
| 11.25833 | | 147.4503 | | | | 0.411026 | | | | 4988.938 | | | 10.96094 | |  | |  | |  |
| 11.26667 | | 147.5387 | | | | 0.412347 | | | | 4988.836 | | | 11.03125 | |  | |  | |  |
| 11.275 | | 147.6253 | | | | 0.412718 | | | | 4988.75 | | | 11.11719 | |  | |  | |  |
| 11.28333 | | 147.7128 | | | | 0.414342 | | | | 4988.641 | | | 11.27344 | |  | |  | |  |
| 11.29167 | | 147.8011 | | | | 0.416413 | | | | 4988.594 | | | 11.39844 | |  | |  | |  |
| 11.3 | | 147.8895 | | | | 0.417155 | | | | 4988.508 | | | 11.32813 | |  | |  | |  |
| 11.30833 | | 147.977 | | | | 0.419106 | | | | 4988.414 | | | 11.41406 | |  | |  | |  |
| 11.31667 | | 148.0635 | | | | 0.420636 | | | | 4988.313 | | | 11.53906 | |  | |  | |  |
| 11.325 | | 148.151 | | | | 0.421228 | | | | 4988.203 | | | 11.55469 | |  | |  | |  |
| 11.33333 | | 148.2385 | | | | 0.422789 | | | | 4988.094 | | | 11.5 | |  | |  | |  |
| 11.34167 | | 148.3251 | | | | 0.424261 | | | | 4987.977 | | | 11.48438 | |  | |  | |  |
| 11.35 | | 148.4107 | | | | 0.4244 | | | | 4987.891 | | | 11.47656 | |  | |  | |  |
| 11.35833 | | 148.4982 | | | | 0.426778 | | | | 4987.781 | | | 11.24219 | |  | |  | |  |
| 11.36667 | | 148.5803 | | | | 0.429151 | | | | 4987.672 | | | 11.02344 | |  | |  | |  |
| 11.375 | | 148.6686 | | | | 0.429865 | | | | 4987.578 | | | 10.90625 | |  | |  | |  |
| 11.38333 | | 148.7543 | | | | 0.432072 | | | | 4987.492 | | | 10.85938 | |  | |  | |  |
| 11.39167 | | 148.8391 | | | | 0.435102 | | | | 4987.391 | | | 10.61719 | |  | |  | |  |
| 11.4 | | 148.9238 | | | | 0.436326 | | | | 4987.305 | | | 10.46875 | |  | |  | |  |
| 11.40833 | | 149.0086 | | | | 0.43835 | | | | 4987.25 | | | 10.39844 | |  | |  | |  |
| 11.41667 | | 149.0943 | | | | 0.441249 | | | | 4987.18 | | | 10.3125 | |  | |  | |  |
| 11.425 | | 149.1817 | | | | 0.442401 | | | | 4987.102 | | | 10.30469 | |  | |  | |  |
| 11.43333 | | 149.2674 | | | | 0.444013 | | | | 4987.023 | | | 10.28906 | |  | |  | |  |
| 11.44167 | | 149.3531 | | | | 0.446071 | | | | 4986.961 | | | 10.25781 | |  | |  | |  |
| 11.45 | | 149.4433 | | | | 0.446144 | | | | 4986.891 | | | 10.32813 | |  | |  | |  |
| 11.45833 | | 149.5307 | | | | 0.447734 | | | | 4986.813 | | | 10.03125 | |  | |  | |  |
| 11.46667 | | 149.6164 | | | | 0.450109 | | | | 4986.742 | | | 9.867188 | |  | |  | |  |
| 11.475 | | 149.7012 | | | | 0.451439 | | | | 4986.664 | | | 9.765625 | |  | |  | |  |
| 11.48333 | | 149.7887 | | | | 0.452758 | | | | 4986.578 | | | 9.59375 | |  | |  | |  |
| 11.49167 | | 149.8797 | | | | 0.454865 | | | | 4986.492 | | | 9.65625 | |  | |  | |  |
| 11.5 | | 149.9681 | | | | 0.456184 | | | | 4986.398 | | | 9.492188 | |  | |  | |  |
| 11.50833 | | 150.0556 | | | | 0.456948 | | | | 4986.367 | | | 9.34375 | |  | |  | |  |
| 11.51667 | | 150.1422 | | | | 0.458688 | | | | 4986.305 | | | 9.226563 | |  | |  | |  |
| 11.525 | | 150.2305 | | | | 0.460238 | | | | 4986.234 | | | 9.070313 | |  | |  | |  |
| 11.53333 | | 150.318 | | | | 0.461195 | | | | 4986.172 | | | 8.984375 | |  | |  | |  |
| 11.54167 | | 150.4037 | | | | 0.462832 | | | | 4986.086 | | | 9.125 | |  | |  | |  |
| 11.55 | | 150.492 | | | | 0.464758 | | | | 4986.023 | | | 9.195313 | |  | |  | |  |
| 11.55833 | | 150.584 | | | | 0.465839 | | | | 4985.969 | | | 9.164063 | |  | |  | |  |
| 11.56667 | | 150.6742 | | | | 0.467844 | | | | 4985.906 | | | 9.460938 | |  | |  | |  |
| 11.575 | | 150.7617 | | | | 0.4713 | | | | 4985.844 | | | 9.445313 | |  | |  | |  |
| 11.58333 | | 150.8482 | | | | 0.472424 | | | | 4985.781 | | | 9.460938 | |  | |  | |  |
| 11.59167 | | 150.9384 | | | | 0.473824 | | | | 4985.68 | | | 9.695313 | |  | |  | |  |
| 11.6 | | 151.0268 | | | | 0.476072 | | | | 4985.594 | | | 9.898438 | |  | |  | |  |
| 11.60833 | | 151.1161 | | | | 0.477036 | | | | 4985.516 | | | 9.835938 | |  | |  | |  |
| 11.61667 | | 151.2035 | | | | 0.477309 | | | | 4985.398 | | | 10.01563 | |  | |  | |  |
| 11.625 | | 151.2901 | | | | 0.479224 | | | | 4985.32 | | | 9.984375 | |  | |  | |  |
| 11.63333 | | 151.3794 | | | | 0.480141 | | | | 4985.227 | | | 9.882813 | |  | |  | |  |
| 11.64167 | | 151.4642 | | | | 0.480523 | | | | 4985.125 | | | 9.75 | |  | |  | |  |
| 11.65 | | 151.5489 | | | | 0.483013 | | | | 4985.008 | | | 9.570313 | |  | |  | |  |
| 11.65833 | | 151.6337 | | | | 0.484653 | | | | 4984.93 | | | 9.265625 | |  | |  | |  |
| 11.66667 | | 151.7257 | | | | 0.4858 | | | | 4984.82 | | | 9.328125 | |  | |  | |  |
| 11.675 | | 151.8104 | | | | 0.488431 | | | | 4984.742 | | | 9.25 | |  | |  | |  |
| 11.68333 | | 151.8988 | | | | 0.489791 | | | | 4984.664 | | | 9.0625 | |  | |  | |  |
| 11.69167 | | 151.9845 | | | | 0.490859 | | | | 4984.602 | | | 8.984375 | |  | |  | |  |
| 11.7 | | 152.0702 | | | | 0.493071 | | | | 4984.547 | | | 9 | |  | |  | |  |
| 11.70833 | | 152.1576 | | | | 0.494509 | | | | 4984.5 | | | 8.890625 | |  | |  | |  |
| 11.71667 | | 152.2442 | | | | 0.495103 | | | | 4984.414 | | | 8.78125 | |  | |  | |  |
| 11.725 | | 152.3308 | | | | 0.497862 | | | | 4984.352 | | | 8.984375 | |  | |  | |  |
| 11.73333 | | 152.4182 | | | | 0.499004 | | | | 4984.297 | | | 8.695313 | |  | |  | |  |
| 11.74167 | | 152.5039 | | | | 0.499427 | | | | 4984.234 | | | 8.609375 | |  | |  | |  |
| 11.75 | | 152.5869 | | | | 0.501449 | | | | 4984.156 | | | 8.609375 | |  | |  | |  |
| 11.75833 | | 152.6707 | | | | 0.502447 | | | | 4984.102 | | | 8.570313 | |  | |  | |  |
| 11.76667 | | 152.7564 | | | | 0.503011 | | | | 4984.047 | | | 8.445313 | |  | |  | |  |
| 11.775 | | 152.843 | | | | 0.505045 | | | | 4983.938 | | | 8.492188 | |  | |  | |  |
| 11.78333 | | 152.9323 | | | | 0.50632 | | | | 4983.906 | | | 8.390625 | |  | |  | |  |
| 11.79167 | | 153.0188 | | | | 0.506808 | | | | 4983.844 | | | 8.375 | |  | |  | |  |
| 11.8 | | 153.1045 | | | | 0.509181 | | | | 4983.773 | | | 8.398438 | |  | |  | |  |
| 11.80833 | | 153.1938 | | | | 0.511079 | | | | 4983.711 | | | 8.3125 | |  | |  | |  |
| 11.81667 | | 153.2831 | | | | 0.512296 | | | | 4983.656 | | | 8.3125 | |  | |  | |  |
| 11.825 | | 153.375 | | | | 0.51486 | | | | 4983.578 | | | 8.320313 | |  | |  | |  |
| 11.83333 | | 153.4634 | | | | 0.517091 | | | | 4983.523 | | | 8.390625 | |  | |  | |  |
| 11.84167 | | 153.5536 | | | | 0.518 | | | | 4983.453 | | | 8.257813 | |  | |  | |  |
| 11.85 | | 153.6456 | | | | 0.519151 | | | | 4983.375 | | | 8.476563 | |  | |  | |  |
| 11.85833 | | 153.7339 | | | | 0.520836 | | | | 4983.328 | | | 8.523438 | |  | |  | |  |
| 11.86667 | | 153.8205 | | | | 0.522015 | | | | 4983.25 | | | 8.414063 | |  | |  | |  |
| 11.875 | | 153.9098 | | | | 0.523894 | | | | 4983.18 | | | 8.195313 | |  | |  | |  |
| 11.88333 | | 154.0009 | | | | 0.526466 | | | | 4983.109 | | | 8.304688 | |  | |  | |  |
| 11.89167 | | 154.0928 | | | | 0.528482 | | | | 4983.047 | | | 8.179688 | |  | |  | |  |
| 11.9 | | 154.1821 | | | | 0.530063 | | | | 4982.945 | | | 8.070313 | |  | |  | |  |
| 11.90833 | | 154.2678 | | | | 0.532112 | | | | 4982.875 | | | 8.328125 | |  | |  | |  |
| 11.91667 | | 154.3544 | | | | 0.53346 | | | | 4982.828 | | | 8.039063 | |  | |  | |  |
| 11.925 | | 154.4436 | | | | 0.534494 | | | | 4982.789 | | | 7.828125 | |  | |  | |  |
| 11.93333 | | 154.5302 | | | | 0.536474 | | | | 4982.703 | | | 7.484375 | |  | |  | |  |
| 11.94167 | | 154.6186 | | | | 0.538417 | | | | 4982.648 | | | 7.398438 | |  | |  | |  |
| 11.95 | | 154.7106 | | | | 0.539999 | | | | 4982.586 | | | 7.4375 | |  | |  | |  |
| 11.95833 | | 154.7971 | | | | 0.542253 | | | | 4982.484 | | | 7.5625 | |  | |  | |  |
| 11.96667 | | 154.8882 | | | | 0.544277 | | | | 4982.445 | | | 7.398438 | |  | |  | |  |
| 11.975 | | 154.9757 | | | | 0.544273 | | | | 4982.414 | | | 7.46875 | |  | |  | |  |
| 11.98333 | | 155.0641 | | | | 0.545205 | | | | 4982.398 | | | 7.390625 | |  | |  | |  |
| 11.99167 | | 155.1543 | | | | 0.547091 | | | | 4982.344 | | | 7.296875 | |  | |  | |  |
| 12 | | 155.2462 | | | | 0.547769 | | | | 4982.266 | | | 7.414063 | |  | |  | |  |
| 12.00833 | | 155.3337 | | | | 0.548707 | | | | 4982.188 | | | 7.804688 | |  | |  | |  |
| 12.01667 | | 155.4221 | | | | 0.550378 | | | | 4982.148 | | | 8.007813 | |  | |  | |  |
| 12.025 | | 155.5087 | | | | 0.551629 | | | | 4982.07 | | | 8.078125 | |  | |  | |  |
| 12.03333 | | 155.5932 | | | | 0.552409 | | | | 4982.016 | | | 7.929688 | |  | |  | |  |
| 12.04167 | | 155.6823 | | | | 0.554362 | | | | 4981.961 | | | 7.898438 | |  | |  | |  |
| 12.05 | | 155.765 | | | | 0.555666 | | | | 4981.891 | | | 7.898438 | |  | |  | |  |
| 12.05833 | | 155.8496 | | | | 0.557343 | | | | 4981.766 | | | 7.835938 | |  | |  | |  |
| 12.06667 | | 155.9351 | | | | 0.559578 | | | | 4981.672 | | | 7.898438 | |  | |  | |  |
| 12.075 | | 156.0205 | | | | 0.559782 | | | | 4981.594 | | | 7.6875 | |  | |  | |  |
| 12.08333 | | 156.1078 | | | | 0.560881 | | | | 4981.547 | | | 7.742188 | |  | |  | |  |
| 12.09167 | | 156.1914 | | | | 0.562158 | | | | 4981.484 | | | 7.28125 | |  | |  | |  |
| 12.1 | | 156.2769 | | | | 0.561971 | | | | 4981.422 | | | 7.101563 | |  | |  | |  |
| 12.10833 | | 156.3659 | | | | 0.562755 | | | | 4981.367 | | | 6.820313 | |  | |  | |  |
| 12.11667 | | 156.4549 | | | | 0.564541 | | | | 4981.297 | | | 6.898438 | |  | |  | |  |
| 12.125 | | 156.5431 | | | | 0.564586 | | | | 4981.25 | | | 6.945313 | |  | |  | |  |
| 12.13333 | | 156.6294 | | | | 0.565188 | | | | 4981.195 | | | 6.875 | |  | |  | |  |
| 12.14167 | | 156.7175 | | | | 0.567304 | | | | 4981.188 | | | 6.789063 | |  | |  | |  |
| 12.15 | | 156.8083 | | | | 0.568117 | | | | 4981.156 | | | 6.75 | |  | |  | |  |
| 12.15833 | | 156.8956 | | | | 0.568542 | | | | 4981.141 | | | 6.789063 | |  | |  | |  |
| 12.16667 | | 156.981 | | | | 0.570383 | | | | 4981.063 | | | 6.757813 | |  | |  | |  |
| 12.175 | | 157.071 | | | | 0.572011 | | | | 4981 | | | 6.921875 | |  | |  | |  |
| 12.18333 | | 157.1591 | | | | 0.572855 | | | | 4980.953 | | | 6.695313 | |  | |  | |  |
| 12.19167 | | 157.2481 | | | | 0.574709 | | | | 4980.906 | | | 6.882813 | |  | |  | |  |
| 12.2 | | 157.3327 | | | | 0.576593 | | | | 4980.852 | | | 6.648438 | |  | |  | |  |
| 12.20833 | | 157.4181 | | | | 0.577902 | | | | 4980.797 | | | 6.617188 | |  | |  | |  |
| 12.21667 | | 157.5045 | | | | 0.580023 | | | | 4980.742 | | | 6.6875 | |  | |  | |  |
| 12.225 | | 157.5926 | | | | 0.581983 | | | | 4980.664 | | | 6.492188 | |  | |  | |  |
| 12.23333 | | 157.6772 | | | | 0.582358 | | | | 4980.625 | | | 6.398438 | |  | |  | |  |
| 12.24167 | | 157.7653 | | | | 0.583876 | | | | 4980.539 | | | 6.390625 | |  | |  | |  |
| 12.25 | | 157.8534 | | | | 0.585555 | | | | 4980.523 | | | 6.460938 | |  | |  | |  |
| 12.25833 | | 157.9442 | | | | 0.586692 | | | | 4980.461 | | | 6.3125 | |  | |  | |  |
| 12.26667 | | 158.0333 | | | | 0.58817 | | | | 4980.398 | | | 6.390625 | |  | |  | |  |
| 12.275 | | 158.1178 | | | | 0.590513 | | | | 4980.375 | | | 6.398438 | |  | |  | |  |
| 12.28333 | | 158.2086 | | | | 0.591414 | | | | 4980.328 | | | 6.5 | |  | |  | |  |
| 12.29167 | | 158.2985 | | | | 0.592511 | | | | 4980.281 | | | 6.34375 | |  | |  | |  |
| 12.3 | | 158.3884 | | | | 0.594812 | | | | 4980.219 | | | 6.3125 | |  | |  | |  |
| 12.30833 | | 158.473 | | | | 0.595884 | | | | 4980.18 | | | 6.570313 | |  | |  | |  |
| 12.31667 | | 158.5611 | | | | 0.596985 | | | | 4980.117 | | | 6.46875 | |  | |  | |  |
| 12.325 | | 158.6466 | | | | 0.599703 | | | | 4980.07 | | | 6.703125 | |  | |  | |  |
| 12.33333 | | 158.7321 | | | | 0.601121 | | | | 4979.992 | | | 6.679688 | |  | |  | |  |
| 12.34167 | | 158.8166 | | | | 0.601124 | | | | 4979.969 | | | 6.6875 | |  | |  | |  |
| 12.35 | | 158.9012 | | | | 0.602707 | | | | 4979.914 | | | 6.445313 | |  | |  | |  |
| 12.35833 | | 158.9902 | | | | 0.604096 | | | | 4979.82 | | | 6.484375 | |  | |  | |  |
| 12.36667 | | 159.0757 | | | | 0.604332 | | | | 4979.773 | | | 6.382813 | |  | |  | |  |
| 12.375 | | 159.1611 | | | | 0.606351 | | | | 4979.695 | | | 6.5 | |  | |  | |  |
| 12.38333 | | 159.2502 | | | | 0.607304 | | | | 4979.648 | | | 6.5625 | |  | |  | |  |
| 12.39167 | | 159.3401 | | | | 0.607727 | | | | 4979.578 | | | 6.453125 | |  | |  | |  |
| 12.4 | | 159.4273 | | | | 0.609125 | | | | 4979.555 | | | 6.445313 | |  | |  | |  |
| 12.40833 | | 159.5181 | | | | 0.610421 | | | | 4979.5 | | | 6.382813 | |  | |  | |  |
| 12.41667 | | 159.608 | | | | 0.610547 | | | | 4979.453 | | | 6.398438 | |  | |  | |  |
| 12.425 | | 159.6962 | | | | 0.612706 | | | | 4979.367 | | | 6.34375 | |  | |  | |  |
| 12.43333 | | 159.787 | | | | 0.615187 | | | | 4979.32 | | | 6.46875 | |  | |  | |  |
| 12.44167 | | 159.8751 | | | | 0.615684 | | | | 4979.273 | | | 6.460938 | |  | |  | |  |
| 12.45 | | 159.965 | | | | 0.617741 | | | | 4979.227 | | | 6.28125 | |  | |  | |  |
| 12.45833 | | 160.054 | | | | 0.619917 | | | | 4979.172 | | | 6.28125 | |  | |  | |  |
| 12.46667 | | 160.1413 | | | | 0.620585 | | | | 4979.125 | | | 6.210938 | |  | |  | |  |
| 12.475 | | 160.2285 | | | | 0.622179 | | | | 4979.07 | | | 5.984375 | |  | |  | |  |
| 12.48333 | | 160.3166 | | | | 0.624072 | | | | 4979 | | | 6 | |  | |  | |  |
| 12.49167 | | 160.4047 | | | | 0.624872 | | | | 4978.953 | | | 5.78125 | |  | |  | |  |
| 12.5 | | 160.4938 | | | | 0.625943 | | | | 4978.922 | | | 5.875 | |  | |  | |  |
| 12.50833 | | 160.5792 | | | | 0.627661 | | | | 4978.875 | | | 5.867188 | |  | |  | |  |
| 12.51667 | | 160.67 | | | | 0.628623 | | | | 4978.836 | | | 5.765625 | |  | |  | |  |
| 12.525 | | 160.759 | | | | 0.629625 | | | | 4978.813 | | | 5.84375 | |  | |  | |  |
| 12.53333 | | 160.8454 | | | | 0.631637 | | | | 4978.758 | | | 5.921875 | |  | |  | |  |
| 12.54167 | | 160.9335 | | | | 0.632705 | | | | 4978.742 | | | 5.78125 | |  | |  | |  |
| 12.55 | | 161.0217 | | | | 0.633574 | | | | 4978.68 | | | 5.914063 | |  | |  | |  |
| 12.55833 | | 161.1116 | | | | 0.63584 | | | | 4978.633 | | | 6.054688 | |  | |  | |  |
| 12.56667 | | 161.1997 | | | | 0.636985 | | | | 4978.594 | | | 6.015625 | |  | |  | |  |
| 12.575 | | 161.2878 | | | | 0.637213 | | | | 4978.539 | | | 6.132813 | |  | |  | |  |
| 12.58333 | | 161.3742 | | | | 0.638847 | | | | 4978.477 | | | 6.046875 | |  | |  | |  |
| 12.59167 | | 161.4632 | | | | 0.640378 | | | | 4978.445 | | | 6.015625 | |  | |  | |  |
| 12.6 | | 161.5469 | | | | 0.640829 | | | | 4978.375 | | | 6.101563 | |  | |  | |  |
| 12.60833 | | 161.635 | | | | 0.642595 | | | | 4978.305 | | | 5.992188 | |  | |  | |  |
| 12.61667 | | 161.7231 | | | | 0.64414 | | | | 4978.266 | | | 5.953125 | |  | |  | |  |
| 12.625 | | 161.8113 | | | | 0.645483 | | | | 4978.195 | | | 5.726563 | |  | |  | |  |
| 12.63333 | | 161.8985 | | | | 0.647298 | | | | 4978.156 | | | 5.679688 | |  | |  | |  |
| 12.64167 | | 161.984 | | | | 0.649494 | | | | 4978.117 | | | 5.789063 | |  | |  | |  |
| 12.65 | | 162.0721 | | | | 0.650216 | | | | 4978.055 | | | 5.882813 | |  | |  | |  |
| 12.65833 | | 162.1584 | | | | 0.652195 | | | | 4978.016 | | | 5.671875 | |  | |  | |  |
| 12.66667 | | 162.2475 | | | | 0.654475 | | | | 4977.969 | | | 5.75 | |  | |  | |  |
| 12.675 | | 162.34 | | | | 0.655051 | | | | 4977.953 | | | 5.859375 | |  | |  | |  |
| 12.68333 | | 162.4291 | | | | 0.656174 | | | | 4977.906 | | | 5.554688 | |  | |  | |  |
| 12.69167 | | 162.5172 | | | | 0.658314 | | | | 4977.852 | | | 5.585938 | |  | |  | |  |
| 12.7 | | 162.6062 | | | | 0.659119 | | | | 4977.789 | | | 5.617188 | |  | |  | |  |
| 12.70833 | | 162.6961 | | | | 0.660154 | | | | 4977.758 | | | 5.75 | |  | |  | |  |
| 12.71667 | | 162.7825 | | | | 0.661578 | | | | 4977.703 | | | 5.726563 | |  | |  | |  |
| 12.725 | | 162.8715 | | | | 0.662263 | | | | 4977.641 | | | 5.546875 | |  | |  | |  |
| 12.73333 | | 162.9605 | | | | 0.663099 | | | | 4977.625 | | | 5.25 | |  | |  | |  |
| 12.74167 | | 163.0513 | | | | 0.664903 | | | | 4977.578 | | | 5.265625 | |  | |  | |  |
| 12.75 | | 163.1403 | | | | 0.666148 | | | | 4977.531 | | | 5.375 | |  | |  | |  |
| 12.75833 | | 163.2267 | | | | 0.666666 | | | | 4977.469 | | | 5.25 | |  | |  | |  |
| 12.76667 | | 163.3157 | | | | 0.668282 | | | | 4977.414 | | | 5.289063 | |  | |  | |  |
| 12.775 | | 163.4029 | | | | 0.669608 | | | | 4977.391 | | | 5.257813 | |  | |  | |  |
| 12.78333 | | 163.4928 | | | | 0.670396 | | | | 4977.383 | | | 5.125 | |  | |  | |  |
| 12.79167 | | 163.5783 | | | | 0.672627 | | | | 4977.336 | | | 5.015625 | |  | |  | |  |
| 12.8 | | 163.6664 | | | | 0.673817 | | | | 4977.273 | | | 4.726563 | |  | |  | |  |
| 12.80833 | | 163.7554 | | | | 0.674433 | | | | 4977.25 | | | 4.875 | |  | |  | |  |
| 12.81667 | | 163.8445 | | | | 0.676585 | | | | 4977.203 | | | 5.429688 | |  | |  | |  |
| 12.825 | | 163.9317 | | | | 0.677145 | | | | 4977.164 | | | 5.539063 | |  | |  | |  |
| 12.83333 | | 164.0207 | | | | 0.677583 | | | | 4977.133 | | | 5.3125 | |  | |  | |  |
| 12.84167 | | 164.108 | | | | 0.67967 | | | | 4977.109 | | | 5.265625 | |  | |  | |  |
| 12.85 | | 164.1943 | | | | 0.681678 | | | | 4977.109 | | | 5.335938 | |  | |  | |  |
| 12.85833 | | 164.2824 | | | | 0.682753 | | | | 4977.047 | | | 5.242188 | |  | |  | |  |
| 12.86667 | | 164.3688 | | | | 0.684918 | | | | 4976.93 | | | 5.28125 | |  | |  | |  |
| 12.875 | | 164.4587 | | | | 0.687035 | | | | 4976.859 | | | 5.351563 | |  | |  | |  |
| 12.88333 | | 164.5495 | | | | 0.687754 | | | | 4976.852 | | | 5.429688 | |  | |  | |  |
| 12.89167 | | 164.6385 | | | | 0.688616 | | | | 4976.82 | | | 5.242188 | |  | |  | |  |
| 12.9 | | 164.7275 | | | | 0.690354 | | | | 4976.766 | | | 4.945313 | |  | |  | |  |
| 12.90833 | | 164.8175 | | | | 0.690493 | | | | 4976.734 | | | 4.765625 | |  | |  | |  |
| 12.91667 | | 164.9047 | | | | 0.691202 | | | | 4976.688 | | | 4.796875 | |  | |  | |  |
| 12.925 | | 164.9982 | | | | 0.692384 | | | | 4976.625 | | | 4.9375 | |  | |  | |  |
| 12.93333 | | 165.0907 | | | | 0.692481 | | | | 4976.563 | | | 5.09375 | |  | |  | |  |
| 12.94167 | | 165.1815 | | | | 0.693106 | | | | 4976.555 | | | 5.21875 | |  | |  | |  |
| 12.95 | | 165.2703 | | | | 0.694852 | | | | 4976.547 | | | 5.25 | |  | |  | |  |
| 12.95833 | | 165.3595 | | | | 0.695609 | | | | 4976.531 | | | 5.46875 | |  | |  | |  |
| 12.96667 | | 165.4495 | | | | 0.695695 | | | | 4976.484 | | | 5.382813 | |  | |  | |  |
| 12.975 | | 165.537 | | | | 0.697863 | | | | 4976.422 | | | 5.5625 | |  | |  | |  |
| 12.98333 | | 165.6227 | | | | 0.699277 | | | | 4976.359 | | | 5.289063 | |  | |  | |  |
| 12.99167 | | 165.7092 | | | | 0.699515 | | | | 4976.297 | | | 5.1875 | |  | |  | |  |
| 13 | | 165.7975 | | | | 0.701907 | | | | 4976.242 | | | 5.148438 | |  | |  | |  |
| 13.00833 | | 165.8841 | | | | 0.704168 | | | | 4976.172 | | | 4.820313 | |  | |  | |  |
| 13.01667 | | 165.9698 | | | | 0.704809 | | | | 4976.133 | | | 4.648438 | |  | |  | |  |
| 13.025 | | 166.0555 | | | | 0.706796 | | | | 4976.063 | | | 4.671875 | |  | |  | |  |
| 13.03333 | | 166.1429 | | | | 0.708841 | | | | 4976.063 | | | 4.59375 | |  | |  | |  |
| 13.04167 | | 166.2286 | | | | 0.70924 | | | | 4976.023 | | | 4.453125 | |  | |  | |  |
| 13.05 | | 166.3152 | | | | 0.710531 | | | | 4975.992 | | | 4.375 | |  | |  | |  |
| 13.05833 | | 166.4035 | | | | 0.712119 | | | | 4975.992 | | | 4.296875 | |  | |  | |  |
| 13.06667 | | 166.491 | | | | 0.71259 | | | | 4975.984 | | | 4.242188 | |  | |  | |  |
| 13.075 | | 166.5801 | | | | 0.713818 | | | | 4975.945 | | | 4.242188 | |  | |  | |  |
| 13.08333 | | 166.6667 | | | | 0.715065 | | | | 4975.914 | | | 4.3125 | |  | |  | |  |
| 13.09167 | | 166.7533 | | | | 0.714956 | | | | 4975.891 | | | 4.46875 | |  | |  | |  |
| 13.1 | | 166.8416 | | | | 0.716178 | | | | 4975.859 | | | 4.375 | |  | |  | |  |
| 13.10833 | | 166.929 | | | | 0.717821 | | | | 4975.836 | | | 4.3125 | |  | |  | |  |
| 13.11667 | | 167.0191 | | | | 0.718085 | | | | 4975.805 | | | 4.195313 | |  | |  | |  |
| 13.125 | | 167.1048 | | | | 0.719493 | | | | 4975.773 | | | 4.148438 | |  | |  | |  |
| 13.13333 | | 167.1896 | | | | 0.72189 | | | | 4975.734 | | | 4.289063 | |  | |  | |  |
| 13.14167 | | 167.2771 | | | | 0.722776 | | | | 4975.672 | | | 4.046875 | |  | |  | |  |
| 13.15 | | 167.3654 | | | | 0.724041 | | | | 4975.641 | | | 4.390625 | |  | |  | |  |
| 13.15833 | | 167.4511 | | | | 0.725787 | | | | 4975.617 | | | 4.539063 | |  | |  | |  |
| 13.16667 | | 167.5385 | | | | 0.726493 | | | | 4975.594 | | | 4.414063 | |  | |  | |  |
| 13.175 | | 167.6242 | | | | 0.727055 | | | | 4975.563 | | | 4.25 | |  | |  | |  |
| 13.18333 | | 167.7108 | | | | 0.728608 | | | | 4975.523 | | | 4.257813 | |  | |  | |  |
| 13.19167 | | 167.7982 | | | | 0.729086 | | | | 4975.508 | | | 4.507813 | |  | |  | |  |
| 13.2 | | 167.8848 | | | | 0.729889 | | | | 4975.43 | | | 4.460938 | |  | |  | |  |
| 13.20833 | | 167.9757 | | | | 0.731573 | | | | 4975.375 | | | 4.351563 | |  | |  | |  |
| 13.21667 | | 168.0693 | | | | 0.732059 | | | | 4975.344 | | | 4.445313 | |  | |  | |  |
| 13.225 | | 168.1576 | | | | 0.731976 | | | | 4975.328 | | | 4.320313 | |  | |  | |  |
| 13.23333 | | 168.2459 | | | | 0.733271 | | | | 4975.289 | | | 4.007813 | |  | |  | |  |
| 13.24167 | | 168.3342 | | | | 0.734724 | | | | 4975.227 | | | 3.84375 | |  | |  | |  |
| 13.25 | | 168.4243 | | | | 0.73601 | | | | 4975.188 | | | 3.898438 | |  | |  | |  |
| 13.25833 | | 168.5161 | | | | 0.73877 | | | | 4975.164 | | | 3.960938 | |  | |  | |  |
| 13.26667 | | 168.6062 | | | | 0.741687 | | | | 4975.117 | | | 4.039063 | |  | |  | |  |
| 13.275 | | 168.698 | | | | 0.742341 | | | | 4975.094 | | | 3.875 | |  | |  | |  |
| 13.28333 | | 168.7872 | | | | 0.743107 | | | | 4975.102 | | | 3.898438 | |  | |  | |  |
| 13.29167 | | 168.8746 | | | | 0.745027 | | | | 4975.094 | | | 3.914063 | |  | |  | |  |
| 13.3 | | 168.9603 | | | | 0.745047 | | | | 4975.063 | | | 3.71875 | |  | |  | |  |
| 13.30833 | | 169.0478 | | | | 0.746941 | | | | 4975.023 | | | 4.078125 | |  | |  | |  |
| 13.31667 | | 169.1352 | | | | 0.750282 | | | | 4974.961 | | | 4.023438 | |  | |  | |  |
| 13.325 | | 169.2226 | | | | 0.751855 | | | | 4974.961 | | | 3.984375 | |  | |  | |  |
| 13.33333 | | 169.3092 | | | | 0.752754 | | | | 4974.914 | | | 3.914063 | |  | |  | |  |
| 13.34167 | | 169.394 | | | | 0.755217 | | | | 4974.883 | | | 4.039063 | |  | |  | |  |
| 13.35 | | 169.4815 | | | | 0.755313 | | | | 4974.875 | | | 3.820313 | |  | |  | |  |
| 13.35833 | | 169.568 | | | | 0.755301 | | | | 4974.789 | | | 3.703125 | |  | |  | |  |
| 13.36667 | | 169.6555 | | | | 0.757115 | | | | 4974.766 | | | 3.539063 | |  | |  | |  |
| 13.375 | | 169.7438 | | | | 0.757454 | | | | 4974.727 | | | 3.625 | |  | |  | |  |
| 13.38333 | | 169.8295 | | | | 0.75807 | | | | 4974.703 | | | 3.710938 | |  | |  | |  |
| 13.39167 | | 169.9196 | | | | 0.759937 | | | | 4974.656 | | | 3.414063 | |  | |  | |  |
| 13.4 | | 170.0087 | | | | 0.760664 | | | | 4974.648 | | | 3.390625 | |  | |  | |  |
| 13.40833 | | 170.0988 | | | | 0.761313 | | | | 4974.641 | | | 3.476563 | |  | |  | |  |
| 13.41667 | | 170.188 | | | | 0.763393 | | | | 4974.641 | | | 3.1875 | |  | |  | |  |
| 13.425 | | 170.2781 | | | | 0.76414 | | | | 4974.602 | | | 3.203125 | |  | |  | |  |
| 13.43333 | | 170.3664 | | | | 0.764675 | | | | 4974.555 | | | 3.125 | |  | |  | |  |
| 13.44167 | | 170.4556 | | | | 0.766406 | | | | 4974.57 | | | 3.296875 | |  | |  | |  |
| 13.45 | | 170.5421 | | | | 0.767689 | | | | 4974.539 | | | 3.492188 | |  | |  | |  |
| 13.45833 | | 170.6296 | | | | 0.768238 | | | | 4974.508 | | | 3.546875 | |  | |  | |  |
| 13.46667 | | 170.7188 | | | | 0.770065 | | | | 4974.516 | | | 3.554688 | |  | |  | |  |
| 13.475 | | 170.8045 | | | | 0.771263 | | | | 4974.484 | | | 3.632813 | |  | |  | |  |
| 13.48333 | | 170.8936 | | | | 0.771689 | | | | 4974.469 | | | 3.71875 | |  | |  | |  |
| 13.49167 | | 170.9802 | | | | 0.773159 | | | | 4974.414 | | | 3.757813 | |  | |  | |  |
| 13.5 | | 171.0659 | | | | 0.773753 | | | | 4974.359 | | | 4.070313 | |  | |  | |  |
| 13.50833 | | 171.1516 | | | | 0.773331 | | | | 4974.313 | | | 4.03125 | |  | |  | |  |
| 13.51667 | | 171.2399 | | | | 0.774902 | | | | 4974.281 | | | 3.992188 | |  | |  | |  |
| 13.525 | | 171.3291 | | | | 0.776222 | | | | 4974.242 | | | 4.179688 | |  | |  | |  |
| 13.53333 | | 171.4201 | | | | 0.776686 | | | | 4974.203 | | | 4.203125 | |  | |  | |  |
| 13.54167 | | 171.5092 | | | | 0.77871 | | | | 4974.164 | | | 4.085938 | |  | |  | |  |
| 13.55 | | 171.5958 | | | | 0.780389 | | | | 4974.086 | | | 3.945313 | |  | |  | |  |
| 13.55833 | | 171.685 | | | | 0.781406 | | | | 4974.055 | | | 3.976563 | |  | |  | |  |
| 13.56667 | | 171.7742 | | | | 0.782391 | | | | 4974.023 | | | 4.226563 | |  | |  | |  |
| 13.575 | | 171.8634 | | | | 0.784644 | | | | 4973.961 | | | 4.015625 | |  | |  | |  |
| 13.58333 | | 171.9561 | | | | 0.78629 | | | | 4973.922 | | | 4.171875 | |  | |  | |  |
| 13.59167 | | 172.0479 | | | | 0.786838 | | | | 4973.906 | | | 4.257813 | |  | |  | |  |
| 13.6 | | 172.1362 | | | | 0.788795 | | | | 4973.898 | | | 4.179688 | |  | |  | |  |
| 13.60833 | | 172.2254 | | | | 0.789784 | | | | 4973.852 | | | 4.140625 | |  | |  | |  |
| 13.61667 | | 172.312 | | | | 0.78921 | | | | 4973.781 | | | 4.007813 | |  | |  | |  |
| 13.625 | | 172.4003 | | | | 0.790555 | | | | 4973.773 | | | 3.9375 | |  | |  | |  |
| 13.63333 | | 172.4938 | | | | 0.791938 | | | | 4973.727 | | | 4.195313 | |  | |  | |  |
| 13.64167 | | 172.583 | | | | 0.791936 | | | | 4973.688 | | | 4.21875 | |  | |  | |  |
| 13.65 | | 172.6705 | | | | 0.793567 | | | | 4973.664 | | | 4.234375 | |  | |  | |  |
| 13.65833 | | 172.7588 | | | | 0.794731 | | | | 4973.633 | | | 4.195313 | |  | |  | |  |
| 13.66667 | | 172.8462 | | | | 0.794517 | | | | 4973.617 | | | 4.046875 | |  | |  | |  |
| 13.675 | | 172.9354 | | | | 0.796341 | | | | 4973.594 | | | 4.070313 | |  | |  | |  |
| 13.68333 | | 173.0237 | | | | 0.797951 | | | | 4973.523 | | | 3.945313 | |  | |  | |  |
| 13.69167 | | 173.1094 | | | | 0.797553 | | | | 4973.484 | | | 3.859375 | |  | |  | |  |
| 13.7 | | 173.1995 | | | | 0.799488 | | | | 4973.453 | | | 3.859375 | |  | |  | |  |
| 13.70833 | | 173.2887 | | | | 0.801286 | | | | 4973.406 | | | 3.882813 | |  | |  | |  |
| 13.71667 | | 173.3735 | | | | 0.800897 | | | | 4973.398 | | | 3.9375 | |  | |  | |  |
| 13.725 | | 173.461 | | | | 0.802144 | | | | 4973.367 | | | 3.960938 | |  | |  | |  |
| 13.73333 | | 173.5484 | | | | 0.804192 | | | | 4973.344 | | | 3.75 | |  | |  | |  |
| 13.74167 | | 173.6376 | | | | 0.804782 | | | | 4973.328 | | | 3.65625 | |  | |  | |  |
| 13.75 | | 173.725 | | | | 0.806493 | | | | 4973.289 | | | 3.945313 | |  | |  | |  |
| 13.75833 | | 173.8125 | | | | 0.808831 | | | | 4973.258 | | | 3.929688 | |  | |  | |  |
| 13.76667 | | 173.899 | | | | 0.808944 | | | | 4973.227 | | | 3.921875 | |  | |  | |  |
| 13.775 | | 173.99 | | | | 0.810792 | | | | 4973.188 | | | 4.054688 | |  | |  | |  |
| 13.78333 | | 174.0783 | | | | 0.812062 | | | | 4973.188 | | | 3.96875 | |  | |  | |  |
| 13.79167 | | 174.1684 | | | | 0.811983 | | | | 4973.172 | | | 3.734375 | |  | |  | |  |
| 13.8 | | 174.2576 | | | | 0.812858 | | | | 4973.102 | | | 3.671875 | |  | |  | |  |
| 13.80833 | | 174.345 | | | | 0.814073 | | | | 4973.07 | | | 3.671875 | |  | |  | |  |
| 13.81667 | | 174.4342 | | | | 0.813225 | | | | 4973.039 | | | 3.632813 | |  | |  | |  |
| 13.825 | | 174.5216 | | | | 0.81408 | | | | 4972.992 | | | 3.734375 | |  | |  | |  |
| 13.83333 | | 174.6108 | | | | 0.814943 | | | | 4972.977 | | | 3.476563 | |  | |  | |  |
| 13.84167 | | 174.6991 | | | | 0.814278 | | | | 4972.969 | | | 3.492188 | |  | |  | |  |
| 13.85 | | 174.7901 | | | | 0.815494 | | | | 4972.945 | | | 3.625 | |  | |  | |  |
| 13.85833 | | 174.8779 | | | | 0.817279 | | | | 4972.914 | | | 3.507813 | |  | |  | |  |
| 13.86667 | | 174.9651 | | | | 0.817435 | | | | 4972.883 | | | 3.539063 | |  | |  | |  |
| 13.875 | | 175.0514 | | | | 0.819278 | | | | 4972.844 | | | 3.710938 | |  | |  | |  |
| 13.88333 | | 175.1404 | | | | 0.822157 | | | | 4972.852 | | | 3.84375 | |  | |  | |  |
| 13.89167 | | 175.2327 | | | | 0.824043 | | | | 4972.813 | | | 3.960938 | |  | |  | |  |
| 13.9 | | 175.3217 | | | | 0.82475 | | | | 4972.758 | | | 4.046875 | |  | |  | |  |
| 13.90833 | | 175.4115 | | | | 0.826568 | | | | 4972.75 | | | 3.984375 | |  | |  | |  |
| 13.91667 | | 175.4995 | | | | 0.827449 | | | | 4972.711 | | | 3.9375 | |  | |  | |  |
| 13.925 | | 175.5885 | | | | 0.827155 | | | | 4972.648 | | | 3.945313 | |  | |  | |  |
| 13.93333 | | 175.6791 | | | | 0.828611 | | | | 4972.602 | | | 3.960938 | |  | |  | |  |
| 13.94167 | | 175.768 | | | | 0.829903 | | | | 4972.555 | | | 3.90625 | |  | |  | |  |
| 13.95 | | 175.8595 | | | | 0.829805 | | | | 4972.508 | | | 4.070313 | |  | |  | |  |
| 13.95833 | | 175.9511 | | | | 0.831121 | | | | 4972.484 | | | 4.078125 | |  | |  | |  |
| 13.96667 | | 176.0434 | | | | 0.832869 | | | | 4972.453 | | | 4.046875 | |  | |  | |  |
| 13.975 | | 176.1306 | | | | 0.832244 | | | | 4972.422 | | | 4.015625 | |  | |  | |  |
| 13.98333 | | 176.2204 | | | | 0.833341 | | | | 4972.391 | | | 3.9375 | |  | |  | |  |
| 13.99167 | | 176.3076 | | | | 0.836287 | | | | 4972.352 | | | 3.9375 | |  | |  | |  |
| 14 | | 176.4009 | | | | 0.837119 | | | | 4972.305 | | | 3.984375 | |  | |  | |  |
| 14.00833 | | 176.4907 | | | | 0.837565 | | | | 4972.273 | | | 4.023438 | |  | |  | |  |
| 14.01667 | | 176.577 | | | | 0.840225 | | | | 4972.242 | | | 3.890625 | |  | |  | |  |
| 14.025 | | 176.6633 | | | | 0.840333 | | | | 4972.219 | | | 4.078125 | |  | |  | |  |
| 14.03333 | | 176.7514 | | | | 0.839912 | | | | 4972.188 | | | 4.132813 | |  | |  | |  |
| 14.04167 | | 176.836 | | | | 0.84195 | | | | 4972.156 | | | 3.960938 | |  | |  | |  |
| 14.05 | | 176.9198 | | | | 0.843176 | | | | 4972.117 | | | 3.945313 | |  | |  | |  |
| 14.05833 | | 177.0061 | | | | 0.844855 | | | | 4972.078 | | | 3.898438 | |  | |  | |  |
| 14.06667 | | 177.0924 | | | | 0.848149 | | | | 4972.063 | | | 3.914063 | |  | |  | |  |
| 14.075 | | 177.1831 | | | | 0.849101 | | | | 4972 | | | 4.054688 | |  | |  | |  |
| 14.08333 | | 177.2642 | | | | 0.849744 | | | | 4971.969 | | | 4.054688 | |  | |  | |  |
| 14.09167 | | 177.3497 | | | | 0.851416 | | | | 4971.953 | | | 4.101563 | |  | |  | |  |
| 14.1 | | 177.4361 | | | | 0.850195 | | | | 4971.922 | | | 3.953125 | |  | |  | |  |
| 14.10833 | | 177.525 | | | | 0.850591 | | | | 4971.898 | | | 3.828125 | |  | |  | |  |
| 14.11667 | | 177.6105 | | | | 0.852695 | | | | 4971.859 | | | 3.71875 | |  | |  | |  |
| 14.125 | | 177.6985 | | | | 0.852861 | | | | 4971.813 | | | 3.773438 | |  | |  | |  |
| 14.13333 | | 177.7892 | | | | 0.853295 | | | | 4971.781 | | | 3.664063 | |  | |  | |  |
| 14.14167 | | 177.8772 | | | | 0.855838 | | | | 4971.742 | | | 3.414063 | |  | |  | |  |
| 14.15 | | 177.9636 | | | | 0.856482 | | | | 4971.719 | | | 3.40625 | |  | |  | |  |
| 14.15833 | | 178.0499 | | | | 0.856674 | | | | 4971.703 | | | 3.390625 | |  | |  | |  |
| 14.16667 | | 178.1414 | | | | 0.858326 | | | | 4971.695 | | | 3.289063 | |  | |  | |  |
| 14.175 | | 178.2321 | | | | 0.85838 | | | | 4971.656 | | | 3.101563 | |  | |  | |  |
| 14.18333 | | 178.3227 | | | | 0.858094 | | | | 4971.641 | | | 3.21875 | |  | |  | |  |
| 14.19167 | | 178.4082 | | | | 0.859166 | | | | 4971.641 | | | 3.203125 | |  | |  | |  |
| 14.2 | | 178.4954 | | | | 0.860306 | | | | 4971.625 | | | 3.023438 | |  | |  | |  |
| 14.20833 | | 178.5809 | | | | 0.860276 | | | | 4971.594 | | | 3.140625 | |  | |  | |  |
| 14.21667 | | 178.6664 | | | | 0.862566 | | | | 4971.586 | | | 3.304688 | |  | |  | |  |
| 14.225 | | 178.7561 | | | | 0.862714 | | | | 4971.578 | | | 3.492188 | |  | |  | |  |
| 14.23333 | | 178.8477 | | | | 0.862808 | | | | 4971.539 | | | 3.585938 | |  | |  | |  |
| 14.24167 | | 178.9348 | | | | 0.865334 | | | | 4971.516 | | | 3.445313 | |  | |  | |  |
| 14.25 | | 179.0238 | | | | 0.86629 | | | | 4971.508 | | | 3.5 | |  | |  | |  |
| 14.25833 | | 179.1101 | | | | 0.866195 | | | | 4971.469 | | | 3.554688 | |  | |  | |  |
| 14.26667 | | 179.199 | | | | 0.867909 | | | | 4971.422 | | | 3.414063 | |  | |  | |  |
| 14.275 | | 179.288 | | | | 0.869044 | | | | 4971.359 | | | 3.429688 | |  | |  | |  |
| 14.28333 | | 179.3786 | | | | 0.869037 | | | | 4971.313 | | | 3.476563 | |  | |  | |  |
| 14.29167 | | 179.471 | | | | 0.870827 | | | | 4971.297 | | | 3.3125 | |  | |  | |  |
| 14.3 | | 179.5625 | | | | 0.872359 | | | | 4971.266 | | | 3.226563 | |  | |  | |  |
| 14.30833 | | 179.6514 | | | | 0.872901 | | | | 4971.227 | | | 3.101563 | |  | |  | |  |
| 14.31667 | | 179.7369 | | | | 0.874091 | | | | 4971.211 | | | 3.070313 | |  | |  | |  |
| 14.325 | | 179.825 | | | | 0.875982 | | | | 4971.18 | | | 2.875 | |  | |  | |  |
| 14.33333 | | 179.9139 | | | | 0.876312 | | | | 4971.148 | | | 3.054688 | |  | |  | |  |
| 14.34167 | | 180.002 | | | | 0.877599 | | | | 4971.141 | | | 3.164063 | |  | |  | |  |
| 14.35 | | 180.0926 | | | | 0.879799 | | | | 4971.133 | | | 3.234375 | |  | |  | |  |
| 14.35833 | | 180.1824 | | | | 0.880415 | | | | 4971.117 | | | 3.273438 | |  | |  | |  |
| 14.36667 | | 180.2679 | | | | 0.880492 | | | | 4971.094 | | | 3.453125 | |  | |  | |  |
| 14.375 | | 180.3568 | | | | 0.881889 | | | | 4971.094 | | | 3.625 | |  | |  | |  |
| 14.38333 | | 180.4449 | | | | 0.882939 | | | | 4971.047 | | | 3.53125 | |  | |  | |  |
| 14.39167 | | 180.5321 | | | | 0.883842 | | | | 4971.008 | | | 3.703125 | |  | |  | |  |
| 14.4 | | 180.6253 | | | | 0.884767 | | | | 4970.969 | | | 3.710938 | |  | |  | |  |
| 14.40833 | | 180.716 | | | | 0.885448 | | | | 4970.93 | | | 3.804688 | |  | |  | |  |
| 14.41667 | | 180.8049 | | | | 0.885995 | | | | 4970.875 | | | 3.789063 | |  | |  | |  |
| 14.425 | | 180.8964 | | | | 0.886733 | | | | 4970.82 | | | 3.578125 | |  | |  | |  |
| 14.43333 | | 180.9802 | | | | 0.887269 | | | | 4970.797 | | | 3.734375 | |  | |  | |  |
| 14.44167 | | 181.0699 | | | | 0.887161 | | | | 4970.742 | | | 3.648438 | |  | |  | |  |
| 14.45 | | 181.1615 | | | | 0.887477 | | | | 4970.711 | | | 3.59375 | |  | |  | |  |
| 14.45833 | | 181.2538 | | | | 0.888809 | | | | 4970.664 | | | 3.507813 | |  | |  | |  |
| 14.46667 | | 181.3402 | | | | 0.88821 | | | | 4970.641 | | | 3.570313 | |  | |  | |  |
| 14.475 | | 181.4283 | | | | 0.889607 | | | | 4970.641 | | | 3.4375 | |  | |  | |  |
| 14.48333 | | 181.5154 | | | | 0.892025 | | | | 4970.586 | | | 3.4375 | |  | |  | |  |
| 14.49167 | | 181.6052 | | | | 0.89366 | | | | 4970.57 | | | 3.398438 | |  | |  | |  |
| 14.5 | | 181.6933 | | | | 0.895455 | | | | 4970.547 | | | 3.398438 | |  | |  | |  |
| 14.50833 | | 181.7814 | | | | 0.897839 | | | | 4970.531 | | | 3.640625 | |  | |  | |  |
| 14.51667 | | 181.8746 | | | | 0.899202 | | | | 4970.492 | | | 3.429688 | |  | |  | |  |
| 14.525 | | 181.9644 | | | | 0.899741 | | | | 4970.484 | | | 3.429688 | |  | |  | |  |
| 14.53333 | | 182.0533 | | | | 0.901029 | | | | 4970.461 | | | 3.460938 | |  | |  | |  |
| 14.54167 | | 182.1397 | | | | 0.900994 | | | | 4970.438 | | | 3.46875 | |  | |  | |  |
| 14.55 | | 182.232 | | | | 0.902213 | | | | 4970.406 | | | 3.476563 | |  | |  | |  |
| 14.55833 | | 182.3227 | | | | 0.904165 | | | | 4970.344 | | | 3.609375 | |  | |  | |  |
| 14.56667 | | 182.4108 | | | | 0.904308 | | | | 4970.344 | | | 3.601563 | |  | |  | |  |
| 14.575 | | 182.4988 | | | | 0.904755 | | | | 4970.32 | | | 3.523438 | |  | |  | |  |
| 14.58333 | | 182.5895 | | | | 0.906112 | | | | 4970.289 | | | 3.507813 | |  | |  | |  |
| 14.59167 | | 182.6749 | | | | 0.9045 | | | | 4970.25 | | | 3.367188 | |  | |  | |  |
| 14.6 | | 182.7613 | | | | 0.904025 | | | | 4970.227 | | | 3.59375 | |  | |  | |  |
| 14.60833 | | 182.8468 | | | | 0.905595 | | | | 4970.18 | | | 3.671875 | |  | |  | |  |
| 14.61667 | | 182.934 | | | | 0.905857 | | | | 4970.148 | | | 3.671875 | |  | |  | |  |
| 14.625 | | 183.0194 | | | | 0.906447 | | | | 4970.117 | | | 3.609375 | |  | |  | |  |
| 14.63333 | | 183.1049 | | | | 0.908383 | | | | 4970.094 | | | 3.734375 | |  | |  | |  |
| 14.64167 | | 183.193 | | | | 0.908397 | | | | 4970.086 | | | 3.625 | |  | |  | |  |
| 14.65 | | 183.2827 | | | | 0.9089 | | | | 4970.023 | | | 3.640625 | |  | |  | |  |
| 14.65833 | | 183.3717 | | | | 0.909863 | | | | 4969.984 | | | 3.5625 | |  | |  | |  |
| 14.66667 | | 183.4563 | | | | 0.908815 | | | | 4969.961 | | | 3.5625 | |  | |  | |  |
| 14.675 | | 183.5478 | | | | 0.908621 | | | | 4969.93 | | | 3.671875 | |  | |  | |  |
| 14.68333 | | 183.6393 | | | | 0.91048 | | | | 4969.883 | | | 3.671875 | |  | |  | |  |
| 14.69167 | | 183.7308 | | | | 0.911129 | | | | 4969.875 | | | 3.546875 | |  | |  | |  |
| 14.7 | | 183.8206 | | | | 0.911913 | | | | 4969.844 | | | 3.539063 | |  | |  | |  |
| 14.70833 | | 183.9147 | | | | 0.914681 | | | | 4969.828 | | | 3.570313 | |  | |  | |  |
| 14.71667 | | 184.0045 | | | | 0.917228 | | | | 4969.797 | | | 3.492188 | |  | |  | |  |
| 14.725 | | 184.0917 | | | | 0.918478 | | | | 4969.758 | | | 3.4375 | |  | |  | |  |
| 14.73333 | | 184.1824 | | | | 0.920546 | | | | 4969.727 | | | 3.375 | |  | |  | |  |
| 14.74167 | | 184.273 | | | | 0.922421 | | | | 4969.703 | | | 3.679688 | |  | |  | |  |
| 14.75 | | 184.3628 | | | | 0.922961 | | | | 4969.68 | | | 3.6875 | |  | |  | |  |
| 14.75833 | | 184.4515 | | | | 0.923662 | | | | 4969.633 | | | 3.671875 | |  | |  | |  |
| 14.76667 | | 184.5368 | | | | 0.924286 | | | | 4969.625 | | | 3.6875 | |  | |  | |  |
| 14.775 | | 184.6255 | | | | 0.923827 | | | | 4969.594 | | | 3.601563 | |  | |  | |  |
| 14.78333 | | 184.7126 | | | | 0.924944 | | | | 4969.578 | | | 3.523438 | |  | |  | |  |
| 14.79167 | | 184.8021 | | | | 0.925838 | | | | 4969.5 | | | 3.40625 | |  | |  | |  |
| 14.8 | | 184.89 | | | | 0.925305 | | | | 4969.469 | | | 3.4375 | |  | |  | |  |
| 14.80833 | | 184.9821 | | | | 0.926285 | | | | 4969.438 | | | 3.398438 | |  | |  | |  |
| 14.81667 | | 185.0674 | | | | 0.927294 | | | | 4969.406 | | | 3.5 | |  | |  | |  |
| 14.825 | | 185.1544 | | | | 0.927122 | | | | 4969.398 | | | 3.359375 | |  | |  | |  |
| 14.83333 | | 185.244 | | | | 0.928223 | | | | 4969.367 | | | 3.46875 | |  | |  | |  |
| 14.84167 | | 185.3319 | | | | 0.930335 | | | | 4969.359 | | | 3.382813 | |  | |  | |  |
| 14.85 | | 185.4249 | | | | 0.931067 | | | | 4969.328 | | | 3.328125 | |  | |  | |  |
| 14.85833 | | 185.5153 | | | | 0.931277 | | | | 4969.305 | | | 3.445313 | |  | |  | |  |
| 14.86667 | | 185.6091 | | | | 0.932956 | | | | 4969.266 | | | 3.726563 | |  | |  | |  |
| 14.875 | | 185.6944 | | | | 0.933184 | | | | 4969.25 | | | 3.734375 | |  | |  | |  |
| 14.88333 | | 185.7866 | | | | 0.93276 | | | | 4969.211 | | | 3.710938 | |  | |  | |  |
| 14.89167 | | 185.8719 | | | | 0.933997 | | | | 4969.188 | | | 3.820313 | |  | |  | |  |
| 14.9 | | 185.9614 | | | | 0.935387 | | | | 4969.172 | | | 3.71875 | |  | |  | |  |
| 14.90833 | | 186.0502 | | | | 0.935869 | | | | 4969.125 | | | 3.796875 | |  | |  | |  |
| 14.91667 | | 186.1397 | | | | 0.937615 | | | | 4969.055 | | | 3.992188 | |  | |  | |  |
| 14.925 | | 186.231 | | | | 0.939451 | | | | 4969.023 | | | 3.984375 | |  | |  | |  |
| 14.93333 | | 186.3172 | | | | 0.93963 | | | | 4968.992 | | | 4.09375 | |  | |  | |  |
| 14.94167 | | 186.405 | | | | 0.941187 | | | | 4968.953 | | | 4.210938 | |  | |  | |  |
| 14.95 | | 186.4929 | | | | 0.942782 | | | | 4968.93 | | | 4.179688 | |  | |  | |  |
| 14.95833 | | 186.585 | | | | 0.942777 | | | | 4968.883 | | | 4.367188 | |  | |  | |  |
| 14.96667 | | 186.6755 | | | | 0.944147 | | | | 4968.828 | | | 4.179688 | |  | |  | |  |
| 14.975 | | 186.7684 | | | | 0.945756 | | | | 4968.789 | | | 4.335938 | |  | |  | |  |
| 14.98333 | | 186.8555 | | | | 0.946418 | | | | 4968.75 | | | 4.421875 | |  | |  | |  |
| 14.99167 | | 186.9467 | | | | 0.946859 | | | | 4968.695 | | | 4.289063 | |  | |  | |  |
| 15 | | 187.0397 | | | | 0.947887 | | | | 4968.664 | | | 4.234375 | |  | |  | |  |
| 15.00833 | | 187.1276 | | | | 0.948784 | | | | 4968.602 | | | 4.148438 | |  | |  | |  |
| 15.01667 | | 187.218 | | | | 0.949066 | | | | 4968.594 | | | 4.140625 | |  | |  | |  |
| 15.025 | | 187.3084 | | | | 0.950514 | | | | 4968.531 | | | 3.867188 | |  | |  | |  |
| 15.03333 | | 187.3989 | | | | 0.951789 | | | | 4968.484 | | | 3.914063 | |  | |  | |  |
| 15.04167 | | 187.4859 | | | | 0.951789 | | | | 4968.469 | | | 3.671875 | |  | |  | |  |
| 15.05 | | 187.572 | | | | 0.953267 | | | | 4968.453 | | | 3.898438 | |  | |  | |  |
| 15.05833 | | 187.6616 | | | | 0.954897 | | | | 4968.422 | | | 3.710938 | |  | |  | |  |
| 15.06667 | | 187.7529 | | | | 0.954898 | | | | 4968.391 | | | 3.6875 | |  | |  | |  |
| 15.075 | | 187.8407 | | | | 0.955519 | | | | 4968.398 | | | 3.757813 | |  | |  | |  |
| 15.08333 | | 187.9252 | | | | 0.957061 | | | | 4968.359 | | | 3.585938 | |  | |  | |  |
| 15.09167 | | 188.0122 | | | | 0.9565 | | | | 4968.359 | | | 3.507813 | |  | |  | |  |
| 15.1 | | 188.0992 | | | | 0.957671 | | | | 4968.297 | | | 3.648438 | |  | |  | |  |
| 15.10833 | | 188.1888 | | | | 0.959209 | | | | 4968.297 | | | 3.84375 | |  | |  | |  |
| 15.11667 | | 188.2758 | | | | 0.958918 | | | | 4968.266 | | | 3.71875 | |  | |  | |  |
| 15.125 | | 188.3654 | | | | 0.959947 | | | | 4968.227 | | | 3.804688 | |  | |  | |  |
| 15.13333 | | 188.4532 | | | | 0.961527 | | | | 4968.219 | | | 3.867188 | |  | |  | |  |
| 15.14167 | | 188.5394 | | | | 0.960891 | | | | 4968.203 | | | 3.953125 | |  | |  | |  |
| 15.15 | | 188.6298 | | | | 0.961702 | | | | 4968.148 | | | 3.9375 | |  | |  | |  |
| 15.15833 | | 188.7177 | | | | 0.962582 | | | | 4968.094 | | | 3.898438 | |  | |  | |  |
| 15.16667 | | 188.8073 | | | | 0.962018 | | | | 4968.07 | | | 4.039063 | |  | |  | |  |
| 15.175 | | 188.8986 | | | | 0.962899 | | | | 4968.031 | | | 4.257813 | |  | |  | |  |
| 15.18333 | | 188.9881 | | | | 0.96445 | | | | 4967.992 | | | 4.0625 | |  | |  | |  |
| 15.19167 | | 189.0751 | | | | 0.964627 | | | | 4967.953 | | | 4.109375 | |  | |  | |  |
| 15.2 | | 189.1638 | | | | 0.965806 | | | | 4967.914 | | | 3.945313 | |  | |  | |  |
| 15.20833 | | 189.2526 | | | | 0.968589 | | | | 4967.891 | | | 4.257813 | |  | |  | |  |
| 15.21667 | | 189.3421 | | | | 0.968776 | | | | 4967.836 | | | 4.203125 | |  | |  | |  |
| 15.225 | | 189.4317 | | | | 0.969458 | | | | 4967.766 | | | 4.3125 | |  | |  | |  |
| 15.23333 | | 189.5196 | | | | 0.971243 | | | | 4967.766 | | | 4.382813 | |  | |  | |  |
| 15.24167 | | 189.6092 | | | | 0.971521 | | | | 4967.727 | | | 4.421875 | |  | |  | |  |
| 15.25 | | 189.6962 | | | | 0.97206 | | | | 4967.719 | | | 4.40625 | |  | |  | |  |
| 15.25833 | | 189.7874 | | | | 0.974321 | | | | 4967.641 | | | 4.4375 | |  | |  | |  |
| 15.26667 | | 189.8762 | | | | 0.974914 | | | | 4967.609 | | | 4.578125 | |  | |  | |  |
| 15.275 | | 189.9649 | | | | 0.976117 | | | | 4967.547 | | | 4.460938 | |  | |  | |  |
| 15.28333 | | 190.0536 | | | | 0.978924 | | | | 4967.508 | | | 4.890625 | |  | |  | |  |
| 15.29167 | | 190.1423 | | | | 0.979178 | | | | 4967.461 | | | 4.710938 | |  | |  | |  |
| 15.3 | | 190.2336 | | | | 0.978489 | | | | 4967.438 | | | 4.59375 | |  | |  | |  |
| 15.30833 | | 190.3215 | | | | 0.979071 | | | | 4967.391 | | | 4.3125 | |  | |  | |  |
| 15.31667 | | 190.411 | | | | 0.97851 | | | | 4967.328 | | | 4.382813 | |  | |  | |  |
| 15.325 | | 190.5015 | | | | 0.978179 | | | | 4967.305 | | | 4.265625 | |  | |  | |  |
| 15.33333 | | 190.5945 | | | | 0.9793 | | | | 4967.211 | | | 4.203125 | |  | |  | |  |
| 15.34167 | | 190.6815 | | | | 0.980601 | | | | 4967.195 | | | 3.835938 | |  | |  | |  |
| 15.35 | | 190.771 | | | | 0.981428 | | | | 4967.172 | | | 3.5625 | |  | |  | |  |
| 15.35833 | | 190.8632 | | | | 0.983202 | | | | 4967.164 | | | 3.53125 | |  | |  | |  |
| 15.36667 | | 190.9536 | | | | 0.984629 | | | | 4967.133 | | | 3.164063 | |  | |  | |  |
| 15.375 | | 191.0423 | | | | 0.985225 | | | | 4967.109 | | | 3.140625 | |  | |  | |  |
| 15.38333 | | 191.1293 | | | | 0.986129 | | | | 4967.078 | | | 2.984375 | |  | |  | |  |
| 15.39167 | | 191.2232 | | | | 0.987552 | | | | 4967.102 | | | 2.96875 | |  | |  | |  |
| 15.4 | | 191.3153 | | | | 0.987865 | | | | 4967.102 | | | 2.835938 | |  | |  | |  |
| 15.40833 | | 191.4049 | | | | 0.987186 | | | | 4967.086 | | | 2.921875 | |  | |  | |  |
| 15.41667 | | 191.4961 | | | | 0.988618 | | | | 4967.109 | | | 2.921875 | |  | |  | |  |
| 15.425 | | 191.5857 | | | | 0.989374 | | | | 4967.094 | | | 3.351563 | |  | |  | |  |
| 15.43333 | | 191.677 | | | | 0.990359 | | | | 4967.086 | | | 3.398438 | |  | |  | |  |
| 15.44167 | | 191.764 | | | | 0.992945 | | | | 4967.07 | | | 3.476563 | |  | |  | |  |
| 15.45 | | 191.851 | | | | 0.99372 | | | | 4967.055 | | | 3.601563 | |  | |  | |  |
| 15.45833 | | 191.9423 | | | | 0.994862 | | | | 4967.023 | | | 3.375 | |  | |  | |  |
| 15.46667 | | 192.0336 | | | | 0.997124 | | | | 4967 | | | 3.625 | |  | |  | |  |
| 15.475 | | 192.1214 | | | | 0.997402 | | | | 4966.906 | | | 3.625 | |  | |  | |  |
| 15.48333 | | 192.2076 | | | | 0.996899 | | | | 4966.875 | | | 3.53125 | |  | |  | |  |
| 15.49167 | | 192.2997 | | | | 0.998835 | | | | 4966.836 | | | 3.453125 | |  | |  | |  |
| 15.5 | | 192.3884 | | | | 0.999316 | | | | 4966.797 | | | 3.523438 | |  | |  | |  |
| 15.50833 | | 192.4789 | | | | 0.998847 | | | | 4966.773 | | | 3.421875 | |  | |  | |  |
| 15.51667 | | 192.5676 | | | | 1.00044 | | | | 4966.727 | | | 3.398438 | |  | |  | |  |
| 15.525 | | 192.6614 | | | | 1.001079 | | | | 4966.695 | | | 3.546875 | |  | |  | |  |
| 15.53333 | | 192.7527 | | | | 1.000119 | | | | 4966.672 | | | 3.75 | |  | |  | |  |
| 15.54167 | | 192.844 | | | | 1.001454 | | | | 4966.648 | | | 3.875 | |  | |  | |  |
| 15.55 | | 192.937 | | | | 1.00298 | | | | 4966.609 | | | 3.890625 | |  | |  | |  |
| 15.55833 | | 193.0282 | | | | 1.003202 | | | | 4966.609 | | | 4.054688 | |  | |  | |  |
| 15.56667 | | 193.1221 | | | | 1.00483 | | | | 4966.563 | | | 4.023438 | |  | |  | |  |
| 15.575 | | 193.2099 | | | | 1.007014 | | | | 4966.516 | | | 4.054688 | |  | |  | |  |
| 15.58333 | | 193.297 | | | | 1.007311 | | | | 4966.453 | | | 4.132813 | |  | |  | |  |
| 15.59167 | | 193.3865 | | | | 1.008576 | | | | 4966.406 | | | 4.171875 | |  | |  | |  |
| 15.6 | | 193.4753 | | | | 1.010049 | | | | 4966.367 | | | 4.195313 | |  | |  | |  |
| 15.60833 | | 193.5597 | | | | 1.009779 | | | | 4966.313 | | | 4.257813 | |  | |  | |  |
| 15.61667 | | 193.6493 | | | | 1.011336 | | | | 4966.281 | | | 4.0625 | |  | |  | |  |
| 15.625 | | 193.7389 | | | | 1.012936 | | | | 4966.242 | | | 4.125 | |  | |  | |  |
| 15.63333 | | 193.8267 | | | | 1.012936 | | | | 4966.195 | | | 4.0625 | |  | |  | |  |
| 15.64167 | | 193.9154 | | | | 1.01359 | | | | 4966.156 | | | 4.007813 | |  | |  | |  |
| 15.65 | | 194.0016 | | | | 1.014841 | | | | 4966.117 | | | 3.976563 | |  | |  | |  |
| 15.65833 | | 194.0878 | | | | 1.014436 | | | | 4966.07 | | | 3.882813 | |  | |  | |  |
| 15.66667 | | 194.1799 | | | | 1.014953 | | | | 4966.063 | | | 3.90625 | |  | |  | |  |
| 15.675 | | 194.2686 | | | | 1.016199 | | | | 4966.023 | | | 3.90625 | |  | |  | |  |
| 15.68333 | | 194.3573 | | | | 1.016718 | | | | 4965.992 | | | 3.664063 | |  | |  | |  |
| 15.69167 | | 194.4495 | | | | 1.017612 | | | | 4965.961 | | | 3.539063 | |  | |  | |  |
| 15.7 | | 194.539 | | | | 1.019349 | | | | 4965.938 | | | 3.617188 | |  | |  | |  |
| 15.70833 | | 194.6277 | | | | 1.020138 | | | | 4965.914 | | | 3.601563 | |  | |  | |  |
| 15.71667 | | 194.7182 | | | | 1.020844 | | | | 4965.883 | | | 3.671875 | |  | |  | |  |
| 15.725 | | 194.8077 | | | | 1.022492 | | | | 4965.852 | | | 3.8125 | |  | |  | |  |
| 15.73333 | | 194.9007 | | | | 1.023363 | | | | 4965.852 | | | 4.140625 | |  | |  | |  |
| 15.74167 | | 194.9963 | | | | 1.023191 | | | | 4965.852 | | | 4.445313 | |  | |  | |  |
| 15.75 | | 195.0824 | | | | 1.024623 | | | | 4965.813 | | | 4.226563 | |  | |  | |  |
| 15.75833 | | 195.1737 | | | | 1.025875 | | | | 4965.781 | | | 4.15625 | |  | |  | |  |
| 15.76667 | | 195.2658 | | | | 1.025921 | | | | 4965.75 | | | 4.328125 | |  | |  | |  |
| 15.775 | | 195.358 | | | | 1.026852 | | | | 4965.695 | | | 4.460938 | |  | |  | |  |
| 15.78333 | | 195.4501 | | | | 1.028284 | | | | 4965.625 | | | 4.320313 | |  | |  | |  |
| 15.79167 | | 195.5388 | | | | 1.027161 | | | | 4965.547 | | | 4.304688 | |  | |  | |  |
| 15.8 | | 195.6267 | | | | 1.027864 | | | | 4965.539 | | | 4.140625 | |  | |  | |  |
| 15.80833 | | 195.718 | | | | 1.029815 | | | | 4965.516 | | | 3.898438 | |  | |  | |  |
| 15.81667 | | 195.8101 | | | | 1.029404 | | | | 4965.461 | | | 3.75 | |  | |  | |  |
| 15.825 | | 195.8997 | | | | 1.030487 | | | | 4965.391 | | | 3.398438 | |  | |  | |  |
| 15.83333 | | 195.9927 | | | | 1.032787 | | | | 4965.383 | | | 3.375 | |  | |  | |  |
| 15.84167 | | 196.0822 | | | | 1.033026 | | | | 4965.344 | | | 3.46875 | |  | |  | |  |
| 15.85 | | 196.1727 | | | | 1.033748 | | | | 4965.336 | | | 3.515625 | |  | |  | |  |
| 15.85833 | | 196.2614 | | | | 1.035706 | | | | 4965.336 | | | 3.484375 | |  | |  | |  |
| 15.86667 | | 196.3509 | | | | 1.035444 | | | | 4965.328 | | | 3.554688 | |  | |  | |  |
| 15.875 | | 196.4422 | | | | 1.035911 | | | | 4965.344 | | | 3.585938 | |  | |  | |  |
| 15.88333 | | 196.5318 | | | | 1.037733 | | | | 4965.32 | | | 3.742188 | |  | |  | |  |
| 15.89167 | | 196.6231 | | | | 1.037249 | | | | 4965.281 | | | 3.90625 | |  | |  | |  |
| 15.9 | | 196.7109 | | | | 1.037194 | | | | 4965.25 | | | 3.875 | |  | |  | |  |
| 15.90833 | | 196.798 | | | | 1.039284 | | | | 4965.227 | | | 4.125 | |  | |  | |  |
| 15.91667 | | 196.8884 | | | | 1.039285 | | | | 4965.18 | | | 4.421875 | |  | |  | |  |
| 15.925 | | 196.9797 | | | | 1.039586 | | | | 4965.141 | | | 4.476563 | |  | |  | |  |
| 15.93333 | | 197.0709 | | | | 1.041767 | | | | 4965.094 | | | 4.570313 | |  | |  | |  |
| 15.94167 | | 197.1614 | | | | 1.04301 | | | | 4965.031 | | | 4.640625 | |  | |  | |  |
| 15.95 | | 197.2518 | | | | 1.043392 | | | | 4965 | | | 4.679688 | |  | |  | |  |
| 15.95833 | | 197.3439 | | | | 1.045653 | | | | 4964.93 | | | 4.546875 | |  | |  | |  |
| 15.96667 | | 197.4395 | | | | 1.047291 | | | | 4964.852 | | | 4.59375 | |  | |  | |  |
| 15.975 | | 197.5282 | | | | 1.047457 | | | | 4964.805 | | | 4.539063 | |  | |  | |  |
| 15.98333 | | 197.6186 | | | | 1.048529 | | | | 4964.758 | | | 4.625 | |  | |  | |  |
| 15.99167 | | 197.7124 | | | | 1.050168 | | | | 4964.703 | | | 4.34375 | |  | |  | |  |
| 16 | | 197.8037 | | | | 1.05034 | | | | 4964.656 | | | 4.382813 | |  | |  | |  |
| 16.00833 | | 197.895 | | | | 1.049904 | | | | 4964.633 | | | 4.375 | |  | |  | |  |
| 16.01667 | | 197.9854 | | | | 1.051548 | | | | 4964.602 | | | 4.09375 | |  | |  | |  |
| 16.025 | | 198.075 | | | | 1.052363 | | | | 4964.57 | | | 4.039063 | |  | |  | |  |
| 16.03333 | | 198.1671 | | | | 1.052342 | | | | 4964.523 | | | 3.859375 | |  | |  | |  |
| 16.04167 | | 198.2567 | | | | 1.054372 | | | | 4964.523 | | | 4.007813 | |  | |  | |  |
| 16.05 | | 198.3437 | | | | 1.0557 | | | | 4964.484 | | | 4.023438 | |  | |  | |  |
| 16.05833 | | 198.4324 | | | | 1.055842 | | | | 4964.453 | | | 3.914063 | |  | |  | |  |
| 16.06667 | | 198.5246 | | | | 1.058305 | | | | 4964.461 | | | 4.070313 | |  | |  | |  |
| 16.075 | | 198.615 | | | | 1.058857 | | | | 4964.43 | | | 4.34375 | |  | |  | |  |
| 16.08333 | | 198.7029 | | | | 1.058317 | | | | 4964.422 | | | 4.359375 | |  | |  | |  |
| 16.09167 | | 198.7899 | | | | 1.059795 | | | | 4964.375 | | | 4.40625 | |  | |  | |  |
| 16.1 | | 198.8794 | | | | 1.059955 | | | | 4964.336 | | | 4.554688 | |  | |  | |  |
| 16.10833 | | 198.9699 | | | | 1.059631 | | | | 4964.32 | | | 4.609375 | |  | |  | |  |
| 16.11667 | | 199.0586 | | | | 1.061129 | | | | 4964.266 | | | 4.640625 | |  | |  | |  |
| 16.125 | | 199.149 | | | | 1.061438 | | | | 4964.188 | | | 4.710938 | |  | |  | |  |
| 16.13333 | | 199.2386 | | | | 1.061922 | | | | 4964.148 | | | 4.703125 | |  | |  | |  |
| 16.14167 | | 199.3307 | | | | 1.063706 | | | | 4964.102 | | | 4.8125 | |  | |  | |  |
| 16.15 | | 199.4169 | | | | 1.064259 | | | | 4964.031 | | | 4.59375 | |  | |  | |  |
| 16.15833 | | 199.5039 | | | | 1.064399 | | | | 4964 | | | 4.609375 | |  | |  | |  |
| 16.16667 | | 199.5943 | | | | 1.066059 | | | | 4963.953 | | | 4.515625 | |  | |  | |  |
| 16.175 | | 199.6856 | | | | 1.066417 | | | | 4963.898 | | | 4.523438 | |  | |  | |  |
| 16.18333 | | 199.7769 | | | | 1.066495 | | | | 4963.859 | | | 4.507813 | |  | |  | |  |
| 16.19167 | | 199.8698 | | | | 1.068627 | | | | 4963.805 | | | 4.570313 | |  | |  | |  |
| 16.2 | | 199.9637 | | | | 1.069948 | | | | 4963.797 | | | 4.679688 | |  | |  | |  |
| 16.20833 | | 200.0533 | | | | 1.069427 | | | | 4963.766 | | | 4.570313 | |  | |  | |  |
| 16.21667 | | 200.1445 | | | | 1.071507 | | | | 4963.75 | | | 4.445313 | |  | |  | |  |
| 16.225 | | 200.2358 | | | | 1.073217 | | | | 4963.719 | | | 4.429688 | |  | |  | |  |
| 16.23333 | | 200.3297 | | | | 1.072813 | | | | 4963.688 | | | 4.367188 | |  | |  | |  |
| 16.24167 | | 200.4218 | | | | 1.074607 | | | | 4963.641 | | | 4.164063 | |  | |  | |  |
| 16.25 | | 200.5131 | | | | 1.076724 | | | | 4963.586 | | | 4.359375 | |  | |  | |  |
| 16.25833 | | 200.6069 | | | | 1.077328 | | | | 4963.555 | | | 4.335938 | |  | |  | |  |
| 16.26667 | | 200.699 | | | | 1.078345 | | | | 4963.539 | | | 4.265625 | |  | |  | |  |
| 16.275 | | 200.7886 | | | | 1.079855 | | | | 4963.5 | | | 4.125 | |  | |  | |  |
| 16.28333 | | 200.8765 | | | | 1.080003 | | | | 4963.484 | | | 4.117188 | |  | |  | |  |
| 16.29167 | | 200.9703 | | | | 1.080831 | | | | 4963.461 | | | 3.914063 | |  | |  | |  |
| 16.3 | | 201.0624 | | | | 1.082315 | | | | 4963.406 | | | 3.945313 | |  | |  | |  |
| 16.30833 | | 201.1563 | | | | 1.082629 | | | | 4963.359 | | | 3.921875 | |  | |  | |  |
| 16.31667 | | 201.2475 | | | | 1.083087 | | | | 4963.328 | | | 4.140625 | |  | |  | |  |
| 16.325 | | 201.3405 | | | | 1.084531 | | | | 4963.32 | | | 4.046875 | |  | |  | |  |
| 16.33333 | | 201.4344 | | | | 1.084859 | | | | 4963.289 | | | 3.953125 | |  | |  | |  |
| 16.34167 | | 201.5239 | | | | 1.085177 | | | | 4963.281 | | | 3.867188 | |  | |  | |  |
| 16.35 | | 201.6109 | | | | 1.08679 | | | | 4963.234 | | | 3.84375 | |  | |  | |  |
| 16.35833 | | 201.7014 | | | | 1.088154 | | | | 4963.211 | | | 3.929688 | |  | |  | |  |
| 16.36667 | | 201.7927 | | | | 1.089245 | | | | 4963.141 | | | 3.890625 | |  | |  | |  |
| 16.375 | | 201.8822 | | | | 1.091145 | | | | 4963.125 | | | 3.8125 | |  | |  | |  |
| 16.38333 | | 201.9727 | | | | 1.091572 | | | | 4963.094 | | | 3.742188 | |  | |  | |  |
| 16.39167 | | 202.0588 | | | | 1.091776 | | | | 4963.086 | | | 3.890625 | |  | |  | |  |
| 16.4 | | 202.1475 | | | | 1.093618 | | | | 4963.055 | | | 3.984375 | |  | |  | |  |
| 16.40833 | | 202.2388 | | | | 1.093815 | | | | 4963.016 | | | 4.125 | |  | |  | |  |
| 16.41667 | | 202.3284 | | | | 1.093871 | | | | 4962.984 | | | 4.039063 | |  | |  | |  |
| 16.425 | | 202.4205 | | | | 1.095371 | | | | 4962.977 | | | 4.289063 | |  | |  | |  |
| 16.43333 | | 202.5144 | | | | 1.096338 | | | | 4962.945 | | | 4.273438 | |  | |  | |  |
| 16.44167 | | 202.6065 | | | | 1.096096 | | | | 4962.898 | | | 4.453125 | |  | |  | |  |
| 16.45 | | 202.6961 | | | | 1.097275 | | | | 4962.844 | | | 4.40625 | |  | |  | |  |
| 16.45833 | | 202.7856 | | | | 1.098883 | | | | 4962.797 | | | 4.710938 | |  | |  | |  |
| 16.46667 | | 202.8752 | | | | 1.098904 | | | | 4962.758 | | | 4.773438 | |  | |  | |  |
| 16.475 | | 202.9682 | | | | 1.0997 | | | | 4962.688 | | | 4.828125 | |  | |  | |  |
| 16.48333 | | 203.0585 | | | | 1.101289 | | | | 4962.656 | | | 4.6875 | |  | |  | |  |
| 16.49167 | | 203.1485 | | | | 1.100881 | | | | 4962.594 | | | 4.78125 | |  | |  | |  |
| 16.5 | | 203.2375 | | | | 1.10144 | | | | 4962.563 | | | 4.695313 | |  | |  | |  |
| 16.50833 | | 203.3274 | | | | 1.103174 | | | | 4962.477 | | | 4.554688 | |  | |  | |  |
| 16.51667 | | 203.419 | | | | 1.103279 | | | | 4962.43 | | | 4.546875 | |  | |  | |  |
| 16.525 | | 203.5089 | | | | 1.103742 | | | | 4962.383 | | | 4.476563 | |  | |  | |  |
| 16.53333 | | 203.5972 | | | | 1.105504 | | | | 4962.359 | | | 4.523438 | |  | |  | |  |
| 16.54167 | | 203.6862 | | | | 1.105356 | | | | 4962.305 | | | 4.523438 | |  | |  | |  |
| 16.55 | | 203.7778 | | | | 1.106219 | | | | 4962.289 | | | 4.515625 | |  | |  | |  |
| 16.55833 | | 203.8677 | | | | 1.108928 | | | | 4962.266 | | | 4.609375 | |  | |  | |  |
| 16.56667 | | 203.9559 | | | | 1.109296 | | | | 4962.227 | | | 4.6875 | |  | |  | |  |
| 16.575 | | 204.0417 | | | | 1.109967 | | | | 4962.211 | | | 4.632813 | |  | |  | |  |
| 16.58333 | | 204.1291 | | | | 1.112943 | | | | 4962.156 | | | 4.820313 | |  | |  | |  |
| 16.59167 | | 204.2165 | | | | 1.113376 | | | | 4962.117 | | | 4.914063 | |  | |  | |  |
| 16.6 | | 204.3048 | | | | 1.113227 | | | | 4962.078 | | | 5.023438 | |  | |  | |  |
| 16.60833 | | 204.3913 | | | | 1.115119 | | | | 4962.031 | | | 5.0625 | |  | |  | |  |
| 16.61667 | | 204.4812 | | | | 1.115525 | | | | 4961.977 | | | 5.15625 | |  | |  | |  |
| 16.625 | | 204.5695 | | | | 1.114834 | | | | 4961.953 | | | 5.023438 | |  | |  | |  |
| 16.63333 | | 204.6569 | | | | 1.117013 | | | | 4961.891 | | | 5.007813 | |  | |  | |  |
| 16.64167 | | 204.746 | | | | 1.118034 | | | | 4961.836 | | | 5.101563 | |  | |  | |  |
| 16.65 | | 204.835 | | | | 1.118682 | | | | 4961.773 | | | 4.945313 | |  | |  | |  |
| 16.65833 | | 204.925 | | | | 1.121016 | | | | 4961.734 | | | 5.109375 | |  | |  | |  |
| 16.66667 | | 205.014 | | | | 1.122091 | | | | 4961.672 | | | 5.039063 | |  | |  | |  |
| 16.675 | | 205.1023 | | | | 1.121808 | | | | 4961.648 | | | 5.09375 | |  | |  | |  |
| 16.68333 | | 205.188 | | | | 1.123424 | | | | 4961.617 | | | 5.007813 | |  | |  | |  |
| 16.69167 | | 205.2796 | | | | 1.124713 | | | | 4961.57 | | | 5.171875 | |  | |  | |  |
| 16.7 | | 205.3703 | | | | 1.125173 | | | | 4961.539 | | | 5.0625 | |  | |  | |  |
| 16.70833 | | 205.4619 | | | | 1.126767 | | | | 4961.477 | | | 5.1875 | |  | |  | |  |
| 16.71667 | | 205.5501 | | | | 1.129205 | | | | 4961.438 | | | 5.320313 | |  | |  | |  |
| 16.725 | | 205.6359 | | | | 1.13002 | | | | 4961.398 | | | 5.289063 | |  | |  | |  |
| 16.73333 | | 205.725 | | | | 1.131272 | | | | 4961.367 | | | 5.226563 | |  | |  | |  |
| 16.74167 | | 205.8174 | | | | 1.133464 | | | | 4961.305 | | | 4.945313 | |  | |  | |  |
| 16.75 | | 205.9064 | | | | 1.134072 | | | | 4961.281 | | | 5.078125 | |  | |  | |  |
| 16.75833 | | 205.9963 | | | | 1.134526 | | | | 4961.219 | | | 5.171875 | |  | |  | |  |
| 16.76667 | | 206.0862 | | | | 1.136198 | | | | 4961.156 | | | 5.078125 | |  | |  | |  |
| 16.775 | | 206.1703 | | | | 1.136542 | | | | 4961.125 | | | 5.09375 | |  | |  | |  |
| 16.78333 | | 206.2586 | | | | 1.137193 | | | | 4961.094 | | | 5.15625 | |  | |  | |  |
| 16.79167 | | 206.346 | | | | 1.138838 | | | | 4961.086 | | | 5.03125 | |  | |  | |  |
| 16.8 | | 206.4342 | | | | 1.138915 | | | | 4961.031 | | | 5.242188 | |  | |  | |  |
| 16.80833 | | 206.5275 | | | | 1.139376 | | | | 4960.969 | | | 5.140625 | |  | |  | |  |
| 16.81667 | | 206.619 | | | | 1.141271 | | | | 4960.938 | | | 5.367188 | |  | |  | |  |
| 16.825 | | 206.7039 | | | | 1.141904 | | | | 4960.883 | | | 5.640625 | |  | |  | |  |
| 16.83333 | | 206.7955 | | | | 1.142089 | | | | 4960.844 | | | 5.453125 | |  | |  | |  |
| 16.84167 | | 206.8854 | | | | 1.143992 | | | | 4960.813 | | | 5.148438 | |  | |  | |  |
| 16.85 | | 206.9762 | | | | 1.145309 | | | | 4960.742 | | | 5.210938 | |  | |  | |  |
| 16.85833 | | 207.0677 | | | | 1.146025 | | | | 4960.703 | | | 5.117188 | |  | |  | |  |
| 16.86667 | | 207.1601 | | | | 1.148164 | | | | 4960.633 | | | 5.15625 | |  | |  | |  |
| 16.875 | | 207.25 | | | | 1.149986 | | | | 4960.547 | | | 5.40625 | |  | |  | |  |
| 16.88333 | | 207.3433 | | | | 1.149835 | | | | 4960.523 | | | 5.21875 | |  | |  | |  |
| 16.89167 | | 207.4299 | | | | 1.151494 | | | | 4960.523 | | | 5.234375 | |  | |  | |  |
| 16.9 | | 207.5164 | | | | 1.15255 | | | | 4960.484 | | | 5.203125 | |  | |  | |  |
| 16.90833 | | 207.608 | | | | 1.151653 | | | | 4960.445 | | | 5.164063 | |  | |  | |  |
| 16.91667 | | 207.6987 | | | | 1.153127 | | | | 4960.398 | | | 5.273438 | |  | |  | |  |
| 16.925 | | 207.7878 | | | | 1.154686 | | | | 4960.328 | | | 5.3125 | |  | |  | |  |
| 16.93333 | | 207.8786 | | | | 1.154853 | | | | 4960.313 | | | 5.414063 | |  | |  | |  |
| 16.94167 | | 207.9693 | | | | 1.155538 | | | | 4960.258 | | | 5.34375 | |  | |  | |  |
| 16.95 | | 208.0559 | | | | 1.157451 | | | | 4960.227 | | | 5.335938 | |  | |  | |  |
| 16.95833 | | 208.1458 | | | | 1.15851 | | | | 4960.18 | | | 5.15625 | |  | |  | |  |
| 16.96667 | | 208.2357 | | | | 1.160133 | | | | 4960.125 | | | 5.15625 | |  | |  | |  |
| 16.975 | | 208.3273 | | | | 1.162391 | | | | 4960.07 | | | 5.148438 | |  | |  | |  |
| 16.98333 | | 208.418 | | | | 1.163481 | | | | 4960.016 | | | 5.125 | |  | |  | |  |
| 16.99167 | | 208.5096 | | | | 1.16373 | | | | 4959.977 | | | 5.09375 | |  | |  | |  |
| 17 | | 208.597 | | | | 1.165081 | | | | 4959.93 | | | 5.125 | |  | |  | |  |
| 17.00833 | | 208.6877 | | | | 1.165835 | | | | 4959.914 | | | 5.140625 | |  | |  | |  |
| 17.01667 | | 208.7751 | | | | 1.165725 | | | | 4959.867 | | | 5.164063 | |  | |  | |  |
| 17.025 | | 208.8634 | | | | 1.167278 | | | | 4959.828 | | | 5.054688 | |  | |  | |  |
| 17.03333 | | 208.9558 | | | | 1.168511 | | | | 4959.789 | | | 5.09375 | |  | |  | |  |
| 17.04167 | | 209.0465 | | | | 1.16837 | | | | 4959.75 | | | 5.03125 | |  | |  | |  |
| 17.05 | | 209.1348 | | | | 1.169194 | | | | 4959.711 | | | 5.109375 | |  | |  | |  |
| 17.05833 | | 209.2238 | | | | 1.170358 | | | | 4959.656 | | | 4.992188 | |  | |  | |  |
| 17.06667 | | 209.3162 | | | | 1.169948 | | | | 4959.617 | | | 5.039063 | |  | |  | |  |
| 17.075 | | 209.4028 | | | | 1.17083 | | | | 4959.586 | | | 4.84375 | |  | |  | |  |
| 17.08333 | | 209.4927 | | | | 1.172361 | | | | 4959.539 | | | 4.921875 | |  | |  | |  |
| 17.09167 | | 209.5776 | | | | 1.171583 | | | | 4959.5 | | | 4.976563 | |  | |  | |  |
| 17.1 | | 209.6667 | | | | 1.172328 | | | | 4959.445 | | | 4.875 | |  | |  | |  |
| 17.10833 | | 209.7575 | | | | 1.174713 | | | | 4959.422 | | | 4.867188 | |  | |  | |  |
| 17.11667 | | 209.844 | | | | 1.174743 | | | | 4959.367 | | | 4.820313 | |  | |  | |  |
| 17.125 | | 209.9306 | | | | 1.175793 | | | | 4959.359 | | | 4.773438 | |  | |  | |  |
| 17.13333 | | 210.0205 | | | | 1.177987 | | | | 4959.313 | | | 4.6875 | |  | |  | |  |
| 17.14167 | | 210.1088 | | | | 1.177422 | | | | 4959.266 | | | 4.65625 | |  | |  | |  |
| 17.15 | | 210.1953 | | | | 1.177812 | | | | 4959.234 | | | 4.601563 | |  | |  | |  |
| 17.15833 | | 210.2861 | | | | 1.179504 | | | | 4959.195 | | | 4.71875 | |  | |  | |  |
| 17.16667 | | 210.3768 | | | | 1.179265 | | | | 4959.164 | | | 4.460938 | |  | |  | |  |
| 17.175 | | 210.4709 | | | | 1.179363 | | | | 4959.125 | | | 4.476563 | |  | |  | |  |
| 17.18333 | | 210.5608 | | | | 1.181957 | | | | 4959.102 | | | 4.539063 | |  | |  | |  |
| 17.19167 | | 210.6507 | | | | 1.182106 | | | | 4959.063 | | | 4.742188 | |  | |  | |  |
| 17.2 | | 210.7414 | | | | 1.182485 | | | | 4959.039 | | | 4.84375 | |  | |  | |  |
| 17.20833 | | 210.833 | | | | 1.184852 | | | | 4958.977 | | | 5.03125 | |  | |  | |  |
| 17.21667 | | 210.9212 | | | | 1.185721 | | | | 4958.969 | | | 5.023438 | |  | |  | |  |
| 17.225 | | 211.0112 | | | | 1.186742 | | | | 4958.93 | | | 5.234375 | |  | |  | |  |
| 17.23333 | | 211.0994 | | | | 1.189255 | | | | 4958.875 | | | 5.273438 | |  | |  | |  |
| 17.24167 | | 211.1876 | | | | 1.189873 | | | | 4958.813 | | | 5.179688 | |  | |  | |  |
| 17.25 | | 211.2784 | | | | 1.190129 | | | | 4958.758 | | | 5.367188 | |  | |  | |  |
| 17.25833 | | 211.3683 | | | | 1.192142 | | | | 4958.688 | | | 5.320313 | |  | |  | |  |
| 17.26667 | | 211.4582 | | | | 1.192347 | | | | 4958.641 | | | 5.195313 | |  | |  | |  |
| 17.275 | | 211.5481 | | | | 1.191689 | | | | 4958.578 | | | 5.242188 | |  | |  | |  |
| 17.28333 | | 211.638 | | | | 1.193707 | | | | 4958.523 | | | 5.34375 | |  | |  | |  |
| 17.29167 | | 211.7287 | | | | 1.193829 | | | | 4958.5 | | | 5.171875 | |  | |  | |  |
| 17.3 | | 211.8203 | | | | 1.194034 | | | | 4958.422 | | | 5.132813 | |  | |  | |  |
| 17.30833 | | 211.9102 | | | | 1.197023 | | | | 4958.391 | | | 5.289063 | |  | |  | |  |
| 17.31667 | | 212.0043 | | | | 1.198306 | | | | 4958.359 | | | 5.203125 | |  | |  | |  |
| 17.325 | | 212.0959 | | | | 1.199317 | | | | 4958.305 | | | 5.398438 | |  | |  | |  |
| 17.33333 | | 212.1824 | | | | 1.200963 | | | | 4958.25 | | | 5.4375 | |  | |  | |  |
| 17.34167 | | 212.2679 | | | | 1.201831 | | | | 4958.234 | | | 5.328125 | |  | |  | |  |
| 17.35 | | 212.3567 | | | | 1.201497 | | | | 4958.188 | | | 5.601563 | |  | |  | |  |
| 17.35833 | | 212.4464 | | | | 1.20242 | | | | 4958.133 | | | 5.46875 | |  | |  | |  |
| 17.36667 | | 212.5376 | | | | 1.203394 | | | | 4958.094 | | | 5.453125 | |  | |  | |  |
| 17.375 | | 212.6272 | | | | 1.203353 | | | | 4958.023 | | | 5.554688 | |  | |  | |  |
| 17.38333 | | 212.7144 | | | | 1.204351 | | | | 4957.977 | | | 5.453125 | |  | |  | |  |
| 17.39167 | | 212.8056 | | | | 1.205814 | | | | 4957.945 | | | 5.320313 | |  | |  | |  |
| 17.4 | | 212.8944 | | | | 1.206346 | | | | 4957.859 | | | 5.3125 | |  | |  | |  |
| 17.40833 | | 212.9841 | | | | 1.207749 | | | | 4957.836 | | | 5.320313 | |  | |  | |  |
| 17.41667 | | 213.0728 | | | | 1.210117 | | | | 4957.789 | | | 5.132813 | |  | |  | |  |
| 17.425 | | 213.1641 | | | | 1.210705 | | | | 4957.734 | | | 5.351563 | |  | |  | |  |
| 17.43333 | | 213.2521 | | | | 1.211443 | | | | 4957.703 | | | 5.484375 | |  | |  | |  |
| 17.44167 | | 213.3401 | | | | 1.213168 | | | | 4957.68 | | | 5.210938 | |  | |  | |  |
| 17.45 | | 213.4288 | | | | 1.213607 | | | | 4957.633 | | | 5.226563 | |  | |  | |  |
| 17.45833 | | 213.5176 | | | | 1.213345 | | | | 4957.586 | | | 5.3125 | |  | |  | |  |
| 17.46667 | | 213.6064 | | | | 1.215257 | | | | 4957.563 | | | 5.453125 | |  | |  | |  |
| 17.475 | | 213.6961 | | | | 1.216206 | | | | 4957.5 | | | 5.460938 | |  | |  | |  |
| 17.48333 | | 213.784 | | | | 1.216237 | | | | 4957.438 | | | 5.6875 | |  | |  | |  |
| 17.49167 | | 213.872 | | | | 1.218674 | | | | 4957.422 | | | 5.75 | |  | |  | |  |
| 17.5 | | 213.9641 | | | | 1.220285 | | | | 4957.391 | | | 6.007813 | |  | |  | |  |
| 17.50833 | | 214.0545 | | | | 1.219918 | | | | 4957.328 | | | 5.953125 | |  | |  | |  |
| 17.51667 | | 214.1466 | | | | 1.221922 | | | | 4957.266 | | | 5.890625 | |  | |  | |  |
| 17.525 | | 214.237 | | | | 1.223222 | | | | 4957.211 | | | 6.40625 | |  | |  | |  |
| 17.53333 | | 214.325 | | | | 1.222493 | | | | 4957.141 | | | 6.71875 | |  | |  | |  |
| 17.54167 | | 214.4113 | | | | 1.223909 | | | | 4957.086 | | | 7.03125 | |  | |  | |  |
| 17.55 | | 214.5018 | | | | 1.2255 | | | | 4957 | | | 7.046875 | |  | |  | |  |
| 17.55833 | | 214.5881 | | | | 1.225605 | | | | 4956.961 | | | 7.164063 | |  | |  | |  |
| 17.56667 | | 214.6769 | | | | 1.227168 | | | | 4956.914 | | | 7.179688 | |  | |  | |  |
| 17.575 | | 214.7641 | | | | 1.22882 | | | | 4956.797 | | | 7.25 | |  | |  | |  |
| 17.58333 | | 214.8496 | | | | 1.228548 | | | | 4956.695 | | | 7.25 | |  | |  | |  |
| 17.59167 | | 214.9375 | | | | 1.230256 | | | | 4956.594 | | | 7.59375 | |  | |  | |  |
| 17.6 | | 215.0239 | | | | 1.231799 | | | | 4956.531 | | | 7.59375 | |  | |  | |  |
| 17.60833 | | 215.1127 | | | | 1.231127 | | | | 4956.453 | | | 7.570313 | |  | |  | |  |
| 17.61667 | | 215.199 | | | | 1.231954 | | | | 4956.383 | | | 7.46875 | |  | |  | |  |
| 17.625 | | 215.2903 | | | | 1.234117 | | | | 4956.32 | | | 7.164063 | |  | |  | |  |
| 17.63333 | | 215.3766 | | | | 1.234039 | | | | 4956.25 | | | 7.242188 | |  | |  | |  |
| 17.64167 | | 215.467 | | | | 1.234496 | | | | 4956.156 | | | 7.257813 | |  | |  | |  |
| 17.65 | | 215.5558 | | | | 1.236302 | | | | 4956.094 | | | 7.304688 | |  | |  | |  |
| 17.65833 | | 215.6454 | | | | 1.235794 | | | | 4956.039 | | | 7.40625 | |  | |  | |  |
| 17.66667 | | 215.7359 | | | | 1.235635 | | | | 4955.992 | | | 7.351563 | |  | |  | |  |
| 17.675 | | 215.823 | | | | 1.237545 | | | | 4955.969 | | | 7.273438 | |  | |  | |  |
| 17.68333 | | 215.9118 | | | | 1.237662 | | | | 4955.898 | | | 7.15625 | |  | |  | |  |
| 17.69167 | | 216.0006 | | | | 1.238159 | | | | 4955.844 | | | 7.226563 | |  | |  | |  |
| 17.7 | | 216.0919 | | | | 1.240477 | | | | 4955.781 | | | 7.164063 | |  | |  | |  |
| 17.70833 | | 216.1815 | | | | 1.240603 | | | | 4955.703 | | | 7.132813 | |  | |  | |  |
| 17.71667 | | 216.2744 | | | | 1.24128 | | | | 4955.648 | | | 7.273438 | |  | |  | |  |
| 17.725 | | 216.3616 | | | | 1.24352 | | | | 4955.602 | | | 7.179688 | |  | |  | |  |
| 17.73333 | | 216.4503 | | | | 1.245081 | | | | 4955.555 | | | 7.078125 | |  | |  | |  |
| 17.74167 | | 216.54 | | | | 1.245652 | | | | 4955.492 | | | 7.046875 | |  | |  | |  |
| 17.75 | | 216.6288 | | | | 1.24734 | | | | 4955.438 | | | 6.953125 | |  | |  | |  |
| 17.75833 | | 216.7184 | | | | 1.248708 | | | | 4955.391 | | | 7.007813 | |  | |  | |  |
| 17.76667 | | 216.808 | | | | 1.248239 | | | | 4955.313 | | | 7.101563 | |  | |  | |  |
| 17.775 | | 216.8976 | | | | 1.248633 | | | | 4955.266 | | | 7.070313 | |  | |  | |  |
| 17.78333 | | 216.9864 | | | | 1.249692 | | | | 4955.227 | | | 7.101563 | |  | |  | |  |
| 17.79167 | | 217.0736 | | | | 1.249481 | | | | 4955.172 | | | 7.195313 | |  | |  | |  |
| 17.8 | | 217.1615 | | | | 1.24994 | | | | 4955.125 | | | 7.195313 | |  | |  | |  |
| 17.80833 | | 217.2528 | | | | 1.250182 | | | | 4955.063 | | | 7.3125 | |  | |  | |  |
| 17.81667 | | 217.3432 | | | | 1.249955 | | | | 4954.992 | | | 7.1875 | |  | |  | |  |
| 17.825 | | 217.4304 | | | | 1.251286 | | | | 4954.938 | | | 7.09375 | |  | |  | |  |
| 17.83333 | | 217.5192 | | | | 1.25274 | | | | 4954.883 | | | 7.1875 | |  | |  | |  |
| 17.84167 | | 217.6104 | | | | 1.253123 | | | | 4954.813 | | | 7.195313 | |  | |  | |  |
| 17.85 | | 217.6984 | | | | 1.254316 | | | | 4954.75 | | | 7.046875 | |  | |  | |  |
| 17.85833 | | 217.788 | | | | 1.255707 | | | | 4954.672 | | | 7.21875 | |  | |  | |  |
| 17.86667 | | 217.8768 | | | | 1.254974 | | | | 4954.633 | | | 7.171875 | |  | |  | |  |
| 17.875 | | 217.9656 | | | | 1.254836 | | | | 4954.586 | | | 6.960938 | |  | |  | |  |
| 17.88333 | | 218.0552 | | | | 1.255408 | | | | 4954.508 | | | 6.65625 | |  | |  | |  |
| 17.89167 | | 218.1465 | | | | 1.255409 | | | | 4954.453 | | | 6.492188 | |  | |  | |  |
| 17.9 | | 218.2336 | | | | 1.256027 | | | | 4954.414 | | | 6.765625 | |  | |  | |  |
| 17.90833 | | 218.3266 | | | | 1.258008 | | | | 4954.328 | | | 6.890625 | |  | |  | |  |
| 17.91667 | | 218.4178 | | | | 1.258755 | | | | 4954.273 | | | 6.71875 | |  | |  | |  |
| 17.925 | | 218.5074 | | | | 1.259003 | | | | 4954.234 | | | 6.71875 | |  | |  | |  |
| 17.93333 | | 218.6003 | | | | 1.261675 | | | | 4954.219 | | | 6.671875 | |  | |  | |  |
| 17.94167 | | 218.6891 | | | | 1.262779 | | | | 4954.188 | | | 6.398438 | |  | |  | |  |
| 17.95 | | 218.7787 | | | | 1.262923 | | | | 4954.086 | | | 6.445313 | |  | |  | |  |
| 17.95833 | | 218.8708 | | | | 1.264318 | | | | 4954.008 | | | 6.804688 | |  | |  | |  |
| 17.96667 | | 218.9629 | | | | 1.26482 | | | | 4953.984 | | | 6.890625 | |  | |  | |  |
| 17.975 | | 219.0493 | | | | 1.263982 | | | | 4953.914 | | | 7.109375 | |  | |  | |  |
| 17.98333 | | 219.1397 | | | | 1.265885 | | | | 4953.867 | | | 7.039063 | |  | |  | |  |
| 17.99167 | | 219.2301 | | | | 1.266226 | | | | 4953.852 | | | 7.09375 | |  | |  | |  |
| 18 | | 219.3189 | | | | 1.265771 | | | | 4953.789 | | | 7.289063 | |  | |  | |  |
| 18.00833 | | 219.4094 | | | | 1.268116 | | | | 4953.68 | | | 7.234375 | |  | |  | |  |
| 18.01667 | | 219.499 | | | | 1.26921 | | | | 4953.609 | | | 7.59375 | |  | |  | |  |
| 18.025 | | 219.5886 | | | | 1.268198 | | | | 4953.523 | | | 7.640625 | |  | |  | |  |
| 18.03333 | | 219.6823 | | | | 1.269996 | | | | 4953.477 | | | 7.695313 | |  | |  | |  |
| 18.04167 | | 219.7728 | | | | 1.271239 | | | | 4953.406 | | | 7.445313 | |  | |  | |  |
| 18.05 | | 219.8591 | | | | 1.271364 | | | | 4953.32 | | | 7.554688 | |  | |  | |  |
| 18.05833 | | 219.9503 | | | | 1.272301 | | | | 4953.25 | | | 7.601563 | |  | |  | |  |
| 18.06667 | | 220.04 | | | | 1.273541 | | | | 4953.156 | | | 7.6875 | |  | |  | |  |
| 18.075 | | 220.1279 | | | | 1.273957 | | | | 4953.078 | | | 7.742188 | |  | |  | |  |
| 18.08333 | | 220.2184 | | | | 1.27526 | | | | 4953.016 | | | 8.007813 | |  | |  | |  |
| 18.09167 | | 220.3064 | | | | 1.277316 | | | | 4952.977 | | | 8.359375 | |  | |  | |  |
| 18.1 | | 220.3935 | | | | 1.277811 | | | | 4952.914 | | | 8.507813 | |  | |  | |  |
| 18.10833 | | 220.4823 | | | | 1.279271 | | | | 4952.844 | | | 8.601563 | |  | |  | |  |
| 18.11667 | | 220.5662 | | | | 1.281083 | | | | 4952.758 | | | 8.859375 | |  | |  | |  |
| 18.125 | | 220.6533 | | | | 1.280843 | | | | 4952.688 | | | 8.71875 | |  | |  | |  |
| 18.13333 | | 220.7429 | | | | 1.281755 | | | | 4952.586 | | | 8.929688 | |  | |  | |  |
| 18.14167 | | 220.8334 | | | | 1.28321 | | | | 4952.469 | | | 8.84375 | |  | |  | |  |
| 18.15 | | 220.9255 | | | | 1.283127 | | | | 4952.383 | | | 8.796875 | |  | |  | |  |
| 18.15833 | | 221.0143 | | | | 1.283716 | | | | 4952.289 | | | 8.742188 | |  | |  | |  |
| 18.16667 | | 221.1039 | | | | 1.28448 | | | | 4952.188 | | | 8.875 | |  | |  | |  |
| 18.175 | | 221.1927 | | | | 1.283635 | | | | 4952.125 | | | 8.53125 | |  | |  | |  |
| 18.18333 | | 221.2847 | | | | 1.284311 | | | | 4952.031 | | | 8.382813 | |  | |  | |  |
| 18.19167 | | 221.3744 | | | | 1.286012 | | | | 4951.969 | | | 8.476563 | |  | |  | |  |
| 18.2 | | 221.4656 | | | | 1.28626 | | | | 4951.906 | | | 8.367188 | |  | |  | |  |
| 18.20833 | | 221.5552 | | | | 1.286979 | | | | 4951.844 | | | 8.476563 | |  | |  | |  |
| 18.21667 | | 221.649 | | | | 1.288919 | | | | 4951.758 | | | 8.375 | |  | |  | |  |
| 18.225 | | 221.7369 | | | | 1.289137 | | | | 4951.734 | | | 8.492188 | |  | |  | |  |
| 18.23333 | | 221.8257 | | | | 1.289218 | | | | 4951.68 | | | 8.546875 | |  | |  | |  |
| 18.24167 | | 221.9162 | | | | 1.291139 | | | | 4951.609 | | | 8.53125 | |  | |  | |  |
| 18.25 | | 222.0091 | | | | 1.29216 | | | | 4951.555 | | | 8.070313 | |  | |  | |  |
| 18.25833 | | 222.102 | | | | 1.292573 | | | | 4951.469 | | | 7.828125 | |  | |  | |  |
| 18.26667 | | 222.1933 | | | | 1.294598 | | | | 4951.406 | | | 7.929688 | |  | |  | |  |
| 18.275 | | 222.2837 | | | | 1.29663 | | | | 4951.328 | | | 7.75 | |  | |  | |  |
| 18.28333 | | 222.375 | | | | 1.295931 | | | | 4951.258 | | | 7.671875 | |  | |  | |  |
| 18.29167 | | 222.4687 | | | | 1.297539 | | | | 4951.195 | | | 7.695313 | |  | |  | |  |
| 18.3 | | 222.5583 | | | | 1.300184 | | | | 4951.188 | | | 7.710938 | |  | |  | |  |
| 18.30833 | | 222.652 | | | | 1.30024 | | | | 4951.148 | | | 7.648438 | |  | |  | |  |
| 18.31667 | | 222.7425 | | | | 1.302031 | | | | 4951.078 | | | 7.398438 | |  | |  | |  |
| 18.325 | | 222.8345 | | | | 1.304723 | | | | 4951.039 | | | 7.515625 | |  | |  | |  |
| 18.33333 | | 222.9233 | | | | 1.304741 | | | | 4950.984 | | | 7.570313 | |  | |  | |  |
| 18.34167 | | 223.0097 | | | | 1.305053 | | | | 4950.914 | | | 7.5625 | |  | |  | |  |
| 18.35 | | 223.0976 | | | | 1.306521 | | | | 4950.859 | | | 7.453125 | |  | |  | |  |
| 18.35833 | | 223.1889 | | | | 1.305503 | | | | 4950.797 | | | 7.578125 | |  | |  | |  |
| 18.36667 | | 223.281 | | | | 1.305111 | | | | 4950.766 | | | 7.695313 | |  | |  | |  |
| 18.375 | | 223.369 | | | | 1.306597 | | | | 4950.695 | | | 7.671875 | |  | |  | |  |
| 18.38333 | | 223.4561 | | | | 1.306052 | | | | 4950.625 | | | 7.679688 | |  | |  | |  |
| 18.39167 | | 223.5441 | | | | 1.306292 | | | | 4950.563 | | | 7.851563 | |  | |  | |  |
| 18.4 | | 223.6329 | | | | 1.308838 | | | | 4950.508 | | | 7.90625 | |  | |  | |  |
| 18.40833 | | 223.725 | | | | 1.308867 | | | | 4950.422 | | | 8.0625 | |  | |  | |  |
| 18.41667 | | 223.8154 | | | | 1.309299 | | | | 4950.336 | | | 8.1875 | |  | |  | |  |
| 18.425 | | 223.9091 | | | | 1.312114 | | | | 4950.273 | | | 8.453125 | |  | |  | |  |
| 18.43333 | | 224.0029 | | | | 1.312827 | | | | 4950.211 | | | 8.617188 | |  | |  | |  |
| 18.44167 | | 224.0982 | | | | 1.31319 | | | | 4950.125 | | | 8.5625 | |  | |  | |  |
| 18.45 | | 224.187 | | | | 1.314875 | | | | 4950.047 | | | 8.648438 | |  | |  | |  |
| 18.45833 | | 224.2799 | | | | 1.315722 | | | | 4949.961 | | | 8.71875 | |  | |  | |  |
| 18.46667 | | 224.3728 | | | | 1.316094 | | | | 4949.867 | | | 8.695313 | |  | |  | |  |
| 18.475 | | 224.4633 | | | | 1.317376 | | | | 4949.758 | | | 8.929688 | |  | |  | |  |
| 18.48333 | | 224.557 | | | | 1.319098 | | | | 4949.664 | | | 9.171875 | |  | |  | |  |
| 18.49167 | | 224.6474 | | | | 1.319233 | | | | 4949.602 | | | 9.140625 | |  | |  | |  |
| 18.5 | | 224.7395 | | | | 1.320496 | | | | 4949.516 | | | 9.179688 | |  | |  | |  |
| 18.50833 | | 224.8291 | | | | 1.322161 | | | | 4949.43 | | | 9.09375 | |  | |  | |  |
| 18.51667 | | 224.9204 | | | | 1.321961 | | | | 4949.344 | | | 9.320313 | |  | |  | |  |
| 18.525 | | 225.0076 | | | | 1.322919 | | | | 4949.234 | | | 9.5 | |  | |  | |  |
| 18.53333 | | 225.0988 | | | | 1.325021 | | | | 4949.133 | | | 9.734375 | |  | |  | |  |
| 18.54167 | | 225.1884 | | | | 1.325097 | | | | 4949.063 | | | 9.773438 | |  | |  | |  |
| 18.55 | | 225.2789 | | | | 1.32528 | | | | 4948.984 | | | 9.78125 | |  | |  | |  |
| 18.55833 | | 225.371 | | | | 1.327888 | | | | 4948.914 | | | 9.65625 | |  | |  | |  |
| 18.56667 | | 225.4589 | | | | 1.328643 | | | | 4948.813 | | | 9.6875 | |  | |  | |  |
| 18.575 | | 225.5477 | | | | 1.329133 | | | | 4948.703 | | | 9.78125 | |  | |  | |  |
| 18.58333 | | 225.6357 | | | | 1.331925 | | | | 4948.602 | | | 9.59375 | |  | |  | |  |
| 18.59167 | | 225.7245 | | | | 1.333156 | | | | 4948.516 | | | 9.625 | |  | |  | |  |
| 18.6 | | 225.8116 | | | | 1.333294 | | | | 4948.43 | | | 9.382813 | |  | |  | |  |
| 18.60833 | | 225.9021 | | | | 1.336267 | | | | 4948.367 | | | 9.171875 | |  | |  | |  |
| 18.61667 | | 225.9933 | | | | 1.336619 | | | | 4948.289 | | | 9.03125 | |  | |  | |  |
| 18.625 | | 226.0863 | | | | 1.335668 | | | | 4948.195 | | | 8.984375 | |  | |  | |  |
| 18.63333 | | 226.1775 | | | | 1.337636 | | | | 4948.141 | | | 8.921875 | |  | |  | |  |
| 18.64167 | | 226.2688 | | | | 1.337888 | | | | 4948.063 | | | 8.804688 | |  | |  | |  |
| 18.65 | | 226.3584 | | | | 1.336458 | | | | 4948.023 | | | 9.007813 | |  | |  | |  |
| 18.65833 | | 226.448 | | | | 1.338408 | | | | 4947.977 | | | 9.109375 | |  | |  | |  |
| 18.66667 | | 226.5393 | | | | 1.339198 | | | | 4947.93 | | | 9.539063 | |  | |  | |  |
| 18.675 | | 226.6305 | | | | 1.338447 | | | | 4947.867 | | | 9.625 | |  | |  | |  |
| 18.68333 | | 226.7226 | | | | 1.34098 | | | | 4947.797 | | | 9.648438 | |  | |  | |  |
| 18.69167 | | 226.8122 | | | | 1.343174 | | | | 4947.742 | | | 9.820313 | |  | |  | |  |
| 18.7 | | 226.9019 | | | | 1.343394 | | | | 4947.641 | | | 9.914063 | |  | |  | |  |
| 18.70833 | | 226.989 | | | | 1.345557 | | | | 4947.547 | | | 9.945313 | |  | |  | |  |
| 18.71667 | | 227.077 | | | | 1.347581 | | | | 4947.422 | | | 10.02344 | |  | |  | |  |
| 18.725 | | 227.1666 | | | | 1.347406 | | | | 4947.328 | | | 10.17969 | |  | |  | |  |
| 18.73333 | | 227.262 | | | | 1.349179 | | | | 4947.242 | | | 10.08594 | |  | |  | |  |
| 18.74167 | | 227.3557 | | | | 1.350998 | | | | 4947.133 | | | 10.04688 | |  | |  | |  |
| 18.75 | | 227.4437 | | | | 1.351147 | | | | 4947.047 | | | 10.08594 | |  | |  | |  |
| 18.75833 | | 227.5366 | | | | 1.353003 | | | | 4946.953 | | | 9.976563 | |  | |  | |  |
| 18.76667 | | 227.6278 | | | | 1.354557 | | | | 4946.859 | | | 10.04688 | |  | |  | |  |
| 18.775 | | 227.7183 | | | | 1.353653 | | | | 4946.75 | | | 9.820313 | |  | |  | |  |
| 18.78333 | | 227.8095 | | | | 1.354844 | | | | 4946.688 | | | 9.914063 | |  | |  | |  |
| 18.79167 | | 227.9016 | | | | 1.35694 | | | | 4946.609 | | | 10.03906 | |  | |  | |  |
| 18.8 | | 227.9945 | | | | 1.357626 | | | | 4946.523 | | | 9.765625 | |  | |  | |  |
| 18.80833 | | 228.0866 | | | | 1.358792 | | | | 4946.461 | | | 9.890625 | |  | |  | |  |
| 18.81667 | | 228.1762 | | | | 1.360906 | | | | 4946.359 | | | 9.882813 | |  | |  | |  |
| 18.825 | | 228.2658 | | | | 1.361624 | | | | 4946.305 | | | 9.835938 | |  | |  | |  |
| 18.83333 | | 228.3579 | | | | 1.362491 | | | | 4946.211 | | | 9.84375 | |  | |  | |  |
| 18.84167 | | 228.45 | | | | 1.364611 | | | | 4946.117 | | | 9.898438 | |  | |  | |  |
| 18.85 | | 228.5405 | | | | 1.365595 | | | | 4946.07 | | | 9.945313 | |  | |  | |  |
| 18.85833 | | 228.6334 | | | | 1.365221 | | | | 4945.961 | | | 10.1875 | |  | |  | |  |
| 18.86667 | | 228.7254 | | | | 1.367403 | | | | 4945.883 | | | 10.25781 | |  | |  | |  |
| 18.875 | | 228.8167 | | | | 1.369255 | | | | 4945.805 | | | 10.16406 | |  | |  | |  |
| 18.88333 | | 228.9055 | | | | 1.368476 | | | | 4945.719 | | | 10.40625 | |  | |  | |  |
| 18.89167 | | 228.9926 | | | | 1.370552 | | | | 4945.633 | | | 10.21875 | |  | |  | |  |
| 18.9 | | 229.0839 | | | | 1.373169 | | | | 4945.539 | | | 10.25781 | |  | |  | |  |
| 18.90833 | | 229.176 | | | | 1.373268 | | | | 4945.422 | | | 10.34375 | |  | |  | |  |
| 18.91667 | | 229.2697 | | | | 1.37488 | | | | 4945.32 | | | 10.3125 | |  | |  | |  |
| 18.925 | | 229.3602 | | | | 1.377123 | | | | 4945.25 | | | 10.5625 | |  | |  | |  |
| 18.93333 | | 229.4506 | | | | 1.376913 | | | | 4945.125 | | | 10.51563 | |  | |  | |  |
| 18.94167 | | 229.541 | | | | 1.378417 | | | | 4945.07 | | | 10.60156 | |  | |  | |  |
| 18.95 | | 229.6339 | | | | 1.380837 | | | | 4944.977 | | | 10.57813 | |  | |  | |  |
| 18.95833 | | 229.7252 | | | | 1.380594 | | | | 4944.891 | | | 10.49219 | |  | |  | |  |
| 18.96667 | | 229.8181 | | | | 1.38269 | | | | 4944.797 | | | 10.53125 | |  | |  | |  |
| 18.975 | | 229.9118 | | | | 1.385782 | | | | 4944.688 | | | 10.76563 | |  | |  | |  |
| 18.98333 | | 229.9998 | | | | 1.385546 | | | | 4944.602 | | | 10.67188 | |  | |  | |  |
| 18.99167 | | 230.0903 | | | | 1.386584 | | | | 4944.508 | | | 10.71094 | |  | |  | |  |
| 19 | | 230.1833 | | | | 1.388787 | | | | 4944.422 | | | 10.60938 | |  | |  | |  |
| 19.00833 | | 230.2723 | | | | 1.389027 | | | | 4944.344 | | | 10.66406 | |  | |  | |  |
| 19.01667 | | 230.3652 | | | | 1.388927 | | | | 4944.258 | | | 10.92188 | |  | |  | |  |
| 19.025 | | 230.455 | | | | 1.38979 | | | | 4944.148 | | | 10.8125 | |  | |  | |  |
| 19.03333 | | 230.5416 | | | | 1.391461 | | | | 4944.07 | | | 10.85938 | |  | |  | |  |
| 19.04167 | | 230.6282 | | | | 1.391607 | | | | 4943.977 | | | 10.98438 | |  | |  | |  |
| 19.05 | | 230.7163 | | | | 1.393364 | | | | 4943.898 | | | 10.99219 | |  | |  | |  |
| 19.05833 | | 230.8061 | | | | 1.395787 | | | | 4943.805 | | | 11.08594 | |  | |  | |  |
| 19.06667 | | 230.8951 | | | | 1.396675 | | | | 4943.68 | | | 11.16406 | |  | |  | |  |
| 19.075 | | 230.9841 | | | | 1.398586 | | | | 4943.594 | | | 11.11719 | |  | |  | |  |
| 19.08333 | | 231.0682 | | | | 1.400429 | | | | 4943.5 | | | 11.30469 | |  | |  | |  |
| 19.09167 | | 231.1556 | | | | 1.400527 | | | | 4943.391 | | | 11.34375 | |  | |  | |  |
| 19.1 | | 231.239 | | | | 1.402062 | | | | 4943.297 | | | 11.13281 | |  | |  | |  |
| 19.10833 | | 231.3271 | | | | 1.403264 | | | | 4943.188 | | | 11.24219 | |  | |  | |  |
| 19.11667 | | 231.4145 | | | | 1.403238 | | | | 4943.086 | | | 11.21094 | |  | |  | |  |
| 19.125 | | 231.5051 | | | | 1.40485 | | | | 4943 | | | 11.21094 | |  | |  | |  |
| 19.13333 | | 231.5933 | | | | 1.406294 | | | | 4942.883 | | | 11.38281 | |  | |  | |  |
| 19.14167 | | 231.6839 | | | | 1.406254 | | | | 4942.789 | | | 11.38281 | |  | |  | |  |
| 19.15 | | 231.7728 | | | | 1.407648 | | | | 4942.711 | | | 11.60156 | |  | |  | |  |
| 19.15833 | | 231.861 | | | | 1.409823 | | | | 4942.617 | | | 11.83594 | |  | |  | |  |
| 19.16667 | | 231.9492 | | | | 1.410428 | | | | 4942.523 | | | 11.92969 | |  | |  | |  |
| 19.175 | | 232.039 | | | | 1.410979 | | | | 4942.438 | | | 11.97656 | |  | |  | |  |
| 19.18333 | | 232.1327 | | | | 1.413518 | | | | 4942.313 | | | 12.05469 | |  | |  | |  |
| 19.19167 | | 232.2217 | | | | 1.41384 | | | | 4942.227 | | | 12.28125 | |  | |  | |  |
| 19.2 | | 232.3131 | | | | 1.413588 | | | | 4942.094 | | | 12.51563 | |  | |  | |  |
| 19.20833 | | 232.4021 | | | | 1.416068 | | | | 4941.969 | | | 12.76563 | |  | |  | |  |
| 19.21667 | | 232.4942 | | | | 1.417155 | | | | 4941.859 | | | 12.78906 | |  | |  | |  |
| 19.225 | | 232.5808 | | | | 1.41704 | | | | 4941.75 | | | 12.99219 | |  | |  | |  |
| 19.23333 | | 232.6722 | | | | 1.419669 | | | | 4941.633 | | | 13.125 | |  | |  | |  |
| 19.24167 | | 232.7612 | | | | 1.420684 | | | | 4941.508 | | | 13.46094 | |  | |  | |  |
| 19.25 | | 232.855 | | | | 1.420567 | | | | 4941.375 | | | 13.44531 | |  | |  | |  |
| 19.25833 | | 232.9464 | | | | 1.422703 | | | | 4941.234 | | | 13.8125 | |  | |  | |  |
| 19.26667 | | 233.0361 | | | | 1.424824 | | | | 4941.117 | | | 13.78125 | |  | |  | |  |
| 19.275 | | 233.1259 | | | | 1.424941 | | | | 4940.984 | | | 13.75781 | |  | |  | |  |
| 19.28333 | | 233.2149 | | | | 1.42665 | | | | 4940.859 | | | 13.97656 | |  | |  | |  |
| 19.29167 | | 233.3071 | | | | 1.42843 | | | | 4940.703 | | | 13.84375 | |  | |  | |  |
| 19.3 | | 233.3952 | | | | 1.428281 | | | | 4940.594 | | | 13.95313 | |  | |  | |  |
| 19.30833 | | 233.4842 | | | | 1.429368 | | | | 4940.438 | | | 13.80469 | |  | |  | |  |
| 19.31667 | | 233.5692 | | | | 1.431384 | | | | 4940.32 | | | 13.78906 | |  | |  | |  |
| 19.325 | | 233.6622 | | | | 1.43245 | | | | 4940.211 | | | 13.75781 | |  | |  | |  |
| 19.33333 | | 233.7503 | | | | 1.433787 | | | | 4940.078 | | | 13.85938 | |  | |  | |  |
| 19.34167 | | 233.8393 | | | | 1.435768 | | | | 4939.969 | | | 13.89844 | |  | |  | |  |
| 19.35 | | 233.9275 | | | | 1.435762 | | | | 4939.844 | | | 14.11719 | |  | |  | |  |
| 19.35833 | | 234.0181 | | | | 1.437127 | | | | 4939.766 | | | 14.45313 | |  | |  | |  |
| 19.36667 | | 234.1094 | | | | 1.439279 | | | | 4939.648 | | | 14.39844 | |  | |  | |  |
| 19.375 | | 234.196 | | | | 1.439243 | | | | 4939.539 | | | 14.49219 | |  | |  | |  |
| 19.38333 | | 234.2874 | | | | 1.440492 | | | | 4939.406 | | | 14.4375 | |  | |  | |  |
| 19.39167 | | 234.3788 | | | | 1.442874 | | | | 4939.289 | | | 14.75781 | |  | |  | |  |
| 19.4 | | 234.4734 | | | | 1.444221 | | | | 4939.148 | | | 14.71875 | |  | |  | |  |
| 19.40833 | | 234.5599 | | | | 1.444287 | | | | 4938.992 | | | 14.69531 | |  | |  | |  |
| 19.41667 | | 234.6489 | | | | 1.44723 | | | | 4938.875 | | | 14.85156 | |  | |  | |  |
| 19.425 | | 234.7379 | | | | 1.449598 | | | | 4938.742 | | | 14.8125 | |  | |  | |  |
| 19.43333 | | 234.8244 | | | | 1.450415 | | | | 4938.633 | | | 14.72656 | |  | |  | |  |
| 19.44167 | | 234.9134 | | | | 1.453411 | | | | 4938.469 | | | 14.8125 | |  | |  | |  |
| 19.45 | | 235.0016 | | | | 1.455841 | | | | 4938.359 | | | 14.9375 | |  | |  | |  |
| 19.45833 | | 235.093 | | | | 1.456843 | | | | 4938.227 | | | 14.98438 | |  | |  | |  |
| 19.46667 | | 235.1804 | | | | 1.459408 | | | | 4938.086 | | | 15.19531 | |  | |  | |  |
| 19.475 | | 235.2701 | | | | 1.46135 | | | | 4937.969 | | | 15.32813 | |  | |  | |  |
| 19.48333 | | 235.3591 | | | | 1.4618 | | | | 4937.867 | | | 15.375 | |  | |  | |  |
| 19.49167 | | 235.4489 | | | | 1.463752 | | | | 4937.734 | | | 15.42969 | |  | |  | |  |
| 19.5 | | 235.5371 | | | | 1.465387 | | | | 4937.602 | | | 15.4375 | |  | |  | |  |
| 19.50833 | | 235.6268 | | | | 1.465305 | | | | 4937.469 | | | 15.49219 | |  | |  | |  |
| 19.51667 | | 235.7198 | | | | 1.466595 | | | | 4937.313 | | | 15.79688 | |  | |  | |  |
| 19.525 | | 235.808 | | | | 1.468376 | | | | 4937.172 | | | 15.83594 | |  | |  | |  |
| 19.53333 | | 235.897 | | | | 1.467728 | | | | 4937.039 | | | 15.86719 | |  | |  | |  |
| 19.54167 | | 235.9843 | | | | 1.469303 | | | | 4936.914 | | | 16.02344 | |  | |  | |  |
| 19.55 | | 236.0733 | | | | 1.471577 | | | | 4936.781 | | | 16.16406 | |  | |  | |  |
| 19.55833 | | 236.1639 | | | | 1.471979 | | | | 4936.641 | | | 16.20313 | |  | |  | |  |
| 19.56667 | | 236.2521 | | | | 1.473429 | | | | 4936.469 | | | 16.32813 | |  | |  | |  |
| 19.575 | | 236.3402 | | | | 1.476804 | | | | 4936.336 | | | 16.47656 | |  | |  | |  |
| 19.58333 | | 236.43 | | | | 1.477656 | | | | 4936.203 | | | 16.40625 | |  | |  | |  |
| 19.59167 | | 236.5198 | | | | 1.479374 | | | | 4936.047 | | | 16.59375 | |  | |  | |  |
| 19.6 | | 236.608 | | | | 1.482565 | | | | 4935.891 | | | 16.61719 | |  | |  | |  |
| 19.60833 | | 236.6994 | | | | 1.483451 | | | | 4935.75 | | | 16.77344 | |  | |  | |  |
| 19.61667 | | 236.7883 | | | | 1.484727 | | | | 4935.594 | | | 16.92969 | |  | |  | |  |
| 19.625 | | 236.8805 | | | | 1.487237 | | | | 4935.438 | | | 16.96094 | |  | |  | |  |
| 19.63333 | | 236.9711 | | | | 1.488141 | | | | 4935.305 | | | 16.89063 | |  | |  | |  |
| 19.64167 | | 237.0569 | | | | 1.489026 | | | | 4935.141 | | | 17.03906 | |  | |  | |  |
| 19.65 | | 237.1474 | | | | 1.491587 | | | | 4935.008 | | | 17.19531 | |  | |  | |  |
| 19.65833 | | 237.2388 | | | | 1.49299 | | | | 4934.844 | | | 17.46094 | |  | |  | |  |
| 19.66667 | | 237.3334 | | | | 1.493781 | | | | 4934.688 | | | 17.59375 | |  | |  | |  |
| 19.675 | | 237.4224 | | | | 1.495638 | | | | 4934.539 | | | 17.58594 | |  | |  | |  |
| 19.68333 | | 237.513 | | | | 1.497865 | | | | 4934.406 | | | 17.73438 | |  | |  | |  |
| 19.69167 | | 237.5995 | | | | 1.49831 | | | | 4934.242 | | | 17.70313 | |  | |  | |  |
| 19.7 | | 237.6893 | | | | 1.500029 | | | | 4934.094 | | | 17.91406 | |  | |  | |  |
| 19.70833 | | 237.7775 | | | | 1.502249 | | | | 4933.914 | | | 17.84375 | |  | |  | |  |
| 19.71667 | | 237.8673 | | | | 1.503006 | | | | 4933.758 | | | 18.20313 | |  | |  | |  |
| 19.725 | | 237.961 | | | | 1.504632 | | | | 4933.602 | | | 18 | |  | |  | |  |
| 19.73333 | | 238.0516 | | | | 1.507097 | | | | 4933.445 | | | 18.07813 | |  | |  | |  |
| 19.74167 | | 238.139 | | | | 1.507218 | | | | 4933.305 | | | 18.26563 | |  | |  | |  |
| 19.75 | | 238.2232 | | | | 1.508407 | | | | 4933.133 | | | 18.67969 | |  | |  | |  |
| 19.75833 | | 238.3129 | | | | 1.511324 | | | | 4932.977 | | | 18.64063 | |  | |  | |  |
| 19.76667 | | 238.4019 | | | | 1.512481 | | | | 4932.781 | | | 18.50781 | |  | |  | |  |
| 19.775 | | 238.4949 | | | | 1.514201 | | | | 4932.664 | | | 18.6875 | |  | |  | |  |
| 19.78333 | | 238.5863 | | | | 1.517594 | | | | 4932.508 | | | 18.65625 | |  | |  | |  |
| 19.79167 | | 238.6769 | | | | 1.518492 | | | | 4932.328 | | | 18.79688 | |  | |  | |  |
| 19.8 | | 238.7682 | | | | 1.519627 | | | | 4932.117 | | | 18.91406 | |  | |  | |  |
| 19.80833 | | 238.8612 | | | | 1.523077 | | | | 4931.977 | | | 19.32031 | |  | |  | |  |
| 19.81667 | | 238.951 | | | | 1.524546 | | | | 4931.828 | | | 19.39063 | |  | |  | |  |
| 19.825 | | 239.044 | | | | 1.52584 | | | | 4931.664 | | | 19.07813 | |  | |  | |  |
| 19.83333 | | 239.133 | | | | 1.529692 | | | | 4931.516 | | | 18.8125 | |  | |  | |  |
| 19.84167 | | 239.2227 | | | | 1.531401 | | | | 4931.336 | | | 19.03125 | |  | |  | |  |
| 19.85 | | 239.3149 | | | | 1.531647 | | | | 4931.172 | | | 18.94531 | |  | |  | |  |
| 19.85833 | | 239.4031 | | | | 1.533621 | | | | 4930.953 | | | 19.14844 | |  | |  | |  |
| 19.86667 | | 239.4945 | | | | 1.535838 | | | | 4930.789 | | | 19.17969 | |  | |  | |  |
| 19.875 | | 239.5842 | | | | 1.537056 | | | | 4930.672 | | | 19.125 | |  | |  | |  |
| 19.88333 | | 239.6748 | | | | 1.539114 | | | | 4930.555 | | | 19.11719 | |  | |  | |  |
| 19.89167 | | 239.7598 | | | | 1.541686 | | | | 4930.375 | | | 18.97656 | |  | |  | |  |
| 19.9 | | 239.8504 | | | | 1.543188 | | | | 4930.219 | | | 19.10156 | |  | |  | |  |
| 19.90833 | | 239.9386 | | | | 1.545361 | | | | 4930.047 | | | 19.23438 | |  | |  | |  |
| 19.91667 | | 240.0291 | | | | 1.547972 | | | | 4929.875 | | | 19.45313 | |  | |  | |  |
| 19.925 | | 240.1197 | | | | 1.549133 | | | | 4929.719 | | | 19.5 | |  | |  | |  |
| 19.93333 | | 240.2055 | | | | 1.550822 | | | | 4929.57 | | | 19.57031 | |  | |  | |  |
| 19.94167 | | 240.2953 | | | | 1.5538 | | | | 4929.43 | | | 19.82813 | |  | |  | |  |
| 19.95 | | 240.385 | | | | 1.554765 | | | | 4929.25 | | | 20.14063 | |  | |  | |  |
| 19.95833 | | 240.474 | | | | 1.556376 | | | | 4929.078 | | | 20.1875 | |  | |  | |  |
| 19.96667 | | 240.5622 | | | | 1.558906 | | | | 4928.883 | | | 20.35156 | |  | |  | |  |
| 19.975 | | 240.6536 | | | | 1.559745 | | | | 4928.719 | | | 20.4375 | |  | |  | |  |
| 19.98333 | | 240.7433 | | | | 1.56124 | | | | 4928.539 | | | 20.53906 | |  | |  | |  |
| 19.99167 | | 240.8339 | | | | 1.56365 | | | | 4928.344 | | | 20.61719 | |  | |  | |  |
| 20 | | 240.9253 | | | | 1.565087 | | | | 4928.141 | | | 20.8125 | |  | |  | |  |
| 20.00833 | | 241.0135 | | | | 1.566075 | | | | 4927.969 | | | 20.90625 | |  | |  | |  |
| 20.01667 | | 241.1024 | | | | 1.569376 | | | | 4927.766 | | | 21.00781 | |  | |  | |  |
| 20.025 | | 241.1922 | | | | 1.571678 | | | | 4927.594 | | | 21.07031 | |  | |  | |  |
| 20.03333 | | 241.2804 | | | | 1.572642 | | | | 4927.414 | | | 20.92969 | |  | |  | |  |
| 20.04167 | | 241.3718 | | | | 1.575569 | | | | 4927.219 | | | 21.15625 | |  | |  | |  |
| 20.05 | | 241.4608 | | | | 1.577978 | | | | 4927.031 | | | 21.07813 | |  | |  | |  |
| 20.05833 | | 241.5489 | | | | 1.57882 | | | | 4926.844 | | | 21.30469 | |  | |  | |  |
| 20.06667 | | 241.6395 | | | | 1.581723 | | | | 4926.648 | | | 21.48438 | |  | |  | |  |
| 20.075 | | 241.7309 | | | | 1.583918 | | | | 4926.469 | | | 21.66406 | |  | |  | |  |
| 20.08333 | | 241.8231 | | | | 1.584532 | | | | 4926.313 | | | 21.57031 | |  | |  | |  |
| 20.09167 | | 241.916 | | | | 1.587507 | | | | 4926.117 | | | 21.66406 | |  | |  | |  |
| 20.1 | | 242.009 | | | | 1.590108 | | | | 4925.945 | | | 21.88281 | |  | |  | |  |
| 20.10833 | | 242.1004 | | | | 1.589795 | | | | 4925.734 | | | 22.03125 | |  | |  | |  |
| 20.11667 | | 242.191 | | | | 1.592079 | | | | 4925.539 | | | 22.17188 | |  | |  | |  |
| 20.125 | | 242.2784 | | | | 1.595429 | | | | 4925.336 | | | 22.36719 | |  | |  | |  |
| 20.13333 | | 242.3705 | | | | 1.596315 | | | | 4925.18 | | | 22.51563 | |  | |  | |  |
| 20.14167 | | 242.4635 | | | | 1.599034 | | | | 4924.969 | | | 22.58594 | |  | |  | |  |
| 20.15 | | 242.5541 | | | | 1.602889 | | | | 4924.766 | | | 22.67969 | |  | |  | |  |
| 20.15833 | | 242.6447 | | | | 1.605215 | | | | 4924.563 | | | 22.65625 | |  | |  | |  |
| 20.16667 | | 242.7345 | | | | 1.607101 | | | | 4924.367 | | | 22.94531 | |  | |  | |  |
| 20.175 | | 242.8242 | | | | 1.610323 | | | | 4924.148 | | | 23.14844 | |  | |  | |  |
| 20.18333 | | 242.9172 | | | | 1.612262 | | | | 4923.953 | | | 23.3125 | |  | |  | |  |
| 20.19167 | | 243.0086 | | | | 1.613315 | | | | 4923.75 | | | 23.40625 | |  | |  | |  |
| 20.2 | | 243.1024 | | | | 1.616187 | | | | 4923.547 | | | 23.89063 | |  | |  | |  |
| 20.20833 | | 243.1962 | | | | 1.618499 | | | | 4923.359 | | | 23.98438 | |  | |  | |  |
| 20.21667 | | 243.2884 | | | | 1.620107 | | | | 4923.133 | | | 23.99219 | |  | |  | |  |
| 20.225 | | 243.3773 | | | | 1.623025 | | | | 4922.922 | | | 24.14063 | |  | |  | |  |
| 20.23333 | | 243.4671 | | | | 1.625631 | | | | 4922.711 | | | 24.21875 | |  | |  | |  |
| 20.24167 | | 243.5561 | | | | 1.626313 | | | | 4922.5 | | | 24.38281 | |  | |  | |  |
| 20.25 | | 243.6483 | | | | 1.62815 | | | | 4922.242 | | | 24.47656 | |  | |  | |  |
| 20.25833 | | 243.7413 | | | | 1.630312 | | | | 4922.031 | | | 24.67969 | |  | |  | |  |
| 20.26667 | | 243.8318 | | | | 1.630946 | | | | 4921.828 | | | 24.70313 | |  | |  | |  |
| 20.275 | | 243.9224 | | | | 1.633099 | | | | 4921.617 | | | 25.02344 | |  | |  | |  |
| 20.28333 | | 244.0082 | | | | 1.636722 | | | | 4921.406 | | | 25.10156 | |  | |  | |  |
| 20.29167 | | 244.0988 | | | | 1.637843 | | | | 4921.188 | | | 25.17188 | |  | |  | |  |
| 20.3 | | 244.1869 | | | | 1.639724 | | | | 4920.969 | | | 25.39063 | |  | |  | |  |
| 20.30833 | | 244.2791 | | | | 1.643215 | | | | 4920.75 | | | 25.41406 | |  | |  | |  |
| 20.31667 | | 244.3705 | | | | 1.644356 | | | | 4920.531 | | | 25.76563 | |  | |  | |  |
| 20.325 | | 244.4627 | | | | 1.646482 | | | | 4920.281 | | | 25.82031 | |  | |  | |  |
| 20.33333 | | 244.5517 | | | | 1.650823 | | | | 4920.07 | | | 26.14844 | |  | |  | |  |
| 20.34167 | | 244.6406 | | | | 1.652615 | | | | 4919.852 | | | 26.10156 | |  | |  | |  |
| 20.35 | | 244.7288 | | | | 1.654615 | | | | 4919.609 | | | 26.15625 | |  | |  | |  |
| 20.35833 | | 244.8218 | | | | 1.65921 | | | | 4919.391 | | | 26.11719 | |  | |  | |  |
| 20.36667 | | 244.9156 | | | | 1.661553 | | | | 4919.141 | | | 25.92188 | |  | |  | |  |
| 20.375 | | 245.007 | | | | 1.662799 | | | | 4918.922 | | | 26.10156 | |  | |  | |  |
| 20.38333 | | 245.0967 | | | | 1.66655 | | | | 4918.648 | | | 26.29688 | |  | |  | |  |
| 20.39167 | | 245.1841 | | | | 1.667814 | | | | 4918.453 | | | 26.51563 | |  | |  | |  |
| 20.4 | | 245.2771 | | | | 1.668834 | | | | 4918.227 | | | 26.57813 | |  | |  | |  |
| 20.40833 | | 245.3685 | | | | 1.672525 | | | | 4918.023 | | | 26.85938 | |  | |  | |  |
| 20.41667 | | 245.4606 | | | | 1.674141 | | | | 4917.828 | | | 26.55469 | |  | |  | |  |
| 20.425 | | 245.552 | | | | 1.675342 | | | | 4917.594 | | | 26.98438 | |  | |  | |  |
| 20.43333 | | 245.645 | | | | 1.679654 | | | | 4917.352 | | | 27.15625 | |  | |  | |  |
| 20.44167 | | 245.7348 | | | | 1.681596 | | | | 4917.109 | | | 27.36719 | |  | |  | |  |
| 20.45 | | 245.827 | | | | 1.682567 | | | | 4916.875 | | | 27.78906 | |  | |  | |  |
| 20.45833 | | 245.9167 | | | | 1.685281 | | | | 4916.617 | | | 27.9375 | |  | |  | |  |
| 20.46667 | | 246.0097 | | | | 1.688346 | | | | 4916.438 | | | 28.21094 | |  | |  | |  |
| 20.475 | | 246.1051 | | | | 1.690388 | | | | 4916.156 | | | 28.35938 | |  | |  | |  |
| 20.48333 | | 246.1957 | | | | 1.692055 | | | | 4915.914 | | | 28.50781 | |  | |  | |  |
| 20.49167 | | 246.2855 | | | | 1.695968 | | | | 4915.664 | | | 28.74219 | |  | |  | |  |
| 20.5 | | 246.3728 | | | | 1.697635 | | | | 4915.375 | | | 29.00781 | |  | |  | |  |
| 20.50833 | | 246.4658 | | | | 1.700324 | | | | 4915.117 | | | 29.10938 | |  | |  | |  |
| 20.51667 | | 246.558 | | | | 1.704436 | | | | 4914.852 | | | 29.27344 | |  | |  | |  |
| 20.525 | | 246.6478 | | | | 1.706317 | | | | 4914.602 | | | 29.21094 | |  | |  | |  |
| 20.53333 | | 246.7368 | | | | 1.708431 | | | | 4914.344 | | | 29.16406 | |  | |  | |  |
| 20.54167 | | 246.8289 | | | | 1.712371 | | | | 4914.078 | | | 29.21875 | |  | |  | |  |
| 20.55 | | 246.9195 | | | | 1.714759 | | | | 4913.797 | | | 28.9375 | |  | |  | |  |
| 20.55833 | | 247.0053 | | | | 1.715786 | | | | 4913.555 | | | 29.01563 | |  | |  | |  |
| 20.56667 | | 247.0935 | | | | 1.719686 | | | | 4913.289 | | | 28.98438 | |  | |  | |  |
| 20.575 | | 247.1865 | | | | 1.722013 | | | | 4913.055 | | | 29.125 | |  | |  | |  |
| 20.58333 | | 247.2811 | | | | 1.723933 | | | | 4912.813 | | | 29.1875 | |  | |  | |  |
| 20.59167 | | 247.37 | | | | 1.727719 | | | | 4912.57 | | | 29.40625 | |  | |  | |  |
| 20.6 | | 247.4574 | | | | 1.730678 | | | | 4912.367 | | | 29.67969 | |  | |  | |  |
| 20.60833 | | 247.5472 | | | | 1.731763 | | | | 4912.117 | | | 29.92969 | |  | |  | |  |
| 20.61667 | | 247.6402 | | | | 1.735024 | | | | 4911.883 | | | 30.27344 | |  | |  | |  |
| 20.625 | | 247.7339 | | | | 1.738828 | | | | 4911.625 | | | 30.51563 | |  | |  | |  |
| 20.63333 | | 247.8285 | | | | 1.739577 | | | | 4911.391 | | | 30.99219 | |  | |  | |  |
| 20.64167 | | 247.9239 | | | | 1.742682 | | | | 4911.102 | | | 31.5625 | |  | |  | |  |
| 20.65 | | 248.0177 | | | | 1.746593 | | | | 4910.828 | | | 31.86719 | |  | |  | |  |
| 20.65833 | | 248.1099 | | | | 1.748307 | | | | 4910.539 | | | 32.13281 | |  | |  | |  |
| 20.66667 | | 248.2013 | | | | 1.749725 | | | | 4910.242 | | | 32.41406 | |  | |  | |  |
| 20.675 | | 248.2918 | | | | 1.752887 | | | | 4909.953 | | | 32.78125 | |  | |  | |  |
| 20.68333 | | 248.3832 | | | | 1.755836 | | | | 4909.633 | | | 32.88281 | |  | |  | |  |
| 20.69167 | | 248.4762 | | | | 1.757629 | | | | 4909.297 | | | 33.35156 | |  | |  | |  |
| 20.7 | | 248.5692 | | | | 1.761358 | | | | 4908.992 | | | 33.39844 | |  | |  | |  |
| 20.70833 | | 248.659 | | | | 1.764341 | | | | 4908.688 | | | 33.8125 | |  | |  | |  |
| 20.71667 | | 248.7487 | | | | 1.765682 | | | | 4908.383 | | | 34.03906 | |  | |  | |  |
| 20.725 | | 248.8385 | | | | 1.768981 | | | | 4908.063 | | | 34.13281 | |  | |  | |  |
| 20.73333 | | 248.9307 | | | | 1.7722 | | | | 4907.773 | | | 34.44531 | |  | |  | |  |
| 20.74167 | | 249.0213 | | | | 1.773729 | | | | 4907.445 | | | 34.60938 | |  | |  | |  |
| 20.75 | | 249.1111 | | | | 1.776763 | | | | 4907.164 | | | 34.92188 | |  | |  | |  |
| 20.75833 | | 249.2048 | | | | 1.780328 | | | | 4906.828 | | | 34.92969 | |  | |  | |  |
| 20.76667 | | 249.2994 | | | | 1.781399 | | | | 4906.523 | | | 35.39063 | |  | |  | |  |
| 20.775 | | 249.3924 | | | | 1.784333 | | | | 4906.227 | | | 35.55469 | |  | |  | |  |
| 20.78333 | | 249.4806 | | | | 1.788791 | | | | 4905.906 | | | 35.92188 | |  | |  | |  |
| 20.79167 | | 249.5712 | | | | 1.791231 | | | | 4905.594 | | | 36.02344 | |  | |  | |  |
| 20.8 | | 249.6633 | | | | 1.794516 | | | | 4905.273 | | | 36.19531 | |  | |  | |  |
| 20.80833 | | 249.7579 | | | | 1.799091 | | | | 4904.977 | | | 36.40625 | |  | |  | |  |
| 20.81667 | | 249.8509 | | | | 1.801629 | | | | 4904.641 | | | 36.6875 | |  | |  | |  |
| 20.825 | | 249.9423 | | | | 1.803652 | | | | 4904.313 | | | 37.02344 | |  | |  | |  |
| 20.83333 | | 250.0345 | | | | 1.807195 | | | | 4903.969 | | | 37.17188 | |  | |  | |  |
| 20.84167 | | 250.1259 | | | | 1.810714 | | | | 4903.664 | | | 37.16406 | |  | |  | |  |
| 20.85 | | 250.218 | | | | 1.812855 | | | | 4903.344 | | | 37.58594 | |  | |  | |  |
| 20.85833 | | 250.3102 | | | | 1.81622 | | | | 4903.008 | | | 37.58594 | |  | |  | |  |
| 20.86667 | | 250.4 | | | | 1.820774 | | | | 4902.672 | | | 37.40625 | |  | |  | |  |
| 20.875 | | 250.4906 | | | | 1.823 | | | | 4902.328 | | | 37.42969 | |  | |  | |  |
| 20.88333 | | 250.582 | | | | 1.825561 | | | | 4901.992 | | | 37.66406 | |  | |  | |  |
| 20.89167 | | 250.6741 | | | | 1.830243 | | | | 4901.688 | | | 37.63281 | |  | |  | |  |
| 20.9 | | 250.7623 | | | | 1.832428 | | | | 4901.328 | | | 37.71094 | |  | |  | |  |
| 20.90833 | | 250.8529 | | | | 1.835833 | | | | 4901.023 | | | 37.83594 | |  | |  | |  |
| 20.91667 | | 250.9459 | | | | 1.839855 | | | | 4900.742 | | | 38.01563 | |  | |  | |  |
| 20.925 | | 251.0357 | | | | 1.842637 | | | | 4900.422 | | | 38.30469 | |  | |  | |  |
| 20.93333 | | 251.1262 | | | | 1.845613 | | | | 4900.102 | | | 38.38281 | |  | |  | |  |
| 20.94167 | | 251.216 | | | | 1.849194 | | | | 4899.789 | | | 38.75781 | |  | |  | |  |
| 20.95 | | 251.309 | | | | 1.851426 | | | | 4899.477 | | | 39.11719 | |  | |  | |  |
| 20.95833 | | 251.3996 | | | | 1.854176 | | | | 4899.156 | | | 39.46094 | |  | |  | |  |
| 20.96667 | | 251.491 | | | | 1.85913 | | | | 4898.82 | | | 39.53125 | |  | |  | |  |
| 20.975 | | 251.5807 | | | | 1.862594 | | | | 4898.469 | | | 39.64844 | |  | |  | |  |
| 20.98333 | | 251.6721 | | | | 1.865284 | | | | 4898.148 | | | 40.13281 | |  | |  | |  |
| 20.99167 | | 251.7659 | | | | 1.870587 | | | | 4897.781 | | | 40.32031 | |  | |  | |  |
| 21 | | 251.8549 | | | | 1.873473 | | | | 4897.398 | | | 40.47656 | |  | |  | |  |
| 21.00833 | | 251.9463 | | | | 1.876038 | | | | 4897.039 | | | 40.96875 | |  | |  | |  |
| 21.01667 | | 252.036 | | | | 1.880134 | | | | 4896.703 | | | 41.23438 | |  | |  | |  |
| 21.025 | | 252.1266 | | | | 1.882751 | | | | 4896.344 | | | 41.29688 | |  | |  | |  |
| 21.03333 | | 252.218 | | | | 1.884332 | | | | 4895.953 | | | 41.625 | |  | |  | |  |
| 21.04167 | | 252.311 | | | | 1.888551 | | | | 4895.594 | | | 42.00781 | |  | |  | |  |
| 21.05 | | 252.4008 | | | | 1.891244 | | | | 4895.234 | | | 42.35156 | |  | |  | |  |
| 21.05833 | | 252.4889 | | | | 1.893133 | | | | 4894.828 | | | 42.73438 | |  | |  | |  |
| 21.06667 | | 252.5819 | | | | 1.897443 | | | | 4894.453 | | | 42.76563 | |  | |  | |  |
| 21.075 | | 252.6741 | | | | 1.901979 | | | | 4894.102 | | | 43.07031 | |  | |  | |  |
| 21.08333 | | 252.7671 | | | | 1.90505 | | | | 4893.711 | | | 43.30469 | |  | |  | |  |
| 21.09167 | | 252.8585 | | | | 1.908168 | | | | 4893.313 | | | 43.33594 | |  | |  | |  |
| 21.1 | | 252.9522 | | | | 1.913411 | | | | 4892.906 | | | 43.76563 | |  | |  | |  |
| 21.10833 | | 253.0476 | | | | 1.916918 | | | | 4892.516 | | | 44.1875 | |  | |  | |  |
| 21.11667 | | 253.1398 | | | | 1.918609 | | | | 4892.156 | | | 44.42188 | |  | |  | |  |
| 21.125 | | 253.2328 | | | | 1.922763 | | | | 4891.758 | | | 44.57031 | |  | |  | |  |
| 21.13333 | | 253.3258 | | | | 1.925846 | | | | 4891.375 | | | 44.69531 | |  | |  | |  |
| 21.14167 | | 253.4204 | | | | 1.928855 | | | | 4891.008 | | | 45.09375 | |  | |  | |  |
| 21.15 | | 253.5134 | | | | 1.933558 | | | | 4890.594 | | | 45.34375 | |  | |  | |  |
| 21.15833 | | 253.6039 | | | | 1.936987 | | | | 4890.172 | | | 45.61719 | |  | |  | |  |
| 21.16667 | | 253.6961 | | | | 1.939703 | | | | 4889.773 | | | 45.84375 | |  | |  | |  |
| 21.175 | | 253.7899 | | | | 1.945004 | | | | 4889.383 | | | 45.92188 | |  | |  | |  |
| 21.18333 | | 253.8813 | | | | 1.949861 | | | | 4888.992 | | | 46.07813 | |  | |  | |  |
| 21.19167 | | 253.9735 | | | | 1.951707 | | | | 4888.578 | | | 45.98438 | |  | |  | |  |
| 21.2 | | 254.0672 | | | | 1.956011 | | | | 4888.164 | | | 46.35938 | |  | |  | |  |
| 21.20833 | | 254.1602 | | | | 1.960693 | | | | 4887.75 | | | 46.50781 | |  | |  | |  |
| 21.21667 | | 254.2524 | | | | 1.963963 | | | | 4887.352 | | | 46.85156 | |  | |  | |  |
| 21.225 | | 254.3462 | | | | 1.966606 | | | | 4886.945 | | | 47.09375 | |  | |  | |  |
| 21.23333 | | 254.436 | | | | 1.971213 | | | | 4886.555 | | | 47.25 | |  | |  | |  |
| 21.24167 | | 254.5274 | | | | 1.973734 | | | | 4886.188 | | | 47.36719 | |  | |  | |  |
| 21.25 | | 254.6219 | | | | 1.977234 | | | | 4885.75 | | | 47.82813 | |  | |  | |  |
| 21.25833 | | 254.7157 | | | | 1.981357 | | | | 4885.352 | | | 48.15625 | |  | |  | |  |
| 21.26667 | | 254.8079 | | | | 1.984666 | | | | 4884.93 | | | 48.48438 | |  | |  | |  |
| 21.275 | | 254.8993 | | | | 1.987729 | | | | 4884.516 | | | 48.79688 | |  | |  | |  |
| 21.28333 | | 254.9907 | | | | 1.992718 | | | | 4884.102 | | | 49.24219 | |  | |  | |  |
| 21.29167 | | 255.082 | | | | 1.997048 | | | | 4883.703 | | | 49.60938 | |  | |  | |  |
| 21.3 | | 255.1742 | | | | 2.000271 | | | | 4883.25 | | | 50.03125 | |  | |  | |  |
| 21.30833 | | 255.264 | | | | 2.004882 | | | | 4882.813 | | | 50.57031 | |  | |  | |  |
| 21.31667 | | 255.3594 | | | | 2.009454 | | | | 4882.367 | | | 51 | |  | |  | |  |
| 21.325 | | 255.4548 | | | | 2.011926 | | | | 4881.922 | | | 51.59375 | |  | |  | |  |
| 21.33333 | | 255.5478 | | | | 2.01616 | | | | 4881.461 | | | 52.01563 | |  | |  | |  |
| 21.34167 | | 255.6391 | | | | 2.021556 | | | | 4881 | | | 52.44531 | |  | |  | |  |
| 21.35 | | 255.7329 | | | | 2.023883 | | | | 4880.523 | | | 52.79688 | |  | |  | |  |
| 21.35833 | | 255.8291 | | | | 2.028327 | | | | 4880.039 | | | 53.22656 | |  | |  | |  |
| 21.36667 | | 255.9245 | | | | 2.034391 | | | | 4879.555 | | | 53.26563 | |  | |  | |  |
| 21.375 | | 256.0191 | | | | 2.038207 | | | | 4879.055 | | | 53.80469 | |  | |  | |  |
| 21.38333 | | 256.1129 | | | | 2.04206 | | | | 4878.57 | | | 54.02344 | |  | |  | |  |
| 21.39167 | | 256.2067 | | | | 2.047354 | | | | 4878.07 | | | 54.27344 | |  | |  | |  |
| 21.4 | | 256.2972 | | | | 2.052183 | | | | 4877.594 | | | 54.63281 | |  | |  | |  |
| 21.40833 | | 256.3894 | | | | 2.054856 | | | | 4877.094 | | | 54.78906 | |  | |  | |  |
| 21.41667 | | 256.4792 | | | | 2.058721 | | | | 4876.656 | | | 55.10938 | |  | |  | |  |
| 21.425 | | 256.5691 | | | | 2.06352 | | | | 4876.141 | | | 55.3125 | |  | |  | |  |
| 21.43333 | | 256.6605 | | | | 2.066767 | | | | 4875.672 | | | 55.6875 | |  | |  | |  |
| 21.44167 | | 256.7473 | | | | 2.070942 | | | | 4875.18 | | | 56.01563 | |  | |  | |  |
| 21.45 | | 256.8379 | | | | 2.075905 | | | | 4874.68 | | | 56.49219 | |  | |  | |  |
| 21.45833 | | 256.927 | | | | 2.079131 | | | | 4874.203 | | | 56.57813 | |  | |  | |  |
| 21.46667 | | 257.0185 | | | | 2.083398 | | | | 4873.727 | | | 56.98438 | |  | |  | |  |
| 21.475 | | 257.11 | | | | 2.088852 | | | | 4873.242 | | | 57.30469 | |  | |  | |  |
| 21.48333 | | 257.203 | | | | 2.091959 | | | | 4872.734 | | | 57.58594 | |  | |  | |  |
| 21.49167 | | 257.2913 | | | | 2.096132 | | | | 4872.227 | | | 57.79688 | |  | |  | |  |
| 21.5 | | 257.3804 | | | | 2.103306 | | | | 4871.703 | | | 58.1875 | |  | |  | |  |
| 21.50833 | | 257.4719 | | | | 2.107568 | | | | 4871.219 | | | 58.60938 | |  | |  | |  |
| 21.51667 | | 257.5641 | | | | 2.111641 | | | | 4870.688 | | | 58.875 | |  | |  | |  |
| 21.525 | | 257.6571 | | | | 2.117569 | | | | 4870.195 | | | 59.21094 | |  | |  | |  |
| 21.53333 | | 257.7447 | | | | 2.12166 | | | | 4869.672 | | | 59.60156 | |  | |  | |  |
| 21.54167 | | 257.8345 | | | | 2.124178 | | | | 4869.172 | | | 60.02344 | |  | |  | |  |
| 21.55 | | 257.9268 | | | | 2.128426 | | | | 4868.641 | | | 60.42969 | |  | |  | |  |
| 21.55833 | | 258.0206 | | | | 2.133064 | | | | 4868.094 | | | 60.92188 | |  | |  | |  |
| 21.56667 | | 258.1105 | | | | 2.13661 | | | | 4867.57 | | | 61.4375 | |  | |  | |  |
| 21.575 | | 258.2036 | | | | 2.141229 | | | | 4867.047 | | | 61.9375 | |  | |  | |  |
| 21.58333 | | 258.3013 | | | | 2.147326 | | | | 4866.516 | | | 62.21094 | |  | |  | |  |
| 21.59167 | | 258.3936 | | | | 2.151758 | | | | 4865.961 | | | 62.71094 | |  | |  | |  |
| 21.6 | | 258.4827 | | | | 2.155066 | | | | 4865.414 | | | 62.94531 | |  | |  | |  |
| 21.60833 | | 258.5725 | | | | 2.161797 | | | | 4864.844 | | | 63.50781 | |  | |  | |  |
| 21.61667 | | 258.668 | | | | 2.166798 | | | | 4864.281 | | | 63.98438 | |  | |  | |  |
| 21.625 | | 258.7594 | | | | 2.170491 | | | | 4863.703 | | | 64.25 | |  | |  | |  |
| 21.63333 | | 258.8485 | | | | 2.176454 | | | | 4863.156 | | | 64.60938 | |  | |  | |  |
| 21.64167 | | 258.9376 | | | | 2.181887 | | | | 4862.578 | | | 64.94531 | |  | |  | |  |
| 21.65 | | 259.0299 | | | | 2.185479 | | | | 4862.016 | | | 65.35938 | |  | |  | |  |
| 21.65833 | | 259.1253 | | | | 2.189788 | | | | 4861.43 | | | 65.60938 | |  | |  | |  |
| 21.66667 | | 259.2136 | | | | 2.194699 | | | | 4860.836 | | | 66.07813 | |  | |  | |  |
| 21.675 | | 259.3051 | | | | 2.198376 | | | | 4860.273 | | | 66.29688 | |  | |  | |  |
| 21.68333 | | 259.3957 | | | | 2.20252 | | | | 4859.695 | | | 66.64063 | |  | |  | |  |
| 21.69167 | | 259.4872 | | | | 2.20786 | | | | 4859.109 | | | 66.96875 | |  | |  | |  |
| 21.7 | | 259.5771 | | | | 2.211549 | | | | 4858.523 | | | 67.30469 | |  | |  | |  |
| 21.70833 | | 259.6701 | | | | 2.216524 | | | | 4857.945 | | | 67.79688 | |  | |  | |  |
| 21.71667 | | 259.7608 | | | | 2.2225 | | | | 4857.328 | | | 68.17188 | |  | |  | |  |
| 21.725 | | 259.8546 | | | | 2.226814 | | | | 4856.758 | | | 68.36719 | |  | |  | |  |
| 21.73333 | | 259.9461 | | | | 2.230869 | | | | 4856.156 | | | 68.57813 | |  | |  | |  |
| 21.74167 | | 260.0359 | | | | 2.236491 | | | | 4855.563 | | | 69.08594 | |  | |  | |  |
| 21.75 | | 260.1282 | | | | 2.240881 | | | | 4854.961 | | | 69.47656 | |  | |  | |  |
| 21.75833 | | 260.2205 | | | | 2.245065 | | | | 4854.344 | | | 69.92969 | |  | |  | |  |
| 21.76667 | | 260.3143 | | | | 2.250861 | | | | 4853.734 | | | 70.32031 | |  | |  | |  |
| 21.775 | | 260.4065 | | | | 2.256421 | | | | 4853.141 | | | 70.84375 | |  | |  | |  |
| 21.78333 | | 260.498 | | | | 2.259878 | | | | 4852.539 | | | 71.19531 | |  | |  | |  |
| 21.79167 | | 260.591 | | | | 2.266138 | | | | 4851.914 | | | 71.82031 | |  | |  | |  |
| 21.8 | | 260.6849 | | | | 2.27234 | | | | 4851.281 | | | 72.22656 | |  | |  | |  |
| 21.80833 | | 260.7755 | | | | 2.276975 | | | | 4850.656 | | | 72.77344 | |  | |  | |  |
| 21.81667 | | 260.8686 | | | | 2.281757 | | | | 4850.023 | | | 73.16406 | |  | |  | |  |
| 21.825 | | 260.9593 | | | | 2.287973 | | | | 4849.375 | | | 73.73438 | |  | |  | |  |
| 21.83333 | | 261.0499 | | | | 2.291725 | | | | 4848.734 | | | 74.07813 | |  | |  | |  |
| 21.84167 | | 261.1414 | | | | 2.295981 | | | | 4848.063 | | | 74.40625 | |  | |  | |  |
| 21.85 | | 261.2344 | | | | 2.302054 | | | | 4847.406 | | | 74.84375 | |  | |  | |  |
| 21.85833 | | 261.3275 | | | | 2.307389 | | | | 4846.742 | | | 75.52344 | |  | |  | |  |
| 21.86667 | | 261.4205 | | | | 2.310946 | | | | 4846.086 | | | 75.92188 | |  | |  | |  |
| 21.875 | | 261.5112 | | | | 2.317138 | | | | 4845.406 | | | 76.05469 | |  | |  | |  |
| 21.88333 | | 261.6034 | | | | 2.32384 | | | | 4844.75 | | | 76.57813 | |  | |  | |  |
| 21.89167 | | 261.6957 | | | | 2.327412 | | | | 4844.094 | | | 77.04688 | |  | |  | |  |
| 21.9 | | 261.7879 | | | | 2.333462 | | | | 4843.422 | | | 77.60938 | |  | |  | |  |
| 21.90833 | | 261.8763 | | | | 2.339871 | | | | 4842.703 | | | 78.01563 | |  | |  | |  |
| 21.91667 | | 261.9701 | | | | 2.343084 | | | | 4842.031 | | | 78.4375 | |  | |  | |  |
| 21.925 | | 262.0631 | | | | 2.348519 | | | | 4841.391 | | | 79.10156 | |  | |  | |  |
| 21.93333 | | 262.1562 | | | | 2.354926 | | | | 4840.688 | | | 79.8125 | |  | |  | |  |
| 21.94167 | | 262.2468 | | | | 2.358831 | | | | 4839.992 | | | 79.83594 | |  | |  | |  |
| 21.95 | | 262.3383 | | | | 2.36441 | | | | 4839.281 | | | 80.42188 | |  | |  | |  |
| 21.95833 | | 262.4313 | | | | 2.371066 | | | | 4838.57 | | | 81.23438 | |  | |  | |  |
| 21.96667 | | 262.5236 | | | | 2.376729 | | | | 4837.867 | | | 81.77344 | |  | |  | |  |
| 21.975 | | 262.6158 | | | | 2.381013 | | | | 4837.133 | | | 82.26563 | |  | |  | |  |
| 21.98333 | | 262.7065 | | | | 2.386766 | | | | 4836.375 | | | 82.8125 | |  | |  | |  |
| 21.99167 | | 262.8011 | | | | 2.392806 | | | | 4835.719 | | | 83.38281 | |  | |  | |  |
| 22 | | 262.891 | | | | 2.397323 | | | | 4834.969 | | | 83.96875 | |  | |  | |  |
| 22.00833 | | 262.984 | | | | 2.403946 | | | | 4834.195 | | | 84.46875 | |  | |  | |  |
| 22.01667 | | 263.0724 | | | | 2.410237 | | | | 4833.453 | | | 84.85938 | |  | |  | |  |
| 22.025 | | 263.1654 | | | | 2.415872 | | | | 4832.703 | | | 85.41406 | |  | |  | |  |
| 22.03333 | | 263.2577 | | | | 2.422313 | | | | 4831.953 | | | 86.01563 | |  | |  | |  |
| 22.04167 | | 263.3515 | | | | 2.428859 | | | | 4831.195 | | | 86.59375 | |  | |  | |  |
| 22.05 | | 263.4445 | | | | 2.433754 | | | | 4830.422 | | | 86.89844 | |  | |  | |  |
| 22.05833 | | 263.5352 | | | | 2.438942 | | | | 4829.656 | | | 87.60938 | |  | |  | |  |
| 22.06667 | | 263.629 | | | | 2.44589 | | | | 4828.906 | | | 88.16406 | |  | |  | |  |
| 22.075 | | 263.7236 | | | | 2.451702 | | | | 4828.133 | | | 88.42969 | |  | |  | |  |
| 22.08333 | | 263.819 | | | | 2.456955 | | | | 4827.344 | | | 88.95313 | |  | |  | |  |
| 22.09167 | | 263.9113 | | | | 2.46333 | | | | 4826.563 | | | 89.50781 | |  | |  | |  |
| 22.1 | | 264.0067 | | | | 2.469285 | | | | 4825.797 | | | 89.70313 | |  | |  | |  |
| 22.10833 | | 264.0989 | | | | 2.474546 | | | | 4824.984 | | | 90.58594 | |  | |  | |  |
| 22.11667 | | 264.1936 | | | | 2.480088 | | | | 4824.188 | | | 91.07813 | |  | |  | |  |
| 22.125 | | 264.285 | | | | 2.488397 | | | | 4823.422 | | | 91.45313 | |  | |  | |  |
| 22.13333 | | 264.3757 | | | | 2.493932 | | | | 4822.625 | | | 92.09375 | |  | |  | |  |
| 22.14167 | | 264.4735 | | | | 2.498877 | | | | 4821.82 | | | 92.38281 | |  | |  | |  |
| 22.15 | | 264.5649 | | | | 2.506027 | | | | 4821.063 | | | 92.89063 | |  | |  | |  |
| 22.15833 | | 264.6588 | | | | 2.511954 | | | | 4820.203 | | | 93.74219 | |  | |  | |  |
| 22.16667 | | 264.7471 | | | | 2.517058 | | | | 4819.383 | | | 94.33594 | |  | |  | |  |
| 22.175 | | 264.8401 | | | | 2.524317 | | | | 4818.578 | | | 95.16406 | |  | |  | |  |
| 22.18333 | | 264.9331 | | | | 2.531273 | | | | 4817.742 | | | 96.29688 | |  | |  | |  |
| 22.19167 | | 265.0262 | | | | 2.536936 | | | | 4816.938 | | | 96.70313 | |  | |  | |  |
| 22.2 | | 265.1185 | | | | 2.542759 | | | | 4816.094 | | | 97.14063 | |  | |  | |  |
| 22.20833 | | 265.2102 | | | | 2.549745 | | | | 4815.211 | | | 97.73438 | |  | |  | |  |
| 22.21667 | | 265.3042 | | | | 2.554623 | | | | 4814.359 | | | 98.4375 | |  | |  | |  |
| 22.225 | | 265.3959 | | | | 2.559789 | | | | 4813.461 | | | 99.32031 | |  | |  | |  |
| 22.23333 | | 265.4892 | | | | 2.567487 | | | | 4812.516 | | | 100.0781 | |  | |  | |  |
| 22.24167 | | 265.5793 | | | | 2.573788 | | | | 4811.672 | | | 100.6953 | |  | |  | |  |
| 22.25 | | 265.6726 | | | | 2.579291 | | | | 4810.813 | | | 101.3828 | |  | |  | |  |
| 22.25833 | | 265.7635 | | | | 2.587124 | | | | 4809.93 | | | 101.8125 | |  | |  | |  |
| 22.26667 | | 265.8545 | | | | 2.594299 | | | | 4809.016 | | | 102.2188 | |  | |  | |  |
| 22.275 | | 265.9485 | | | | 2.5997 | | | | 4808.094 | | | 102.9063 | |  | |  | |  |
| 22.28333 | | 266.0402 | | | | 2.606564 | | | | 4807.172 | | | 103.3984 | |  | |  | |  |
| 22.29167 | | 266.1343 | | | | 2.613011 | | | | 4806.258 | | | 104.2891 | |  | |  | |  |
| 22.3 | | 266.226 | | | | 2.61821 | | | | 4805.336 | | | 104.7578 | |  | |  | |  |
| 22.30833 | | 266.3161 | | | | 2.623194 | | | | 4804.445 | | | 105.125 | |  | |  | |  |
| 22.31667 | | 266.4086 | | | | 2.630105 | | | | 4803.547 | | | 105.7109 | |  | |  | |  |
| 22.325 | | 266.5027 | | | | 2.635245 | | | | 4802.609 | | | 106.5 | |  | |  | |  |
| 22.33333 | | 266.5959 | | | | 2.641663 | | | | 4801.688 | | | 107.375 | |  | |  | |  |
| 22.34167 | | 266.6884 | | | | 2.649429 | | | | 4800.719 | | | 108.0391 | |  | |  | |  |
| 22.35 | | 266.7794 | | | | 2.655478 | | | | 4799.789 | | | 108.6172 | |  | |  | |  |
| 22.35833 | | 266.8711 | | | | 2.662077 | | | | 4798.883 | | | 109.2188 | |  | |  | |  |
| 22.36667 | | 266.9636 | | | | 2.669506 | | | | 4797.93 | | | 109.8906 | |  | |  | |  |
| 22.375 | | 267.0553 | | | | 2.67726 | | | | 4796.945 | | | 110.5234 | |  | |  | |  |
| 22.38333 | | 267.1493 | | | | 2.682903 | | | | 4795.945 | | | 111.2813 | |  | |  | |  |
| 22.39167 | | 267.241 | | | | 2.689672 | | | | 4794.961 | | | 112.0625 | |  | |  | |  |
| 22.4 | | 267.3359 | | | | 2.697773 | | | | 4793.992 | | | 112.7578 | |  | |  | |  |
| 22.40833 | | 267.426 | | | | 2.703369 | | | | 4793.016 | | | 113.1016 | |  | |  | |  |
| 22.41667 | | 267.5216 | | | | 2.711409 | | | | 4792.016 | | | 113.5313 | |  | |  | |  |
| 22.425 | | 267.6133 | | | | 2.719586 | | | | 4791.031 | | | 114.1406 | |  | |  | |  |
| 22.43333 | | 267.7066 | | | | 2.725912 | | | | 4790.016 | | | 114.8906 | |  | |  | |  |
| 22.44167 | | 267.7991 | | | | 2.732749 | | | | 4788.984 | | | 115.6719 | |  | |  | |  |
| 22.45 | | 267.8916 | | | | 2.740698 | | | | 4787.969 | | | 116.4453 | |  | |  | |  |
| 22.45833 | | 267.9841 | | | | 2.747618 | | | | 4786.984 | | | 117.1719 | |  | |  | |  |
| 22.46667 | | 268.075 | | | | 2.752066 | | | | 4786 | | | 117.8359 | |  | |  | |  |
| 22.475 | | 268.1698 | | | | 2.759413 | | | | 4784.984 | | | 118.5078 | |  | |  | |  |
| 22.48333 | | 268.26 | | | | 2.767634 | | | | 4783.938 | | | 118.9453 | |  | |  | |  |
| 22.49167 | | 268.3524 | | | | 2.772943 | | | | 4782.883 | | | 119.6328 | |  | |  | |  |
| 22.5 | | 268.4442 | | | | 2.779911 | | | | 4781.82 | | | 120.2656 | |  | |  | |  |
| 22.50833 | | 268.5382 | | | | 2.787846 | | | | 4780.766 | | | 121.0156 | |  | |  | |  |
| 22.51667 | | 268.6339 | | | | 2.795145 | | | | 4779.703 | | | 121.6797 | |  | |  | |  |
| 22.525 | | 268.7263 | | | | 2.801104 | | | | 4778.648 | | | 122.3594 | |  | |  | |  |
| 22.53333 | | 268.8196 | | | | 2.809409 | | | | 4777.609 | | | 122.9531 | |  | |  | |  |
| 22.54167 | | 268.9105 | | | | 2.817184 | | | | 4776.539 | | | 123.7656 | |  | |  | |  |
| 22.55 | | 269.0046 | | | | 2.823667 | | | | 4775.461 | | | 124.5469 | |  | |  | |  |
| 22.55833 | | 269.0971 | | | | 2.831717 | | | | 4774.367 | | | 125.3203 | |  | |  | |  |
| 22.56667 | | 269.1911 | | | | 2.839681 | | | | 4773.281 | | | 126.1016 | |  | |  | |  |
| 22.575 | | 269.2852 | | | | 2.844745 | | | | 4772.172 | | | 126.7891 | |  | |  | |  |
| 22.58333 | | 269.3792 | | | | 2.851537 | | | | 4771.07 | | | 127.5781 | |  | |  | |  |
| 22.59167 | | 269.4749 | | | | 2.860234 | | | | 4769.953 | | | 128.4688 | |  | |  | |  |
| 22.6 | | 269.5689 | | | | 2.866638 | | | | 4768.82 | | | 129.3047 | |  | |  | |  |
| 22.60833 | | 269.6622 | | | | 2.873247 | | | | 4767.688 | | | 129.8984 | |  | |  | |  |
| 22.61667 | | 269.757 | | | | 2.880772 | | | | 4766.547 | | | 130.7891 | |  | |  | |  |
| 22.625 | | 269.8542 | | | | 2.88906 | | | | 4765.398 | | | 131.6094 | |  | |  | |  |
| 22.63333 | | 269.9507 | | | | 2.894546 | | | | 4764.242 | | | 132.2578 | |  | |  | |  |
| 22.64167 | | 270.0439 | | | | 2.901382 | | | | 4763.07 | | | 132.9297 | |  | |  | |  |
| 22.65 | | 270.1356 | | | | 2.910526 | | | | 4761.883 | | | 133.8438 | |  | |  | |  |
| 22.65833 | | 270.2312 | | | | 2.916979 | | | | 4760.742 | | | 134.7031 | |  | |  | |  |
| 22.66667 | | 270.3237 | | | | 2.923962 | | | | 4759.547 | | | 135.5313 | |  | |  | |  |
| 22.675 | | 270.4162 | | | | 2.932511 | | | | 4758.359 | | | 136.25 | |  | |  | |  |
| 22.68333 | | 270.5063 | | | | 2.939382 | | | | 4757.18 | | | 136.9766 | |  | |  | |  |
| 22.69167 | | 270.5988 | | | | 2.945823 | | | | 4755.992 | | | 137.9063 | |  | |  | |  |
| 22.7 | | 270.6937 | | | | 2.953116 | | | | 4754.773 | | | 138.7188 | |  | |  | |  |
| 22.70833 | | 270.7862 | | | | 2.961718 | | | | 4753.555 | | | 139.5625 | |  | |  | |  |
| 22.71667 | | 270.8763 | | | | 2.967537 | | | | 4752.32 | | | 140.3203 | |  | |  | |  |
| 22.725 | | 270.968 | | | | 2.973866 | | | | 4751.109 | | | 141.3047 | |  | |  | |  |
| 22.73333 | | 271.0636 | | | | 2.982116 | | | | 4749.883 | | | 142.125 | |  | |  | |  |
| 22.74167 | | 271.1569 | | | | 2.987289 | | | | 4748.625 | | | 143.1016 | |  | |  | |  |
| 22.75 | | 271.251 | | | | 2.992949 | | | | 4747.367 | | | 143.8438 | |  | |  | |  |
| 22.75833 | | 271.3442 | | | | 3.00147 | | | | 4746.109 | | | 144.8906 | |  | |  | |  |
| 22.76667 | | 271.4414 | | | | 3.00814 | | | | 4744.844 | | | 145.9844 | |  | |  | |  |
| 22.775 | | 271.537 | | | | 3.014486 | | | | 4743.555 | | | 147.0625 | |  | |  | |  |
| 22.78333 | | 271.628 | | | | 3.022583 | | | | 4742.266 | | | 147.75 | |  | |  | |  |
| 22.79167 | | 271.7205 | | | | 3.030673 | | | | 4740.961 | | | 148.5625 | |  | |  | |  |
| 22.8 | | 271.8138 | | | | 3.036631 | | | | 4739.68 | | | 149.5703 | |  | |  | |  |
| 22.80833 | | 271.9094 | | | | 3.043199 | | | | 4738.352 | | | 150.3359 | |  | |  | |  |
| 22.81667 | | 272.0019 | | | | 3.052146 | | | | 4737.008 | | | 151.2813 | |  | |  | |  |
| 22.825 | | 272.0928 | | | | 3.058824 | | | | 4735.656 | | | 151.9453 | |  | |  | |  |
| 22.83333 | | 272.1845 | | | | 3.065436 | | | | 4734.344 | | | 152.8125 | |  | |  | |  |
| 22.84167 | | 272.2793 | | | | 3.074284 | | | | 4733.008 | | | 153.4766 | |  | |  | |  |
| 22.85 | | 272.3718 | | | | 3.081102 | | | | 4731.648 | | | 154.3984 | |  | |  | |  |
| 22.85833 | | 272.4643 | | | | 3.087988 | | | | 4730.297 | | | 155.0703 | |  | |  | |  |
| 22.86667 | | 272.5552 | | | | 3.096703 | | | | 4728.938 | | | 156.0078 | |  | |  | |  |
| 22.875 | | 272.6501 | | | | 3.104016 | | | | 4727.602 | | | 156.9141 | |  | |  | |  |
| 22.88333 | | 272.7426 | | | | 3.11024 | | | | 4726.234 | | | 157.8594 | |  | |  | |  |
| 22.89167 | | 272.8351 | | | | 3.118916 | | | | 4724.883 | | | 158.8906 | |  | |  | |  |
| 22.9 | | 272.9275 | | | | 3.127061 | | | | 4723.492 | | | 159.6875 | |  | |  | |  |
| 22.90833 | | 273.0239 | | | | 3.132945 | | | | 4722.125 | | | 160.5938 | |  | |  | |  |
| 22.91667 | | 273.1196 | | | | 3.140547 | | | | 4720.719 | | | 161.5625 | |  | |  | |  |
| 22.925 | | 273.2121 | | | | 3.149096 | | | | 4719.305 | | | 162.6563 | |  | |  | |  |
| 22.93333 | | 273.303 | | | | 3.155668 | | | | 4717.875 | | | 163.4141 | |  | |  | |  |
| 22.94167 | | 273.3955 | | | | 3.163363 | | | | 4716.438 | | | 164.4453 | |  | |  | |  |
| 22.95 | | 273.4934 | | | | 3.17284 | | | | 4715.016 | | | 165.3047 | |  | |  | |  |
| 22.95833 | | 273.5851 | | | | 3.181104 | | | | 4713.57 | | | 166.1641 | |  | |  | |  |
| 22.96667 | | 273.6808 | | | | 3.187465 | | | | 4712.109 | | | 167.1406 | |  | |  | |  |
| 22.975 | | 273.7734 | | | | 3.196275 | | | | 4710.617 | | | 167.8594 | |  | |  | |  |
| 22.98333 | | 273.8659 | | | | 3.203818 | | | | 4709.172 | | | 168.8672 | |  | |  | |  |
| 22.99167 | | 273.9561 | | | | 3.209905 | | | | 4707.695 | | | 169.9375 | |  | |  | |  |
| 23 | | 274.0486 | | | | 3.217804 | | | | 4706.211 | | | 170.9375 | |  | |  | |  |
| 23.00833 | | 274.1387 | | | | 3.225964 | | | | 4704.734 | | | 171.9375 | |  | |  | |  |
| 23.01667 | | 274.232 | | | | 3.233211 | | | | 4703.227 | | | 172.8438 | |  | |  | |  |
| 23.025 | | 274.3268 | | | | 3.240019 | | | | 4701.75 | | | 173.7266 | |  | |  | |  |
| 23.03333 | | 274.4193 | | | | 3.248617 | | | | 4700.227 | | | 174.8906 | |  | |  | |  |
| 23.04167 | | 274.5117 | | | | 3.256757 | | | | 4698.68 | | | 175.9609 | |  | |  | |  |
| 23.05 | | 274.6035 | | | | 3.262993 | | | | 4697.141 | | | 176.9063 | |  | |  | |  |
| 23.05833 | | 274.6967 | | | | 3.271564 | | | | 4695.594 | | | 178.0469 | |  | |  | |  |
| 23.06667 | | 274.7915 | | | | 3.280557 | | | | 4694.047 | | | 179.1172 | |  | |  | |  |
| 23.075 | | 274.8855 | | | | 3.288183 | | | | 4692.508 | | | 180.3359 | |  | |  | |  |
| 23.08333 | | 274.9765 | | | | 3.294974 | | | | 4690.906 | | | 181.5313 | |  | |  | |  |
| 23.09167 | | 275.0713 | | | | 3.30334 | | | | 4689.313 | | | 182.4063 | |  | |  | |  |
| 23.1 | | 275.1653 | | | | 3.312014 | | | | 4687.727 | | | 183.6016 | |  | |  | |  |
| 23.10833 | | 275.2562 | | | | 3.318297 | | | | 4686.117 | | | 185.125 | |  | |  | |  |
| 23.11667 | | 275.3464 | | | | 3.326082 | | | | 4684.484 | | | 186.0469 | |  | |  | |  |
| 23.125 | | 275.4427 | | | | 3.334278 | | | | 4682.844 | | | 186.9766 | |  | |  | |  |
| 23.13333 | | 275.5368 | | | | 3.340337 | | | | 4681.188 | | | 188.0781 | |  | |  | |  |
| 23.14167 | | 275.6308 | | | | 3.34862 | | | | 4679.563 | | | 189.4219 | |  | |  | |  |
| 23.15 | | 275.7225 | | | | 3.357759 | | | | 4677.898 | | | 190.4375 | |  | |  | |  |
| 23.15833 | | 275.8142 | | | | 3.365943 | | | | 4676.164 | | | 191.4844 | |  | |  | |  |
| 23.16667 | | 275.9082 | | | | 3.373937 | | | | 4674.508 | | | 192.4844 | |  | |  | |  |
| 23.175 | | 276.0023 | | | | 3.382678 | | | | 4672.844 | | | 193.5234 | |  | |  | |  |
| 23.18333 | | 276.0932 | | | | 3.39141 | | | | 4671.141 | | | 194.7969 | |  | |  | |  |
| 23.19167 | | 276.188 | | | | 3.397616 | | | | 4669.406 | | | 195.6953 | |  | |  | |  |
| 23.2 | | 276.2813 | | | | 3.405352 | | | | 4667.703 | | | 196.875 | |  | |  | |  |
| 23.20833 | | 276.3707 | | | | 3.414937 | | | | 4665.984 | | | 198.0703 | |  | |  | |  |
| 23.21667 | | 276.4624 | | | | 3.421241 | | | | 4664.266 | | | 199.5234 | |  | |  | |  |
| 23.225 | | 276.5541 | | | | 3.42866 | | | | 4662.539 | | | 200.6875 | |  | |  | |  |
| 23.23333 | | 276.6458 | | | | 3.438075 | | | | 4660.766 | | | 201.6641 | |  | |  | |  |
| 23.24167 | | 276.7383 | | | | 3.444688 | | | | 4659.039 | | | 202.7031 | |  | |  | |  |
| 23.25 | | 276.8315 | | | | 3.452507 | | | | 4657.266 | | | 203.8984 | |  | |  | |  |
| 23.25833 | | 276.9186 | | | | 3.461574 | | | | 4655.477 | | | 205.3047 | |  | |  | |  |
| 23.26667 | | 277.0111 | | | | 3.470015 | | | | 4653.641 | | | 206.6172 | |  | |  | |  |
| 23.275 | | 277.099 | | | | 3.476884 | | | | 4651.836 | | | 207.8828 | |  | |  | |  |
| 23.28333 | | 277.1914 | | | | 3.485243 | | | | 4650.047 | | | 209.0703 | |  | |  | |  |
| 23.29167 | | 277.2855 | | | | 3.494537 | | | | 4648.234 | | | 210.5859 | |  | |  | |  |
| 23.3 | | 277.378 | | | | 3.501032 | | | | 4646.398 | | | 211.6406 | |  | |  | |  |
| 23.30833 | | 277.4697 | | | | 3.508149 | | | | 4644.523 | | | 212.7813 | |  | |  | |  |
| 23.31667 | | 277.5629 | | | | 3.517513 | | | | 4642.641 | | | 214.1797 | |  | |  | |  |
| 23.325 | | 277.657 | | | | 3.524174 | | | | 4640.766 | | | 215.5 | |  | |  | |  |
| 23.33333 | | 277.7494 | | | | 3.530414 | | | | 4638.883 | | | 216.8359 | |  | |  | |  |
| 23.34167 | | 277.845 | | | | 3.538353 | | | | 4636.953 | | | 218.1484 | |  | |  | |  |
| 23.35 | | 277.9375 | | | | 3.54643 | | | | 4635.063 | | | 219.0938 | |  | |  | |  |
| 23.35833 | | 278.0323 | | | | 3.553457 | | | | 4633.164 | | | 220.25 | |  | |  | |  |
| 23.36667 | | 278.1263 | | | | 3.561234 | | | | 4631.211 | | | 221.4922 | |  | |  | |  |
| 23.375 | | 278.2195 | | | | 3.571363 | | | | 4629.266 | | | 222.7578 | |  | |  | |  |
| 23.38333 | | 278.312 | | | | 3.579012 | | | | 4627.297 | | | 223.8047 | |  | |  | |  |
| 23.39167 | | 278.4045 | | | | 3.586097 | | | | 4625.344 | | | 224.9766 | |  | |  | |  |
| 23.4 | | 278.497 | | | | 3.595176 | | | | 4623.414 | | | 226.3438 | |  | |  | |  |
| 23.40833 | | 278.588 | | | | 3.602276 | | | | 4621.445 | | | 227.7109 | |  | |  | |  |
| 23.41667 | | 278.6781 | | | | 3.608851 | | | | 4619.453 | | | 228.7656 | |  | |  | |  |
| 23.425 | | 278.7721 | | | | 3.617334 | | | | 4617.469 | | | 230.0156 | |  | |  | |  |
| 23.43333 | | 278.8677 | | | | 3.625721 | | | | 4615.469 | | | 231.4453 | |  | |  | |  |
| 23.44167 | | 278.9617 | | | | 3.633093 | | | | 4613.469 | | | 233.0313 | |  | |  | |  |
| 23.45 | | 279.0542 | | | | 3.640569 | | | | 4611.414 | | | 234.2969 | |  | |  | |  |
| 23.45833 | | 279.1444 | | | | 3.649736 | | | | 4609.367 | | | 235.6094 | |  | |  | |  |
| 23.46667 | | 279.2384 | | | | 3.656607 | | | | 4607.344 | | | 236.8359 | |  | |  | |  |
| 23.475 | | 279.3309 | | | | 3.662634 | | | | 4605.281 | | | 238.5313 | |  | |  | |  |
| 23.48333 | | 279.4234 | | | | 3.67159 | | | | 4603.18 | | | 239.9063 | |  | |  | |  |
| 23.49167 | | 279.5143 | | | | 3.679439 | | | | 4601.063 | | | 241.5078 | |  | |  | |  |
| 23.5 | | 279.6091 | | | | 3.686478 | | | | 4598.945 | | | 242.9688 | |  | |  | |  |
| 23.50833 | | 279.7016 | | | | 3.695629 | | | | 4596.828 | | | 244.4766 | |  | |  | |  |
| 23.51667 | | 279.7925 | | | | 3.704549 | | | | 4594.719 | | | 246.0625 | |  | |  | |  |
| 23.525 | | 279.8866 | | | | 3.711598 | | | | 4592.531 | | | 247.3047 | |  | |  | |  |
| 23.53333 | | 279.979 | | | | 3.719583 | | | | 4590.367 | | | 248.6797 | |  | |  | |  |
| 23.54167 | | 280.0746 | | | | 3.728579 | | | | 4588.164 | | | 250.1641 | |  | |  | |  |
| 23.55 | | 280.1686 | | | | 3.736525 | | | | 4585.969 | | | 252.0313 | |  | |  | |  |
| 23.55833 | | 280.2619 | | | | 3.743534 | | | | 4583.75 | | | 253.5938 | |  | |  | |  |
| 23.56667 | | 280.3544 | | | | 3.753051 | | | | 4581.523 | | | 254.9219 | |  | |  | |  |
| 23.575 | | 280.4492 | | | | 3.761081 | | | | 4579.32 | | | 256.2813 | |  | |  | |  |
| 23.58333 | | 280.5424 | | | | 3.76723 | | | | 4577.094 | | | 257.8672 | |  | |  | |  |
| 23.59167 | | 280.6357 | | | | 3.775424 | | | | 4574.836 | | | 259.5547 | |  | |  | |  |
| 23.6 | | 280.7266 | | | | 3.78418 | | | | 4572.508 | | | 261.0781 | |  | |  | |  |
| 23.60833 | | 280.8191 | | | | 3.791841 | | | | 4570.219 | | | 262.6016 | |  | |  | |  |
| 23.61667 | | 280.9131 | | | | 3.799565 | | | | 4567.93 | | | 264.1016 | |  | |  | |  |
| 23.625 | | 281.0071 | | | | 3.808909 | | | | 4565.648 | | | 265.8125 | |  | |  | |  |
| 23.63333 | | 281.0988 | | | | 3.816451 | | | | 4563.305 | | | 267.1875 | |  | |  | |  |
| 23.64167 | | 281.1913 | | | | 3.823191 | | | | 4560.953 | | | 268.6172 | |  | |  | |  |
| 23.65 | | 281.2854 | | | | 3.832731 | | | | 4558.594 | | | 270.1328 | |  | |  | |  |
| 23.65833 | | 281.3794 | | | | 3.842063 | | | | 4556.234 | | | 271.8438 | |  | |  | |  |
| 23.66667 | | 281.4734 | | | | 3.847973 | | | | 4553.859 | | | 273.6484 | |  | |  | |  |
| 23.675 | | 281.5628 | | | | 3.857135 | | | | 4551.438 | | | 275.0469 | |  | |  | |  |
| 23.68333 | | 281.6561 | | | | 3.866355 | | | | 4549.063 | | | 276.7031 | |  | |  | |  |
| 23.69167 | | 281.7493 | | | | 3.873059 | | | | 4546.656 | | | 278.5625 | |  | |  | |  |
| 23.7 | | 281.8403 | | | | 3.881051 | | | | 4544.234 | | | 280.4453 | |  | |  | |  |
| 23.70833 | | 281.9312 | | | | 3.89039 | | | | 4541.766 | | | 282.0234 | |  | |  | |  |
| 23.71667 | | 282.0229 | | | | 3.898443 | | | | 4539.273 | | | 283.6406 | |  | |  | |  |
| 23.725 | | 282.1139 | | | | 3.906071 | | | | 4536.813 | | | 285.4375 | |  | |  | |  |
| 23.73333 | | 282.2044 | | | | 3.915289 | | | | 4534.32 | | | 287.5391 | |  | |  | |  |
| 23.74167 | | 282.2953 | | | | 3.922604 | | | | 4531.781 | | | 289.3359 | |  | |  | |  |
| 23.75 | | 282.3853 | | | | 3.92928 | | | | 4529.219 | | | 290.9922 | |  | |  | |  |
| 23.75833 | | 282.4801 | | | | 3.938329 | | | | 4526.688 | | | 292.7188 | |  | |  | |  |
| 23.76667 | | 282.5756 | | | | 3.947551 | | | | 4524.141 | | | 294.4531 | |  | |  | |  |
| 23.775 | | 282.6688 | | | | 3.953819 | | | | 4521.547 | | | 296.5469 | |  | |  | |  |
| 23.78333 | | 282.7635 | | | | 3.961963 | | | | 4518.906 | | | 298.3594 | |  | |  | |  |
| 23.79167 | | 282.856 | | | | 3.971682 | | | | 4516.289 | | | 299.9844 | |  | |  | |  |
| 23.8 | | 282.9499 | | | | 3.978456 | | | | 4513.672 | | | 301.6719 | |  | |  | |  |
| 23.80833 | | 283.0462 | | | | 3.986034 | | | | 4511.039 | | | 303.7813 | |  | |  | |  |
| 23.81667 | | 283.141 | | | | 3.99516 | | | | 4508.383 | | | 305.6953 | |  | |  | |  |
| 23.825 | | 283.2373 | | | | 4.004313 | | | | 4505.664 | | | 307.5547 | |  | |  | |  |
| 23.83333 | | 283.332 | | | | 4.01166 | | | | 4502.977 | | | 309.25 | |  | |  | |  |
| 23.84167 | | 283.4275 | | | | 4.019997 | | | | 4500.281 | | | 311.1953 | |  | |  | |  |
| 23.85 | | 283.52 | | | | 4.030048 | | | | 4497.578 | | | 313.3594 | |  | |  | |  |
| 23.85833 | | 283.6124 | | | | 4.036956 | | | | 4494.797 | | | 315.1016 | |  | |  | |  |
| 23.86667 | | 283.7056 | | | | 4.045314 | | | | 4492.031 | | | 317.1016 | |  | |  | |  |
| 23.875 | | 283.8018 | | | | 4.05499 | | | | 4489.25 | | | 318.9375 | |  | |  | |  |
| 23.88333 | | 283.8981 | | | | 4.063797 | | | | 4486.477 | | | 321.375 | |  | |  | |  |
| 23.89167 | | 283.9906 | | | | 4.071811 | | | | 4483.648 | | | 323.2188 | |  | |  | |  |
| 23.9 | | 284.1063 | | | | 4.082431 | | | | 4480.227 | | | 325.1797 | |  | |  | |  |
| 23.90833 | | 284.2002 | | | | 4.09265 | | | | 4477.375 | | | 327.0625 | |  | |  | |  |
| 23.91667 | | 284.2779 | | | | 4.099038 | | | | 4475.086 | | | 328.9453 | |  | |  | |  |
| 23.925 | | 284.3719 | | | | 4.107237 | | | | 4472.211 | | | 331.0313 | |  | |  | |  |
| 23.93333 | | 284.4674 | | | | 4.11782 | | | | 4469.242 | | | 333.0469 | |  | |  | |  |
| 23.94167 | | 284.5637 | | | | 4.126481 | | | | 4466.352 | | | 334.9844 | |  | |  | |  |
| 23.95 | | 284.6584 | | | | 4.133805 | | | | 4463.461 | | | 336.9609 | |  | |  | |  |
| 23.95833 | | 284.7501 | | | | 4.142506 | | | | 4460.523 | | | 339.2813 | |  | |  | |  |
| 23.96667 | | 284.8433 | | | | 4.151178 | | | | 4457.508 | | | 341.2109 | |  | |  | |  |
| 23.975 | | 284.9388 | | | | 4.158338 | | | | 4454.508 | | | 343.1172 | |  | |  | |  |
| 23.98333 | | 285.0327 | | | | 4.165487 | | | | 4451.5 | | | 345.2344 | |  | |  | |  |
| 23.99167 | | 285.1275 | | | | 4.175804 | | | | 4448.492 | | | 347.5234 | |  | |  | |  |
| 24 | | 285.2408 | | | | 4.186092 | | | | 4444.828 | | | 350.0391 | |  | |  | |  |
| 24.00833 | | 285.317 | | | | 4.191615 | | | | 4442.352 | | | 351.8203 | |  | |  | |  |
| 24.01667 | | 285.4117 | | | | 4.201338 | | | | 4439.289 | | | 353.8984 | |  | |  | |  |
| 24.025 | | 285.5235 | | | | 4.212595 | | | | 4435.586 | | | 356.7891 | |  | |  | |  |
| 24.03333 | | 285.6222 | | | | 4.221542 | | | | 4432.484 | | | 359.2578 | |  | |  | |  |
| 24.04167 | | 285.6998 | | | | 4.227672 | | | | 4429.93 | | | 361.1563 | |  | |  | |  |
| 24.05 | | 285.8132 | | | | 4.239344 | | | | 4426.148 | | | 364 | |  | |  | |  |
| 24.05833 | | 285.8916 | | | | 4.246737 | | | | 4423.586 | | | 365.8828 | |  | |  | |  |
| 24.06667 | | 285.9864 | | | | 4.254641 | | | | 4420.398 | | | 368.625 | |  | |  | |  |
| 24.075 | | 286.0819 | | | | 4.264295 | | | | 4417.164 | | | 370.8516 | |  | |  | |  |
| 24.08333 | | 286.1937 | | | | 4.275097 | | | | 4413.211 | | | 373.7969 | |  | |  | |  |
| 24.09167 | | 286.2683 | | | | 4.28078 | | | | 4410.57 | | | 375.4609 | |  | |  | |  |
| 24.1 | | 286.3622 | | | | 4.289608 | | | | 4407.258 | | | 377.9141 | |  | |  | |  |
| 24.10833 | | 286.4578 | | | | 4.30038 | | | | 4403.938 | | | 380.3984 | |  | |  | |  |
| 24.11667 | | 286.5696 | | | | 4.311543 | | | | 4399.867 | | | 383.3594 | |  | |  | |  |
| 24.125 | | 286.6426 | | | | 4.31726 | | | | 4397.195 | | | 385.2031 | |  | |  | |  |
| 24.13333 | | 286.7552 | | | | 4.328853 | | | | 4393.117 | | | 387.9844 | |  | |  | |  |
| 24.14167 | | 286.8282 | | | | 4.338156 | | | | 4390.422 | | | 390.1641 | |  | |  | |  |
| 24.15 | | 286.9416 | | | | 4.349107 | | | | 4386.281 | | | 393.1641 | |  | |  | |  |
| 24.15833 | | 287.0169 | | | | 4.355776 | | | | 4383.523 | | | 395.2422 | |  | |  | |  |
| 24.16667 | | 287.1124 | | | | 4.365622 | | | | 4380.031 | | | 397.7969 | |  | |  | |  |
| 24.175 | | 287.2281 | | | | 4.37698 | | | | 4375.852 | | | 400.9609 | |  | |  | |  |
| 24.18333 | | 287.3058 | | | | 4.382891 | | | | 4373.086 | | | 403.125 | |  | |  | |  |
| 24.19167 | | 287.3997 | | | | 4.392604 | | | | 4369.508 | | | 405.7578 | |  | |  | |  |
| 24.2 | | 287.4914 | | | | 4.402426 | | | | 4365.953 | | | 408.1797 | |  | |  | |  |
| 24.20833 | | 287.6079 | | | | 4.413718 | | | | 4361.617 | | | 411.5547 | |  | |  | |  |
| 24.21667 | | 287.7057 | | | | 4.422207 | | | | 4358.031 | | | 414.7188 | |  | |  | |  |
| 24.225 | | 287.802 | | | | 4.432897 | | | | 4354.359 | | | 417.3125 | |  | |  | |  |
| 24.23333 | | 287.8944 | | | | 4.442698 | | | | 4350.68 | | | 419.875 | |  | |  | |  |
| 24.24167 | | 287.9908 | | | | 4.450895 | | | | 4346.984 | | | 422.7188 | |  | |  | |  |
| 24.25 | | 288.0653 | | | | 4.459079 | | | | 4344.055 | | | 425.3125 | |  | |  | |  |
| 24.25833 | | 288.1608 | | | | 4.469963 | | | | 4340.305 | | | 428.3984 | |  | |  | |  |
| 24.26667 | | 288.2548 | | | | 4.477892 | | | | 4336.461 | | | 431.1719 | |  | |  | |  |
| 24.275 | | 288.372 | | | | 4.490197 | | | | 4331.922 | | | 434.2813 | |  | |  | |  |
| 24.28333 | | 288.4528 | | | | 4.500034 | | | | 4328.875 | | | 436.6406 | |  | |  | |  |
| 24.29167 | | 288.5662 | | | | 4.513225 | | | | 4324.281 | | | 440.3203 | |  | |  | |  |
| 24.3 | | 288.6415 | | | | 4.519403 | | | | 4321.133 | | | 442.5938 | |  | |  | |  |
| 24.30833 | | 288.7363 | | | | 4.530769 | | | | 4317.203 | | | 445.5781 | |  | |  | |  |
| 24.31667 | | 288.8349 | | | | 4.542954 | | | | 4313.289 | | | 448.6953 | |  | |  | |  |
| 24.325 | | 288.9483 | | | | 4.555334 | | | | 4308.586 | | | 452.8594 | |  | |  | |  |
| 24.33333 | | 289.0251 | | | | 4.563302 | | | | 4305.398 | | | 455.2266 | |  | |  | |  |
| 24.34167 | | 289.1207 | | | | 4.575173 | | | | 4301.367 | | | 458.1094 | |  | |  | |  |
| 24.35 | | 289.2364 | | | | 4.588033 | | | | 4296.547 | | | 461.8047 | |  | |  | |  |
| 24.35833 | | 289.3102 | | | | 4.594395 | | | | 4293.289 | | | 464.4922 | |  | |  | |  |
| 24.36667 | | 289.4236 | | | | 4.606884 | | | | 4288.352 | | | 468.6406 | |  | |  | |  |
| 24.375 | | 289.4981 | | | | 4.616418 | | | | 4285.016 | | | 471.0703 | |  | |  | |  |
| 24.38333 | | 289.5921 | | | | 4.624914 | | | | 4280.875 | | | 474.0938 | |  | |  | |  |
| 24.39167 | | 289.6907 | | | | 4.63595 | | | | 4276.719 | | | 476.8672 | |  | |  | |  |
| 24.4 | | 289.8072 | | | | 4.649905 | | | | 4271.672 | | | 480.7891 | |  | |  | |  |
| 24.40833 | | 289.8841 | | | | 4.658115 | | | | 4268.258 | | | 483.3594 | |  | |  | |  |
| 24.41667 | | 289.9796 | | | | 4.668692 | | | | 4263.93 | | | 486.5625 | |  | |  | |  |
| 24.425 | | 290.0743 | | | | 4.680753 | | | | 4259.656 | | | 489.8828 | |  | |  | |  |
| 24.43333 | | 290.1714 | | | | 4.692744 | | | | 4255.359 | | | 493.2656 | |  | |  | |  |
| 24.44167 | | 290.2902 | | | | 4.706196 | | | | 4250.195 | | | 497.7656 | |  | |  | |  |
| 24.45 | | 290.3849 | | | | 4.716779 | | | | 4245.813 | | | 500.8438 | |  | |  | |  |
| 24.45833 | | 290.4836 | | | | 4.729037 | | | | 4241.414 | | | 504.0859 | |  | |  | |  |
| 24.46667 | | 290.5822 | | | | 4.742153 | | | | 4236.961 | | | 507.5859 | |  | |  | |  |
| 24.475 | | 290.6569 | | | | 4.75012 | | | | 4233.398 | | | 511.1094 | |  | |  | |  |
| 24.48333 | | 290.7682 | | | | 4.7646 | | | | 4227.961 | | | 515.3516 | |  | |  | |  |
| 24.49167 | | 290.8642 | | | | 4.778423 | | | | 4223.383 | | | 518.6406 | |  | |  | |  |
| 24.5 | | 290.9633 | | | | 4.790508 | | | | 4218.859 | | | 521.9766 | |  | |  | |  |
| 24.50833 | | 291.0408 | | | | 4.799245 | | | | 4215.195 | | | 525.2422 | |  | |  | |  |
| 24.51667 | | 291.1353 | | | | 4.812317 | | | | 4210.586 | | | 528.9922 | |  | |  | |  |
| 24.525 | | 291.2458 | | | | 4.82761 | | | | 4204.883 | | | 533.0625 | |  | |  | |  |
| 24.53333 | | 291.3226 | | | | 4.835377 | | | | 4201.117 | | | 536.0234 | |  | |  | |  |
| 24.54167 | | 291.4185 | | | | 4.847359 | | | | 4196.406 | | | 539.8281 | |  | |  | |  |
| 24.55 | | 291.5145 | | | | 4.861376 | | | | 4191.695 | | | 544.3047 | |  | |  | |  |
| 24.55833 | | 291.6089 | | | | 4.872627 | | | | 4186.844 | | | 547.75 | |  | |  | |  |
| 24.56667 | | 291.718 | | | | 4.887503 | | | | 4181.047 | | | 552.2109 | |  | |  | |  |
| 24.575 | | 291.7924 | | | | 4.898918 | | | | 4177.172 | | | 555.0156 | |  | |  | |  |
| 24.58333 | | 291.9045 | | | | 4.915349 | | | | 4171.313 | | | 560.3281 | |  | |  | |  |
| 24.59167 | | 291.979 | | | | 4.924298 | | | | 4167.344 | | | 563.2188 | |  | |  | |  |
| 24.6 | | 292.0919 | | | | 4.939059 | | | | 4161.281 | | | 567.8438 | |  | |  | |  |
| 24.60833 | | 292.1702 | | | | 4.951427 | | | | 4157.32 | | | 570.8125 | |  | |  | |  |
| 24.61667 | | 292.2685 | | | | 4.964458 | | | | 4152.289 | | | 574.7422 | |  | |  | |  |
| 24.625 | | 292.3621 | | | | 4.976317 | | | | 4147.258 | | | 579.0625 | |  | |  | |  |
| 24.63333 | | 292.4727 | | | | 4.993865 | | | | 4141.023 | | | 583.25 | |  | |  | |  |
| 24.64167 | | 292.5487 | | | | 5.005531 | | | | 4136.938 | | | 586.2969 | |  | |  | |  |
| 24.65 | | 292.6462 | | | | 5.018799 | | | | 4131.758 | | | 590.3516 | |  | |  | |  |
| 24.65833 | | 292.7637 | | | | 5.034808 | | | | 4125.555 | | | 595.7813 | |  | |  | |  |
| 24.66667 | | 292.8589 | | | | 5.050256 | | | | 4120.25 | | | 599.5859 | |  | |  | |  |
| 24.675 | | 292.9556 | | | | 5.065047 | | | | 4114.953 | | | 603.6328 | |  | |  | |  |
| 24.68333 | | 293.0516 | | | | 5.078501 | | | | 4109.695 | | | 607.7031 | |  | |  | |  |
| 24.69167 | | 293.1483 | | | | 5.092418 | | | | 4104.344 | | | 612.5313 | |  | |  | |  |
| 24.7 | | 293.2251 | | | | 5.106092 | | | | 4100.055 | | | 615.9688 | |  | |  | |  |
| 24.70833 | | 293.3234 | | | | 5.121085 | | | | 4094.531 | | | 620.0625 | |  | |  | |  |
| 24.71667 | | 293.4408 | | | | 5.138476 | | | | 4088.016 | | | 625.0938 | |  | |  | |  |
| 24.725 | | 293.5191 | | | | 5.150821 | | | | 4083.602 | | | 628.6641 | |  | |  | |  |
| 24.73333 | | 293.6174 | | | | 5.16673 | | | | 4078.094 | | | 633.3828 | |  | |  | |  |
| 24.74167 | | 293.7318 | | | | 5.185445 | | | | 4071.313 | | | 638.625 | |  | |  | |  |
| 24.75 | | 293.8308 | | | | 5.199701 | | | | 4065.672 | | | 643.0625 | |  | |  | |  |
| 24.75833 | | 293.9115 | | | | 5.213259 | | | | 4061.156 | | | 646.3516 | |  | |  | |  |
| 24.76667 | | 294.0097 | | | | 5.228823 | | | | 4055.477 | | | 651.1875 | |  | |  | |  |
| 24.775 | | 294.1264 | | | | 5.247304 | | | | 4048.57 | | | 656.8281 | |  | |  | |  |
| 24.78333 | | 294.2224 | | | | 5.262384 | | | | 4042.719 | | | 661.1797 | |  | |  | |  |
| 24.79167 | | 294.2969 | | | | 5.276608 | | | | 4038.07 | | | 664.4453 | |  | |  | |  |
| 24.8 | | 294.4174 | | | | 5.296654 | | | | 4031.031 | | | 670.1875 | |  | |  | |  |
| 24.80833 | | 294.4957 | | | | 5.30851 | | | | 4026.344 | | | 673.9453 | |  | |  | |  |
| 24.81667 | | 294.614 | | | | 5.329895 | | | | 4019.141 | | | 679.1016 | |  | |  | |  |
| 24.825 | | 294.7115 | | | | 5.348593 | | | | 4013.125 | | | 683.1563 | |  | |  | |  |
| 24.83333 | | 294.8082 | | | | 5.366968 | | | | 4007.125 | | | 687.3203 | |  | |  | |  |
| 24.84167 | | 294.8842 | | | | 5.380007 | | | | 4002.313 | | | 691.3203 | |  | |  | |  |
| 24.85 | | 294.9817 | | | | 5.399477 | | | | 3996.211 | | | 695.875 | |  | |  | |  |
| 24.85833 | | 295.0977 | | | | 5.420932 | | | | 3988.781 | | | 700.6797 | |  | |  | |  |
| 24.86667 | | 295.1767 | | | | 5.433952 | | | | 3983.875 | | | 704.1563 | |  | |  | |  |
| 24.875 | | 295.2996 | | | | 5.455346 | | | | 3976.492 | | | 709.7734 | |  | |  | |  |
| 24.88333 | | 295.3955 | | | | 5.474811 | | | | 3970.289 | | | 714.2266 | |  | |  | |  |
| 24.89167 | | 295.4946 | | | | 5.494365 | | | | 3963.922 | | | 718.3984 | |  | |  | |  |
| 24.9 | | 295.5729 | | | | 5.507923 | | | | 3958.898 | | | 721.8672 | |  | |  | |  |
| 24.90833 | | 295.6935 | | | | 5.530911 | | | | 3951.352 | | | 727.1563 | |  | |  | |  |
| 24.91667 | | 295.7733 | | | | 5.547889 | | | | 3946.258 | | | 731.0703 | |  | |  | |  |
| 24.925 | | 295.8708 | | | | 5.566338 | | | | 3939.828 | | | 735.5391 | |  | |  | |  |
| 24.93333 | | 295.9729 | | | | 5.583534 | | | | 3933.359 | | | 739.625 | |  | |  | |  |
| 24.94167 | | 296.075 | | | | 5.603255 | | | | 3926.891 | | | 743.9219 | |  | |  | |  |
| 24.95 | | 296.1956 | | | | 5.627707 | | | | 3919.094 | | | 749.4531 | |  | |  | |  |
| 24.95833 | | 296.2923 | | | | 5.64902 | | | | 3912.531 | | | 754.0625 | |  | |  | |  |
| 24.96667 | | 296.3906 | | | | 5.668361 | | | | 3905.852 | | | 758.3125 | |  | |  | |  |
| 24.975 | | 296.4689 | | | | 5.686159 | | | | 3900.57 | | | 761.3203 | |  | |  | |  |
| 24.98333 | | 296.5695 | | | | 5.708779 | | | | 3893.93 | | | 765.3359 | |  | |  | |  |
| 24.99167 | | 296.6892 | | | | 5.735248 | | | | 3885.914 | | | 770.7578 | |  | |  | |  |
| 25 | | 296.7691 | | | | 5.75067 | | | | 3880.492 | | | 774.0859 | |  | |  | |  |
| 25.00833 | | 296.8889 | | | | 5.775943 | | | | 3872.32 | | | 778.5 | |  | |  | |  |
| 25.01667 | | 296.9656 | | | | 5.794354 | | | | 3866.883 | | | 781.4844 | |  | |  | |  |
| 25.025 | | 297.0654 | | | | 5.815967 | | | | 3860.117 | | | 786.0078 | |  | |  | |  |
| 25.03333 | | 297.1637 | | | | 5.836784 | | | | 3853.313 | | | 789.8203 | |  | |  | |  |
| 25.04167 | | 297.2635 | | | | 5.85871 | | | | 3846.336 | | | 793.3672 | |  | |  | |  |
| 25.05 | | 297.3848 | | | | 5.88647 | | | | 3838.086 | | | 797.25 | |  | |  | |  |
| 25.05833 | | 297.4654 | | | | 5.905509 | | | | 3832.555 | | | 800.1875 | |  | |  | |  |
| 25.06667 | | 297.5829 | | | | 5.932497 | | | | 3824.242 | | | 804.9063 | |  | |  | |  |
| 25.075 | | 297.6827 | | | | 5.955626 | | | | 3817.188 | | | 808.1016 | |  | |  | |  |
| 25.08333 | | 297.7802 | | | | 5.979713 | | | | 3810.195 | | | 810.9922 | |  | |  | |  |
| 25.09167 | | 297.8608 | | | | 5.999371 | | | | 3804.586 | | | 813.25 | |  | |  | |  |
| 25.1 | | 297.9829 | | | | 6.027885 | | | | 3796.219 | | | 817.5078 | |  | |  | |  |
| 25.10833 | | 298.0605 | | | | 6.046539 | | | | 3790.563 | | | 819.6875 | |  | |  | |  |
| 25.11667 | | 298.1602 | | | | 6.071677 | | | | 3783.398 | | | 822.3125 | |  | |  | |  |
| 25.125 | | 298.2624 | | | | 6.094654 | | | | 3776.336 | | | 824.625 | |  | |  | |  |
| 25.13333 | | 298.386 | | | | 6.123641 | | | | 3767.859 | | | 827.9844 | |  | |  | |  |
| 25.14167 | | 298.4666 | | | | 6.142035 | | | | 3762.211 | | | 830.3203 | |  | |  | |  |
| 25.15 | | 298.571 | | | | 6.1679 | | | | 3754.969 | | | 832.3047 | |  | |  | |  |
| 25.15833 | | 298.6923 | | | | 6.196708 | | | | 3746.453 | | | 834.5547 | |  | |  | |  |
| 25.16667 | | 298.796 | | | | 6.219951 | | | | 3739.352 | | | 836.6094 | |  | |  | |  |
| 25.175 | | 298.8773 | | | | 6.237751 | | | | 3733.664 | | | 838.8125 | |  | |  | |  |
| 25.18333 | | 298.9787 | | | | 6.263514 | | | | 3726.477 | | | 840.2266 | |  | |  | |  |
| 25.19167 | | 299.079 | | | | 6.287647 | | | | 3719.258 | | | 841.5703 | |  | |  | |  |
| 25.2 | | 299.1799 | | | | 6.311575 | | | | 3712.141 | | | 842.6953 | |  | |  | |  |
| 25.20833 | | 299.2982 | | | | 6.341256 | | | | 3703.586 | | | 844.6797 | |  | |  | |  |
| 25.21667 | | 299.3968 | | | | 6.365737 | | | | 3696.43 | | | 845.7891 | |  | |  | |  |
| 25.225 | | 299.4757 | | | | 6.387811 | | | | 3690.594 | | | 846.1172 | |  | |  | |  |
| 25.23333 | | 299.5743 | | | | 6.412188 | | | | 3683.5 | | | 846.7031 | |  | |  | |  |
| 25.24167 | | 299.6744 | | | | 6.435525 | | | | 3676.375 | | | 846.9609 | |  | |  | |  |
| 25.25 | | 299.7746 | | | | 6.46127 | | | | 3669.281 | | | 847.6797 | |  | |  | |  |
| 25.25833 | | 299.8739 | | | | 6.485922 | | | | 3662.102 | | | 846.9219 | |  | |  | |  |
| 25.26667 | | 299.9937 | | | | 6.51474 | | | | 3653.547 | | | 846.3672 | |  | |  | |  |
| 25.275 | | 300.0954 | | | | 6.537422 | | | | 3646.492 | | | 845.5391 | |  | |  | |  |
| 25.28333 | | 300.197 | | | | 6.561024 | | | | 3639.453 | | | 845.3203 | |  | |  | |  |
| 25.29167 | | 300.2979 | | | | 6.587225 | | | | 3632.398 | | | 844.0938 | |  | |  | |  |
| 25.3 | | 300.376 | | | | 6.606875 | | | | 3626.727 | | | 842.8828 | |  | |  | |  |
| 25.30833 | | 300.4784 | | | | 6.628777 | | | | 3619.836 | | | 841.0938 | |  | |  | |  |
| 25.31667 | | 300.6028 | | | | 6.656698 | | | | 3611.516 | | | 839.0859 | |  | |  | |  |
| 25.325 | | 300.7022 | | | | 6.681143 | | | | 3604.633 | | | 837.5313 | |  | |  | |  |
| 25.33333 | | 300.7841 | | | | 6.700018 | | | | 3599.07 | | | 835.6406 | |  | |  | |  |
| 25.34167 | | 300.8857 | | | | 6.722098 | | | | 3592.211 | | | 832.9688 | |  | |  | |  |
| 25.35 | | 301.0071 | | | | 6.749055 | | | | 3584.086 | | | 829.4453 | |  | |  | |  |
| 25.35833 | | 301.0852 | | | | 6.768825 | | | | 3578.719 | | | 827.7969 | |  | |  | |  |
| 25.36667 | | 301.1998 | | | | 6.795446 | | | | 3570.625 | | | 824.0859 | |  | |  | |  |
| 25.375 | | 301.2771 | | | | 6.811098 | | | | 3565.227 | | | 821.3203 | |  | |  | |  |
| 25.38333 | | 301.3962 | | | | 6.836351 | | | | 3557.297 | | | 816.9531 | |  | |  | |  |
| 25.39167 | | 301.4948 | | | | 6.858632 | | | | 3550.742 | | | 813.5703 | |  | |  | |  |
| 25.4 | | 301.5934 | | | | 6.880151 | | | | 3544.258 | | | 809.8906 | |  | |  | |  |
| 25.40833 | | 301.67 | | | | 6.894812 | | | | 3538.977 | | | 806.4922 | |  | |  | |  |
| 25.41667 | | 301.7823 | | | | 6.917966 | | | | 3531.258 | | | 801.3516 | |  | |  | |  |
| 25.425 | | 301.8794 | | | | 6.93772 | | | | 3524.883 | | | 797.0234 | |  | |  | |  |
| 25.43333 | | 301.9575 | | | | 6.953363 | | | | 3519.836 | | | 794.0703 | |  | |  | |  |
| 25.44167 | | 302.0561 | | | | 6.969785 | | | | 3513.523 | | | 789.3359 | |  | |  | |  |
| 25.45 | | 302.1547 | | | | 6.988357 | | | | 3507.242 | | | 784.1563 | |  | |  | |  |
| 25.45833 | | 302.2533 | | | | 7.007204 | | | | 3501.07 | | | 779.0625 | |  | |  | |  |
| 25.46667 | | 302.3678 | | | | 7.027783 | | | | 3493.734 | | | 773.4922 | |  | |  | |  |
| 25.475 | | 302.4626 | | | | 7.042142 | | | | 3487.648 | | | 768.4063 | |  | |  | |  |
| 25.48333 | | 302.5362 | | | | 7.056038 | | | | 3482.742 | | | 763.9375 | |  | |  | |  |
| 25.49167 | | 302.6333 | | | | 7.071017 | | | | 3476.781 | | | 758.0469 | |  | |  | |  |
| 25.5 | | 302.7509 | | | | 7.088046 | | | | 3469.727 | | | 751.4063 | |  | |  | |  |
| 25.50833 | | 302.8472 | | | | 7.10217 | | | | 3463.906 | | | 746.1563 | |  | |  | |  |
| 25.51667 | | 302.942 | | | | 7.116946 | | | | 3458.008 | | | 740.0156 | |  | |  | |  |
| 25.525 | | 303.0368 | | | | 7.131187 | | | | 3452.258 | | | 733.8594 | |  | |  | |  |
| 25.53333 | | 303.1119 | | | | 7.140364 | | | | 3447.719 | | | 729.0391 | |  | |  | |  |
| 25.54167 | | 303.2265 | | | | 7.155422 | | | | 3441.016 | | | 722.0547 | |  | |  | |  |
| 25.55 | | 303.3053 | | | | 7.165987 | | | | 3436.555 | | | 716.7891 | |  | |  | |  |
| 25.55833 | | 303.3986 | | | | 7.175769 | | | | 3430.953 | | | 710.1328 | |  | |  | |  |
| 25.56667 | | 303.5132 | | | | 7.188674 | | | | 3424.445 | | | 702.5313 | |  | |  | |  |
| 25.575 | | 303.6087 | | | | 7.199466 | | | | 3419.07 | | | 696.2891 | |  | |  | |  |
| 25.58333 | | 303.7028 | | | | 7.209328 | | | | 3413.734 | | | 690.3047 | |  | |  | |  |
| 25.59167 | | 303.7961 | | | | 7.217227 | | | | 3408.406 | | | 683.6094 | |  | |  | |  |
| 25.6 | | 303.8917 | | | | 7.226322 | | | | 3403.25 | | | 676.9609 | |  | |  | |  |
| 25.60833 | | 303.9887 | | | | 7.23577 | | | | 3398.125 | | | 670.3125 | |  | |  | |  |
| 25.61667 | | 304.0858 | | | | 7.241643 | | | | 3393.023 | | | 663.9375 | |  | |  | |  |
| 25.625 | | 304.1799 | | | | 7.248205 | | | | 3387.953 | | | 657.4609 | |  | |  | |  |
| 25.63333 | | 304.2755 | | | | 7.255806 | | | | 3382.898 | | | 650.8516 | |  | |  | |  |
| 25.64167 | | 304.3703 | | | | 7.262512 | | | | 3377.992 | | | 644.2813 | |  | |  | |  |
| 25.65 | | 304.4613 | | | | 7.267154 | | | | 3373.133 | | | 637.4609 | |  | |  | |  |
| 25.65833 | | 304.5538 | | | | 7.272919 | | | | 3368.336 | | | 630.9922 | |  | |  | |  |
| 25.66667 | | 304.6464 | | | | 7.279697 | | | | 3363.531 | | | 624.0391 | |  | |  | |  |
| 25.675 | | 304.7389 | | | | 7.282756 | | | | 3358.805 | | | 617.0078 | |  | |  | |  |
| 25.68333 | | 304.8292 | | | | 7.286584 | | | | 3354.141 | | | 610.2578 | |  | |  | |  |
| 25.69167 | | 304.9186 | | | | 7.291819 | | | | 3349.539 | | | 603.7109 | |  | |  | |  |
| 25.7 | | 305.0044 | | | | 7.293534 | | | | 3345.016 | | | 596.9375 | |  | |  | |  |
| 25.70833 | | 305.0969 | | | | 7.296379 | | | | 3340.5 | | | 590.1328 | |  | |  | |  |
| 25.71667 | | 305.1849 | | | | 7.300007 | | | | 3336.102 | | | 583.1953 | |  | |  | |  |
| 25.725 | | 305.2774 | | | | 7.300644 | | | | 3331.773 | | | 576.6563 | |  | |  | |  |
| 25.73333 | | 305.3707 | | | | 7.301465 | | | | 3327.461 | | | 570.6797 | |  | |  | |  |
| 25.74167 | | 305.461 | | | | 7.304762 | | | | 3323.18 | | | 564.0938 | |  | |  | |  |
| 25.75 | | 305.5535 | | | | 7.304522 | | | | 3318.984 | | | 557.6641 | |  | |  | |  |
| 25.75833 | | 305.643 | | | | 7.303853 | | | | 3314.828 | | | 551.5 | |  | |  | |  |
| 25.76667 | | 305.7348 | | | | 7.306271 | | | | 3310.773 | | | 545.7109 | |  | |  | |  |
| 25.775 | | 305.8258 | | | | 7.305573 | | | | 3306.703 | | | 539.8828 | |  | |  | |  |
| 25.78333 | | 305.9206 | | | | 7.303742 | | | | 3302.617 | | | 533.7656 | |  | |  | |  |
| 25.79167 | | 306.0101 | | | | 7.304297 | | | | 3298.656 | | | 527.6875 | |  | |  | |  |
| 25.8 | | 306.1034 | | | | 7.302718 | | | | 3294.727 | | | 522.0547 | |  | |  | |  |
| 25.80833 | | 306.1937 | | | | 7.300088 | | | | 3290.828 | | | 516.3516 | |  | |  | |  |
| 25.81667 | | 306.2855 | | | | 7.298496 | | | | 3286.914 | | | 510.3047 | |  | |  | |  |
| 25.825 | | 306.3772 | | | | 7.297908 | | | | 3283.047 | | | 504.4141 | |  | |  | |  |
| 25.83333 | | 306.4652 | | | | 7.294731 | | | | 3279.266 | | | 498.5156 | |  | |  | |  |
| 25.84167 | | 306.5562 | | | | 7.291776 | | | | 3275.555 | | | 493.2734 | |  | |  | |  |
| 25.85 | | 306.6465 | | | | 7.291288 | | | | 3271.813 | | | 487.625 | |  | |  | |  |
| 25.85833 | | 306.7353 | | | | 7.288168 | | | | 3268.148 | | | 482.0234 | |  | |  | |  |
| 25.86667 | | 306.824 | | | | 7.283915 | | | | 3264.555 | | | 476.1875 | |  | |  | |  |
| 25.875 | | 306.9135 | | | | 7.282708 | | | | 3261 | | | 470.8984 | |  | |  | |  |
| 25.88333 | | 307.0007 | | | | 7.279031 | | | | 3257.5 | | | 465.9141 | |  | |  | |  |
| 25.89167 | | 307.0872 | | | | 7.274238 | | | | 3253.961 | | | 460.75 | |  | |  | |  |
| 25.9 | | 307.1729 | | | | 7.271383 | | | | 3250.516 | | | 455.625 | |  | |  | |  |
| 25.90833 | | 307.2609 | | | | 7.26648 | | | | 3247.109 | | | 450.6563 | |  | |  | |  |
| 25.91667 | | 307.3504 | | | | 7.261429 | | | | 3243.797 | | | 446.0547 | |  | |  | |  |
| 25.925 | | 307.4399 | | | | 7.258767 | | | | 3240.438 | | | 440.8984 | |  | |  | |  |
| 25.93333 | | 307.5256 | | | | 7.253468 | | | | 3237.102 | | | 435.9531 | |  | |  | |  |
| 25.94167 | | 307.6143 | | | | 7.248229 | | | | 3233.813 | | | 431.1875 | |  | |  | |  |
| 25.95 | | 307.7039 | | | | 7.244185 | | | | 3230.578 | | | 427.0781 | |  | |  | |  |
| 25.95833 | | 307.7933 | | | | 7.237791 | | | | 3227.367 | | | 422.8672 | |  | |  | |  |
| 25.96667 | | 307.8813 | | | | 7.232121 | | | | 3224.133 | | | 418.2578 | |  | |  | |  |
| 25.975 | | 307.9708 | | | | 7.227368 | | | | 3221.023 | | | 413.5234 | |  | |  | |  |
| 25.98333 | | 308.0626 | | | | 7.22078 | | | | 3217.922 | | | 409.2656 | |  | |  | |  |
| 25.99167 | | 308.1521 | | | | 7.214896 | | | | 3214.852 | | | 405.1406 | |  | |  | |  |
| 26 | | 308.2393 | | | | 7.210188 | | | | 3211.711 | | | 400.9063 | |  | |  | |  |
| 26.00833 | | 308.325 | | | | 7.203448 | | | | 3208.641 | | | 396.8438 | |  | |  | |  |
| 26.01667 | | 308.4153 | | | | 7.197372 | | | | 3205.648 | | | 392.7344 | |  | |  | |  |
| 26.025 | | 308.5078 | | | | 7.192299 | | | | 3202.719 | | | 388.9453 | |  | |  | |  |
| 26.03333 | | 308.5958 | | | | 7.185387 | | | | 3199.766 | | | 385.1719 | |  | |  | |  |
| 26.04167 | | 308.6823 | | | | 7.179128 | | | | 3196.828 | | | 381.0625 | |  | |  | |  |
| 26.05 | | 308.7695 | | | | 7.17406 | | | | 3193.945 | | | 377.0938 | |  | |  | |  |
| 26.05833 | | 308.859 | | | | 7.166834 | | | | 3191.078 | | | 373.4141 | |  | |  | |  |
| 26.06667 | | 308.9455 | | | | 7.159976 | | | | 3188.25 | | | 369.8906 | |  | |  | |  |
| 26.075 | | 309.035 | | | | 7.154758 | | | | 3185.414 | | | 366.1328 | |  | |  | |  |
| 26.08333 | | 309.1237 | | | | 7.146941 | | | | 3182.602 | | | 362.3125 | |  | |  | |  |
| 26.09167 | | 309.2148 | | | | 7.13987 | | | | 3179.883 | | | 358.7266 | |  | |  | |  |
| 26.1 | | 309.3027 | | | | 7.134259 | | | | 3177.172 | | | 355.3359 | |  | |  | |  |
| 26.10833 | | 309.3885 | | | | 7.126624 | | | | 3174.453 | | | 352.0156 | |  | |  | |  |
| 26.11667 | | 309.4757 | | | | 7.119791 | | | | 3171.766 | | | 348.5625 | |  | |  | |  |
| 26.125 | | 309.5637 | | | | 7.11472 | | | | 3169.125 | | | 345.0781 | |  | |  | |  |
| 26.13333 | | 309.6547 | | | | 7.106851 | | | | 3166.508 | | | 341.7656 | |  | |  | |  |
| 26.14167 | | 309.7411 | | | | 7.099519 | | | | 3163.922 | | | 338.8672 | |  | |  | |  |
| 26.15 | | 309.8299 | | | | 7.093766 | | | | 3161.32 | | | 335.7969 | |  | |  | |  |
| 26.15833 | | 309.9156 | | | | 7.085198 | | | | 3158.75 | | | 332.7813 | |  | |  | |  |
| 26.16667 | | 310.0051 | | | | 7.077815 | | | | 3156.211 | | | 329.7734 | |  | |  | |  |
| 26.175 | | 310.0923 | | | | 7.071728 | | | | 3153.711 | | | 327.0156 | |  | |  | |  |
| 26.18333 | | 310.1788 | | | | 7.06371 | | | | 3151.219 | | | 324.1641 | |  | |  | |  |
| 26.19167 | | 310.2653 | | | | 7.05644 | | | | 3148.695 | | | 321.0313 | |  | |  | |  |
| 26.2 | | 310.354 | | | | 7.051301 | | | | 3146.227 | | | 317.8672 | |  | |  | |  |
| 26.20833 | | 310.4443 | | | | 7.043044 | | | | 3143.773 | | | 315.5234 | |  | |  | |  |
| 26.21667 | | 310.5315 | | | | 7.035668 | | | | 3141.352 | | | 313.2109 | |  | |  | |  |
| 26.225 | | 310.6218 | | | | 7.030517 | | | | 3138.914 | | | 310.3906 | |  | |  | |  |
| 26.23333 | | 310.709 | | | | 7.022332 | | | | 3136.531 | | | 307.7344 | |  | |  | |  |
| 26.24167 | | 310.7993 | | | | 7.015006 | | | | 3134.195 | | | 305.2109 | |  | |  | |  |
| 26.25 | | 310.888 | | | | 7.009927 | | | | 3131.891 | | | 303.0078 | |  | |  | |  |
| 26.25833 | | 310.976 | | | | 7.001237 | | | | 3129.508 | | | 300.6484 | |  | |  | |  |
| 26.26667 | | 311.0647 | | | | 6.993594 | | | | 3127.141 | | | 298.1094 | |  | |  | |  |
| 26.275 | | 311.1542 | | | | 6.989121 | | | | 3124.852 | | | 295.7734 | |  | |  | |  |
| 26.28333 | | 311.2422 | | | | 6.980719 | | | | 3122.578 | | | 293.7813 | |  | |  | |  |
| 26.29167 | | 311.3279 | | | | 6.973012 | | | | 3120.305 | | | 291.5547 | |  | |  | |  |
| 26.3 | | 311.4167 | | | | 6.968188 | | | | 3118.008 | | | 289.0391 | |  | |  | |  |
| 26.30833 | | 311.5024 | | | | 6.959403 | | | | 3115.758 | | | 286.8125 | |  | |  | |  |
| 26.31667 | | 311.5904 | | | | 6.95195 | | | | 3113.539 | | | 284.6563 | |  | |  | |  |
| 26.325 | | 311.6783 | | | | 6.947273 | | | | 3111.328 | | | 282.5703 | |  | |  | |  |
| 26.33333 | | 311.7633 | | | | 6.938889 | | | | 3109.086 | | | 280.2344 | |  | |  | |  |
| 26.34167 | | 311.8513 | | | | 6.931872 | | | | 3106.891 | | | 277.8828 | |  | |  | |  |
| 26.35 | | 311.9385 | | | | 6.927688 | | | | 3104.75 | | | 276.0078 | |  | |  | |  |
| 26.35833 | | 312.0242 | | | | 6.919585 | | | | 3102.602 | | | 274.3125 | |  | |  | |  |
| 26.36667 | | 312.1122 | | | | 6.912957 | | | | 3100.461 | | | 272.4453 | |  | |  | |  |
| 26.375 | | 312.1979 | | | | 6.907397 | | | | 3098.328 | | | 270.5 | |  | |  | |  |
| 26.38333 | | 312.2829 | | | | 6.899625 | | | | 3096.258 | | | 268.5547 | |  | |  | |  |
| 26.39167 | | 312.3724 | | | | 6.893657 | | | | 3094.195 | | | 266.7656 | |  | |  | |  |
| 26.4 | | 312.4596 | | | | 6.887947 | | | | 3092.086 | | | 265.2109 | |  | |  | |  |
| 26.40833 | | 312.543 | | | | 6.880141 | | | | 3089.969 | | | 263.4375 | |  | |  | |  |
| 26.41667 | | 312.631 | | | | 6.874576 | | | | 3087.898 | | | 261.8203 | |  | |  | |  |
| 26.425 | | 312.7182 | | | | 6.868981 | | | | 3085.859 | | | 260.0391 | |  | |  | |  |
| 26.43333 | | 312.807 | | | | 6.861794 | | | | 3083.828 | | | 258.3594 | |  | |  | |  |
| 26.44167 | | 312.8957 | | | | 6.85761 | | | | 3081.789 | | | 256.3203 | |  | |  | |  |
| 26.45 | | 312.983 | | | | 6.851328 | | | | 3079.75 | | | 254.5391 | |  | |  | |  |
| 26.45833 | | 313.0724 | | | | 6.844103 | | | | 3077.742 | | | 252.7578 | |  | |  | |  |
| 26.46667 | | 313.1604 | | | | 6.83968 | | | | 3075.727 | | | 251.1797 | |  | |  | |  |
| 26.475 | | 313.2484 | | | | 6.83319 | | | | 3073.758 | | | 249.5938 | |  | |  | |  |
| 26.48333 | | 313.3387 | | | | 6.824713 | | | | 3071.789 | | | 247.8906 | |  | |  | |  |
| 26.49167 | | 313.4274 | | | | 6.819547 | | | | 3069.883 | | | 246.0703 | |  | |  | |  |
| 26.5 | | 313.5139 | | | | 6.813765 | | | | 3067.953 | | | 244.0156 | |  | |  | |  |
| 26.50833 | | 313.6019 | | | | 6.80529 | | | | 3066.055 | | | 242.6953 | |  | |  | |  |
| 26.51667 | | 313.6906 | | | | 6.801126 | | | | 3064.125 | | | 241.2266 | |  | |  | |  |
| 26.525 | | 313.7779 | | | | 6.795212 | | | | 3062.227 | | | 239.6797 | |  | |  | |  |
| 26.53333 | | 313.8651 | | | | 6.787708 | | | | 3060.352 | | | 237.9375 | |  | |  | |  |
| 26.54167 | | 313.9546 | | | | 6.783831 | | | | 3058.508 | | | 236.5313 | |  | |  | |  |
| 26.55 | | 314.0441 | | | | 6.778188 | | | | 3056.703 | | | 235.1406 | |  | |  | |  |
| 26.55833 | | 314.1298 | | | | 6.770905 | | | | 3054.82 | | | 233.6094 | |  | |  | |  |
| 26.56667 | | 314.2163 | | | | 6.767052 | | | | 3052.969 | | | 232.0234 | |  | |  | |  |
| 26.575 | | 314.305 | | | | 6.761107 | | | | 3051.141 | | | 230.7813 | |  | |  | |  |
| 26.58333 | | 314.3945 | | | | 6.754795 | | | | 3049.359 | | | 229.9375 | |  | |  | |  |
| 26.59167 | | 314.4818 | | | | 6.750773 | | | | 3047.539 | | | 228.7109 | |  | |  | |  |
| 26.6 | | 314.5674 | | | | 6.744349 | | | | 3045.734 | | | 227.1719 | |  | |  | |  |
| 26.60833 | | 314.6562 | | | | 6.738893 | | | | 3043.961 | | | 225.9766 | |  | |  | |  |
| 26.61667 | | 314.7465 | | | | 6.734726 | | | | 3042.188 | | | 224.8594 | |  | |  | |  |
| 26.625 | | 314.8329 | | | | 6.728569 | | | | 3040.398 | | | 223.6328 | |  | |  | |  |
| 26.63333 | | 314.9194 | | | | 6.722995 | | | | 3038.547 | | | 222.5078 | |  | |  | |  |
| 26.64167 | | 315.0081 | | | | 6.718556 | | | | 3036.781 | | | 221.2031 | |  | |  | |  |
| 26.65 | | 315.0969 | | | | 6.71239 | | | | 3035.07 | | | 220.0156 | |  | |  | |  |
| 26.65833 | | 315.1856 | | | | 6.707607 | | | | 3033.313 | | | 219.0469 | |  | |  | |  |
| 26.66667 | | 315.2736 | | | | 6.704056 | | | | 3031.539 | | | 217.6094 | |  | |  | |  |
| 26.675 | | 315.3616 | | | | 6.697631 | | | | 3029.813 | | | 216.2734 | |  | |  | |  |
| 26.68333 | | 315.4511 | | | | 6.693757 | | | | 3028.07 | | | 215.1953 | |  | |  | |  |
| 26.69167 | | 315.5391 | | | | 6.690317 | | | | 3026.375 | | | 214.1563 | |  | |  | |  |
| 26.7 | | 315.624 | | | | 6.683343 | | | | 3024.672 | | | 213.0078 | |  | |  | |  |
| 26.70833 | | 315.7094 | | | | 6.678978 | | | | 3022.953 | | | 212 | |  | |  | |  |
| 26.71667 | | 315.794 | | | | 6.674681 | | | | 3021.297 | | | 210.6406 | |  | |  | |  |
| 26.725 | | 315.8824 | | | | 6.6672 | | | | 3019.641 | | | 209.7813 | |  | |  | |  |
| 26.73333 | | 315.9679 | | | | 6.663608 | | | | 3017.969 | | | 208.8906 | |  | |  | |  |
| 26.74167 | | 316.0518 | | | | 6.660782 | | | | 3016.305 | | | 207.75 | |  | |  | |  |
| 26.75 | | 316.1387 | | | | 6.654089 | | | | 3014.641 | | | 206.6953 | |  | |  | |  |
| 26.75833 | | 316.2241 | | | | 6.650737 | | | | 3012.977 | | | 205.8594 | |  | |  | |  |
| 26.76667 | | 316.3118 | | | | 6.647875 | | | | 3011.375 | | | 204.9453 | |  | |  | |  |
| 26.775 | | 316.3987 | | | | 6.640779 | | | | 3009.719 | | | 204.0234 | |  | |  | |  |
| 26.78333 | | 316.4871 | | | | 6.636628 | | | | 3008.07 | | | 203.0313 | |  | |  | |  |
| 26.79167 | | 316.574 | | | | 6.63291 | | | | 3006.469 | | | 201.8438 | |  | |  | |  |
| 26.8 | | 316.6639 | | | | 6.626477 | | | | 3004.852 | | | 201.1016 | |  | |  | |  |
| 26.80833 | | 316.7508 | | | | 6.622846 | | | | 3003.219 | | | 200.0859 | |  | |  | |  |
| 26.81667 | | 316.8385 | | | | 6.619454 | | | | 3001.602 | | | 199.2266 | |  | |  | |  |
| 26.825 | | 316.9284 | | | | 6.613596 | | | | 3000 | | | 198.3047 | |  | |  | |  |
| 26.83333 | | 317.0168 | | | | 6.610825 | | | | 2998.422 | | | 197.2188 | |  | |  | |  |
| 26.84167 | | 317.1052 | | | | 6.608273 | | | | 2996.883 | | | 196.4141 | |  | |  | |  |
| 26.85 | | 317.1921 | | | | 6.603615 | | | | 2995.273 | | | 195.6797 | |  | |  | |  |
| 26.85833 | | 317.2775 | | | | 6.601029 | | | | 2993.719 | | | 194.7266 | |  | |  | |  |
| 26.86667 | | 317.3637 | | | | 6.598185 | | | | 2992.156 | | | 193.9531 | |  | |  | |  |
| 26.875 | | 317.4551 | | | | 6.593173 | | | | 2990.602 | | | 193.4688 | |  | |  | |  |
| 26.88333 | | 317.5435 | | | | 6.590473 | | | | 2989.094 | | | 192.7656 | |  | |  | |  |
| 26.89167 | | 317.6304 | | | | 6.587628 | | | | 2987.539 | | | 192 | |  | |  | |  |
| 26.9 | | 317.7158 | | | | 6.582635 | | | | 2985.992 | | | 191.1719 | |  | |  | |  |
| 26.90833 | | 317.8035 | | | | 6.58018 | | | | 2984.469 | | | 190.3906 | |  | |  | |  |
| 26.91667 | | 317.8904 | | | | 6.576771 | | | | 2982.945 | | | 189.8516 | |  | |  | |  |
| 26.925 | | 317.9788 | | | | 6.571763 | | | | 2981.367 | | | 188.9141 | |  | |  | |  |
| 26.93333 | | 318.0657 | | | | 6.568469 | | | | 2979.844 | | | 187.9297 | |  | |  | |  |
| 26.94167 | | 318.1556 | | | | 6.564981 | | | | 2978.328 | | | 186.9844 | |  | |  | |  |
| 26.95 | | 318.2455 | | | | 6.559709 | | | | 2976.836 | | | 186.2656 | |  | |  | |  |
| 26.95833 | | 318.3317 | | | | 6.557005 | | | | 2975.328 | | | 185.2969 | |  | |  | |  |
| 26.96667 | | 318.4164 | | | | 6.552878 | | | | 2973.797 | | | 184.3828 | |  | |  | |  |
| 26.975 | | 318.504 | | | | 6.547985 | | | | 2972.336 | | | 183.7969 | |  | |  | |  |
| 26.98333 | | 318.5947 | | | | 6.545801 | | | | 2970.875 | | | 183.0313 | |  | |  | |  |
| 26.99167 | | 318.6808 | | | | 6.542429 | | | | 2969.422 | | | 182.3828 | |  | |  | |  |
| 27 | | 318.7685 | | | | 6.537604 | | | | 2967.953 | | | 181.5547 | |  | |  | |  |
| 27.00833 | | 318.8569 | | | | 6.535314 | | | | 2966.523 | | | 180.6875 | |  | |  | |  |
| 27.01667 | | 318.946 | | | | 6.532757 | | | | 2965.086 | | | 180.0234 | |  | |  | |  |
| 27.025 | | 319.0337 | | | | 6.528604 | | | | 2963.617 | | | 179.5078 | |  | |  | |  |
| 27.03333 | | 319.1229 | | | | 6.526655 | | | | 2962.172 | | | 178.7969 | |  | |  | |  |
| 27.04167 | | 319.2113 | | | | 6.524402 | | | | 2960.719 | | | 178.2031 | |  | |  | |  |
| 27.05 | | 319.3004 | | | | 6.520403 | | | | 2959.305 | | | 177.4688 | |  | |  | |  |
| 27.05833 | | 319.3881 | | | | 6.518138 | | | | 2957.898 | | | 176.7266 | |  | |  | |  |
| 27.06667 | | 319.4757 | | | | 6.516028 | | | | 2956.477 | | | 176.3438 | |  | |  | |  |
| 27.075 | | 319.5627 | | | | 6.512027 | | | | 2955.031 | | | 175.6016 | |  | |  | |  |
| 27.08333 | | 319.6503 | | | | 6.51011 | | | | 2953.625 | | | 175.1563 | |  | |  | |  |
| 27.09167 | | 319.7387 | | | | 6.508494 | | | | 2952.195 | | | 174.625 | |  | |  | |  |
| 27.1 | | 319.8249 | | | | 6.504726 | | | | 2950.805 | | | 173.9453 | |  | |  | |  |
| 27.10833 | | 319.9125 | | | | 6.503503 | | | | 2949.406 | | | 173.3984 | |  | |  | |  |
| 27.11667 | | 319.998 | | | | 6.501117 | | | | 2947.977 | | | 172.7891 | |  | |  | |  |
| 27.125 | | 320.0849 | | | | 6.496521 | | | | 2946.602 | | | 172 | |  | |  | |  |
| 27.13333 | | 320.1748 | | | | 6.495307 | | | | 2945.172 | | | 171.6172 | |  | |  | |  |
| 27.14167 | | 320.2624 | | | | 6.492808 | | | | 2943.773 | | | 171.0156 | |  | |  | |  |
| 27.15 | | 320.3493 | | | | 6.488394 | | | | 2942.406 | | | 170.2344 | |  | |  | |  |
| 27.15833 | | 320.44 | | | | 6.487669 | | | | 2941.016 | | | 169.6719 | |  | |  | |  |
| 27.16667 | | 320.5291 | | | | 6.486601 | | | | 2939.641 | | | 168.9453 | |  | |  | |  |
| 27.175 | | 320.6183 | | | | 6.482118 | | | | 2938.297 | | | 168.4766 | |  | |  | |  |
| 27.18333 | | 320.7052 | | | | 6.481125 | | | | 2936.898 | | | 167.9453 | |  | |  | |  |
| 27.19167 | | 320.7906 | | | | 6.479345 | | | | 2935.555 | | | 167.2578 | |  | |  | |  |
| 27.2 | | 320.8775 | | | | 6.475153 | | | | 2934.219 | | | 166.7891 | |  | |  | |  |
| 27.20833 | | 320.9674 | | | | 6.474564 | | | | 2932.867 | | | 166.4375 | |  | |  | |  |
| 27.21667 | | 321.0551 | | | | 6.472675 | | | | 2931.555 | | | 165.7656 | |  | |  | |  |
| 27.225 | | 321.1443 | | | | 6.468947 | | | | 2930.195 | | | 165.1563 | |  | |  | |  |
| 27.23333 | | 321.2304 | | | | 6.468544 | | | | 2928.852 | | | 164.5 | |  | |  | |  |
| 27.24167 | | 321.3181 | | | | 6.466935 | | | | 2927.539 | | | 164.0313 | |  | |  | |  |
| 27.25 | | 321.4065 | | | | 6.462241 | | | | 2926.195 | | | 163.8125 | |  | |  | |  |
| 27.25833 | | 321.4926 | | | | 6.462545 | | | | 2924.844 | | | 163.2891 | |  | |  | |  |
| 27.26667 | | 321.5803 | | | | 6.461354 | | | | 2923.531 | | | 162.7344 | |  | |  | |  |
| 27.275 | | 321.6672 | | | | 6.457219 | | | | 2922.219 | | | 162.5234 | |  | |  | |  |
| 27.28333 | | 321.7549 | | | | 6.45676 | | | | 2920.93 | | | 162.0625 | |  | |  | |  |
| 27.29167 | | 321.8395 | | | | 6.45502 | | | | 2919.602 | | | 161.4766 | |  | |  | |  |
| 27.3 | | 321.9257 | | | | 6.451587 | | | | 2918.258 | | | 161.0859 | |  | |  | |  |
| 27.30833 | | 322.0119 | | | | 6.451188 | | | | 2916.953 | | | 160.7266 | |  | |  | |  |
| 27.31667 | | 322.1033 | | | | 6.448662 | | | | 2915.648 | | | 160.4922 | |  | |  | |  |
| 27.325 | | 322.1917 | | | | 6.446037 | | | | 2914.32 | | | 159.9609 | |  | |  | |  |
| 27.33333 | | 322.2786 | | | | 6.445497 | | | | 2913.023 | | | 159.2578 | |  | |  | |  |
| 27.34167 | | 322.3662 | | | | 6.443202 | | | | 2911.742 | | | 158.6094 | |  | |  | |  |
| 27.35 | | 322.4569 | | | | 6.44116 | | | | 2910.445 | | | 158.1406 | |  | |  | |  |
| 27.35833 | | 322.5506 | | | | 6.440929 | | | | 2909.141 | | | 157.9219 | |  | |  | |  |
| 27.36667 | | 322.6412 | | | | 6.440126 | | | | 2907.828 | | | 157.5547 | |  | |  | |  |
| 27.375 | | 322.7311 | | | | 6.436934 | | | | 2906.555 | | | 157.1172 | |  | |  | |  |
| 27.38333 | | 322.8203 | | | | 6.436812 | | | | 2905.32 | | | 156.6484 | |  | |  | |  |
| 27.39167 | | 322.9109 | | | | 6.435597 | | | | 2904.063 | | | 156.1094 | |  | |  | |  |
| 27.4 | | 322.9979 | | | | 6.432481 | | | | 2902.797 | | | 155.6797 | |  | |  | |  |
| 27.40833 | | 323.084 | | | | 6.432154 | | | | 2901.508 | | | 155.3047 | |  | |  | |  |
| 27.41667 | | 323.1709 | | | | 6.431224 | | | | 2900.234 | | | 154.9844 | |  | |  | |  |
| 27.425 | | 323.2586 | | | | 6.428932 | | | | 2898.969 | | | 154.6797 | |  | |  | |  |
| 27.43333 | | 323.3463 | | | | 6.428596 | | | | 2897.719 | | | 154.4922 | |  | |  | |  |
| 27.44167 | | 323.4324 | | | | 6.427254 | | | | 2896.477 | | | 153.7891 | |  | |  | |  |
| 27.45 | | 323.5208 | | | | 6.424169 | | | | 2895.234 | | | 153.3281 | |  | |  | |  |
| 27.45833 | | 323.6107 | | | | 6.423694 | | | | 2893.977 | | | 152.6875 | |  | |  | |  |
| 27.46667 | | 323.6999 | | | | 6.422379 | | | | 2892.711 | | | 152.0547 | |  | |  | |  |
| 27.475 | | 323.786 | | | | 6.41986 | | | | 2891.453 | | | 151.7578 | |  | |  | |  |
| 27.48333 | | 323.8752 | | | | 6.419647 | | | | 2890.18 | | | 151.5547 | |  | |  | |  |
| 27.49167 | | 323.9666 | | | | 6.418875 | | | | 2888.984 | | | 151.1953 | |  | |  | |  |
| 27.5 | | 324.0573 | | | | 6.416725 | | | | 2887.758 | | | 150.6719 | |  | |  | |  |
| 27.50833 | | 324.1479 | | | | 6.416189 | | | | 2886.563 | | | 150.4609 | |  | |  | |  |
| 27.51667 | | 324.2371 | | | | 6.415892 | | | | 2885.367 | | | 150.0703 | |  | |  | |  |
| 27.525 | | 324.3247 | | | | 6.413492 | | | | 2884.141 | | | 149.6406 | |  | |  | |  |
| 27.53333 | | 324.4139 | | | | 6.41365 | | | | 2882.891 | | | 149.2656 | |  | |  | |  |
| 27.54167 | | 324.5008 | | | | 6.413268 | | | | 2881.68 | | | 149.1406 | |  | |  | |  |
| 27.55 | | 324.5892 | | | | 6.410424 | | | | 2880.477 | | | 149.0469 | |  | |  | |  |
| 27.55833 | | 324.6799 | | | | 6.409174 | | | | 2879.25 | | | 148.6797 | |  | |  | |  |
| 27.56667 | | 324.7675 | | | | 6.409403 | | | | 2878.039 | | | 148.0625 | |  | |  | |  |
| 27.575 | | 324.8582 | | | | 6.40708 | | | | 2876.844 | | | 147.6328 | |  | |  | |  |
| 27.58333 | | 324.9481 | | | | 6.406057 | | | | 2875.641 | | | 147.2813 | |  | |  | |  |
| 27.59167 | | 325.0365 | | | | 6.406285 | | | | 2874.406 | | | 146.9531 | |  | |  | |  |
| 27.6 | | 325.1257 | | | | 6.403359 | | | | 2873.164 | | | 146.4922 | |  | |  | |  |
| 27.60833 | | 325.2163 | | | | 6.402586 | | | | 2871.969 | | | 146.3906 | |  | |  | |  |
| 27.61667 | | 325.3047 | | | | 6.403205 | | | | 2870.813 | | | 146.0391 | |  | |  | |  |
| 27.625 | | 325.3946 | | | | 6.400702 | | | | 2869.633 | | | 145.6797 | |  | |  | |  |
| 27.63333 | | 325.483 | | | | 6.399672 | | | | 2868.438 | | | 145.3203 | |  | |  | |  |
| 27.64167 | | 325.5722 | | | | 6.400987 | | | | 2867.25 | | | 144.8828 | |  | |  | |  |
| 27.65 | | 325.6621 | | | | 6.398216 | | | | 2866.086 | | | 144.5234 | |  | |  | |  |
| 27.65833 | | 325.7475 | | | | 6.396825 | | | | 2864.875 | | | 144.1172 | |  | |  | |  |
| 27.66667 | | 325.8352 | | | | 6.396522 | | | | 2863.695 | | | 143.75 | |  | |  | |  |
| 27.675 | | 325.9221 | | | | 6.394676 | | | | 2862.516 | | | 143.2891 | |  | |  | |  |
| 27.68333 | | 326.0105 | | | | 6.394979 | | | | 2861.344 | | | 142.9141 | |  | |  | |  |
| 27.69167 | | 326.0989 | | | | 6.395766 | | | | 2860.188 | | | 142.7578 | |  | |  | |  |
| 27.7 | | 326.1873 | | | | 6.393743 | | | | 2859.031 | | | 142.3906 | |  | |  | |  |
| 27.70833 | | 326.2772 | | | | 6.392948 | | | | 2857.867 | | | 142.0391 | |  | |  | |  |
| 27.71667 | | 326.3641 | | | | 6.393959 | | | | 2856.703 | | | 141.6875 | |  | |  | |  |
| 27.725 | | 326.451 | | | | 6.392154 | | | | 2855.57 | | | 141.2266 | |  | |  | |  |
| 27.73333 | | 326.5417 | | | | 6.392292 | | | | 2854.422 | | | 140.9297 | |  | |  | |  |
| 27.74167 | | 326.6301 | | | | 6.39307 | | | | 2853.25 | | | 140.7031 | |  | |  | |  |
| 27.75 | | 326.7178 | | | | 6.391529 | | | | 2852.109 | | | 140.3047 | |  | |  | |  |
| 27.75833 | | 326.8054 | | | | 6.391256 | | | | 2850.961 | | | 139.8281 | |  | |  | |  |
| 27.76667 | | 326.8953 | | | | 6.391127 | | | | 2849.82 | | | 139.6406 | |  | |  | |  |
| 27.775 | | 326.9845 | | | | 6.388423 | | | | 2848.695 | | | 139.0859 | |  | |  | |  |
| 27.78333 | | 327.0721 | | | | 6.387097 | | | | 2847.555 | | | 138.75 | |  | |  | |  |
| 27.79167 | | 327.1583 | | | | 6.388036 | | | | 2846.406 | | | 138.1406 | |  | |  | |  |
| 27.8 | | 327.249 | | | | 6.38609 | | | | 2845.289 | | | 137.5547 | |  | |  | |  |
| 27.80833 | | 327.3396 | | | | 6.385866 | | | | 2844.18 | | | 137.5078 | |  | |  | |  |
| 27.81667 | | 327.4258 | | | | 6.386699 | | | | 2843.031 | | | 137.5078 | |  | |  | |  |
| 27.825 | | 327.5134 | | | | 6.384692 | | | | 2841.938 | | | 137.125 | |  | |  | |  |
| 27.83333 | | 327.6041 | | | | 6.385212 | | | | 2840.813 | | | 136.8281 | |  | |  | |  |
| 27.84167 | | 327.6978 | | | | 6.385749 | | | | 2839.742 | | | 136.7969 | |  | |  | |  |
| 27.85 | | 327.7847 | | | | 6.383943 | | | | 2838.672 | | | 136.6484 | |  | |  | |  |
| 27.85833 | | 327.8738 | | | | 6.38365 | | | | 2837.531 | | | 136.4844 | |  | |  | |  |
| 27.86667 | | 327.9622 | | | | 6.384187 | | | | 2836.375 | | | 136.2109 | |  | |  | |  |
| 27.875 | | 328.0544 | | | | 6.382154 | | | | 2835.273 | | | 136.2344 | |  | |  | |  |
| 27.88333 | | 328.1451 | | | | 6.380882 | | | | 2834.164 | | | 136.3438 | |  | |  | |  |
| 27.89167 | | 328.2342 | | | | 6.380931 | | | | 2833.016 | | | 135.8672 | |  | |  | |  |
| 27.9 | | 328.3249 | | | | 6.379948 | | | | 2831.898 | | | 135.3984 | |  | |  | |  |
| 27.90833 | | 328.417 | | | | 6.377653 | | | | 2830.773 | | | 135.3438 | |  | |  | |  |
| 27.91667 | | 328.5062 | | | | 6.378816 | | | | 2829.656 | | | 135.2266 | |  | |  | |  |
| 27.925 | | 328.5931 | | | | 6.379093 | | | | 2828.523 | | | 134.6953 | |  | |  | |  |
| 27.93333 | | 328.683 | | | | 6.376589 | | | | 2827.359 | | | 134.2656 | |  | |  | |  |
| 27.94167 | | 328.7714 | | | | 6.377719 | | | | 2826.281 | | | 133.7813 | |  | |  | |  |
| 27.95 | | 328.8643 | | | | 6.377734 | | | | 2825.203 | | | 133.3984 | |  | |  | |  |
| 27.95833 | | 328.9512 | | | | 6.37582 | | | | 2824.078 | | | 132.9375 | |  | |  | |  |
| 27.96667 | | 329.0411 | | | | 6.377478 | | | | 2822.961 | | | 132.4766 | |  | |  | |  |
| 27.975 | | 329.1295 | | | | 6.377834 | | | | 2821.914 | | | 132.375 | |  | |  | |  |
| 27.98333 | | 329.2187 | | | | 6.375985 | | | | 2820.844 | | | 131.9063 | |  | |  | |  |
| 27.99167 | | 329.3071 | | | | 6.376824 | | | | 2819.789 | | | 131.4609 | |  | |  | |  |
| 28 | | 329.3962 | | | | 6.376613 | | | | 2818.719 | | | 131.1094 | |  | |  | |  |
| 28.00833 | | 329.4869 | | | | 6.374523 | | | | 2817.672 | | | 131.0313 | |  | |  | |  |
| 28.01667 | | 329.5791 | | | | 6.375319 | | | | 2816.617 | | | 130.7734 | |  | |  | |  |
| 28.025 | | 329.6697 | | | | 6.375822 | | | | 2815.531 | | | 130.8125 | |  | |  | |  |
| 28.03333 | | 329.7581 | | | | 6.374212 | | | | 2814.477 | | | 130.6797 | |  | |  | |  |
| 28.04167 | | 329.848 | | | | 6.375655 | | | | 2813.445 | | | 130.5547 | |  | |  | |  |
| 28.05 | | 329.9357 | | | | 6.376341 | | | | 2812.391 | | | 130.1953 | |  | |  | |  |
| 28.05833 | | 330.0263 | | | | 6.374741 | | | | 2811.305 | | | 130 | |  | |  | |  |
| 28.06667 | | 330.117 | | | | 6.37608 | | | | 2810.234 | | | 129.5859 | |  | |  | |  |
| 28.075 | | 330.2054 | | | | 6.376996 | | | | 2809.141 | | | 129.3672 | |  | |  | |  |
| 28.08333 | | 330.2946 | | | | 6.374248 | | | | 2808.063 | | | 129.3594 | |  | |  | |  |
| 28.09167 | | 330.3845 | | | | 6.374562 | | | | 2806.969 | | | 129.1641 | |  | |  | |  |
| 28.1 | | 330.4729 | | | | 6.375925 | | | | 2805.938 | | | 129.1094 | |  | |  | |  |
| 28.10833 | | 330.5605 | | | | 6.372636 | | | | 2804.875 | | | 128.8438 | |  | |  | |  |
| 28.11667 | | 330.6475 | | | | 6.372191 | | | | 2803.844 | | | 128.4688 | |  | |  | |  |
| 28.125 | | 330.7366 | | | | 6.373765 | | | | 2802.797 | | | 127.9922 | |  | |  | |  |
| 28.13333 | | 330.8265 | | | | 6.370573 | | | | 2801.719 | | | 128.1016 | |  | |  | |  |
| 28.14167 | | 330.9157 | | | | 6.370238 | | | | 2800.648 | | | 127.8516 | |  | |  | |  |
| 28.15 | | 331.0018 | | | | 6.371813 | | | | 2799.586 | | | 127.8594 | |  | |  | |  |
| 28.15833 | | 331.088 | | | | 6.370078 | | | | 2798.539 | | | 127.6953 | |  | |  | |  |
| 28.16667 | | 331.1757 | | | | 6.370452 | | | | 2797.508 | | | 127.1016 | |  | |  | |  |
| 28.175 | | 331.2641 | | | | 6.370102 | | | | 2796.508 | | | 127.0078 | |  | |  | |  |
| 28.18333 | | 331.351 | | | | 6.368194 | | | | 2795.414 | | | 126.7734 | |  | |  | |  |
| 28.19167 | | 331.4401 | | | | 6.368322 | | | | 2794.391 | | | 126.3828 | |  | |  | |  |
| 28.2 | | 331.5293 | | | | 6.366819 | | | | 2793.328 | | | 125.9531 | |  | |  | |  |
| 28.20833 | | 331.6192 | | | | 6.364678 | | | | 2792.273 | | | 126.2656 | |  | |  | |  |
| 28.21667 | | 331.7083 | | | | 6.365277 | | | | 2791.281 | | | 125.9922 | |  | |  | |  |
| 28.225 | | 331.796 | | | | 6.365705 | | | | 2790.234 | | | 125.7578 | |  | |  | |  |
| 28.23333 | | 331.8867 | | | | 6.364238 | | | | 2789.211 | | | 125.5781 | |  | |  | |  |
| 28.24167 | | 331.9781 | | | | 6.364875 | | | | 2788.195 | | | 125.2422 | |  | |  | |  |
| 28.25 | | 332.0695 | | | | 6.366893 | | | | 2787.195 | | | 125.1484 | |  | |  | |  |
| 28.25833 | | 332.1586 | | | | 6.366003 | | | | 2786.094 | | | 124.8281 | |  | |  | |  |
| 28.26667 | | 332.2478 | | | | 6.36605 | | | | 2785.07 | | | 124.4688 | |  | |  | |  |
| 28.275 | | 332.3377 | | | | 6.366974 | | | | 2784.047 | | | 124.2734 | |  | |  | |  |
| 28.28333 | | 332.4299 | | | | 6.365009 | | | | 2783.008 | | | 124.3438 | |  | |  | |  |
| 28.29167 | | 332.5198 | | | | 6.365021 | | | | 2782.008 | | | 123.7969 | |  | |  | |  |
| 28.3 | | 332.6074 | | | | 6.366646 | | | | 2780.977 | | | 123.5234 | |  | |  | |  |
| 28.30833 | | 332.6966 | | | | 6.365037 | | | | 2779.969 | | | 123.5078 | |  | |  | |  |
| 28.31667 | | 332.7857 | | | | 6.365438 | | | | 2778.969 | | | 123.4609 | |  | |  | |  |
| 28.325 | | 332.8771 | | | | 6.367712 | | | | 2777.961 | | | 123.4531 | |  | |  | |  |
| 28.33333 | | 332.9641 | | | | 6.366315 | | | | 2776.922 | | | 123.4063 | |  | |  | |  |
| 28.34167 | | 333.0525 | | | | 6.365391 | | | | 2775.961 | | | 123.0313 | |  | |  | |  |
| 28.35 | | 333.1431 | | | | 6.36606 | | | | 2774.969 | | | 122.7813 | |  | |  | |  |
| 28.35833 | | 333.2323 | | | | 6.364661 | | | | 2773.93 | | | 122.4922 | |  | |  | |  |
| 28.36667 | | 333.3207 | | | | 6.364536 | | | | 2772.906 | | | 122.3906 | |  | |  | |  |
| 28.375 | | 333.4091 | | | | 6.364837 | | | | 2771.875 | | | 122.1484 | |  | |  | |  |
| 28.38333 | | 333.4983 | | | | 6.362634 | | | | 2770.852 | | | 121.7266 | |  | |  | |  |
| 28.39167 | | 333.5889 | | | | 6.36207 | | | | 2769.875 | | | 121.3438 | |  | |  | |  |
| 28.4 | | 333.6788 | | | | 6.362957 | | | | 2768.891 | | | 120.9922 | |  | |  | |  |
| 28.40833 | | 333.7702 | | | | 6.361904 | | | | 2767.891 | | | 120.6406 | |  | |  | |  |
| 28.41667 | | 333.8624 | | | | 6.361207 | | | | 2766.891 | | | 120.4141 | |  | |  | |  |
| 28.425 | | 333.9545 | | | | 6.363512 | | | | 2765.898 | | | 120.2422 | |  | |  | |  |
| 28.43333 | | 334.0459 | | | | 6.362517 | | | | 2764.938 | | | 120.2344 | |  | |  | |  |
| 28.44167 | | 334.1359 | | | | 6.361284 | | | | 2763.969 | | | 120.0781 | |  | |  | |  |
| 28.45 | | 334.2258 | | | | 6.362522 | | | | 2763 | | | 119.8438 | |  | |  | |  |
| 28.45833 | | 334.3172 | | | | 6.361399 | | | | 2762.039 | | | 119.6953 | |  | |  | |  |
| 28.46667 | | 334.4101 | | | | 6.359696 | | | | 2761.063 | | | 119.75 | |  | |  | |  |
| 28.475 | | 334.5022 | | | | 6.362928 | | | | 2760.078 | | | 119.5938 | |  | |  | |  |
| 28.48333 | | 334.5929 | | | | 6.363371 | | | | 2759.07 | | | 119.2422 | |  | |  | |  |
| 28.49167 | | 334.6835 | | | | 6.361269 | | | | 2758.086 | | | 119.0469 | |  | |  | |  |
| 28.5 | | 334.7757 | | | | 6.362426 | | | | 2757.109 | | | 118.8438 | |  | |  | |  |
| 28.50833 | | 334.8648 | | | | 6.364011 | | | | 2756.125 | | | 118.5703 | |  | |  | |  |
| 28.51667 | | 334.9532 | | | | 6.360328 | | | | 2755.117 | | | 118.3359 | |  | |  | |  |
| 28.525 | | 335.0432 | | | | 6.361622 | | | | 2754.148 | | | 118.2344 | |  | |  | |  |
| 28.53333 | | 335.1323 | | | | 6.362642 | | | | 2753.188 | | | 117.8594 | |  | |  | |  |
| 28.54167 | | 335.22 | | | | 6.361147 | | | | 2752.219 | | | 117.5781 | |  | |  | |  |
| 28.55 | | 335.3091 | | | | 6.362419 | | | | 2751.258 | | | 117.3594 | |  | |  | |  |
| 28.55833 | | 335.3968 | | | | 6.363846 | | | | 2750.297 | | | 117 | |  | |  | |  |
| 28.56667 | | 335.486 | | | | 6.362457 | | | | 2749.336 | | | 116.8594 | |  | |  | |  |
| 28.575 | | 335.5729 | | | | 6.36317 | | | | 2748.359 | | | 116.8906 | |  | |  | |  |
| 28.58333 | | 335.6605 | | | | 6.362893 | | | | 2747.43 | | | 116.7031 | |  | |  | |  |
| 28.59167 | | 335.7504 | | | | 6.361018 | | | | 2746.484 | | | 116.6172 | |  | |  | |  |
| 28.6 | | 335.8403 | | | | 6.361322 | | | | 2745.539 | | | 116.5547 | |  | |  | |  |
| 28.60833 | | 335.9295 | | | | 6.36147 | | | | 2744.594 | | | 116.2031 | |  | |  | |  |
| 28.61667 | | 336.0216 | | | | 6.359679 | | | | 2743.633 | | | 116.2188 | |  | |  | |  |
| 28.625 | | 336.1123 | | | | 6.360531 | | | | 2742.648 | | | 116.3438 | |  | |  | |  |
| 28.63333 | | 336.2015 | | | | 6.362471 | | | | 2741.703 | | | 116.2031 | |  | |  | |  |
| 28.64167 | | 336.2906 | | | | 6.360441 | | | | 2740.727 | | | 115.9219 | |  | |  | |  |
| 28.65 | | 336.3805 | | | | 6.360086 | | | | 2739.766 | | | 115.9453 | |  | |  | |  |
| 28.65833 | | 336.4682 | | | | 6.361503 | | | | 2738.836 | | | 115.9688 | |  | |  | |  |
| 28.66667 | | 336.5558 | | | | 6.359527 | | | | 2737.867 | | | 115.8438 | |  | |  | |  |
| 28.675 | | 336.6458 | | | | 6.359764 | | | | 2736.867 | | | 115.8906 | |  | |  | |  |
| 28.68333 | | 336.7349 | | | | 6.36061 | | | | 2735.922 | | | 115.6328 | |  | |  | |  |
| 28.69167 | | 336.8263 | | | | 6.359348 | | | | 2734.992 | | | 115.5781 | |  | |  | |  |
| 28.7 | | 336.9132 | | | | 6.361497 | | | | 2734.023 | | | 115.5859 | |  | |  | |  |
| 28.70833 | | 337.0016 | | | | 6.362455 | | | | 2733.031 | | | 115.0625 | |  | |  | |  |
| 28.71667 | | 337.0908 | | | | 6.360077 | | | | 2732.078 | | | 115.0781 | |  | |  | |  |
| 28.725 | | 337.183 | | | | 6.36082 | | | | 2731.102 | | | 115.1172 | |  | |  | |  |
| 28.73333 | | 337.2744 | | | | 6.362097 | | | | 2730.172 | | | 114.8984 | |  | |  | |  |
| 28.74167 | | 337.365 | | | | 6.35985 | | | | 2729.211 | | | 114.6094 | |  | |  | |  |
| 28.75 | | 337.4557 | | | | 6.360199 | | | | 2728.242 | | | 114.3438 | |  | |  | |  |
| 28.75833 | | 337.5448 | | | | 6.362501 | | | | 2727.352 | | | 113.9219 | |  | |  | |  |
| 28.76667 | | 337.6362 | | | | 6.361502 | | | | 2726.391 | | | 113.8594 | |  | |  | |  |
| 28.775 | | 337.7261 | | | | 6.36226 | | | | 2725.422 | | | 113.75 | |  | |  | |  |
| 28.78333 | | 337.8153 | | | | 6.365292 | | | | 2724.492 | | | 113.4375 | |  | |  | |  |
| 28.79167 | | 337.9052 | | | | 6.363881 | | | | 2723.578 | | | 113.4766 | |  | |  | |  |
| 28.8 | | 337.9981 | | | | 6.363537 | | | | 2722.656 | | | 113.1641 | |  | |  | |  |
| 28.80833 | | 338.0873 | | | | 6.365787 | | | | 2721.766 | | | 113.0938 | |  | |  | |  |
| 28.81667 | | 338.1749 | | | | 6.364581 | | | | 2720.82 | | | 113.0391 | |  | |  | |  |
| 28.825 | | 338.2686 | | | | 6.364224 | | | | 2719.891 | | | 112.6328 | |  | |  | |  |
| 28.83333 | | 338.3615 | | | | 6.366567 | | | | 2718.977 | | | 112.3594 | |  | |  | |  |
| 28.84167 | | 338.4499 | | | | 6.365472 | | | | 2718.031 | | | 112.3594 | |  | |  | |  |
| 28.85 | | 338.5398 | | | | 6.363723 | | | | 2717.117 | | | 112.3984 | |  | |  | |  |
| 28.85833 | | 338.6282 | | | | 6.365819 | | | | 2716.18 | | | 112.0781 | |  | |  | |  |
| 28.86667 | | 338.7219 | | | | 6.364328 | | | | 2715.242 | | | 111.6797 | |  | |  | |  |
| 28.875 | | 338.8155 | | | | 6.362095 | | | | 2714.359 | | | 111.4219 | |  | |  | |  |
| 28.88333 | | 338.9032 | | | | 6.364612 | | | | 2713.453 | | | 111.4844 | |  | |  | |  |
| 28.89167 | | 338.9923 | | | | 6.364621 | | | | 2712.516 | | | 111.2969 | |  | |  | |  |
| 28.9 | | 339.0815 | | | | 6.364416 | | | | 2711.57 | | | 111 | |  | |  | |  |
| 28.90833 | | 339.1714 | | | | 6.366795 | | | | 2710.68 | | | 111 | |  | |  | |  |
| 28.91667 | | 339.2606 | | | | 6.366636 | | | | 2709.797 | | | 110.8594 | |  | |  | |  |
| 28.925 | | 339.352 | | | | 6.364944 | | | | 2708.906 | | | 110.5 | |  | |  | |  |
| 28.93333 | | 339.4419 | | | | 6.367489 | | | | 2707.961 | | | 110.0469 | |  | |  | |  |
| 28.94167 | | 339.534 | | | | 6.367631 | | | | 2707.055 | | | 109.6328 | |  | |  | |  |
| 28.95 | | 339.6224 | | | | 6.366116 | | | | 2706.172 | | | 109.875 | |  | |  | |  |
| 28.95833 | | 339.7101 | | | | 6.369041 | | | | 2705.25 | | | 109.8984 | |  | |  | |  |
| 28.96667 | | 339.8015 | | | | 6.369422 | | | | 2704.344 | | | 109.4922 | |  | |  | |  |
| 28.975 | | 339.8936 | | | | 6.367787 | | | | 2703.461 | | | 109.4219 | |  | |  | |  |
| 28.98333 | | 339.985 | | | | 6.369186 | | | | 2702.609 | | | 109.3047 | |  | |  | |  |
| 28.99167 | | 340.0727 | | | | 6.368654 | | | | 2701.75 | | | 109.3906 | |  | |  | |  |
| 29 | | 340.1626 | | | | 6.367047 | | | | 2700.789 | | | 109.3203 | |  | |  | |  |
| 29.00833 | | 340.2503 | | | | 6.368144 | | | | 2699.867 | | | 109.1406 | |  | |  | |  |
| 29.01667 | | 340.3405 | | | | 6.368429 | | | | 2699.008 | | | 109.1016 | |  | |  | |  |
| 29.025 | | 340.4301 | | | | 6.36631 | | | | 2698.094 | | | 109.4063 | |  | |  | |  |
| 29.03333 | | 340.5174 | | | | 6.368004 | | | | 2697.188 | | | 109.125 | |  | |  | |  |
| 29.04167 | | 340.6076 | | | | 6.368433 | | | | 2696.266 | | | 109.0703 | |  | |  | |  |
| 29.05 | | 340.6957 | | | | 6.367551 | | | | 2695.359 | | | 108.9531 | |  | |  | |  |
| 29.05833 | | 340.7808 | | | | 6.368712 | | | | 2694.469 | | | 108.9531 | |  | |  | |  |
| 29.06667 | | 340.8703 | | | | 6.369372 | | | | 2693.555 | | | 108.9922 | |  | |  | |  |
| 29.075 | | 340.9606 | | | | 6.367119 | | | | 2692.594 | | | 108.8047 | |  | |  | |  |
| 29.08333 | | 341.0472 | | | | 6.368192 | | | | 2691.727 | | | 108.7578 | |  | |  | |  |
| 29.09167 | | 341.1397 | | | | 6.368479 | | | | 2690.82 | | | 108.5625 | |  | |  | |  |
| 29.1 | | 341.2292 | | | | 6.366572 | | | | 2689.914 | | | 108.6172 | |  | |  | |  |
| 29.10833 | | 341.318 | | | | 6.367811 | | | | 2689.008 | | | 108.5313 | |  | |  | |  |
| 29.11667 | | 341.4098 | | | | 6.368538 | | | | 2688.094 | | | 108.2031 | |  | |  | |  |
| 29.125 | | 341.5 | | | | 6.367353 | | | | 2687.195 | | | 107.9297 | |  | |  | |  |
| 29.13333 | | 341.5925 | | | | 6.36809 | | | | 2686.297 | | | 107.8906 | |  | |  | |  |
| 29.14167 | | 341.6843 | | | | 6.369425 | | | | 2685.414 | | | 107.4375 | |  | |  | |  |
| 29.15 | | 341.7731 | | | | 6.368097 | | | | 2684.5 | | | 107.1875 | |  | |  | |  |
| 29.15833 | | 341.8619 | | | | 6.367252 | | | | 2683.617 | | | 106.9297 | |  | |  | |  |
| 29.16667 | | 341.9521 | | | | 6.368864 | | | | 2682.75 | | | 106.5781 | |  | |  | |  |
| 29.175 | | 342.0417 | | | | 6.368663 | | | | 2681.883 | | | 106.6797 | |  | |  | |  |
| 29.18333 | | 342.1312 | | | | 6.366792 | | | | 2680.992 | | | 106.6016 | |  | |  | |  |
| 29.19167 | | 342.2193 | | | | 6.368612 | | | | 2680.156 | | | 106.1953 | |  | |  | |  |
| 29.2 | | 342.3066 | | | | 6.36935 | | | | 2679.297 | | | 106.1016 | |  | |  | |  |
| 29.20833 | | 342.3947 | | | | 6.36738 | | | | 2678.438 | | | 106.1719 | |  | |  | |  |
| 29.21667 | | 342.4827 | | | | 6.368412 | | | | 2677.586 | | | 106.125 | |  | |  | |  |
| 29.225 | | 342.573 | | | | 6.368794 | | | | 2676.688 | | | 106.0938 | |  | |  | |  |
| 29.23333 | | 342.6618 | | | | 6.368162 | | | | 2675.813 | | | 105.9375 | |  | |  | |  |
| 29.24167 | | 342.7498 | | | | 6.369631 | | | | 2674.969 | | | 105.6094 | |  | |  | |  |
| 29.25 | | 342.8386 | | | | 6.369508 | | | | 2674.094 | | | 105.6953 | |  | |  | |  |
| 29.25833 | | 342.9274 | | | | 6.368878 | | | | 2673.195 | | | 105.4375 | |  | |  | |  |
| 29.26667 | | 343.0155 | | | | 6.37057 | | | | 2672.32 | | | 105.4063 | |  | |  | |  |
| 29.275 | | 343.1057 | | | | 6.371323 | | | | 2671.445 | | | 105.4375 | |  | |  | |  |
| 29.28333 | | 343.1967 | | | | 6.369889 | | | | 2670.578 | | | 105.5234 | |  | |  | |  |
| 29.29167 | | 343.2878 | | | | 6.370291 | | | | 2669.734 | | | 105.5156 | |  | |  | |  |
| 29.3 | | 343.3751 | | | | 6.372047 | | | | 2668.844 | | | 105.3438 | |  | |  | |  |
| 29.30833 | | 343.4631 | | | | 6.370232 | | | | 2667.992 | | | 105.1875 | |  | |  | |  |
| 29.31667 | | 343.5549 | | | | 6.370166 | | | | 2667.117 | | | 105.1563 | |  | |  | |  |
| 29.325 | | 343.6474 | | | | 6.37256 | | | | 2666.234 | | | 105.0859 | |  | |  | |  |
| 29.33333 | | 343.7391 | | | | 6.37109 | | | | 2665.344 | | | 105.0078 | |  | |  | |  |
| 29.34167 | | 343.8272 | | | | 6.371564 | | | | 2664.477 | | | 104.875 | |  | |  | |  |
| 29.35 | | 343.916 | | | | 6.374517 | | | | 2663.602 | | | 104.7188 | |  | |  | |  |
| 29.35833 | | 344.0063 | | | | 6.373162 | | | | 2662.75 | | | 104.5547 | |  | |  | |  |
| 29.36667 | | 344.0966 | | | | 6.372903 | | | | 2661.883 | | | 104.2422 | |  | |  | |  |
| 29.375 | | 344.1839 | | | | 6.376257 | | | | 2661.008 | | | 103.8281 | |  | |  | |  |
| 29.38333 | | 344.2726 | | | | 6.374933 | | | | 2660.141 | | | 103.6094 | |  | |  | |  |
| 29.39167 | | 344.3637 | | | | 6.374365 | | | | 2659.281 | | | 103.5625 | |  | |  | |  |
| 29.4 | | 344.4503 | | | | 6.377519 | | | | 2658.43 | | | 103.2578 | |  | |  | |  |
| 29.40833 | | 344.5368 | | | | 6.376447 | | | | 2657.57 | | | 103.125 | |  | |  | |  |
| 29.41667 | | 344.6256 | | | | 6.375654 | | | | 2656.742 | | | 102.7266 | |  | |  | |  |
| 29.425 | | 344.7166 | | | | 6.378016 | | | | 2655.93 | | | 102.3203 | |  | |  | |  |
| 29.43333 | | 344.8076 | | | | 6.375958 | | | | 2655.094 | | | 102.1953 | |  | |  | |  |
| 29.44167 | | 344.8972 | | | | 6.374748 | | | | 2654.234 | | | 101.8516 | |  | |  | |  |
| 29.45 | | 344.9845 | | | | 6.377153 | | | | 2653.406 | | | 101.4766 | |  | |  | |  |
| 29.45833 | | 345.0748 | | | | 6.376175 | | | | 2652.563 | | | 101.4063 | |  | |  | |  |
| 29.46667 | | 345.1673 | | | | 6.374999 | | | | 2651.758 | | | 101.3281 | |  | |  | |  |
| 29.475 | | 345.2553 | | | | 6.377223 | | | | 2650.953 | | | 101.2891 | |  | |  | |  |
| 29.48333 | | 345.3456 | | | | 6.375878 | | | | 2650.125 | | | 101.1328 | |  | |  | |  |
| 29.49167 | | 345.4366 | | | | 6.373699 | | | | 2649.313 | | | 100.9453 | |  | |  | |  |
| 29.5 | | 345.5262 | | | | 6.375058 | | | | 2648.508 | | | 100.8359 | |  | |  | |  |
| 29.50833 | | 345.6164 | | | | 6.374212 | | | | 2647.68 | | | 100.7969 | |  | |  | |  |
| 29.51667 | | 345.7067 | | | | 6.372256 | | | | 2646.836 | | | 100.6875 | |  | |  | |  |
| 29.525 | | 345.7962 | | | | 6.372445 | | | | 2646 | | | 100.6016 | |  | |  | |  |
| 29.53333 | | 345.885 | | | | 6.372943 | | | | 2645.172 | | | 100.75 | |  | |  | |  |
| 29.54167 | | 345.9745 | | | | 6.371319 | | | | 2644.359 | | | 100.9063 | |  | |  | |  |
| 29.55 | | 346.0641 | | | | 6.371356 | | | | 2643.531 | | | 100.7891 | |  | |  | |  |
| 29.55833 | | 346.1536 | | | | 6.372669 | | | | 2642.688 | | | 100.8125 | |  | |  | |  |
| 29.56667 | | 346.2432 | | | | 6.37135 | | | | 2641.867 | | | 100.8359 | |  | |  | |  |
| 29.575 | | 346.3334 | | | | 6.373087 | | | | 2641.031 | | | 100.9141 | |  | |  | |  |
| 29.58333 | | 346.4222 | | | | 6.374418 | | | | 2640.164 | | | 100.8828 | |  | |  | |  |
| 29.59167 | | 346.5088 | | | | 6.374031 | | | | 2639.305 | | | 100.7656 | |  | |  | |  |
| 29.6 | | 346.5969 | | | | 6.376072 | | | | 2638.477 | | | 100.4922 | |  | |  | |  |
| 29.60833 | | 346.6842 | | | | 6.377913 | | | | 2637.625 | | | 100.4297 | |  | |  | |  |
| 29.61667 | | 346.7789 | | | | 6.377033 | | | | 2636.781 | | | 100.3047 | |  | |  | |  |
| 29.625 | | 346.8677 | | | | 6.376331 | | | | 2635.922 | | | 100.0547 | |  | |  | |  |
| 29.63333 | | 346.9543 | | | | 6.377803 | | | | 2635.078 | | | 99.84375 | |  | |  | |  |
| 29.64167 | | 347.0438 | | | | 6.376589 | | | | 2634.258 | | | 99.46094 | |  | |  | |  |
| 29.65 | | 347.1333 | | | | 6.376473 | | | | 2633.453 | | | 99.17969 | |  | |  | |  |
| 29.65833 | | 347.2229 | | | | 6.377913 | | | | 2632.617 | | | 98.97656 | |  | |  | |  |
| 29.66667 | | 347.3094 | | | | 6.377456 | | | | 2631.797 | | | 98.84375 | |  | |  | |  |
| 29.675 | | 347.3997 | | | | 6.377075 | | | | 2630.992 | | | 98.82813 | |  | |  | |  |
| 29.68333 | | 347.4885 | | | | 6.378751 | | | | 2630.188 | | | 98.96875 | |  | |  | |  |
| 29.69167 | | 347.5803 | | | | 6.37833 | | | | 2629.406 | | | 99.125 | |  | |  | |  |
| 29.7 | | 347.6654 | | | | 6.377839 | | | | 2628.609 | | | 99.13281 | |  | |  | |  |
| 29.70833 | | 347.7527 | | | | 6.379938 | | | | 2627.813 | | | 98.89063 | |  | |  | |  |
| 29.71667 | | 347.8415 | | | | 6.379379 | | | | 2627.008 | | | 98.625 | |  | |  | |  |
| 29.725 | | 347.931 | | | | 6.378673 | | | | 2626.172 | | | 98.60938 | |  | |  | |  |
| 29.73333 | | 348.0198 | | | | 6.380313 | | | | 2625.328 | | | 98.54688 | |  | |  | |  |
| 29.74167 | | 348.1078 | | | | 6.37925 | | | | 2624.469 | | | 98.32813 | |  | |  | |  |
| 29.75 | | 348.1989 | | | | 6.378158 | | | | 2623.641 | | | 98.32813 | |  | |  | |  |
| 29.75833 | | 348.2906 | | | | 6.379479 | | | | 2622.844 | | | 98.1875 | |  | |  | |  |
| 29.76667 | | 348.3787 | | | | 6.380098 | | | | 2622.063 | | | 98.125 | |  | |  | |  |
| 29.775 | | 348.4639 | | | | 6.379355 | | | | 2621.242 | | | 97.78906 | |  | |  | |  |
| 29.78333 | | 348.5516 | | | | 6.381405 | | | | 2620.438 | | | 97.41406 | |  | |  | |  |
| 29.79167 | | 348.6429 | | | | 6.381294 | | | | 2619.641 | | | 97.19531 | |  | |  | |  |
| 29.8 | | 348.7328 | | | | 6.381083 | | | | 2618.82 | | | 97.41406 | |  | |  | |  |
| 29.80833 | | 348.822 | | | | 6.38263 | | | | 2618.016 | | | 97.28125 | |  | |  | |  |
| 29.81667 | | 348.9111 | | | | 6.381997 | | | | 2617.211 | | | 97.22656 | |  | |  | |  |
| 29.825 | | 349.001 | | | | 6.381251 | | | | 2616.445 | | | 97.39063 | |  | |  | |  |
| 29.83333 | | 349.0916 | | | | 6.382592 | | | | 2615.68 | | | 97.32813 | |  | |  | |  |
| 29.84167 | | 349.1786 | | | | 6.38313 | | | | 2614.898 | | | 97.36719 | |  | |  | |  |
| 29.85 | | 349.2699 | | | | 6.381436 | | | | 2614.063 | | | 97.17969 | |  | |  | |  |
| 29.85833 | | 349.3619 | | | | 6.382136 | | | | 2613.266 | | | 96.89063 | |  | |  | |  |
| 29.86667 | | 349.4518 | | | | 6.383718 | | | | 2612.453 | | | 96.80469 | |  | |  | |  |
| 29.875 | | 349.5388 | | | | 6.381694 | | | | 2611.617 | | | 96.97656 | |  | |  | |  |
| 29.88333 | | 349.6272 | | | | 6.38251 | | | | 2610.813 | | | 96.89063 | |  | |  | |  |
| 29.89167 | | 349.7156 | | | | 6.385232 | | | | 2610 | | | 96.875 | |  | |  | |  |
| 29.9 | | 349.8047 | | | | 6.383049 | | | | 2609.211 | | | 96.76563 | |  | |  | |  |
| 29.90833 | | 349.8954 | | | | 6.383534 | | | | 2608.438 | | | 96.44531 | |  | |  | |  |
| 29.91667 | | 349.983 | | | | 6.385561 | | | | 2607.648 | | | 96.41406 | |  | |  | |  |
| 29.925 | | 350.0707 | | | | 6.383605 | | | | 2606.805 | | | 96.32813 | |  | |  | |  |
| 29.93333 | | 350.157 | | | | 6.382695 | | | | 2606 | | | 96.10938 | |  | |  | |  |
| 29.94167 | | 350.2446 | | | | 6.385533 | | | | 2605.188 | | | 96.07031 | |  | |  | |  |
| 29.95 | | 350.3345 | | | | 6.384703 | | | | 2604.398 | | | 96.20313 | |  | |  | |  |
| 29.95833 | | 350.4236 | | | | 6.384262 | | | | 2603.633 | | | 95.875 | |  | |  | |  |
| 29.96667 | | 350.5128 | | | | 6.386612 | | | | 2602.836 | | | 95.58594 | |  | |  | |  |
| 29.975 | | 350.6005 | | | | 6.386281 | | | | 2602.039 | | | 95.3125 | |  | |  | |  |
| 29.98333 | | 350.6874 | | | | 6.385895 | | | | 2601.266 | | | 95.27344 | |  | |  | |  |
| 29.99167 | | 350.7737 | | | | 6.388066 | | | | 2600.461 | | | 95.375 | |  | |  | |  |
| 30 | | 350.8628 | | | | 6.387183 | | | | 2599.641 | | | 95.33594 | |  | |  | |  |
| 30.00833 | | 350.9527 | | | | 6.386717 | | | | 2598.891 | | | 95.29688 | |  | |  | |  |
| 30.01667 | | 351.0425 | | | | 6.389337 | | | | 2598.125 | | | 95.46094 | |  | |  | |  |
| 30.025 | | 351.1317 | | | | 6.387665 | | | | 2597.375 | | | 95.45313 | |  | |  | |  |
| 30.03333 | | 351.2208 | | | | 6.386094 | | | | 2596.578 | | | 95.46875 | |  | |  | |  |
| 30.04167 | | 351.3129 | | | | 6.387722 | | | | 2595.773 | | | 95.63281 | |  | |  | |  |
| 30.05 | | 351.4013 | | | | 6.387284 | | | | 2594.977 | | | 95.63281 | |  | |  | |  |
| 30.05833 | | 351.4926 | | | | 6.386471 | | | | 2594.195 | | | 96.08594 | |  | |  | |  |
| 30.06667 | | 351.5833 | | | | 6.387115 | | | | 2593.375 | | | 96.15625 | |  | |  | |  |
| 30.075 | | 351.6709 | | | | 6.387836 | | | | 2592.57 | | | 95.94531 | |  | |  | |  |
| 30.08333 | | 351.7586 | | | | 6.386297 | | | | 2591.766 | | | 95.72656 | |  | |  | |  |
| 30.09167 | | 351.8492 | | | | 6.385812 | | | | 2590.938 | | | 95.73438 | |  | |  | |  |
| 30.1 | | 351.9384 | | | | 6.387709 | | | | 2590.133 | | | 95.64844 | |  | |  | |  |
| 30.10833 | | 352.0261 | | | | 6.386264 | | | | 2589.273 | | | 95.5 | |  | |  | |  |
| 30.11667 | | 352.113 | | | | 6.386521 | | | | 2588.461 | | | 95.52344 | |  | |  | |  |
| 30.125 | | 352.1985 | | | | 6.388149 | | | | 2587.695 | | | 95.375 | |  | |  | |  |
| 30.13333 | | 352.2884 | | | | 6.386993 | | | | 2586.922 | | | 95.5625 | |  | |  | |  |
| 30.14167 | | 352.3731 | | | | 6.386656 | | | | 2586.133 | | | 95.28906 | |  | |  | |  |
| 30.15 | | 352.4594 | | | | 6.388391 | | | | 2585.344 | | | 94.89844 | |  | |  | |  |
| 30.15833 | | 352.5492 | | | | 6.387969 | | | | 2584.563 | | | 94.89063 | |  | |  | |  |
| 30.16667 | | 352.6376 | | | | 6.38841 | | | | 2583.766 | | | 94.98438 | |  | |  | |  |
| 30.175 | | 352.7246 | | | | 6.390414 | | | | 2582.984 | | | 94.85938 | |  | |  | |  |
| 30.18333 | | 352.813 | | | | 6.389166 | | | | 2582.172 | | | 94.63281 | |  | |  | |  |
| 30.19167 | | 352.9007 | | | | 6.389982 | | | | 2581.414 | | | 94.50781 | |  | |  | |  |
| 30.2 | | 352.9876 | | | | 6.391874 | | | | 2580.672 | | | 94.14063 | |  | |  | |  |
| 30.20833 | | 353.0775 | | | | 6.391449 | | | | 2579.891 | | | 94.07813 | |  | |  | |  |
| 30.21667 | | 353.1652 | | | | 6.391492 | | | | 2579.086 | | | 93.80469 | |  | |  | |  |
| 30.225 | | 353.2565 | | | | 6.39284 | | | | 2578.305 | | | 93.60156 | |  | |  | |  |
| 30.23333 | | 353.3479 | | | | 6.392096 | | | | 2577.547 | | | 93.44531 | |  | |  | |  |
| 30.24167 | | 353.437 | | | | 6.390673 | | | | 2576.773 | | | 93.54688 | |  | |  | |  |
| 30.25 | | 353.5262 | | | | 6.392137 | | | | 2576.039 | | | 93.46875 | |  | |  | |  |
| 30.25833 | | 353.6153 | | | | 6.391804 | | | | 2575.266 | | | 93.35156 | |  | |  | |  |
| 30.26667 | | 353.7023 | | | | 6.390909 | | | | 2574.531 | | | 93.28906 | |  | |  | |  |
| 30.275 | | 353.7914 | | | | 6.393018 | | | | 2573.766 | | | 93.36719 | |  | |  | |  |
| 30.28333 | | 353.882 | | | | 6.393878 | | | | 2573.008 | | | 93.75 | |  | |  | |  |
| 30.29167 | | 353.9682 | | | | 6.391613 | | | | 2572.203 | | | 93.60156 | |  | |  | |  |
| 30.3 | | 354.0566 | | | | 6.39369 | | | | 2571.43 | | | 93.42969 | |  | |  | |  |
| 30.30833 | | 354.1451 | | | | 6.394662 | | | | 2570.68 | | | 93.21875 | |  | |  | |  |
| 30.31667 | | 354.2349 | | | | 6.392442 | | | | 2569.906 | | | 93.27344 | |  | |  | |  |
| 30.325 | | 354.3226 | | | | 6.394089 | | | | 2569.109 | | | 93.10938 | |  | |  | |  |
| 30.33333 | | 354.4125 | | | | 6.394414 | | | | 2568.281 | | | 92.80469 | |  | |  | |  |
| 30.34167 | | 354.498 | | | | 6.392497 | | | | 2567.516 | | | 92.6875 | |  | |  | |  |
| 30.35 | | 354.5879 | | | | 6.394351 | | | | 2566.75 | | | 92.38281 | |  | |  | |  |
| 30.35833 | | 354.6785 | | | | 6.394431 | | | | 2566.016 | | | 92.07813 | |  | |  | |  |
| 30.36667 | | 354.7661 | | | | 6.3915 | | | | 2565.227 | | | 91.64844 | |  | |  | |  |
| 30.375 | | 354.8538 | | | | 6.39324 | | | | 2564.484 | | | 91.44531 | |  | |  | |  |
| 30.38333 | | 354.9422 | | | | 6.394111 | | | | 2563.742 | | | 91.28906 | |  | |  | |  |
| 30.39167 | | 355.0299 | | | | 6.391359 | | | | 2562.984 | | | 91.38281 | |  | |  | |  |
| 30.4 | | 355.1176 | | | | 6.393175 | | | | 2562.258 | | | 91.23438 | |  | |  | |  |
| 30.40833 | | 355.2016 | | | | 6.394355 | | | | 2561.523 | | | 90.85156 | |  | |  | |  |
| 30.41667 | | 355.2871 | | | | 6.392496 | | | | 2560.82 | | | 90.94531 | |  | |  | |  |
| 30.425 | | 355.3785 | | | | 6.394337 | | | | 2560.078 | | | 90.76563 | |  | |  | |  |
| 30.43333 | | 355.4676 | | | | 6.395824 | | | | 2559.336 | | | 91.10156 | |  | |  | |  |
| 30.44167 | | 355.5553 | | | | 6.392912 | | | | 2558.547 | | | 90.9375 | |  | |  | |  |
| 30.45 | | 355.6466 | | | | 6.393827 | | | | 2557.813 | | | 90.61719 | |  | |  | |  |
| 30.45833 | | 355.7387 | | | | 6.395897 | | | | 2557.086 | | | 90.52344 | |  | |  | |  |
| 30.46667 | | 355.8286 | | | | 6.393813 | | | | 2556.32 | | | 90.53125 | |  | |  | |  |
| 30.475 | | 355.9185 | | | | 6.394673 | | | | 2555.578 | | | 90.30469 | |  | |  | |  |
| 30.48333 | | 356.0061 | | | | 6.397276 | | | | 2554.773 | | | 90.32031 | |  | |  | |  |
| 30.49167 | | 356.0975 | | | | 6.396209 | | | | 2554.039 | | | 90.3125 | |  | |  | |  |
| 30.5 | | 356.1888 | | | | 6.396747 | | | | 2553.328 | | | 90.02344 | |  | |  | |  |
| 30.50833 | | 356.2772 | | | | 6.399441 | | | | 2552.578 | | | 90.03125 | |  | |  | |  |
| 30.51667 | | 356.3678 | | | | 6.398151 | | | | 2551.813 | | | 89.80469 | |  | |  | |  |
| 30.525 | | 356.4581 | | | | 6.398537 | | | | 2551.094 | | | 89.75781 | |  | |  | |  |
| 30.53333 | | 356.5474 | | | | 6.400607 | | | | 2550.328 | | | 89.57031 | |  | |  | |  |
| 30.54167 | | 356.6382 | | | | 6.400815 | | | | 2549.586 | | | 89.38281 | |  | |  | |  |
| 30.55 | | 356.729 | | | | 6.39988 | | | | 2548.875 | | | 89.29688 | |  | |  | |  |
| 30.55833 | | 356.8184 | | | | 6.401656 | | | | 2548.117 | | | 89.32031 | |  | |  | |  |
| 30.56667 | | 356.9114 | | | | 6.402439 | | | | 2547.398 | | | 89.19531 | |  | |  | |  |
| 30.575 | | 357.0067 | | | | 6.401033 | | | | 2546.648 | | | 88.90625 | |  | |  | |  |
| 30.58333 | | 357.0975 | | | | 6.401805 | | | | 2545.922 | | | 88.92969 | |  | |  | |  |
| 30.59167 | | 357.1891 | | | | 6.402985 | | | | 2545.203 | | | 88.59375 | |  | |  | |  |
| 30.6 | | 357.2776 | | | | 6.400966 | | | | 2544.477 | | | 88.48438 | |  | |  | |  |
| 30.60833 | | 357.367 | | | | 6.401258 | | | | 2543.727 | | | 88.19531 | |  | |  | |  |
| 30.61667 | | 357.4556 | | | | 6.404117 | | | | 2543.008 | | | 88.50781 | |  | |  | |  |
| 30.625 | | 357.5435 | | | | 6.402183 | | | | 2542.289 | | | 88.76563 | |  | |  | |  |
| 30.63333 | | 357.6343 | | | | 6.402289 | | | | 2541.555 | | | 88.64844 | |  | |  | |  |
| 30.64167 | | 357.7251 | | | | 6.405166 | | | | 2540.852 | | | 88.59375 | |  | |  | |  |
| 30.65 | | 357.8137 | | | | 6.403515 | | | | 2540.125 | | | 88.78125 | |  | |  | |  |
| 30.65833 | | 357.903 | | | | 6.403372 | | | | 2539.422 | | | 88.95313 | |  | |  | |  |
| 30.66667 | | 357.9938 | | | | 6.404845 | | | | 2538.641 | | | 89.30469 | |  | |  | |  |
| 30.675 | | 358.0847 | | | | 6.402987 | | | | 2537.867 | | | 89.36719 | |  | |  | |  |
| 30.68333 | | 358.1762 | | | | 6.402266 | | | | 2537.125 | | | 89.35938 | |  | |  | |  |
| 30.69167 | | 358.2678 | | | | 6.404534 | | | | 2536.391 | | | 89.50781 | |  | |  | |  |
| 30.7 | | 358.3608 | | | | 6.403668 | | | | 2535.625 | | | 89.39063 | |  | |  | |  |
| 30.70833 | | 358.4516 | | | | 6.403227 | | | | 2534.852 | | | 89.14063 | |  | |  | |  |
| 30.71667 | | 358.5424 | | | | 6.406476 | | | | 2534.063 | | | 89.11719 | |  | |  | |  |
| 30.725 | | 358.6355 | | | | 6.406463 | | | | 2533.305 | | | 89.07813 | |  | |  | |  |
| 30.73333 | | 358.7263 | | | | 6.405869 | | | | 2532.555 | | | 89.03125 | |  | |  | |  |
| 30.74167 | | 358.8134 | | | | 6.407098 | | | | 2531.789 | | | 88.875 | |  | |  | |  |
| 30.75 | | 358.9028 | | | | 6.407606 | | | | 2531.063 | | | 88.59375 | |  | |  | |  |
| 30.75833 | | 358.9914 | | | | 6.40609 | | | | 2530.359 | | | 88.57031 | |  | |  | |  |
| 30.76667 | | 359.0815 | | | | 6.407531 | | | | 2529.625 | | | 88.38281 | |  | |  | |  |
| 30.775 | | 359.17 | | | | 6.408102 | | | | 2528.883 | | | 88.36719 | |  | |  | |  |
| 30.78333 | | 359.2579 | | | | 6.406091 | | | | 2528.148 | | | 88.21094 | |  | |  | |  |
| 30.79167 | | 359.3495 | | | | 6.406986 | | | | 2527.422 | | | 88.13281 | |  | |  | |  |
| 30.8 | | 359.4366 | | | | 6.40791 | | | | 2526.727 | | | 88.21094 | |  | |  | |  |
| 30.80833 | | 359.5252 | | | | 6.407094 | | | | 2525.992 | | | 88.26563 | |  | |  | |  |
| 30.81667 | | 359.616 | | | | 6.407596 | | | | 2525.281 | | | 88.21875 | |  | |  | |  |
| 30.825 | | 359.7068 | | | | 6.408315 | | | | 2524.539 | | | 88.07813 | |  | |  | |  |
| 30.83333 | | 359.7976 | | | | 6.407326 | | | | 2523.82 | | | 88.17188 | |  | |  | |  |
| 30.84167 | | 359.8877 | | | | 6.406048 | | | | 2523.102 | | | 88.35156 | |  | |  | |  |
| 30.85 | | 359.9785 | | | | 6.408077 | | | | 2522.352 | | | 88.44531 | |  | |  | |  |
| 30.85833 | | 360.0694 | | | | 6.407829 | | | | 2521.602 | | | 88.35156 | |  | |  | |  |
| 30.86667 | | 360.1572 | | | | 6.407076 | | | | 2520.875 | | | 88.50781 | |  | |  | |  |
| 30.875 | | 360.2473 | | | | 6.409911 | | | | 2520.164 | | | 88.69531 | |  | |  | |  |
| 30.88333 | | 360.3374 | | | | 6.409466 | | | | 2519.414 | | | 88.52344 | |  | |  | |  |
| 30.89167 | | 360.4252 | | | | 6.408519 | | | | 2518.656 | | | 88.33594 | |  | |  | |  |
| 30.9 | | 360.5161 | | | | 6.410287 | | | | 2517.898 | | | 88.20313 | |  | |  | |  |
| 30.90833 | | 360.6084 | | | | 6.409979 | | | | 2517.18 | | | 88.21094 | |  | |  | |  |
| 30.91667 | | 360.6947 | | | | 6.410134 | | | | 2516.414 | | | 88.14844 | |  | |  | |  |
| 30.925 | | 360.7826 | | | | 6.411926 | | | | 2515.656 | | | 87.9375 | |  | |  | |  |
| 30.93333 | | 360.8734 | | | | 6.411259 | | | | 2514.938 | | | 87.6875 | |  | |  | |  |
| 30.94167 | | 360.9628 | | | | 6.410864 | | | | 2514.234 | | | 87.5 | |  | |  | |  |
| 30.95 | | 361.058 | | | | 6.412873 | | | | 2513.523 | | | 87.25781 | |  | |  | |  |
| 30.95833 | | 361.1466 | | | | 6.41354 | | | | 2512.781 | | | 87.65625 | |  | |  | |  |
| 30.96667 | | 361.2367 | | | | 6.411308 | | | | 2512.055 | | | 87.26563 | |  | |  | |  |
| 30.975 | | 361.3268 | | | | 6.412863 | | | | 2511.359 | | | 87.03906 | |  | |  | |  |
| 30.98333 | | 361.4146 | | | | 6.413868 | | | | 2510.656 | | | 86.875 | |  | |  | |  |
| 30.99167 | | 361.501 | | | | 6.412039 | | | | 2509.961 | | | 86.77344 | |  | |  | |  |
| 31 | | 361.5926 | | | | 6.413493 | | | | 2509.266 | | | 86.60938 | |  | |  | |  |
| 31.00833 | | 361.6849 | | | | 6.414527 | | | | 2508.484 | | | 86.39063 | |  | |  | |  |
| 31.01667 | | 361.7727 | | | | 6.412078 | | | | 2507.805 | | | 86.45313 | |  | |  | |  |
| 31.025 | | 361.865 | | | | 6.413093 | | | | 2507.102 | | | 86.5 | |  | |  | |  |
| 31.03333 | | 361.9514 | | | | 6.414081 | | | | 2506.406 | | | 86.48438 | |  | |  | |  |
| 31.04167 | | 362.0415 | | | | 6.410967 | | | | 2505.688 | | | 85.80469 | |  | |  | |  |
| 31.05 | | 362.1353 | | | | 6.412046 | | | | 2504.992 | | | 85.875 | |  | |  | |  |
| 31.05833 | | 362.2276 | | | | 6.413567 | | | | 2504.289 | | | 86.03906 | |  | |  | |  |
| 31.06667 | | 362.3162 | | | | 6.41066 | | | | 2503.555 | | | 85.82813 | |  | |  | |  |
| 31.075 | | 362.4063 | | | | 6.41124 | | | | 2502.82 | | | 85.74219 | |  | |  | |  |
| 31.08333 | | 362.4941 | | | | 6.412912 | | | | 2502.109 | | | 85.67188 | |  | |  | |  |
| 31.09167 | | 362.5849 | | | | 6.410588 | | | | 2501.477 | | | 85.67188 | |  | |  | |  |
| 31.1 | | 362.6735 | | | | 6.411642 | | | | 2500.75 | | | 85.71875 | |  | |  | |  |
| 31.10833 | | 362.7629 | | | | 6.413708 | | | | 2500.016 | | | 85.64844 | |  | |  | |  |
| 31.11667 | | 362.8552 | | | | 6.41178 | | | | 2499.32 | | | 85.73438 | |  | |  | |  |
| 31.125 | | 362.9482 | | | | 6.412536 | | | | 2498.617 | | | 85.40625 | |  | |  | |  |
| 31.13333 | | 363.0338 | | | | 6.415347 | | | | 2497.914 | | | 85.47656 | |  | |  | |  |
| 31.14167 | | 363.1217 | | | | 6.414103 | | | | 2497.195 | | | 85.35938 | |  | |  | |  |
| 31.15 | | 363.2148 | | | | 6.415183 | | | | 2496.484 | | | 85.46094 | |  | |  | |  |
| 31.15833 | | 363.3078 | | | | 6.416988 | | | | 2495.773 | | | 85.35156 | |  | |  | |  |
| 31.16667 | | 363.4023 | | | | 6.416313 | | | | 2495.055 | | | 85.32813 | |  | |  | |  |
| 31.175 | | 363.4938 | | | | 6.414954 | | | | 2494.375 | | | 85.32813 | |  | |  | |  |
| 31.18333 | | 363.5847 | | | | 6.415002 | | | | 2493.648 | | | 85.26563 | |  | |  | |  |
| 31.19167 | | 363.6711 | | | | 6.415605 | | | | 2492.961 | | | 85.14063 | |  | |  | |  |
| 31.2 | | 363.7604 | | | | 6.414602 | | | | 2492.227 | | | 85.03125 | |  | |  | |  |
| 31.20833 | | 363.8512 | | | | 6.416994 | | | | 2491.523 | | | 85.41406 | |  | |  | |  |
| 31.21667 | | 363.9442 | | | | 6.41923 | | | | 2490.813 | | | 85.22656 | |  | |  | |  |
| 31.225 | | 364.0351 | | | | 6.418131 | | | | 2490.102 | | | 85.21875 | |  | |  | |  |
| 31.23333 | | 364.1244 | | | | 6.419252 | | | | 2489.398 | | | 85.32813 | |  | |  | |  |
| 31.24167 | | 364.2159 | | | | 6.420937 | | | | 2488.695 | | | 85.23438 | |  | |  | |  |
| 31.25 | | 364.3023 | | | | 6.418731 | | | | 2488.008 | | | 85.28125 | |  | |  | |  |
| 31.25833 | | 364.3895 | | | | 6.419323 | | | | 2487.234 | | | 84.97656 | |  | |  | |  |
| 31.26667 | | 364.4803 | | | | 6.419771 | | | | 2486.547 | | | 84.79688 | |  | |  | |  |
| 31.275 | | 364.5735 | | | | 6.4197 | | | | 2485.82 | | | 84.75 | |  | |  | |  |
| 31.28333 | | 364.661 | | | | 6.420647 | | | | 2485.102 | | | 84.625 | |  | |  | |  |
| 31.29167 | | 364.747 | | | | 6.422243 | | | | 2484.398 | | | 84.42188 | |  | |  | |  |
| 31.3 | | 364.8331 | | | | 6.420634 | | | | 2483.688 | | | 84.23438 | |  | |  | |  |
| 31.30833 | | 364.9199 | | | | 6.420811 | | | | 2483.016 | | | 83.75 | |  | |  | |  |
| 31.31667 | | 365.0081 | | | | 6.42207 | | | | 2482.336 | | | 83.61719 | |  | |  | |  |
| 31.325 | | 365.0949 | | | | 6.420912 | | | | 2481.641 | | | 83.44531 | |  | |  | |  |
| 31.33333 | | 365.1868 | | | | 6.421189 | | | | 2480.945 | | | 83.32813 | |  | |  | |  |
| 31.34167 | | 365.275 | | | | 6.422991 | | | | 2480.281 | | | 83.1875 | |  | |  | |  |
| 31.35 | | 365.3647 | | | | 6.421844 | | | | 2479.602 | | | 83.19531 | |  | |  | |  |
| 31.35833 | | 365.4544 | | | | 6.420691 | | | | 2478.961 | | | 83.25 | |  | |  | |  |
| 31.36667 | | 365.5455 | | | | 6.422467 | | | | 2478.281 | | | 83.21094 | |  | |  | |  |
| 31.375 | | 365.633 | | | | 6.420634 | | | | 2477.617 | | | 83.03125 | |  | |  | |  |
| 31.38333 | | 365.7227 | | | | 6.419384 | | | | 2476.938 | | | 83.07031 | |  | |  | |  |
| 31.39167 | | 365.8124 | | | | 6.4222 | | | | 2476.258 | | | 83.42188 | |  | |  | |  |
| 31.4 | | 365.9006 | | | | 6.421997 | | | | 2475.563 | | | 83.28125 | |  | |  | |  |
| 31.40833 | | 365.9881 | | | | 6.420649 | | | | 2474.859 | | | 83.375 | |  | |  | |  |
| 31.41667 | | 366.0764 | | | | 6.424213 | | | | 2474.164 | | | 83.28906 | |  | |  | |  |
| 31.425 | | 366.166 | | | | 6.42537 | | | | 2473.5 | | | 83.47656 | |  | |  | |  |
| 31.43333 | | 366.2521 | | | | 6.424245 | | | | 2472.797 | | | 83.6875 | |  | |  | |  |
| 31.44167 | | 366.3382 | | | | 6.42789 | | | | 2472.063 | | | 83.53125 | |  | |  | |  |
| 31.45 | | 366.4264 | | | | 6.427896 | | | | 2471.375 | | | 83.3125 | |  | |  | |  |
| 31.45833 | | 366.5168 | | | | 6.425797 | | | | 2470.664 | | | 83.23438 | |  | |  | |  |
| 31.46667 | | 366.605 | | | | 6.427945 | | | | 2469.984 | | | 83.28125 | |  | |  | |  |
| 31.475 | | 366.6933 | | | | 6.427968 | | | | 2469.258 | | | 83.4375 | |  | |  | |  |
| 31.48333 | | 366.7837 | | | | 6.425784 | | | | 2468.539 | | | 83.46875 | |  | |  | |  |
| 31.49167 | | 366.8727 | | | | 6.428366 | | | | 2467.859 | | | 83.5625 | |  | |  | |  |
| 31.5 | | 366.9609 | | | | 6.428296 | | | | 2467.188 | | | 83.52344 | |  | |  | |  |
| 31.50833 | | 367.0498 | | | | 6.42529 | | | | 2466.508 | | | 83.10938 | |  | |  | |  |
| 31.51667 | | 367.1381 | | | | 6.427218 | | | | 2465.805 | | | 83.07031 | |  | |  | |  |
| 31.525 | | 367.227 | | | | 6.427584 | | | | 2465.086 | | | 82.94531 | |  | |  | |  |
| 31.53333 | | 367.3153 | | | | 6.424857 | | | | 2464.375 | | | 83 | |  | |  | |  |
| 31.54167 | | 367.4035 | | | | 6.425586 | | | | 2463.672 | | | 83.15625 | |  | |  | |  |
| 31.55 | | 367.4925 | | | | 6.427541 | | | | 2462.977 | | | 83.21875 | |  | |  | |  |
| 31.55833 | | 367.5829 | | | | 6.426129 | | | | 2462.336 | | | 82.80469 | |  | |  | |  |
| 31.56667 | | 367.6704 | | | | 6.426374 | | | | 2461.656 | | | 82.52344 | |  | |  | |  |
| 31.575 | | 367.7601 | | | | 6.428539 | | | | 2460.977 | | | 82.6875 | |  | |  | |  |
| 31.58333 | | 367.8483 | | | | 6.42835 | | | | 2460.273 | | | 82.79688 | |  | |  | |  |
| 31.59167 | | 367.9351 | | | | 6.428191 | | | | 2459.555 | | | 83 | |  | |  | |  |
| 31.6 | | 368.0247 | | | | 6.430143 | | | | 2458.852 | | | 82.875 | |  | |  | |  |
| 31.60833 | | 368.1137 | | | | 6.4299 | | | | 2458.219 | | | 83.08594 | |  | |  | |  |
| 31.61667 | | 368.202 | | | | 6.428674 | | | | 2457.563 | | | 83.07813 | |  | |  | |  |
| 31.625 | | 368.2895 | | | | 6.430627 | | | | 2456.852 | | | 82.96094 | |  | |  | |  |
| 31.63333 | | 368.3784 | | | | 6.430878 | | | | 2456.141 | | | 82.92188 | |  | |  | |  |
| 31.64167 | | 368.4681 | | | | 6.430341 | | | | 2455.414 | | | 83.09375 | |  | |  | |  |
| 31.65 | | 368.5571 | | | | 6.433162 | | | | 2454.742 | | | 83.125 | |  | |  | |  |
| 31.65833 | | 368.6453 | | | | 6.434172 | | | | 2454.023 | | | 82.97656 | |  | |  | |  |
| 31.66667 | | 368.7335 | | | | 6.433646 | | | | 2453.336 | | | 82.91406 | |  | |  | |  |
| 31.675 | | 368.8225 | | | | 6.435474 | | | | 2452.656 | | | 82.57031 | |  | |  | |  |
| 31.68333 | | 368.9122 | | | | 6.43697 | | | | 2451.969 | | | 82.625 | |  | |  | |  |
| 31.69167 | | 368.9997 | | | | 6.434809 | | | | 2451.258 | | | 82.39844 | |  | |  | |  |
| 31.7 | | 369.0872 | | | | 6.436956 | | | | 2450.563 | | | 82.44531 | |  | |  | |  |
| 31.70833 | | 369.1754 | | | | 6.439339 | | | | 2449.898 | | | 82.25 | |  | |  | |  |
| 31.71667 | | 369.2615 | | | | 6.436559 | | | | 2449.219 | | | 82.15625 | |  | |  | |  |
| 31.725 | | 369.3497 | | | | 6.437494 | | | | 2448.578 | | | 82 | |  | |  | |  |
| 31.73333 | | 369.4387 | | | | 6.439426 | | | | 2447.891 | | | 81.89844 | |  | |  | |  |
| 31.74167 | | 369.5269 | | | | 6.436271 | | | | 2447.234 | | | 81.78906 | |  | |  | |  |
| 31.75 | | 369.6137 | | | | 6.436102 | | | | 2446.539 | | | 81.65625 | |  | |  | |  |
| 31.75833 | | 369.7041 | | | | 6.437966 | | | | 2445.875 | | | 81.76563 | |  | |  | |  |
| 31.76667 | | 369.7931 | | | | 6.435612 | | | | 2445.203 | | | 81.82813 | |  | |  | |  |
| 31.775 | | 369.8828 | | | | 6.43632 | | | | 2444.539 | | | 81.78125 | |  | |  | |  |
| 31.78333 | | 369.9717 | | | | 6.438727 | | | | 2443.875 | | | 81.65625 | |  | |  | |  |
| 31.79167 | | 370.0607 | | | | 6.437332 | | | | 2443.203 | | | 81.71875 | |  | |  | |  |
| 31.8 | | 370.1533 | | | | 6.437856 | | | | 2442.531 | | | 81.72656 | |  | |  | |  |
| 31.80833 | | 370.2393 | | | | 6.440652 | | | | 2441.844 | | | 81.50781 | |  | |  | |  |
| 31.81667 | | 370.3268 | | | | 6.43945 | | | | 2441.148 | | | 81.50781 | |  | |  | |  |
| 31.825 | | 370.4129 | | | | 6.439184 | | | | 2440.469 | | | 81.5625 | |  | |  | |  |
| 31.83333 | | 370.5069 | | | | 6.441181 | | | | 2439.805 | | | 81.60938 | |  | |  | |  |
| 31.84167 | | 370.5952 | | | | 6.441327 | | | | 2439.117 | | | 81.74219 | |  | |  | |  |
| 31.85 | | 370.6826 | | | | 6.440944 | | | | 2438.438 | | | 81.625 | |  | |  | |  |
| 31.85833 | | 370.7694 | | | | 6.443317 | | | | 2437.781 | | | 81.71094 | |  | |  | |  |
| 31.86667 | | 370.8584 | | | | 6.442936 | | | | 2437.094 | | | 81.60156 | |  | |  | |  |
| 31.875 | | 370.9474 | | | | 6.442544 | | | | 2436.406 | | | 81.57031 | |  | |  | |  |
| 31.88333 | | 371.0334 | | | | 6.445058 | | | | 2435.719 | | | 81.29688 | |  | |  | |  |
| 31.89167 | | 371.1173 | | | | 6.444532 | | | | 2435.008 | | | 81.09375 | |  | |  | |  |
| 31.9 | | 371.2056 | | | | 6.443637 | | | | 2434.344 | | | 81.1875 | |  | |  | |  |
| 31.90833 | | 371.2996 | | | | 6.445162 | | | | 2433.656 | | | 81.03906 | |  | |  | |  |
| 31.91667 | | 371.3871 | | | | 6.445032 | | | | 2432.992 | | | 81.03906 | |  | |  | |  |
| 31.925 | | 371.476 | | | | 6.443719 | | | | 2432.32 | | | 80.79688 | |  | |  | |  |
| 31.93333 | | 371.5643 | | | | 6.444934 | | | | 2431.68 | | | 80.67969 | |  | |  | |  |
| 31.94167 | | 371.6533 | | | | 6.444352 | | | | 2431.016 | | | 80.69531 | |  | |  | |  |
| 31.95 | | 371.74 | | | | 6.442857 | | | | 2430.336 | | | 80.86719 | |  | |  | |  |
| 31.95833 | | 371.8275 | | | | 6.445538 | | | | 2429.68 | | | 80.64844 | |  | |  | |  |
| 31.96667 | | 371.9179 | | | | 6.445635 | | | | 2429.008 | | | 80.72656 | |  | |  | |  |
| 31.975 | | 372.0141 | | | | 6.444502 | | | | 2428.367 | | | 80.89063 | |  | |  | |  |
| 31.98333 | | 372.1038 | | | | 6.445989 | | | | 2427.703 | | | 80.90625 | |  | |  | |  |
| 31.99167 | | 372.1906 | | | | 6.448008 | | | | 2427.023 | | | 81.07813 | |  | |  | |  |
| 32 | | 372.2774 | | | | 6.447306 | | | | 2426.328 | | | 80.89844 | |  | |  | |  |
| 32.00833 | | 372.3664 | | | | 6.448294 | | | | 2425.68 | | | 80.85156 | |  | |  | |  |
| 32.01667 | | 372.4599 | | | | 6.45059 | | | | 2424.992 | | | 80.85938 | |  | |  | |  |
| 32.025 | | 372.552 | | | | 6.45036 | | | | 2424.297 | | | 80.74219 | |  | |  | |  |
| 32.03333 | | 372.6433 | | | | 6.449705 | | | | 2423.609 | | | 80.79688 | |  | |  | |  |
| 32.04167 | | 372.7331 | | | | 6.452515 | | | | 2422.914 | | | 80.97656 | |  | |  | |  |
| 32.05 | | 372.8237 | | | | 6.452126 | | | | 2422.266 | | | 81.04688 | |  | |  | |  |
| 32.05833 | | 372.9143 | | | | 6.450781 | | | | 2421.594 | | | 81.03906 | |  | |  | |  |
| 32.06667 | | 373.0085 | | | | 6.453027 | | | | 2420.922 | | | 81.03906 | |  | |  | |  |
| 32.075 | | 373.0976 | | | | 6.453713 | | | | 2420.266 | | | 80.79688 | |  | |  | |  |
| 32.08333 | | 373.1904 | | | | 6.451981 | | | | 2419.586 | | | 80.77344 | |  | |  | |  |
| 32.09167 | | 373.2802 | | | | 6.45342 | | | | 2418.891 | | | 80.92188 | |  | |  | |  |
| 32.1 | | 373.3628 | | | | 6.454115 | | | | 2418.203 | | | 80.85938 | |  | |  | |  |
| 32.10833 | | 373.4504 | | | | 6.452349 | | | | 2417.523 | | | 80.85156 | |  | |  | |  |
| 32.11667 | | 373.5403 | | | | 6.454681 | | | | 2416.859 | | | 80.57813 | |  | |  | |  |
| 32.125 | | 373.6324 | | | | 6.455759 | | | | 2416.211 | | | 80.47656 | |  | |  | |  |
| 32.13333 | | 373.7207 | | | | 6.455251 | | | | 2415.539 | | | 80.5 | |  | |  | |  |
| 32.14167 | | 373.8069 | | | | 6.456496 | | | | 2414.852 | | | 80.35156 | |  | |  | |  |
| 32.15 | | 373.8902 | | | | 6.458056 | | | | 2414.188 | | | 80.20313 | |  | |  | |  |
| 32.15833 | | 373.9793 | | | | 6.455894 | | | | 2413.508 | | | 80.24219 | |  | |  | |  |
| 32.16667 | | 374.0685 | | | | 6.45693 | | | | 2412.875 | | | 80.125 | |  | |  | |  |
| 32.175 | | 374.1576 | | | | 6.457848 | | | | 2412.219 | | | 80.10938 | |  | |  | |  |
| 32.18333 | | 374.2489 | | | | 6.456049 | | | | 2411.547 | | | 80.14063 | |  | |  | |  |
| 32.19167 | | 374.3372 | | | | 6.457746 | | | | 2410.891 | | | 79.95313 | |  | |  | |  |
| 32.2 | | 374.4286 | | | | 6.459779 | | | | 2410.242 | | | 80.125 | |  | |  | |  |
| 32.20833 | | 374.517 | | | | 6.459207 | | | | 2409.57 | | | 80.09375 | |  | |  | |  |
| 32.21667 | | 374.6068 | | | | 6.460683 | | | | 2408.914 | | | 79.8125 | |  | |  | |  |
| 32.225 | | 374.6981 | | | | 6.462659 | | | | 2408.25 | | | 79.80469 | |  | |  | |  |
| 32.23333 | | 374.7894 | | | | 6.461556 | | | | 2407.578 | | | 79.94531 | |  | |  | |  |
| 32.24167 | | 374.8829 | | | | 6.461474 | | | | 2406.93 | | | 79.89844 | |  | |  | |  |
| 32.25 | | 374.9713 | | | | 6.463104 | | | | 2406.234 | | | 79.9375 | |  | |  | |  |
| 32.25833 | | 375.0626 | | | | 6.462033 | | | | 2405.57 | | | 79.71875 | |  | |  | |  |
| 32.26667 | | 375.1525 | | | | 6.461831 | | | | 2404.945 | | | 79.60938 | |  | |  | |  |
| 32.275 | | 375.2438 | | | | 6.463989 | | | | 2404.273 | | | 79.65625 | |  | |  | |  |
| 32.28333 | | 375.3329 | | | | 6.465034 | | | | 2403.594 | | | 79.50781 | |  | |  | |  |
| 32.29167 | | 375.4235 | | | | 6.464067 | | | | 2402.938 | | | 79.48438 | |  | |  | |  |
| 32.3 | | 375.5126 | | | | 6.466267 | | | | 2402.266 | | | 79.50781 | |  | |  | |  |
| 32.30833 | | 375.6017 | | | | 6.468023 | | | | 2401.633 | | | 79.58594 | |  | |  | |  |
| 32.31667 | | 375.6938 | | | | 6.46569 | | | | 2400.984 | | | 79.54688 | |  | |  | |  |
| 32.325 | | 375.7814 | | | | 6.46753 | | | | 2400.313 | | | 79.70313 | |  | |  | |  |
| 32.33333 | | 375.8705 | | | | 6.468805 | | | | 2399.672 | | | 79.63281 | |  | |  | |  |
| 32.34167 | | 375.9589 | | | | 6.466727 | | | | 2399.016 | | | 79.72656 | |  | |  | |  |
| 32.35 | | 376.051 | | | | 6.468095 | | | | 2398.336 | | | 79.71875 | |  | |  | |  |
| 32.35833 | | 376.1408 | | | | 6.469354 | | | | 2397.664 | | | 79.3125 | |  | |  | |  |
| 32.36667 | | 376.2314 | | | | 6.467611 | | | | 2397.008 | | | 79.53906 | |  | |  | |  |
| 32.375 | | 376.3213 | | | | 6.469342 | | | | 2396.32 | | | 79.53125 | |  | |  | |  |
| 32.38333 | | 376.4126 | | | | 6.471574 | | | | 2395.664 | | | 79.41406 | |  | |  | |  |
| 32.39167 | | 376.5017 | | | | 6.468828 | | | | 2394.992 | | | 79.125 | |  | |  | |  |
| 32.4 | | 376.5872 | | | | 6.469762 | | | | 2394.32 | | | 78.95313 | |  | |  | |  |
| 32.40833 | | 376.6755 | | | | 6.472574 | | | | 2393.719 | | | 78.67188 | |  | |  | |  |
| 32.41667 | | 376.7698 | | | | 6.470749 | | | | 2393.016 | | | 78.57813 | |  | |  | |  |
| 32.425 | | 376.8596 | | | | 6.471699 | | | | 2392.359 | | | 78.53906 | |  | |  | |  |
| 32.43333 | | 376.9502 | | | | 6.475278 | | | | 2391.711 | | | 78.25 | |  | |  | |  |
| 32.44167 | | 377.0415 | | | | 6.474085 | | | | 2391.086 | | | 78.65625 | |  | |  | |  |
| 32.45 | | 377.1314 | | | | 6.474069 | | | | 2390.445 | | | 78.49219 | |  | |  | |  |
| 32.45833 | | 377.2205 | | | | 6.476392 | | | | 2389.836 | | | 78.50781 | |  | |  | |  |
| 32.46667 | | 377.3096 | | | | 6.475853 | | | | 2389.188 | | | 78.60156 | |  | |  | |  |
| 32.475 | | 377.3987 | | | | 6.475631 | | | | 2388.547 | | | 78.64844 | |  | |  | |  |
| 32.48333 | | 377.4879 | | | | 6.478461 | | | | 2387.922 | | | 78.70313 | |  | |  | |  |
| 32.49167 | | 377.5777 | | | | 6.47821 | | | | 2387.211 | | | 78.65625 | |  | |  | |  |
| 32.5 | | 377.6661 | | | | 6.477365 | | | | 2386.586 | | | 78.90625 | |  | |  | |  |
| 32.50833 | | 377.7552 | | | | 6.479676 | | | | 2385.922 | | | 78.89063 | |  | |  | |  |
| 32.51667 | | 377.8458 | | | | 6.479267 | | | | 2385.25 | | | 79.5 | |  | |  | |  |
| 32.525 | | 377.9356 | | | | 6.478274 | | | | 2384.594 | | | 79.42188 | |  | |  | |  |
| 32.53333 | | 378.0277 | | | | 6.47974 | | | | 2383.93 | | | 79.25781 | |  | |  | |  |
| 32.54167 | | 378.1183 | | | | 6.480256 | | | | 2383.273 | | | 79.14844 | |  | |  | |  |
| 32.55 | | 378.2081 | | | | 6.478743 | | | | 2382.578 | | | 79.03906 | |  | |  | |  |
| 32.55833 | | 378.2994 | | | | 6.479726 | | | | 2381.914 | | | 79.28125 | |  | |  | |  |
| 32.56667 | | 378.3914 | | | | 6.482532 | | | | 2381.172 | | | 79.24219 | |  | |  | |  |
| 32.575 | | 378.4813 | | | | 6.481357 | | | | 2380.539 | | | 79.36719 | |  | |  | |  |
| 32.58333 | | 378.5682 | | | | 6.482526 | | | | 2379.883 | | | 79.15625 | |  | |  | |  |
| 32.59167 | | 378.6581 | | | | 6.484444 | | | | 2379.242 | | | 79.21875 | |  | |  | |  |
| 32.6 | | 378.7443 | | | | 6.483442 | | | | 2378.594 | | | 78.74219 | |  | |  | |  |
| 32.60833 | | 378.8341 | | | | 6.484382 | | | | 2377.906 | | | 78.71875 | |  | |  | |  |
| 32.61667 | | 378.9196 | | | | 6.48612 | | | | 2377.25 | | | 78.80469 | |  | |  | |  |
| 32.625 | | 379.0109 | | | | 6.485494 | | | | 2376.57 | | | 78.82031 | |  | |  | |  |
| 32.63333 | | 379.1022 | | | | 6.486244 | | | | 2375.938 | | | 78.60938 | |  | |  | |  |
| 32.64167 | | 379.195 | | | | 6.488538 | | | | 2375.266 | | | 78.57031 | |  | |  | |  |
| 32.65 | | 379.2863 | | | | 6.487326 | | | | 2374.68 | | | 78.5625 | |  | |  | |  |
| 32.65833 | | 379.3761 | | | | 6.487021 | | | | 2374.023 | | | 78.39844 | |  | |  | |  |
| 32.66667 | | 379.466 | | | | 6.4905 | | | | 2373.359 | | | 78.375 | |  | |  | |  |
| 32.675 | | 379.558 | | | | 6.49078 | | | | 2372.695 | | | 78.21875 | |  | |  | |  |
| 32.68333 | | 379.653 | | | | 6.489705 | | | | 2372.07 | | | 78.20313 | |  | |  | |  |
| 32.69167 | | 379.7429 | | | | 6.492281 | | | | 2371.422 | | | 78.28125 | |  | |  | |  |
| 32.7 | | 379.832 | | | | 6.493021 | | | | 2370.773 | | | 78.25781 | |  | |  | |  |
| 32.70833 | | 379.916 | | | | 6.49122 | | | | 2370.133 | | | 78.20313 | |  | |  | |  |
| 32.71667 | | 380.0036 | | | | 6.493844 | | | | 2369.492 | | | 78.39844 | |  | |  | |  |
| 32.725 | | 380.0935 | | | | 6.4937 | | | | 2368.859 | | | 78.19531 | |  | |  | |  |
| 32.73333 | | 380.1797 | | | | 6.491857 | | | | 2368.195 | | | 78.35938 | |  | |  | |  |
| 32.74167 | | 380.2695 | | | | 6.495393 | | | | 2367.531 | | | 78.42188 | |  | |  | |  |
| 32.75 | | 380.363 | | | | 6.495938 | | | | 2366.883 | | | 78.33594 | |  | |  | |  |
| 32.75833 | | 380.4505 | | | | 6.493668 | | | | 2366.25 | | | 78.64063 | |  | |  | |  |
| 32.76667 | | 380.5371 | | | | 6.496764 | | | | 2365.563 | | | 78.69531 | |  | |  | |  |
| 32.775 | | 380.6251 | | | | 6.497313 | | | | 2364.93 | | | 78.57031 | |  | |  | |  |
| 32.78333 | | 380.716 | | | | 6.494944 | | | | 2364.266 | | | 78.4375 | |  | |  | |  |
| 32.79167 | | 380.8083 | | | | 6.496989 | | | | 2363.602 | | | 78.60156 | |  | |  | |  |
| 32.8 | | 380.8985 | | | | 6.498122 | | | | 2362.961 | | | 78.58594 | |  | |  | |  |
| 32.80833 | | 380.9837 | | | | 6.49634 | | | | 2362.266 | | | 78.75781 | |  | |  | |  |
| 32.81667 | | 381.076 | | | | 6.498407 | | | | 2361.609 | | | 78.79688 | |  | |  | |  |
| 32.825 | | 381.1691 | | | | 6.49993 | | | | 2360.969 | | | 78.875 | |  | |  | |  |
| 32.83333 | | 381.2578 | | | | 6.498779 | | | | 2360.344 | | | 79 | |  | |  | |  |
| 32.84167 | | 381.3502 | | | | 6.498627 | | | | 2359.648 | | | 78.83594 | |  | |  | |  |
| 32.85 | | 381.4382 | | | | 6.501251 | | | | 2359 | | | 78.76563 | |  | |  | |  |
| 32.85833 | | 381.5291 | | | | 6.50165 | | | | 2358.32 | | | 78.71094 | |  | |  | |  |
| 32.86667 | | 381.62 | | | | 6.5003 | | | | 2357.656 | | | 79.19531 | |  | |  | |  |
| 32.875 | | 381.7123 | | | | 6.502396 | | | | 2356.984 | | | 79 | |  | |  | |  |
| 32.88333 | | 381.8032 | | | | 6.503363 | | | | 2356.313 | | | 78.92969 | |  | |  | |  |
| 32.89167 | | 381.8919 | | | | 6.50132 | | | | 2355.68 | | | 78.72656 | |  | |  | |  |
| 32.9 | | 381.9821 | | | | 6.503251 | | | | 2355.031 | | | 78.51563 | |  | |  | |  |
| 32.90833 | | 382.0665 | | | | 6.504715 | | | | 2354.375 | | | 78.25781 | |  | |  | |  |
| 32.91667 | | 382.1545 | | | | 6.502455 | | | | 2353.641 | | | 78.10938 | |  | |  | |  |
| 32.925 | | 382.2404 | | | | 6.504801 | | | | 2353.016 | | | 78.00781 | |  | |  | |  |
| 32.93333 | | 382.3284 | | | | 6.507022 | | | | 2352.359 | | | 77.96875 | |  | |  | |  |
| 32.94167 | | 382.4178 | | | | 6.504425 | | | | 2351.742 | | | 77.96875 | |  | |  | |  |
| 32.95 | | 382.5052 | | | | 6.506284 | | | | 2351.117 | | | 77.625 | |  | |  | |  |
| 32.95833 | | 382.5924 | | | | 6.509205 | | | | 2350.492 | | | 77.71875 | |  | |  | |  |
| 32.96667 | | 382.6754 | | | | 6.507385 | | | | 2349.859 | | | 77.80469 | |  | |  | |  |
| 32.975 | | 382.7656 | | | | 6.508818 | | | | 2349.219 | | | 77.79688 | |  | |  | |  |
| 32.98333 | | 382.8536 | | | | 6.511145 | | | | 2348.578 | | | 77.76563 | |  | |  | |  |
| 32.99167 | | 382.9438 | | | | 6.509382 | | | | 2347.93 | | | 77.85938 | |  | |  | |  |
| 33 | | 383.0339 | | | | 6.510203 | | | | 2347.344 | | | 78.03125 | |  | |  | |  |
| 33.00833 | | 383.1248 | | | | 6.512573 | | | | 2346.672 | | | 78.11719 | |  | |  | |  |
| 33.01667 | | 383.2136 | | | | 6.512448 | | | | 2346.016 | | | 78.10156 | |  | |  | |  |
| 33.025 | | 383.2987 | | | | 6.513371 | | | | 2345.367 | | | 78.24219 | |  | |  | |  |
| 33.03333 | | 383.3889 | | | | 6.516773 | | | | 2344.719 | | | 78.22656 | |  | |  | |  |
| 33.04167 | | 383.4783 | | | | 6.516074 | | | | 2344.055 | | | 78.08594 | |  | |  | |  |
| 33.05 | | 383.5736 | | | | 6.51608 | | | | 2343.391 | | | 78.20313 | |  | |  | |  |
| 33.05833 | | 383.6616 | | | | 6.518431 | | | | 2342.719 | | | 78.32813 | |  | |  | |  |
| 33.06667 | | 383.7496 | | | | 6.518551 | | | | 2342.078 | | | 77.92969 | |  | |  | |  |
| 33.075 | | 383.8354 | | | | 6.518348 | | | | 2341.406 | | | 77.85938 | |  | |  | |  |
| 33.08333 | | 383.9213 | | | | 6.521435 | | | | 2340.758 | | | 77.80469 | |  | |  | |  |
| 33.09167 | | 384.0086 | | | | 6.521266 | | | | 2340.117 | | | 77.64063 | |  | |  | |  |
| 33.1 | | 384.1002 | | | | 6.521193 | | | | 2339.453 | | | 77.65625 | |  | |  | |  |
| 33.10833 | | 384.1911 | | | | 6.52269 | | | | 2338.781 | | | 77.53906 | |  | |  | |  |
| 33.11667 | | 384.2762 | | | | 6.524052 | | | | 2338.18 | | | 77.69531 | |  | |  | |  |
| 33.125 | | 384.3621 | | | | 6.523043 | | | | 2337.547 | | | 77.60156 | |  | |  | |  |
| 33.13333 | | 384.4494 | | | | 6.524929 | | | | 2336.914 | | | 77.53906 | |  | |  | |  |
| 33.14167 | | 384.541 | | | | 6.525553 | | | | 2336.281 | | | 77.67969 | |  | |  | |  |
| 33.15 | | 384.629 | | | | 6.524279 | | | | 2335.633 | | | 78.19531 | |  | |  | |  |
| 33.15833 | | 384.7199 | | | | 6.525826 | | | | 2334.992 | | | 78.52344 | |  | |  | |  |
| 33.16667 | | 384.8123 | | | | 6.527502 | | | | 2334.328 | | | 78.78906 | |  | |  | |  |
| 33.175 | | 384.9024 | | | | 6.52613 | | | | 2333.688 | | | 78.9375 | |  | |  | |  |
| 33.18333 | | 384.9926 | | | | 6.526231 | | | | 2333.055 | | | 79.10938 | |  | |  | |  |
| 33.19167 | | 385.0792 | | | | 6.529476 | | | | 2332.383 | | | 79.07813 | |  | |  | |  |
| 33.2 | | 385.173 | | | | 6.52877 | | | | 2331.656 | | | 78.96094 | |  | |  | |  |
| 33.20833 | | 385.2682 | | | | 6.52824 | | | | 2330.961 | | | 79.10156 | |  | |  | |  |
| 33.21667 | | 385.3598 | | | | 6.531646 | | | | 2330.258 | | | 79.0625 | |  | |  | |  |
| 33.225 | | 385.4471 | | | | 6.531284 | | | | 2329.578 | | | 79.01563 | |  | |  | |  |
| 33.23333 | | 385.5359 | | | | 6.530258 | | | | 2328.898 | | | 78.98438 | |  | |  | |  |
| 33.24167 | | 385.6275 | | | | 6.533787 | | | | 2328.25 | | | 78.77344 | |  | |  | |  |
| 33.25 | | 385.7155 | | | | 6.533713 | | | | 2327.602 | | | 78.5 | |  | |  | |  |
| 33.25833 | | 385.8078 | | | | 6.532676 | | | | 2326.922 | | | 78.53906 | |  | |  | |  |
| 33.26667 | | 385.8922 | | | | 6.536606 | | | | 2326.258 | | | 78.28906 | |  | |  | |  |
| 33.275 | | 385.986 | | | | 6.536241 | | | | 2325.609 | | | 78.30469 | |  | |  | |  |
| 33.28333 | | 386.0733 | | | | 6.533951 | | | | 2324.961 | | | 78.29688 | |  | |  | |  |
| 33.29167 | | 386.1555 | | | | 6.536767 | | | | 2324.344 | | | 78.29688 | |  | |  | |  |
| 33.3 | | 386.2414 | | | | 6.535593 | | | | 2323.719 | | | 78.22656 | |  | |  | |  |
| 33.30833 | | 386.3323 | | | | 6.533952 | | | | 2323.078 | | | 78.1875 | |  | |  | |  |
| 33.31667 | | 386.4203 | | | | 6.537167 | | | | 2322.445 | | | 78.21094 | |  | |  | |  |
| 33.325 | | 386.5069 | | | | 6.537122 | | | | 2321.805 | | | 78.3125 | |  | |  | |  |
| 33.33333 | | 386.5949 | | | | 6.535597 | | | | 2321.148 | | | 78.32813 | |  | |  | |  |
| 33.34167 | | 386.6786 | | | | 6.539265 | | | | 2320.5 | | | 78.22656 | |  | |  | |  |
| 33.35 | | 386.7716 | | | | 6.539408 | | | | 2319.867 | | | 78.42969 | |  | |  | |  |
| 33.35833 | | 386.8582 | | | | 6.537621 | | | | 2319.219 | | | 78.47656 | |  | |  | |  |
| 33.36667 | | 386.9484 | | | | 6.540395 | | | | 2318.555 | | | 78.47656 | |  | |  | |  |
| 33.375 | | 387.0451 | | | | 6.541211 | | | | 2317.891 | | | 78.29688 | |  | |  | |  |
| 33.38333 | | 387.1374 | | | | 6.54042 | | | | 2317.234 | | | 78.29688 | |  | |  | |  |
| 33.39167 | | 387.2261 | | | | 6.541804 | | | | 2316.602 | | | 78.25781 | |  | |  | |  |
| 33.4 | | 387.3178 | | | | 6.54426 | | | | 2315.914 | | | 77.90625 | |  | |  | |  |
| 33.40833 | | 387.4058 | | | | 6.544193 | | | | 2315.25 | | | 77.79688 | |  | |  | |  |
| 33.41667 | | 387.4945 | | | | 6.54628 | | | | 2314.602 | | | 77.76563 | |  | |  | |  |
| 33.425 | | 387.5934 | | | | 6.548784 | | | | 2313.977 | | | 77.89844 | |  | |  | |  |
| 33.43333 | | 387.6828 | | | | 6.54931 | | | | 2313.32 | | | 77.83594 | |  | |  | |  |
| 33.44167 | | 387.7715 | | | | 6.548977 | | | | 2312.68 | | | 77.75781 | |  | |  | |  |
| 33.45 | | 387.8617 | | | | 6.551886 | | | | 2312.063 | | | 77.82813 | |  | |  | |  |
| 33.45833 | | 387.9505 | | | | 6.552936 | | | | 2311.438 | | | 77.95313 | |  | |  | |  |
| 33.46667 | | 388.0406 | | | | 6.550262 | | | | 2310.789 | | | 78 | |  | |  | |  |
| 33.475 | | 388.1279 | | | | 6.552751 | | | | 2310.133 | | | 78.00781 | |  | |  | |  |
| 33.48333 | | 388.2188 | | | | 6.554697 | | | | 2309.492 | | | 78.01563 | |  | |  | |  |
| 33.49167 | | 388.3126 | | | | 6.553037 | | | | 2308.859 | | | 78.08594 | |  | |  | |  |
| 33.5 | | 388.405 | | | | 6.555461 | | | | 2308.195 | | | 78.05469 | |  | |  | |  |
| 33.50833 | | 388.4886 | | | | 6.5578 | | | | 2307.531 | | | 77.96875 | |  | |  | |  |
| 33.51667 | | 388.5745 | | | | 6.55599 | | | | 2306.867 | | | 77.92969 | |  | |  | |  |
| 33.525 | | 388.6654 | | | | 6.559262 | | | | 2306.227 | | | 77.89063 | |  | |  | |  |
| 33.53333 | | 388.7505 | | | | 6.561675 | | | | 2305.586 | | | 77.70313 | |  | |  | |  |
| 33.54167 | | 388.8342 | | | | 6.559098 | | | | 2304.914 | | | 77.9375 | |  | |  | |  |
| 33.55 | | 388.9215 | | | | 6.560433 | | | | 2304.258 | | | 77.94531 | |  | |  | |  |
| 33.55833 | | 389.0124 | | | | 6.562287 | | | | 2303.609 | | | 77.99219 | |  | |  | |  |
| 33.56667 | | 389.0982 | | | | 6.559571 | | | | 2302.961 | | | 78.125 | |  | |  | |  |
| 33.575 | | 389.1862 | | | | 6.560277 | | | | 2302.32 | | | 78.3125 | |  | |  | |  |
| 33.58333 | | 389.2743 | | | | 6.562813 | | | | 2301.688 | | | 78.40625 | |  | |  | |  |
| 33.59167 | | 389.3623 | | | | 6.561897 | | | | 2301.008 | | | 78.42969 | |  | |  | |  |
| 33.6 | | 389.4532 | | | | 6.563546 | | | | 2300.359 | | | 78.34375 | |  | |  | |  |
| 33.60833 | | 389.5441 | | | | 6.566196 | | | | 2299.695 | | | 78.53125 | |  | |  | |  |
| 33.61667 | | 389.635 | | | | 6.565296 | | | | 2299.023 | | | 78.67969 | |  | |  | |  |
| 33.625 | | 389.7259 | | | | 6.565355 | | | | 2298.344 | | | 78.5 | |  | |  | |  |
| 33.63333 | | 389.8175 | | | | 6.568011 | | | | 2297.688 | | | 78.63281 | |  | |  | |  |
| 33.64167 | | 389.907 | | | | 6.568098 | | | | 2297.023 | | | 78.64844 | |  | |  | |  |
| 33.65 | | 389.9986 | | | | 6.567455 | | | | 2296.391 | | | 78.51563 | |  | |  | |  |
| 33.65833 | | 390.0888 | | | | 6.569535 | | | | 2295.711 | | | 78.22656 | |  | |  | |  |
| 33.66667 | | 390.1761 | | | | 6.570994 | | | | 2295.031 | | | 78.09375 | |  | |  | |  |
| 33.675 | | 390.2662 | | | | 6.570413 | | | | 2294.406 | | | 77.97656 | |  | |  | |  |
| 33.68333 | | 390.3564 | | | | 6.572121 | | | | 2293.734 | | | 77.96875 | |  | |  | |  |
| 33.69167 | | 390.4437 | | | | 6.573186 | | | | 2293.078 | | | 77.875 | |  | |  | |  |
| 33.7 | | 390.5324 | | | | 6.572133 | | | | 2292.445 | | | 77.55469 | |  | |  | |  |
| 33.70833 | | 390.6233 | | | | 6.573026 | | | | 2291.828 | | | 77.5 | |  | |  | |  |
| 33.71667 | | 390.7099 | | | | 6.57399 | | | | 2291.203 | | | 77.16406 | |  | |  | |  |
| 33.725 | | 390.7994 | | | | 6.572582 | | | | 2290.57 | | | 77.03125 | |  | |  | |  |
| 33.73333 | | 390.8896 | | | | 6.573737 | | | | 2289.914 | | | 77.21094 | |  | |  | |  |
| 33.74167 | | 390.9783 | | | | 6.57721 | | | | 2289.281 | | | 77.21875 | |  | |  | |  |
| 33.75 | | 391.075 | | | | 6.576108 | | | | 2288.672 | | | 77.49219 | |  | |  | |  |
| 33.75833 | | 391.1666 | | | | 6.575522 | | | | 2288.039 | | | 77.46094 | |  | |  | |  |
| 33.76667 | | 391.2546 | | | | 6.578109 | | | | 2287.445 | | | 77.44531 | |  | |  | |  |
| 33.775 | | 391.3448 | | | | 6.576584 | | | | 2286.813 | | | 77.6875 | |  | |  | |  |
| 33.78333 | | 391.4342 | | | | 6.576597 | | | | 2286.148 | | | 77.8125 | |  | |  | |  |
| 33.79167 | | 391.5229 | | | | 6.579171 | | | | 2285.5 | | | 77.82813 | |  | |  | |  |
| 33.8 | | 391.611 | | | | 6.5795 | | | | 2284.828 | | | 78.0625 | |  | |  | |  |
| 33.80833 | | 391.7019 | | | | 6.579187 | | | | 2284.18 | | | 78.17188 | |  | |  | |  |
| 33.81667 | | 391.787 | | | | 6.581882 | | | | 2283.539 | | | 78.03125 | |  | |  | |  |
| 33.825 | | 391.8793 | | | | 6.582502 | | | | 2282.859 | | | 78.03125 | |  | |  | |  |
| 33.83333 | | 391.9659 | | | | 6.581356 | | | | 2282.195 | | | 77.85938 | |  | |  | |  |
| 33.84167 | | 392.0547 | | | | 6.584218 | | | | 2281.547 | | | 78.04688 | |  | |  | |  |
| 33.85 | | 392.1427 | | | | 6.585258 | | | | 2280.859 | | | 78.04688 | |  | |  | |  |
| 33.85833 | | 392.2307 | | | | 6.58432 | | | | 2280.188 | | | 77.64844 | |  | |  | |  |
| 33.86667 | | 392.3194 | | | | 6.585457 | | | | 2279.547 | | | 77.60156 | |  | |  | |  |
| 33.875 | | 392.4067 | | | | 6.586964 | | | | 2278.906 | | | 77.48438 | |  | |  | |  |
| 33.88333 | | 392.4969 | | | | 6.585884 | | | | 2278.273 | | | 77.40625 | |  | |  | |  |
| 33.89167 | | 392.5863 | | | | 6.586466 | | | | 2277.602 | | | 77.08594 | |  | |  | |  |
| 33.9 | | 392.6794 | | | | 6.58903 | | | | 2276.953 | | | 76.92188 | |  | |  | |  |
| 33.90833 | | 392.7653 | | | | 6.588067 | | | | 2276.359 | | | 76.92188 | |  | |  | |  |
| 33.91667 | | 392.8496 | | | | 6.589913 | | | | 2275.727 | | | 76.89063 | |  | |  | |  |
| 33.925 | | 392.9348 | | | | 6.591417 | | | | 2275.086 | | | 76.65625 | |  | |  | |  |
| 33.93333 | | 393.0286 | | | | 6.59108 | | | | 2274.461 | | | 76.39063 | |  | |  | |  |
| 33.94167 | | 393.1195 | | | | 6.593467 | | | | 2273.867 | | | 76.5625 | |  | |  | |  |
| 33.95 | | 393.2097 | | | | 6.596165 | | | | 2273.234 | | | 76.67969 | |  | |  | |  |
| 33.95833 | | 393.3005 | | | | 6.596105 | | | | 2272.594 | | | 76.75 | |  | |  | |  |
| 33.96667 | | 393.3914 | | | | 6.597714 | | | | 2271.961 | | | 76.71094 | |  | |  | |  |
| 33.975 | | 393.4816 | | | | 6.600253 | | | | 2271.344 | | | 76.77344 | |  | |  | |  |
| 33.98333 | | 393.5754 | | | | 6.599791 | | | | 2270.742 | | | 77.07813 | |  | |  | |  |
| 33.99167 | | 393.6678 | | | | 6.597549 | | | | 2270.07 | | | 77.25 | |  | |  | |  |
| 34 | | 393.7594 | | | | 6.600556 | | | | 2269.422 | | | 77.54688 | |  | |  | |  |
| 34.00833 | | 393.8531 | | | | 6.601525 | | | | 2268.766 | | | 77.85938 | |  | |  | |  |
| 34.01667 | | 393.9469 | | | | 6.600521 | | | | 2268.125 | | | 78.23438 | |  | |  | |  |
| 34.025 | | 394.0414 | | | | 6.602026 | | | | 2267.477 | | | 78.49219 | |  | |  | |  |
| 34.03333 | | 394.128 | | | | 6.604335 | | | | 2266.797 | | | 78.61719 | |  | |  | |  |
| 34.04167 | | 394.2146 | | | | 6.603403 | | | | 2266.125 | | | 78.96094 | |  | |  | |  |
| 34.05 | | 394.3033 | | | | 6.605572 | | | | 2265.438 | | | 79.07031 | |  | |  | |  |
| 34.05833 | | 394.3957 | | | | 6.607351 | | | | 2264.75 | | | 79.27344 | |  | |  | |  |
| 34.06667 | | 394.4866 | | | | 6.606404 | | | | 2264.039 | | | 79.29688 | |  | |  | |  |
| 34.075 | | 394.5768 | | | | 6.607378 | | | | 2263.359 | | | 79.39844 | |  | |  | |  |
| 34.08333 | | 394.6619 | | | | 6.609943 | | | | 2262.68 | | | 79.40625 | |  | |  | |  |
| 34.09167 | | 394.7506 | | | | 6.608563 | | | | 2261.984 | | | 79.40625 | |  | |  | |  |
| 34.1 | | 394.8394 | | | | 6.610397 | | | | 2261.313 | | | 79.28125 | |  | |  | |  |
| 34.10833 | | 394.9237 | | | | 6.613008 | | | | 2260.617 | | | 78.98438 | |  | |  | |  |
| 34.11667 | | 395.0168 | | | | 6.612028 | | | | 2259.969 | | | 78.73438 | |  | |  | |  |
| 34.125 | | 395.1135 | | | | 6.612864 | | | | 2259.289 | | | 78.44531 | |  | |  | |  |
| 34.13333 | | 395.2037 | | | | 6.614637 | | | | 2258.625 | | | 78.5 | |  | |  | |  |
| 34.14167 | | 395.2888 | | | | 6.614249 | | | | 2257.969 | | | 78.42188 | |  | |  | |  |
| 34.15 | | 395.3754 | | | | 6.613653 | | | | 2257.328 | | | 78.40625 | |  | |  | |  |
| 34.15833 | | 395.4641 | | | | 6.615399 | | | | 2256.711 | | | 78.3125 | |  | |  | |  |
| 34.16667 | | 395.5593 | | | | 6.615705 | | | | 2256.094 | | | 78.45313 | |  | |  | |  |
| 34.175 | | 395.6509 | | | | 6.613098 | | | | 2255.477 | | | 78.53125 | |  | |  | |  |
| 34.18333 | | 395.7404 | | | | 6.61523 | | | | 2254.813 | | | 78.48438 | |  | |  | |  |
| 34.19167 | | 395.8284 | | | | 6.617462 | | | | 2254.172 | | | 78.67188 | |  | |  | |  |
| 34.2 | | 395.9251 | | | | 6.615648 | | | | 2253.516 | | | 79.07813 | |  | |  | |  |
| 34.20833 | | 396.0146 | | | | 6.616481 | | | | 2252.875 | | | 79.20313 | |  | |  | |  |
| 34.21667 | | 396.1026 | | | | 6.618876 | | | | 2252.195 | | | 79.33594 | |  | |  | |  |
| 34.225 | | 396.1942 | | | | 6.617495 | | | | 2251.539 | | | 79.39844 | |  | |  | |  |
| 34.23333 | | 396.2825 | | | | 6.617852 | | | | 2250.891 | | | 79.32031 | |  | |  | |  |
| 34.24167 | | 396.3687 | | | | 6.620727 | | | | 2250.203 | | | 79.42969 | |  | |  | |  |
| 34.25 | | 396.457 | | | | 6.620264 | | | | 2249.492 | | | 79.19531 | |  | |  | |  |
| 34.25833 | | 396.5431 | | | | 6.62111 | | | | 2248.82 | | | 79.0625 | |  | |  | |  |
| 34.26667 | | 396.6314 | | | | 6.624338 | | | | 2248.148 | | | 78.99219 | |  | |  | |  |
| 34.275 | | 396.7226 | | | | 6.624382 | | | | 2247.477 | | | 79.11719 | |  | |  | |  |
| 34.28333 | | 396.8109 | | | | 6.624581 | | | | 2246.828 | | | 78.95313 | |  | |  | |  |
| 34.29167 | | 396.8964 | | | | 6.627202 | | | | 2246.164 | | | 79.02344 | |  | |  | |  |
| 34.3 | | 396.9875 | | | | 6.628518 | | | | 2245.539 | | | 78.94531 | |  | |  | |  |
| 34.30833 | | 397.0751 | | | | 6.627992 | | | | 2244.906 | | | 79.04688 | |  | |  | |  |
| 34.31667 | | 397.1649 | | | | 6.630004 | | | | 2244.25 | | | 79.25781 | |  | |  | |  |
| 34.325 | | 397.2517 | | | | 6.631381 | | | | 2243.578 | | | 79.14063 | |  | |  | |  |
| 34.33333 | | 397.3379 | | | | 6.629856 | | | | 2242.953 | | | 79.24219 | |  | |  | |  |
| 34.34167 | | 397.4255 | | | | 6.63155 | | | | 2242.289 | | | 79.20313 | |  | |  | |  |
| 34.35 | | 397.5117 | | | | 6.633092 | | | | 2241.641 | | | 79.34375 | |  | |  | |  |
| 34.35833 | | 397.6035 | | | | 6.631817 | | | | 2240.969 | | | 79.21094 | |  | |  | |  |
| 34.36667 | | 397.6847 | | | | 6.633463 | | | | 2240.273 | | | 79.21875 | |  | |  | |  |
| 34.375 | | 397.7744 | | | | 6.635693 | | | | 2239.633 | | | 79.32031 | |  | |  | |  |
| 34.38333 | | 397.8642 | | | | 6.634856 | | | | 2238.953 | | | 79.28906 | |  | |  | |  |
| 34.39167 | | 397.9554 | | | | 6.635236 | | | | 2238.297 | | | 79.15625 | |  | |  | |  |
| 34.4 | | 398.0429 | | | | 6.638649 | | | | 2237.617 | | | 79.07031 | |  | |  | |  |
| 34.40833 | | 398.1334 | | | | 6.637815 | | | | 2236.969 | | | 79.32813 | |  | |  | |  |
| 34.41667 | | 398.2231 | | | | 6.638418 | | | | 2236.297 | | | 79.22656 | |  | |  | |  |
| 34.425 | | 398.3072 | | | | 6.642076 | | | | 2235.625 | | | 79.26563 | |  | |  | |  |
| 34.43333 | | 398.3983 | | | | 6.640802 | | | | 2234.961 | | | 79.30469 | |  | |  | |  |
| 34.44167 | | 398.4859 | | | | 6.641175 | | | | 2234.32 | | | 79.21094 | |  | |  | |  |
| 34.45 | | 398.5771 | | | | 6.644125 | | | | 2233.672 | | | 79.0625 | |  | |  | |  |
| 34.45833 | | 398.6682 | | | | 6.643101 | | | | 2232.969 | | | 78.83594 | |  | |  | |  |
| 34.46667 | | 398.753 | | | | 6.641828 | | | | 2232.336 | | | 78.79688 | |  | |  | |  |
| 34.475 | | 398.842 | | | | 6.645026 | | | | 2231.656 | | | 78.66406 | |  | |  | |  |
| 34.48333 | | 398.9303 | | | | 6.644333 | | | | 2230.992 | | | 78.53125 | |  | |  | |  |
| 34.49167 | | 399.0243 | | | | 6.643868 | | | | 2230.344 | | | 78.14844 | |  | |  | |  |
| 34.5 | | 399.1147 | | | | 6.646381 | | | | 2229.711 | | | 78.08594 | |  | |  | |  |
| 34.50833 | | 399.2052 | | | | 6.64638 | | | | 2229.086 | | | 78.17188 | |  | |  | |  |
| 34.51667 | | 399.2878 | | | | 6.644799 | | | | 2228.445 | | | 78.16406 | |  | |  | |  |
| 34.525 | | 399.3761 | | | | 6.646992 | | | | 2227.813 | | | 78.19531 | |  | |  | |  |
| 34.53333 | | 399.4666 | | | | 6.647777 | | | | 2227.18 | | | 78.52344 | |  | |  | |  |
| 34.54167 | | 399.5534 | | | | 6.646304 | | | | 2226.578 | | | 78.54688 | |  | |  | |  |
| 34.55 | | 399.6446 | | | | 6.648734 | | | | 2225.938 | | | 78.71875 | |  | |  | |  |
| 34.55833 | | 399.7293 | | | | 6.651028 | | | | 2225.273 | | | 78.8125 | |  | |  | |  |
| 34.56667 | | 399.8184 | | | | 6.650283 | | | | 2224.617 | | | 79 | |  | |  | |  |
| 34.575 | | 399.9052 | | | | 6.65216 | | | | 2223.961 | | | 79.33594 | |  | |  | |  |
| 34.58333 | | 399.995 | | | | 6.653217 | | | | 2223.266 | | | 79.52344 | |  | |  | |  |
| 34.59167 | | 400.0904 | | | | 6.652837 | | | | 2222.594 | | | 79.46094 | |  | |  | |  |
| 34.6 | | 400.1837 | | | | 6.652626 | | | | 2221.914 | | | 79.4375 | |  | |  | |  |
| 34.60833 | | 400.2727 | | | | 6.656118 | | | | 2221.234 | | | 79.53906 | |  | |  | |  |
| 34.61667 | | 400.3653 | | | | 6.656475 | | | | 2220.555 | | | 79.46875 | |  | |  | |  |
| 34.625 | | 400.4579 | | | | 6.654717 | | | | 2219.844 | | | 79.50781 | |  | |  | |  |
| 34.63333 | | 400.5469 | | | | 6.658001 | | | | 2219.156 | | | 79.4375 | |  | |  | |  |
| 34.64167 | | 400.6373 | | | | 6.659814 | | | | 2218.5 | | | 79.39063 | |  | |  | |  |
| 34.65 | | 400.7242 | | | | 6.658142 | | | | 2217.844 | | | 79.4375 | |  | |  | |  |
| 34.65833 | | 400.8097 | | | | 6.660174 | | | | 2217.18 | | | 79.15625 | |  | |  | |  |
| 34.66667 | | 400.8973 | | | | 6.662759 | | | | 2216.523 | | | 79.07813 | |  | |  | |  |
| 34.675 | | 400.9813 | | | | 6.661649 | | | | 2215.852 | | | 79.10156 | |  | |  | |  |
| 34.68333 | | 401.0696 | | | | 6.663469 | | | | 2215.211 | | | 79.00781 | |  | |  | |  |
| 34.69167 | | 401.1572 | | | | 6.664662 | | | | 2214.547 | | | 79.22656 | |  | |  | |  |
| 34.7 | | 401.2384 | | | | 6.66262 | | | | 2213.891 | | | 79.15625 | |  | |  | |  |
| 34.70833 | | 401.3203 | | | | 6.665489 | | | | 2213.266 | | | 79.14844 | |  | |  | |  |
| 34.71667 | | 401.4079 | | | | 6.665685 | | | | 2212.609 | | | 79.19531 | |  | |  | |  |
| 34.725 | | 401.4955 | | | | 6.662478 | | | | 2211.953 | | | 79.125 | |  | |  | |  |
| 34.73333 | | 401.5852 | | | | 6.665528 | | | | 2211.305 | | | 79.35938 | |  | |  | |  |
| 34.74167 | | 401.6728 | | | | 6.667437 | | | | 2210.609 | | | 79.34375 | |  | |  | |  |
| 34.75 | | 401.7625 | | | | 6.66574 | | | | 2209.961 | | | 79.27344 | |  | |  | |  |
| 34.75833 | | 401.8572 | | | | 6.668276 | | | | 2209.297 | | | 78.98438 | |  | |  | |  |
| 34.76667 | | 401.9456 | | | | 6.670787 | | | | 2208.633 | | | 78.97656 | |  | |  | |  |
| 34.775 | | 402.0339 | | | | 6.670184 | | | | 2207.977 | | | 78.64844 | |  | |  | |  |
| 34.78333 | | 402.125 | | | | 6.671258 | | | | 2207.289 | | | 78.46094 | |  | |  | |  |
| 34.79167 | | 402.2162 | | | | 6.673733 | | | | 2206.625 | | | 78.35938 | |  | |  | |  |
| 34.8 | | 402.3059 | | | | 6.673927 | | | | 2205.992 | | | 78.14063 | |  | |  | |  |
| 34.80833 | | 402.3942 | | | | 6.67326 | | | | 2205.359 | | | 78.16406 | |  | |  | |  |
| 34.81667 | | 402.4861 | | | | 6.675788 | | | | 2204.703 | | | 78.07813 | |  | |  | |  |
| 34.825 | | 402.5829 | | | | 6.678043 | | | | 2204.094 | | | 78.1875 | |  | |  | |  |
| 34.83333 | | 402.6713 | | | | 6.674957 | | | | 2203.461 | | | 78.46875 | |  | |  | |  |
| 34.84167 | | 402.7553 | | | | 6.677783 | | | | 2202.828 | | | 78.89063 | |  | |  | |  |
| 34.85 | | 402.8443 | | | | 6.680432 | | | | 2202.203 | | | 79.16406 | |  | |  | |  |
| 34.85833 | | 402.9348 | | | | 6.678671 | | | | 2201.555 | | | 79.60938 | |  | |  | |  |
| 34.86667 | | 403.0245 | | | | 6.681135 | | | | 2200.914 | | | 80.17188 | |  | |  | |  |
| 34.875 | | 403.1149 | | | | 6.684227 | | | | 2200.258 | | | 80.53906 | |  | |  | |  |
| 34.88333 | | 403.2018 | | | | 6.682126 | | | | 2199.555 | | | 81.28125 | |  | |  | |  |
| 34.89167 | | 403.2887 | | | | 6.683709 | | | | 2198.844 | | | 81.8125 | |  | |  | |  |
| 34.9 | | 403.3741 | | | | 6.687407 | | | | 2198.148 | | | 82.00781 | |  | |  | |  |
| 34.90833 | | 403.4553 | | | | 6.684893 | | | | 2197.422 | | | 82.48438 | |  | |  | |  |
| 34.91667 | | 403.5465 | | | | 6.686383 | | | | 2196.68 | | | 82.76563 | |  | |  | |  |
| 34.925 | | 403.6384 | | | | 6.69015 | | | | 2195.953 | | | 83 | |  | |  | |  |
| 34.93333 | | 403.7274 | | | | 6.688532 | | | | 2195.164 | | | 83.17188 | |  | |  | |  |
| 34.94167 | | 403.8143 | | | | 6.689453 | | | | 2194.422 | | | 83.24219 | |  | |  | |  |
| 34.95 | | 403.9019 | | | | 6.693336 | | | | 2193.695 | | | 83.32813 | |  | |  | |  |
| 34.95833 | | 403.9902 | | | | 6.691038 | | | | 2192.945 | | | 83.5625 | |  | |  | |  |
| 34.96667 | | 404.0806 | | | | 6.69192 | | | | 2192.219 | | | 83.48438 | |  | |  | |  |
| 34.975 | | 404.1749 | | | | 6.695137 | | | | 2191.492 | | | 83.39844 | |  | |  | |  |
| 34.98333 | | 404.2684 | | | | 6.695762 | | | | 2190.781 | | | 83.5625 | |  | |  | |  |
| 34.99167 | | 404.3654 | | | | 6.694524 | | | | 2190.078 | | | 83.36719 | |  | |  | |  |
| 35 | | 404.4554 | | | | 6.695587 | | | | 2189.375 | | | 83.125 | |  | |  | |  |
| 35.00833 | | 404.5418 | | | | 6.698742 | | | | 2188.648 | | | 82.94531 | |  | |  | |  |
| 35.01667 | | 404.6296 | | | | 6.697251 | | | | 2187.977 | | | 83.16406 | |  | |  | |  |
| 35.025 | | 404.7188 | | | | 6.698424 | | | | 2187.289 | | | 82.98438 | |  | |  | |  |
| 35.03333 | | 404.8116 | | | | 6.701677 | | | | 2186.578 | | | 83 | |  | |  | |  |
| 35.04167 | | 404.8987 | | | | 6.700717 | | | | 2185.906 | | | 82.83594 | |  | |  | |  |
| 35.05 | | 404.9901 | | | | 6.701938 | | | | 2185.258 | | | 82.6875 | |  | |  | |  |
| 35.05833 | | 405.0793 | | | | 6.706602 | | | | 2184.602 | | | 82.58594 | |  | |  | |  |
| 35.06667 | | 405.165 | | | | 6.705776 | | | | 2183.875 | | | 82.40625 | |  | |  | |  |
| 35.075 | | 405.2485 | | | | 6.705664 | | | | 2183.219 | | | 82.17188 | |  | |  | |  |
| 35.08333 | | 405.3342 | | | | 6.710473 | | | | 2182.531 | | | 82.17969 | |  | |  | |  |
| 35.09167 | | 405.4248 | | | | 6.709526 | | | | 2181.867 | | | 82.32031 | |  | |  | |  |
| 35.1 | | 405.5197 | | | | 6.70888 | | | | 2181.195 | | | 82.25781 | |  | |  | |  |
| 35.10833 | | 405.6133 | | | | 6.712182 | | | | 2180.523 | | | 82.07813 | |  | |  | |  |
| 35.11667 | | 405.7003 | | | | 6.712689 | | | | 2179.875 | | | 81.97656 | |  | |  | |  |
| 35.125 | | 405.7946 | | | | 6.711786 | | | | 2179.227 | | | 81.8125 | |  | |  | |  |
| 35.13333 | | 405.8824 | | | | 6.713883 | | | | 2178.531 | | | 81.52344 | |  | |  | |  |
| 35.14167 | | 405.9702 | | | | 6.714635 | | | | 2177.836 | | | 81.45313 | |  | |  | |  |
| 35.15 | | 406.0602 | | | | 6.71379 | | | | 2177.164 | | | 81.38281 | |  | |  | |  |
| 35.15833 | | 406.1494 | | | | 6.71582 | | | | 2176.508 | | | 81.32813 | |  | |  | |  |
| 35.16667 | | 406.2393 | | | | 6.718662 | | | | 2175.844 | | | 81.53906 | |  | |  | |  |
| 35.175 | | 406.3307 | | | | 6.718292 | | | | 2175.195 | | | 81.48438 | |  | |  | |  |
| 35.18333 | | 406.4164 | | | | 6.718638 | | | | 2174.555 | | | 81.32031 | |  | |  | |  |
| 35.19167 | | 406.5049 | | | | 6.722275 | | | | 2173.906 | | | 81.40625 | |  | |  | |  |
| 35.2 | | 406.5941 | | | | 6.721944 | | | | 2173.242 | | | 81.25781 | |  | |  | |  |
| 35.20833 | | 406.679 | | | | 6.721426 | | | | 2172.57 | | | 81.3125 | |  | |  | |  |
| 35.21667 | | 406.7661 | | | | 6.724258 | | | | 2171.867 | | | 81.46094 | |  | |  | |  |
| 35.225 | | 406.8546 | | | | 6.724269 | | | | 2171.203 | | | 81.60938 | |  | |  | |  |
| 35.23333 | | 406.9453 | | | | 6.724413 | | | | 2170.539 | | | 81.39063 | |  | |  | |  |
| 35.24167 | | 407.036 | | | | 6.727963 | | | | 2169.844 | | | 81.47656 | |  | |  | |  |
| 35.25 | | 407.1273 | | | | 6.728188 | | | | 2169.18 | | | 81.38281 | |  | |  | |  |
| 35.25833 | | 407.213 | | | | 6.727216 | | | | 2168.5 | | | 81.3125 | |  | |  | |  |
| 35.26667 | | 407.2979 | | | | 6.731098 | | | | 2167.789 | | | 81.16406 | |  | |  | |  |
| 35.275 | | 407.385 | | | | 6.731044 | | | | 2167.086 | | | 81.05469 | |  | |  | |  |
| 35.28333 | | 407.4764 | | | | 6.730671 | | | | 2166.414 | | | 81.125 | |  | |  | |  |
| 35.29167 | | 407.5656 | | | | 6.734296 | | | | 2165.719 | | | 81.0625 | |  | |  | |  |
| 35.3 | | 407.6542 | | | | 6.735405 | | | | 2165.047 | | | 81.09375 | |  | |  | |  |
| 35.30833 | | 407.7448 | | | | 6.732874 | | | | 2164.375 | | | 81.16406 | |  | |  | |  |
| 35.31667 | | 407.8326 | | | | 6.735622 | | | | 2163.719 | | | 81.28125 | |  | |  | |  |
| 35.325 | | 407.9211 | | | | 6.736259 | | | | 2163.047 | | | 81.35938 | |  | |  | |  |
| 35.33333 | | 408.0104 | | | | 6.733123 | | | | 2162.359 | | | 81.28906 | |  | |  | |  |
| 35.34167 | | 408.0989 | | | | 6.736286 | | | | 2161.68 | | | 81.35156 | |  | |  | |  |
| 35.35 | | 408.1903 | | | | 6.737841 | | | | 2160.992 | | | 81.46094 | |  | |  | |  |
| 35.35833 | | 408.2831 | | | | 6.735651 | | | | 2160.305 | | | 81.59375 | |  | |  | |  |
| 35.36667 | | 408.3723 | | | | 6.737668 | | | | 2159.609 | | | 81.67188 | |  | |  | |  |
| 35.375 | | 408.4665 | | | | 6.739703 | | | | 2158.922 | | | 81.70313 | |  | |  | |  |
| 35.38333 | | 408.5586 | | | | 6.739265 | | | | 2158.25 | | | 81.71875 | |  | |  | |  |
| 35.39167 | | 408.6486 | | | | 6.738852 | | | | 2157.563 | | | 82.10156 | |  | |  | |  |
| 35.4 | | 408.7385 | | | | 6.740785 | | | | 2156.875 | | | 82.17188 | |  | |  | |  |
| 35.40833 | | 408.8277 | | | | 6.740926 | | | | 2156.172 | | | 82.07031 | |  | |  | |  |
| 35.41667 | | 408.9155 | | | | 6.741164 | | | | 2155.484 | | | 82.11719 | |  | |  | |  |
| 35.425 | | 409.0105 | | | | 6.743354 | | | | 2154.805 | | | 82.1875 | |  | |  | |  |
| 35.43333 | | 409.1026 | | | | 6.744882 | | | | 2154.109 | | | 82.15625 | |  | |  | |  |
| 35.44167 | | 409.1861 | | | | 6.744367 | | | | 2153.375 | | | 82.14844 | |  | |  | |  |
| 35.45 | | 409.271 | | | | 6.747608 | | | | 2152.695 | | | 82.07031 | |  | |  | |  |
| 35.45833 | | 409.3567 | | | | 6.748085 | | | | 2152.016 | | | 82.02344 | |  | |  | |  |
| 35.46667 | | 409.4438 | | | | 6.747915 | | | | 2151.328 | | | 82.09375 | |  | |  | |  |
| 35.475 | | 409.533 | | | | 6.749722 | | | | 2150.641 | | | 81.54688 | |  | |  | |  |
| 35.48333 | | 409.6208 | | | | 6.750578 | | | | 2149.961 | | | 81.63281 | |  | |  | |  |
| 35.49167 | | 409.7086 | | | | 6.749569 | | | | 2149.273 | | | 81.71875 | |  | |  | |  |
| 35.5 | | 409.795 | | | | 6.751784 | | | | 2148.609 | | | 81.82813 | |  | |  | |  |
| 35.50833 | | 409.8828 | | | | 6.752965 | | | | 2147.93 | | | 81.88281 | |  | |  | |  |
| 35.51667 | | 409.9727 | | | | 6.751651 | | | | 2147.25 | | | 81.90625 | |  | |  | |  |
| 35.525 | | 410.0648 | | | | 6.754049 | | | | 2146.633 | | | 81.96094 | |  | |  | |  |
| 35.53333 | | 410.1605 | | | | 6.756823 | | | | 2145.945 | | | 82.28125 | |  | |  | |  |
| 35.54167 | | 410.2511 | | | | 6.753929 | | | | 2145.258 | | | 82.49219 | |  | |  | |  |
| 35.55 | | 410.3411 | | | | 6.755512 | | | | 2144.563 | | | 82.4375 | |  | |  | |  |
| 35.55833 | | 410.4303 | | | | 6.7587 | | | | 2143.883 | | | 82.50781 | |  | |  | |  |
| 35.56667 | | 410.5217 | | | | 6.757244 | | | | 2143.188 | | | 82.5 | |  | |  | |  |
| 35.575 | | 410.6124 | | | | 6.757318 | | | | 2142.492 | | | 82.46875 | |  | |  | |  |
| 35.58333 | | 410.703 | | | | 6.759028 | | | | 2141.773 | | | 82.48438 | |  | |  | |  |
| 35.59167 | | 410.7894 | | | | 6.758564 | | | | 2141.055 | | | 82.53125 | |  | |  | |  |
| 35.6 | | 410.8729 | | | | 6.757818 | | | | 2140.375 | | | 82.6875 | |  | |  | |  |
| 35.60833 | | 410.96 | | | | 6.759965 | | | | 2139.68 | | | 82.66406 | |  | |  | |  |
| 35.61667 | | 411.0464 | | | | 6.75824 | | | | 2138.992 | | | 82.51563 | |  | |  | |  |
| 35.625 | | 411.137 | | | | 6.758748 | | | | 2138.305 | | | 82.67188 | |  | |  | |  |
| 35.63333 | | 411.2291 | | | | 6.762256 | | | | 2137.617 | | | 82.49219 | |  | |  | |  |
| 35.64167 | | 411.3191 | | | | 6.762356 | | | | 2136.922 | | | 82.40625 | |  | |  | |  |
| 35.65 | | 411.409 | | | | 6.761331 | | | | 2136.211 | | | 82.39063 | |  | |  | |  |
| 35.65833 | | 411.4997 | | | | 6.763093 | | | | 2135.523 | | | 82.42969 | |  | |  | |  |
| 35.66667 | | 411.5853 | | | | 6.762989 | | | | 2134.859 | | | 82.52344 | |  | |  | |  |
| 35.675 | | 411.6781 | | | | 6.761763 | | | | 2134.156 | | | 82.80469 | |  | |  | |  |
| 35.68333 | | 411.7716 | | | | 6.761967 | | | | 2133.492 | | | 82.82031 | |  | |  | |  |
| 35.69167 | | 411.8587 | | | | 6.763848 | | | | 2132.82 | | | 82.91406 | |  | |  | |  |
| 35.7 | | 411.9434 | | | | 6.762093 | | | | 2132.133 | | | 82.85156 | |  | |  | |  |
| 35.70833 | | 412.0293 | | | | 6.763174 | | | | 2131.438 | | | 82.58594 | |  | |  | |  |
| 35.71667 | | 412.1153 | | | | 6.764208 | | | | 2130.734 | | | 82.78906 | |  | |  | |  |
| 35.725 | | 412.2034 | | | | 6.763035 | | | | 2130.008 | | | 82.85938 | |  | |  | |  |
| 35.73333 | | 412.2943 | | | | 6.763929 | | | | 2129.32 | | | 82.92188 | |  | |  | |  |
| 35.74167 | | 412.3874 | | | | 6.767237 | | | | 2128.617 | | | 82.74219 | |  | |  | |  |
| 35.75 | | 412.4847 | | | | 6.766618 | | | | 2127.938 | | | 82.70313 | |  | |  | |  |
| 35.75833 | | 412.5707 | | | | 6.765346 | | | | 2127.281 | | | 82.60938 | |  | |  | |  |
| 35.76667 | | 412.6574 | | | | 6.769018 | | | | 2126.563 | | | 82.49219 | |  | |  | |  |
| 35.775 | | 412.7462 | | | | 6.768167 | | | | 2125.859 | | | 82.42188 | |  | |  | |  |
| 35.78333 | | 412.8336 | | | | 6.766912 | | | | 2125.164 | | | 82.50781 | |  | |  | |  |
| 35.79167 | | 412.9217 | | | | 6.771252 | | | | 2124.492 | | | 82.73438 | |  | |  | |  |
| 35.8 | | 413.0133 | | | | 6.772171 | | | | 2123.805 | | | 82.67188 | |  | |  | |  |
| 35.80833 | | 413.0993 | | | | 6.77162 | | | | 2123.141 | | | 82.73438 | |  | |  | |  |
| 35.81667 | | 413.1845 | | | | 6.776695 | | | | 2122.461 | | | 82.69531 | |  | |  | |  |
| 35.825 | | 413.2691 | | | | 6.777641 | | | | 2121.789 | | | 82.78125 | |  | |  | |  |
| 35.83333 | | 413.3543 | | | | 6.77707 | | | | 2121.086 | | | 82.75781 | |  | |  | |  |
| 35.84167 | | 413.441 | | | | 6.780634 | | | | 2120.367 | | | 82.71875 | |  | |  | |  |
| 35.85 | | 413.5313 | | | | 6.780222 | | | | 2119.695 | | | 82.64844 | |  | |  | |  |
| 35.85833 | | 413.6215 | | | | 6.778576 | | | | 2118.992 | | | 82.57031 | |  | |  | |  |
| 35.86667 | | 413.7145 | | | | 6.782125 | | | | 2118.313 | | | 82.42188 | |  | |  | |  |
| 35.875 | | 413.8012 | | | | 6.782597 | | | | 2117.609 | | | 82.39063 | |  | |  | |  |
| 35.88333 | | 413.8914 | | | | 6.78075 | | | | 2116.93 | | | 82.42188 | |  | |  | |  |
| 35.89167 | | 413.981 | | | | 6.784634 | | | | 2116.242 | | | 82.19531 | |  | |  | |  |
| 35.9 | | 414.0691 | | | | 6.785605 | | | | 2115.563 | | | 82.32813 | |  | |  | |  |
| 35.90833 | | 414.1565 | | | | 6.784163 | | | | 2114.891 | | | 82.47656 | |  | |  | |  |
| 35.91667 | | 414.2431 | | | | 6.786996 | | | | 2114.227 | | | 82.67969 | |  | |  | |  |
| 35.925 | | 414.3348 | | | | 6.788543 | | | | 2113.547 | | | 83.17188 | |  | |  | |  |
| 35.93333 | | 414.4243 | | | | 6.788358 | | | | 2112.852 | | | 83.10938 | |  | |  | |  |
| 35.94167 | | 414.5145 | | | | 6.788258 | | | | 2112.188 | | | 83.32031 | |  | |  | |  |
| 35.95 | | 414.6033 | | | | 6.791263 | | | | 2111.477 | | | 83.63281 | |  | |  | |  |
| 35.95833 | | 414.6964 | | | | 6.791456 | | | | 2110.773 | | | 83.78906 | |  | |  | |  |
| 35.96667 | | 414.7831 | | | | 6.79063 | | | | 2110.055 | | | 83.88281 | |  | |  | |  |
| 35.975 | | 414.874 | | | | 6.793642 | | | | 2109.297 | | | 84.14063 | |  | |  | |  |
| 35.98333 | | 414.965 | | | | 6.794327 | | | | 2108.609 | | | 84.0625 | |  | |  | |  |
| 35.99167 | | 415.0524 | | | | 6.793348 | | | | 2107.883 | | | 83.97656 | |  | |  | |  |
| 36 | | 415.1412 | | | | 6.795929 | | | | 2107.141 | | | 83.9375 | |  | |  | |  |
| 36.00833 | | 415.2271 | | | | 6.796466 | | | | 2106.422 | | | 83.42188 | |  | |  | |  |
| 36.01667 | | 415.3145 | | | | 6.795167 | | | | 2105.711 | | | 83.57813 | |  | |  | |  |
| 36.025 | | 415.404 | | | | 6.797742 | | | | 2104.977 | | | 83.67969 | |  | |  | |  |
| 36.03333 | | 415.4957 | | | | 6.799439 | | | | 2104.289 | | | 83.55469 | |  | |  | |  |
| 36.04167 | | 415.5845 | | | | 6.797859 | | | | 2103.602 | | | 83.47656 | |  | |  | |  |
| 36.05 | | 415.6761 | | | | 6.801602 | | | | 2102.922 | | | 83.41406 | |  | |  | |  |
| 36.05833 | | 415.7664 | | | | 6.80464 | | | | 2102.281 | | | 83.67188 | |  | |  | |  |
| 36.06667 | | 415.8545 | | | | 6.801906 | | | | 2101.57 | | | 83.74219 | |  | |  | |  |
| 36.075 | | 415.944 | | | | 6.804658 | | | | 2100.867 | | | 83.85938 | |  | |  | |  |
| 36.08333 | | 416.0378 | | | | 6.807377 | | | | 2100.172 | | | 83.83594 | |  | |  | |  |
| 36.09167 | | 416.1301 | | | | 6.805912 | | | | 2099.5 | | | 84.0625 | |  | |  | |  |
| 36.1 | | 416.2232 | | | | 6.806256 | | | | 2098.805 | | | 83.92188 | |  | |  | |  |
| 36.10833 | | 416.3127 | | | | 6.808008 | | | | 2098.078 | | | 84.20313 | |  | |  | |  |
| 36.11667 | | 416.398 | | | | 6.807916 | | | | 2097.367 | | | 84.29688 | |  | |  | |  |
| 36.125 | | 416.4861 | | | | 6.807408 | | | | 2096.656 | | | 84.50781 | |  | |  | |  |
| 36.13333 | | 416.5735 | | | | 6.80953 | | | | 2095.961 | | | 84.49219 | |  | |  | |  |
| 36.14167 | | 416.6623 | | | | 6.809868 | | | | 2095.234 | | | 84.15625 | |  | |  | |  |
| 36.15 | | 416.749 | | | | 6.809353 | | | | 2094.555 | | | 84.46094 | |  | |  | |  |
| 36.15833 | | 416.8385 | | | | 6.811884 | | | | 2093.813 | | | 84.41406 | |  | |  | |  |
| 36.16667 | | 416.9251 | | | | 6.812242 | | | | 2093.102 | | | 84.39844 | |  | |  | |  |
| 36.175 | | 417.0154 | | | | 6.811442 | | | | 2092.359 | | | 84.29688 | |  | |  | |  |
| 36.18333 | | 417.0999 | | | | 6.814218 | | | | 2091.664 | | | 84.51563 | |  | |  | |  |
| 36.19167 | | 417.1902 | | | | 6.815988 | | | | 2091.008 | | | 84.28125 | |  | |  | |  |
| 36.2 | | 417.2811 | | | | 6.815563 | | | | 2090.273 | | | 84.22656 | |  | |  | |  |
| 36.20833 | | 417.3692 | | | | 6.817275 | | | | 2089.578 | | | 84.28906 | |  | |  | |  |
| 36.21667 | | 417.4559 | | | | 6.818355 | | | | 2088.875 | | | 84.5625 | |  | |  | |  |
| 36.225 | | 417.5418 | | | | 6.816443 | | | | 2088.188 | | | 84.8125 | |  | |  | |  |
| 36.23333 | | 417.6335 | | | | 6.818157 | | | | 2087.445 | | | 84.67969 | |  | |  | |  |
| 36.24167 | | 417.7266 | | | | 6.82093 | | | | 2086.789 | | | 85.08594 | |  | |  | |  |
| 36.25 | | 417.8168 | | | | 6.819073 | | | | 2086.094 | | | 85.11719 | |  | |  | |  |
| 36.25833 | | 417.9013 | | | | 6.820247 | | | | 2085.391 | | | 85.28906 | |  | |  | |  |
| 36.26667 | | 417.9915 | | | | 6.824585 | | | | 2084.641 | | | 85.45313 | |  | |  | |  |
| 36.275 | | 418.0804 | | | | 6.823224 | | | | 2083.906 | | | 85.50781 | |  | |  | |  |
| 36.28333 | | 418.1706 | | | | 6.823412 | | | | 2083.219 | | | 85.69531 | |  | |  | |  |
| 36.29167 | | 418.2601 | | | | 6.827521 | | | | 2082.469 | | | 85.67969 | |  | |  | |  |
| 36.3 | | 418.3489 | | | | 6.82738 | | | | 2081.742 | | | 85.70313 | |  | |  | |  |
| 36.30833 | | 418.4391 | | | | 6.827887 | | | | 2081.008 | | | 85.85938 | |  | |  | |  |
| 36.31667 | | 418.5273 | | | | 6.831238 | | | | 2080.273 | | | 86.03906 | |  | |  | |  |
| 36.325 | | 418.6139 | | | | 6.829767 | | | | 2079.547 | | | 86.01563 | |  | |  | |  |
| 36.33333 | | 418.702 | | | | 6.829063 | | | | 2078.813 | | | 86.05469 | |  | |  | |  |
| 36.34167 | | 418.7915 | | | | 6.833195 | | | | 2078.094 | | | 85.96094 | |  | |  | |  |
| 36.35 | | 418.8782 | | | | 6.833284 | | | | 2077.375 | | | 86.16406 | |  | |  | |  |
| 36.35833 | | 418.9649 | | | | 6.8325 | | | | 2076.633 | | | 86.22656 | |  | |  | |  |
| 36.36667 | | 419.0523 | | | | 6.836428 | | | | 2075.898 | | | 86.29688 | |  | |  | |  |
| 36.375 | | 419.1425 | | | | 6.836675 | | | | 2075.18 | | | 86.40625 | |  | |  | |  |
| 36.38333 | | 419.2342 | | | | 6.835801 | | | | 2074.461 | | | 86.46094 | |  | |  | |  |
| 36.39167 | | 419.3237 | | | | 6.83829 | | | | 2073.766 | | | 86.40625 | |  | |  | |  |
| 36.4 | | 419.4118 | | | | 6.838722 | | | | 2073.023 | | | 86.39844 | |  | |  | |  |
| 36.40833 | | 419.4992 | | | | 6.837707 | | | | 2072.297 | | | 86.4375 | |  | |  | |  |
| 36.41667 | | 419.5894 | | | | 6.84086 | | | | 2071.57 | | | 86.54688 | |  | |  | |  |
| 36.425 | | 419.681 | | | | 6.84193 | | | | 2070.844 | | | 86.84375 | |  | |  | |  |
| 36.43333 | | 419.7749 | | | | 6.840519 | | | | 2070.117 | | | 86.625 | |  | |  | |  |
| 36.44167 | | 419.8651 | | | | 6.841833 | | | | 2069.406 | | | 86.63281 | |  | |  | |  |
| 36.45 | | 419.954 | | | | 6.844269 | | | | 2068.688 | | | 86.625 | |  | |  | |  |
| 36.45833 | | 420.0428 | | | | 6.843509 | | | | 2067.961 | | | 86.58594 | |  | |  | |  |
| 36.46667 | | 420.131 | | | | 6.844049 | | | | 2067.227 | | | 86.53125 | |  | |  | |  |
| 36.475 | | 420.2184 | | | | 6.84752 | | | | 2066.469 | | | 86.60156 | |  | |  | |  |
| 36.48333 | | 420.3115 | | | | 6.848034 | | | | 2065.773 | | | 86.51563 | |  | |  | |  |
| 36.49167 | | 420.4032 | | | | 6.847315 | | | | 2065.055 | | | 86.44531 | |  | |  | |  |
| 36.5 | | 420.4913 | | | | 6.851418 | | | | 2064.336 | | | 86.53125 | |  | |  | |  |
| 36.50833 | | 420.5808 | | | | 6.851966 | | | | 2063.609 | | | 86.54688 | |  | |  | |  |
| 36.51667 | | 420.6661 | | | | 6.850584 | | | | 2062.898 | | | 86.59375 | |  | |  | |  |
| 36.525 | | 420.7542 | | | | 6.854738 | | | | 2062.172 | | | 86.63281 | |  | |  | |  |
| 36.53333 | | 420.8431 | | | | 6.855158 | | | | 2061.469 | | | 86.50781 | |  | |  | |  |
| 36.54167 | | 420.9305 | | | | 6.854827 | | | | 2060.758 | | | 86.63281 | |  | |  | |  |
| 36.55 | | 421.0201 | | | | 6.857905 | | | | 2060.023 | | | 86.69531 | |  | |  | |  |
| 36.55833 | | 421.1103 | | | | 6.858867 | | | | 2059.313 | | | 86.71875 | |  | |  | |  |
| 36.56667 | | 421.1992 | | | | 6.85696 | | | | 2058.578 | | | 86.82813 | |  | |  | |  |
| 36.575 | | 421.2866 | | | | 6.861295 | | | | 2057.852 | | | 86.97656 | |  | |  | |  |
| 36.58333 | | 421.3762 | | | | 6.862889 | | | | 2057.148 | | | 87.07031 | |  | |  | |  |
| 36.59167 | | 421.4657 | | | | 6.861451 | | | | 2056.422 | | | 87.07031 | |  | |  | |  |
| 36.6 | | 421.5574 | | | | 6.864266 | | | | 2055.688 | | | 86.97656 | |  | |  | |  |
| 36.60833 | | 421.6469 | | | | 6.865877 | | | | 2054.969 | | | 87.29688 | |  | |  | |  |
| 36.61667 | | 421.7372 | | | | 6.863354 | | | | 2054.227 | | | 87.57813 | |  | |  | |  |
| 36.625 | | 421.8275 | | | | 6.865706 | | | | 2053.477 | | | 87.32813 | |  | |  | |  |
| 36.63333 | | 421.9149 | | | | 6.868168 | | | | 2052.75 | | | 87.40625 | |  | |  | |  |
| 36.64167 | | 422.0059 | | | | 6.867133 | | | | 2052.016 | | | 87.70313 | |  | |  | |  |
| 36.65 | | 422.0933 | | | | 6.868453 | | | | 2051.313 | | | 87.66406 | |  | |  | |  |
| 36.65833 | | 422.1828 | | | | 6.87126 | | | | 2050.523 | | | 87.6875 | |  | |  | |  |
| 36.66667 | | 422.2717 | | | | 6.870438 | | | | 2049.758 | | | 87.78125 | |  | |  | |  |
| 36.675 | | 422.3612 | | | | 6.872252 | | | | 2049.063 | | | 87.89844 | |  | |  | |  |
| 36.68333 | | 422.4494 | | | | 6.875623 | | | | 2048.32 | | | 88.21094 | |  | |  | |  |
| 36.69167 | | 422.5389 | | | | 6.875791 | | | | 2047.555 | | | 87.83594 | |  | |  | |  |
| 36.7 | | 422.6285 | | | | 6.876166 | | | | 2046.828 | | | 87.90625 | |  | |  | |  |
| 36.70833 | | 422.7202 | | | | 6.879856 | | | | 2046.094 | | | 88.03906 | |  | |  | |  |
| 36.71667 | | 422.8118 | | | | 6.880235 | | | | 2045.344 | | | 88.08594 | |  | |  | |  |
| 36.725 | | 422.8993 | | | | 6.880099 | | | | 2044.602 | | | 88.03125 | |  | |  | |  |
| 36.73333 | | 422.9881 | | | | 6.883471 | | | | 2043.828 | | | 88.21094 | |  | |  | |  |
| 36.74167 | | 423.0769 | | | | 6.885013 | | | | 2043.148 | | | 88.32813 | |  | |  | |  |
| 36.75 | | 423.1665 | | | | 6.883805 | | | | 2042.406 | | | 88.35938 | |  | |  | |  |
| 36.75833 | | 423.256 | | | | 6.886139 | | | | 2041.648 | | | 88.40625 | |  | |  | |  |
| 36.76667 | | 423.3463 | | | | 6.888656 | | | | 2040.914 | | | 88.46094 | |  | |  | |  |
| 36.775 | | 423.4359 | | | | 6.88575 | | | | 2040.188 | | | 88.57813 | |  | |  | |  |
| 36.78333 | | 423.5233 | | | | 6.887508 | | | | 2039.43 | | | 88.35938 | |  | |  | |  |
| 36.79167 | | 423.6107 | | | | 6.890354 | | | | 2038.68 | | | 88.51563 | |  | |  | |  |
| 36.8 | | 423.6996 | | | | 6.888716 | | | | 2037.945 | | | 88.63281 | |  | |  | |  |
| 36.80833 | | 423.7898 | | | | 6.890224 | | | | 2037.195 | | | 88.50781 | |  | |  | |  |
| 36.81667 | | 423.8801 | | | | 6.892922 | | | | 2036.445 | | | 88.53125 | |  | |  | |  |
| 36.825 | | 423.9703 | | | | 6.89123 | | | | 2035.695 | | | 88.375 | |  | |  | |  |
| 36.83333 | | 424.0599 | | | | 6.892817 | | | | 2034.992 | | | 88.38281 | |  | |  | |  |
| 36.84167 | | 424.1466 | | | | 6.896534 | | | | 2034.242 | | | 88.42969 | |  | |  | |  |
| 36.85 | | 424.2347 | | | | 6.894927 | | | | 2033.484 | | | 88.15625 | |  | |  | |  |
| 36.85833 | | 424.3257 | | | | 6.896096 | | | | 2032.766 | | | 88.13281 | |  | |  | |  |
| 36.86667 | | 424.4195 | | | | 6.899548 | | | | 2032.031 | | | 88.50781 | |  | |  | |  |
| 36.875 | | 424.5077 | | | | 6.900548 | | | | 2031.32 | | | 88.625 | |  | |  | |  |
| 36.88333 | | 424.5944 | | | | 6.900533 | | | | 2030.578 | | | 88.6875 | |  | |  | |  |
| 36.89167 | | 424.6846 | | | | 6.904241 | | | | 2029.844 | | | 88.75 | |  | |  | |  |
| 36.9 | | 424.7756 | | | | 6.904258 | | | | 2029.141 | | | 88.74219 | |  | |  | |  |
| 36.90833 | | 424.8666 | | | | 6.90448 | | | | 2028.414 | | | 88.9375 | |  | |  | |  |
| 36.91667 | | 424.9561 | | | | 6.907124 | | | | 2027.625 | | | 88.89063 | |  | |  | |  |
| 36.925 | | 425.0478 | | | | 6.907522 | | | | 2026.875 | | | 89 | |  | |  | |  |
| 36.93333 | | 425.1388 | | | | 6.90683 | | | | 2026.141 | | | 89.00781 | |  | |  | |  |
| 36.94167 | | 425.2283 | | | | 6.909454 | | | | 2025.383 | | | 89.0625 | |  | |  | |  |
| 36.95 | | 425.3158 | | | | 6.912769 | | | | 2024.641 | | | 89.05469 | |  | |  | |  |
| 36.95833 | | 425.4089 | | | | 6.912803 | | | | 2023.883 | | | 88.89844 | |  | |  | |  |
| 36.96667 | | 425.5005 | | | | 6.913915 | | | | 2023.141 | | | 88.95313 | |  | |  | |  |
| 36.975 | | 425.5915 | | | | 6.918286 | | | | 2022.391 | | | 89.05469 | |  | |  | |  |
| 36.98333 | | 425.6804 | | | | 6.919048 | | | | 2021.648 | | | 89.125 | |  | |  | |  |
| 36.99167 | | 425.7699 | | | | 6.918121 | | | | 2020.891 | | | 89.17188 | |  | |  | |  |
| 37 | | 425.8602 | | | | 6.922813 | | | | 2020.148 | | | 89.17969 | |  | |  | |  |
| 37.00833 | | 425.9497 | | | | 6.924493 | | | | 2019.43 | | | 89.17969 | |  | |  | |  |
| 37.01667 | | 426.04 | | | | 6.925019 | | | | 2018.68 | | | 89.32813 | |  | |  | |  |
| 37.025 | | 426.1302 | | | | 6.929701 | | | | 2017.93 | | | 89.55469 | |  | |  | |  |
| 37.03333 | | 426.2191 | | | | 6.931135 | | | | 2017.172 | | | 89.58594 | |  | |  | |  |
| 37.04167 | | 426.3065 | | | | 6.929978 | | | | 2016.422 | | | 89.71875 | |  | |  | |  |
| 37.05 | | 426.3975 | | | | 6.934464 | | | | 2015.672 | | | 89.77344 | |  | |  | |  |
| 37.05833 | | 426.4871 | | | | 6.935728 | | | | 2014.938 | | | 89.73438 | |  | |  | |  |
| 37.06667 | | 426.5752 | | | | 6.935294 | | | | 2014.164 | | | 89.82031 | |  | |  | |  |
| 37.075 | | 426.664 | | | | 6.938275 | | | | 2013.391 | | | 89.84375 | |  | |  | |  |
| 37.08333 | | 426.7536 | | | | 6.940511 | | | | 2012.633 | | | 89.80469 | |  | |  | |  |
| 37.09167 | | 426.8417 | | | | 6.94004 | | | | 2011.883 | | | 89.99219 | |  | |  | |  |
| 37.1 | | 426.9291 | | | | 6.942437 | | | | 2011.125 | | | 90.03906 | |  | |  | |  |
| 37.10833 | | 427.0158 | | | | 6.945401 | | | | 2010.383 | | | 89.96094 | |  | |  | |  |
| 37.11667 | | 427.1054 | | | | 6.945319 | | | | 2009.625 | | | 89.91406 | |  | |  | |  |
| 37.125 | | 427.1971 | | | | 6.946353 | | | | 2008.875 | | | 90.1875 | |  | |  | |  |
| 37.13333 | | 427.2859 | | | | 6.95034 | | | | 2008.133 | | | 90.35156 | |  | |  | |  |
| 37.14167 | | 427.3734 | | | | 6.950973 | | | | 2007.352 | | | 90.3125 | |  | |  | |  |
| 37.15 | | 427.4606 | | | | 6.950194 | | | | 2006.602 | | | 90.45313 | |  | |  | |  |
| 37.15833 | | 427.5501 | | | | 6.954972 | | | | 2005.859 | | | 90.60156 | |  | |  | |  |
| 37.16667 | | 427.6381 | | | | 6.955134 | | | | 2005.117 | | | 90.74219 | |  | |  | |  |
| 37.175 | | 427.7297 | | | | 6.954957 | | | | 2004.328 | | | 90.75781 | |  | |  | |  |
| 37.18333 | | 427.8212 | | | | 6.957918 | | | | 2003.563 | | | 90.94531 | |  | |  | |  |
| 37.19167 | | 427.9121 | | | | 6.96086 | | | | 2002.805 | | | 90.92188 | |  | |  | |  |
| 37.2 | | 428.0022 | | | | 6.960119 | | | | 2002.039 | | | 91.03906 | |  | |  | |  |
| 37.20833 | | 428.0882 | | | | 6.963246 | | | | 2001.266 | | | 91.07813 | |  | |  | |  |
| 37.21667 | | 428.179 | | | | 6.966279 | | | | 2000.492 | | | 91.07813 | |  | |  | |  |
| 37.225 | | 428.2713 | | | | 6.967225 | | | | 1999.734 | | | 90.92969 | |  | |  | |  |
| 37.23333 | | 428.3643 | | | | 6.969031 | | | | 1998.953 | | | 91.16406 | |  | |  | |  |
| 37.24167 | | 428.4544 | | | | 6.972793 | | | | 1998.203 | | | 91.28125 | |  | |  | |  |
| 37.25 | | 428.5446 | | | | 6.973717 | | | | 1997.438 | | | 91.25 | |  | |  | |  |
| 37.25833 | | 428.6326 | | | | 6.974155 | | | | 1996.672 | | | 91.25781 | |  | |  | |  |
| 37.26667 | | 428.722 | | | | 6.977697 | | | | 1995.914 | | | 91.25781 | |  | |  | |  |
| 37.275 | | 428.8115 | | | | 6.979687 | | | | 1995.164 | | | 91.59375 | |  | |  | |  |
| 37.28333 | | 428.8988 | | | | 6.979625 | | | | 1994.383 | | | 91.77344 | |  | |  | |  |
| 37.29167 | | 428.9904 | | | | 6.983822 | | | | 1993.617 | | | 91.83594 | |  | |  | |  |
| 37.3 | | 429.0777 | | | | 6.986145 | | | | 1992.852 | | | 91.94531 | |  | |  | |  |
| 37.30833 | | 429.1644 | | | | 6.985576 | | | | 1992.086 | | | 92.32813 | |  | |  | |  |
| 37.31667 | | 429.2538 | | | | 6.988655 | | | | 1991.328 | | | 92.29688 | |  | |  | |  |
| 37.325 | | 429.3411 | | | | 6.990843 | | | | 1990.523 | | | 92.39844 | |  | |  | |  |
| 37.33333 | | 429.4299 | | | | 6.989536 | | | | 1989.742 | | | 92.61719 | |  | |  | |  |
| 37.34167 | | 429.52 | | | | 6.992082 | | | | 1988.969 | | | 92.49219 | |  | |  | |  |
| 37.35 | | 429.6067 | | | | 6.995612 | | | | 1988.18 | | | 92.50781 | |  | |  | |  |
| 37.35833 | | 429.6947 | | | | 6.994003 | | | | 1987.367 | | | 92.60156 | |  | |  | |  |
| 37.36667 | | 429.7863 | | | | 6.995176 | | | | 1986.602 | | | 92.5625 | |  | |  | |  |
| 37.375 | | 429.8743 | | | | 6.998172 | | | | 1985.82 | | | 92.38281 | |  | |  | |  |
| 37.38333 | | 429.9651 | | | | 6.997931 | | | | 1985.016 | | | 92.35938 | |  | |  | |  |
| 37.39167 | | 430.0546 | | | | 6.998195 | | | | 1984.266 | | | 92.38281 | |  | |  | |  |
| 37.4 | | 430.1419 | | | | 7.001833 | | | | 1983.5 | | | 92.35938 | |  | |  | |  |
| 37.40833 | | 430.2314 | | | | 7.002833 | | | | 1982.719 | | | 92.35156 | |  | |  | |  |
| 37.41667 | | 430.318 | | | | 7.003633 | | | | 1981.953 | | | 92.35938 | |  | |  | |  |
| 37.425 | | 430.4075 | | | | 7.007473 | | | | 1981.203 | | | 92.85156 | |  | |  | |  |
| 37.43333 | | 430.499 | | | | 7.008158 | | | | 1980.438 | | | 92.82031 | |  | |  | |  |
| 37.44167 | | 430.5864 | | | | 7.008271 | | | | 1979.672 | | | 92.61719 | |  | |  | |  |
| 37.45 | | 430.673 | | | | 7.013025 | | | | 1978.906 | | | 92.71875 | |  | |  | |  |
| 37.45833 | | 430.7617 | | | | 7.014954 | | | | 1978.141 | | | 92.89844 | |  | |  | |  |
| 37.46667 | | 430.8484 | | | | 7.015376 | | | | 1977.367 | | | 93.01563 | |  | |  | |  |
| 37.475 | | 430.9364 | | | | 7.019842 | | | | 1976.531 | | | 93.0625 | |  | |  | |  |
| 37.48333 | | 431.0251 | | | | 7.022143 | | | | 1975.766 | | | 93.11719 | |  | |  | |  |
| 37.49167 | | 431.1132 | | | | 7.022506 | | | | 1975.016 | | | 93.20313 | |  | |  | |  |
| 37.5 | | 431.204 | | | | 7.025159 | | | | 1974.234 | | | 93.28125 | |  | |  | |  |
| 37.50833 | | 431.2935 | | | | 7.027839 | | | | 1973.438 | | | 93.0625 | |  | |  | |  |
| 37.51667 | | 431.3801 | | | | 7.026948 | | | | 1972.648 | | | 93.17188 | |  | |  | |  |
| 37.525 | | 431.4724 | | | | 7.029668 | | | | 1971.859 | | | 93.35156 | |  | |  | |  |
| 37.53333 | | 431.5646 | | | | 7.032583 | | | | 1971.078 | | | 93.32031 | |  | |  | |  |
| 37.54167 | | 431.6534 | | | | 7.032664 | | | | 1970.297 | | | 93.32813 | |  | |  | |  |
| 37.55 | | 431.7421 | | | | 7.033757 | | | | 1969.508 | | | 93.3125 | |  | |  | |  |
| 37.55833 | | 431.8309 | | | | 7.038565 | | | | 1968.766 | | | 93.25 | |  | |  | |  |
| 37.56667 | | 431.9225 | | | | 7.038886 | | | | 1967.969 | | | 93.29688 | |  | |  | |  |
| 37.575 | | 432.0126 | | | | 7.038113 | | | | 1967.172 | | | 93.38281 | |  | |  | |  |
| 37.58333 | | 432.1021 | | | | 7.043269 | | | | 1966.406 | | | 93.44531 | |  | |  | |  |
[truncated: 243,890 more chars]
