# Supplementary material for: Characterization dataset for pre- and post-irradiated shrimp waste chitosan
Source: Data Brief. 2020 Jul 25;32:106081. doi: 10.1016/j.dib.2020.106081 (PMC7397402; doi:10.1016/j.dib.2020.106081)
Supplement: Supplementary file 1 [file mmc1.zip › TGA-CH10.docx]

| Module | | | TG/DTA | | | | | | | | | |  | | | |  | | |  | |  |  |  |  |  |
| --- | --- | --- | --- | --- | --- | --- | --- | --- | --- | --- | --- | --- | --- | --- | --- | --- | --- | --- | --- | --- | --- | --- | --- | --- | --- | --- |
| Channel | | | 1 | | | | | | | | | |  | | | |  | | |  | |  |  |  |  |  |
| Data Name | | | S-Ch-2,(30-03-2017) | | | | | | | | | | | | | |  | | |  | |  |  |  |  |  |
| Measurement Time | | | 3/30/2017 6:06:56 PM | | | | | | | | | | | | | | | | |  | |  |  |  |  |  |
| Sample Name | | | S-Ch-2 | | | | | | | | | |  | | | |  | | |  | |  |  |  |  |  |
| Sample Weight | | | 5.255 | | | | | | | | | | mg | | | |  | | |  | |  |  |  |  |  |
| Reference Name | | | Alumina | | | | | | | | | |  | | | |  | | |  | |  |  |  |  |  |
| Reference Weight | | | 5.5 | | | | | | | | | | mg | | | |  | | |  | |  |  |  |  |  |
| Temperature Program | | | | | | | | | | | | | Cel | | | | Cel | | | Cel/min | | min | s | Gas1 | Gas2 | Store |
|  | | | 1 | | | | | | | | | | 50 | | | | 650 | | | 10 | | 0 | 0.5 | Off | Off | On |
|  | | | Temperature Program Mode | | | | | | | | | | Lamp | | | |  | | |  | |  |  |  |  |  |
|  | |  | | | |  | | |  | | |  | | | |  | | |  | |
|  | |  | | | |  | | |  | | |  | | | |  | | |  | |
|  | |  | | | |  | | |  | | |  | | | |  | | |  | |
|  | |  | | | |  | | |  | | |  | | | |  | | |  | |
|  | |  | | |  | | |  | | |  | | | |  | | |
|  |  | | |  | | |  | | |  | | | |
|  | |  | | | |  | | |  | | |  | | | |  | | |  | |
|  | | |  | | | | | | | | | |  | | | |  | | |  | |  |  |  |  |  |
|  | | |  | | | | | | | | | |  | | | |  | | |  | |  |  |  |  |  |
| Time | | | Temp. | | | | | | | | | | DTA | | | | TG | | | DTG | |  |  |  |  |  |
| min | | | Cel | | | | | | | | | | uV | | | | ug | | | ug/min | |  |  |  |  |  |
| 0.008333 | | | 50.21592 | | | | | | | | | | -0.70781 | | | | 5248.742 | | | 53.98438 | |  |  |  |  |  |
| 0.016667 | | | 50.25343 | | | | | | | | | | -0.70751 | | | | 5248.281 | | | 54.16406 | |  |  |  |  |  |
| 0.025 | | | 50.28411 | | | | | | | | | | -0.70785 | | | | 5247.828 | | | 54.125 | |  |  |  |  |  |
| 0.033333 | | | 50.31821 | | | | | | | | | | -0.70793 | | | | 5247.359 | | | 54.28125 | |  |  |  |  |  |
| 0.041667 | | | 50.35344 | | | | | | | | | | -0.70809 | | | | 5246.875 | | | 54.34375 | |  |  |  |  |  |
| 0.05 | | | 50.38981 | | | | | | | | | | -0.7082 | | | | 5246.406 | | | 54.39063 | |  |  |  |  |  |
| 0.058333 | | | 50.41595 | | | | | | | | | | -0.70916 | | | | 5245.938 | | | 54.47656 | |  |  |  |  |  |
| 0.066667 | | | 50.44663 | | | | | | | | | | -0.70975 | | | | 5245.461 | | | 54.48438 | |  |  |  |  |  |
| 0.075 | | | 50.47732 | | | | | | | | | | -0.7101 | | | | 5245.031 | | | 54.47656 | |  |  |  |  |  |
| 0.083333 | | | 50.50573 | | | | | | | | | | -0.71027 | | | | 5244.547 | | | 54.28125 | |  |  |  |  |  |
| 0.091667 | | | 50.53415 | | | | | | | | | | -0.71057 | | | | 5244.102 | | | 54.17969 | |  |  |  |  |  |
| 0.1 | | | 50.56028 | | | | | | | | | | -0.71116 | | | | 5243.641 | | | 54.09375 | |  |  |  |  |  |
| 0.108333 | | | 50.59438 | | | | | | | | | | -0.71131 | | | | 5243.172 | | | 54.21094 | |  |  |  |  |  |
| 0.116667 | | | 50.62961 | | | | | | | | | | -0.711 | | | | 5242.727 | | | 54.04688 | |  |  |  |  |  |
| 0.125 | | | 50.65575 | | | | | | | | | | -0.71118 | | | | 5242.281 | | | 54.26563 | |  |  |  |  |  |
| 0.133333 | | | 50.68189 | | | | | | | | | | -0.71135 | | | | 5241.852 | | | 54.42969 | |  |  |  |  |  |
| 0.141667 | | | 50.71371 | | | | | | | | | | -0.71085 | | | | 5241.414 | | | 54.33594 | |  |  |  |  |  |
| 0.15 | | | 50.73985 | | | | | | | | | | -0.71086 | | | | 5240.969 | | | 54.28125 | |  |  |  |  |  |
| 0.158333 | | | 50.76941 | | | | | | | | | | -0.71085 | | | | 5240.5 | | | 54.34375 | |  |  |  |  |  |
| 0.166667 | | | 50.80236 | | | | | | | | | | -0.7109 | | | | 5240.078 | | | 54.36719 | |  |  |  |  |  |
| 0.175 | | | 50.83419 | | | | | | | | | | -0.71087 | | | | 5239.602 | | | 54.38281 | |  |  |  |  |  |
| 0.183333 | | | 50.86487 | | | | | | | | | | -0.71072 | | | | 5239.125 | | | 54.45313 | |  |  |  |  |  |
| 0.191667 | | | 50.89328 | | | | | | | | | | -0.7107 | | | | 5238.68 | | | 54.41406 | |  |  |  |  |  |
| 0.2 | | | 50.91942 | | | | | | | | | | -0.71097 | | | | 5238.242 | | | 54.47656 | |  |  |  |  |  |
| 0.208333 | | | 50.94897 | | | | | | | | | | -0.7109 | | | | 5237.773 | | | 54.51563 | |  |  |  |  |  |
| 0.216667 | | | 50.97625 | | | | | | | | | | -0.7112 | | | | 5237.32 | | | 54.375 | |  |  |  |  |  |
| 0.225 | | | 51.01716 | | | | | | | | | | -0.71119 | | | | 5236.773 | | | 54.32031 | |  |  |  |  |  |
| 0.233333 | | | 51.05012 | | | | | | | | | | -0.71104 | | | | 5236.32 | | | 54.46875 | |  |  |  |  |  |
| 0.241667 | | | 51.07626 | | | | | | | | | | -0.711 | | | | 5235.961 | | | 54.41406 | |  |  |  |  |  |
| 0.25 | | | 51.10695 | | | | | | | | | | -0.71091 | | | | 5235.492 | | | 54.65625 | |  |  |  |  |  |
| 0.258333 | | | 51.13763 | | | | | | | | | | -0.71048 | | | | 5235.039 | | | 54.90625 | |  |  |  |  |  |
| 0.266667 | | | 51.17514 | | | | | | | | | | -0.70991 | | | | 5234.523 | | | 54.85938 | |  |  |  |  |  |
| 0.275 | | | 51.20583 | | | | | | | | | | -0.7096 | | | | 5234.07 | | | 55.125 | |  |  |  |  |  |
| 0.283333 | | | 51.23196 | | | | | | | | | | -0.70915 | | | | 5233.688 | | | 55.25781 | |  |  |  |  |  |
| 0.291667 | | | 51.27174 | | | | | | | | | | -0.70817 | | | | 5233.141 | | | 55.25 | |  |  |  |  |  |
| 0.3 | | | 51.30016 | | | | | | | | | | -0.7078 | | | | 5232.672 | | | 55.03906 | |  |  |  |  |  |
| 0.308333 | | | 51.32857 | | | | | | | | | | -0.70735 | | | | 5232.18 | | | 55.02344 | |  |  |  |  |  |
| 0.316667 | | | 51.35812 | | | | | | | | | | -0.70654 | | | | 5231.719 | | | 54.9375 | |  |  |  |  |  |
| 0.325 | | | 51.37858 | | | | | | | | | | -0.706 | | | | 5231.336 | | | 54.84375 | |  |  |  |  |  |
| 0.333333 | | | 51.4104 | | | | | | | | | | -0.70543 | | | | 5230.844 | | | 54.74219 | |  |  |  |  |  |
| 0.341667 | | | 51.43881 | | | | | | | | | | -0.70482 | | | | 5230.406 | | | 54.60938 | |  |  |  |  |  |
| 0.35 | | | 51.46722 | | | | | | | | | | -0.70391 | | | | 5229.945 | | | 54.46094 | |  |  |  |  |  |
| 0.358333 | | | 51.50132 | | | | | | | | | | -0.70304 | | | | 5229.406 | | | 54.14063 | |  |  |  |  |  |
| 0.366667 | | | 51.52973 | | | | | | | | | | -0.70234 | | | | 5228.969 | | | 54.16406 | |  |  |  |  |  |
| 0.375 | | | 51.55133 | | | | | | | | | | -0.70198 | | | | 5228.609 | | | 54.10938 | |  |  |  |  |  |
| 0.383333 | | | 51.58088 | | | | | | | | | | -0.70115 | | | | 5228.164 | | | 54.03906 | |  |  |  |  |  |
| 0.391667 | | | 51.60929 | | | | | | | | | | -0.70046 | | | | 5227.727 | | | 54.0625 | |  |  |  |  |  |
| 0.4 | | | 51.64452 | | | | | | | | | | -0.69969 | | | | 5227.195 | | | 54.32813 | |  |  |  |  |  |
| 0.408333 | | | 51.6718 | | | | | | | | | | -0.69875 | | | | 5226.844 | | | 54.17969 | |  |  |  |  |  |
| 0.416667 | | | 51.70589 | | | | | | | | | | -0.69755 | | | | 5226.336 | | | 54.20313 | |  |  |  |  |  |
| 0.425 | | | 51.73544 | | | | | | | | | | -0.69686 | | | | 5225.891 | | | 54.07031 | |  |  |  |  |  |
| 0.433333 | | | 51.75931 | | | | | | | | | | -0.69645 | | | | 5225.539 | | | 54.10938 | |  |  |  |  |  |
| 0.441667 | | | 51.78431 | | | | | | | | | | -0.69607 | | | | 5225.078 | | | 54.20313 | |  |  |  |  |  |
| 0.45 | | | 51.81727 | | | | | | | | | | -0.69575 | | | | 5224.5 | | | 54.24219 | |  |  |  |  |  |
| 0.458333 | | | 51.84909 | | | | | | | | | | -0.69524 | | | | 5224.07 | | | 54.07031 | |  |  |  |  |  |
| 0.466667 | | | 51.8741 | | | | | | | | | | -0.69484 | | | | 5223.703 | | | 54.08594 | |  |  |  |  |  |
| 0.475 | | | 51.91501 | | | | | | | | | | -0.6935 | | | | 5223.188 | | | 54.10938 | |  |  |  |  |  |
| 0.483333 | | | 51.94683 | | | | | | | | | | -0.69247 | | | | 5222.727 | | | 53.83594 | |  |  |  |  |  |
| 0.491667 | | | 51.96843 | | | | | | | | | | -0.69171 | | | | 5222.367 | | | 53.74219 | |  |  |  |  |  |
| 0.5 | | | 52.00025 | | | | | | | | | | -0.69079 | | | | 5221.82 | | | 53.58594 | |  |  |  |  |  |
| 0.508333 | | | 52.02639 | | | | | | | | | | -0.69003 | | | | 5221.477 | | | 53.70313 | |  |  |  |  |  |
| 0.516667 | | | 52.06049 | | | | | | | | | | -0.68929 | | | | 5220.938 | | | 53.41406 | |  |  |  |  |  |
| 0.525 | | | 52.09117 | | | | | | | | | | -0.68837 | | | | 5220.547 | | | 53.375 | |  |  |  |  |  |
| 0.533333 | | | 52.13322 | | | | | | | | | | -0.68687 | | | | 5220.063 | | | 53.34375 | |  |  |  |  |  |
| 0.541667 | | | 52.15823 | | | | | | | | | | -0.68618 | | | | 5219.719 | | | 53.27344 | |  |  |  |  |  |
| 0.55 | | | 52.18777 | | | | | | | | | | -0.68545 | | | | 5219.297 | | | 53.20313 | |  |  |  |  |  |
| 0.558333 | | | 52.21846 | | | | | | | | | | -0.68458 | | | | 5218.828 | | | 53.0625 | |  |  |  |  |  |
| 0.566667 | | | 52.2446 | | | | | | | | | | -0.68402 | | | | 5218.422 | | | 53.33594 | |  |  |  |  |  |
| 0.575 | | | 52.27529 | | | | | | | | | | -0.68341 | | | | 5217.977 | | | 53.41406 | |  |  |  |  |  |
| 0.583333 | | | 52.31393 | | | | | | | | | | -0.68244 | | | | 5217.461 | | | 53.51563 | |  |  |  |  |  |
| 0.591667 | | | 52.33666 | | | | | | | | | | -0.68167 | | | | 5217.102 | | | 53.46094 | |  |  |  |  |  |
| 0.6 | | | 52.37075 | | | | | | | | | | -0.68027 | | | | 5216.664 | | | 53.65625 | |  |  |  |  |  |
| 0.608333 | | | 52.3969 | | | | | | | | | | -0.67928 | | | | 5216.25 | | | 53.89063 | |  |  |  |  |  |
| 0.616667 | | | 52.42872 | | | | | | | | | | -0.67796 | | | | 5215.773 | | | 53.98438 | |  |  |  |  |  |
| 0.625 | | | 52.46054 | | | | | | | | | | -0.67655 | | | | 5215.313 | | | 54.24219 | |  |  |  |  |  |
| 0.633333 | | | 52.49009 | | | | | | | | | | -0.6754 | | | | 5214.859 | | | 54.21875 | |  |  |  |  |  |
| 0.641667 | | | 52.51737 | | | | | | | | | | -0.67458 | | | | 5214.406 | | | 54.28125 | |  |  |  |  |  |
| 0.65 | | | 52.5526 | | | | | | | | | | -0.67307 | | | | 5213.93 | | | 54.26563 | |  |  |  |  |  |
| 0.658333 | | | 52.59124 | | | | | | | | | | -0.67144 | | | | 5213.352 | | | 54.27344 | |  |  |  |  |  |
| 0.666667 | | | 52.62306 | | | | | | | | | | -0.67031 | | | | 5212.883 | | | 54.25781 | |  |  |  |  |  |
| 0.675 | | | 52.65261 | | | | | | | | | | -0.66918 | | | | 5212.477 | | | 54.20313 | |  |  |  |  |  |
| 0.683333 | | | 52.69239 | | | | | | | | | | -0.66826 | | | | 5211.945 | | | 54.32813 | |  |  |  |  |  |
| 0.691667 | | | 52.72535 | | | | | | | | | | -0.66777 | | | | 5211.477 | | | 53.8125 | |  |  |  |  |  |
| 0.7 | | | 52.74808 | | | | | | | | | | -0.66759 | | | | 5211.125 | | | 53.57813 | |  |  |  |  |  |
| 0.708333 | | | 52.7799 | | | | | | | | | | -0.66653 | | | | 5210.68 | | | 53.50781 | |  |  |  |  |  |
| 0.716667 | | | 52.81286 | | | | | | | | | | -0.66549 | | | | 5210.211 | | | 53.80469 | |  |  |  |  |  |
| 0.725 | | | 52.84696 | | | | | | | | | | -0.66433 | | | | 5209.773 | | | 53.92969 | |  |  |  |  |  |
| 0.733333 | | | 52.87423 | | | | | | | | | | -0.66351 | | | | 5209.313 | | | 54.07031 | |  |  |  |  |  |
| 0.741667 | | | 52.90719 | | | | | | | | | | -0.66228 | | | | 5208.922 | | | 54.25781 | |  |  |  |  |  |
| 0.75 | | | 52.94015 | | | | | | | | | | -0.66114 | | | | 5208.516 | | | 54.28906 | |  |  |  |  |  |
| 0.758333 | | | 52.97425 | | | | | | | | | | -0.65977 | | | | 5207.992 | | | 54.6875 | |  |  |  |  |  |
| 0.766667 | | | 53.00493 | | | | | | | | | | -0.65815 | | | | 5207.5 | | | 54.86719 | |  |  |  |  |  |
| 0.775 | | | 53.03562 | | | | | | | | | | -0.65675 | | | | 5207.039 | | | 55.23438 | |  |  |  |  |  |
| 0.783333 | | | 53.06858 | | | | | | | | | | -0.65525 | | | | 5206.555 | | | 55.42188 | |  |  |  |  |  |
| 0.791667 | | | 53.09017 | | | | | | | | | | -0.65434 | | | | 5206.18 | | | 55.60156 | |  |  |  |  |  |
| 0.8 | | | 53.12313 | | | | | | | | | | -0.65332 | | | | 5205.711 | | | 55.57813 | |  |  |  |  |  |
| 0.808333 | | | 53.15495 | | | | | | | | | | -0.6525 | | | | 5205.219 | | | 55.67188 | |  |  |  |  |  |
| 0.816667 | | | 53.18905 | | | | | | | | | | -0.65113 | | | | 5204.734 | | | 55.67969 | |  |  |  |  |  |
| 0.825 | | | 53.2311 | | | | | | | | | | -0.64918 | | | | 5204.125 | | | 55.67969 | |  |  |  |  |  |
| 0.833333 | | | 53.25724 | | | | | | | | | | -0.64828 | | | | 5203.727 | | | 55.61719 | |  |  |  |  |  |
| 0.841667 | | | 53.28906 | | | | | | | | | | -0.64742 | | | | 5203.242 | | | 55.53125 | |  |  |  |  |  |
| 0.85 | | | 53.33339 | | | | | | | | | | -0.64606 | | | | 5202.68 | | | 55.67969 | |  |  |  |  |  |
| 0.858333 | | | 53.35952 | | | | | | | | | | -0.64549 | | | | 5202.313 | | | 55.85156 | |  |  |  |  |  |
| 0.866667 | | | 53.40044 | | | | | | | | | | -0.64445 | | | | 5201.758 | | | 55.98438 | |  |  |  |  |  |
| 0.875 | | | 53.43113 | | | | | | | | | | -0.64341 | | | | 5201.391 | | | 55.75 | |  |  |  |  |  |
| 0.883333 | | | 53.46181 | | | | | | | | | | -0.64268 | | | | 5200.938 | | | 55.84375 | |  |  |  |  |  |
| 0.891667 | | | 53.50841 | | | | | | | | | | -0.64104 | | | | 5200.383 | | | 55.98438 | |  |  |  |  |  |
| 0.9 | | | 53.53796 | | | | | | | | | | -0.64061 | | | | 5200.008 | | | 56.01563 | |  |  |  |  |  |
| 0.908333 | | | 53.57546 | | | | | | | | | | -0.64075 | | | | 5199.414 | | | 56.01563 | |  |  |  |  |  |
| 0.916667 | | | 53.60728 | | | | | | | | | | -0.64057 | | | | 5198.945 | | | 56.125 | |  |  |  |  |  |
| 0.925 | | | 53.63456 | | | | | | | | | | -0.64018 | | | | 5198.609 | | | 56.09375 | |  |  |  |  |  |
| 0.933333 | | | 53.67093 | | | | | | | | | | -0.64008 | | | | 5198.039 | | | 55.88281 | |  |  |  |  |  |
| 0.941667 | | | 53.7073 | | | | | | | | | | -0.63948 | | | | 5197.555 | | | 55.61719 | |  |  |  |  |  |
| 0.95 | | | 53.73344 | | | | | | | | | | -0.63921 | | | | 5197.18 | | | 55.45313 | |  |  |  |  |  |
| 0.958333 | | | 53.76413 | | | | | | | | | | -0.63903 | | | | 5196.703 | | | 55.60156 | |  |  |  |  |  |
| 0.966667 | | | 53.80163 | | | | | | | | | | -0.63834 | | | | 5196.227 | | | 55.52344 | |  |  |  |  |  |
| 0.975 | | | 53.83345 | | | | | | | | | | -0.63768 | | | | 5195.773 | | | 55.28125 | |  |  |  |  |  |
| 0.983333 | | | 53.87209 | | | | | | | | | | -0.63658 | | | | 5195.242 | | | 55.04688 | |  |  |  |  |  |
| 0.991667 | | | 53.90846 | | | | | | | | | | -0.63525 | | | | 5194.82 | | | 55.28125 | |  |  |  |  |  |
| 1 | | | 53.93687 | | | | | | | | | | -0.63437 | | | | 5194.477 | | | 55.20313 | |  |  |  |  |  |
| 1.008333 | | | 53.9812 | | | | | | | | | | -0.63298 | | | | 5193.891 | | | 55.20313 | |  |  |  |  |  |
| 1.016667 | | | 54.00961 | | | | | | | | | | -0.63228 | | | | 5193.531 | | | 55.125 | |  |  |  |  |  |
| 1.025 | | | 54.0528 | | | | | | | | | | -0.63085 | | | | 5193.008 | | | 55.3125 | |  |  |  |  |  |
| 1.033333 | | | 54.09258 | | | | | | | | | | -0.62971 | | | | 5192.586 | | | 55.38281 | |  |  |  |  |  |
| 1.041667 | | | 54.1244 | | | | | | | | | | -0.62918 | | | | 5192.18 | | | 55.375 | |  |  |  |  |  |
| 1.05 | | | 54.16077 | | | | | | | | | | -0.62867 | | | | 5191.734 | | | 55.64063 | |  |  |  |  |  |
| 1.058333 | | | 54.20737 | | | | | | | | | | -0.62849 | | | | 5191.18 | | | 55.83594 | |  |  |  |  |  |
| 1.066667 | | | 54.24601 | | | | | | | | | | -0.62858 | | | | 5190.734 | | | 55.73438 | |  |  |  |  |  |
| 1.075 | | | 54.2767 | | | | | | | | | | -0.62813 | | | | 5190.359 | | | 55.4375 | |  |  |  |  |  |
| 1.083333 | | | 54.32329 | | | | | | | | | | -0.62742 | | | | 5189.781 | | | 55.47656 | |  |  |  |  |  |
| 1.091667 | | | 54.35966 | | | | | | | | | | -0.62734 | | | | 5189.328 | | | 55.53906 | |  |  |  |  |  |
| 1.1 | | | 54.3983 | | | | | | | | | | -0.62711 | | | | 5188.82 | | | 55.64063 | |  |  |  |  |  |
| 1.108333 | | | 54.43808 | | | | | | | | | | -0.62699 | | | | 5188.336 | | | 55.78125 | |  |  |  |  |  |
| 1.116667 | | | 54.47445 | | | | | | | | | | -0.62682 | | | | 5187.891 | | | 55.85938 | |  |  |  |  |  |
| 1.125 | | | 54.50286 | | | | | | | | | | -0.62627 | | | | 5187.555 | | | 55.96875 | |  |  |  |  |  |
| 1.133333 | | | 54.54832 | | | | | | | | | | -0.62559 | | | | 5186.992 | | | 55.58594 | |  |  |  |  |  |
| 1.141667 | | | 54.58583 | | | | | | | | | | -0.62528 | | | | 5186.523 | | | 55.40625 | |  |  |  |  |  |
| 1.15 | | | 54.61083 | | | | | | | | | | -0.62535 | | | | 5186.133 | | | 55.58594 | |  |  |  |  |  |
| 1.158333 | | | 54.64947 | | | | | | | | | | -0.62533 | | | | 5185.664 | | | 55.78906 | |  |  |  |  |  |
| 1.166667 | | | 54.68925 | | | | | | | | | | -0.62503 | | | | 5185.18 | | | 55.64844 | |  |  |  |  |  |
| 1.175 | | | 54.73698 | | | | | | | | | | -0.62443 | | | | 5184.602 | | | 55.84375 | |  |  |  |  |  |
| 1.183333 | | | 54.76994 | | | | | | | | | | -0.6242 | | | | 5184.273 | | | 55.89063 | |  |  |  |  |  |
| 1.191667 | | | 54.81086 | | | | | | | | | | -0.62383 | | | | 5183.836 | | | 55.78125 | |  |  |  |  |  |
| 1.2 | | | 54.84836 | | | | | | | | | | -0.62392 | | | | 5183.344 | | | 55.5625 | |  |  |  |  |  |
| 1.208333 | | | 54.887 | | | | | | | | | | -0.6241 | | | | 5182.852 | | | 55.40625 | |  |  |  |  |  |
| 1.216667 | | | 54.92905 | | | | | | | | | | -0.62284 | | | | 5182.406 | | | 55.5625 | |  |  |  |  |  |
| 1.225 | | | 54.96883 | | | | | | | | | | -0.6214 | | | | 5181.922 | | | 55.77344 | |  |  |  |  |  |
| 1.233333 | | | 55.02225 | | | | | | | | | | -0.62001 | | | | 5181.367 | | | 55.88281 | |  |  |  |  |  |
| 1.241667 | | | 55.0518 | | | | | | | | | | -0.61991 | | | | 5181 | | | 55.80469 | |  |  |  |  |  |
| 1.25 | | | 55.09726 | | | | | | | | | | -0.61971 | | | | 5180.469 | | | 56.25 | |  |  |  |  |  |
| 1.258333 | | | 55.12908 | | | | | | | | | | -0.61903 | | | | 5180.117 | | | 56.28906 | |  |  |  |  |  |
| 1.266667 | | | 55.17682 | | | | | | | | | | -0.6185 | | | | 5179.531 | | | 56.35938 | |  |  |  |  |  |
| 1.275 | | | 55.2075 | | | | | | | | | | -0.6188 | | | | 5179.141 | | | 56.35938 | |  |  |  |  |  |
| 1.283333 | | | 55.2541 | | | | | | | | | | -0.61915 | | | | 5178.563 | | | 56.26563 | |  |  |  |  |  |
| 1.291667 | | | 55.29615 | | | | | | | | | | -0.6196 | | | | 5178.086 | | | 56.45313 | |  |  |  |  |  |
| 1.3 | | | 55.32684 | | | | | | | | | | -0.62024 | | | | 5177.656 | | | 56.28906 | |  |  |  |  |  |
| 1.308333 | | | 55.36889 | | | | | | | | | | -0.62043 | | | | 5177.195 | | | 56.21875 | |  |  |  |  |  |
| 1.316667 | | | 55.41662 | | | | | | | | | | -0.62121 | | | | 5176.617 | | | 56.23438 | |  |  |  |  |  |
| 1.325 | | | 55.44844 | | | | | | | | | | -0.62165 | | | | 5176.25 | | | 56.3125 | |  |  |  |  |  |
| 1.333333 | | | 55.49959 | | | | | | | | | | -0.62233 | | | | 5175.695 | | | 56.32813 | |  |  |  |  |  |
| 1.341667 | | | 55.54391 | | | | | | | | | | -0.62243 | | | | 5175.211 | | | 56.20313 | |  |  |  |  |  |
| 1.35 | | | 55.57914 | | | | | | | | | | -0.62186 | | | | 5174.859 | | | 56.15625 | |  |  |  |  |  |
| 1.358333 | | | 55.6246 | | | | | | | | | | -0.62167 | | | | 5174.391 | | | 56.24219 | |  |  |  |  |  |
| 1.366667 | | | 55.66893 | | | | | | | | | | -0.62212 | | | | 5173.938 | | | 56.38281 | |  |  |  |  |  |
| 1.375 | | | 55.71439 | | | | | | | | | | -0.62259 | | | | 5173.453 | | | 56.35938 | |  |  |  |  |  |
| 1.383333 | | | 55.75985 | | | | | | | | | | -0.62292 | | | | 5172.984 | | | 56.44531 | |  |  |  |  |  |
| 1.391667 | | | 55.80645 | | | | | | | | | | -0.62228 | | | | 5172.523 | | | 56.57031 | |  |  |  |  |  |
| 1.4 | | | 55.8485 | | | | | | | | | | -0.62203 | | | | 5172.07 | | | 56.60156 | |  |  |  |  |  |
| 1.408333 | | | 55.89396 | | | | | | | | | | -0.62181 | | | | 5171.594 | | | 56.46094 | |  |  |  |  |  |
| 1.416667 | | | 55.94397 | | | | | | | | | | -0.62223 | | | | 5171.023 | | | 56.42969 | |  |  |  |  |  |
| 1.425 | | | 55.98715 | | | | | | | | | | -0.62257 | | | | 5170.539 | | | 56.4375 | |  |  |  |  |  |
| 1.433333 | | | 56.02125 | | | | | | | | | | -0.6229 | | | | 5170.156 | | | 56.63281 | |  |  |  |  |  |
| 1.441667 | | | 56.07012 | | | | | | | | | | -0.62355 | | | | 5169.555 | | | 56.79688 | |  |  |  |  |  |
| 1.45 | | | 56.11671 | | | | | | | | | | -0.62383 | | | | 5169.102 | | | 57.07813 | |  |  |  |  |  |
| 1.458333 | | | 56.15308 | | | | | | | | | | -0.62419 | | | | 5168.727 | | | 56.94531 | |  |  |  |  |  |
| 1.466667 | | | 56.19627 | | | | | | | | | | -0.62469 | | | | 5168.266 | | | 56.89063 | |  |  |  |  |  |
| 1.475 | | | 56.24628 | | | | | | | | | | -0.62505 | | | | 5167.703 | | | 56.64844 | |  |  |  |  |  |
| 1.483333 | | | 56.28719 | | | | | | | | | | -0.62489 | | | | 5167.305 | | | 56.71094 | |  |  |  |  |  |
| 1.491667 | | | 56.33152 | | | | | | | | | | -0.62573 | | | | 5166.805 | | | 56.89063 | |  |  |  |  |  |
| 1.5 | | | 56.37811 | | | | | | | | | | -0.62648 | | | | 5166.305 | | | 56.88281 | |  |  |  |  |  |
| 1.508333 | | | 56.42471 | | | | | | | | | | -0.62715 | | | | 5165.836 | | | 57.14063 | |  |  |  |  |  |
| 1.516667 | | | 56.47472 | | | | | | | | | | -0.62733 | | | | 5165.266 | | | 57.10156 | |  |  |  |  |  |
| 1.525 | | | 56.52018 | | | | | | | | | | -0.62741 | | | | 5164.836 | | | 57.17188 | |  |  |  |  |  |
| 1.533333 | | | 56.55428 | | | | | | | | | | -0.62807 | | | | 5164.453 | | | 56.91406 | |  |  |  |  |  |
| 1.541667 | | | 56.5986 | | | | | | | | | | -0.62903 | | | | 5163.961 | | | 56.97656 | |  |  |  |  |  |
| 1.55 | | | 56.64065 | | | | | | | | | | -0.63043 | | | | 5163.477 | | | 57.375 | |  |  |  |  |  |
| 1.558333 | | | 56.69407 | | | | | | | | | | -0.63112 | | | | 5162.875 | | | 57.48438 | |  |  |  |  |  |
| 1.566667 | | | 56.73498 | | | | | | | | | | -0.63145 | | | | 5162.406 | | | 57.73438 | |  |  |  |  |  |
| 1.575 | | | 56.78613 | | | | | | | | | | -0.63146 | | | | 5161.922 | | | 58 | |  |  |  |  |  |
| 1.583333 | | | 56.83158 | | | | | | | | | | -0.63218 | | | | 5161.484 | | | 57.84375 | |  |  |  |  |  |
| 1.591667 | | | 56.86682 | | | | | | | | | | -0.63302 | | | | 5161.086 | | | 58.20313 | |  |  |  |  |  |
| 1.6 | | | 56.92591 | | | | | | | | | | -0.63374 | | | | 5160.469 | | | 58.27344 | |  |  |  |  |  |
| 1.608333 | | | 56.96115 | | | | | | | | | | -0.63453 | | | | 5160.078 | | | 58 | |  |  |  |  |  |
| 1.616667 | | | 57.0157 | | | | | | | | | | -0.63643 | | | | 5159.461 | | | 58.34375 | |  |  |  |  |  |
| 1.625 | | | 57.0532 | | | | | | | | | | -0.63755 | | | | 5159.039 | | | 58.49219 | |  |  |  |  |  |
| 1.633333 | | | 57.10321 | | | | | | | | | | -0.63845 | | | | 5158.57 | | | 58.60156 | |  |  |  |  |  |
| 1.641667 | | | 57.16117 | | | | | | | | | | -0.63936 | | | | 5157.945 | | | 58.78125 | |  |  |  |  |  |
| 1.65 | | | 57.20322 | | | | | | | | | | -0.63963 | | | | 5157.547 | | | 58.59375 | |  |  |  |  |  |
| 1.658333 | | | 57.24755 | | | | | | | | | | -0.641 | | | | 5157.094 | | | 58.625 | |  |  |  |  |  |
| 1.666667 | | | 57.3021 | | | | | | | | | | -0.64222 | | | | 5156.461 | | | 59.03125 | |  |  |  |  |  |
| 1.675 | | | 57.33961 | | | | | | | | | | -0.64291 | | | | 5156.055 | | | 58.82813 | |  |  |  |  |  |
| 1.683333 | | | 57.3953 | | | | | | | | | | -0.64363 | | | | 5155.453 | | | 59.00781 | |  |  |  |  |  |
| 1.691667 | | | 57.43735 | | | | | | | | | | -0.64379 | | | | 5155.047 | | | 59.30469 | |  |  |  |  |  |
| 1.7 | | | 57.49304 | | | | | | | | | | -0.64526 | | | | 5154.484 | | | 59.46875 | |  |  |  |  |  |
| 1.708333 | | | 57.53281 | | | | | | | | | | -0.64613 | | | | 5154.094 | | | 59.47656 | |  |  |  |  |  |
| 1.716667 | | | 57.58168 | | | | | | | | | | -0.64742 | | | | 5153.57 | | | 59.57031 | |  |  |  |  |  |
| 1.725 | | | 57.63737 | | | | | | | | | | -0.64849 | | | | 5152.984 | | | 59.60156 | |  |  |  |  |  |
| 1.733333 | | | 57.67261 | | | | | | | | | | -0.64927 | | | | 5152.578 | | | 59.92188 | |  |  |  |  |  |
| 1.741667 | | | 57.73057 | | | | | | | | | | -0.65035 | | | | 5151.93 | | | 59.875 | |  |  |  |  |  |
| 1.75 | | | 57.77944 | | | | | | | | | | -0.6515 | | | | 5151.43 | | | 59.99219 | |  |  |  |  |  |
| 1.758333 | | | 57.81922 | | | | | | | | | | -0.65242 | | | | 5151.031 | | | 59.89063 | |  |  |  |  |  |
| 1.766667 | | | 57.86809 | | | | | | | | | | -0.65317 | | | | 5150.523 | | | 60.10938 | |  |  |  |  |  |
| 1.775 | | | 57.92832 | | | | | | | | | | -0.65434 | | | | 5149.922 | | | 60.22656 | |  |  |  |  |  |
| 1.783333 | | | 57.98288 | | | | | | | | | | -0.6553 | | | | 5149.383 | | | 60.21875 | |  |  |  |  |  |
| 1.791667 | | | 58.01925 | | | | | | | | | | -0.65638 | | | | 5148.977 | | | 60.26563 | |  |  |  |  |  |
| 1.8 | | | 58.06925 | | | | | | | | | | -0.65658 | | | | 5148.477 | | | 60.20313 | |  |  |  |  |  |
| 1.808333 | | | 58.13176 | | | | | | | | | | -0.65725 | | | | 5147.891 | | | 60.52344 | |  |  |  |  |  |
| 1.816667 | | | 58.18404 | | | | | | | | | | -0.65809 | | | | 5147.383 | | | 60.49219 | |  |  |  |  |  |
| 1.825 | | | 58.23518 | | | | | | | | | | -0.65891 | | | | 5146.859 | | | 60.54688 | |  |  |  |  |  |
| 1.833333 | | | 58.28489 | | | | | | | | | | -0.6594 | | | | 5146.352 | | | 60.45313 | |  |  |  |  |  |
| 1.841667 | | | 58.33424 | | | | | | | | | | -0.66028 | | | | 5145.852 | | | 60.63281 | |  |  |  |  |  |
| 1.85 | | | 58.3825 | | | | | | | | | | -0.66164 | | | | 5145.344 | | | 60.60938 | |  |  |  |  |  |
| 1.858333 | | | 58.43076 | | | | | | | | | | -0.663 | | | | 5144.813 | | | 60.5 | |  |  |  |  |  |
| 1.866667 | | | 58.47573 | | | | | | | | | | -0.6643 | | | | 5144.313 | | | 60.40625 | |  |  |  |  |  |
| 1.875 | | | 58.52509 | | | | | | | | | | -0.66503 | | | | 5143.813 | | | 60.57813 | |  |  |  |  |  |
| 1.883333 | | | 58.57444 | | | | | | | | | | -0.66658 | | | | 5143.32 | | | 60.61719 | |  |  |  |  |  |
| 1.891667 | | | 58.62599 | | | | | | | | | | -0.66801 | | | | 5142.789 | | | 60.42969 | |  |  |  |  |  |
| 1.9 | | | 58.67754 | | | | | | | | | | -0.66946 | | | | 5142.289 | | | 60.27344 | |  |  |  |  |  |
| 1.908333 | | | 58.73019 | | | | | | | | | | -0.6705 | | | | 5141.805 | | | 60.17188 | |  |  |  |  |  |
| 1.916667 | | | 58.78393 | | | | | | | | | | -0.67155 | | | | 5141.32 | | | 60.34375 | |  |  |  |  |  |
| 1.925 | | | 58.83219 | | | | | | | | | | -0.67343 | | | | 5140.789 | | | 60.20313 | |  |  |  |  |  |
| 1.933333 | | | 58.88374 | | | | | | | | | | -0.67509 | | | | 5140.289 | | | 60.41406 | |  |  |  |  |  |
| 1.941667 | | | 58.93529 | | | | | | | | | | -0.67644 | | | | 5139.813 | | | 60.48438 | |  |  |  |  |  |
| 1.95 | | | 58.98684 | | | | | | | | | | -0.67738 | | | | 5139.336 | | | 60.625 | |  |  |  |  |  |
| 1.958333 | | | 59.0351 | | | | | | | | | | -0.67876 | | | | 5138.844 | | | 60.83594 | |  |  |  |  |  |
| 1.966667 | | | 59.08665 | | | | | | | | | | -0.67995 | | | | 5138.313 | | | 60.82813 | |  |  |  |  |  |
| 1.975 | | | 59.13491 | | | | | | | | | | -0.68109 | | | | 5137.828 | | | 61.04688 | |  |  |  |  |  |
| 1.983333 | | | 59.18426 | | | | | | | | | | -0.68219 | | | | 5137.305 | | | 61.33594 | |  |  |  |  |  |
| 1.991667 | | | 59.23362 | | | | | | | | | | -0.68356 | | | | 5136.789 | | | 61.74219 | |  |  |  |  |  |
| 2 | | | 59.28188 | | | | | | | | | | -0.68548 | | | | 5136.258 | | | 61.82031 | |  |  |  |  |  |
| 2.008333 | | | 59.33452 | | | | | | | | | | -0.68716 | | | | 5135.734 | | | 62.0625 | |  |  |  |  |  |
| 2.016667 | | | 59.38607 | | | | | | | | | | -0.68862 | | | | 5135.211 | | | 62.10156 | |  |  |  |  |  |
| 2.025 | | | 59.43433 | | | | | | | | | | -0.6902 | | | | 5134.688 | | | 62.27344 | |  |  |  |  |  |
| 2.033333 | | | 59.48808 | | | | | | | | | | -0.6923 | | | | 5134.125 | | | 62.1875 | |  |  |  |  |  |
| 2.041667 | | | 59.54292 | | | | | | | | | | -0.69395 | | | | 5133.555 | | | 62.15625 | |  |  |  |  |  |
| 2.05 | | | 59.59446 | | | | | | | | | | -0.69549 | | | | 5133.039 | | | 62.375 | |  |  |  |  |  |
| 2.058333 | | | 59.64602 | | | | | | | | | | -0.6967 | | | | 5132.492 | | | 62.5625 | |  |  |  |  |  |
| 2.066667 | | | 59.69866 | | | | | | | | | | -0.69811 | | | | 5131.961 | | | 62.86719 | |  |  |  |  |  |
| 2.075 | | | 59.75021 | | | | | | | | | | -0.69963 | | | | 5131.43 | | | 62.91406 | |  |  |  |  |  |
| 2.083333 | | | 59.80286 | | | | | | | | | | -0.701 | | | | 5130.914 | | | 62.84375 | |  |  |  |  |  |
| 2.091667 | | | 59.85441 | | | | | | | | | | -0.70222 | | | | 5130.406 | | | 62.9375 | |  |  |  |  |  |
| 2.1 | | | 59.90705 | | | | | | | | | | -0.70365 | | | | 5129.859 | | | 63.09375 | |  |  |  |  |  |
| 2.108333 | | | 59.9597 | | | | | | | | | | -0.70488 | | | | 5129.305 | | | 63.41406 | |  |  |  |  |  |
| 2.116667 | | | 60.01125 | | | | | | | | | | -0.70592 | | | | 5128.742 | | | 63.59375 | |  |  |  |  |  |
| 2.125 | | | 60.0639 | | | | | | | | | | -0.70689 | | | | 5128.227 | | | 63.84375 | |  |  |  |  |  |
| 2.133333 | | | 60.11874 | | | | | | | | | | -0.70843 | | | | 5127.703 | | | 63.82031 | |  |  |  |  |  |
| 2.141667 | | | 60.16809 | | | | | | | | | | -0.71012 | | | | 5127.164 | | | 63.80469 | |  |  |  |  |  |
| 2.15 | | | 60.22293 | | | | | | | | | | -0.71126 | | | | 5126.617 | | | 63.625 | |  |  |  |  |  |
| 2.158333 | | | 60.27668 | | | | | | | | | | -0.71255 | | | | 5126.039 | | | 63.64063 | |  |  |  |  |  |
| 2.166667 | | | 60.33261 | | | | | | | | | | -0.71428 | | | | 5125.5 | | | 63.65625 | |  |  |  |  |  |
| 2.175 | | | 60.38416 | | | | | | | | | | -0.71627 | | | | 5124.93 | | | 63.69531 | |  |  |  |  |  |
| 2.183333 | | | 60.439 | | | | | | | | | | -0.71812 | | | | 5124.406 | | | 63.8125 | |  |  |  |  |  |
| 2.191667 | | | 60.49384 | | | | | | | | | | -0.71937 | | | | 5123.875 | | | 63.60938 | |  |  |  |  |  |
| 2.2 | | | 60.54978 | | | | | | | | | | -0.72075 | | | | 5123.383 | | | 63.40625 | |  |  |  |  |  |
| 2.208333 | | | 60.60681 | | | | | | | | | | -0.72229 | | | | 5122.836 | | | 63.42969 | |  |  |  |  |  |
| 2.216667 | | | 60.65836 | | | | | | | | | | -0.72408 | | | | 5122.313 | | | 63.5625 | |  |  |  |  |  |
| 2.225 | | | 60.71539 | | | | | | | | | | -0.72544 | | | | 5121.773 | | | 63.61719 | |  |  |  |  |  |
| 2.233333 | | | 60.76914 | | | | | | | | | | -0.72733 | | | | 5121.242 | | | 63.78125 | |  |  |  |  |  |
| 2.241667 | | | 60.82288 | | | | | | | | | | -0.72934 | | | | 5120.734 | | | 63.78125 | |  |  |  |  |  |
| 2.25 | | | 60.87333 | | | | | | | | | | -0.73112 | | | | 5120.242 | | | 64.23438 | |  |  |  |  |  |
| 2.258333 | | | 60.92817 | | | | | | | | | | -0.73246 | | | | 5119.711 | | | 64.30469 | |  |  |  |  |  |
| 2.266667 | | | 60.98192 | | | | | | | | | | -0.73384 | | | | 5119.172 | | | 64.39844 | |  |  |  |  |  |
| 2.275 | | | 61.03785 | | | | | | | | | | -0.73563 | | | | 5118.633 | | | 64.45313 | |  |  |  |  |  |
| 2.283333 | | | 61.09489 | | | | | | | | | | -0.7374 | | | | 5118.078 | | | 64.76563 | |  |  |  |  |  |
| 2.291667 | | | 61.14753 | | | | | | | | | | -0.73879 | | | | 5117.547 | | | 64.99219 | |  |  |  |  |  |
| 2.3 | | | 61.20347 | | | | | | | | | | -0.7398 | | | | 5116.953 | | | 65.25781 | |  |  |  |  |  |
| 2.308333 | | | 61.2616 | | | | | | | | | | -0.74179 | | | | 5116.422 | | | 65.54688 | |  |  |  |  |  |
| 2.316667 | | | 61.31644 | | | | | | | | | | -0.74385 | | | | 5115.875 | | | 65.80469 | |  |  |  |  |  |
| 2.325 | | | 61.37567 | | | | | | | | | | -0.74555 | | | | 5115.328 | | | 66.11719 | |  |  |  |  |  |
| 2.333333 | | | 61.43489 | | | | | | | | | | -0.74719 | | | | 5114.75 | | | 65.89844 | |  |  |  |  |  |
| 2.341667 | | | 61.49303 | | | | | | | | | | -0.74895 | | | | 5114.18 | | | 65.95313 | |  |  |  |  |  |
| 2.35 | | | 61.54896 | | | | | | | | | | -0.75064 | | | | 5113.594 | | | 65.91406 | |  |  |  |  |  |
| 2.358333 | | | 61.60929 | | | | | | | | | | -0.75191 | | | | 5113.008 | | | 66.14844 | |  |  |  |  |  |
| 2.366667 | | | 61.66303 | | | | | | | | | | -0.75359 | | | | 5112.422 | | | 66.35938 | |  |  |  |  |  |
| 2.375 | | | 61.72006 | | | | | | | | | | -0.75572 | | | | 5111.844 | | | 66.17969 | |  |  |  |  |  |
| 2.383333 | | | 61.7771 | | | | | | | | | | -0.7571 | | | | 5111.32 | | | 66.08594 | |  |  |  |  |  |
| 2.391667 | | | 61.83523 | | | | | | | | | | -0.75854 | | | | 5110.766 | | | 65.82813 | |  |  |  |  |  |
| 2.4 | | | 61.88897 | | | | | | | | | | -0.76065 | | | | 5110.219 | | | 65.52344 | |  |  |  |  |  |
| 2.408333 | | | 61.94381 | | | | | | | | | | -0.76318 | | | | 5109.633 | | | 65.375 | |  |  |  |  |  |
| 2.416667 | | | 62.00194 | | | | | | | | | | -0.7647 | | | | 5109.047 | | | 65.46094 | |  |  |  |  |  |
| 2.425 | | | 62.06117 | | | | | | | | | | -0.76584 | | | | 5108.531 | | | 65.74219 | |  |  |  |  |  |
| 2.433333 | | | 62.1193 | | | | | | | | | | -0.76748 | | | | 5108 | | | 65.82813 | |  |  |  |  |  |
| 2.441667 | | | 62.17195 | | | | | | | | | | -0.76967 | | | | 5107.492 | | | 65.61719 | |  |  |  |  |  |
| 2.45 | | | 62.23008 | | | | | | | | | | -0.77139 | | | | 5106.992 | | | 65.46094 | |  |  |  |  |  |
| 2.458333 | | | 62.28821 | | | | | | | | | | -0.77289 | | | | 5106.453 | | | 65.80469 | |  |  |  |  |  |
| 2.466667 | | | 62.34853 | | | | | | | | | | -0.77473 | | | | 5105.906 | | | 66.11719 | |  |  |  |  |  |
| 2.475 | | | 62.40337 | | | | | | | | | | -0.7769 | | | | 5105.32 | | | 66.17188 | |  |  |  |  |  |
| 2.483333 | | | 62.46589 | | | | | | | | | | -0.77839 | | | | 5104.766 | | | 66.33594 | |  |  |  |  |  |
| 2.491667 | | | 62.52512 | | | | | | | | | | -0.77993 | | | | 5104.242 | | | 66.46875 | |  |  |  |  |  |
| 2.5 | | | 62.57996 | | | | | | | | | | -0.78242 | | | | 5103.719 | | | 66.66406 | |  |  |  |  |  |
| 2.508333 | | | 62.63699 | | | | | | | | | | -0.78494 | | | | 5103.125 | | | 66.65625 | |  |  |  |  |  |
| 2.516667 | | | 62.69622 | | | | | | | | | | -0.78682 | | | | 5102.531 | | | 66.96094 | |  |  |  |  |  |
| 2.525 | | | 62.75764 | | | | | | | | | | -0.78827 | | | | 5101.961 | | | 67.29688 | |  |  |  |  |  |
| 2.533333 | | | 62.81686 | | | | | | | | | | -0.79084 | | | | 5101.391 | | | 67.375 | |  |  |  |  |  |
| 2.541667 | | | 62.87938 | | | | | | | | | | -0.79307 | | | | 5100.82 | | | 67.41406 | |  |  |  |  |  |
| 2.55 | | | 62.93642 | | | | | | | | | | -0.79543 | | | | 5100.25 | | | 67.48438 | |  |  |  |  |  |
| 2.558333 | | | 62.99674 | | | | | | | | | | -0.79736 | | | | 5099.695 | | | 67.63281 | |  |  |  |  |  |
| 2.566667 | | | 63.05706 | | | | | | | | | | -0.79972 | | | | 5099.094 | | | 67.53125 | |  |  |  |  |  |
| 2.575 | | | 63.11739 | | | | | | | | | | -0.80172 | | | | 5098.492 | | | 67.4375 | |  |  |  |  |  |
| 2.583333 | | | 63.181 | | | | | | | | | | -0.80343 | | | | 5097.914 | | | 67.67969 | |  |  |  |  |  |
| 2.591667 | | | 63.23913 | | | | | | | | | | -0.80518 | | | | 5097.359 | | | 67.73438 | |  |  |  |  |  |
| 2.6 | | | 63.30165 | | | | | | | | | | -0.80727 | | | | 5096.797 | | | 67.47656 | |  |  |  |  |  |
| 2.608333 | | | 63.36088 | | | | | | | | | | -0.80917 | | | | 5096.211 | | | 67.25 | |  |  |  |  |  |
| 2.616667 | | | 63.4201 | | | | | | | | | | -0.81071 | | | | 5095.664 | | | 67.42188 | |  |  |  |  |  |
| 2.625 | | | 63.47823 | | | | | | | | | | -0.8123 | | | | 5095.117 | | | 67.82813 | |  |  |  |  |  |
| 2.633333 | | | 63.53965 | | | | | | | | | | -0.81496 | | | | 5094.523 | | | 67.92188 | |  |  |  |  |  |
| 2.641667 | | | 63.60108 | | | | | | | | | | -0.81679 | | | | 5093.953 | | | 67.92188 | |  |  |  |  |  |
| 2.65 | | | 63.65921 | | | | | | | | | | -0.81857 | | | | 5093.43 | | | 68.05469 | |  |  |  |  |  |
| 2.658333 | | | 63.71734 | | | | | | | | | | -0.82061 | | | | 5092.898 | | | 68.32813 | |  |  |  |  |  |
| 2.666667 | | | 63.77766 | | | | | | | | | | -0.82306 | | | | 5092.313 | | | 68.41406 | |  |  |  |  |  |
| 2.675 | | | 63.84127 | | | | | | | | | | -0.82437 | | | | 5091.695 | | | 68.5625 | |  |  |  |  |  |
| 2.683333 | | | 63.9005 | | | | | | | | | | -0.82608 | | | | 5091.117 | | | 68.74219 | |  |  |  |  |  |
| 2.691667 | | | 63.96302 | | | | | | | | | | -0.82775 | | | | 5090.547 | | | 68.95313 | |  |  |  |  |  |
| 2.7 | | | 64.02663 | | | | | | | | | | -0.8293 | | | | 5089.953 | | | 69.27344 | |  |  |  |  |  |
| 2.708333 | | | 64.08915 | | | | | | | | | | -0.83057 | | | | 5089.352 | | | 69.0625 | |  |  |  |  |  |
| 2.716667 | | | 64.14948 | | | | | | | | | | -0.83199 | | | | 5088.766 | | | 69.15625 | |  |  |  |  |  |
| 2.725 | | | 64.2098 | | | | | | | | | | -0.83422 | | | | 5088.172 | | | 69.35156 | |  |  |  |  |  |
| 2.733333 | | | 64.27451 | | | | | | | | | | -0.83556 | | | | 5087.578 | | | 69.50781 | |  |  |  |  |  |
| 2.741667 | | | 64.33813 | | | | | | | | | | -0.83679 | | | | 5086.977 | | | 69.75 | |  |  |  |  |  |
| 2.75 | | | 64.40064 | | | | | | | | | | -0.83864 | | | | 5086.359 | | | 69.67969 | |  |  |  |  |  |
| 2.758333 | | | 64.46317 | | | | | | | | | | -0.8405 | | | | 5085.813 | | | 69.67969 | |  |  |  |  |  |
| 2.766667 | | | 64.52568 | | | | | | | | | | -0.84142 | | | | 5085.219 | | | 69.77344 | |  |  |  |  |  |
| 2.775 | | | 64.58601 | | | | | | | | | | -0.84305 | | | | 5084.617 | | | 69.59375 | |  |  |  |  |  |
| 2.783333 | | | 64.64742 | | | | | | | | | | -0.84563 | | | | 5084.023 | | | 69.44531 | |  |  |  |  |  |
| 2.791667 | | | 64.70775 | | | | | | | | | | -0.84785 | | | | 5083.414 | | | 69.34375 | |  |  |  |  |  |
| 2.8 | | | 64.76807 | | | | | | | | | | -0.85002 | | | | 5082.852 | | | 69.14063 | |  |  |  |  |  |
| 2.808333 | | | 64.83279 | | | | | | | | | | -0.85198 | | | | 5082.281 | | | 68.96875 | |  |  |  |  |  |
| 2.816667 | | | 64.8953 | | | | | | | | | | -0.85445 | | | | 5081.688 | | | 68.875 | |  |  |  |  |  |
| 2.825 | | | 64.96001 | | | | | | | | | | -0.85597 | | | | 5081.133 | | | 68.64063 | |  |  |  |  |  |
| 2.833333 | | | 65.02253 | | | | | | | | | | -0.85789 | | | | 5080.586 | | | 68.69531 | |  |  |  |  |  |
| 2.841667 | | | 65.08614 | | | | | | | | | | -0.86034 | | | | 5080.023 | | | 68.82813 | |  |  |  |  |  |
| 2.85 | | | 65.14757 | | | | | | | | | | -0.86284 | | | | 5079.484 | | | 68.82031 | |  |  |  |  |  |
| 2.858333 | | | 65.21557 | | | | | | | | | | -0.86462 | | | | 5078.922 | | | 69.08594 | |  |  |  |  |  |
| 2.866667 | | | 65.27589 | | | | | | | | | | -0.86713 | | | | 5078.367 | | | 68.9375 | |  |  |  |  |  |
| 2.875 | | | 65.3428 | | | | | | | | | | -0.86969 | | | | 5077.828 | | | 68.85938 | |  |  |  |  |  |
| 2.883333 | | | 65.4119 | | | | | | | | | | -0.87144 | | | | 5077.25 | | | 69.09375 | |  |  |  |  |  |
| 2.891667 | | | 65.47331 | | | | | | | | | | -0.87319 | | | | 5076.664 | | | 69.07813 | |  |  |  |  |  |
| 2.9 | | | 65.53364 | | | | | | | | | | -0.87516 | | | | 5076.086 | | | 69.39844 | |  |  |  |  |  |
| 2.908333 | | | 65.59725 | | | | | | | | | | -0.87744 | | | | 5075.469 | | | 69.42969 | |  |  |  |  |  |
| 2.916667 | | | 65.66087 | | | | | | | | | | -0.87879 | | | | 5074.922 | | | 69.39844 | |  |  |  |  |  |
| 2.925 | | | 65.72668 | | | | | | | | | | -0.88047 | | | | 5074.344 | | | 69.46875 | |  |  |  |  |  |
| 2.933333 | | | 65.79139 | | | | | | | | | | -0.88246 | | | | 5073.742 | | | 69.5625 | |  |  |  |  |  |
| 2.941667 | | | 65.855 | | | | | | | | | | -0.88431 | | | | 5073.172 | | | 69.59375 | |  |  |  |  |  |
| 2.95 | | | 65.923 | | | | | | | | | | -0.88563 | | | | 5072.547 | | | 69.89844 | |  |  |  |  |  |
| 2.958333 | | | 65.98442 | | | | | | | | | | -0.88791 | | | | 5071.961 | | | 70.35938 | |  |  |  |  |  |
| 2.966667 | | | 66.04803 | | | | | | | | | | -0.8903 | | | | 5071.398 | | | 70.4375 | |  |  |  |  |  |
| 2.975 | | | 66.11494 | | | | | | | | | | -0.89185 | | | | 5070.805 | | | 70.51563 | |  |  |  |  |  |
| 2.983333 | | | 66.18185 | | | | | | | | | | -0.89347 | | | | 5070.211 | | | 70.60938 | |  |  |  |  |  |
| 2.991667 | | | 66.24875 | | | | | | | | | | -0.89573 | | | | 5069.633 | | | 70.69531 | |  |  |  |  |  |
| 3 | | | 66.31237 | | | | | | | | | | -0.89849 | | | | 5069.016 | | | 70.95313 | |  |  |  |  |  |
| 3.008333 | | | 66.37379 | | | | | | | | | | -0.90025 | | | | 5068.359 | | | 70.92969 | |  |  |  |  |  |
| 3.016667 | | | 66.44179 | | | | | | | | | | -0.90181 | | | | 5067.773 | | | 70.80469 | |  |  |  |  |  |
| 3.025 | | | 66.5087 | | | | | | | | | | -0.90393 | | | | 5067.164 | | | 70.85156 | |  |  |  |  |  |
| 3.033333 | | | 66.5734 | | | | | | | | | | -0.90574 | | | | 5066.563 | | | 70.94531 | |  |  |  |  |  |
| 3.041667 | | | 66.64141 | | | | | | | | | | -0.90652 | | | | 5065.961 | | | 70.75 | |  |  |  |  |  |
| 3.05 | | | 66.70721 | | | | | | | | | | -0.90818 | | | | 5065.336 | | | 70.70313 | |  |  |  |  |  |
| 3.058333 | | | 66.77412 | | | | | | | | | | -0.91001 | | | | 5064.75 | | | 70.47656 | |  |  |  |  |  |
| 3.066667 | | | 66.83774 | | | | | | | | | | -0.91141 | | | | 5064.172 | | | 70.27344 | |  |  |  |  |  |
| 3.075 | | | 66.89806 | | | | | | | | | | -0.91364 | | | | 5063.578 | | | 70.50781 | |  |  |  |  |  |
| 3.083333 | | | 66.96497 | | | | | | | | | | -0.91571 | | | | 5062.969 | | | 70.46875 | |  |  |  |  |  |
| 3.091667 | | | 67.03407 | | | | | | | | | | -0.91711 | | | | 5062.406 | | | 70.23438 | |  |  |  |  |  |
| 3.1 | | | 67.09877 | | | | | | | | | | -0.91877 | | | | 5061.828 | | | 70.32031 | |  |  |  |  |  |
| 3.108333 | | | 67.16348 | | | | | | | | | | -0.9209 | | | | 5061.273 | | | 70.36719 | |  |  |  |  |  |
| 3.116667 | | | 67.22929 | | | | | | | | | | -0.92345 | | | | 5060.719 | | | 70.17188 | |  |  |  |  |  |
| 3.125 | | | 67.2951 | | | | | | | | | | -0.92473 | | | | 5060.094 | | | 70.21094 | |  |  |  |  |  |
| 3.133333 | | | 67.3642 | | | | | | | | | | -0.92625 | | | | 5059.516 | | | 70.4375 | |  |  |  |  |  |
| 3.141667 | | | 67.42891 | | | | | | | | | | -0.92842 | | | | 5058.953 | | | 70.84375 | |  |  |  |  |  |
| 3.15 | | | 67.49911 | | | | | | | | | | -0.93006 | | | | 5058.359 | | | 70.9375 | |  |  |  |  |  |
| 3.158333 | | | 67.56821 | | | | | | | | | | -0.93121 | | | | 5057.766 | | | 70.85156 | |  |  |  |  |  |
| 3.166667 | | | 67.6373 | | | | | | | | | | -0.93269 | | | | 5057.211 | | | 70.92969 | |  |  |  |  |  |
| 3.175 | | | 67.70201 | | | | | | | | | | -0.93488 | | | | 5056.625 | | | 71.375 | |  |  |  |  |  |
| 3.183333 | | | 67.76892 | | | | | | | | | | -0.9357 | | | | 5056.008 | | | 71.46094 | |  |  |  |  |  |
| 3.191667 | | | 67.83363 | | | | | | | | | | -0.93703 | | | | 5055.367 | | | 71.42188 | |  |  |  |  |  |
| 3.2 | | | 67.90273 | | | | | | | | | | -0.93837 | | | | 5054.758 | | | 71.53125 | |  |  |  |  |  |
| 3.208333 | | | 67.97073 | | | | | | | | | | -0.93945 | | | | 5054.195 | | | 71.60938 | |  |  |  |  |  |
| 3.216667 | | | 68.03654 | | | | | | | | | | -0.94042 | | | | 5053.594 | | | 71.71094 | |  |  |  |  |  |
| 3.225 | | | 68.10454 | | | | | | | | | | -0.9421 | | | | 5052.938 | | | 71.57031 | |  |  |  |  |  |
| 3.233333 | | | 68.17035 | | | | | | | | | | -0.94435 | | | | 5052.336 | | | 71.57813 | |  |  |  |  |  |
| 3.241667 | | | 68.24054 | | | | | | | | | | -0.94542 | | | | 5051.75 | | | 71.80469 | |  |  |  |  |  |
| 3.25 | | | 68.30525 | | | | | | | | | | -0.94766 | | | | 5051.141 | | | 71.875 | |  |  |  |  |  |
| 3.258333 | | | 68.37435 | | | | | | | | | | -0.94999 | | | | 5050.523 | | | 71.79688 | |  |  |  |  |  |
| 3.266667 | | | 68.44455 | | | | | | | | | | -0.95198 | | | | 5049.93 | | | 72.04688 | |  |  |  |  |  |
| 3.275 | | | 68.51255 | | | | | | | | | | -0.95388 | | | | 5049.344 | | | 72.07813 | |  |  |  |  |  |
| 3.283333 | | | 68.57945 | | | | | | | | | | -0.95657 | | | | 5048.742 | | | 72.09375 | |  |  |  |  |  |
| 3.291667 | | | 68.64635 | | | | | | | | | | -0.95882 | | | | 5048.117 | | | 72.29688 | |  |  |  |  |  |
| 3.3 | | | 68.71545 | | | | | | | | | | -0.95983 | | | | 5047.508 | | | 72.36719 | |  |  |  |  |  |
| 3.308333 | | | 68.78236 | | | | | | | | | | -0.96149 | | | | 5046.914 | | | 72.38281 | |  |  |  |  |  |
| 3.316667 | | | 68.85255 | | | | | | | | | | -0.96334 | | | | 5046.289 | | | 72.30469 | |  |  |  |  |  |
| 3.325 | | | 68.91946 | | | | | | | | | | -0.96451 | | | | 5045.68 | | | 72.28125 | |  |  |  |  |  |
| 3.333333 | | | 68.98746 | | | | | | | | | | -0.96616 | | | | 5045.078 | | | 72.25781 | |  |  |  |  |  |
| 3.341667 | | | 69.05656 | | | | | | | | | | -0.96791 | | | | 5044.453 | | | 72.22656 | |  |  |  |  |  |
| 3.35 | | | 69.12566 | | | | | | | | | | -0.96907 | | | | 5043.836 | | | 71.88281 | |  |  |  |  |  |
| 3.358333 | | | 69.19585 | | | | | | | | | | -0.96978 | | | | 5043.219 | | | 72.01563 | |  |  |  |  |  |
| 3.366667 | | | 69.26605 | | | | | | | | | | -0.97121 | | | | 5042.641 | | | 72.14063 | |  |  |  |  |  |
| 3.375 | | | 69.33405 | | | | | | | | | | -0.97247 | | | | 5042.039 | | | 72.08594 | |  |  |  |  |  |
| 3.383333 | | | 69.40096 | | | | | | | | | | -0.97373 | | | | 5041.438 | | | 72.01563 | |  |  |  |  |  |
| 3.391667 | | | 69.47444 | | | | | | | | | | -0.97548 | | | | 5040.836 | | | 72.07813 | |  |  |  |  |  |
| 3.4 | | | 69.54354 | | | | | | | | | | -0.9777 | | | | 5040.289 | | | 72.35156 | |  |  |  |  |  |
| 3.408333 | | | 69.61374 | | | | | | | | | | -0.97946 | | | | 5039.672 | | | 72.21094 | |  |  |  |  |  |
| 3.416667 | | | 69.68283 | | | | | | | | | | -0.98101 | | | | 5039.047 | | | 72.24219 | |  |  |  |  |  |
| 3.425 | | | 69.74974 | | | | | | | | | | -0.98343 | | | | 5038.461 | | | 72.25781 | |  |  |  |  |  |
| 3.433333 | | | 69.81774 | | | | | | | | | | -0.98557 | | | | 5037.867 | | | 72.30469 | |  |  |  |  |  |
| 3.441667 | | | 69.88574 | | | | | | | | | | -0.98685 | | | | 5037.258 | | | 72.42188 | |  |  |  |  |  |
| 3.45 | | | 69.95045 | | | | | | | | | | -0.98901 | | | | 5036.625 | | | 72.44531 | |  |  |  |  |  |
| 3.458333 | | | 70.02174 | | | | | | | | | | -0.99123 | | | | 5036.047 | | | 72.46875 | |  |  |  |  |  |
| 3.466667 | | | 70.09194 | | | | | | | | | | -0.99344 | | | | 5035.445 | | | 72.75781 | |  |  |  |  |  |
| 3.475 | | | 70.15994 | | | | | | | | | | -0.99546 | | | | 5034.844 | | | 73.00781 | |  |  |  |  |  |
| 3.483333 | | | 70.22904 | | | | | | | | | | -0.99814 | | | | 5034.227 | | | 72.94531 | |  |  |  |  |  |
| 3.491667 | | | 70.29691 | | | | | | | | | | -1.00043 | | | | 5033.617 | | | 72.89063 | |  |  |  |  |  |
| 3.5 | | | 70.36667 | | | | | | | | | | -1.00142 | | | | 5033.016 | | | 72.79688 | |  |  |  |  |  |
| 3.508333 | | | 70.43961 | | | | | | | | | | -1.00274 | | | | 5032.406 | | | 73.04688 | |  |  |  |  |  |
| 3.516667 | | | 70.50726 | | | | | | | | | | -1.00473 | | | | 5031.766 | | | 73.1875 | |  |  |  |  |  |
| 3.525 | | | 70.57597 | | | | | | | | | | -1.0061 | | | | 5031.125 | | | 73.125 | |  |  |  |  |  |
| 3.533333 | | | 70.64997 | | | | | | | | | | -1.00692 | | | | 5030.516 | | | 73.13281 | |  |  |  |  |  |
| 3.541667 | | | 70.71762 | | | | | | | | | | -1.00881 | | | | 5029.922 | | | 73.10156 | |  |  |  |  |  |
| 3.55 | | | 70.78528 | | | | | | | | | | -1.00989 | | | | 5029.32 | | | 73.00781 | |  |  |  |  |  |
| 3.558333 | | | 70.85188 | | | | | | | | | | -1.01024 | | | | 5028.688 | | | 72.74219 | |  |  |  |  |  |
| 3.566667 | | | 70.91953 | | | | | | | | | | -1.01147 | | | | 5028.055 | | | 72.98438 | |  |  |  |  |  |
| 3.575 | | | 70.99035 | | | | | | | | | | -1.01242 | | | | 5027.453 | | | 73.1875 | |  |  |  |  |  |
| 3.583333 | | | 71.06223 | | | | | | | | | | -1.01262 | | | | 5026.844 | | | 73.22656 | |  |  |  |  |  |
| 3.591667 | | | 71.12883 | | | | | | | | | | -1.01417 | | | | 5026.242 | | | 73.29688 | |  |  |  |  |  |
| 3.6 | | | 71.19754 | | | | | | | | | | -1.01576 | | | | 5025.648 | | | 73.14063 | |  |  |  |  |  |
| 3.608333 | | | 71.26625 | | | | | | | | | | -1.01678 | | | | 5025.078 | | | 72.94531 | |  |  |  |  |  |
| 3.616667 | | | 71.3339 | | | | | | | | | | -1.01806 | | | | 5024.438 | | | 72.83594 | |  |  |  |  |  |
| 3.625 | | | 71.40261 | | | | | | | | | | -1.01991 | | | | 5023.797 | | | 72.92188 | |  |  |  |  |  |
| 3.633333 | | | 71.47343 | | | | | | | | | | -1.0212 | | | | 5023.18 | | | 72.97656 | |  |  |  |  |  |
| 3.641667 | | | 71.54214 | | | | | | | | | | -1.02193 | | | | 5022.563 | | | 72.78125 | |  |  |  |  |  |
| 3.65 | | | 71.61614 | | | | | | | | | | -1.02403 | | | | 5021.977 | | | 72.57031 | |  |  |  |  |  |
| 3.658333 | | | 71.6838 | | | | | | | | | | -1.02602 | | | | 5021.391 | | | 72.69531 | |  |  |  |  |  |
| 3.666667 | | | 71.7504 | | | | | | | | | | -1.02729 | | | | 5020.797 | | | 72.69531 | |  |  |  |  |  |
| 3.675 | | | 71.82016 | | | | | | | | | | -1.02897 | | | | 5020.18 | | | 72.52344 | |  |  |  |  |  |
| 3.683333 | | | 71.89098 | | | | | | | | | | -1.03088 | | | | 5019.57 | | | 72.61719 | |  |  |  |  |  |
| 3.691667 | | | 71.96498 | | | | | | | | | | -1.03211 | | | | 5018.984 | | | 73.02344 | |  |  |  |  |  |
| 3.7 | | | 72.0358 | | | | | | | | | | -1.03298 | | | | 5018.398 | | | 73.0625 | |  |  |  |  |  |
| 3.708333 | | | 72.10769 | | | | | | | | | | -1.03433 | | | | 5017.789 | | | 72.875 | |  |  |  |  |  |
| 3.716667 | | | 72.17745 | | | | | | | | | | -1.03548 | | | | 5017.18 | | | 72.75 | |  |  |  |  |  |
| 3.725 | | | 72.2504 | | | | | | | | | | -1.03608 | | | | 5016.602 | | | 72.96875 | |  |  |  |  |  |
| 3.733333 | | | 72.31911 | | | | | | | | | | -1.03771 | | | | 5015.977 | | | 72.90625 | |  |  |  |  |  |
| 3.741667 | | | 72.39098 | | | | | | | | | | -1.03833 | | | | 5015.32 | | | 72.64844 | |  |  |  |  |  |
| 3.75 | | | 72.45864 | | | | | | | | | | -1.03887 | | | | 5014.711 | | | 72.54688 | |  |  |  |  |  |
| 3.758333 | | | 72.5284 | | | | | | | | | | -1.04039 | | | | 5014.125 | | | 72.53125 | |  |  |  |  |  |
| 3.766667 | | | 72.60135 | | | | | | | | | | -1.04191 | | | | 5013.539 | | | 72.39844 | |  |  |  |  |  |
| 3.775 | | | 72.66794 | | | | | | | | | | -1.04309 | | | | 5012.898 | | | 72.28125 | |  |  |  |  |  |
| 3.783333 | | | 72.73982 | | | | | | | | | | -1.04408 | | | | 5012.297 | | | 72.21094 | |  |  |  |  |  |
| 3.791667 | | | 72.80748 | | | | | | | | | | -1.04578 | | | | 5011.727 | | | 72.01563 | |  |  |  |  |  |
| 3.8 | | | 72.88042 | | | | | | | | | | -1.0468 | | | | 5011.133 | | | 72.14844 | |  |  |  |  |  |
| 3.808333 | | | 72.95124 | | | | | | | | | | -1.04692 | | | | 5010.539 | | | 72.07813 | |  |  |  |  |  |
| 3.816667 | | | 73.02101 | | | | | | | | | | -1.0486 | | | | 5009.953 | | | 72.10156 | |  |  |  |  |  |
| 3.825 | | | 73.09184 | | | | | | | | | | -1.04986 | | | | 5009.367 | | | 72.28906 | |  |  |  |  |  |
| 3.833333 | | | 73.16689 | | | | | | | | | | -1.05004 | | | | 5008.773 | | | 72.23438 | |  |  |  |  |  |
| 3.841667 | | | 73.23771 | | | | | | | | | | -1.05127 | | | | 5008.203 | | | 72.48438 | |  |  |  |  |  |
| 3.85 | | | 73.30748 | | | | | | | | | | -1.05227 | | | | 5007.578 | | | 72.78125 | |  |  |  |  |  |
| 3.858333 | | | 73.37513 | | | | | | | | | | -1.05302 | | | | 5006.992 | | | 72.88281 | |  |  |  |  |  |
| 3.866667 | | | 73.4449 | | | | | | | | | | -1.05391 | | | | 5006.375 | | | 72.92969 | |  |  |  |  |  |
| 3.875 | | | 73.51678 | | | | | | | | | | -1.05526 | | | | 5005.75 | | | 73.10156 | |  |  |  |  |  |
| 3.883333 | | | 73.58443 | | | | | | | | | | -1.05635 | | | | 5005.148 | | | 72.97656 | |  |  |  |  |  |
| 3.891667 | | | 73.65632 | | | | | | | | | | -1.05659 | | | | 5004.516 | | | 72.97656 | |  |  |  |  |  |
| 3.9 | | | 73.72608 | | | | | | | | | | -1.05812 | | | | 5003.867 | | | 72.79688 | |  |  |  |  |  |
| 3.908333 | | | 73.80008 | | | | | | | | | | -1.05871 | | | | 5003.242 | | | 72.5625 | |  |  |  |  |  |
| 3.916667 | | | 73.86879 | | | | | | | | | | -1.05939 | | | | 5002.617 | | | 72.72656 | |  |  |  |  |  |
| 3.925 | | | 73.94173 | | | | | | | | | | -1.06157 | | | | 5001.992 | | | 72.60938 | |  |  |  |  |  |
| 3.933333 | | | 74.0115 | | | | | | | | | | -1.06305 | | | | 5001.398 | | | 72.35156 | |  |  |  |  |  |
| 3.941667 | | | 74.08549 | | | | | | | | | | -1.06375 | | | | 5000.789 | | | 72.17188 | |  |  |  |  |  |
| 3.95 | | | 74.15843 | | | | | | | | | | -1.06519 | | | | 5000.211 | | | 72.13281 | |  |  |  |  |  |
| 3.958333 | | | 74.23243 | | | | | | | | | | -1.06613 | | | | 4999.633 | | | 72.19531 | |  |  |  |  |  |
| 3.966667 | | | 74.30325 | | | | | | | | | | -1.06665 | | | | 4999.016 | | | 72.36719 | |  |  |  |  |  |
| 3.975 | | | 74.37302 | | | | | | | | | | -1.06786 | | | | 4998.414 | | | 72.57031 | |  |  |  |  |  |
| 3.983333 | | | 74.44913 | | | | | | | | | | -1.0694 | | | | 4997.844 | | | 72.5625 | |  |  |  |  |  |
| 3.991667 | | | 74.51784 | | | | | | | | | | -1.07072 | | | | 4997.266 | | | 72.875 | |  |  |  |  |  |
| 4 | | | 74.59077 | | | | | | | | | | -1.0712 | | | | 4996.68 | | | 72.9375 | |  |  |  |  |  |
| 4.008333 | | | 74.66372 | | | | | | | | | | -1.07296 | | | | 4996.063 | | | 72.85156 | |  |  |  |  |  |
| 4.016667 | | | 74.73666 | | | | | | | | | | -1.07365 | | | | 4995.445 | | | 72.72656 | |  |  |  |  |  |
| 4.025 | | | 74.81593 | | | | | | | | | | -1.07348 | | | | 4994.805 | | | 72.84375 | |  |  |  |  |  |
| 4.033333 | | | 74.88464 | | | | | | | | | | -1.07492 | | | | 4994.203 | | | 72.78125 | |  |  |  |  |  |
| 4.041667 | | | 74.95759 | | | | | | | | | | -1.07523 | | | | 4993.555 | | | 72.82813 | |  |  |  |  |  |
| 4.05 | | | 75.0337 | | | | | | | | | | -1.07545 | | | | 4992.945 | | | 72.71875 | |  |  |  |  |  |
| 4.058333 | | | 75.1098 | | | | | | | | | | -1.07662 | | | | 4992.344 | | | 72.41406 | |  |  |  |  |  |
| 4.066667 | | | 75.18275 | | | | | | | | | | -1.07724 | | | | 4991.758 | | | 72.35156 | |  |  |  |  |  |
| 4.075 | | | 75.25674 | | | | | | | | | | -1.0771 | | | | 4991.133 | | | 72.07031 | |  |  |  |  |  |
| 4.083333 | | | 75.32862 | | | | | | | | | | -1.07808 | | | | 4990.539 | | | 72.32813 | |  |  |  |  |  |
| 4.091667 | | | 75.40051 | | | | | | | | | | -1.07904 | | | | 4989.914 | | | 72.32813 | |  |  |  |  |  |
| 4.1 | | | 75.47767 | | | | | | | | | | -1.0792 | | | | 4989.32 | | | 72.34375 | |  |  |  |  |  |
| 4.108333 | | | 75.54638 | | | | | | | | | | -1.0801 | | | | 4988.766 | | | 72.20313 | |  |  |  |  |  |
| 4.116667 | | | 75.62144 | | | | | | | | | | -1.08121 | | | | 4988.172 | | | 72.03906 | |  |  |  |  |  |
| 4.125 | | | 75.69437 | | | | | | | | | | -1.08181 | | | | 4987.602 | | | 71.91406 | |  |  |  |  |  |
| 4.133333 | | | 75.76414 | | | | | | | | | | -1.08272 | | | | 4986.969 | | | 71.99219 | |  |  |  |  |  |
| 4.141667 | | | 75.83497 | | | | | | | | | | -1.08441 | | | | 4986.375 | | | 72.21875 | |  |  |  |  |  |
| 4.15 | | | 75.90896 | | | | | | | | | | -1.08489 | | | | 4985.758 | | | 72.38281 | |  |  |  |  |  |
| 4.158333 | | | 75.9819 | | | | | | | | | | -1.0853 | | | | 4985.18 | | | 72.375 | |  |  |  |  |  |
| 4.166667 | | | 76.05907 | | | | | | | | | | -1.08631 | | | | 4984.602 | | | 72.16406 | |  |  |  |  |  |
| 4.175 | | | 76.12989 | | | | | | | | | | -1.0869 | | | | 4984.031 | | | 72.0625 | |  |  |  |  |  |
| 4.183333 | | | 76.20177 | | | | | | | | | | -1.08698 | | | | 4983.414 | | | 72.10156 | |  |  |  |  |  |
| 4.191667 | | | 76.27471 | | | | | | | | | | -1.08761 | | | | 4982.797 | | | 72.00781 | |  |  |  |  |  |
| 4.2 | | | 76.35082 | | | | | | | | | | -1.08776 | | | | 4982.164 | | | 72.21875 | |  |  |  |  |  |
| 4.208333 | | | 76.42693 | | | | | | | | | | -1.08753 | | | | 4981.555 | | | 72.375 | |  |  |  |  |  |
| 4.216667 | | | 76.5041 | | | | | | | | | | -1.08807 | | | | 4980.984 | | | 72.19531 | |  |  |  |  |  |
| 4.225 | | | 76.57809 | | | | | | | | | | -1.08882 | | | | 4980.398 | | | 72.07031 | |  |  |  |  |  |
| 4.233333 | | | 76.65103 | | | | | | | | | | -1.08919 | | | | 4979.797 | | | 71.72656 | |  |  |  |  |  |
| 4.241667 | | | 76.72926 | | | | | | | | | | -1.08951 | | | | 4979.195 | | | 71.60156 | |  |  |  |  |  |
| 4.25 | | | 76.8022 | | | | | | | | | | -1.09068 | | | | 4978.555 | | | 71.54688 | |  |  |  |  |  |
| 4.258333 | | | 76.88148 | | | | | | | | | | -1.09059 | | | | 4977.93 | | | 71.49219 | |  |  |  |  |  |
| 4.266667 | | | 76.95759 | | | | | | | | | | -1.09106 | | | | 4977.352 | | | 71.39063 | |  |  |  |  |  |
| 4.275 | | | 77.03581 | | | | | | | | | | -1.09202 | | | | 4976.766 | | | 71.33594 | |  |  |  |  |  |
| 4.283333 | | | 77.11192 | | | | | | | | | | -1.09209 | | | | 4976.219 | | | 70.96875 | |  |  |  |  |  |
| 4.291667 | | | 77.18697 | | | | | | | | | | -1.09251 | | | | 4975.633 | | | 70.71094 | |  |  |  |  |  |
| 4.3 | | | 77.26414 | | | | | | | | | | -1.09337 | | | | 4975.047 | | | 70.5 | |  |  |  |  |  |
| 4.308333 | | | 77.34131 | | | | | | | | | | -1.09311 | | | | 4974.469 | | | 70.32031 | |  |  |  |  |  |
| 4.316667 | | | 77.41531 | | | | | | | | | | -1.09326 | | | | 4973.883 | | | 70.23438 | |  |  |  |  |  |
| 4.325 | | | 77.49142 | | | | | | | | | | -1.09433 | | | | 4973.305 | | | 69.85156 | |  |  |  |  |  |
| 4.333333 | | | 77.56541 | | | | | | | | | | -1.09458 | | | | 4972.75 | | | 69.53906 | |  |  |  |  |  |
| 4.341667 | | | 77.63624 | | | | | | | | | | -1.09477 | | | | 4972.211 | | | 69.33594 | |  |  |  |  |  |
| 4.35 | | | 77.70811 | | | | | | | | | | -1.09541 | | | | 4971.648 | | | 69.07813 | |  |  |  |  |  |
| 4.358333 | | | 77.78211 | | | | | | | | | | -1.096 | | | | 4971.086 | | | 68.80469 | |  |  |  |  |  |
| 4.366667 | | | 77.854 | | | | | | | | | | -1.09613 | | | | 4970.508 | | | 68.73438 | |  |  |  |  |  |
| 4.375 | | | 77.92799 | | | | | | | | | | -1.09669 | | | | 4969.969 | | | 68.75781 | |  |  |  |  |  |
| 4.383333 | | | 78.00304 | | | | | | | | | | -1.09766 | | | | 4969.43 | | | 68.75 | |  |  |  |  |  |
| 4.391667 | | | 78.08233 | | | | | | | | | | -1.09732 | | | | 4968.883 | | | 69.03125 | |  |  |  |  |  |
| 4.4 | | | 78.15843 | | | | | | | | | | -1.09764 | | | | 4968.336 | | | 69.17969 | |  |  |  |  |  |
| 4.408333 | | | 78.23032 | | | | | | | | | | -1.09888 | | | | 4967.797 | | | 69.53125 | |  |  |  |  |  |
| 4.416667 | | | 78.31065 | | | | | | | | | | -1.09857 | | | | 4967.227 | | | 69.75781 | |  |  |  |  |  |
| 4.425 | | | 78.38888 | | | | | | | | | | -1.09913 | | | | 4966.656 | | | 70.07813 | |  |  |  |  |  |
| 4.433333 | | | 78.4671 | | | | | | | | | | -1.10015 | | | | 4966.078 | | | 70.13281 | |  |  |  |  |  |
| 4.441667 | | | 78.5411 | | | | | | | | | | -1.09971 | | | | 4965.453 | | | 70.57813 | |  |  |  |  |  |
| 4.45 | | | 78.62144 | | | | | | | | | | -1.09963 | | | | 4964.852 | | | 70.77344 | |  |  |  |  |  |
| 4.458333 | | | 78.69543 | | | | | | | | | | -1.10058 | | | | 4964.227 | | | 70.89063 | |  |  |  |  |  |
| 4.466667 | | | 78.76943 | | | | | | | | | | -1.1005 | | | | 4963.609 | | | 70.97656 | |  |  |  |  |  |
| 4.475 | | | 78.84026 | | | | | | | | | | -1.10084 | | | | 4962.984 | | | 70.77344 | |  |  |  |  |  |
| 4.483333 | | | 78.91108 | | | | | | | | | | -1.10194 | | | | 4962.383 | | | 70.90625 | |  |  |  |  |  |
| 4.491667 | | | 78.98824 | | | | | | | | | | -1.10232 | | | | 4961.727 | | | 71 | |  |  |  |  |  |
| 4.5 | | | 79.06224 | | | | | | | | | | -1.10177 | | | | 4961.109 | | | 70.9375 | |  |  |  |  |  |
| 4.508333 | | | 79.13835 | | | | | | | | | | -1.10202 | | | | 4960.5 | | | 70.84375 | |  |  |  |  |  |
| 4.516667 | | | 79.21235 | | | | | | | | | | -1.10223 | | | | 4959.898 | | | 70.97656 | |  |  |  |  |  |
| 4.525 | | | 79.28846 | | | | | | | | | | -1.10158 | | | | 4959.344 | | | 70.64844 | |  |  |  |  |  |
| 4.533333 | | | 79.36246 | | | | | | | | | | -1.10199 | | | | 4958.734 | | | 70.50781 | |  |  |  |  |  |
| 4.541667 | | | 79.43962 | | | | | | | | | | -1.10235 | | | | 4958.117 | | | 70.27344 | |  |  |  |  |  |
| 4.55 | | | 79.51467 | | | | | | | | | | -1.10164 | | | | 4957.547 | | | 70.15625 | |  |  |  |  |  |
| 4.558333 | | | 79.5929 | | | | | | | | | | -1.10162 | | | | 4956.953 | | | 69.97656 | |  |  |  |  |  |
| 4.566667 | | | 79.67535 | | | | | | | | | | -1.10179 | | | | 4956.352 | | | 69.85938 | |  |  |  |  |  |
| 4.575 | | | 79.75146 | | | | | | | | | | -1.10092 | | | | 4955.813 | | | 69.54688 | |  |  |  |  |  |
| 4.583333 | | | 79.82863 | | | | | | | | | | -1.10088 | | | | 4955.227 | | | 69.53906 | |  |  |  |  |  |
| 4.591667 | | | 79.90791 | | | | | | | | | | -1.10118 | | | | 4954.68 | | | 69.45313 | |  |  |  |  |  |
| 4.6 | | | 79.98613 | | | | | | | | | | -1.1008 | | | | 4954.109 | | | 69.3125 | |  |  |  |  |  |
| 4.608333 | | | 80.06541 | | | | | | | | | | -1.10071 | | | | 4953.539 | | | 69.41406 | |  |  |  |  |  |
| 4.616667 | | | 80.14046 | | | | | | | | | | -1.10132 | | | | 4952.977 | | | 69.16406 | |  |  |  |  |  |
| 4.625 | | | 80.21869 | | | | | | | | | | -1.10077 | | | | 4952.445 | | | 69.25781 | |  |  |  |  |  |
| 4.633333 | | | 80.29797 | | | | | | | | | | -1.1005 | | | | 4951.867 | | | 69.14844 | |  |  |  |  |  |
| 4.641667 | | | 80.37408 | | | | | | | | | | -1.10089 | | | | 4951.297 | | | 69.125 | |  |  |  |  |  |
| 4.65 | | | 80.45125 | | | | | | | | | | -1.10037 | | | | 4950.742 | | | 69.16406 | |  |  |  |  |  |
| 4.658333 | | | 80.52947 | | | | | | | | | | -1.10007 | | | | 4950.156 | | | 69.21875 | |  |  |  |  |  |
| 4.666667 | | | 80.60558 | | | | | | | | | | -1.10044 | | | | 4949.609 | | | 69.21875 | |  |  |  |  |  |
| 4.675 | | | 80.68063 | | | | | | | | | | -1.10024 | | | | 4949.016 | | | 69.11719 | |  |  |  |  |  |
| 4.683333 | | | 80.75886 | | | | | | | | | | -1.0997 | | | | 4948.453 | | | 69.15625 | |  |  |  |  |  |
| 4.691667 | | | 80.83497 | | | | | | | | | | -1.10019 | | | | 4947.891 | | | 69.01563 | |  |  |  |  |  |
| 4.7 | | | 80.91425 | | | | | | | | | | -1.10017 | | | | 4947.305 | | | 68.83594 | |  |  |  |  |  |
| 4.708333 | | | 80.99142 | | | | | | | | | | -1.09956 | | | | 4946.727 | | | 68.53906 | |  |  |  |  |  |
| 4.716667 | | | 81.06647 | | | | | | | | | | -1.09995 | | | | 4946.148 | | | 68.30469 | |  |  |  |  |  |
| 4.725 | | | 81.1447 | | | | | | | | | | -1.09998 | | | | 4945.594 | | | 68.24219 | |  |  |  |  |  |
| 4.733333 | | | 81.22292 | | | | | | | | | | -1.09897 | | | | 4945.016 | | | 68.09375 | |  |  |  |  |  |
| 4.741667 | | | 81.29692 | | | | | | | | | | -1.09938 | | | | 4944.461 | | | 67.89063 | |  |  |  |  |  |
| 4.75 | | | 81.37408 | | | | | | | | | | -1.09947 | | | | 4943.914 | | | 67.78125 | |  |  |  |  |  |
| 4.758333 | | | 81.45337 | | | | | | | | | | -1.09821 | | | | 4943.391 | | | 67.64844 | |  |  |  |  |  |
| 4.766667 | | | 81.53265 | | | | | | | | | | -1.09815 | | | | 4942.852 | | | 67.65625 | |  |  |  |  |  |
| 4.775 | | | 81.60876 | | | | | | | | | | -1.09812 | | | | 4942.297 | | | 67.32813 | |  |  |  |  |  |
| 4.783333 | | | 81.68804 | | | | | | | | | | -1.09653 | | | | 4941.75 | | | 67.47656 | |  |  |  |  |  |
| 4.791667 | | | 81.77155 | | | | | | | | | | -1.09614 | | | | 4941.203 | | | 67.67188 | |  |  |  |  |  |
| 4.8 | | | 81.85188 | | | | | | | | | | -1.09644 | | | | 4940.656 | | | 67.60938 | |  |  |  |  |  |
| 4.808333 | | | 81.92876 | | | | | | | | | | -1.09553 | | | | 4940.102 | | | 67.53125 | |  |  |  |  |  |
| 4.816667 | | | 82.00606 | | | | | | | | | | -1.09563 | | | | 4939.531 | | | 67.28125 | |  |  |  |  |  |
| 4.825 | | | 82.08129 | | | | | | | | | | -1.09578 | | | | 4939 | | | 67.39844 | |  |  |  |  |  |
| 4.833333 | | | 82.15755 | | | | | | | | | | -1.09492 | | | | 4938.422 | | | 66.96875 | |  |  |  |  |  |
| 4.841667 | | | 82.23485 | | | | | | | | | | -1.09497 | | | | 4937.82 | | | 66.78906 | |  |  |  |  |  |
| 4.85 | | | 82.30802 | | | | | | | | | | -1.09544 | | | | 4937.25 | | | 66.83594 | |  |  |  |  |  |
| 4.858333 | | | 82.38841 | | | | | | | | | | -1.09483 | | | | 4936.695 | | | 66.97656 | |  |  |  |  |  |
| 4.866667 | | | 82.46468 | | | | | | | | | | -1.09473 | | | | 4936.164 | | | 66.82813 | |  |  |  |  |  |
| 4.875 | | | 82.53579 | | | | | | | | | | -1.09506 | | | | 4935.594 | | | 66.92969 | |  |  |  |  |  |
| 4.883333 | | | 82.61411 | | | | | | | | | | -1.0941 | | | | 4935.086 | | | 66.92969 | |  |  |  |  |  |
| 4.891667 | | | 82.69038 | | | | | | | | | | -1.09359 | | | | 4934.547 | | | 66.94531 | |  |  |  |  |  |
| 4.9 | | | 82.76562 | | | | | | | | | | -1.09401 | | | | 4933.984 | | | 67.17188 | |  |  |  |  |  |
| 4.908333 | | | 82.84909 | | | | | | | | | | -1.09311 | | | | 4933.398 | | | 66.96094 | |  |  |  |  |  |
| 4.916667 | | | 82.92742 | | | | | | | | | | -1.09245 | | | | 4932.859 | | | 67.17969 | |  |  |  |  |  |
| 4.925 | | | 83.00574 | | | | | | | | | | -1.0922 | | | | 4932.281 | | | 67.21094 | |  |  |  |  |  |
| 4.933333 | | | 83.08614 | | | | | | | | | | -1.09098 | | | | 4931.727 | | | 67.25781 | |  |  |  |  |  |
| 4.941667 | | | 83.16446 | | | | | | | | | | -1.08997 | | | | 4931.164 | | | 67.17969 | |  |  |  |  |  |
| 4.95 | | | 83.24278 | | | | | | | | | | -1.09 | | | | 4930.578 | | | 67.28125 | |  |  |  |  |  |
| 4.958333 | | | 83.32214 | | | | | | | | | | -1.08963 | | | | 4930.039 | | | 66.99219 | |  |  |  |  |  |
| 4.966667 | | | 83.39841 | | | | | | | | | | -1.0889 | | | | 4929.453 | | | 66.83594 | |  |  |  |  |  |
| 4.975 | | | 83.47776 | | | | | | | | | | -1.08882 | | | | 4928.883 | | | 66.92969 | |  |  |  |  |  |
| 4.983333 | | | 83.55506 | | | | | | | | | | -1.08788 | | | | 4928.32 | | | 66.78125 | |  |  |  |  |  |
| 4.991667 | | | 83.63235 | | | | | | | | | | -1.08695 | | | | 4927.773 | | | 66.82031 | |  |  |  |  |  |
| 5 | | | 83.71068 | | | | | | | | | | -1.08702 | | | | 4927.195 | | | 66.57813 | |  |  |  |  |  |
| 5.008333 | | | 83.78694 | | | | | | | | | | -1.08683 | | | | 4926.68 | | | 66.36719 | |  |  |  |  |  |
| 5.016667 | | | 83.8663 | | | | | | | | | | -1.08596 | | | | 4926.133 | | | 65.96875 | |  |  |  |  |  |
| 5.025 | | | 83.94566 | | | | | | | | | | -1.08608 | | | | 4925.57 | | | 65.92969 | |  |  |  |  |  |
| 5.033333 | | | 84.02296 | | | | | | | | | | -1.08579 | | | | 4925.031 | | | 65.73438 | |  |  |  |  |  |
| 5.041667 | | | 84.10128 | | | | | | | | | | -1.08429 | | | | 4924.469 | | | 65.49219 | |  |  |  |  |  |
| 5.05 | | | 84.1827 | | | | | | | | | | -1.08464 | | | | 4923.945 | | | 65.40625 | |  |  |  |  |  |
| 5.058333 | | | 84.25896 | | | | | | | | | | -1.0846 | | | | 4923.414 | | | 65.01563 | |  |  |  |  |  |
| 5.066667 | | | 84.33729 | | | | | | | | | | -1.0838 | | | | 4922.922 | | | 64.9375 | |  |  |  |  |  |
| 5.075 | | | 84.41252 | | | | | | | | | | -1.08437 | | | | 4922.375 | | | 64.78906 | |  |  |  |  |  |
| 5.083333 | | | 84.48981 | | | | | | | | | | -1.08402 | | | | 4921.859 | | | 64.80469 | |  |  |  |  |  |
| 5.091667 | | | 84.57021 | | | | | | | | | | -1.08255 | | | | 4921.336 | | | 64.88281 | |  |  |  |  |  |
| 5.1 | | | 84.64647 | | | | | | | | | | -1.08241 | | | | 4920.805 | | | 64.875 | |  |  |  |  |  |
| 5.108333 | | | 84.7217 | | | | | | | | | | -1.08208 | | | | 4920.32 | | | 64.78906 | |  |  |  |  |  |
| 5.116667 | | | 84.80209 | | | | | | | | | | -1.08077 | | | | 4919.789 | | | 64.67969 | |  |  |  |  |  |
| 5.125 | | | 84.88042 | | | | | | | | | | -1.08046 | | | | 4919.273 | | | 64.72656 | |  |  |  |  |  |
| 5.133333 | | | 84.95978 | | | | | | | | | | -1.07972 | | | | 4918.727 | | | 64.79688 | |  |  |  |  |  |
| 5.141667 | | | 85.03913 | | | | | | | | | | -1.07815 | | | | 4918.172 | | | 64.72656 | |  |  |  |  |  |
| 5.15 | | | 85.12055 | | | | | | | | | | -1.07745 | | | | 4917.633 | | | 64.75781 | |  |  |  |  |  |
| 5.158333 | | | 85.20197 | | | | | | | | | | -1.07647 | | | | 4917.102 | | | 64.79688 | |  |  |  |  |  |
| 5.166667 | | | 85.28236 | | | | | | | | | | -1.075 | | | | 4916.578 | | | 64.75 | |  |  |  |  |  |
| 5.175 | | | 85.36171 | | | | | | | | | | -1.07477 | | | | 4916.031 | | | 64.75781 | |  |  |  |  |  |
| 5.183333 | | | 85.44004 | | | | | | | | | | -1.07439 | | | | 4915.477 | | | 64.92969 | |  |  |  |  |  |
| 5.191667 | | | 85.51836 | | | | | | | | | | -1.07309 | | | | 4914.938 | | | 64.78906 | |  |  |  |  |  |
| 5.2 | | | 85.59566 | | | | | | | | | | -1.07255 | | | | 4914.398 | | | 64.64063 | |  |  |  |  |  |
| 5.208333 | | | 85.67399 | | | | | | | | | | -1.07204 | | | | 4913.852 | | | 64.60938 | |  |  |  |  |  |
| 5.216667 | | | 85.75128 | | | | | | | | | | -1.07107 | | | | 4913.313 | | | 64.60156 | |  |  |  |  |  |
| 5.225 | | | 85.83064 | | | | | | | | | | -1.07049 | | | | 4912.773 | | | 64.75781 | |  |  |  |  |  |
| 5.233333 | | | 85.90691 | | | | | | | | | | -1.07024 | | | | 4912.203 | | | 64.52344 | |  |  |  |  |  |
| 5.241667 | | | 85.98626 | | | | | | | | | | -1.0691 | | | | 4911.688 | | | 64.28125 | |  |  |  |  |  |
| 5.25 | | | 86.06561 | | | | | | | | | | -1.06814 | | | | 4911.156 | | | 64.14063 | |  |  |  |  |  |
| 5.258333 | | | 86.14394 | | | | | | | | | | -1.06782 | | | | 4910.625 | | | 63.96094 | |  |  |  |  |  |
| 5.266667 | | | 86.22433 | | | | | | | | | | -1.06648 | | | | 4910.086 | | | 63.66406 | |  |  |  |  |  |
| 5.275 | | | 86.30472 | | | | | | | | | | -1.0654 | | | | 4909.523 | | | 63.8125 | |  |  |  |  |  |
| 5.283333 | | | 86.38613 | | | | | | | | | | -1.06535 | | | | 4909.008 | | | 63.8125 | |  |  |  |  |  |
| 5.291667 | | | 86.46755 | | | | | | | | | | -1.06412 | | | | 4908.508 | | | 63.72656 | |  |  |  |  |  |
| 5.3 | | | 86.54485 | | | | | | | | | | -1.06326 | | | | 4907.992 | | | 63.375 | |  |  |  |  |  |
| 5.308333 | | | 86.62524 | | | | | | | | | | -1.06312 | | | | 4907.484 | | | 63.05469 | |  |  |  |  |  |
| 5.316667 | | | 86.70459 | | | | | | | | | | -1.06189 | | | | 4906.984 | | | 62.96875 | |  |  |  |  |  |
| 5.325 | | | 86.78395 | | | | | | | | | | -1.06074 | | | | 4906.43 | | | 63.07031 | |  |  |  |  |  |
| 5.333333 | | | 86.86433 | | | | | | | | | | -1.06069 | | | | 4905.891 | | | 62.92188 | |  |  |  |  |  |
| 5.341667 | | | 86.9437 | | | | | | | | | | -1.05939 | | | | 4905.375 | | | 62.85156 | |  |  |  |  |  |
| 5.35 | | | 87.02099 | | | | | | | | | | -1.05814 | | | | 4904.891 | | | 62.8125 | |  |  |  |  |  |
| 5.358333 | | | 87.10241 | | | | | | | | | | -1.05811 | | | | 4904.406 | | | 62.57813 | |  |  |  |  |  |
| 5.366667 | | | 87.1797 | | | | | | | | | | -1.05716 | | | | 4903.891 | | | 62.39844 | |  |  |  |  |  |
| 5.375 | | | 87.25803 | | | | | | | | | | -1.05552 | | | | 4903.352 | | | 62.14063 | |  |  |  |  |  |
| 5.383333 | | | 87.33739 | | | | | | | | | | -1.05529 | | | | 4902.844 | | | 62.07813 | |  |  |  |  |  |
| 5.391667 | | | 87.4219 | | | | | | | | | | -1.0535 | | | | 4902.328 | | | 62.25781 | |  |  |  |  |  |
| 5.4 | | | 87.50537 | | | | | | | | | | -1.05205 | | | | 4901.805 | | | 62.26563 | |  |  |  |  |  |
| 5.408333 | | | 87.5837 | | | | | | | | | | -1.05192 | | | | 4901.313 | | | 62.19531 | |  |  |  |  |  |
| 5.416667 | | | 87.661 | | | | | | | | | | -1.05036 | | | | 4900.828 | | | 62.26563 | |  |  |  |  |  |
| 5.425 | | | 87.73932 | | | | | | | | | | -1.04906 | | | | 4900.344 | | | 62.42188 | |  |  |  |  |  |
| 5.433333 | | | 87.82177 | | | | | | | | | | -1.04912 | | | | 4899.836 | | | 62.32031 | |  |  |  |  |  |
| 5.441667 | | | 87.90319 | | | | | | | | | | -1.0477 | | | | 4899.281 | | | 62.1875 | |  |  |  |  |  |
| 5.45 | | | 87.9846 | | | | | | | | | | -1.04612 | | | | 4898.766 | | | 62.30469 | |  |  |  |  |  |
| 5.458333 | | | 88.06087 | | | | | | | | | | -1.04642 | | | | 4898.258 | | | 62.27344 | |  |  |  |  |  |
| 5.466667 | | | 88.14229 | | | | | | | | | | -1.045 | | | | 4897.727 | | | 62.22656 | |  |  |  |  |  |
| 5.475 | | | 88.21958 | | | | | | | | | | -1.04372 | | | | 4897.195 | | | 61.92969 | |  |  |  |  |  |
| 5.483333 | | | 88.29997 | | | | | | | | | | -1.0433 | | | | 4896.688 | | | 61.82031 | |  |  |  |  |  |
| 5.491667 | | | 88.38139 | | | | | | | | | | -1.0417 | | | | 4896.18 | | | 62.02344 | |  |  |  |  |  |
| 5.5 | | | 88.46487 | | | | | | | | | | -1.04041 | | | | 4895.641 | | | 61.88281 | |  |  |  |  |  |
| 5.508333 | | | 88.54835 | | | | | | | | | | -1.04024 | | | | 4895.117 | | | 61.65625 | |  |  |  |  |  |
| 5.516667 | | | 88.6277 | | | | | | | | | | -1.03879 | | | | 4894.609 | | | 61.78906 | |  |  |  |  |  |
| 5.525 | | | 88.705 | | | | | | | | | | -1.03742 | | | | 4894.133 | | | 61.50781 | |  |  |  |  |  |
| 5.533333 | | | 88.78539 | | | | | | | | | | -1.037 | | | | 4893.625 | | | 61.39844 | |  |  |  |  |  |
| 5.541667 | | | 88.87093 | | | | | | | | | | -1.03526 | | | | 4893.078 | | | 61.32031 | |  |  |  |  |  |
| 5.55 | | | 88.95132 | | | | | | | | | | -1.03424 | | | | 4892.586 | | | 61.3125 | |  |  |  |  |  |
| 5.558333 | | | 89.03067 | | | | | | | | | | -1.03426 | | | | 4892.086 | | | 61.17188 | |  |  |  |  |  |
| 5.566667 | | | 89.10899 | | | | | | | | | | -1.0332 | | | | 4891.555 | | | 60.85938 | |  |  |  |  |  |
| 5.575 | | | 89.19144 | | | | | | | | | | -1.03206 | | | | 4891.078 | | | 60.69531 | |  |  |  |  |  |
| 5.583333 | | | 89.26978 | | | | | | | | | | -1.03178 | | | | 4890.586 | | | 60.59375 | |  |  |  |  |  |
| 5.591667 | | | 89.3481 | | | | | | | | | | -1.03053 | | | | 4890.078 | | | 60.64063 | |  |  |  |  |  |
| 5.6 | | | 89.42951 | | | | | | | | | | -1.02905 | | | | 4889.563 | | | 60.32813 | |  |  |  |  |  |
| 5.608333 | | | 89.5099 | | | | | | | | | | -1.0285 | | | | 4889.078 | | | 60.33594 | |  |  |  |  |  |
| 5.616667 | | | 89.58926 | | | | | | | | | | -1.02739 | | | | 4888.602 | | | 60.32031 | |  |  |  |  |  |
| 5.625 | | | 89.66862 | | | | | | | | | | -1.02626 | | | | 4888.125 | | | 60.29688 | |  |  |  |  |  |
| 5.633333 | | | 89.75004 | | | | | | | | | | -1.02563 | | | | 4887.625 | | | 60.20313 | |  |  |  |  |  |
| 5.641667 | | | 89.83042 | | | | | | | | | | -1.02442 | | | | 4887.117 | | | 60.25 | |  |  |  |  |  |
| 5.65 | | | 89.91493 | | | | | | | | | | -1.02264 | | | | 4886.648 | | | 60.375 | |  |  |  |  |  |
| 5.658333 | | | 89.99738 | | | | | | | | | | -1.02192 | | | | 4886.148 | | | 60.07813 | |  |  |  |  |  |
| 5.666667 | | | 90.07983 | | | | | | | | | | -1.02079 | | | | 4885.656 | | | 60.03906 | |  |  |  |  |  |
| 5.675 | | | 90.16331 | | | | | | | | | | -1.01927 | | | | 4885.141 | | | 59.85938 | |  |  |  |  |  |
| 5.683333 | | | 90.24576 | | | | | | | | | | -1.01848 | | | | 4884.648 | | | 59.78906 | |  |  |  |  |  |
| 5.691667 | | | 90.3282 | | | | | | | | | | -1.01727 | | | | 4884.141 | | | 59.95313 | |  |  |  |  |  |
| 5.7 | | | 90.40962 | | | | | | | | | | -1.01573 | | | | 4883.625 | | | 59.89844 | |  |  |  |  |  |
| 5.708333 | | | 90.49001 | | | | | | | | | | -1.01474 | | | | 4883.164 | | | 59.60938 | |  |  |  |  |  |
| 5.716667 | | | 90.57246 | | | | | | | | | | -1.01328 | | | | 4882.664 | | | 59.53125 | |  |  |  |  |  |
| 5.725 | | | 90.65697 | | | | | | | | | | -1.01141 | | | | 4882.195 | | | 59.48438 | |  |  |  |  |  |
| 5.733333 | | | 90.73736 | | | | | | | | | | -1.01084 | | | | 4881.695 | | | 59.46094 | |  |  |  |  |  |
| 5.741667 | | | 90.81774 | | | | | | | | | | -1.00995 | | | | 4881.18 | | | 59.71094 | |  |  |  |  |  |
| 5.75 | | | 90.90019 | | | | | | | | | | -1.00808 | | | | 4880.688 | | | 59.51563 | |  |  |  |  |  |
| 5.758333 | | | 90.98058 | | | | | | | | | | -1.00713 | | | | 4880.219 | | | 59.38281 | |  |  |  |  |  |
| 5.766667 | | | 91.06303 | | | | | | | | | | -1.00605 | | | | 4879.75 | | | 59.28906 | |  |  |  |  |  |
| 5.775 | | | 91.14651 | | | | | | | | | | -1.00387 | | | | 4879.25 | | | 59.21094 | |  |  |  |  |  |
| 5.783333 | | | 91.22792 | | | | | | | | | | -1.00293 | | | | 4878.766 | | | 59.25781 | |  |  |  |  |  |
| 5.791667 | | | 91.3145 | | | | | | | | | | -1.00188 | | | | 4878.219 | | | 59.17969 | |  |  |  |  |  |
| 5.8 | | | 91.39797 | | | | | | | | | | -1.00042 | | | | 4877.75 | | | 59.25781 | |  |  |  |  |  |
| 5.808333 | | | 91.4763 | | | | | | | | | | -0.99973 | | | | 4877.273 | | | 59.03125 | |  |  |  |  |  |
| 5.816667 | | | 91.55875 | | | | | | | | | | -0.99855 | | | | 4876.789 | | | 58.875 | |  |  |  |  |  |
| 5.825 | | | 91.63811 | | | | | | | | | | -0.99714 | | | | 4876.297 | | | 58.53906 | |  |  |  |  |  |
| 5.833333 | | | 91.72262 | | | | | | | | | | -0.99641 | | | | 4875.789 | | | 58.59375 | |  |  |  |  |  |
| 5.841667 | | | 91.803 | | | | | | | | | | -0.99575 | | | | 4875.305 | | | 58.58594 | |  |  |  |  |  |
| 5.85 | | | 91.88442 | | | | | | | | | | -0.99404 | | | | 4874.805 | | | 58.5 | |  |  |  |  |  |
| 5.858333 | | | 91.96275 | | | | | | | | | | -0.99368 | | | | 4874.336 | | | 58.32813 | |  |  |  |  |  |
| 5.866667 | | | 92.04623 | | | | | | | | | | -0.99333 | | | | 4873.867 | | | 58.26563 | |  |  |  |  |  |
| 5.875 | | | 92.12352 | | | | | | | | | | -0.99163 | | | | 4873.43 | | | 58.25 | |  |  |  |  |  |
| 5.883333 | | | 92.20391 | | | | | | | | | | -0.9905 | | | | 4872.93 | | | 58.11719 | |  |  |  |  |  |
| 5.891667 | | | 92.28635 | | | | | | | | | | -0.98952 | | | | 4872.445 | | | 58.23438 | |  |  |  |  |  |
| 5.9 | | | 92.36674 | | | | | | | | | | -0.98725 | | | | 4871.961 | | | 58.32813 | |  |  |  |  |  |
| 5.908333 | | | 92.45538 | | | | | | | | | | -0.98539 | | | | 4871.492 | | | 58.28906 | |  |  |  |  |  |
| 5.916667 | | | 92.53577 | | | | | | | | | | -0.98422 | | | | 4871.008 | | | 58.35156 | |  |  |  |  |  |
| 5.925 | | | 92.61821 | | | | | | | | | | -0.98217 | | | | 4870.531 | | | 58.33594 | |  |  |  |  |  |
| 5.933333 | | | 92.69963 | | | | | | | | | | -0.98139 | | | | 4870.055 | | | 57.9375 | |  |  |  |  |  |
| 5.941667 | | | 92.78723 | | | | | | | | | | -0.97977 | | | | 4869.563 | | | 57.78125 | |  |  |  |  |  |
| 5.95 | | | 92.8738 | | | | | | | | | | -0.97718 | | | | 4869.063 | | | 57.51563 | |  |  |  |  |  |
| 5.958333 | | | 92.96037 | | | | | | | | | | -0.97594 | | | | 4868.578 | | | 57.23438 | |  |  |  |  |  |
| 5.966667 | | | 93.04282 | | | | | | | | | | -0.97473 | | | | 4868.078 | | | 57.10938 | |  |  |  |  |  |
| 5.975 | | | 93.12939 | | | | | | | | | | -0.97292 | | | | 4867.602 | | | 56.92969 | |  |  |  |  |  |
| 5.983333 | | | 93.21027 | | | | | | | | | | -0.97229 | | | | 4867.172 | | | 56.88281 | |  |  |  |  |  |
| 5.991667 | | | 93.28691 | | | | | | | | | | -0.9713 | | | | 4866.703 | | | 56.66406 | |  |  |  |  |  |
| 6 | | | 93.36654 | | | | | | | | | | -0.96925 | | | | 4866.266 | | | 56.35938 | |  |  |  |  |  |
| 6.008333 | | | 93.44815 | | | | | | | | | | -0.96831 | | | | 4865.828 | | | 56.16406 | |  |  |  |  |  |
| 6.016667 | | | 93.53176 | | | | | | | | | | -0.96676 | | | | 4865.367 | | | 56.19531 | |  |  |  |  |  |
| 6.025 | | | 93.60939 | | | | | | | | | | -0.96497 | | | | 4864.914 | | | 55.99219 | |  |  |  |  |  |
| 6.033333 | | | 93.69002 | | | | | | | | | | -0.96421 | | | | 4864.445 | | | 55.84375 | |  |  |  |  |  |
| 6.041667 | | | 93.76865 | | | | | | | | | | -0.96326 | | | | 4863.992 | | | 55.91406 | |  |  |  |  |  |
| 6.05 | | | 93.84927 | | | | | | | | | | -0.96107 | | | | 4863.57 | | | 55.69531 | |  |  |  |  |  |
| 6.058333 | | | 93.9269 | | | | | | | | | | -0.96006 | | | | 4863.125 | | | 55.39063 | |  |  |  |  |  |
| 6.066667 | | | 94.00652 | | | | | | | | | | -0.95853 | | | | 4862.648 | | | 55.14844 | |  |  |  |  |  |
| 6.075 | | | 94.08516 | | | | | | | | | | -0.95613 | | | | 4862.211 | | | 55.02344 | |  |  |  |  |  |
| 6.083333 | | | 94.16677 | | | | | | | | | | -0.95482 | | | | 4861.766 | | | 55.15625 | |  |  |  |  |  |
| 6.091667 | | | 94.24739 | | | | | | | | | | -0.95312 | | | | 4861.289 | | | 55.04688 | |  |  |  |  |  |
| 6.1 | | | 94.32503 | | | | | | | | | | -0.95052 | | | | 4860.852 | | | 55.01563 | |  |  |  |  |  |
| 6.108333 | | | 94.40466 | | | | | | | | | | -0.94949 | | | | 4860.422 | | | 54.98438 | |  |  |  |  |  |
| 6.116667 | | | 94.4793 | | | | | | | | | | -0.94855 | | | | 4860 | | | 54.94531 | |  |  |  |  |  |
| 6.125 | | | 94.55694 | | | | | | | | | | -0.94626 | | | | 4859.555 | | | 54.88281 | |  |  |  |  |  |
| 6.133333 | | | 94.63457 | | | | | | | | | | -0.94545 | | | | 4859.07 | | | 54.89063 | |  |  |  |  |  |
| 6.141667 | | | 94.7132 | | | | | | | | | | -0.94436 | | | | 4858.617 | | | 54.97656 | |  |  |  |  |  |
| 6.15 | | | 94.79681 | | | | | | | | | | -0.94176 | | | | 4858.164 | | | 54.82031 | |  |  |  |  |  |
| 6.158333 | | | 94.87843 | | | | | | | | | | -0.94098 | | | | 4857.711 | | | 54.89844 | |  |  |  |  |  |
| 6.166667 | | | 94.95805 | | | | | | | | | | -0.93994 | | | | 4857.25 | | | 54.80469 | |  |  |  |  |  |
| 6.175 | | | 95.03867 | | | | | | | | | | -0.93746 | | | | 4856.797 | | | 54.77344 | |  |  |  |  |  |
| 6.183333 | | | 95.12029 | | | | | | | | | | -0.93685 | | | | 4856.336 | | | 54.78906 | |  |  |  |  |  |
| 6.191667 | | | 95.2009 | | | | | | | | | | -0.93585 | | | | 4855.859 | | | 54.71094 | |  |  |  |  |  |
| 6.2 | | | 95.28452 | | | | | | | | | | -0.93326 | | | | 4855.414 | | | 54.65625 | |  |  |  |  |  |
| 6.208333 | | | 95.36713 | | | | | | | | | | -0.93202 | | | | 4854.953 | | | 54.6875 | |  |  |  |  |  |
| 6.216667 | | | 95.45073 | | | | | | | | | | -0.93057 | | | | 4854.508 | | | 54.45313 | |  |  |  |  |  |
| 6.225 | | | 95.53434 | | | | | | | | | | -0.92791 | | | | 4854.047 | | | 54.32813 | |  |  |  |  |  |
| 6.233333 | | | 95.61297 | | | | | | | | | | -0.92719 | | | | 4853.586 | | | 54.60938 | |  |  |  |  |  |
| 6.241667 | | | 95.69061 | | | | | | | | | | -0.92588 | | | | 4853.141 | | | 54.44531 | |  |  |  |  |  |
| 6.25 | | | 95.77222 | | | | | | | | | | -0.92354 | | | | 4852.695 | | | 54.36719 | |  |  |  |  |  |
| 6.258333 | | | 95.85284 | | | | | | | | | | -0.9228 | | | | 4852.227 | | | 54.17188 | |  |  |  |  |  |
| 6.266667 | | | 95.93147 | | | | | | | | | | -0.92176 | | | | 4851.813 | | | 54.0625 | |  |  |  |  |  |
| 6.275 | | | 96.01408 | | | | | | | | | | -0.91929 | | | | 4851.367 | | | 54.13281 | |  |  |  |  |  |
| 6.283333 | | | 96.09669 | | | | | | | | | | -0.91819 | | | | 4850.875 | | | 54.375 | |  |  |  |  |  |
| 6.291667 | | | 96.17931 | | | | | | | | | | -0.9167 | | | | 4850.43 | | | 54.35938 | |  |  |  |  |  |
| 6.3 | | | 96.2659 | | | | | | | | | | -0.91405 | | | | 4849.992 | | | 54.51563 | |  |  |  |  |  |
| 6.308333 | | | 96.3495 | | | | | | | | | | -0.91344 | | | | 4849.555 | | | 54.47656 | |  |  |  |  |  |
| 6.316667 | | | 96.43311 | | | | | | | | | | -0.91203 | | | | 4849.117 | | | 54.25 | |  |  |  |  |  |
| 6.325 | | | 96.51971 | | | | | | | | | | -0.90975 | | | | 4848.656 | | | 54.19531 | |  |  |  |  |  |
| 6.333333 | | | 96.60132 | | | | | | | | | | -0.90916 | | | | 4848.172 | | | 54.10938 | |  |  |  |  |  |
| 6.341667 | | | 96.68095 | | | | | | | | | | -0.90777 | | | | 4847.711 | | | 54.03125 | |  |  |  |  |  |
| 6.35 | | | 96.76555 | | | | | | | | | | -0.90495 | | | | 4847.242 | | | 54.02344 | |  |  |  |  |  |
| 6.358333 | | | 96.84816 | | | | | | | | | | -0.9042 | | | | 4846.789 | | | 53.78906 | |  |  |  |  |  |
| 6.366667 | | | 96.92878 | | | | | | | | | | -0.90277 | | | | 4846.375 | | | 53.53906 | |  |  |  |  |  |
| 6.375 | | | 97.01238 | | | | | | | | | | -0.90028 | | | | 4845.93 | | | 53.52344 | |  |  |  |  |  |
| 6.383333 | | | 97.09102 | | | | | | | | | | -0.89984 | | | | 4845.492 | | | 53.52344 | |  |  |  |  |  |
| 6.391667 | | | 97.17064 | | | | | | | | | | -0.89837 | | | | 4845.047 | | | 53.21875 | |  |  |  |  |  |
| 6.4 | | | 97.25325 | | | | | | | | | | -0.89611 | | | | 4844.602 | | | 53.16406 | |  |  |  |  |  |
| 6.408333 | | | 97.33586 | | | | | | | | | | -0.89502 | | | | 4844.18 | | | 53.38281 | |  |  |  |  |  |
| 6.416667 | | | 97.41649 | | | | | | | | | | -0.89343 | | | | 4843.781 | | | 53.125 | |  |  |  |  |  |
| 6.425 | | | 97.50307 | | | | | | | | | | -0.89066 | | | | 4843.328 | | | 53.04688 | |  |  |  |  |  |
| 6.433333 | | | 97.58469 | | | | | | | | | | -0.88985 | | | | 4842.891 | | | 53.08594 | |  |  |  |  |  |
| 6.441667 | | | 97.66432 | | | | | | | | | | -0.88812 | | | | 4842.477 | | | 53.02344 | |  |  |  |  |  |
| 6.45 | | | 97.74693 | | | | | | | | | | -0.88525 | | | | 4842.047 | | | 53.11719 | |  |  |  |  |  |
| 6.458333 | | | 97.82954 | | | | | | | | | | -0.88439 | | | | 4841.578 | | | 53.08594 | |  |  |  |  |  |
| 6.466667 | | | 97.91216 | | | | | | | | | | -0.88223 | | | | 4841.172 | | | 52.95313 | |  |  |  |  |  |
| 6.475 | | | 97.99477 | | | | | | | | | | -0.8794 | | | | 4840.742 | | | 52.92969 | |  |  |  |  |  |
| 6.483333 | | | 98.07539 | | | | | | | | | | -0.87836 | | | | 4840.289 | | | 52.72656 | |  |  |  |  |  |
| 6.491667 | | | 98.15401 | | | | | | | | | | -0.87667 | | | | 4839.844 | | | 52.47656 | |  |  |  |  |  |
| 6.5 | | | 98.23663 | | | | | | | | | | -0.87405 | | | | 4839.391 | | | 52.53125 | |  |  |  |  |  |
| 6.508333 | | | 98.31924 | | | | | | | | | | -0.87334 | | | | 4838.953 | | | 52.5625 | |  |  |  |  |  |
| 6.516667 | | | 98.40185 | | | | | | | | | | -0.87154 | | | | 4838.523 | | | 52.1875 | |  |  |  |  |  |
| 6.525 | | | 98.48546 | | | | | | | | | | -0.86935 | | | | 4838.086 | | | 52.04688 | |  |  |  |  |  |
| 6.533333 | | | 98.56807 | | | | | | | | | | -0.86907 | | | | 4837.672 | | | 52.00781 | |  |  |  |  |  |
| 6.541667 | | | 98.65267 | | | | | | | | | | -0.86689 | | | | 4837.266 | | | 51.51563 | |  |  |  |  |  |
| 6.55 | | | 98.73428 | | | | | | | | | | -0.86466 | | | | 4836.82 | | | 51.35938 | |  |  |  |  |  |
| 6.558333 | | | 98.81789 | | | | | | | | | | -0.86406 | | | | 4836.375 | | | 51.21094 | |  |  |  |  |  |
| 6.566667 | | | 98.90051 | | | | | | | | | | -0.86214 | | | | 4835.992 | | | 51.4375 | |  |  |  |  |  |
| 6.575 | | | 98.9871 | | | | | | | | | | -0.85982 | | | | 4835.578 | | | 51.1875 | |  |  |  |  |  |
| 6.583333 | | | 99.0717 | | | | | | | | | | -0.85895 | | | | 4835.148 | | | 51.03125 | |  |  |  |  |  |
| 6.591667 | | | 99.1553 | | | | | | | | | | -0.85678 | | | | 4834.781 | | | 50.65625 | |  |  |  |  |  |
| 6.6 | | | 99.23891 | | | | | | | | | | -0.85471 | | | | 4834.375 | | | 50.48438 | |  |  |  |  |  |
| 6.608333 | | | 99.31953 | | | | | | | | | | -0.85401 | | | | 4833.961 | | | 50.3125 | |  |  |  |  |  |
| 6.616667 | | | 99.40015 | | | | | | | | | | -0.85187 | | | | 4833.508 | | | 49.86719 | |  |  |  |  |  |
| 6.625 | | | 99.48177 | | | | | | | | | | -0.85023 | | | | 4833.109 | | | 49.89063 | |  |  |  |  |  |
| 6.633333 | | | 99.56339 | | | | | | | | | | -0.85 | | | | 4832.695 | | | 49.84375 | |  |  |  |  |  |
| 6.641667 | | | 99.645 | | | | | | | | | | -0.84804 | | | | 4832.328 | | | 49.53125 | |  |  |  |  |  |
| 6.65 | | | 99.72961 | | | | | | | | | | -0.84592 | | | | 4831.938 | | | 49.15625 | |  |  |  |  |  |
| 6.658333 | | | 99.80923 | | | | | | | | | | -0.84554 | | | | 4831.539 | | | 49.11719 | |  |  |  |  |  |
| 6.666667 | | | 99.89184 | | | | | | | | | | -0.84349 | | | | 4831.18 | | | 49.02344 | |  |  |  |  |  |
| 6.675 | | | 99.96947 | | | | | | | | | | -0.84158 | | | | 4830.75 | | | 49.01563 | |  |  |  |  |  |
| 6.683333 | | | 100.0511 | | | | | | | | | | -0.84117 | | | | 4830.344 | | | 49.13281 | |  |  |  |  |  |
| 6.691667 | | | 100.1337 | | | | | | | | | | -0.83891 | | | | 4829.969 | | | 49.09375 | |  |  |  |  |  |
| 6.7 | | | 100.2173 | | | | | | | | | | -0.83679 | | | | 4829.602 | | | 49.0625 | |  |  |  |  |  |
| 6.708333 | | | 100.2969 | | | | | | | | | | -0.83581 | | | | 4829.195 | | | 48.84375 | |  |  |  |  |  |
| 6.716667 | | | 100.3825 | | | | | | | | | | -0.83296 | | | | 4828.789 | | | 48.65625 | |  |  |  |  |  |
| 6.725 | | | 100.4671 | | | | | | | | | | -0.83077 | | | | 4828.383 | | | 48.86719 | |  |  |  |  |  |
| 6.733333 | | | 100.5507 | | | | | | | | | | -0.82983 | | | | 4827.961 | | | 48.625 | |  |  |  |  |  |
| 6.741667 | | | 100.6363 | | | | | | | | | | -0.82745 | | | | 4827.539 | | | 48.42188 | |  |  |  |  |  |
| 6.75 | | | 100.718 | | | | | | | | | | -0.82546 | | | | 4827.133 | | | 48.22656 | |  |  |  |  |  |
| 6.758333 | | | 100.8046 | | | | | | | | | | -0.82443 | | | | 4826.766 | | | 48.07813 | |  |  |  |  |  |
| 6.766667 | | | 100.8892 | | | | | | | | | | -0.82177 | | | | 4826.383 | | | 48.07813 | |  |  |  |  |  |
| 6.775 | | | 100.9738 | | | | | | | | | | -0.81997 | | | | 4825.938 | | | 47.89063 | |  |  |  |  |  |
| 6.783333 | | | 101.0554 | | | | | | | | | | -0.8192 | | | | 4825.563 | | | 47.83594 | |  |  |  |  |  |
| 6.791667 | | | 101.14 | | | | | | | | | | -0.8168 | | | | 4825.188 | | | 47.99219 | |  |  |  |  |  |
| 6.8 | | | 101.2236 | | | | | | | | | | -0.81514 | | | | 4824.805 | | | 47.95313 | |  |  |  |  |  |
| 6.808333 | | | 101.3072 | | | | | | | | | | -0.81419 | | | | 4824.422 | | | 47.65625 | |  |  |  |  |  |
| 6.816667 | | | 101.3868 | | | | | | | | | | -0.81231 | | | | 4824.016 | | | 47.75 | |  |  |  |  |  |
| 6.825 | | | 101.4654 | | | | | | | | | | -0.8109 | | | | 4823.648 | | | 47.78125 | |  |  |  |  |  |
| 6.833333 | | | 101.55 | | | | | | | | | | -0.81 | | | | 4823.25 | | | 47.74219 | |  |  |  |  |  |
| 6.841667 | | | 101.6287 | | | | | | | | | | -0.80782 | | | | 4822.82 | | | 47.8125 | |  |  |  |  |  |
| 6.85 | | | 101.7103 | | | | | | | | | | -0.80574 | | | | 4822.422 | | | 47.72656 | |  |  |  |  |  |
| 6.858333 | | | 101.7949 | | | | | | | | | | -0.80435 | | | | 4822.055 | | | 47.55469 | |  |  |  |  |  |
| 6.866667 | | | 101.8805 | | | | | | | | | | -0.80139 | | | | 4821.641 | | | 47.61719 | |  |  |  |  |  |
| 6.875 | | | 101.9621 | | | | | | | | | | -0.79988 | | | | 4821.234 | | | 47.67188 | |  |  |  |  |  |
| 6.883333 | | | 102.0427 | | | | | | | | | | -0.79905 | | | | 4820.844 | | | 47.64844 | |  |  |  |  |  |
| 6.891667 | | | 102.1233 | | | | | | | | | | -0.79638 | | | | 4820.422 | | | 47.75 | |  |  |  |  |  |
| 6.9 | | | 102.2069 | | | | | | | | | | -0.79419 | | | | 4820.039 | | | 47.63281 | |  |  |  |  |  |
| 6.908333 | | | 102.2935 | | | | | | | | | | -0.793 | | | | 4819.664 | | | 47.65625 | |  |  |  |  |  |
| 6.916667 | | | 102.3762 | | | | | | | | | | -0.79039 | | | | 4819.25 | | | 47.55469 | |  |  |  |  |  |
| 6.925 | | | 102.4627 | | | | | | | | | | -0.78861 | | | | 4818.852 | | | 47.42969 | |  |  |  |  |  |
| 6.933333 | | | 102.5483 | | | | | | | | | | -0.78789 | | | | 4818.445 | | | 47.35156 | |  |  |  |  |  |
| 6.941667 | | | 102.63 | | | | | | | | | | -0.78548 | | | | 4818.039 | | | 47.47656 | |  |  |  |  |  |
| 6.95 | | | 102.7126 | | | | | | | | | | -0.78385 | | | | 4817.656 | | | 47.21094 | |  |  |  |  |  |
| 6.958333 | | | 102.7972 | | | | | | | | | | -0.78254 | | | | 4817.258 | | | 47 | |  |  |  |  |  |
| 6.966667 | | | 102.8818 | | | | | | | | | | -0.78005 | | | | 4816.875 | | | 46.76563 | |  |  |  |  |  |
| 6.975 | | | 102.9644 | | | | | | | | | | -0.77851 | | | | 4816.492 | | | 46.57813 | |  |  |  |  |  |
| 6.983333 | | | 103.05 | | | | | | | | | | -0.77777 | | | | 4816.102 | | | 46.55469 | |  |  |  |  |  |
| 6.991667 | | | 103.1326 | | | | | | | | | | -0.77504 | | | | 4815.695 | | | 46.4375 | |  |  |  |  |  |
| 7 | | | 103.2172 | | | | | | | | | | -0.77317 | | | | 4815.336 | | | 46.25 | |  |  |  |  |  |
| 7.008333 | | | 103.3008 | | | | | | | | | | -0.77209 | | | | 4814.977 | | | 46.03125 | |  |  |  |  |  |
| 7.016667 | | | 103.3844 | | | | | | | | | | -0.76921 | | | | 4814.625 | | | 46.08594 | |  |  |  |  |  |
| 7.025 | | | 103.472 | | | | | | | | | | -0.76757 | | | | 4814.25 | | | 45.89844 | |  |  |  |  |  |
| 7.033333 | | | 103.5566 | | | | | | | | | | -0.76667 | | | | 4813.867 | | | 45.71094 | |  |  |  |  |  |
| 7.041667 | | | 103.6402 | | | | | | | | | | -0.76423 | | | | 4813.492 | | | 45.55469 | |  |  |  |  |  |
| 7.05 | | | 103.7238 | | | | | | | | | | -0.76268 | | | | 4813.148 | | | 45.57031 | |  |  |  |  |  |
| 7.058333 | | | 103.8104 | | | | | | | | | | -0.76145 | | | | 4812.789 | | | 45.60156 | |  |  |  |  |  |
| 7.066667 | | | 103.895 | | | | | | | | | | -0.75837 | | | | 4812.391 | | | 45.32031 | |  |  |  |  |  |
| 7.075 | | | 103.9806 | | | | | | | | | | -0.75663 | | | | 4812.031 | | | 45.02344 | |  |  |  |  |  |
| 7.083333 | | | 104.0642 | | | | | | | | | | -0.75539 | | | | 4811.672 | | | 44.82031 | |  |  |  |  |  |
| 7.091667 | | | 104.1518 | | | | | | | | | | -0.75255 | | | | 4811.305 | | | 44.60156 | |  |  |  |  |  |
| 7.1 | | | 104.2314 | | | | | | | | | | -0.75127 | | | | 4810.93 | | | 44.24219 | |  |  |  |  |  |
| 7.108333 | | | 104.313 | | | | | | | | | | -0.75009 | | | | 4810.547 | | | 44.36719 | |  |  |  |  |  |
| 7.116667 | | | 104.3965 | | | | | | | | | | -0.74771 | | | | 4810.203 | | | 44.38281 | |  |  |  |  |  |
| 7.125 | | | 104.4811 | | | | | | | | | | -0.74615 | | | | 4809.867 | | | 44.32031 | |  |  |  |  |  |
| 7.133333 | | | 104.5685 | | | | | | | | | | -0.74433 | | | | 4809.516 | | | 44.15625 | |  |  |  |  |  |
| 7.141667 | | | 104.6511 | | | | | | | | | | -0.74182 | | | | 4809.18 | | | 44.01563 | |  |  |  |  |  |
| 7.15 | | | 104.7366 | | | | | | | | | | -0.74028 | | | | 4808.859 | | | 44.14844 | |  |  |  |  |  |
| 7.158333 | | | 104.8202 | | | | | | | | | | -0.73874 | | | | 4808.469 | | | 44.3125 | |  |  |  |  |  |
| 7.166667 | | | 104.9027 | | | | | | | | | | -0.73622 | | | | 4808.102 | | | 44.57813 | |  |  |  |  |  |
| 7.175 | | | 104.9813 | | | | | | | | | | -0.73501 | | | | 4807.734 | | | 44.72656 | |  |  |  |  |  |
| 7.183333 | | | 105.0678 | | | | | | | | | | -0.73346 | | | | 4807.391 | | | 44.78125 | |  |  |  |  |  |
| 7.191667 | | | 105.1534 | | | | | | | | | | -0.73078 | | | | 4807.031 | | | 44.5 | |  |  |  |  |  |
| 7.2 | | | 105.2369 | | | | | | | | | | -0.72926 | | | | 4806.641 | | | 44.35938 | |  |  |  |  |  |
| 7.208333 | | | 105.3195 | | | | | | | | | | -0.72761 | | | | 4806.25 | | | 44.32031 | |  |  |  |  |  |
| 7.216667 | | | 105.3991 | | | | | | | | | | -0.72513 | | | | 4805.828 | | | 44.53906 | |  |  |  |  |  |
| 7.225 | | | 105.4826 | | | | | | | | | | -0.72367 | | | | 4805.43 | | | 44.35156 | |  |  |  |  |  |
| 7.233333 | | | 105.5612 | | | | | | | | | | -0.72238 | | | | 4805.047 | | | 44.03125 | |  |  |  |  |  |
| 7.241667 | | | 105.6418 | | | | | | | | | | -0.71981 | | | | 4804.719 | | | 43.89063 | |  |  |  |  |  |
| 7.25 | | | 105.7303 | | | | | | | | | | -0.71837 | | | | 4804.359 | | | 43.67969 | |  |  |  |  |  |
| 7.258333 | | | 105.8207 | | | | | | | | | | -0.71646 | | | | 4803.992 | | | 43.42969 | |  |  |  |  |  |
| 7.266667 | | | 105.9023 | | | | | | | | | | -0.71455 | | | | 4803.586 | | | 43.17969 | |  |  |  |  |  |
| 7.275 | | | 105.9829 | | | | | | | | | | -0.71343 | | | | 4803.25 | | | 43.19531 | |  |  |  |  |  |
| 7.283333 | | | 106.0674 | | | | | | | | | | -0.7116 | | | | 4802.922 | | | 42.96094 | |  |  |  |  |  |
| 7.291667 | | | 106.151 | | | | | | | | | | -0.70897 | | | | 4802.57 | | | 42.99219 | |  |  |  |  |  |
| 7.3 | | | 106.2355 | | | | | | | | | | -0.70756 | | | | 4802.227 | | | 42.5625 | |  |  |  |  |  |
| 7.308333 | | | 106.32 | | | | | | | | | | -0.70573 | | | | 4801.898 | | | 42.61719 | |  |  |  |  |  |
| 7.316667 | | | 106.4065 | | | | | | | | | | -0.70315 | | | | 4801.57 | | | 42.5625 | |  |  |  |  |  |
| 7.325 | | | 106.495 | | | | | | | | | | -0.70183 | | | | 4801.211 | | | 42.41406 | |  |  |  |  |  |
| 7.333333 | | | 106.5756 | | | | | | | | | | -0.70015 | | | | 4800.875 | | | 42.27344 | |  |  |  |  |  |
| 7.341667 | | | 106.6542 | | | | | | | | | | -0.69771 | | | | 4800.508 | | | 42.33594 | |  |  |  |  |  |
| 7.35 | | | 106.7417 | | | | | | | | | | -0.6958 | | | | 4800.211 | | | 42.29688 | |  |  |  |  |  |
| 7.358333 | | | 106.8291 | | | | | | | | | | -0.69314 | | | | 4799.852 | | | 42.08594 | |  |  |  |  |  |
| 7.366667 | | | 106.9137 | | | | | | | | | | -0.69052 | | | | 4799.508 | | | 41.92969 | |  |  |  |  |  |
| 7.375 | | | 106.9992 | | | | | | | | | | -0.68903 | | | | 4799.172 | | | 41.71094 | |  |  |  |  |  |
| 7.383333 | | | 107.0807 | | | | | | | | | | -0.68687 | | | | 4798.828 | | | 41.85938 | |  |  |  |  |  |
| 7.391667 | | | 107.1663 | | | | | | | | | | -0.68442 | | | | 4798.469 | | | 41.80469 | |  |  |  |  |  |
| 7.4 | | | 107.2528 | | | | | | | | | | -0.68297 | | | | 4798.125 | | | 41.98438 | |  |  |  |  |  |
| 7.408333 | | | 107.3334 | | | | | | | | | | -0.68092 | | | | 4797.805 | | | 41.96875 | |  |  |  |  |  |
| 7.416667 | | | 107.4198 | | | | | | | | | | -0.67844 | | | | 4797.469 | | | 41.95313 | |  |  |  |  |  |
| 7.425 | | | 107.5073 | | | | | | | | | | -0.67674 | | | | 4797.148 | | | 41.77344 | |  |  |  |  |  |
| 7.433333 | | | 107.5899 | | | | | | | | | | -0.67433 | | | | 4796.773 | | | 41.74219 | |  |  |  |  |  |
| 7.441667 | | | 107.6715 | | | | | | | | | | -0.67242 | | | | 4796.438 | | | 41.73438 | |  |  |  |  |  |
| 7.45 | | | 107.755 | | | | | | | | | | -0.67096 | | | | 4796.055 | | | 41.90625 | |  |  |  |  |  |
| 7.458333 | | | 107.8385 | | | | | | | | | | -0.66851 | | | | 4795.711 | | | 41.875 | |  |  |  |  |  |
| 7.466667 | | | 107.925 | | | | | | | | | | -0.66624 | | | | 4795.359 | | | 41.8125 | |  |  |  |  |  |
| 7.475 | | | 108.0056 | | | | | | | | | | -0.66486 | | | | 4795.039 | | | 41.80469 | |  |  |  |  |  |
| 7.483333 | | | 108.0852 | | | | | | | | | | -0.6626 | | | | 4794.695 | | | 41.625 | |  |  |  |  |  |
| 7.491667 | | | 108.1727 | | | | | | | | | | -0.66 | | | | 4794.344 | | | 41.77344 | |  |  |  |  |  |
| 7.5 | | | 108.2543 | | | | | | | | | | -0.65857 | | | | 4793.977 | | | 41.66406 | |  |  |  |  |  |
| 7.508333 | | | 108.3378 | | | | | | | | | | -0.65624 | | | | 4793.633 | | | 41.84375 | |  |  |  |  |  |
| 7.516667 | | | 108.4243 | | | | | | | | | | -0.6542 | | | | 4793.289 | | | 41.9375 | |  |  |  |  |  |
| 7.525 | | | 108.5108 | | | | | | | | | | -0.65271 | | | | 4792.938 | | | 41.90625 | |  |  |  |  |  |
| 7.533333 | | | 108.5983 | | | | | | | | | | -0.65031 | | | | 4792.609 | | | 41.73438 | |  |  |  |  |  |
| 7.541667 | | | 108.6858 | | | | | | | | | | -0.64848 | | | | 4792.25 | | | 41.74219 | |  |  |  |  |  |
| 7.55 | | | 108.7693 | | | | | | | | | | -0.64732 | | | | 4791.906 | | | 41.39844 | |  |  |  |  |  |
| 7.558333 | | | 108.8578 | | | | | | | | | | -0.64479 | | | | 4791.523 | | | 41.57031 | |  |  |  |  |  |
| 7.566667 | | | 108.9462 | | | | | | | | | | -0.64302 | | | | 4791.156 | | | 41.28125 | |  |  |  |  |  |
| 7.575 | | | 109.0327 | | | | | | | | | | -0.64139 | | | | 4790.805 | | | 41.125 | |  |  |  |  |  |
| 7.583333 | | | 109.1192 | | | | | | | | | | -0.63863 | | | | 4790.484 | | | 41.08594 | |  |  |  |  |  |
| 7.591667 | | | 109.2047 | | | | | | | | | | -0.63677 | | | | 4790.133 | | | 40.79688 | |  |  |  |  |  |
| 7.6 | | | 109.2893 | | | | | | | | | | -0.63504 | | | | 4789.828 | | | 40.53125 | |  |  |  |  |  |
| 7.608333 | | | 109.3797 | | | | | | | | | | -0.63195 | | | | 4789.453 | | | 40.51563 | |  |  |  |  |  |
| 7.616667 | | | 109.4622 | | | | | | | | | | -0.63025 | | | | 4789.148 | | | 40.55469 | |  |  |  |  |  |
| 7.625 | | | 109.5428 | | | | | | | | | | -0.62885 | | | | 4788.828 | | | 40.39844 | |  |  |  |  |  |
| 7.633333 | | | 109.6274 | | | | | | | | | | -0.62645 | | | | 4788.492 | | | 40.49219 | |  |  |  |  |  |
| 7.641667 | | | 109.7099 | | | | | | | | | | -0.62466 | | | | 4788.195 | | | 40.25781 | |  |  |  |  |  |
| 7.65 | | | 109.7944 | | | | | | | | | | -0.62314 | | | | 4787.891 | | | 40.39063 | |  |  |  |  |  |
| 7.658333 | | | 109.879 | | | | | | | | | | -0.62053 | | | | 4787.555 | | | 40.42969 | |  |  |  |  |  |
| 7.666667 | | | 109.9665 | | | | | | | | | | -0.61825 | | | | 4787.219 | | | 40.25 | |  |  |  |  |  |
| 7.675 | | | 110.05 | | | | | | | | | | -0.6167 | | | | 4786.891 | | | 40.125 | |  |  |  |  |  |
| 7.683333 | | | 110.1355 | | | | | | | | | | -0.61415 | | | | 4786.547 | | | 40.09375 | |  |  |  |  |  |
| 7.691667 | | | 110.2161 | | | | | | | | | | -0.61237 | | | | 4786.234 | | | 39.97656 | |  |  |  |  |  |
| 7.7 | | | 110.3006 | | | | | | | | | | -0.61096 | | | | 4785.883 | | | 39.88281 | |  |  |  |  |  |
| 7.708333 | | | 110.3901 | | | | | | | | | | -0.60841 | | | | 4785.539 | | | 39.85938 | |  |  |  |  |  |
| 7.716667 | | | 110.4775 | | | | | | | | | | -0.6065 | | | | 4785.234 | | | 39.77344 | |  |  |  |  |  |
| 7.725 | | | 110.5621 | | | | | | | | | | -0.60492 | | | | 4784.914 | | | 39.35156 | |  |  |  |  |  |
| 7.733333 | | | 110.6456 | | | | | | | | | | -0.60241 | | | | 4784.586 | | | 39.10156 | |  |  |  |  |  |
| 7.741667 | | | 110.7282 | | | | | | | | | | -0.60121 | | | | 4784.266 | | | 39.05469 | |  |  |  |  |  |
| 7.75 | | | 110.8107 | | | | | | | | | | -0.60015 | | | | 4783.945 | | | 39.03906 | |  |  |  |  |  |
| 7.758333 | | | 110.8982 | | | | | | | | | | -0.59797 | | | | 4783.609 | | | 39.01563 | |  |  |  |  |  |
| 7.766667 | | | 110.9808 | | | | | | | | | | -0.59677 | | | | 4783.305 | | | 38.92188 | |  |  |  |  |  |
| 7.775 | | | 111.0633 | | | | | | | | | | -0.5956 | | | | 4783.031 | | | 38.875 | |  |  |  |  |  |
| 7.783333 | | | 111.1508 | | | | | | | | | | -0.59331 | | | | 4782.727 | | | 38.8125 | |  |  |  |  |  |
| 7.791667 | | | 111.2353 | | | | | | | | | | -0.59167 | | | | 4782.414 | | | 38.70313 | |  |  |  |  |  |
| 7.8 | | | 111.3238 | | | | | | | | | | -0.5901 | | | | 4782.086 | | | 38.73438 | |  |  |  |  |  |
| 7.808333 | | | 111.4123 | | | | | | | | | | -0.58731 | | | | 4781.758 | | | 38.78906 | |  |  |  |  |  |
| 7.816667 | | | 111.5007 | | | | | | | | | | -0.5854 | | | | 4781.438 | | | 38.67188 | |  |  |  |  |  |
| 7.825 | | | 111.5872 | | | | | | | | | | -0.58335 | | | | 4781.133 | | | 38.48438 | |  |  |  |  |  |
| 7.833333 | | | 111.6727 | | | | | | | | | | -0.58043 | | | | 4780.805 | | | 38.53125 | |  |  |  |  |  |
| 7.841667 | | | 111.7582 | | | | | | | | | | -0.57893 | | | | 4780.5 | | | 38.47656 | |  |  |  |  |  |
| 7.85 | | | 111.8428 | | | | | | | | | | -0.57742 | | | | 4780.172 | | | 38.3125 | |  |  |  |  |  |
| 7.858333 | | | 111.9322 | | | | | | | | | | -0.57467 | | | | 4779.836 | | | 38.07031 | |  |  |  |  |  |
| 7.866667 | | | 112.0206 | | | | | | | | | | -0.57307 | | | | 4779.523 | | | 37.88281 | |  |  |  |  |  |
| 7.875 | | | 112.1101 | | | | | | | | | | -0.57073 | | | | 4779.227 | | | 37.57031 | |  |  |  |  |  |
| 7.883333 | | | 112.1966 | | | | | | | | | | -0.56777 | | | | 4778.898 | | | 37.22656 | |  |  |  |  |  |
| 7.891667 | | | 112.2821 | | | | | | | | | | -0.5666 | | | | 4778.57 | | | 36.97656 | |  |  |  |  |  |
| 7.9 | | | 112.3696 | | | | | | | | | | -0.56448 | | | | 4778.281 | | | 36.82813 | |  |  |  |  |  |
| 7.908333 | | | 112.458 | | | | | | | | | | -0.56201 | | | | 4777.984 | | | 36.67188 | |  |  |  |  |  |
| 7.916667 | | | 112.5435 | | | | | | | | | | -0.56087 | | | | 4777.703 | | | 36.38281 | |  |  |  |  |  |
| 7.925 | | | 112.6281 | | | | | | | | | | -0.5585 | | | | 4777.422 | | | 36.14063 | |  |  |  |  |  |
| 7.933333 | | | 112.7165 | | | | | | | | | | -0.55543 | | | | 4777.164 | | | 35.89844 | |  |  |  |  |  |
| 7.941667 | | | 112.805 | | | | | | | | | | -0.55396 | | | | 4776.883 | | | 35.78125 | |  |  |  |  |  |
| 7.95 | | | 112.8895 | | | | | | | | | | -0.55123 | | | | 4776.594 | | | 35.86719 | |  |  |  |  |  |
| 7.958333 | | | 112.973 | | | | | | | | | | -0.54847 | | | | 4776.305 | | | 35.61719 | |  |  |  |  |  |
| 7.966667 | | | 113.0586 | | | | | | | | | | -0.54737 | | | | 4776.047 | | | 35.76563 | |  |  |  |  |  |
| 7.975 | | | 113.1451 | | | | | | | | | | -0.54488 | | | | 4775.773 | | | 35.70313 | |  |  |  |  |  |
| 7.983333 | | | 113.2315 | | | | | | | | | | -0.54239 | | | | 4775.5 | | | 35.44531 | |  |  |  |  |  |
| 7.991667 | | | 113.3161 | | | | | | | | | | -0.5417 | | | | 4775.219 | | | 35.45313 | |  |  |  |  |  |
| 8 | | | 113.4045 | | | | | | | | | | -0.53928 | | | | 4774.914 | | | 35.49219 | |  |  |  |  |  |
| 8.008333 | | | 113.4891 | | | | | | | | | | -0.53687 | | | | 4774.641 | | | 35.49219 | |  |  |  |  |  |
| 8.016667 | | | 113.5765 | | | | | | | | | | -0.5357 | | | | 4774.328 | | | 35.71875 | |  |  |  |  |  |
| 8.025 | | | 113.6611 | | | | | | | | | | -0.53325 | | | | 4774.023 | | | 35.92969 | |  |  |  |  |  |
| 8.033333 | | | 113.7466 | | | | | | | | | | -0.53122 | | | | 4773.766 | | | 35.77344 | |  |  |  |  |  |
| 8.041667 | | | 113.8321 | | | | | | | | | | -0.53071 | | | | 4773.461 | | | 35.8125 | |  |  |  |  |  |
| 8.05 | | | 113.9166 | | | | | | | | | | -0.52823 | | | | 4773.156 | | | 35.84375 | |  |  |  |  |  |
| 8.058333 | | | 114.0031 | | | | | | | | | | -0.52611 | | | | 4772.852 | | | 35.69531 | |  |  |  |  |  |
| 8.066667 | | | 114.0857 | | | | | | | | | | -0.5254 | | | | 4772.523 | | | 35.92188 | |  |  |  |  |  |
| 8.075 | | | 114.1702 | | | | | | | | | | -0.52295 | | | | 4772.188 | | | 35.91406 | |  |  |  |  |  |
| 8.083333 | | | 114.2567 | | | | | | | | | | -0.52071 | | | | 4771.914 | | | 35.71875 | |  |  |  |  |  |
| 8.091667 | | | 114.3461 | | | | | | | | | | -0.51989 | | | | 4771.602 | | | 35.59375 | |  |  |  |  |  |
| 8.1 | | | 114.4346 | | | | | | | | | | -0.51754 | | | | 4771.297 | | | 35.46875 | |  |  |  |  |  |
| 8.108333 | | | 114.5171 | | | | | | | | | | -0.51588 | | | | 4771.016 | | | 35.30469 | |  |  |  |  |  |
| 8.116667 | | | 114.6026 | | | | | | | | | | -0.51543 | | | | 4770.68 | | | 35.52344 | |  |  |  |  |  |
| 8.125 | | | 114.6931 | | | | | | | | | | -0.51255 | | | | 4770.391 | | | 35.4375 | |  |  |  |  |  |
| 8.133333 | | | 114.7805 | | | | | | | | | | -0.51052 | | | | 4770.117 | | | 35.54688 | |  |  |  |  |  |
| 8.141667 | | | 114.8671 | | | | | | | | | | -0.50933 | | | | 4769.836 | | | 35.50781 | |  |  |  |  |  |
| 8.15 | | | 114.9524 | | | | | | | | | | -0.50623 | | | | 4769.555 | | | 35.38281 | |  |  |  |  |  |
| 8.158333 | | | 115.0396 | | | | | | | | | | -0.50383 | | | | 4769.273 | | | 35.42969 | |  |  |  |  |  |
| 8.166667 | | | 115.124 | | | | | | | | | | -0.50262 | | | | 4768.953 | | | 35.30469 | |  |  |  |  |  |
| 8.175 | | | 115.2065 | | | | | | | | | | -0.49993 | | | | 4768.664 | | | 35.25 | |  |  |  |  |  |
| 8.183333 | | | 115.2852 | | | | | | | | | | -0.49807 | | | | 4768.352 | | | 35.22656 | |  |  |  |  |  |
| 8.191667 | | | 115.3696 | | | | | | | | | | -0.49679 | | | | 4768.063 | | | 35.07031 | |  |  |  |  |  |
| 8.2 | | | 115.454 | | | | | | | | | | -0.49343 | | | | 4767.773 | | | 35.19531 | |  |  |  |  |  |
| 8.208333 | | | 115.5364 | | | | | | | | | | -0.4912 | | | | 4767.469 | | | 34.97656 | |  |  |  |  |  |
| 8.216667 | | | 115.6218 | | | | | | | | | | -0.48974 | | | | 4767.188 | | | 34.74219 | |  |  |  |  |  |
| 8.225 | | | 115.7043 | | | | | | | | | | -0.48704 | | | | 4766.898 | | | 34.69531 | |  |  |  |  |  |
| 8.233333 | | | 115.7868 | | | | | | | | | | -0.4851 | | | | 4766.609 | | | 34.39063 | |  |  |  |  |  |
| 8.241667 | | | 115.8683 | | | | | | | | | | -0.4839 | | | | 4766.336 | | | 34.29688 | |  |  |  |  |  |
| 8.25 | | | 115.9517 | | | | | | | | | | -0.48018 | | | | 4766.023 | | | 34.22656 | |  |  |  |  |  |
| 8.258333 | | | 116.0361 | | | | | | | | | | -0.47752 | | | | 4765.766 | | | 34.14844 | |  |  |  |  |  |
| 8.266667 | | | 116.1214 | | | | | | | | | | -0.47586 | | | | 4765.508 | | | 33.83594 | |  |  |  |  |  |
| 8.275 | | | 116.2058 | | | | | | | | | | -0.47217 | | | | 4765.227 | | | 33.72656 | |  |  |  |  |  |
| 8.283333 | | | 116.2911 | | | | | | | | | | -0.46988 | | | | 4764.992 | | | 33.35156 | |  |  |  |  |  |
| 8.291667 | | | 116.3765 | | | | | | | | | | -0.46828 | | | | 4764.711 | | | 33.28906 | |  |  |  |  |  |
| 8.3 | | | 116.4552 | | | | | | | | | | -0.46549 | | | | 4764.438 | | | 33.375 | |  |  |  |  |  |
| 8.308333 | | | 116.5396 | | | | | | | | | | -0.46358 | | | | 4764.172 | | | 33.27344 | |  |  |  |  |  |
| 8.316667 | | | 116.6268 | | | | | | | | | | -0.46208 | | | | 4763.922 | | | 33.32031 | |  |  |  |  |  |
| 8.325 | | | 116.7093 | | | | | | | | | | -0.45929 | | | | 4763.664 | | | 33.03906 | |  |  |  |  |  |
| 8.333333 | | | 116.7918 | | | | | | | | | | -0.45764 | | | | 4763.438 | | | 32.89844 | |  |  |  |  |  |
| 8.341667 | | | 116.8752 | | | | | | | | | | -0.45625 | | | | 4763.172 | | | 32.97656 | |  |  |  |  |  |
| 8.35 | | | 116.9586 | | | | | | | | | | -0.45349 | | | | 4762.867 | | | 32.83594 | |  |  |  |  |  |
| 8.358333 | | | 117.0421 | | | | | | | | | | -0.45228 | | | | 4762.617 | | | 32.8125 | |  |  |  |  |  |
| 8.366667 | | | 117.1264 | | | | | | | | | | -0.45119 | | | | 4762.32 | | | 32.71875 | |  |  |  |  |  |
| 8.375 | | | 117.2089 | | | | | | | | | | -0.44857 | | | | 4762.094 | | | 32.72656 | |  |  |  |  |  |
| 8.383333 | | | 117.299 | | | | | | | | | | -0.44678 | | | | 4761.836 | | | 32.60938 | |  |  |  |  |  |
| 8.391667 | | | 117.3843 | | | | | | | | | | -0.44527 | | | | 4761.555 | | | 32.80469 | |  |  |  |  |  |
| 8.4 | | | 117.4668 | | | | | | | | | | -0.44269 | | | | 4761.281 | | | 32.92188 | |  |  |  |  |  |
| 8.408333 | | | 117.554 | | | | | | | | | | -0.44086 | | | | 4761.016 | | | 33.10938 | |  |  |  |  |  |
| 8.416667 | | | 117.6375 | | | | | | | | | | -0.43914 | | | | 4760.75 | | | 33.16406 | |  |  |  |  |  |
| 8.425 | | | 117.7238 | | | | | | | | | | -0.43656 | | | | 4760.461 | | | 33.13281 | |  |  |  |  |  |
| 8.433333 | | | 117.811 | | | | | | | | | | -0.43516 | | | | 4760.203 | | | 32.96094 | |  |  |  |  |  |
| 8.441667 | | | 117.8973 | | | | | | | | | | -0.43321 | | | | 4759.906 | | | 33.03125 | |  |  |  |  |  |
| 8.45 | | | 117.9807 | | | | | | | | | | -0.43079 | | | | 4759.602 | | | 32.92188 | |  |  |  |  |  |
| 8.458333 | | | 118.0632 | | | | | | | | | | -0.4293 | | | | 4759.305 | | | 33.01563 | |  |  |  |  |  |
| 8.466667 | | | 118.1457 | | | | | | | | | | -0.42713 | | | | 4759.016 | | | 32.74219 | |  |  |  |  |  |
| 8.475 | | | 118.23 | | | | | | | | | | -0.42401 | | | | 4758.742 | | | 32.625 | |  |  |  |  |  |
| 8.483333 | | | 118.3125 | | | | | | | | | | -0.42213 | | | | 4758.492 | | | 32.25781 | |  |  |  |  |  |
| 8.491667 | | | 118.3969 | | | | | | | | | | -0.4201 | | | | 4758.203 | | | 32.13281 | |  |  |  |  |  |
| 8.5 | | | 118.487 | | | | | | | | | | -0.41771 | | | | 4757.945 | | | 32.07031 | |  |  |  |  |  |
| 8.508333 | | | 118.5685 | | | | | | | | | | -0.41668 | | | | 4757.648 | | | 32.03906 | |  |  |  |  |  |
| 8.516667 | | | 118.6558 | | | | | | | | | | -0.41416 | | | | 4757.414 | | | 32.02344 | |  |  |  |  |  |
| 8.525 | | | 118.7411 | | | | | | | | | | -0.41146 | | | | 4757.156 | | | 31.82813 | |  |  |  |  |  |
| 8.533333 | | | 118.8255 | | | | | | | | | | -0.40979 | | | | 4756.938 | | | 32.11719 | |  |  |  |  |  |
| 8.541667 | | | 118.908 | | | | | | | | | | -0.40738 | | | | 4756.68 | | | 31.63281 | |  |  |  |  |  |
| 8.55 | | | 118.9933 | | | | | | | | | | -0.40521 | | | | 4756.422 | | | 31.36719 | |  |  |  |  |  |
| 8.558333 | | | 119.0777 | | | | | | | | | | -0.40385 | | | | 4756.156 | | | 31.28906 | |  |  |  |  |  |
| 8.566667 | | | 119.1649 | | | | | | | | | | -0.4016 | | | | 4755.883 | | | 31.17188 | |  |  |  |  |  |
| 8.575 | | | 119.2521 | | | | | | | | | | -0.39912 | | | | 4755.633 | | | 31.16406 | |  |  |  |  |  |
| 8.583333 | | | 119.3346 | | | | | | | | | | -0.3973 | | | | 4755.344 | | | 31.10938 | |  |  |  |  |  |
| 8.591667 | | | 119.4228 | | | | | | | | | | -0.39414 | | | | 4755.141 | | | 30.85938 | |  |  |  |  |  |
| 8.6 | | | 119.5024 | | | | | | | | | | -0.39194 | | | | 4754.906 | | | 30.5625 | |  |  |  |  |  |
| 8.608333 | | | 119.5821 | | | | | | | | | | -0.39054 | | | | 4754.664 | | | 30.46875 | |  |  |  |  |  |
| 8.616667 | | | 119.6674 | | | | | | | | | | -0.38827 | | | | 4754.414 | | | 30.33594 | |  |  |  |  |  |
| 8.625 | | | 119.7556 | | | | | | | | | | -0.38594 | | | | 4754.164 | | | 30.44531 | |  |  |  |  |  |
| 8.633333 | | | 119.8371 | | | | | | | | | | -0.38473 | | | | 4753.914 | | | 30.41406 | |  |  |  |  |  |
| 8.641667 | | | 119.9215 | | | | | | | | | | -0.38252 | | | | 4753.688 | | | 30.32813 | |  |  |  |  |  |
| 8.65 | | | 120.0059 | | | | | | | | | | -0.38038 | | | | 4753.477 | | | 30.46875 | |  |  |  |  |  |
| 8.658333 | | | 120.0865 | | | | | | | | | | -0.37948 | | | | 4753.227 | | | 30.38281 | |  |  |  |  |  |
| 8.666667 | | | 120.1671 | | | | | | | | | | -0.37793 | | | | 4752.992 | | | 30.14063 | |  |  |  |  |  |
| 8.675 | | | 120.2486 | | | | | | | | | | -0.3759 | | | | 4752.727 | | | 30.15625 | |  |  |  |  |  |
| 8.683333 | | | 120.3358 | | | | | | | | | | -0.37483 | | | | 4752.484 | | | 30.28906 | |  |  |  |  |  |
| 8.691667 | | | 120.4259 | | | | | | | | | | -0.37216 | | | | 4752.227 | | | 30.34375 | |  |  |  |  |  |
| 8.7 | | | 120.5084 | | | | | | | | | | -0.37003 | | | | 4751.953 | | | 30.42969 | |  |  |  |  |  |
| 8.708333 | | | 120.5937 | | | | | | | | | | -0.36848 | | | | 4751.711 | | | 30.45313 | |  |  |  |  |  |
| 8.716667 | | | 120.6809 | | | | | | | | | | -0.36592 | | | | 4751.492 | | | 30.46094 | |  |  |  |  |  |
| 8.725 | | | 120.7644 | | | | | | | | | | -0.36437 | | | | 4751.234 | | | 30.46875 | |  |  |  |  |  |
| 8.733333 | | | 120.8497 | | | | | | | | | | -0.36326 | | | | 4750.969 | | | 30.38281 | |  |  |  |  |  |
| 8.741667 | | | 120.9388 | | | | | | | | | | -0.3606 | | | | 4750.695 | | | 30.23438 | |  |  |  |  |  |
| 8.75 | | | 121.026 | | | | | | | | | | -0.35922 | | | | 4750.438 | | | 30.28906 | |  |  |  |  |  |
| 8.758333 | | | 121.1152 | | | | | | | | | | -0.35811 | | | | 4750.18 | | | 30.125 | |  |  |  |  |  |
| 8.766667 | | | 121.2014 | | | | | | | | | | -0.35574 | | | | 4749.922 | | | 30.29688 | |  |  |  |  |  |
| 8.775 | | | 121.2887 | | | | | | | | | | -0.35447 | | | | 4749.656 | | | 30.15625 | |  |  |  |  |  |
| 8.783333 | | | 121.3749 | | | | | | | | | | -0.3533 | | | | 4749.422 | | | 29.96875 | |  |  |  |  |  |
| 8.791667 | | | 121.4622 | | | | | | | | | | -0.35073 | | | | 4749.18 | | | 29.73438 | |  |  |  |  |  |
| 8.8 | | | 121.5475 | | | | | | | | | | -0.34922 | | | | 4748.914 | | | 29.8125 | |  |  |  |  |  |
| 8.808333 | | | 121.6347 | | | | | | | | | | -0.34766 | | | | 4748.672 | | | 29.59375 | |  |  |  |  |  |
| 8.816667 | | | 121.7191 | | | | | | | | | | -0.34473 | | | | 4748.398 | | | 29.44531 | |  |  |  |  |  |
| 8.825 | | | 121.8026 | | | | | | | | | | -0.34321 | | | | 4748.172 | | | 29.28906 | |  |  |  |  |  |
| 8.833333 | | | 121.8898 | | | | | | | | | | -0.34128 | | | | 4747.945 | | | 29.27344 | |  |  |  |  |  |
| 8.841667 | | | 121.977 | | | | | | | | | | -0.33811 | | | | 4747.727 | | | 29.10938 | |  |  |  |  |  |
| 8.85 | | | 122.0623 | | | | | | | | | | -0.33629 | | | | 4747.469 | | | 28.875 | |  |  |  |  |  |
| 8.858333 | | | 122.1486 | | | | | | | | | | -0.33462 | | | | 4747.242 | | | 28.49219 | |  |  |  |  |  |
| 8.866667 | | | 122.2358 | | | | | | | | | | -0.33198 | | | | 4747.016 | | | 28.47656 | |  |  |  |  |  |
| 8.875 | | | 122.3202 | | | | | | | | | | -0.33079 | | | | 4746.805 | | | 28.26563 | |  |  |  |  |  |
| 8.883333 | | | 122.4065 | | | | | | | | | | -0.32921 | | | | 4746.555 | | | 28.00781 | |  |  |  |  |  |
| 8.891667 | | | 122.4899 | | | | | | | | | | -0.3268 | | | | 4746.328 | | | 28 | |  |  |  |  |  |
| 8.9 | | | 122.5781 | | | | | | | | | | -0.32568 | | | | 4746.125 | | | 27.92969 | |  |  |  |  |  |
| 8.908333 | | | 122.6644 | | | | | | | | | | -0.32392 | | | | 4745.93 | | | 27.94531 | |  |  |  |  |  |
| 8.916667 | | | 122.7488 | | | | | | | | | | -0.32128 | | | | 4745.703 | | | 27.6875 | |  |  |  |  |  |
| 8.925 | | | 122.8303 | | | | | | | | | | -0.32033 | | | | 4745.5 | | | 27.75 | |  |  |  |  |  |
| 8.933333 | | | 122.9137 | | | | | | | | | | -0.31865 | | | | 4745.281 | | | 27.72656 | |  |  |  |  |  |
| 8.941667 | | | 122.9962 | | | | | | | | | | -0.31637 | | | | 4745.063 | | | 28.02344 | |  |  |  |  |  |
| 8.95 | | | 123.0797 | | | | | | | | | | -0.31537 | | | | 4744.836 | | | 27.875 | |  |  |  |  |  |
| 8.958333 | | | 123.1603 | | | | | | | | | | -0.31394 | | | | 4744.594 | | | 27.85938 | |  |  |  |  |  |
| 8.966667 | | | 123.2446 | | | | | | | | | | -0.31141 | | | | 4744.398 | | | 27.66406 | |  |  |  |  |  |
| 8.975 | | | 123.3328 | | | | | | | | | | -0.3098 | | | | 4744.156 | | | 27.53125 | |  |  |  |  |  |
| 8.983333 | | | 123.4162 | | | | | | | | | | -0.30823 | | | | 4743.93 | | | 27.55469 | |  |  |  |  |  |
| 8.991667 | | | 123.4978 | | | | | | | | | | -0.30636 | | | | 4743.648 | | | 27.21094 | |  |  |  |  |  |
| 9 | | | 123.5812 | | | | | | | | | | -0.30556 | | | | 4743.445 | | | 27.23438 | |  |  |  |  |  |
| 9.008333 | | | 123.6665 | | | | | | | | | | -0.30467 | | | | 4743.211 | | | 27.07031 | |  |  |  |  |  |
| 9.016667 | | | 123.7538 | | | | | | | | | | -0.30271 | | | | 4743.008 | | | 26.86719 | |  |  |  |  |  |
| 9.025 | | | 123.8401 | | | | | | | | | | -0.30163 | | | | 4742.797 | | | 26.57813 | |  |  |  |  |  |
| 9.033333 | | | 123.9282 | | | | | | | | | | -0.30018 | | | | 4742.563 | | | 26.59375 | |  |  |  |  |  |
| 9.041667 | | | 124.0183 | | | | | | | | | | -0.29768 | | | | 4742.375 | | | 26.6875 | |  |  |  |  |  |
| 9.05 | | | 124.1055 | | | | | | | | | | -0.29677 | | | | 4742.141 | | | 26.84375 | |  |  |  |  |  |
| 9.058333 | | | 124.1928 | | | | | | | | | | -0.29552 | | | | 4741.938 | | | 26.59375 | |  |  |  |  |  |
| 9.066667 | | | 124.279 | | | | | | | | | | -0.2934 | | | | 4741.742 | | | 26.1875 | |  |  |  |  |  |
| 9.075 | | | 124.3691 | | | | | | | | | | -0.29208 | | | | 4741.563 | | | 26.15625 | |  |  |  |  |  |
| 9.083333 | | | 124.4601 | | | | | | | | | | -0.28991 | | | | 4741.336 | | | 25.95313 | |  |  |  |  |  |
| 9.091667 | | | 124.5473 | | | | | | | | | | -0.28703 | | | | 4741.086 | | | 25.85156 | |  |  |  |  |  |
| 9.1 | | | 124.6317 | | | | | | | | | | -0.28573 | | | | 4740.844 | | | 25.89844 | |  |  |  |  |  |
| 9.108333 | | | 124.719 | | | | | | | | | | -0.28283 | | | | 4740.648 | | | 25.64063 | |  |  |  |  |  |
| 9.116667 | | | 124.8014 | | | | | | | | | | -0.28005 | | | | 4740.484 | | | 25.51563 | |  |  |  |  |  |
| 9.125 | | | 124.8906 | | | | | | | | | | -0.2791 | | | | 4740.258 | | | 25.1875 | |  |  |  |  |  |
| 9.133333 | | | 124.9797 | | | | | | | | | | -0.27629 | | | | 4740.07 | | | 25.125 | |  |  |  |  |  |
| 9.141667 | | | 125.066 | | | | | | | | | | -0.27351 | | | | 4739.867 | | | 25.14063 | |  |  |  |  |  |
| 9.15 | | | 125.1522 | | | | | | | | | | -0.27235 | | | | 4739.641 | | | 25.25 | |  |  |  |  |  |
| 9.158333 | | | 125.2393 | | | | | | | | | | -0.26941 | | | | 4739.453 | | | 25.21094 | |  |  |  |  |  |
| 9.166667 | | | 125.3236 | | | | | | | | | | -0.26687 | | | | 4739.258 | | | 25.3125 | |  |  |  |  |  |
| 9.175 | | | 125.4079 | | | | | | | | | | -0.26594 | | | | 4739.086 | | | 25.41406 | |  |  |  |  |  |
| 9.183333 | | | 125.495 | | | | | | | | | | -0.2634 | | | | 4738.891 | | | 25.29688 | |  |  |  |  |  |
| 9.191667 | | | 125.5774 | | | | | | | | | | -0.2614 | | | | 4738.672 | | | 25.28906 | |  |  |  |  |  |
| 9.2 | | | 125.6673 | | | | | | | | | | -0.26024 | | | | 4738.438 | | | 25.55469 | |  |  |  |  |  |
| 9.208333 | | | 125.7516 | | | | | | | | | | -0.25801 | | | | 4738.234 | | | 25.39063 | |  |  |  |  |  |
| 9.216667 | | | 125.8312 | | | | | | | | | | -0.25652 | | | | 4738.008 | | | 25.28125 | |  |  |  |  |  |
| 9.225 | | | 125.9136 | | | | | | | | | | -0.25573 | | | | 4737.781 | | | 25.17188 | |  |  |  |  |  |
| 9.233333 | | | 125.9998 | | | | | | | | | | -0.25315 | | | | 4737.586 | | | 25.05469 | |  |  |  |  |  |
| 9.241667 | | | 126.0859 | | | | | | | | | | -0.25085 | | | | 4737.367 | | | 25.13281 | |  |  |  |  |  |
| 9.25 | | | 126.1702 | | | | | | | | | | -0.24974 | | | | 4737.125 | | | 25.03125 | |  |  |  |  |  |
| 9.258333 | | | 126.2583 | | | | | | | | | | -0.24681 | | | | 4736.938 | | | 24.72656 | |  |  |  |  |  |
| 9.266667 | | | 126.3444 | | | | | | | | | | -0.24477 | | | | 4736.742 | | | 24.71094 | |  |  |  |  |  |
| 9.275 | | | 126.4334 | | | | | | | | | | -0.24366 | | | | 4736.547 | | | 24.64063 | |  |  |  |  |  |
| 9.283333 | | | 126.5186 | | | | | | | | | | -0.2412 | | | | 4736.352 | | | 24.25 | |  |  |  |  |  |
| 9.291667 | | | 126.6001 | | | | | | | | | | -0.23922 | | | | 4736.133 | | | 24.03906 | |  |  |  |  |  |
| 9.3 | | | 126.6872 | | | | | | | | | | -0.23795 | | | | 4735.938 | | | 24.09375 | |  |  |  |  |  |
| 9.308333 | | | 126.7752 | | | | | | | | | | -0.23514 | | | | 4735.758 | | | 24.21875 | |  |  |  |  |  |
| 9.316667 | | | 126.8605 | | | | | | | | | | -0.23318 | | | | 4735.57 | | | 23.97656 | |  |  |  |  |  |
| 9.325 | | | 126.9466 | | | | | | | | | | -0.23208 | | | | 4735.367 | | | 23.79688 | |  |  |  |  |  |
| 9.333333 | | | 127.0328 | | | | | | | | | | -0.22919 | | | | 4735.211 | | | 23.71094 | |  |  |  |  |  |
| 9.341667 | | | 127.1189 | | | | | | | | | | -0.22744 | | | | 4735.031 | | | 23.625 | |  |  |  |  |  |
| 9.35 | | | 127.2051 | | | | | | | | | | -0.22643 | | | | 4734.82 | | | 23.625 | |  |  |  |  |  |
| 9.358333 | | | 127.2894 | | | | | | | | | | -0.22387 | | | | 4734.602 | | | 23.49219 | |  |  |  |  |  |
| 9.366667 | | | 127.3737 | | | | | | | | | | -0.22208 | | | | 4734.422 | | | 23.57813 | |  |  |  |  |  |
| 9.375 | | | 127.4608 | | | | | | | | | | -0.22042 | | | | 4734.258 | | | 23.66406 | |  |  |  |  |  |
| 9.383333 | | | 127.5497 | | | | | | | | | | -0.21744 | | | | 4734.07 | | | 23.64063 | |  |  |  |  |  |
| 9.391667 | | | 127.6387 | | | | | | | | | | -0.21549 | | | | 4733.875 | | | 23.21875 | |  |  |  |  |  |
| 9.4 | | | 127.7258 | | | | | | | | | | -0.214 | | | | 4733.688 | | | 23.00781 | |  |  |  |  |  |
| 9.408333 | | | 127.811 | | | | | | | | | | -0.21164 | | | | 4733.508 | | | 22.85156 | |  |  |  |  |  |
| 9.416667 | | | 127.8963 | | | | | | | | | | -0.21046 | | | | 4733.289 | | | 22.82031 | |  |  |  |  |  |
| 9.425 | | | 127.9824 | | | | | | | | | | -0.20891 | | | | 4733.086 | | | 22.54688 | |  |  |  |  |  |
| 9.433333 | | | 128.0695 | | | | | | | | | | -0.20631 | | | | 4732.898 | | | 22.32813 | |  |  |  |  |  |
| 9.441667 | | | 128.1547 | | | | | | | | | | -0.20486 | | | | 4732.758 | | | 22.375 | |  |  |  |  |  |
| 9.45 | | | 128.2409 | | | | | | | | | | -0.20317 | | | | 4732.586 | | | 22.10938 | |  |  |  |  |  |
| 9.458333 | | | 128.3299 | | | | | | | | | | -0.20067 | | | | 4732.414 | | | 21.96094 | |  |  |  |  |  |
| 9.466667 | | | 128.4179 | | | | | | | | | | -0.19931 | | | | 4732.234 | | | 21.82031 | |  |  |  |  |  |
| 9.475 | | | 128.5041 | | | | | | | | | | -0.19774 | | | | 4732.078 | | | 21.84375 | |  |  |  |  |  |
| 9.483333 | | | 128.5893 | | | | | | | | | | -0.19523 | | | | 4731.922 | | | 22.03125 | |  |  |  |  |  |
| 9.491667 | | | 128.6745 | | | | | | | | | | -0.19391 | | | | 4731.727 | | | 22 | |  |  |  |  |  |
| 9.5 | | | 128.7616 | | | | | | | | | | -0.19214 | | | | 4731.578 | | | 21.99219 | |  |  |  |  |  |
| 9.508333 | | | 128.845 | | | | | | | | | | -0.18991 | | | | 4731.414 | | | 22.00781 | |  |  |  |  |  |
| 9.516667 | | | 128.9302 | | | | | | | | | | -0.18851 | | | | 4731.25 | | | 22.14063 | |  |  |  |  |  |
| 9.525 | | | 129.0201 | | | | | | | | | | -0.18688 | | | | 4731.063 | | | 21.9375 | |  |  |  |  |  |
| 9.533333 | | | 129.11 | | | | | | | | | | -0.18432 | | | | 4730.852 | | | 22.07813 | |  |  |  |  |  |
| 9.541667 | | | 129.198 | | | | | | | | | | -0.183 | | | | 4730.672 | | | 22.25 | |  |  |  |  |  |
| 9.55 | | | 129.2833 | | | | | | | | | | -0.18114 | | | | 4730.477 | | | 22.20313 | |  |  |  |  |  |
| 9.558333 | | | 129.3676 | | | | | | | | | | -0.17882 | | | | 4730.289 | | | 22.4375 | |  |  |  |  |  |
| 9.566667 | | | 129.4537 | | | | | | | | | | -0.17759 | | | | 4730.078 | | | 22.34375 | |  |  |  |  |  |
| 9.575 | | | 129.5399 | | | | | | | | | | -0.17572 | | | | 4729.922 | | | 22.23438 | |  |  |  |  |  |
| 9.583333 | | | 129.627 | | | | | | | | | | -0.17334 | | | | 4729.727 | | | 22.25 | |  |  |  |  |  |
| 9.591667 | | | 129.715 | | | | | | | | | | -0.17172 | | | | 4729.508 | | | 22.11719 | |  |  |  |  |  |
| 9.6 | | | 129.803 | | | | | | | | | | -0.16914 | | | | 4729.313 | | | 22.00781 | |  |  |  |  |  |
| 9.608333 | | | 129.8855 | | | | | | | | | | -0.16697 | | | | 4729.102 | | | 21.92188 | |  |  |  |  |  |
| 9.616667 | | | 129.9688 | | | | | | | | | | -0.16581 | | | | 4728.922 | | | 21.72656 | |  |  |  |  |  |
| 9.625 | | | 130.0503 | | | | | | | | | | -0.16416 | | | | 4728.75 | | | 21.54688 | |  |  |  |  |  |
| 9.633333 | | | 130.1318 | | | | | | | | | | -0.16198 | | | | 4728.57 | | | 21.64844 | |  |  |  |  |  |
| 9.641667 | | | 130.2189 | | | | | | | | | | -0.16081 | | | | 4728.398 | | | 21.29688 | |  |  |  |  |  |
| 9.65 | | | 130.3041 | | | | | | | | | | -0.15935 | | | | 4728.234 | | | 21.38281 | |  |  |  |  |  |
| 9.658333 | | | 130.3902 | | | | | | | | | | -0.157 | | | | 4728.055 | | | 21.51563 | |  |  |  |  |  |
| 9.666667 | | | 130.4755 | | | | | | | | | | -0.15572 | | | | 4727.898 | | | 21.25781 | |  |  |  |  |  |
| 9.675 | | | 130.5626 | | | | | | | | | | -0.15432 | | | | 4727.734 | | | 21.28125 | |  |  |  |  |  |
| 9.683333 | | | 130.6469 | | | | | | | | | | -0.15159 | | | | 4727.555 | | | 21.22656 | |  |  |  |  |  |
| 9.691667 | | | 130.7358 | | | | | | | | | | -0.14991 | | | | 4727.422 | | | 21.28125 | |  |  |  |  |  |
| 9.7 | | | 130.8229 | | | | | | | | | | -0.14828 | | | | 4727.227 | | | 21.42969 | |  |  |  |  |  |
| 9.708333 | | | 130.9128 | | | | | | | | | | -0.14524 | | | | 4727.023 | | | 21.16406 | |  |  |  |  |  |
| 9.716667 | | | 131.0018 | | | | | | | | | | -0.1438 | | | | 4726.883 | | | 20.86719 | |  |  |  |  |  |
| 9.725 | | | 131.0889 | | | | | | | | | | -0.14184 | | | | 4726.703 | | | 20.84375 | |  |  |  |  |  |
| 9.733333 | | | 131.1797 | | | | | | | | | | -0.1391 | | | | 4726.531 | | | 20.64063 | |  |  |  |  |  |
| 9.741667 | | | 131.2668 | | | | | | | | | | -0.13773 | | | | 4726.344 | | | 20.36719 | |  |  |  |  |  |
| 9.75 | | | 131.3568 | | | | | | | | | | -0.13531 | | | | 4726.148 | | | 20.60156 | |  |  |  |  |  |
| 9.758333 | | | 131.4438 | | | | | | | | | | -0.13319 | | | | 4726.008 | | | 20.32813 | |  |  |  |  |  |
| 9.766667 | | | 131.5309 | | | | | | | | | | -0.13234 | | | | 4725.875 | | | 20.22656 | |  |  |  |  |  |
| 9.775 | | | 131.6208 | | | | | | | | | | -0.12975 | | | | 4725.695 | | | 20.14844 | |  |  |  |  |  |
| 9.783333 | | | 131.7061 | | | | | | | | | | -0.12824 | | | | 4725.547 | | | 19.875 | |  |  |  |  |  |
| 9.791667 | | | 131.7904 | | | | | | | | | | -0.12739 | | | | 4725.414 | | | 20.00781 | |  |  |  |  |  |
| 9.8 | | | 131.8784 | | | | | | | | | | -0.12492 | | | | 4725.219 | | | 20.01563 | |  |  |  |  |  |
| 9.808333 | | | 131.9646 | | | | | | | | | | -0.12349 | | | | 4725.094 | | | 20.00781 | |  |  |  |  |  |
| 9.816667 | | | 132.0498 | | | | | | | | | | -0.12245 | | | | 4724.93 | | | 19.96875 | |  |  |  |  |  |
| 9.825 | | | 132.1341 | | | | | | | | | | -0.11966 | | | | 4724.781 | | | 19.97656 | |  |  |  |  |  |
| 9.833333 | | | 132.2184 | | | | | | | | | | -0.11827 | | | | 4724.648 | | | 19.66406 | |  |  |  |  |  |
| 9.841667 | | | 132.3055 | | | | | | | | | | -0.11712 | | | | 4724.453 | | | 19.85156 | |  |  |  |  |  |
| 9.85 | | | 132.3897 | | | | | | | | | | -0.1141 | | | | 4724.289 | | | 19.80469 | |  |  |  |  |  |
| 9.858333 | | | 132.475 | | | | | | | | | | -0.11308 | | | | 4724.125 | | | 19.625 | |  |  |  |  |  |
| 9.866667 | | | 132.5639 | | | | | | | | | | -0.11227 | | | | 4723.961 | | | 19.71875 | |  |  |  |  |  |
| 9.875 | | | 132.6539 | | | | | | | | | | -0.10972 | | | | 4723.805 | | | 19.53906 | |  |  |  |  |  |
| 9.883333 | | | 132.7391 | | | | | | | | | | -0.10887 | | | | 4723.672 | | | 19.4375 | |  |  |  |  |  |
| 9.891667 | | | 132.8243 | | | | | | | | | | -0.10794 | | | | 4723.477 | | | 19.38281 | |  |  |  |  |  |
| 9.9 | | | 132.9123 | | | | | | | | | | -0.10539 | | | | 4723.32 | | | 19.27344 | |  |  |  |  |  |
| 9.908333 | | | 133.0041 | | | | | | | | | | -0.10387 | | | | 4723.172 | | | 19.17188 | |  |  |  |  |  |
| 9.916667 | | | 133.094 | | | | | | | | | | -0.10254 | | | | 4722.992 | | | 19.14844 | |  |  |  |  |  |
| 9.925 | | | 133.1802 | | | | | | | | | | -0.09963 | | | | 4722.852 | | | 18.97656 | |  |  |  |  |  |
| 9.933333 | | | 133.2701 | | | | | | | | | | -0.09838 | | | | 4722.703 | | | 19.00781 | |  |  |  |  |  |
| 9.941667 | | | 133.3572 | | | | | | | | | | -0.09682 | | | | 4722.547 | | | 19.07813 | |  |  |  |  |  |
| 9.95 | | | 133.4452 | | | | | | | | | | -0.09304 | | | | 4722.398 | | | 19.01563 | |  |  |  |  |  |
| 9.958333 | | | 133.5314 | | | | | | | | | | -0.09117 | | | | 4722.242 | | | 19.09375 | |  |  |  |  |  |
| 9.966667 | | | 133.6213 | | | | | | | | | | -0.08865 | | | | 4722.086 | | | 19.125 | |  |  |  |  |  |
| 9.975 | | | 133.7121 | | | | | | | | | | -0.08472 | | | | 4721.945 | | | 18.99219 | |  |  |  |  |  |
| 9.983333 | | | 133.802 | | | | | | | | | | -0.08323 | | | | 4721.781 | | | 18.78125 | |  |  |  |  |  |
| 9.991667 | | | 133.891 | | | | | | | | | | -0.08135 | | | | 4721.609 | | | 18.71875 | |  |  |  |  |  |
| 10 | | | 133.979 | | | | | | | | | | -0.07878 | | | | 4721.461 | | | 18.73438 | |  |  |  |  |  |
| 10.00833 | | | 134.0699 | | | | | | | | | | -0.07802 | | | | 4721.289 | | | 18.64063 | |  |  |  |  |  |
| 10.01667 | | | 134.1589 | | | | | | | | | | -0.07652 | | | | 4721.125 | | | 18.53125 | |  |  |  |  |  |
| 10.025 | | | 134.2469 | | | | | | | | | | -0.0742 | | | | 4720.992 | | | 18.33594 | |  |  |  |  |  |
| 10.03333 | | | 134.3368 | | | | | | | | | | -0.07356 | | | | 4720.859 | | | 18.25781 | |  |  |  |  |  |
| 10.04167 | | | 134.4239 | | | | | | | | | | -0.07216 | | | | 4720.711 | | | 18.10938 | |  |  |  |  |  |
| 10.05 | | | 134.5119 | | | | | | | | | | -0.07041 | | | | 4720.547 | | | 17.96094 | |  |  |  |  |  |
| 10.05833 | | | 134.599 | | | | | | | | | | -0.07011 | | | | 4720.398 | | | 17.91406 | |  |  |  |  |  |
| 10.06667 | | | 134.6842 | | | | | | | | | | -0.06876 | | | | 4720.266 | | | 17.82813 | |  |  |  |  |  |
| 10.075 | | | 134.7695 | | | | | | | | | | -0.06647 | | | | 4720.133 | | | 17.72656 | |  |  |  |  |  |
| 10.08333 | | | 134.8556 | | | | | | | | | | -0.06547 | | | | 4719.984 | | | 17.69531 | |  |  |  |  |  |
| 10.09167 | | | 134.9427 | | | | | | | | | | -0.06364 | | | | 4719.852 | | | 17.6875 | |  |  |  |  |  |
| 10.1 | | | 135.0326 | | | | | | | | | | -0.06054 | | | | 4719.719 | | | 17.53906 | |  |  |  |  |  |
| 10.10833 | | | 135.1197 | | | | | | | | | | -0.05945 | | | | 4719.578 | | | 17.53125 | |  |  |  |  |  |
| 10.11667 | | | 135.2059 | | | | | | | | | | -0.05753 | | | | 4719.438 | | | 17.46094 | |  |  |  |  |  |
| 10.125 | | | 135.2958 | | | | | | | | | | -0.05491 | | | | 4719.305 | | | 17.51563 | |  |  |  |  |  |
| 10.13333 | | | 135.3829 | | | | | | | | | | -0.05382 | | | | 4719.172 | | | 17.69531 | |  |  |  |  |  |
| 10.14167 | | | 135.4709 | | | | | | | | | | -0.05157 | | | | 4719.016 | | | 17.70313 | |  |  |  |  |  |
| 10.15 | | | 135.5599 | | | | | | | | | | -0.04881 | | | | 4718.891 | | | 17.92188 | |  |  |  |  |  |
| 10.15833 | | | 135.6506 | | | | | | | | | | -0.04751 | | | | 4718.742 | | | 18.00781 | |  |  |  |  |  |
| 10.16667 | | | 135.7366 | | | | | | | | | | -0.0454 | | | | 4718.609 | | | 18.09375 | |  |  |  |  |  |
| 10.175 | | | 135.8245 | | | | | | | | | | -0.04287 | | | | 4718.453 | | | 18.03125 | |  |  |  |  |  |
| 10.18333 | | | 135.9096 | | | | | | | | | | -0.042 | | | | 4718.281 | | | 18.14844 | |  |  |  |  |  |
| 10.19167 | | | 135.9956 | | | | | | | | | | -0.03995 | | | | 4718.125 | | | 17.99219 | |  |  |  |  |  |
| 10.2 | | | 136.0844 | | | | | | | | | | -0.03745 | | | | 4717.953 | | | 17.89063 | |  |  |  |  |  |
| 10.20833 | | | 136.1685 | | | | | | | | | | -0.03639 | | | | 4717.781 | | | 17.55469 | |  |  |  |  |  |
| 10.21667 | | | 136.2564 | | | | | | | | | | -0.03379 | | | | 4717.617 | | | 17.42969 | |  |  |  |  |  |
| 10.225 | | | 136.3452 | | | | | | | | | | -0.03137 | | | | 4717.469 | | | 17.25 | |  |  |  |  |  |
| 10.23333 | | | 136.4322 | | | | | | | | | | -0.03035 | | | | 4717.305 | | | 17.07813 | |  |  |  |  |  |
| 10.24167 | | | 136.5173 | | | | | | | | | | -0.02825 | | | | 4717.172 | | | 16.96094 | |  |  |  |  |  |
| 10.25 | | | 136.606 | | | | | | | | | | -0.02636 | | | | 4717.039 | | | 16.63281 | |  |  |  |  |  |
| 10.25833 | | | 136.6911 | | | | | | | | | | -0.0254 | | | | 4716.938 | | | 16.36719 | |  |  |  |  |  |
| 10.26667 | | | 136.7744 | | | | | | | | | | -0.02305 | | | | 4716.805 | | | 16.41406 | |  |  |  |  |  |
| 10.275 | | | 136.8623 | | | | | | | | | | -0.02087 | | | | 4716.688 | | | 16.22656 | |  |  |  |  |  |
| 10.28333 | | | 136.9474 | | | | | | | | | | -0.01955 | | | | 4716.578 | | | 16.125 | |  |  |  |  |  |
| 10.29167 | | | 137.0343 | | | | | | | | | | -0.01693 | | | | 4716.453 | | | 16.05469 | |  |  |  |  |  |
| 10.3 | | | 137.1203 | | | | | | | | | | -0.01528 | | | | 4716.352 | | | 16.03125 | |  |  |  |  |  |
| 10.30833 | | | 137.2054 | | | | | | | | | | -0.01421 | | | | 4716.25 | | | 16.0625 | |  |  |  |  |  |
| 10.31667 | | | 137.2887 | | | | | | | | | | -0.0121 | | | | 4716.102 | | | 16.07031 | |  |  |  |  |  |
| 10.325 | | | 137.3738 | | | | | | | | | | -0.01014 | | | | 4716 | | | 16.02344 | |  |  |  |  |  |
| 10.33333 | | | 137.457 | | | | | | | | | | -0.00918 | | | | 4715.875 | | | 16.07813 | |  |  |  |  |  |
| 10.34167 | | | 137.5403 | | | | | | | | | | -0.00685 | | | | 4715.742 | | | 16.33594 | |  |  |  |  |  |
| 10.35 | | | 137.6309 | | | | | | | | | | -0.00478 | | | | 4715.617 | | | 16.25 | |  |  |  |  |  |
| 10.35833 | | | 137.7188 | | | | | | | | | | -0.00383 | | | | 4715.469 | | | 16.49219 | |  |  |  |  |  |
| 10.36667 | | | 137.8048 | | | | | | | | | | -0.00177 | | | | 4715.328 | | | 16.6875 | |  |  |  |  |  |
| 10.375 | | | 137.8945 | | | | | | | | | | -6.2E-05 | | | | 4715.203 | | | 16.67188 | |  |  |  |  |  |
| 10.38333 | | | 137.9824 | | | | | | | | | | 0.0011 | | | | 4715.063 | | | 16.61719 | |  |  |  |  |  |
| 10.39167 | | | 138.0693 | | | | | | | | | | 0.003471 | | | | 4714.883 | | | 16.52344 | |  |  |  |  |  |
| 10.4 | | | 138.1581 | | | | | | | | | | 0.005079 | | | | 4714.75 | | | 16.59375 | |  |  |  |  |  |
| 10.40833 | | | 138.2432 | | | | | | | | | | 0.005931 | | | | 4714.578 | | | 16.39063 | |  |  |  |  |  |
| 10.41667 | | | 138.332 | | | | | | | | | | 0.008008 | | | | 4714.422 | | | 16.35156 | |  |  |  |  |  |
| 10.425 | | | 138.4227 | | | | | | | | | | 0.009373 | | | | 4714.273 | | | 16 | |  |  |  |  |  |
| 10.43333 | | | 138.5105 | | | | | | | | | | 0.0103 | | | | 4714.148 | | | 15.86719 | |  |  |  |  |  |
| 10.44167 | | | 138.5984 | | | | | | | | | | 0.01274 | | | | 4714.016 | | | 15.70313 | |  |  |  |  |  |
| 10.45 | | | 138.6853 | | | | | | | | | | 0.014348 | | | | 4713.875 | | | 15.40625 | |  |  |  |  |  |
| 10.45833 | | | 138.7741 | | | | | | | | | | 0.015834 | | | | 4713.773 | | | 15.21875 | |  |  |  |  |  |
| 10.46667 | | | 138.8629 | | | | | | | | | | 0.018113 | | | | 4713.633 | | | 15.14063 | |  |  |  |  |  |
| 10.475 | | | 138.9499 | | | | | | | | | | 0.019284 | | | | 4713.555 | | | 14.73438 | |  |  |  |  |  |
| 10.48333 | | | 139.0378 | | | | | | | | | | 0.020644 | | | | 4713.445 | | | 14.55469 | |  |  |  |  |  |
| 10.49167 | | | 139.133 | | | | | | | | | | 0.02369 | | | | 4713.328 | | | 14.60156 | |  |  |  |  |  |
| 10.5 | | | 139.2227 | | | | | | | | | | 0.024991 | | | | 4713.25 | | | 14.42188 | |  |  |  |  |  |
| 10.50833 | | | 139.3097 | | | | | | | | | | 0.026861 | | | | 4713.141 | | | 14.38281 | |  |  |  |  |  |
| 10.51667 | | | 139.3966 | | | | | | | | | | 0.029173 | | | | 4713.023 | | | 14.45313 | |  |  |  |  |  |
| 10.525 | | | 139.4817 | | | | | | | | | | 0.029882 | | | | 4712.953 | | | 14.22656 | |  |  |  |  |  |
| 10.53333 | | | 139.5742 | | | | | | | | | | 0.031787 | | | | 4712.852 | | | 14.26563 | |  |  |  |  |  |
| 10.54167 | | | 139.6612 | | | | | | | | | | 0.034041 | | | | 4712.719 | | | 14.23438 | |  |  |  |  |  |
| 10.55 | | | 139.75 | | | | | | | | | | 0.035138 | | | | 4712.625 | | | 14.1875 | |  |  |  |  |  |
| 10.55833 | | | 139.8369 | | | | | | | | | | 0.037328 | | | | 4712.492 | | | 14.25781 | |  |  |  |  |  |
| 10.56667 | | | 139.9238 | | | | | | | | | | 0.039457 | | | | 4712.367 | | | 13.99219 | |  |  |  |  |  |
| 10.575 | | | 140.0099 | | | | | | | | | | 0.040083 | | | | 4712.266 | | | 13.80469 | |  |  |  |  |  |
| 10.58333 | | | 140.0977 | | | | | | | | | | 0.041898 | | | | 4712.141 | | | 14.01563 | |  |  |  |  |  |
| 10.59167 | | | 140.1828 | | | | | | | | | | 0.044147 | | | | 4712.023 | | | 13.99219 | |  |  |  |  |  |
| 10.6 | | | 140.2698 | | | | | | | | | | 0.045272 | | | | 4711.914 | | | 13.91406 | |  |  |  |  |  |
| 10.60833 | | | 140.3595 | | | | | | | | | | 0.047164 | | | | 4711.789 | | | 13.75 | |  |  |  |  |  |
| 10.61667 | | | 140.4455 | | | | | | | | | | 0.049445 | | | | 4711.711 | | | 13.70313 | |  |  |  |  |  |
| 10.625 | | | 140.5325 | | | | | | | | | | 0.0505 | | | | 4711.617 | | | 13.42969 | |  |  |  |  |  |
| 10.63333 | | | 140.6185 | | | | | | | | | | 0.052574 | | | | 4711.477 | | | 13.30469 | |  |  |  |  |  |
| 10.64167 | | | 140.7082 | | | | | | | | | | 0.055426 | | | | 4711.352 | | | 13.41406 | |  |  |  |  |  |
| 10.65 | | | 140.7979 | | | | | | | | | | 0.056992 | | | | 4711.258 | | | 13.58594 | |  |  |  |  |  |
| 10.65833 | | | 140.8812 | | | | | | | | | | 0.059715 | | | | 4711.156 | | | 13.53125 | |  |  |  |  |  |
| 10.66667 | | | 140.9681 | | | | | | | | | | 0.062522 | | | | 4711.063 | | | 13.32031 | |  |  |  |  |  |
| 10.675 | | | 141.0569 | | | | | | | | | | 0.063814 | | | | 4710.984 | | | 13.1875 | |  |  |  |  |  |
| 10.68333 | | | 141.1485 | | | | | | | | | | 0.066359 | | | | 4710.891 | | | 13.03906 | |  |  |  |  |  |
| 10.69167 | | | 141.2373 | | | | | | | | | | 0.068619 | | | | 4710.75 | | | 13.1875 | |  |  |  |  |  |
| 10.7 | | | 141.3224 | | | | | | | | | | 0.06919 | | | | 4710.617 | | | 13.375 | |  |  |  |  |  |
| 10.70833 | | | 141.4093 | | | | | | | | | | 0.07114 | | | | 4710.508 | | | 13.50781 | |  |  |  |  |  |
| 10.71667 | | | 141.499 | | | | | | | | | | 0.072948 | | | | 4710.43 | | | 13.53906 | |  |  |  |  |  |
| 10.725 | | | 141.586 | | | | | | | | | | 0.073378 | | | | 4710.328 | | | 13.40625 | |  |  |  |  |  |
| 10.73333 | | | 141.6729 | | | | | | | | | | 0.075429 | | | | 4710.242 | | | 13.46094 | |  |  |  |  |  |
| 10.74167 | | | 141.7617 | | | | | | | | | | 0.077135 | | | | 4710.102 | | | 13.51563 | |  |  |  |  |  |
| 10.75 | | | 141.8514 | | | | | | | | | | 0.077672 | | | | 4709.953 | | | 13.67188 | |  |  |  |  |  |
| 10.75833 | | | 141.9412 | | | | | | | | | | 0.079915 | | | | 4709.82 | | | 13.46094 | |  |  |  |  |  |
| 10.76667 | | | 142.0281 | | | | | | | | | | 0.081729 | | | | 4709.695 | | | 13.49219 | |  |  |  |  |  |
| 10.775 | | | 142.1141 | | | | | | | | | | 0.082324 | | | | 4709.602 | | | 13.42969 | |  |  |  |  |  |
| 10.78333 | | | 142.202 | | | | | | | | | | 0.084634 | | | | 4709.477 | | | 13.34375 | |  |  |  |  |  |
| 10.79167 | | | 142.2889 | | | | | | | | | | 0.086601 | | | | 4709.352 | | | 13.42969 | |  |  |  |  |  |
| 10.8 | | | 142.3731 | | | | | | | | | | 0.087383 | | | | 4709.211 | | | 13.53125 | |  |  |  |  |  |
| 10.80833 | | | 142.4601 | | | | | | | | | | 0.089739 | | | | 4709.133 | | | 13.69531 | |  |  |  |  |  |
| 10.81667 | | | 142.5479 | | | | | | | | | | 0.091456 | | | | 4709 | | | 13.36719 | |  |  |  |  |  |
| 10.825 | | | 142.6367 | | | | | | | | | | 0.092161 | | | | 4708.922 | | | 13.3125 | |  |  |  |  |  |
| 10.83333 | | | 142.7237 | | | | | | | | | | 0.094679 | | | | 4708.813 | | | 13.10156 | |  |  |  |  |  |
| 10.84167 | | | 142.8125 | | | | | | | | | | 0.096817 | | | | 4708.688 | | | 13.04688 | |  |  |  |  |  |
| 10.85 | | | 142.8966 | | | | | | | | | | 0.097659 | | | | 4708.563 | | | 13.10156 | |  |  |  |  |  |
| 10.85833 | | | 142.9827 | | | | | | | | | | 0.100327 | | | | 4708.438 | | | 13.03125 | |  |  |  |  |  |
| 10.86667 | | | 143.0733 | | | | | | | | | | 0.102017 | | | | 4708.359 | | | 12.70313 | |  |  |  |  |  |
| 10.875 | | | 143.1649 | | | | | | | | | | 0.102637 | | | | 4708.266 | | | 12.5 | |  |  |  |  |  |
| 10.88333 | | | 143.2564 | | | | | | | | | | 0.105482 | | | | 4708.188 | | | 12.33594 | |  |  |  |  |  |
| 10.89167 | | | 143.3443 | | | | | | | | | | 0.106993 | | | | 4708.078 | | | 12.03906 | |  |  |  |  |  |
| 10.9 | | | 143.434 | | | | | | | | | | 0.107751 | | | | 4707.977 | | | 12.07031 | |  |  |  |  |  |
| 10.90833 | | | 143.5237 | | | | | | | | | | 0.110733 | | | | 4707.867 | | | 12.07813 | |  |  |  |  |  |
| 10.91667 | | | 143.6116 | | | | | | | | | | 0.111744 | | | | 4707.805 | | | 12.00781 | |  |  |  |  |  |
| 10.925 | | | 143.6976 | | | | | | | | | | 0.112062 | | | | 4707.727 | | | 12.125 | |  |  |  |  |  |
| 10.93333 | | | 143.7874 | | | | | | | | | | 0.114814 | | | | 4707.648 | | | 11.92188 | |  |  |  |  |  |
| 10.94167 | | | 143.8799 | | | | | | | | | | 0.115825 | | | | 4707.586 | | | 11.65625 | |  |  |  |  |  |
| 10.95 | | | 143.9686 | | | | | | | | | | 0.116729 | | | | 4707.477 | | | 11.75781 | |  |  |  |  |  |
| 10.95833 | | | 144.0547 | | | | | | | | | | 0.119742 | | | | 4707.375 | | | 11.99219 | |  |  |  |  |  |
| 10.96667 | | | 144.1407 | | | | | | | | | | 0.120833 | | | | 4707.289 | | | 12.10938 | |  |  |  |  |  |
| 10.975 | | | 144.2332 | | | | | | | | | | 0.121911 | | | | 4707.172 | | | 12.10156 | |  |  |  |  |  |
| 10.98333 | | | 144.321 | | | | | | | | | | 0.124907 | | | | 4707.094 | | | 11.97656 | |  |  |  |  |  |
| 10.99167 | | | 144.408 | | | | | | | | | | 0.125961 | | | | 4707.031 | | | 11.71875 | |  |  |  |  |  |
| 11 | | | 144.4959 | | | | | | | | | | 0.126668 | | | | 4706.922 | | | 11.61719 | |  |  |  |  |  |
| 11.00833 | | | 144.5856 | | | | | | | | | | 0.129762 | | | | 4706.789 | | | 11.33594 | |  |  |  |  |  |
| 11.01667 | | | 144.6753 | | | | | | | | | | 0.130643 | | | | 4706.672 | | | 11.4375 | |  |  |  |  |  |
| 11.025 | | | 144.7623 | | | | | | | | | | 0.131653 | | | | 4706.57 | | | 11.625 | |  |  |  |  |  |
| 11.03333 | | | 144.851 | | | | | | | | | | 0.134422 | | | | 4706.492 | | | 11.76563 | |  |  |  |  |  |
| 11.04167 | | | 144.9389 | | | | | | | | | | 0.135121 | | | | 4706.43 | | | 11.65625 | |  |  |  |  |  |
| 11.05 | | | 145.0277 | | | | | | | | | | 0.135794 | | | | 4706.336 | | | 11.29688 | |  |  |  |  |  |
| 11.05833 | | | 145.1147 | | | | | | | | | | 0.138542 | | | | 4706.289 | | | 11.57031 | |  |  |  |  |  |
| 11.06667 | | | 145.2044 | | | | | | | | | | 0.139152 | | | | 4706.172 | | | 11.57813 | |  |  |  |  |  |
| 11.075 | | | 145.2941 | | | | | | | | | | 0.13988 | | | | 4706.055 | | | 11.79688 | |  |  |  |  |  |
| 11.08333 | | | 145.3847 | | | | | | | | | | 0.142565 | | | | 4705.93 | | | 11.82813 | |  |  |  |  |  |
| 11.09167 | | | 145.4754 | | | | | | | | | | 0.14334 | | | | 4705.852 | | | 12.07813 | |  |  |  |  |  |
| 11.1 | | | 145.5688 | | | | | | | | | | 0.145054 | | | | 4705.797 | | | 11.99219 | |  |  |  |  |  |
| 11.10833 | | | 145.6594 | | | | | | | | | | 0.14811 | | | | 4705.656 | | | 11.63281 | |  |  |  |  |  |
| 11.11667 | | | 145.7442 | | | | | | | | | | 0.149263 | | | | 4705.547 | | | 11.375 | |  |  |  |  |  |
| 11.125 | | | 145.8353 | | | | | | | | | | 0.151271 | | | | 4705.43 | | | 11.32031 | |  |  |  |  |  |
| 11.13333 | | | 145.9209 | | | | | | | | | | 0.153826 | | | | 4705.32 | | | 11.30469 | |  |  |  |  |  |
| 11.14167 | | | 146.0075 | | | | | | | | | | 0.154863 | | | | 4705.18 | | | 11.08594 | |  |  |  |  |  |
| 11.15 | | | 146.0914 | | | | | | | | | | 0.156677 | | | | 4705.086 | | | 11.01563 | |  |  |  |  |  |
| 11.15833 | | | 146.1797 | | | | | | | | | | 0.159701 | | | | 4705.039 | | | 10.94531 | |  |  |  |  |  |
| 11.16667 | | | 146.2654 | | | | | | | | | | 0.160788 | | | | 4704.977 | | | 10.96094 | |  |  |  |  |  |
| 11.175 | | | 146.352 | | | | | | | | | | 0.162739 | | | | 4704.891 | | | 10.70313 | |  |  |  |  |  |
| 11.18333 | | | 146.4349 | | | | | | | | | | 0.164912 | | | | 4704.797 | | | 10.67969 | |  |  |  |  |  |
| 11.19167 | | | 146.5224 | | | | | | | | | | 0.165773 | | | | 4704.734 | | | 11.21094 | |  |  |  |  |  |
| 11.2 | | | 146.6099 | | | | | | | | | | 0.167652 | | | | 4704.648 | | | 11.05469 | |  |  |  |  |  |
| 11.20833 | | | 146.6937 | | | | | | | | | | 0.170267 | | | | 4704.563 | | | 10.88281 | |  |  |  |  |  |
| 11.21667 | | | 146.7812 | | | | | | | | | | 0.171201 | | | | 4704.477 | | | 11.02344 | |  |  |  |  |  |
| 11.225 | | | 146.8696 | | | | | | | | | | 0.173047 | | | | 4704.414 | | | 10.98438 | |  |  |  |  |  |
| 11.23333 | | | 146.9598 | | | | | | | | | | 0.175361 | | | | 4704.328 | | | 10.84375 | |  |  |  |  |  |
| 11.24167 | | | 147.0463 | | | | | | | | | | 0.176023 | | | | 4704.164 | | | 10.78125 | |  |  |  |  |  |
| 11.25 | | | 147.1329 | | | | | | | | | | 0.17788 | | | | 4704.086 | | | 10.89063 | |  |  |  |  |  |
| 11.25833 | | | 147.2213 | | | | | | | | | | 0.180407 | | | | 4704.016 | | | 11.09375 | |  |  |  |  |  |
| 11.26667 | | | 147.3078 | | | | | | | | | | 0.181442 | | | | 4703.922 | | | 11.22656 | |  |  |  |  |  |
| 11.275 | | | 147.3935 | | | | | | | | | | 0.18344 | | | | 4703.828 | | | 10.86719 | |  |  |  |  |  |
| 11.28333 | | | 147.4801 | | | | | | | | | | 0.185555 | | | | 4703.758 | | | 11.02344 | |  |  |  |  |  |
| 11.29167 | | | 147.5667 | | | | | | | | | | 0.186514 | | | | 4703.68 | | | 10.97656 | |  |  |  |  |  |
| 11.3 | | | 147.6568 | | | | | | | | | | 0.188956 | | | | 4703.57 | | | 10.85156 | |  |  |  |  |  |
| 11.30833 | | | 147.7434 | | | | | | | | | | 0.191121 | | | | 4703.445 | | | 10.57813 | |  |  |  |  |  |
| 11.31667 | | | 147.83 | | | | | | | | | | 0.191998 | | | | 4703.344 | | | 10.51563 | |  |  |  |  |  |
| 11.325 | | | 147.9156 | | | | | | | | | | 0.193814 | | | | 4703.297 | | | 10.55469 | |  |  |  |  |  |
| 11.33333 | | | 148.0031 | | | | | | | | | | 0.195785 | | | | 4703.188 | | | 10.375 | |  |  |  |  |  |
| 11.34167 | | | 148.0897 | | | | | | | | | | 0.196355 | | | | 4703.109 | | | 9.984375 | |  |  |  |  |  |
| 11.35 | | | 148.179 | | | | | | | | | | 0.198619 | | | | 4703.023 | | | 9.859375 | |  |  |  |  |  |
| 11.35833 | | | 148.2646 | | | | | | | | | | 0.200795 | | | | 4702.969 | | | 10.14063 | |  |  |  |  |  |
| 11.36667 | | | 148.3521 | | | | | | | | | | 0.201485 | | | | 4702.883 | | | 10 | |  |  |  |  |  |
| 11.375 | | | 148.4414 | | | | | | | | | | 0.203484 | | | | 4702.797 | | | 10.16406 | |  |  |  |  |  |
| 11.38333 | | | 148.528 | | | | | | | | | | 0.205347 | | | | 4702.734 | | | 10.03125 | |  |  |  |  |  |
| 11.39167 | | | 148.6163 | | | | | | | | | | 0.206316 | | | | 4702.703 | | | 10.05469 | |  |  |  |  |  |
| 11.4 | | | 148.7029 | | | | | | | | | | 0.208912 | | | | 4702.641 | | | 10.21094 | |  |  |  |  |  |
| 11.40833 | | | 148.7886 | | | | | | | | | | 0.211286 | | | | 4702.523 | | | 10.15625 | |  |  |  |  |  |
| 11.41667 | | | 148.8743 | | | | | | | | | | 0.212848 | | | | 4702.461 | | | 10.26563 | |  |  |  |  |  |
| 11.425 | | | 148.9599 | | | | | | | | | | 0.215545 | | | | 4702.352 | | | 10.32031 | |  |  |  |  |  |
| 11.43333 | | | 149.0465 | | | | | | | | | | 0.217032 | | | | 4702.273 | | | 10.38281 | |  |  |  |  |  |
| 11.44167 | | | 149.134 | | | | | | | | | | 0.218219 | | | | 4702.18 | | | 10.09375 | |  |  |  |  |  |
| 11.45 | | | 149.2196 | | | | | | | | | | 0.220558 | | | | 4702.086 | | | 10.10156 | |  |  |  |  |  |
| 11.45833 | | | 149.3071 | | | | | | | | | | 0.221975 | | | | 4702 | | | 10.02344 | |  |  |  |  |  |
| 11.46667 | | | 149.3928 | | | | | | | | | | 0.222738 | | | | 4701.898 | | | 10.05469 | |  |  |  |  |  |
| 11.475 | | | 149.4775 | | | | | | | | | | 0.224774 | | | | 4701.789 | | | 9.953125 | |  |  |  |  |  |
| 11.48333 | | | 149.5623 | | | | | | | | | | 0.225961 | | | | 4701.695 | | | 9.882813 | |  |  |  |  |  |
| 11.49167 | | | 149.6498 | | | | | | | | | | 0.226799 | | | | 4701.648 | | | 9.796875 | |  |  |  |  |  |
| 11.5 | | | 149.7381 | | | | | | | | | | 0.228925 | | | | 4701.563 | | | 9.578125 | |  |  |  |  |  |
| 11.50833 | | | 149.8247 | | | | | | | | | | 0.230165 | | | | 4701.492 | | | 9.507813 | |  |  |  |  |  |
| 11.51667 | | | 149.9104 | | | | | | | | | | 0.230894 | | | | 4701.414 | | | 9.578125 | |  |  |  |  |  |
| 11.525 | | | 149.9979 | | | | | | | | | | 0.233083 | | | | 4701.336 | | | 9.46875 | |  |  |  |  |  |
| 11.53333 | | | 150.0853 | | | | | | | | | | 0.234514 | | | | 4701.266 | | | 9.210938 | |  |  |  |  |  |
| 11.54167 | | | 150.1692 | | | | | | | | | | 0.235655 | | | | 4701.203 | | | 9.117188 | |  |  |  |  |  |
| 11.55 | | | 150.2558 | | | | | | | | | | 0.238285 | | | | 4701.148 | | | 9.03125 | |  |  |  |  |  |
| 11.55833 | | | 150.3414 | | | | | | | | | | 0.239627 | | | | 4701.078 | | | 8.859375 | |  |  |  |  |  |
| 11.56667 | | | 150.4316 | | | | | | | | | | 0.240851 | | | | 4700.984 | | | 8.789063 | |  |  |  |  |  |
| 11.575 | | | 150.52 | | | | | | | | | | 0.243034 | | | | 4700.93 | | | 8.640625 | |  |  |  |  |  |
| 11.58333 | | | 150.6084 | | | | | | | | | | 0.243981 | | | | 4700.883 | | | 8.5 | |  |  |  |  |  |
| 11.59167 | | | 150.6976 | | | | | | | | | | 0.244997 | | | | 4700.813 | | | 8.210938 | |  |  |  |  |  |
| 11.6 | | | 150.7878 | | | | | | | | | | 0.247391 | | | | 4700.75 | | | 7.898438 | |  |  |  |  |  |
| 11.60833 | | | 150.8753 | | | | | | | | | | 0.248447 | | | | 4700.703 | | | 7.929688 | |  |  |  |  |  |
| 11.61667 | | | 150.9646 | | | | | | | | | | 0.249825 | | | | 4700.633 | | | 8 | |  |  |  |  |  |
| 11.625 | | | 151.0529 | | | | | | | | | | 0.252167 | | | | 4700.578 | | | 7.804688 | |  |  |  |  |  |
| 11.63333 | | | 151.1431 | | | | | | | | | | 0.253068 | | | | 4700.531 | | | 7.664063 | |  |  |  |  |  |
| 11.64167 | | | 151.236 | | | | | | | | | | 0.254716 | | | | 4700.508 | | | 7.773438 | |  |  |  |  |  |
| 11.65 | | | 151.3226 | | | | | | | | | | 0.256739 | | | | 4700.461 | | | 7.78125 | |  |  |  |  |  |
| 11.65833 | | | 151.41 | | | | | | | | | | 0.257313 | | | | 4700.398 | | | 7.9375 | |  |  |  |  |  |
| 11.66667 | | | 151.4993 | | | | | | | | | | 0.258663 | | | | 4700.313 | | | 7.804688 | |  |  |  |  |  |
| 11.675 | | | 151.5877 | | | | | | | | | | 0.260683 | | | | 4700.273 | | | 7.890625 | |  |  |  |  |  |
| 11.68333 | | | 151.6734 | | | | | | | | | | 0.261008 | | | | 4700.227 | | | 7.789063 | |  |  |  |  |  |
| 11.69167 | | | 151.7635 | | | | | | | | | | 0.262466 | | | | 4700.148 | | | 7.828125 | |  |  |  |  |  |
| 11.7 | | | 151.8519 | | | | | | | | | | 0.264677 | | | | 4700.078 | | | 7.828125 | |  |  |  |  |  |
| 11.70833 | | | 151.9412 | | | | | | | | | | 0.264959 | | | | 4699.992 | | | 7.898438 | |  |  |  |  |  |
| 11.71667 | | | 152.0278 | | | | | | | | | | 0.26623 | | | | 4699.938 | | | 7.835938 | |  |  |  |  |  |
| 11.725 | | | 152.1134 | | | | | | | | | | 0.268227 | | | | 4699.859 | | | 7.492188 | |  |  |  |  |  |
| 11.73333 | | | 152.2018 | | | | | | | | | | 0.268775 | | | | 4699.813 | | | 7.273438 | |  |  |  |  |  |
| 11.74167 | | | 152.2902 | | | | | | | | | | 0.270113 | | | | 4699.742 | | | 7.179688 | |  |  |  |  |  |
| 11.75 | | | 152.3759 | | | | | | | | | | 0.272082 | | | | 4699.68 | | | 7.171875 | |  |  |  |  |  |
| 11.75833 | | | 152.4633 | | | | | | | | | | 0.272561 | | | | 4699.594 | | | 6.929688 | |  |  |  |  |  |
| 11.76667 | | | 152.549 | | | | | | | | | | 0.274271 | | | | 4699.539 | | | 6.789063 | |  |  |  |  |  |
| 11.775 | | | 152.6356 | | | | | | | | | | 0.276317 | | | | 4699.523 | | | 6.710938 | |  |  |  |  |  |
| 11.78333 | | | 152.7212 | | | | | | | | | | 0.276629 | | | | 4699.484 | | | 6.648438 | |  |  |  |  |  |
| 11.79167 | | | 152.8096 | | | | | | | | | | 0.278781 | | | | 4699.438 | | | 6.359375 | |  |  |  |  |  |
| 11.8 | | | 152.8971 | | | | | | | | | | 0.281137 | | | | 4699.383 | | | 6.289063 | |  |  |  |  |  |
| 11.80833 | | | 152.9882 | | | | | | | | | | 0.281598 | | | | 4699.352 | | | 6.304688 | |  |  |  |  |  |
| 11.81667 | | | 153.0747 | | | | | | | | | | 0.283542 | | | | 4699.305 | | | 6.257813 | |  |  |  |  |  |
| 11.825 | | | 153.1631 | | | | | | | | | | 0.28591 | | | | 4699.25 | | | 5.992188 | |  |  |  |  |  |
| 11.83333 | | | 153.2515 | | | | | | | | | | 0.286687 | | | | 4699.211 | | | 5.726563 | |  |  |  |  |  |
| 11.84167 | | | 153.3363 | | | | | | | | | | 0.28891 | | | | 4699.195 | | | 5.828125 | |  |  |  |  |  |
| 11.85 | | | 153.4273 | | | | | | | | | | 0.291417 | | | | 4699.148 | | | 5.859375 | |  |  |  |  |  |
| 11.85833 | | | 153.5139 | | | | | | | | | | 0.29196 | | | | 4699.094 | | | 5.835938 | |  |  |  |  |  |
| 11.86667 | | | 153.6059 | | | | | | | | | | 0.294233 | | | | 4699.047 | | | 5.726563 | |  |  |  |  |  |
| 11.875 | | | 153.6961 | | | | | | | | | | 0.296034 | | | | 4699.031 | | | 5.90625 | |  |  |  |  |  |
| 11.88333 | | | 153.7835 | | | | | | | | | | 0.296267 | | | | 4699.016 | | | 6.039063 | |  |  |  |  |  |
| 11.89167 | | | 153.8674 | | | | | | | | | | 0.297826 | | | | 4698.945 | | | 6.148438 | |  |  |  |  |  |
| 11.9 | | | 153.9576 | | | | | | | | | | 0.299876 | | | | 4698.891 | | | 6.414063 | |  |  |  |  |  |
| 11.90833 | | | 154.0442 | | | | | | | | | | 0.300433 | | | | 4698.844 | | | 6.804688 | |  |  |  |  |  |
| 11.91667 | | | 154.1316 | | | | | | | | | | 0.302188 | | | | 4698.813 | | | 7.171875 | |  |  |  |  |  |
| 11.925 | | | 154.2227 | | | | | | | | | | 0.304199 | | | | 4698.727 | | | 7.203125 | |  |  |  |  |  |
| 11.93333 | | | 154.3075 | | | | | | | | | | 0.304521 | | | | 4698.656 | | | 7.484375 | |  |  |  |  |  |
| 11.94167 | | | 154.3958 | | | | | | | | | | 0.306581 | | | | 4698.586 | | | 7.484375 | |  |  |  |  |  |
| 11.95 | | | 154.4806 | | | | | | | | | | 0.308311 | | | | 4698.508 | | | 7.898438 | |  |  |  |  |  |
| 11.95833 | | | 154.5636 | | | | | | | | | | 0.30841 | | | | 4698.391 | | | 7.882813 | |  |  |  |  |  |
| 11.96667 | | | 154.6538 | | | | | | | | | | 0.309721 | | | | 4698.273 | | | 7.90625 | |  |  |  |  |  |
| 11.975 | | | 154.7439 | | | | | | | | | | 0.311274 | | | | 4698.203 | | | 8.007813 | |  |  |  |  |  |
| 11.98333 | | | 154.8305 | | | | | | | | | | 0.311629 | | | | 4698.109 | | | 8.039063 | |  |  |  |  |  |
| 11.99167 | | | 154.918 | | | | | | | | | | 0.313121 | | | | 4698.047 | | | 7.828125 | |  |  |  |  |  |
| 12 | | | 155.0045 | | | | | | | | | | 0.314754 | | | | 4697.938 | | | 7.742188 | |  |  |  |  |  |
| 12.00833 | | | 155.0929 | | | | | | | | | | 0.315372 | | | | 4697.867 | | | 7.726563 | |  |  |  |  |  |
| 12.01667 | | | 155.184 | | | | | | | | | | 0.317048 | | | | 4697.797 | | | 7.484375 | |  |  |  |  |  |
| 12.025 | | | 155.2733 | | | | | | | | | | 0.318698 | | | | 4697.719 | | | 7.320313 | |  |  |  |  |  |
| 12.03333 | | | 155.3625 | | | | | | | | | | 0.318964 | | | | 4697.648 | | | 7.289063 | |  |  |  |  |  |
| 12.04167 | | | 155.4518 | | | | | | | | | | 0.321027 | | | | 4697.609 | | | 7.078125 | |  |  |  |  |  |
| 12.05 | | | 155.5389 | | | | | | | | | | 0.322624 | | | | 4697.563 | | | 6.875 | |  |  |  |  |  |
| 12.05833 | | | 155.6244 | | | | | | | | | | 0.323017 | | | | 4697.492 | | | 6.804688 | |  |  |  |  |  |
| 12.06667 | | | 155.7125 | | | | | | | | | | 0.325225 | | | | 4697.461 | | | 6.601563 | |  |  |  |  |  |
| 12.075 | | | 155.8006 | | | | | | | | | | 0.326424 | | | | 4697.422 | | | 6.617188 | |  |  |  |  |  |
| 12.08333 | | | 155.8906 | | | | | | | | | | 0.326391 | | | | 4697.367 | | | 6.570313 | |  |  |  |  |  |
| 12.09167 | | | 155.9778 | | | | | | | | | | 0.328586 | | | | 4697.336 | | | 6.585938 | |  |  |  |  |  |
| 12.1 | | | 156.0632 | | | | | | | | | | 0.329242 | | | | 4697.305 | | | 6.554688 | |  |  |  |  |  |
| 12.10833 | | | 156.1496 | | | | | | | | | | 0.329104 | | | | 4697.258 | | | 6.78125 | |  |  |  |  |  |
| 12.11667 | | | 156.2351 | | | | | | | | | | 0.331524 | | | | 4697.234 | | | 6.804688 | |  |  |  |  |  |
| 12.125 | | | 156.3259 | | | | | | | | | | 0.332586 | | | | 4697.18 | | | 6.820313 | |  |  |  |  |  |
| 12.13333 | | | 156.4149 | | | | | | | | | | 0.332775 | | | | 4697.141 | | | 6.9375 | |  |  |  |  |  |
| 12.14167 | | | 156.503 | | | | | | | | | | 0.335205 | | | | 4697.078 | | | 6.789063 | |  |  |  |  |  |
| 12.15 | | | 156.5911 | | | | | | | | | | 0.336179 | | | | 4697.031 | | | 6.5625 | |  |  |  |  |  |
| 12.15833 | | | 156.6802 | | | | | | | | | | 0.336562 | | | | 4696.945 | | | 6.65625 | |  |  |  |  |  |
| 12.16667 | | | 156.7665 | | | | | | | | | | 0.339382 | | | | 4696.883 | | | 6.570313 | |  |  |  |  |  |
| 12.175 | | | 156.8537 | | | | | | | | | | 0.340104 | | | | 4696.82 | | | 6.414063 | |  |  |  |  |  |
| 12.18333 | | | 156.9428 | | | | | | | | | | 0.339971 | | | | 4696.758 | | | 6.46875 | |  |  |  |  |  |
| 12.19167 | | | 157.0327 | | | | | | | | | | 0.342517 | | | | 4696.719 | | | 6.359375 | |  |  |  |  |  |
| 12.2 | | | 157.1217 | | | | | | | | | | 0.342824 | | | | 4696.695 | | | 5.992188 | |  |  |  |  |  |
| 12.20833 | | | 157.2063 | | | | | | | | | | 0.342701 | | | | 4696.633 | | | 5.8125 | |  |  |  |  |  |
| 12.21667 | | | 157.2917 | | | | | | | | | | 0.345166 | | | | 4696.594 | | | 5.820313 | |  |  |  |  |  |
| 12.225 | | | 157.379 | | | | | | | | | | 0.345684 | | | | 4696.563 | | | 5.921875 | |  |  |  |  |  |
| 12.23333 | | | 157.4662 | | | | | | | | | | 0.345979 | | | | 4696.508 | | | 6.257813 | |  |  |  |  |  |
| 12.24167 | | | 157.5552 | | | | | | | | | | 0.348294 | | | | 4696.469 | | | 6.328125 | |  |  |  |  |  |
| 12.25 | | | 157.6451 | | | | | | | | | | 0.348673 | | | | 4696.469 | | | 6.210938 | |  |  |  |  |  |
| 12.25833 | | | 157.7342 | | | | | | | | | | 0.3485 | | | | 4696.445 | | | 6.109375 | |  |  |  |  |  |
| 12.26667 | | | 157.8223 | | | | | | | | | | 0.351066 | | | | 4696.398 | | | 6.078125 | |  |  |  |  |  |
| 12.275 | | | 157.9077 | | | | | | | | | | 0.35136 | | | | 4696.328 | | | 6.007813 | |  |  |  |  |  |
| 12.28333 | | | 157.995 | | | | | | | | | | 0.351626 | | | | 4696.227 | | | 6.132813 | |  |  |  |  |  |
| 12.29167 | | | 158.0849 | | | | | | | | | | 0.354278 | | | | 4696.164 | | | 6.289063 | |  |  |  |  |  |
| 12.3 | | | 158.1739 | | | | | | | | | | 0.354746 | | | | 4696.125 | | | 6.234375 | |  |  |  |  |  |
| 12.30833 | | | 158.262 | | | | | | | | | | 0.355356 | | | | 4696.07 | | | 6.164063 | |  |  |  |  |  |
| 12.31667 | | | 158.3493 | | | | | | | | | | 0.357832 | | | | 4696.023 | | | 5.96875 | |  |  |  |  |  |
| 12.325 | | | 158.4356 | | | | | | | | | | 0.358233 | | | | 4695.977 | | | 5.617188 | |  |  |  |  |  |
| 12.33333 | | | 158.5229 | | | | | | | | | | 0.358567 | | | | 4695.906 | | | 5.445313 | |  |  |  |  |  |
| 12.34167 | | | 158.6101 | | | | | | | | | | 0.361424 | | | | 4695.828 | | | 5.296875 | |  |  |  |  |  |
| 12.35 | | | 158.6973 | | | | | | | | | | 0.361755 | | | | 4695.781 | | | 5.046875 | |  |  |  |  |  |
| 12.35833 | | | 158.7855 | | | | | | | | | | 0.362318 | | | | 4695.734 | | | 5.015625 | |  |  |  |  |  |
| 12.36667 | | | 158.8754 | | | | | | | | | | 0.365178 | | | | 4695.719 | | | 5.070313 | |  |  |  |  |  |
| 12.375 | | | 158.9608 | | | | | | | | | | 0.365485 | | | | 4695.711 | | | 4.882813 | |  |  |  |  |  |
| 12.38333 | | | 159.0463 | | | | | | | | | | 0.365881 | | | | 4695.695 | | | 4.726563 | |  |  |  |  |  |
| 12.39167 | | | 159.1335 | | | | | | | | | | 0.368319 | | | | 4695.664 | | | 4.820313 | |  |  |  |  |  |
| 12.4 | | | 159.2217 | | | | | | | | | | 0.368684 | | | | 4695.656 | | | 4.9375 | |  |  |  |  |  |
| 12.40833 | | | 159.308 | | | | | | | | | | 0.369184 | | | | 4695.609 | | | 5.101563 | |  |  |  |  |  |
| 12.41667 | | | 159.397 | | | | | | | | | | 0.372215 | | | | 4695.57 | | | 5.289063 | |  |  |  |  |  |
| 12.425 | | | 159.4843 | | | | | | | | | | 0.372722 | | | | 4695.555 | | | 5.382813 | |  |  |  |  |  |
| 12.43333 | | | 159.5724 | | | | | | | | | | 0.373735 | | | | 4695.531 | | | 5.429688 | |  |  |  |  |  |
| 12.44167 | | | 159.6614 | | | | | | | | | | 0.376576 | | | | 4695.477 | | | 5.085938 | |  |  |  |  |  |
| 12.45 | | | 159.7505 | | | | | | | | | | 0.376935 | | | | 4695.43 | | | 5.1875 | |  |  |  |  |  |
| 12.45833 | | | 159.8386 | | | | | | | | | | 0.377972 | | | | 4695.359 | | | 5.34375 | |  |  |  |  |  |
| 12.46667 | | | 159.9267 | | | | | | | | | | 0.38123 | | | | 4695.289 | | | 5.773438 | |  |  |  |  |  |
| 12.475 | | | 160.0148 | | | | | | | | | | 0.381722 | | | | 4695.234 | | | 5.515625 | |  |  |  |  |  |
| 12.48333 | | | 160.1021 | | | | | | | | | | 0.382858 | | | | 4695.18 | | | 5.53125 | |  |  |  |  |  |
| 12.49167 | | | 160.192 | | | | | | | | | | 0.385988 | | | | 4695.18 | | | 5.570313 | |  |  |  |  |  |
| 12.5 | | | 160.2775 | | | | | | | | | | 0.386011 | | | | 4695.133 | | | 5.75 | |  |  |  |  |  |
| 12.50833 | | | 160.3665 | | | | | | | | | | 0.387106 | | | | 4695.055 | | | 5.609375 | |  |  |  |  |  |
| 12.51667 | | | 160.4564 | | | | | | | | | | 0.389633 | | | | 4694.945 | | | 5.929688 | |  |  |  |  |  |
| 12.525 | | | 160.5445 | | | | | | | | | | 0.389498 | | | | 4694.93 | | | 6.226563 | |  |  |  |  |  |
| 12.53333 | | | 160.6326 | | | | | | | | | | 0.390637 | | | | 4694.875 | | | 6.085938 | |  |  |  |  |  |
| 12.54167 | | | 160.7216 | | | | | | | | | | 0.392683 | | | | 4694.828 | | | 5.992188 | |  |  |  |  |  |
| 12.55 | | | 160.8133 | | | | | | | | | | 0.392582 | | | | 4694.758 | | | 5.804688 | |  |  |  |  |  |
| 12.55833 | | | 160.9024 | | | | | | | | | | 0.393924 | | | | 4694.727 | | | 5.773438 | |  |  |  |  |  |
| 12.56667 | | | 160.9949 | | | | | | | | | | 0.39619 | | | | 4694.625 | | | 5.90625 | |  |  |  |  |  |
| 12.575 | | | 161.0804 | | | | | | | | | | 0.396308 | | | | 4694.547 | | | 6.054688 | |  |  |  |  |  |
| 12.58333 | | | 161.1694 | | | | | | | | | | 0.397854 | | | | 4694.508 | | | 5.8125 | |  |  |  |  |  |
| 12.59167 | | | 161.2584 | | | | | | | | | | 0.399955 | | | | 4694.484 | | | 5.96875 | |  |  |  |  |  |
| 12.6 | | | 161.3448 | | | | | | | | | | 0.399982 | | | | 4694.461 | | | 5.78125 | |  |  |  |  |  |
| 12.60833 | | | 161.4329 | | | | | | | | | | 0.401827 | | | | 4694.414 | | | 5.804688 | |  |  |  |  |  |
| 12.61667 | | | 161.5184 | | | | | | | | | | 0.403897 | | | | 4694.344 | | | 5.914063 | |  |  |  |  |  |
| 12.625 | | | 161.6065 | | | | | | | | | | 0.403817 | | | | 4694.281 | | | 5.796875 | |  |  |  |  |  |
| 12.63333 | | | 161.6938 | | | | | | | | | | 0.405281 | | | | 4694.25 | | | 5.757813 | |  |  |  |  |  |
| 12.64167 | | | 161.7828 | | | | | | | | | | 0.407399 | | | | 4694.18 | | | 5.679688 | |  |  |  |  |  |
| 12.65 | | | 161.8682 | | | | | | | | | | 0.406952 | | | | 4694.164 | | | 5.460938 | |  |  |  |  |  |
| 12.65833 | | | 161.9572 | | | | | | | | | | 0.407931 | | | | 4694.109 | | | 5.460938 | |  |  |  |  |  |
| 12.66667 | | | 162.0454 | | | | | | | | | | 0.409925 | | | | 4694.039 | | | 5.398438 | |  |  |  |  |  |
| 12.675 | | | 162.1291 | | | | | | | | | | 0.409311 | | | | 4694.008 | | | 5.570313 | |  |  |  |  |  |
| 12.68333 | | | 162.2163 | | | | | | | | | | 0.410501 | | | | 4693.969 | | | 5.515625 | |  |  |  |  |  |
| 12.69167 | | | 162.3035 | | | | | | | | | | 0.412135 | | | | 4693.93 | | | 5.507813 | |  |  |  |  |  |
| 12.7 | | | 162.3908 | | | | | | | | | | 0.41174 | | | | 4693.906 | | | 5.296875 | |  |  |  |  |  |
| 12.70833 | | | 162.4816 | | | | | | | | | | 0.413339 | | | | 4693.867 | | | 5.476563 | |  |  |  |  |  |
| 12.71667 | | | 162.5724 | | | | | | | | | | 0.415458 | | | | 4693.836 | | | 5.5 | |  |  |  |  |  |
| 12.725 | | | 162.6605 | | | | | | | | | | 0.415175 | | | | 4693.766 | | | 5.554688 | |  |  |  |  |  |
| 12.73333 | | | 162.7477 | | | | | | | | | | 0.416601 | | | | 4693.734 | | | 5.320313 | |  |  |  |  |  |
| 12.74167 | | | 162.8359 | | | | | | | | | | 0.418587 | | | | 4693.688 | | | 5.164063 | |  |  |  |  |  |
| 12.75 | | | 162.9249 | | | | | | | | | | 0.418056 | | | | 4693.68 | | | 5.148438 | |  |  |  |  |  |
| 12.75833 | | | 163.0184 | | | | | | | | | | 0.419734 | | | | 4693.602 | | | 4.828125 | |  |  |  |  |  |
| 12.76667 | | | 163.1074 | | | | | | | | | | 0.421811 | | | | 4693.563 | | | 4.914063 | |  |  |  |  |  |
| 12.775 | | | 163.1973 | | | | | | | | | | 0.421863 | | | | 4693.508 | | | 4.796875 | |  |  |  |  |  |
| 12.78333 | | | 163.2908 | | | | | | | | | | 0.424029 | | | | 4693.5 | | | 4.851563 | |  |  |  |  |  |
| 12.79167 | | | 163.378 | | | | | | | | | | 0.425599 | | | | 4693.477 | | | 4.726563 | |  |  |  |  |  |
| 12.8 | | | 163.4644 | | | | | | | | | | 0.42533 | | | | 4693.445 | | | 4.695313 | |  |  |  |  |  |
| 12.80833 | | | 163.5543 | | | | | | | | | | 0.426671 | | | | 4693.438 | | | 4.65625 | |  |  |  |  |  |
| 12.81667 | | | 163.6433 | | | | | | | | | | 0.428031 | | | | 4693.391 | | | 4.710938 | |  |  |  |  |  |
| 12.825 | | | 163.7341 | | | | | | | | | | 0.428022 | | | | 4693.359 | | | 4.632813 | |  |  |  |  |  |
| 12.83333 | | | 163.824 | | | | | | | | | | 0.429775 | | | | 4693.313 | | | 4.695313 | |  |  |  |  |  |
| 12.84167 | | | 163.9121 | | | | | | | | | | 0.431161 | | | | 4693.289 | | | 4.554688 | |  |  |  |  |  |
| 12.85 | | | 164.0011 | | | | | | | | | | 0.431366 | | | | 4693.258 | | | 4.546875 | |  |  |  |  |  |
| 12.85833 | | | 164.0875 | | | | | | | | | | 0.43299 | | | | 4693.219 | | | 4.328125 | |  |  |  |  |  |
| 12.86667 | | | 164.1712 | | | | | | | | | | 0.433919 | | | | 4693.164 | | | 4.234375 | |  |  |  |  |  |
| 12.875 | | | 164.2602 | | | | | | | | | | 0.433367 | | | | 4693.133 | | | 4.085938 | |  |  |  |  |  |
| 12.88333 | | | 164.3492 | | | | | | | | | | 0.434763 | | | | 4693.086 | | | 4.101563 | |  |  |  |  |  |
| 12.89167 | | | 164.4329 | | | | | | | | | | 0.435699 | | | | 4693.063 | | | 3.914063 | |  |  |  |  |  |
| 12.9 | | | 164.5246 | | | | | | | | | | 0.435526 | | | | 4693.031 | | | 3.921875 | |  |  |  |  |  |
| 12.90833 | | | 164.6145 | | | | | | | | | | 0.437417 | | | | 4693.023 | | | 4.070313 | |  |  |  |  |  |
| 12.91667 | | | 164.7008 | | | | | | | | | | 0.438738 | | | | 4692.984 | | | 4.234375 | |  |  |  |  |  |
| 12.925 | | | 164.789 | | | | | | | | | | 0.438554 | | | | 4692.977 | | | 4.320313 | |  |  |  |  |  |
| 12.93333 | | | 164.8798 | | | | | | | | | | 0.440423 | | | | 4692.938 | | | 4.140625 | |  |  |  |  |  |
| 12.94167 | | | 164.9706 | | | | | | | | | | 0.44175 | | | | 4692.93 | | | 4.132813 | |  |  |  |  |  |
| 12.95 | | | 165.0623 | | | | | | | | | | 0.441941 | | | | 4692.898 | | | 4.039063 | |  |  |  |  |  |
| 12.95833 | | | 165.1513 | | | | | | | | | | 0.444 | | | | 4692.836 | | | 4.015625 | |  |  |  |  |  |
| 12.96667 | | | 165.2358 | | | | | | | | | | 0.445335 | | | | 4692.781 | | | 4.070313 | |  |  |  |  |  |
| 12.975 | | | 165.3271 | | | | | | | | | | 0.445941 | | | | 4692.719 | | | 4.007813 | |  |  |  |  |  |
| 12.98333 | | | 165.4119 | | | | | | | | | | 0.448433 | | | | 4692.719 | | | 3.773438 | |  |  |  |  |  |
| 12.99167 | | | 165.4985 | | | | | | | | | | 0.44997 | | | | 4692.68 | | | 3.53125 | |  |  |  |  |  |
| 13 | | | 165.5851 | | | | | | | | | | 0.450417 | | | | 4692.664 | | | 3.445313 | |  |  |  |  |  |
| 13.00833 | | | 165.6716 | | | | | | | | | | 0.452419 | | | | 4692.633 | | | 3.101563 | |  |  |  |  |  |
| 13.01667 | | | 165.7564 | | | | | | | | | | 0.453132 | | | | 4692.586 | | | 3.171875 | |  |  |  |  |  |
| 13.025 | | | 165.8456 | | | | | | | | | | 0.453496 | | | | 4692.555 | | | 3.070313 | |  |  |  |  |  |
| 13.03333 | | | 165.9322 | | | | | | | | | | 0.45544 | | | | 4692.563 | | | 3.34375 | |  |  |  |  |  |
| 13.04167 | | | 166.0196 | | | | | | | | | | 0.456518 | | | | 4692.555 | | | 3.304688 | |  |  |  |  |  |
| 13.05 | | | 166.1123 | | | | | | | | | | 0.457478 | | | | 4692.547 | | | 3.265625 | |  |  |  |  |  |
| 13.05833 | | | 166.2006 | | | | | | | | | | 0.459727 | | | | 4692.57 | | | 3.421875 | |  |  |  |  |  |
| 13.06667 | | | 166.2907 | | | | | | | | | | 0.460488 | | | | 4692.539 | | | 3.734375 | |  |  |  |  |  |
| 13.075 | | | 166.3755 | | | | | | | | | | 0.460935 | | | | 4692.508 | | | 3.796875 | |  |  |  |  |  |
| 13.08333 | | | 166.4682 | | | | | | | | | | 0.463103 | | | | 4692.445 | | | 3.914063 | |  |  |  |  |  |
| 13.09167 | | | 166.5583 | | | | | | | | | | 0.463792 | | | | 4692.422 | | | 4.171875 | |  |  |  |  |  |
| 13.1 | | | 166.6475 | | | | | | | | | | 0.464815 | | | | 4692.398 | | | 4.328125 | |  |  |  |  |  |
| 13.10833 | | | 166.7358 | | | | | | | | | | 0.466905 | | | | 4692.344 | | | 4.304688 | |  |  |  |  |  |
| 13.11667 | | | 166.8267 | | | | | | | | | | 0.46759 | | | | 4692.273 | | | 3.976563 | |  |  |  |  |  |
| 13.125 | | | 166.915 | | | | | | | | | | 0.468908 | | | | 4692.227 | | | 4.03125 | |  |  |  |  |  |
| 13.13333 | | | 166.9999 | | | | | | | | | | 0.470781 | | | | 4692.18 | | | 3.921875 | |  |  |  |  |  |
| 13.14167 | | | 167.0864 | | | | | | | | | | 0.471069 | | | | 4692.109 | | | 3.734375 | |  |  |  |  |  |
| 13.15 | | | 167.1721 | | | | | | | | | | 0.472087 | | | | 4692.047 | | | 3.695313 | |  |  |  |  |  |
| 13.15833 | | | 167.2639 | | | | | | | | | | 0.473859 | | | | 4692.023 | | | 3.539063 | |  |  |  |  |  |
| 13.16667 | | | 167.3522 | | | | | | | | | | 0.473754 | | | | 4692.031 | | | 3.5 | |  |  |  |  |  |
| 13.175 | | | 167.4397 | | | | | | | | | | 0.474702 | | | | 4692 | | | 3.601563 | |  |  |  |  |  |
| 13.18333 | | | 167.5245 | | | | | | | | | | 0.476025 | | | | 4691.977 | | | 3.671875 | |  |  |  |  |  |
| 13.19167 | | | 167.6111 | | | | | | | | | | 0.475993 | | | | 4691.969 | | | 3.78125 | |  |  |  |  |  |
| 13.2 | | | 167.6985 | | | | | | | | | | 0.476902 | | | | 4691.945 | | | 3.859375 | |  |  |  |  |  |
| 13.20833 | | | 167.786 | | | | | | | | | | 0.478256 | | | | 4691.938 | | | 4.015625 | |  |  |  |  |  |
| 13.21667 | | | 167.8743 | | | | | | | | | | 0.478315 | | | | 4691.914 | | | 4.023438 | |  |  |  |  |  |
| 13.225 | | | 167.9617 | | | | | | | | | | 0.479135 | | | | 4691.859 | | | 4.289063 | |  |  |  |  |  |
| 13.23333 | | | 168.0509 | | | | | | | | | | 0.480999 | | | | 4691.82 | | | 4.359375 | |  |  |  |  |  |
| 13.24167 | | | 168.1384 | | | | | | | | | | 0.481265 | | | | 4691.773 | | | 4.570313 | |  |  |  |  |  |
| 13.25 | | | 168.2206 | | | | | | | | | | 0.482135 | | | | 4691.727 | | | 4.59375 | |  |  |  |  |  |
| 13.25833 | | | 168.308 | | | | | | | | | | 0.484216 | | | | 4691.672 | | | 4.421875 | |  |  |  |  |  |
| 13.26667 | | | 168.4016 | | | | | | | | | | 0.485136 | | | | 4691.641 | | | 4.445313 | |  |  |  |  |  |
| 13.275 | | | 168.4925 | | | | | | | | | | 0.486698 | | | | 4691.578 | | | 4.296875 | |  |  |  |  |  |
| 13.28333 | | | 168.5808 | | | | | | | | | | 0.488669 | | | | 4691.523 | | | 4.398438 | |  |  |  |  |  |
| 13.29167 | | | 168.6691 | | | | | | | | | | 0.489228 | | | | 4691.461 | | | 4.148438 | |  |  |  |  |  |
| 13.3 | | | 168.7574 | | | | | | | | | | 0.490812 | | | | 4691.422 | | | 4.109375 | |  |  |  |  |  |
| 13.30833 | | | 168.8493 | | | | | | | | | | 0.492528 | | | | 4691.406 | | | 4.015625 | |  |  |  |  |  |
| 13.31667 | | | 168.9358 | | | | | | | | | | 0.492585 | | | | 4691.359 | | | 3.8125 | |  |  |  |  |  |
| 13.325 | | | 169.0241 | | | | | | | | | | 0.494031 | | | | 4691.336 | | | 3.648438 | |  |  |  |  |  |
| 13.33333 | | | 169.116 | | | | | | | | | | 0.495777 | | | | 4691.305 | | | 3.546875 | |  |  |  |  |  |
| 13.34167 | | | 169.2008 | | | | | | | | | | 0.495753 | | | | 4691.305 | | | 3.445313 | |  |  |  |  |  |
| 13.35 | | | 169.2856 | | | | | | | | | | 0.496775 | | | | 4691.266 | | | 3.382813 | |  |  |  |  |  |
| 13.35833 | | | 169.3713 | | | | | | | | | | 0.498249 | | | | 4691.258 | | | 3.539063 | |  |  |  |  |  |
| 13.36667 | | | 169.457 | | | | | | | | | | 0.498515 | | | | 4691.242 | | | 3.492188 | |  |  |  |  |  |
| 13.375 | | | 169.5453 | | | | | | | | | | 0.499827 | | | | 4691.234 | | | 3.734375 | |  |  |  |  |  |
| 13.38333 | | | 169.6354 | | | | | | | | | | 0.501383 | | | | 4691.227 | | | 3.671875 | |  |  |  |  |  |
| 13.39167 | | | 169.7219 | | | | | | | | | | 0.50146 | | | | 4691.211 | | | 3.71875 | |  |  |  |  |  |
| 13.4 | | | 169.8102 | | | | | | | | | | 0.502952 | | | | 4691.203 | | | 3.578125 | |  |  |  |  |  |
| 13.40833 | | | 169.8994 | | | | | | | | | | 0.504751 | | | | 4691.156 | | | 3.617188 | |  |  |  |  |  |
| 13.41667 | | | 169.986 | | | | | | | | | | 0.50525 | | | | 4691.133 | | | 3.710938 | |  |  |  |  |  |
| 13.425 | | | 170.0813 | | | | | | | | | | 0.507282 | | | | 4691.07 | | | 3.546875 | |  |  |  |  |  |
| 13.43333 | | | 170.1705 | | | | | | | | | | 0.508906 | | | | 4691.039 | | | 3.375 | |  |  |  |  |  |
| 13.44167 | | | 170.2553 | | | | | | | | | | 0.509127 | | | | 4691 | | | 3.28125 | |  |  |  |  |  |
| 13.45 | | | 170.3454 | | | | | | | | | | 0.510856 | | | | 4690.992 | | | 3.460938 | |  |  |  |  |  |
| 13.45833 | | | 170.4355 | | | | | | | | | | 0.512097 | | | | 4690.953 | | | 3.367188 | |  |  |  |  |  |
| 13.46667 | | | 170.5229 | | | | | | | | | | 0.512022 | | | | 4690.906 | | | 3.390625 | |  |  |  |  |  |
| 13.475 | | | 170.6103 | | | | | | | | | | 0.513427 | | | | 4690.898 | | | 3.34375 | |  |  |  |  |  |
| 13.48333 | | | 170.6978 | | | | | | | | | | 0.514472 | | | | 4690.891 | | | 3.632813 | |  |  |  |  |  |
| 13.49167 | | | 170.787 | | | | | | | | | | 0.514329 | | | | 4690.867 | | | 3.75 | |  |  |  |  |  |
| 13.5 | | | 170.8762 | | | | | | | | | | 0.515796 | | | | 4690.813 | | | 3.859375 | |  |  |  |  |  |
| 13.50833 | | | 170.9601 | | | | | | | | | | 0.516879 | | | | 4690.797 | | | 4.132813 | |  |  |  |  |  |
| 13.51667 | | | 171.0484 | | | | | | | | | | 0.517171 | | | | 4690.773 | | | 4.109375 | |  |  |  |  |  |
| 13.525 | | | 171.1394 | | | | | | | | | | 0.518892 | | | | 4690.75 | | | 3.992188 | |  |  |  |  |  |
| 13.53333 | | | 171.2286 | | | | | | | | | | 0.52009 | | | | 4690.672 | | | 3.84375 | |  |  |  |  |  |
| 13.54167 | | | 171.3151 | | | | | | | | | | 0.520511 | | | | 4690.633 | | | 3.890625 | |  |  |  |  |  |
| 13.55 | | | 171.4026 | | | | | | | | | | 0.522585 | | | | 4690.594 | | | 4 | |  |  |  |  |  |
| 13.55833 | | | 171.4944 | | | | | | | | | | 0.524227 | | | | 4690.516 | | | 3.992188 | |  |  |  |  |  |
| 13.56667 | | | 171.5853 | | | | | | | | | | 0.524971 | | | | 4690.484 | | | 3.9375 | |  |  |  |  |  |
| 13.575 | | | 171.6719 | | | | | | | | | | 0.527342 | | | | 4690.469 | | | 3.789063 | |  |  |  |  |  |
| 13.58333 | | | 171.7619 | | | | | | | | | | 0.528269 | | | | 4690.453 | | | 3.625 | |  |  |  |  |  |
| 13.59167 | | | 171.8529 | | | | | | | | | | 0.528751 | | | | 4690.414 | | | 3.773438 | |  |  |  |  |  |
| 13.6 | | | 171.9438 | | | | | | | | | | 0.531546 | | | | 4690.352 | | | 3.6875 | |  |  |  |  |  |
| 13.60833 | | | 172.033 | | | | | | | | | | 0.531865 | | | | 4690.328 | | | 3.734375 | |  |  |  |  |  |
| 13.61667 | | | 172.1187 | | | | | | | | | | 0.531929 | | | | 4690.305 | | | 3.726563 | |  |  |  |  |  |
| 13.625 | | | 172.2062 | | | | | | | | | | 0.534139 | | | | 4690.289 | | | 3.796875 | |  |  |  |  |  |
| 13.63333 | | | 172.2971 | | | | | | | | | | 0.53414 | | | | 4690.273 | | | 3.507813 | |  |  |  |  |  |
| 13.64167 | | | 172.3819 | | | | | | | | | | 0.534274 | | | | 4690.219 | | | 3.382813 | |  |  |  |  |  |
| 13.65 | | | 172.4694 | | | | | | | | | | 0.536257 | | | | 4690.203 | | | 3.289063 | |  |  |  |  |  |
| 13.65833 | | | 172.5585 | | | | | | | | | | 0.536422 | | | | 4690.172 | | | 3.375 | |  |  |  |  |  |
| 13.66667 | | | 172.6469 | | | | | | | | | | 0.536725 | | | | 4690.141 | | | 3.375 | |  |  |  |  |  |
| 13.675 | | | 172.7343 | | | | | | | | | | 0.538746 | | | | 4690.094 | | | 3.179688 | |  |  |  |  |  |
| 13.68333 | | | 172.8252 | | | | | | | | | | 0.538984 | | | | 4690.109 | | | 3.320313 | |  |  |  |  |  |
| 13.69167 | | | 172.9153 | | | | | | | | | | 0.539237 | | | | 4690.094 | | | 3.335938 | |  |  |  |  |  |
| 13.7 | | | 173.0054 | | | | | | | | | | 0.541558 | | | | 4690.086 | | | 3.335938 | |  |  |  |  |  |
| 13.70833 | | | 173.0963 | | | | | | | | | | 0.542224 | | | | 4690.047 | | | 3.34375 | |  |  |  |  |  |
| 13.71667 | | | 173.1838 | | | | | | | | | | 0.543174 | | | | 4690.016 | | | 3.453125 | |  |  |  |  |  |
| 13.725 | | | 173.2756 | | | | | | | | | | 0.545471 | | | | 4690.016 | | | 3.34375 | |  |  |  |  |  |
| 13.73333 | | | 173.3665 | | | | | | | | | | 0.545385 | | | | 4689.977 | | | 3.398438 | |  |  |  |  |  |
| 13.74167 | | | 173.4539 | | | | | | | | | | 0.546347 | | | | 4689.945 | | | 3.195313 | |  |  |  |  |  |
| 13.75 | | | 173.5432 | | | | | | | | | | 0.548326 | | | | 4689.922 | | | 3.1875 | |  |  |  |  |  |
| 13.75833 | | | 173.6341 | | | | | | | | | | 0.548041 | | | | 4689.891 | | | 3.023438 | |  |  |  |  |  |
| 13.76667 | | | 173.7198 | | | | | | | | | | 0.548553 | | | | 4689.844 | | | 2.960938 | |  |  |  |  |  |
| 13.775 | | | 173.8046 | | | | | | | | | | 0.550605 | | | | 4689.828 | | | 2.867188 | |  |  |  |  |  |
| 13.78333 | | | 173.8938 | | | | | | | | | | 0.550557 | | | | 4689.789 | | | 2.921875 | |  |  |  |  |  |
| 13.79167 | | | 173.9812 | | | | | | | | | | 0.551328 | | | | 4689.797 | | | 2.695313 | |  |  |  |  |  |
| 13.8 | | | 174.0704 | | | | | | | | | | 0.553259 | | | | 4689.766 | | | 2.617188 | |  |  |  |  |  |
| 13.80833 | | | 174.1579 | | | | | | | | | | 0.553042 | | | | 4689.766 | | | 2.523438 | |  |  |  |  |  |
| 13.81667 | | | 174.2462 | | | | | | | | | | 0.553776 | | | | 4689.742 | | | 2.429688 | |  |  |  |  |  |
| 13.825 | | | 174.338 | | | | | | | | | | 0.555611 | | | | 4689.742 | | | 2.59375 | |  |  |  |  |  |
| 13.83333 | | | 174.4263 | | | | | | | | | | 0.555479 | | | | 4689.703 | | | 2.351563 | |  |  |  |  |  |
| 13.84167 | | | 174.5129 | | | | | | | | | | 0.556392 | | | | 4689.719 | | | 2.523438 | |  |  |  |  |  |
| 13.85 | | | 174.6029 | | | | | | | | | | 0.558314 | | | | 4689.703 | | | 2.539063 | |  |  |  |  |  |
| 13.85833 | | | 174.6965 | | | | | | | | | | 0.558214 | | | | 4689.695 | | | 2.59375 | |  |  |  |  |  |
| 13.86667 | | | 174.7848 | | | | | | | | | | 0.559244 | | | | 4689.688 | | | 2.46875 | |  |  |  |  |  |
| 13.875 | | | 174.8745 | | | | | | | | | | 0.561729 | | | | 4689.648 | | | 2.539063 | |  |  |  |  |  |
| 13.88333 | | | 174.9591 | | | | | | | | | | 0.561475 | | | | 4689.648 | | | 2.554688 | |  |  |  |  |  |
| 13.89167 | | | 175.0488 | | | | | | | | | | 0.562353 | | | | 4689.594 | | | 2.539063 | |  |  |  |  |  |
| 13.9 | | | 175.1378 | | | | | | | | | | 0.564413 | | | | 4689.57 | | | 2.460938 | |  |  |  |  |  |
| 13.90833 | | | 175.2241 | | | | | | | | | | 0.564492 | | | | 4689.539 | | | 2.523438 | |  |  |  |  |  |
| 13.91667 | | | 175.3096 | | | | | | | | | | 0.564945 | | | | 4689.539 | | | 2.445313 | |  |  |  |  |  |
| 13.925 | | | 175.3959 | | | | | | | | | | 0.566679 | | | | 4689.508 | | | 2.195313 | |  |  |  |  |  |
| 13.93333 | | | 175.4831 | | | | | | | | | | 0.566841 | | | | 4689.484 | | | 2.273438 | |  |  |  |  |  |
| 13.94167 | | | 175.5703 | | | | | | | | | | 0.56761 | | | | 4689.461 | | | 2.328125 | |  |  |  |  |  |
| 13.95 | | | 175.6567 | | | | | | | | | | 0.569492 | | | | 4689.438 | | | 2.539063 | |  |  |  |  |  |
| 13.95833 | | | 175.7447 | | | | | | | | | | 0.569039 | | | | 4689.414 | | | 2.726563 | |  |  |  |  |  |
| 13.96667 | | | 175.8371 | | | | | | | | | | 0.570059 | | | | 4689.406 | | | 2.75 | |  |  |  |  |  |
| 13.975 | | | 175.9252 | | | | | | | | | | 0.572099 | | | | 4689.422 | | | 2.75 | |  |  |  |  |  |
| 13.98333 | | | 176.0124 | | | | | | | | | | 0.572019 | | | | 4689.391 | | | 2.882813 | |  |  |  |  |  |
| 13.99167 | | | 176.1022 | | | | | | | | | | 0.57317 | | | | 4689.359 | | | 3.039063 | |  |  |  |  |  |
| 14 | | | 176.1954 | | | | | | | | | | 0.575671 | | | | 4689.305 | | | 3.304688 | |  |  |  |  |  |
| 14.00833 | | | 176.2869 | | | | | | | | | | 0.575894 | | | | 4689.258 | | | 3.21875 | |  |  |  |  |  |
| 14.01667 | | | 176.3758 | | | | | | | | | | 0.577407 | | | | 4689.234 | | | 3.398438 | |  |  |  |  |  |
| 14.025 | | | 176.4648 | | | | | | | | | | 0.579021 | | | | 4689.203 | | | 3.5 | |  |  |  |  |  |
| 14.03333 | | | 176.5546 | | | | | | | | | | 0.578985 | | | | 4689.164 | | | 3.164063 | |  |  |  |  |  |
| 14.04167 | | | 176.6443 | | | | | | | | | | 0.580922 | | | | 4689.117 | | | 3.179688 | |  |  |  |  |  |
| 14.05 | | | 176.7341 | | | | | | | | | | 0.582846 | | | | 4689.063 | | | 3.289063 | |  |  |  |  |  |
| 14.05833 | | | 176.8213 | | | | | | | | | | 0.582557 | | | | 4689.047 | | | 3.28125 | |  |  |  |  |  |
| 14.06667 | | | 176.9068 | | | | | | | | | | 0.584001 | | | | 4688.984 | | | 3.335938 | |  |  |  |  |  |
| 14.075 | | | 176.9914 | | | | | | | | | | 0.585981 | | | | 4688.953 | | | 3.289063 | |  |  |  |  |  |
| 14.08333 | | | 177.076 | | | | | | | | | | 0.585317 | | | | 4688.953 | | | 3.289063 | |  |  |  |  |  |
| 14.09167 | | | 177.165 | | | | | | | | | | 0.586935 | | | | 4688.945 | | | 3.492188 | |  |  |  |  |  |
| 14.1 | | | 177.2513 | | | | | | | | | | 0.58906 | | | | 4688.898 | | | 3.226563 | |  |  |  |  |  |
| 14.10833 | | | 177.3385 | | | | | | | | | | 0.588486 | | | | 4688.875 | | | 3.296875 | |  |  |  |  |  |
| 14.11667 | | | 177.4266 | | | | | | | | | | 0.590135 | | | | 4688.828 | | | 3.453125 | |  |  |  |  |  |
| 14.125 | | | 177.5129 | | | | | | | | | | 0.591578 | | | | 4688.813 | | | 3.585938 | |  |  |  |  |  |
| 14.13333 | | | 177.5984 | | | | | | | | | | 0.590596 | | | | 4688.781 | | | 3.570313 | |  |  |  |  |  |
| 14.14167 | | | 177.6838 | | | | | | | | | | 0.592239 | | | | 4688.734 | | | 3.960938 | |  |  |  |  |  |
| 14.15 | | | 177.7736 | | | | | | | | | | 0.593508 | | | | 4688.734 | | | 3.921875 | |  |  |  |  |  |
| 14.15833 | | | 177.8643 | | | | | | | | | | 0.593231 | | | | 4688.703 | | | 4.25 | |  |  |  |  |  |
| 14.16667 | | | 177.9558 | | | | | | | | | | 0.594899 | | | | 4688.656 | | | 4.203125 | |  |  |  |  |  |
| 14.175 | | | 178.0404 | | | | | | | | | | 0.595985 | | | | 4688.609 | | | 4.25 | |  |  |  |  |  |
| 14.18333 | | | 178.1267 | | | | | | | | | | 0.595663 | | | | 4688.578 | | | 4.578125 | |  |  |  |  |  |
| 14.19167 | | | 178.2157 | | | | | | | | | | 0.596892 | | | | 4688.5 | | | 4.421875 | |  |  |  |  |  |
| 14.2 | | | 178.3046 | | | | | | | | | | 0.598468 | | | | 4688.469 | | | 4.523438 | |  |  |  |  |  |
| 14.20833 | | | 178.3901 | | | | | | | | | | 0.597775 | | | | 4688.391 | | | 4.414063 | |  |  |  |  |  |
| 14.21667 | | | 178.4764 | | | | | | | | | | 0.599297 | | | | 4688.367 | | | 4.484375 | |  |  |  |  |  |
| 14.225 | | | 178.5662 | | | | | | | | | | 0.600964 | | | | 4688.32 | | | 4.28125 | |  |  |  |  |  |
| 14.23333 | | | 178.656 | | | | | | | | | | 0.600535 | | | | 4688.25 | | | 4.148438 | |  |  |  |  |  |
| 14.24167 | | | 178.7449 | | | | | | | | | | 0.602148 | | | | 4688.227 | | | 3.757813 | |  |  |  |  |  |
| 14.25 | | | 178.8313 | | | | | | | | | | 0.603414 | | | | 4688.188 | | | 3.476563 | |  |  |  |  |  |
| 14.25833 | | | 178.921 | | | | | | | | | | 0.603172 | | | | 4688.156 | | | 3.414063 | |  |  |  |  |  |
| 14.26667 | | | 179.0117 | | | | | | | | | | 0.605148 | | | | 4688.117 | | | 3.070313 | |  |  |  |  |  |
| 14.275 | | | 179.0989 | | | | | | | | | | 0.606537 | | | | 4688.109 | | | 3.148438 | |  |  |  |  |  |
| 14.28333 | | | 179.1878 | | | | | | | | | | 0.606496 | | | | 4688.094 | | | 3.125 | |  |  |  |  |  |
| 14.29167 | | | 179.2793 | | | | | | | | | | 0.608646 | | | | 4688.117 | | | 3.015625 | |  |  |  |  |  |
| 14.3 | | | 179.3665 | | | | | | | | | | 0.60968 | | | | 4688.125 | | | 2.976563 | |  |  |  |  |  |
| 14.30833 | | | 179.4555 | | | | | | | | | | 0.60941 | | | | 4688.109 | | | 2.835938 | |  |  |  |  |  |
| 14.31667 | | | 179.5444 | | | | | | | | | | 0.611914 | | | | 4688.141 | | | 2.765625 | |  |  |  |  |  |
| 14.325 | | | 179.6324 | | | | | | | | | | 0.613297 | | | | 4688.102 | | | 2.757813 | |  |  |  |  |  |
| 14.33333 | | | 179.7205 | | | | | | | | | | 0.61323 | | | | 4688.086 | | | 3.078125 | |  |  |  |  |  |
| 14.34167 | | | 179.8112 | | | | | | | | | | 0.615832 | | | | 4688.07 | | | 3.210938 | |  |  |  |  |  |
| 14.35 | | | 179.9035 | | | | | | | | | | 0.617059 | | | | 4688.047 | | | 3.554688 | |  |  |  |  |  |
| 14.35833 | | | 179.9951 | | | | | | | | | | 0.616966 | | | | 4688.039 | | | 3.578125 | |  |  |  |  |  |
| 14.36667 | | | 180.0831 | | | | | | | | | | 0.619561 | | | | 4688.031 | | | 3.359375 | |  |  |  |  |  |
| 14.375 | | | 180.1721 | | | | | | | | | | 0.620423 | | | | 4688.008 | | | 3.359375 | |  |  |  |  |  |
| 14.38333 | | | 180.2662 | | | | | | | | | | 0.620221 | | | | 4687.93 | | | 3.1875 | |  |  |  |  |  |
| 14.39167 | | | 180.3551 | | | | | | | | | | 0.622197 | | | | 4687.883 | | | 3.023438 | |  |  |  |  |  |
| 14.4 | | | 180.4414 | | | | | | | | | | 0.622945 | | | | 4687.797 | | | 3.265625 | |  |  |  |  |  |
| 14.40833 | | | 180.5295 | | | | | | | | | | 0.622945 | | | | 4687.766 | | | 3.367188 | |  |  |  |  |  |
| 14.41667 | | | 180.6219 | | | | | | | | | | 0.625707 | | | | 4687.766 | | | 3.132813 | |  |  |  |  |  |
| 14.425 | | | 180.7073 | | | | | | | | | | 0.626172 | | | | 4687.742 | | | 2.96875 | |  |  |  |  |  |
| 14.43333 | | | 180.7954 | | | | | | | | | | 0.626172 | | | | 4687.742 | | | 3.03125 | |  |  |  |  |  |
| 14.44167 | | | 180.8835 | | | | | | | | | | 0.628575 | | | | 4687.742 | | | 2.859375 | |  |  |  |  |  |
| 14.45 | | | 180.9733 | | | | | | | | | | 0.629152 | | | | 4687.672 | | | 3.117188 | |  |  |  |  |  |
| 14.45833 | | | 181.0622 | | | | | | | | | | 0.628555 | | | | 4687.633 | | | 3.304688 | |  |  |  |  |  |
| 14.46667 | | | 181.1494 | | | | | | | | | | 0.630588 | | | | 4687.641 | | | 3.382813 | |  |  |  |  |  |
| 14.475 | | | 181.2418 | | | | | | | | | | 0.631099 | | | | 4687.641 | | | 3.453125 | |  |  |  |  |  |
| 14.48333 | | | 181.3333 | | | | | | | | | | 0.630955 | | | | 4687.602 | | | 3.414063 | |  |  |  |  |  |
| 14.49167 | | | 181.4239 | | | | | | | | | | 0.633263 | | | | 4687.594 | | | 3.203125 | |  |  |  |  |  |
| 14.5 | | | 181.512 | | | | | | | | | | 0.633413 | | | | 4687.539 | | | 3.296875 | |  |  |  |  |  |
| 14.50833 | | | 181.6035 | | | | | | | | | | 0.633359 | | | | 4687.492 | | | 3.15625 | |  |  |  |  |  |
| 14.51667 | | | 181.6907 | | | | | | | | | | 0.635524 | | | | 4687.445 | | | 3.078125 | |  |  |  |  |  |
| 14.525 | | | 181.7788 | | | | | | | | | | 0.636106 | | | | 4687.414 | | | 3.054688 | |  |  |  |  |  |
| 14.53333 | | | 181.8686 | | | | | | | | | | 0.63612 | | | | 4687.383 | | | 2.8125 | |  |  |  |  |  |
| 14.54167 | | | 181.9601 | | | | | | | | | | 0.63909 | | | | 4687.383 | | | 2.703125 | |  |  |  |  |  |
| 14.55 | | | 182.0499 | | | | | | | | | | 0.640124 | | | | 4687.336 | | | 2.5625 | |  |  |  |  |  |
| 14.55833 | | | 182.1379 | | | | | | | | | | 0.640996 | | | | 4687.32 | | | 2.375 | |  |  |  |  |  |
| 14.56667 | | | 182.2269 | | | | | | | | | | 0.643872 | | | | 4687.305 | | | 2.28125 | |  |  |  |  |  |
| 14.575 | | | 182.3167 | | | | | | | | | | 0.64477 | | | | 4687.289 | | | 2.453125 | |  |  |  |  |  |
| 14.58333 | | | 182.4047 | | | | | | | | | | 0.645083 | | | | 4687.289 | | | 2.304688 | |  |  |  |  |  |
| 14.59167 | | | 182.4945 | | | | | | | | | | 0.647801 | | | | 4687.289 | | | 2.476563 | |  |  |  |  |  |
| 14.6 | | | 182.5834 | | | | | | | | | | 0.648234 | | | | 4687.281 | | | 2.257813 | |  |  |  |  |  |
| 14.60833 | | | 182.6732 | | | | | | | | | | 0.648132 | | | | 4687.289 | | | 2.421875 | |  |  |  |  |  |
| 14.61667 | | | 182.7656 | | | | | | | | | | 0.65058 | | | | 4687.273 | | | 2.625 | |  |  |  |  |  |
| 14.625 | | | 182.8519 | | | | | | | | | | 0.650858 | | | | 4687.242 | | | 2.671875 | |  |  |  |  |  |
| 14.63333 | | | 182.9426 | | | | | | | | | | 0.650889 | | | | 4687.227 | | | 2.617188 | |  |  |  |  |  |
| 14.64167 | | | 183.0289 | | | | | | | | | | 0.65298 | | | | 4687.195 | | | 2.734375 | |  |  |  |  |  |
| 14.65 | | | 183.1204 | | | | | | | | | | 0.653125 | | | | 4687.211 | | | 2.789063 | |  |  |  |  |  |
| 14.65833 | | | 183.2076 | | | | | | | | | | 0.653311 | | | | 4687.172 | | | 2.804688 | |  |  |  |  |  |
| 14.66667 | | | 183.2957 | | | | | | | | | | 0.655647 | | | | 4687.125 | | | 2.820313 | |  |  |  |  |  |
| 14.675 | | | 183.3838 | | | | | | | | | | 0.655278 | | | | 4687.102 | | | 2.90625 | |  |  |  |  |  |
| 14.68333 | | | 183.4744 | | | | | | | | | | 0.655699 | | | | 4687.078 | | | 2.664063 | |  |  |  |  |  |
| 14.69167 | | | 183.5633 | | | | | | | | | | 0.657422 | | | | 4687.039 | | | 2.679688 | |  |  |  |  |  |
| 14.7 | | | 183.6505 | | | | | | | | | | 0.657404 | | | | 4687.008 | | | 2.4375 | |  |  |  |  |  |
| 14.70833 | | | 183.7386 | | | | | | | | | | 0.658112 | | | | 4686.984 | | | 2.453125 | |  |  |  |  |  |
| 14.71667 | | | 183.8267 | | | | | | | | | | 0.65992 | | | | 4686.961 | | | 2.570313 | |  |  |  |  |  |
| 14.725 | | | 183.9147 | | | | | | | | | | 0.659749 | | | | 4686.93 | | | 2.546875 | |  |  |  |  |  |
| 14.73333 | | | 184.0002 | | | | | | | | | | 0.65973 | | | | 4686.922 | | | 2.492188 | |  |  |  |  |  |
| 14.74167 | | | 184.09 | | | | | | | | | | 0.661839 | | | | 4686.906 | | | 2.515625 | |  |  |  |  |  |
| 14.75 | | | 184.1824 | | | | | | | | | | 0.661379 | | | | 4686.906 | | | 2.71875 | |  |  |  |  |  |
| 14.75833 | | | 184.2748 | | | | | | | | | | 0.662142 | | | | 4686.883 | | | 2.71875 | |  |  |  |  |  |
| 14.76667 | | | 184.3628 | | | | | | | | | | 0.663654 | | | | 4686.852 | | | 2.984375 | |  |  |  |  |  |
| 14.775 | | | 184.4515 | | | | | | | | | | 0.663359 | | | | 4686.828 | | | 3 | |  |  |  |  |  |
| 14.78333 | | | 184.5402 | | | | | | | | | | 0.663837 | | | | 4686.82 | | | 3.140625 | |  |  |  |  |  |
| 14.79167 | | | 184.629 | | | | | | | | | | 0.665545 | | | | 4686.797 | | | 3.359375 | |  |  |  |  |  |
| 14.8 | | | 184.716 | | | | | | | | | | 0.665221 | | | | 4686.734 | | | 3.429688 | |  |  |  |  |  |
| 14.80833 | | | 184.8047 | | | | | | | | | | 0.665808 | | | | 4686.711 | | | 3.335938 | |  |  |  |  |  |
| 14.81667 | | | 184.8943 | | | | | | | | | | 0.667656 | | | | 4686.656 | | | 3.359375 | |  |  |  |  |  |
| 14.825 | | | 184.9847 | | | | | | | | | | 0.667423 | | | | 4686.625 | | | 3.234375 | |  |  |  |  |  |
| 14.83333 | | | 185.0717 | | | | | | | | | | 0.667973 | | | | 4686.578 | | | 3.070313 | |  |  |  |  |  |
| 14.84167 | | | 185.1596 | | | | | | | | | | 0.669883 | | | | 4686.531 | | | 3.140625 | |  |  |  |  |  |
| 14.85 | | | 185.2457 | | | | | | | | | | 0.669307 | | | | 4686.492 | | | 2.90625 | |  |  |  |  |  |
| 14.85833 | | | 185.3353 | | | | | | | | | | 0.670264 | | | | 4686.461 | | | 2.859375 | |  |  |  |  |  |
| 14.86667 | | | 185.4249 | | | | | | | | | | 0.672887 | | | | 4686.438 | | | 2.851563 | |  |  |  |  |  |
| 14.875 | | | 185.5153 | | | | | | | | | | 0.672738 | | | | 4686.422 | | | 3.117188 | |  |  |  |  |  |
| 14.88333 | | | 185.6074 | | | | | | | | | | 0.673309 | | | | 4686.414 | | | 3.132813 | |  |  |  |  |  |
| 14.89167 | | | 185.6987 | | | | | | | | | | 0.674894 | | | | 4686.391 | | | 3.1875 | |  |  |  |  |  |
| 14.9 | | | 185.7866 | | | | | | | | | | 0.674277 | | | | 4686.391 | | | 3.445313 | |  |  |  |  |  |
| 14.90833 | | | 185.8744 | | | | | | | | | | 0.675186 | | | | 4686.375 | | | 3.492188 | |  |  |  |  |  |
| 14.91667 | | | 185.9632 | | | | | | | | | | 0.677493 | | | | 4686.359 | | | 3.664063 | |  |  |  |  |  |
| 14.925 | | | 186.0519 | | | | | | | | | | 0.676946 | | | | 4686.297 | | | 3.789063 | |  |  |  |  |  |
| 14.93333 | | | 186.1457 | | | | | | | | | | 0.677857 | | | | 4686.266 | | | 3.960938 | |  |  |  |  |  |
| 14.94167 | | | 186.2344 | | | | | | | | | | 0.679439 | | | | 4686.234 | | | 4.109375 | |  |  |  |  |  |
| 14.95 | | | 186.3248 | | | | | | | | | | 0.678969 | | | | 4686.172 | | | 4.015625 | |  |  |  |  |  |
| 14.95833 | | | 186.4153 | | | | | | | | | | 0.680303 | | | | 4686.141 | | | 3.742188 | |  |  |  |  |  |
| 14.96667 | | | 186.5014 | | | | | | | | | | 0.682304 | | | | 4686.094 | | | 3.59375 | |  |  |  |  |  |
| 14.975 | | | 186.5867 | | | | | | | | | | 0.681814 | | | | 4686.039 | | | 3.726563 | |  |  |  |  |  |
| 14.98333 | | | 186.678 | | | | | | | | | | 0.682704 | | | | 4685.984 | | | 3.742188 | |  |  |  |  |  |
| 14.99167 | | | 186.7659 | | | | | | | | | | 0.684202 | | | | 4685.93 | | | 3.65625 | |  |  |  |  |  |
| 15 | | | 186.8546 | | | | | | | | | | 0.683713 | | | | 4685.914 | | | 3.523438 | |  |  |  |  |  |
| 15.00833 | | | 186.9433 | | | | | | | | | | 0.684586 | | | | 4685.922 | | | 3.234375 | |  |  |  |  |  |
| 15.01667 | | | 187.032 | | | | | | | | | | 0.686316 | | | | 4685.906 | | | 3.203125 | |  |  |  |  |  |
| 15.025 | | | 187.1216 | | | | | | | | | | 0.686138 | | | | 4685.867 | | | 3.132813 | |  |  |  |  |  |
| 15.03333 | | | 187.2078 | | | | | | | | | | 0.687056 | | | | 4685.836 | | | 3.289063 | |  |  |  |  |  |
| 15.04167 | | | 187.2965 | | | | | | | | | | 0.689027 | | | | 4685.805 | | | 3.453125 | |  |  |  |  |  |
| 15.05 | | | 187.3852 | | | | | | | | | | 0.688258 | | | | 4685.805 | | | 3.421875 | |  |  |  |  |  |
| 15.05833 | | | 187.4782 | | | | | | | | | | 0.689136 | | | | 4685.813 | | | 3.296875 | |  |  |  |  |  |
| 15.06667 | | | 187.5669 | | | | | | | | | | 0.690637 | | | | 4685.789 | | | 3.054688 | |  |  |  |  |  |
| 15.075 | | | 187.6582 | | | | | | | | | | 0.689911 | | | | 4685.781 | | | 3.25 | |  |  |  |  |  |
| 15.08333 | | | 187.7469 | | | | | | | | | | 0.690932 | | | | 4685.719 | | | 3.140625 | |  |  |  |  |  |
| 15.09167 | | | 187.8365 | | | | | | | | | | 0.692737 | | | | 4685.68 | | | 3.257813 | |  |  |  |  |  |
| 15.1 | | | 187.9235 | | | | | | | | | | 0.691793 | | | | 4685.656 | | | 3.25 | |  |  |  |  |  |
| 15.10833 | | | 188.0088 | | | | | | | | | | 0.693251 | | | | 4685.656 | | | 3.265625 | |  |  |  |  |  |
| 15.11667 | | | 188.0967 | | | | | | | | | | 0.69467 | | | | 4685.656 | | | 3.226563 | |  |  |  |  |  |
| 15.125 | | | 188.1854 | | | | | | | | | | 0.694311 | | | | 4685.594 | | | 3.140625 | |  |  |  |  |  |
| 15.13333 | | | 188.2767 | | | | | | | | | | 0.695922 | | | | 4685.594 | | | 3.210938 | |  |  |  |  |  |
| 15.14167 | | | 188.3611 | | | | | | | | | | 0.69791 | | | | 4685.547 | | | 3.539063 | |  |  |  |  |  |
| 15.15 | | | 188.4473 | | | | | | | | | | 0.697855 | | | | 4685.516 | | | 3.546875 | |  |  |  |  |  |
| 15.15833 | | | 188.5334 | | | | | | | | | | 0.699397 | | | | 4685.484 | | | 3.390625 | |  |  |  |  |  |
| 15.16667 | | | 188.6205 | | | | | | | | | | 0.701323 | | | | 4685.469 | | | 3.648438 | |  |  |  |  |  |
| 15.175 | | | 188.7075 | | | | | | | | | | 0.700702 | | | | 4685.445 | | | 3.578125 | |  |  |  |  |  |
| 15.18333 | | | 188.7953 | | | | | | | | | | 0.702188 | | | | 4685.406 | | | 3.601563 | |  |  |  |  |  |
| 15.19167 | | | 188.884 | | | | | | | | | | 0.703763 | | | | 4685.328 | | | 3.59375 | |  |  |  |  |  |
| 15.2 | | | 188.9719 | | | | | | | | | | 0.703 | | | | 4685.305 | | | 3.609375 | |  |  |  |  |  |
| 15.20833 | | | 189.0606 | | | | | | | | | | 0.703704 | | | | 4685.289 | | | 3.453125 | |  |  |  |  |  |
| 15.21667 | | | 189.1485 | | | | | | | | | | 0.704988 | | | | 4685.227 | | | 3.46875 | |  |  |  |  |  |
| 15.225 | | | 189.2406 | | | | | | | | | | 0.70365 | | | | 4685.203 | | | 3.46875 | |  |  |  |  |  |
| 15.23333 | | | 189.3311 | | | | | | | | | | 0.705059 | | | | 4685.18 | | | 3.40625 | |  |  |  |  |  |
| 15.24167 | | | 189.4223 | | | | | | | | | | 0.706514 | | | | 4685.148 | | | 3.25 | |  |  |  |  |  |
| 15.25 | | | 189.5128 | | | | | | | | | | 0.705697 | | | | 4685.117 | | | 3.210938 | |  |  |  |  |  |
| 15.25833 | | | 189.5998 | | | | | | | | | | 0.707486 | | | | 4685.109 | | | 3.132813 | |  |  |  |  |  |
| 15.26667 | | | 189.6885 | | | | | | | | | | 0.708572 | | | | 4685.086 | | | 3.046875 | |  |  |  |  |  |
| 15.275 | | | 189.7772 | | | | | | | | | | 0.70774 | | | | 4685.055 | | | 3.085938 | |  |  |  |  |  |
| 15.28333 | | | 189.8668 | | | | | | | | | | 0.709504 | | | | 4685.031 | | | 2.976563 | |  |  |  |  |  |
| 15.29167 | | | 189.9547 | | | | | | | | | | 0.71041 | | | | 4685.023 | | | 3.1875 | |  |  |  |  |  |
| 15.3 | | | 190.0451 | | | | | | | | | | 0.710415 | | | | 4685 | | | 3.359375 | |  |  |  |  |  |
| 15.30833 | | | 190.1364 | | | | | | | | | | 0.712276 | | | | 4684.977 | | | 3.320313 | |  |  |  |  |  |
| 15.31667 | | | 190.2276 | | | | | | | | | | 0.712914 | | | | 4684.969 | | | 3.59375 | |  |  |  |  |  |
| 15.325 | | | 190.3189 | | | | | | | | | | 0.712793 | | | | 4684.938 | | | 3.625 | |  |  |  |  |  |
| 15.33333 | | | 190.4085 | | | | | | | | | | 0.714463 | | | | 4684.922 | | | 3.617188 | |  |  |  |  |  |
| 15.34167 | | | 190.5006 | | | | | | | | | | 0.714856 | | | | 4684.859 | | | 3.578125 | |  |  |  |  |  |
| 15.35 | | | 190.5919 | | | | | | | | | | 0.714424 | | | | 4684.813 | | | 3.90625 | |  |  |  |  |  |
| 15.35833 | | | 190.684 | | | | | | | | | | 0.71662 | | | | 4684.789 | | | 3.804688 | |  |  |  |  |  |
| 15.36667 | | | 190.7736 | | | | | | | | | | 0.717183 | | | | 4684.727 | | | 3.65625 | |  |  |  |  |  |
| 15.375 | | | 190.8657 | | | | | | | | | | 0.71725 | | | | 4684.688 | | | 3.570313 | |  |  |  |  |  |
| 15.38333 | | | 190.9544 | | | | | | | | | | 0.719288 | | | | 4684.656 | | | 3.4375 | |  |  |  |  |  |
| 15.39167 | | | 191.044 | | | | | | | | | | 0.719697 | | | | 4684.625 | | | 3.453125 | |  |  |  |  |  |
| 15.4 | | | 191.1344 | | | | | | | | | | 0.719832 | | | | 4684.563 | | | 3.265625 | |  |  |  |  |  |
| 15.40833 | | | 191.2197 | | | | | | | | | | 0.721891 | | | | 4684.547 | | | 3.25 | |  |  |  |  |  |
| 15.41667 | | | 191.311 | | | | | | | | | | 0.722616 | | | | 4684.531 | | | 3.375 | |  |  |  |  |  |
| 15.425 | | | 191.4014 | | | | | | | | | | 0.723255 | | | | 4684.523 | | | 3.398438 | |  |  |  |  |  |
| 15.43333 | | | 191.491 | | | | | | | | | | 0.725499 | | | | 4684.508 | | | 3.210938 | |  |  |  |  |  |
| 15.44167 | | | 191.5823 | | | | | | | | | | 0.72613 | | | | 4684.477 | | | 3.257813 | |  |  |  |  |  |
| 15.45 | | | 191.6727 | | | | | | | | | | 0.726941 | | | | 4684.484 | | | 3.382813 | |  |  |  |  |  |
| 15.45833 | | | 191.7623 | | | | | | | | | | 0.728971 | | | | 4684.453 | | | 3.34375 | |  |  |  |  |  |
| 15.46667 | | | 191.851 | | | | | | | | | | 0.72939 | | | | 4684.406 | | | 3.289063 | |  |  |  |  |  |
| 15.475 | | | 191.938 | | | | | | | | | | 0.729603 | | | | 4684.375 | | | 3.265625 | |  |  |  |  |  |
| 15.48333 | | | 192.0233 | | | | | | | | | | 0.731643 | | | | 4684.375 | | | 3.242188 | |  |  |  |  |  |
| 15.49167 | | | 192.1172 | | | | | | | | | | 0.731838 | | | | 4684.336 | | | 3.054688 | |  |  |  |  |  |
| 15.5 | | | 192.2085 | | | | | | | | | | 0.731621 | | | | 4684.289 | | | 3.070313 | |  |  |  |  |  |
| 15.50833 | | | 192.2972 | | | | | | | | | | 0.733108 | | | | 4684.266 | | | 3.039063 | |  |  |  |  |  |
| 15.51667 | | | 192.385 | | | | | | | | | | 0.732804 | | | | 4684.258 | | | 3.015625 | |  |  |  |  |  |
| 15.525 | | | 192.472 | | | | | | | | | | 0.732073 | | | | 4684.227 | | | 3.09375 | |  |  |  |  |  |
| 15.53333 | | | 192.565 | | | | | | | | | | 0.733579 | | | | 4684.203 | | | 3.210938 | |  |  |  |  |  |
| 15.54167 | | | 192.6537 | | | | | | | | | | 0.733369 | | | | 4684.195 | | | 3.164063 | |  |  |  |  |  |
| 15.55 | | | 192.745 | | | | | | | | | | 0.733213 | | | | 4684.172 | | | 3.085938 | |  |  |  |  |  |
| 15.55833 | | | 192.8397 | | | | | | | | | | 0.735089 | | | | 4684.148 | | | 3.203125 | |  |  |  |  |  |
| 15.56667 | | | 192.9344 | | | | | | | | | | 0.734985 | | | | 4684.125 | | | 3.390625 | |  |  |  |  |  |
| 15.575 | | | 193.0223 | | | | | | | | | | 0.735135 | | | | 4684.078 | | | 3.453125 | |  |  |  |  |  |
| 15.58333 | | | 193.1101 | | | | | | | | | | 0.736574 | | | | 4684.039 | | | 3.484375 | |  |  |  |  |  |
| 15.59167 | | | 193.1997 | | | | | | | | | | 0.736392 | | | | 4684.023 | | | 3.617188 | |  |  |  |  |  |
| 15.6 | | | 193.2901 | | | | | | | | | | 0.73649 | | | | 4684 | | | 3.625 | |  |  |  |  |  |
| 15.60833 | | | 193.3789 | | | | | | | | | | 0.738681 | | | | 4683.969 | | | 3.664063 | |  |  |  |  |  |
| 15.61667 | | | 193.4642 | | | | | | | | | | 0.738883 | | | | 4683.922 | | | 3.546875 | |  |  |  |  |  |
| 15.625 | | | 193.5537 | | | | | | | | | | 0.739026 | | | | 4683.875 | | | 3.820313 | |  |  |  |  |  |
| 15.63333 | | | 193.6416 | | | | | | | | | | 0.740972 | | | | 4683.836 | | | 4.015625 | |  |  |  |  |  |
| 15.64167 | | | 193.7278 | | | | | | | | | | 0.740694 | | | | 4683.797 | | | 4.101563 | |  |  |  |  |  |
| 15.65 | | | 193.8122 | | | | | | | | | | 0.740212 | | | | 4683.766 | | | 4.148438 | |  |  |  |  |  |
| 15.65833 | | | 193.9001 | | | | | | | | | | 0.741958 | | | | 4683.734 | | | 4.023438 | |  |  |  |  |  |
| 15.66667 | | | 193.9897 | | | | | | | | | | 0.741545 | | | | 4683.711 | | | 4.289063 | |  |  |  |  |  |
| 15.675 | | | 194.0801 | | | | | | | | | | 0.74122 | | | | 4683.648 | | | 4.351563 | |  |  |  |  |  |
| 15.68333 | | | 194.168 | | | | | | | | | | 0.7432 | | | | 4683.586 | | | 4.617188 | |  |  |  |  |  |
| 15.69167 | | | 194.2592 | | | | | | | | | | 0.742806 | | | | 4683.539 | | | 4.765625 | |  |  |  |  |  |
| 15.7 | | | 194.3497 | | | | | | | | | | 0.742412 | | | | 4683.5 | | | 4.742188 | |  |  |  |  |  |
| 15.70833 | | | 194.4375 | | | | | | | | | | 0.744335 | | | | 4683.469 | | | 4.898438 | |  |  |  |  |  |
| 15.71667 | | | 194.5296 | | | | | | | | | | 0.743769 | | | | 4683.406 | | | 5 | |  |  |  |  |  |
| 15.725 | | | 194.6209 | | | | | | | | | | 0.743532 | | | | 4683.359 | | | 5.132813 | |  |  |  |  |  |
| 15.73333 | | | 194.7113 | | | | | | | | | | 0.745526 | | | | 4683.281 | | | 4.976563 | |  |  |  |  |  |
| 15.74167 | | | 194.8001 | | | | | | | | | | 0.745333 | | | | 4683.219 | | | 4.976563 | |  |  |  |  |  |
| 15.75 | | | 194.8896 | | | | | | | | | | 0.745946 | | | | 4683.18 | | | 4.804688 | |  |  |  |  |  |
| 15.75833 | | | 194.9818 | | | | | | | | | | 0.747993 | | | | 4683.117 | | | 4.609375 | |  |  |  |  |  |
| 15.76667 | | | 195.0748 | | | | | | | | | | 0.747793 | | | | 4683.055 | | | 4.328125 | |  |  |  |  |  |
| 15.775 | | | 195.1643 | | | | | | | | | | 0.748389 | | | | 4683.016 | | | 4.171875 | |  |  |  |  |  |
| 15.78333 | | | 195.2556 | | | | | | | | | | 0.749827 | | | | 4683 | | | 4.070313 | |  |  |  |  |  |
| 15.79167 | | | 195.352 | | | | | | | | | | 0.7492 | | | | 4682.953 | | | 3.851563 | |  |  |  |  |  |
| 15.8 | | | 195.4424 | | | | | | | | | | 0.749794 | | | | 4682.953 | | | 3.664063 | |  |  |  |  |  |
| 15.80833 | | | 195.5329 | | | | | | | | | | 0.751492 | | | | 4682.93 | | | 3.492188 | |  |  |  |  |  |
| 15.81667 | | | 195.625 | | | | | | | | | | 0.751117 | | | | 4682.93 | | | 3.5 | |  |  |  |  |  |
| 15.825 | | | 195.7163 | | | | | | | | | | 0.751857 | | | | 4682.93 | | | 3.460938 | |  |  |  |  |  |
| 15.83333 | | | 195.8092 | | | | | | | | | | 0.753367 | | | | 4682.914 | | | 3.4375 | |  |  |  |  |  |
| 15.84167 | | | 195.8997 | | | | | | | | | | 0.75309 | | | | 4682.906 | | | 3.484375 | |  |  |  |  |  |
| 15.85 | | | 195.9884 | | | | | | | | | | 0.753894 | | | | 4682.906 | | | 3.515625 | |  |  |  |  |  |
| 15.85833 | | | 196.0805 | | | | | | | | | | 0.755528 | | | | 4682.898 | | | 3.515625 | |  |  |  |  |  |
| 15.86667 | | | 196.1684 | | | | | | | | | | 0.755525 | | | | 4682.867 | | | 3.570313 | |  |  |  |  |  |
| 15.875 | | | 196.2537 | | | | | | | | | | 0.756667 | | | | 4682.852 | | | 3.515625 | |  |  |  |  |  |
| 15.88333 | | | 196.345 | | | | | | | | | | 0.758587 | | | | 4682.828 | | | 3.515625 | |  |  |  |  |  |
| 15.89167 | | | 196.4328 | | | | | | | | | | 0.758005 | | | | 4682.797 | | | 3.484375 | |  |  |  |  |  |
| 15.9 | | | 196.5258 | | | | | | | | | | 0.758758 | | | | 4682.758 | | | 3.578125 | |  |  |  |  |  |
| 15.90833 | | | 196.6188 | | | | | | | | | | 0.759996 | | | | 4682.734 | | | 3.671875 | |  |  |  |  |  |
| 15.91667 | | | 196.7067 | | | | | | | | | | 0.759318 | | | | 4682.688 | | | 3.59375 | |  |  |  |  |  |
| 15.925 | | | 196.7937 | | | | | | | | | | 0.760109 | | | | 4682.664 | | | 3.890625 | |  |  |  |  |  |
| 15.93333 | | | 196.8858 | | | | | | | | | | 0.761136 | | | | 4682.633 | | | 3.929688 | |  |  |  |  |  |
| 15.94167 | | | 196.9754 | | | | | | | | | | 0.760506 | | | | 4682.609 | | | 3.726563 | |  |  |  |  |  |
| 15.95 | | | 197.0692 | | | | | | | | | | 0.761262 | | | | 4682.563 | | | 3.898438 | |  |  |  |  |  |
| 15.95833 | | | 197.1605 | | | | | | | | | | 0.762208 | | | | 4682.523 | | | 3.96875 | |  |  |  |  |  |
| 15.96667 | | | 197.2492 | | | | | | | | | | 0.761405 | | | | 4682.5 | | | 4.03125 | |  |  |  |  |  |
| 15.975 | | | 197.3422 | | | | | | | | | | 0.762748 | | | | 4682.43 | | | 4.046875 | |  |  |  |  |  |
| 15.98333 | | | 197.4301 | | | | | | | | | | 0.764059 | | | | 4682.391 | | | 4.03125 | |  |  |  |  |  |
| 15.99167 | | | 197.5171 | | | | | | | | | | 0.763643 | | | | 4682.383 | | | 4.117188 | |  |  |  |  |  |
| 16 | | | 197.6067 | | | | | | | | | | 0.764814 | | | | 4682.32 | | | 4.179688 | |  |  |  |  |  |
| 16.00833 | | | 197.7014 | | | | | | | | | | 0.766473 | | | | 4682.281 | | | 4.15625 | |  |  |  |  |  |
| 16.01667 | | | 197.7926 | | | | | | | | | | 0.766328 | | | | 4682.242 | | | 4.140625 | |  |  |  |  |  |
| 16.025 | | | 197.8865 | | | | | | | | | | 0.767764 | | | | 4682.203 | | | 4.460938 | |  |  |  |  |  |
| 16.03333 | | | 197.9794 | | | | | | | | | | 0.769022 | | | | 4682.164 | | | 4.21875 | |  |  |  |  |  |
| 16.04167 | | | 198.0699 | | | | | | | | | | 0.769025 | | | | 4682.117 | | | 4.015625 | |  |  |  |  |  |
| 16.05 | | | 198.1611 | | | | | | | | | | 0.770822 | | | | 4682.07 | | | 3.960938 | |  |  |  |  |  |
| 16.05833 | | | 198.249 | | | | | | | | | | 0.771776 | | | | 4682.047 | | | 3.882813 | |  |  |  |  |  |
| 16.06667 | | | 198.3403 | | | | | | | | | | 0.771683 | | | | 4682.008 | | | 3.78125 | |  |  |  |  |  |
| 16.075 | | | 198.4333 | | | | | | | | | | 0.773508 | | | | 4681.922 | | | 3.554688 | |  |  |  |  |  |
| 16.08333 | | | 198.5263 | | | | | | | | | | 0.774635 | | | | 4681.922 | | | 3.585938 | |  |  |  |  |  |
| 16.09167 | | | 198.615 | | | | | | | | | | 0.774353 | | | | 4681.906 | | | 3.453125 | |  |  |  |  |  |
| 16.1 | | | 198.7046 | | | | | | | | | | 0.775594 | | | | 4681.883 | | | 3.210938 | |  |  |  |  |  |
| 16.10833 | | | 198.7899 | | | | | | | | | | 0.776815 | | | | 4681.859 | | | 3.15625 | |  |  |  |  |  |
| 16.11667 | | | 198.876 | | | | | | | | | | 0.776276 | | | | 4681.836 | | | 3.148438 | |  |  |  |  |  |
| 16.125 | | | 198.9673 | | | | | | | | | | 0.777405 | | | | 4681.844 | | | 3.382813 | |  |  |  |  |  |
| 16.13333 | | | 199.0569 | | | | | | | | | | 0.778314 | | | | 4681.805 | | | 3.296875 | |  |  |  |  |  |
| 16.14167 | | | 199.1464 | | | | | | | | | | 0.777202 | | | | 4681.797 | | | 3.359375 | |  |  |  |  |  |
| 16.15 | | | 199.236 | | | | | | | | | | 0.778185 | | | | 4681.797 | | | 3.492188 | |  |  |  |  |  |
| 16.15833 | | | 199.3281 | | | | | | | | | | 0.779327 | | | | 4681.789 | | | 3.734375 | |  |  |  |  |  |
| 16.16667 | | | 199.4152 | | | | | | | | | | 0.778424 | | | | 4681.758 | | | 3.867188 | |  |  |  |  |  |
| 16.175 | | | 199.503 | | | | | | | | | | 0.779433 | | | | 4681.711 | | | 3.8125 | |  |  |  |  |  |
| 16.18333 | | | 199.5909 | | | | | | | | | | 0.78056 | | | | 4681.695 | | | 4.289063 | |  |  |  |  |  |
| 16.19167 | | | 199.6813 | | | | | | | | | | 0.779787 | | | | 4681.648 | | | 4.320313 | |  |  |  |  |  |
| 16.2 | | | 199.7717 | | | | | | | | | | 0.780851 | | | | 4681.609 | | | 4.375 | |  |  |  |  |  |
| 16.20833 | | | 199.8639 | | | | | | | | | | 0.782041 | | | | 4681.547 | | | 4.28125 | |  |  |  |  |  |
| 16.21667 | | | 199.9517 | | | | | | | | | | 0.781489 | | | | 4681.5 | | | 4.445313 | |  |  |  |  |  |
| 16.225 | | | 200.0456 | | | | | | | | | | 0.783342 | | | | 4681.477 | | | 4.476563 | |  |  |  |  |  |
| 16.23333 | | | 200.1377 | | | | | | | | | | 0.784579 | | | | 4681.375 | | | 4.445313 | |  |  |  |  |  |
| 16.24167 | | | 200.2264 | | | | | | | | | | 0.783996 | | | | 4681.336 | | | 4.5 | |  |  |  |  |  |
| 16.25 | | | 200.3168 | | | | | | | | | | 0.785517 | | | | 4681.289 | | | 4.445313 | |  |  |  |  |  |
| 16.25833 | | | 200.4107 | | | | | | | | | | 0.786848 | | | | 4681.273 | | | 4.40625 | |  |  |  |  |  |
| 16.26667 | | | 200.5054 | | | | | | | | | | 0.786835 | | | | 4681.203 | | | 4.25 | |  |  |  |  |  |
| 16.275 | | | 200.6018 | | | | | | | | | | 0.788731 | | | | 4681.172 | | | 4.171875 | |  |  |  |  |  |
| 16.28333 | | | 200.6948 | | | | | | | | | | 0.78968 | | | | 4681.133 | | | 4.203125 | |  |  |  |  |  |
| 16.29167 | | | 200.7843 | | | | | | | | | | 0.790105 | | | | 4681.086 | | | 4.320313 | |  |  |  |  |  |
| 16.3 | | | 200.8807 | | | | | | | | | | 0.792502 | | | | 4681.063 | | | 4.351563 | |  |  |  |  |  |
| 16.30833 | | | 200.9729 | | | | | | | | | | 0.792642 | | | | 4681.023 | | | 4.5 | |  |  |  |  |  |
| 16.31667 | | | 201.0599 | | | | | | | | | | 0.793068 | | | | 4681.016 | | | 4.453125 | |  |  |  |  |  |
| 16.325 | | | 201.1503 | | | | | | | | | | 0.794887 | | | | 4680.992 | | | 4.390625 | |  |  |  |  |  |
| 16.33333 | | | 201.2424 | | | | | | | | | | 0.795021 | | | | 4680.945 | | | 4.289063 | |  |  |  |  |  |
| 16.34167 | | | 201.3311 | | | | | | | | | | 0.795268 | | | | 4680.898 | | | 4.453125 | |  |  |  |  |  |
| 16.35 | | | 201.4207 | | | | | | | | | | 0.796811 | | | | 4680.852 | | | 4.398438 | |  |  |  |  |  |
| 16.35833 | | | 201.506 | | | | | | | | | | 0.796942 | | | | 4680.797 | | | 4.375 | |  |  |  |  |  |
| 16.36667 | | | 201.5973 | | | | | | | | | | 0.797173 | | | | 4680.766 | | | 4.390625 | |  |  |  |  |  |
| 16.375 | | | 201.6877 | | | | | | | | | | 0.798874 | | | | 4680.742 | | | 4.3125 | |  |  |  |  |  |
| 16.38333 | | | 201.7764 | | | | | | | | | | 0.79887 | | | | 4680.711 | | | 4.203125 | |  |  |  |  |  |
| 16.39167 | | | 201.8669 | | | | | | | | | | 0.799338 | | | | 4680.648 | | | 4.09375 | |  |  |  |  |  |
| 16.4 | | | 201.9581 | | | | | | | | | | 0.801247 | | | | 4680.617 | | | 4.046875 | |  |  |  |  |  |
| 16.40833 | | | 202.0494 | | | | | | | | | | 0.801639 | | | | 4680.586 | | | 4.1875 | |  |  |  |  |  |
| 16.41667 | | | 202.1407 | | | | | | | | | | 0.801762 | | | | 4680.555 | | | 4.484375 | |  |  |  |  |  |
| 16.425 | | | 202.2294 | | | | | | | | | | 0.803074 | | | | 4680.516 | | | 4.453125 | |  |  |  |  |  |
| 16.43333 | | | 202.3207 | | | | | | | | | | 0.803159 | | | | 4680.508 | | | 4.609375 | |  |  |  |  |  |
| 16.44167 | | | 202.4154 | | | | | | | | | | 0.803318 | | | | 4680.484 | | | 4.84375 | |  |  |  |  |  |
| 16.45 | | | 202.5033 | | | | | | | | | | 0.804875 | | | | 4680.461 | | | 4.984375 | |  |  |  |  |  |
| 16.45833 | | | 202.5928 | | | | | | | | | | 0.805039 | | | | 4680.406 | | | 4.679688 | |  |  |  |  |  |
| 16.46667 | | | 202.6833 | | | | | | | | | | 0.805024 | | | | 4680.336 | | | 4.820313 | |  |  |  |  |  |
| 16.475 | | | 202.7737 | | | | | | | | | | 0.80656 | | | | 4680.297 | | | 4.710938 | |  |  |  |  |  |
| 16.48333 | | | 202.8684 | | | | | | | | | | 0.806621 | | | | 4680.242 | | | 4.929688 | |  |  |  |  |  |
| 16.49167 | | | 202.9597 | | | | | | | | | | 0.806825 | | | | 4680.164 | | | 4.90625 | |  |  |  |  |  |
| 16.5 | | | 203.0527 | | | | | | | | | | 0.808623 | | | | 4680.117 | | | 4.601563 | |  |  |  |  |  |
| 16.50833 | | | 203.1443 | | | | | | | | | | 0.80907 | | | | 4680.125 | | | 4.304688 | |  |  |  |  |  |
| 16.51667 | | | 203.2309 | | | | | | | | | | 0.81021 | | | | 4680.07 | | | 4.210938 | |  |  |  |  |  |
| 16.525 | | | 203.3183 | | | | | | | | | | 0.812118 | | | | 4680.047 | | | 4.101563 | |  |  |  |  |  |
| 16.53333 | | | 203.4073 | | | | | | | | | | 0.811779 | | | | 4679.977 | | | 4.070313 | |  |  |  |  |  |
| 16.54167 | | | 203.4998 | | | | | | | | | | 0.812872 | | | | 4679.93 | | | 4.0625 | |  |  |  |  |  |
| 16.55 | | | 203.5888 | | | | | | | | | | 0.814553 | | | | 4679.938 | | | 4.109375 | |  |  |  |  |  |
| 16.55833 | | | 203.6762 | | | | | | | | | | 0.81444 | | | | 4679.938 | | | 4.367188 | |  |  |  |  |  |
| 16.56667 | | | 203.7661 | | | | | | | | | | 0.815676 | | | | 4679.914 | | | 4.179688 | |  |  |  |  |  |
| 16.575 | | | 203.856 | | | | | | | | | | 0.817496 | | | | 4679.898 | | | 4.15625 | |  |  |  |  |  |
| 16.58333 | | | 203.941 | | | | | | | | | | 0.81748 | | | | 4679.859 | | | 4.132813 | |  |  |  |  |  |
| 16.59167 | | | 204.0292 | | | | | | | | | | 0.818467 | | | | 4679.82 | | | 4.382813 | |  |  |  |  |  |
| 16.6 | | | 204.1216 | | | | | | | | | | 0.820402 | | | | 4679.781 | | | 4.484375 | |  |  |  |  |  |
| 16.60833 | | | 204.2124 | | | | | | | | | | 0.820536 | | | | 4679.719 | | | 4.59375 | |  |  |  |  |  |
| 16.61667 | | | 204.3014 | | | | | | | | | | 0.821719 | | | | 4679.695 | | | 4.8125 | |  |  |  |  |  |
| 16.625 | | | 204.3855 | | | | | | | | | | 0.823314 | | | | 4679.672 | | | 5.078125 | |  |  |  |  |  |
| 16.63333 | | | 204.4738 | | | | | | | | | | 0.823207 | | | | 4679.633 | | | 5.1875 | |  |  |  |  |  |
| 16.64167 | | | 204.5637 | | | | | | | | | | 0.823759 | | | | 4679.57 | | | 5.15625 | |  |  |  |  |  |
| 16.65 | | | 204.6519 | | | | | | | | | | 0.825208 | | | | 4679.516 | | | 5.125 | |  |  |  |  |  |
| 16.65833 | | | 204.7385 | | | | | | | | | | 0.82494 | | | | 4679.461 | | | 4.953125 | |  |  |  |  |  |
| 16.66667 | | | 204.8292 | | | | | | | | | | 0.825966 | | | | 4679.398 | | | 5.109375 | |  |  |  |  |  |
| 16.675 | | | 204.9175 | | | | | | | | | | 0.827537 | | | | 4679.313 | | | 5.039063 | |  |  |  |  |  |
| 16.68333 | | | 205.0049 | | | | | | | | | | 0.827167 | | | | 4679.258 | | | 5.023438 | |  |  |  |  |  |
| 16.69167 | | | 205.0906 | | | | | | | | | | 0.827897 | | | | 4679.219 | | | 5 | |  |  |  |  |  |
| 16.7 | | | 205.1797 | | | | | | | | | | 0.829141 | | | | 4679.18 | | | 4.945313 | |  |  |  |  |  |
| 16.70833 | | | 205.2696 | | | | | | | | | | 0.828771 | | | | 4679.156 | | | 4.796875 | |  |  |  |  |  |
| 16.71667 | | | 205.362 | | | | | | | | | | 0.829436 | | | | 4679.102 | | | 4.90625 | |  |  |  |  |  |
| 16.725 | | | 205.4494 | | | | | | | | | | 0.830637 | | | | 4679.07 | | | 4.820313 | |  |  |  |  |  |
| 16.73333 | | | 205.5368 | | | | | | | | | | 0.830204 | | | | 4679.023 | | | 5.3125 | |  |  |  |  |  |
| 16.74167 | | | 205.6284 | | | | | | | | | | 0.831449 | | | | 4678.984 | | | 5.507813 | |  |  |  |  |  |
| 16.75 | | | 205.7183 | | | | | | | | | | 0.832675 | | | | 4678.953 | | | 5.53125 | |  |  |  |  |  |
| 16.75833 | | | 205.809 | | | | | | | | | | 0.832172 | | | | 4678.938 | | | 5.296875 | |  |  |  |  |  |
| 16.76667 | | | 205.8973 | | | | | | | | | | 0.833663 | | | | 4678.883 | | | 5.328125 | |  |  |  |  |  |
| 16.775 | | | 205.9888 | | | | | | | | | | 0.834825 | | | | 4678.852 | | | 5.21875 | |  |  |  |  |  |
| 16.78333 | | | 206.0788 | | | | | | | | | | 0.834473 | | | | 4678.742 | | | 5.28125 | |  |  |  |  |  |
| 16.79167 | | | 206.1703 | | | | | | | | | | 0.836073 | | | | 4678.68 | | | 5.34375 | |  |  |  |  |  |
| 16.8 | | | 206.2561 | | | | | | | | | | 0.837146 | | | | 4678.625 | | | 5.0625 | |  |  |  |  |  |
| 16.80833 | | | 206.366 | | | | | | | | | | 0.837132 | | | | 4678.586 | | | 5.171875 | |  |  |  |  |  |
| 16.81667 | | | 206.4384 | | | | | | | | | | 0.83895 | | | | 4678.555 | | | 4.835938 | |  |  |  |  |  |
| 16.825 | | | 206.5266 | | | | | | | | | | 0.840375 | | | | 4678.531 | | | 4.71875 | |  |  |  |  |  |
| 16.83333 | | | 206.614 | | | | | | | | | | 0.840108 | | | | 4678.477 | | | 4.65625 | |  |  |  |  |  |
| 16.84167 | | | 206.7023 | | | | | | | | | | 0.841214 | | | | 4678.422 | | | 4.875 | |  |  |  |  |  |
| 16.85 | | | 206.7905 | | | | | | | | | | 0.842265 | | | | 4678.414 | | | 4.867188 | |  |  |  |  |  |
| 16.85833 | | | 206.8813 | | | | | | | | | | 0.841829 | | | | 4678.352 | | | 4.796875 | |  |  |  |  |  |
| 16.86667 | | | 206.9928 | | | | | | | | | | 0.843706 | | | | 4678.383 | | | 4.875 | |  |  |  |  |  |
| 16.875 | | | 207.0811 | | | | | | | | | | 0.844354 | | | | 4678.344 | | | 4.921875 | |  |  |  |  |  |
| 16.88333 | | | 207.1701 | | | | | | | | | | 0.843878 | | | | 4678.297 | | | 5.054688 | |  |  |  |  |  |
| 16.89167 | | | 207.2584 | | | | | | | | | | 0.845694 | | | | 4678.234 | | | 5.15625 | |  |  |  |  |  |
| 16.9 | | | 207.3275 | | | | | | | | | | 0.846088 | | | | 4678.211 | | | 5.359375 | |  |  |  |  |  |
| 16.90833 | | | 207.4357 | | | | | | | | | | 0.845742 | | | | 4678.164 | | | 5.773438 | |  |  |  |  |  |
| 16.91667 | | | 207.5273 | | | | | | | | | | 0.847962 | | | | 4678.133 | | | 5.820313 | |  |  |  |  |  |
| 16.925 | | | 207.618 | | | | | | | | | | 0.848483 | | | | 4678.086 | | | 6.226563 | |  |  |  |  |  |
| 16.93333 | | | 207.6913 | | | | | | | | | | 0.848213 | | | | 4678.047 | | | 6.398438 | |  |  |  |  |  |
| 16.94167 | | | 207.7961 | | | | | | | | | | 0.8504 | | | | 4677.961 | | | 6.335938 | |  |  |  |  |  |
| 16.95 | | | 207.8669 | | | | | | | | | | 0.851077 | | | | 4677.898 | | | 6.328125 | |  |  |  |  |  |
| 16.95833 | | | 207.9552 | | | | | | | | | | 0.851067 | | | | 4677.797 | | | 6.28125 | |  |  |  |  |  |
| 16.96667 | | | 208.0642 | | | | | | | | | | 0.853687 | | | | 4677.727 | | | 6.039063 | |  |  |  |  |  |
| 16.975 | | | 208.135 | | | | | | | | | | 0.854175 | | | | 4677.641 | | | 5.945313 | |  |  |  |  |  |
| 16.98333 | | | 208.2265 | | | | | | | | | | 0.853977 | | | | 4677.563 | | | 5.851563 | |  |  |  |  |  |
| 16.99167 | | | 208.3181 | | | | | | | | | | 0.855959 | | | | 4677.508 | | | 5.648438 | |  |  |  |  |  |
| 17 | | | 208.4055 | | | | | | | | | | 0.856542 | | | | 4677.461 | | | 5.476563 | |  |  |  |  |  |
| 17.00833 | | | 208.4954 | | | | | | | | | | 0.856295 | | | | 4677.414 | | | 5.179688 | |  |  |  |  |  |
| 17.01667 | | | 208.602 | | | | | | | | | | 0.858727 | | | | 4677.391 | | | 4.851563 | |  |  |  |  |  |
| 17.025 | | | 208.6927 | | | | | | | | | | 0.858285 | | | | 4677.336 | | | 5 | |  |  |  |  |  |
| 17.03333 | | | 208.7635 | | | | | | | | | | 0.858047 | | | | 4677.32 | | | 5.15625 | |  |  |  |  |  |
| 17.04167 | | | 208.8559 | | | | | | | | | | 0.860035 | | | | 4677.297 | | | 5.125 | |  |  |  |  |  |
| 17.05 | | | 208.9491 | | | | | | | | | | 0.86063 | | | | 4677.281 | | | 5.296875 | |  |  |  |  |  |
| 17.05833 | | | 209.0399 | | | | | | | | | | 0.860647 | | | | 4677.281 | | | 5.1875 | |  |  |  |  |  |
| 17.06667 | | | 209.1289 | | | | | | | | | | 0.863052 | | | | 4677.266 | | | 5.117188 | |  |  |  |  |  |
| 17.075 | | | 209.2355 | | | | | | | | | | 0.863183 | | | | 4677.211 | | | 5.070313 | |  |  |  |  |  |
| 17.08333 | | | 209.3079 | | | | | | | | | | 0.86307 | | | | 4677.156 | | | 4.96875 | |  |  |  |  |  |
| 17.09167 | | | 209.4161 | | | | | | | | | | 0.865697 | | | | 4677.109 | | | 4.828125 | |  |  |  |  |  |
| 17.1 | | | 209.4902 | | | | | | | | | | 0.865344 | | | | 4677.055 | | | 4.953125 | |  |  |  |  |  |
| 17.10833 | | | 209.5926 | | | | | | | | | | 0.865401 | | | | 4677.016 | | | 5.046875 | |  |  |  |  |  |
| 17.11667 | | | 209.6817 | | | | | | | | | | 0.867753 | | | | 4676.984 | | | 5.023438 | |  |  |  |  |  |
| 17.125 | | | 209.7508 | | | | | | | | | | 0.8671 | | | | 4676.969 | | | 4.96875 | |  |  |  |  |  |
| 17.13333 | | | 209.8374 | | | | | | | | | | 0.866884 | | | | 4676.922 | | | 4.984375 | |  |  |  |  |  |
| 17.14167 | | | 209.9431 | | | | | | | | | | 0.869451 | | | | 4676.898 | | | 5.085938 | |  |  |  |  |  |
| 17.15 | | | 210.0164 | | | | | | | | | | 0.869269 | | | | 4676.836 | | | 5.265625 | |  |  |  |  |  |
| 17.15833 | | | 210.1046 | | | | | | | | | | 0.869055 | | | | 4676.789 | | | 5.257813 | |  |  |  |  |  |
| 17.16667 | | | 210.1937 | | | | | | | | | | 0.870948 | | | | 4676.766 | | | 5.40625 | |  |  |  |  |  |
| 17.175 | | | 210.3002 | | | | | | | | | | 0.87149 | | | | 4676.695 | | | 5.679688 | |  |  |  |  |  |
| 17.18333 | | | 210.3702 | | | | | | | | | | 0.871725 | | | | 4676.672 | | | 5.578125 | |  |  |  |  |  |
| 17.19167 | | | 210.4642 | | | | | | | | | | 0.874163 | | | | 4676.625 | | | 5.546875 | |  |  |  |  |  |
| 17.2 | | | 210.5541 | | | | | | | | | | 0.875117 | | | | 4676.547 | | | 5.78125 | |  |  |  |  |  |
| 17.20833 | | | 210.644 | | | | | | | | | | 0.87569 | | | | 4676.508 | | | 5.945313 | |  |  |  |  |  |
| 17.21667 | | | 210.7506 | | | | | | | | | | 0.878257 | | | | 4676.43 | | | 5.992188 | |  |  |  |  |  |
| 17.225 | | | 210.823 | | | | | | | | | | 0.877852 | | | | 4676.367 | | | 6.210938 | |  |  |  |  |  |
| 17.23333 | | | 210.9129 | | | | | | | | | | 0.877636 | | | | 4676.336 | | | 6.117188 | |  |  |  |  |  |
| 17.24167 | | | 211.0037 | | | | | | | | | | 0.879462 | | | | 4676.281 | | | 6.09375 | |  |  |  |  |  |
| 17.25 | | | 211.0911 | | | | | | | | | | 0.879515 | | | | 4676.211 | | | 6 | |  |  |  |  |  |
| 17.25833 | | | 211.1793 | | | | | | | | | | 0.879855 | | | | 4676.133 | | | 6.132813 | |  |  |  |  |  |
| 17.26667 | | | 211.2884 | | | | | | | | | | 0.88249 | | | | 4676.063 | | | 6.375 | |  |  |  |  |  |
| 17.275 | | | 211.3591 | | | | | | | | | | 0.881808 | | | | 4676 | | | 6.304688 | |  |  |  |  |  |
| 17.28333 | | | 211.4649 | | | | | | | | | | 0.881884 | | | | 4675.945 | | | 6.1875 | |  |  |  |  |  |
| 17.29167 | | | 211.5548 | | | | | | | | | | 0.884023 | | | | 4675.906 | | | 5.84375 | |  |  |  |  |  |
| 17.3 | | | 211.6488 | | | | | | | | | | 0.883084 | | | | 4675.859 | | | 5.734375 | |  |  |  |  |  |
| 17.30833 | | | 211.7213 | | | | | | | | | | 0.883495 | | | | 4675.797 | | | 5.609375 | |  |  |  |  |  |
| 17.31667 | | | 211.8295 | | | | | | | | | | 0.88624 | | | | 4675.695 | | | 5.851563 | |  |  |  |  |  |
| 17.325 | | | 211.9027 | | | | | | | | | | 0.885571 | | | | 4675.656 | | | 5.765625 | |  |  |  |  |  |
| 17.33333 | | | 211.9935 | | | | | | | | | | 0.885971 | | | | 4675.617 | | | 5.96875 | |  |  |  |  |  |
| 17.34167 | | | 212.0851 | | | | | | | | | | 0.887993 | | | | 4675.617 | | | 5.9375 | |  |  |  |  |  |
| 17.35 | | | 212.1939 | | | | | | | | | | 0.887441 | | | | 4675.57 | | | 5.742188 | |  |  |  |  |  |
| 17.35833 | | | 212.2638 | | | | | | | | | | 0.888439 | | | | 4675.555 | | | 5.734375 | |  |  |  |  |  |
| 17.36667 | | | 212.3526 | | | | | | | | | | 0.890837 | | | | 4675.492 | | | 5.8125 | |  |  |  |  |  |
| 17.375 | | | 212.4414 | | | | | | | | | | 0.890767 | | | | 4675.438 | | | 5.953125 | |  |  |  |  |  |
| 17.38333 | | | 212.5458 | | | | | | | | | | 0.891803 | | | | 4675.344 | | | 5.773438 | |  |  |  |  |  |
| 17.39167 | | | 212.6157 | | | | | | | | | | 0.893994 | | | | 4675.328 | | | 5.992188 | |  |  |  |  |  |
| 17.4 | | | 212.7226 | | | | | | | | | | 0.893874 | | | | 4675.297 | | | 5.8125 | |  |  |  |  |  |
| 17.40833 | | | 212.7958 | | | | | | | | | | 0.894894 | | | | 4675.273 | | | 6.007813 | |  |  |  |  |  |
| 17.41667 | | | 212.8879 | | | | | | | | | | 0.897459 | | | | 4675.211 | | | 5.875 | |  |  |  |  |  |
| 17.425 | | | 212.9915 | | | | | | | | | | 0.897149 | | | | 4675.148 | | | 6.015625 | |  |  |  |  |  |
| 17.43333 | | | 213.0605 | | | | | | | | | | 0.898096 | | | | 4675.109 | | | 5.96875 | |  |  |  |  |  |
| 17.44167 | | | 213.169 | | | | | | | | | | 0.900367 | | | | 4675.023 | | | 6.242188 | |  |  |  |  |  |
| 17.45 | | | 213.2619 | | | | | | | | | | 0.899336 | | | | 4675 | | | 6.09375 | |  |  |  |  |  |
| 17.45833 | | | 213.3532 | | | | | | | | | | 0.900449 | | | | 4674.93 | | | 6.25 | |  |  |  |  |  |
| 17.46667 | | | 213.4247 | | | | | | | | | | 0.902406 | | | | 4674.906 | | | 6.414063 | |  |  |  |  |  |
| 17.475 | | | 213.5152 | | | | | | | | | | 0.902093 | | | | 4674.836 | | | 6.460938 | |  |  |  |  |  |
| 17.48333 | | | 213.6023 | | | | | | | | | | 0.903181 | | | | 4674.797 | | | 6.429688 | |  |  |  |  |  |
| 17.49167 | | | 213.6911 | | | | | | | | | | 0.905063 | | | | 4674.727 | | | 6.515625 | |  |  |  |  |  |
| 17.5 | | | 213.7807 | | | | | | | | | | 0.904254 | | | | 4674.68 | | | 6.484375 | |  |  |  |  |  |
| 17.50833 | | | 213.8695 | | | | | | | | | | 0.904889 | | | | 4674.617 | | | 6.257813 | |  |  |  |  |  |
| 17.51667 | | | 213.9616 | | | | | | | | | | 0.906792 | | | | 4674.531 | | | 6.367188 | |  |  |  |  |  |
| 17.525 | | | 214.0537 | | | | | | | | | | 0.906311 | | | | 4674.469 | | | 6.328125 | |  |  |  |  |  |
| 17.53333 | | | 214.14 | | | | | | | | | | 0.907334 | | | | 4674.422 | | | 6.453125 | |  |  |  |  |  |
| 17.54167 | | | 214.228 | | | | | | | | | | 0.909377 | | | | 4674.359 | | | 6.523438 | |  |  |  |  |  |
| 17.55 | | | 214.3176 | | | | | | | | | | 0.908975 | | | | 4674.305 | | | 6.78125 | |  |  |  |  |  |
| 17.55833 | | | 214.4072 | | | | | | | | | | 0.909756 | | | | 4674.281 | | | 6.59375 | |  |  |  |  |  |
| 17.56667 | | | 214.4993 | | | | | | | | | | 0.911597 | | | | 4674.219 | | | 6.71875 | |  |  |  |  |  |
| 17.575 | | | 214.5856 | | | | | | | | | | 0.911089 | | | | 4674.164 | | | 6.78125 | |  |  |  |  |  |
| 17.58333 | | | 214.6934 | | | | | | | | | | 0.912747 | | | | 4674.078 | | | 6.898438 | |  |  |  |  |  |
| 17.59167 | | | 214.7846 | | | | | | | | | | 0.914901 | | | | 4674.031 | | | 6.96875 | |  |  |  |  |  |
| 17.6 | | | 214.857 | | | | | | | | | | 0.914132 | | | | 4673.938 | | | 6.945313 | |  |  |  |  |  |
| 17.60833 | | | 214.9474 | | | | | | | | | | 0.915727 | | | | 4673.906 | | | 6.960938 | |  |  |  |  |  |
| 17.61667 | | | 215.037 | | | | | | | | | | 0.917719 | | | | 4673.828 | | | 6.632813 | |  |  |  |  |  |
| 17.625 | | | 215.1242 | | | | | | | | | | 0.9165 | | | | 4673.773 | | | 6.421875 | |  |  |  |  |  |
| 17.63333 | | | 215.2113 | | | | | | | | | | 0.917913 | | | | 4673.703 | | | 6.226563 | |  |  |  |  |  |
| 17.64167 | | | 215.3018 | | | | | | | | | | 0.919766 | | | | 4673.633 | | | 6.375 | |  |  |  |  |  |
| 17.65 | | | 215.407 | | | | | | | | | | 0.918667 | | | | 4673.563 | | | 6.609375 | |  |  |  |  |  |
| 17.65833 | | | 215.4966 | | | | | | | | | | 0.921037 | | | | 4673.547 | | | 6.625 | |  |  |  |  |  |
| 17.66667 | | | 215.5665 | | | | | | | | | | 0.922386 | | | | 4673.5 | | | 6.632813 | |  |  |  |  |  |
| 17.675 | | | 215.6726 | | | | | | | | | | 0.921494 | | | | 4673.453 | | | 6.625 | |  |  |  |  |  |
| 17.68333 | | | 215.74 | | | | | | | | | | 0.922978 | | | | 4673.445 | | | 6.515625 | |  |  |  |  |  |
| 17.69167 | | | 215.8255 | | | | | | | | | | 0.924698 | | | | 4673.383 | | | 6.578125 | |  |  |  |  |  |
| 17.7 | | | 215.9324 | | | | | | | | | | 0.924083 | | | | 4673.289 | | | 6.65625 | |  |  |  |  |  |
| 17.70833 | | | 216.0236 | | | | | | | | | | 0.926467 | | | | 4673.234 | | | 6.890625 | |  |  |  |  |  |
| 17.71667 | | | 216.0952 | | | | | | | | | | 0.927861 | | | | 4673.188 | | | 6.71875 | |  |  |  |  |  |
| 17.725 | | | 216.2021 | | | | | | | | | | 0.927316 | | | | 4673.125 | | | 6.640625 | |  |  |  |  |  |
| 17.73333 | | | 216.2908 | | | | | | | | | | 0.930012 | | | | 4673.07 | | | 6.601563 | |  |  |  |  |  |
| 17.74167 | | | 216.3813 | | | | | | | | | | 0.931089 | | | | 4673 | | | 6.570313 | |  |  |  |  |  |
| 17.75 | | | 216.4725 | | | | | | | | | | 0.930433 | | | | 4672.945 | | | 6.414063 | |  |  |  |  |  |
| 17.75833 | | | 216.5433 | | | | | | | | | | 0.932274 | | | | 4672.883 | | | 6.570313 | |  |  |  |  |  |
| 17.76667 | | | 216.6353 | | | | | | | | | | 0.933503 | | | | 4672.844 | | | 6.710938 | |  |  |  |  |  |
| 17.775 | | | 216.725 | | | | | | | | | | 0.932166 | | | | 4672.805 | | | 6.5625 | |  |  |  |  |  |
| 17.78333 | | | 216.8146 | | | | | | | | | | 0.93407 | | | | 4672.758 | | | 6.75 | |  |  |  |  |  |
| 17.79167 | | | 216.9025 | | | | | | | | | | 0.935216 | | | | 4672.703 | | | 6.945313 | |  |  |  |  |  |
| 17.8 | | | 216.9946 | | | | | | | | | | 0.933764 | | | | 4672.664 | | | 7.078125 | |  |  |  |  |  |
| 17.80833 | | | 217.1015 | | | | | | | | | | 0.936032 | | | | 4672.563 | | | 7.195313 | |  |  |  |  |  |
| 17.81667 | | | 217.1969 | | | | | | | | | | 0.936524 | | | | 4672.516 | | | 7.453125 | |  |  |  |  |  |
| 17.825 | | | 217.2849 | | | | | | | | | | 0.935856 | | | | 4672.461 | | | 7.359375 | |  |  |  |  |  |
| 17.83333 | | | 217.3728 | | | | | | | | | | 0.937987 | | | | 4672.383 | | | 7.359375 | |  |  |  |  |  |
| 17.84167 | | | 217.4468 | | | | | | | | | | 0.938805 | | | | 4672.313 | | | 7.28125 | |  |  |  |  |  |
| 17.85 | | | 217.5381 | | | | | | | | | | 0.93807 | | | | 4672.242 | | | 7.34375 | |  |  |  |  |  |
| 17.85833 | | | 217.6277 | | | | | | | | | | 0.940633 | | | | 4672.148 | | | 7.773438 | |  |  |  |  |  |
| 17.86667 | | | 217.7371 | | | | | | | | | | 0.94133 | | | | 4672.039 | | | 7.742188 | |  |  |  |  |  |
| 17.875 | | | 217.8094 | | | | | | | | | | 0.940872 | | | | 4672 | | | 7.578125 | |  |  |  |  |  |
| 17.88333 | | | 217.9138 | | | | | | | | | | 0.943826 | | | | 4671.938 | | | 7.671875 | |  |  |  |  |  |
| 17.89167 | | | 218.0026 | | | | | | | | | | 0.944385 | | | | 4671.891 | | | 7.757813 | |  |  |  |  |  |
| 17.9 | | | 218.0889 | | | | | | | | | | 0.943992 | | | | 4671.82 | | | 7.742188 | |  |  |  |  |  |
| 17.90833 | | | 218.1629 | | | | | | | | | | 0.946357 | | | | 4671.711 | | | 7.898438 | |  |  |  |  |  |
| 17.91667 | | | 218.2526 | | | | | | | | | | 0.947342 | | | | 4671.648 | | | 8.140625 | |  |  |  |  |  |
| 17.925 | | | 218.3578 | | | | | | | | | | 0.947041 | | | | 4671.594 | | | 8.25 | |  |  |  |  |  |
| 17.93333 | | | 218.4458 | | | | | | | | | | 0.949357 | | | | 4671.523 | | | 8.203125 | |  |  |  |  |  |
| 17.94167 | | | 218.5214 | | | | | | | | | | 0.950012 | | | | 4671.469 | | | 8 | |  |  |  |  |  |
| 17.95 | | | 218.611 | | | | | | | | | | 0.949318 | | | | 4671.398 | | | 7.867188 | |  |  |  |  |  |
| 17.95833 | | | 218.7023 | | | | | | | | | | 0.952358 | | | | 4671.305 | | | 7.960938 | |  |  |  |  |  |
| 17.96667 | | | 218.7944 | | | | | | | | | | 0.953432 | | | | 4671.203 | | | 7.9375 | |  |  |  |  |  |
| 17.975 | | | 218.9054 | | | | | | | | | | 0.953507 | | | | 4671.102 | | | 8.164063 | |  |  |  |  |  |
| 17.98333 | | | 218.9761 | | | | | | | | | | 0.955785 | | | | 4671.063 | | | 8.132813 | |  |  |  |  |  |
| 17.99167 | | | 219.0838 | | | | | | | | | | 0.956049 | | | | 4671.016 | | | 8.117188 | |  |  |  |  |  |
| 18 | | | 219.1586 | | | | | | | | | | 0.9561 | | | | 4670.969 | | | 8.078125 | |  |  |  |  |  |
| 18.00833 | | | 219.249 | | | | | | | | | | 0.958602 | | | | 4670.891 | | | 7.992188 | |  |  |  |  |  |
| 18.01667 | | | 219.3584 | | | | | | | | | | 0.958995 | | | | 4670.805 | | | 8.15625 | |  |  |  |  |  |
| 18.025 | | | 219.4464 | | | | | | | | | | 0.95942 | | | | 4670.734 | | | 8.25 | |  |  |  |  |  |
| 18.03333 | | | 219.5171 | | | | | | | | | | 0.961563 | | | | 4670.695 | | | 8.226563 | |  |  |  |  |  |
| 18.04167 | | | 219.6067 | | | | | | | | | | 0.961854 | | | | 4670.617 | | | 8.210938 | |  |  |  |  |  |
| 18.05 | | | 219.7152 | | | | | | | | | | 0.961677 | | | | 4670.547 | | | 8.117188 | |  |  |  |  |  |
| 18.05833 | | | 219.7859 | | | | | | | | | | 0.963974 | | | | 4670.5 | | | 8.03125 | |  |  |  |  |  |
| 18.06667 | | | 219.8764 | | | | | | | | | | 0.964343 | | | | 4670.398 | | | 7.882813 | |  |  |  |  |  |
| 18.075 | | | 219.9668 | | | | | | | | | | 0.96413 | | | | 4670.344 | | | 7.726563 | |  |  |  |  |  |
| 18.08333 | | | 220.0729 | | | | | | | | | | 0.966787 | | | | 4670.25 | | | 7.59375 | |  |  |  |  |  |
| 18.09167 | | | 220.1436 | | | | | | | | | | 0.966835 | | | | 4670.203 | | | 7.625 | |  |  |  |  |  |
| 18.1 | | | 220.2324 | | | | | | | | | | 0.966328 | | | | 4670.148 | | | 7.601563 | |  |  |  |  |  |
| 18.10833 | | | 220.3212 | | | | | | | | | | 0.968806 | | | | 4670.094 | | | 7.679688 | |  |  |  |  |  |
| 18.11667 | | | 220.4099 | | | | | | | | | | 0.968852 | | | | 4670.047 | | | 7.8125 | |  |  |  |  |  |
| 18.125 | | | 220.5004 | | | | | | | | | | 0.968604 | | | | 4670.008 | | | 8.101563 | |  |  |  |  |  |
| 18.13333 | | | 220.6064 | | | | | | | | | | 0.971963 | | | | 4669.961 | | | 8.09375 | |  |  |  |  |  |
| 18.14167 | | | 220.6961 | | | | | | | | | | 0.972267 | | | | 4669.891 | | | 8.164063 | |  |  |  |  |  |
| 18.15 | | | 220.7668 | | | | | | | | | | 0.972196 | | | | 4669.844 | | | 8.273438 | |  |  |  |  |  |
| 18.15833 | | | 220.872 | | | | | | | | | | 0.9751 | | | | 4669.742 | | | 8.476563 | |  |  |  |  |  |
| 18.16667 | | | 220.9435 | | | | | | | | | | 0.975224 | | | | 4669.68 | | | 8.601563 | |  |  |  |  |  |
| 18.175 | | | 221.0315 | | | | | | | | | | 0.9754 | | | | 4669.586 | | | 8.835938 | |  |  |  |  |  |
| 18.18333 | | | 221.1211 | | | | | | | | | | 0.97902 | | | | 4669.508 | | | 8.914063 | |  |  |  |  |  |
| 18.19167 | | | 221.2313 | | | | | | | | | | 0.980104 | | | | 4669.422 | | | 8.953125 | |  |  |  |  |  |
| 18.2 | | | 221.3045 | | | | | | | | | | 0.980251 | | | | 4669.344 | | | 8.898438 | |  |  |  |  |  |
| 18.20833 | | | 221.4097 | | | | | | | | | | 0.983268 | | | | 4669.227 | | | 8.5625 | |  |  |  |  |  |
| 18.21667 | | | 221.4985 | | | | | | | | | | 0.982872 | | | | 4669.109 | | | 8.695313 | |  |  |  |  |  |
| 18.225 | | | 221.5881 | | | | | | | | | | 0.982936 | | | | 4669.023 | | | 8.742188 | |  |  |  |  |  |
| 18.23333 | | | 221.6613 | | | | | | | | | | 0.984892 | | | | 4668.961 | | | 8.640625 | |  |  |  |  |  |
| 18.24167 | | | 221.7509 | | | | | | | | | | 0.984269 | | | | 4668.891 | | | 8.609375 | |  |  |  |  |  |
| 18.25 | | | 221.843 | | | | | | | | | | 0.984254 | | | | 4668.82 | | | 8.492188 | |  |  |  |  |  |
| 18.25833 | | | 221.9581 | | | | | | | | | | 0.986669 | | | | 4668.773 | | | 8.414063 | |  |  |  |  |  |
| 18.26667 | | | 222.0288 | | | | | | | | | | 0.986149 | | | | 4668.703 | | | 8.460938 | |  |  |  |  |  |
| 18.275 | | | 222.139 | | | | | | | | | | 0.986576 | | | | 4668.609 | | | 8.851563 | |  |  |  |  |  |
| 18.28333 | | | 222.2113 | | | | | | | | | | 0.988242 | | | | 4668.555 | | | 8.859375 | |  |  |  |  |  |
| 18.29167 | | | 222.319 | | | | | | | | | | 0.987576 | | | | 4668.484 | | | 9.046875 | |  |  |  |  |  |
| 18.3 | | | 222.4103 | | | | | | | | | | 0.988497 | | | | 4668.438 | | | 9 | |  |  |  |  |  |
| 18.30833 | | | 222.4835 | | | | | | | | | | 0.990267 | | | | 4668.367 | | | 9.085938 | |  |  |  |  |  |
| 18.31667 | | | 222.5739 | | | | | | | | | | 0.990149 | | | | 4668.297 | | | 8.976563 | |  |  |  |  |  |
| 18.325 | | | 222.6652 | | | | | | | | | | 0.991317 | | | | 4668.172 | | | 9.1875 | |  |  |  |  |  |
| 18.33333 | | | 222.7548 | | | | | | | | | | 0.993472 | | | | 4668.094 | | | 9.265625 | |  |  |  |  |  |
| 18.34167 | | | 222.8411 | | | | | | | | | | 0.993172 | | | | 4668.008 | | | 9.242188 | |  |  |  |  |  |
| 18.35 | | | 222.9513 | | | | | | | | | | 0.994654 | | | | 4667.914 | | | 9.296875 | |  |  |  |  |  |
| 18.35833 | | | 223.022 | | | | | | | | | | 0.995924 | | | | 4667.852 | | | 9.140625 | |  |  |  |  |  |
| 18.36667 | | | 223.1108 | | | | | | | | | | 0.995435 | | | | 4667.789 | | | 9.148438 | |  |  |  |  |  |
| 18.375 | | | 223.2037 | | | | | | | | | | 0.996416 | | | | 4667.688 | | | 9.296875 | |  |  |  |  |  |
| 18.38333 | | | 223.3122 | | | | | | | | | | 0.998516 | | | | 4667.57 | | | 9.390625 | |  |  |  |  |  |
| 18.39167 | | | 223.4019 | | | | | | | | | | 0.998459 | | | | 4667.5 | | | 9.679688 | |  |  |  |  |  |
| 18.4 | | | 223.4734 | | | | | | | | | | 0.999295 | | | | 4667.445 | | | 9.796875 | |  |  |  |  |  |
| 18.40833 | | | 223.5836 | | | | | | | | | | 1.001455 | | | | 4667.375 | | | 9.890625 | |  |  |  |  |  |
| 18.41667 | | | 223.6707 | | | | | | | | | | 1.001235 | | | | 4667.289 | | | 9.765625 | |  |  |  |  |  |
| 18.425 | | | 223.7447 | | | | | | | | | | 1.002063 | | | | 4667.203 | | | 9.90625 | |  |  |  |  |  |
| 18.43333 | | | 223.8343 | | | | | | | | | | 1.004199 | | | | 4667.102 | | | 9.9375 | |  |  |  |  |  |
| 18.44167 | | | 223.9437 | | | | | | | | | | 1.003856 | | | | 4666.977 | | | 9.890625 | |  |  |  |  |  |
| 18.45 | | | 224.0366 | | | | | | | | | | 1.005477 | | | | 4666.875 | | | 10.00781 | |  |  |  |  |  |
| 18.45833 | | | 224.1254 | | | | | | | | | | 1.006862 | | | | 4666.789 | | | 9.945313 | |  |  |  |  |  |
| 18.46667 | | | 224.1969 | | | | | | | | | | 1.006522 | | | | 4666.742 | | | 9.882813 | |  |  |  |  |  |
| 18.475 | | | 224.3079 | | | | | | | | | | 1.008148 | | | | 4666.641 | | | 9.429688 | |  |  |  |  |  |
| 18.48333 | | | 224.4024 | | | | | | | | | | 1.009323 | | | | 4666.563 | | | 9.484375 | |  |  |  |  |  |
| 18.49167 | | | 224.4953 | | | | | | | | | | 1.009191 | | | | 4666.477 | | | 9.382813 | |  |  |  |  |  |
| 18.5 | | | 224.5693 | | | | | | | | | | 1.010536 | | | | 4666.398 | | | 9.476563 | |  |  |  |  |  |
| 18.50833 | | | 224.6795 | | | | | | | | | | 1.011964 | | | | 4666.305 | | | 9.523438 | |  |  |  |  |  |
| 18.51667 | | | 224.7708 | | | | | | | | | | 1.012333 | | | | 4666.25 | | | 9.546875 | |  |  |  |  |  |
| 18.525 | | | 224.8629 | | | | | | | | | | 1.014516 | | | | 4666.219 | | | 9.460938 | |  |  |  |  |  |
| 18.53333 | | | 224.9533 | | | | | | | | | | 1.014877 | | | | 4666.125 | | | 9.4375 | |  |  |  |  |  |
| 18.54167 | | | 225.0454 | | | | | | | | | | 1.015395 | | | | 4666.063 | | | 9.492188 | |  |  |  |  |  |
| 18.55 | | | 225.1366 | | | | | | | | | | 1.017978 | | | | 4665.969 | | | 9.796875 | |  |  |  |  |  |
| 18.55833 | | | 225.2271 | | | | | | | | | | 1.01852 | | | | 4665.883 | | | 9.914063 | |  |  |  |  |  |
| 18.56667 | | | 225.3142 | | | | | | | | | | 1.019289 | | | | 4665.797 | | | 9.84375 | |  |  |  |  |  |
| 18.575 | | | 225.3997 | | | | | | | | | | 1.021711 | | | | 4665.734 | | | 10.09375 | |  |  |  |  |  |
| 18.58333 | | | 225.4918 | | | | | | | | | | 1.022452 | | | | 4665.656 | | | 10.19531 | |  |  |  |  |  |
| 18.59167 | | | 225.5798 | | | | | | | | | | 1.022779 | | | | 4665.57 | | | 10.25781 | |  |  |  |  |  |
| 18.6 | | | 225.6686 | | | | | | | | | | 1.024642 | | | | 4665.445 | | | 10.42969 | |  |  |  |  |  |
| 18.60833 | | | 225.7574 | | | | | | | | | | 1.025196 | | | | 4665.352 | | | 10.57031 | |  |  |  |  |  |
| 18.61667 | | | 225.8462 | | | | | | | | | | 1.025552 | | | | 4665.266 | | | 10.48438 | |  |  |  |  |  |
| 18.625 | | | 225.935 | | | | | | | | | | 1.02765 | | | | 4665.164 | | | 10.4375 | |  |  |  |  |  |
| 18.63333 | | | 226.0262 | | | | | | | | | | 1.02795 | | | | 4665.063 | | | 10.35938 | |  |  |  |  |  |
| 18.64167 | | | 226.115 | | | | | | | | | | 1.028129 | | | | 4664.969 | | | 10.38281 | |  |  |  |  |  |
| 18.65 | | | 226.2071 | | | | | | | | | | 1.030017 | | | | 4664.859 | | | 10.35938 | |  |  |  |  |  |
| 18.65833 | | | 226.2992 | | | | | | | | | | 1.029841 | | | | 4664.758 | | | 10.33594 | |  |  |  |  |  |
| 18.66667 | | | 226.3872 | | | | | | | | | | 1.030108 | | | | 4664.68 | | | 10.35938 | |  |  |  |  |  |
| 18.675 | | | 226.4784 | | | | | | | | | | 1.032257 | | | | 4664.602 | | | 10.20313 | |  |  |  |  |  |
| 18.68333 | | | 226.5672 | | | | | | | | | | 1.032767 | | | | 4664.516 | | | 10.14063 | |  |  |  |  |  |
| 18.69167 | | | 226.656 | | | | | | | | | | 1.033973 | | | | 4664.43 | | | 10.125 | |  |  |  |  |  |
| 18.7 | | | 226.7473 | | | | | | | | | | 1.036817 | | | | 4664.344 | | | 10.22656 | |  |  |  |  |  |
| 18.70833 | | | 226.841 | | | | | | | | | | 1.037347 | | | | 4664.266 | | | 10.42188 | |  |  |  |  |  |
| 18.71667 | | | 226.9306 | | | | | | | | | | 1.038498 | | | | 4664.18 | | | 10.46875 | |  |  |  |  |  |
| 18.725 | | | 227.0202 | | | | | | | | | | 1.040881 | | | | 4664.117 | | | 10.57031 | |  |  |  |  |  |
| 18.73333 | | | 227.1099 | | | | | | | | | | 1.041546 | | | | 4664.039 | | | 10.82031 | |  |  |  |  |  |
| 18.74167 | | | 227.2011 | | | | | | | | | | 1.042064 | | | | 4663.961 | | | 10.72656 | |  |  |  |  |  |
| 18.75 | | | 227.2965 | | | | | | | | | | 1.044327 | | | | 4663.859 | | | 10.76563 | |  |  |  |  |  |
| 18.75833 | | | 227.3878 | | | | | | | | | | 1.044903 | | | | 4663.742 | | | 10.89063 | |  |  |  |  |  |
| 18.76667 | | | 227.4798 | | | | | | | | | | 1.045059 | | | | 4663.648 | | | 11.04688 | |  |  |  |  |  |
| 18.775 | | | 227.5728 | | | | | | | | | | 1.046687 | | | | 4663.547 | | | 11.11719 | |  |  |  |  |  |
| 18.78333 | | | 227.6656 | | | | | | | | | | 1.046597 | | | | 4663.422 | | | 11.17188 | |  |  |  |  |  |
| 18.79167 | | | 227.7586 | | | | | | | | | | 1.046813 | | | | 4663.344 | | | 10.96875 | |  |  |  |  |  |
| 18.8 | | | 227.8482 | | | | | | | | | | 1.048599 | | | | 4663.242 | | | 11.07031 | |  |  |  |  |  |
| 18.80833 | | | 227.9419 | | | | | | | | | | 1.048478 | | | | 4663.141 | | | 11.02344 | |  |  |  |  |  |
| 18.81667 | | | 228.034 | | | | | | | | | | 1.048886 | | | | 4663.023 | | | 11.02344 | |  |  |  |  |  |
| 18.825 | | | 228.1261 | | | | | | | | | | 1.050913 | | | | 4662.93 | | | 11.29688 | |  |  |  |  |  |
| 18.83333 | | | 228.2149 | | | | | | | | | | 1.050588 | | | | 4662.828 | | | 11.24219 | |  |  |  |  |  |
| 18.84167 | | | 228.307 | | | | | | | | | | 1.051651 | | | | 4662.766 | | | 11.46094 | |  |  |  |  |  |
| 18.85 | | | 228.4023 | | | | | | | | | | 1.054159 | | | | 4662.656 | | | 11.32031 | |  |  |  |  |  |
| 18.85833 | | | 228.4952 | | | | | | | | | | 1.054189 | | | | 4662.57 | | | 11.36719 | |  |  |  |  |  |
| 18.86667 | | | 228.5873 | | | | | | | | | | 1.055789 | | | | 4662.492 | | | 11.45313 | |  |  |  |  |  |
| 18.875 | | | 228.6794 | | | | | | | | | | 1.057515 | | | | 4662.367 | | | 11.67969 | |  |  |  |  |  |
| 18.88333 | | | 228.7731 | | | | | | | | | | 1.056517 | | | | 4662.273 | | | 11.78125 | |  |  |  |  |  |
| 18.89167 | | | 228.8652 | | | | | | | | | | 1.057576 | | | | 4662.156 | | | 11.82813 | |  |  |  |  |  |
| 18.9 | | | 228.9573 | | | | | | | | | | 1.059445 | | | | 4662.078 | | | 12.15625 | |  |  |  |  |  |
| 18.90833 | | | 229.0494 | | | | | | | | | | 1.058803 | | | | 4661.977 | | | 12.24219 | |  |  |  |  |  |
| 18.91667 | | | 229.1456 | | | | | | | | | | 1.060383 | | | | 4661.875 | | | 12.35156 | |  |  |  |  |  |
| 18.925 | | | 229.236 | | | | | | | | | | 1.062219 | | | | 4661.75 | | | 12.39063 | |  |  |  |  |  |
| 18.93333 | | | 229.3264 | | | | | | | | | | 1.061329 | | | | 4661.633 | | | 12.63281 | |  |  |  |  |  |
| 18.94167 | | | 229.4185 | | | | | | | | | | 1.062834 | | | | 4661.523 | | | 12.67969 | |  |  |  |  |  |
| 18.95 | | | 229.5114 | | | | | | | | | | 1.064647 | | | | 4661.383 | | | 12.71875 | |  |  |  |  |  |
| 18.95833 | | | 229.597 | | | | | | | | | | 1.064059 | | | | 4661.266 | | | 12.75 | |  |  |  |  |  |
| 18.96667 | | | 229.689 | | | | | | | | | | 1.06633 | | | | 4661.148 | | | 12.53906 | |  |  |  |  |  |
| 18.975 | | | 229.7811 | | | | | | | | | | 1.068908 | | | | 4661.047 | | | 12.70313 | |  |  |  |  |  |
| 18.98333 | | | 229.8732 | | | | | | | | | | 1.069069 | | | | 4660.906 | | | 12.71875 | |  |  |  |  |  |
| 18.99167 | | | 229.9645 | | | | | | | | | | 1.07193 | | | | 4660.797 | | | 12.6875 | |  |  |  |  |  |
| 19 | | | 230.0533 | | | | | | | | | | 1.074081 | | | | 4660.68 | | | 12.48438 | |  |  |  |  |  |
| 19.00833 | | | 230.1456 | | | | | | | | | | 1.073409 | | | | 4660.578 | | | 12.71094 | |  |  |  |  |  |
| 19.01667 | | | 230.233 | | | | | | | | | | 1.075749 | | | | 4660.492 | | | 12.4375 | |  |  |  |  |  |
| 19.025 | | | 230.3204 | | | | | | | | | | 1.077431 | | | | 4660.367 | | | 12.53125 | |  |  |  |  |  |
| 19.03333 | | | 230.4061 | | | | | | | | | | 1.076736 | | | | 4660.266 | | | 12.59375 | |  |  |  |  |  |
| 19.04167 | | | 230.4991 | | | | | | | | | | 1.079385 | | | | 4660.164 | | | 12.54688 | |  |  |  |  |  |
| 19.05 | | | 230.5873 | | | | | | | | | | 1.081193 | | | | 4660.086 | | | 12.35156 | |  |  |  |  |  |
| 19.05833 | | | 230.673 | | | | | | | | | | 1.080989 | | | | 4659.961 | | | 12.20313 | |  |  |  |  |  |
| 19.06667 | | | 230.7588 | | | | | | | | | | 1.083262 | | | | 4659.898 | | | 12.04688 | |  |  |  |  |  |
| 19.075 | | | 230.847 | | | | | | | | | | 1.085473 | | | | 4659.781 | | | 11.94531 | |  |  |  |  |  |
| 19.08333 | | | 230.9352 | | | | | | | | | | 1.085376 | | | | 4659.672 | | | 12.09375 | |  |  |  |  |  |
| 19.09167 | | | 231.0249 | | | | | | | | | | 1.087906 | | | | 4659.578 | | | 12.11719 | |  |  |  |  |  |
| 19.1 | | | 231.1123 | | | | | | | | | | 1.090028 | | | | 4659.508 | | | 12.20313 | |  |  |  |  |  |
| 19.10833 | | | 231.1981 | | | | | | | | | | 1.090142 | | | | 4659.422 | | | 12.33594 | |  |  |  |  |  |
| 19.11667 | | | 231.2879 | | | | | | | | | | 1.092648 | | | | 4659.344 | | | 12.33594 | |  |  |  |  |  |
| 19.125 | | | 231.376 | | | | | | | | | | 1.09405 | | | | 4659.258 | | | 12.70313 | |  |  |  |  |  |
| 19.13333 | | | 231.461 | | | | | | | | | | 1.093379 | | | | 4659.133 | | | 12.85156 | |  |  |  |  |  |
| 19.14167 | | | 231.55 | | | | | | | | | | 1.095298 | | | | 4659.031 | | | 12.91406 | |  |  |  |  |  |
| 19.15 | | | 231.6414 | | | | | | | | | | 1.096639 | | | | 4658.914 | | | 12.92969 | |  |  |  |  |  |
| 19.15833 | | | 231.7295 | | | | | | | | | | 1.095829 | | | | 4658.797 | | | 12.92969 | |  |  |  |  |  |
| 19.16667 | | | 231.8169 | | | | | | | | | | 1.098045 | | | | 4658.688 | | | 13.08594 | |  |  |  |  |  |
| 19.175 | | | 231.9011 | | | | | | | | | | 1.099297 | | | | 4658.539 | | | 13.14063 | |  |  |  |  |  |
| 19.18333 | | | 231.9917 | | | | | | | | | | 1.098662 | | | | 4658.406 | | | 13.22656 | |  |  |  |  |  |
| 19.19167 | | | 232.0838 | | | | | | | | | | 1.1008 | | | | 4658.281 | | | 13.30469 | |  |  |  |  |  |
| 19.2 | | | 232.172 | | | | | | | | | | 1.102044 | | | | 4658.18 | | | 13.30469 | |  |  |  |  |  |
| 19.20833 | | | 232.2594 | | | | | | | | | | 1.101972 | | | | 4658.078 | | | 13.25781 | |  |  |  |  |  |
| 19.21667 | | | 232.3516 | | | | | | | | | | 1.104996 | | | | 4657.953 | | | 13.52344 | |  |  |  |  |  |
| 19.225 | | | 232.4438 | | | | | | | | | | 1.106921 | | | | 4657.828 | | | 13.32813 | |  |  |  |  |  |
| 19.23333 | | | 232.5327 | | | | | | | | | | 1.106653 | | | | 4657.711 | | | 13.51563 | |  |  |  |  |  |
| 19.24167 | | | 232.6225 | | | | | | | | | | 1.10996 | | | | 4657.586 | | | 13.75781 | |  |  |  |  |  |
| 19.25 | | | 232.7107 | | | | | | | | | | 1.111342 | | | | 4657.484 | | | 13.625 | |  |  |  |  |  |
| 19.25833 | | | 232.7997 | | | | | | | | | | 1.111315 | | | | 4657.383 | | | 13.72656 | |  |  |  |  |  |
| 19.26667 | | | 232.8894 | | | | | | | | | | 1.114714 | | | | 4657.227 | | | 13.78125 | |  |  |  |  |  |
| 19.275 | | | 232.9784 | | | | | | | | | | 1.115672 | | | | 4657.141 | | | 13.97656 | |  |  |  |  |  |
| 19.28333 | | | 233.0714 | | | | | | | | | | 1.115412 | | | | 4657 | | | 14.16406 | |  |  |  |  |  |
| 19.29167 | | | 233.1604 | | | | | | | | | | 1.118456 | | | | 4656.859 | | | 14.08594 | |  |  |  |  |  |
| 19.3 | | | 233.2509 | | | | | | | | | | 1.119277 | | | | 4656.758 | | | 14.125 | |  |  |  |  |  |
| 19.30833 | | | 233.3407 | | | | | | | | | | 1.119117 | | | | 4656.633 | | | 14.35938 | |  |  |  |  |  |
| 19.31667 | | | 233.4289 | | | | | | | | | | 1.122589 | | | | 4656.508 | | | 14.51563 | |  |  |  |  |  |
| 19.325 | | | 233.5179 | | | | | | | | | | 1.123317 | | | | 4656.359 | | | 14.5625 | |  |  |  |  |  |
| 19.33333 | | | 233.6093 | | | | | | | | | | 1.123018 | | | | 4656.219 | | | 14.77344 | |  |  |  |  |  |
| 19.34167 | | | 233.7014 | | | | | | | | | | 1.126574 | | | | 4656.102 | | | 14.91406 | |  |  |  |  |  |
| 19.35 | | | 233.792 | | | | | | | | | | 1.127416 | | | | 4655.984 | | | 15.24219 | |  |  |  |  |  |
| 19.35833 | | | 233.8818 | | | | | | | | | | 1.128266 | | | | 4655.836 | | | 15.03125 | |  |  |  |  |  |
| 19.36667 | | | 233.9668 | | | | | | | | | | 1.132412 | | | | 4655.695 | | | 15.125 | |  |  |  |  |  |
| 19.375 | | | 234.0589 | | | | | | | | | | 1.133653 | | | | 4655.57 | | | 15.21094 | |  |  |  |  |  |
| 19.38333 | | | 234.1479 | | | | | | | | | | 1.133753 | | | | 4655.414 | | | 15.39063 | |  |  |  |  |  |
| 19.39167 | | | 234.2369 | | | | | | | | | | 1.137297 | | | | 4655.266 | | | 15.61719 | |  |  |  |  |  |
| 19.4 | | | 234.3283 | | | | | | | | | | 1.137979 | | | | 4655.109 | | | 15.67969 | |  |  |  |  |  |
| 19.40833 | | | 234.4196 | | | | | | | | | | 1.138392 | | | | 4655.016 | | | 15.92188 | |  |  |  |  |  |
| 19.41667 | | | 234.5094 | | | | | | | | | | 1.142035 | | | | 4654.875 | | | 15.89844 | |  |  |  |  |  |
| 19.425 | | | 234.6008 | | | | | | | | | | 1.142606 | | | | 4654.734 | | | 15.92188 | |  |  |  |  |  |
| 19.43333 | | | 234.6874 | | | | | | | | | | 1.143339 | | | | 4654.586 | | | 15.92188 | |  |  |  |  |  |
| 19.44167 | | | 234.7772 | | | | | | | | | | 1.146833 | | | | 4654.43 | | | 16.0625 | |  |  |  |  |  |
| 19.45 | | | 234.8701 | | | | | | | | | | 1.147143 | | | | 4654.297 | | | 16.10156 | |  |  |  |  |  |
| 19.45833 | | | 234.9599 | | | | | | | | | | 1.148165 | | | | 4654.141 | | | 16.25781 | |  |  |  |  |  |
| 19.46667 | | | 235.0505 | | | | | | | | | | 1.151694 | | | | 4654.008 | | | 16.36719 | |  |  |  |  |  |
| 19.475 | | | 235.1395 | | | | | | | | | | 1.15189 | | | | 4653.883 | | | 16.53906 | |  |  |  |  |  |
| 19.48333 | | | 235.2301 | | | | | | | | | | 1.152713 | | | | 4653.75 | | | 16.61719 | |  |  |  |  |  |
| 19.49167 | | | 235.3214 | | | | | | | | | | 1.156017 | | | | 4653.602 | | | 16.69531 | |  |  |  |  |  |
| 19.5 | | | 235.4104 | | | | | | | | | | 1.15633 | | | | 4653.461 | | | 16.78125 | |  |  |  |  |  |
| 19.50833 | | | 235.5002 | | | | | | | | | | 1.157502 | | | | 4653.297 | | | 16.89063 | |  |  |  |  |  |
| 19.51667 | | | 235.5932 | | | | | | | | | | 1.160659 | | | | 4653.156 | | | 17 | |  |  |  |  |  |
| 19.525 | | | 235.6846 | | | | | | | | | | 1.160718 | | | | 4652.992 | | | 17.0625 | |  |  |  |  |  |
| 19.53333 | | | 235.7743 | | | | | | | | | | 1.162103 | | | | 4652.844 | | | 17.07813 | |  |  |  |  |  |
| 19.54167 | | | 235.8625 | | | | | | | | | | 1.165241 | | | | 4652.688 | | | 17.04688 | |  |  |  |  |  |
| 19.55 | | | 235.9507 | | | | | | | | | | 1.165248 | | | | 4652.547 | | | 17.27344 | |  |  |  |  |  |
| 19.55833 | | | 236.0388 | | | | | | | | | | 1.166255 | | | | 4652.391 | | | 17.17969 | |  |  |  |  |  |
| 19.56667 | | | 236.1262 | | | | | | | | | | 1.169879 | | | | 4652.242 | | | 17.10156 | |  |  |  |  |  |
| 19.575 | | | 236.2104 | | | | | | | | | | 1.16951 | | | | 4652.086 | | | 17.03125 | |  |  |  |  |  |
| 19.58333 | | | 236.301 | | | | | | | | | | 1.170306 | | | | 4651.945 | | | 17.07813 | |  |  |  |  |  |
| 19.59167 | | | 236.3883 | | | | | | | | | | 1.173441 | | | | 4651.813 | | | 17.14844 | |  |  |  |  |  |
| 19.6 | | | 236.4765 | | | | | | | | | | 1.173345 | | | | 4651.641 | | | 17.53906 | |  |  |  |  |  |
| 19.60833 | | | 236.5671 | | | | | | | | | | 1.174366 | | | | 4651.523 | | | 17.5625 | |  |  |  |  |  |
| 19.61667 | | | 236.6545 | | | | | | | | | | 1.177098 | | | | 4651.383 | | | 17.59375 | |  |  |  |  |  |
| 19.625 | | | 236.745 | | | | | | | | | | 1.176371 | | | | 4651.258 | | | 17.6875 | |  |  |  |  |  |
| 19.63333 | | | 236.838 | | | | | | | | | | 1.177499 | | | | 4651.109 | | | 17.5625 | |  |  |  |  |  |
| 19.64167 | | | 236.931 | | | | | | | | | | 1.180583 | | | | 4650.953 | | | 17.60156 | |  |  |  |  |  |
| 19.65 | | | 237.0232 | | | | | | | | | | 1.180593 | | | | 4650.766 | | | 17.61719 | |  |  |  |  |  |
| 19.65833 | | | 237.1138 | | | | | | | | | | 1.182668 | | | | 4650.617 | | | 17.66406 | |  |  |  |  |  |
| 19.66667 | | | 237.2027 | | | | | | | | | | 1.185331 | | | | 4650.461 | | | 18.07031 | |  |  |  |  |  |
| 19.675 | | | 237.2941 | | | | | | | | | | 1.185595 | | | | 4650.305 | | | 18.05469 | |  |  |  |  |  |
| 19.68333 | | | 237.3855 | | | | | | | | | | 1.187849 | | | | 4650.172 | | | 17.85938 | |  |  |  |  |  |
| 19.69167 | | | 237.4737 | | | | | | | | | | 1.1902 | | | | 4650.031 | | | 18.10938 | |  |  |  |  |  |
| 19.7 | | | 237.5635 | | | | | | | | | | 1.190792 | | | | 4649.883 | | | 18.1875 | |  |  |  |  |  |
| 19.70833 | | | 237.6556 | | | | | | | | | | 1.193249 | | | | 4649.719 | | | 18.28906 | |  |  |  |  |  |
| 19.71667 | | | 237.743 | | | | | | | | | | 1.195204 | | | | 4649.523 | | | 18.35156 | |  |  |  |  |  |
| 19.725 | | | 237.8296 | | | | | | | | | | 1.195138 | | | | 4649.375 | | | 18.67188 | |  |  |  |  |  |
| 19.73333 | | | 237.9186 | | | | | | | | | | 1.197149 | | | | 4649.25 | | | 18.75 | |  |  |  |  |  |
| 19.74167 | | | 238.0116 | | | | | | | | | | 1.199089 | | | | 4649.063 | | | 18.9375 | |  |  |  |  |  |
| 19.75 | | | 238.1013 | | | | | | | | | | 1.199047 | | | | 4648.898 | | | 18.95313 | |  |  |  |  |  |
| 19.75833 | | | 238.1919 | | | | | | | | | | 1.201012 | | | | 4648.742 | | | 19.38281 | |  |  |  |  |  |
| 19.76667 | | | 238.2809 | | | | | | | | | | 1.203256 | | | | 4648.586 | | | 19.59375 | |  |  |  |  |  |
| 19.775 | | | 238.3715 | | | | | | | | | | 1.203707 | | | | 4648.383 | | | 19.32813 | |  |  |  |  |  |
| 19.78333 | | | 238.4652 | | | | | | | | | | 1.206276 | | | | 4648.219 | | | 19.66406 | |  |  |  |  |  |
| 19.79167 | | | 238.5534 | | | | | | | | | | 1.208846 | | | | 4648.031 | | | 19.89063 | |  |  |  |  |  |
| 19.8 | | | 238.6448 | | | | | | | | | | 1.209503 | | | | 4647.875 | | | 19.9375 | |  |  |  |  |  |
| 19.80833 | | | 238.737 | | | | | | | | | | 1.212252 | | | | 4647.656 | | | 19.92188 | |  |  |  |  |  |
| 19.81667 | | | 238.83 | | | | | | | | | | 1.214621 | | | | 4647.469 | | | 20.0625 | |  |  |  |  |  |
| 19.825 | | | 238.9213 | | | | | | | | | | 1.215353 | | | | 4647.336 | | | 20.33594 | |  |  |  |  |  |
| 19.83333 | | | 239.0151 | | | | | | | | | | 1.218547 | | | | 4647.141 | | | 20.40625 | |  |  |  |  |  |
| 19.84167 | | | 239.1041 | | | | | | | | | | 1.220532 | | | | 4646.945 | | | 20.21094 | |  |  |  |  |  |
| 19.85 | | | 239.1955 | | | | | | | | | | 1.221583 | | | | 4646.766 | | | 20.17188 | |  |  |  |  |  |
| 19.85833 | | | 239.2861 | | | | | | | | | | 1.22442 | | | | 4646.609 | | | 20.5 | |  |  |  |  |  |
| 19.86667 | | | 239.3774 | | | | | | | | | | 1.225882 | | | | 4646.422 | | | 20.46875 | |  |  |  |  |  |
| 19.875 | | | 239.4672 | | | | | | | | | | 1.226933 | | | | 4646.219 | | | 20.50781 | |  |  |  |  |  |
| 19.88333 | | | 239.5586 | | | | | | | | | | 1.229872 | | | | 4646.039 | | | 20.4375 | |  |  |  |  |  |
| 19.89167 | | | 239.6484 | | | | | | | | | | 1.231125 | | | | 4645.891 | | | 20.55469 | |  |  |  |  |  |
| 19.9 | | | 239.7357 | | | | | | | | | | 1.231993 | | | | 4645.734 | | | 20.42969 | |  |  |  |  |  |
| 19.90833 | | | 239.8223 | | | | | | | | | | 1.234421 | | | | 4645.523 | | | 20.19531 | |  |  |  |  |  |
| 19.91667 | | | 239.9089 | | | | | | | | | | 1.235646 | | | | 4645.359 | | | 20.24219 | |  |  |  |  |  |
| 19.925 | | | 240.0003 | | | | | | | | | | 1.236009 | | | | 4645.188 | | | 20.50781 | |  |  |  |  |  |
| 19.93333 | | | 240.09 | | | | | | | | | | 1.238113 | | | | 4645.031 | | | 20.72656 | |  |  |  |  |  |
| 19.94167 | | | 240.1774 | | | | | | | | | | 1.238988 | | | | 4644.836 | | | 20.90625 | |  |  |  |  |  |
| 19.95 | | | 240.2648 | | | | | | | | | | 1.23886 | | | | 4644.695 | | | 20.92969 | |  |  |  |  |  |
| 19.95833 | | | 240.3546 | | | | | | | | | | 1.240728 | | | | 4644.555 | | | 21.17969 | |  |  |  |  |  |
| 19.96667 | | | 240.4427 | | | | | | | | | | 1.241587 | | | | 4644.398 | | | 21.25 | |  |  |  |  |  |
| 19.975 | | | 240.5341 | | | | | | | | | | 1.242247 | | | | 4644.188 | | | 21.38281 | |  |  |  |  |  |
| 19.98333 | | | 240.6231 | | | | | | | | | | 1.244823 | | | | 4643.984 | | | 21.57031 | |  |  |  |  |  |
| 19.99167 | | | 240.7145 | | | | | | | | | | 1.245986 | | | | 4643.789 | | | 21.77344 | |  |  |  |  |  |
| 20 | | | 240.8075 | | | | | | | | | | 1.246848 | | | | 4643.609 | | | 22.03125 | |  |  |  |  |  |
| 20.00833 | | | 240.8956 | | | | | | | | | | 1.249503 | | | | 4643.398 | | | 21.95313 | |  |  |  |  |  |
| 20.01667 | | | 240.9854 | | | | | | | | | | 1.250923 | | | | 4643.211 | | | 22.0625 | |  |  |  |  |  |
| 20.025 | | | 241.0752 | | | | | | | | | | 1.252305 | | | | 4643.016 | | | 21.97656 | |  |  |  |  |  |
| 20.03333 | | | 241.161 | | | | | | | | | | 1.255581 | | | | 4642.813 | | | 22.07031 | |  |  |  |  |  |
| 20.04167 | | | 241.2523 | | | | | | | | | | 1.257495 | | | | 4642.602 | | | 22.09375 | |  |  |  |  |  |
| 20.05 | | | 241.3413 | | | | | | | | | | 1.258709 | | | | 4642.391 | | | 22.35938 | |  |  |  |  |  |
| 20.05833 | | | 241.4271 | | | | | | | | | | 1.261609 | | | | 4642.227 | | | 22.66406 | |  |  |  |  |  |
| 20.06667 | | | 241.5169 | | | | | | | | | | 1.263408 | | | | 4642.031 | | | 22.98438 | |  |  |  |  |  |
| 20.075 | | | 241.6082 | | | | | | | | | | 1.265083 | | | | 4641.859 | | | 23.19531 | |  |  |  |  |  |
| 20.08333 | | | 241.6972 | | | | | | | | | | 1.268635 | | | | 4641.664 | | | 23.20313 | |  |  |  |  |  |
| 20.09167 | | | 241.7886 | | | | | | | | | | 1.270417 | | | | 4641.477 | | | 23.3125 | |  |  |  |  |  |
| 20.1 | | | 241.88 | | | | | | | | | | 1.271847 | | | | 4641.258 | | | 23.39844 | |  |  |  |  |  |
| 20.10833 | | | 241.969 | | | | | | | | | | 1.274954 | | | | 4641.031 | | | 23.46094 | |  |  |  |  |  |
| 20.11667 | | | 242.0619 | | | | | | | | | | 1.276584 | | | | 4640.797 | | | 23.90625 | |  |  |  |  |  |
| 20.125 | | | 242.1517 | | | | | | | | | | 1.278029 | | | | 4640.57 | | | 23.99219 | |  |  |  |  |  |
| 20.13333 | | | 242.2439 | | | | | | | | | | 1.281617 | | | | 4640.383 | | | 23.94531 | |  |  |  |  |  |
| 20.14167 | | | 242.3369 | | | | | | | | | | 1.283458 | | | | 4640.172 | | | 23.96094 | |  |  |  |  |  |
| 20.15 | | | 242.4291 | | | | | | | | | | 1.285259 | | | | 4639.969 | | | 24.0625 | |  |  |  |  |  |
| 20.15833 | | | 242.5196 | | | | | | | | | | 1.288132 | | | | 4639.758 | | | 24.17188 | |  |  |  |  |  |
| 20.16667 | | | 242.6086 | | | | | | | | | | 1.288962 | | | | 4639.5 | | | 24.33594 | |  |  |  |  |  |
| 20.175 | | | 242.6992 | | | | | | | | | | 1.290589 | | | | 4639.297 | | | 24.34375 | |  |  |  |  |  |
| 20.18333 | | | 242.789 | | | | | | | | | | 1.293679 | | | | 4639.102 | | | 24.53906 | |  |  |  |  |  |
| 20.19167 | | | 242.882 | | | | | | | | | | 1.294785 | | | | 4638.906 | | | 24.71094 | |  |  |  |  |  |
| 20.2 | | | 242.9741 | | | | | | | | | | 1.296895 | | | | 4638.688 | | | 24.67188 | |  |  |  |  |  |
| 20.20833 | | | 243.0647 | | | | | | | | | | 1.300068 | | | | 4638.477 | | | 24.74219 | |  |  |  |  |  |
| 20.21667 | | | 243.1569 | | | | | | | | | | 1.300769 | | | | 4638.25 | | | 24.88281 | |  |  |  |  |  |
| 20.225 | | | 243.2507 | | | | | | | | | | 1.303389 | | | | 4638.055 | | | 24.88281 | |  |  |  |  |  |
| 20.23333 | | | 243.3437 | | | | | | | | | | 1.306488 | | | | 4637.813 | | | 25.03125 | |  |  |  |  |  |
| 20.24167 | | | 243.431 | | | | | | | | | | 1.307467 | | | | 4637.594 | | | 25.17188 | |  |  |  |  |  |
| 20.25 | | | 243.5224 | | | | | | | | | | 1.31098 | | | | 4637.391 | | | 25.32813 | |  |  |  |  |  |
| 20.25833 | | | 243.6138 | | | | | | | | | | 1.31411 | | | | 4637.188 | | | 25.55469 | |  |  |  |  |  |
| 20.26667 | | | 243.706 | | | | | | | | | | 1.31457 | | | | 4636.961 | | | 25.53125 | |  |  |  |  |  |
| 20.275 | | | 243.7982 | | | | | | | | | | 1.318007 | | | | 4636.75 | | | 25.82813 | |  |  |  |  |  |
| 20.28333 | | | 243.8896 | | | | | | | | | | 1.32089 | | | | 4636.531 | | | 25.85938 | |  |  |  |  |  |
| 20.29167 | | | 243.9793 | | | | | | | | | | 1.321223 | | | | 4636.305 | | | 26.30469 | |  |  |  |  |  |
| 20.3 | | | 244.0715 | | | | | | | | | | 1.325032 | | | | 4636.078 | | | 26.51563 | |  |  |  |  |  |
| 20.30833 | | | 244.1613 | | | | | | | | | | 1.327469 | | | | 4635.852 | | | 26.6875 | |  |  |  |  |  |
| 20.31667 | | | 244.2503 | | | | | | | | | | 1.327553 | | | | 4635.633 | | | 26.9375 | |  |  |  |  |  |
| 20.325 | | | 244.3424 | | | | | | | | | | 1.330464 | | | | 4635.383 | | | 27.14063 | |  |  |  |  |  |
| 20.33333 | | | 244.4338 | | | | | | | | | | 1.33274 | | | | 4635.164 | | | 27.1875 | |  |  |  |  |  |
| 20.34167 | | | 244.5244 | | | | | | | | | | 1.332638 | | | | 4634.883 | | | 27.54688 | |  |  |  |  |  |
| 20.35 | | | 244.6142 | | | | | | | | | | 1.335609 | | | | 4634.641 | | | 27.82031 | |  |  |  |  |  |
| 20.35833 | | | 244.704 | | | | | | | | | | 1.337916 | | | | 4634.383 | | | 27.6875 | |  |  |  |  |  |
| 20.36667 | | | 244.7945 | | | | | | | | | | 1.337919 | | | | 4634.141 | | | 27.90625 | |  |  |  |  |  |
| 20.375 | | | 244.8899 | | | | | | | | | | 1.34135 | | | | 4633.883 | | | 27.98438 | |  |  |  |  |  |
| 20.38333 | | | 244.9781 | | | | | | | | | | 1.343412 | | | | 4633.656 | | | 28.23438 | |  |  |  |  |  |
| 20.39167 | | | 245.0671 | | | | | | | | | | 1.343876 | | | | 4633.375 | | | 28.21094 | |  |  |  |  |  |
| 20.4 | | | 245.1561 | | | | | | | | | | 1.347397 | | | | 4633.109 | | | 28.33594 | |  |  |  |  |  |
| 20.40833 | | | 245.2506 | | | | | | | | | | 1.350253 | | | | 4632.906 | | | 28.28906 | |  |  |  |  |  |
| 20.41667 | | | 245.3412 | | | | | | | | | | 1.351274 | | | | 4632.641 | | | 28.42969 | |  |  |  |  |  |
| 20.425 | | | 245.4326 | | | | | | | | | | 1.356052 | | | | 4632.406 | | | 28.34375 | |  |  |  |  |  |
| 20.43333 | | | 245.5232 | | | | | | | | | | 1.358843 | | | | 4632.148 | | | 28.45313 | |  |  |  |  |  |
| 20.44167 | | | 245.6145 | | | | | | | | | | 1.359699 | | | | 4631.914 | | | 28.875 | |  |  |  |  |  |
| 20.45 | | | 245.7059 | | | | | | | | | | 1.364636 | | | | 4631.664 | | | 29.0625 | |  |  |  |  |  |
| 20.45833 | | | 245.7965 | | | | | | | | | | 1.366884 | | | | 4631.43 | | | 29.28125 | |  |  |  |  |  |
| 20.46667 | | | 245.8887 | | | | | | | | | | 1.367325 | | | | 4631.172 | | | 29.38281 | |  |  |  |  |  |
| 20.475 | | | 245.9777 | | | | | | | | | | 1.371578 | | | | 4630.953 | | | 29.57813 | |  |  |  |  |  |
| 20.48333 | | | 246.0715 | | | | | | | | | | 1.373437 | | | | 4630.711 | | | 29.77344 | |  |  |  |  |  |
| 20.49167 | | | 246.1612 | | | | | | | | | | 1.373869 | | | | 4630.406 | | | 30.01563 | |  |  |  |  |  |
| 20.5 | | | 246.2526 | | | | | | | | | | 1.377683 | | | | 4630.148 | | | 30.17188 | |  |  |  |  |  |
| 20.50833 | | | 246.3424 | | | | | | | | | | 1.379238 | | | | 4629.883 | | | 30.76563 | |  |  |  |  |  |
| 20.51667 | | | 246.433 | | | | | | | | | | 1.379975 | | | | 4629.617 | | | 30.80469 | |  |  |  |  |  |
| 20.525 | | | 246.5244 | | | | | | | | | | 1.384156 | | | | 4629.344 | | | 30.83594 | |  |  |  |  |  |
| 20.53333 | | | 246.6125 | | | | | | | | | | 1.385518 | | | | 4629.078 | | | 30.86719 | |  |  |  |  |  |
| 20.54167 | | | 246.6999 | | | | | | | | | | 1.386192 | | | | 4628.805 | | | 30.86719 | |  |  |  |  |  |
| 20.55 | | | 246.7873 | | | | | | | | | | 1.390536 | | | | 4628.523 | | | 31.03906 | |  |  |  |  |  |
| 20.55833 | | | 246.8819 | | | | | | | | | | 1.391848 | | | | 4628.195 | | | 31.125 | |  |  |  |  |  |
| 20.56667 | | | 246.9708 | | | | | | | | | | 1.392816 | | | | 4627.93 | | | 31.17188 | |  |  |  |  |  |
| 20.575 | | | 247.0606 | | | | | | | | | | 1.397862 | | | | 4627.68 | | | 31.32031 | |  |  |  |  |  |
| 20.58333 | | | 247.1512 | | | | | | | | | | 1.400053 | | | | 4627.43 | | | 31.54688 | |  |  |  |  |  |
| 20.59167 | | | 247.2418 | | | | | | | | | | 1.401111 | | | | 4627.164 | | | 31.54688 | |  |  |  |  |  |
| 20.6 | | | 247.334 | | | | | | | | | | 1.40616 | | | | 4626.898 | | | 31.78125 | |  |  |  |  |  |
| 20.60833 | | | 247.4253 | | | | | | | | | | 1.407924 | | | | 4626.625 | | | 32.17188 | |  |  |  |  |  |
| 20.61667 | | | 247.5183 | | | | | | | | | | 1.40956 | | | | 4626.359 | | | 32.41406 | |  |  |  |  |  |
| 20.625 | | | 247.6097 | | | | | | | | | | 1.414305 | | | | 4626.086 | | | 32.61719 | |  |  |  |  |  |
| 20.63333 | | | 247.7019 | | | | | | | | | | 1.416191 | | | | 4625.797 | | | 32.84375 | |  |  |  |  |  |
| 20.64167 | | | 247.7917 | | | | | | | | | | 1.41834 | | | | 4625.547 | | | 33.09375 | |  |  |  |  |  |
| 20.65 | | | 247.8838 | | | | | | | | | | 1.423299 | | | | 4625.242 | | | 33.21875 | |  |  |  |  |  |
| 20.65833 | | | 247.9752 | | | | | | | | | | 1.425214 | | | | 4624.922 | | | 33.34375 | |  |  |  |  |  |
| 20.66667 | | | 248.0682 | | | | | | | | | | 1.427167 | | | | 4624.625 | | | 33.48438 | |  |  |  |  |  |
| 20.675 | | | 248.1644 | | | | | | | | | | 1.43199 | | | | 4624.328 | | | 33.60156 | |  |  |  |  |  |
| 20.68333 | | | 248.2566 | | | | | | | | | | 1.433701 | | | | 4624.031 | | | 33.50781 | |  |  |  |  |  |
| 20.69167 | | | 248.3472 | | | | | | | | | | 1.435875 | | | | 4623.711 | | | 33.54688 | |  |  |  |  |  |
| 20.7 | | | 248.4401 | | | | | | | | | | 1.440368 | | | | 4623.414 | | | 33.66406 | |  |  |  |  |  |
| 20.70833 | | | 248.5339 | | | | | | | | | | 1.442256 | | | | 4623.125 | | | 33.91406 | |  |  |  |  |  |
| 20.71667 | | | 248.6285 | | | | | | | | | | 1.445251 | | | | 4622.836 | | | 33.85156 | |  |  |  |  |  |
| 20.725 | | | 248.7223 | | | | | | | | | | 1.449423 | | | | 4622.547 | | | 34.10156 | |  |  |  |  |  |
| 20.73333 | | | 248.8129 | | | | | | | | | | 1.450811 | | | | 4622.289 | | | 34.22656 | |  |  |  |  |  |
| 20.74167 | | | 248.9059 | | | | | | | | | | 1.453541 | | | | 4622 | | | 34.5625 | |  |  |  |  |  |
| 20.75 | | | 248.9972 | | | | | | | | | | 1.456961 | | | | 4621.703 | | | 34.89063 | |  |  |  |  |  |
| 20.75833 | | | 249.087 | | | | | | | | | | 1.458401 | | | | 4621.398 | | | 35.02344 | |  |  |  |  |  |
| 20.76667 | | | 249.18 | | | | | | | | | | 1.461836 | | | | 4621.117 | | | 35.34375 | |  |  |  |  |  |
| 20.775 | | | 249.2754 | | | | | | | | | | 1.465522 | | | | 4620.805 | | | 35.50781 | |  |  |  |  |  |
| 20.78333 | | | 249.3668 | | | | | | | | | | 1.467426 | | | | 4620.508 | | | 35.90625 | |  |  |  |  |  |
| 20.79167 | | | 249.4573 | | | | | | | | | | 1.471155 | | | | 4620.172 | | | 36.03906 | |  |  |  |  |  |
| 20.8 | | | 249.5495 | | | | | | | | | | 1.474711 | | | | 4619.859 | | | 36.35156 | |  |  |  |  |  |
| 20.80833 | | | 249.6385 | | | | | | | | | | 1.476818 | | | | 4619.539 | | | 36.39844 | |  |  |  |  |  |
| 20.81667 | | | 249.7331 | | | | | | | | | | 1.480607 | | | | 4619.211 | | | 36.73438 | |  |  |  |  |  |
| 20.825 | | | 249.8261 | | | | | | | | | | 1.484092 | | | | 4618.891 | | | 36.79688 | |  |  |  |  |  |
| 20.83333 | | | 249.9174 | | | | | | | | | | 1.485846 | | | | 4618.547 | | | 37.02344 | |  |  |  |  |  |
| 20.84167 | | | 250.0072 | | | | | | | | | | 1.48953 | | | | 4618.227 | | | 37.34375 | |  |  |  |  |  |
| 20.85 | | | 250.0986 | | | | | | | | | | 1.492767 | | | | 4617.891 | | | 37.46875 | |  |  |  |  |  |
| 20.85833 | | | 250.1892 | | | | | | | | | | 1.494112 | | | | 4617.578 | | | 37.5 | |  |  |  |  |  |
| 20.86667 | | | 250.2782 | | | | | | | | | | 1.497239 | | | | 4617.234 | | | 37.79688 | |  |  |  |  |  |
| 20.875 | | | 250.3695 | | | | | | | | | | 1.499865 | | | | 4616.914 | | | 38.03125 | |  |  |  |  |  |
| 20.88333 | | | 250.4577 | | | | | | | | | | 1.500815 | | | | 4616.586 | | | 38.13281 | |  |  |  |  |  |
| 20.89167 | | | 250.5507 | | | | | | | | | | 1.503986 | | | | 4616.234 | | | 38.67188 | |  |  |  |  |  |
| 20.9 | | | 250.6397 | | | | | | | | | | 1.507188 | | | | 4615.906 | | | 38.89844 | |  |  |  |  |  |
| 20.90833 | | | 250.7303 | | | | | | | | | | 1.509032 | | | | 4615.586 | | | 39.14844 | |  |  |  |  |  |
| 20.91667 | | | 250.8208 | | | | | | | | | | 1.512692 | | | | 4615.234 | | | 39.42969 | |  |  |  |  |  |
| 20.925 | | | 250.913 | | | | | | | | | | 1.51616 | | | | 4614.891 | | | 39.46875 | |  |  |  |  |  |
| 20.93333 | | | 251.0052 | | | | | | | | | | 1.518121 | | | | 4614.563 | | | 40.00781 | |  |  |  |  |  |
| 20.94167 | | | 251.0934 | | | | | | | | | | 1.52209 | | | | 4614.172 | | | 40.20313 | |  |  |  |  |  |
| 20.95 | | | 251.1864 | | | | | | | | | | 1.525458 | | | | 4613.828 | | | 40.24219 | |  |  |  |  |  |
| 20.95833 | | | 251.2785 | | | | | | | | | | 1.527765 | | | | 4613.469 | | | 40.41406 | |  |  |  |  |  |
| 20.96667 | | | 251.3723 | | | | | | | | | | 1.532419 | | | | 4613.109 | | | 40.85938 | |  |  |  |  |  |
| 20.975 | | | 251.4669 | | | | | | | | | | 1.535318 | | | | 4612.773 | | | 40.71094 | |  |  |  |  |  |
| 20.98333 | | | 251.5607 | | | | | | | | | | 1.537312 | | | | 4612.375 | | | 40.92188 | |  |  |  |  |  |
| 20.99167 | | | 251.6513 | | | | | | | | | | 1.541433 | | | | 4612.023 | | | 40.96094 | |  |  |  |  |  |
| 21 | | | 251.7427 | | | | | | | | | | 1.54353 | | | | 4611.68 | | | 41.14844 | |  |  |  |  |  |
| 21.00833 | | | 251.8356 | | | | | | | | | | 1.545425 | | | | 4611.32 | | | 41.46875 | |  |  |  |  |  |
| 21.01667 | | | 251.9238 | | | | | | | | | | 1.549529 | | | | 4610.93 | | | 41.625 | |  |  |  |  |  |
| 21.025 | | | 252.0176 | | | | | | | | | | 1.55174 | | | | 4610.609 | | | 41.89844 | |  |  |  |  |  |
| 21.03333 | | | 252.109 | | | | | | | | | | 1.554696 | | | | 4610.25 | | | 41.91406 | |  |  |  |  |  |
| 21.04167 | | | 252.2004 | | | | | | | | | | 1.559577 | | | | 4609.898 | | | 42.19531 | |  |  |  |  |  |
| 21.05 | | | 252.2917 | | | | | | | | | | 1.561613 | | | | 4609.523 | | | 42.24219 | |  |  |  |  |  |
| 21.05833 | | | 252.3799 | | | | | | | | | | 1.564177 | | | | 4609.156 | | | 42.40625 | |  |  |  |  |  |
| 21.06667 | | | 252.4697 | | | | | | | | | | 1.568764 | | | | 4608.789 | | | 42.63281 | |  |  |  |  |  |
| 21.075 | | | 252.5635 | | | | | | | | | | 1.571295 | | | | 4608.414 | | | 43.05469 | |  |  |  |  |  |
| 21.08333 | | | 252.6565 | | | | | | | | | | 1.574517 | | | | 4608.063 | | | 43.27344 | |  |  |  |  |  |
| 21.09167 | | | 252.7478 | | | | | | | | | | 1.580008 | | | | 4607.688 | | | 43.51563 | |  |  |  |  |  |
| 21.1 | | | 252.84 | | | | | | | | | | 1.582518 | | | | 4607.328 | | | 43.69531 | |  |  |  |  |  |
| 21.10833 | | | 252.9314 | | | | | | | | | | 1.585823 | | | | 4606.961 | | | 44.25 | |  |  |  |  |  |
| 21.11667 | | | 253.0244 | | | | | | | | | | 1.590545 | | | | 4606.578 | | | 44.66406 | |  |  |  |  |  |
| 21.125 | | | 253.115 | | | | | | | | | | 1.591881 | | | | 4606.172 | | | 44.95313 | |  |  |  |  |  |
| 21.13333 | | | 253.2047 | | | | | | | | | | 1.595118 | | | | 4605.781 | | | 45.23438 | |  |  |  |  |  |
| 21.14167 | | | 253.2961 | | | | | | | | | | 1.600004 | | | | 4605.391 | | | 45.60156 | |  |  |  |  |  |
| 21.15 | | | 253.3923 | | | | | | | | | | 1.601516 | | | | 4605.008 | | | 45.82031 | |  |  |  |  |  |
| 21.15833 | | | 253.4853 | | | | | | | | | | 1.605101 | | | | 4604.563 | | | 45.96094 | |  |  |  |  |  |
| 21.16667 | | | 253.5783 | | | | | | | | | | 1.610049 | | | | 4604.141 | | | 46.14063 | |  |  |  |  |  |
| 21.175 | | | 253.6697 | | | | | | | | | | 1.611226 | | | | 4603.727 | | | 46.59375 | |  |  |  |  |  |
| 21.18333 | | | 253.7643 | | | | | | | | | | 1.615621 | | | | 4603.32 | | | 47.00781 | |  |  |  |  |  |
| 21.19167 | | | 253.8572 | | | | | | | | | | 1.621108 | | | | 4602.891 | | | 46.97656 | |  |  |  |  |  |
| 21.2 | | | 253.951 | | | | | | | | | | 1.622527 | | | | 4602.484 | | | 47.21875 | |  |  |  |  |  |
| 21.20833 | | | 254.044 | | | | | | | | | | 1.627319 | | | | 4602.086 | | | 47.50781 | |  |  |  |  |  |
| 21.21667 | | | 254.1394 | | | | | | | | | | 1.632495 | | | | 4601.68 | | | 47.72656 | |  |  |  |  |  |
| 21.225 | | | 254.2372 | | | | | | | | | | 1.63394 | | | | 4601.234 | | | 47.99219 | |  |  |  |  |  |
| 21.23333 | | | 254.327 | | | | | | | | | | 1.638124 | | | | 4600.805 | | | 48.3125 | |  |  |  |  |  |
| 21.24167 | | | 254.4159 | | | | | | | | | | 1.642909 | | | | 4600.414 | | | 48.80469 | |  |  |  |  |  |
| 21.25 | | | 254.5065 | | | | | | | | | | 1.643698 | | | | 4599.984 | | | 49.14063 | |  |  |  |  |  |
| 21.25833 | | | 254.6003 | | | | | | | | | | 1.647983 | | | | 4599.563 | | | 49.32813 | |  |  |  |  |  |
| 21.26667 | | | 254.6925 | | | | | | | | | | 1.652162 | | | | 4599.141 | | | 49.3125 | |  |  |  |  |  |
| 21.275 | | | 254.7847 | | | | | | | | | | 1.653107 | | | | 4598.719 | | | 49.67188 | |  |  |  |  |  |
| 21.28333 | | | 254.8752 | | | | | | | | | | 1.657498 | | | | 4598.266 | | | 49.98438 | |  |  |  |  |  |
| 21.29167 | | | 254.9698 | | | | | | | | | | 1.661733 | | | | 4597.797 | | | 50.29688 | |  |  |  |  |  |
| 21.3 | | | 255.0644 | | | | | | | | | | 1.663721 | | | | 4597.352 | | | 50.72656 | |  |  |  |  |  |
| 21.30833 | | | 255.1542 | | | | | | | | | | 1.669235 | | | | 4596.922 | | | 51.05469 | |  |  |  |  |  |
| 21.31667 | | | 255.2488 | | | | | | | | | | 1.674147 | | | | 4596.516 | | | 51.20313 | |  |  |  |  |  |
| 21.325 | | | 255.341 | | | | | | | | | | 1.676767 | | | | 4596.055 | | | 51.36719 | |  |  |  |  |  |
| 21.33333 | | | 255.438 | | | | | | | | | | 1.682688 | | | | 4595.602 | | | 51.73438 | |  |  |  |  |  |
| 21.34167 | | | 255.5317 | | | | | | | | | | 1.686906 | | | | 4595.141 | | | 52.16406 | |  |  |  |  |  |
| 21.35 | | | 255.6263 | | | | | | | | | | 1.690027 | | | | 4594.672 | | | 52.52344 | |  |  |  |  |  |
| 21.35833 | | | 255.7201 | | | | | | | | | | 1.695552 | | | | 4594.203 | | | 52.71094 | |  |  |  |  |  |
| 21.36667 | | | 255.8131 | | | | | | | | | | 1.699372 | | | | 4593.758 | | | 52.94531 | |  |  |  |  |  |
| 21.375 | | | 255.9069 | | | | | | | | | | 1.702343 | | | | 4593.32 | | | 53.27344 | |  |  |  |  |  |
| 21.38333 | | | 255.9967 | | | | | | | | | | 1.707222 | | | | 4592.844 | | | 53.625 | |  |  |  |  |  |
| 21.39167 | | | 256.0896 | | | | | | | | | | 1.71012 | | | | 4592.359 | | | 53.91406 | |  |  |  |  |  |
| 21.4 | | | 256.1802 | | | | | | | | | | 1.712827 | | | | 4591.875 | | | 54.13281 | |  |  |  |  |  |
| 21.40833 | | | 256.2748 | | | | | | | | | | 1.718051 | | | | 4591.422 | | | 54.45313 | |  |  |  |  |  |
| 21.41667 | | | 256.3678 | | | | | | | | | | 1.721215 | | | | 4590.953 | | | 54.875 | |  |  |  |  |  |
| 21.425 | | | 256.4576 | | | | | | | | | | 1.724496 | | | | 4590.469 | | | 55.20313 | |  |  |  |  |  |
| 21.43333 | | | 256.5446 | | | | | | | | | | 1.729426 | | | | 4589.984 | | | 55.34375 | |  |  |  |  |  |
| 21.44167 | | | 256.6329 | | | | | | | | | | 1.732486 | | | | 4589.5 | | | 55.8125 | |  |  |  |  |  |
| 21.45 | | | 256.7252 | | | | | | | | | | 1.73598 | | | | 4589.031 | | | 56.03906 | |  |  |  |  |  |
| 21.45833 | | | 256.8151 | | | | | | | | | | 1.74132 | | | | 4588.539 | | | 56.38281 | |  |  |  |  |  |
| 21.46667 | | | 256.9089 | | | | | | | | | | 1.744777 | | | | 4588.031 | | | 56.72656 | |  |  |  |  |  |
| 21.475 | | | 256.9995 | | | | | | | | | | 1.749 | | | | 4587.539 | | | 57.07031 | |  |  |  |  |  |
| 21.48333 | | | 257.091 | | | | | | | | | | 1.754886 | | | | 4587.063 | | | 57.4375 | |  |  |  |  |  |
| 21.49167 | | | 257.1825 | | | | | | | | | | 1.758268 | | | | 4586.539 | | | 57.66406 | |  |  |  |  |  |
| 21.5 | | | 257.2716 | | | | | | | | | | 1.762115 | | | | 4586.047 | | | 57.75 | |  |  |  |  |  |
| 21.50833 | | | 257.363 | | | | | | | | | | 1.767705 | | | | 4585.531 | | | 57.96875 | |  |  |  |  |  |
| 21.51667 | | | 257.4553 | | | | | | | | | | 1.771126 | | | | 4585.016 | | | 58.42188 | |  |  |  |  |  |
| 21.525 | | | 257.5475 | | | | | | | | | | 1.775501 | | | | 4584.508 | | | 58.78906 | |  |  |  |  |  |
| 21.53333 | | | 257.6359 | | | | | | | | | | 1.780739 | | | | 4583.984 | | | 59.14844 | |  |  |  |  |  |
| 21.54167 | | | 257.7257 | | | | | | | | | | 1.783907 | | | | 4583.469 | | | 59.59375 | |  |  |  |  |  |
| 21.55 | | | 257.8156 | | | | | | | | | | 1.788302 | | | | 4582.992 | | | 60 | |  |  |  |  |  |
| 21.55833 | | | 257.9071 | | | | | | | | | | 1.793851 | | | | 4582.484 | | | 60.32813 | |  |  |  |  |  |
| 21.56667 | | | 257.9985 | | | | | | | | | | 1.797021 | | | | 4581.938 | | | 60.77344 | |  |  |  |  |  |
| 21.575 | | | 258.0892 | | | | | | | | | | 1.801873 | | | | 4581.398 | | | 61.21875 | |  |  |  |  |  |
| 21.58333 | | | 258.1783 | | | | | | | | | | 1.80703 | | | | 4580.867 | | | 61.70313 | |  |  |  |  |  |
| 21.59167 | | | 258.2706 | | | | | | | | | | 1.81008 | | | | 4580.32 | | | 62.10156 | |  |  |  |  |  |
| 21.6 | | | 258.3628 | | | | | | | | | | 1.815154 | | | | 4579.766 | | | 62.48438 | |  |  |  |  |  |
| 21.60833 | | | 258.4559 | | | | | | | | | | 1.820029 | | | | 4579.227 | | | 62.75781 | |  |  |  |  |  |
| 21.61667 | | | 258.5505 | | | | | | | | | | 1.822911 | | | | 4578.672 | | | 63.24219 | |  |  |  |  |  |
| 21.625 | | | 258.6451 | | | | | | | | | | 1.829013 | | | | 4578.109 | | | 63.29688 | |  |  |  |  |  |
| 21.63333 | | | 258.7373 | | | | | | | | | | 1.833437 | | | | 4577.531 | | | 63.57813 | |  |  |  |  |  |
| 21.64167 | | | 258.8304 | | | | | | | | | | 1.835523 | | | | 4576.961 | | | 64.13281 | |  |  |  |  |  |
| 21.65 | | | 258.925 | | | | | | | | | | 1.841966 | | | | 4576.398 | | | 64.59375 | |  |  |  |  |  |
| 21.65833 | | | 259.0157 | | | | | | | | | | 1.845959 | | | | 4575.852 | | | 64.75 | |  |  |  |  |  |
| 21.66667 | | | 259.1111 | | | | | | | | | | 1.848583 | | | | 4575.266 | | | 65.04688 | |  |  |  |  |  |
| 21.675 | | | 259.2041 | | | | | | | | | | 1.855099 | | | | 4574.742 | | | 65.46875 | |  |  |  |  |  |
| 21.68333 | | | 259.2964 | | | | | | | | | | 1.859381 | | | | 4574.172 | | | 65.97656 | |  |  |  |  |  |
| 21.69167 | | | 259.3894 | | | | | | | | | | 1.862502 | | | | 4573.57 | | | 66.34375 | |  |  |  |  |  |
| 21.7 | | | 259.4793 | | | | | | | | | | 1.869333 | | | | 4572.977 | | | 66.59375 | |  |  |  |  |  |
| 21.70833 | | | 259.5715 | | | | | | | | | | 1.87377 | | | | 4572.422 | | | 67.14844 | |  |  |  |  |  |
| 21.71667 | | | 259.663 | | | | | | | | | | 1.877126 | | | | 4571.852 | | | 67.45313 | |  |  |  |  |  |
| 21.725 | | | 259.7513 | | | | | | | | | | 1.884367 | | | | 4571.258 | | | 67.57813 | |  |  |  |  |  |
| 21.73333 | | | 259.8412 | | | | | | | | | | 1.888497 | | | | 4570.641 | | | 67.86719 | |  |  |  |  |  |
| 21.74167 | | | 259.9366 | | | | | | | | | | 1.891543 | | | | 4570.055 | | | 68.52344 | |  |  |  |  |  |
| 21.75 | | | 260.0281 | | | | | | | | | | 1.898285 | | | | 4569.453 | | | 68.83594 | |  |  |  |  |  |
| 21.75833 | | | 260.1195 | | | | | | | | | | 1.902058 | | | | 4568.844 | | | 69.25781 | |  |  |  |  |  |
| 21.76667 | | | 260.211 | | | | | | | | | | 1.905201 | | | | 4568.242 | | | 69.53125 | |  |  |  |  |  |
| 21.775 | | | 260.3025 | | | | | | | | | | 1.911393 | | | | 4567.68 | | | 70.0625 | |  |  |  |  |  |
| 21.78333 | | | 260.3963 | | | | | | | | | | 1.914581 | | | | 4567.078 | | | 70.26563 | |  |  |  |  |  |
| 21.79167 | | | 260.4862 | | | | | | | | | | 1.917958 | | | | 4566.43 | | | 70.6875 | |  |  |  |  |  |
| 21.8 | | | 260.576 | | | | | | | | | | 1.924131 | | | | 4565.813 | | | 71.13281 | |  |  |  |  |  |
| 21.80833 | | | 260.6675 | | | | | | | | | | 1.927426 | | | | 4565.195 | | | 71.71094 | |  |  |  |  |  |
| 21.81667 | | | 260.7606 | | | | | | | | | | 1.931714 | | | | 4564.578 | | | 71.96094 | |  |  |  |  |  |
| 21.825 | | | 260.8544 | | | | | | | | | | 1.937813 | | | | 4563.938 | | | 72.30469 | |  |  |  |  |  |
| 21.83333 | | | 260.9443 | | | | | | | | | | 1.941501 | | | | 4563.328 | | | 72.9375 | |  |  |  |  |  |
| 21.84167 | | | 261.0373 | | | | | | | | | | 1.946222 | | | | 4562.688 | | | 73.14844 | |  |  |  |  |  |
| 21.85 | | | 261.1319 | | | | | | | | | | 1.952564 | | | | 4562.039 | | | 73.39844 | |  |  |  |  |  |
| 21.85833 | | | 261.2234 | | | | | | | | | | 1.95678 | | | | 4561.375 | | | 74.01563 | |  |  |  |  |  |
| 21.86667 | | | 261.3164 | | | | | | | | | | 1.962283 | | | | 4560.75 | | | 74.63281 | |  |  |  |  |  |
| 21.875 | | | 261.4102 | | | | | | | | | | 1.96808 | | | | 4560.109 | | | 75.10156 | |  |  |  |  |  |
| 21.88333 | | | 261.5041 | | | | | | | | | | 1.971753 | | | | 4559.422 | | | 75.375 | |  |  |  |  |  |
| 21.89167 | | | 261.5995 | | | | | | | | | | 1.977867 | | | | 4558.797 | | | 75.91406 | |  |  |  |  |  |
| 21.9 | | | 261.6917 | | | | | | | | | | 1.982971 | | | | 4558.156 | | | 76.57031 | |  |  |  |  |  |
| 21.90833 | | | 261.7816 | | | | | | | | | | 1.986812 | | | | 4557.469 | | | 77.1875 | |  |  |  |  |  |
| 21.91667 | | | 261.8755 | | | | | | | | | | 1.993118 | | | | 4556.766 | | | 77.35938 | |  |  |  |  |  |
| 21.925 | | | 261.9685 | | | | | | | | | | 1.997927 | | | | 4556.094 | | | 78.14063 | |  |  |  |  |  |
| 21.93333 | | | 262.0584 | | | | | | | | | | 2.00206 | | | | 4555.43 | | | 78.5625 | |  |  |  |  |  |
| 21.94167 | | | 262.149 | | | | | | | | | | 2.00883 | | | | 4554.727 | | | 78.67969 | |  |  |  |  |  |
| 21.95 | | | 262.2405 | | | | | | | | | | 2.013796 | | | | 4554.008 | | | 78.9375 | |  |  |  |  |  |
| 21.95833 | | | 262.332 | | | | | | | | | | 2.017779 | | | | 4553.289 | | | 79.5625 | |  |  |  |  |  |
| 21.96667 | | | 262.425 | | | | | | | | | | 2.024275 | | | | 4552.625 | | | 80.14063 | |  |  |  |  |  |
| 21.975 | | | 262.5173 | | | | | | | | | | 2.028588 | | | | 4551.883 | | | 80.64063 | |  |  |  |  |  |
| 21.98333 | | | 262.6071 | | | | | | | | | | 2.032816 | | | | 4551.18 | | | 80.92188 | |  |  |  |  |  |
| 21.99167 | | | 262.6971 | | | | | | | | | | 2.039626 | | | | 4550.508 | | | 81.4375 | |  |  |  |  |  |
| 22 | | | 262.7901 | | | | | | | | | | 2.044598 | | | | 4549.828 | | | 82.04688 | |  |  |  |  |  |
| 22.00833 | | | 262.8808 | | | | | | | | | | 2.048974 | | | | 4549.094 | | | 82.64063 | |  |  |  |  |  |
| 22.01667 | | | 262.9738 | | | | | | | | | | 2.055701 | | | | 4548.352 | | | 83.26563 | |  |  |  |  |  |
| 22.025 | | | 263.0692 | | | | | | | | | | 2.060115 | | | | 4547.625 | | | 83.6875 | |  |  |  |  |  |
| 22.03333 | | | 263.1607 | | | | | | | | | | 2.064265 | | | | 4546.922 | | | 83.9375 | |  |  |  |  |  |
| 22.04167 | | | 263.2529 | | | | | | | | | | 2.07148 | | | | 4546.18 | | | 84.42188 | |  |  |  |  |  |
| 22.05 | | | 263.3444 | | | | | | | | | | 2.07609 | | | | 4545.422 | | | 84.75 | |  |  |  |  |  |
| 22.05833 | | | 263.4366 | | | | | | | | | | 2.080836 | | | | 4544.656 | | | 84.80469 | |  |  |  |  |  |
| 22.06667 | | | 263.5289 | | | | | | | | | | 2.08801 | | | | 4543.898 | | | 85.42969 | |  |  |  |  |  |
| 22.075 | | | 263.6259 | | | | | | | | | | 2.092446 | | | | 4543.148 | | | 85.71875 | |  |  |  |  |  |
| 22.08333 | | | 263.7181 | | | | | | | | | | 2.097143 | | | | 4542.422 | | | 86.08594 | |  |  |  |  |  |
| 22.09167 | | | 263.8104 | | | | | | | | | | 2.104793 | | | | 4541.664 | | | 86.34375 | |  |  |  |  |  |
| 22.1 | | | 263.901 | | | | | | | | | | 2.10822 | | | | 4540.922 | | | 86.85938 | |  |  |  |  |  |
| 22.10833 | | | 263.9933 | | | | | | | | | | 2.113318 | | | | 4540.219 | | | 87.49219 | |  |  |  |  |  |
| 22.11667 | | | 264.0871 | | | | | | | | | | 2.121447 | | | | 4539.438 | | | 88.19531 | |  |  |  |  |  |
| 22.125 | | | 264.1794 | | | | | | | | | | 2.125573 | | | | 4538.703 | | | 88.61719 | |  |  |  |  |  |
| 22.13333 | | | 264.2701 | | | | | | | | | | 2.13149 | | | | 4537.938 | | | 89.1875 | |  |  |  |  |  |
| 22.14167 | | | 264.3631 | | | | | | | | | | 2.138949 | | | | 4537.203 | | | 89.875 | |  |  |  |  |  |
| 22.15 | | | 264.4585 | | | | | | | | | | 2.143086 | | | | 4536.406 | | | 90.54688 | |  |  |  |  |  |
| 22.15833 | | | 264.55 | | | | | | | | | | 2.149061 | | | | 4535.602 | | | 91.1875 | |  |  |  |  |  |
| 22.16667 | | | 264.643 | | | | | | | | | | 2.155781 | | | | 4534.781 | | | 91.73438 | |  |  |  |  |  |
| 22.175 | | | 264.7376 | | | | | | | | | | 2.16023 | | | | 4533.992 | | | 92.47656 | |  |  |  |  |  |
| 22.18333 | | | 264.8369 | | | | | | | | | | 2.167007 | | | | 4533.188 | | | 92.86719 | |  |  |  |  |  |
| 22.19167 | | | 264.9284 | | | | | | | | | | 2.172829 | | | | 4532.352 | | | 93.57813 | |  |  |  |  |  |
| 22.2 | | | 265.0207 | | | | | | | | | | 2.177137 | | | | 4531.516 | | | 94.20313 | |  |  |  |  |  |
| 22.20833 | | | 265.1114 | | | | | | | | | | 2.183571 | | | | 4530.68 | | | 94.85938 | |  |  |  |  |  |
| 22.21667 | | | 265.2078 | | | | | | | | | | 2.189504 | | | | 4529.852 | | | 95.51563 | |  |  |  |  |  |
| 22.225 | | | 265.3027 | | | | | | | | | | 2.194818 | | | | 4528.992 | | | 96.34375 | |  |  |  |  |  |
| 22.23333 | | | 265.3959 | | | | | | | | | | 2.202124 | | | | 4528.172 | | | 96.66406 | |  |  |  |  |  |
| 22.24167 | | | 265.4861 | | | | | | | | | | 2.20738 | | | | 4527.305 | | | 96.89844 | |  |  |  |  |  |
| 22.25 | | | 265.5801 | | | | | | | | | | 2.212138 | | | | 4526.453 | | | 97.45313 | |  |  |  |  |  |
| 22.25833 | | | 265.6711 | | | | | | | | | | 2.219143 | | | | 4525.578 | | | 97.78906 | |  |  |  |  |  |
| 22.26667 | | | 265.7612 | | | | | | | | | | 2.224118 | | | | 4524.703 | | | 98.35938 | |  |  |  |  |  |
| 22.275 | | | 265.856 | | | | | | | | | | 2.229463 | | | | 4523.797 | | | 98.91406 | |  |  |  |  |  |
| 22.28333 | | | 265.9478 | | | | | | | | | | 2.237138 | | | | 4522.961 | | | 99.32813 | |  |  |  |  |  |
| 22.29167 | | | 266.0426 | | | | | | | | | | 2.241968 | | | | 4522.133 | | | 100.0703 | |  |  |  |  |  |
| 22.3 | | | 266.1351 | | | | | | | | | | 2.247838 | | | | 4521.258 | | | 100.4922 | |  |  |  |  |  |
| 22.30833 | | | 266.226 | | | | | | | | | | 2.254921 | | | | 4520.398 | | | 100.7188 | |  |  |  |  |  |
| 22.31667 | | | 266.3185 | | | | | | | | | | 2.259227 | | | | 4519.516 | | | 101.5859 | |  |  |  |  |  |
| 22.325 | | | 266.4141 | | | | | | | | | | 2.265456 | | | | 4518.625 | | | 102.5234 | |  |  |  |  |  |
| 22.33333 | | | 266.505 | | | | | | | | | | 2.273651 | | | | 4517.75 | | | 103.3359 | |  |  |  |  |  |
| 22.34167 | | | 266.5991 | | | | | | | | | | 2.277972 | | | | 4516.836 | | | 104.1719 | |  |  |  |  |  |
| 22.35 | | | 266.6877 | | | | | | | | | | 2.284703 | | | | 4515.953 | | | 104.7891 | |  |  |  |  |  |
| 22.35833 | | | 266.777 | | | | | | | | | | 2.292698 | | | | 4515.094 | | | 105.4531 | |  |  |  |  |  |
| 22.36667 | | | 266.8695 | | | | | | | | | | 2.296914 | | | | 4514.141 | | | 106.2891 | |  |  |  |  |  |
| 22.375 | | | 266.9636 | | | | | | | | | | 2.303877 | | | | 4513.164 | | | 106.8828 | |  |  |  |  |  |
| 22.38333 | | | 267.0561 | | | | | | | | | | 2.311778 | | | | 4512.203 | | | 107.7266 | |  |  |  |  |  |
| 22.39167 | | | 267.1493 | | | | | | | | | | 2.315754 | | | | 4511.234 | | | 108.5625 | |  |  |  |  |  |
| 22.4 | | | 267.2395 | | | | | | | | | | 2.322681 | | | | 4510.289 | | | 109.1641 | |  |  |  |  |  |
| 22.40833 | | | 267.3304 | | | | | | | | | | 2.330559 | | | | 4509.328 | | | 109.7656 | |  |  |  |  |  |
| 22.41667 | | | 267.4221 | | | | | | | | | | 2.334701 | | | | 4508.336 | | | 110.4141 | |  |  |  |  |  |
| 22.425 | | | 267.513 | | | | | | | | | | 2.342235 | | | | 4507.383 | | | 111.0156 | |  |  |  |  |  |
| 22.43333 | | | 267.6063 | | | | | | | | | | 2.349422 | | | | 4506.383 | | | 111.7188 | |  |  |  |  |  |
| 22.44167 | | | 267.7011 | | | | | | | | | | 2.354286 | | | | 4505.383 | | | 112.3438 | |  |  |  |  |  |
| 22.45 | | | 267.7936 | | | | | | | | | | 2.361569 | | | | 4504.406 | | | 112.8594 | |  |  |  |  |  |
| 22.45833 | | | 267.8838 | | | | | | | | | | 2.367855 | | | | 4503.43 | | | 113.3594 | |  |  |  |  |  |
| 22.46667 | | | 267.9731 | | | | | | | | | | 2.372689 | | | | 4502.438 | | | 114.0234 | |  |  |  |  |  |
| 22.475 | | | 268.0617 | | | | | | | | | | 2.380141 | | | | 4501.445 | | | 114.6875 | |  |  |  |  |  |
| 22.48333 | | | 268.1526 | | | | | | | | | | 2.386992 | | | | 4500.438 | | | 115.4219 | |  |  |  |  |  |
| 22.49167 | | | 268.2466 | | | | | | | | | | 2.391757 | | | | 4499.43 | | | 116.1641 | |  |  |  |  |  |
| 22.5 | | | 268.3391 | | | | | | | | | | 2.399414 | | | | 4498.43 | | | 116.6875 | |  |  |  |  |  |
| 22.50833 | | | 268.4324 | | | | | | | | | | 2.405837 | | | | 4497.43 | | | 117.3203 | |  |  |  |  |  |
| 22.51667 | | | 268.5265 | | | | | | | | | | 2.410867 | | | | 4496.406 | | | 117.9688 | |  |  |  |  |  |
| 22.525 | | | 268.619 | | | | | | | | | | 2.418317 | | | | 4495.375 | | | 118.7422 | |  |  |  |  |  |
| 22.53333 | | | 268.7138 | | | | | | | | | | 2.424308 | | | | 4494.328 | | | 119.4219 | |  |  |  |  |  |
| 22.54167 | | | 268.8071 | | | | | | | | | | 2.429856 | | | | 4493.273 | | | 120.3203 | |  |  |  |  |  |
| 22.55 | | | 268.9011 | | | | | | | | | | 2.437452 | | | | 4492.234 | | | 121.0078 | |  |  |  |  |  |
| 22.55833 | | | 268.9975 | | | | | | | | | | 2.443234 | | | | 4491.195 | | | 121.6719 | |  |  |  |  |  |
| 22.56667 | | | 269.0932 | | | | | | | | | | 2.449729 | | | | 4490.133 | | | 122.4297 | |  |  |  |  |  |
| 22.575 | | | 269.1849 | | | | | | | | | | 2.457697 | | | | 4489.055 | | | 123.2734 | |  |  |  |  |  |
| 22.58333 | | | 269.2797 | | | | | | | | | | 2.462931 | | | | 4487.977 | | | 124.0313 | |  |  |  |  |  |
| 22.59167 | | | 269.3737 | | | | | | | | | | 2.469936 | | | | 4486.867 | | | 124.9766 | |  |  |  |  |  |
| 22.6 | | | 269.4662 | | | | | | | | | | 2.477472 | | | | 4485.773 | | | 125.8516 | |  |  |  |  |  |
| 22.60833 | | | 269.5595 | | | | | | | | | | 2.482313 | | | | 4484.688 | | | 126.8594 | |  |  |  |  |  |
| 22.61667 | | | 269.6512 | | | | | | | | | | 2.489758 | | | | 4483.578 | | | 127.5078 | |  |  |  |  |  |
| 22.625 | | | 269.7453 | | | | | | | | | | 2.497232 | | | | 4482.438 | | | 128.3125 | |  |  |  |  |  |
| 22.63333 | | | 269.8409 | | | | | | | | | | 2.501703 | | | | 4481.32 | | | 129.2109 | |  |  |  |  |  |
| 22.64167 | | | 269.9342 | | | | | | | | | | 2.509478 | | | | 4480.172 | | | 130.0234 | |  |  |  |  |  |
| 22.65 | | | 270.0259 | | | | | | | | | | 2.516878 | | | | 4479.023 | | | 130.7266 | |  |  |  |  |  |
| 22.65833 | | | 270.1207 | | | | | | | | | | 2.520862 | | | | 4477.852 | | | 131.3438 | |  |  |  |  |  |
| 22.66667 | | | 270.2132 | | | | | | | | | | 2.529366 | | | | 4476.711 | | | 132 | |  |  |  |  |  |
| 22.675 | | | 270.3065 | | | | | | | | | | 2.536608 | | | | 4475.547 | | | 132.8047 | |  |  |  |  |  |
| 22.68333 | | | 270.4021 | | | | | | | | | | 2.541137 | | | | 4474.367 | | | 133.4297 | |  |  |  |  |  |
| 22.69167 | | | 270.4938 | | | | | | | | | | 2.549881 | | | | 4473.188 | | | 133.8672 | |  |  |  |  |  |
| 22.7 | | | 270.5894 | | | | | | | | | | 2.55719 | | | | 4472.016 | | | 134.4922 | |  |  |  |  |  |
| 22.70833 | | | 270.6843 | | | | | | | | | | 2.562548 | | | | 4470.867 | | | 135.3281 | |  |  |  |  |  |
| 22.71667 | | | 270.7784 | | | | | | | | | | 2.571468 | | | | 4469.695 | | | 136.1484 | |  |  |  |  |  |
| 22.725 | | | 270.8724 | | | | | | | | | | 2.578557 | | | | 4468.492 | | | 136.9453 | |  |  |  |  |  |
| 22.73333 | | | 270.9665 | | | | | | | | | | 2.583878 | | | | 4467.305 | | | 137.7109 | |  |  |  |  |  |
| 22.74167 | | | 271.0613 | | | | | | | | | | 2.592843 | | | | 4466.148 | | | 138.5859 | |  |  |  |  |  |
| 22.75 | | | 271.1561 | | | | | | | | | | 2.599195 | | | | 4464.953 | | | 139.6563 | |  |  |  |  |  |
| 22.75833 | | | 271.251 | | | | | | | | | | 2.605863 | | | | 4463.727 | | | 140.3281 | |  |  |  |  |  |
| 22.76667 | | | 271.3427 | | | | | | | | | | 2.614144 | | | | 4462.492 | | | 141.0391 | |  |  |  |  |  |
| 22.775 | | | 271.4383 | | | | | | | | | | 2.619987 | | | | 4461.258 | | | 141.9297 | |  |  |  |  |  |
| 22.78333 | | | 271.5308 | | | | | | | | | | 2.627169 | | | | 4460.016 | | | 143.1094 | |  |  |  |  |  |
| 22.79167 | | | 271.6233 | | | | | | | | | | 2.635274 | | | | 4458.758 | | | 143.7969 | |  |  |  |  |  |
| 22.8 | | | 271.715 | | | | | | | | | | 2.641067 | | | | 4457.469 | | | 144.6484 | |  |  |  |  |  |
| 22.80833 | | | 271.8067 | | | | | | | | | | 2.648561 | | | | 4456.227 | | | 145.6172 | |  |  |  |  |  |
| 22.81667 | | | 271.9023 | | | | | | | | | | 2.656751 | | | | 4454.969 | | | 146.4922 | |  |  |  |  |  |
| 22.825 | | | 271.9932 | | | | | | | | | | 2.662152 | | | | 4453.68 | | | 147.3438 | |  |  |  |  |  |
| 22.83333 | | | 272.0857 | | | | | | | | | | 2.669746 | | | | 4452.344 | | | 148.0781 | |  |  |  |  |  |
| 22.84167 | | | 272.179 | | | | | | | | | | 2.67723 | | | | 4451.078 | | | 148.9063 | |  |  |  |  |  |
| 22.85 | | | 272.2738 | | | | | | | | | | 2.682626 | | | | 4449.773 | | | 149.9922 | |  |  |  |  |  |
| 22.85833 | | | 272.3679 | | | | | | | | | | 2.6905 | | | | 4448.445 | | | 150.9531 | |  |  |  |  |  |
| 22.86667 | | | 272.4612 | | | | | | | | | | 2.698022 | | | | 4447.125 | | | 151.6016 | |  |  |  |  |  |
| 22.875 | | | 272.5529 | | | | | | | | | | 2.703294 | | | | 4445.797 | | | 152.7109 | |  |  |  |  |  |
| 22.88333 | | | 272.6446 | | | | | | | | | | 2.711566 | | | | 4444.484 | | | 153.3438 | |  |  |  |  |  |
| 22.89167 | | | 272.7371 | | | | | | | | | | 2.719184 | | | | 4443.148 | | | 154.25 | |  |  |  |  |  |
| 22.9 | | | 272.8296 | | | | | | | | | | 2.72465 | | | | 4441.766 | | | 155.2891 | |  |  |  |  |  |
| 22.90833 | | | 272.9221 | | | | | | | | | | 2.733531 | | | | 4440.398 | | | 156.25 | |  |  |  |  |  |
| 22.91667 | | | 273.013 | | | | | | | | | | 2.74054 | | | | 4439.063 | | | 157.3906 | |  |  |  |  |  |
| 22.925 | | | 273.1039 | | | | | | | | | | 2.746299 | | | | 4437.656 | | | 158.3359 | |  |  |  |  |  |
| 22.93333 | | | 273.1972 | | | | | | | | | | 2.75568 | | | | 4436.313 | | | 159.25 | |  |  |  |  |  |
| 22.94167 | | | 273.2889 | | | | | | | | | | 2.762943 | | | | 4434.914 | | | 160.2031 | |  |  |  |  |  |
| 22.95 | | | 273.3813 | | | | | | | | | | 2.768505 | | | | 4433.508 | | | 161.4375 | |  |  |  |  |  |
| 22.95833 | | | 273.4762 | | | | | | | | | | 2.777796 | | | | 4432.086 | | | 162.1094 | |  |  |  |  |  |
| 22.96667 | | | 273.5718 | | | | | | | | | | 2.78459 | | | | 4430.648 | | | 163.3828 | |  |  |  |  |  |
| 22.975 | | | 273.6674 | | | | | | | | | | 2.79007 | | | | 4429.211 | | | 164.1875 | |  |  |  |  |  |
| 22.98333 | | | 273.7588 | | | | | | | | | | 2.799896 | | | | 4427.773 | | | 165.1172 | |  |  |  |  |  |
| 22.99167 | | | 273.8521 | | | | | | | | | | 2.806256 | | | | 4426.328 | | | 165.8438 | |  |  |  |  |  |
| 23 | | | 273.9468 | | | | | | | | | | 2.813073 | | | | 4424.836 | | | 166.6875 | |  |  |  |  |  |
| 23.00833 | | | 274.0432 | | | | | | | | | | 2.822175 | | | | 4423.406 | | | 167.8438 | |  |  |  |  |  |
| 23.01667 | | | 274.1357 | | | | | | | | | | 2.82808 | | | | 4421.891 | | | 168.8516 | |  |  |  |  |  |
| 23.025 | | | 274.2289 | | | | | | | | | | 2.83537 | | | | 4420.438 | | | 169.7656 | |  |  |  |  |  |
| 23.03333 | | | 274.3222 | | | | | | | | | | 2.843626 | | | | 4418.945 | | | 170.6172 | |  |  |  |  |  |
| 23.04167 | | | 274.4154 | | | | | | | | | | 2.84982 | | | | 4417.484 | | | 171.9141 | |  |  |  |  |  |
| 23.05 | | | 274.5056 | | | | | | | | | | 2.857793 | | | | 4415.992 | | | 172.6953 | |  |  |  |  |  |
| 23.05833 | | | 274.5981 | | | | | | | | | | 2.865748 | | | | 4414.469 | | | 173.7188 | |  |  |  |  |  |
| 23.06667 | | | 274.6936 | | | | | | | | | | 2.871546 | | | | 4412.945 | | | 174.6797 | |  |  |  |  |  |
| 23.075 | | | 274.7869 | | | | | | | | | | 2.879912 | | | | 4411.43 | | | 175.9297 | |  |  |  |  |  |
| 23.08333 | | | 274.8786 | | | | | | | | | | 2.887227 | | | | 4409.906 | | | 177.0625 | |  |  |  |  |  |
| 23.09167 | | | 274.968 | | | | | | | | | | 2.893284 | | | | 4408.328 | | | 177.9375 | |  |  |  |  |  |
| 23.1 | | | 275.0582 | | | | | | | | | | 2.902325 | | | | 4406.813 | | | 178.8125 | |  |  |  |  |  |
| 23.10833 | | | 275.1507 | | | | | | | | | | 2.910061 | | | | 4405.234 | | | 179.8594 | |  |  |  |  |  |
| 23.11667 | | | 275.2447 | | | | | | | | | | 2.916414 | | | | 4403.672 | | | 181.1641 | |  |  |  |  |  |
| 23.125 | | | 275.3372 | | | | | | | | | | 2.925629 | | | | 4402.055 | | | 182.1719 | |  |  |  |  |  |
| 23.13333 | | | 275.4304 | | | | | | | | | | 2.932289 | | | | 4400.445 | | | 183.3359 | |  |  |  |  |  |
| 23.14167 | | | 275.5229 | | | | | | | | | | 2.938404 | | | | 4398.875 | | | 184.3672 | |  |  |  |  |  |
| 23.15 | | | 275.6131 | | | | | | | | | | 2.947968 | | | | 4397.281 | | | 185.5859 | |  |  |  |  |  |
| 23.15833 | | | 275.7048 | | | | | | | | | | 2.953767 | | | | 4395.656 | | | 186.5781 | |  |  |  |  |  |
| 23.16667 | | | 275.798 | | | | | | | | | | 2.960294 | | | | 4394 | | | 187.7422 | |  |  |  |  |  |
| 23.175 | | | 275.8897 | | | | | | | | | | 2.970127 | | | | 4392.367 | | | 189.1875 | |  |  |  |  |  |
| 23.18333 | | | 275.9861 | | | | | | | | | | 2.975819 | | | | 4390.695 | | | 190.2578 | |  |  |  |  |  |
| 23.19167 | | | 276.0801 | | | | | | | | | | 2.98288 | | | | 4389.039 | | | 191.6016 | |  |  |  |  |  |
| 23.2 | | | 276.1734 | | | | | | | | | | 2.992536 | | | | 4387.344 | | | 192.375 | |  |  |  |  |  |
| 23.20833 | | | 276.2651 | | | | | | | | | | 2.998653 | | | | 4385.68 | | | 193.4531 | |  |  |  |  |  |
| 23.21667 | | | 276.3568 | | | | | | | | | | 3.006351 | | | | 4383.977 | | | 194.5313 | |  |  |  |  |  |
| 23.225 | | | 276.45 | | | | | | | | | | 3.01593 | | | | 4382.227 | | | 195.8359 | |  |  |  |  |  |
| 23.23333 | | | 276.5433 | | | | | | | | | | 3.022334 | | | | 4380.523 | | | 197.0859 | |  |  |  |  |  |
| 23.24167 | | | 276.635 | | | | | | | | | | 3.030862 | | | | 4378.766 | | | 198.2109 | |  |  |  |  |  |
| 23.25 | | | 276.7267 | | | | | | | | | | 3.040544 | | | | 4377.078 | | | 199.1797 | |  |  |  |  |  |
| 23.25833 | | | 276.8223 | | | | | | | | | | 3.047267 | | | | 4375.344 | | | 200.1406 | |  |  |  |  |  |
| 23.26667 | | | 276.914 | | | | | | | | | | 3.055775 | | | | 4373.602 | | | 201.4141 | |  |  |  |  |  |
| 23.275 | | | 277.0034 | | | | | | | | | | 3.064746 | | | | 4371.813 | | | 202.5469 | |  |  |  |  |  |
| 23.28333 | | | 277.0943 | | | | | | | | | | 3.071073 | | | | 4370.031 | | | 203.8594 | |  |  |  |  |  |
| 23.29167 | | | 277.1876 | | | | | | | | | | 3.079419 | | | | 4368.25 | | | 204.9375 | |  |  |  |  |  |
| 23.3 | | | 277.2839 | | | | | | | | | | 3.088232 | | | | 4366.492 | | | 206.3047 | |  |  |  |  |  |
| 23.30833 | | | 277.3764 | | | | | | | | | | 3.094747 | | | | 4364.703 | | | 207.5078 | |  |  |  |  |  |
| 23.31667 | | | 277.4673 | | | | | | | | | | 3.103736 | | | | 4362.875 | | | 208.5 | |  |  |  |  |  |
| 23.325 | | | 277.5591 | | | | | | | | | | 3.111855 | | | | 4361.055 | | | 209.7031 | |  |  |  |  |  |
| 23.33333 | | | 277.6539 | | | | | | | | | | 3.118561 | | | | 4359.203 | | | 211.1563 | |  |  |  |  |  |
| 23.34167 | | | 277.7433 | | | | | | | | | | 3.127347 | | | | 4357.367 | | | 212.5156 | |  |  |  |  |  |
| 23.35 | | | 277.8334 | | | | | | | | | | 3.134612 | | | | 4355.484 | | | 213.9844 | |  |  |  |  |  |
| 23.35833 | | | 277.9282 | | | | | | | | | | 3.141283 | | | | 4353.617 | | | 215.3359 | |  |  |  |  |  |
| 23.36667 | | | 278.023 | | | | | | | | | | 3.150717 | | | | 4351.766 | | | 216.5156 | |  |  |  |  |  |
| 23.375 | | | 278.117 | | | | | | | | | | 3.156856 | | | | 4349.883 | | | 218.2031 | |  |  |  |  |  |
| 23.38333 | | | 278.2095 | | | | | | | | | | 3.16414 | | | | 4347.953 | | | 219.5547 | |  |  |  |  |  |
| 23.39167 | | | 278.3028 | | | | | | | | | | 3.174014 | | | | 4346.016 | | | 220.7031 | |  |  |  |  |  |
| 23.4 | | | 278.3976 | | | | | | | | | | 3.17952 | | | | 4344.063 | | | 222.2266 | |  |  |  |  |  |
| 23.40833 | | | 278.4947 | | | | | | | | | | 3.18751 | | | | 4342.109 | | | 223.9141 | |  |  |  |  |  |
| 23.41667 | | | 278.588 | | | | | | | | | | 3.196322 | | | | 4340.164 | | | 225.3281 | |  |  |  |  |  |
| 23.425 | | | 278.6827 | | | | | | | | | | 3.202047 | | | | 4338.148 | | | 226.625 | |  |  |  |  |  |
| 23.43333 | | | 278.7775 | | | | | | | | | | 3.210204 | | | | 4336.164 | | | 227.8359 | |  |  |  |  |  |
| 23.44167 | | | 278.8716 | | | | | | | | | | 3.218744 | | | | 4334.195 | | | 228.9141 | |  |  |  |  |  |
| 23.45 | | | 278.9641 | | | | | | | | | | 3.224968 | | | | 4332.164 | | | 230.4063 | |  |  |  |  |  |
| 23.45833 | | | 279.0565 | | | | | | | | | | 3.233855 | | | | 4330.086 | | | 231.7969 | |  |  |  |  |  |
| 23.46667 | | | 279.1475 | | | | | | | | | | 3.241571 | | | | 4328.055 | | | 233.1563 | |  |  |  |  |  |
| 23.475 | | | 279.2392 | | | | | | | | | | 3.247817 | | | | 4326.008 | | | 234.4609 | |  |  |  |  |  |
| 23.48333 | | | 279.3324 | | | | | | | | | | 3.256554 | | | | 4323.984 | | | 235.75 | |  |  |  |  |  |
| 23.49167 | | | 279.4234 | | | | | | | | | | 3.26401 | | | | 4321.953 | | | 237 | |  |  |  |  |  |
| 23.5 | | | 279.5151 | | | | | | | | | | 3.270583 | | | | 4319.859 | | | 238.4219 | |  |  |  |  |  |
| 23.50833 | | | 279.6076 | | | | | | | | | | 3.279463 | | | | 4317.766 | | | 239.6953 | |  |  |  |  |  |
| 23.51667 | | | 279.7024 | | | | | | | | | | 3.286857 | | | | 4315.672 | | | 241.2578 | |  |  |  |  |  |
| 23.525 | | | 279.7949 | | | | | | | | | | 3.294413 | | | | 4313.563 | | | 243.0078 | |  |  |  |  |  |
| 23.53333 | | | 279.885 | | | | | | | | | | 3.304114 | | | | 4311.453 | | | 244.7109 | |  |  |  |  |  |
| 23.54167 | | | 279.9775 | | | | | | | | | | 3.311709 | | | | 4309.336 | | | 245.9922 | |  |  |  |  |  |
| 23.55 | | | 280.0746 | | | | | | | | | | 3.31964 | | | | 4307.18 | | | 247.4531 | |  |  |  |  |  |
| 23.55833 | | | 280.1686 | | | | | | | | | | 3.329401 | | | | 4305.039 | | | 249.2578 | |  |  |  |  |  |
| 23.56667 | | | 280.2627 | | | | | | | | | | 3.335927 | | | | 4302.836 | | | 251.1406 | |  |  |  |  |  |
| 23.575 | | | 280.3575 | | | | | | | | | | 3.344022 | | | | 4300.602 | | | 252.6406 | |  |  |  |  |  |
| 23.58333 | | | 280.4499 | | | | | | | | | | 3.353528 | | | | 4298.367 | | | 254.1484 | |  |  |  |  |  |
| 23.59167 | | | 280.5424 | | | | | | | | | | 3.359273 | | | | 4296.172 | | | 255.9531 | |  |  |  |  |  |
| 23.6 | | | 280.6341 | | | | | | | | | | 3.368414 | | | | 4293.945 | | | 257.625 | |  |  |  |  |  |
| 23.60833 | | | 280.7281 | | | | | | | | | | 3.377742 | | | | 4291.648 | | | 259.0625 | |  |  |  |  |  |
| 23.61667 | | | 280.8245 | | | | | | | | | | 3.382989 | | | | 4289.328 | | | 260.5547 | |  |  |  |  |  |
| 23.625 | | | 280.9185 | | | | | | | | | | 3.391998 | | | | 4287.055 | | | 262.0234 | |  |  |  |  |  |
| 23.63333 | | | 281.0102 | | | | | | | | | | 3.400723 | | | | 4284.758 | | | 263.6406 | |  |  |  |  |  |
| 23.64167 | | | 281.1027 | | | | | | | | | | 3.406552 | | | | 4282.414 | | | 265.1641 | |  |  |  |  |  |
| 23.65 | | | 281.1952 | | | | | | | | | | 3.415243 | | | | 4280.07 | | | 266.5078 | |  |  |  |  |  |
| 23.65833 | | | 281.2846 | | | | | | | | | | 3.424008 | | | | 4277.758 | | | 268.0391 | |  |  |  |  |  |
| 23.66667 | | | 281.3778 | | | | | | | | | | 3.430184 | | | | 4275.406 | | | 269.8828 | |  |  |  |  |  |
| 23.675 | | | 281.4727 | | | | | | | | | | 3.439646 | | | | 4273.063 | | | 271.6641 | |  |  |  |  |  |
| 23.68333 | | | 281.5644 | | | | | | | | | | 3.447679 | | | | 4270.672 | | | 273.1875 | |  |  |  |  |  |
| 23.69167 | | | 281.6545 | | | | | | | | | | 3.453859 | | | | 4268.297 | | | 274.9453 | |  |  |  |  |  |
| 23.7 | | | 281.7455 | | | | | | | | | | 3.462771 | | | | 4265.922 | | | 276.3203 | |  |  |  |  |  |
| 23.70833 | | | 281.838 | | | | | | | | | | 3.47043 | | | | 4263.516 | | | 278.3672 | |  |  |  |  |  |
| 23.71667 | | | 281.9297 | | | | | | | | | | 3.477014 | | | | 4261.047 | | | 280.0625 | |  |  |  |  |  |
| 23.725 | | | 282.0229 | | | | | | | | | | 3.486628 | | | | 4258.586 | | | 281.8438 | |  |  |  |  |  |
| 23.73333 | | | 282.1146 | | | | | | | | | | 3.495055 | | | | 4256.125 | | | 283.3828 | |  |  |  |  |  |
| 23.74167 | | | 282.2106 | | | | | | | | | | 3.503106 | | | | 4253.641 | | | 285.3281 | |  |  |  |  |  |
| 23.75 | | | 282.3069 | | | | | | | | | | 3.513454 | | | | 4251.172 | | | 287 | |  |  |  |  |  |
| 23.75833 | | | 282.3993 | | | | | | | | | | 3.521328 | | | | 4248.609 | | | 288.5078 | |  |  |  |  |  |
| 23.76667 | | | 282.4933 | | | | | | | | | | 3.529429 | | | | 4246.078 | | | 290.0234 | |  |  |  |  |  |
| 23.775 | | | 282.5896 | | | | | | | | | | 3.539067 | | | | 4243.516 | | | 291.7188 | |  |  |  |  |  |
| 23.78333 | | | 282.6851 | | | | | | | | | | 3.545873 | | | | 4240.977 | | | 293.6406 | |  |  |  |  |  |
| 23.79167 | | | 282.7791 | | | | | | | | | | 3.554529 | | | | 4238.375 | | | 295.4688 | |  |  |  |  |  |
| 23.8 | | | 282.8715 | | | | | | | | | | 3.563603 | | | | 4235.789 | | | 296.875 | |  |  |  |  |  |
| 23.80833 | | | 282.9655 | | | | | | | | | | 3.5695 | | | | 4233.211 | | | 298.3125 | |  |  |  |  |  |
| 23.81667 | | | 283.061 | | | | | | | | | | 3.578179 | | | | 4230.625 | | | 300.1641 | |  |  |  |  |  |
| 23.825 | | | 283.155 | | | | | | | | | | 3.586516 | | | | 4228 | | | 301.8047 | |  |  |  |  |  |
| 23.83333 | | | 283.2466 | | | | | | | | | | 3.591726 | | | | 4225.336 | | | 303.7656 | |  |  |  |  |  |
| 23.84167 | | | 283.3421 | | | | | | | | | | 3.601556 | | | | 4222.664 | | | 305.5547 | |  |  |  |  |  |
| 23.85 | | | 283.4392 | | | | | | | | | | 3.610038 | | | | 4220.039 | | | 307.2109 | |  |  |  |  |  |
| 23.85833 | | | 283.5355 | | | | | | | | | | 3.616292 | | | | 4217.391 | | | 309.2891 | |  |  |  |  |  |
| 23.86667 | | | 283.6279 | | | | | | | | | | 3.626124 | | | | 4214.672 | | | 310.9609 | |  |  |  |  |  |
| 23.875 | | | 283.7226 | | | | | | | | | | 3.633993 | | | | 4211.969 | | | 312.5 | |  |  |  |  |  |
| 23.88333 | | | 283.8174 | | | | | | | | | | 3.641112 | | | | 4209.211 | | | 314.1484 | |  |  |  |  |  |
| 23.89167 | | | 283.9113 | | | | | | | | | | 3.65162 | | | | 4206.461 | | | 316.125 | |  |  |  |  |  |
| 23.9 | | | 284.0038 | | | | | | | | | | 3.659958 | | | | 4203.711 | | | 318.1719 | |  |  |  |  |  |
| 23.90833 | | | 284.0985 | | | | | | | | | | 3.667734 | | | | 4200.898 | | | 319.8672 | |  |  |  |  |  |
| 23.91667 | | | 284.1917 | | | | | | | | | | 3.677674 | | | | 4198.109 | | | 321.3906 | |  |  |  |  |  |
| 23.925 | | | 284.2864 | | | | | | | | | | 3.685125 | | | | 4195.336 | | | 323.5234 | |  |  |  |  |  |
| 23.93333 | | | 284.3781 | | | | | | | | | | 3.693295 | | | | 4192.531 | | | 325.7578 | |  |  |  |  |  |
| 23.94167 | | | 284.4705 | | | | | | | | | | 3.703278 | | | | 4189.664 | | | 327.8672 | |  |  |  |  |  |
| 23.95 | | | 284.5668 | | | | | | | | | | 3.711206 | | | | 4186.773 | | | 329.6328 | |  |  |  |  |  |
| 23.95833 | | | 284.6646 | | | | | | | | | | 3.720673 | | | | 4183.914 | | | 331.4688 | |  |  |  |  |  |
| 23.96667 | | | 284.7617 | | | | | | | | | | 3.730611 | | | | 4181.055 | | | 333.9766 | |  |  |  |  |  |
| 23.975 | | | 284.8596 | | | | | | | | | | 3.737328 | | | | 4178.102 | | | 336.3125 | |  |  |  |  |  |
| 23.98333 | | | 284.9551 | | | | | | | | | | 3.747478 | | | | 4175.125 | | | 338.3438 | |  |  |  |  |  |
| 23.99167 | | | 285.049 | | | | | | | | | | 3.75659 | | | | 4172.148 | | | 340.5 | |  |  |  |  |  |
| 24 | | | 285.1461 | | | | | | | | | | 3.763343 | | | | 4169.195 | | | 342.9141 | |  |  |  |  |  |
| 24.00833 | | | 285.2416 | | | | | | | | | | 3.774384 | | | | 4166.219 | | | 345.0859 | |  |  |  |  |  |
| 24.01667 | | | 285.3379 | | | | | | | | | | 3.783182 | | | | 4163.125 | | | 347.125 | |  |  |  |  |  |
| 24.025 | | | 285.4311 | | | | | | | | | | 3.790216 | | | | 4160.055 | | | 349.4141 | |  |  |  |  |  |
| 24.03333 | | | 285.5259 | | | | | | | | | | 3.801743 | | | | 4156.992 | | | 351.8125 | |  |  |  |  |  |
| 24.04167 | | | 285.6175 | | | | | | | | | | 3.810896 | | | | 4153.906 | | | 354.5 | |  |  |  |  |  |
| 24.05 | | | 285.7115 | | | | | | | | | | 3.818501 | | | | 4150.766 | | | 356.8203 | |  |  |  |  |  |
| 24.05833 | | | 285.8031 | | | | | | | | | | 3.830048 | | | | 4147.633 | | | 358.9844 | |  |  |  |  |  |
| 24.06667 | | | 285.8986 | | | | | | | | | | 3.838374 | | | | 4144.5 | | | 361.1953 | |  |  |  |  |  |
| 24.075 | | | 285.9949 | | | | | | | | | | 3.846214 | | | | 4141.32 | | | 363.7344 | |  |  |  |  |  |
| 24.08333 | | | 286.0897 | | | | | | | | | | 3.856587 | | | | 4138.102 | | | 366.1953 | |  |  |  |  |  |
| 24.09167 | | | 286.1836 | | | | | | | | | | 3.864262 | | | | 4134.82 | | | 368.6016 | |  |  |  |  |  |
| 24.1 | | | 286.2753 | | | | | | | | | | 3.87261 | | | | 4131.578 | | | 371.25 | |  |  |  |  |  |
| 24.10833 | | | 286.3708 | | | | | | | | | | 3.882983 | | | | 4128.328 | | | 373.3203 | |  |  |  |  |  |
| 24.11667 | | | 286.4632 | | | | | | | | | | 3.890629 | | | | 4125.063 | | | 376.1016 | |  |  |  |  |  |
| 24.125 | | | 286.5564 | | | | | | | | | | 3.899207 | | | | 4121.727 | | | 378.6406 | |  |  |  |  |  |
| 24.13333 | | | 286.6504 | | | | | | | | | | 3.90928 | | | | 4118.391 | | | 381 | |  |  |  |  |  |
| 24.14167 | | | 286.7474 | | | | | | | | | | 3.916963 | | | | 4115.031 | | | 383.5391 | |  |  |  |  |  |
| 24.15 | | | 286.8414 | | | | | | | | | | 3.926843 | | | | 4111.625 | | | 386.2578 | |  |  |  |  |  |
| 24.15833 | | | 286.9354 | | | | | | | | | | 3.937 | | | | 4108.273 | | | 389.0469 | |  |  |  |  |  |
| 24.16667 | | | 287.0286 | | | | | | | | | | 3.94532 | | | | 4104.813 | | | 391.4609 | |  |  |  |  |  |
| 24.175 | | | 287.1194 | | | | | | | | | | 3.955819 | | | | 4101.359 | | | 393.8203 | |  |  |  |  |  |
| 24.18333 | | | 287.2173 | | | | | | | | | | 3.966216 | | | | 4097.906 | | | 396.1875 | |  |  |  |  |  |
| 24.19167 | | | 287.3112 | | | | | | | | | | 3.974517 | | | | 4094.422 | | | 398.875 | |  |  |  |  |  |
| 24.2 | | | 287.4075 | | | | | | | | | | 3.986045 | | | | 4090.875 | | | 401.375 | |  |  |  |  |  |
| 24.20833 | | | 287.5023 | | | | | | | | | | 3.995205 | | | | 4087.32 | | | 403.6563 | |  |  |  |  |  |
| 24.21667 | | | 287.5978 | | | | | | | | | | 4.002693 | | | | 4083.781 | | | 406.125 | |  |  |  |  |  |
| 24.225 | | | 287.6918 | | | | | | | | | | 4.014712 | | | | 4080.227 | | | 409.1641 | |  |  |  |  |  |
| 24.23333 | | | 287.7881 | | | | | | | | | | 4.023224 | | | | 4076.656 | | | 411.7188 | |  |  |  |  |  |
| 24.24167 | | | 287.8813 | | | | | | | | | | 4.032111 | | | | 4073.016 | | | 414.25 | |  |  |  |  |  |
| 24.25 | | | 287.9768 | | | | | | | | | | 4.044186 | | | | 4069.391 | | | 416.6719 | |  |  |  |  |  |
| 24.25833 | | | 288.0769 | | | | | | | | | | 4.052996 | | | | 4065.773 | | | 419.3281 | |  |  |  |  |  |
| 24.26667 | | | 288.174 | | | | | | | | | | 4.062666 | | | | 4062.102 | | | 422.3203 | |  |  |  |  |  |
| 24.275 | | | 288.2688 | | | | | | | | | | 4.073889 | | | | 4058.328 | | | 424.9531 | |  |  |  |  |  |
| 24.28333 | | | 288.3635 | | | | | | | | | | 4.082549 | | | | 4054.609 | | | 427.6016 | |  |  |  |  |  |
| 24.29167 | | | 288.4614 | | | | | | | | | | 4.093245 | | | | 4050.875 | | | 430.1875 | |  |  |  |  |  |
| 24.3 | | | 288.5545 | | | | | | | | | | 4.104584 | | | | 4047.133 | | | 433.3125 | |  |  |  |  |  |
| 24.30833 | | | 288.6501 | | | | | | | | | | 4.113763 | | | | 4043.328 | | | 435.8203 | |  |  |  |  |  |
| 24.31667 | | | 288.7433 | | | | | | | | | | 4.125444 | | | | 4039.461 | | | 438.6953 | |  |  |  |  |  |
| 24.325 | | | 288.8403 | | | | | | | | | | 4.136373 | | | | 4035.625 | | | 441.5625 | |  |  |  |  |  |
| 24.33333 | | | 288.9366 | | | | | | | | | | 4.1459 | | | | 4031.75 | | | 444.8125 | |  |  |  |  |  |
| 24.34167 | | | 289.0298 | | | | | | | | | | 4.158695 | | | | 4027.875 | | | 447.9688 | |  |  |  |  |  |
| 24.35 | | | 289.1215 | | | | | | | | | | 4.169456 | | | | 4023.891 | | | 450.7578 | |  |  |  |  |  |
| 24.35833 | | | 289.2185 | | | | | | | | | | 4.179159 | | | | 4019.977 | | | 453.7109 | |  |  |  |  |  |
| 24.36667 | | | 289.3164 | | | | | | | | | | 4.192766 | | | | 4015.984 | | | 456.5469 | |  |  |  |  |  |
| 24.375 | | | 289.4103 | | | | | | | | | | 4.202709 | | | | 4011.977 | | | 460.1797 | |  |  |  |  |  |
| 24.38333 | | | 289.5097 | | | | | | | | | | 4.212754 | | | | 4007.883 | | | 463.1563 | |  |  |  |  |  |
| 24.39167 | | | 289.6053 | | | | | | | | | | 4.226592 | | | | 4003.781 | | | 466.0391 | |  |  |  |  |  |
| 24.4 | | | 289.7039 | | | | | | | | | | 4.236371 | | | | 3999.695 | | | 468.8359 | |  |  |  |  |  |
| 24.40833 | | | 289.7994 | | | | | | | | | | 4.247612 | | | | 3995.578 | | | 472.0938 | |  |  |  |  |  |
| 24.41667 | | | 289.8972 | | | | | | | | | | 4.261001 | | | | 3991.445 | | | 475.2734 | |  |  |  |  |  |
| 24.425 | | | 289.9959 | | | | | | | | | | 4.270687 | | | | 3987.172 | | | 478.1953 | |  |  |  |  |  |
| 24.43333 | | | 290.0914 | | | | | | | | | | 4.282125 | | | | 3982.961 | | | 481.3281 | |  |  |  |  |  |
| 24.44167 | | | 290.1893 | | | | | | | | | | 4.294657 | | | | 3978.75 | | | 484.3438 | |  |  |  |  |  |
| 24.45 | | | 290.2848 | | | | | | | | | | 4.30469 | | | | 3974.523 | | | 487.9219 | |  |  |  |  |  |
| 24.45833 | | | 290.3834 | | | | | | | | | | 4.317324 | | | | 3970.203 | | | 490.8047 | |  |  |  |  |  |
| 24.46667 | | | 290.4789 | | | | | | | | | | 4.328896 | | | | 3965.875 | | | 493.8047 | |  |  |  |  |  |
| 24.475 | | | 290.5744 | | | | | | | | | | 4.339297 | | | | 3961.555 | | | 496.8516 | |  |  |  |  |  |
| 24.48333 | | | 290.6723 | | | | | | | | | | 4.352747 | | | | 3957.18 | | | 500.3281 | |  |  |  |  |  |
| 24.49167 | | | 290.769 | | | | | | | | | | 4.363533 | | | | 3952.789 | | | 503.4688 | |  |  |  |  |  |
| 24.5 | | | 290.8635 | | | | | | | | | | 4.37421 | | | | 3948.297 | | | 506.4141 | |  |  |  |  |  |
| 24.50833 | | | 290.9586 | | | | | | | | | | 4.388295 | | | | 3943.883 | | | 509.5469 | |  |  |  |  |  |
| 24.51667 | | | 291.0585 | | | | | | | | | | 4.398723 | | | | 3939.43 | | | 512.7656 | |  |  |  |  |  |
| 24.525 | | | 291.1521 | | | | | | | | | | 4.411155 | | | | 3934.93 | | | 516.3828 | |  |  |  |  |  |
| 24.53333 | | | 291.2466 | | | | | | | | | | 4.426285 | | | | 3930.352 | | | 519.6797 | |  |  |  |  |  |
| 24.54167 | | | 291.3402 | | | | | | | | | | 4.437067 | | | | 3925.797 | | | 522.9219 | |  |  |  |  |  |
| 24.55 | | | 291.4354 | | | | | | | | | | 4.450033 | | | | 3921.234 | | | 526.2344 | |  |  |  |  |  |
| 24.55833 | | | 291.5299 | | | | | | | | | | 4.465357 | | | | 3916.633 | | | 529.9688 | |  |  |  |  |  |
| 24.56667 | | | 291.6228 | | | | | | | | | | 4.47673 | | | | 3911.984 | | | 533.3125 | |  |  |  |  |  |
| 24.575 | | | 291.7149 | | | | | | | | | | 4.49012 | | | | 3907.258 | | | 536.5078 | |  |  |  |  |  |
| 24.58333 | | | 291.8093 | | | | | | | | | | 4.504694 | | | | 3902.547 | | | 539.7969 | |  |  |  |  |  |
| 24.59167 | | | 291.9038 | | | | | | | | | | 4.516504 | | | | 3897.813 | | | 543.3438 | |  |  |  |  |  |
| 24.6 | | | 291.9959 | | | | | | | | | | 4.530898 | | | | 3893.047 | | | 547.1563 | |  |  |  |  |  |
| 24.60833 | | | 292.0903 | | | | | | | | | | 4.544969 | | | | 3888.195 | | | 550.625 | |  |  |  |  |  |
| 24.61667 | | | 292.1878 | | | | | | | | | | 4.557256 | | | | 3883.375 | | | 553.8672 | |  |  |  |  |  |
| 24.625 | | | 292.2846 | | | | | | | | | | 4.572383 | | | | 3878.531 | | | 557.1094 | |  |  |  |  |  |
| 24.63333 | | | 292.3782 | | | | | | | | | | 4.586019 | | | | 3873.664 | | | 560.9922 | |  |  |  |  |  |
| 24.64167 | | | 292.4742 | | | | | | | | | | 4.598684 | | | | 3868.719 | | | 564.5469 | |  |  |  |  |  |
| 24.65 | | | 292.5717 | | | | | | | | | | 4.613958 | | | | 3863.719 | | | 567.9063 | |  |  |  |  |  |
| 24.65833 | | | 292.6708 | | | | | | | | | | 4.626526 | | | | 3858.734 | | | 571.4375 | |  |  |  |  |  |
| 24.66667 | | | 292.766 | | | | | | | | | | 4.640824 | | | | 3853.742 | | | 575.2813 | |  |  |  |  |  |
| 24.675 | | | 292.8596 | | | | | | | | | | 4.657339 | | | | 3848.734 | | | 579.1563 | |  |  |  |  |  |
| 24.68333 | | | 292.9579 | | | | | | | | | | 4.669844 | | | | 3843.602 | | | 582.375 | |  |  |  |  |  |
| 24.69167 | | | 293.0562 | | | | | | | | | | 4.685474 | | | | 3838.5 | | | 585.5703 | |  |  |  |  |  |
| 24.7 | | | 293.1522 | | | | | | | | | | 4.701731 | | | | 3833.367 | | | 588.7891 | |  |  |  |  |  |
| 24.70833 | | | 293.2481 | | | | | | | | | | 4.714721 | | | | 3828.211 | | | 593.0703 | |  |  |  |  |  |
| 24.71667 | | | 293.3448 | | | | | | | | | | 4.730523 | | | | 3822.953 | | | 596.5234 | |  |  |  |  |  |
| 24.725 | | | 293.4431 | | | | | | | | | | 4.746521 | | | | 3817.688 | | | 599.875 | |  |  |  |  |  |
| 24.73333 | | | 293.5383 | | | | | | | | | | 4.76059 | | | | 3812.469 | | | 603.25 | |  |  |  |  |  |
| 24.74167 | | | 293.6335 | | | | | | | | | | 4.777906 | | | | 3807.234 | | | 607.2734 | |  |  |  |  |  |
| 24.75 | | | 293.7318 | | | | | | | | | | 4.793304 | | | | 3801.953 | | | 611.0156 | |  |  |  |  |  |
| 24.75833 | | | 293.8308 | | | | | | | | | | 4.808398 | | | | 3796.516 | | | 614.4297 | |  |  |  |  |  |
| 24.76667 | | | 293.9276 | | | | | | | | | | 4.825745 | | | | 3791.148 | | | 617.9922 | |  |  |  |  |  |
| 24.775 | | | 294.0243 | | | | | | | | | | 4.839963 | | | | 3785.758 | | | 621.6016 | |  |  |  |  |  |
| 24.78333 | | | 294.1203 | | | | | | | | | | 4.857034 | | | | 3780.352 | | | 625.7109 | |  |  |  |  |  |
| 24.79167 | | | 294.217 | | | | | | | | | | 4.874963 | | | | 3774.813 | | | 628.9297 | |  |  |  |  |  |
| 24.8 | | | 294.3176 | | | | | | | | | | 4.889565 | | | | 3769.305 | | | 632.1641 | |  |  |  |  |  |
| 24.80833 | | | 294.4167 | | | | | | | | | | 4.907763 | | | | 3763.789 | | | 635.4688 | |  |  |  |  |  |
| 24.81667 | | | 294.5149 | | | | | | | | | | 4.92505 | | | | 3758.234 | | | 639.375 | |  |  |  |  |  |
| 24.825 | | | 294.614 | | | | | | | | | | 4.940783 | | | | 3752.641 | | | 642.75 | |  |  |  |  |  |
| 24.83333 | | | 294.713 | | | | | | | | | | 4.959407 | | | | 3746.945 | | | 645.8359 | |  |  |  |  |  |
| 24.84167 | | | 294.809 | | | | | | | | | | 4.975727 | | | | 3741.352 | | | 648.8594 | |  |  |  |  |  |
| 24.85 | | | 294.908 | | | | | | | | | | 4.992779 | | | | 3735.711 | | | 652.2266 | |  |  |  |  |  |
| 24.85833 | | | 295.0048 | | | | | | | | | | 5.011624 | | | | 3730.055 | | | 655.9297 | |  |  |  |  |  |
| 24.86667 | | | 295.1015 | | | | | | | | | | 5.027253 | | | | 3724.273 | | | 658.875 | |  |  |  |  |  |
| 24.875 | | | 295.1998 | | | | | | | | | | 5.045317 | | | | 3718.539 | | | 661.9375 | |  |  |  |  |  |
| 24.88333 | | | 295.298 | | | | | | | | | | 5.064629 | | | | 3712.82 | | | 664.875 | |  |  |  |  |  |
| 24.89167 | | | 295.3963 | | | | | | | | | | 5.079703 | | | | 3707.086 | | | 668.4766 | |  |  |  |  |  |
| 24.9 | | | 295.4923 | | | | | | | | | | 5.099363 | | | | 3701.266 | | | 671.5078 | |  |  |  |  |  |
| 24.90833 | | | 295.5898 | | | | | | | | | | 5.118058 | | | | 3695.383 | | | 674.1328 | |  |  |  |  |  |
| 24.91667 | | | 295.6881 | | | | | | | | | | 5.134324 | | | | 3689.57 | | | 677.3047 | |  |  |  |  |  |
| 24.925 | | | 295.7894 | | | | | | | | | | 5.154966 | | | | 3683.719 | | | 680.4688 | |  |  |  |  |  |
| 24.93333 | | | 295.8877 | | | | | | | | | | 5.173973 | | | | 3677.859 | | | 683.6094 | |  |  |  |  |  |
| 24.94167 | | | 295.9867 | | | | | | | | | | 5.192103 | | | | 3671.867 | | | 686.1875 | |  |  |  |  |  |
| 24.95 | | | 296.0888 | | | | | | | | | | 5.212753 | | | | 3665.953 | | | 688.6484 | |  |  |  |  |  |
| 24.95833 | | | 296.1902 | | | | | | | | | | 5.230351 | | | | 3660.047 | | | 691.3047 | |  |  |  |  |  |
| 24.96667 | | | 296.2877 | | | | | | | | | | 5.250351 | | | | 3654.055 | | | 694.4766 | |  |  |  |  |  |
| 24.975 | | | 296.3844 | | | | | | | | | | 5.270949 | | | | 3648.031 | | | 696.9453 | |  |  |  |  |  |
| 24.98333 | | | 296.4858 | | | | | | | | | | 5.28784 | | | | 3642 | | | 699.1563 | |  |  |  |  |  |
| 24.99167 | | | 296.5856 | | | | | | | | | | 5.309117 | | | | 3636 | | | 701.1328 | |  |  |  |  |  |
| 25 | | | 296.68 | | | | | | | | | | 5.328836 | | | | 3630 | | | 703.4766 | |  |  |  |  |  |
| 25.00833 | | | 296.7775 | | | | | | | | | | 5.345959 | | | | 3623.961 | | | 705.8438 | |  |  |  |  |  |
| 25.01667 | | | 296.875 | | | | | | | | | | 5.366763 | | | | 3617.813 | | | 707.7109 | |  |  |  |  |  |
| 25.025 | | | 296.9733 | | | | | | | | | | 5.38569 | | | | 3611.75 | | | 709.3906 | |  |  |  |  |  |
| 25.03333 | | | 297.0708 | | | | | | | | | | 5.404839 | | | | 3605.695 | | | 711.4297 | |  |  |  |  |  |
| 25.04167 | | | 297.1675 | | | | | | | | | | 5.426442 | | | | 3599.664 | | | 713.8047 | |  |  |  |  |  |
| 25.05 | | | 297.265 | | | | | | | | | | 5.444976 | | | | 3593.547 | | | 715.5234 | |  |  |  |  |  |
| 25.05833 | | | 297.3641 | | | | | | | | | | 5.465264 | | | | 3587.43 | | | 717.1719 | |  |  |  |  |  |
| 25.06667 | | | 297.4616 | | | | | | | | | | 5.485762 | | | | 3581.336 | | | 718.8828 | |  |  |  |  |  |
| 25.075 | | | 297.5622 | | | | | | | | | | 5.503833 | | | | 3575.266 | | | 721.0391 | |  |  |  |  |  |
| 25.08333 | | | 297.6643 | | | | | | | | | | 5.526195 | | | | 3569.125 | | | 722.6406 | |  |  |  |  |  |
| 25.09167 | | | 297.7618 | | | | | | | | | | 5.546941 | | | | 3562.93 | | | 724.0469 | |  |  |  |  |  |
| 25.1 | | | 297.8585 | | | | | | | | | | 5.565043 | | | | 3556.805 | | | 724.9609 | |  |  |  |  |  |
| 25.10833 | | | 297.9606 | | | | | | | | | | 5.587423 | | | | 3550.664 | | | 726.25 | |  |  |  |  |  |
| 25.11667 | | | 298.0597 | | | | | | | | | | 5.607028 | | | | 3544.508 | | | 727.6641 | |  |  |  |  |  |
| 25.125 | | | 298.158 | | | | | | | | | | 5.626158 | | | | 3538.266 | | | 728.5156 | |  |  |  |  |  |
| 25.13333 | | | 298.2562 | | | | | | | | | | 5.647481 | | | | 3532.086 | | | 728.9844 | |  |  |  |  |  |
| 25.14167 | | | 298.356 | | | | | | | | | | 5.665912 | | | | 3525.906 | | | 729.3359 | |  |  |  |  |  |
| 25.15 | | | 298.4566 | | | | | | | | | | 5.686856 | | | | 3519.813 | | | 730.1406 | |  |  |  |  |  |
| 25.15833 | | | 298.5541 | | | | | | | | | | 5.707832 | | | | 3513.625 | | | 730.875 | |  |  |  |  |  |
| 25.16667 | | | 298.6539 | | | | | | | | | | 5.725834 | | | | 3507.438 | | | 730.7188 | |  |  |  |  |  |
| 25.175 | | | 298.7568 | | | | | | | | | | 5.748203 | | | | 3501.297 | | | 730.5625 | |  |  |  |  |  |
| 25.18333 | | | 298.862 | | | | | | | | | | 5.767943 | | | | 3495.211 | | | 730.9688 | |  |  |  |  |  |
| 25.19167 | | | 298.9587 | | | | | | | | | | 5.787405 | | | | 3489.133 | | | 731.1719 | |  |  |  |  |  |
| 25.2 | | | 299.0578 | | | | | | | | | | 5.809247 | | | | 3482.984 | | | 730.8828 | |  |  |  |  |  |
| 25.20833 | | | 299.1541 | | | | | | | | | | 5.827746 | | | | 3476.859 | | | 730.1875 | |  |  |  |  |  |
| 25.21667 | | | 299.252 | | | | | | | | | | 5.848035 | | | | 3470.82 | | | 729.6563 | |  |  |  |  |  |
| 25.225 | | | 299.3483 | | | | | | | | | | 5.869044 | | | | 3464.805 | | | 729.5078 | |  |  |  |  |  |
| 25.23333 | | | 299.4461 | | | | | | | | | | 5.886819 | | | | 3458.695 | | | 728.9063 | |  |  |  |  |  |
| 25.24167 | | | 299.5424 | | | | | | | | | | 5.907542 | | | | 3452.609 | | | 727.625 | |  |  |  |  |  |
| 25.25 | | | 299.6388 | | | | | | | | | | 5.926947 | | | | 3446.602 | | | 726.4688 | |  |  |  |  |  |
| 25.25833 | | | 299.7343 | | | | | | | | | | 5.944125 | | | | 3440.633 | | | 725.7813 | |  |  |  |  |  |
| 25.26667 | | | 299.8269 | | | | | | | | | | 5.965576 | | | | 3434.672 | | | 724.9531 | |  |  |  |  |  |
| 25.275 | | | 299.924 | | | | | | | | | | 5.983231 | | | | 3428.633 | | | 723.7344 | |  |  |  |  |  |
| 25.28333 | | | 300.0211 | | | | | | | | | | 5.999946 | | | | 3422.68 | | | 722.2266 | |  |  |  |  |  |
| 25.29167 | | | 300.1212 | | | | | | | | | | 6.020543 | | | | 3416.797 | | | 720.7891 | |  |  |  |  |  |
| 25.3 | | | 300.2221 | | | | | | | | | | 6.037713 | | | | 3410.93 | | | 719.8906 | |  |  |  |  |  |
| 25.30833 | | | 300.3214 | | | | | | | | | | 6.056065 | | | | 3405 | | | 718.1484 | |  |  |  |  |  |
| 25.31667 | | | 300.4185 | | | | | | | | | | 6.075283 | | | | 3399.078 | | | 716.0625 | |  |  |  |  |  |
| 25.325 | | | 300.5171 | | | | | | | | | | 6.091539 | | | | 3393.227 | | | 714.1016 | |  |  |  |  |  |
| 25.33333 | | | 300.615 | | | | | | | | | | 6.110792 | | | | 3387.414 | | | 712.6172 | |  |  |  |  |  |
| 25.34167 | | | 300.7113 | | | | | | | | | | 6.128267 | | | | 3381.617 | | | 710.5781 | |  |  |  |  |  |
| 25.35 | | | 300.8091 | | | | | | | | | | 6.143687 | | | | 3375.742 | | | 708.0547 | |  |  |  |  |  |
| 25.35833 | | | 300.907 | | | | | | | | | | 6.16375 | | | | 3370.008 | | | 705.5078 | |  |  |  |  |  |
| 25.36667 | | | 301.0079 | | | | | | | | | | 6.179712 | | | | 3364.313 | | | 702.9219 | |  |  |  |  |  |
| 25.375 | | | 301.1065 | | | | | | | | | | 6.196339 | | | | 3358.633 | | | 700.6953 | |  |  |  |  |  |
| 25.38333 | | | 301.2028 | | | | | | | | | | 6.214537 | | | | 3352.891 | | | 697.8516 | |  |  |  |  |  |
| 25.39167 | | | 301.2968 | | | | | | | | | | 6.229175 | | | | 3347.25 | | | 694.9609 | |  |  |  |  |  |
| 25.4 | | | 301.3924 | | | | | | | | | | 6.244841 | | | | 3341.672 | | | 692 | |  |  |  |  |  |
| 25.40833 | | | 301.4903 | | | | | | | | | | 6.261159 | | | | 3336.141 | | | 689.5 | |  |  |  |  |  |
| 25.41667 | | | 301.5873 | | | | | | | | | | 6.274472 | | | | 3330.609 | | | 686.4531 | |  |  |  |  |  |
| 25.425 | | | 301.6859 | | | | | | | | | | 6.290611 | | | | 3325.063 | | | 683.1484 | |  |  |  |  |  |
| 25.43333 | | | 301.7823 | | | | | | | | | | 6.304442 | | | | 3319.617 | | | 679.5859 | |  |  |  |  |  |
| 25.44167 | | | 301.8801 | | | | | | | | | | 6.317482 | | | | 3314.188 | | | 676.4219 | |  |  |  |  |  |
| 25.45 | | | 301.9742 | | | | | | | | | | 6.334106 | | | | 3308.789 | | | 673.5938 | |  |  |  |  |  |
| 25.45833 | | | 302.0659 | | | | | | | | | | 6.346628 | | | | 3303.359 | | | 669.9766 | |  |  |  |  |  |
| 25.46667 | | | 302.157 | | | | | | | | | | 6.358787 | | | | 3298.023 | | | 666.3906 | |  |  |  |  |  |
| 25.475 | | | 302.2541 | | | | | | | | | | 6.374245 | | | | 3292.742 | | | 662.6094 | |  |  |  |  |  |
| 25.48333 | | | 302.3489 | | | | | | | | | | 6.3853 | | | | 3287.516 | | | 659.3984 | |  |  |  |  |  |
| 25.49167 | | | 302.4399 | | | | | | | | | | 6.396729 | | | | 3282.266 | | | 655.875 | |  |  |  |  |  |
| 25.5 | | | 302.5332 | | | | | | | | | | 6.410392 | | | | 3277 | | | 651.7969 | |  |  |  |  |  |
| 25.50833 | | | 302.6257 | | | | | | | | | | 6.420203 | | | | 3271.859 | | | 647.7891 | |  |  |  |  |  |
| 25.51667 | | | 302.7206 | | | | | | | | | | 6.43195 | | | | 3266.742 | | | 644.0547 | |  |  |  |  |  |
| 25.525 | | | 302.8161 | | | | | | | | | | 6.444213 | | | | 3261.68 | | | 640.2422 | |  |  |  |  |  |
| 25.53333 | | | 302.9079 | | | | | | | | | | 6.453346 | | | | 3256.563 | | | 635.6484 | |  |  |  |  |  |
| 25.54167 | | | 303.002 | | | | | | | | | | 6.464297 | | | | 3251.531 | | | 631.2422 | |  |  |  |  |  |
| 25.55 | | | 303.099 | | | | | | | | | | 6.474575 | | | | 3246.594 | | | 626.8203 | |  |  |  |  |  |
| 25.55833 | | | 303.1946 | | | | | | | | | | 6.482983 | | | | 3241.688 | | | 622.8828 | |  |  |  |  |  |
| 25.56667 | | | 303.2902 | | | | | | | | | | 6.49396 | | | | 3236.758 | | | 618.2891 | |  |  |  |  |  |
| 25.575 | | | 303.3857 | | | | | | | | | | 6.502733 | | | | 3231.898 | | | 613.5859 | |  |  |  |  |  |
| 25.58333 | | | 303.4806 | | | | | | | | | | 6.510961 | | | | 3227.141 | | | 608.7734 | |  |  |  |  |  |
| 25.59167 | | | 303.5754 | | | | | | | | | | 6.521989 | | | | 3222.414 | | | 604.5469 | |  |  |  |  |  |
| 25.6 | | | 303.6687 | | | | | | | | | | 6.528719 | | | | 3217.727 | | | 600.0391 | |  |  |  |  |  |
| 25.60833 | | | 303.762 | | | | | | | | | | 6.535743 | | | | 3213 | | | 595.0703 | |  |  |  |  |  |
| 25.61667 | | | 303.8537 | | | | | | | | | | 6.545641 | | | | 3208.406 | | | 589.9766 | |  |  |  |  |  |
| 25.625 | | | 303.9493 | | | | | | | | | | 6.551052 | | | | 3203.852 | | | 585.3203 | |  |  |  |  |  |
| 25.63333 | | | 304.0449 | | | | | | | | | | 6.557365 | | | | 3199.359 | | | 580.875 | |  |  |  |  |  |
| 25.64167 | | | 304.1374 | | | | | | | | | | 6.566567 | | | | 3194.82 | | | 575.7188 | |  |  |  |  |  |
| 25.65 | | | 304.2284 | | | | | | | | | | 6.570808 | | | | 3190.359 | | | 570.5391 | |  |  |  |  |  |
| 25.65833 | | | 304.3217 | | | | | | | | | | 6.576893 | | | | 3185.984 | | | 565.5234 | |  |  |  |  |  |
| 25.66667 | | | 304.4173 | | | | | | | | | | 6.584606 | | | | 3181.672 | | | 561.0938 | |  |  |  |  |  |
| 25.675 | | | 304.5106 | | | | | | | | | | 6.588475 | | | | 3177.336 | | | 556.5 | |  |  |  |  |  |
| 25.68333 | | | 304.6024 | | | | | | | | | | 6.594048 | | | | 3173.023 | | | 551.1484 | |  |  |  |  |  |
| 25.69167 | | | 304.6919 | | | | | | | | | | 6.600178 | | | | 3168.836 | | | 546.0781 | |  |  |  |  |  |
| 25.7 | | | 304.7874 | | | | | | | | | | 6.602624 | | | | 3164.68 | | | 541.4219 | |  |  |  |  |  |
| 25.70833 | | | 304.8792 | | | | | | | | | | 6.607329 | | | | 3160.563 | | | 536.5859 | |  |  |  |  |  |
| 25.71667 | | | 304.9687 | | | | | | | | | | 6.612637 | | | | 3156.391 | | | 531.8047 | |  |  |  |  |  |
| 25.725 | | | 305.0597 | | | | | | | | | | 6.614481 | | | | 3152.281 | | | 527.0703 | |  |  |  |  |  |
| 25.73333 | | | 305.1523 | | | | | | | | | | 6.618745 | | | | 3148.313 | | | 522.0859 | |  |  |  |  |  |
| 25.74167 | | | 305.2456 | | | | | | | | | | 6.622696 | | | | 3144.344 | | | 517.3906 | |  |  |  |  |  |
| 25.75 | | | 305.3336 | | | | | | | | | | 6.623618 | | | | 3140.375 | | | 512.5313 | |  |  |  |  |  |
| 25.75833 | | | 305.4238 | | | | | | | | | | 6.626471 | | | | 3136.453 | | | 507.2734 | |  |  |  |  |  |
| 25.76667 | | | 305.5148 | | | | | | | | | | 6.629111 | | | | 3132.578 | | | 502.5156 | |  |  |  |  |  |
| 25.775 | | | 305.6081 | | | | | | | | | | 6.629197 | | | | 3128.719 | | | 498.0234 | |  |  |  |  |  |
| 25.78333 | | | 305.6999 | | | | | | | | | | 6.631528 | | | | 3124.945 | | | 493.4297 | |  |  |  |  |  |
| 25.79167 | | | 305.7909 | | | | | | | | | | 6.632868 | | | | 3121.164 | | | 488.5781 | |  |  |  |  |  |
| 25.8 | | | 305.8819 | | | | | | | | | | 6.632092 | | | | 3117.453 | | | 483.6484 | |  |  |  |  |  |
| 25.80833 | | | 305.9753 | | | | | | | | | | 6.634184 | | | | 3113.828 | | | 478.9609 | |  |  |  |  |  |
| 25.81667 | | | 306.0685 | | | | | | | | | | 6.634661 | | | | 3110.172 | | | 474.5156 | |  |  |  |  |  |
| 25.825 | | | 306.1595 | | | | | | | | | | 6.633947 | | | | 3106.531 | | | 469.6797 | |  |  |  |  |  |
| 25.83333 | | | 306.2506 | | | | | | | | | | 6.635245 | | | | 3102.93 | | | 464.75 | |  |  |  |  |  |
| 25.84167 | | | 306.3423 | | | | | | | | | | 6.633998 | | | | 3099.414 | | | 459.9844 | |  |  |  |  |  |
| 25.85 | | | 306.4341 | | | | | | | | | | 6.63236 | | | | 3095.938 | | | 455.5234 | |  |  |  |  |  |
| 25.85833 | | | 306.5236 | | | | | | | | | | 6.632997 | | | | 3092.469 | | | 450.9922 | |  |  |  |  |  |
| 25.86667 | | | 306.6116 | | | | | | | | | | 6.630991 | | | | 3089.008 | | | 446.2109 | |  |  |  |  |  |
| 25.875 | | | 306.7003 | | | | | | | | | | 6.629547 | | | | 3085.648 | | | 441.6094 | |  |  |  |  |  |
| 25.88333 | | | 306.7899 | | | | | | | | | | 6.629851 | | | | 3082.32 | | | 437.3125 | |  |  |  |  |  |
| 25.89167 | | | 306.8778 | | | | | | | | | | 6.627283 | | | | 3079.016 | | | 433.0547 | |  |  |  |  |  |
| 25.9 | | | 306.9643 | | | | | | | | | | 6.624447 | | | | 3075.719 | | | 428.5781 | |  |  |  |  |  |
| 25.90833 | | | 307.0523 | | | | | | | | | | 6.62336 | | | | 3072.469 | | | 424.3125 | |  |  |  |  |  |
| 25.91667 | | | 307.1411 | | | | | | | | | | 6.62039 | | | | 3069.281 | | | 420.125 | |  |  |  |  |  |
| 25.925 | | | 307.2305 | | | | | | | | | | 6.6164 | | | | 3066.109 | | | 416.1172 | |  |  |  |  |  |
| 25.93333 | | | 307.3208 | | | | | | | | | | 6.614685 | | | | 3062.953 | | | 412.0859 | |  |  |  |  |  |
| 25.94167 | | | 307.4103 | | | | | | | | | | 6.611249 | | | | 3059.813 | | | 407.625 | |  |  |  |  |  |
| 25.95 | | | 307.4998 | | | | | | | | | | 6.606539 | | | | 3056.742 | | | 403.2813 | |  |  |  |  |  |
| 25.95833 | | | 307.5901 | | | | | | | | | | 6.604326 | | | | 3053.664 | | | 399.4297 | |  |  |  |  |  |
| 25.96667 | | | 307.6788 | | | | | | | | | | 6.600458 | | | | 3050.633 | | | 395.4375 | |  |  |  |  |  |
| 25.975 | | | 307.7675 | | | | | | | | | | 6.595734 | | | | 3047.602 | | | 391.4688 | |  |  |  |  |  |
| 25.98333 | | | 307.8593 | | | | | | | | | | 6.593478 | | | | 3044.625 | | | 387.4766 | |  |  |  |  |  |
| 25.99167 | | | 307.9496 | | | | | | | | | | 6.589462 | | | | 3041.711 | | | 383.3438 | |  |  |  |  |  |
| 26 | | | 308.0421 | | | | | | | | | | 6.584374 | | | | 3038.836 | | | 379.6563 | |  |  |  |  |  |
| 26.00833 | | | 308.1316 | | | | | | | | | | 6.581833 | | | | 3035.93 | | | 375.8047 | |  |  |  |  |  |
| 26.01667 | | | 308.2189 | | | | | | | | | | 6.577852 | | | | 3033.086 | | | 371.8984 | |  |  |  |  |  |
| 26.025 | | | 308.3076 | | | | | | | | | | 6.572799 | | | | 3030.25 | | | 368.0703 | |  |  |  |  |  |
| 26.03333 | | | 308.3986 | | | | | | | | | | 6.570264 | | | | 3027.461 | | | 364.8672 | |  |  |  |  |  |
| 26.04167 | | | 308.4889 | | | | | | | | | | 6.565866 | | | | 3024.734 | | | 361.4219 | |  |  |  |  |  |
| 26.05 | | | 308.5769 | | | | | | | | | | 6.560099 | | | | 3021.969 | | | 357.8047 | |  |  |  |  |  |
| 26.05833 | | | 308.6641 | | | | | | | | | | 6.556888 | | | | 3019.266 | | | 354.2266 | |  |  |  |  |  |
| 26.06667 | | | 308.7551 | | | | | | | | | | 6.551804 | | | | 3016.602 | | | 350.7422 | |  |  |  |  |  |
| 26.075 | | | 308.8439 | | | | | | | | | | 6.545632 | | | | 3013.945 | | | 347.6953 | |  |  |  |  |  |
| 26.08333 | | | 308.9288 | | | | | | | | | | 6.5423 | | | | 3011.25 | | | 344.6172 | |  |  |  |  |  |
| 26.09167 | | | 309.0153 | | | | | | | | | | 6.537811 | | | | 3008.617 | | | 341.3516 | |  |  |  |  |  |
| 26.1 | | | 309.1033 | | | | | | | | | | 6.531146 | | | | 3006.023 | | | 338.2656 | |  |  |  |  |  |
| 26.10833 | | | 309.192 | | | | | | | | | | 6.527687 | | | | 3003.469 | | | 335.5078 | |  |  |  |  |  |
| 26.11667 | | | 309.28 | | | | | | | | | | 6.522996 | | | | 3000.93 | | | 332.5234 | |  |  |  |  |  |
| 26.125 | | | 309.368 | | | | | | | | | | 6.515632 | | | | 2998.344 | | | 329.3203 | |  |  |  |  |  |
| 26.13333 | | | 309.4575 | | | | | | | | | | 6.512118 | | | | 2995.805 | | | 326.3125 | |  |  |  |  |  |
| 26.14167 | | | 309.5485 | | | | | | | | | | 6.507131 | | | | 2993.305 | | | 323.2734 | |  |  |  |  |  |
| 26.15 | | | 309.6342 | | | | | | | | | | 6.499615 | | | | 2990.813 | | | 320.8125 | |  |  |  |  |  |
| 26.15833 | | | 309.7222 | | | | | | | | | | 6.49615 | | | | 2988.32 | | | 317.9844 | |  |  |  |  |  |
| 26.16667 | | | 309.8117 | | | | | | | | | | 6.491594 | | | | 2985.859 | | | 314.7422 | |  |  |  |  |  |
| 26.175 | | | 309.9005 | | | | | | | | | | 6.483912 | | | | 2983.469 | | | 312.125 | |  |  |  |  |  |
| 26.18333 | | | 309.993 | | | | | | | | | | 6.480329 | | | | 2981.063 | | | 309.5 | |  |  |  |  |  |
| 26.19167 | | | 310.0802 | | | | | | | | | | 6.475446 | | | | 2978.711 | | | 306.9297 | |  |  |  |  |  |
| 26.2 | | | 310.1682 | | | | | | | | | | 6.467537 | | | | 2976.281 | | | 304.0547 | |  |  |  |  |  |
| 26.20833 | | | 310.2577 | | | | | | | | | | 6.464157 | | | | 2973.938 | | | 301.7188 | |  |  |  |  |  |
| 26.21667 | | | 310.3487 | | | | | | | | | | 6.459095 | | | | 2971.672 | | | 299.2969 | |  |  |  |  |  |
| 26.225 | | | 310.4382 | | | | | | | | | | 6.451046 | | | | 2969.359 | | | 296.9063 | |  |  |  |  |  |
| 26.23333 | | | 310.5255 | | | | | | | | | | 6.447227 | | | | 2967.063 | | | 294.1563 | |  |  |  |  |  |
| 26.24167 | | | 310.6127 | | | | | | | | | | 6.441747 | | | | 2964.781 | | | 291.6328 | |  |  |  |  |  |
| 26.25 | | | 310.7022 | | | | | | | | | | 6.433229 | | | | 2962.555 | | | 289.4375 | |  |  |  |  |  |
| 26.25833 | | | 310.7894 | | | | | | | | | | 6.429195 | | | | 2960.289 | | | 287.1406 | |  |  |  |  |  |
| 26.26667 | | | 310.8766 | | | | | | | | | | 6.424215 | | | | 2958.055 | | | 284.8672 | |  |  |  |  |  |
| 26.275 | | | 310.9631 | | | | | | | | | | 6.416394 | | | | 2955.828 | | | 282.4063 | |  |  |  |  |  |
| 26.28333 | | | 311.0503 | | | | | | | | | | 6.412019 | | | | 2953.688 | | | 280.0938 | |  |  |  |  |  |
| 26.29167 | | | 311.1383 | | | | | | | | | | 6.40706 | | | | 2951.523 | | | 277.9375 | |  |  |  |  |  |
| 26.3 | | | 311.2233 | | | | | | | | | | 6.399314 | | | | 2949.352 | | | 275.7188 | |  |  |  |  |  |
| 26.30833 | | | 311.3074 | | | | | | | | | | 6.394282 | | | | 2947.203 | | | 273.5703 | |  |  |  |  |  |
| 26.31667 | | | 311.3962 | | | | | | | | | | 6.389717 | | | | 2945.078 | | | 271.7109 | |  |  |  |  |  |
| 26.325 | | | 311.4857 | | | | | | | | | | 6.382619 | | | | 2942.984 | | | 269.3594 | |  |  |  |  |  |
| 26.33333 | | | 311.5744 | | | | | | | | | | 6.377338 | | | | 2940.898 | | | 267.3984 | |  |  |  |  |  |
| 26.34167 | | | 311.6624 | | | | | | | | | | 6.372912 | | | | 2938.828 | | | 265.3594 | |  |  |  |  |  |
| 26.35 | | | 311.7489 | | | | | | | | | | 6.365944 | | | | 2936.773 | | | 263.3281 | |  |  |  |  |  |
| 26.35833 | | | 311.8384 | | | | | | | | | | 6.360721 | | | | 2934.719 | | | 261.2969 | |  |  |  |  |  |
| 26.36667 | | | 311.9279 | | | | | | | | | | 6.356132 | | | | 2932.664 | | | 259.5469 | |  |  |  |  |  |
| 26.375 | | | 312.0151 | | | | | | | | | | 6.349176 | | | | 2930.664 | | | 257.7109 | |  |  |  |  |  |
| 26.38333 | | | 312.1077 | | | | | | | | | | 6.344245 | | | | 2928.648 | | | 255.8594 | |  |  |  |  |  |
| 26.39167 | | | 312.1987 | | | | | | | | | | 6.340048 | | | | 2926.672 | | | 253.9922 | |  |  |  |  |  |
| 26.4 | | | 312.2874 | | | | | | | | | | 6.333375 | | | | 2924.695 | | | 252.1641 | |  |  |  |  |  |
| 26.40833 | | | 312.3739 | | | | | | | | | | 6.328657 | | | | 2922.742 | | | 250.7891 | |  |  |  |  |  |
| 26.41667 | | | 312.4611 | | | | | | | | | | 6.324617 | | | | 2920.766 | | | 249.0156 | |  |  |  |  |  |
| 26.425 | | | 312.5499 | | | | | | | | | | 6.318117 | | | | 2918.82 | | | 247.2422 | |  |  |  |  |  |
| 26.43333 | | | 312.6401 | | | | | | | | | | 6.312927 | | | | 2916.883 | | | 245.4063 | |  |  |  |  |  |
| 26.44167 | | | 312.7296 | | | | | | | | | | 6.308789 | | | | 2914.969 | | | 243.9688 | |  |  |  |  |  |
| 26.45 | | | 312.8168 | | | | | | | | | | 6.302207 | | | | 2913.07 | | | 242.2656 | |  |  |  |  |  |
| 26.45833 | | | 312.9026 | | | | | | | | | | 6.296638 | | | | 2911.117 | | | 240.7266 | |  |  |  |  |  |
| 26.46667 | | | 312.9883 | | | | | | | | | | 6.292883 | | | | 2909.242 | | | 239.1563 | |  |  |  |  |  |
| 26.475 | | | 313.0755 | | | | | | | | | | 6.286064 | | | | 2907.367 | | | 237.4688 | |  |  |  |  |  |
| 26.48333 | | | 313.1627 | | | | | | | | | | 6.279535 | | | | 2905.523 | | | 235.875 | |  |  |  |  |  |
| 26.49167 | | | 313.2484 | | | | | | | | | | 6.275692 | | | | 2903.641 | | | 234.1797 | |  |  |  |  |  |
| 26.5 | | | 313.3341 | | | | | | | | | | 6.26932 | | | | 2901.797 | | | 232.4453 | |  |  |  |  |  |
| 26.50833 | | | 313.4244 | | | | | | | | | | 6.262544 | | | | 2899.961 | | | 231.1641 | |  |  |  |  |  |
| 26.51667 | | | 313.5132 | | | | | | | | | | 6.259397 | | | | 2898.133 | | | 229.9609 | |  |  |  |  |  |
| 26.525 | | | 313.6004 | | | | | | | | | | 6.253194 | | | | 2896.344 | | | 228.5859 | |  |  |  |  |  |
| 26.53333 | | | 313.6891 | | | | | | | | | | 6.246231 | | | | 2894.547 | | | 227.0547 | |  |  |  |  |  |
| 26.54167 | | | 313.7786 | | | | | | | | | | 6.244178 | | | | 2892.789 | | | 225.3594 | |  |  |  |  |  |
| 26.55 | | | 313.8674 | | | | | | | | | | 6.238117 | | | | 2891.031 | | | 224.1328 | |  |  |  |  |  |
| 26.55833 | | | 313.9554 | | | | | | | | | | 6.231399 | | | | 2889.25 | | | 222.9219 | |  |  |  |  |  |
| 26.56667 | | | 314.0449 | | | | | | | | | | 6.229748 | | | | 2887.461 | | | 221.6875 | |  |  |  |  |  |
| 26.575 | | | 314.1374 | | | | | | | | | | 6.224348 | | | | 2885.703 | | | 220.3125 | |  |  |  |  |  |
| 26.58333 | | | 314.2254 | | | | | | | | | | 6.218008 | | | | 2883.977 | | | 219.125 | |  |  |  |  |  |
| 26.59167 | | | 314.3126 | | | | | | | | | | 6.215986 | | | | 2882.289 | | | 217.9922 | |  |  |  |  |  |
| 26.6 | | | 314.3983 | | | | | | | | | | 6.210549 | | | | 2880.555 | | | 216.6094 | |  |  |  |  |  |
| 26.60833 | | | 314.4886 | | | | | | | | | | 6.204026 | | | | 2878.813 | | | 215.2813 | |  |  |  |  |  |
| 26.61667 | | | 314.5735 | | | | | | | | | | 6.201595 | | | | 2877.102 | | | 214.2656 | |  |  |  |  |  |
| 26.625 | | | 314.6623 | | | | | | | | | | 6.196252 | | | | 2875.414 | | | 213.1484 | |  |  |  |  |  |
| 26.63333 | | | 314.7502 | | | | | | | | | | 6.190043 | | | | 2873.719 | | | 211.8906 | |  |  |  |  |  |
| 26.64167 | | | 314.8367 | | | | | | | | | | 6.187521 | | | | 2872.023 | | | 210.6641 | |  |  |  |  |  |
| 26.65 | | | 314.9232 | | | | | | | | | | 6.182533 | | | | 2870.367 | | | 209.7031 | |  |  |  |  |  |
| 26.65833 | | | 315.0119 | | | | | | | | | | 6.176386 | | | | 2868.719 | | | 208.6016 | |  |  |  |  |  |
| 26.66667 | | | 315.1045 | | | | | | | | | | 6.173357 | | | | 2867.039 | | | 207.7031 | |  |  |  |  |  |
| 26.675 | | | 315.1947 | | | | | | | | | | 6.168698 | | | | 2865.375 | | | 206.4922 | |  |  |  |  |  |
| 26.68333 | | | 315.282 | | | | | | | | | | 6.162531 | | | | 2863.75 | | | 205.3359 | |  |  |  |  |  |
| 26.69167 | | | 315.3684 | | | | | | | | | | 6.159498 | | | | 2862.133 | | | 204.4375 | |  |  |  |  |  |
| 26.7 | | | 315.4617 | | | | | | | | | | 6.155051 | | | | 2860.5 | | | 203.2891 | |  |  |  |  |  |
| 26.70833 | | | 315.5512 | | | | | | | | | | 6.149396 | | | | 2858.875 | | | 202.2891 | |  |  |  |  |  |
| 26.71667 | | | 315.6407 | | | | | | | | | | 6.146793 | | | | 2857.227 | | | 201.1094 | |  |  |  |  |  |
| 26.725 | | | 315.7311 | | | | | | | | | | 6.142605 | | | | 2855.648 | | | 199.9922 | |  |  |  |  |  |
| 26.73333 | | | 315.8195 | | | | | | | | | | 6.137378 | | | | 2854.055 | | | 198.7734 | |  |  |  |  |  |
| 26.74167 | | | 315.9057 | | | | | | | | | | 6.134719 | | | | 2852.445 | | | 197.8203 | |  |  |  |  |  |
| 26.75 | | | 315.9904 | | | | | | | | | | 6.131052 | | | | 2850.891 | | | 196.8203 | |  |  |  |  |  |
| 26.75833 | | | 316.0742 | | | | | | | | | | 6.125966 | | | | 2849.305 | | | 195.9219 | |  |  |  |  |  |
| 26.76667 | | | 316.1627 | | | | | | | | | | 6.123302 | | | | 2847.766 | | | 194.9844 | |  |  |  |  |  |
| 26.775 | | | 316.2511 | | | | | | | | | | 6.120466 | | | | 2846.227 | | | 193.9453 | |  |  |  |  |  |
| 26.78333 | | | 316.3357 | | | | | | | | | | 6.115659 | | | | 2844.703 | | | 193.1719 | |  |  |  |  |  |
| 26.79167 | | | 316.4226 | | | | | | | | | | 6.112814 | | | | 2843.156 | | | 192.1641 | |  |  |  |  |  |
| 26.8 | | | 316.5073 | | | | | | | | | | 6.110293 | | | | 2841.641 | | | 191.375 | |  |  |  |  |  |
| 26.80833 | | | 316.5935 | | | | | | | | | | 6.1051 | | | | 2840.086 | | | 190.5078 | |  |  |  |  |  |
| 26.81667 | | | 316.6811 | | | | | | | | | | 6.101573 | | | | 2838.563 | | | 189.6797 | |  |  |  |  |  |
| 26.825 | | | 316.7695 | | | | | | | | | | 6.099848 | | | | 2837.063 | | | 188.875 | |  |  |  |  |  |
| 26.83333 | | | 316.8617 | | | | | | | | | | 6.094587 | | | | 2835.539 | | | 188.0625 | |  |  |  |  |  |
| 26.84167 | | | 316.9531 | | | | | | | | | | 6.091271 | | | | 2834.039 | | | 186.875 | |  |  |  |  |  |
| 26.85 | | | 317.0423 | | | | | | | | | | 6.089074 | | | | 2832.539 | | | 186.125 | |  |  |  |  |  |
| 26.85833 | | | 317.1284 | | | | | | | | | | 6.08376 | | | | 2831.039 | | | 185.2656 | |  |  |  |  |  |
| 26.86667 | | | 317.2176 | | | | | | | | | | 6.080742 | | | | 2829.539 | | | 184.2266 | |  |  |  |  |  |
| 26.875 | | | 317.3052 | | | | | | | | | | 6.07863 | | | | 2828.063 | | | 183.3594 | |  |  |  |  |  |
| 26.88333 | | | 317.3959 | | | | | | | | | | 6.073316 | | | | 2826.586 | | | 182.5547 | |  |  |  |  |  |
| 26.89167 | | | 317.4843 | | | | | | | | | | 6.070597 | | | | 2825.172 | | | 181.7813 | |  |  |  |  |  |
| 26.9 | | | 317.5727 | | | | | | | | | | 6.068955 | | | | 2823.695 | | | 180.9297 | |  |  |  |  |  |
| 26.90833 | | | 317.6634 | | | | | | | | | | 6.064318 | | | | 2822.25 | | | 179.9375 | |  |  |  |  |  |
| 26.91667 | | | 317.7503 | | | | | | | | | | 6.061605 | | | | 2820.828 | | | 179.0313 | |  |  |  |  |  |
| 26.925 | | | 317.8379 | | | | | | | | | | 6.060439 | | | | 2819.398 | | | 178.5859 | |  |  |  |  |  |
| 26.93333 | | | 317.9271 | | | | | | | | | | 6.055392 | | | | 2817.961 | | | 177.7969 | |  |  |  |  |  |
| 26.94167 | | | 318.0193 | | | | | | | | | | 6.052529 | | | | 2816.539 | | | 177.0938 | |  |  |  |  |  |
| 26.95 | | | 318.1069 | | | | | | | | | | 6.051166 | | | | 2815.125 | | | 176.3359 | |  |  |  |  |  |
| 26.95833 | | | 318.1946 | | | | | | | | | | 6.045819 | | | | 2813.734 | | | 175.6719 | |  |  |  |  |  |
| 26.96667 | | | 318.2815 | | | | | | | | | | 6.0433 | | | | 2812.344 | | | 175.0781 | |  |  |  |  |  |
| 26.975 | | | 318.3706 | | | | | | | | | | 6.042574 | | | | 2810.898 | | | 174.3281 | |  |  |  |  |  |
| 26.98333 | | | 318.4583 | | | | | | | | | | 6.037282 | | | | 2809.5 | | | 173.6875 | |  |  |  |  |  |
| 26.99167 | | | 318.5422 | | | | | | | | | | 6.034179 | | | | 2808.102 | | | 173.2031 | |  |  |  |  |  |
| 27 | | | 318.6306 | | | | | | | | | | 6.033427 | | | | 2806.711 | | | 172.4531 | |  |  |  |  |  |
| 27.00833 | | | 318.7168 | | | | | | | | | | 6.027549 | | | | 2805.32 | | | 171.6328 | |  |  |  |  |  |
| 27.01667 | | | 318.8029 | | | | | | | | | | 6.024491 | | | | 2803.922 | | | 170.9531 | |  |  |  |  |  |
| 27.025 | | | 318.8883 | | | | | | | | | | 6.02424 | | | | 2802.547 | | | 170.2031 | |  |  |  |  |  |
| 27.03333 | | | 318.9767 | | | | | | | | | | 6.01829 | | | | 2801.164 | | | 169.6797 | |  |  |  |  |  |
| 27.04167 | | | 319.0652 | | | | | | | | | | 6.015972 | | | | 2799.773 | | | 169.0625 | |  |  |  |  |  |
| 27.05 | | | 319.1528 | | | | | | | | | | 6.015397 | | | | 2798.422 | | | 168.3828 | |  |  |  |  |  |
| 27.05833 | | | 319.2405 | | | | | | | | | | 6.010227 | | | | 2797.078 | | | 167.7031 | |  |  |  |  |  |
| 27.06667 | | | 319.3297 | | | | | | | | | | 6.007908 | | | | 2795.734 | | | 167.1016 | |  |  |  |  |  |
| 27.075 | | | 319.4188 | | | | | | | | | | 6.007424 | | | | 2794.398 | | | 166.7578 | |  |  |  |  |  |
| 27.08333 | | | 319.505 | | | | | | | | | | 6.002618 | | | | 2793.039 | | | 166.4688 | |  |  |  |  |  |
| 27.09167 | | | 319.5956 | | | | | | | | | | 6.000561 | | | | 2791.688 | | | 165.8047 | |  |  |  |  |  |
| 27.1 | | | 319.6855 | | | | | | | | | | 6.000541 | | | | 2790.375 | | | 165.1016 | |  |  |  |  |  |
| 27.10833 | | | 319.7762 | | | | | | | | | | 5.99577 | | | | 2789.039 | | | 164.5703 | |  |  |  |  |  |
| 27.11667 | | | 319.8661 | | | | | | | | | | 5.994141 | | | | 2787.719 | | | 164.2266 | |  |  |  |  |  |
| 27.125 | | | 319.9553 | | | | | | | | | | 5.993927 | | | | 2786.359 | | | 163.6484 | |  |  |  |  |  |
| 27.13333 | | | 320.0452 | | | | | | | | | | 5.989816 | | | | 2784.992 | | | 163.125 | |  |  |  |  |  |
| 27.14167 | | | 320.1336 | | | | | | | | | | 5.988007 | | | | 2783.688 | | | 162.5703 | |  |  |  |  |  |
| 27.15 | | | 320.2227 | | | | | | | | | | 5.987708 | | | | 2782.391 | | | 162.2656 | |  |  |  |  |  |
| 27.15833 | | | 320.3081 | | | | | | | | | | 5.983854 | | | | 2781.07 | | | 161.6875 | |  |  |  |  |  |
| 27.16667 | | | 320.3958 | | | | | | | | | | 5.981661 | | | | 2779.742 | | | 161.1406 | |  |  |  |  |  |
| 27.175 | | | 320.4835 | | | | | | | | | | 5.981536 | | | | 2778.445 | | | 160.7813 | |  |  |  |  |  |
| 27.18333 | | | 320.5711 | | | | | | | | | | 5.97798 | | | | 2777.141 | | | 160.2422 | |  |  |  |  |  |
| 27.19167 | | | 320.6565 | | | | | | | | | | 5.976133 | | | | 2775.844 | | | 159.75 | |  |  |  |  |  |
| 27.2 | | | 320.7419 | | | | | | | | | | 5.97585 | | | | 2774.508 | | | 159.2734 | |  |  |  |  |  |
| 27.20833 | | | 320.8296 | | | | | | | | | | 5.972739 | | | | 2773.234 | | | 158.6953 | |  |  |  |  |  |
| 27.21667 | | | 320.9165 | | | | | | | | | | 5.970377 | | | | 2771.945 | | | 158.1719 | |  |  |  |  |  |
| 27.225 | | | 321.0042 | | | | | | | | | | 5.96993 | | | | 2770.648 | | | 157.7969 | |  |  |  |  |  |
| 27.23333 | | | 321.0903 | | | | | | | | | | 5.96715 | | | | 2769.367 | | | 157.1016 | |  |  |  |  |  |
| 27.24167 | | | 321.1825 | | | | | | | | | | 5.964621 | | | | 2768.094 | | | 156.4688 | |  |  |  |  |  |
| 27.25 | | | 321.2686 | | | | | | | | | | 5.964303 | | | | 2766.82 | | | 155.9375 | |  |  |  |  |  |
| 27.25833 | | | 321.3563 | | | | | | | | | | 5.961715 | | | | 2765.563 | | | 155.4063 | |  |  |  |  |  |
| 27.26667 | | | 321.4417 | | | | | | | | | | 5.959071 | | | | 2764.297 | | | 154.9375 | |  |  |  |  |  |
| 27.275 | | | 321.5309 | | | | | | | | | | 5.959058 | | | | 2763.031 | | | 154.4297 | |  |  |  |  |  |
| 27.28333 | | | 321.62 | | | | | | | | | | 5.956882 | | | | 2761.813 | | | 153.8047 | |  |  |  |  |  |
| 27.29167 | | | 321.7099 | | | | | | | | | | 5.954817 | | | | 2760.578 | | | 153.2031 | |  |  |  |  |  |
| 27.3 | | | 321.7976 | | | | | | | | | | 5.954413 | | | | 2759.344 | | | 152.8438 | |  |  |  |  |  |
| 27.30833 | | | 321.886 | | | | | | | | | | 5.951906 | | | | 2758.102 | | | 152.2969 | |  |  |  |  |  |
| 27.31667 | | | 321.9722 | | | | | | | | | | 5.948969 | | | | 2756.867 | | | 151.7813 | |  |  |  |  |  |
| 27.325 | | | 322.0621 | | | | | | | | | | 5.94847 | | | | 2755.633 | | | 151.2422 | |  |  |  |  |  |
| 27.33333 | | | 322.152 | | | | | | | | | | 5.945963 | | | | 2754.43 | | | 150.7891 | |  |  |  |  |  |
| 27.34167 | | | 322.2389 | | | | | | | | | | 5.94287 | | | | 2753.219 | | | 150.3438 | |  |  |  |  |  |
| 27.35 | | | 322.3288 | | | | | | | | | | 5.94252 | | | | 2751.984 | | | 150 | |  |  |  |  |  |
| 27.35833 | | | 322.4179 | | | | | | | | | | 5.940595 | | | | 2750.781 | | | 149.5156 | |  |  |  |  |  |
| 27.36667 | | | 322.5071 | | | | | | | | | | 5.937407 | | | | 2749.57 | | | 149.125 | |  |  |  |  |  |
| 27.375 | | | 322.5963 | | | | | | | | | | 5.93733 | | | | 2748.367 | | | 148.9219 | |  |  |  |  |  |
| 27.38333 | | | 322.6862 | | | | | | | | | | 5.935237 | | | | 2747.172 | | | 148.3516 | |  |  |  |  |  |
| 27.39167 | | | 322.7746 | | | | | | | | | | 5.932288 | | | | 2745.961 | | | 148.1719 | |  |  |  |  |  |
| 27.4 | | | 322.8667 | | | | | | | | | | 5.932318 | | | | 2744.75 | | | 147.6172 | |  |  |  |  |  |
| 27.40833 | | | 322.9514 | | | | | | | | | | 5.93074 | | | | 2743.563 | | | 147.2734 | |  |  |  |  |  |
| 27.41667 | | | 323.0421 | | | | | | | | | | 5.928043 | | | | 2742.359 | | | 146.8594 | |  |  |  |  |  |
| 27.425 | | | 323.1305 | | | | | | | | | | 5.92879 | | | | 2741.133 | | | 146.2266 | |  |  |  |  |  |
| 27.43333 | | | 323.2204 | | | | | | | | | | 5.927792 | | | | 2739.961 | | | 145.6406 | |  |  |  |  |  |
| 27.44167 | | | 323.3088 | | | | | | | | | | 5.925108 | | | | 2738.742 | | | 145.2656 | |  |  |  |  |  |
| 27.45 | | | 323.3972 | | | | | | | | | | 5.925734 | | | | 2737.578 | | | 144.9453 | |  |  |  |  |  |
| 27.45833 | | | 323.4834 | | | | | | | | | | 5.92435 | | | | 2736.383 | | | 144.3203 | |  |  |  |  |  |
| 27.46667 | | | 323.571 | | | | | | | | | | 5.92133 | | | | 2735.211 | | | 143.9531 | |  |  |  |  |  |
| 27.475 | | | 323.6632 | | | | | | | | | | 5.92234 | | | | 2734.063 | | | 143.6094 | |  |  |  |  |  |
| 27.48333 | | | 323.7523 | | | | | | | | | | 5.921562 | | | | 2732.922 | | | 143.4766 | |  |  |  |  |  |
| 27.49167 | | | 323.8452 | | | | | | | | | | 5.918599 | | | | 2731.742 | | | 143.2188 | |  |  |  |  |  |
| 27.5 | | | 323.9329 | | | | | | | | | | 5.919743 | | | | 2730.578 | | | 142.9922 | |  |  |  |  |  |
| 27.50833 | | | 324.0243 | | | | | | | | | | 5.918614 | | | | 2729.445 | | | 142.6641 | |  |  |  |  |  |
| 27.51667 | | | 324.1127 | | | | | | | | | | 5.915488 | | | | 2728.281 | | | 142.2188 | |  |  |  |  |  |
| 27.525 | | | 324.2026 | | | | | | | | | | 5.916327 | | | | 2727.125 | | | 141.2344 | |  |  |  |  |  |
| 27.53333 | | | 324.294 | | | | | | | | | | 5.914613 | | | | 2725.93 | | | 139.8672 | |  |  |  |  |  |
| 27.54167 | | | 324.3847 | | | | | | | | | | 5.911523 | | | | 2724.773 | | | 138.125 | |  |  |  |  |  |
| 27.55 | | | 324.4753 | | | | | | | | | | 5.91224 | | | | 2723.586 | | | 136.4375 | |  |  |  |  |  |
| 27.55833 | | | 324.563 | | | | | | | | | | 5.910932 | | | | 2722.438 | | | 134.6328 | |  |  |  |  |  |
| 27.56667 | | | 324.6522 | | | | | | | | | | 5.90829 | | | | 2721.305 | | | 132.6484 | |  |  |  |  |  |
| 27.575 | | | 324.7405 | | | | | | | | | | 5.909778 | | | | 2720.25 | | | 130.9297 | |  |  |  |  |  |
| 27.58333 | | | 324.8327 | | | | | | | | | | 5.909063 | | | | 2719.273 | | | 128.8203 | |  |  |  |  |  |
| 27.59167 | | | 324.9219 | | | | | | | | | | 5.907616 | | | | 2718.344 | | | 127.3828 | |  |  |  |  |  |
| 27.6 | | | 325.0133 | | | | | | | | | | 5.910638 | | | | 2717.438 | | | 125.9844 | |  |  |  |  |  |
| 27.60833 | | | 325.1024 | | | | | | | | | | 5.911249 | | | | 2716.563 | | | 124.8125 | |  |  |  |  |  |
| 27.61667 | | | 325.1916 | | | | | | | | | | 5.911474 | | | | 2715.719 | | | 124.3516 | |  |  |  |  |  |
| 27.625 | | | 325.2815 | | | | | | | | | | 5.915404 | | | | 2714.844 | | | 124.3438 | |  |  |  |  |  |
| 27.63333 | | | 325.3722 | | | | | | | | | | 5.916827 | | | | 2714.055 | | | 124.2813 | |  |  |  |  |  |
| 27.64167 | | | 325.4598 | | | | | | | | | | 5.916873 | | | | 2713.172 | | | 123.9766 | |  |  |  |  |  |
| 27.65 | | | 325.546 | | | | | | | | | | 5.920914 | | | | 2712.313 | | | 124.1563 | |  |  |  |  |  |
| 27.65833 | | | 325.6321 | | | | | | | | | | 5.922631 | | | | 2711.398 | | | 124.25 | |  |  |  |  |  |
| 27.66667 | | | 325.7206 | | | | | | | | | | 5.921983 | | | | 2710.414 | | | 124.6406 | |  |  |  |  |  |
| 27.675 | | | 325.8097 | | | | | | | | | | 5.925926 | | | | 2709.352 | | | 124.7656 | |  |  |  |  |  |
| 27.68333 | | | 325.8959 | | | | | | | | | | 5.926357 | | | | 2708.305 | | | 125.1094 | |  |  |  |  |  |
| 27.69167 | | | 325.985 | | | | | | | | | | 5.924564 | | | | 2707.289 | | | 125.3672 | |  |  |  |  |  |
| 27.7 | | | 326.0742 | | | | | | | | | | 5.927616 | | | | 2706.211 | | | 125.5859 | |  |  |  |  |  |
| 27.70833 | | | 326.1641 | | | | | | | | | | 5.927457 | | | | 2705.141 | | | 125.6328 | |  |  |  |  |  |
| 27.71667 | | | 326.2532 | | | | | | | | | | 5.925707 | | | | 2704.016 | | | 125.6719 | |  |  |  |  |  |
| 27.725 | | | 326.3446 | | | | | | | | | | 5.929187 | | | | 2702.945 | | | 125.9766 | |  |  |  |  |  |
| 27.73333 | | | 326.4361 | | | | | | | | | | 5.929028 | | | | 2701.836 | | | 126.125 | |  |  |  |  |  |
| 27.74167 | | | 326.5275 | | | | | | | | | | 5.927197 | | | | 2700.734 | | | 126.1016 | |  |  |  |  |  |
| 27.75 | | | 326.6166 | | | | | | | | | | 5.929453 | | | | 2699.641 | | | 126.0391 | |  |  |  |  |  |
| 27.75833 | | | 326.7043 | | | | | | | | | | 5.928343 | | | | 2698.57 | | | 125.9609 | |  |  |  |  |  |
| 27.76667 | | | 326.7957 | | | | | | | | | | 5.926295 | | | | 2697.492 | | | 125.7813 | |  |  |  |  |  |
| 27.775 | | | 326.8864 | | | | | | | | | | 5.928444 | | | | 2696.375 | | | 125.9375 | |  |  |  |  |  |
| 27.78333 | | | 326.9755 | | | | | | | | | | 5.92706 | | | | 2695.289 | | | 125.8125 | |  |  |  |  |  |
| 27.79167 | | | 327.0654 | | | | | | | | | | 5.92504 | | | | 2694.219 | | | 125.6172 | |  |  |  |  |  |
| 27.8 | | | 327.1538 | | | | | | | | | | 5.927296 | | | | 2693.164 | | | 125.5781 | |  |  |  |  |  |
| 27.80833 | | | 327.2445 | | | | | | | | | | 5.926003 | | | | 2692.109 | | | 125.6172 | |  |  |  |  |  |
| 27.81667 | | | 327.3351 | | | | | | | | | | 5.923739 | | | | 2691.07 | | | 125.5313 | |  |  |  |  |  |
| 27.825 | | | 327.425 | | | | | | | | | | 5.926588 | | | | 2689.984 | | | 125.4531 | |  |  |  |  |  |
| 27.83333 | | | 327.5134 | | | | | | | | | | 5.925596 | | | | 2688.953 | | | 125.3359 | |  |  |  |  |  |
| 27.84167 | | | 327.6026 | | | | | | | | | | 5.923807 | | | | 2687.906 | | | 125.0703 | |  |  |  |  |  |
| 27.85 | | | 327.691 | | | | | | | | | | 5.926398 | | | | 2686.859 | | | 125.1719 | |  |  |  |  |  |
| 27.85833 | | | 327.7794 | | | | | | | | | | 5.925514 | | | | 2685.797 | | | 124.8516 | |  |  |  |  |  |
| 27.86667 | | | 327.8686 | | | | | | | | | | 5.923855 | | | | 2684.75 | | | 124.9297 | |  |  |  |  |  |
| 27.875 | | | 327.957 | | | | | | | | | | 5.926526 | | | | 2683.695 | | | 124.7422 | |  |  |  |  |  |
| 27.88333 | | | 328.0476 | | | | | | | | | | 5.925834 | | | | 2682.656 | | | 124.75 | |  |  |  |  |  |
| 27.89167 | | | 328.1368 | | | | | | | | | | 5.924179 | | | | 2681.625 | | | 124.6563 | |  |  |  |  |  |
| 27.9 | | | 328.226 | | | | | | | | | | 5.92705 | | | | 2680.563 | | | 124.6016 | |  |  |  |  |  |
| 27.90833 | | | 328.3151 | | | | | | | | | | 5.925002 | | | | 2679.547 | | | 124.5234 | |  |  |  |  |  |
| 27.91667 | | | 328.405 | | | | | | | | | | 5.923138 | | | | 2678.484 | | | 124.3984 | |  |  |  |  |  |
| 27.925 | | | 328.4979 | | | | | | | | | | 5.925324 | | | | 2677.461 | | | 124.6484 | |  |  |  |  |  |
| 27.93333 | | | 328.5893 | | | | | | | | | | 5.923206 | | | | 2676.438 | | | 124.7969 | |  |  |  |  |  |
| 27.94167 | | | 328.6792 | | | | | | | | | | 5.922088 | | | | 2675.422 | | | 124.8906 | |  |  |  |  |  |
| 27.95 | | | 328.7684 | | | | | | | | | | 5.92432 | | | | 2674.414 | | | 124.8516 | |  |  |  |  |  |
| 27.95833 | | | 328.8598 | | | | | | | | | | 5.92269 | | | | 2673.406 | | | 124.9688 | |  |  |  |  |  |
| 27.96667 | | | 328.9512 | | | | | | | | | | 5.921597 | | | | 2672.398 | | | 124.8125 | |  |  |  |  |  |
| 27.975 | | | 329.0404 | | | | | | | | | | 5.924364 | | | | 2671.344 | | | 124.7266 | |  |  |  |  |  |
| 27.98333 | | | 329.1288 | | | | | | | | | | 5.922698 | | | | 2670.32 | | | 124.7578 | |  |  |  |  |  |
| 27.99167 | | | 329.2202 | | | | | | | | | | 5.921701 | | | | 2669.281 | | | 124.5078 | |  |  |  |  |  |
| 28 | | | 329.3108 | | | | | | | | | | 5.924989 | | | | 2668.273 | | | 124.6094 | |  |  |  |  |  |
| 28.00833 | | | 329.3992 | | | | | | | | | | 5.923295 | | | | 2667.234 | | | 124.3906 | |  |  |  |  |  |
| 28.01667 | | | 329.4891 | | | | | | | | | | 5.922517 | | | | 2666.219 | | | 123.9297 | |  |  |  |  |  |
| 28.025 | | | 329.5783 | | | | | | | | | | 5.925751 | | | | 2665.188 | | | 123.5313 | |  |  |  |  |  |
| 28.03333 | | | 329.6682 | | | | | | | | | | 5.924322 | | | | 2664.148 | | | 123.3672 | |  |  |  |  |  |
| 28.04167 | | | 329.7566 | | | | | | | | | | 5.923249 | | | | 2663.141 | | | 122.9453 | |  |  |  |  |  |
| 28.05 | | | 329.8443 | | | | | | | | | | 5.925605 | | | | 2662.086 | | | 122.7734 | |  |  |  |  |  |
| 28.05833 | | | 329.9334 | | | | | | | | | | 5.923446 | | | | 2661.078 | | | 122.5547 | |  |  |  |  |  |
| 28.06667 | | | 330.0233 | | | | | | | | | | 5.922095 | | | | 2660.094 | | | 122.1484 | |  |  |  |  |  |
| 28.075 | | | 330.111 | | | | | | | | | | 5.924902 | | | | 2659.117 | | | 121.9063 | |  |  |  |  |  |
| 28.08333 | | | 330.1972 | | | | | | | | | | 5.923549 | | | | 2658.109 | | | 121.5938 | |  |  |  |  |  |
| 28.09167 | | | 330.2856 | | | | | | | | | | 5.922744 | | | | 2657.133 | | | 121.2109 | |  |  |  |  |  |
| 28.1 | | | 330.3755 | | | | | | | | | | 5.925033 | | | | 2656.117 | | | 120.9297 | |  |  |  |  |  |
| 28.10833 | | | 330.4654 | | | | | | | | | | 5.923872 | | | | 2655.141 | | | 120.7422 | |  |  |  |  |  |
| 28.11667 | | | 330.5538 | | | | | | | | | | 5.922727 | | | | 2654.164 | | | 120.5156 | |  |  |  |  |  |
| 28.125 | | | 330.6429 | | | | | | | | | | 5.924687 | | | | 2653.164 | | | 120.4141 | |  |  |  |  |  |
| 28.13333 | | | 330.7284 | | | | | | | | | | 5.923888 | | | | 2652.188 | | | 120.2891 | |  |  |  |  |  |
| 28.14167 | | | 330.8175 | | | | | | | | | | 5.92274 | | | | 2651.234 | | | 120.0781 | |  |  |  |  |  |
| 28.15 | | | 330.9074 | | | | | | | | | | 5.924594 | | | | 2650.25 | | | 119.7188 | |  |  |  |  |  |
| 28.15833 | | | 330.9988 | | | | | | | | | | 5.923186 | | | | 2649.266 | | | 119.5703 | |  |  |  |  |  |
| 28.16667 | | | 331.091 | | | | | | | | | | 5.922135 | | | | 2648.289 | | | 119.4063 | |  |  |  |  |  |
| 28.175 | | | 331.1816 | | | | | | | | | | 5.924165 | | | | 2647.289 | | | 119.2656 | |  |  |  |  |  |
| 28.18333 | | | 331.2708 | | | | | | | | | | 5.922994 | | | | 2646.305 | | | 119.2031 | |  |  |  |  |  |
| 28.19167 | | | 331.3592 | | | | | | | | | | 5.922203 | | | | 2645.336 | | | 119.0859 | |  |  |  |  |  |
| 28.2 | | | 331.4506 | | | | | | | | | | 5.924341 | | | | 2644.375 | | | 118.8125 | |  |  |  |  |  |
| 28.20833 | | | 331.5383 | | | | | | | | | | 5.923474 | | | | 2643.391 | | | 118.7188 | |  |  |  |  |  |
| 28.21667 | | | 331.6334 | | | | | | | | | | 5.922398 | | | | 2642.406 | | | 118.3828 | |  |  |  |  |  |
| 28.225 | | | 331.7218 | | | | | | | | | | 5.924512 | | | | 2641.438 | | | 118.3203 | |  |  |  |  |  |
| 28.23333 | | | 331.8117 | | | | | | | | | | 5.923398 | | | | 2640.445 | | | 118.3516 | |  |  |  |  |  |
| 28.24167 | | | 331.8987 | | | | | | | | | | 5.922742 | | | | 2639.469 | | | 118.1094 | |  |  |  |  |  |
| 28.25 | | | 331.9886 | | | | | | | | | | 5.924715 | | | | 2638.5 | | | 117.7188 | |  |  |  |  |  |
| 28.25833 | | | 332.0785 | | | | | | | | | | 5.923398 | | | | 2637.523 | | | 117.6328 | |  |  |  |  |  |
| 28.26667 | | | 332.1661 | | | | | | | | | | 5.922348 | | | | 2636.578 | | | 117.3516 | |  |  |  |  |  |
| 28.275 | | | 332.2545 | | | | | | | | | | 5.923837 | | | | 2635.594 | | | 117.0938 | |  |  |  |  |  |
| 28.28333 | | | 332.3422 | | | | | | | | | | 5.922589 | | | | 2634.602 | | | 116.9297 | |  |  |  |  |  |
| 28.29167 | | | 332.4329 | | | | | | | | | | 5.921219 | | | | 2633.648 | | | 116.6484 | |  |  |  |  |  |
| 28.3 | | | 332.522 | | | | | | | | | | 5.922589 | | | | 2632.703 | | | 116.6563 | |  |  |  |  |  |
| 28.30833 | | | 332.6127 | | | | | | | | | | 5.921986 | | | | 2631.742 | | | 116.2969 | |  |  |  |  |  |
| 28.31667 | | | 332.7041 | | | | | | | | | | 5.920836 | | | | 2630.797 | | | 115.9219 | |  |  |  |  |  |
| 28.325 | | | 332.7955 | | | | | | | | | | 5.922478 | | | | 2629.852 | | | 115.6719 | |  |  |  |  |  |
| 28.33333 | | | 332.8846 | | | | | | | | | | 5.921625 | | | | 2628.898 | | | 115.6484 | |  |  |  |  |  |
| 28.34167 | | | 332.9745 | | | | | | | | | | 5.920793 | | | | 2627.953 | | | 115.5781 | |  |  |  |  |  |
| 28.35 | | | 333.0645 | | | | | | | | | | 5.922714 | | | | 2626.969 | | | 115.5938 | |  |  |  |  |  |
| 28.35833 | | | 333.1566 | | | | | | | | | | 5.922349 | | | | 2626.055 | | | 115.4141 | |  |  |  |  |  |
| 28.36667 | | | 333.248 | | | | | | | | | | 5.921822 | | | | 2625.133 | | | 115.1797 | |  |  |  |  |  |
| 28.375 | | | 333.3372 | | | | | | | | | | 5.923607 | | | | 2624.195 | | | 114.8438 | |  |  |  |  |  |
| 28.38333 | | | 333.4263 | | | | | | | | | | 5.922912 | | | | 2623.227 | | | 114.4531 | |  |  |  |  |  |
| 28.39167 | | | 333.5155 | | | | | | | | | | 5.922463 | | | | 2622.266 | | | 114.2734 | |  |  |  |  |  |
| 28.4 | | | 333.6046 | | | | | | | | | | 5.924383 | | | | 2621.289 | | | 113.9297 | |  |  |  |  |  |
| 28.40833 | | | 333.6931 | | | | | | | | | | 5.924401 | | | | 2620.352 | | | 113.9219 | |  |  |  |  |  |
| 28.41667 | | | 333.7852 | | | | | | | | | | 5.923832 | | | | 2619.414 | | | 113.3594 | |  |  |  |  |  |
| 28.425 | | | 333.8759 | | | | | | | | | | 5.925875 | | | | 2618.508 | | | 113.1016 | |  |  |  |  |  |
| 28.43333 | | | 333.968 | | | | | | | | | | 5.925469 | | | | 2617.609 | | | 112.6641 | |  |  |  |  |  |
| 28.44167 | | | 334.0594 | | | | | | | | | | 5.924368 | | | | 2616.68 | | | 112.7109 | |  |  |  |  |  |
| 28.45 | | | 334.1478 | | | | | | | | | | 5.925583 | | | | 2615.766 | | | 112.6094 | |  |  |  |  |  |
| 28.45833 | | | 334.2408 | | | | | | | | | | 5.924611 | | | | 2614.813 | | | 112.6016 | |  |  |  |  |  |
| 28.46667 | | | 334.3299 | | | | | | | | | | 5.923447 | | | | 2613.945 | | | 112.3516 | |  |  |  |  |  |
| 28.475 | | | 334.4191 | | | | | | | | | | 5.924674 | | | | 2613.031 | | | 112.3281 | |  |  |  |  |  |
| 28.48333 | | | 334.5097 | | | | | | | | | | 5.923398 | | | | 2612.148 | | | 112.3359 | |  |  |  |  |  |
| 28.49167 | | | 334.6011 | | | | | | | | | | 5.922149 | | | | 2611.195 | | | 111.8438 | |  |  |  |  |  |
| 28.5 | | | 334.6873 | | | | | | | | | | 5.92318 | | | | 2610.273 | | | 111.8516 | |  |  |  |  |  |
| 28.50833 | | | 334.7742 | | | | | | | | | | 5.921914 | | | | 2609.336 | | | 111.5781 | |  |  |  |  |  |
| 28.51667 | | | 334.8641 | | | | | | | | | | 5.921047 | | | | 2608.422 | | | 111.3672 | |  |  |  |  |  |
| 28.525 | | | 334.9532 | | | | | | | | | | 5.922658 | | | | 2607.477 | | | 111.1172 | |  |  |  |  |  |
| 28.53333 | | | 335.0446 | | | | | | | | | | 5.921802 | | | | 2606.547 | | | 110.7813 | |  |  |  |  |  |
| 28.54167 | | | 335.1316 | | | | | | | | | | 5.921585 | | | | 2605.664 | | | 110.2969 | |  |  |  |  |  |
| 28.55 | | | 335.223 | | | | | | | | | | 5.923238 | | | | 2604.734 | | | 110.1484 | |  |  |  |  |  |
| 28.55833 | | | 335.3129 | | | | | | | | | | 5.92207 | | | | 2603.836 | | | 110.0547 | |  |  |  |  |  |
| 28.56667 | | | 335.402 | | | | | | | | | | 5.921652 | | | | 2602.938 | | | 109.7109 | |  |  |  |  |  |
| 28.575 | | | 335.4919 | | | | | | | | | | 5.923183 | | | | 2602.039 | | | 109.6016 | |  |  |  |  |  |
| 28.58333 | | | 335.5826 | | | | | | | | | | 5.921803 | | | | 2601.156 | | | 109.5859 | |  |  |  |  |  |
| 28.59167 | | | 335.6763 | | | | | | | | | | 5.920733 | | | | 2600.297 | | | 109.5469 | |  |  |  |  |  |
| 28.6 | | | 335.7624 | | | | | | | | | | 5.921538 | | | | 2599.398 | | | 109.625 | |  |  |  |  |  |
| 28.60833 | | | 335.8523 | | | | | | | | | | 5.920225 | | | | 2598.484 | | | 109.2031 | |  |  |  |  |  |
| 28.61667 | | | 335.9422 | | | | | | | | | | 5.918968 | | | | 2597.609 | | | 109.1797 | |  |  |  |  |  |
| 28.625 | | | 336.0321 | | | | | | | | | | 5.92029 | | | | 2596.703 | | | 109.1406 | |  |  |  |  |  |
| 28.63333 | | | 336.1205 | | | | | | | | | | 5.919607 | | | | 2595.781 | | | 109.0156 | |  |  |  |  |  |
| 28.64167 | | | 336.2127 | | | | | | | | | | 5.918955 | | | | 2594.875 | | | 108.9141 | |  |  |  |  |  |
| 28.65 | | | 336.3041 | | | | | | | | | | 5.920758 | | | | 2593.945 | | | 108.6563 | |  |  |  |  |  |
| 28.65833 | | | 336.3925 | | | | | | | | | | 5.920204 | | | | 2593.086 | | | 108.6641 | |  |  |  |  |  |
| 28.66667 | | | 336.4817 | | | | | | | | | | 5.919388 | | | | 2592.18 | | | 108.5391 | |  |  |  |  |  |
| 28.675 | | | 336.5708 | | | | | | | | | | 5.921521 | | | | 2591.266 | | | 108.2422 | |  |  |  |  |  |
| 28.68333 | | | 336.6622 | | | | | | | | | | 5.921324 | | | | 2590.367 | | | 108 | |  |  |  |  |  |
| 28.69167 | | | 336.7506 | | | | | | | | | | 5.920466 | | | | 2589.477 | | | 107.7969 | |  |  |  |  |  |
| 28.7 | | | 336.8413 | | | | | | | | | | 5.922529 | | | | 2588.602 | | | 107.5313 | |  |  |  |  |  |
| 28.70833 | | | 336.932 | | | | | | | | | | 5.922349 | | | | 2587.688 | | | 107.6641 | |  |  |  |  |  |
| 28.71667 | | | 337.0234 | | | | | | | | | | 5.921105 | | | | 2586.797 | | | 107.5156 | |  |  |  |  |  |
| 28.725 | | | 337.1133 | | | | | | | | | | 5.923218 | | | | 2585.93 | | | 107.3438 | |  |  |  |  |  |
| 28.73333 | | | 337.2024 | | | | | | | | | | 5.922893 | | | | 2585.063 | | | 107.4531 | |  |  |  |  |  |
| 28.74167 | | | 337.2931 | | | | | | | | | | 5.921638 | | | | 2584.18 | | | 106.9609 | |  |  |  |  |  |
| 28.75 | | | 337.386 | | | | | | | | | | 5.923792 | | | | 2583.313 | | | 106.7813 | |  |  |  |  |  |
| 28.75833 | | | 337.4744 | | | | | | | | | | 5.923236 | | | | 2582.391 | | | 106.6875 | |  |  |  |  |  |
| 28.76667 | | | 337.5651 | | | | | | | | | | 5.922202 | | | | 2581.516 | | | 106.3047 | |  |  |  |  |  |
| 28.775 | | | 337.6565 | | | | | | | | | | 5.924234 | | | | 2580.633 | | | 106.3281 | |  |  |  |  |  |
| 28.78333 | | | 337.7441 | | | | | | | | | | 5.923849 | | | | 2579.734 | | | 106.3203 | |  |  |  |  |  |
| 28.79167 | | | 337.8363 | | | | | | | | | | 5.923049 | | | | 2578.898 | | | 106.25 | |  |  |  |  |  |
| 28.8 | | | 337.9262 | | | | | | | | | | 5.924903 | | | | 2578.039 | | | 106.2109 | |  |  |  |  |  |
| 28.80833 | | | 338.0191 | | | | | | | | | | 5.923847 | | | | 2577.156 | | | 105.8203 | |  |  |  |  |  |
| 28.81667 | | | 338.1075 | | | | | | | | | | 5.923011 | | | | 2576.328 | | | 105.7891 | |  |  |  |  |  |
| 28.825 | | | 338.1967 | | | | | | | | | | 5.924876 | | | | 2575.43 | | | 105.5938 | |  |  |  |  |  |
| 28.83333 | | | 338.2851 | | | | | | | | | | 5.923702 | | | | 2574.547 | | | 105.4141 | |  |  |  |  |  |
| 28.84167 | | | 338.3765 | | | | | | | | | | 5.92273 | | | | 2573.656 | | | 105.2344 | |  |  |  |  |  |
| 28.85 | | | 338.4671 | | | | | | | | | | 5.924559 | | | | 2572.781 | | | 105.4141 | |  |  |  |  |  |
| 28.85833 | | | 338.5548 | | | | | | | | | | 5.923801 | | | | 2571.945 | | | 105.1875 | |  |  |  |  |  |
| 28.86667 | | | 338.6447 | | | | | | | | | | 5.922778 | | | | 2571.07 | | | 104.7109 | |  |  |  |  |  |
| 28.875 | | | 338.7331 | | | | | | | | | | 5.925155 | | | | 2570.203 | | | 104.5313 | |  |  |  |  |  |
| 28.88333 | | | 338.823 | | | | | | | | | | 5.924807 | | | | 2569.344 | | | 104.5313 | |  |  |  |  |  |
| 28.89167 | | | 338.9107 | | | | | | | | | | 5.923708 | | | | 2568.484 | | | 104.5938 | |  |  |  |  |  |
| 28.9 | | | 339.0028 | | | | | | | | | | 5.926429 | | | | 2567.578 | | | 104.5 | |  |  |  |  |  |
| 28.90833 | | | 339.0957 | | | | | | | | | | 5.925737 | | | | 2566.734 | | | 104.2656 | |  |  |  |  |  |
| 28.91667 | | | 339.1886 | | | | | | | | | | 5.924752 | | | | 2565.914 | | | 104.0313 | |  |  |  |  |  |
| 28.925 | | | 339.2785 | | | | | | | | | | 5.926801 | | | | 2565.078 | | | 104.1953 | |  |  |  |  |  |
| 28.93333 | | | 339.3699 | | | | | | | | | | 5.925936 | | | | 2564.203 | | | 104.0078 | |  |  |  |  |  |
| 28.94167 | | | 339.4591 | | | | | | | | | | 5.92521 | | | | 2563.32 | | | 103.6484 | |  |  |  |  |  |
| 28.95 | | | 339.5513 | | | | | | | | | | 5.927364 | | | | 2562.469 | | | 103.6484 | |  |  |  |  |  |
| 28.95833 | | | 339.6419 | | | | | | | | | | 5.926355 | | | | 2561.617 | | | 103.5625 | |  |  |  |  |  |
| 28.96667 | | | 339.7318 | | | | | | | | | | 5.925617 | | | | 2560.789 | | | 103.4219 | |  |  |  |  |  |
| 28.975 | | | 339.8195 | | | | | | | | | | 5.927597 | | | | 2559.891 | | | 103.0547 | |  |  |  |  |  |
| 28.98333 | | | 339.9071 | | | | | | | | | | 5.926377 | | | | 2559.047 | | | 102.8047 | |  |  |  |  |  |
| 28.99167 | | | 339.9955 | | | | | | | | | | 5.925563 | | | | 2558.227 | | | 102.5313 | |  |  |  |  |  |
| 29 | | | 340.084 | | | | | | | | | | 5.927757 | | | | 2557.352 | | | 102.6484 | |  |  |  |  |  |
| 29.00833 | | | 340.1754 | | | | | | | | | | 5.926813 | | | | 2556.5 | | | 102.3359 | |  |  |  |  |  |
| 29.01667 | | | 340.2653 | | | | | | | | | | 5.926073 | | | | 2555.664 | | | 102.1172 | |  |  |  |  |  |
| 29.025 | | | 340.356 | | | | | | | | | | 5.927899 | | | | 2554.836 | | | 101.9844 | |  |  |  |  |  |
| 29.03333 | | | 340.4441 | | | | | | | | | | 5.926624 | | | | 2554.016 | | | 102.1172 | |  |  |  |  |  |
| 29.04167 | | | 340.5322 | | | | | | | | | | 5.925904 | | | | 2553.195 | | | 101.9375 | |  |  |  |  |  |
| 29.05 | | | 340.621 | | | | | | | | | | 5.927384 | | | | 2552.32 | | | 101.7578 | |  |  |  |  |  |
| 29.05833 | | | 340.7097 | | | | | | | | | | 5.925535 | | | | 2551.508 | | | 101.5938 | |  |  |  |  |  |
| 29.06667 | | | 340.8008 | | | | | | | | | | 5.924669 | | | | 2550.688 | | | 101.6094 | |  |  |  |  |  |
| 29.075 | | | 340.8881 | | | | | | | | | | 5.925954 | | | | 2549.844 | | | 101.6016 | |  |  |  |  |  |
| 29.08333 | | | 340.9761 | | | | | | | | | | 5.924148 | | | | 2548.977 | | | 101.5078 | |  |  |  |  |  |
| 29.09167 | | | 341.0634 | | | | | | | | | | 5.923192 | | | | 2548.148 | | | 101.4063 | |  |  |  |  |  |
| 29.1 | | | 341.1523 | | | | | | | | | | 5.925529 | | | | 2547.32 | | | 101.3672 | |  |  |  |  |  |
| 29.10833 | | | 341.2425 | | | | | | | | | | 5.924184 | | | | 2546.484 | | | 101.5234 | |  |  |  |  |  |
| 29.11667 | | | 341.3328 | | | | | | | | | | 5.922869 | | | | 2545.641 | | | 101.1172 | |  |  |  |  |  |
| 29.125 | | | 341.4238 | | | | | | | | | | 5.924992 | | | | 2544.789 | | | 100.9375 | |  |  |  |  |  |
| 29.13333 | | | 341.5126 | | | | | | | | | | 5.923264 | | | | 2543.953 | | | 100.6563 | |  |  |  |  |  |
| 29.14167 | | | 341.6021 | | | | | | | | | | 5.922306 | | | | 2543.125 | | | 100.4531 | |  |  |  |  |  |
| 29.15 | | | 341.6917 | | | | | | | | | | 5.924641 | | | | 2542.273 | | | 100.3438 | |  |  |  |  |  |
| 29.15833 | | | 341.7834 | | | | | | | | | | 5.9232 | | | | 2541.406 | | | 100.2109 | |  |  |  |  |  |
| 29.16667 | | | 341.8722 | | | | | | | | | | 5.923126 | | | | 2540.625 | | | 99.91406 | |  |  |  |  |  |
| 29.175 | | | 341.9618 | | | | | | | | | | 5.925719 | | | | 2539.797 | | | 99.66406 | |  |  |  |  |  |
| 29.18333 | | | 342.0491 | | | | | | | | | | 5.924721 | | | | 2539 | | | 99.55469 | |  |  |  |  |  |
| 29.19167 | | | 342.1393 | | | | | | | | | | 5.924324 | | | | 2538.18 | | | 99.35156 | |  |  |  |  |  |
| 29.2 | | | 342.2289 | | | | | | | | | | 5.926652 | | | | 2537.352 | | | 99.13281 | |  |  |  |  |  |
| 29.20833 | | | 342.3155 | | | | | | | | | | 5.92486 | | | | 2536.539 | | | 98.96094 | |  |  |  |  |  |
| 29.21667 | | | 342.4035 | | | | | | | | | | 5.923964 | | | | 2535.742 | | | 98.72656 | |  |  |  |  |  |
| 29.225 | | | 342.493 | | | | | | | | | | 5.92649 | | | | 2534.938 | | | 98.63281 | |  |  |  |  |  |
| 29.23333 | | | 342.5796 | | | | | | | | | | 5.924583 | | | | 2534.125 | | | 98.53906 | |  |  |  |  |  |
| 29.24167 | | | 342.6699 | | | | | | | | | | 5.923721 | | | | 2533.32 | | | 98.25 | |  |  |  |  |  |
| 29.25 | | | 342.7609 | | | | | | | | | | 5.926531 | | | | 2532.516 | | | 98.09375 | |  |  |  |  |  |
| 29.25833 | | | 342.849 | | | | | | | | | | 5.924518 | | | | 2531.719 | | | 97.80469 | |  |  |  |  |  |
| 29.26667 | | | 342.937 | | | | | | | | | | 5.923051 | | | | 2530.922 | | | 97.58594 | |  |  |  |  |  |
| 29.275 | | | 343.0258 | | | | | | | | | | 5.925866 | | | | 2530.117 | | | 97.34375 | |  |  |  |  |  |
| 29.28333 | | | 343.1124 | | | | | | | | | | 5.923558 | | | | 2529.305 | | | 97.17188 | |  |  |  |  |  |
| 29.29167 | | | 343.2027 | | | | | | | | | | 5.922147 | | | | 2528.516 | | | 96.99219 | |  |  |  |  |  |
| 29.3 | | | 343.2922 | | | | | | | | | | 5.925055 | | | | 2527.719 | | | 96.96094 | |  |  |  |  |  |
| 29.30833 | | | 343.3817 | | | | | | | | | | 5.922634 | | | | 2526.93 | | | 97.02344 | |  |  |  |  |  |
| 29.31667 | | | 343.4713 | | | | | | | | | | 5.921889 | | | | 2526.148 | | | 96.85156 | |  |  |  |  |  |
| 29.325 | | | 343.5593 | | | | | | | | | | 5.924825 | | | | 2525.367 | | | 96.75781 | |  |  |  |  |  |
| 29.33333 | | | 343.6489 | | | | | | | | | | 5.922866 | | | | 2524.578 | | | 96.6875 | |  |  |  |  |  |
| 29.34167 | | | 343.7406 | | | | | | | | | | 5.921978 | | | | 2523.789 | | | 96.59375 | |  |  |  |  |  |
| 29.35 | | | 343.8331 | | | | | | | | | | 5.924522 | | | | 2522.977 | | | 96.21875 | |  |  |  |  |  |
| 29.35833 | | | 343.9227 | | | | | | | | | | 5.922014 | | | | 2522.156 | | | 96.11719 | |  |  |  |  |  |
| 29.36667 | | | 344.0129 | | | | | | | | | | 5.921099 | | | | 2521.375 | | | 96.09375 | |  |  |  |  |  |
| 29.375 | | | 344.1025 | | | | | | | | | | 5.923889 | | | | 2520.57 | | | 96.22656 | |  |  |  |  |  |
| 29.38333 | | | 344.1913 | | | | | | | | | | 5.921679 | | | | 2519.773 | | | 96.08594 | |  |  |  |  |  |
| 29.39167 | | | 344.2815 | | | | | | | | | | 5.921454 | | | | 2518.977 | | | 95.92188 | |  |  |  |  |  |
| 29.4 | | | 344.3733 | | | | | | | | | | 5.924222 | | | | 2518.219 | | | 95.72656 | |  |  |  |  |  |
| 29.40833 | | | 344.4628 | | | | | | | | | | 5.921689 | | | | 2517.438 | | | 95.76563 | |  |  |  |  |  |
| 29.41667 | | | 344.5501 | | | | | | | | | | 5.921006 | | | | 2516.641 | | | 95.85156 | |  |  |  |  |  |
| 29.425 | | | 344.6352 | | | | | | | | | | 5.923406 | | | | 2515.805 | | | 95.65625 | |  |  |  |  |  |
| 29.43333 | | | 344.7226 | | | | | | | | | | 5.920383 | | | | 2515.023 | | | 95.92188 | |  |  |  |  |  |
| 29.44167 | | | 344.8106 | | | | | | | | | | 5.919575 | | | | 2514.242 | | | 95.9375 | |  |  |  |  |  |
| 29.45 | | | 344.8994 | | | | | | | | | | 5.92201 | | | | 2513.469 | | | 96.03906 | |  |  |  |  |  |
| 29.45833 | | | 344.9867 | | | | | | | | | | 5.919061 | | | | 2512.664 | | | 95.53125 | |  |  |  |  |  |
| 29.46667 | | | 345.0748 | | | | | | | | | | 5.918753 | | | | 2511.844 | | | 95.40625 | |  |  |  |  |  |
| 29.475 | | | 345.1636 | | | | | | | | | | 5.920774 | | | | 2511.063 | | | 95.21094 | |  |  |  |  |  |
| 29.48333 | | | 345.2523 | | | | | | | | | | 5.918231 | | | | 2510.227 | | | 95.44531 | |  |  |  |  |  |
| 29.49167 | | | 345.3404 | | | | | | | | | | 5.917652 | | | | 2509.422 | | | 95.28906 | |  |  |  |  |  |
| 29.5 | | | 345.43 | | | | | | | | | | 5.919702 | | | | 2508.602 | | | 94.96094 | |  |  |  |  |  |
| 29.50833 | | | 345.5225 | | | | | | | | | | 5.916926 | | | | 2507.875 | | | 94.88281 | |  |  |  |  |  |
| 29.51667 | | | 345.6142 | | | | | | | | | | 5.916721 | | | | 2507.094 | | | 94.71094 | |  |  |  |  |  |
| 29.525 | | | 345.7045 | | | | | | | | | | 5.919468 | | | | 2506.32 | | | 94.64063 | |  |  |  |  |  |
| 29.53333 | | | 345.794 | | | | | | | | | | 5.916877 | | | | 2505.484 | | | 94.19531 | |  |  |  |  |  |
| 29.54167 | | | 345.885 | | | | | | | | | | 5.91743 | | | | 2504.719 | | | 94.40625 | |  |  |  |  |  |
| 29.55 | | | 345.9768 | | | | | | | | | | 5.920373 | | | | 2503.961 | | | 94.28906 | |  |  |  |  |  |
| 29.55833 | | | 346.0656 | | | | | | | | | | 5.918201 | | | | 2503.188 | | | 94.21094 | |  |  |  |  |  |
| 29.56667 | | | 346.1544 | | | | | | | | | | 5.918831 | | | | 2502.422 | | | 93.74219 | |  |  |  |  |  |
| 29.575 | | | 346.2432 | | | | | | | | | | 5.921417 | | | | 2501.641 | | | 93.625 | |  |  |  |  |  |
| 29.58333 | | | 346.332 | | | | | | | | | | 5.918882 | | | | 2500.906 | | | 93.47656 | |  |  |  |  |  |
| 29.59167 | | | 346.4193 | | | | | | | | | | 5.919375 | | | | 2500.094 | | | 93.48438 | |  |  |  |  |  |
| 29.6 | | | 346.5059 | | | | | | | | | | 5.921942 | | | | 2499.32 | | | 93.32813 | |  |  |  |  |  |
| 29.60833 | | | 346.5917 | | | | | | | | | | 5.919616 | | | | 2498.547 | | | 93.21875 | |  |  |  |  |  |
| 29.61667 | | | 346.6805 | | | | | | | | | | 5.920083 | | | | 2497.828 | | | 93.22656 | |  |  |  |  |  |
| 29.625 | | | 346.7685 | | | | | | | | | | 5.922071 | | | | 2497.063 | | | 93.17969 | |  |  |  |  |  |
| 29.63333 | | | 346.8559 | | | | | | | | | | 5.920045 | | | | 2496.305 | | | 93.23438 | |  |  |  |  |  |
| 29.64167 | | | 346.9432 | | | | | | | | | | 5.919806 | | | | 2495.516 | | | 93.16406 | |  |  |  |  |  |
| 29.65 | | | 347.0298 | | | | | | | | | | 5.921156 | | | | 2494.758 | | | 93.15625 | |  |  |  |  |  |
| 29.65833 | | | 347.1223 | | | | | | | | | | 5.918958 | | | | 2494 | | | 93.14063 | |  |  |  |  |  |
| 29.66667 | | | 347.2096 | | | | | | | | | | 5.91801 | | | | 2493.219 | | | 93.20313 | |  |  |  |  |  |
| 29.675 | | | 347.2976 | | | | | | | | | | 5.919232 | | | | 2492.453 | | | 93.1875 | |  |  |  |  |  |
| 29.68333 | | | 347.3864 | | | | | | | | | | 5.9179 | | | | 2491.664 | | | 93.20313 | |  |  |  |  |  |
| 29.69167 | | | 347.4767 | | | | | | | | | | 5.917112 | | | | 2490.898 | | | 93.0625 | |  |  |  |  |  |
| 29.7 | | | 347.5662 | | | | | | | | | | 5.918893 | | | | 2490.117 | | | 93.02344 | |  |  |  |  |  |
| 29.70833 | | | 347.6572 | | | | | | | | | | 5.918279 | | | | 2489.336 | | | 92.91406 | |  |  |  |  |  |
| 29.71667 | | | 347.7468 | | | | | | | | | | 5.917736 | | | | 2488.555 | | | 92.82813 | |  |  |  |  |  |
| 29.725 | | | 347.8385 | | | | | | | | | | 5.91951 | | | | 2487.773 | | | 92.63281 | |  |  |  |  |  |
| 29.73333 | | | 347.9325 | | | | | | | | | | 5.918486 | | | | 2486.984 | | | 92.64844 | |  |  |  |  |  |
| 29.74167 | | | 348.0191 | | | | | | | | | | 5.918029 | | | | 2486.227 | | | 92.65625 | |  |  |  |  |  |
| 29.75 | | | 348.1123 | | | | | | | | | | 5.920102 | | | | 2485.461 | | | 92.49219 | |  |  |  |  |  |
| 29.75833 | | | 348.2041 | | | | | | | | | | 5.919123 | | | | 2484.711 | | | 92.48438 | |  |  |  |  |  |
| 29.76667 | | | 348.2943 | | | | | | | | | | 5.919396 | | | | 2483.938 | | | 92.32031 | |  |  |  |  |  |
| 29.775 | | | 348.3853 | | | | | | | | | | 5.921212 | | | | 2483.188 | | | 92.39063 | |  |  |  |  |  |
| 29.78333 | | | 348.4756 | | | | | | | | | | 5.919082 | | | | 2482.414 | | | 92.24219 | |  |  |  |  |  |
| 29.79167 | | | 348.5648 | | | | | | | | | | 5.919442 | | | | 2481.641 | | | 92.35156 | |  |  |  |  |  |
| 29.8 | | | 348.6546 | | | | | | | | | | 5.920665 | | | | 2480.891 | | | 91.99219 | |  |  |  |  |  |
| 29.80833 | | | 348.743 | | | | | | | | | | 5.917741 | | | | 2480.117 | | | 91.92188 | |  |  |  |  |  |
| 29.81667 | | | 348.8286 | | | | | | | | | | 5.918165 | | | | 2479.367 | | | 91.67188 | |  |  |  |  |  |
| 29.825 | | | 348.9184 | | | | | | | | | | 5.919664 | | | | 2478.586 | | | 91.35938 | |  |  |  |  |  |
| 29.83333 | | | 349.0046 | | | | | | | | | | 5.917377 | | | | 2477.828 | | | 91.13281 | |  |  |  |  |  |
| 29.84167 | | | 349.0916 | | | | | | | | | | 5.91803 | | | | 2477.039 | | | 90.82031 | |  |  |  |  |  |
| 29.85 | | | 349.1829 | | | | | | | | | | 5.919454 | | | | 2476.32 | | | 90.71875 | |  |  |  |  |  |
| 29.85833 | | | 349.2713 | | | | | | | | | | 5.91688 | | | | 2475.563 | | | 90.53125 | |  |  |  |  |  |
| 29.86667 | | | 349.3576 | | | | | | | | | | 5.91768 | | | | 2474.836 | | | 90.36719 | |  |  |  |  |  |
| 29.875 | | | 349.4445 | | | | | | | | | | 5.918698 | | | | 2474.109 | | | 90.05469 | |  |  |  |  |  |
| 29.88333 | | | 349.5344 | | | | | | | | | | 5.916526 | | | | 2473.383 | | | 90.01563 | |  |  |  |  |  |
| 29.89167 | | | 349.6243 | | | | | | | | | | 5.91737 | | | | 2472.664 | | | 90.07813 | |  |  |  |  |  |
| 29.9 | | | 349.7119 | | | | | | | | | | 5.918717 | | | | 2471.93 | | | 90.04688 | |  |  |  |  |  |
| 29.90833 | | | 349.7989 | | | | | | | | | | 5.916528 | | | | 2471.188 | | | 89.9375 | |  |  |  |  |  |
| 29.91667 | | | 349.8902 | | | | | | | | | | 5.917472 | | | | 2470.461 | | | 90.17969 | |  |  |  |  |  |
| 29.925 | | | 349.9801 | | | | | | | | | | 5.91861 | | | | 2469.758 | | | 90.03125 | |  |  |  |  |  |
| 29.93333 | | | 350.0649 | | | | | | | | | | 5.91619 | | | | 2469.016 | | | 90.14844 | |  |  |  |  |  |
| 29.94167 | | | 350.1511 | | | | | | | | | | 5.916752 | | | | 2468.242 | | | 90.10156 | |  |  |  |  |  |
| 29.95 | | | 350.2395 | | | | | | | | | | 5.917707 | | | | 2467.5 | | | 90.23438 | |  |  |  |  |  |
| 29.95833 | | | 350.3294 | | | | | | | | | | 5.915526 | | | | 2466.75 | | | 90.23438 | |  |  |  |  |  |
| 29.96667 | | | 350.42 | | | | | | | | | | 5.915613 | | | | 2465.977 | | | 90.16406 | |  |  |  |  |  |
| 29.975 | | | 350.507 | | | | | | | | | | 5.9165 | | | | 2465.242 | | | 90.00781 | |  |  |  |  |  |
| 29.98333 | | | 350.5946 | | | | | | | | | | 5.914526 | | | | 2464.469 | | | 90.13281 | |  |  |  |  |  |
| 29.99167 | | | 350.6852 | | | | | | | | | | 5.914941 | | | | 2463.719 | | | 90.05469 | |  |  |  |  |  |
| 30 | | | 350.7715 | | | | | | | | | | 5.915971 | | | | 2462.945 | | | 90.0625 | |  |  |  |  |  |
| 30.00833 | | | 350.8591 | | | | | | | | | | 5.913882 | | | | 2462.203 | | | 89.86719 | |  |  |  |  |  |
| 30.01667 | | | 350.9505 | | | | | | | | | | 5.913903 | | | | 2461.453 | | | 89.64063 | |  |  |  |  |  |
| 30.025 | | | 351.0418 | | | | | | | | | | 5.914896 | | | | 2460.734 | | | 89.35156 | |  |  |  |  |  |
| 30.03333 | | | 351.131 | | | | | | | | | | 5.9129 | | | | 2459.953 | | | 89.00781 | |  |  |  |  |  |
| 30.04167 | | | 351.2186 | | | | | | | | | | 5.913419 | | | | 2459.211 | | | 89.10938 | |  |  |  |  |  |
| 30.05 | | | 351.3049 | | | | | | | | | | 5.914407 | | | | 2458.445 | | | 88.89844 | |  |  |  |  |  |
| 30.05833 | | | 351.3925 | | | | | | | | | | 5.912506 | | | | 2457.727 | | | 88.77344 | |  |  |  |  |  |
| 30.06667 | | | 351.4846 | | | | | | | | | | 5.912433 | | | | 2457 | | | 88.42969 | |  |  |  |  |  |
| 30.075 | | | 351.5716 | | | | | | | | | | 5.913406 | | | | 2456.305 | | | 88.39063 | |  |  |  |  |  |
| 30.08333 | | | 351.6614 | | | | | | | | | | 5.911484 | | | | 2455.594 | | | 88.0625 | |  |  |  |  |  |
| 30.09167 | | | 351.7484 | | | | | | | | | | 5.911735 | | | | 2454.852 | | | 87.99219 | |  |  |  |  |  |
| 30.1 | | | 351.8339 | | | | | | | | | | 5.913104 | | | | 2454.125 | | | 87.71875 | |  |  |  |  |  |
| 30.10833 | | | 351.9238 | | | | | | | | | | 5.911467 | | | | 2453.414 | | | 87.84375 | |  |  |  |  |  |
| 30.11667 | | | 352.0122 | | | | | | | | | | 5.911538 | | | | 2452.711 | | | 87.92188 | |  |  |  |  |  |
| 30.125 | | | 352.1021 | | | | | | | | | | 5.912664 | | | | 2451.977 | | | 87.85156 | |  |  |  |  |  |
| 30.13333 | | | 352.1905 | | | | | | | | | | 5.911024 | | | | 2451.289 | | | 87.59375 | |  |  |  |  |  |
| 30.14167 | | | 352.2803 | | | | | | | | | | 5.911633 | | | | 2450.563 | | | 87.57813 | |  |  |  |  |  |
| 30.15 | | | 352.3665 | | | | | | | | | | 5.912977 | | | | 2449.859 | | | 87.34375 | |  |  |  |  |  |
| 30.15833 | | | 352.4528 | | | | | | | | | | 5.911541 | | | | 2449.109 | | | 87.17188 | |  |  |  |  |  |
| 30.16667 | | | 352.5397 | | | | | | | | | | 5.911598 | | | | 2448.359 | | | 87.22656 | |  |  |  |  |  |
| 30.175 | | | 352.6318 | | | | | | | | | | 5.913172 | | | | 2447.633 | | | 87.09375 | |  |  |  |  |  |
| 30.18333 | | | 352.7231 | | | | | | | | | | 5.911538 | | | | 2446.938 | | | 87.11719 | |  |  |  |  |  |
| 30.19167 | | | 352.8108 | | | | | | | | | | 5.911983 | | | | 2446.211 | | | 87.03906 | |  |  |  |  |  |
| 30.2 | | | 352.9014 | | | | | | | | | | 5.913146 | | | | 2445.508 | | | 86.82031 | |  |  |  |  |  |
| 30.20833 | | | 352.9876 | | | | | | | | | | 5.911551 | | | | 2444.797 | | | 86.65625 | |  |  |  |  |  |
| 30.21667 | | | 353.0746 | | | | | | | | | | 5.911567 | | | | 2444.07 | | | 86.57813 | |  |  |  |  |  |
| 30.225 | | | 353.1616 | | | | | | | | | | 5.913303 | | | | 2443.359 | | | 86.75 | |  |  |  |  |  |
| 30.23333 | | | 353.25 | | | | | | | | | | 5.912289 | | | | 2442.633 | | | 86.67969 | |  |  |  |  |  |
| 30.24167 | | | 353.3398 | | | | | | | | | | 5.912033 | | | | 2441.914 | | | 86.53125 | |  |  |  |  |  |
| 30.25 | | | 353.4305 | | | | | | | | | | 5.914401 | | | | 2441.227 | | | 86.28906 | |  |  |  |  |  |
| 30.25833 | | | 353.5181 | | | | | | | | | | 5.912588 | | | | 2440.531 | | | 86.34375 | |  |  |  |  |  |
| 30.26667 | | | 353.6058 | | | | | | | | | | 5.911184 | | | | 2439.813 | | | 86.24219 | |  |  |  |  |  |
| 30.275 | | | 353.6927 | | | | | | | | | | 5.913273 | | | | 2439.07 | | | 86.00781 | |  |  |  |  |  |
| 30.28333 | | | 353.7805 | | | | | | | | | | 5.911499 | | | | 2438.352 | | | 86.00781 | |  |  |  |  |  |
| 30.29167 | | | 353.871 | | | | | | | | | | 5.91162 | | | | 2437.648 | | | 86.04688 | |  |  |  |  |  |
| 30.3 | | | 353.9595 | | | | | | | | | | 5.91423 | | | | 2436.953 | | | 85.91406 | |  |  |  |  |  |
| 30.30833 | | | 354.0486 | | | | | | | | | | 5.912517 | | | | 2436.227 | | | 85.85938 | |  |  |  |  |  |
| 30.31667 | | | 354.137 | | | | | | | | | | 5.912256 | | | | 2435.516 | | | 85.72656 | |  |  |  |  |  |
| 30.325 | | | 354.2262 | | | | | | | | | | 5.913901 | | | | 2434.828 | | | 85.875 | |  |  |  |  |  |
| 30.33333 | | | 354.3138 | | | | | | | | | | 5.912306 | | | | 2434.109 | | | 85.67188 | |  |  |  |  |  |
| 30.34167 | | | 354.4015 | | | | | | | | | | 5.911749 | | | | 2433.383 | | | 85.95313 | |  |  |  |  |  |
| 30.35 | | | 354.4885 | | | | | | | | | | 5.914238 | | | | 2432.68 | | | 85.64063 | |  |  |  |  |  |
| 30.35833 | | | 354.5762 | | | | | | | | | | 5.912029 | | | | 2431.969 | | | 85.72656 | |  |  |  |  |  |
| 30.36667 | | | 354.6631 | | | | | | | | | | 5.910856 | | | | 2431.266 | | | 85.60938 | |  |  |  |  |  |
| 30.375 | | | 354.7508 | | | | | | | | | | 5.913598 | | | | 2430.531 | | | 85.5 | |  |  |  |  |  |
| 30.38333 | | | 354.8392 | | | | | | | | | | 5.91116 | | | | 2429.836 | | | 85.48438 | |  |  |  |  |  |
| 30.39167 | | | 354.9276 | | | | | | | | | | 5.910097 | | | | 2429.078 | | | 85.40625 | |  |  |  |  |  |
| 30.4 | | | 355.0168 | | | | | | | | | | 5.912385 | | | | 2428.398 | | | 85.24219 | |  |  |  |  |  |
| 30.40833 | | | 355.1052 | | | | | | | | | | 5.910135 | | | | 2427.672 | | | 85.125 | |  |  |  |  |  |
| 30.41667 | | | 355.1921 | | | | | | | | | | 5.909595 | | | | 2426.977 | | | 85.16406 | |  |  |  |  |  |
| 30.425 | | | 355.2805 | | | | | | | | | | 5.9126 | | | | 2426.273 | | | 84.71094 | |  |  |  |  |  |
| 30.43333 | | | 355.3712 | | | | | | | | | | 5.909676 | | | | 2425.57 | | | 84.625 | |  |  |  |  |  |
| 30.44167 | | | 355.4603 | | | | | | | | | | 5.909587 | | | | 2424.867 | | | 84.46094 | |  |  |  |  |  |
| 30.45 | | | 355.5494 | | | | | | | | | | 5.911801 | | | | 2424.18 | | | 84.35938 | |  |  |  |  |  |
| 30.45833 | | | 355.6378 | | | | | | | | | | 5.910069 | | | | 2423.477 | | | 84.19531 | |  |  |  |  |  |
| 30.46667 | | | 355.727 | | | | | | | | | | 5.909847 | | | | 2422.766 | | | 84.17969 | |  |  |  |  |  |
| 30.475 | | | 355.8162 | | | | | | | | | | 5.911629 | | | | 2422.117 | | | 84.05469 | |  |  |  |  |  |
| 30.48333 | | | 355.906 | | | | | | | | | | 5.909693 | | | | 2421.43 | | | 84.00781 | |  |  |  |  |  |
| 30.49167 | | | 355.9959 | | | | | | | | | | 5.909185 | | | | 2420.742 | | | 83.86719 | |  |  |  |  |  |
| 30.5 | | | 356.0865 | | | | | | | | | | 5.911166 | | | | 2420.063 | | | 83.72656 | |  |  |  |  |  |
| 30.50833 | | | 356.1764 | | | | | | | | | | 5.908909 | | | | 2419.375 | | | 83.80469 | |  |  |  |  |  |
| 30.51667 | | | 356.2655 | | | | | | | | | | 5.908549 | | | | 2418.68 | | | 83.89063 | |  |  |  |  |  |
| 30.525 | | | 356.3554 | | | | | | | | | | 5.911143 | | | | 2417.977 | | | 83.9375 | |  |  |  |  |  |
| 30.53333 | | | 356.4455 | | | | | | | | | | 5.908297 | | | | 2417.289 | | | 83.96094 | |  |  |  |  |  |
| 30.54167 | | | 356.5341 | | | | | | | | | | 5.907837 | | | | 2416.609 | | | 83.80469 | |  |  |  |  |  |
| 30.55 | | | 356.6227 | | | | | | | | | | 5.910169 | | | | 2415.93 | | | 83.89063 | |  |  |  |  |  |
| 30.55833 | | | 356.7128 | | | | | | | | | | 5.907461 | | | | 2415.227 | | | 83.8125 | |  |  |  |  |  |
| 30.56667 | | | 356.7999 | | | | | | | | | | 5.906463 | | | | 2414.508 | | | 83.92969 | |  |  |  |  |  |
| 30.575 | | | 356.8878 | | | | | | | | | | 5.909098 | | | | 2413.797 | | | 83.69531 | |  |  |  |  |  |
| 30.58333 | | | 356.9764 | | | | | | | | | | 5.906521 | | | | 2413.094 | | | 83.64844 | |  |  |  |  |  |
| 30.59167 | | | 357.065 | | | | | | | | | | 5.906357 | | | | 2412.406 | | | 83.60938 | |  |  |  |  |  |
| 30.6 | | | 357.1536 | | | | | | | | | | 5.908862 | | | | 2411.703 | | | 83.60156 | |  |  |  |  |  |
| 30.60833 | | | 357.243 | | | | | | | | | | 5.906065 | | | | 2411.016 | | | 83.5625 | |  |  |  |  |  |
| 30.61667 | | | 357.333 | | | | | | | | | | 5.905893 | | | | 2410.305 | | | 83.625 | |  |  |  |  |  |
| 30.625 | | | 357.4253 | | | | | | | | | | 5.908563 | | | | 2409.625 | | | 83.73438 | |  |  |  |  |  |
| 30.63333 | | | 357.5183 | | | | | | | | | | 5.906841 | | | | 2408.93 | | | 83.59375 | |  |  |  |  |  |
| 30.64167 | | | 357.6077 | | | | | | | | | | 5.906661 | | | | 2408.242 | | | 83.39844 | |  |  |  |  |  |
| 30.65 | | | 357.7 | | | | | | | | | | 5.909726 | | | | 2407.547 | | | 83.4375 | |  |  |  |  |  |
| 30.65833 | | | 357.7908 | | | | | | | | | | 5.906747 | | | | 2406.844 | | | 83.4375 | |  |  |  |  |  |
| 30.66667 | | | 357.8824 | | | | | | | | | | 5.907293 | | | | 2406.141 | | | 83.52344 | |  |  |  |  |  |
| 30.675 | | | 357.9717 | | | | | | | | | | 5.909958 | | | | 2405.43 | | | 83.48438 | |  |  |  |  |  |
| 30.68333 | | | 358.061 | | | | | | | | | | 5.907533 | | | | 2404.75 | | | 83.30469 | |  |  |  |  |  |
| 30.69167 | | | 358.1496 | | | | | | | | | | 5.908297 | | | | 2404.078 | | | 82.97656 | |  |  |  |  |  |
| 30.7 | | | 358.2397 | | | | | | | | | | 5.910153 | | | | 2403.375 | | | 83.01563 | |  |  |  |  |  |
| 30.70833 | | | 358.3268 | | | | | | | | | | 5.907436 | | | | 2402.688 | | | 83.10156 | |  |  |  |  |  |
| 30.71667 | | | 358.4132 | | | | | | | | | | 5.907594 | | | | 2401.977 | | | 82.89844 | |  |  |  |  |  |
| 30.725 | | | 358.5033 | | | | | | | | | | 5.910037 | | | | 2401.273 | | | 83 | |  |  |  |  |  |
| 30.73333 | | | 358.5941 | | | | | | | | | | 5.907388 | | | | 2400.602 | | | 82.75781 | |  |  |  |  |  |
| 30.74167 | | | 358.6827 | | | | | | | | | | 5.907603 | | | | 2399.953 | | | 82.45313 | |  |  |  |  |  |
| 30.75 | | | 358.7699 | | | | | | | | | | 5.910458 | | | | 2399.258 | | | 82.33594 | |  |  |  |  |  |
| 30.75833 | | | 358.8599 | | | | | | | | | | 5.907472 | | | | 2398.555 | | | 82.24219 | |  |  |  |  |  |
| 30.76667 | | | 358.95 | | | | | | | | | | 5.908835 | | | | 2397.883 | | | 82 | |  |  |  |  |  |
| 30.775 | | | 359.0423 | | | | | | | | | | 5.911476 | | | | 2397.18 | | | 82.03906 | |  |  |  |  |  |
| 30.78333 | | | 359.1339 | | | | | | | | | | 5.90903 | | | | 2396.523 | | | 81.90625 | |  |  |  |  |  |
| 30.79167 | | | 359.2247 | | | | | | | | | | 5.910038 | | | | 2395.875 | | | 81.86719 | |  |  |  |  |  |
| 30.8 | | | 359.3155 | | | | | | | | | | 5.912538 | | | | 2395.203 | | | 81.83594 | |  |  |  |  |  |
| 30.80833 | | | 359.4056 | | | | | | | | | | 5.909822 | | | | 2394.531 | | | 81.60938 | |  |  |  |  |  |
| 30.81667 | | | 359.4949 | | | | | | | | | | 5.910172 | | | | 2393.883 | | | 81.5 | |  |  |  |  |  |
| 30.825 | | | 359.5857 | | | | | | | | | | 5.912097 | | | | 2393.195 | | | 81.71094 | |  |  |  |  |  |
| 30.83333 | | | 359.6758 | | | | | | | | | | 5.909468 | | | | 2392.531 | | | 81.61719 | |  |  |  |  |  |
| 30.84167 | | | 359.7689 | | | | | | | | | | 5.911221 | | | | 2391.852 | | | 81.38281 | |  |  |  |  |  |
| 30.85 | | | 359.8582 | | | | | | | | | | 5.91219 | | | | 2391.172 | | | 81.58594 | |  |  |  |  |  |
| 30.85833 | | | 359.949 | | | | | | | | | | 5.909284 | | | | 2390.523 | | | 81.49219 | |  |  |  |  |  |
| 30.86667 | | | 360.0354 | | | | | | | | | | 5.910522 | | | | 2389.859 | | | 81.27344 | |  |  |  |  |  |
| 30.875 | | | 360.124 | | | | | | | | | | 5.911562 | | | | 2389.141 | | | 80.90625 | |  |  |  |  |  |
| 30.88333 | | | 360.2148 | | | | | | | | | | 5.908584 | | | | 2388.477 | | | 80.875 | |  |  |  |  |  |
| 30.89167 | | | 360.3041 | | | | | | | | | | 5.909479 | | | | 2387.828 | | | 80.82813 | |  |  |  |  |  |
| 30.9 | | | 360.3905 | | | | | | | | | | 5.911896 | | | | 2387.133 | | | 80.84375 | |  |  |  |  |  |
| 30.90833 | | | 360.4806 | | | | | | | | | | 5.909155 | | | | 2386.469 | | | 80.71094 | |  |  |  |  |  |
| 30.91667 | | | 360.5722 | | | | | | | | | | 5.910148 | | | | 2385.813 | | | 80.44531 | |  |  |  |  |  |
| 30.925 | | | 360.66 | | | | | | | | | | 5.912175 | | | | 2385.18 | | | 80.60156 | |  |  |  |  |  |
| 30.93333 | | | 360.7516 | | | | | | | | | | 5.909918 | | | | 2384.508 | | | 80.375 | |  |  |  |  |  |
| 30.94167 | | | 360.8402 | | | | | | | | | | 5.911228 | | | | 2383.836 | | | 80.51563 | |  |  |  |  |  |
| 30.95 | | | 360.9303 | | | | | | | | | | 5.912747 | | | | 2383.156 | | | 80.52344 | |  |  |  |  |  |
| 30.95833 | | | 361.0211 | | | | | | | | | | 5.91096 | | | | 2382.508 | | | 80.625 | |  |  |  |  |  |
| 30.96667 | | | 361.1134 | | | | | | | | | | 5.91167 | | | | 2381.867 | | | 80.57813 | |  |  |  |  |  |
| 30.975 | | | 361.2042 | | | | | | | | | | 5.913133 | | | | 2381.172 | | | 80.54688 | |  |  |  |  |  |
| 30.98333 | | | 361.2957 | | | | | | | | | | 5.911367 | | | | 2380.531 | | | 80.5 | |  |  |  |  |  |
| 30.99167 | | | 361.3881 | | | | | | | | | | 5.91287 | | | | 2379.836 | | | 80.49219 | |  |  |  |  |  |
| 31 | | | 361.4789 | | | | | | | | | | 5.914941 | | | | 2379.164 | | | 80.625 | |  |  |  |  |  |
| 31.00833 | | | 361.5689 | | | | | | | | | | 5.91234 | | | | 2378.477 | | | 80.54688 | |  |  |  |  |  |
| 31.01667 | | | 361.6576 | | | | | | | | | | 5.913946 | | | | 2377.797 | | | 80.60938 | |  |  |  |  |  |
| 31.025 | | | 361.7484 | | | | | | | | | | 5.915408 | | | | 2377.125 | | | 80.26563 | |  |  |  |  |  |
| 31.03333 | | | 361.8384 | | | | | | | | | | 5.912326 | | | | 2376.461 | | | 80.25781 | |  |  |  |  |  |
| 31.04167 | | | 361.9285 | | | | | | | | | | 5.914005 | | | | 2375.797 | | | 80.29688 | |  |  |  |  |  |
| 31.05 | | | 362.0157 | | | | | | | | | | 5.915142 | | | | 2375.094 | | | 80.30469 | |  |  |  |  |  |
| 31.05833 | | | 362.105 | | | | | | | | | | 5.912304 | | | | 2374.438 | | | 80.33594 | |  |  |  |  |  |
| 31.06667 | | | 362.1951 | | | | | | | | | | 5.914068 | | | | 2373.742 | | | 80.34375 | |  |  |  |  |  |
| 31.075 | | | 362.2829 | | | | | | | | | | 5.915586 | | | | 2373.125 | | | 80.17188 | |  |  |  |  |  |
| 31.08333 | | | 362.3716 | | | | | | | | | | 5.913569 | | | | 2372.453 | | | 80.0625 | |  |  |  |  |  |
| 31.09167 | | | 362.4609 | | | | | | | | | | 5.914287 | | | | 2371.789 | | | 80.20313 | |  |  |  |  |  |
| 31.1 | | | 362.5532 | | | | | | | | | | 5.915245 | | | | 2371.109 | | | 80.32031 | |  |  |  |  |  |
| 31.10833 | | | 362.6432 | | | | | | | | | | 5.913341 | | | | 2370.445 | | | 80.46094 | |  |  |  |  |  |
| 31.11667 | | | 362.7333 | | | | | | | | | | 5.914201 | | | | 2369.766 | | | 80.48438 | |  |  |  |  |  |
| 31.125 | | | 362.8205 | | | | | | | | | | 5.915169 | | | | 2369.117 | | | 80.45313 | |  |  |  |  |  |
| 31.13333 | | | 362.9113 | | | | | | | | | | 5.913958 | | | | 2368.461 | | | 80.35156 | |  |  |  |  |  |
| 31.14167 | | | 363.0036 | | | | | | | | | | 5.915471 | | | | 2367.789 | | | 80.21875 | |  |  |  |  |  |
| 31.15 | | | 363.0944 | | | | | | | | | | 5.916531 | | | | 2367.094 | | | 80.20313 | |  |  |  |  |  |
| 31.15833 | | | 363.1852 | | | | | | | | | | 5.914531 | | | | 2366.398 | | | 80.14063 | |  |  |  |  |  |
| 31.16667 | | | 363.2753 | | | | | | | | | | 5.915637 | | | | 2365.727 | | | 80.11719 | |  |  |  |  |  |
| 31.175 | | | 363.3661 | | | | | | | | | | 5.916346 | | | | 2365.055 | | | 80.22656 | |  |  |  |  |  |
| 31.18333 | | | 363.4562 | | | | | | | | | | 5.913215 | | | | 2364.398 | | | 80.01563 | |  |  |  |  |  |
| 31.19167 | | | 363.5455 | | | | | | | | | | 5.914407 | | | | 2363.758 | | | 79.90625 | |  |  |  |  |  |
| 31.2 | | | 363.6356 | | | | | | | | | | 5.915143 | | | | 2363.086 | | | 79.625 | |  |  |  |  |  |
| 31.20833 | | | 363.7301 | | | | | | | | | | 5.912221 | | | | 2362.43 | | | 79.42969 | |  |  |  |  |  |
| 31.21667 | | | 363.8224 | | | | | | | | | | 5.914112 | | | | 2361.766 | | | 79.27344 | |  |  |  |  |  |
| 31.225 | | | 363.9117 | | | | | | | | | | 5.915031 | | | | 2361.078 | | | 79.49219 | |  |  |  |  |  |
| 31.23333 | | | 364.0033 | | | | | | | | | | 5.912536 | | | | 2360.445 | | | 79.27344 | |  |  |  |  |  |
| 31.24167 | | | 364.0927 | | | | | | | | | | 5.914164 | | | | 2359.789 | | | 79.19531 | |  |  |  |  |  |
| 31.25 | | | 364.1842 | | | | | | | | | | 5.913912 | | | | 2359.164 | | | 79.02344 | |  |  |  |  |  |
| 31.25833 | | | 364.2713 | | | | | | | | | | 5.911979 | | | | 2358.531 | | | 78.83594 | |  |  |  |  |  |
| 31.26667 | | | 364.3592 | | | | | | | | | | 5.913603 | | | | 2357.891 | | | 78.76563 | |  |  |  |  |  |
| 31.275 | | | 364.4485 | | | | | | | | | | 5.91379 | | | | 2357.195 | | | 78.69531 | |  |  |  |  |  |
| 31.28333 | | | 364.5395 | | | | | | | | | | 5.912321 | | | | 2356.578 | | | 78.79688 | |  |  |  |  |  |
| 31.29167 | | | 364.6284 | | | | | | | | | | 5.913821 | | | | 2355.922 | | | 78.78906 | |  |  |  |  |  |
| 31.3 | | | 364.7145 | | | | | | | | | | 5.914155 | | | | 2355.289 | | | 78.71875 | |  |  |  |  |  |
| 31.30833 | | | 364.8006 | | | | | | | | | | 5.91199 | | | | 2354.656 | | | 78.47656 | |  |  |  |  |  |
| 31.31667 | | | 364.8874 | | | | | | | | | | 5.913654 | | | | 2354.016 | | | 78.50781 | |  |  |  |  |  |
| 31.325 | | | 364.9771 | | | | | | | | | | 5.914069 | | | | 2353.359 | | | 78.60938 | |  |  |  |  |  |
| 31.33333 | | | 365.0645 | | | | | | | | | | 5.912277 | | | | 2352.695 | | | 78.72656 | |  |  |  |  |  |
| 31.34167 | | | 365.1557 | | | | | | | | | | 5.913502 | | | | 2352.023 | | | 78.61719 | |  |  |  |  |  |
| 31.35 | | | 365.2439 | | | | | | | | | | 5.91362 | | | | 2351.391 | | | 78.66406 | |  |  |  |  |  |
| 31.35833 | | | 365.33 | | | | | | | | | | 5.911938 | | | | 2350.758 | | | 78.57813 | |  |  |  |  |  |
| 31.36667 | | | 365.4168 | | | | | | | | | | 5.913315 | | | | 2350.094 | | | 78.625 | |  |  |  |  |  |
| 31.375 | | | 365.5021 | | | | | | | | | | 5.914508 | | | | 2349.422 | | | 78.38281 | |  |  |  |  |  |
| 31.38333 | | | 365.5911 | | | | | | | | | | 5.912197 | | | | 2348.75 | | | 78.49219 | |  |  |  |  |  |
| 31.39167 | | | 365.68 | | | | | | | | | | 5.913469 | | | | 2348.117 | | | 78.57813 | |  |  |  |  |  |
| 31.4 | | | 365.769 | | | | | | | | | | 5.913612 | | | | 2347.445 | | | 78.60938 | |  |  |  |  |  |
| 31.40833 | | | 365.8572 | | | | | | | | | | 5.911511 | | | | 2346.797 | | | 78.67969 | |  |  |  |  |  |
| 31.41667 | | | 365.944 | | | | | | | | | | 5.912585 | | | | 2346.141 | | | 78.73438 | |  |  |  |  |  |
| 31.425 | | | 366.0315 | | | | | | | | | | 5.91319 | | | | 2345.516 | | | 78.95313 | |  |  |  |  |  |
| 31.43333 | | | 366.1212 | | | | | | | | | | 5.910852 | | | | 2344.844 | | | 78.90625 | |  |  |  |  |  |
| 31.44167 | | | 366.2116 | | | | | | | | | | 5.912537 | | | | 2344.188 | | | 78.67188 | |  |  |  |  |  |
| 31.45 | | | 366.3027 | | | | | | | | | | 5.913249 | | | | 2343.508 | | | 78.58594 | |  |  |  |  |  |
| 31.45833 | | | 366.3924 | | | | | | | | | | 5.91203 | | | | 2342.852 | | | 78.49219 | |  |  |  |  |  |
| 31.46667 | | | 366.4785 | | | | | | | | | | 5.913502 | | | | 2342.188 | | | 78.52344 | |  |  |  |  |  |
| 31.475 | | | 366.5689 | | | | | | | | | | 5.914008 | | | | 2341.492 | | | 78.5625 | |  |  |  |  |  |
| 31.48333 | | | 366.6586 | | | | | | | | | | 5.912169 | | | | 2340.852 | | | 78.375 | |  |  |  |  |  |
| 31.49167 | | | 366.7468 | | | | | | | | | | 5.914466 | | | | 2340.219 | | | 78.14844 | |  |  |  |  |  |
| 31.5 | | | 366.8358 | | | | | | | | | | 5.915776 | | | | 2339.578 | | | 77.98438 | |  |  |  |  |  |
| 31.50833 | | | 366.9269 | | | | | | | | | | 5.914264 | | | | 2338.922 | | | 77.85156 | |  |  |  |  |  |
| 31.51667 | | | 367.0144 | | | | | | | | | | 5.91676 | | | | 2338.258 | | | 77.88281 | |  |  |  |  |  |
| 31.525 | | | 367.1048 | | | | | | | | | | 5.917582 | | | | 2337.602 | | | 78.03125 | |  |  |  |  |  |
| 31.53333 | | | 367.1923 | | | | | | | | | | 5.915678 | | | | 2336.984 | | | 78.16406 | |  |  |  |  |  |
| 31.54167 | | | 367.2798 | | | | | | | | | | 5.91813 | | | | 2336.359 | | | 78.28906 | |  |  |  |  |  |
| 31.55 | | | 367.3709 | | | | | | | | | | 5.918292 | | | | 2335.727 | | | 78.15625 | |  |  |  |  |  |
| 31.55833 | | | 367.4592 | | | | | | | | | | 5.916922 | | | | 2335.102 | | | 78.09375 | |  |  |  |  |  |
| 31.56667 | | | 367.5474 | | | | | | | | | | 5.918696 | | | | 2334.445 | | | 78.10156 | |  |  |  |  |  |
| 31.575 | | | 367.6342 | | | | | | | | | | 5.919315 | | | | 2333.766 | | | 77.97656 | |  |  |  |  |  |
| 31.58333 | | | 367.7232 | | | | | | | | | | 5.917487 | | | | 2333.094 | | | 77.95313 | |  |  |  |  |  |
| 31.59167 | | | 367.8092 | | | | | | | | | | 5.919831 | | | | 2332.43 | | | 77.78906 | |  |  |  |  |  |
| 31.6 | | | 367.8975 | | | | | | | | | | 5.91963 | | | | 2331.797 | | | 77.49219 | |  |  |  |  |  |
| 31.60833 | | | 367.9828 | | | | | | | | | | 5.918112 | | | | 2331.156 | | | 77.25781 | |  |  |  |  |  |
| 31.61667 | | | 368.0703 | | | | | | | | | | 5.920033 | | | | 2330.508 | | | 77.20313 | |  |  |  |  |  |
| 31.625 | | | 368.1607 | | | | | | | | | | 5.920154 | | | | 2329.875 | | | 77.10156 | |  |  |  |  |  |
| 31.63333 | | | 368.2475 | | | | | | | | | | 5.918293 | | | | 2329.219 | | | 77.03125 | |  |  |  |  |  |
| 31.64167 | | | 368.3358 | | | | | | | | | | 5.920575 | | | | 2328.602 | | | 76.88281 | |  |  |  |  |  |
| 31.65 | | | 368.4218 | | | | | | | | | | 5.920142 | | | | 2328 | | | 76.85156 | |  |  |  |  |  |
| 31.65833 | | | 368.5122 | | | | | | | | | | 5.919134 | | | | 2327.383 | | | 76.92969 | |  |  |  |  |  |
| 31.66667 | | | 368.6019 | | | | | | | | | | 5.921276 | | | | 2326.75 | | | 76.78906 | |  |  |  |  |  |
| 31.675 | | | 368.6909 | | | | | | | | | | 5.920664 | | | | 2326.125 | | | 76.77344 | |  |  |  |  |  |
| 31.68333 | | | 368.7798 | | | | | | | | | | 5.920456 | | | | 2325.484 | | | 76.74219 | |  |  |  |  |  |
| 31.69167 | | | 368.8695 | | | | | | | | | | 5.923239 | | | | 2324.859 | | | 76.57031 | |  |  |  |  |  |
| 31.7 | | | 368.9577 | | | | | | | | | | 5.923308 | | | | 2324.227 | | | 76.65625 | |  |  |  |  |  |
| 31.70833 | | | 369.0424 | | | | | | | | | | 5.922852 | | | | 2323.578 | | | 76.71875 | |  |  |  |  |  |
| 31.71667 | | | 369.1313 | | | | | | | | | | 5.924854 | | | | 2322.953 | | | 76.64844 | |  |  |  |  |  |
| 31.725 | | | 369.2188 | | | | | | | | | | 5.925014 | | | | 2322.313 | | | 76.71094 | |  |  |  |  |  |
| 31.73333 | | | 369.3107 | | | | | | | | | | 5.924164 | | | | 2321.68 | | | 76.70313 | |  |  |  |  |  |
| 31.74167 | | | 369.3982 | | | | | | | | | | 5.926426 | | | | 2321.055 | | | 76.64844 | |  |  |  |  |  |
| 31.75 | | | 369.485 | | | | | | | | | | 5.927117 | | | | 2320.398 | | | 76.69531 | |  |  |  |  |  |
| 31.75833 | | | 369.5718 | | | | | | | | | | 5.9255 | | | | 2319.758 | | | 76.64844 | |  |  |  |  |  |
| 31.76667 | | | 369.6614 | | | | | | | | | | 5.927867 | | | | 2319.117 | | | 76.875 | |  |  |  |  |  |
| 31.775 | | | 369.749 | | | | | | | | | | 5.927808 | | | | 2318.477 | | | 76.91406 | |  |  |  |  |  |
| 31.78333 | | | 369.8365 | | | | | | | | | | 5.926363 | | | | 2317.836 | | | 76.53125 | |  |  |  |  |  |
| 31.79167 | | | 369.9276 | | | | | | | | | | 5.92872 | | | | 2317.203 | | | 76.47656 | |  |  |  |  |  |
| 31.8 | | | 370.0158 | | | | | | | | | | 5.928017 | | | | 2316.555 | | | 76.46094 | |  |  |  |  |  |
[truncated: 353,083 more chars]
