# Supplementary material for: Characterization dataset for pre- and post-irradiated shrimp waste chitosan
Source: Data Brief. 2020 Jul 25;32:106081. doi: 10.1016/j.dib.2020.106081 (PMC7397402; doi:10.1016/j.dib.2020.106081)
Supplement: Supplementary file 1 [file mmc1.zip › TGA-CH20.docx]

| Module | | TG/DTA |  |  |  |  |  |  |  |  |
| --- | --- | --- | --- | --- | --- | --- | --- | --- | --- | --- |
| Channel | | 1 |  |  |  |  |  |  |  |  |
| Data Name | | S-Ch-3,(01-04-2017) | |  |  |  |  |  |  |  |
| Measurement Time | | 4/1/2017 7:19:33 PM | |  |  |  |  |  |  |  |
| Sample Name | | S-Ch-3 |  |  |  |  |  |  |  |  |
| Sample Weight | | 5.175 | mg |  |  |  |  |  |  |  |
| Reference Name | | Alumina |  |  |  |  |  |  |  |  |
| Reference Weight | | 5.5 | mg |  |  |  |  |  |  |  |
| Temperature Program | | | Cel | Cel | Cel/min | min | s | Gas1 | Gas2 | Store |
|  | | 1 | 50 | 650 | 10 | 0 | 0.5 | Off | Off | On |
|  | | Temperature Program Mode | Lamp |  |  |  |  |  |  |  |
|  |
|  |
|  |
|  |
|  |
|  |
|  |
|  | |  |  |  |  |  |  |  |  |  |
|  | |  |  |  |  |  |  |  |  |  |
| Time | | Temp. | DTA | TG | DTG |  |  |  |  |  |
| min | | Cel | uV | ug | ug/min |  |  |  |  |  |
| 0.008333 | | 52.88446 | -0.69382 | 5168.781 | 54.26563 |  |  |  |  |  |
| 0.016667 | | 52.8981 | -0.69214 | 5168.313 | 54.32031 |  |  |  |  |  |
| 0.025 | | 52.91515 | -0.69046 | 5167.844 | 54.27344 |  |  |  |  |  |
| 0.033333 | | 52.92879 | -0.68897 | 5167.391 | 54.29688 |  |  |  |  |  |
| 0.041667 | | 52.93788 | -0.68771 | 5166.93 | 54.50781 |  |  |  |  |  |
| 0.05 | | 52.95493 | -0.68587 | 5166.484 | 54.375 |  |  |  |  |  |
| 0.058333 | | 52.97084 | -0.68399 | 5166.047 | 54.3125 |  |  |  |  |  |
| 0.066667 | | 52.98902 | -0.68171 | 5165.578 | 54.08594 |  |  |  |  |  |
| 0.075 | | 53.00266 | -0.67946 | 5165.133 | 54.04688 |  |  |  |  |  |
| 0.083333 | | 53.01743 | -0.67703 | 5164.672 | 53.88281 |  |  |  |  |  |
| 0.091667 | | 53.03675 | -0.6744 | 5164.188 | 53.76563 |  |  |  |  |  |
| 0.1 | | 53.05153 | -0.67217 | 5163.75 | 53.46875 |  |  |  |  |  |
| 0.108333 | | 53.06744 | -0.66965 | 5163.297 | 53.36719 |  |  |  |  |  |
| 0.116667 | | 53.0879 | -0.66689 | 5162.883 | 53.1875 |  |  |  |  |  |
| 0.125 | | 53.10949 | -0.66432 | 5162.422 | 52.9375 |  |  |  |  |  |
| 0.133333 | | 53.11972 | -0.66238 | 5161.992 | 52.84375 |  |  |  |  |  |
| 0.141667 | | 53.13449 | -0.65995 | 5161.547 | 52.70313 |  |  |  |  |  |
| 0.15 | | 53.14813 | -0.65769 | 5161.148 | 52.92188 |  |  |  |  |  |
| 0.158333 | | 53.1595 | -0.65572 | 5160.711 | 52.71094 |  |  |  |  |  |
| 0.166667 | | 53.17427 | -0.65327 | 5160.289 | 52.67188 |  |  |  |  |  |
| 0.175 | | 53.19019 | -0.65089 | 5159.875 | 52.58594 |  |  |  |  |  |
| 0.183333 | | 53.20723 | -0.64853 | 5159.453 | 52.49219 |  |  |  |  |  |
| 0.191667 | | 53.22542 | -0.64619 | 5159.031 | 52.35156 |  |  |  |  |  |
| 0.2 | | 53.23906 | -0.64418 | 5158.555 | 52.28125 |  |  |  |  |  |
| 0.208333 | | 53.25156 | -0.64237 | 5158.148 | 52.34375 |  |  |  |  |  |
| 0.216667 | | 53.27201 | -0.6399 | 5157.727 | 52.14844 |  |  |  |  |  |
| 0.225 | | 53.28906 | -0.63783 | 5157.313 | 52.125 |  |  |  |  |  |
| 0.233333 | | 53.30497 | -0.63611 | 5156.891 | 51.83594 |  |  |  |  |  |
| 0.241667 | | 53.3243 | -0.63445 | 5156.484 | 51.94531 |  |  |  |  |  |
| 0.25 | | 53.3368 | -0.63315 | 5156.063 | 51.9375 |  |  |  |  |  |
| 0.258333 | | 53.3493 | -0.63129 | 5155.625 | 52.04688 |  |  |  |  |  |
| 0.266667 | | 53.36521 | -0.62919 | 5155.211 | 52.10156 |  |  |  |  |  |
| 0.275 | | 53.37543 | -0.62747 | 5154.789 | 52.04688 |  |  |  |  |  |
| 0.283333 | | 53.39021 | -0.62511 | 5154.398 | 52.03125 |  |  |  |  |  |
| 0.291667 | | 53.40499 | -0.62266 | 5153.953 | 51.85156 |  |  |  |  |  |
| 0.3 | | 53.4209 | -0.62014 | 5153.516 | 51.86719 |  |  |  |  |  |
| 0.308333 | | 53.43681 | -0.6175 | 5153.07 | 51.83594 |  |  |  |  |  |
| 0.316667 | | 53.45386 | -0.61446 | 5152.617 | 51.71094 |  |  |  |  |  |
| 0.325 | | 53.47091 | -0.61131 | 5152.172 | 51.28906 |  |  |  |  |  |
| 0.333333 | | 53.48682 | -0.60825 | 5151.742 | 51.09375 |  |  |  |  |  |
| 0.341667 | | 53.50386 | -0.60535 | 5151.32 | 50.82813 |  |  |  |  |  |
| 0.35 | | 53.51637 | -0.60282 | 5150.891 | 50.60156 |  |  |  |  |  |
| 0.358333 | | 53.53682 | -0.59985 | 5150.453 | 50.35938 |  |  |  |  |  |
| 0.366667 | | 53.5516 | -0.59733 | 5150.039 | 50.52344 |  |  |  |  |  |
| 0.375 | | 53.57092 | -0.59417 | 5149.672 | 50.5 |  |  |  |  |  |
| 0.383333 | | 53.58456 | -0.59155 | 5149.266 | 50.32813 |  |  |  |  |  |
| 0.391667 | | 53.6016 | -0.58869 | 5148.875 | 50.27344 |  |  |  |  |  |
| 0.4 | | 53.61524 | -0.58612 | 5148.484 | 50.19531 |  |  |  |  |  |
| 0.408333 | | 53.63115 | -0.58346 | 5148.094 | 50.40625 |  |  |  |  |  |
| 0.416667 | | 53.65047 | -0.58091 | 5147.648 | 50.21094 |  |  |  |  |  |
| 0.425 | | 53.66525 | -0.57874 | 5147.227 | 50.17188 |  |  |  |  |  |
| 0.433333 | | 53.68571 | -0.57605 | 5146.82 | 50.14063 |  |  |  |  |  |
| 0.441667 | | 53.70162 | -0.57355 | 5146.406 | 50.0625 |  |  |  |  |  |
| 0.45 | | 53.72207 | -0.57082 | 5145.992 | 49.96875 |  |  |  |  |  |
| 0.458333 | | 53.74026 | -0.56847 | 5145.531 | 49.67969 |  |  |  |  |  |
| 0.466667 | | 53.75503 | -0.56641 | 5145.148 | 49.49219 |  |  |  |  |  |
| 0.475 | | 53.76867 | -0.56443 | 5144.727 | 49.10156 |  |  |  |  |  |
| 0.483333 | | 53.78799 | -0.56203 | 5144.313 | 48.90625 |  |  |  |  |  |
| 0.491667 | | 53.80504 | -0.55967 | 5143.906 | 48.64063 |  |  |  |  |  |
| 0.5 | | 53.81641 | -0.55745 | 5143.508 | 48.55469 |  |  |  |  |  |
| 0.508333 | | 53.83118 | -0.55508 | 5143.133 | 48.45313 |  |  |  |  |  |
| 0.516667 | | 53.84254 | -0.55307 | 5142.75 | 48.45313 |  |  |  |  |  |
| 0.525 | | 53.85846 | -0.55071 | 5142.313 | 48.23438 |  |  |  |  |  |
| 0.533333 | | 53.86982 | -0.5487 | 5141.938 | 48.11719 |  |  |  |  |  |
| 0.541667 | | 53.88005 | -0.54718 | 5141.641 | 48.22656 |  |  |  |  |  |
| 0.55 | | 53.8971 | -0.54499 | 5141.234 | 48.41406 |  |  |  |  |  |
| 0.558333 | | 53.91642 | -0.54256 | 5140.844 | 48.50781 |  |  |  |  |  |
| 0.566667 | | 53.93574 | -0.54016 | 5140.445 | 48.53125 |  |  |  |  |  |
| 0.575 | | 53.95506 | -0.53764 | 5140.063 | 48.57031 |  |  |  |  |  |
| 0.583333 | | 53.98006 | -0.53487 | 5139.672 | 48.65625 |  |  |  |  |  |
| 0.591667 | | 54.00166 | -0.53211 | 5139.266 | 48.63281 |  |  |  |  |  |
| 0.6 | | 54.02552 | -0.52877 | 5138.836 | 48.54688 |  |  |  |  |  |
| 0.608333 | | 54.04598 | -0.52556 | 5138.414 | 48.60938 |  |  |  |  |  |
| 0.616667 | | 54.06758 | -0.52263 | 5138 | 48.51563 |  |  |  |  |  |
| 0.625 | | 54.09372 | -0.51953 | 5137.508 | 48.63281 |  |  |  |  |  |
| 0.633333 | | 54.1119 | -0.51743 | 5137.18 | 48.625 |  |  |  |  |  |
| 0.641667 | | 54.1369 | -0.51449 | 5136.688 | 48.71094 |  |  |  |  |  |
| 0.65 | | 54.15281 | -0.5125 | 5136.367 | 48.84375 |  |  |  |  |  |
| 0.658333 | | 54.17213 | -0.51038 | 5135.945 | 48.75 |  |  |  |  |  |
| 0.666667 | | 54.19146 | -0.5082 | 5135.555 | 48.8125 |  |  |  |  |  |
| 0.675 | | 54.21078 | -0.5063 | 5135.133 | 48.76563 |  |  |  |  |  |
| 0.683333 | | 54.23237 | -0.50441 | 5134.719 | 48.82031 |  |  |  |  |  |
| 0.691667 | | 54.25396 | -0.50229 | 5134.297 | 48.75 |  |  |  |  |  |
| 0.7 | | 54.2767 | -0.49966 | 5133.875 | 48.71875 |  |  |  |  |  |
| 0.708333 | | 54.30056 | -0.49661 | 5133.383 | 48.32813 |  |  |  |  |  |
| 0.716667 | | 54.31647 | -0.49469 | 5133.055 | 48 |  |  |  |  |  |
| 0.725 | | 54.34034 | -0.49226 | 5132.578 | 47.8125 |  |  |  |  |  |
| 0.733333 | | 54.36193 | -0.49034 | 5132.156 | 47.70313 |  |  |  |  |  |
| 0.741667 | | 54.38125 | -0.48866 | 5131.781 | 47.64844 |  |  |  |  |  |
| 0.75 | | 54.40398 | -0.4869 | 5131.383 | 47.46094 |  |  |  |  |  |
| 0.758333 | | 54.42217 | -0.48547 | 5131.078 | 47.30469 |  |  |  |  |  |
| 0.766667 | | 54.44263 | -0.48375 | 5130.68 | 46.96875 |  |  |  |  |  |
| 0.775 | | 54.46309 | -0.48221 | 5130.289 | 46.88281 |  |  |  |  |  |
| 0.783333 | | 54.48013 | -0.4807 | 5130 | 46.69531 |  |  |  |  |  |
| 0.791667 | | 54.50968 | -0.47797 | 5129.523 | 46.74219 |  |  |  |  |  |
| 0.8 | | 54.53127 | -0.47612 | 5129.211 | 46.57813 |  |  |  |  |  |
| 0.808333 | | 54.55969 | -0.47349 | 5128.773 | 46.57031 |  |  |  |  |  |
| 0.816667 | | 54.57674 | -0.47188 | 5128.5 | 46.66406 |  |  |  |  |  |
| 0.825 | | 54.60401 | -0.46941 | 5128.117 | 46.49219 |  |  |  |  |  |
| 0.833333 | | 54.62333 | -0.46764 | 5127.75 | 46.67188 |  |  |  |  |  |
| 0.841667 | | 54.64379 | -0.46583 | 5127.375 | 46.79688 |  |  |  |  |  |
| 0.85 | | 54.66993 | -0.46366 | 5126.984 | 46.76563 |  |  |  |  |  |
| 0.858333 | | 54.69493 | -0.46122 | 5126.602 | 46.92188 |  |  |  |  |  |
| 0.866667 | | 54.7188 | -0.45894 | 5126.203 | 47.02344 |  |  |  |  |  |
| 0.875 | | 54.74267 | -0.45662 | 5125.758 | 47.04688 |  |  |  |  |  |
| 0.883333 | | 54.76426 | -0.45469 | 5125.344 | 47.20313 |  |  |  |  |  |
| 0.891667 | | 54.78358 | -0.45326 | 5125.023 | 47.13281 |  |  |  |  |  |
| 0.9 | | 54.81313 | -0.45114 | 5124.555 | 47.0625 |  |  |  |  |  |
| 0.908333 | | 54.83472 | -0.44976 | 5124.141 | 47.09375 |  |  |  |  |  |
| 0.916667 | | 54.85632 | -0.44855 | 5123.813 | 46.94531 |  |  |  |  |  |
| 0.925 | | 54.88814 | -0.44691 | 5123.344 | 46.78906 |  |  |  |  |  |
| 0.933333 | | 54.91428 | -0.44503 | 5122.93 | 46.70313 |  |  |  |  |  |
| 0.941667 | | 54.93587 | -0.44321 | 5122.617 | 46.48438 |  |  |  |  |  |
| 0.95 | | 54.9677 | -0.44078 | 5122.156 | 46.28906 |  |  |  |  |  |
| 0.958333 | | 54.99384 | -0.43914 | 5121.758 | 46.25781 |  |  |  |  |  |
| 0.966667 | | 55.01316 | -0.438 | 5121.469 | 46.35156 |  |  |  |  |  |
| 0.975 | | 55.04384 | -0.43618 | 5121.016 | 46.23438 |  |  |  |  |  |
| 0.983333 | | 55.06771 | -0.43461 | 5120.625 | 46.24219 |  |  |  |  |  |
| 0.991667 | | 55.08931 | -0.43321 | 5120.352 | 46.1875 |  |  |  |  |  |
| 1 | | 55.11772 | -0.43116 | 5120 | 46.39063 |  |  |  |  |  |
| 1.008333 | | 55.14386 | -0.42928 | 5119.602 | 46.49219 |  |  |  |  |  |
| 1.016667 | | 55.17 | -0.42776 | 5119.203 | 46.71875 |  |  |  |  |  |
| 1.025 | | 55.19614 | -0.42665 | 5118.82 | 46.98438 |  |  |  |  |  |
| 1.033333 | | 55.22001 | -0.42555 | 5118.445 | 47.29688 |  |  |  |  |  |
| 1.041667 | | 55.2541 | -0.42393 | 5117.984 | 47.51563 |  |  |  |  |  |
| 1.05 | | 55.28479 | -0.42241 | 5117.555 | 47.49219 |  |  |  |  |  |
| 1.058333 | | 55.30524 | -0.4215 | 5117.234 | 47.35156 |  |  |  |  |  |
| 1.066667 | | 55.3382 | -0.41983 | 5116.813 | 47.50781 |  |  |  |  |  |
| 1.075 | | 55.36548 | -0.41857 | 5116.383 | 47.50781 |  |  |  |  |  |
| 1.083333 | | 55.39162 | -0.41745 | 5115.938 | 47.19531 |  |  |  |  |  |
| 1.091667 | | 55.42912 | -0.41584 | 5115.438 | 47.16406 |  |  |  |  |  |
| 1.1 | | 55.4564 | -0.41486 | 5115.031 | 47.10156 |  |  |  |  |  |
| 1.108333 | | 55.48595 | -0.41395 | 5114.664 | 47.17188 |  |  |  |  |  |
| 1.116667 | | 55.51777 | -0.41316 | 5114.258 | 46.91406 |  |  |  |  |  |
| 1.125 | | 55.5405 | -0.41258 | 5113.945 | 46.74219 |  |  |  |  |  |
| 1.133333 | | 55.57801 | -0.41113 | 5113.539 | 46.39844 |  |  |  |  |  |
| 1.141667 | | 55.60301 | -0.41014 | 5113.203 | 46.78125 |  |  |  |  |  |
| 1.15 | | 55.63256 | -0.40954 | 5112.813 | 46.84375 |  |  |  |  |  |
| 1.158333 | | 55.66779 | -0.40854 | 5112.43 | 46.6875 |  |  |  |  |  |
| 1.166667 | | 55.69507 | -0.40813 | 5112.078 | 46.71094 |  |  |  |  |  |
| 1.175 | | 55.72575 | -0.40764 | 5111.711 | 46.57031 |  |  |  |  |  |
| 1.183333 | | 55.75417 | -0.40722 | 5111.375 | 46.60156 |  |  |  |  |  |
| 1.191667 | | 55.78599 | -0.40602 | 5110.93 | 46.65625 |  |  |  |  |  |
| 1.2 | | 55.81213 | -0.40507 | 5110.531 | 46.625 |  |  |  |  |  |
| 1.208333 | | 55.83827 | -0.4043 | 5110.164 | 46.58594 |  |  |  |  |  |
| 1.216667 | | 55.86782 | -0.40362 | 5109.766 | 46.75781 |  |  |  |  |  |
| 1.225 | | 55.89964 | -0.40294 | 5109.398 | 46.66406 |  |  |  |  |  |
| 1.233333 | | 55.93601 | -0.40254 | 5108.922 | 46.77344 |  |  |  |  |  |
| 1.241667 | | 55.9576 | -0.40247 | 5108.617 | 46.875 |  |  |  |  |  |
| 1.25 | | 55.99738 | -0.40166 | 5108.164 | 47.125 |  |  |  |  |  |
| 1.258333 | | 56.02125 | -0.40127 | 5107.836 | 47.08594 |  |  |  |  |  |
| 1.266667 | | 56.06216 | -0.40045 | 5107.352 | 47.16406 |  |  |  |  |  |
| 1.275 | | 56.09626 | -0.39995 | 5106.977 | 47.25 |  |  |  |  |  |
| 1.283333 | | 56.13035 | -0.39949 | 5106.563 | 47.30469 |  |  |  |  |  |
| 1.291667 | | 56.16217 | -0.39891 | 5106.234 | 47.125 |  |  |  |  |  |
| 1.3 | | 56.19173 | -0.39867 | 5105.828 | 47.20313 |  |  |  |  |  |
| 1.308333 | | 56.22809 | -0.39789 | 5105.336 | 47.39063 |  |  |  |  |  |
| 1.316667 | | 56.25196 | -0.39718 | 5105.016 | 47.38281 |  |  |  |  |  |
| 1.325 | | 56.28492 | -0.39628 | 5104.625 | 47.39844 |  |  |  |  |  |
| 1.333333 | | 56.31788 | -0.39581 | 5104.219 | 47.29688 |  |  |  |  |  |
| 1.341667 | | 56.3588 | -0.39516 | 5103.781 | 47.22656 |  |  |  |  |  |
| 1.35 | | 56.39175 | -0.39489 | 5103.367 | 47.25 |  |  |  |  |  |
| 1.358333 | | 56.41676 | -0.39493 | 5103.031 | 47.35156 |  |  |  |  |  |
| 1.366667 | | 56.45426 | -0.3943 | 5102.641 | 47.52344 |  |  |  |  |  |
| 1.375 | | 56.4929 | -0.39345 | 5102.242 | 47.61719 |  |  |  |  |  |
| 1.383333 | | 56.53609 | -0.39332 | 5101.805 | 47.51563 |  |  |  |  |  |
| 1.391667 | | 56.5645 | -0.39345 | 5101.492 | 47.35938 |  |  |  |  |  |
| 1.4 | | 56.60996 | -0.39359 | 5101.008 | 47.34375 |  |  |  |  |  |
| 1.408333 | | 56.64633 | -0.39378 | 5100.602 | 47.22656 |  |  |  |  |  |
| 1.416667 | | 56.68611 | -0.3935 | 5100.172 | 47.0625 |  |  |  |  |  |
| 1.425 | | 56.72475 | -0.39293 | 5099.742 | 46.95313 |  |  |  |  |  |
| 1.433333 | | 56.75544 | -0.39248 | 5099.445 | 46.9375 |  |  |  |  |  |
| 1.441667 | | 56.79408 | -0.39185 | 5099.07 | 46.49219 |  |  |  |  |  |
| 1.45 | | 56.83386 | -0.39142 | 5098.688 | 46.22656 |  |  |  |  |  |
| 1.458333 | | 56.86454 | -0.3919 | 5098.305 | 46.25781 |  |  |  |  |  |
| 1.466667 | | 56.90205 | -0.39186 | 5097.914 | 46.32031 |  |  |  |  |  |
| 1.475 | | 56.94183 | -0.39173 | 5097.547 | 46.29688 |  |  |  |  |  |
| 1.483333 | | 56.9907 | -0.39198 | 5097.102 | 46.39063 |  |  |  |  |  |
| 1.491667 | | 57.02934 | -0.3924 | 5096.766 | 46.53125 |  |  |  |  |  |
| 1.5 | | 57.05661 | -0.39278 | 5096.5 | 46.39844 |  |  |  |  |  |
| 1.508333 | | 57.0998 | -0.39318 | 5096.031 | 46.75781 |  |  |  |  |  |
| 1.516667 | | 57.13844 | -0.3933 | 5095.648 | 46.82813 |  |  |  |  |  |
| 1.525 | | 57.17368 | -0.39358 | 5095.25 | 46.85938 |  |  |  |  |  |
| 1.533333 | | 57.2055 | -0.39375 | 5094.93 | 46.83594 |  |  |  |  |  |
| 1.541667 | | 57.24869 | -0.39415 | 5094.539 | 46.71875 |  |  |  |  |  |
| 1.55 | | 57.29642 | -0.39488 | 5094.055 | 46.625 |  |  |  |  |  |
| 1.558333 | | 57.3362 | -0.39544 | 5093.648 | 46.30469 |  |  |  |  |  |
| 1.566667 | | 57.36348 | -0.39607 | 5093.32 | 46.13281 |  |  |  |  |  |
| 1.575 | | 57.40098 | -0.39638 | 5092.922 | 45.96094 |  |  |  |  |  |
| 1.583333 | | 57.44189 | -0.39706 | 5092.539 | 45.85938 |  |  |  |  |  |
| 1.591667 | | 57.48963 | -0.39845 | 5092.094 | 45.52344 |  |  |  |  |  |
| 1.6 | | 57.52372 | -0.39909 | 5091.789 | 45.34375 |  |  |  |  |  |
| 1.608333 | | 57.56237 | -0.40012 | 5091.438 | 45.30469 |  |  |  |  |  |
| 1.616667 | | 57.60442 | -0.40063 | 5091.086 | 45.64844 |  |  |  |  |  |
| 1.625 | | 57.6442 | -0.40083 | 5090.734 | 45.67969 |  |  |  |  |  |
| 1.633333 | | 57.6817 | -0.40141 | 5090.359 | 45.66406 |  |  |  |  |  |
| 1.641667 | | 57.72148 | -0.40187 | 5090 | 45.97656 |  |  |  |  |  |
| 1.65 | | 57.77489 | -0.40215 | 5089.594 | 46.17188 |  |  |  |  |  |
| 1.658333 | | 57.81467 | -0.40254 | 5089.203 | 46.375 |  |  |  |  |  |
| 1.666667 | | 57.85672 | -0.40222 | 5088.781 | 46.63281 |  |  |  |  |  |
| 1.675 | | 57.89082 | -0.402 | 5088.469 | 46.875 |  |  |  |  |  |
| 1.683333 | | 57.94196 | -0.40251 | 5088 | 47.125 |  |  |  |  |  |
| 1.691667 | | 57.97947 | -0.40263 | 5087.664 | 47.20313 |  |  |  |  |  |
| 1.7 | | 58.01697 | -0.40324 | 5087.258 | 47.24219 |  |  |  |  |  |
| 1.708333 | | 58.06129 | -0.40254 | 5086.82 | 47.30469 |  |  |  |  |  |
| 1.716667 | | 58.1113 | -0.40229 | 5086.32 | 47.60156 |  |  |  |  |  |
| 1.725 | | 58.14767 | -0.40237 | 5085.977 | 47.46875 |  |  |  |  |  |
| 1.733333 | | 58.19768 | -0.40256 | 5085.469 | 47.5 |  |  |  |  |  |
| 1.741667 | | 58.24314 | -0.40266 | 5085.063 | 47.67969 |  |  |  |  |  |
| 1.75 | | 58.27721 | -0.40275 | 5084.75 | 47.79688 |  |  |  |  |  |
| 1.758333 | | 58.31669 | -0.40337 | 5084.336 | 47.78906 |  |  |  |  |  |
| 1.766667 | | 58.36715 | -0.40489 | 5083.82 | 47.90625 |  |  |  |  |  |
| 1.775 | | 58.40005 | -0.40597 | 5083.523 | 47.92188 |  |  |  |  |  |
| 1.783333 | | 58.44941 | -0.40746 | 5083.039 | 47.99219 |  |  |  |  |  |
| 1.791667 | | 58.48779 | -0.40843 | 5082.625 | 48.04688 |  |  |  |  |  |
| 1.8 | | 58.52618 | -0.40831 | 5082.297 | 48.03125 |  |  |  |  |  |
| 1.808333 | | 58.57444 | -0.40978 | 5081.789 | 48.22656 |  |  |  |  |  |
| 1.816667 | | 58.60954 | -0.41085 | 5081.477 | 48.25781 |  |  |  |  |  |
| 1.825 | | 58.65231 | -0.41239 | 5081.078 | 48.39844 |  |  |  |  |  |
| 1.833333 | | 58.6918 | -0.41418 | 5080.672 | 48.3125 |  |  |  |  |  |
| 1.841667 | | 58.74664 | -0.41517 | 5080.188 | 48.07813 |  |  |  |  |  |
| 1.85 | | 58.78722 | -0.41637 | 5079.805 | 48.14844 |  |  |  |  |  |
| 1.858333 | | 58.83438 | -0.41763 | 5079.375 | 48.03125 |  |  |  |  |  |
| 1.866667 | | 58.87167 | -0.41882 | 5079.047 | 48.03125 |  |  |  |  |  |
| 1.875 | | 58.91774 | -0.4205 | 5078.633 | 48.02344 |  |  |  |  |  |
| 1.883333 | | 58.97039 | -0.42236 | 5078.164 | 48.09375 |  |  |  |  |  |
| 1.891667 | | 59.01645 | -0.42357 | 5077.789 | 48.08594 |  |  |  |  |  |
| 1.9 | | 59.06252 | -0.42506 | 5077.375 | 48.26563 |  |  |  |  |  |
| 1.908333 | | 59.09652 | -0.4259 | 5077.07 | 48.39844 |  |  |  |  |  |
| 1.916667 | | 59.15465 | -0.42634 | 5076.602 | 48.47656 |  |  |  |  |  |
| 1.925 | | 59.18755 | -0.42717 | 5076.273 | 48.89844 |  |  |  |  |  |
| 1.933333 | | 59.24568 | -0.42838 | 5075.789 | 49.16406 |  |  |  |  |  |
| 1.941667 | | 59.27639 | -0.42979 | 5075.461 | 49.5625 |  |  |  |  |  |
| 1.95 | | 59.32136 | -0.43084 | 5075.047 | 49.77344 |  |  |  |  |  |
| 1.958333 | | 59.36523 | -0.4321 | 5074.625 | 49.94531 |  |  |  |  |  |
| 1.966667 | | 59.42227 | -0.43266 | 5074.133 | 50.10938 |  |  |  |  |  |
| 1.975 | | 59.45846 | -0.43364 | 5073.758 | 50 |  |  |  |  |  |
| 1.983333 | | 59.51111 | -0.43543 | 5073.234 | 49.74219 |  |  |  |  |  |
| 1.991667 | | 59.55169 | -0.43631 | 5072.852 | 49.83594 |  |  |  |  |  |
| 2 | | 59.59666 | -0.43758 | 5072.414 | 49.91406 |  |  |  |  |  |
| 2.008333 | | 59.65588 | -0.43824 | 5071.898 | 49.64063 |  |  |  |  |  |
| 2.016667 | | 59.69318 | -0.43903 | 5071.563 | 49.42188 |  |  |  |  |  |
| 2.025 | | 59.7546 | -0.44014 | 5071.055 | 49.17188 |  |  |  |  |  |
| 2.033333 | | 59.80396 | -0.44106 | 5070.68 | 49.10156 |  |  |  |  |  |
| 2.041667 | | 59.84015 | -0.44178 | 5070.336 | 49.22656 |  |  |  |  |  |
| 2.05 | | 59.88622 | -0.4431 | 5069.914 | 49.34375 |  |  |  |  |  |
| 2.058333 | | 59.94764 | -0.44418 | 5069.445 | 49.36719 |  |  |  |  |  |
| 2.066667 | | 59.98712 | -0.44499 | 5069.148 | 49.53906 |  |  |  |  |  |
| 2.075 | | 60.03318 | -0.44571 | 5068.773 | 49.57813 |  |  |  |  |  |
| 2.083333 | | 60.08254 | -0.44658 | 5068.375 | 49.625 |  |  |  |  |  |
| 2.091667 | | 60.13848 | -0.44833 | 5067.875 | 49.74219 |  |  |  |  |  |
| 2.1 | | 60.18674 | -0.4497 | 5067.461 | 50.02344 |  |  |  |  |  |
| 2.108333 | | 60.22293 | -0.4508 | 5067.109 | 50.22656 |  |  |  |  |  |
| 2.116667 | | 60.27338 | -0.45271 | 5066.57 | 50.32031 |  |  |  |  |  |
| 2.125 | | 60.32274 | -0.45439 | 5066.172 | 50.3125 |  |  |  |  |  |
| 2.133333 | | 60.37319 | -0.4562 | 5065.734 | 50.39844 |  |  |  |  |  |
| 2.141667 | | 60.41048 | -0.45788 | 5065.398 | 50.625 |  |  |  |  |  |
| 2.15 | | 60.46313 | -0.45998 | 5064.859 | 50.51563 |  |  |  |  |  |
| 2.158333 | | 60.51249 | -0.46123 | 5064.414 | 50.66406 |  |  |  |  |  |
| 2.166667 | | 60.56404 | -0.46285 | 5063.977 | 50.60938 |  |  |  |  |  |
| 2.175 | | 60.61668 | -0.46423 | 5063.563 | 50.54688 |  |  |  |  |  |
| 2.183333 | | 60.66714 | -0.46572 | 5063.125 | 50.51563 |  |  |  |  |  |
| 2.191667 | | 60.71869 | -0.46704 | 5062.688 | 50.5 |  |  |  |  |  |
| 2.2 | | 60.76914 | -0.46874 | 5062.281 | 50.46875 |  |  |  |  |  |
| 2.208333 | | 60.81959 | -0.47075 | 5061.836 | 50.28125 |  |  |  |  |  |
| 2.216667 | | 60.86675 | -0.4727 | 5061.422 | 50.11719 |  |  |  |  |  |
| 2.225 | | 60.9194 | -0.474 | 5061.016 | 50.13281 |  |  |  |  |  |
| 2.233333 | | 60.97095 | -0.47535 | 5060.602 | 49.96875 |  |  |  |  |  |
| 2.241667 | | 61.02359 | -0.47708 | 5060.188 | 49.75 |  |  |  |  |  |
| 2.25 | | 61.07295 | -0.47893 | 5059.773 | 49.95313 |  |  |  |  |  |
| 2.258333 | | 61.11792 | -0.48103 | 5059.383 | 50.19531 |  |  |  |  |  |
| 2.266667 | | 61.16947 | -0.48195 | 5058.992 | 50.46094 |  |  |  |  |  |
| 2.275 | | 61.22102 | -0.4837 | 5058.578 | 50.50781 |  |  |  |  |  |
| 2.283333 | | 61.27695 | -0.48561 | 5058.18 | 50.6875 |  |  |  |  |  |
| 2.291667 | | 61.32851 | -0.48729 | 5057.789 | 51.1875 |  |  |  |  |  |
| 2.3 | | 61.38225 | -0.48876 | 5057.359 | 51.59375 |  |  |  |  |  |
| 2.308333 | | 61.42612 | -0.49094 | 5056.898 | 51.88281 |  |  |  |  |  |
| 2.316667 | | 61.47877 | -0.4927 | 5056.445 | 52.52344 |  |  |  |  |  |
| 2.325 | | 61.52703 | -0.49487 | 5056.016 | 52.96094 |  |  |  |  |  |
| 2.333333 | | 61.57638 | -0.49681 | 5055.563 | 53.34375 |  |  |  |  |  |
| 2.341667 | | 61.62793 | -0.49803 | 5055.055 | 53.75781 |  |  |  |  |  |
| 2.35 | | 61.67729 | -0.49972 | 5054.57 | 53.96094 |  |  |  |  |  |
| 2.358333 | | 61.72774 | -0.50148 | 5054.094 | 54.46094 |  |  |  |  |  |
| 2.366667 | | 61.78039 | -0.50264 | 5053.563 | 54.96094 |  |  |  |  |  |
| 2.375 | | 61.83194 | -0.50419 | 5053.063 | 55.07813 |  |  |  |  |  |
| 2.383333 | | 61.88239 | -0.506 | 5052.57 | 55.24219 |  |  |  |  |  |
| 2.391667 | | 61.94271 | -0.50784 | 5052.07 | 55.32031 |  |  |  |  |  |
| 2.4 | | 62.00414 | -0.50872 | 5051.594 | 55.32031 |  |  |  |  |  |
| 2.408333 | | 62.06227 | -0.50909 | 5051.078 | 55.10938 |  |  |  |  |  |
| 2.416667 | | 62.1182 | -0.5099 | 5050.555 | 54.98438 |  |  |  |  |  |
| 2.425 | | 62.17633 | -0.51129 | 5050.086 | 54.88281 |  |  |  |  |  |
| 2.433333 | | 62.23227 | -0.51275 | 5049.617 | 54.73438 |  |  |  |  |  |
| 2.441667 | | 62.28821 | -0.51417 | 5049.156 | 54.58594 |  |  |  |  |  |
| 2.45 | | 62.34305 | -0.516 | 5048.695 | 54.25781 |  |  |  |  |  |
| 2.458333 | | 62.40228 | -0.51772 | 5048.266 | 54.0625 |  |  |  |  |  |
| 2.466667 | | 62.45931 | -0.51938 | 5047.82 | 54.07813 |  |  |  |  |  |
| 2.475 | | 62.51634 | -0.52103 | 5047.398 | 54.13281 |  |  |  |  |  |
| 2.483333 | | 62.56789 | -0.52362 | 5046.961 | 54.0625 |  |  |  |  |  |
| 2.491667 | | 62.62164 | -0.52644 | 5046.523 | 54.01563 |  |  |  |  |  |
| 2.5 | | 62.67648 | -0.52874 | 5046.125 | 53.97656 |  |  |  |  |  |
| 2.508333 | | 62.7346 | -0.53049 | 5045.695 | 54.24219 |  |  |  |  |  |
| 2.516667 | | 62.79164 | -0.53275 | 5045.25 | 54.4375 |  |  |  |  |  |
| 2.525 | | 62.84538 | -0.53557 | 5044.797 | 54.70313 |  |  |  |  |  |
| 2.533333 | | 62.89803 | -0.53803 | 5044.359 | 55.0625 |  |  |  |  |  |
| 2.541667 | | 62.95287 | -0.54017 | 5043.914 | 55.375 |  |  |  |  |  |
| 2.55 | | 63.00771 | -0.54217 | 5043.469 | 55.64844 |  |  |  |  |  |
| 2.558333 | | 63.06255 | -0.54472 | 5042.984 | 55.88281 |  |  |  |  |  |
| 2.566667 | | 63.12068 | -0.5466 | 5042.508 | 55.98438 |  |  |  |  |  |
| 2.575 | | 63.1821 | -0.54818 | 5042.023 | 56.3125 |  |  |  |  |  |
| 2.583333 | | 63.24242 | -0.54984 | 5041.508 | 56.66406 |  |  |  |  |  |
| 2.591667 | | 63.29617 | -0.55252 | 5041.016 | 56.53125 |  |  |  |  |  |
| 2.6 | | 63.3543 | -0.55444 | 5040.516 | 56.52344 |  |  |  |  |  |
| 2.608333 | | 63.41572 | -0.5557 | 5040.031 | 56.40625 |  |  |  |  |  |
| 2.616667 | | 63.47823 | -0.55663 | 5039.555 | 56.10156 |  |  |  |  |  |
| 2.625 | | 63.53637 | -0.55926 | 5039.039 | 56.01563 |  |  |  |  |  |
| 2.633333 | | 63.59449 | -0.56127 | 5038.531 | 56.04688 |  |  |  |  |  |
| 2.641667 | | 63.64933 | -0.56327 | 5038.086 | 55.82813 |  |  |  |  |  |
| 2.65 | | 63.70198 | -0.56532 | 5037.625 | 55.73438 |  |  |  |  |  |
| 2.658333 | | 63.75792 | -0.56773 | 5037.172 | 55.75 |  |  |  |  |  |
| 2.666667 | | 63.81495 | -0.56962 | 5036.75 | 55.63281 |  |  |  |  |  |
| 2.675 | | 63.87089 | -0.57111 | 5036.297 | 55.71875 |  |  |  |  |  |
| 2.683333 | | 63.92573 | -0.57281 | 5035.828 | 55.82031 |  |  |  |  |  |
| 2.691667 | | 63.98386 | -0.57474 | 5035.391 | 55.92969 |  |  |  |  |  |
| 2.7 | | 64.03979 | -0.57661 | 5034.938 | 55.96094 |  |  |  |  |  |
| 2.708333 | | 64.09573 | -0.57839 | 5034.461 | 56.17188 |  |  |  |  |  |
| 2.716667 | | 64.14838 | -0.58042 | 5034.008 | 56.34375 |  |  |  |  |  |
| 2.725 | | 64.20541 | -0.58274 | 5033.531 | 56.625 |  |  |  |  |  |
| 2.733333 | | 64.26244 | -0.58435 | 5033.047 | 56.98438 |  |  |  |  |  |
| 2.741667 | | 64.31838 | -0.58583 | 5032.563 | 56.91406 |  |  |  |  |  |
| 2.75 | | 64.37761 | -0.58739 | 5032.086 | 57.25781 |  |  |  |  |  |
| 2.758333 | | 64.43903 | -0.58948 | 5031.586 | 57.3125 |  |  |  |  |  |
| 2.766667 | | 64.49936 | -0.59059 | 5031.086 | 57.51563 |  |  |  |  |  |
| 2.775 | | 64.55529 | -0.5927 | 5030.563 | 57.64063 |  |  |  |  |  |
| 2.783333 | | 64.61343 | -0.59514 | 5030.047 | 57.80469 |  |  |  |  |  |
| 2.791667 | | 64.67374 | -0.59777 | 5029.578 | 57.92188 |  |  |  |  |  |
| 2.8 | | 64.74065 | -0.59916 | 5029.063 | 57.74219 |  |  |  |  |  |
| 2.808333 | | 64.80098 | -0.60091 | 5028.578 | 57.61719 |  |  |  |  |  |
| 2.816667 | | 64.8624 | -0.60305 | 5028.07 | 57.46875 |  |  |  |  |  |
| 2.825 | | 64.92491 | -0.60501 | 5027.586 | 57.47656 |  |  |  |  |  |
| 2.833333 | | 64.98305 | -0.60669 | 5027.078 | 57.28906 |  |  |  |  |  |
| 2.841667 | | 65.04118 | -0.6089 | 5026.594 | 57.26563 |  |  |  |  |  |
| 2.85 | | 65.1015 | -0.61149 | 5026.141 | 57.21094 |  |  |  |  |  |
| 2.858333 | | 65.16621 | -0.61319 | 5025.688 | 56.86719 |  |  |  |  |  |
| 2.866667 | | 65.22873 | -0.61493 | 5025.234 | 56.55469 |  |  |  |  |  |
| 2.875 | | 65.29015 | -0.61714 | 5024.758 | 56.47656 |  |  |  |  |  |
| 2.883333 | | 65.35157 | -0.61996 | 5024.305 | 56.15625 |  |  |  |  |  |
| 2.891667 | | 65.41409 | -0.62188 | 5023.844 | 56.19531 |  |  |  |  |  |
| 2.9 | | 65.47112 | -0.6239 | 5023.375 | 56.09375 |  |  |  |  |  |
| 2.908333 | | 65.52925 | -0.62592 | 5022.961 | 56.04688 |  |  |  |  |  |
| 2.916667 | | 65.59068 | -0.62848 | 5022.516 | 55.84375 |  |  |  |  |  |
| 2.925 | | 65.65209 | -0.63032 | 5022.063 | 55.96875 |  |  |  |  |  |
| 2.933333 | | 65.71022 | -0.6325 | 5021.633 | 56.05469 |  |  |  |  |  |
| 2.941667 | | 65.76616 | -0.63498 | 5021.156 | 56.32813 |  |  |  |  |  |
| 2.95 | | 65.82648 | -0.63757 | 5020.695 | 56.74219 |  |  |  |  |  |
| 2.958333 | | 65.8901 | -0.63926 | 5020.234 | 56.90625 |  |  |  |  |  |
| 2.966667 | | 65.95042 | -0.64151 | 5019.789 | 57.33594 |  |  |  |  |  |
| 2.975 | | 66.01184 | -0.64403 | 5019.305 | 57.36719 |  |  |  |  |  |
| 2.983333 | | 66.07875 | -0.6464 | 5018.82 | 57.625 |  |  |  |  |  |
| 2.991667 | | 66.14236 | -0.64806 | 5018.313 | 57.83594 |  |  |  |  |  |
| 3 | | 66.20488 | -0.65026 | 5017.781 | 57.92188 |  |  |  |  |  |
| 3.008333 | | 66.27069 | -0.65242 | 5017.289 | 58.14063 |  |  |  |  |  |
| 3.016667 | | 66.3354 | -0.65437 | 5016.758 | 58.17188 |  |  |  |  |  |
| 3.025 | | 66.40231 | -0.65571 | 5016.273 | 58.15625 |  |  |  |  |  |
| 3.033333 | | 66.46372 | -0.65759 | 5015.758 | 57.85938 |  |  |  |  |  |
| 3.041667 | | 66.52625 | -0.65985 | 5015.266 | 57.90625 |  |  |  |  |  |
| 3.05 | | 66.59205 | -0.6609 | 5014.75 | 57.72656 |  |  |  |  |  |
| 3.058333 | | 66.65676 | -0.66201 | 5014.25 | 57.52344 |  |  |  |  |  |
| 3.066667 | | 66.72257 | -0.66372 | 5013.758 | 57.47656 |  |  |  |  |  |
| 3.075 | | 66.78728 | -0.6656 | 5013.281 | 57.5625 |  |  |  |  |  |
| 3.083333 | | 66.8509 | -0.667 | 5012.836 | 57.71094 |  |  |  |  |  |
| 3.091667 | | 66.91013 | -0.66935 | 5012.352 | 57.82031 |  |  |  |  |  |
| 3.1 | | 66.97593 | -0.67147 | 5011.891 | 58.00781 |  |  |  |  |  |
| 3.108333 | | 67.03735 | -0.67314 | 5011.445 | 58.79688 |  |  |  |  |  |
| 3.116667 | | 67.10426 | -0.67452 | 5010.969 | 59.26563 |  |  |  |  |  |
| 3.125 | | 67.16458 | -0.67679 | 5010.469 | 59.50781 |  |  |  |  |  |
| 3.133333 | | 67.22381 | -0.67946 | 5009.961 | 60 |  |  |  |  |  |
| 3.141667 | | 67.29181 | -0.6805 | 5009.469 | 60.65625 |  |  |  |  |  |
| 3.15 | | 67.35542 | -0.68208 | 5008.953 | 61.07031 |  |  |  |  |  |
| 3.158333 | | 67.41685 | -0.68411 | 5008.352 | 61.55469 |  |  |  |  |  |
| 3.166667 | | 67.47717 | -0.68651 | 5007.789 | 61.85938 |  |  |  |  |  |
| 3.175 | | 67.54517 | -0.68776 | 5007.258 | 62.09375 |  |  |  |  |  |
| 3.183333 | | 67.60988 | -0.68991 | 5006.688 | 62.32813 |  |  |  |  |  |
| 3.191667 | | 67.67679 | -0.69218 | 5006.094 | 61.94531 |  |  |  |  |  |
| 3.2 | | 67.7415 | -0.69391 | 5005.531 | 61.875 |  |  |  |  |  |
| 3.208333 | | 67.81059 | -0.69479 | 5004.953 | 61.75 |  |  |  |  |  |
| 3.216667 | | 67.8786 | -0.69676 | 5004.414 | 61.64063 |  |  |  |  |  |
| 3.225 | | 67.94112 | -0.69901 | 5003.859 | 61.52344 |  |  |  |  |  |
| 3.233333 | | 68.00363 | -0.70037 | 5003.313 | 61.36719 |  |  |  |  |  |
| 3.241667 | | 68.07273 | -0.70193 | 5002.859 | 61.26563 |  |  |  |  |  |
| 3.25 | | 68.14073 | -0.70404 | 5002.367 | 61.30469 |  |  |  |  |  |
| 3.258333 | | 68.20216 | -0.70602 | 5001.875 | 61.17188 |  |  |  |  |  |
| 3.266667 | | 68.26796 | -0.70731 | 5001.383 | 60.90625 |  |  |  |  |  |
| 3.275 | | 68.33157 | -0.70951 | 5000.891 | 60.88281 |  |  |  |  |  |
| 3.283333 | | 68.39629 | -0.71225 | 5000.414 | 60.92969 |  |  |  |  |  |
| 3.291667 | | 68.461 | -0.71413 | 4999.922 | 61 |  |  |  |  |  |
| 3.3 | | 68.52571 | -0.71618 | 4999.406 | 61.125 |  |  |  |  |  |
| 3.308333 | | 68.59042 | -0.71854 | 4998.93 | 61.05469 |  |  |  |  |  |
| 3.316667 | | 68.65952 | -0.7203 | 4998.461 | 61 |  |  |  |  |  |
| 3.325 | | 68.72423 | -0.72163 | 4997.961 | 61.01563 |  |  |  |  |  |
| 3.333333 | | 68.79114 | -0.7233 | 4997.453 | 60.875 |  |  |  |  |  |
| 3.341667 | | 68.86133 | -0.72525 | 4996.93 | 60.92969 |  |  |  |  |  |
| 3.35 | | 68.92604 | -0.72626 | 4996.406 | 60.90625 |  |  |  |  |  |
| 3.358333 | | 68.99295 | -0.72794 | 4995.906 | 60.71875 |  |  |  |  |  |
| 3.366667 | | 69.05875 | -0.72983 | 4995.406 | 60.61719 |  |  |  |  |  |
| 3.375 | | 69.12457 | -0.73129 | 4994.898 | 60.46875 |  |  |  |  |  |
| 3.383333 | | 69.19147 | -0.7321 | 4994.406 | 60.24219 |  |  |  |  |  |
| 3.391667 | | 69.25837 | -0.73362 | 4993.891 | 60.03125 |  |  |  |  |  |
| 3.4 | | 69.32308 | -0.7351 | 4993.391 | 59.82813 |  |  |  |  |  |
| 3.408333 | | 69.39108 | -0.73643 | 4992.906 | 59.42188 |  |  |  |  |  |
| 3.416667 | | 69.46018 | -0.7384 | 4992.414 | 59.24219 |  |  |  |  |  |
| 3.425 | | 69.52599 | -0.74063 | 4991.938 | 59.10938 |  |  |  |  |  |
| 3.433333 | | 69.59509 | -0.74275 | 4991.477 | 59.21094 |  |  |  |  |  |
| 3.441667 | | 69.66199 | -0.74448 | 4991.008 | 59.33594 |  |  |  |  |  |
| 3.45 | | 69.73438 | -0.74662 | 4990.547 | 59.59375 |  |  |  |  |  |
| 3.458333 | | 69.80238 | -0.74884 | 4990.109 | 59.84375 |  |  |  |  |  |
| 3.466667 | | 69.87258 | -0.7505 | 4989.648 | 60.19531 |  |  |  |  |  |
| 3.475 | | 69.94497 | -0.75242 | 4989.172 | 60.74219 |  |  |  |  |  |
| 3.483333 | | 70.01187 | -0.75478 | 4988.664 | 61.22656 |  |  |  |  |  |
| 3.491667 | | 70.08097 | -0.75668 | 4988.148 | 61.85156 |  |  |  |  |  |
| 3.5 | | 70.14897 | -0.75816 | 4987.617 | 62.47656 |  |  |  |  |  |
| 3.508333 | | 70.21917 | -0.76027 | 4987.07 | 62.60938 |  |  |  |  |  |
| 3.516667 | | 70.29046 | -0.76212 | 4986.5 | 63.04688 |  |  |  |  |  |
| 3.525 | | 70.35715 | -0.76309 | 4985.922 | 63.35938 |  |  |  |  |  |
| 3.533333 | | 70.42164 | -0.76424 | 4985.336 | 63.33594 |  |  |  |  |  |
| 3.541667 | | 70.49246 | -0.76572 | 4984.719 | 63.53906 |  |  |  |  |  |
| 3.55 | | 70.56117 | -0.7673 | 4984.109 | 63.40625 |  |  |  |  |  |
| 3.558333 | | 70.62672 | -0.76837 | 4983.547 | 63.17969 |  |  |  |  |  |
| 3.566667 | | 70.69754 | -0.77035 | 4982.961 | 63.08594 |  |  |  |  |  |
| 3.575 | | 70.76308 | -0.77238 | 4982.391 | 62.78125 |  |  |  |  |  |
| 3.583333 | | 70.83073 | -0.77349 | 4981.867 | 62.50781 |  |  |  |  |  |
| 3.591667 | | 70.89416 | -0.77556 | 4981.313 | 62.58594 |  |  |  |  |  |
| 3.6 | | 70.95864 | -0.77774 | 4980.805 | 62.23438 |  |  |  |  |  |
| 3.608333 | | 71.03052 | -0.77907 | 4980.32 | 62 |  |  |  |  |  |
| 3.616667 | | 71.09818 | -0.78074 | 4979.813 | 61.95313 |  |  |  |  |  |
| 3.625 | | 71.16371 | -0.78338 | 4979.336 | 61.625 |  |  |  |  |  |
| 3.633333 | | 71.22608 | -0.7856 | 4978.859 | 61.8125 |  |  |  |  |  |
| 3.641667 | | 71.28951 | -0.78658 | 4978.328 | 61.89063 |  |  |  |  |  |
| 3.65 | | 71.35399 | -0.78839 | 4977.859 | 62 |  |  |  |  |  |
| 3.658333 | | 71.41847 | -0.79031 | 4977.383 | 62.03125 |  |  |  |  |  |
| 3.666667 | | 71.48506 | -0.79129 | 4976.875 | 62.35938 |  |  |  |  |  |
| 3.675 | | 71.55272 | -0.79192 | 4976.406 | 62.25 |  |  |  |  |  |
| 3.683333 | | 71.6172 | -0.79411 | 4975.867 | 62.39063 |  |  |  |  |  |
| 3.691667 | | 71.67957 | -0.79595 | 4975.344 | 62.84375 |  |  |  |  |  |
| 3.7 | | 71.74828 | -0.79693 | 4974.813 | 62.92188 |  |  |  |  |  |
| 3.708333 | | 71.81593 | -0.79817 | 4974.297 | 63.15625 |  |  |  |  |  |
| 3.716667 | | 71.88253 | -0.79977 | 4973.727 | 63.07031 |  |  |  |  |  |
| 3.725 | | 71.95229 | -0.80091 | 4973.227 | 63.32813 |  |  |  |  |  |
| 3.733333 | | 72.02418 | -0.80147 | 4972.688 | 63.49219 |  |  |  |  |  |
| 3.741667 | | 72.09395 | -0.80304 | 4972.109 | 63.60938 |  |  |  |  |  |
| 3.75 | | 72.16371 | -0.80435 | 4971.563 | 63.46094 |  |  |  |  |  |
| 3.758333 | | 72.2356 | -0.80523 | 4971.016 | 63.57813 |  |  |  |  |  |
| 3.766667 | | 72.31065 | -0.80664 | 4970.508 | 63.71875 |  |  |  |  |  |
| 3.775 | | 72.38147 | -0.80777 | 4969.953 | 63.77344 |  |  |  |  |  |
| 3.783333 | | 72.44701 | -0.8087 | 4969.406 | 63.95313 |  |  |  |  |  |
| 3.791667 | | 72.51784 | -0.80991 | 4968.867 | 64.04688 |  |  |  |  |  |
| 3.8 | | 72.59184 | -0.81156 | 4968.359 | 63.89063 |  |  |  |  |  |
| 3.808333 | | 72.66477 | -0.81297 | 4967.828 | 63.9375 |  |  |  |  |  |
| 3.816667 | | 72.7356 | -0.81427 | 4967.281 | 63.82813 |  |  |  |  |  |
| 3.825 | | 72.80642 | -0.81604 | 4966.758 | 63.77344 |  |  |  |  |  |
| 3.833333 | | 72.87408 | -0.81781 | 4966.195 | 63.67188 |  |  |  |  |  |
| 3.841667 | | 72.94595 | -0.81807 | 4965.656 | 63.57031 |  |  |  |  |  |
| 3.85 | | 73.0115 | -0.82001 | 4965.141 | 63.32813 |  |  |  |  |  |
| 3.858333 | | 73.07703 | -0.8215 | 4964.602 | 63.03125 |  |  |  |  |  |
| 3.866667 | | 73.14892 | -0.82228 | 4964.086 | 62.88281 |  |  |  |  |  |
| 3.875 | | 73.21657 | -0.82399 | 4963.555 | 62.96875 |  |  |  |  |  |
| 3.883333 | | 73.28634 | -0.82597 | 4963.031 | 63.13281 |  |  |  |  |  |
| 3.891667 | | 73.35081 | -0.82753 | 4962.5 | 63.17188 |  |  |  |  |  |
| 3.9 | | 73.41847 | -0.82856 | 4962.008 | 63.21094 |  |  |  |  |  |
| 3.908333 | | 73.49247 | -0.83001 | 4961.508 | 63.10156 |  |  |  |  |  |
| 3.916667 | | 73.56541 | -0.8309 | 4961 | 63.08594 |  |  |  |  |  |
| 3.925 | | 73.63306 | -0.83116 | 4960.461 | 63.3125 |  |  |  |  |  |
| 3.933333 | | 73.70388 | -0.83267 | 4959.906 | 63.48438 |  |  |  |  |  |
| 3.941667 | | 73.77894 | -0.83357 | 4959.375 | 63.47656 |  |  |  |  |  |
| 3.95 | | 73.85188 | -0.83388 | 4958.844 | 63.58594 |  |  |  |  |  |
| 3.958333 | | 73.92165 | -0.83551 | 4958.336 | 63.65625 |  |  |  |  |  |
| 3.966667 | | 73.99141 | -0.83698 | 4957.813 | 63.60938 |  |  |  |  |  |
| 3.975 | | 74.06435 | -0.8377 | 4957.258 | 63.52344 |  |  |  |  |  |
| 3.983333 | | 74.13623 | -0.83847 | 4956.719 | 63.60938 |  |  |  |  |  |
| 3.991667 | | 74.20389 | -0.84037 | 4956.18 | 63.71875 |  |  |  |  |  |
| 4 | | 74.27471 | -0.84111 | 4955.648 | 64.125 |  |  |  |  |  |
| 4.008333 | | 74.3487 | -0.84193 | 4955.117 | 64.15625 |  |  |  |  |  |
| 4.016667 | | 74.42059 | -0.84383 | 4954.594 | 64.08594 |  |  |  |  |  |
| 4.025 | | 74.49352 | -0.84497 | 4954.086 | 64.22656 |  |  |  |  |  |
| 4.033333 | | 74.56223 | -0.84596 | 4953.547 | 64.26563 |  |  |  |  |  |
| 4.041667 | | 74.63518 | -0.84749 | 4952.992 | 64.39844 |  |  |  |  |  |
| 4.05 | | 74.70494 | -0.84914 | 4952.406 | 64.46875 |  |  |  |  |  |
| 4.058333 | | 74.77682 | -0.84986 | 4951.875 | 64.54688 |  |  |  |  |  |
| 4.066667 | | 74.84765 | -0.85082 | 4951.328 | 64.65625 |  |  |  |  |  |
| 4.075 | | 74.91847 | -0.85198 | 4950.781 | 64.69531 |  |  |  |  |  |
| 4.083333 | | 74.98718 | -0.85243 | 4950.242 | 64.375 |  |  |  |  |  |
| 4.091667 | | 75.05589 | -0.8529 | 4949.68 | 64.15625 |  |  |  |  |  |
| 4.1 | | 75.12672 | -0.85448 | 4949.133 | 64.1875 |  |  |  |  |  |
| 4.108333 | | 75.19966 | -0.855 | 4948.578 | 64.03906 |  |  |  |  |  |
| 4.116667 | | 75.27048 | -0.85516 | 4948.031 | 63.89063 |  |  |  |  |  |
| 4.125 | | 75.33813 | -0.85677 | 4947.492 | 63.5625 |  |  |  |  |  |
| 4.133333 | | 75.41213 | -0.85745 | 4947 | 63.28906 |  |  |  |  |  |
| 4.141667 | | 75.48824 | -0.85754 | 4946.492 | 63.23438 |  |  |  |  |  |
| 4.15 | | 75.55907 | -0.85855 | 4945.953 | 63.125 |  |  |  |  |  |
| 4.158333 | | 75.63095 | -0.85966 | 4945.445 | 62.94531 |  |  |  |  |  |
| 4.166667 | | 75.70389 | -0.86024 | 4944.922 | 62.90625 |  |  |  |  |  |
| 4.175 | | 75.78 | -0.86121 | 4944.445 | 62.82813 |  |  |  |  |  |
| 4.183333 | | 75.854 | -0.86257 | 4943.953 | 62.55469 |  |  |  |  |  |
| 4.191667 | | 75.92271 | -0.86325 | 4943.43 | 62.27344 |  |  |  |  |  |
| 4.2 | | 75.99776 | -0.86371 | 4942.922 | 61.98438 |  |  |  |  |  |
| 4.208333 | | 76.07703 | -0.86515 | 4942.422 | 61.80469 |  |  |  |  |  |
| 4.216667 | | 76.15103 | -0.86557 | 4941.906 | 61.88281 |  |  |  |  |  |
| 4.225 | | 76.21974 | -0.86601 | 4941.391 | 61.74219 |  |  |  |  |  |
| 4.233333 | | 76.2948 | -0.8668 | 4940.906 | 61.3125 |  |  |  |  |  |
| 4.241667 | | 76.36985 | -0.86666 | 4940.43 | 61.33594 |  |  |  |  |  |
| 4.25 | | 76.44596 | -0.86658 | 4939.953 | 61.10938 |  |  |  |  |  |
| 4.258333 | | 76.51678 | -0.86773 | 4939.469 | 60.86719 |  |  |  |  |  |
| 4.266667 | | 76.59184 | -0.86804 | 4938.938 | 60.61719 |  |  |  |  |  |
| 4.275 | | 76.66372 | -0.86822 | 4938.445 | 60.85156 |  |  |  |  |  |
| 4.283333 | | 76.73666 | -0.86905 | 4937.984 | 60.69531 |  |  |  |  |  |
| 4.291667 | | 76.80854 | -0.87039 | 4937.469 | 60.86719 |  |  |  |  |  |
| 4.3 | | 76.88359 | -0.87075 | 4936.992 | 60.65625 |  |  |  |  |  |
| 4.308333 | | 76.95759 | -0.87172 | 4936.523 | 60.35156 |  |  |  |  |  |
| 4.316667 | | 77.02841 | -0.87352 | 4936.047 | 60.39844 |  |  |  |  |  |
| 4.325 | | 77.10346 | -0.87411 | 4935.5 | 60.28906 |  |  |  |  |  |
| 4.333333 | | 77.17746 | -0.87467 | 4935.016 | 60.35938 |  |  |  |  |  |
| 4.341667 | | 77.25146 | -0.87562 | 4934.477 | 60.39844 |  |  |  |  |  |
| 4.35 | | 77.32334 | -0.8762 | 4934.008 | 60.60938 |  |  |  |  |  |
| 4.358333 | | 77.40051 | -0.87645 | 4933.531 | 60.48438 |  |  |  |  |  |
| 4.366667 | | 77.47556 | -0.87743 | 4933.031 | 60.82031 |  |  |  |  |  |
| 4.375 | | 77.5485 | -0.87786 | 4932.531 | 60.72656 |  |  |  |  |  |
| 4.383333 | | 77.61721 | -0.87791 | 4932.016 | 60.88281 |  |  |  |  |  |
| 4.391667 | | 77.69543 | -0.87781 | 4931.492 | 61.25 |  |  |  |  |  |
| 4.4 | | 77.77155 | -0.87836 | 4930.953 | 61.46875 |  |  |  |  |  |
| 4.408333 | | 77.8466 | -0.87831 | 4930.469 | 61.46094 |  |  |  |  |  |
| 4.416667 | | 77.92059 | -0.879 | 4929.914 | 61.63281 |  |  |  |  |  |
| 4.425 | | 77.99459 | -0.8805 | 4929.414 | 61.85156 |  |  |  |  |  |
| 4.433333 | | 78.07069 | -0.88073 | 4928.875 | 61.97656 |  |  |  |  |  |
| 4.441667 | | 78.1468 | -0.88127 | 4928.313 | 62.32031 |  |  |  |  |  |
| 4.45 | | 78.22186 | -0.8826 | 4927.758 | 62.24219 |  |  |  |  |  |
| 4.458333 | | 78.29903 | -0.8827 | 4927.242 | 62.32031 |  |  |  |  |  |
| 4.466667 | | 78.37408 | -0.88357 | 4926.703 | 62.5625 |  |  |  |  |  |
| 4.475 | | 78.44385 | -0.88538 | 4926.148 | 62.55469 |  |  |  |  |  |
| 4.483333 | | 78.52101 | -0.88572 | 4925.609 | 62.54688 |  |  |  |  |  |
| 4.491667 | | 78.59396 | -0.88598 | 4925.055 | 62.61719 |  |  |  |  |  |
| 4.5 | | 78.66689 | -0.88676 | 4924.539 | 62.58594 |  |  |  |  |  |
| 4.508333 | | 78.74723 | -0.88683 | 4924.016 | 62.45313 |  |  |  |  |  |
| 4.516667 | | 78.82439 | -0.88675 | 4923.461 | 62.53125 |  |  |  |  |  |
| 4.525 | | 78.89839 | -0.88755 | 4922.945 | 62.75 |  |  |  |  |  |
| 4.533333 | | 78.97028 | -0.88845 | 4922.422 | 62.66406 |  |  |  |  |  |
| 4.541667 | | 79.04744 | -0.88778 | 4921.891 | 62.63281 |  |  |  |  |  |
| 4.55 | | 79.12672 | -0.88802 | 4921.375 | 62.42188 |  |  |  |  |  |
| 4.558333 | | 79.20389 | -0.88844 | 4920.875 | 62.21875 |  |  |  |  |  |
| 4.566667 | | 79.27789 | -0.88819 | 4920.344 | 62.20313 |  |  |  |  |  |
| 4.575 | | 79.35294 | -0.88894 | 4919.797 | 62.11719 |  |  |  |  |  |
| 4.583333 | | 79.43117 | -0.88996 | 4919.289 | 62.08594 |  |  |  |  |  |
| 4.591667 | | 79.50516 | -0.88986 | 4918.773 | 62.125 |  |  |  |  |  |
| 4.6 | | 79.58444 | -0.88996 | 4918.297 | 62.20313 |  |  |  |  |  |
| 4.608333 | | 79.66267 | -0.89057 | 4917.805 | 61.97656 |  |  |  |  |  |
| 4.616667 | | 79.74194 | -0.8901 | 4917.289 | 61.89063 |  |  |  |  |  |
| 4.625 | | 79.81594 | -0.89033 | 4916.789 | 61.875 |  |  |  |  |  |
| 4.633333 | | 79.89205 | -0.8909 | 4916.281 | 62.11719 |  |  |  |  |  |
| 4.641667 | | 79.97028 | -0.8906 | 4915.75 | 62.34375 |  |  |  |  |  |
| 4.65 | | 80.04639 | -0.89022 | 4915.227 | 62.22656 |  |  |  |  |  |
| 4.658333 | | 80.12461 | -0.8904 | 4914.758 | 62.28906 |  |  |  |  |  |
| 4.666667 | | 80.20284 | -0.88984 | 4914.25 | 62.29688 |  |  |  |  |  |
| 4.675 | | 80.28 | -0.8893 | 4913.734 | 62.21094 |  |  |  |  |  |
| 4.683333 | | 80.35294 | -0.88962 | 4913.188 | 61.75 |  |  |  |  |  |
| 4.691667 | | 80.42693 | -0.88978 | 4912.633 | 61.75 |  |  |  |  |  |
| 4.7 | | 80.50516 | -0.88927 | 4912.133 | 61.74219 |  |  |  |  |  |
| 4.708333 | | 80.58233 | -0.88975 | 4911.617 | 61.66406 |  |  |  |  |  |
| 4.716667 | | 80.65949 | -0.89001 | 4911.094 | 61.28906 |  |  |  |  |  |
| 4.725 | | 80.73666 | -0.88938 | 4910.594 | 61.21875 |  |  |  |  |  |
| 4.733333 | | 80.81489 | -0.88981 | 4910.141 | 61.20313 |  |  |  |  |  |
| 4.741667 | | 80.891 | -0.8904 | 4909.633 | 61.35938 |  |  |  |  |  |
| 4.75 | | 80.965 | -0.88983 | 4909.109 | 61.16406 |  |  |  |  |  |
| 4.758333 | | 81.03899 | -0.89038 | 4908.609 | 60.85156 |  |  |  |  |  |
| 4.766667 | | 81.11404 | -0.89127 | 4908.148 | 61.07031 |  |  |  |  |  |
| 4.775 | | 81.19121 | -0.89093 | 4907.648 | 61.05469 |  |  |  |  |  |
| 4.783333 | | 81.26521 | -0.89141 | 4907.133 | 61.10156 |  |  |  |  |  |
| 4.791667 | | 81.3392 | -0.89202 | 4906.594 | 60.96094 |  |  |  |  |  |
| 4.8 | | 81.41319 | -0.89176 | 4906.109 | 60.89844 |  |  |  |  |  |
| 4.808333 | | 81.48825 | -0.89151 | 4905.641 | 60.89844 |  |  |  |  |  |
| 4.816667 | | 81.56541 | -0.89177 | 4905.109 | 60.60156 |  |  |  |  |  |
| 4.825 | | 81.64575 | -0.89125 | 4904.594 | 60.27344 |  |  |  |  |  |
| 4.833333 | | 81.72504 | -0.89066 | 4904.07 | 60.10156 |  |  |  |  |  |
| 4.841667 | | 81.80431 | -0.89112 | 4903.578 | 60.3125 |  |  |  |  |  |
| 4.85 | | 81.88033 | -0.89113 | 4903.07 | 60.28906 |  |  |  |  |  |
| 4.858333 | | 81.95453 | -0.89031 | 4902.57 | 60.11719 |  |  |  |  |  |
| 4.866667 | | 82.03182 | -0.89028 | 4902.102 | 60.15625 |  |  |  |  |  |
| 4.875 | | 82.10912 | -0.88985 | 4901.641 | 60.22656 |  |  |  |  |  |
| 4.883333 | | 82.18641 | -0.88898 | 4901.156 | 60.32813 |  |  |  |  |  |
| 4.891667 | | 82.26577 | -0.88918 | 4900.633 | 60.3125 |  |  |  |  |  |
| 4.9 | | 82.33997 | -0.88945 | 4900.133 | 60.58594 |  |  |  |  |  |
| 4.908333 | | 82.41521 | -0.88868 | 4899.648 | 60.70313 |  |  |  |  |  |
| 4.916667 | | 82.49044 | -0.88897 | 4899.133 | 60.70313 |  |  |  |  |  |
| 4.925 | | 82.56464 | -0.88898 | 4898.625 | 60.80469 |  |  |  |  |  |
| 4.933333 | | 82.64194 | -0.88801 | 4898.102 | 60.82031 |  |  |  |  |  |
| 4.941667 | | 82.71821 | -0.88849 | 4897.602 | 61.04688 |  |  |  |  |  |
| 4.95 | | 82.79653 | -0.88855 | 4897.055 | 60.88281 |  |  |  |  |  |
| 4.958333 | | 82.87486 | -0.88764 | 4896.539 | 61 |  |  |  |  |  |
| 4.966667 | | 82.95319 | -0.88753 | 4896.023 | 60.85938 |  |  |  |  |  |
| 4.975 | | 83.02842 | -0.88763 | 4895.508 | 61.10938 |  |  |  |  |  |
| 4.983333 | | 83.10674 | -0.88692 | 4895 | 60.875 |  |  |  |  |  |
| 4.991667 | | 83.18198 | -0.88696 | 4894.461 | 60.73438 |  |  |  |  |  |
| 5 | | 83.25824 | -0.88729 | 4893.977 | 60.76563 |  |  |  |  |  |
| 5.008333 | | 83.33451 | -0.88701 | 4893.445 | 60.53125 |  |  |  |  |  |
| 5.016667 | | 83.4118 | -0.88679 | 4892.961 | 60.39063 |  |  |  |  |  |
| 5.025 | | 83.49219 | -0.8869 | 4892.422 | 59.92188 |  |  |  |  |  |
| 5.033333 | | 83.56536 | -0.88677 | 4891.945 | 59.77344 |  |  |  |  |  |
| 5.041667 | | 83.6406 | -0.88679 | 4891.445 | 59.49219 |  |  |  |  |  |
| 5.05 | | 83.71893 | -0.88723 | 4890.938 | 59.22656 |  |  |  |  |  |
| 5.058333 | | 83.79622 | -0.88706 | 4890.453 | 58.69531 |  |  |  |  |  |
| 5.066667 | | 83.87351 | -0.88629 | 4889.984 | 58.625 |  |  |  |  |  |
| 5.075 | | 83.94978 | -0.88643 | 4889.539 | 58.52344 |  |  |  |  |  |
| 5.083333 | | 84.02502 | -0.88639 | 4889.07 | 58.375 |  |  |  |  |  |
| 5.091667 | | 84.10128 | -0.8851 | 4888.617 | 58.17969 |  |  |  |  |  |
| 5.1 | | 84.1796 | -0.88564 | 4888.156 | 58.23438 |  |  |  |  |  |
| 5.108333 | | 84.2569 | -0.88544 | 4887.742 | 58.29688 |  |  |  |  |  |
| 5.116667 | | 84.33729 | -0.88434 | 4887.266 | 58.05469 |  |  |  |  |  |
| 5.125 | | 84.41768 | -0.88439 | 4886.789 | 57.67188 |  |  |  |  |  |
| 5.133333 | | 84.49394 | -0.88411 | 4886.328 | 57.42188 |  |  |  |  |  |
| 5.141667 | | 84.57227 | -0.88256 | 4885.859 | 57.49219 |  |  |  |  |  |
| 5.15 | | 84.6475 | -0.88241 | 4885.367 | 57.23438 |  |  |  |  |  |
| 5.158333 | | 84.72376 | -0.88202 | 4884.875 | 56.89063 |  |  |  |  |  |
| 5.166667 | | 84.80312 | -0.88085 | 4884.422 | 56.78906 |  |  |  |  |  |
| 5.175 | | 84.88248 | -0.88083 | 4883.984 | 56.74219 |  |  |  |  |  |
| 5.183333 | | 84.95978 | -0.88042 | 4883.539 | 56.66406 |  |  |  |  |  |
| 5.191667 | | 85.0381 | -0.87901 | 4883.047 | 56.46094 |  |  |  |  |  |
| 5.2 | | 85.11642 | -0.87844 | 4882.609 | 56.60156 |  |  |  |  |  |
| 5.208333 | | 85.19476 | -0.87802 | 4882.18 | 56.88281 |  |  |  |  |  |
| 5.216667 | | 85.27308 | -0.87715 | 4881.719 | 57.19531 |  |  |  |  |  |
| 5.225 | | 85.3514 | -0.87732 | 4881.25 | 57.28125 |  |  |  |  |  |
| 5.233333 | | 85.43385 | -0.8774 | 4880.797 | 57.32813 |  |  |  |  |  |
| 5.241667 | | 85.51424 | -0.87654 | 4880.336 | 57.59375 |  |  |  |  |  |
| 5.25 | | 85.59566 | -0.87618 | 4879.852 | 57.60156 |  |  |  |  |  |
| 5.258333 | | 85.67914 | -0.87562 | 4879.328 | 57.78906 |  |  |  |  |  |
| 5.266667 | | 85.76056 | -0.87458 | 4878.805 | 57.64844 |  |  |  |  |  |
| 5.275 | | 85.83888 | -0.87451 | 4878.313 | 57.5 |  |  |  |  |  |
| 5.283333 | | 85.91618 | -0.87434 | 4877.82 | 57.61719 |  |  |  |  |  |
| 5.291667 | | 85.99451 | -0.8735 | 4877.297 | 57.79688 |  |  |  |  |  |
| 5.3 | | 86.07489 | -0.87277 | 4876.805 | 57.78125 |  |  |  |  |  |
| 5.308333 | | 86.15218 | -0.87269 | 4876.297 | 57.51563 |  |  |  |  |  |
| 5.316667 | | 86.22948 | -0.8718 | 4875.836 | 57.47656 |  |  |  |  |  |
| 5.325 | | 86.30884 | -0.871 | 4875.367 | 57.46875 |  |  |  |  |  |
| 5.333333 | | 86.38613 | -0.87098 | 4874.875 | 57.64844 |  |  |  |  |  |
| 5.341667 | | 86.46343 | -0.86977 | 4874.359 | 57.50781 |  |  |  |  |  |
| 5.35 | | 86.54176 | -0.86855 | 4873.883 | 57.78125 |  |  |  |  |  |
| 5.358333 | | 86.62008 | -0.86847 | 4873.43 | 57.89844 |  |  |  |  |  |
| 5.366667 | | 86.70047 | -0.86758 | 4872.953 | 58.16406 |  |  |  |  |  |
| 5.375 | | 86.77879 | -0.8665 | 4872.469 | 58.1875 |  |  |  |  |  |
| 5.383333 | | 86.85609 | -0.86669 | 4871.969 | 58.28125 |  |  |  |  |  |
| 5.391667 | | 86.93648 | -0.86551 | 4871.5 | 58.49219 |  |  |  |  |  |
| 5.4 | | 87.01584 | -0.86431 | 4870.977 | 58.75 |  |  |  |  |  |
| 5.408333 | | 87.0921 | -0.8648 | 4870.477 | 58.89844 |  |  |  |  |  |
| 5.416667 | | 87.16837 | -0.86433 | 4869.953 | 58.77344 |  |  |  |  |  |
| 5.425 | | 87.2436 | -0.86362 | 4869.477 | 58.96094 |  |  |  |  |  |
| 5.433333 | | 87.31883 | -0.86389 | 4868.969 | 58.60938 |  |  |  |  |  |
| 5.441667 | | 87.39922 | -0.86315 | 4868.461 | 58.41406 |  |  |  |  |  |
| 5.45 | | 87.47961 | -0.86177 | 4867.938 | 58.11719 |  |  |  |  |  |
| 5.458333 | | 87.5569 | -0.86163 | 4867.438 | 57.69531 |  |  |  |  |  |
| 5.466667 | | 87.63523 | -0.86047 | 4866.961 | 57.36719 |  |  |  |  |  |
| 5.475 | | 87.71561 | -0.85893 | 4866.461 | 57.20313 |  |  |  |  |  |
| 5.483333 | | 87.79601 | -0.85841 | 4866.008 | 57.10156 |  |  |  |  |  |
| 5.491667 | | 87.87742 | -0.8576 | 4865.555 | 56.5625 |  |  |  |  |  |
| 5.5 | | 87.9609 | -0.85613 | 4865.117 | 56.53125 |  |  |  |  |  |
| 5.508333 | | 88.04541 | -0.85569 | 4864.695 | 56.25 |  |  |  |  |  |
| 5.516667 | | 88.12786 | -0.85472 | 4864.266 | 56.04688 |  |  |  |  |  |
| 5.525 | | 88.20721 | -0.85343 | 4863.813 | 55.98438 |  |  |  |  |  |
| 5.533333 | | 88.28452 | -0.85324 | 4863.352 | 55.75 |  |  |  |  |  |
| 5.541667 | | 88.3649 | -0.85237 | 4862.961 | 55.66406 |  |  |  |  |  |
| 5.55 | | 88.44838 | -0.85105 | 4862.492 | 55.64844 |  |  |  |  |  |
| 5.558333 | | 88.52567 | -0.85104 | 4862.078 | 55.67188 |  |  |  |  |  |
| 5.566667 | | 88.60606 | -0.85037 | 4861.633 | 55.3125 |  |  |  |  |  |
| 5.575 | | 88.68954 | -0.8489 | 4861.18 | 55.46875 |  |  |  |  |  |
| 5.583333 | | 88.76993 | -0.84852 | 4860.75 | 55.42188 |  |  |  |  |  |
| 5.591667 | | 88.84619 | -0.84788 | 4860.297 | 55.49219 |  |  |  |  |  |
| 5.6 | | 88.92451 | -0.84684 | 4859.82 | 55.25781 |  |  |  |  |  |
| 5.608333 | | 89.00285 | -0.84674 | 4859.367 | 55.20313 |  |  |  |  |  |
| 5.616667 | | 89.08323 | -0.8461 | 4858.938 | 55.07813 |  |  |  |  |  |
| 5.625 | | 89.16774 | -0.84466 | 4858.445 | 55.16406 |  |  |  |  |  |
| 5.633333 | | 89.24607 | -0.84441 | 4857.984 | 54.83594 |  |  |  |  |  |
| 5.641667 | | 89.32748 | -0.84368 | 4857.508 | 54.47656 |  |  |  |  |  |
| 5.65 | | 89.40685 | -0.84223 | 4857.078 | 54.35156 |  |  |  |  |  |
| 5.658333 | | 89.48723 | -0.8415 | 4856.625 | 54.24219 |  |  |  |  |  |
| 5.666667 | | 89.56762 | -0.84065 | 4856.188 | 53.82031 |  |  |  |  |  |
| 5.675 | | 89.65213 | -0.83926 | 4855.711 | 53.75 |  |  |  |  |  |
| 5.683333 | | 89.7387 | -0.83853 | 4855.297 | 53.67969 |  |  |  |  |  |
| 5.691667 | | 89.8263 | -0.83727 | 4854.875 | 53.50781 |  |  |  |  |  |
| 5.7 | | 89.90565 | -0.83598 | 4854.453 | 53.58594 |  |  |  |  |  |
| 5.708333 | | 89.98502 | -0.83549 | 4854.008 | 53.38281 |  |  |  |  |  |
| 5.716667 | | 90.07056 | -0.83466 | 4853.609 | 53.21875 |  |  |  |  |  |
| 5.725 | | 90.15197 | -0.8334 | 4853.18 | 52.98438 |  |  |  |  |  |
| 5.733333 | | 90.23339 | -0.83313 | 4852.734 | 52.85156 |  |  |  |  |  |
| 5.741667 | | 90.31583 | -0.83248 | 4852.305 | 52.625 |  |  |  |  |  |
| 5.75 | | 90.39622 | -0.8313 | 4851.836 | 52.35156 |  |  |  |  |  |
| 5.758333 | | 90.47558 | -0.83054 | 4851.414 | 52.29688 |  |  |  |  |  |
| 5.766667 | | 90.55494 | -0.82976 | 4850.984 | 52.15625 |  |  |  |  |  |
| 5.775 | | 90.63223 | -0.82823 | 4850.57 | 52.36719 |  |  |  |  |  |
| 5.783333 | | 90.71674 | -0.82751 | 4850.141 | 52.28125 |  |  |  |  |  |
| 5.791667 | | 90.79919 | -0.82704 | 4849.727 | 52.17969 |  |  |  |  |  |
| 5.8 | | 90.87649 | -0.82539 | 4849.32 | 52.26563 |  |  |  |  |  |
| 5.808333 | | 90.95894 | -0.82406 | 4848.891 | 52.50781 |  |  |  |  |  |
| 5.816667 | | 91.04035 | -0.82323 | 4848.461 | 52.92188 |  |  |  |  |  |
| 5.825 | | 91.11765 | -0.82102 | 4847.992 | 53.02344 |  |  |  |  |  |
| 5.833333 | | 91.2001 | -0.81958 | 4847.563 | 52.96875 |  |  |  |  |  |
| 5.841667 | | 91.28358 | -0.81893 | 4847.141 | 53.21875 |  |  |  |  |  |
| 5.85 | | 91.36603 | -0.81687 | 4846.695 | 53.39063 |  |  |  |  |  |
| 5.858333 | | 91.45157 | -0.81548 | 4846.219 | 53.25781 |  |  |  |  |  |
| 5.866667 | | 91.53504 | -0.81462 | 4845.727 | 53.14063 |  |  |  |  |  |
| 5.875 | | 91.61646 | -0.81292 | 4845.266 | 53.35156 |  |  |  |  |  |
| 5.883333 | | 91.69788 | -0.81227 | 4844.828 | 53.58594 |  |  |  |  |  |
| 5.891667 | | 91.78239 | -0.81218 | 4844.352 | 53.57813 |  |  |  |  |  |
| 5.9 | | 91.8669 | -0.81019 | 4843.891 | 53.35156 |  |  |  |  |  |
| 5.908333 | | 91.95141 | -0.80936 | 4843.453 | 53.16406 |  |  |  |  |  |
| 5.916667 | | 92.03283 | -0.80949 | 4843.039 | 53.4375 |  |  |  |  |  |
| 5.925 | | 92.11631 | -0.80749 | 4842.555 | 53.23438 |  |  |  |  |  |
| 5.933333 | | 92.19875 | -0.8066 | 4842.086 | 53.20313 |  |  |  |  |  |
| 5.941667 | | 92.27708 | -0.80633 | 4841.633 | 53.16406 |  |  |  |  |  |
| 5.95 | | 92.35953 | -0.80409 | 4841.227 | 53.19531 |  |  |  |  |  |
| 5.958333 | | 92.44404 | -0.8025 | 4840.797 | 53.03906 |  |  |  |  |  |
| 5.966667 | | 92.52855 | -0.80114 | 4840.32 | 52.98438 |  |  |  |  |  |
| 5.975 | | 92.60997 | -0.79888 | 4839.898 | 52.71094 |  |  |  |  |  |
| 5.983333 | | 92.69241 | -0.79797 | 4839.453 | 52.69531 |  |  |  |  |  |
| 5.991667 | | 92.77589 | -0.79724 | 4839.016 | 52.64844 |  |  |  |  |  |
| 6 | | 92.86143 | -0.79474 | 4838.57 | 52.63281 |  |  |  |  |  |
| 6.008333 | | 92.94183 | -0.79389 | 4838.148 | 52.42188 |  |  |  |  |  |
| 6.016667 | | 93.02221 | -0.79327 | 4837.719 | 51.97656 |  |  |  |  |  |
| 6.025 | | 93.10672 | -0.79131 | 4837.313 | 51.54688 |  |  |  |  |  |
| 6.033333 | | 93.18838 | -0.79056 | 4836.875 | 51.375 |  |  |  |  |  |
| 6.041667 | | 93.26701 | -0.78996 | 4836.453 | 51.0625 |  |  |  |  |  |
| 6.05 | | 93.34364 | -0.78774 | 4836.008 | 50.55469 |  |  |  |  |  |
| 6.058333 | | 93.42426 | -0.78675 | 4835.609 | 50.32813 |  |  |  |  |  |
| 6.066667 | | 93.50289 | -0.78587 | 4835.234 | 50.27344 |  |  |  |  |  |
| 6.075 | | 93.58153 | -0.78352 | 4834.875 | 50.05469 |  |  |  |  |  |
| 6.083333 | | 93.65916 | -0.78262 | 4834.469 | 49.77344 |  |  |  |  |  |
| 6.091667 | | 93.73978 | -0.78195 | 4834.078 | 49.71875 |  |  |  |  |  |
| 6.1 | | 93.8204 | -0.78001 | 4833.727 | 50.00781 |  |  |  |  |  |
| 6.108333 | | 93.89704 | -0.77911 | 4833.336 | 50.22656 |  |  |  |  |  |
| 6.116667 | | 93.97667 | -0.7783 | 4832.93 | 50.22656 |  |  |  |  |  |
| 6.125 | | 94.05331 | -0.77668 | 4832.539 | 50.02344 |  |  |  |  |  |
| 6.133333 | | 94.13393 | -0.77565 | 4832.172 | 50.20313 |  |  |  |  |  |
| 6.141667 | | 94.21056 | -0.77513 | 4831.758 | 50.125 |  |  |  |  |  |
| 6.15 | | 94.2882 | -0.77318 | 4831.297 | 49.76563 |  |  |  |  |  |
| 6.158333 | | 94.36882 | -0.77183 | 4830.852 | 49.76563 |  |  |  |  |  |
| 6.166667 | | 94.44845 | -0.77087 | 4830.438 | 49.625 |  |  |  |  |  |
| 6.175 | | 94.53006 | -0.76866 | 4830.039 | 49.30469 |  |  |  |  |  |
| 6.183333 | | 94.60969 | -0.76745 | 4829.586 | 49.22656 |  |  |  |  |  |
| 6.191667 | | 94.69031 | -0.76628 | 4829.188 | 49.08594 |  |  |  |  |  |
| 6.2 | | 94.76994 | -0.76412 | 4828.82 | 49.17188 |  |  |  |  |  |
| 6.208333 | | 94.85255 | -0.76258 | 4828.398 | 49.4375 |  |  |  |  |  |
| 6.216667 | | 94.93416 | -0.76137 | 4827.984 | 49.375 |  |  |  |  |  |
| 6.225 | | 95.01279 | -0.7592 | 4827.617 | 49.48438 |  |  |  |  |  |
| 6.233333 | | 95.09541 | -0.75793 | 4827.219 | 49.60156 |  |  |  |  |  |
| 6.241667 | | 95.17802 | -0.75695 | 4826.82 | 49.85938 |  |  |  |  |  |
| 6.25 | | 95.26063 | -0.75518 | 4826.391 | 49.85156 |  |  |  |  |  |
| 6.258333 | | 95.34025 | -0.75383 | 4825.938 | 50.11719 |  |  |  |  |  |
| 6.266667 | | 95.41888 | -0.75277 | 4825.531 | 50.03906 |  |  |  |  |  |
| 6.275 | | 95.4995 | -0.75091 | 4825.102 | 50.0625 |  |  |  |  |  |
| 6.283333 | | 95.57813 | -0.74947 | 4824.656 | 50.0625 |  |  |  |  |  |
| 6.291667 | | 95.65776 | -0.74837 | 4824.203 | 49.95313 |  |  |  |  |  |
| 6.3 | | 95.74037 | -0.74631 | 4823.797 | 49.99219 |  |  |  |  |  |
| 6.308333 | | 95.82099 | -0.74498 | 4823.328 | 50 |  |  |  |  |  |
| 6.316667 | | 95.90062 | -0.74429 | 4822.922 | 49.97656 |  |  |  |  |  |
| 6.325 | | 95.97925 | -0.74298 | 4822.5 | 49.65625 |  |  |  |  |  |
| 6.333333 | | 96.05987 | -0.74148 | 4822.078 | 49.78906 |  |  |  |  |  |
| 6.341667 | | 96.14148 | -0.74058 | 4821.672 | 49.78125 |  |  |  |  |  |
| 6.35 | | 96.22509 | -0.73889 | 4821.25 | 49.54688 |  |  |  |  |  |
| 6.358333 | | 96.31268 | -0.73749 | 4820.828 | 49.45313 |  |  |  |  |  |
| 6.366667 | | 96.39629 | -0.73673 | 4820.414 | 49.21094 |  |  |  |  |  |
| 6.375 | | 96.47691 | -0.73536 | 4820.047 | 48.9375 |  |  |  |  |  |
| 6.383333 | | 96.55553 | -0.73435 | 4819.609 | 49.08594 |  |  |  |  |  |
| 6.391667 | | 96.63914 | -0.73351 | 4819.195 | 48.76563 |  |  |  |  |  |
| 6.4 | | 96.72375 | -0.73144 | 4818.813 | 48.46094 |  |  |  |  |  |
| 6.408333 | | 96.80536 | -0.72976 | 4818.422 | 48.28906 |  |  |  |  |  |
| 6.416667 | | 96.88698 | -0.72866 | 4818.047 | 48.01563 |  |  |  |  |  |
| 6.425 | | 96.96859 | -0.7267 | 4817.68 | 47.53906 |  |  |  |  |  |
| 6.433333 | | 97.04822 | -0.72524 | 4817.25 | 47.28125 |  |  |  |  |  |
| 6.441667 | | 97.12585 | -0.72431 | 4816.898 | 47.27344 |  |  |  |  |  |
| 6.45 | | 97.21046 | -0.72204 | 4816.539 | 46.92188 |  |  |  |  |  |
| 6.458333 | | 97.29605 | -0.7201 | 4816.156 | 46.69531 |  |  |  |  |  |
| 6.466667 | | 97.37966 | -0.71895 | 4815.797 | 46.30469 |  |  |  |  |  |
| 6.475 | | 97.46127 | -0.71709 | 4815.477 | 46.60938 |  |  |  |  |  |
| 6.483333 | | 97.54189 | -0.71579 | 4815.109 | 46.72656 |  |  |  |  |  |
| 6.491667 | | 97.62351 | -0.71503 | 4814.727 | 46.89844 |  |  |  |  |  |
| 6.5 | | 97.70512 | -0.71319 | 4814.375 | 46.89844 |  |  |  |  |  |
| 6.508333 | | 97.78575 | -0.71188 | 4814.016 | 47.16406 |  |  |  |  |  |
| 6.516667 | | 97.87135 | -0.7108 | 4813.672 | 47.46094 |  |  |  |  |  |
| 6.525 | | 97.95495 | -0.70876 | 4813.242 | 47.77344 |  |  |  |  |  |
| 6.533333 | | 98.03159 | -0.70778 | 4812.828 | 48.03906 |  |  |  |  |  |
| 6.541667 | | 98.11221 | -0.70697 | 4812.414 | 48.17188 |  |  |  |  |  |
| 6.55 | | 98.19382 | -0.70514 | 4812.023 | 48.125 |  |  |  |  |  |
| 6.558333 | | 98.27644 | -0.70357 | 4811.586 | 48.15625 |  |  |  |  |  |
| 6.566667 | | 98.36204 | -0.70224 | 4811.148 | 47.82031 |  |  |  |  |  |
| 6.575 | | 98.44366 | -0.70006 | 4810.711 | 47.61719 |  |  |  |  |  |
| 6.583333 | | 98.52726 | -0.6985 | 4810.273 | 47.52344 |  |  |  |  |  |
| 6.591667 | | 98.61186 | -0.69684 | 4809.852 | 47.14844 |  |  |  |  |  |
| 6.6 | | 98.69248 | -0.69458 | 4809.453 | 46.90625 |  |  |  |  |  |
| 6.608333 | | 98.77111 | -0.69348 | 4809.047 | 46.46875 |  |  |  |  |  |
| 6.616667 | | 98.85571 | -0.69223 | 4808.688 | 46.47656 |  |  |  |  |  |
| 6.625 | | 98.93633 | -0.69035 | 4808.32 | 46.28906 |  |  |  |  |  |
| 6.633333 | | 99.01795 | -0.68887 | 4807.938 | 46.0625 |  |  |  |  |  |
| 6.641667 | | 99.10056 | -0.68736 | 4807.578 | 45.79688 |  |  |  |  |  |
| 6.65 | | 99.18019 | -0.68526 | 4807.227 | 45.90625 |  |  |  |  |  |
| 6.658333 | | 99.26479 | -0.68392 | 4806.891 | 45.64844 |  |  |  |  |  |
| 6.666667 | | 99.34641 | -0.68292 | 4806.5 | 45.71875 |  |  |  |  |  |
| 6.675 | | 99.42802 | -0.68111 | 4806.133 | 45.71094 |  |  |  |  |  |
| 6.683333 | | 99.51064 | -0.68002 | 4805.773 | 45.57031 |  |  |  |  |  |
| 6.691667 | | 99.59822 | -0.67888 | 4805.438 | 45.79688 |  |  |  |  |  |
| 6.7 | | 99.68083 | -0.67679 | 4805.031 | 45.42188 |  |  |  |  |  |
| 6.708333 | | 99.76643 | -0.67537 | 4804.68 | 45.35156 |  |  |  |  |  |
| 6.716667 | | 99.85004 | -0.67409 | 4804.281 | 45.36719 |  |  |  |  |  |
| 6.725 | | 99.93464 | -0.67197 | 4803.914 | 45.375 |  |  |  |  |  |
| 6.733333 | | 100.0173 | -0.67094 | 4803.531 | 45.38281 |  |  |  |  |  |
| 6.741667 | | 100.0979 | -0.66933 | 4803.125 | 45.50781 |  |  |  |  |  |
| 6.75 | | 100.1795 | -0.66695 | 4802.789 | 45.38281 |  |  |  |  |  |
| 6.758333 | | 100.2591 | -0.66548 | 4802.422 | 45.375 |  |  |  |  |  |
| 6.766667 | | 100.3417 | -0.66346 | 4802.047 | 45.29688 |  |  |  |  |  |
| 6.775 | | 100.4204 | -0.66147 | 4801.656 | 45.02344 |  |  |  |  |  |
| 6.783333 | | 100.506 | -0.66017 | 4801.281 | 45.03906 |  |  |  |  |  |
| 6.791667 | | 100.5856 | -0.65891 | 4800.883 | 44.88281 |  |  |  |  |  |
| 6.8 | | 100.6692 | -0.65681 | 4800.531 | 44.99219 |  |  |  |  |  |
| 6.808333 | | 100.7498 | -0.65555 | 4800.156 | 45.03906 |  |  |  |  |  |
| 6.816667 | | 100.8314 | -0.65399 | 4799.789 | 45.02344 |  |  |  |  |  |
| 6.825 | | 100.915 | -0.65148 | 4799.461 | 44.98438 |  |  |  |  |  |
| 6.833333 | | 100.9976 | -0.65014 | 4799.086 | 44.73438 |  |  |  |  |  |
| 6.841667 | | 101.0832 | -0.64867 | 4798.727 | 44.73438 |  |  |  |  |  |
| 6.85 | | 101.1668 | -0.6466 | 4798.328 | 44.72656 |  |  |  |  |  |
| 6.858333 | | 101.2495 | -0.64547 | 4797.953 | 44.60938 |  |  |  |  |  |
| 6.866667 | | 101.3281 | -0.64409 | 4797.586 | 44.14844 |  |  |  |  |  |
| 6.875 | | 101.4127 | -0.64165 | 4797.219 | 43.9375 |  |  |  |  |  |
| 6.883333 | | 101.4943 | -0.64026 | 4796.875 | 43.60156 |  |  |  |  |  |
| 6.891667 | | 101.5779 | -0.6385 | 4796.492 | 43.03906 |  |  |  |  |  |
| 6.9 | | 101.6635 | -0.63595 | 4796.117 | 42.6875 |  |  |  |  |  |
| 6.908333 | | 101.7471 | -0.63461 | 4795.758 | 42.57031 |  |  |  |  |  |
| 6.916667 | | 101.8307 | -0.63277 | 4795.445 | 42.42188 |  |  |  |  |  |
| 6.925 | | 101.9123 | -0.63035 | 4795.094 | 42.14063 |  |  |  |  |  |
| 6.933333 | | 101.9949 | -0.62893 | 4794.781 | 41.83594 |  |  |  |  |  |
| 6.941667 | | 102.0805 | -0.62668 | 4794.492 | 41.59375 |  |  |  |  |  |
| 6.95 | | 102.1651 | -0.62435 | 4794.188 | 41.6875 |  |  |  |  |  |
| 6.958333 | | 102.2497 | -0.62315 | 4793.836 | 41.67969 |  |  |  |  |  |
| 6.966667 | | 102.3334 | -0.62155 | 4793.5 | 41.63281 |  |  |  |  |  |
| 6.975 | | 102.413 | -0.61997 | 4793.18 | 41.79688 |  |  |  |  |  |
| 6.983333 | | 102.4936 | -0.61938 | 4792.883 | 41.88281 |  |  |  |  |  |
| 6.991667 | | 102.5732 | -0.61842 | 4792.563 | 41.64063 |  |  |  |  |  |
| 7 | | 102.6558 | -0.61668 | 4792.195 | 41.63281 |  |  |  |  |  |
| 7.008333 | | 102.7394 | -0.61586 | 4791.867 | 41.76563 |  |  |  |  |  |
| 7.016667 | | 102.8201 | -0.61485 | 4791.516 | 41.97656 |  |  |  |  |  |
| 7.025 | | 102.9037 | -0.6129 | 4791.141 | 42.26563 |  |  |  |  |  |
| 7.033333 | | 102.9873 | -0.61132 | 4790.773 | 42.35156 |  |  |  |  |  |
| 7.041667 | | 103.0679 | -0.60995 | 4790.461 | 42.1875 |  |  |  |  |  |
| 7.05 | | 103.1535 | -0.60682 | 4790.117 | 42.0625 |  |  |  |  |  |
| 7.058333 | | 103.2411 | -0.60475 | 4789.742 | 41.96094 |  |  |  |  |  |
| 7.066667 | | 103.3277 | -0.60267 | 4789.359 | 41.97656 |  |  |  |  |  |
| 7.075 | | 103.4163 | -0.59977 | 4788.961 | 42.00781 |  |  |  |  |  |
| 7.083333 | | 103.5019 | -0.59833 | 4788.594 | 42.17969 |  |  |  |  |  |
| 7.091667 | | 103.5885 | -0.59673 | 4788.258 | 41.67188 |  |  |  |  |  |
| 7.1 | | 103.676 | -0.59448 | 4787.922 | 41.52344 |  |  |  |  |  |
| 7.108333 | | 103.7616 | -0.59326 | 4787.586 | 41.05469 |  |  |  |  |  |
| 7.116667 | | 103.8492 | -0.59122 | 4787.234 | 41.14063 |  |  |  |  |  |
| 7.125 | | 103.9338 | -0.58911 | 4786.875 | 41.35156 |  |  |  |  |  |
| 7.133333 | | 104.0194 | -0.58839 | 4786.5 | 41.25 |  |  |  |  |  |
| 7.141667 | | 104.1046 | -0.58662 | 4786.211 | 41.11719 |  |  |  |  |  |
| 7.15 | | 104.1852 | -0.58504 | 4785.891 | 41.08594 |  |  |  |  |  |
| 7.158333 | | 104.2658 | -0.58407 | 4785.609 | 41.0625 |  |  |  |  |  |
| 7.166667 | | 104.3494 | -0.58197 | 4785.25 | 40.95313 |  |  |  |  |  |
| 7.175 | | 104.4319 | -0.57993 | 4784.883 | 41.47656 |  |  |  |  |  |
| 7.183333 | | 104.5174 | -0.57826 | 4784.547 | 41.76563 |  |  |  |  |  |
| 7.191667 | | 104.601 | -0.57579 | 4784.219 | 42.10938 |  |  |  |  |  |
| 7.2 | | 104.6835 | -0.57382 | 4783.867 | 42.01563 |  |  |  |  |  |
| 7.208333 | | 104.772 | -0.57224 | 4783.531 | 42.0625 |  |  |  |  |  |
| 7.216667 | | 104.8526 | -0.5699 | 4783.195 | 42.41406 |  |  |  |  |  |
| 7.225 | | 104.9352 | -0.56778 | 4782.773 | 42.60938 |  |  |  |  |  |
| 7.233333 | | 105.0197 | -0.56672 | 4782.391 | 42.53906 |  |  |  |  |  |
| 7.241667 | | 105.1072 | -0.56453 | 4781.992 | 42.21875 |  |  |  |  |  |
| 7.25 | | 105.1917 | -0.56246 | 4781.656 | 42 |  |  |  |  |  |
| 7.258333 | | 105.2762 | -0.56092 | 4781.289 | 41.65625 |  |  |  |  |  |
| 7.266667 | | 105.3597 | -0.55818 | 4780.891 | 41.41406 |  |  |  |  |  |
| 7.275 | | 105.4413 | -0.55612 | 4780.508 | 41.07813 |  |  |  |  |  |
| 7.283333 | | 105.5209 | -0.55492 | 4780.172 | 40.88281 |  |  |  |  |  |
| 7.291667 | | 105.6006 | -0.55292 | 4779.867 | 40.47656 |  |  |  |  |  |
| 7.3 | | 105.6841 | -0.55148 | 4779.563 | 40.21094 |  |  |  |  |  |
| 7.308333 | | 105.7686 | -0.55042 | 4779.258 | 39.96875 |  |  |  |  |  |
| 7.316667 | | 105.8531 | -0.54798 | 4778.953 | 39.72656 |  |  |  |  |  |
| 7.325 | | 105.9347 | -0.5467 | 4778.656 | 39.71875 |  |  |  |  |  |
| 7.333333 | | 106.0183 | -0.54592 | 4778.352 | 39.54688 |  |  |  |  |  |
| 7.341667 | | 106.1028 | -0.5438 | 4778.07 | 39.10156 |  |  |  |  |  |
| 7.35 | | 106.1863 | -0.54237 | 4777.773 | 38.88281 |  |  |  |  |  |
| 7.358333 | | 106.2699 | -0.54122 | 4777.469 | 38.71875 |  |  |  |  |  |
| 7.366667 | | 106.3564 | -0.53853 | 4777.18 | 38.32031 |  |  |  |  |  |
| 7.375 | | 106.4419 | -0.5366 | 4776.836 | 38 |  |  |  |  |  |
| 7.383333 | | 106.5284 | -0.53496 | 4776.523 | 37.59375 |  |  |  |  |  |
| 7.391667 | | 106.6119 | -0.53242 | 4776.266 | 37.03125 |  |  |  |  |  |
| 7.4 | | 106.6935 | -0.53051 | 4775.969 | 36.85156 |  |  |  |  |  |
| 7.408333 | | 106.779 | -0.52832 | 4775.664 | 36.375 |  |  |  |  |  |
| 7.416667 | | 106.8645 | -0.52461 | 4775.391 | 36.15625 |  |  |  |  |  |
| 7.425 | | 106.9451 | -0.52261 | 4775.117 | 35.90625 |  |  |  |  |  |
| 7.433333 | | 107.0306 | -0.52084 | 4774.859 | 35.57813 |  |  |  |  |  |
| 7.441667 | | 107.1161 | -0.51813 | 4774.617 | 35.45313 |  |  |  |  |  |
| 7.45 | | 107.1977 | -0.51711 | 4774.336 | 35.5 |  |  |  |  |  |
| 7.458333 | | 107.2793 | -0.51616 | 4774.102 | 35.63281 |  |  |  |  |  |
| 7.466667 | | 107.3658 | -0.5137 | 4773.813 | 35.53125 |  |  |  |  |  |
| 7.475 | | 107.4493 | -0.5123 | 4773.547 | 35.64844 |  |  |  |  |  |
| 7.483333 | | 107.5348 | -0.51065 | 4773.289 | 35.78125 |  |  |  |  |  |
| 7.491667 | | 107.6154 | -0.50824 | 4773.016 | 35.94531 |  |  |  |  |  |
| 7.5 | | 107.698 | -0.50702 | 4772.703 | 36.13281 |  |  |  |  |  |
| 7.508333 | | 107.7855 | -0.50552 | 4772.391 | 36.4375 |  |  |  |  |  |
| 7.516667 | | 107.868 | -0.50283 | 4772.102 | 36.65625 |  |  |  |  |  |
| 7.525 | | 107.9506 | -0.50136 | 4771.773 | 36.75781 |  |  |  |  |  |
| 7.533333 | | 108.0371 | -0.49949 | 4771.453 | 36.74219 |  |  |  |  |  |
| 7.541667 | | 108.1255 | -0.49592 | 4771.125 | 36.92969 |  |  |  |  |  |
| 7.55 | | 108.2071 | -0.4942 | 4770.797 | 37.27344 |  |  |  |  |  |
| 7.558333 | | 108.2926 | -0.49197 | 4770.438 | 37.29688 |  |  |  |  |  |
| 7.566667 | | 108.3772 | -0.48871 | 4770.102 | 37.24219 |  |  |  |  |  |
| 7.575 | | 108.4666 | -0.48729 | 4769.773 | 37.34375 |  |  |  |  |  |
| 7.583333 | | 108.5511 | -0.48585 | 4769.453 | 37.36719 |  |  |  |  |  |
| 7.591667 | | 108.6366 | -0.48304 | 4769.109 | 37.24219 |  |  |  |  |  |
| 7.6 | | 108.7202 | -0.48224 | 4768.742 | 37.29688 |  |  |  |  |  |
| 7.608333 | | 108.8067 | -0.48021 | 4768.43 | 36.91406 |  |  |  |  |  |
| 7.616667 | | 108.8951 | -0.47699 | 4768.109 | 36.73438 |  |  |  |  |  |
| 7.625 | | 108.9806 | -0.47604 | 4767.773 | 36.48438 |  |  |  |  |  |
| 7.633333 | | 109.0652 | -0.4741 | 4767.469 | 36.11719 |  |  |  |  |  |
| 7.641667 | | 109.1487 | -0.47098 | 4767.164 | 35.99219 |  |  |  |  |  |
| 7.65 | | 109.2342 | -0.46997 | 4766.852 | 35.67969 |  |  |  |  |  |
| 7.658333 | | 109.3197 | -0.46783 | 4766.586 | 35.50781 |  |  |  |  |  |
| 7.666667 | | 109.4062 | -0.46466 | 4766.313 | 35.17188 |  |  |  |  |  |
| 7.675 | | 109.4868 | -0.46344 | 4766.047 | 35.02344 |  |  |  |  |  |
| 7.683333 | | 109.5723 | -0.46134 | 4765.805 | 34.8125 |  |  |  |  |  |
| 7.691667 | | 109.6559 | -0.4589 | 4765.516 | 34.85156 |  |  |  |  |  |
| 7.7 | | 109.7374 | -0.45774 | 4765.266 | 34.83594 |  |  |  |  |  |
| 7.708333 | | 109.8239 | -0.45557 | 4764.992 | 34.70313 |  |  |  |  |  |
| 7.716667 | | 109.9085 | -0.45242 | 4764.75 | 34.99219 |  |  |  |  |  |
| 7.725 | | 109.995 | -0.45094 | 4764.477 | 35.19531 |  |  |  |  |  |
| 7.733333 | | 110.0815 | -0.44886 | 4764.211 | 35.59375 |  |  |  |  |  |
| 7.741667 | | 110.164 | -0.44627 | 4763.914 | 35.75781 |  |  |  |  |  |
| 7.75 | | 110.2495 | -0.4448 | 4763.625 | 36.3125 |  |  |  |  |  |
| 7.758333 | | 110.337 | -0.44292 | 4763.352 | 36.60156 |  |  |  |  |  |
| 7.766667 | | 110.4235 | -0.44028 | 4763.031 | 37 |  |  |  |  |  |
| 7.775 | | 110.508 | -0.43942 | 4762.703 | 37.4375 |  |  |  |  |  |
| 7.783333 | | 110.5925 | -0.43751 | 4762.359 | 37.75 |  |  |  |  |  |
| 7.791667 | | 110.6751 | -0.43479 | 4762.031 | 38.17969 |  |  |  |  |  |
| 7.8 | | 110.7636 | -0.43403 | 4761.664 | 38.14063 |  |  |  |  |  |
| 7.808333 | | 110.851 | -0.43205 | 4761.32 | 38.05469 |  |  |  |  |  |
| 7.816667 | | 110.9356 | -0.42954 | 4760.961 | 37.96094 |  |  |  |  |  |
| 7.825 | | 111.0221 | -0.42869 | 4760.594 | 37.90625 |  |  |  |  |  |
| 7.833333 | | 111.1026 | -0.4268 | 4760.25 | 37.72656 |  |  |  |  |  |
| 7.841667 | | 111.1891 | -0.42402 | 4759.875 | 37.5625 |  |  |  |  |  |
| 7.85 | | 111.2756 | -0.42288 | 4759.555 | 37.00781 |  |  |  |  |  |
| 7.858333 | | 111.3631 | -0.42025 | 4759.25 | 36.39844 |  |  |  |  |  |
| 7.866667 | | 111.4476 | -0.41705 | 4758.945 | 35.78906 |  |  |  |  |  |
| 7.875 | | 111.5331 | -0.41591 | 4758.641 | 35.34375 |  |  |  |  |  |
| 7.883333 | | 111.6167 | -0.41361 | 4758.352 | 35.10156 |  |  |  |  |  |
| 7.891667 | | 111.6973 | -0.41104 | 4758.055 | 34.67188 |  |  |  |  |  |
| 7.9 | | 111.7779 | -0.41039 | 4757.828 | 34.35156 |  |  |  |  |  |
| 7.908333 | | 111.8614 | -0.40868 | 4757.609 | 33.78906 |  |  |  |  |  |
| 7.916667 | | 111.9528 | -0.40585 | 4757.391 | 33.16406 |  |  |  |  |  |
| 7.925 | | 112.0374 | -0.40501 | 4757.148 | 32.58594 |  |  |  |  |  |
| 7.933333 | | 112.1229 | -0.40265 | 4756.891 | 32.28125 |  |  |  |  |  |
| 7.941667 | | 112.2064 | -0.39994 | 4756.648 | 32.05469 |  |  |  |  |  |
| 7.95 | | 112.2929 | -0.39913 | 4756.406 | 32.02344 |  |  |  |  |  |
| 7.958333 | | 112.3784 | -0.39697 | 4756.211 | 31.66406 |  |  |  |  |  |
| 7.966667 | | 112.4629 | -0.39444 | 4756.008 | 31.42969 |  |  |  |  |  |
| 7.975 | | 112.5534 | -0.39291 | 4755.813 | 31.25 |  |  |  |  |  |
| 7.983333 | | 112.6438 | -0.38973 | 4755.578 | 31.14063 |  |  |  |  |  |
| 7.991667 | | 112.7322 | -0.38638 | 4755.344 | 31.32813 |  |  |  |  |  |
| 8 | | 112.8138 | -0.38505 | 4755.07 | 31.28906 |  |  |  |  |  |
| 8.008333 | | 112.8993 | -0.38181 | 4754.859 | 31.45313 |  |  |  |  |  |
| 8.016667 | | 112.9848 | -0.3788 | 4754.633 | 31.64844 |  |  |  |  |  |
| 8.025 | | 113.0704 | -0.37775 | 4754.398 | 31.97656 |  |  |  |  |  |
| 8.033333 | | 113.1568 | -0.37466 | 4754.156 | 31.91406 |  |  |  |  |  |
| 8.041667 | | 113.2424 | -0.37205 | 4753.867 | 31.92188 |  |  |  |  |  |
| 8.05 | | 113.3288 | -0.37093 | 4753.609 | 32 |  |  |  |  |  |
| 8.058333 | | 113.4124 | -0.36855 | 4753.313 | 31.9375 |  |  |  |  |  |
| 8.066667 | | 113.4999 | -0.36618 | 4753.031 | 31.9375 |  |  |  |  |  |
| 8.075 | | 113.5854 | -0.36512 | 4752.719 | 31.74219 |  |  |  |  |  |
| 8.083333 | | 113.6738 | -0.36259 | 4752.461 | 31.65625 |  |  |  |  |  |
| 8.091667 | | 113.7613 | -0.36069 | 4752.188 | 31.27344 |  |  |  |  |  |
| 8.1 | | 113.8468 | -0.35966 | 4751.914 | 31.02344 |  |  |  |  |  |
| 8.108333 | | 113.9382 | -0.35695 | 4751.641 | 30.75781 |  |  |  |  |  |
| 8.116667 | | 114.0267 | -0.35534 | 4751.383 | 30.53125 |  |  |  |  |  |
| 8.125 | | 114.1132 | -0.35406 | 4751.133 | 30.53906 |  |  |  |  |  |
| 8.133333 | | 114.2016 | -0.35168 | 4750.875 | 30.07031 |  |  |  |  |  |
| 8.141667 | | 114.2921 | -0.3499 | 4750.656 | 29.85938 |  |  |  |  |  |
| 8.15 | | 114.3766 | -0.34857 | 4750.422 | 29.79688 |  |  |  |  |  |
| 8.158333 | | 114.4631 | -0.34608 | 4750.195 | 29.45313 |  |  |  |  |  |
| 8.166667 | | 114.5486 | -0.34457 | 4749.961 | 29.3125 |  |  |  |  |  |
| 8.175 | | 114.6351 | -0.34302 | 4749.688 | 29.46094 |  |  |  |  |  |
| 8.183333 | | 114.7186 | -0.3406 | 4749.492 | 29.41406 |  |  |  |  |  |
| 8.191667 | | 114.8031 | -0.33863 | 4749.258 | 29.28125 |  |  |  |  |  |
| 8.2 | | 114.8898 | -0.33644 | 4749.008 | 29.39844 |  |  |  |  |  |
| 8.208333 | | 114.9733 | -0.33339 | 4748.805 | 29.22656 |  |  |  |  |  |
| 8.216667 | | 115.0558 | -0.33129 | 4748.57 | 29.22656 |  |  |  |  |  |
| 8.225 | | 115.1344 | -0.32968 | 4748.289 | 29.25781 |  |  |  |  |  |
| 8.233333 | | 115.2198 | -0.3267 | 4748.047 | 29.26563 |  |  |  |  |  |
| 8.241667 | | 115.3013 | -0.32499 | 4747.82 | 29.46875 |  |  |  |  |  |
| 8.25 | | 115.381 | -0.32375 | 4747.563 | 29.57031 |  |  |  |  |  |
| 8.258333 | | 115.4653 | -0.32078 | 4747.352 | 29.32031 |  |  |  |  |  |
| 8.266667 | | 115.5497 | -0.31854 | 4747.102 | 29.49219 |  |  |  |  |  |
| 8.275 | | 115.6303 | -0.31705 | 4746.859 | 29.5 |  |  |  |  |  |
| 8.283333 | | 115.7119 | -0.31466 | 4746.617 | 29.57031 |  |  |  |  |  |
| 8.291667 | | 115.7924 | -0.31292 | 4746.336 | 29.74219 |  |  |  |  |  |
| 8.3 | | 115.8721 | -0.31186 | 4746.086 | 29.85156 |  |  |  |  |  |
| 8.308333 | | 115.9603 | -0.3085 | 4745.875 | 30.11719 |  |  |  |  |  |
| 8.316667 | | 116.0465 | -0.30626 | 4745.609 | 30.0625 |  |  |  |  |  |
| 8.325 | | 116.1338 | -0.30432 | 4745.375 | 29.96094 |  |  |  |  |  |
| 8.333333 | | 116.2219 | -0.30065 | 4745.109 | 30.07031 |  |  |  |  |  |
| 8.341667 | | 116.3082 | -0.29841 | 4744.844 | 30.03125 |  |  |  |  |  |
| 8.35 | | 116.3954 | -0.29657 | 4744.57 | 29.78125 |  |  |  |  |  |
| 8.358333 | | 116.4827 | -0.29388 | 4744.297 | 29.67969 |  |  |  |  |  |
| 8.366667 | | 116.568 | -0.29223 | 4744.047 | 29.27344 |  |  |  |  |  |
| 8.375 | | 116.6562 | -0.28998 | 4743.813 | 28.86719 |  |  |  |  |  |
| 8.383333 | | 116.7434 | -0.28749 | 4743.547 | 28.80469 |  |  |  |  |  |
| 8.391667 | | 116.8221 | -0.2863 | 4743.305 | 28.50781 |  |  |  |  |  |
| 8.4 | | 116.9008 | -0.28447 | 4743.086 | 28.48438 |  |  |  |  |  |
| 8.408333 | | 116.9804 | -0.28223 | 4742.852 | 28.39844 |  |  |  |  |  |
| 8.416667 | | 117.0658 | -0.28088 | 4742.664 | 28.16406 |  |  |  |  |  |
| 8.425 | | 117.1454 | -0.27927 | 4742.461 | 27.97656 |  |  |  |  |  |
| 8.433333 | | 117.2326 | -0.27625 | 4742.234 | 27.8125 |  |  |  |  |  |
| 8.441667 | | 117.3161 | -0.27444 | 4742.031 | 28.00781 |  |  |  |  |  |
| 8.45 | | 117.3976 | -0.27238 | 4741.789 | 28.02344 |  |  |  |  |  |
| 8.458333 | | 117.481 | -0.26969 | 4741.563 | 28.13281 |  |  |  |  |  |
| 8.466667 | | 117.5673 | -0.26793 | 4741.352 | 27.88281 |  |  |  |  |  |
| 8.475 | | 117.6526 | -0.26571 | 4741.156 | 27.76563 |  |  |  |  |  |
| 8.483333 | | 117.7437 | -0.26297 | 4740.938 | 27.64063 |  |  |  |  |  |
| 8.491667 | | 117.8309 | -0.26166 | 4740.68 | 27.70313 |  |  |  |  |  |
| 8.5 | | 117.9181 | -0.25918 | 4740.445 | 27.71094 |  |  |  |  |  |
| 8.508333 | | 117.9997 | -0.25731 | 4740.203 | 27.89063 |  |  |  |  |  |
| 8.516667 | | 118.0774 | -0.25632 | 4740 | 28.10938 |  |  |  |  |  |
| 8.525 | | 118.1637 | -0.25387 | 4739.781 | 27.85156 |  |  |  |  |  |
| 8.533333 | | 118.2452 | -0.25154 | 4739.57 | 27.72656 |  |  |  |  |  |
| 8.541667 | | 118.3315 | -0.2499 | 4739.328 | 27.74219 |  |  |  |  |  |
| 8.55 | | 118.4168 | -0.24722 | 4739.094 | 27.40625 |  |  |  |  |  |
| 8.558333 | | 118.505 | -0.24468 | 4738.836 | 27.27344 |  |  |  |  |  |
| 8.566667 | | 118.5856 | -0.24339 | 4738.578 | 27.02344 |  |  |  |  |  |
| 8.575 | | 118.6719 | -0.24029 | 4738.375 | 26.82813 |  |  |  |  |  |
| 8.583333 | | 118.7534 | -0.23789 | 4738.156 | 26.48438 |  |  |  |  |  |
| 8.591667 | | 118.8378 | -0.23602 | 4737.93 | 26.41406 |  |  |  |  |  |
| 8.6 | | 118.9193 | -0.23335 | 4737.742 | 26.13281 |  |  |  |  |  |
| 8.608333 | | 119.0018 | -0.2312 | 4737.531 | 25.95313 |  |  |  |  |  |
| 8.616667 | | 119.09 | -0.22958 | 4737.344 | 26.07031 |  |  |  |  |  |
| 8.625 | | 119.1744 | -0.22676 | 4737.148 | 25.86719 |  |  |  |  |  |
| 8.633333 | | 119.2569 | -0.22436 | 4736.969 | 25.9375 |  |  |  |  |  |
| 8.641667 | | 119.3384 | -0.22283 | 4736.75 | 25.90625 |  |  |  |  |  |
| 8.65 | | 119.4228 | -0.22016 | 4736.578 | 26.10938 |  |  |  |  |  |
| 8.658333 | | 119.5034 | -0.21809 | 4736.375 | 26.00781 |  |  |  |  |  |
| 8.666667 | | 119.5887 | -0.21667 | 4736.141 | 26.15625 |  |  |  |  |  |
| 8.675 | | 119.6759 | -0.2141 | 4735.953 | 26.17188 |  |  |  |  |  |
| 8.683333 | | 119.7632 | -0.21172 | 4735.719 | 26.10938 |  |  |  |  |  |
| 8.691667 | | 119.8447 | -0.21036 | 4735.5 | 26.07031 |  |  |  |  |  |
| 8.7 | | 119.9319 | -0.20768 | 4735.242 | 25.90625 |  |  |  |  |  |
| 8.708333 | | 120.0125 | -0.20615 | 4735.039 | 26.17188 |  |  |  |  |  |
| 8.716667 | | 120.0969 | -0.20525 | 4734.797 | 26.21875 |  |  |  |  |  |
| 8.725 | | 120.1841 | -0.20294 | 4734.578 | 26.32813 |  |  |  |  |  |
| 8.733333 | | 120.2675 | -0.20084 | 4734.359 | 26.17969 |  |  |  |  |  |
| 8.741667 | | 120.3548 | -0.19907 | 4734.141 | 26.10938 |  |  |  |  |  |
| 8.75 | | 120.4373 | -0.19609 | 4733.945 | 26.01563 |  |  |  |  |  |
| 8.758333 | | 120.5226 | -0.19412 | 4733.695 | 26.09375 |  |  |  |  |  |
| 8.766667 | | 120.6127 | -0.19217 | 4733.469 | 26.26563 |  |  |  |  |  |
| 8.775 | | 120.7037 | -0.18912 | 4733.234 | 26.40625 |  |  |  |  |  |
| 8.783333 | | 120.7852 | -0.18782 | 4733.039 | 26.58594 |  |  |  |  |  |
| 8.791667 | | 120.8725 | -0.18597 | 4732.828 | 26.53125 |  |  |  |  |  |
| 8.8 | | 120.9549 | -0.18277 | 4732.625 | 26.67188 |  |  |  |  |  |
| 8.808333 | | 121.0384 | -0.1813 | 4732.398 | 26.49219 |  |  |  |  |  |
| 8.816667 | | 121.1294 | -0.17957 | 4732.156 | 26.40625 |  |  |  |  |  |
| 8.825 | | 121.2157 | -0.17676 | 4731.914 | 26.42969 |  |  |  |  |  |
| 8.833333 | | 121.3038 | -0.17563 | 4731.664 | 26.28906 |  |  |  |  |  |
| 8.841667 | | 121.393 | -0.17377 | 4731.445 | 25.97656 |  |  |  |  |  |
| 8.85 | | 121.4745 | -0.17119 | 4731.211 | 25.88281 |  |  |  |  |  |
| 8.858333 | | 121.5589 | -0.17009 | 4731.008 | 25.58594 |  |  |  |  |  |
| 8.866667 | | 121.6442 | -0.16872 | 4730.805 | 25.39063 |  |  |  |  |  |
| 8.875 | | 121.7324 | -0.1657 | 4730.578 | 25.17969 |  |  |  |  |  |
| 8.883333 | | 121.8168 | -0.16464 | 4730.383 | 25.01563 |  |  |  |  |  |
| 8.891667 | | 121.9031 | -0.16291 | 4730.203 | 24.90625 |  |  |  |  |  |
| 8.9 | | 121.9827 | -0.16023 | 4730 | 24.85938 |  |  |  |  |  |
| 8.908333 | | 122.069 | -0.15878 | 4729.82 | 24.78125 |  |  |  |  |  |
| 8.916667 | | 122.1534 | -0.15704 | 4729.633 | 24.8125 |  |  |  |  |  |
| 8.925 | | 122.2358 | -0.1545 | 4729.453 | 24.70313 |  |  |  |  |  |
| 8.933333 | | 122.324 | -0.15298 | 4729.266 | 24.64844 |  |  |  |  |  |
| 8.941667 | | 122.4103 | -0.15091 | 4729.078 | 24.48438 |  |  |  |  |  |
| 8.95 | | 122.4975 | -0.14809 | 4728.875 | 24.24219 |  |  |  |  |  |
| 8.958333 | | 122.5819 | -0.14728 | 4728.672 | 24.11719 |  |  |  |  |  |
| 8.966667 | | 122.672 | -0.14517 | 4728.469 | 23.89063 |  |  |  |  |  |
| 8.975 | | 122.7564 | -0.14219 | 4728.273 | 23.82031 |  |  |  |  |  |
| 8.983333 | | 122.8426 | -0.1411 | 4728.086 | 23.88281 |  |  |  |  |  |
| 8.991667 | | 122.9213 | -0.13909 | 4727.906 | 23.72656 |  |  |  |  |  |
| 9 | | 123.0057 | -0.13638 | 4727.742 | 23.375 |  |  |  |  |  |
| 9.008333 | | 123.091 | -0.13534 | 4727.555 | 23.23438 |  |  |  |  |  |
| 9.016667 | | 123.1783 | -0.13319 | 4727.383 | 23 |  |  |  |  |  |
| 9.025 | | 123.2617 | -0.13012 | 4727.203 | 22.92969 |  |  |  |  |  |
| 9.033333 | | 123.3537 | -0.12823 | 4726.992 | 22.96094 |  |  |  |  |  |
| 9.041667 | | 123.439 | -0.12579 | 4726.813 | 22.80469 |  |  |  |  |  |
| 9.05 | | 123.5253 | -0.12284 | 4726.656 | 22.84375 |  |  |  |  |  |
| 9.058333 | | 123.6153 | -0.12195 | 4726.477 | 22.75 |  |  |  |  |  |
| 9.066667 | | 123.7054 | -0.1197 | 4726.32 | 22.42969 |  |  |  |  |  |
| 9.075 | | 123.7974 | -0.11701 | 4726.133 | 22.38281 |  |  |  |  |  |
| 9.083333 | | 123.8856 | -0.11612 | 4725.93 | 22.60156 |  |  |  |  |  |
| 9.091667 | | 123.9728 | -0.11388 | 4725.766 | 22.5625 |  |  |  |  |  |
| 9.1 | | 124.0581 | -0.11152 | 4725.563 | 22.55469 |  |  |  |  |  |
| 9.108333 | | 124.1453 | -0.11084 | 4725.383 | 22.53125 |  |  |  |  |  |
| 9.116667 | | 124.2231 | -0.10909 | 4725.234 | 22.39063 |  |  |  |  |  |
| 9.125 | | 124.3084 | -0.10641 | 4725.047 | 22.35156 |  |  |  |  |  |
| 9.133333 | | 124.3957 | -0.10568 | 4724.828 | 22.125 |  |  |  |  |  |
| 9.141667 | | 124.481 | -0.10322 | 4724.633 | 22.35156 |  |  |  |  |  |
| 9.15 | | 124.5663 | -0.10079 | 4724.453 | 22.24219 |  |  |  |  |  |
| 9.158333 | | 124.6507 | -0.10003 | 4724.266 | 21.99219 |  |  |  |  |  |
| 9.166667 | | 124.7351 | -0.09724 | 4724.086 | 22.04688 |  |  |  |  |  |
| 9.175 | | 124.8166 | -0.09487 | 4723.906 | 21.97656 |  |  |  |  |  |
| 9.183333 | | 124.901 | -0.09425 | 4723.734 | 22.00781 |  |  |  |  |  |
| 9.191667 | | 124.9873 | -0.09167 | 4723.516 | 22.03906 |  |  |  |  |  |
| 9.2 | | 125.0764 | -0.0892 | 4723.344 | 21.94531 |  |  |  |  |  |
| 9.208333 | | 125.1665 | -0.08794 | 4723.188 | 21.79688 |  |  |  |  |  |
| 9.216667 | | 125.2506 | -0.08551 | 4723.008 | 21.76563 |  |  |  |  |  |
| 9.225 | | 125.333 | -0.08362 | 4722.828 | 21.67188 |  |  |  |  |  |
| 9.233333 | | 125.4154 | -0.08247 | 4722.625 | 22 |  |  |  |  |  |
| 9.241667 | | 125.5062 | -0.07961 | 4722.445 | 22.125 |  |  |  |  |  |
| 9.25 | | 125.5933 | -0.0779 | 4722.273 | 22.02344 |  |  |  |  |  |
| 9.258333 | | 125.6842 | -0.07627 | 4722.109 | 22.27344 |  |  |  |  |  |
| 9.266667 | | 125.7675 | -0.0738 | 4721.93 | 22.24219 |  |  |  |  |  |
| 9.275 | | 125.849 | -0.07224 | 4721.766 | 22.26563 |  |  |  |  |  |
| 9.283333 | | 125.9342 | -0.07092 | 4721.539 | 22.57813 |  |  |  |  |  |
| 9.291667 | | 126.0148 | -0.06866 | 4721.336 | 22.82031 |  |  |  |  |  |
| 9.3 | | 126.0991 | -0.06688 | 4721.156 | 22.72656 |  |  |  |  |  |
| 9.308333 | | 126.1843 | -0.06547 | 4720.93 | 22.72656 |  |  |  |  |  |
| 9.316667 | | 126.2676 | -0.06297 | 4720.75 | 22.35938 |  |  |  |  |  |
| 9.325 | | 126.3472 | -0.06122 | 4720.563 | 22.32813 |  |  |  |  |  |
| 9.333333 | | 126.4315 | -0.05981 | 4720.336 | 22.22656 |  |  |  |  |  |
| 9.341667 | | 126.5177 | -0.05732 | 4720.109 | 21.82031 |  |  |  |  |  |
| 9.35 | | 126.6048 | -0.0553 | 4719.938 | 21.78125 |  |  |  |  |  |
| 9.358333 | | 126.6956 | -0.05392 | 4719.75 | 21.67969 |  |  |  |  |  |
| 9.366667 | | 126.7818 | -0.05116 | 4719.617 | 21.44531 |  |  |  |  |  |
| 9.375 | | 126.868 | -0.04906 | 4719.43 | 21.35156 |  |  |  |  |  |
| 9.383333 | | 126.956 | -0.0473 | 4719.266 | 21.40625 |  |  |  |  |  |
| 9.391667 | | 127.0468 | -0.04415 | 4719.133 | 21.49219 |  |  |  |  |  |
| 9.4 | | 127.1377 | -0.04259 | 4718.961 | 21.47656 |  |  |  |  |  |
| 9.408333 | | 127.2248 | -0.04104 | 4718.797 | 21.34375 |  |  |  |  |  |
| 9.416667 | | 127.3109 | -0.03866 | 4718.656 | 21.19531 |  |  |  |  |  |
| 9.425 | | 127.3943 | -0.03705 | 4718.492 | 20.92969 |  |  |  |  |  |
| 9.433333 | | 127.4786 | -0.03501 | 4718.305 | 20.98438 |  |  |  |  |  |
| 9.441667 | | 127.5629 | -0.03216 | 4718.109 | 20.88281 |  |  |  |  |  |
| 9.45 | | 127.649 | -0.03034 | 4717.93 | 20.78125 |  |  |  |  |  |
| 9.458333 | | 127.7342 | -0.02871 | 4717.766 | 20.44531 |  |  |  |  |  |
| 9.466667 | | 127.8204 | -0.0264 | 4717.617 | 20.29688 |  |  |  |  |  |
| 9.475 | | 127.9038 | -0.02517 | 4717.477 | 20.0625 |  |  |  |  |  |
| 9.483333 | | 127.9871 | -0.02336 | 4717.289 | 20.08594 |  |  |  |  |  |
| 9.491667 | | 128.0733 | -0.02079 | 4717.125 | 20.17188 |  |  |  |  |  |
| 9.5 | | 128.1622 | -0.01924 | 4716.969 | 20.25781 |  |  |  |  |  |
| 9.508333 | | 128.2484 | -0.01737 | 4716.844 | 20.40625 |  |  |  |  |  |
| 9.516667 | | 128.3364 | -0.01491 | 4716.695 | 20.44531 |  |  |  |  |  |
| 9.525 | | 128.4217 | -0.0137 | 4716.555 | 20.42969 |  |  |  |  |  |
| 9.533333 | | 128.5069 | -0.0119 | 4716.383 | 20.38281 |  |  |  |  |  |
| 9.541667 | | 128.5959 | -0.00894 | 4716.195 | 20.51563 |  |  |  |  |  |
| 9.55 | | 128.6792 | -0.00746 | 4716.023 | 20.66406 |  |  |  |  |  |
| 9.558333 | | 128.7644 | -0.00581 | 4715.828 | 20.625 |  |  |  |  |  |
| 9.566667 | | 128.8506 | -0.00359 | 4715.656 | 20.49219 |  |  |  |  |  |
| 9.575 | | 128.9377 | -0.00232 | 4715.484 | 20.38281 |  |  |  |  |  |
| 9.583333 | | 129.022 | -0.00091 | 4715.313 | 20.41406 |  |  |  |  |  |
| 9.591667 | | 129.1091 | 0.001509 | 4715.125 | 20.24219 |  |  |  |  |  |
| 9.6 | | 129.1943 | 0.002813 | 4714.93 | 19.85938 |  |  |  |  |  |
| 9.608333 | | 129.2823 | 0.004645 | 4714.766 | 19.67188 |  |  |  |  |  |
| 9.616667 | | 129.3694 | 0.006851 | 4714.609 | 19.58594 |  |  |  |  |  |
| 9.625 | | 129.4556 | 0.007982 | 4714.461 | 19.5 |  |  |  |  |  |
| 9.633333 | | 129.5446 | 0.009989 | 4714.281 | 19.30469 |  |  |  |  |  |
| 9.641667 | | 129.6345 | 0.012882 | 4714.133 | 19.15625 |  |  |  |  |  |
| 9.65 | | 129.7225 | 0.014849 | 4714.016 | 19.14063 |  |  |  |  |  |
| 9.658333 | | 129.8096 | 0.018139 | 4713.867 | 19.09375 |  |  |  |  |  |
| 9.666667 | | 129.8967 | 0.02092 | 4713.719 | 18.875 |  |  |  |  |  |
| 9.675 | | 129.9838 | 0.022419 | 4713.57 | 18.85938 |  |  |  |  |  |
| 9.683333 | | 130.0756 | 0.025295 | 4713.422 | 18.83594 |  |  |  |  |  |
| 9.691667 | | 130.1627 | 0.027401 | 4713.281 | 18.96875 |  |  |  |  |  |
| 9.7 | | 130.2479 | 0.028382 | 4713.117 | 18.85156 |  |  |  |  |  |
| 9.708333 | | 130.3341 | 0.030788 | 4712.961 | 18.8125 |  |  |  |  |  |
| 9.716667 | | 130.4193 | 0.032706 | 4712.836 | 18.75 |  |  |  |  |  |
| 9.725 | | 130.5064 | 0.033668 | 4712.68 | 18.47656 |  |  |  |  |  |
| 9.733333 | | 130.5944 | 0.0358 | 4712.516 | 18.28125 |  |  |  |  |  |
| 9.741667 | | 130.6815 | 0.037908 | 4712.352 | 18.21875 |  |  |  |  |  |
| 9.75 | | 130.7658 | 0.038745 | 4712.203 | 18.09375 |  |  |  |  |  |
| 9.758333 | | 130.8529 | 0.040914 | 4712.063 | 17.94531 |  |  |  |  |  |
| 9.766667 | | 130.9381 | 0.042995 | 4711.914 | 18.04688 |  |  |  |  |  |
| 9.775 | | 131.0233 | 0.044008 | 4711.797 | 17.875 |  |  |  |  |  |
| 9.783333 | | 131.1095 | 0.046268 | 4711.664 | 17.70313 |  |  |  |  |  |
| 9.791667 | | 131.1975 | 0.048288 | 4711.516 | 17.8125 |  |  |  |  |  |
| 9.8 | | 131.2846 | 0.049433 | 4711.375 | 17.78125 |  |  |  |  |  |
| 9.808333 | | 131.3652 | 0.051786 | 4711.25 | 17.84375 |  |  |  |  |  |
| 9.816667 | | 131.4485 | 0.053683 | 4711.086 | 17.89844 |  |  |  |  |  |
| 9.825 | | 131.5328 | 0.054596 | 4710.969 | 18.00781 |  |  |  |  |  |
| 9.833333 | | 131.6199 | 0.057419 | 4710.836 | 18.20313 |  |  |  |  |  |
| 9.841667 | | 131.7051 | 0.059461 | 4710.664 | 18.47656 |  |  |  |  |  |
| 9.85 | | 131.7894 | 0.060694 | 4710.523 | 18.5625 |  |  |  |  |  |
| 9.858333 | | 131.8737 | 0.063625 | 4710.367 | 18.77344 |  |  |  |  |  |
| 9.866667 | | 131.9608 | 0.065759 | 4710.219 | 18.85938 |  |  |  |  |  |
| 9.875 | | 132.046 | 0.067091 | 4710.047 | 18.76563 |  |  |  |  |  |
| 9.883333 | | 132.1331 | 0.070173 | 4709.867 | 18.8125 |  |  |  |  |  |
| 9.891667 | | 132.224 | 0.071809 | 4709.664 | 18.83594 |  |  |  |  |  |
| 9.9 | | 132.3111 | 0.073034 | 4709.5 | 18.92969 |  |  |  |  |  |
| 9.908333 | | 132.3982 | 0.076394 | 4709.305 | 18.71875 |  |  |  |  |  |
| 9.916667 | | 132.4834 | 0.077176 | 4709.133 | 18.72656 |  |  |  |  |  |
| 9.925 | | 132.5696 | 0.077706 | 4708.984 | 18.5 |  |  |  |  |  |
| 9.933333 | | 132.6595 | 0.080234 | 4708.828 | 18.28125 |  |  |  |  |  |
| 9.941667 | | 132.7484 | 0.080858 | 4708.656 | 17.83594 |  |  |  |  |  |
| 9.95 | | 132.8365 | 0.081809 | 4708.477 | 17.71094 |  |  |  |  |  |
| 9.958333 | | 132.9245 | 0.084747 | 4708.359 | 17.42969 |  |  |  |  |  |
| 9.966667 | | 133.0107 | 0.086096 | 4708.203 | 17.22656 |  |  |  |  |  |
| 9.975 | | 133.0968 | 0.087678 | 4708.078 | 16.85938 |  |  |  |  |  |
| 9.983333 | | 133.1867 | 0.091135 | 4707.961 | 16.71875 |  |  |  |  |  |
| 9.991667 | | 133.2757 | 0.092459 | 4707.867 | 16.47656 |  |  |  |  |  |
| 10 | | 133.3628 | 0.094475 | 4707.742 | 16.0625 |  |  |  |  |  |
| 10.00833 | | 133.4518 | 0.098669 | 4707.641 | 15.6875 |  |  |  |  |  |
| 10.01667 | | 133.5379 | 0.100226 | 4707.516 | 15.64844 |  |  |  |  |  |
| 10.025 | | 133.625 | 0.102472 | 4707.43 | 15.53906 |  |  |  |  |  |
| 10.03333 | | 133.7112 | 0.106125 | 4707.305 | 15.20313 |  |  |  |  |  |
| 10.04167 | | 133.8011 | 0.107513 | 4707.211 | 14.9375 |  |  |  |  |  |
| 10.05 | | 133.8891 | 0.109106 | 4707.133 | 14.53906 |  |  |  |  |  |
| 10.05833 | | 133.9753 | 0.111767 | 4707.047 | 14.60938 |  |  |  |  |  |
| 10.06667 | | 134.0596 | 0.112508 | 4706.914 | 14.15625 |  |  |  |  |  |
| 10.075 | | 134.1448 | 0.113831 | 4706.805 | 14.10938 |  |  |  |  |  |
| 10.08333 | | 134.2338 | 0.116562 | 4706.727 | 13.98438 |  |  |  |  |  |
| 10.09167 | | 134.3228 | 0.117029 | 4706.633 | 13.78125 |  |  |  |  |  |
| 10.1 | | 134.4089 | 0.118573 | 4706.563 | 13.61719 |  |  |  |  |  |
| 10.10833 | | 134.496 | 0.121242 | 4706.438 | 13.49219 |  |  |  |  |  |
| 10.11667 | | 134.5831 | 0.121658 | 4706.375 | 13.51563 |  |  |  |  |  |
| 10.125 | | 134.6683 | 0.123002 | 4706.266 | 13.73438 |  |  |  |  |  |
| 10.13333 | | 134.7573 | 0.125759 | 4706.164 | 13.8125 |  |  |  |  |  |
| 10.14167 | | 134.8463 | 0.126569 | 4706.07 | 13.85156 |  |  |  |  |  |
| 10.15 | | 134.9371 | 0.128144 | 4705.984 | 14.17188 |  |  |  |  |  |
| 10.15833 | | 135.027 | 0.13117 | 4705.883 | 14.0625 |  |  |  |  |  |
| 10.16667 | | 135.1169 | 0.13209 | 4705.766 | 13.98438 |  |  |  |  |  |
| 10.175 | | 135.2031 | 0.134097 | 4705.617 | 14.07031 |  |  |  |  |  |
| 10.18333 | | 135.293 | 0.136921 | 4705.484 | 13.85156 |  |  |  |  |  |
| 10.19167 | | 135.381 | 0.137897 | 4705.359 | 13.85156 |  |  |  |  |  |
| 10.2 | | 135.4719 | 0.140251 | 4705.188 | 13.8125 |  |  |  |  |  |
| 10.20833 | | 135.5609 | 0.142861 | 4705.086 | 13.90625 |  |  |  |  |  |
| 10.21667 | | 135.646 | 0.143775 | 4704.977 | 13.69531 |  |  |  |  |  |
| 10.225 | | 135.7301 | 0.145725 | 4704.836 | 13.46094 |  |  |  |  |  |
| 10.23333 | | 135.8143 | 0.148376 | 4704.742 | 13.04688 |  |  |  |  |  |
| 10.24167 | | 135.9003 | 0.149477 | 4704.617 | 12.9375 |  |  |  |  |  |
| 10.25 | | 135.9845 | 0.151666 | 4704.508 | 12.97656 |  |  |  |  |  |
| 10.25833 | | 136.0714 | 0.154509 | 4704.367 | 12.75781 |  |  |  |  |  |
| 10.26667 | | 136.1537 | 0.155125 | 4704.273 | 12.75781 |  |  |  |  |  |
| 10.275 | | 136.2426 | 0.15748 | 4704.195 | 12.72656 |  |  |  |  |  |
| 10.28333 | | 136.3286 | 0.160082 | 4704.141 | 13.02344 |  |  |  |  |  |
| 10.29167 | | 136.4109 | 0.160733 | 4704.031 | 12.84375 |  |  |  |  |  |
| 10.3 | | 136.4997 | 0.163271 | 4703.914 | 12.92969 |  |  |  |  |  |
| 10.30833 | | 136.5885 | 0.165751 | 4703.828 | 12.96094 |  |  |  |  |  |
| 10.31667 | | 136.6773 | 0.166205 | 4703.727 | 13.09375 |  |  |  |  |  |
| 10.325 | | 136.7661 | 0.168122 | 4703.609 | 13.125 |  |  |  |  |  |
| 10.33333 | | 136.853 | 0.170095 | 4703.469 | 13.21875 |  |  |  |  |  |
| 10.34167 | | 136.94 | 0.171116 | 4703.391 | 13.3125 |  |  |  |  |  |
| 10.35 | | 137.0269 | 0.173826 | 4703.266 | 13.26563 |  |  |  |  |  |
| 10.35833 | | 137.112 | 0.175935 | 4703.148 | 13.10938 |  |  |  |  |  |
| 10.36667 | | 137.198 | 0.177098 | 4703.023 | 12.8125 |  |  |  |  |  |
| 10.375 | | 137.2859 | 0.179651 | 4702.906 | 12.625 |  |  |  |  |  |
| 10.38333 | | 137.3719 | 0.181779 | 4702.789 | 12.46094 |  |  |  |  |  |
| 10.39167 | | 137.4607 | 0.183035 | 4702.664 | 12.42969 |  |  |  |  |  |
| 10.4 | | 137.5486 | 0.18574 | 4702.563 | 12.28125 |  |  |  |  |  |
| 10.40833 | | 137.6337 | 0.187497 | 4702.477 | 12.20313 |  |  |  |  |  |
| 10.41667 | | 137.7215 | 0.188471 | 4702.414 | 12.16406 |  |  |  |  |  |
| 10.425 | | 137.8085 | 0.1906 | 4702.336 | 12.0625 |  |  |  |  |  |
| 10.43333 | | 137.8982 | 0.191986 | 4702.258 | 11.96094 |  |  |  |  |  |
| 10.44167 | | 137.9852 | 0.192818 | 4702.164 | 11.875 |  |  |  |  |  |
| 10.45 | | 138.0703 | 0.194875 | 4702.078 | 12.03906 |  |  |  |  |  |
| 10.45833 | | 138.16 | 0.196255 | 4701.984 | 12.25 |  |  |  |  |  |
| 10.46667 | | 138.2488 | 0.197103 | 4701.898 | 12.16406 |  |  |  |  |  |
| 10.475 | | 138.3329 | 0.198934 | 4701.805 | 12.11719 |  |  |  |  |  |
| 10.48333 | | 138.4162 | 0.200399 | 4701.719 | 12.10938 |  |  |  |  |  |
| 10.49167 | | 138.5031 | 0.20148 | 4701.641 | 12.25 |  |  |  |  |  |
| 10.5 | | 138.5891 | 0.204167 | 4701.508 | 12.35938 |  |  |  |  |  |
| 10.50833 | | 138.6752 | 0.20618 | 4701.391 | 12.39844 |  |  |  |  |  |
| 10.51667 | | 138.764 | 0.207559 | 4701.289 | 12.75 |  |  |  |  |  |
| 10.525 | | 138.8528 | 0.210115 | 4701.195 | 12.89063 |  |  |  |  |  |
| 10.53333 | | 138.9388 | 0.211727 | 4701.086 | 13 |  |  |  |  |  |
| 10.54167 | | 139.0239 | 0.212755 | 4700.961 | 13.04688 |  |  |  |  |  |
| 10.55 | | 139.109 | 0.215452 | 4700.836 | 13.19531 |  |  |  |  |  |
| 10.55833 | | 139.1959 | 0.217446 | 4700.727 | 13.32813 |  |  |  |  |  |
| 10.56667 | | 139.2847 | 0.218876 | 4700.578 | 13.27344 |  |  |  |  |  |
| 10.575 | | 139.3744 | 0.221568 | 4700.438 | 13.30469 |  |  |  |  |  |
| 10.58333 | | 139.466 | 0.222732 | 4700.313 | 13.46875 |  |  |  |  |  |
| 10.59167 | | 139.552 | 0.223467 | 4700.195 | 13.46875 |  |  |  |  |  |
| 10.6 | | 139.6362 | 0.225481 | 4700.07 | 13.13281 |  |  |  |  |  |
| 10.60833 | | 139.7213 | 0.226735 | 4699.938 | 13.03906 |  |  |  |  |  |
| 10.61667 | | 139.8138 | 0.228273 | 4699.828 | 12.99219 |  |  |  |  |  |
| 10.625 | | 139.9007 | 0.230861 | 4699.719 | 12.73438 |  |  |  |  |  |
| 10.63333 | | 139.9877 | 0.23237 | 4699.586 | 12.59375 |  |  |  |  |  |
| 10.64167 | | 140.0792 | 0.234134 | 4699.477 | 12.63281 |  |  |  |  |  |
| 10.65 | | 140.1662 | 0.236713 | 4699.414 | 12.42188 |  |  |  |  |  |
| 10.65833 | | 140.2559 | 0.238206 | 4699.328 | 12.17188 |  |  |  |  |  |
| 10.66667 | | 140.3391 | 0.23973 | 4699.227 | 11.82813 |  |  |  |  |  |
| 10.675 | | 140.4279 | 0.242698 | 4699.156 | 11.70313 |  |  |  |  |  |
| 10.68333 | | 140.5186 | 0.2446 | 4699.07 | 11.89844 |  |  |  |  |  |
| 10.69167 | | 140.6074 | 0.246661 | 4698.953 | 11.94531 |  |  |  |  |  |
| 10.7 | | 140.6934 | 0.249253 | 4698.883 | 11.77344 |  |  |  |  |  |
| 10.70833 | | 140.7822 | 0.250583 | 4698.805 | 12.0625 |  |  |  |  |  |
| 10.71667 | | 140.8738 | 0.252819 | 4698.766 | 12.07813 |  |  |  |  |  |
| 10.725 | | 140.9579 | 0.255156 | 4698.672 | 11.9375 |  |  |  |  |  |
| 10.73333 | | 141.0467 | 0.256214 | 4698.547 | 12.10938 |  |  |  |  |  |
| 10.74167 | | 141.1309 | 0.257894 | 4698.438 | 11.85938 |  |  |  |  |  |
| 10.75 | | 141.2206 | 0.26029 | 4698.359 | 12.08594 |  |  |  |  |  |
| 10.75833 | | 141.3113 | 0.261323 | 4698.219 | 12.14844 |  |  |  |  |  |
| 10.76667 | | 141.3982 | 0.262821 | 4698.125 | 12.16406 |  |  |  |  |  |
| 10.775 | | 141.4852 | 0.264642 | 4698.047 | 12.24219 |  |  |  |  |  |
| 10.78333 | | 141.5712 | 0.265377 | 4697.938 | 12.28125 |  |  |  |  |  |
| 10.79167 | | 141.6581 | 0.267185 | 4697.852 | 12.125 |  |  |  |  |  |
| 10.8 | | 141.7441 | 0.269118 | 4697.727 | 11.95313 |  |  |  |  |  |
| 10.80833 | | 141.832 | 0.269843 | 4697.617 | 11.9375 |  |  |  |  |  |
| 10.81667 | | 141.919 | 0.271674 | 4697.516 | 11.89844 |  |  |  |  |  |
| 10.825 | | 142.005 | 0.273859 | 4697.414 | 11.91406 |  |  |  |  |  |
| 10.83333 | | 142.0965 | 0.274899 | 4697.297 | 11.85938 |  |  |  |  |  |
| 10.84167 | | 142.1853 | 0.276581 | 4697.227 | 11.38281 |  |  |  |  |  |
| 10.85 | | 142.2732 | 0.278686 | 4697.148 | 11.20313 |  |  |  |  |  |
| 10.85833 | | 142.3611 | 0.279508 | 4697.047 | 11.01563 |  |  |  |  |  |
| 10.86667 | | 142.4517 | 0.281601 | 4696.953 | 10.80469 |  |  |  |  |  |
| 10.875 | | 142.5405 | 0.283205 | 4696.852 | 10.75781 |  |  |  |  |  |
| 10.88333 | | 142.6275 | 0.283805 | 4696.766 | 11.11719 |  |  |  |  |  |
| 10.89167 | | 142.7181 | 0.286283 | 4696.727 | 11.13281 |  |  |  |  |  |
| 10.9 | | 142.8069 | 0.288301 | 4696.664 | 11.03906 |  |  |  |  |  |
| 10.90833 | | 142.8957 | 0.28918 | 4696.586 | 10.73438 |  |  |  |  |  |
| 10.91667 | | 142.9808 | 0.291692 | 4696.523 | 10.55469 |  |  |  |  |  |
| 10.925 | | 143.0677 | 0.29345 | 4696.43 | 10.28125 |  |  |  |  |  |
| 10.93333 | | 143.1547 | 0.294308 | 4696.281 | 9.96875 |  |  |  |  |  |
| 10.94167 | | 143.2463 | 0.297359 | 4696.188 | 9.6875 |  |  |  |  |  |
| 10.95 | | 143.3351 | 0.299562 | 4696.102 | 9.460938 |  |  |  |  |  |
| 10.95833 | | 143.4238 | 0.300742 | 4696.055 | 9.085938 |  |  |  |  |  |
| 10.96667 | | 143.5126 | 0.30353 | 4695.992 | 8.414063 |  |  |  |  |  |
| 10.975 | | 143.6024 | 0.304921 | 4695.938 | 8.09375 |  |  |  |  |  |
| 10.98333 | | 143.6921 | 0.30558 | 4695.891 | 7.992188 |  |  |  |  |  |
| 10.99167 | | 143.7827 | 0.308453 | 4695.852 | 7.929688 |  |  |  |  |  |
| 11 | | 143.866 | 0.309521 | 4695.805 | 7.859375 |  |  |  |  |  |
| 11.00833 | | 143.9511 | 0.310399 | 4695.781 | 8.03125 |  |  |  |  |  |
| 11.01667 | | 144.0408 | 0.313507 | 4695.797 | 8.117188 |  |  |  |  |  |
| 11.025 | | 144.1268 | 0.3148 | 4695.781 | 8.085938 |  |  |  |  |  |
| 11.03333 | | 144.2128 | 0.315351 | 4695.727 | 8.070313 |  |  |  |  |  |
| 11.04167 | | 144.297 | 0.317719 | 4695.664 | 8.164063 |  |  |  |  |  |
| 11.05 | | 144.3839 | 0.319288 | 4695.602 | 8.328125 |  |  |  |  |  |
| 11.05833 | | 144.469 | 0.31993 | 4695.516 | 8.242188 |  |  |  |  |  |
| 11.06667 | | 144.5532 | 0.322061 | 4695.438 | 8.140625 |  |  |  |  |  |
| 11.075 | | 144.6392 | 0.323609 | 4695.367 | 8.140625 |  |  |  |  |  |
| 11.08333 | | 144.7327 | 0.324615 | 4695.289 | 7.882813 |  |  |  |  |  |
| 11.09167 | | 144.8242 | 0.327187 | 4695.211 | 7.789063 |  |  |  |  |  |
| 11.1 | | 144.9121 | 0.328342 | 4695.109 | 7.859375 |  |  |  |  |  |
| 11.10833 | | 145 | 0.329038 | 4695.055 | 7.757813 |  |  |  |  |  |
| 11.11667 | | 145.0869 | 0.331352 | 4694.992 | 7.742188 |  |  |  |  |  |
| 11.125 | | 145.1794 | 0.332884 | 4694.922 | 7.835938 |  |  |  |  |  |
| 11.13333 | | 145.2701 | 0.333588 | 4694.891 | 7.742188 |  |  |  |  |  |
| 11.14167 | | 145.357 | 0.336029 | 4694.836 | 8 |  |  |  |  |  |
| 11.15 | | 145.4458 | 0.337111 | 4694.75 | 8.453125 |  |  |  |  |  |
| 11.15833 | | 145.5355 | 0.338013 | 4694.695 | 8.765625 |  |  |  |  |  |
| 11.16667 | | 145.6251 | 0.340669 | 4694.625 | 9.320313 |  |  |  |  |  |
| 11.175 | | 145.7108 | 0.341802 | 4694.547 | 9.640625 |  |  |  |  |  |
| 11.18333 | | 145.7965 | 0.3428 | 4694.492 | 9.773438 |  |  |  |  |  |
| 11.19167 | | 145.8812 | 0.345441 | 4694.383 | 9.984375 |  |  |  |  |  |
| 11.2 | | 145.9696 | 0.346847 | 4694.25 | 10.27344 |  |  |  |  |  |
| 11.20833 | | 146.0571 | 0.348173 | 4694.117 | 10.40625 |  |  |  |  |  |
| 11.21667 | | 146.1427 | 0.351418 | 4693.969 | 10.47656 |  |  |  |  |  |
| 11.225 | | 146.2338 | 0.352854 | 4693.844 | 10.36719 |  |  |  |  |  |
| 11.23333 | | 146.3222 | 0.354315 | 4693.734 | 10.17188 |  |  |  |  |  |
| 11.24167 | | 146.4079 | 0.357494 | 4693.633 | 9.851563 |  |  |  |  |  |
| 11.25 | | 146.4935 | 0.358379 | 4693.508 | 9.664063 |  |  |  |  |  |
| 11.25833 | | 146.5801 | 0.359818 | 4693.398 | 9.59375 |  |  |  |  |  |
| 11.26667 | | 146.6667 | 0.362853 | 4693.305 | 9.453125 |  |  |  |  |  |
| 11.275 | | 146.7542 | 0.363692 | 4693.234 | 9.382813 |  |  |  |  |  |
| 11.28333 | | 146.8425 | 0.36487 | 4693.172 | 8.851563 |  |  |  |  |  |
| 11.29167 | | 146.9282 | 0.36761 | 4693.133 | 8.773438 |  |  |  |  |  |
| 11.3 | | 147.0166 | 0.367952 | 4693.078 | 8.59375 |  |  |  |  |  |
| 11.30833 | | 147.1013 | 0.368967 | 4693.008 | 8.640625 |  |  |  |  |  |
| 11.31667 | | 147.1924 | 0.371991 | 4692.953 | 8.625 |  |  |  |  |  |
| 11.325 | | 147.2817 | 0.372517 | 4692.898 | 8.648438 |  |  |  |  |  |
| 11.33333 | | 147.3665 | 0.373785 | 4692.891 | 8.84375 |  |  |  |  |  |
| 11.34167 | | 147.4548 | 0.376332 | 4692.836 | 8.984375 |  |  |  |  |  |
| 11.35 | | 147.5432 | 0.376568 | 4692.797 | 9.257813 |  |  |  |  |  |
| 11.35833 | | 147.6307 | 0.378188 | 4692.719 | 9.367188 |  |  |  |  |  |
| 11.36667 | | 147.7182 | 0.380959 | 4692.656 | 9.71875 |  |  |  |  |  |
| 11.375 | | 147.8056 | 0.381399 | 4692.594 | 9.679688 |  |  |  |  |  |
| 11.38333 | | 147.8886 | 0.382638 | 4692.492 | 9.890625 |  |  |  |  |  |
| 11.39167 | | 147.977 | 0.385622 | 4692.398 | 9.78125 |  |  |  |  |  |
| 11.4 | | 148.0617 | 0.386041 | 4692.289 | 9.648438 |  |  |  |  |  |
| 11.40833 | | 148.1474 | 0.387883 | 4692.188 | 9.640625 |  |  |  |  |  |
| 11.41667 | | 148.2349 | 0.390913 | 4692.055 | 9.226563 |  |  |  |  |  |
| 11.425 | | 148.3242 | 0.391485 | 4691.984 | 8.679688 |  |  |  |  |  |
| 11.43333 | | 148.4134 | 0.393158 | 4691.875 | 8.21875 |  |  |  |  |  |
| 11.44167 | | 148.5027 | 0.395722 | 4691.805 | 7.742188 |  |  |  |  |  |
| 11.45 | | 148.5893 | 0.395919 | 4691.734 | 7.078125 |  |  |  |  |  |
| 11.45833 | | 148.6759 | 0.397824 | 4691.672 | 6.796875 |  |  |  |  |  |
| 11.46667 | | 148.7651 | 0.400733 | 4691.648 | 6.21875 |  |  |  |  |  |
| 11.475 | | 148.8535 | 0.401575 | 4691.656 | 5.867188 |  |  |  |  |  |
| 11.48333 | | 148.9383 | 0.403759 | 4691.664 | 5.3125 |  |  |  |  |  |
| 11.49167 | | 149.0275 | 0.405852 | 4691.664 | 5.046875 |  |  |  |  |  |
| 11.5 | | 149.115 | 0.406536 | 4691.688 | 5.046875 |  |  |  |  |  |
| 11.50833 | | 149.2016 | 0.408524 | 4691.68 | 5.125 |  |  |  |  |  |
| 11.51667 | | 149.2846 | 0.410365 | 4691.703 | 5.148438 |  |  |  |  |  |
| 11.525 | | 149.3702 | 0.410763 | 4691.703 | 5.507813 |  |  |  |  |  |
| 11.53333 | | 149.4577 | 0.412521 | 4691.742 | 5.609375 |  |  |  |  |  |
| 11.54167 | | 149.5452 | 0.414044 | 4691.719 | 5.804688 |  |  |  |  |  |
| 11.55 | | 149.6353 | 0.414581 | 4691.672 | 6.242188 |  |  |  |  |  |
| 11.55833 | | 149.7183 | 0.416315 | 4691.602 | 6.414063 |  |  |  |  |  |
| 11.56667 | | 149.8085 | 0.418204 | 4691.547 | 7.007813 |  |  |  |  |  |
| 11.575 | | 149.8951 | 0.418621 | 4691.445 | 7.210938 |  |  |  |  |  |
| 11.58333 | | 149.9843 | 0.420718 | 4691.375 | 7.414063 |  |  |  |  |  |
| 11.59167 | | 150.0691 | 0.422555 | 4691.289 | 7.65625 |  |  |  |  |  |
| 11.6 | | 150.1593 | 0.423635 | 4691.164 | 7.945313 |  |  |  |  |  |
| 11.60833 | | 150.2495 | 0.426379 | 4691.078 | 7.945313 |  |  |  |  |  |
| 11.61667 | | 150.3378 | 0.428172 | 4690.93 | 8.148438 |  |  |  |  |  |
| 11.625 | | 150.4235 | 0.429331 | 4690.836 | 8.296875 |  |  |  |  |  |
| 11.63333 | | 150.5092 | 0.431514 | 4690.75 | 8.179688 |  |  |  |  |  |
| 11.64167 | | 150.6012 | 0.43293 | 4690.656 | 8.289063 |  |  |  |  |  |
| 11.65 | | 150.6886 | 0.433721 | 4690.547 | 8.140625 |  |  |  |  |  |
| 11.65833 | | 150.7761 | 0.435784 | 4690.484 | 7.953125 |  |  |  |  |  |
| 11.66667 | | 150.8645 | 0.437174 | 4690.383 | 7.664063 |  |  |  |  |  |
| 11.675 | | 150.951 | 0.438195 | 4690.305 | 7.515625 |  |  |  |  |  |
| 11.68333 | | 151.0385 | 0.44048 | 4690.258 | 7.414063 |  |  |  |  |  |
| 11.69167 | | 151.126 | 0.441644 | 4690.172 | 7.554688 |  |  |  |  |  |
| 11.7 | | 151.2117 | 0.442495 | 4690.125 | 7.59375 |  |  |  |  |  |
| 11.70833 | | 151.2991 | 0.444692 | 4690.086 | 7.609375 |  |  |  |  |  |
| 11.71667 | | 151.3875 | 0.44587 | 4690.063 | 7.617188 |  |  |  |  |  |
| 11.725 | | 151.475 | 0.446706 | 4690.023 | 7.679688 |  |  |  |  |  |
| 11.73333 | | 151.5634 | 0.448641 | 4689.969 | 7.773438 |  |  |  |  |  |
| 11.74167 | | 151.6508 | 0.449922 | 4689.875 | 7.90625 |  |  |  |  |  |
| 11.75 | | 151.7383 | 0.450818 | 4689.805 | 8.289063 |  |  |  |  |  |
| 11.75833 | | 151.8276 | 0.453251 | 4689.727 | 8.304688 |  |  |  |  |  |
| 11.76667 | | 151.916 | 0.45461 | 4689.656 | 8.195313 |  |  |  |  |  |
| 11.775 | | 152.0061 | 0.455845 | 4689.594 | 8.15625 |  |  |  |  |  |
| 11.78333 | | 152.0936 | 0.45828 | 4689.508 | 8.109375 |  |  |  |  |  |
| 11.79167 | | 152.1856 | 0.459814 | 4689.422 | 8.140625 |  |  |  |  |  |
| 11.8 | | 152.2758 | 0.461115 | 4689.297 | 8.335938 |  |  |  |  |  |
| 11.80833 | | 152.3623 | 0.463157 | 4689.234 | 8.335938 |  |  |  |  |  |
| 11.81667 | | 152.4471 | 0.464007 | 4689.18 | 8.5625 |  |  |  |  |  |
| 11.825 | | 152.5355 | 0.465252 | 4689.133 | 8.554688 |  |  |  |  |  |
| 11.83333 | | 152.6257 | 0.467471 | 4689.07 | 8.609375 |  |  |  |  |  |
| 11.84167 | | 152.7149 | 0.468083 | 4689.008 | 8.539063 |  |  |  |  |  |
| 11.85 | | 152.8006 | 0.46952 | 4688.922 | 8.59375 |  |  |  |  |  |
| 11.85833 | | 152.8854 | 0.471376 | 4688.859 | 8.429688 |  |  |  |  |  |
| 11.86667 | | 152.9746 | 0.471658 | 4688.773 | 8.1875 |  |  |  |  |  |
| 11.875 | | 153.063 | 0.472712 | 4688.711 | 7.945313 |  |  |  |  |  |
| 11.88333 | | 153.1523 | 0.474669 | 4688.648 | 7.875 |  |  |  |  |  |
| 11.89167 | | 153.2407 | 0.475377 | 4688.586 | 7.875 |  |  |  |  |  |
| 11.9 | | 153.3326 | 0.477288 | 4688.508 | 7.476563 |  |  |  |  |  |
| 11.90833 | | 153.4228 | 0.479361 | 4688.461 | 7.304688 |  |  |  |  |  |
| 11.91667 | | 153.5085 | 0.479543 | 4688.43 | 6.96875 |  |  |  |  |  |
| 11.925 | | 153.5942 | 0.480964 | 4688.391 | 6.867188 |  |  |  |  |  |
| 11.93333 | | 153.6807 | 0.482495 | 4688.336 | 6.601563 |  |  |  |  |  |
| 11.94167 | | 153.77 | 0.482784 | 4688.273 | 6.8125 |  |  |  |  |  |
| 11.95 | | 153.8602 | 0.483941 | 4688.258 | 6.835938 |  |  |  |  |  |
| 11.95833 | | 153.9477 | 0.485674 | 4688.219 | 6.945313 |  |  |  |  |  |
| 11.96667 | | 154.0306 | 0.485981 | 4688.195 | 6.929688 |  |  |  |  |  |
| 11.975 | | 154.1208 | 0.487129 | 4688.148 | 6.664063 |  |  |  |  |  |
| 11.98333 | | 154.2101 | 0.488828 | 4688.125 | 6.632813 |  |  |  |  |  |
| 11.99167 | | 154.2939 | 0.489032 | 4688.031 | 6.625 |  |  |  |  |  |
| 12 | | 154.3805 | 0.490268 | 4687.969 | 6.65625 |  |  |  |  |  |
| 12.00833 | | 154.468 | 0.492076 | 4687.891 | 6.859375 |  |  |  |  |  |
| 12.01667 | | 154.5582 | 0.49241 | 4687.836 | 7.0625 |  |  |  |  |  |
| 12.025 | | 154.6447 | 0.493468 | 4687.813 | 6.828125 |  |  |  |  |  |
| 12.03333 | | 154.7295 | 0.495053 | 4687.766 | 6.671875 |  |  |  |  |  |
| 12.04167 | | 154.817 | 0.495604 | 4687.703 | 6.609375 |  |  |  |  |  |
| 12.05 | | 154.9044 | 0.49691 | 4687.648 | 6.601563 |  |  |  |  |  |
| 12.05833 | | 154.9892 | 0.498663 | 4687.555 | 6.78125 |  |  |  |  |  |
| 12.06667 | | 155.0731 | 0.499442 | 4687.477 | 7.03125 |  |  |  |  |  |
| 12.075 | | 155.1597 | 0.500552 | 4687.461 | 7.140625 |  |  |  |  |  |
| 12.08333 | | 155.2471 | 0.502854 | 4687.422 | 7.171875 |  |  |  |  |  |
| 12.09167 | | 155.3373 | 0.503635 | 4687.375 | 6.953125 |  |  |  |  |  |
| 12.1 | | 155.423 | 0.504792 | 4687.313 | 6.828125 |  |  |  |  |  |
| 12.10833 | | 155.5104 | 0.507139 | 4687.227 | 7.007813 |  |  |  |  |  |
| 12.11667 | | 155.595 | 0.508031 | 4687.133 | 7.070313 |  |  |  |  |  |
| 12.125 | | 155.6831 | 0.509777 | 4687.055 | 6.859375 |  |  |  |  |  |
| 12.13333 | | 155.7713 | 0.511864 | 4686.992 | 6.851563 |  |  |  |  |  |
| 12.14167 | | 155.8594 | 0.512436 | 4686.961 | 6.757813 |  |  |  |  |  |
| 12.15 | | 155.9484 | 0.514098 | 4686.914 | 6.773438 |  |  |  |  |  |
| 12.15833 | | 156.0383 | 0.515427 | 4686.828 | 6.59375 |  |  |  |  |  |
| 12.16667 | | 156.1265 | 0.515543 | 4686.766 | 6.664063 |  |  |  |  |  |
| 12.175 | | 156.2137 | 0.517308 | 4686.727 | 6.585938 |  |  |  |  |  |
| 12.18333 | | 156.3027 | 0.518554 | 4686.68 | 6.460938 |  |  |  |  |  |
| 12.19167 | | 156.39 | 0.518592 | 4686.633 | 6.34375 |  |  |  |  |  |
| 12.2 | | 156.4816 | 0.520188 | 4686.57 | 6.664063 |  |  |  |  |  |
| 12.20833 | | 156.5671 | 0.521132 | 4686.539 | 7.070313 |  |  |  |  |  |
| 12.21667 | | 156.6535 | 0.521148 | 4686.469 | 7.132813 |  |  |  |  |  |
| 12.225 | | 156.7407 | 0.522762 | 4686.43 | 7.492188 |  |  |  |  |  |
| 12.23333 | | 156.8288 | 0.524011 | 4686.383 | 7.453125 |  |  |  |  |  |
| 12.24167 | | 156.9196 | 0.524175 | 4686.352 | 7.515625 |  |  |  |  |  |
| 12.25 | | 157.0095 | 0.526495 | 4686.25 | 7.671875 |  |  |  |  |  |
| 12.25833 | | 157.0968 | 0.527669 | 4686.133 | 7.835938 |  |  |  |  |  |
| 12.26667 | | 157.1849 | 0.528073 | 4686.07 | 7.765625 |  |  |  |  |  |
| 12.275 | | 157.2739 | 0.53035 | 4685.953 | 7.726563 |  |  |  |  |  |
| 12.28333 | | 157.362 | 0.531555 | 4685.898 | 7.34375 |  |  |  |  |  |
| 12.29167 | | 157.4493 | 0.532158 | 4685.828 | 6.757813 |  |  |  |  |  |
| 12.3 | | 157.5374 | 0.534521 | 4685.742 | 6.5625 |  |  |  |  |  |
| 12.30833 | | 157.6247 | 0.535979 | 4685.656 | 6.164063 |  |  |  |  |  |
| 12.31667 | | 157.7128 | 0.536237 | 4685.609 | 6.242188 |  |  |  |  |  |
| 12.325 | | 157.7991 | 0.538019 | 4685.555 | 6.375 |  |  |  |  |  |
| 12.33333 | | 157.8882 | 0.538831 | 4685.555 | 5.929688 |  |  |  |  |  |
| 12.34167 | | 157.9736 | 0.53889 | 4685.578 | 6.210938 |  |  |  |  |  |
| 12.35 | | 158.0626 | 0.541131 | 4685.547 | 6.421875 |  |  |  |  |  |
| 12.35833 | | 158.1517 | 0.542154 | 4685.547 | 6.421875 |  |  |  |  |  |
| 12.36667 | | 158.238 | 0.542274 | 4685.477 | 6.53125 |  |  |  |  |  |
| 12.375 | | 158.327 | 0.544135 | 4685.422 | 6.820313 |  |  |  |  |  |
| 12.38333 | | 158.4187 | 0.545048 | 4685.422 | 6.773438 |  |  |  |  |  |
| 12.39167 | | 158.5077 | 0.545105 | 4685.336 | 6.867188 |  |  |  |  |  |
| 12.4 | | 158.5941 | 0.547294 | 4685.258 | 6.515625 |  |  |  |  |  |
| 12.40833 | | 158.6813 | 0.548159 | 4685.195 | 6.429688 |  |  |  |  |  |
| 12.41667 | | 158.7659 | 0.548624 | 4685.125 | 6.601563 |  |  |  |  |  |
| 12.425 | | 158.8558 | 0.550847 | 4685.031 | 6.359375 |  |  |  |  |  |
| 12.43333 | | 158.9448 | 0.551473 | 4684.977 | 6.296875 |  |  |  |  |  |
| 12.44167 | | 159.0321 | 0.551688 | 4684.922 | 6.515625 |  |  |  |  |  |
| 12.45 | | 159.1211 | 0.554121 | 4684.906 | 6.570313 |  |  |  |  |  |
| 12.45833 | | 159.2092 | 0.554803 | 4684.852 | 6.578125 |  |  |  |  |  |
| 12.46667 | | 159.2956 | 0.555013 | 4684.773 | 6.773438 |  |  |  |  |  |
| 12.475 | | 159.3846 | 0.557338 | 4684.758 | 6.851563 |  |  |  |  |  |
| 12.48333 | | 159.4736 | 0.557855 | 4684.711 | 6.835938 |  |  |  |  |  |
| 12.49167 | | 159.5608 | 0.558373 | 4684.633 | 6.851563 |  |  |  |  |  |
| 12.5 | | 159.6507 | 0.560588 | 4684.57 | 6.78125 |  |  |  |  |  |
| 12.50833 | | 159.7398 | 0.560995 | 4684.508 | 6.617188 |  |  |  |  |  |
| 12.51667 | | 159.8297 | 0.561732 | 4684.414 | 6.664063 |  |  |  |  |  |
| 12.525 | | 159.9169 | 0.564608 | 4684.359 | 6.398438 |  |  |  |  |  |
| 12.53333 | | 160.0068 | 0.565495 | 4684.297 | 6.210938 |  |  |  |  |  |
| 12.54167 | | 160.0958 | 0.566582 | 4684.242 | 6.085938 |  |  |  |  |  |
| 12.55 | | 160.1831 | 0.569631 | 4684.195 | 5.734375 |  |  |  |  |  |
| 12.55833 | | 160.2703 | 0.569853 | 4684.164 | 5.65625 |  |  |  |  |  |
| 12.56667 | | 160.3558 | 0.570781 | 4684.102 | 5.507813 |  |  |  |  |  |
| 12.575 | | 160.4448 | 0.573476 | 4684.094 | 5.390625 |  |  |  |  |  |
| 12.58333 | | 160.5312 | 0.573168 | 4684.063 | 5.234375 |  |  |  |  |  |
| 12.59167 | | 160.6184 | 0.574003 | 4684.031 | 5.3125 |  |  |  |  |  |
| 12.6 | | 160.7003 | 0.57626 | 4684.023 | 5.234375 |  |  |  |  |  |
| 12.60833 | | 160.7893 | 0.575985 | 4683.984 | 5.289063 |  |  |  |  |  |
| 12.61667 | | 160.8766 | 0.576558 | 4683.961 | 5.398438 |  |  |  |  |  |
| 12.625 | | 160.9647 | 0.578865 | 4683.914 | 5.421875 |  |  |  |  |  |
| 12.63333 | | 161.0546 | 0.578921 | 4683.898 | 5.515625 |  |  |  |  |  |
| 12.64167 | | 161.1418 | 0.579716 | 4683.828 | 5.757813 |  |  |  |  |  |
| 12.65 | | 161.23 | 0.582045 | 4683.805 | 6.046875 |  |  |  |  |  |
| 12.65833 | | 161.3181 | 0.581914 | 4683.75 | 6.023438 |  |  |  |  |  |
| 12.66667 | | 161.408 | 0.583038 | 4683.68 | 6.3125 |  |  |  |  |  |
| 12.675 | | 161.4979 | 0.585442 | 4683.633 | 6.476563 |  |  |  |  |  |
| 12.68333 | | 161.5905 | 0.585374 | 4683.578 | 6.71875 |  |  |  |  |  |
| 12.69167 | | 161.6795 | 0.586477 | 4683.508 | 6.53125 |  |  |  |  |  |
| 12.7 | | 161.765 | 0.588713 | 4683.422 | 6.375 |  |  |  |  |  |
| 12.70833 | | 161.8504 | 0.58853 | 4683.367 | 6.273438 |  |  |  |  |  |
| 12.71667 | | 161.9368 | 0.589354 | 4683.281 | 6.179688 |  |  |  |  |  |
| 12.725 | | 162.0258 | 0.591515 | 4683.203 | 6.101563 |  |  |  |  |  |
| 12.73333 | | 162.1184 | 0.591583 | 4683.117 | 5.875 |  |  |  |  |  |
| 12.74167 | | 162.2038 | 0.592889 | 4683.078 | 5.679688 |  |  |  |  |  |
| 12.75 | | 162.2902 | 0.594997 | 4683.047 | 5.445313 |  |  |  |  |  |
| 12.75833 | | 162.3792 | 0.595018 | 4683.016 | 5.046875 |  |  |  |  |  |
| 12.76667 | | 162.4673 | 0.596311 | 4682.977 | 4.804688 |  |  |  |  |  |
| 12.775 | | 162.5528 | 0.598365 | 4682.93 | 5 |  |  |  |  |  |
| 12.78333 | | 162.6445 | 0.598416 | 4682.914 | 4.953125 |  |  |  |  |  |
| 12.79167 | | 162.7362 | 0.599668 | 4682.891 | 4.945313 |  |  |  |  |  |
| 12.8 | | 162.8261 | 0.60164 | 4682.883 | 4.90625 |  |  |  |  |  |
| 12.80833 | | 162.9178 | 0.601277 | 4682.891 | 4.640625 |  |  |  |  |  |
| 12.81667 | | 163.0032 | 0.602564 | 4682.883 | 4.5 |  |  |  |  |  |
| 12.825 | | 163.094 | 0.604948 | 4682.813 | 4.59375 |  |  |  |  |  |
| 12.83333 | | 163.1848 | 0.604998 | 4682.781 | 4.453125 |  |  |  |  |  |
| 12.84167 | | 163.2721 | 0.606733 | 4682.734 | 4.3125 |  |  |  |  |  |
| 12.85 | | 163.3602 | 0.608865 | 4682.695 | 4.1875 |  |  |  |  |  |
| 12.85833 | | 163.4492 | 0.608194 | 4682.695 | 3.976563 |  |  |  |  |  |
| 12.86667 | | 163.5374 | 0.609168 | 4682.672 | 3.90625 |  |  |  |  |  |
| 12.875 | | 163.6228 | 0.610563 | 4682.617 | 3.953125 |  |  |  |  |  |
| 12.88333 | | 163.7136 | 0.609929 | 4682.594 | 4.179688 |  |  |  |  |  |
| 12.89167 | | 163.7991 | 0.611047 | 4682.57 | 4.289063 |  |  |  |  |  |
| 12.9 | | 163.8916 | 0.612842 | 4682.555 | 4.484375 |  |  |  |  |  |
| 12.90833 | | 163.9825 | 0.612176 | 4682.555 | 4.4375 |  |  |  |  |  |
| 12.91667 | | 164.0679 | 0.613553 | 4682.523 | 4.898438 |  |  |  |  |  |
| 12.925 | | 164.1587 | 0.614972 | 4682.484 | 5.21875 |  |  |  |  |  |
| 12.93333 | | 164.2486 | 0.614232 | 4682.422 | 5.65625 |  |  |  |  |  |
| 12.94167 | | 164.343 | 0.615952 | 4682.375 | 5.960938 |  |  |  |  |  |
| 12.95 | | 164.4302 | 0.617552 | 4682.313 | 6.367188 |  |  |  |  |  |
| 12.95833 | | 164.5201 | 0.617685 | 4682.281 | 6.367188 |  |  |  |  |  |
| 12.96667 | | 164.6065 | 0.619696 | 4682.18 | 6.3125 |  |  |  |  |  |
| 12.975 | | 164.6964 | 0.621683 | 4682.086 | 6.40625 |  |  |  |  |  |
| 12.98333 | | 164.7827 | 0.621527 | 4681.977 | 6.414063 |  |  |  |  |  |
| 12.99167 | | 164.8727 | 0.623584 | 4681.883 | 6.453125 |  |  |  |  |  |
| 13 | | 164.9634 | 0.625241 | 4681.773 | 6.148438 |  |  |  |  |  |
| 13.00833 | | 165.0525 | 0.625235 | 4681.719 | 6.0625 |  |  |  |  |  |
| 13.01667 | | 165.1424 | 0.627318 | 4681.672 | 5.890625 |  |  |  |  |  |
| 13.025 | | 165.2296 | 0.628883 | 4681.609 | 5.789063 |  |  |  |  |  |
| 13.03333 | | 165.3166 | 0.62874 | 4681.555 | 5.609375 |  |  |  |  |  |
| 13.04167 | | 165.4049 | 0.631104 | 4681.492 | 5.617188 |  |  |  |  |  |
| 13.05 | | 165.4932 | 0.632658 | 4681.484 | 5.609375 |  |  |  |  |  |
| 13.05833 | | 165.5807 | 0.632719 | 4681.453 | 5.632813 |  |  |  |  |  |
| 13.06667 | | 165.6673 | 0.634615 | 4681.414 | 5.507813 |  |  |  |  |  |
| 13.075 | | 165.7512 | 0.635265 | 4681.375 | 5.25 |  |  |  |  |  |
| 13.08333 | | 165.8386 | 0.635157 | 4681.352 | 5.234375 |  |  |  |  |  |
| 13.09167 | | 165.9226 | 0.636754 | 4681.305 | 5.085938 |  |  |  |  |  |
| 13.1 | | 166.01 | 0.637874 | 4681.258 | 4.859375 |  |  |  |  |  |
| 13.10833 | | 166.0948 | 0.637984 | 4681.219 | 4.523438 |  |  |  |  |  |
| 13.11667 | | 166.1832 | 0.64023 | 4681.188 | 4.320313 |  |  |  |  |  |
| 13.125 | | 166.2688 | 0.641316 | 4681.172 | 4.054688 |  |  |  |  |  |
| 13.13333 | | 166.3537 | 0.641127 | 4681.133 | 4.007813 |  |  |  |  |  |
| 13.14167 | | 166.4394 | 0.643232 | 4681.117 | 4.15625 |  |  |  |  |  |
| 13.15 | | 166.5268 | 0.644372 | 4681.109 | 4.15625 |  |  |  |  |  |
| 13.15833 | | 166.6151 | 0.644317 | 4681.133 | 4.1875 |  |  |  |  |  |
| 13.16667 | | 166.7026 | 0.646464 | 4681.125 | 4.28125 |  |  |  |  |  |
| 13.175 | | 166.79 | 0.647843 | 4681.125 | 4.414063 |  |  |  |  |  |
| 13.18333 | | 166.8748 | 0.647571 | 4681.102 | 4.476563 |  |  |  |  |  |
| 13.19167 | | 166.964 | 0.650001 | 4681.039 | 4.703125 |  |  |  |  |  |
| 13.2 | | 167.0549 | 0.651132 | 4681.016 | 4.828125 |  |  |  |  |  |
| 13.20833 | | 167.1441 | 0.65114 | 4680.969 | 4.953125 |  |  |  |  |  |
| 13.21667 | | 167.2307 | 0.652865 | 4680.914 | 5.039063 |  |  |  |  |  |
| 13.225 | | 167.3155 | 0.653363 | 4680.867 | 4.992188 |  |  |  |  |  |
| 13.23333 | | 167.4047 | 0.653173 | 4680.828 | 5.210938 |  |  |  |  |  |
| 13.24167 | | 167.4948 | 0.654955 | 4680.75 | 5.320313 |  |  |  |  |  |
| 13.25 | | 167.584 | 0.655612 | 4680.695 | 5.460938 |  |  |  |  |  |
| 13.25833 | | 167.6723 | 0.655783 | 4680.641 | 5.898438 |  |  |  |  |  |
| 13.26667 | | 167.7615 | 0.657966 | 4680.586 | 6.054688 |  |  |  |  |  |
| 13.275 | | 167.8489 | 0.658918 | 4680.555 | 6.226563 |  |  |  |  |  |
| 13.28333 | | 167.9337 | 0.658964 | 4680.477 | 6.203125 |  |  |  |  |  |
| 13.29167 | | 168.0221 | 0.661578 | 4680.414 | 6.398438 |  |  |  |  |  |
| 13.3 | | 168.1121 | 0.662575 | 4680.344 | 6.296875 |  |  |  |  |  |
| 13.30833 | | 168.2013 | 0.662997 | 4680.234 | 6.390625 |  |  |  |  |  |
| 13.31667 | | 168.2879 | 0.665465 | 4680.156 | 6.3125 |  |  |  |  |  |
| 13.325 | | 168.3727 | 0.666582 | 4680.07 | 6.34375 |  |  |  |  |  |
| 13.33333 | | 168.4584 | 0.66682 | 4680.023 | 6.210938 |  |  |  |  |  |
| 13.34167 | | 168.545 | 0.669055 | 4679.938 | 5.835938 |  |  |  |  |  |
| 13.35 | | 168.6342 | 0.669687 | 4679.891 | 6.125 |  |  |  |  |  |
| 13.35833 | | 168.7207 | 0.669376 | 4679.82 | 5.867188 |  |  |  |  |  |
| 13.36667 | | 168.8099 | 0.671464 | 4679.781 | 6.0625 |  |  |  |  |  |
| 13.375 | | 168.8974 | 0.671438 | 4679.727 | 6.054688 |  |  |  |  |  |
| 13.38333 | | 168.9865 | 0.671167 | 4679.688 | 6.289063 |  |  |  |  |  |
| 13.39167 | | 169.0775 | 0.673277 | 4679.68 | 6.25 |  |  |  |  |  |
| 13.4 | | 169.1658 | 0.673537 | 4679.609 | 6.117188 |  |  |  |  |  |
| 13.40833 | | 169.2559 | 0.673547 | 4679.594 | 6.046875 |  |  |  |  |  |
| 13.41667 | | 169.3477 | 0.675691 | 4679.508 | 5.984375 |  |  |  |  |  |
| 13.425 | | 169.4377 | 0.676204 | 4679.469 | 6.15625 |  |  |  |  |  |
| 13.43333 | | 169.5234 | 0.676677 | 4679.391 | 5.914063 |  |  |  |  |  |
| 13.44167 | | 169.6118 | 0.678679 | 4679.344 | 5.84375 |  |  |  |  |  |
| 13.45 | | 169.6992 | 0.678697 | 4679.313 | 5.820313 |  |  |  |  |  |
| 13.45833 | | 169.7866 | 0.678973 | 4679.266 | 5.960938 |  |  |  |  |  |
| 13.46667 | | 169.8741 | 0.680853 | 4679.227 | 5.789063 |  |  |  |  |  |
| 13.475 | | 169.9615 | 0.68119 | 4679.156 | 5.546875 |  |  |  |  |  |
| 13.48333 | | 170.0498 | 0.68166 | 4679.148 | 5.796875 |  |  |  |  |  |
| 13.49167 | | 170.1373 | 0.683559 | 4679.109 | 5.773438 |  |  |  |  |  |
| 13.5 | | 170.2265 | 0.683622 | 4679.078 | 5.789063 |  |  |  |  |  |
| 13.50833 | | 170.3174 | 0.684016 | 4679.008 | 5.734375 |  |  |  |  |  |
| 13.51667 | | 170.4057 | 0.685748 | 4678.992 | 5.984375 |  |  |  |  |  |
| 13.525 | | 170.4958 | 0.685598 | 4678.977 | 6.03125 |  |  |  |  |  |
| 13.53333 | | 170.5832 | 0.686002 | 4678.906 | 5.96875 |  |  |  |  |  |
| 13.54167 | | 170.6716 | 0.687728 | 4678.859 | 5.664063 |  |  |  |  |  |
| 13.55 | | 170.759 | 0.687561 | 4678.813 | 5.875 |  |  |  |  |  |
| 13.55833 | | 170.8456 | 0.687853 | 4678.766 | 5.945313 |  |  |  |  |  |
| 13.56667 | | 170.933 | 0.689802 | 4678.688 | 5.875 |  |  |  |  |  |
| 13.575 | | 171.0239 | 0.69019 | 4678.625 | 6.023438 |  |  |  |  |  |
| 13.58333 | | 171.1079 | 0.69102 | 4678.578 | 6.03125 |  |  |  |  |  |
| 13.59167 | | 171.1962 | 0.693399 | 4678.578 | 6.304688 |  |  |  |  |  |
| 13.6 | | 171.2845 | 0.693772 | 4678.5 | 6.09375 |  |  |  |  |  |
| 13.60833 | | 171.3728 | 0.6949 | 4678.445 | 5.890625 |  |  |  |  |  |
| 13.61667 | | 171.4612 | 0.697367 | 4678.398 | 5.867188 |  |  |  |  |  |
| 13.625 | | 171.5512 | 0.698009 | 4678.328 | 5.890625 |  |  |  |  |  |
| 13.63333 | | 171.6404 | 0.6997 | 4678.273 | 5.78125 |  |  |  |  |  |
| 13.64167 | | 171.7287 | 0.701967 | 4678.188 | 5.734375 |  |  |  |  |  |
| 13.65 | | 171.8214 | 0.702499 | 4678.164 | 5.679688 |  |  |  |  |  |
| 13.65833 | | 171.9071 | 0.704116 | 4678.133 | 5.460938 |  |  |  |  |  |
| 13.66667 | | 171.9954 | 0.705525 | 4678.078 | 5.5 |  |  |  |  |  |
| 13.675 | | 172.0802 | 0.705206 | 4678.023 | 5.328125 |  |  |  |  |  |
| 13.68333 | | 172.1703 | 0.706508 | 4677.992 | 5.335938 |  |  |  |  |  |
| 13.69167 | | 172.2578 | 0.707733 | 4677.953 | 5.4375 |  |  |  |  |  |
| 13.7 | | 172.3461 | 0.707909 | 4677.906 | 5.25 |  |  |  |  |  |
| 13.70833 | | 172.4318 | 0.70924 | 4677.891 | 5.375 |  |  |  |  |  |
| 13.71667 | | 172.5183 | 0.710711 | 4677.836 | 5.21875 |  |  |  |  |  |
| 13.725 | | 172.6075 | 0.7107 | 4677.82 | 5.171875 |  |  |  |  |  |
| 13.73333 | | 172.6932 | 0.711847 | 4677.773 | 5.023438 |  |  |  |  |  |
| 13.74167 | | 172.7815 | 0.713412 | 4677.719 | 5.109375 |  |  |  |  |  |
| 13.75 | | 172.8716 | 0.713296 | 4677.695 | 4.953125 |  |  |  |  |  |
| 13.75833 | | 172.9617 | 0.714401 | 4677.641 | 5.023438 |  |  |  |  |  |
| 13.76667 | | 173.0473 | 0.716255 | 4677.609 | 5.148438 |  |  |  |  |  |
| 13.775 | | 173.1365 | 0.716208 | 4677.578 | 5.34375 |  |  |  |  |  |
| 13.78333 | | 173.2249 | 0.717149 | 4677.539 | 5.46875 |  |  |  |  |  |
| 13.79167 | | 173.3176 | 0.718467 | 4677.492 | 5.507813 |  |  |  |  |  |
| 13.8 | | 173.4076 | 0.718403 | 4677.469 | 5.601563 |  |  |  |  |  |
| 13.80833 | | 173.4977 | 0.719606 | 4677.422 | 5.703125 |  |  |  |  |  |
| 13.81667 | | 173.586 | 0.72058 | 4677.367 | 5.71875 |  |  |  |  |  |
| 13.825 | | 173.6787 | 0.72062 | 4677.289 | 5.710938 |  |  |  |  |  |
| 13.83333 | | 173.7696 | 0.722137 | 4677.234 | 5.679688 |  |  |  |  |  |
| 13.84167 | | 173.8623 | 0.723617 | 4677.172 | 5.804688 |  |  |  |  |  |
| 13.85 | | 173.9532 | 0.724083 | 4677.117 | 5.828125 |  |  |  |  |  |
| 13.85833 | | 174.0407 | 0.725791 | 4677.055 | 5.5 |  |  |  |  |  |
| 13.86667 | | 174.1299 | 0.726864 | 4677.008 | 5.46875 |  |  |  |  |  |
| 13.875 | | 174.2156 | 0.726995 | 4676.961 | 5.4375 |  |  |  |  |  |
| 13.88333 | | 174.3048 | 0.728852 | 4676.922 | 5.367188 |  |  |  |  |  |
| 13.89167 | | 174.3913 | 0.729822 | 4676.852 | 5.4375 |  |  |  |  |  |
| 13.9 | | 174.4788 | 0.729639 | 4676.797 | 5.460938 |  |  |  |  |  |
| 13.90833 | | 174.5653 | 0.730762 | 4676.797 | 5.484375 |  |  |  |  |  |
| 13.91667 | | 174.6519 | 0.731666 | 4676.75 | 5.304688 |  |  |  |  |  |
| 13.925 | | 174.7359 | 0.731075 | 4676.719 | 5.25 |  |  |  |  |  |
| 13.93333 | | 174.8242 | 0.732477 | 4676.672 | 5.328125 |  |  |  |  |  |
| 13.94167 | | 174.9124 | 0.733424 | 4676.625 | 5.328125 |  |  |  |  |  |
| 13.95 | | 175.0031 | 0.732649 | 4676.578 | 5.429688 |  |  |  |  |  |
| 13.95833 | | 175.0903 | 0.734171 | 4676.531 | 5.585938 |  |  |  |  |  |
| 13.96667 | | 175.1784 | 0.735507 | 4676.5 | 5.507813 |  |  |  |  |  |
| 13.975 | | 175.2673 | 0.734471 | 4676.469 | 5.765625 |  |  |  |  |  |
| 13.98333 | | 175.3571 | 0.735804 | 4676.406 | 5.921875 |  |  |  |  |  |
| 13.99167 | | 175.4434 | 0.73709 | 4676.367 | 5.96875 |  |  |  |  |  |
| 14 | | 175.5323 | 0.73671 | 4676.305 | 5.914063 |  |  |  |  |  |
| 14.00833 | | 175.6213 | 0.737932 | 4676.242 | 5.898438 |  |  |  |  |  |
| 14.01667 | | 175.7085 | 0.738824 | 4676.195 | 5.78125 |  |  |  |  |  |
| 14.025 | | 175.7991 | 0.739 | 4676.109 | 6.0625 |  |  |  |  |  |
| 14.03333 | | 175.8855 | 0.740676 | 4676.039 | 6.28125 |  |  |  |  |  |
| 14.04167 | | 175.9752 | 0.741987 | 4675.984 | 6.492188 |  |  |  |  |  |
| 14.05 | | 176.0624 | 0.742202 | 4675.945 | 7.03125 |  |  |  |  |  |
| 14.05833 | | 176.1557 | 0.744742 | 4675.898 | 6.914063 |  |  |  |  |  |
| 14.06667 | | 176.2429 | 0.746642 | 4675.867 | 7.117188 |  |  |  |  |  |
| 14.075 | | 176.3309 | 0.747565 | 4675.789 | 7.375 |  |  |  |  |  |
| 14.08333 | | 176.4199 | 0.749914 | 4675.703 | 7.679688 |  |  |  |  |  |
| 14.09167 | | 176.5097 | 0.751974 | 4675.617 | 7.734375 |  |  |  |  |  |
| 14.1 | | 176.5986 | 0.752538 | 4675.492 | 7.84375 |  |  |  |  |  |
| 14.10833 | | 176.6841 | 0.755249 | 4675.438 | 7.820313 |  |  |  |  |  |
| 14.11667 | | 176.7713 | 0.756525 | 4675.367 | 7.515625 |  |  |  |  |  |
| 14.125 | | 176.8611 | 0.756747 | 4675.266 | 7.203125 |  |  |  |  |  |
| 14.13333 | | 176.95 | 0.758534 | 4675.156 | 6.664063 |  |  |  |  |  |
| 14.14167 | | 177.0337 | 0.75907 | 4675.094 | 6.53125 |  |  |  |  |  |
| 14.15 | | 177.1227 | 0.759326 | 4675.016 | 6.359375 |  |  |  |  |  |
| 14.15833 | | 177.2124 | 0.761303 | 4674.961 | 6.125 |  |  |  |  |  |
| 14.16667 | | 177.2962 | 0.761574 | 4674.938 | 6 |  |  |  |  |  |
| 14.175 | | 177.3817 | 0.761468 | 4674.922 | 5.875 |  |  |  |  |  |
| 14.18333 | | 177.4697 | 0.763453 | 4674.938 | 5.90625 |  |  |  |  |  |
| 14.19167 | | 177.5578 | 0.763931 | 4674.906 | 5.507813 |  |  |  |  |  |
| 14.2 | | 177.6458 | 0.764527 | 4674.875 | 5.601563 |  |  |  |  |  |
| 14.20833 | | 177.7305 | 0.76598 | 4674.859 | 5.429688 |  |  |  |  |  |
| 14.21667 | | 177.8185 | 0.766967 | 4674.828 | 5.578125 |  |  |  |  |  |
| 14.225 | | 177.9066 | 0.767514 | 4674.797 | 5.460938 |  |  |  |  |  |
| 14.23333 | | 177.9946 | 0.769989 | 4674.742 | 5.601563 |  |  |  |  |  |
| 14.24167 | | 178.0827 | 0.770741 | 4674.75 | 5.695313 |  |  |  |  |  |
| 14.25 | | 178.1716 | 0.770778 | 4674.695 | 5.5625 |  |  |  |  |  |
| 14.25833 | | 178.2606 | 0.773141 | 4674.68 | 5.539063 |  |  |  |  |  |
| 14.26667 | | 178.3469 | 0.773823 | 4674.609 | 5.421875 |  |  |  |  |  |
| 14.275 | | 178.4393 | 0.774401 | 4674.578 | 5.632813 |  |  |  |  |  |
| 14.28333 | | 178.5265 | 0.776623 | 4674.516 | 5.617188 |  |  |  |  |  |
| 14.29167 | | 178.6171 | 0.77763 | 4674.453 | 5.742188 |  |  |  |  |  |
| 14.3 | | 178.7052 | 0.778475 | 4674.422 | 5.664063 |  |  |  |  |  |
| 14.30833 | | 178.795 | 0.780583 | 4674.383 | 5.585938 |  |  |  |  |  |
| 14.31667 | | 178.8848 | 0.781038 | 4674.344 | 5.335938 |  |  |  |  |  |
| 14.325 | | 178.972 | 0.781492 | 4674.281 | 5.132813 |  |  |  |  |  |
| 14.33333 | | 179.0635 | 0.783308 | 4674.234 | 5.140625 |  |  |  |  |  |
| 14.34167 | | 179.1507 | 0.783836 | 4674.164 | 5.09375 |  |  |  |  |  |
| 14.35 | | 179.2405 | 0.7844 | 4674.133 | 4.710938 |  |  |  |  |  |
| 14.35833 | | 179.3251 | 0.786515 | 4674.094 | 4.640625 |  |  |  |  |  |
| 14.36667 | | 179.4114 | 0.786533 | 4674.078 | 4.609375 |  |  |  |  |  |
| 14.375 | | 179.4995 | 0.787399 | 4674.063 | 4.351563 |  |  |  |  |  |
| 14.38333 | | 179.591 | 0.789112 | 4674.008 | 4.117188 |  |  |  |  |  |
| 14.39167 | | 179.6773 | 0.789039 | 4673.977 | 3.828125 |  |  |  |  |  |
| 14.4 | | 179.7645 | 0.78955 | 4673.984 | 3.75 |  |  |  |  |  |
| 14.40833 | | 179.8517 | 0.791481 | 4673.953 | 3.757813 |  |  |  |  |  |
| 14.41667 | | 179.9389 | 0.79171 | 4673.938 | 3.960938 |  |  |  |  |  |
| 14.425 | | 180.0279 | 0.792614 | 4673.93 | 4.054688 |  |  |  |  |  |
| 14.43333 | | 180.1168 | 0.794182 | 4673.914 | 4.53125 |  |  |  |  |  |
| 14.44167 | | 180.2057 | 0.794034 | 4673.922 | 4.96875 |  |  |  |  |  |
| 14.45 | | 180.2981 | 0.79469 | 4673.883 | 5.140625 |  |  |  |  |  |
| 14.45833 | | 180.3879 | 0.796054 | 4673.852 | 5.523438 |  |  |  |  |  |
| 14.46667 | | 180.4742 | 0.796284 | 4673.789 | 5.976563 |  |  |  |  |  |
| 14.475 | | 180.5623 | 0.797223 | 4673.727 | 6.21875 |  |  |  |  |  |
| 14.48333 | | 180.6521 | 0.799559 | 4673.609 | 6.335938 |  |  |  |  |  |
| 14.49167 | | 180.741 | 0.799749 | 4673.508 | 6.578125 |  |  |  |  |  |
| 14.5 | | 180.8308 | 0.800665 | 4673.43 | 6.351563 |  |  |  |  |  |
| 14.50833 | | 180.9214 | 0.802433 | 4673.336 | 6.609375 |  |  |  |  |  |
| 14.51667 | | 181.0095 | 0.802124 | 4673.219 | 6.265625 |  |  |  |  |  |
| 14.525 | | 181.0984 | 0.802592 | 4673.141 | 5.953125 |  |  |  |  |  |
| 14.53333 | | 181.1908 | 0.803842 | 4673.07 | 5.84375 |  |  |  |  |  |
| 14.54167 | | 181.2789 | 0.803851 | 4672.992 | 5.53125 |  |  |  |  |  |
| 14.55 | | 181.3687 | 0.804627 | 4672.977 | 5.28125 |  |  |  |  |  |
| 14.55833 | | 181.4585 | 0.805955 | 4672.891 | 5.28125 |  |  |  |  |  |
| 14.56667 | | 181.5439 | 0.806122 | 4672.891 | 5.28125 |  |  |  |  |  |
| 14.575 | | 181.6337 | 0.806599 | 4672.883 | 5.109375 |  |  |  |  |  |
| 14.58333 | | 181.7218 | 0.808689 | 4672.859 | 5.25 |  |  |  |  |  |
| 14.59167 | | 181.8116 | 0.809117 | 4672.852 | 5.15625 |  |  |  |  |  |
| 14.6 | | 181.9014 | 0.810178 | 4672.844 | 5.09375 |  |  |  |  |  |
| 14.60833 | | 181.992 | 0.812249 | 4672.797 | 5.046875 |  |  |  |  |  |
| 14.61667 | | 182.0784 | 0.812861 | 4672.766 | 5.0625 |  |  |  |  |  |
| 14.625 | | 182.169 | 0.813781 | 4672.734 | 5.210938 |  |  |  |  |  |
| 14.63333 | | 182.2554 | 0.816163 | 4672.672 | 5.289063 |  |  |  |  |  |
| 14.64167 | | 182.346 | 0.816314 | 4672.648 | 5.398438 |  |  |  |  |  |
| 14.65 | | 182.4367 | 0.81731 | 4672.609 | 5.445313 |  |  |  |  |  |
| 14.65833 | | 182.5264 | 0.818852 | 4672.57 | 5.453125 |  |  |  |  |  |
| 14.66667 | | 182.6154 | 0.81863 | 4672.523 | 5.398438 |  |  |  |  |  |
| 14.675 | | 182.7017 | 0.818928 | 4672.461 | 5.101563 |  |  |  |  |  |
| 14.68333 | | 182.7941 | 0.820664 | 4672.414 | 5.257813 |  |  |  |  |  |
| 14.69167 | | 182.8856 | 0.820909 | 4672.336 | 5.476563 |  |  |  |  |  |
| 14.7 | | 182.9728 | 0.8219 | 4672.297 | 5.601563 |  |  |  |  |  |
| 14.70833 | | 183.0591 | 0.823281 | 4672.242 | 5.570313 |  |  |  |  |  |
| 14.71667 | | 183.1498 | 0.823679 | 4672.219 | 5.546875 |  |  |  |  |  |
| 14.725 | | 183.2379 | 0.824802 | 4672.211 | 5.328125 |  |  |  |  |  |
| 14.73333 | | 183.3251 | 0.826193 | 4672.148 | 5.421875 |  |  |  |  |  |
| 14.74167 | | 183.4097 | 0.825849 | 4672.07 | 5.375 |  |  |  |  |  |
| 14.75 | | 183.4969 | 0.827066 | 4672.016 | 5.34375 |  |  |  |  |  |
| 14.75833 | | 183.5875 | 0.828402 | 4671.977 | 5.46875 |  |  |  |  |  |
| 14.76667 | | 183.6739 | 0.828215 | 4671.938 | 5.359375 |  |  |  |  |  |
| 14.775 | | 183.7585 | 0.829044 | 4671.922 | 5.390625 |  |  |  |  |  |
| 14.78333 | | 183.8491 | 0.830047 | 4671.875 | 5.5625 |  |  |  |  |  |
| 14.79167 | | 183.9389 | 0.829878 | 4671.844 | 5.546875 |  |  |  |  |  |
| 14.8 | | 184.0278 | 0.830861 | 4671.805 | 5.625 |  |  |  |  |  |
| 14.80833 | | 184.1202 | 0.832227 | 4671.742 | 5.601563 |  |  |  |  |  |
| 14.81667 | | 184.2126 | 0.832002 | 4671.711 | 5.664063 |  |  |  |  |  |
| 14.825 | | 184.3031 | 0.83378 | 4671.648 | 5.789063 |  |  |  |  |  |
| 14.83333 | | 184.3944 | 0.834665 | 4671.578 | 5.984375 |  |  |  |  |  |
| 14.84167 | | 184.4814 | 0.834564 | 4671.531 | 6.125 |  |  |  |  |  |
| 14.85 | | 184.5735 | 0.836371 | 4671.469 | 6.171875 |  |  |  |  |  |
| 14.85833 | | 184.6614 | 0.837747 | 4671.414 | 5.820313 |  |  |  |  |  |
| 14.86667 | | 184.7492 | 0.838385 | 4671.352 | 5.789063 |  |  |  |  |  |
| 14.875 | | 184.8371 | 0.840029 | 4671.281 | 6.03125 |  |  |  |  |  |
| 14.88333 | | 184.9284 | 0.840965 | 4671.195 | 5.921875 |  |  |  |  |  |
| 14.89167 | | 185.0154 | 0.841058 | 4671.125 | 5.867188 |  |  |  |  |  |
| 14.9 | | 185.105 | 0.842588 | 4671.07 | 6.015625 |  |  |  |  |  |
| 14.90833 | | 185.1971 | 0.843465 | 4671.063 | 6.109375 |  |  |  |  |  |
| 14.91667 | | 185.2858 | 0.843455 | 4671.031 | 5.929688 |  |  |  |  |  |
| 14.925 | | 185.3771 | 0.845447 | 4670.953 | 5.765625 |  |  |  |  |  |
| 14.93333 | | 185.4615 | 0.846501 | 4670.914 | 5.820313 |  |  |  |  |  |
| 14.94167 | | 185.5528 | 0.846621 | 4670.883 | 5.875 |  |  |  |  |  |
| 14.95 | | 185.6432 | 0.847926 | 4670.82 | 5.953125 |  |  |  |  |  |
| 14.95833 | | 185.7328 | 0.848312 | 4670.75 | 5.84375 |  |  |  |  |  |
| 14.96667 | | 185.8207 | 0.84788 | 4670.734 | 5.851563 |  |  |  |  |  |
| 14.975 | | 185.9086 | 0.849328 | 4670.703 | 6.15625 |  |  |  |  |  |
| 14.98333 | | 185.9956 | 0.85007 | 4670.656 | 6.328125 |  |  |  |  |  |
| 14.99167 | | 186.0843 | 0.850066 | 4670.594 | 6.257813 |  |  |  |  |  |
| 15 | | 186.1704 | 0.851211 | 4670.539 | 6.3125 |  |  |  |  |  |
| 15.00833 | | 186.2583 | 0.85199 | 4670.5 | 6.648438 |  |  |  |  |  |
| 15.01667 | | 186.3487 | 0.851984 | 4670.453 | 6.867188 |  |  |  |  |  |
| 15.025 | | 186.4357 | 0.853415 | 4670.359 | 7.039063 |  |  |  |  |  |
| 15.03333 | | 186.5228 | 0.854488 | 4670.281 | 7.148438 |  |  |  |  |  |
| 15.04167 | | 186.6123 | 0.854131 | 4670.234 | 7.179688 |  |  |  |  |  |
| 15.05 | | 186.6985 | 0.85539 | 4670.164 | 7.273438 |  |  |  |  |  |
| 15.05833 | | 186.7864 | 0.856032 | 4670.07 | 7.28125 |  |  |  |  |  |
| 15.06667 | | 186.8725 | 0.855661 | 4669.984 | 7.195313 |  |  |  |  |  |
| 15.075 | | 186.9595 | 0.856406 | 4669.914 | 7.242188 |  |  |  |  |  |
| 15.08333 | | 187.05 | 0.857034 | 4669.828 | 7.234375 |  |  |  |  |  |
| 15.09167 | | 187.1395 | 0.856674 | 4669.773 | 7.085938 |  |  |  |  |  |
| 15.1 | | 187.2265 | 0.857462 | 4669.688 | 6.945313 |  |  |  |  |  |
| 15.10833 | | 187.3153 | 0.858382 | 4669.633 | 6.789063 |  |  |  |  |  |
| 15.11667 | | 187.4014 | 0.857596 | 4669.586 | 6.335938 |  |  |  |  |  |
| 15.125 | | 187.4901 | 0.858392 | 4669.523 | 5.984375 |  |  |  |  |  |
| 15.13333 | | 187.5789 | 0.859334 | 4669.469 | 5.828125 |  |  |  |  |  |
| 15.14167 | | 187.6693 | 0.858711 | 4669.438 | 5.429688 |  |  |  |  |  |
| 15.15 | | 187.7606 | 0.860013 | 4669.398 | 5.132813 |  |  |  |  |  |
| 15.15833 | | 187.8459 | 0.861359 | 4669.367 | 4.851563 |  |  |  |  |  |
| 15.16667 | | 187.9346 | 0.860691 | 4669.383 | 4.6875 |  |  |  |  |  |
| 15.175 | | 188.0242 | 0.862447 | 4669.375 | 4.671875 |  |  |  |  |  |
| 15.18333 | | 188.112 | 0.863264 | 4669.344 | 4.398438 |  |  |  |  |  |
| 15.19167 | | 188.1999 | 0.862809 | 4669.359 | 4.453125 |  |  |  |  |  |
| 15.2 | | 188.2903 | 0.864121 | 4669.359 | 4.5625 |  |  |  |  |  |
| 15.20833 | | 188.379 | 0.86511 | 4669.352 | 4.609375 |  |  |  |  |  |
| 15.21667 | | 188.4695 | 0.864858 | 4669.336 | 4.445313 |  |  |  |  |  |
| 15.225 | | 188.5556 | 0.866148 | 4669.305 | 4.640625 |  |  |  |  |  |
| 15.23333 | | 188.6435 | 0.867391 | 4669.289 | 4.84375 |  |  |  |  |  |
| 15.24167 | | 188.7356 | 0.867227 | 4669.25 | 4.765625 |  |  |  |  |  |
| 15.25 | | 188.8252 | 0.868705 | 4669.188 | 4.960938 |  |  |  |  |  |
| 15.25833 | | 188.9113 | 0.869223 | 4669.148 | 4.960938 |  |  |  |  |  |
| 15.26667 | | 189.0026 | 0.869128 | 4669.141 | 5.203125 |  |  |  |  |  |
| 15.275 | | 189.093 | 0.870805 | 4669.063 | 5.382813 |  |  |  |  |  |
| 15.28333 | | 189.1826 | 0.87152 | 4668.992 | 5.71875 |  |  |  |  |  |
| 15.29167 | | 189.2713 | 0.871595 | 4668.969 | 6.015625 |  |  |  |  |  |
| 15.3 | | 189.3592 | 0.873625 | 4668.898 | 6.382813 |  |  |  |  |  |
| 15.30833 | | 189.4505 | 0.874369 | 4668.852 | 6.578125 |  |  |  |  |  |
| 15.31667 | | 189.5426 | 0.874416 | 4668.766 | 6.5625 |  |  |  |  |  |
| 15.325 | | 189.6339 | 0.875967 | 4668.703 | 6.96875 |  |  |  |  |  |
| 15.33333 | | 189.7218 | 0.875721 | 4668.609 | 7.054688 |  |  |  |  |  |
| 15.34167 | | 189.8122 | 0.875946 | 4668.516 | 7.3125 |  |  |  |  |  |
| 15.35 | | 189.9009 | 0.877742 | 4668.406 | 7.273438 |  |  |  |  |  |
| 15.35833 | | 189.9922 | 0.877246 | 4668.328 | 7.4375 |  |  |  |  |  |
| 15.36667 | | 190.0835 | 0.878039 | 4668.273 | 7.460938 |  |  |  |  |  |
| 15.375 | | 190.1739 | 0.879493 | 4668.164 | 7.460938 |  |  |  |  |  |
| 15.38333 | | 190.266 | 0.878764 | 4668.078 | 7.375 |  |  |  |  |  |
| 15.39167 | | 190.3556 | 0.879857 | 4667.984 | 7.492188 |  |  |  |  |  |
| 15.4 | | 190.4435 | 0.880931 | 4667.93 | 7.460938 |  |  |  |  |  |
| 15.40833 | | 190.5313 | 0.880634 | 4667.844 | 7.28125 |  |  |  |  |  |
| 15.41667 | | 190.6217 | 0.881347 | 4667.773 | 7.054688 |  |  |  |  |  |
| 15.425 | | 190.7113 | 0.882909 | 4667.711 | 6.742188 |  |  |  |  |  |
| 15.43333 | | 190.8026 | 0.882721 | 4667.656 | 6.601563 |  |  |  |  |  |
| 15.44167 | | 190.8913 | 0.883569 | 4667.57 | 6.320313 |  |  |  |  |  |
| 15.45 | | 190.9783 | 0.884985 | 4667.508 | 6.085938 |  |  |  |  |  |
| 15.45833 | | 191.0653 | 0.884538 | 4667.484 | 5.757813 |  |  |  |  |  |
| 15.46667 | | 191.1541 | 0.88492 | 4667.461 | 5.390625 |  |  |  |  |  |
| 15.475 | | 191.2419 | 0.886246 | 4667.453 | 5.203125 |  |  |  |  |  |
| 15.48333 | | 191.3289 | 0.886209 | 4667.414 | 5.070313 |  |  |  |  |  |
| 15.49167 | | 191.4185 | 0.887079 | 4667.398 | 5.015625 |  |  |  |  |  |
| 15.5 | | 191.5089 | 0.888675 | 4667.383 | 5.0625 |  |  |  |  |  |
| 15.50833 | | 191.5968 | 0.888369 | 4667.383 | 5.132813 |  |  |  |  |  |
| 15.51667 | | 191.6864 | 0.889047 | 4667.398 | 5.125 |  |  |  |  |  |
| 15.525 | | 191.7725 | 0.890311 | 4667.383 | 4.820313 |  |  |  |  |  |
| 15.53333 | | 191.863 | 0.890102 | 4667.359 | 4.601563 |  |  |  |  |  |
| 15.54167 | | 191.9551 | 0.890781 | 4667.336 | 4.726563 |  |  |  |  |  |
| 15.55 | | 192.0421 | 0.892318 | 4667.281 | 4.78125 |  |  |  |  |  |
| 15.55833 | | 192.1317 | 0.892026 | 4667.242 | 4.59375 |  |  |  |  |  |
| 15.56667 | | 192.223 | 0.892175 | 4667.211 | 4.664063 |  |  |  |  |  |
| 15.575 | | 192.3117 | 0.893299 | 4667.211 | 4.960938 |  |  |  |  |  |
| 15.58333 | | 192.3978 | 0.892615 | 4667.203 | 4.734375 |  |  |  |  |  |
| 15.59167 | | 192.4874 | 0.892497 | 4667.156 | 4.804688 |  |  |  |  |  |
| 15.6 | | 192.5736 | 0.893788 | 4667.102 | 4.945313 |  |  |  |  |  |
| 15.60833 | | 192.6631 | 0.893294 | 4667.086 | 5.46875 |  |  |  |  |  |
| 15.61667 | | 192.7527 | 0.893376 | 4667.031 | 5.625 |  |  |  |  |  |
| 15.625 | | 192.8389 | 0.89476 | 4666.953 | 5.78125 |  |  |  |  |  |
| 15.63333 | | 192.9284 | 0.894394 | 4666.938 | 6.179688 |  |  |  |  |  |
| 15.64167 | | 193.0172 | 0.894186 | 4666.891 | 6.390625 |  |  |  |  |  |
| 15.65 | | 193.1084 | 0.895783 | 4666.828 | 6.53125 |  |  |  |  |  |
| 15.65833 | | 193.1997 | 0.895397 | 4666.703 | 6.710938 |  |  |  |  |  |
| 15.66667 | | 193.2893 | 0.89544 | 4666.641 | 7.023438 |  |  |  |  |  |
| 15.675 | | 193.378 | 0.897876 | 4666.563 | 7.195313 |  |  |  |  |  |
| 15.68333 | | 193.4684 | 0.898126 | 4666.453 | 7.351563 |  |  |  |  |  |
| 15.69167 | | 193.5606 | 0.898738 | 4666.359 | 7.21875 |  |  |  |  |  |
| 15.7 | | 193.6518 | 0.900598 | 4666.273 | 7.578125 |  |  |  |  |  |
| 15.70833 | | 193.7423 | 0.900386 | 4666.195 | 7.75 |  |  |  |  |  |
| 15.71667 | | 193.8361 | 0.900984 | 4666.094 | 7.664063 |  |  |  |  |  |
| 15.725 | | 193.9274 | 0.902459 | 4666 | 7.796875 |  |  |  |  |  |
| 15.73333 | | 194.017 | 0.902238 | 4665.914 | 7.6875 |  |  |  |  |  |
| 15.74167 | | 194.1065 | 0.903159 | 4665.867 | 7.546875 |  |  |  |  |  |
| 15.75 | | 194.1995 | 0.904484 | 4665.75 | 7.328125 |  |  |  |  |  |
| 15.75833 | | 194.2899 | 0.903953 | 4665.672 | 7.273438 |  |  |  |  |  |
| 15.76667 | | 194.3812 | 0.905138 | 4665.602 | 7.09375 |  |  |  |  |  |
| 15.775 | | 194.4708 | 0.906391 | 4665.523 | 7.382813 |  |  |  |  |  |
| 15.78333 | | 194.5578 | 0.906001 | 4665.477 | 7 |  |  |  |  |  |
| 15.79167 | | 194.6482 | 0.907123 | 4665.438 | 6.96875 |  |  |  |  |  |
| 15.8 | | 194.7335 | 0.908353 | 4665.406 | 7 |  |  |  |  |  |
| 15.80833 | | 194.8223 | 0.908175 | 4665.359 | 6.617188 |  |  |  |  |  |
| 15.81667 | | 194.9093 | 0.909465 | 4665.32 | 6.859375 |  |  |  |  |  |
| 15.825 | | 194.998 | 0.910979 | 4665.227 | 6.945313 |  |  |  |  |  |
| 15.83333 | | 195.0858 | 0.910993 | 4665.227 | 6.984375 |  |  |  |  |  |
| 15.84167 | | 195.1746 | 0.912066 | 4665.18 | 7.117188 |  |  |  |  |  |
| 15.85 | | 195.2633 | 0.913259 | 4665.117 | 7.09375 |  |  |  |  |  |
| 15.85833 | | 195.352 | 0.912721 | 4665.117 | 6.820313 |  |  |  |  |  |
| 15.86667 | | 195.445 | 0.913568 | 4665.047 | 7.054688 |  |  |  |  |  |
| 15.875 | | 195.5371 | 0.915268 | 4664.984 | 7.085938 |  |  |  |  |  |
| 15.88333 | | 195.6284 | 0.915165 | 4664.914 | 7.15625 |  |  |  |  |  |
| 15.89167 | | 195.7205 | 0.916461 | 4664.844 | 7.492188 |  |  |  |  |  |
| 15.9 | | 195.8161 | 0.917935 | 4664.789 | 8.046875 |  |  |  |  |  |
| 15.90833 | | 195.9056 | 0.917736 | 4664.781 | 8.375 |  |  |  |  |  |
| 15.91667 | | 195.9978 | 0.91892 | 4664.68 | 8.851563 |  |  |  |  |  |
| 15.925 | | 196.0908 | 0.920328 | 4664.625 | 9.257813 |  |  |  |  |  |
| 15.93333 | | 196.1795 | 0.92021 | 4664.563 | 9.703125 |  |  |  |  |  |
| 15.94167 | | 196.2708 | 0.921954 | 4664.453 | 10.28125 |  |  |  |  |  |
| 15.95 | | 196.3595 | 0.922907 | 4664.313 | 10.57813 |  |  |  |  |  |
| 15.95833 | | 196.4482 | 0.922351 | 4664.203 | 10.99219 |  |  |  |  |  |
| 15.96667 | | 196.5403 | 0.923407 | 4664.063 | 11.15625 |  |  |  |  |  |
| 15.975 | | 196.6316 | 0.92399 | 4663.93 | 11.36719 |  |  |  |  |  |
| 15.98333 | | 196.7186 | 0.923753 | 4663.781 | 11.21875 |  |  |  |  |  |
| 15.99167 | | 196.8099 | 0.925415 | 4663.617 | 11.35156 |  |  |  |  |  |
| 16 | | 196.8969 | 0.926083 | 4663.492 | 11.42188 |  |  |  |  |  |
| 16.00833 | | 196.9873 | 0.925756 | 4663.344 | 11.52344 |  |  |  |  |  |
| 16.01667 | | 197.0803 | 0.927026 | 4663.227 | 11.28906 |  |  |  |  |  |
| 16.025 | | 197.1716 | 0.927732 | 4663.109 | 11.1875 |  |  |  |  |  |
| 16.03333 | | 197.2629 | 0.927613 | 4663.039 | 11.51563 |  |  |  |  |  |
| 16.04167 | | 197.3524 | 0.929569 | 4662.914 | 11.55469 |  |  |  |  |  |
| 16.05 | | 197.4429 | 0.930715 | 4662.813 | 11.51563 |  |  |  |  |  |
| 16.05833 | | 197.5333 | 0.930676 | 4662.711 | 11.65625 |  |  |  |  |  |
| 16.06667 | | 197.6237 | 0.932244 | 4662.648 | 11.64063 |  |  |  |  |  |
| 16.075 | | 197.7141 | 0.933102 | 4662.563 | 11.39063 |  |  |  |  |  |
| 16.08333 | | 197.8063 | 0.93315 | 4662.43 | 11.28125 |  |  |  |  |  |
| 16.09167 | | 197.895 | 0.934708 | 4662.328 | 11.24219 |  |  |  |  |  |
| 16.1 | | 197.9854 | 0.935627 | 4662.234 | 11.45313 |  |  |  |  |  |
| 16.10833 | | 198.0767 | 0.935665 | 4662.117 | 11.35156 |  |  |  |  |  |
| 16.11667 | | 198.1646 | 0.937309 | 4662.031 | 10.95313 |  |  |  |  |  |
| 16.125 | | 198.2576 | 0.938168 | 4661.969 | 10.82813 |  |  |  |  |  |
| 16.13333 | | 198.348 | 0.938051 | 4661.898 | 10.75 |  |  |  |  |  |
| 16.14167 | | 198.4384 | 0.939621 | 4661.813 | 10.39844 |  |  |  |  |  |
| 16.15 | | 198.5297 | 0.940377 | 4661.703 | 10.21094 |  |  |  |  |  |
| 16.15833 | | 198.6201 | 0.940064 | 4661.625 | 10.17188 |  |  |  |  |  |
| 16.16667 | | 198.7122 | 0.941251 | 4661.594 | 9.90625 |  |  |  |  |  |
| 16.175 | | 198.8061 | 0.941945 | 4661.523 | 9.625 |  |  |  |  |  |
| 16.18333 | | 198.8965 | 0.941467 | 4661.453 | 9.3125 |  |  |  |  |  |
| 16.19167 | | 198.9827 | 0.942555 | 4661.422 | 9.085938 |  |  |  |  |  |
| 16.2 | | 199.0739 | 0.94302 | 4661.359 | 9.046875 |  |  |  |  |  |
| 16.20833 | | 199.1601 | 0.942124 | 4661.297 | 9.054688 |  |  |  |  |  |
| 16.21667 | | 199.2497 | 0.943216 | 4661.258 | 8.804688 |  |  |  |  |  |
| 16.225 | | 199.3375 | 0.943868 | 4661.219 | 8.773438 |  |  |  |  |  |
| 16.23333 | | 199.4271 | 0.943338 | 4661.18 | 8.65625 |  |  |  |  |  |
| 16.24167 | | 199.5175 | 0.944803 | 4661.141 | 8.523438 |  |  |  |  |  |
| 16.25 | | 199.6097 | 0.945448 | 4661.078 | 8.609375 |  |  |  |  |  |
| 16.25833 | | 199.695 | 0.945215 | 4661.008 | 8.4375 |  |  |  |  |  |
| 16.26667 | | 199.7837 | 0.946802 | 4660.961 | 8.359375 |  |  |  |  |  |
| 16.275 | | 199.8775 | 0.947777 | 4660.898 | 8.515625 |  |  |  |  |  |
| 16.28333 | | 199.9671 | 0.947479 | 4660.852 | 8.523438 |  |  |  |  |  |
| 16.29167 | | 200.0575 | 0.949306 | 4660.781 | 8.390625 |  |  |  |  |  |
| 16.3 | | 200.1445 | 0.95029 | 4660.688 | 8.429688 |  |  |  |  |  |
| 16.30833 | | 200.2341 | 0.950233 | 4660.641 | 8.375 |  |  |  |  |  |
| 16.31667 | | 200.3203 | 0.951713 | 4660.563 | 8.359375 |  |  |  |  |  |
| 16.325 | | 200.4107 | 0.952918 | 4660.469 | 8.351563 |  |  |  |  |  |
| 16.33333 | | 200.4968 | 0.952624 | 4660.391 | 8.03125 |  |  |  |  |  |
| 16.34167 | | 200.5907 | 0.954114 | 4660.336 | 8.023438 |  |  |  |  |  |
| 16.35 | | 200.682 | 0.954917 | 4660.242 | 7.953125 |  |  |  |  |  |
| 16.35833 | | 200.7715 | 0.954534 | 4660.172 | 7.75 |  |  |  |  |  |
| 16.36667 | | 200.8628 | 0.956382 | 4660.102 | 7.546875 |  |  |  |  |  |
| 16.375 | | 200.9549 | 0.956967 | 4660.031 | 7.539063 |  |  |  |  |  |
| 16.38333 | | 201.0496 | 0.957037 | 4660.008 | 7.546875 |  |  |  |  |  |
| 16.39167 | | 201.1426 | 0.958798 | 4659.93 | 7.5 |  |  |  |  |  |
| 16.4 | | 201.2382 | 0.958932 | 4659.867 | 7.703125 |  |  |  |  |  |
| 16.40833 | | 201.3294 | 0.958961 | 4659.828 | 7.757813 |  |  |  |  |  |
| 16.41667 | | 201.4224 | 0.960679 | 4659.797 | 7.796875 |  |  |  |  |  |
| 16.425 | | 201.5128 | 0.960922 | 4659.734 | 7.640625 |  |  |  |  |  |
| 16.43333 | | 201.6024 | 0.961708 | 4659.664 | 7.65625 |  |  |  |  |  |
| 16.44167 | | 201.692 | 0.96364 | 4659.594 | 7.492188 |  |  |  |  |  |
| 16.45 | | 201.7833 | 0.963858 | 4659.5 | 7.3125 |  |  |  |  |  |
| 16.45833 | | 201.8771 | 0.964779 | 4659.422 | 7.351563 |  |  |  |  |  |
| 16.46667 | | 201.9684 | 0.966642 | 4659.352 | 7.140625 |  |  |  |  |  |
| 16.475 | | 202.058 | 0.966903 | 4659.305 | 7.179688 |  |  |  |  |  |
| 16.48333 | | 202.1467 | 0.967505 | 4659.242 | 7.03125 |  |  |  |  |  |
| 16.49167 | | 202.238 | 0.969036 | 4659.203 | 7.015625 |  |  |  |  |  |
| 16.5 | | 202.3292 | 0.96888 | 4659.156 | 6.898438 |  |  |  |  |  |
| 16.50833 | | 202.4214 | 0.969184 | 4659.094 | 7.085938 |  |  |  |  |  |
| 16.51667 | | 202.5152 | 0.970753 | 4659.063 | 7.273438 |  |  |  |  |  |
| 16.525 | | 202.6056 | 0.970509 | 4659 | 7.382813 |  |  |  |  |  |
| 16.53333 | | 202.6986 | 0.971133 | 4658.953 | 7.6875 |  |  |  |  |  |
| 16.54167 | | 202.7873 | 0.972681 | 4658.906 | 7.710938 |  |  |  |  |  |
| 16.55 | | 202.8769 | 0.972466 | 4658.859 | 7.796875 |  |  |  |  |  |
| 16.55833 | | 202.9699 | 0.973005 | 4658.773 | 7.617188 |  |  |  |  |  |
| 16.56667 | | 203.0577 | 0.974545 | 4658.688 | 7.585938 |  |  |  |  |  |
| 16.575 | | 203.1443 | 0.974793 | 4658.609 | 7.53125 |  |  |  |  |  |
| 16.58333 | | 203.2309 | 0.975971 | 4658.516 | 7.40625 |  |  |  |  |  |
| 16.59167 | | 203.3158 | 0.977959 | 4658.453 | 7.296875 |  |  |  |  |  |
| 16.6 | | 203.4024 | 0.978109 | 4658.367 | 7.039063 |  |  |  |  |  |
| 16.60833 | | 203.4939 | 0.97934 | 4658.328 | 6.992188 |  |  |  |  |  |
| 16.61667 | | 203.5813 | 0.981018 | 4658.273 | 6.867188 |  |  |  |  |  |
| 16.625 | | 203.6704 | 0.98091 | 4658.219 | 6.90625 |  |  |  |  |  |
| 16.63333 | | 203.7587 | 0.981745 | 4658.18 | 6.710938 |  |  |  |  |  |
| 16.64167 | | 203.8461 | 0.983399 | 4658.125 | 7.148438 |  |  |  |  |  |
| 16.65 | | 203.936 | 0.983496 | 4658.102 | 7.28125 |  |  |  |  |  |
| 16.65833 | | 204.0242 | 0.984065 | 4658.047 | 7.296875 |  |  |  |  |  |
| 16.66667 | | 204.1141 | 0.985853 | 4658.008 | 7.367188 |  |  |  |  |  |
| 16.675 | | 204.2082 | 0.986017 | 4657.938 | 7.242188 |  |  |  |  |  |
| 16.68333 | | 204.2964 | 0.986784 | 4657.906 | 7.335938 |  |  |  |  |  |
| 16.69167 | | 204.3847 | 0.988415 | 4657.789 | 7.382813 |  |  |  |  |  |
| 16.7 | | 204.4738 | 0.988228 | 4657.711 | 7.359375 |  |  |  |  |  |
| 16.70833 | | 204.5603 | 0.988799 | 4657.641 | 7.28125 |  |  |  |  |  |
| 16.71667 | | 204.6519 | 0.990325 | 4657.57 | 7.203125 |  |  |  |  |  |
| 16.725 | | 204.7435 | 0.990079 | 4657.523 | 6.726563 |  |  |  |  |  |
| 16.73333 | | 204.835 | 0.991494 | 4657.453 | 6.820313 |  |  |  |  |  |
| 16.74167 | | 204.9283 | 0.993423 | 4657.383 | 6.90625 |  |  |  |  |  |
| 16.75 | | 205.0199 | 0.993293 | 4657.328 | 6.992188 |  |  |  |  |  |
| 16.75833 | | 205.1064 | 0.995159 | 4657.281 | 7.164063 |  |  |  |  |  |
| 16.76667 | | 205.1963 | 0.996243 | 4657.234 | 7.28125 |  |  |  |  |  |
| 16.775 | | 205.2838 | 0.995731 | 4657.242 | 7.257813 |  |  |  |  |  |
| 16.78333 | | 205.372 | 0.996996 | 4657.172 | 7.71875 |  |  |  |  |  |
| 16.79167 | | 205.4644 | 0.998255 | 4657.102 | 7.9375 |  |  |  |  |  |
| 16.8 | | 205.551 | 0.997823 | 4657.031 | 8.265625 |  |  |  |  |  |
| 16.80833 | | 205.6384 | 0.999681 | 4656.945 | 8.648438 |  |  |  |  |  |
| 16.81667 | | 205.7241 | 1.000741 | 4656.859 | 8.796875 |  |  |  |  |  |
| 16.825 | | 205.8099 | 1.000351 | 4656.805 | 8.710938 |  |  |  |  |  |
| 16.83333 | | 205.8964 | 1.0016 | 4656.68 | 8.609375 |  |  |  |  |  |
| 16.84167 | | 205.9847 | 1.002421 | 4656.578 | 8.398438 |  |  |  |  |  |
| 16.85 | | 206.0746 | 1.001922 | 4656.461 | 7.914063 |  |  |  |  |  |
| 16.85833 | | 206.167 | 1.003543 | 4656.344 | 7.789063 |  |  |  |  |  |
| 16.86667 | | 206.2577 | 1.005005 | 4656.258 | 7.375 |  |  |  |  |  |
| 16.875 | | 206.3452 | 1.004813 | 4656.203 | 6.9375 |  |  |  |  |  |
| 16.88333 | | 206.4359 | 1.006347 | 4656.141 | 6.492188 |  |  |  |  |  |
| 16.89167 | | 206.5258 | 1.00775 | 4656.109 | 6.21875 |  |  |  |  |  |
| 16.9 | | 206.6199 | 1.007593 | 4656.102 | 5.601563 |  |  |  |  |  |
| 16.90833 | | 206.7114 | 1.009127 | 4656.055 | 5.515625 |  |  |  |  |  |
| 16.91667 | | 206.7997 | 1.010272 | 4656.055 | 5.414063 |  |  |  |  |  |
| 16.925 | | 206.8896 | 1.010173 | 4656.055 | 5.453125 |  |  |  |  |  |
| 16.93333 | | 206.9778 | 1.012058 | 4656.055 | 5.515625 |  |  |  |  |  |
| 16.94167 | | 207.0661 | 1.013159 | 4656.039 | 5.5 |  |  |  |  |  |
| 16.95 | | 207.1535 | 1.012736 | 4656.078 | 5.476563 |  |  |  |  |  |
| 16.95833 | | 207.2434 | 1.014366 | 4656.039 | 5.203125 |  |  |  |  |  |
| 16.96667 | | 207.33 | 1.015326 | 4656.016 | 4.976563 |  |  |  |  |  |
| 16.975 | | 207.4182 | 1.014955 | 4655.969 | 4.867188 |  |  |  |  |  |
| 16.98333 | | 207.5056 | 1.015996 | 4655.922 | 5.085938 |  |  |  |  |  |
| 16.99167 | | 207.5939 | 1.016787 | 4655.859 | 4.773438 |  |  |  |  |  |
| 17 | | 207.6829 | 1.016108 | 4655.828 | 4.796875 |  |  |  |  |  |
| 17.00833 | | 207.7728 | 1.017089 | 4655.82 | 4.710938 |  |  |  |  |  |
| 17.01667 | | 207.8644 | 1.018037 | 4655.813 | 4.632813 |  |  |  |  |  |
| 17.025 | | 207.9535 | 1.01779 | 4655.781 | 4.859375 |  |  |  |  |  |
| 17.03333 | | 208.0442 | 1.01982 | 4655.711 | 4.976563 |  |  |  |  |  |
| 17.04167 | | 208.135 | 1.021238 | 4655.703 | 5.34375 |  |  |  |  |  |
| 17.05 | | 208.2265 | 1.021368 | 4655.664 | 5.71875 |  |  |  |  |  |
| 17.05833 | | 208.3165 | 1.023692 | 4655.625 | 5.96875 |  |  |  |  |  |
| 17.06667 | | 208.4055 | 1.024842 | 4655.594 | 6.109375 |  |  |  |  |  |
| 17.075 | | 208.4971 | 1.025146 | 4655.523 | 6.554688 |  |  |  |  |  |
| 17.08333 | | 208.5903 | 1.0278 | 4655.453 | 6.8125 |  |  |  |  |  |
| 17.09167 | | 208.6786 | 1.028326 | 4655.359 | 6.875 |  |  |  |  |  |
| 17.1 | | 208.7676 | 1.028615 | 4655.25 | 6.898438 |  |  |  |  |  |
| 17.10833 | | 208.8559 | 1.030929 | 4655.172 | 6.921875 |  |  |  |  |  |
| 17.11667 | | 208.9441 | 1.031698 | 4655.102 | 7.023438 |  |  |  |  |  |
| 17.125 | | 209.034 | 1.031566 | 4654.984 | 6.953125 |  |  |  |  |  |
| 17.13333 | | 209.1248 | 1.033815 | 4654.898 | 7.03125 |  |  |  |  |  |
| 17.14167 | | 209.2139 | 1.034196 | 4654.82 | 7.132813 |  |  |  |  |  |
| 17.15 | | 209.3038 | 1.034156 | 4654.758 | 7.335938 |  |  |  |  |  |
| 17.15833 | | 209.3937 | 1.036214 | 4654.695 | 7.429688 |  |  |  |  |  |
| 17.16667 | | 209.4811 | 1.036162 | 4654.625 | 7.585938 |  |  |  |  |  |
| 17.175 | | 209.5718 | 1.036017 | 4654.57 | 7.890625 |  |  |  |  |  |
| 17.18333 | | 209.6617 | 1.038043 | 4654.492 | 8.335938 |  |  |  |  |  |
| 17.19167 | | 209.7525 | 1.038075 | 4654.414 | 8.46875 |  |  |  |  |  |
| 17.2 | | 209.8457 | 1.037974 | 4654.32 | 8.53125 |  |  |  |  |  |
| 17.20833 | | 209.9523 | 1.040724 | 4654.211 | 8.890625 |  |  |  |  |  |
| 17.21667 | | 210.0247 | 1.040267 | 4654.148 | 8.710938 |  |  |  |  |  |
| 17.225 | | 210.1163 | 1.040133 | 4654.039 | 8.726563 |  |  |  |  |  |
| 17.23333 | | 210.2078 | 1.042302 | 4653.914 | 8.617188 |  |  |  |  |  |
| 17.24167 | | 210.2961 | 1.04207 | 4653.828 | 8.375 |  |  |  |  |  |
| 17.25 | | 210.3851 | 1.04246 | 4653.75 | 8.34375 |  |  |  |  |  |
| 17.25833 | | 210.4759 | 1.044986 | 4653.641 | 8.15625 |  |  |  |  |  |
| 17.26667 | | 210.5658 | 1.04523 | 4653.594 | 8.015625 |  |  |  |  |  |
| 17.275 | | 210.6582 | 1.04579 | 4653.523 | 7.9375 |  |  |  |  |  |
| 17.28333 | | 210.7481 | 1.048116 | 4653.484 | 8.070313 |  |  |  |  |  |
| 17.29167 | | 210.853 | 1.047401 | 4653.438 | 8.109375 |  |  |  |  |  |
| 17.3 | | 210.9229 | 1.047403 | 4653.398 | 8.273438 |  |  |  |  |  |
| 17.30833 | | 211.0145 | 1.049662 | 4653.359 | 8.570313 |  |  |  |  |  |
| 17.31667 | | 211.1219 | 1.049134 | 4653.305 | 8.9375 |  |  |  |  |  |
| 17.325 | | 211.1943 | 1.049942 | 4653.266 | 9.164063 |  |  |  |  |  |
| 17.33333 | | 211.2859 | 1.052507 | 4653.18 | 9.46875 |  |  |  |  |  |
| 17.34167 | | 211.3949 | 1.052009 | 4653.094 | 9.648438 |  |  |  |  |  |
| 17.35 | | 211.4665 | 1.052564 | 4653.008 | 9.84375 |  |  |  |  |  |
| 17.35833 | | 211.5723 | 1.054745 | 4652.883 | 10.07031 |  |  |  |  |  |
| 17.36667 | | 211.6438 | 1.053898 | 4652.805 | 10.17969 |  |  |  |  |  |
| 17.375 | | 211.7537 | 1.054763 | 4652.656 | 10.3125 |  |  |  |  |  |
| 17.38333 | | 211.827 | 1.057095 | 4652.57 | 10.35156 |  |  |  |  |  |
| 17.39167 | | 211.9327 | 1.056607 | 4652.445 | 10.27344 |  |  |  |  |  |
| 17.4 | | 212.0035 | 1.057447 | 4652.359 | 10.10938 |  |  |  |  |  |
| 17.40833 | | 212.0959 | 1.060006 | 4652.25 | 10.14063 |  |  |  |  |  |
| 17.41667 | | 212.1865 | 1.059559 | 4652.164 | 10.11719 |  |  |  |  |  |
| 17.425 | | 212.2745 | 1.06076 | 4652.063 | 9.992188 |  |  |  |  |  |
| 17.43333 | | 212.3814 | 1.063604 | 4651.945 | 10.04688 |  |  |  |  |  |
| 17.44167 | | 212.4513 | 1.063027 | 4651.883 | 10.08594 |  |  |  |  |  |
| 17.45 | | 212.5417 | 1.063952 | 4651.828 | 10.02344 |  |  |  |  |  |
| 17.45833 | | 212.6346 | 1.066839 | 4651.742 | 9.796875 |  |  |  |  |  |
| 17.46667 | | 212.7242 | 1.066211 | 4651.664 | 9.78125 |  |  |  |  |  |
| 17.475 | | 212.8303 | 1.067918 | 4651.563 | 9.953125 |  |  |  |  |  |
| 17.48333 | | 212.901 | 1.070158 | 4651.492 | 9.960938 |  |  |  |  |  |
| 17.49167 | | 213.0095 | 1.069536 | 4651.391 | 9.96875 |  |  |  |  |  |
| 17.5 | | 213.0992 | 1.070898 | 4651.32 | 9.851563 |  |  |  |  |  |
| 17.50833 | | 213.1871 | 1.072509 | 4651.273 | 9.695313 |  |  |  |  |  |
| 17.51667 | | 213.2595 | 1.071443 | 4651.211 | 9.523438 |  |  |  |  |  |
| 17.525 | | 213.3507 | 1.072269 | 4651.148 | 9.453125 |  |  |  |  |  |
| 17.53333 | | 213.4552 | 1.074497 | 4651.008 | 9.539063 |  |  |  |  |  |
| 17.54167 | | 213.5407 | 1.073576 | 4650.93 | 9.578125 |  |  |  |  |  |
| 17.55 | | 213.6311 | 1.074905 | 4650.867 | 9.59375 |  |  |  |  |  |
| 17.55833 | | 213.7035 | 1.076337 | 4650.797 | 9.695313 |  |  |  |  |  |
| 17.56667 | | 213.8128 | 1.075032 | 4650.734 | 9.617188 |  |  |  |  |  |
| 17.575 | | 213.881 | 1.076002 | 4650.688 | 9.734375 |  |  |  |  |  |
| 17.58333 | | 213.9707 | 1.077832 | 4650.594 | 9.90625 |  |  |  |  |  |
| 17.59167 | | 214.0784 | 1.07676 | 4650.492 | 10.10938 |  |  |  |  |  |
| 17.6 | | 214.1491 | 1.077918 | 4650.422 | 10.34375 |  |  |  |  |  |
| 17.60833 | | 214.2379 | 1.080232 | 4650.32 | 10.53906 |  |  |  |  |  |
| 17.61667 | | 214.3291 | 1.079546 | 4650.258 | 10.42969 |  |  |  |  |  |
| 17.625 | | 214.4377 | 1.081379 | 4650.141 | 10.66406 |  |  |  |  |  |
| 17.63333 | | 214.5092 | 1.083183 | 4650.063 | 10.54688 |  |  |  |  |  |
| 17.64167 | | 214.6185 | 1.082416 | 4649.93 | 10.71094 |  |  |  |  |  |
| 17.65 | | 214.6892 | 1.083914 | 4649.836 | 10.63281 |  |  |  |  |  |
| 17.65833 | | 214.7797 | 1.08586 | 4649.727 | 10.27344 |  |  |  |  |  |
| 17.66667 | | 214.8693 | 1.085234 | 4649.664 | 10.10938 |  |  |  |  |  |
| 17.675 | | 214.977 | 1.088248 | 4649.531 | 9.789063 |  |  |  |  |  |
| 17.68333 | | 215.0477 | 1.089942 | 4649.477 | 9.585938 |  |  |  |  |  |
| 17.69167 | | 215.1538 | 1.089353 | 4649.344 | 9.140625 |  |  |  |  |  |
| 17.7 | | 215.2409 | 1.0914 | 4649.281 | 9.164063 |  |  |  |  |  |
| 17.70833 | | 215.3108 | 1.092971 | 4649.25 | 9.085938 |  |  |  |  |  |
| 17.71667 | | 215.4169 | 1.092564 | 4649.172 | 9.171875 |  |  |  |  |  |
| 17.725 | | 215.4876 | 1.094908 | 4649.141 | 8.84375 |  |  |  |  |  |
| 17.73333 | | 215.578 | 1.096827 | 4649.094 | 8.929688 |  |  |  |  |  |
| 17.74167 | | 215.6652 | 1.096286 | 4649.063 | 9.15625 |  |  |  |  |  |
| 17.75 | | 215.7737 | 1.098751 | 4648.977 | 9.3125 |  |  |  |  |  |
| 17.75833 | | 215.8625 | 1.099323 | 4648.906 | 9.695313 |  |  |  |  |  |
| 17.76667 | | 215.9546 | 1.099285 | 4648.813 | 10.01563 |  |  |  |  |  |
| 17.775 | | 216.0278 | 1.101047 | 4648.797 | 10.39063 |  |  |  |  |  |
| 17.78333 | | 216.1322 | 1.102051 | 4648.695 | 10.58594 |  |  |  |  |  |
| 17.79167 | | 216.2062 | 1.10171 | 4648.602 | 10.71094 |  |  |  |  |  |
| 17.8 | | 216.2974 | 1.10429 | 4648.508 | 10.67188 |  |  |  |  |  |
| 17.80833 | | 216.3854 | 1.105119 | 4648.383 | 10.69531 |  |  |  |  |  |
| 17.81667 | | 216.4717 | 1.104498 | 4648.258 | 10.77344 |  |  |  |  |  |
| 17.825 | | 216.5622 | 1.106904 | 4648.102 | 10.82813 |  |  |  |  |  |
| 17.83333 | | 216.6493 | 1.107959 | 4647.992 | 10.85938 |  |  |  |  |  |
| 17.84167 | | 216.7554 | 1.107763 | 4647.859 | 10.91406 |  |  |  |  |  |
| 17.85 | | 216.8417 | 1.109856 | 4647.789 | 10.74219 |  |  |  |  |  |
| 17.85833 | | 216.9132 | 1.111003 | 4647.711 | 10.47656 |  |  |  |  |  |
| 17.86667 | | 217.0053 | 1.110441 | 4647.609 | 10.5 |  |  |  |  |  |
| 17.875 | | 217.0949 | 1.113183 | 4647.516 | 10.57031 |  |  |  |  |  |
| 17.88333 | | 217.1994 | 1.114056 | 4647.398 | 10.8125 |  |  |  |  |  |
| 17.89167 | | 217.2684 | 1.113191 | 4647.32 | 10.96094 |  |  |  |  |  |
| 17.9 | | 217.3605 | 1.11579 | 4647.25 | 11.0625 |  |  |  |  |  |
| 17.90833 | | 217.4509 | 1.116663 | 4647.203 | 11.24219 |  |  |  |  |  |
| 17.91667 | | 217.5414 | 1.116179 | 4647.117 | 11.17969 |  |  |  |  |  |
| 17.925 | | 217.6466 | 1.118826 | 4647.008 | 11.24219 |  |  |  |  |  |
| 17.93333 | | 217.7198 | 1.119441 | 4646.914 | 11.375 |  |  |  |  |  |
| 17.94167 | | 217.8259 | 1.118683 | 4646.766 | 11.64844 |  |  |  |  |  |
| 17.95 | | 217.8974 | 1.120343 | 4646.68 | 11.58594 |  |  |  |  |  |
| 17.95833 | | 218.0051 | 1.120384 | 4646.539 | 11.55469 |  |  |  |  |  |
| 17.96667 | | 218.0766 | 1.119443 | 4646.477 | 11.58594 |  |  |  |  |  |
| 17.975 | | 218.1884 | 1.122269 | 4646.359 | 11.30469 |  |  |  |  |  |
| 17.98333 | | 218.26 | 1.122666 | 4646.258 | 11.28125 |  |  |  |  |  |
| 17.99167 | | 218.3504 | 1.122052 | 4646.133 | 11.05469 |  |  |  |  |  |
| 18 | | 218.4375 | 1.124589 | 4646.031 | 11.53906 |  |  |  |  |  |
| 18.00833 | | 218.5305 | 1.125376 | 4645.953 | 11.52344 |  |  |  |  |  |
| 18.01667 | | 218.6398 | 1.125753 | 4645.828 | 11 |  |  |  |  |  |
| 18.025 | | 218.713 | 1.128106 | 4645.781 | 11.03906 |  |  |  |  |  |
| 18.03333 | | 218.819 | 1.12914 | 4645.727 | 10.76563 |  |  |  |  |  |
| 18.04167 | | 218.8914 | 1.129116 | 4645.641 | 10.82031 |  |  |  |  |  |
| 18.05 | | 219.0007 | 1.132437 | 4645.453 | 10.70313 |  |  |  |  |  |
| 18.05833 | | 219.0706 | 1.13281 | 4645.398 | 10.72656 |  |  |  |  |  |
| 18.06667 | | 219.1759 | 1.133559 | 4645.344 | 10.79688 |  |  |  |  |  |
| 18.075 | | 219.2655 | 1.135999 | 4645.281 | 10.57031 |  |  |  |  |  |
| 18.08333 | | 219.3551 | 1.136221 | 4645.219 | 10.22656 |  |  |  |  |  |
| 18.09167 | | 219.4274 | 1.135943 | 4645.148 | 10.19531 |  |  |  |  |  |
| 18.1 | | 219.5368 | 1.139224 | 4645.063 | 10.51563 |  |  |  |  |  |
| 18.10833 | | 219.6083 | 1.139635 | 4644.984 | 10.67188 |  |  |  |  |  |
| 18.11667 | | 219.7021 | 1.139387 | 4644.898 | 11.05469 |  |  |  |  |  |
| 18.125 | | 219.7925 | 1.142467 | 4644.82 | 11.32813 |  |  |  |  |  |
| 18.13333 | | 219.8796 | 1.142975 | 4644.789 | 11.75 |  |  |  |  |  |
| 18.14167 | | 219.9882 | 1.143544 | 4644.695 | 11.92188 |  |  |  |  |  |
| 18.15 | | 220.077 | 1.145692 | 4644.563 | 12.29688 |  |  |  |  |  |
| 18.15833 | | 220.1493 | 1.145619 | 4644.469 | 12.5625 |  |  |  |  |  |
| 18.16667 | | 220.2578 | 1.145983 | 4644.281 | 12.90625 |  |  |  |  |  |
| 18.175 | | 220.3442 | 1.147698 | 4644.141 | 13.3125 |  |  |  |  |  |
| 18.18333 | | 220.4141 | 1.147684 | 4644.016 | 13.61719 |  |  |  |  |  |
| 18.19167 | | 220.4987 | 1.147142 | 4643.891 | 13.85938 |  |  |  |  |  |
| 18.2 | | 220.5843 | 1.150169 | 4643.75 | 13.89063 |  |  |  |  |  |
| 18.20833 | | 220.6903 | 1.150118 | 4643.57 | 14.15625 |  |  |  |  |  |
| 18.21667 | | 220.7627 | 1.149906 | 4643.453 | 14.03125 |  |  |  |  |  |
| 18.225 | | 220.8539 | 1.153073 | 4643.289 | 14.20313 |  |  |  |  |  |
| 18.23333 | | 220.9633 | 1.153532 | 4643.086 | 14.53125 |  |  |  |  |  |
| 18.24167 | | 221.034 | 1.153712 | 4642.969 | 14.54688 |  |  |  |  |  |
| 18.25 | | 221.1219 | 1.157317 | 4642.852 | 14.64063 |  |  |  |  |  |
| 18.25833 | | 221.2321 | 1.158401 | 4642.688 | 14.66406 |  |  |  |  |  |
| 18.26667 | | 221.3045 | 1.158731 | 4642.609 | 14.30469 |  |  |  |  |  |
| 18.275 | | 221.3974 | 1.162503 | 4642.477 | 14.125 |  |  |  |  |  |
| 18.28333 | | 221.4886 | 1.162564 | 4642.305 | 14 |  |  |  |  |  |
| 18.29167 | | 221.5791 | 1.162882 | 4642.18 | 13.67969 |  |  |  |  |  |
| 18.3 | | 221.6876 | 1.165328 | 4642.023 | 13.32813 |  |  |  |  |  |
| 18.30833 | | 221.7591 | 1.165113 | 4641.922 | 13.14844 |  |  |  |  |  |
| 18.31667 | | 221.8677 | 1.166367 | 4641.828 | 12.5625 |  |  |  |  |  |
| 18.325 | | 221.94 | 1.168591 | 4641.766 | 12.29688 |  |  |  |  |  |
| 18.33333 | | 222.0469 | 1.168508 | 4641.648 | 11.92969 |  |  |  |  |  |
| 18.34167 | | 222.1192 | 1.16889 | 4641.594 | 11.65625 |  |  |  |  |  |
| 18.35 | | 222.227 | 1.17148 | 4641.516 | 11.375 |  |  |  |  |  |
| 18.35833 | | 222.3009 | 1.171352 | 4641.453 | 11.22656 |  |  |  |  |  |
| 18.36667 | | 222.4095 | 1.172665 | 4641.414 | 11.03906 |  |  |  |  |  |
| 18.375 | | 222.481 | 1.174692 | 4641.375 | 11.07813 |  |  |  |  |  |
| 18.38333 | | 222.5698 | 1.174621 | 4641.328 | 11.48438 |  |  |  |  |  |
| 18.39167 | | 222.6767 | 1.176202 | 4641.273 | 11.42188 |  |  |  |  |  |
| 18.4 | | 222.7638 | 1.177891 | 4641.188 | 11.39844 |  |  |  |  |  |
| 18.40833 | | 222.8551 | 1.177758 | 4641.125 | 11.63281 |  |  |  |  |  |
| 18.41667 | | 222.9307 | 1.178724 | 4641.078 | 11.79688 |  |  |  |  |  |
| 18.425 | | 223.0228 | 1.180997 | 4640.984 | 12.03125 |  |  |  |  |  |
| 18.43333 | | 223.1133 | 1.180767 | 4640.844 | 12.41406 |  |  |  |  |  |
| 18.44167 | | 223.2218 | 1.182254 | 4640.734 | 12.72656 |  |  |  |  |  |
| 18.45 | | 223.2933 | 1.183832 | 4640.656 | 12.9375 |  |  |  |  |  |
| 18.45833 | | 223.3846 | 1.183625 | 4640.547 | 13.23438 |  |  |  |  |  |
| 18.46667 | | 223.4775 | 1.184859 | 4640.422 | 13.28125 |  |  |  |  |  |
| 18.475 | | 223.5836 | 1.187129 | 4640.25 | 13.59375 |  |  |  |  |  |
| 18.48333 | | 223.6765 | 1.18717 | 4640.117 | 13.94531 |  |  |  |  |  |
| 18.49167 | | 223.7702 | 1.188912 | 4639.969 | 14.28125 |  |  |  |  |  |
| 18.5 | | 223.8615 | 1.190299 | 4639.813 | 14.28125 |  |  |  |  |  |
| 18.50833 | | 223.9494 | 1.190331 | 4639.672 | 14.41406 |  |  |  |  |  |
| 18.51667 | | 224.0399 | 1.192381 | 4639.539 | 14.53906 |  |  |  |  |  |
| 18.525 | | 224.113 | 1.194111 | 4639.43 | 14.69531 |  |  |  |  |  |
| 18.53333 | | 224.2027 | 1.19411 | 4639.25 | 14.875 |  |  |  |  |  |
| 18.54167 | | 224.2947 | 1.195546 | 4639.086 | 14.83594 |  |  |  |  |  |
| 18.55 | | 224.4033 | 1.19741 | 4638.93 | 14.82813 |  |  |  |  |  |
| 18.55833 | | 224.4962 | 1.197701 | 4638.781 | 14.90625 |  |  |  |  |  |
| 18.56667 | | 224.5685 | 1.199215 | 4638.664 | 14.88281 |  |  |  |  |  |
| 18.575 | | 224.6581 | 1.201162 | 4638.516 | 14.70313 |  |  |  |  |  |
| 18.58333 | | 224.765 | 1.201451 | 4638.336 | 14.75781 |  |  |  |  |  |
| 18.59167 | | 224.839 | 1.203094 | 4638.242 | 14.60938 |  |  |  |  |  |
| 18.6 | | 224.9303 | 1.204554 | 4638.117 | 14.5 |  |  |  |  |  |
| 18.60833 | | 225.0405 | 1.204988 | 4637.953 | 14.14844 |  |  |  |  |  |
| 18.61667 | | 225.1136 | 1.207144 | 4637.859 | 14.07813 |  |  |  |  |  |
| 18.625 | | 225.2024 | 1.209153 | 4637.766 | 14.125 |  |  |  |  |  |
| 18.63333 | | 225.2945 | 1.209956 | 4637.633 | 14.07813 |  |  |  |  |  |
| 18.64167 | | 225.3858 | 1.212345 | 4637.523 | 13.9375 |  |  |  |  |  |
| 18.65 | | 225.4787 | 1.21375 | 4637.414 | 13.74219 |  |  |  |  |  |
| 18.65833 | | 225.5708 | 1.213997 | 4637.336 | 13.97656 |  |  |  |  |  |
| 18.66667 | | 225.6628 | 1.216213 | 4637.242 | 13.77344 |  |  |  |  |  |
| 18.675 | | 225.7549 | 1.21718 | 4637.125 | 13.61719 |  |  |  |  |  |
| 18.68333 | | 225.8454 | 1.217754 | 4637.023 | 13.49219 |  |  |  |  |  |
| 18.69167 | | 225.9366 | 1.220067 | 4636.93 | 13.46094 |  |  |  |  |  |
| 18.7 | | 226.046 | 1.220206 | 4636.813 | 13.375 |  |  |  |  |  |
| 18.70833 | | 226.1372 | 1.221204 | 4636.695 | 13.28125 |  |  |  |  |  |
| 18.71667 | | 226.2104 | 1.223172 | 4636.625 | 13.24219 |  |  |  |  |  |
| 18.725 | | 226.2976 | 1.22357 | 4636.539 | 13.17969 |  |  |  |  |  |
| 18.73333 | | 226.388 | 1.224669 | 4636.453 | 13.36719 |  |  |  |  |  |
| 18.74167 | | 226.476 | 1.227438 | 4636.352 | 13.00781 |  |  |  |  |  |
| 18.75 | | 226.5672 | 1.228291 | 4636.266 | 13.21875 |  |  |  |  |  |
| 18.75833 | | 226.6741 | 1.230196 | 4636.141 | 12.875 |  |  |  |  |  |
| 18.76667 | | 226.7465 | 1.232596 | 4636.07 | 12.90625 |  |  |  |  |  |
| 18.775 | | 226.8353 | 1.233461 | 4635.969 | 13.03906 |  |  |  |  |  |
| 18.78333 | | 226.9249 | 1.234747 | 4635.828 | 13.00781 |  |  |  |  |  |
| 18.79167 | | 227.035 | 1.238041 | 4635.719 | 13.21094 |  |  |  |  |  |
| 18.8 | | 227.1247 | 1.238069 | 4635.617 | 13.24219 |  |  |  |  |  |
| 18.80833 | | 227.2168 | 1.238642 | 4635.547 | 13.28125 |  |  |  |  |  |
| 18.81667 | | 227.2883 | 1.239945 | 4635.461 | 13.34375 |  |  |  |  |  |
| 18.825 | | 227.396 | 1.240289 | 4635.305 | 13.65625 |  |  |  |  |  |
| 18.83333 | | 227.4872 | 1.240841 | 4635.195 | 13.85156 |  |  |  |  |  |
| 18.84167 | | 227.5785 | 1.242678 | 4635.055 | 14.46094 |  |  |  |  |  |
| 18.85 | | 227.6689 | 1.242553 | 4634.938 | 14.79688 |  |  |  |  |  |
| 18.85833 | | 227.7627 | 1.242778 | 4634.805 | 14.99219 |  |  |  |  |  |
| 18.86667 | | 227.8531 | 1.244449 | 4634.695 | 15.46094 |  |  |  |  |  |
| 18.875 | | 227.9477 | 1.244335 | 4634.523 | 15.79688 |  |  |  |  |  |
| 18.88333 | | 228.0381 | 1.245213 | 4634.383 | 16.34375 |  |  |  |  |  |
| 18.89167 | | 228.1302 | 1.247629 | 4634.164 | 16.57031 |  |  |  |  |  |
| 18.9 | | 228.2239 | 1.24758 | 4633.992 | 16.96094 |  |  |  |  |  |
| 18.90833 | | 228.316 | 1.249244 | 4633.836 | 16.89844 |  |  |  |  |  |
| 18.91667 | | 228.4064 | 1.251723 | 4633.641 | 17.23438 |  |  |  |  |  |
| 18.925 | | 228.4969 | 1.251928 | 4633.453 | 17.21094 |  |  |  |  |  |
| 18.93333 | | 228.5898 | 1.254111 | 4633.242 | 17.35938 |  |  |  |  |  |
| 18.94167 | | 228.6802 | 1.25633 | 4633.07 | 17.46875 |  |  |  |  |  |
| 18.95 | | 228.7698 | 1.25607 | 4632.883 | 17.5 |  |  |  |  |  |
| 18.95833 | | 228.8578 | 1.257159 | 4632.742 | 17.51563 |  |  |  |  |  |
| 18.96667 | | 228.9507 | 1.259179 | 4632.555 | 17.25 |  |  |  |  |  |
| 18.975 | | 229.0428 | 1.258983 | 4632.414 | 17.03125 |  |  |  |  |  |
| 18.98333 | | 229.1135 | 1.259619 | 4632.289 | 17.07813 |  |  |  |  |  |
| 18.99167 | | 229.2262 | 1.262228 | 4632.102 | 17.10938 |  |  |  |  |  |
| 19 | | 229.319 | 1.261711 | 4631.961 | 17.04688 |  |  |  |  |  |
| 19.00833 | | 229.4103 | 1.262703 | 4631.82 | 17.1875 |  |  |  |  |  |
| 19.01667 | | 229.4991 | 1.264235 | 4631.711 | 17.36719 |  |  |  |  |  |
| 19.025 | | 229.5904 | 1.263855 | 4631.602 | 17.35938 |  |  |  |  |  |
| 19.03333 | | 229.6816 | 1.265334 | 4631.477 | 17.71094 |  |  |  |  |  |
| 19.04167 | | 229.7713 | 1.267496 | 4631.32 | 17.77344 |  |  |  |  |  |
| 19.05 | | 229.8633 | 1.267573 | 4631.195 | 18.03906 |  |  |  |  |  |
| 19.05833 | | 229.9538 | 1.269445 | 4631.031 | 18.57813 |  |  |  |  |  |
| 19.06667 | | 230.045 | 1.271276 | 4630.875 | 18.70313 |  |  |  |  |  |
| 19.075 | | 230.1336 | 1.270357 | 4630.734 | 18.69531 |  |  |  |  |  |
| 19.08333 | | 230.2218 | 1.271822 | 4630.547 | 18.78125 |  |  |  |  |  |
| 19.09167 | | 230.3131 | 1.27351 | 4630.391 | 18.85938 |  |  |  |  |  |
| 19.1 | | 230.4021 | 1.272819 | 4630.211 | 18.89844 |  |  |  |  |  |
| 19.10833 | | 230.4911 | 1.274821 | 4629.984 | 18.66406 |  |  |  |  |  |
| 19.11667 | | 230.5809 | 1.27647 | 4629.82 | 18.13281 |  |  |  |  |  |
| 19.125 | | 230.6698 | 1.276013 | 4629.672 | 17.99219 |  |  |  |  |  |
| 19.13333 | | 230.7572 | 1.278134 | 4629.508 | 17.78906 |  |  |  |  |  |
| 19.14167 | | 230.8446 | 1.280324 | 4629.344 | 17.375 |  |  |  |  |  |
| 19.15 | | 230.9328 | 1.280382 | 4629.188 | 17.44531 |  |  |  |  |  |
| 19.15833 | | 231.0217 | 1.282864 | 4629.078 | 17.44531 |  |  |  |  |  |
| 19.16667 | | 231.1099 | 1.28508 | 4629 | 17.47656 |  |  |  |  |  |
| 19.175 | | 231.1965 | 1.285045 | 4628.875 | 17.61719 |  |  |  |  |  |
| 19.18333 | | 231.2847 | 1.287339 | 4628.766 | 17.33594 |  |  |  |  |  |
| 19.19167 | | 231.3736 | 1.288863 | 4628.672 | 17.53906 |  |  |  |  |  |
| 19.2 | | 231.4642 | 1.288219 | 4628.523 | 17.96875 |  |  |  |  |  |
| 19.20833 | | 231.558 | 1.290383 | 4628.383 | 18.36719 |  |  |  |  |  |
| 19.21667 | | 231.6486 | 1.291386 | 4628.234 | 18.83594 |  |  |  |  |  |
| 19.225 | | 231.7376 | 1.290451 | 4628.07 | 18.95313 |  |  |  |  |  |
| 19.23333 | | 231.8289 | 1.293554 | 4627.961 | 19.14063 |  |  |  |  |  |
| 19.24167 | | 231.9195 | 1.294758 | 4627.773 | 19.39844 |  |  |  |  |  |
| 19.25 | | 232.0093 | 1.294045 | 4627.57 | 19.53906 |  |  |  |  |  |
| 19.25833 | | 232.0991 | 1.297249 | 4627.359 | 19.5 |  |  |  |  |  |
| 19.26667 | | 232.1897 | 1.298211 | 4627.133 | 20.0625 |  |  |  |  |  |
| 19.275 | | 232.2786 | 1.297804 | 4626.938 | 20.38281 |  |  |  |  |  |
| 19.28333 | | 232.3668 | 1.301539 | 4626.758 | 20.51563 |  |  |  |  |  |
| 19.29167 | | 232.455 | 1.30325 | 4626.555 | 20.46875 |  |  |  |  |  |
| 19.3 | | 232.5455 | 1.302937 | 4626.367 | 20.375 |  |  |  |  |  |
| 19.30833 | | 232.6393 | 1.306319 | 4626.203 | 20.32813 |  |  |  |  |  |
| 19.31667 | | 232.7299 | 1.306916 | 4625.961 | 20.40625 |  |  |  |  |  |
| 19.325 | | 232.8189 | 1.307018 | 4625.742 | 20.52344 |  |  |  |  |  |
| 19.33333 | | 232.9063 | 1.309994 | 4625.563 | 20.73438 |  |  |  |  |  |
| 19.34167 | | 232.9952 | 1.31052 | 4625.398 | 20.75 |  |  |  |  |  |
| 19.35 | | 233.085 | 1.310645 | 4625.25 | 20.71875 |  |  |  |  |  |
| 19.35833 | | 233.1732 | 1.314141 | 4625.086 | 20.625 |  |  |  |  |  |
| 19.36667 | | 233.2614 | 1.315022 | 4624.906 | 20.78125 |  |  |  |  |  |
| 19.375 | | 233.3471 | 1.315014 | 4624.734 | 20.76563 |  |  |  |  |  |
| 19.38333 | | 233.4345 | 1.318888 | 4624.539 | 20.73438 |  |  |  |  |  |
| 19.39167 | | 233.5195 | 1.32004 | 4624.367 | 20.92188 |  |  |  |  |  |
| 19.4 | | 233.6068 | 1.320058 | 4624.203 | 20.94531 |  |  |  |  |  |
| 19.40833 | | 233.695 | 1.323795 | 4624.047 | 21.00781 |  |  |  |  |  |
| 19.41667 | | 233.7872 | 1.325046 | 4623.844 | 21.13281 |  |  |  |  |  |
| 19.425 | | 233.8786 | 1.325732 | 4623.68 | 21.16406 |  |  |  |  |  |
| 19.43333 | | 233.9644 | 1.32972 | 4623.5 | 21.27344 |  |  |  |  |  |
| 19.44167 | | 234.0525 | 1.330538 | 4623.313 | 21.50781 |  |  |  |  |  |
| 19.45 | | 234.1431 | 1.330497 | 4623.133 | 21.5625 |  |  |  |  |  |
| 19.45833 | | 234.2329 | 1.333908 | 4622.945 | 21.64844 |  |  |  |  |  |
| 19.46667 | | 234.3235 | 1.33465 | 4622.75 | 21.82031 |  |  |  |  |  |
| 19.475 | | 234.4116 | 1.334629 | 4622.563 | 22.08594 |  |  |  |  |  |
| 19.48333 | | 234.4998 | 1.338561 | 4622.375 | 22.14844 |  |  |  |  |  |
| 19.49167 | | 234.5904 | 1.338814 | 4622.172 | 22.13281 |  |  |  |  |  |
| 19.5 | | 234.681 | 1.339309 | 4621.984 | 22.20313 |  |  |  |  |  |
| 19.50833 | | 234.7683 | 1.343287 | 4621.797 | 22.35938 |  |  |  |  |  |
| 19.51667 | | 234.8597 | 1.343955 | 4621.602 | 22.32031 |  |  |  |  |  |
| 19.525 | | 234.9535 | 1.34467 | 4621.391 | 22.53906 |  |  |  |  |  |
| 19.53333 | | 235.0433 | 1.348333 | 4621.211 | 22.59375 |  |  |  |  |  |
| 19.54167 | | 235.1331 | 1.348847 | 4621.039 | 22.47656 |  |  |  |  |  |
| 19.55 | | 235.222 | 1.34953 | 4620.852 | 22.48438 |  |  |  |  |  |
| 19.55833 | | 235.311 | 1.353284 | 4620.648 | 22.52344 |  |  |  |  |  |
| 19.56667 | | 235.4024 | 1.353594 | 4620.469 | 22.53906 |  |  |  |  |  |
| 19.575 | | 235.4954 | 1.354656 | 4620.25 | 22.66406 |  |  |  |  |  |
| 19.58333 | | 235.5827 | 1.357849 | 4620.063 | 22.59375 |  |  |  |  |  |
| 19.59167 | | 235.6725 | 1.357663 | 4619.898 | 22.59375 |  |  |  |  |  |
| 19.6 | | 235.7663 | 1.358594 | 4619.695 | 22.625 |  |  |  |  |  |
| 19.60833 | | 235.8537 | 1.362284 | 4619.508 | 22.33594 |  |  |  |  |  |
| 19.61667 | | 235.9435 | 1.362653 | 4619.313 | 22.22656 |  |  |  |  |  |
| 19.625 | | 236.0364 | 1.363673 | 4619.102 | 22.36719 |  |  |  |  |  |
| 19.63333 | | 236.1254 | 1.367251 | 4618.93 | 22.25781 |  |  |  |  |  |
| 19.64167 | | 236.2208 | 1.366961 | 4618.734 | 22.21094 |  |  |  |  |  |
| 19.65 | | 236.3098 | 1.368049 | 4618.547 | 22.17969 |  |  |  |  |  |
| 19.65833 | | 236.3964 | 1.370935 | 4618.398 | 22.28125 |  |  |  |  |  |
| 19.66667 | | 236.4861 | 1.371255 | 4618.219 | 22.28906 |  |  |  |  |  |
| 19.675 | | 236.5783 | 1.373026 | 4618.008 | 22.36719 |  |  |  |  |  |
| 19.68333 | | 236.6625 | 1.375823 | 4617.844 | 22.39063 |  |  |  |  |  |
| 19.69167 | | 236.7523 | 1.375551 | 4617.664 | 22.4375 |  |  |  |  |  |
| 19.7 | | 236.8436 | 1.37676 | 4617.492 | 22.49219 |  |  |  |  |  |
| 19.70833 | | 236.9302 | 1.379443 | 4617.297 | 22.78906 |  |  |  |  |  |
| 19.71667 | | 237.02 | 1.379384 | 4617.102 | 23.17969 |  |  |  |  |  |
| 19.725 | | 237.1066 | 1.381047 | 4616.906 | 23.29688 |  |  |  |  |  |
| 19.73333 | | 237.1947 | 1.384124 | 4616.727 | 23.64063 |  |  |  |  |  |
| 19.74167 | | 237.2837 | 1.384489 | 4616.523 | 23.92188 |  |  |  |  |  |
| 19.75 | | 237.3743 | 1.386553 | 4616.344 | 24.33594 |  |  |  |  |  |
| 19.75833 | | 237.4633 | 1.389388 | 4616.102 | 24.36719 |  |  |  |  |  |
| 19.76667 | | 237.5562 | 1.389806 | 4615.867 | 24.77344 |  |  |  |  |  |
| 19.775 | | 237.6452 | 1.391937 | 4615.648 | 24.96875 |  |  |  |  |  |
| 19.78333 | | 237.7342 | 1.394899 | 4615.406 | 25.39063 |  |  |  |  |  |
| 19.79167 | | 237.828 | 1.395667 | 4615.156 | 25.46094 |  |  |  |  |  |
| 19.8 | | 237.9178 | 1.398375 | 4614.906 | 25.67969 |  |  |  |  |  |
| 19.80833 | | 238.0091 | 1.401194 | 4614.695 | 25.77344 |  |  |  |  |  |
| 19.81667 | | 238.1005 | 1.401706 | 4614.445 | 25.92188 |  |  |  |  |  |
| 19.825 | | 238.1911 | 1.404147 | 4614.203 | 25.83594 |  |  |  |  |  |
| 19.83333 | | 238.2817 | 1.407006 | 4613.938 | 25.83594 |  |  |  |  |  |
| 19.84167 | | 238.3707 | 1.407665 | 4613.719 | 25.99219 |  |  |  |  |  |
| 19.85 | | 238.4596 | 1.410039 | 4613.469 | 25.94531 |  |  |  |  |  |
| 19.85833 | | 238.5534 | 1.412837 | 4613.25 | 26.10156 |  |  |  |  |  |
| 19.86667 | | 238.6448 | 1.413322 | 4613.016 | 26 |  |  |  |  |  |
| 19.875 | | 238.733 | 1.415451 | 4612.813 | 25.99219 |  |  |  |  |  |
| 19.88333 | | 238.8235 | 1.417693 | 4612.602 | 25.91406 |  |  |  |  |  |
| 19.89167 | | 238.9133 | 1.417861 | 4612.367 | 26.38281 |  |  |  |  |  |
| 19.9 | | 239.0047 | 1.420872 | 4612.156 | 26.39844 |  |  |  |  |  |
| 19.90833 | | 239.0977 | 1.423368 | 4611.922 | 26.64844 |  |  |  |  |  |
| 19.91667 | | 239.1867 | 1.424272 | 4611.727 | 26.9375 |  |  |  |  |  |
| 19.925 | | 239.2756 | 1.427282 | 4611.508 | 27.03906 |  |  |  |  |  |
| 19.93333 | | 239.3702 | 1.429664 | 4611.305 | 27.15625 |  |  |  |  |  |
| 19.94167 | | 239.46 | 1.430839 | 4611.031 | 27.17188 |  |  |  |  |  |
| 19.95 | | 239.549 | 1.433834 | 4610.805 | 27.57813 |  |  |  |  |  |
| 19.95833 | | 239.6388 | 1.43587 | 4610.547 | 27.97656 |  |  |  |  |  |
| 19.96667 | | 239.7333 | 1.437032 | 4610.289 | 28.07813 |  |  |  |  |  |
| 19.975 | | 239.8239 | 1.440158 | 4610.055 | 27.96094 |  |  |  |  |  |
| 19.98333 | | 239.9169 | 1.441508 | 4609.82 | 28.16406 |  |  |  |  |  |
| 19.99167 | | 240.0059 | 1.442439 | 4609.586 | 28.07031 |  |  |  |  |  |
| 20 | | 240.0957 | 1.445004 | 4609.305 | 27.74219 |  |  |  |  |  |
| 20.00833 | | 240.1886 | 1.445824 | 4609.031 | 27.95313 |  |  |  |  |  |
| 20.01667 | | 240.2752 | 1.446517 | 4608.781 | 28.0625 |  |  |  |  |  |
| 20.025 | | 240.3658 | 1.449135 | 4608.57 | 27.95313 |  |  |  |  |  |
| 20.03333 | | 240.4556 | 1.450133 | 4608.313 | 27.70313 |  |  |  |  |  |
| 20.04167 | | 240.5461 | 1.451751 | 4608.109 | 27.48438 |  |  |  |  |  |
| 20.05 | | 240.6367 | 1.454918 | 4607.922 | 27.50781 |  |  |  |  |  |
| 20.05833 | | 240.7289 | 1.456054 | 4607.672 | 27.49219 |  |  |  |  |  |
| 20.06667 | | 240.8155 | 1.457833 | 4607.422 | 27.17188 |  |  |  |  |  |
| 20.075 | | 240.9053 | 1.46081 | 4607.211 | 27.25 |  |  |  |  |  |
| 20.08333 | | 240.9982 | 1.461828 | 4607.016 | 27.28906 |  |  |  |  |  |
| 20.09167 | | 241.0872 | 1.463648 | 4606.836 | 27.17969 |  |  |  |  |  |
| 20.1 | | 241.177 | 1.466978 | 4606.594 | 27.125 |  |  |  |  |  |
| 20.10833 | | 241.2636 | 1.468287 | 4606.375 | 27.48438 |  |  |  |  |  |
| 20.11667 | | 241.3557 | 1.470099 | 4606.188 | 27.67969 |  |  |  |  |  |
| 20.125 | | 241.4455 | 1.473489 | 4605.945 | 28.23438 |  |  |  |  |  |
| 20.13333 | | 241.5305 | 1.474731 | 4605.719 | 28.39844 |  |  |  |  |  |
| 20.14167 | | 241.6179 | 1.476654 | 4605.508 | 28.77344 |  |  |  |  |  |
| 20.15 | | 241.7101 | 1.479911 | 4605.273 | 29.05469 |  |  |  |  |  |
| 20.15833 | | 241.8014 | 1.481326 | 4605 | 29.49219 |  |  |  |  |  |
| 20.16667 | | 241.8896 | 1.483397 | 4604.742 | 30.0625 |  |  |  |  |  |
| 20.175 | | 241.9786 | 1.48692 | 4604.422 | 30.25781 |  |  |  |  |  |
| 20.18333 | | 242.07 | 1.488632 | 4604.164 | 30.53125 |  |  |  |  |  |
| 20.19167 | | 242.1629 | 1.490891 | 4603.867 | 30.53906 |  |  |  |  |  |
| 20.2 | | 242.2503 | 1.494478 | 4603.586 | 30.67188 |  |  |  |  |  |
| 20.20833 | | 242.3417 | 1.495986 | 4603.281 | 30.61719 |  |  |  |  |  |
| 20.21667 | | 242.4323 | 1.498101 | 4602.953 | 30.8125 |  |  |  |  |  |
| 20.225 | | 242.5245 | 1.501332 | 4602.68 | 30.84375 |  |  |  |  |  |
| 20.23333 | | 242.6142 | 1.502638 | 4602.391 | 31.01563 |  |  |  |  |  |
| 20.24167 | | 242.704 | 1.504689 | 4602.133 | 31.17969 |  |  |  |  |  |
| 20.25 | | 242.7954 | 1.507963 | 4601.859 | 31.34375 |  |  |  |  |  |
| 20.25833 | | 242.8876 | 1.508994 | 4601.617 | 31.83594 |  |  |  |  |  |
| 20.26667 | | 242.983 | 1.511724 | 4601.344 | 32.28906 |  |  |  |  |  |
| 20.275 | | 243.0751 | 1.515111 | 4601.094 | 32.78906 |  |  |  |  |  |
| 20.28333 | | 243.1713 | 1.515623 | 4600.805 | 33.3125 |  |  |  |  |  |
| 20.29167 | | 243.2627 | 1.519044 | 4600.523 | 33.6875 |  |  |  |  |  |
| 20.3 | | 243.3541 | 1.522201 | 4600.242 | 33.82813 |  |  |  |  |  |
| 20.30833 | | 243.4455 | 1.522867 | 4599.914 | 34.50781 |  |  |  |  |  |
| 20.31667 | | 243.5368 | 1.526929 | 4599.578 | 34.96094 |  |  |  |  |  |
| 20.325 | | 243.6282 | 1.530068 | 4599.234 | 35.22656 |  |  |  |  |  |
| 20.33333 | | 243.7196 | 1.530334 | 4598.891 | 35.46875 |  |  |  |  |  |
| 20.34167 | | 243.8126 | 1.534339 | 4598.57 | 35.60156 |  |  |  |  |  |
| 20.35 | | 243.9008 | 1.536866 | 4598.258 | 35.84375 |  |  |  |  |  |
| 20.35833 | | 243.9922 | 1.536794 | 4597.883 | 35.6875 |  |  |  |  |  |
| 20.36667 | | 244.0811 | 1.541018 | 4597.523 | 35.90625 |  |  |  |  |  |
| 20.375 | | 244.1733 | 1.543235 | 4597.211 | 36.0625 |  |  |  |  |  |
| 20.38333 | | 244.2671 | 1.54321 | 4596.883 | 35.95313 |  |  |  |  |  |
| 20.39167 | | 244.3569 | 1.546717 | 4596.57 | 35.91406 |  |  |  |  |  |
| 20.4 | | 244.4499 | 1.548985 | 4596.258 | 35.70313 |  |  |  |  |  |
| 20.40833 | | 244.538 | 1.548981 | 4595.984 | 35.64063 |  |  |  |  |  |
| 20.41667 | | 244.6294 | 1.553235 | 4595.664 | 35.46094 |  |  |  |  |  |
| 20.425 | | 244.7184 | 1.555822 | 4595.352 | 35.28125 |  |  |  |  |  |
| 20.43333 | | 244.8098 | 1.556086 | 4595.07 | 35.17188 |  |  |  |  |  |
| 20.44167 | | 244.8987 | 1.560468 | 4594.805 | 35.42969 |  |  |  |  |  |
| 20.45 | | 244.9901 | 1.56267 | 4594.539 | 35.22656 |  |  |  |  |  |
| 20.45833 | | 245.0775 | 1.563399 | 4594.266 | 35.08594 |  |  |  |  |  |
| 20.46667 | | 245.1657 | 1.567698 | 4594 | 35.00781 |  |  |  |  |  |
| 20.475 | | 245.2587 | 1.570656 | 4593.742 | 35.07813 |  |  |  |  |  |
| 20.48333 | | 245.3452 | 1.571758 | 4593.469 | 35.03125 |  |  |  |  |  |
| 20.49167 | | 245.439 | 1.576741 | 4593.156 | 35.125 |  |  |  |  |  |
| 20.5 | | 245.5296 | 1.579089 | 4592.891 | 35.34375 |  |  |  |  |  |
| 20.50833 | | 245.625 | 1.580091 | 4592.625 | 35.44531 |  |  |  |  |  |
| 20.51667 | | 245.7164 | 1.584617 | 4592.344 | 35.63281 |  |  |  |  |  |
| 20.525 | | 245.8101 | 1.586643 | 4592.047 | 35.77344 |  |  |  |  |  |
| 20.53333 | | 245.8999 | 1.587895 | 4591.758 | 36.04688 |  |  |  |  |  |
| 20.54167 | | 245.9913 | 1.591922 | 4591.453 | 36.41406 |  |  |  |  |  |
| 20.55 | | 246.0843 | 1.593623 | 4591.125 | 36.64063 |  |  |  |  |  |
| 20.55833 | | 246.1749 | 1.594966 | 4590.813 | 36.69531 |  |  |  |  |  |
| 20.56667 | | 246.2702 | 1.598881 | 4590.492 | 36.96875 |  |  |  |  |  |
| 20.575 | | 246.36 | 1.600677 | 4590.18 | 36.9375 |  |  |  |  |  |
| 20.58333 | | 246.4522 | 1.602901 | 4589.828 | 36.94531 |  |  |  |  |  |
| 20.59167 | | 246.5412 | 1.607079 | 4589.492 | 37.1875 |  |  |  |  |  |
| 20.6 | | 246.6342 | 1.608851 | 4589.148 | 37.17969 |  |  |  |  |  |
| 20.60833 | | 246.7247 | 1.610955 | 4588.844 | 37.23438 |  |  |  |  |  |
| 20.61667 | | 246.8177 | 1.614587 | 4588.5 | 37.05469 |  |  |  |  |  |
| 20.625 | | 246.9115 | 1.616023 | 4588.211 | 37.10156 |  |  |  |  |  |
| 20.63333 | | 247.0061 | 1.618812 | 4587.898 | 37.32813 |  |  |  |  |  |
| 20.64167 | | 247.0999 | 1.622981 | 4587.563 | 37.5625 |  |  |  |  |  |
| 20.65 | | 247.1881 | 1.624837 | 4587.25 | 37.71875 |  |  |  |  |  |
| 20.65833 | | 247.2778 | 1.627416 | 4586.93 | 37.86719 |  |  |  |  |  |
| 20.66667 | | 247.3684 | 1.631213 | 4586.648 | 38.05469 |  |  |  |  |  |
| 20.675 | | 247.4574 | 1.63291 | 4586.32 | 38.14063 |  |  |  |  |  |
| 20.68333 | | 247.5464 | 1.635855 | 4585.977 | 38.38281 |  |  |  |  |  |
| 20.69167 | | 247.6353 | 1.639997 | 4585.625 | 38.54688 |  |  |  |  |  |
| 20.7 | | 247.7275 | 1.64229 | 4585.289 | 38.8125 |  |  |  |  |  |
| 20.70833 | | 247.8205 | 1.646166 | 4584.945 | 39.28906 |  |  |  |  |  |
| 20.71667 | | 247.9095 | 1.650396 | 4584.602 | 39.67969 |  |  |  |  |  |
| 20.725 | | 247.9969 | 1.652318 | 4584.266 | 39.92969 |  |  |  |  |  |
| 20.73333 | | 248.089 | 1.655274 | 4583.922 | 40.21875 |  |  |  |  |  |
| 20.74167 | | 248.1844 | 1.659613 | 4583.57 | 40.70313 |  |  |  |  |  |
| 20.75 | | 248.2806 | 1.661485 | 4583.211 | 41.14844 |  |  |  |  |  |
| 20.75833 | | 248.3736 | 1.664777 | 4582.82 | 41.64844 |  |  |  |  |  |
| 20.76667 | | 248.4642 | 1.66841 | 4582.43 | 42.08594 |  |  |  |  |  |
| 20.775 | | 248.558 | 1.670255 | 4582.078 | 42.28125 |  |  |  |  |  |
| 20.78333 | | 248.6485 | 1.674102 | 4581.703 | 42.75781 |  |  |  |  |  |
| 20.79167 | | 248.7391 | 1.677575 | 4581.297 | 42.99219 |  |  |  |  |  |
| 20.8 | | 248.8313 | 1.679238 | 4580.898 | 43.24219 |  |  |  |  |  |
| 20.80833 | | 248.9251 | 1.682944 | 4580.492 | 43.92188 |  |  |  |  |  |
| 20.81667 | | 249.0173 | 1.685763 | 4580.086 | 44.36719 |  |  |  |  |  |
| 20.825 | | 249.1078 | 1.687409 | 4579.719 | 44.76563 |  |  |  |  |  |
| 20.83333 | | 249.196 | 1.691571 | 4579.305 | 45.39844 |  |  |  |  |  |
| 20.84167 | | 249.2874 | 1.694914 | 4578.93 | 45.78906 |  |  |  |  |  |
| 20.85 | | 249.3812 | 1.697103 | 4578.531 | 46.19531 |  |  |  |  |  |
| 20.85833 | | 249.4726 | 1.701471 | 4578.094 | 46.80469 |  |  |  |  |  |
| 20.86667 | | 249.5656 | 1.704221 | 4577.656 | 47.53125 |  |  |  |  |  |
| 20.875 | | 249.6561 | 1.706246 | 4577.242 | 47.89063 |  |  |  |  |  |
| 20.88333 | | 249.7467 | 1.710295 | 4576.781 | 48.125 |  |  |  |  |  |
| 20.89167 | | 249.8397 | 1.713261 | 4576.359 | 48.46094 |  |  |  |  |  |
| 20.9 | | 249.9343 | 1.7155 | 4575.922 | 48.59375 |  |  |  |  |  |
| 20.90833 | | 250.0281 | 1.7201 | 4575.453 | 48.91406 |  |  |  |  |  |
| 20.91667 | | 250.1194 | 1.722843 | 4574.969 | 48.96094 |  |  |  |  |  |
| 20.925 | | 250.2116 | 1.724869 | 4574.523 | 49.21094 |  |  |  |  |  |
| 20.93333 | | 250.3022 | 1.729332 | 4574.094 | 49.42969 |  |  |  |  |  |
| 20.94167 | | 250.3992 | 1.731852 | 4573.656 | 49.53125 |  |  |  |  |  |
| 20.95 | | 250.4914 | 1.734278 | 4573.234 | 49.35938 |  |  |  |  |  |
| 20.95833 | | 250.5868 | 1.739428 | 4572.797 | 49.375 |  |  |  |  |  |
| 20.96667 | | 250.6806 | 1.741839 | 4572.383 | 49.49219 |  |  |  |  |  |
| 20.975 | | 250.7743 | 1.744848 | 4571.961 | 49.71875 |  |  |  |  |  |
| 20.98333 | | 250.8657 | 1.750354 | 4571.523 | 50.07813 |  |  |  |  |  |
| 20.99167 | | 250.9579 | 1.752059 | 4571.117 | 50.33594 |  |  |  |  |  |
| 21 | | 251.0477 | 1.754856 | 4570.734 | 50.50781 |  |  |  |  |  |
| 21.00833 | | 251.1399 | 1.760023 | 4570.328 | 50.85938 |  |  |  |  |  |
| 21.01667 | | 251.232 | 1.761472 | 4569.914 | 51.21094 |  |  |  |  |  |
| 21.025 | | 251.3218 | 1.764502 | 4569.484 | 51.64844 |  |  |  |  |  |
| 21.03333 | | 251.41 | 1.769918 | 4569.016 | 51.98438 |  |  |  |  |  |
| 21.04167 | | 251.4982 | 1.771243 | 4568.57 | 52.40625 |  |  |  |  |  |
| 21.05 | | 251.5912 | 1.774173 | 4568.133 | 52.78125 |  |  |  |  |  |
| 21.05833 | | 251.6817 | 1.779396 | 4567.672 | 53.10938 |  |  |  |  |  |
| 21.06667 | | 251.7739 | 1.780323 | 4567.203 | 53.125 |  |  |  |  |  |
| 21.075 | | 251.8661 | 1.7836 | 4566.719 | 53.39063 |  |  |  |  |  |
| 21.08333 | | 251.9607 | 1.788884 | 4566.25 | 53.71875 |  |  |  |  |  |
| 21.09167 | | 252.0537 | 1.790747 | 4565.766 | 53.78125 |  |  |  |  |  |
| 21.1 | | 252.1475 | 1.794885 | 4565.273 | 53.84375 |  |  |  |  |  |
| 21.10833 | | 252.2364 | 1.799948 | 4564.797 | 54 |  |  |  |  |  |
| 21.11667 | | 252.3326 | 1.801607 | 4564.359 | 54.25 |  |  |  |  |  |
| 21.125 | | 252.4256 | 1.805677 | 4563.883 | 54.46875 |  |  |  |  |  |
| 21.13333 | | 252.5154 | 1.810603 | 4563.406 | 54.61719 |  |  |  |  |  |
| 21.14167 | | 252.6052 | 1.812556 | 4562.945 | 54.89844 |  |  |  |  |  |
| 21.15 | | 252.6965 | 1.817174 | 4562.5 | 54.97656 |  |  |  |  |  |
| 21.15833 | | 252.7879 | 1.822439 | 4562.031 | 54.82031 |  |  |  |  |  |
| 21.16667 | | 252.8769 | 1.824361 | 4561.547 | 54.79688 |  |  |  |  |  |
| 21.175 | | 252.9683 | 1.828735 | 4561.07 | 54.82813 |  |  |  |  |  |
| 21.18333 | | 253.0573 | 1.833195 | 4560.602 | 54.96875 |  |  |  |  |  |
| 21.19167 | | 253.1494 | 1.835052 | 4560.117 | 54.88281 |  |  |  |  |  |
| 21.2 | | 253.2416 | 1.839501 | 4559.656 | 54.82031 |  |  |  |  |  |
| 21.20833 | | 253.3354 | 1.843916 | 4559.219 | 55.07031 |  |  |  |  |  |
| 21.21667 | | 253.4292 | 1.845978 | 4558.766 | 55.13281 |  |  |  |  |  |
| 21.225 | | 253.5238 | 1.850495 | 4558.313 | 55.29688 |  |  |  |  |  |
| 21.23333 | | 253.6143 | 1.854263 | 4557.836 | 55.75781 |  |  |  |  |  |
| 21.24167 | | 253.7081 | 1.856685 | 4557.391 | 56.35938 |  |  |  |  |  |
| 21.25 | | 253.8059 | 1.861803 | 4556.945 | 56.96094 |  |  |  |  |  |
| 21.25833 | | 253.9013 | 1.865589 | 4556.453 | 57.40625 |  |  |  |  |  |
| 21.26667 | | 253.9967 | 1.868788 | 4555.977 | 57.78906 |  |  |  |  |  |
| 21.275 | | 254.0905 | 1.873946 | 4555.492 | 58.45313 |  |  |  |  |  |
| 21.28333 | | 254.1787 | 1.877085 | 4554.977 | 59.10938 |  |  |  |  |  |
| 21.29167 | | 254.2724 | 1.88003 | 4554.422 | 59.38281 |  |  |  |  |  |
| 21.3 | | 254.3638 | 1.885112 | 4553.867 | 59.77344 |  |  |  |  |  |
| 21.30833 | | 254.4552 | 1.888706 | 4553.328 | 60.11719 |  |  |  |  |  |
| 21.31667 | | 254.5482 | 1.891668 | 4552.805 | 60.53125 |  |  |  |  |  |
| 21.325 | | 254.6396 | 1.896525 | 4552.227 | 60.65625 |  |  |  |  |  |
| 21.33333 | | 254.7318 | 1.899709 | 4551.648 | 60.75 |  |  |  |  |  |
| 21.34167 | | 254.8231 | 1.902793 | 4551.125 | 61.28125 |  |  |  |  |  |
| 21.35 | | 254.9129 | 1.90774 | 4550.586 | 61.71094 |  |  |  |  |  |
| 21.35833 | | 255.0059 | 1.911173 | 4550.047 | 61.91406 |  |  |  |  |  |
| 21.36667 | | 255.1029 | 1.915146 | 4549.5 | 62.14844 |  |  |  |  |  |
| 21.375 | | 255.1959 | 1.92103 | 4548.977 | 62.5 |  |  |  |  |  |
| 21.38333 | | 255.2881 | 1.924446 | 4548.461 | 62.88281 |  |  |  |  |  |
| 21.39167 | | 255.3786 | 1.928934 | 4547.891 | 63.17969 |  |  |  |  |  |
| 21.4 | | 255.4724 | 1.934663 | 4547.328 | 63.42188 |  |  |  |  |  |
| 21.40833 | | 255.5662 | 1.937814 | 4546.781 | 63.71875 |  |  |  |  |  |
| 21.41667 | | 255.6616 | 1.94267 | 4546.234 | 64.15625 |  |  |  |  |  |
| 21.425 | | 255.7546 | 1.948119 | 4545.664 | 64.54688 |  |  |  |  |  |
| 21.43333 | | 255.8508 | 1.950706 | 4545.094 | 64.75781 |  |  |  |  |  |
| 21.44167 | | 255.9445 | 1.95555 | 4544.531 | 64.86719 |  |  |  |  |  |
| 21.45 | | 256.0391 | 1.960669 | 4543.969 | 65.14063 |  |  |  |  |  |
| 21.45833 | | 256.1321 | 1.962566 | 4543.406 | 65.53125 |  |  |  |  |  |
| 21.46667 | | 256.2267 | 1.967864 | 4542.82 | 65.86719 |  |  |  |  |  |
| 21.475 | | 256.3229 | 1.972803 | 4542.234 | 66.4375 |  |  |  |  |  |
| 21.48333 | | 256.4167 | 1.974292 | 4541.68 | 67.04688 |  |  |  |  |  |
| 21.49167 | | 256.5083 | 1.980085 | 4541.125 | 67.69531 |  |  |  |  |  |
| 21.5 | | 256.599 | 1.984876 | 4540.555 | 68.30469 |  |  |  |  |  |
| 21.50833 | | 256.6889 | 1.987201 | 4539.969 | 68.74219 |  |  |  |  |  |
| 21.51667 | | 256.7788 | 1.993327 | 4539.367 | 69.76563 |  |  |  |  |  |
| 21.525 | | 256.871 | 1.998072 | 4538.742 | 70.57031 |  |  |  |  |  |
| 21.53333 | | 256.9601 | 2.000549 | 4538.117 | 71.17188 |  |  |  |  |  |
| 21.54167 | | 257.0508 | 2.007079 | 4537.461 | 71.8125 |  |  |  |  |  |
| 21.55 | | 257.1431 | 2.01191 | 4536.828 | 72.35938 |  |  |  |  |  |
| 21.55833 | | 257.2353 | 2.014107 | 4536.203 | 72.67969 |  |  |  |  |  |
| 21.56667 | | 257.3299 | 2.020718 | 4535.492 | 73.08594 |  |  |  |  |  |
| 21.575 | | 257.423 | 2.02502 | 4534.805 | 73.32031 |  |  |  |  |  |
| 21.58333 | | 257.5113 | 2.027529 | 4534.141 | 73.63281 |  |  |  |  |  |
| 21.59167 | | 257.6035 | 2.033555 | 4533.477 | 74.04688 |  |  |  |  |  |
| 21.6 | | 257.6974 | 2.037798 | 4532.805 | 74.28125 |  |  |  |  |  |
| 21.60833 | | 257.7872 | 2.041239 | 4532.18 | 74.5 |  |  |  |  |  |
| 21.61667 | | 257.8787 | 2.047692 | 4531.523 | 75.26563 |  |  |  |  |  |
| 21.625 | | 257.9694 | 2.052037 | 4530.891 | 75.64844 |  |  |  |  |  |
| 21.63333 | | 258.0601 | 2.055901 | 4530.242 | 75.96875 |  |  |  |  |  |
| 21.64167 | | 258.1507 | 2.062305 | 4529.578 | 76.5 |  |  |  |  |  |
| 21.65 | | 258.2383 | 2.066187 | 4528.938 | 76.8125 |  |  |  |  |  |
| 21.65833 | | 258.3281 | 2.07009 | 4528.281 | 77.17969 |  |  |  |  |  |
| 21.66667 | | 258.422 | 2.076094 | 4527.57 | 77.63281 |  |  |  |  |  |
| 21.675 | | 258.5166 | 2.080112 | 4526.891 | 78.00781 |  |  |  |  |  |
| 21.68333 | | 258.6096 | 2.085142 | 4526.219 | 78.29688 |  |  |  |  |  |
| 21.69167 | | 258.6979 | 2.091226 | 4525.516 | 78.70313 |  |  |  |  |  |
| 21.7 | | 258.7886 | 2.094891 | 4524.844 | 78.71875 |  |  |  |  |  |
| 21.70833 | | 258.8809 | 2.09957 | 4524.164 | 79.17188 |  |  |  |  |  |
| 21.71667 | | 258.9723 | 2.105312 | 4523.461 | 79.55469 |  |  |  |  |  |
| 21.725 | | 259.0645 | 2.109083 | 4522.773 | 79.9375 |  |  |  |  |  |
| 21.73333 | | 259.1568 | 2.11447 | 4522.094 | 80.44531 |  |  |  |  |  |
| 21.74167 | | 259.2514 | 2.120494 | 4521.398 | 81.11719 |  |  |  |  |  |
| 21.75 | | 259.3445 | 2.12461 | 4520.742 | 81.8125 |  |  |  |  |  |
| 21.75833 | | 259.4336 | 2.130416 | 4520.039 | 82.41406 |  |  |  |  |  |
| 21.76667 | | 259.525 | 2.136317 | 4519.336 | 82.70313 |  |  |  |  |  |
| 21.775 | | 259.622 | 2.140158 | 4518.617 | 83.21875 |  |  |  |  |  |
| 21.78333 | | 259.7159 | 2.146186 | 4517.883 | 83.9375 |  |  |  |  |  |
| 21.79167 | | 259.8089 | 2.151963 | 4517.125 | 84.46094 |  |  |  |  |  |
| 21.8 | | 259.9019 | 2.155602 | 4516.359 | 85.00781 |  |  |  |  |  |
| 21.80833 | | 259.9918 | 2.16145 | 4515.609 | 85.55469 |  |  |  |  |  |
| 21.81667 | | 260.084 | 2.166929 | 4514.883 | 86.11719 |  |  |  |  |  |
| 21.825 | | 260.174 | 2.170992 | 4514.141 | 86.35156 |  |  |  |  |  |
| 21.83333 | | 260.2622 | 2.177457 | 4513.352 | 86.6875 |  |  |  |  |  |
| 21.84167 | | 260.3561 | 2.182745 | 4512.586 | 87.07813 |  |  |  |  |  |
| 21.85 | | 260.4467 | 2.186385 | 4511.813 | 87.65625 |  |  |  |  |  |
| 21.85833 | | 260.539 | 2.193364 | 4511.039 | 88.27344 |  |  |  |  |  |
| 21.86667 | | 260.6305 | 2.198406 | 4510.258 | 88.53906 |  |  |  |  |  |
| 21.875 | | 260.7203 | 2.202184 | 4509.516 | 88.86719 |  |  |  |  |  |
| 21.88333 | | 260.8102 | 2.209372 | 4508.758 | 89.39063 |  |  |  |  |  |
| 21.89167 | | 260.9056 | 2.214356 | 4507.984 | 89.67188 |  |  |  |  |  |
| 21.9 | | 261.001 | 2.218615 | 4507.18 | 90 |  |  |  |  |  |
| 21.90833 | | 261.0941 | 2.226333 | 4506.375 | 90.51563 |  |  |  |  |  |
| 21.91667 | | 261.1871 | 2.230797 | 4505.602 | 90.67188 |  |  |  |  |  |
| 21.925 | | 261.277 | 2.234427 | 4504.828 | 90.91406 |  |  |  |  |  |
| 21.93333 | | 261.3693 | 2.241808 | 4504.016 | 91.0625 |  |  |  |  |  |
| 21.94167 | | 261.4615 | 2.245412 | 4503.234 | 91.21094 |  |  |  |  |  |
| 21.95 | | 261.5545 | 2.249779 | 4502.445 | 91.27344 |  |  |  |  |  |
| 21.95833 | | 261.6484 | 2.256918 | 4501.633 | 91.3125 |  |  |  |  |  |
| 21.96667 | | 261.7438 | 2.260779 | 4500.875 | 91.25781 |  |  |  |  |  |
| 21.975 | | 261.8368 | 2.266239 | 4500.102 | 91.28125 |  |  |  |  |  |
| 21.98333 | | 261.9251 | 2.272885 | 4499.328 | 91.34375 |  |  |  |  |  |
| 21.99167 | | 262.0166 | 2.277089 | 4498.555 | 91.49219 |  |  |  |  |  |
| 22 | | 262.1104 | 2.282642 | 4497.805 | 91.75 |  |  |  |  |  |
| 22.00833 | | 262.2058 | 2.290039 | 4497.047 | 92.17969 |  |  |  |  |  |
| 22.01667 | | 262.2989 | 2.295053 | 4496.305 | 92.74219 |  |  |  |  |  |
| 22.025 | | 262.3895 | 2.301246 | 4495.547 | 93.07031 |  |  |  |  |  |
| 22.03333 | | 262.4842 | 2.30775 | 4494.789 | 93.64063 |  |  |  |  |  |
| 22.04167 | | 262.5772 | 2.312593 | 4494.008 | 94.35938 |  |  |  |  |  |
| 22.05 | | 262.6694 | 2.319474 | 4493.219 | 95.03906 |  |  |  |  |  |
| 22.05833 | | 262.7609 | 2.325934 | 4492.406 | 95.77344 |  |  |  |  |  |
| 22.06667 | | 262.8547 | 2.330741 | 4491.563 | 96.52344 |  |  |  |  |  |
| 22.075 | | 262.9462 | 2.337094 | 4490.75 | 97.05469 |  |  |  |  |  |
| 22.08333 | | 263.04 | 2.343677 | 4489.891 | 97.67969 |  |  |  |  |  |
| 22.09167 | | 263.1331 | 2.348298 | 4489.016 | 98.29688 |  |  |  |  |  |
| 22.1 | | 263.2245 | 2.354863 | 4488.141 | 98.58594 |  |  |  |  |  |
| 22.10833 | | 263.3176 | 2.361101 | 4487.258 | 98.98438 |  |  |  |  |  |
| 22.11667 | | 263.4114 | 2.366026 | 4486.352 | 99.29688 |  |  |  |  |  |
| 22.125 | | 263.5037 | 2.373058 | 4485.484 | 99.88281 |  |  |  |  |  |
| 22.13333 | | 263.5927 | 2.379037 | 4484.586 | 100.3594 |  |  |  |  |  |
| 22.14167 | | 263.6858 | 2.384079 | 4483.688 | 100.9141 |  |  |  |  |  |
| 22.15 | | 263.782 | 2.391471 | 4482.836 | 101.5781 |  |  |  |  |  |
| 22.15833 | | 263.8774 | 2.396977 | 4481.961 | 102.4531 |  |  |  |  |  |
| 22.16667 | | 263.9696 | 2.401953 | 4481.102 | 103.5156 |  |  |  |  |  |
| 22.175 | | 264.0619 | 2.409641 | 4480.195 | 104.625 |  |  |  |  |  |
| 22.18333 | | 264.1565 | 2.414591 | 4479.297 | 105.6641 |  |  |  |  |  |
| 22.19167 | | 264.2503 | 2.420472 | 4478.375 | 106.8516 |  |  |  |  |  |
| 22.2 | | 264.3387 | 2.428781 | 4477.453 | 107.9922 |  |  |  |  |  |
| 22.20833 | | 264.4325 | 2.433292 | 4476.484 | 108.9922 |  |  |  |  |  |
| 22.21667 | | 264.5279 | 2.439067 | 4475.477 | 109.6797 |  |  |  |  |  |
| 22.225 | | 264.6193 | 2.447218 | 4474.461 | 110.3984 |  |  |  |  |  |
| 22.23333 | | 264.71 | 2.451495 | 4473.438 | 111.125 |  |  |  |  |  |
| 22.24167 | | 264.8038 | 2.457563 | 4472.398 | 111.8594 |  |  |  |  |  |
| 22.25 | | 264.8953 | 2.465311 | 4471.344 | 112.4141 |  |  |  |  |  |
| 22.25833 | | 264.9844 | 2.469223 | 4470.313 | 112.9063 |  |  |  |  |  |
| 22.26667 | | 265.0751 | 2.475531 | 4469.313 | 113.4219 |  |  |  |  |  |
| 22.275 | | 265.1663 | 2.483481 | 4468.313 | 113.9219 |  |  |  |  |  |
| 22.28333 | | 265.2588 | 2.487927 | 4467.297 | 114.5078 |  |  |  |  |  |
| 22.29167 | | 265.3505 | 2.494526 | 4466.273 | 114.9531 |  |  |  |  |  |
| 22.3 | | 265.4453 | 2.502154 | 4465.273 | 115.7734 |  |  |  |  |  |
| 22.30833 | | 265.5394 | 2.506374 | 4464.281 | 116.5234 |  |  |  |  |  |
| 22.31667 | | 265.6319 | 2.512838 | 4463.289 | 117.0391 |  |  |  |  |  |
| 22.325 | | 265.7212 | 2.520069 | 4462.289 | 117.8906 |  |  |  |  |  |
| 22.33333 | | 265.8153 | 2.52455 | 4461.273 | 118.3438 |  |  |  |  |  |
| 22.34167 | | 265.9101 | 2.532338 | 4460.273 | 118.8594 |  |  |  |  |  |
| 22.35 | | 266.0065 | 2.539549 | 4459.219 | 119.5 |  |  |  |  |  |
| 22.35833 | | 266.1037 | 2.544976 | 4458.172 | 120.1172 |  |  |  |  |  |
| 22.36667 | | 266.1978 | 2.552567 | 4457.141 | 120.4766 |  |  |  |  |  |
| 22.375 | | 266.2934 | 2.558719 | 4456.07 | 121.0078 |  |  |  |  |  |
| 22.38333 | | 266.3835 | 2.564808 | 4455.047 | 121.6172 |  |  |  |  |  |
| 22.39167 | | 266.4768 | 2.572818 | 4454.008 | 122.0938 |  |  |  |  |  |
| 22.4 | | 266.567 | 2.578991 | 4452.945 | 122.6406 |  |  |  |  |  |
| 22.40833 | | 266.6626 | 2.585488 | 4451.875 | 123 |  |  |  |  |  |
| 22.41667 | | 266.7535 | 2.594167 | 4450.844 | 123.9688 |  |  |  |  |  |
| 22.425 | | 266.846 | 2.599993 | 4449.781 | 124.9844 |  |  |  |  |  |
| 22.43333 | | 266.9361 | 2.606345 | 4448.703 | 125.7031 |  |  |  |  |  |
| 22.44167 | | 267.0255 | 2.614325 | 4447.641 | 126.5703 |  |  |  |  |  |
| 22.45 | | 267.118 | 2.619604 | 4446.547 | 127.4766 |  |  |  |  |  |
| 22.45833 | | 267.2073 | 2.625709 | 4445.492 | 128.7109 |  |  |  |  |  |
| 22.46667 | | 267.3022 | 2.63401 | 4444.328 | 129.5 |  |  |  |  |  |
| 22.475 | | 267.3923 | 2.639304 | 4443.172 | 130.4922 |  |  |  |  |  |
| 22.48333 | | 267.4879 | 2.646428 | 4442.031 | 131.2813 |  |  |  |  |  |
| 22.49167 | | 267.5789 | 2.654533 | 4440.883 | 132.2031 |  |  |  |  |  |
| 22.5 | | 267.6721 | 2.658828 | 4439.711 | 132.625 |  |  |  |  |  |
| 22.50833 | | 267.7646 | 2.666277 | 4438.484 | 133.0156 |  |  |  |  |  |
| 22.51667 | | 267.8571 | 2.67419 | 4437.32 | 133.7734 |  |  |  |  |  |
| 22.525 | | 267.9543 | 2.678588 | 4436.117 | 134.3984 |  |  |  |  |  |
| 22.53333 | | 268.0499 | 2.686716 | 4434.938 | 135.4922 |  |  |  |  |  |
| 22.54167 | | 268.144 | 2.694581 | 4433.727 | 135.9453 |  |  |  |  |  |
| 22.55 | | 268.2365 | 2.69943 | 4432.578 | 136.6094 |  |  |  |  |  |
| 22.55833 | | 268.3313 | 2.707749 | 4431.422 | 137.4453 |  |  |  |  |  |
| 22.56667 | | 268.4261 | 2.715441 | 4430.219 | 138.5625 |  |  |  |  |  |
| 22.575 | | 268.521 | 2.721119 | 4429.023 | 139.5 |  |  |  |  |  |
| 22.58333 | | 268.6166 | 2.729627 | 4427.758 | 140.4688 |  |  |  |  |  |
| 22.59167 | | 268.7154 | 2.736316 | 4426.563 | 141.2734 |  |  |  |  |  |
| 22.6 | | 268.8118 | 2.742735 | 4425.344 | 141.9453 |  |  |  |  |  |
| 22.60833 | | 268.9027 | 2.75092 | 4424.086 | 142.9219 |  |  |  |  |  |
| 22.61667 | | 268.9944 | 2.757122 | 4422.789 | 143.0625 |  |  |  |  |  |
| 22.625 | | 269.0892 | 2.763933 | 4421.516 | 143.9297 |  |  |  |  |  |
| 22.63333 | | 269.1833 | 2.772333 | 4420.227 | 144.6875 |  |  |  |  |  |
| 22.64167 | | 269.275 | 2.778059 | 4418.961 | 145.2969 |  |  |  |  |  |
| 22.65 | | 269.3659 | 2.78517 | 4417.703 | 145.7656 |  |  |  |  |  |
| 22.65833 | | 269.4592 | 2.793131 | 4416.398 | 146.3281 |  |  |  |  |  |
| 22.66667 | | 269.5525 | 2.798546 | 4415.203 | 147.0313 |  |  |  |  |  |
| 22.675 | | 269.6418 | 2.806193 | 4413.906 | 147.8281 |  |  |  |  |  |
| 22.68333 | | 269.732 | 2.814136 | 4412.617 | 148.75 |  |  |  |  |  |
| 22.69167 | | 269.8229 | 2.819522 | 4411.344 | 149.6953 |  |  |  |  |  |
| 22.7 | | 269.9154 | 2.827013 | 4410.086 | 150.7188 |  |  |  |  |  |
| 22.70833 | | 270.0078 | 2.835077 | 4408.797 | 151.5 |  |  |  |  |  |
| 22.71667 | | 270.1027 | 2.840283 | 4407.5 | 152.5859 |  |  |  |  |  |
| 22.725 | | 270.1991 | 2.848508 | 4406.164 | 153.7656 |  |  |  |  |  |
| 22.73333 | | 270.2932 | 2.856403 | 4404.828 | 154.7109 |  |  |  |  |  |
| 22.74167 | | 270.388 | 2.861833 | 4403.461 | 155.8047 |  |  |  |  |  |
| 22.75 | | 270.4813 | 2.870559 | 4402.086 | 156.9141 |  |  |  |  |  |
| 22.75833 | | 270.5753 | 2.87852 | 4400.742 | 157.9141 |  |  |  |  |  |
| 22.76667 | | 270.6694 | 2.883614 | 4399.336 | 158.9453 |  |  |  |  |  |
| 22.775 | | 270.7627 | 2.892957 | 4397.922 | 159.6875 |  |  |  |  |  |
| 22.78333 | | 270.8583 | 2.900998 | 4396.523 | 160.7422 |  |  |  |  |  |
| 22.79167 | | 270.9516 | 2.906028 | 4395.102 | 161.9141 |  |  |  |  |  |
| 22.8 | | 271.044 | 2.915551 | 4393.664 | 162.9844 |  |  |  |  |  |
| 22.80833 | | 271.1342 | 2.922556 | 4392.234 | 163.8047 |  |  |  |  |  |
| 22.81667 | | 271.2274 | 2.928162 | 4390.773 | 164.8516 |  |  |  |  |  |
| 22.825 | | 271.3192 | 2.937076 | 4389.359 | 165.9609 |  |  |  |  |  |
| 22.83333 | | 271.4093 | 2.943809 | 4387.891 | 166.9141 |  |  |  |  |  |
| 22.84167 | | 271.5018 | 2.94976 | 4386.406 | 167.7969 |  |  |  |  |  |
| 22.85 | | 271.5943 | 2.958786 | 4384.906 | 168.7422 |  |  |  |  |  |
| 22.85833 | | 271.6876 | 2.965685 | 4383.453 | 169.8281 |  |  |  |  |  |
| 22.86667 | | 271.78 | 2.972075 | 4381.953 | 170.8906 |  |  |  |  |  |
| 22.875 | | 271.8717 | 2.981256 | 4380.43 | 171.7656 |  |  |  |  |  |
| 22.88333 | | 271.9642 | 2.988078 | 4378.93 | 172.3672 |  |  |  |  |  |
| 22.89167 | | 272.0583 | 2.995032 | 4377.43 | 173.3828 |  |  |  |  |  |
| 22.9 | | 272.1516 | 3.004233 | 4375.922 | 174.4609 |  |  |  |  |  |
| 22.90833 | | 272.2464 | 3.01082 | 4374.383 | 175 |  |  |  |  |  |
| 22.91667 | | 272.342 | 3.017924 | 4372.836 | 175.5156 |  |  |  |  |  |
| 22.925 | | 272.4369 | 3.026906 | 4371.305 | 176.0938 |  |  |  |  |  |
| 22.93333 | | 272.5333 | 3.033532 | 4369.805 | 176.9453 |  |  |  |  |  |
| 22.94167 | | 272.6297 | 3.041725 | 4368.242 | 177.3828 |  |  |  |  |  |
| 22.95 | | 272.7214 | 3.050337 | 4366.672 | 177.9844 |  |  |  |  |  |
| 22.95833 | | 272.8155 | 3.056368 | 4365.156 | 178.6172 |  |  |  |  |  |
| 22.96667 | | 272.9087 | 3.064766 | 4363.641 | 179.4063 |  |  |  |  |  |
| 22.975 | | 273.0036 | 3.072539 | 4362.109 | 180.1875 |  |  |  |  |  |
| 22.98333 | | 273.0984 | 3.078641 | 4360.539 | 180.6797 |  |  |  |  |  |
| 22.99167 | | 273.1917 | 3.087926 | 4359.023 | 181.6172 |  |  |  |  |  |
| 23 | | 273.2865 | 3.095641 | 4357.484 | 182.6563 |  |  |  |  |  |
| 23.00833 | | 273.3798 | 3.101629 | 4355.93 | 184.0859 |  |  |  |  |  |
| 23.01667 | | 273.4707 | 3.110939 | 4354.352 | 185.1563 |  |  |  |  |  |
| 23.025 | | 273.5624 | 3.118066 | 4352.773 | 186.1797 |  |  |  |  |  |
| 23.03333 | | 273.6557 | 3.123701 | 4351.219 | 187.4219 |  |  |  |  |  |
| 23.04167 | | 273.7495 | 3.13428 | 4349.602 | 188.5547 |  |  |  |  |  |
| 23.05 | | 273.8436 | 3.141346 | 4347.953 | 189.8438 |  |  |  |  |  |
| 23.05833 | | 273.9345 | 3.1481 | 4346.25 | 190.8984 |  |  |  |  |  |
| 23.06667 | | 274.0262 | 3.158135 | 4344.586 | 192.1875 |  |  |  |  |  |
| 23.075 | | 274.1202 | 3.164943 | 4342.906 | 193.3281 |  |  |  |  |  |
| 23.08333 | | 274.2135 | 3.172338 | 4341.203 | 194.4688 |  |  |  |  |  |
| 23.09167 | | 274.3075 | 3.181795 | 4339.492 | 195.5313 |  |  |  |  |  |
| 23.1 | | 274.4031 | 3.188412 | 4337.75 | 196.6641 |  |  |  |  |  |
| 23.10833 | | 274.4948 | 3.196522 | 4336.039 | 197.7578 |  |  |  |  |  |
| 23.11667 | | 274.5904 | 3.20577 | 4334.281 | 198.9453 |  |  |  |  |  |
| 23.125 | | 274.6828 | 3.212267 | 4332.539 | 200.125 |  |  |  |  |  |
| 23.13333 | | 274.773 | 3.220602 | 4330.773 | 201.1094 |  |  |  |  |  |
| 23.14167 | | 274.8686 | 3.229144 | 4329.016 | 202.1406 |  |  |  |  |  |
| 23.15 | | 274.9603 | 3.235738 | 4327.242 | 203.2422 |  |  |  |  |  |
| 23.15833 | | 275.0497 | 3.24492 | 4325.469 | 204.7109 |  |  |  |  |  |
| 23.16667 | | 275.1406 | 3.253622 | 4323.656 | 205.875 |  |  |  |  |  |
| 23.175 | | 275.2324 | 3.260336 | 4321.852 | 206.9219 |  |  |  |  |  |
| 23.18333 | | 275.3217 | 3.269477 | 4320.07 | 207.9063 |  |  |  |  |  |
| 23.19167 | | 275.4158 | 3.277479 | 4318.258 | 209.2891 |  |  |  |  |  |
| 23.2 | | 275.509 | 3.284037 | 4316.422 | 210.5547 |  |  |  |  |  |
| 23.20833 | | 275.6023 | 3.293635 | 4314.539 | 211.6719 |  |  |  |  |  |
| 23.21667 | | 275.6955 | 3.300307 | 4312.688 | 213.0859 |  |  |  |  |  |
| 23.225 | | 275.788 | 3.307037 | 4310.844 | 214.3203 |  |  |  |  |  |
| 23.23333 | | 275.8828 | 3.317237 | 4309 | 215.6563 |  |  |  |  |  |
| 23.24167 | | 275.9776 | 3.323555 | 4307.078 | 216.5625 |  |  |  |  |  |
| 23.25 | | 276.0693 | 3.330505 | 4305.18 | 217.875 |  |  |  |  |  |
| 23.25833 | | 276.1633 | 3.341037 | 4303.273 | 218.8984 |  |  |  |  |  |
| 23.26667 | | 276.2581 | 3.347504 | 4301.328 | 220.1641 |  |  |  |  |  |
| 23.275 | | 276.3499 | 3.355236 | 4299.391 | 221.2578 |  |  |  |  |  |
| 23.28333 | | 276.4408 | 3.36537 | 4297.453 | 222.2422 |  |  |  |  |  |
| 23.29167 | | 276.5333 | 3.371725 | 4295.539 | 223.3516 |  |  |  |  |  |
| 23.3 | | 276.6265 | 3.380265 | 4293.57 | 224.375 |  |  |  |  |  |
| 23.30833 | | 276.7198 | 3.390558 | 4291.641 | 225.6719 |  |  |  |  |  |
| 23.31667 | | 276.8123 | 3.396999 | 4289.656 | 227.0469 |  |  |  |  |  |
| 23.325 | | 276.9024 | 3.404725 | 4287.703 | 228.3984 |  |  |  |  |  |
| 23.33333 | | 276.9965 | 3.413841 | 4285.742 | 229.75 |  |  |  |  |  |
| 23.34167 | | 277.0866 | 3.420089 | 4283.766 | 231.4219 |  |  |  |  |  |
| 23.35 | | 277.1814 | 3.428186 | 4281.789 | 232.7656 |  |  |  |  |  |
| 23.35833 | | 277.2762 | 3.437247 | 4279.758 | 234.3281 |  |  |  |  |  |
| 23.36667 | | 277.368 | 3.443587 | 4277.711 | 235.7891 |  |  |  |  |  |
| 23.375 | | 277.462 | 3.452425 | 4275.656 | 237.3984 |  |  |  |  |  |
| 23.38333 | | 277.5544 | 3.460845 | 4273.586 | 239.3672 |  |  |  |  |  |
| 23.39167 | | 277.6454 | 3.467575 | 4271.461 | 240.8672 |  |  |  |  |  |
| 23.4 | | 277.7379 | 3.476731 | 4269.367 | 242.3125 |  |  |  |  |  |
| 23.40833 | | 277.8334 | 3.48483 | 4267.219 | 243.7969 |  |  |  |  |  |
| 23.41667 | | 277.9298 | 3.492183 | 4265.086 | 245.2656 |  |  |  |  |  |
| 23.425 | | 278.0246 | 3.501918 | 4262.914 | 246.6875 |  |  |  |  |  |
| 23.43333 | | 278.1147 | 3.508288 | 4260.672 | 248.0781 |  |  |  |  |  |
| 23.44167 | | 278.2057 | 3.515963 | 4258.492 | 249.5781 |  |  |  |  |  |
| 23.45 | | 278.2989 | 3.526237 | 4256.297 | 250.9531 |  |  |  |  |  |
| 23.45833 | | 278.3899 | 3.532469 | 4254.094 | 252.5 |  |  |  |  |  |
| 23.46667 | | 278.4831 | 3.540469 | 4251.875 | 253.5859 |  |  |  |  |  |
| 23.475 | | 278.5748 | 3.550234 | 4249.648 | 254.9688 |  |  |  |  |  |
| 23.48333 | | 278.6658 | 3.556029 | 4247.422 | 256.2969 |  |  |  |  |  |
| 23.49167 | | 278.7567 | 3.564195 | 4245.172 | 257.8359 |  |  |  |  |  |
| 23.5 | | 278.8477 | 3.574262 | 4242.922 | 259.4453 |  |  |  |  |  |
| 23.50833 | | 278.9371 | 3.580168 | 4240.633 | 260.7813 |  |  |  |  |  |
| 23.51667 | | 279.028 | 3.58903 | 4238.406 | 262.3125 |  |  |  |  |  |
| 23.525 | | 279.1228 | 3.598677 | 4236.125 | 263.9453 |  |  |  |  |  |
| 23.53333 | | 279.2168 | 3.604955 | 4233.844 | 265.7656 |  |  |  |  |  |
| 23.54167 | | 279.3085 | 3.613708 | 4231.508 | 267.1875 |  |  |  |  |  |
| 23.55 | | 279.4002 | 3.622699 | 4229.172 | 268.7344 |  |  |  |  |  |
| 23.55833 | | 279.4951 | 3.629127 | 4226.836 | 270.0859 |  |  |  |  |  |
| 23.56667 | | 279.5898 | 3.638235 | 4224.469 | 271.8359 |  |  |  |  |  |
| 23.575 | | 279.6823 | 3.646868 | 4222.078 | 273.3125 |  |  |  |  |  |
| 23.58333 | | 279.7733 | 3.653733 | 4219.648 | 274.7656 |  |  |  |  |  |
| 23.59167 | | 279.8673 | 3.663219 | 4217.258 | 276.2344 |  |  |  |  |  |
| 23.6 | | 279.9629 | 3.67161 | 4214.844 | 278.1406 |  |  |  |  |  |
| 23.60833 | | 280.0569 | 3.679028 | 4212.438 | 279.9766 |  |  |  |  |  |
| 23.61667 | | 280.1478 | 3.688477 | 4209.961 | 281.875 |  |  |  |  |  |
| 23.625 | | 280.2426 | 3.696163 | 4207.516 | 283.6094 |  |  |  |  |  |
| 23.63333 | | 280.3374 | 3.704144 | 4205.063 | 285.5547 |  |  |  |  |  |
| 23.64167 | | 280.4291 | 3.714051 | 4202.594 | 287.625 |  |  |  |  |  |
| 23.65 | | 280.5201 | 3.72105 | 4200.055 | 289.3125 |  |  |  |  |  |
| 23.65833 | | 280.6149 | 3.729427 | 4197.508 | 290.8047 |  |  |  |  |  |
| 23.66667 | | 280.7081 | 3.739027 | 4194.922 | 292.3203 |  |  |  |  |  |
| 23.675 | | 280.8029 | 3.745497 | 4192.359 | 294.1563 |  |  |  |  |  |
| 23.68333 | | 280.8954 | 3.754517 | 4189.758 | 295.6563 |  |  |  |  |  |
| 23.69167 | | 280.9856 | 3.763987 | 4187.109 | 296.9375 |  |  |  |  |  |
| 23.7 | | 281.0796 | 3.770163 | 4184.516 | 298.0313 |  |  |  |  |  |
| 23.70833 | | 281.1736 | 3.779278 | 4181.938 | 299.625 |  |  |  |  |  |
| 23.71667 | | 281.2661 | 3.789137 | 4179.32 | 301.2422 |  |  |  |  |  |
| 23.725 | | 281.3578 | 3.795226 | 4176.656 | 302.6797 |  |  |  |  |  |
| 23.73333 | | 281.4518 | 3.804951 | 4174.031 | 304.2188 |  |  |  |  |  |
| 23.74167 | | 281.5451 | 3.814613 | 4171.414 | 305.9297 |  |  |  |  |  |
| 23.75 | | 281.6391 | 3.821178 | 4168.828 | 307.9375 |  |  |  |  |  |
| 23.75833 | | 281.7331 | 3.831146 | 4166.141 | 309.9141 |  |  |  |  |  |
| 23.76667 | | 281.8241 | 3.84 | 4163.453 | 311.7969 |  |  |  |  |  |
| 23.775 | | 281.9181 | 3.846731 | 4160.758 | 313.5859 |  |  |  |  |  |
| 23.78333 | | 282.0114 | 3.856759 | 4158.039 | 315.6797 |  |  |  |  |  |
| 23.79167 | | 282.1046 | 3.865531 | 4155.297 | 317.6094 |  |  |  |  |  |
| 23.8 | | 282.1967 | 3.872854 | 4152.484 | 319.3906 |  |  |  |  |  |
| 23.80833 | | 282.2922 | 3.883374 | 4149.672 | 321.1797 |  |  |  |  |  |
| 23.81667 | | 282.3869 | 3.892119 | 4146.852 | 323.2266 |  |  |  |  |  |
| 23.825 | | 282.4809 | 3.90028 | 4144.031 | 325.5547 |  |  |  |  |  |
| 23.83333 | | 282.5733 | 3.910321 | 4141.148 | 327.5234 |  |  |  |  |  |
| 23.84167 | | 282.6657 | 3.918176 | 4138.273 | 329.2891 |  |  |  |  |  |
| 23.85 | | 282.7643 | 3.926555 | 4135.406 | 331.1563 |  |  |  |  |  |
| 23.85833 | | 282.8599 | 3.936514 | 4132.523 | 333.3828 |  |  |  |  |  |
| 23.86667 | | 282.9561 | 3.943966 | 4129.594 | 335.3047 |  |  |  |  |  |
| 23.875 | | 283.0493 | 3.953158 | 4126.594 | 337.3828 |  |  |  |  |  |
| 23.88333 | | 283.1456 | 3.96266 | 4123.648 | 339.3359 |  |  |  |  |  |
| 23.89167 | | 283.2419 | 3.968899 | 4120.703 | 341.4063 |  |  |  |  |  |
| 23.9 | | 283.3367 | 3.979231 | 4117.727 | 343.6953 |  |  |  |  |  |
| 23.90833 | | 283.4306 | 3.98858 | 4114.68 | 345.6016 |  |  |  |  |  |
| 23.91667 | | 283.5262 | 3.995121 | 4111.664 | 347.5781 |  |  |  |  |  |
| 23.925 | | 283.6201 | 4.005844 | 4108.609 | 349.7188 |  |  |  |  |  |
| 23.93333 | | 283.7133 | 4.01498 | 4105.555 | 352.0625 |  |  |  |  |  |
| 23.94167 | | 283.805 | 4.02208 | 4102.461 | 354.3828 |  |  |  |  |  |
| 23.95 | | 283.8974 | 4.032797 | 4099.336 | 356.5078 |  |  |  |  |  |
| 23.95833 | | 283.9929 | 4.041875 | 4096.242 | 358.3984 |  |  |  |  |  |
| 23.96667 | | 284.0876 | 4.049833 | 4093.109 | 360.3984 |  |  |  |  |  |
| 23.975 | | 284.1801 | 4.060719 | 4089.945 | 362.6719 |  |  |  |  |  |
| 23.98333 | | 284.2748 | 4.068789 | 4086.727 | 364.6953 |  |  |  |  |  |
| 23.99167 | | 284.3672 | 4.076243 | 4083.508 | 366.5859 |  |  |  |  |  |
| 24 | | 284.4619 | 4.086447 | 4080.289 | 368.1797 |  |  |  |  |  |
| 24.00833 | | 284.559 | 4.094694 | 4077.086 | 370.3203 |  |  |  |  |  |
| 24.01667 | | 284.653 | 4.103779 | 4073.844 | 372.3438 |  |  |  |  |  |
| 24.025 | | 284.7477 | 4.114106 | 4070.563 | 374.1328 |  |  |  |  |  |
| 24.03333 | | 284.8417 | 4.122055 | 4067.289 | 376.0547 |  |  |  |  |  |
| 24.04167 | | 284.9349 | 4.131623 | 4064.016 | 378.2813 |  |  |  |  |  |
| 24.05 | | 285.0304 | 4.141675 | 4060.766 | 380.8203 |  |  |  |  |  |
| 24.05833 | | 285.1267 | 4.149463 | 4057.438 | 382.9688 |  |  |  |  |  |
| 24.06667 | | 285.2207 | 4.160117 | 4054.117 | 385.1719 |  |  |  |  |  |
| 24.075 | | 285.3193 | 4.170533 | 4050.789 | 387.6875 |  |  |  |  |  |
| 24.08333 | | 285.4171 | 4.178701 | 4047.453 | 390.7422 |  |  |  |  |  |
| 24.09167 | | 285.5096 | 4.190499 | 4044.039 | 393.3281 |  |  |  |  |  |
| 24.1 | | 285.6035 | 4.200416 | 4040.578 | 395.8672 |  |  |  |  |  |
| 24.10833 | | 285.7021 | 4.208185 | 4037.141 | 398.3047 |  |  |  |  |  |
| 24.11667 | | 285.8008 | 4.220465 | 4033.68 | 401.0234 |  |  |  |  |  |
| 24.125 | | 285.8963 | 4.229535 | 4030.156 | 403.8672 |  |  |  |  |  |
| 24.13333 | | 285.991 | 4.237603 | 4026.531 | 406.5703 |  |  |  |  |  |
| 24.14167 | | 286.085 | 4.248275 | 4022.945 | 409.2891 |  |  |  |  |  |
| 24.15 | | 286.1813 | 4.25652 | 4019.344 | 412.1094 |  |  |  |  |  |
| 24.15833 | | 286.2761 | 4.265611 | 4015.734 | 415.0156 |  |  |  |  |  |
| 24.16667 | | 286.3669 | 4.276469 | 4012.07 | 417.2422 |  |  |  |  |  |
| 24.175 | | 286.4648 | 4.284656 | 4008.367 | 419.5469 |  |  |  |  |  |
| 24.18333 | | 286.5626 | 4.294185 | 4004.656 | 421.9922 |  |  |  |  |  |
| 24.19167 | | 286.6581 | 4.304902 | 4000.922 | 424.6016 |  |  |  |  |  |
| 24.2 | | 286.7513 | 4.313565 | 3997.156 | 427.2734 |  |  |  |  |  |
| 24.20833 | | 286.8492 | 4.324465 | 3993.359 | 429.4063 |  |  |  |  |  |
| 24.21667 | | 286.9455 | 4.335212 | 3989.625 | 431.7734 |  |  |  |  |  |
| 24.225 | | 287.0425 | 4.34428 | 3985.859 | 433.9531 |  |  |  |  |  |
| 24.23333 | | 287.1373 | 4.356625 | 3982.063 | 436.6094 |  |  |  |  |  |
| 24.24167 | | 287.2297 | 4.367151 | 3978.227 | 439.0859 |  |  |  |  |  |
| 24.25 | | 287.326 | 4.375995 | 3974.359 | 441.6016 |  |  |  |  |  |
| 24.25833 | | 287.4192 | 4.388818 | 3970.539 | 444.2813 |  |  |  |  |  |
| 24.26667 | | 287.5132 | 4.398244 | 3966.68 | 447.3438 |  |  |  |  |  |
| 24.275 | | 287.6079 | 4.406728 | 3962.813 | 450.1563 |  |  |  |  |  |
| 24.28333 | | 287.7018 | 4.419221 | 3958.867 | 452.7266 |  |  |  |  |  |
| 24.29167 | | 287.7958 | 4.428596 | 3954.93 | 455.2969 |  |  |  |  |  |
| 24.3 | | 287.8906 | 4.43909 | 3950.953 | 457.9609 |  |  |  |  |  |
| 24.30833 | | 287.9861 | 4.451887 | 3946.945 | 461.2266 |  |  |  |  |  |
| 24.31667 | | 288.0824 | 4.461467 | 3942.844 | 463.8594 |  |  |  |  |  |
| 24.325 | | 288.1794 | 4.472106 | 3938.773 | 466.2266 |  |  |  |  |  |
| 24.33333 | | 288.2765 | 4.48402 | 3934.695 | 468.6719 |  |  |  |  |  |
| 24.34167 | | 288.3728 | 4.4939 | 3930.609 | 471.8594 |  |  |  |  |  |
| 24.35 | | 288.4707 | 4.506197 | 3926.477 | 474.1875 |  |  |  |  |  |
| 24.35833 | | 288.5646 | 4.5177 | 3922.25 | 476.9219 |  |  |  |  |  |
| 24.36667 | | 288.6625 | 4.527785 | 3918.094 | 479.6563 |  |  |  |  |  |
| 24.375 | | 288.7596 | 4.541018 | 3913.945 | 482.6016 |  |  |  |  |  |
| 24.38333 | | 288.8543 | 4.551968 | 3909.758 | 485.7813 |  |  |  |  |  |
| 24.39167 | | 288.9498 | 4.562241 | 3905.453 | 488.8281 |  |  |  |  |  |
| 24.4 | | 289.0461 | 4.576281 | 3901.258 | 491.7813 |  |  |  |  |  |
| 24.40833 | | 289.1424 | 4.586906 | 3896.977 | 494.7031 |  |  |  |  |  |
| 24.41667 | | 289.2379 | 4.598007 | 3892.664 | 498.0938 |  |  |  |  |  |
| 24.425 | | 289.335 | 4.612393 | 3888.305 | 500.8672 |  |  |  |  |  |
| 24.43333 | | 289.4282 | 4.622672 | 3883.898 | 504.2891 |  |  |  |  |  |
| 24.44167 | | 289.5261 | 4.634373 | 3879.461 | 507.2422 |  |  |  |  |  |
| 24.45 | | 289.6223 | 4.648307 | 3875.023 | 510.2734 |  |  |  |  |  |
| 24.45833 | | 289.7187 | 4.658817 | 3870.57 | 513.6484 |  |  |  |  |  |
| 24.46667 | | 289.8157 | 4.671073 | 3866.016 | 516.5 |  |  |  |  |  |
| 24.475 | | 289.9097 | 4.684049 | 3861.523 | 519.1172 |  |  |  |  |  |
| 24.48333 | | 290.0052 | 4.694512 | 3856.906 | 521.6641 |  |  |  |  |  |
| 24.49167 | | 290.1007 | 4.707172 | 3852.344 | 525.4453 |  |  |  |  |  |
| 24.5 | | 290.1978 | 4.720012 | 3847.727 | 528.4219 |  |  |  |  |  |
| 24.50833 | | 290.2933 | 4.731066 | 3843.055 | 530.9531 |  |  |  |  |  |
| 24.51667 | | 290.3904 | 4.745048 | 3838.414 | 533.5 |  |  |  |  |  |
| 24.525 | | 290.4883 | 4.757569 | 3833.805 | 536.6875 |  |  |  |  |  |
| 24.53333 | | 290.583 | 4.769467 | 3829.164 | 539.8672 |  |  |  |  |  |
| 24.54167 | | 290.6754 | 4.784131 | 3824.328 | 542.4922 |  |  |  |  |  |
| 24.55 | | 290.7698 | 4.795921 | 3819.578 | 545.4297 |  |  |  |  |  |
| 24.55833 | | 290.8696 | 4.808141 | 3814.867 | 548.3828 |  |  |  |  |  |
| 24.56667 | | 290.9656 | 4.8243 | 3810.148 | 552.1016 |  |  |  |  |  |
| 24.575 | | 291.0608 | 4.835684 | 3805.297 | 554.9141 |  |  |  |  |  |
| 24.58333 | | 291.1544 | 4.848961 | 3800.438 | 557.8672 |  |  |  |  |  |
| 24.59167 | | 291.2489 | 4.864943 | 3795.617 | 561.1484 |  |  |  |  |  |
| 24.6 | | 291.3464 | 4.87694 | 3790.742 | 564.7969 |  |  |  |  |  |
| 24.60833 | | 291.44 | 4.890868 | 3785.836 | 568.1328 |  |  |  |  |  |
| 24.61667 | | 291.5368 | 4.906121 | 3780.805 | 571.0547 |  |  |  |  |  |
| 24.625 | | 291.6343 | 4.918133 | 3775.867 | 574.4375 |  |  |  |  |  |
| 24.63333 | | 291.7326 | 4.933264 | 3770.875 | 577.9844 |  |  |  |  |  |
| 24.64167 | | 291.8285 | 4.947885 | 3765.828 | 581.9297 |  |  |  |  |  |
| 24.65 | | 291.9245 | 4.960882 | 3760.688 | 585.1563 |  |  |  |  |  |
| 24.65833 | | 292.0235 | 4.976968 | 3755.578 | 588.4063 |  |  |  |  |  |
| 24.66667 | | 292.1218 | 4.99061 | 3750.469 | 591.4219 |  |  |  |  |  |
| 24.675 | | 292.2209 | 5.005163 | 3745.297 | 595.5313 |  |  |  |  |  |
| 24.68333 | | 292.3168 | 5.022083 | 3740.047 | 598.8906 |  |  |  |  |  |
| 24.69167 | | 292.4128 | 5.035222 | 3734.734 | 601.7891 |  |  |  |  |  |
| 24.7 | | 292.5072 | 5.050635 | 3729.5 | 605.1406 |  |  |  |  |  |
| 24.70833 | | 292.6063 | 5.068671 | 3724.211 | 608.5703 |  |  |  |  |  |
| 24.71667 | | 292.7007 | 5.082328 | 3718.945 | 611.8516 |  |  |  |  |  |
| 24.725 | | 292.7951 | 5.099086 | 3713.492 | 614.5938 |  |  |  |  |  |
| 24.73333 | | 292.8926 | 5.115988 | 3708.125 | 617.4609 |  |  |  |  |  |
| 24.74167 | | 292.9909 | 5.130515 | 3702.773 | 620.3203 |  |  |  |  |  |
| 24.75 | | 293.0854 | 5.148115 | 3697.367 | 624.1563 |  |  |  |  |  |
| 24.75833 | | 293.1813 | 5.164228 | 3691.891 | 626.9063 |  |  |  |  |  |
| 24.76667 | | 293.2781 | 5.179 | 3686.43 | 629.9141 |  |  |  |  |  |
| 24.775 | | 293.3748 | 5.196342 | 3681.008 | 632.7969 |  |  |  |  |  |
| 24.78333 | | 293.4738 | 5.211944 | 3675.547 | 635.75 |  |  |  |  |  |
| 24.79167 | | 293.5698 | 5.228009 | 3670.055 | 638.7266 |  |  |  |  |  |
| 24.8 | | 293.6673 | 5.246507 | 3664.406 | 641.3203 |  |  |  |  |  |
| 24.80833 | | 293.7656 | 5.260863 | 3658.875 | 643.8359 |  |  |  |  |  |
| 24.81667 | | 293.8631 | 5.278445 | 3653.281 | 646.3438 |  |  |  |  |  |
| 24.825 | | 293.9591 | 5.296775 | 3647.695 | 649.5234 |  |  |  |  |  |
| 24.83333 | | 294.0573 | 5.311616 | 3642.063 | 651.5547 |  |  |  |  |  |
| 24.84167 | | 294.1541 | 5.330038 | 3636.414 | 653.7266 |  |  |  |  |  |
| 24.85 | | 294.2485 | 5.347114 | 3630.773 | 655.9219 |  |  |  |  |  |
| 24.85833 | | 294.3483 | 5.363836 | 3625.148 | 658.6875 |  |  |  |  |  |
| 24.86667 | | 294.4466 | 5.383086 | 3619.477 | 661.3594 |  |  |  |  |  |
| 24.875 | | 294.5449 | 5.399431 | 3613.719 | 663.5 |  |  |  |  |  |
| 24.88333 | | 294.6401 | 5.416909 | 3608.094 | 665.7188 |  |  |  |  |  |
| 24.89167 | | 294.7368 | 5.436195 | 3602.414 | 668.25 |  |  |  |  |  |
| 24.9 | | 294.8336 | 5.452226 | 3596.711 | 671.0781 |  |  |  |  |  |
| 24.90833 | | 294.9272 | 5.47072 | 3590.93 | 673.1953 |  |  |  |  |  |
| 24.91667 | | 295.0232 | 5.489885 | 3585.141 | 675.3281 |  |  |  |  |  |
| 24.925 | | 295.1207 | 5.505102 | 3579.391 | 677.375 |  |  |  |  |  |
| 24.93333 | | 295.222 | 5.52502 | 3573.602 | 679.5938 |  |  |  |  |  |
| 24.94167 | | 295.3195 | 5.544053 | 3567.766 | 682.0156 |  |  |  |  |  |
| 24.95 | | 295.4171 | 5.559908 | 3561.859 | 683.8203 |  |  |  |  |  |
| 24.95833 | | 295.513 | 5.57945 | 3556.031 | 685.5547 |  |  |  |  |  |
| 24.96667 | | 295.6151 | 5.597492 | 3550.172 | 687.2891 |  |  |  |  |  |
| 24.975 | | 295.7149 | 5.615368 | 3544.32 | 689.4922 |  |  |  |  |  |
| 24.98333 | | 295.8124 | 5.635508 | 3538.414 | 690.7031 |  |  |  |  |  |
| 24.99167 | | 295.9115 | 5.653264 | 3532.469 | 691.8672 |  |  |  |  |  |
| 25 | | 296.0121 | 5.672623 | 3526.586 | 693.0234 |  |  |  |  |  |
| 25.00833 | | 296.1119 | 5.693492 | 3520.695 | 694.7969 |  |  |  |  |  |
| 25.01667 | | 296.2094 | 5.709771 | 3514.797 | 695.9922 |  |  |  |  |  |
| 25.025 | | 296.3076 | 5.730494 | 3508.813 | 696.7109 |  |  |  |  |  |
| 25.03333 | | 296.4067 | 5.750185 | 3502.945 | 697.7188 |  |  |  |  |  |
| 25.04167 | | 296.5103 | 5.767512 | 3497.063 | 698.9141 |  |  |  |  |  |
| 25.05 | | 296.6071 | 5.788094 | 3491.195 | 700.2969 |  |  |  |  |  |
| 25.05833 | | 296.7046 | 5.805977 | 3485.203 | 700.7188 |  |  |  |  |  |
| 25.06667 | | 296.8021 | 5.823494 | 3479.305 | 701.3125 |  |  |  |  |  |
| 25.075 | | 296.9019 | 5.843465 | 3473.438 | 701.9297 |  |  |  |  |  |
| 25.08333 | | 297.0017 | 5.860496 | 3467.547 | 703.3828 |  |  |  |  |  |
| 25.09167 | | 297.1023 | 5.880072 | 3461.594 | 703.8047 |  |  |  |  |  |
| 25.1 | | 297.2006 | 5.899725 | 3455.617 | 703.9922 |  |  |  |  |  |
| 25.10833 | | 297.2996 | 5.915665 | 3449.766 | 703.9375 |  |  |  |  |  |
| 25.11667 | | 297.3986 | 5.936384 | 3443.883 | 704.3047 |  |  |  |  |  |
| 25.125 | | 297.4938 | 5.954473 | 3437.992 | 704.4766 |  |  |  |  |  |
| 25.13333 | | 297.5944 | 5.971056 | 3431.977 | 704.0391 |  |  |  |  |  |
| 25.14167 | | 297.6904 | 5.991346 | 3426.102 | 704.0703 |  |  |  |  |  |
| 25.15 | | 297.7925 | 6.0094 | 3420.227 | 704.0156 |  |  |  |  |  |
| 25.15833 | | 297.8923 | 6.02741 | 3414.414 | 704.5859 |  |  |  |  |  |
| 25.16667 | | 297.9921 | 6.046923 | 3408.523 | 704.1328 |  |  |  |  |  |
| 25.175 | | 298.0873 | 6.063337 | 3402.672 | 703.2266 |  |  |  |  |  |
| 25.18333 | | 298.1864 | 6.081786 | 3396.883 | 702.4375 |  |  |  |  |  |
| 25.19167 | | 298.2869 | 6.100439 | 3391.039 | 702.25 |  |  |  |  |  |
| 25.2 | | 298.3867 | 6.116049 | 3385.211 | 701.6406 |  |  |  |  |  |
| 25.20833 | | 298.485 | 6.135768 | 3379.297 | 700.4141 |  |  |  |  |  |
| 25.21667 | | 298.581 | 6.153244 | 3373.516 | 699.1094 |  |  |  |  |  |
| 25.225 | | 298.6823 | 6.169213 | 3367.789 | 697.4688 |  |  |  |  |  |
| 25.23333 | | 298.7798 | 6.188365 | 3362.078 | 696.0781 |  |  |  |  |  |
| 25.24167 | | 298.8743 | 6.204308 | 3356.273 | 693.8203 |  |  |  |  |  |
| 25.25 | | 298.9687 | 6.220667 | 3350.539 | 691.4297 |  |  |  |  |  |
| 25.25833 | | 299.0684 | 6.238462 | 3344.875 | 689.2266 |  |  |  |  |  |
| 25.26667 | | 299.1662 | 6.253496 | 3339.242 | 687.8359 |  |  |  |  |  |
| 25.275 | | 299.2618 | 6.270422 | 3333.672 | 685.5391 |  |  |  |  |  |
| 25.28333 | | 299.3574 | 6.286829 | 3328.07 | 683.0859 |  |  |  |  |  |
| 25.29167 | | 299.4545 | 6.300607 | 3322.602 | 680.2578 |  |  |  |  |  |
| 25.3 | | 299.5515 | 6.318066 | 3317.164 | 677.9766 |  |  |  |  |  |
| 25.30833 | | 299.6448 | 6.333084 | 3311.719 | 675.8594 |  |  |  |  |  |
| 25.31667 | | 299.7374 | 6.345601 | 3306.18 | 672.9766 |  |  |  |  |  |
| 25.325 | | 299.836 | 6.36272 | 3300.781 | 670.1484 |  |  |  |  |  |
| 25.33333 | | 299.9354 | 6.375836 | 3295.406 | 667.4453 |  |  |  |  |  |
| 25.34167 | | 300.0324 | 6.388945 | 3290.109 | 664.9375 |  |  |  |  |  |
| 25.35 | | 300.1273 | 6.404439 | 3284.758 | 661.8281 |  |  |  |  |  |
| 25.35833 | | 300.2198 | 6.416758 | 3279.398 | 658.6172 |  |  |  |  |  |
| 25.36667 | | 300.3176 | 6.43 | 3274.156 | 655.0781 |  |  |  |  |  |
| 25.375 | | 300.4132 | 6.444346 | 3268.93 | 652.2734 |  |  |  |  |  |
| 25.38333 | | 300.5065 | 6.455593 | 3263.727 | 649.3984 |  |  |  |  |  |
| 25.39167 | | 300.6051 | 6.46891 | 3258.492 | 645.9531 |  |  |  |  |  |
| 25.4 | | 300.7045 | 6.481508 | 3253.375 | 642.5078 |  |  |  |  |  |
| 25.40833 | | 300.8023 | 6.491765 | 3248.289 | 639.1563 |  |  |  |  |  |
| 25.41667 | | 300.8964 | 6.506109 | 3243.273 | 636.2031 |  |  |  |  |  |
| 25.425 | | 300.9897 | 6.516968 | 3238.188 | 632.3828 |  |  |  |  |  |
| 25.43333 | | 301.0822 | 6.526628 | 3233.133 | 628.3438 |  |  |  |  |  |
| 25.44167 | | 301.1778 | 6.540202 | 3228.172 | 624.4688 |  |  |  |  |  |
| 25.45 | | 301.2703 | 6.549496 | 3223.234 | 621.0703 |  |  |  |  |  |
| 25.45833 | | 301.3621 | 6.558087 | 3218.328 | 617.3281 |  |  |  |  |  |
| 25.46667 | | 301.4599 | 6.569928 | 3213.367 | 612.8984 |  |  |  |  |  |
| 25.475 | | 301.554 | 6.577532 | 3208.57 | 608.5938 |  |  |  |  |  |
| 25.48333 | | 301.6503 | 6.58642 | 3203.805 | 604.5781 |  |  |  |  |  |
| 25.49167 | | 301.7428 | 6.596425 | 3199.078 | 600.6016 |  |  |  |  |  |
| 25.5 | | 301.8362 | 6.603478 | 3194.305 | 596.0703 |  |  |  |  |  |
| 25.50833 | | 301.9272 | 6.612203 | 3189.617 | 591.6406 |  |  |  |  |  |
| 25.51667 | | 302.0242 | 6.620993 | 3185.039 | 587.3828 |  |  |  |  |  |
| 25.525 | | 302.1176 | 6.6273 | 3180.484 | 583.4922 |  |  |  |  |  |
| 25.53333 | | 302.2093 | 6.635569 | 3175.922 | 579.1563 |  |  |  |  |  |
| 25.54167 | | 302.3049 | 6.642204 | 3171.391 | 574.6328 |  |  |  |  |  |
| 25.55 | | 302.3951 | 6.647347 | 3166.969 | 569.9297 |  |  |  |  |  |
| 25.55833 | | 302.4892 | 6.655316 | 3162.547 | 565.6016 |  |  |  |  |  |
| 25.56667 | | 302.5802 | 6.660526 | 3158.148 | 561.6172 |  |  |  |  |  |
| 25.575 | | 302.6728 | 6.664785 | 3153.734 | 557.1406 |  |  |  |  |  |
| 25.58333 | | 302.7676 | 6.672184 | 3149.422 | 552.2891 |  |  |  |  |  |
| 25.59167 | | 302.8616 | 6.675926 | 3145.156 | 547.7188 |  |  |  |  |  |
| 25.6 | | 302.9549 | 6.679498 | 3140.953 | 543.4297 |  |  |  |  |  |
| 25.60833 | | 303.0467 | 6.68662 | 3136.742 | 538.8672 |  |  |  |  |  |
| 25.61667 | | 303.1415 | 6.688922 | 3132.516 | 534.1719 |  |  |  |  |  |
| 25.625 | | 303.234 | 6.692246 | 3128.398 | 529.4219 |  |  |  |  |  |
| 25.63333 | | 303.3296 | 6.698217 | 3124.367 | 525.1563 |  |  |  |  |  |
| 25.64167 | | 303.4221 | 6.699833 | 3120.344 | 521.0547 |  |  |  |  |  |
| 25.65 | | 303.5132 | 6.703051 | 3116.313 | 516.3594 |  |  |  |  |  |
| 25.65833 | | 303.6034 | 6.70772 | 3112.352 | 511.9297 |  |  |  |  |  |
| 25.66667 | | 303.6929 | 6.708354 | 3108.438 | 507.6406 |  |  |  |  |  |
| 25.675 | | 303.7847 | 6.710452 | 3104.586 | 503.7031 |  |  |  |  |  |
| 25.68333 | | 303.8727 | 6.714207 | 3100.695 | 499.2969 |  |  |  |  |  |
| 25.69167 | | 303.9622 | 6.713793 | 3096.82 | 494.8906 |  |  |  |  |  |
| 25.7 | | 304.0487 | 6.714943 | 3093.063 | 490.625 |  |  |  |  |  |
| 25.70833 | | 304.1397 | 6.718584 | 3089.289 | 486.3984 |  |  |  |  |  |
| 25.71667 | | 304.2292 | 6.717639 | 3085.555 | 482.4688 |  |  |  |  |  |
| 25.725 | | 304.3164 | 6.718371 | 3081.789 | 477.7344 |  |  |  |  |  |
| 25.73333 | | 304.4036 | 6.721359 | 3078.133 | 473.2188 |  |  |  |  |  |
| 25.74167 | | 304.4939 | 6.720083 | 3074.492 | 468.6719 |  |  |  |  |  |
| 25.75 | | 304.5857 | 6.720658 | 3070.898 | 464.9141 |  |  |  |  |  |
| 25.75833 | | 304.6767 | 6.723353 | 3067.313 | 460.9922 |  |  |  |  |  |
| 25.76667 | | 304.7692 | 6.721242 | 3063.742 | 456.6953 |  |  |  |  |  |
| 25.775 | | 304.8618 | 6.721086 | 3060.297 | 452.3984 |  |  |  |  |  |
| 25.78333 | | 304.9566 | 6.722979 | 3056.852 | 448.5781 |  |  |  |  |  |
| 25.79167 | | 305.0468 | 6.72055 | 3053.469 | 444.7266 |  |  |  |  |  |
| 25.8 | | 305.1364 | 6.720567 | 3050 | 440.5391 |  |  |  |  |  |
| 25.80833 | | 305.2251 | 6.721758 | 3046.586 | 436.5781 |  |  |  |  |  |
| 25.81667 | | 305.3169 | 6.71852 | 3043.258 | 432.8281 |  |  |  |  |  |
| 25.825 | | 305.4102 | 6.717547 | 3039.977 | 429.3359 |  |  |  |  |  |
| 25.83333 | | 305.4997 | 6.717577 | 3036.656 | 425.3594 |  |  |  |  |  |
| 25.84167 | | 305.5899 | 6.713685 | 3033.375 | 420.9766 |  |  |  |  |  |
| 25.85 | | 305.6817 | 6.712344 | 3030.164 | 417.4375 |  |  |  |  |  |
| 25.85833 | | 305.7712 | 6.711738 | 3026.961 | 413.9375 |  |  |  |  |  |
| 25.86667 | | 305.8584 | 6.707198 | 3023.773 | 410.1875 |  |  |  |  |  |
| 25.875 | | 305.9487 | 6.705189 | 3020.57 | 406.2813 |  |  |  |  |  |
| 25.88333 | | 306.0397 | 6.703587 | 3017.469 | 402.6172 |  |  |  |  |  |
| 25.89167 | | 306.1353 | 6.69905 | 3014.461 | 399.4766 |  |  |  |  |  |
| 25.9 | | 306.2263 | 6.697343 | 3011.375 | 396.125 |  |  |  |  |  |
| 25.90833 | | 306.3135 | 6.694846 | 3008.297 | 392.7109 |  |  |  |  |  |
| 25.91667 | | 306.4023 | 6.689498 | 3005.305 | 389.4219 |  |  |  |  |  |
| 25.925 | | 306.4918 | 6.686907 | 3002.344 | 386.4297 |  |  |  |  |  |
| 25.93333 | | 306.5782 | 6.683928 | 2999.391 | 383.4141 |  |  |  |  |  |
| 25.94167 | | 306.6693 | 6.679044 | 2996.391 | 380.3047 |  |  |  |  |  |
| 25.95 | | 306.7595 | 6.677149 | 2993.461 | 377.3516 |  |  |  |  |  |
| 25.95833 | | 306.8467 | 6.675166 | 2990.555 | 374.0938 |  |  |  |  |  |
| 25.96667 | | 306.9355 | 6.670326 | 2987.664 | 371.4141 |  |  |  |  |  |
| 25.975 | | 307.0219 | 6.667838 | 2984.766 | 368.1172 |  |  |  |  |  |
| 25.98333 | | 307.1115 | 6.66544 | 2981.891 | 364.8594 |  |  |  |  |  |
| 25.99167 | | 307.2017 | 6.659754 | 2979.063 | 361.8828 |  |  |  |  |  |
| 26 | | 307.292 | 6.657454 | 2976.242 | 358.8516 |  |  |  |  |  |
| 26.00833 | | 307.3807 | 6.655069 | 2973.484 | 355.9844 |  |  |  |  |  |
| 26.01667 | | 307.4695 | 6.648734 | 2970.672 | 352.8984 |  |  |  |  |  |
| 26.025 | | 307.5575 | 6.645878 | 2967.977 | 349.7422 |  |  |  |  |  |
| 26.03333 | | 307.6454 | 6.642945 | 2965.289 | 346.7422 |  |  |  |  |  |
| 26.04167 | | 307.7342 | 6.635792 | 2962.602 | 344.3672 |  |  |  |  |  |
| 26.05 | | 307.8237 | 6.632635 | 2959.945 | 341.3984 |  |  |  |  |  |
| 26.05833 | | 307.9155 | 6.629658 | 2957.289 | 338.7266 |  |  |  |  |  |
| 26.06667 | | 308.0034 | 6.622478 | 2954.695 | 335.9688 |  |  |  |  |  |
| 26.075 | | 308.0937 | 6.618896 | 2952.125 | 333.3438 |  |  |  |  |  |
| 26.08333 | | 308.1825 | 6.616033 | 2949.57 | 330.9688 |  |  |  |  |  |
| 26.09167 | | 308.2712 | 6.609177 | 2946.938 | 328.1641 |  |  |  |  |  |
| 26.1 | | 308.3592 | 6.606488 | 2944.43 | 325.3438 |  |  |  |  |  |
| 26.10833 | | 308.451 | 6.60378 | 2941.891 | 322.625 |  |  |  |  |  |
| 26.11667 | | 308.542 | 6.596797 | 2939.391 | 320.2578 |  |  |  |  |  |
| 26.125 | | 308.6322 | 6.593849 | 2936.906 | 317.8594 |  |  |  |  |  |
| 26.13333 | | 308.7217 | 6.590497 | 2934.406 | 315.4688 |  |  |  |  |  |
| 26.14167 | | 308.8082 | 6.583087 | 2931.992 | 313.0469 |  |  |  |  |  |
| 26.15 | | 308.8969 | 6.579698 | 2929.586 | 310.8594 |  |  |  |  |  |
| 26.15833 | | 308.9842 | 6.576611 | 2927.195 | 308.5313 |  |  |  |  |  |
| 26.16667 | | 309.0729 | 6.569549 | 2924.781 | 305.9219 |  |  |  |  |  |
| 26.175 | | 309.1632 | 6.565784 | 2922.398 | 303.5547 |  |  |  |  |  |
| 26.18333 | | 309.2542 | 6.562179 | 2920.031 | 301.5078 |  |  |  |  |  |
| 26.19167 | | 309.3422 | 6.554664 | 2917.68 | 299.7813 |  |  |  |  |  |
| 26.2 | | 309.4286 | 6.550964 | 2915.328 | 297.5625 |  |  |  |  |  |
| 26.20833 | | 309.5151 | 6.54705 | 2913.016 | 295.1406 |  |  |  |  |  |
| 26.21667 | | 309.6024 | 6.540371 | 2910.766 | 293.2266 |  |  |  |  |  |
| 26.225 | | 309.6896 | 6.536189 | 2908.484 | 291.2109 |  |  |  |  |  |
| 26.23333 | | 309.7791 | 6.532833 | 2906.195 | 289.3203 |  |  |  |  |  |
| 26.24167 | | 309.8663 | 6.526414 | 2903.875 | 287.4375 |  |  |  |  |  |
| 26.25 | | 309.9528 | 6.52243 | 2901.648 | 285.5938 |  |  |  |  |  |
| 26.25833 | | 310.04 | 6.519274 | 2899.453 | 283.7656 |  |  |  |  |  |
| 26.26667 | | 310.128 | 6.51366 | 2897.211 | 281.7813 |  |  |  |  |  |
| 26.275 | | 310.213 | 6.509799 | 2895.008 | 279.75 |  |  |  |  |  |
| 26.28333 | | 310.3017 | 6.506873 | 2892.797 | 277.8828 |  |  |  |  |  |
| 26.29167 | | 310.3912 | 6.501427 | 2890.609 | 276 |  |  |  |  |  |
| 26.3 | | 310.4792 | 6.496828 | 2888.414 | 274.2188 |  |  |  |  |  |
| 26.30833 | | 310.5694 | 6.493709 | 2886.25 | 272.3828 |  |  |  |  |  |
| 26.31667 | | 310.6544 | 6.48833 | 2884.109 | 270.3125 |  |  |  |  |  |
| 26.325 | | 310.7439 | 6.483194 | 2882.008 | 268.3281 |  |  |  |  |  |
| 26.33333 | | 310.8357 | 6.480019 | 2879.891 | 266.4219 |  |  |  |  |  |
| 26.34167 | | 310.9244 | 6.474482 | 2877.789 | 264.6172 |  |  |  |  |  |
| 26.35 | | 311.0147 | 6.468604 | 2875.695 | 262.4766 |  |  |  |  |  |
| 26.35833 | | 311.1057 | 6.465232 | 2873.633 | 260.5391 |  |  |  |  |  |
| 26.36667 | | 311.1944 | 6.459456 | 2871.602 | 258.3516 |  |  |  |  |  |
| 26.375 | | 311.2847 | 6.453763 | 2869.586 | 256.5156 |  |  |  |  |  |
| 26.38333 | | 311.3727 | 6.450564 | 2867.586 | 254.5391 |  |  |  |  |  |
| 26.39167 | | 311.4569 | 6.445351 | 2865.578 | 252.5 |  |  |  |  |  |
| 26.4 | | 311.5456 | 6.439722 | 2863.633 | 250.6484 |  |  |  |  |  |
| 26.40833 | | 311.6336 | 6.437141 | 2861.68 | 249.0547 |  |  |  |  |  |
| 26.41667 | | 311.7201 | 6.432305 | 2859.789 | 247.6328 |  |  |  |  |  |
| 26.425 | | 311.8096 | 6.42645 | 2857.852 | 246.25 |  |  |  |  |  |
| 26.43333 | | 311.896 | 6.424084 | 2855.969 | 244.8047 |  |  |  |  |  |
| 26.44167 | | 311.9833 | 6.419017 | 2854.086 | 243.3516 |  |  |  |  |  |
| 26.45 | | 312.0728 | 6.412938 | 2852.219 | 242.5469 |  |  |  |  |  |
| 26.45833 | | 312.16 | 6.410604 | 2850.305 | 241.1563 |  |  |  |  |  |
| 26.46667 | | 312.2495 | 6.405727 | 2848.398 | 239.9688 |  |  |  |  |  |
| 26.475 | | 312.342 | 6.400052 | 2846.484 | 238.9063 |  |  |  |  |  |
| 26.48333 | | 312.433 | 6.397763 | 2844.594 | 237.8516 |  |  |  |  |  |
| 26.49167 | | 312.5241 | 6.393078 | 2842.727 | 236.4141 |  |  |  |  |  |
| 26.5 | | 312.6121 | 6.387539 | 2840.773 | 235.0703 |  |  |  |  |  |
| 26.50833 | | 312.6985 | 6.386148 | 2838.922 | 233.5313 |  |  |  |  |  |
| 26.51667 | | 312.788 | 6.381568 | 2837.031 | 232.2656 |  |  |  |  |  |
| 26.525 | | 312.8775 | 6.375924 | 2835.148 | 231.3047 |  |  |  |  |  |
| 26.53333 | | 312.9655 | 6.37482 | 2833.266 | 230.0469 |  |  |  |  |  |
| 26.54167 | | 313.0543 | 6.370577 | 2831.461 | 229.0313 |  |  |  |  |  |
| 26.55 | | 313.143 | 6.365005 | 2829.641 | 227.5938 |  |  |  |  |  |
| 26.55833 | | 313.2318 | 6.363663 | 2827.875 | 226.3828 |  |  |  |  |  |
| 26.56667 | | 313.3205 | 6.359332 | 2826.07 | 225.2813 |  |  |  |  |  |
| 26.575 | | 313.407 | 6.353407 | 2824.242 | 224.1406 |  |  |  |  |  |
| 26.58333 | | 313.498 | 6.352576 | 2822.453 | 223.1172 |  |  |  |  |  |
| 26.59167 | | 313.589 | 6.348131 | 2820.641 | 222.1797 |  |  |  |  |  |
| 26.6 | | 313.6793 | 6.342399 | 2818.906 | 221.0938 |  |  |  |  |  |
| 26.60833 | | 313.7695 | 6.34178 | 2817.164 | 220.0234 |  |  |  |  |  |
| 26.61667 | | 313.8598 | 6.337416 | 2815.391 | 218.6172 |  |  |  |  |  |
| 26.625 | | 313.95 | 6.331875 | 2813.648 | 217.3594 |  |  |  |  |  |
| 26.63333 | | 314.0396 | 6.33102 | 2811.898 | 216.4609 |  |  |  |  |  |
| 26.64167 | | 314.129 | 6.327001 | 2810.133 | 215.375 |  |  |  |  |  |
| 26.65 | | 314.2178 | 6.321755 | 2808.398 | 214.1016 |  |  |  |  |  |
| 26.65833 | | 314.3065 | 6.320597 | 2806.68 | 212.9531 |  |  |  |  |  |
| 26.66667 | | 314.3953 | 6.316363 | 2805.023 | 211.8516 |  |  |  |  |  |
| 26.675 | | 314.4832 | 6.310946 | 2803.352 | 210.4531 |  |  |  |  |  |
| 26.68333 | | 314.5697 | 6.30937 | 2801.641 | 209.4141 |  |  |  |  |  |
| 26.69167 | | 314.6562 | 6.305659 | 2799.961 | 208.0469 |  |  |  |  |  |
| 26.7 | | 314.7457 | 6.300744 | 2798.32 | 206.9688 |  |  |  |  |  |
| 26.70833 | | 314.8337 | 6.299387 | 2796.68 | 205.8359 |  |  |  |  |  |
| 26.71667 | | 314.9224 | 6.29614 | 2795.039 | 204.7422 |  |  |  |  |  |
| 26.725 | | 315.0112 | 6.291707 | 2793.445 | 203.6172 |  |  |  |  |  |
| 26.73333 | | 315.1014 | 6.290355 | 2791.82 | 202.5781 |  |  |  |  |  |
| 26.74167 | | 315.1932 | 6.287322 | 2790.25 | 201.8359 |  |  |  |  |  |
| 26.75 | | 315.2804 | 6.282771 | 2788.648 | 200.7578 |  |  |  |  |  |
| 26.75833 | | 315.3699 | 6.281229 | 2787.07 | 199.9375 |  |  |  |  |  |
| 26.76667 | | 315.4602 | 6.278168 | 2785.492 | 198.8672 |  |  |  |  |  |
| 26.775 | | 315.552 | 6.273781 | 2783.93 | 198.0938 |  |  |  |  |  |
| 26.78333 | | 315.64 | 6.272378 | 2782.367 | 197.3594 |  |  |  |  |  |
| 26.79167 | | 315.7274 | 6.269203 | 2780.766 | 196.4766 |  |  |  |  |  |
| 26.8 | | 315.8143 | 6.264764 | 2779.219 | 195.5703 |  |  |  |  |  |
| 26.80833 | | 315.9012 | 6.262748 | 2777.641 | 194.6641 |  |  |  |  |  |
| 26.81667 | | 315.9896 | 6.259915 | 2776.102 | 193.8516 |  |  |  |  |  |
| 26.825 | | 316.075 | 6.2556 | 2774.531 | 192.8906 |  |  |  |  |  |
| 26.83333 | | 316.1642 | 6.253913 | 2772.961 | 192.1016 |  |  |  |  |  |
| 26.84167 | | 316.2518 | 6.252005 | 2771.414 | 191.2109 |  |  |  |  |  |
| 26.85 | | 316.341 | 6.248102 | 2769.875 | 190.4297 |  |  |  |  |  |
| 26.85833 | | 316.4272 | 6.246306 | 2768.359 | 189.6328 |  |  |  |  |  |
| 26.86667 | | 316.5163 | 6.244372 | 2766.828 | 188.75 |  |  |  |  |  |
| 26.875 | | 316.607 | 6.240253 | 2765.32 | 187.7891 |  |  |  |  |  |
| 26.88333 | | 316.6961 | 6.238453 | 2763.797 | 186.8594 |  |  |  |  |  |
| 26.89167 | | 316.7838 | 6.236817 | 2762.305 | 186.1797 |  |  |  |  |  |
| 26.9 | | 316.8707 | 6.232955 | 2760.805 | 185.2891 |  |  |  |  |  |
| 26.90833 | | 316.9591 | 6.231012 | 2759.313 | 184.4063 |  |  |  |  |  |
| 26.91667 | | 317.046 | 6.229586 | 2757.844 | 183.2422 |  |  |  |  |  |
| 26.925 | | 317.1352 | 6.22542 | 2756.375 | 182.4219 |  |  |  |  |  |
| 26.93333 | | 317.2206 | 6.223502 | 2754.922 | 181.5234 |  |  |  |  |  |
| 26.94167 | | 317.309 | 6.222322 | 2753.445 | 180.5156 |  |  |  |  |  |
| 26.95 | | 317.3936 | 6.218167 | 2751.992 | 179.9297 |  |  |  |  |  |
| 26.95833 | | 317.4806 | 6.216553 | 2750.563 | 179.25 |  |  |  |  |  |
| 26.96667 | | 317.5697 | 6.215965 | 2749.18 | 178.7813 |  |  |  |  |  |
| 26.975 | | 317.6596 | 6.212243 | 2747.742 | 178.4844 |  |  |  |  |  |
| 26.98333 | | 317.748 | 6.21025 | 2746.328 | 178.0547 |  |  |  |  |  |
| 26.99167 | | 317.8372 | 6.209997 | 2744.93 | 177.6641 |  |  |  |  |  |
| 27 | | 317.9248 | 6.205609 | 2743.492 | 177.75 |  |  |  |  |  |
| 27.00833 | | 318.0125 | 6.203316 | 2742.063 | 177.5703 |  |  |  |  |  |
| 27.01667 | | 318.1024 | 6.203281 | 2740.617 | 177.3438 |  |  |  |  |  |
| 27.025 | | 318.1901 | 6.198741 | 2739.148 | 177.3125 |  |  |  |  |  |
| 27.03333 | | 318.2822 | 6.197162 | 2737.711 | 176.8984 |  |  |  |  |  |
| 27.04167 | | 318.3714 | 6.197351 | 2736.25 | 176.6563 |  |  |  |  |  |
| 27.05 | | 318.4598 | 6.193227 | 2734.734 | 176.2656 |  |  |  |  |  |
| 27.05833 | | 318.5482 | 6.191442 | 2733.266 | 175.6484 |  |  |  |  |  |
| 27.06667 | | 318.6359 | 6.19188 | 2731.797 | 175.3047 |  |  |  |  |  |
| 27.075 | | 318.7258 | 6.187693 | 2730.305 | 174.7969 |  |  |  |  |  |
| 27.08333 | | 318.8164 | 6.18645 | 2728.875 | 174.2266 |  |  |  |  |  |
| 27.09167 | | 318.9033 | 6.186493 | 2727.422 | 173.375 |  |  |  |  |  |
| 27.1 | | 318.9933 | 6.18258 | 2726 | 172.7266 |  |  |  |  |  |
| 27.10833 | | 319.0847 | 6.182013 | 2724.609 | 171.7344 |  |  |  |  |  |
| 27.11667 | | 319.1716 | 6.181545 | 2723.188 | 171.3594 |  |  |  |  |  |
| 27.125 | | 319.2615 | 6.178046 | 2721.781 | 170.6563 |  |  |  |  |  |
| 27.13333 | | 319.3514 | 6.177182 | 2720.406 | 169.8047 |  |  |  |  |  |
| 27.14167 | | 319.4383 | 6.176561 | 2719.055 | 169.25 |  |  |  |  |  |
| 27.15 | | 319.5274 | 6.172633 | 2717.703 | 168.4844 |  |  |  |  |  |
| 27.15833 | | 319.6151 | 6.171637 | 2716.383 | 168.0625 |  |  |  |  |  |
| 27.16667 | | 319.7028 | 6.171803 | 2715.008 | 167.3906 |  |  |  |  |  |
| 27.175 | | 319.7927 | 6.167559 | 2713.664 | 166.6406 |  |  |  |  |  |
| 27.18333 | | 319.8818 | 6.166917 | 2712.344 | 165.9688 |  |  |  |  |  |
| 27.19167 | | 319.9717 | 6.167249 | 2711 | 165.5391 |  |  |  |  |  |
| 27.2 | | 320.0602 | 6.163052 | 2709.688 | 164.9297 |  |  |  |  |  |
| 27.20833 | | 320.1471 | 6.162184 | 2708.336 | 164.4219 |  |  |  |  |  |
| 27.21667 | | 320.2332 | 6.162452 | 2707.023 | 164.1406 |  |  |  |  |  |
| 27.225 | | 320.3216 | 6.158547 | 2705.719 | 163.7813 |  |  |  |  |  |
| 27.23333 | | 320.4123 | 6.157548 | 2704.422 | 163.3594 |  |  |  |  |  |
| 27.24167 | | 320.4999 | 6.158041 | 2703.086 | 162.8906 |  |  |  |  |  |
| 27.25 | | 320.5891 | 6.154174 | 2701.781 | 162.4219 |  |  |  |  |  |
| 27.25833 | | 320.6783 | 6.154004 | 2700.461 | 162.25 |  |  |  |  |  |
| 27.26667 | | 320.7629 | 6.154521 | 2699.133 | 162.2344 |  |  |  |  |  |
| 27.275 | | 320.8468 | 6.151099 | 2697.805 | 161.8281 |  |  |  |  |  |
| 27.28333 | | 320.9375 | 6.150525 | 2696.492 | 161.3984 |  |  |  |  |  |
| 27.29167 | | 321.0266 | 6.15104 | 2695.18 | 160.9141 |  |  |  |  |  |
| 27.3 | | 321.1165 | 6.147645 | 2693.883 | 160.4297 |  |  |  |  |  |
| 27.30833 | | 321.205 | 6.146861 | 2692.555 | 159.6484 |  |  |  |  |  |
| 27.31667 | | 321.2934 | 6.147378 | 2691.195 | 159.0156 |  |  |  |  |  |
| 27.325 | | 321.381 | 6.144403 | 2689.891 | 158.1406 |  |  |  |  |  |
| 27.33333 | | 321.4709 | 6.144002 | 2688.594 | 157.4219 |  |  |  |  |  |
| 27.34167 | | 321.5586 | 6.144748 | 2687.32 | 156.9531 |  |  |  |  |  |
| 27.35 | | 321.6493 | 6.141886 | 2686.047 | 156.1016 |  |  |  |  |  |
| 27.35833 | | 321.7399 | 6.142005 | 2684.813 | 155.6172 |  |  |  |  |  |
| 27.36667 | | 321.8291 | 6.142171 | 2683.555 | 155.1406 |  |  |  |  |  |
| 27.375 | | 321.9175 | 6.138773 | 2682.352 | 154.8125 |  |  |  |  |  |
| 27.38333 | | 322.0059 | 6.138324 | 2681.125 | 154.4609 |  |  |  |  |  |
| 27.39167 | | 322.0965 | 6.138716 | 2679.875 | 154.1406 |  |  |  |  |  |
| 27.4 | | 322.1864 | 6.135487 | 2678.664 | 153.875 |  |  |  |  |  |
| 27.40833 | | 322.2779 | 6.135048 | 2677.414 | 153.7031 |  |  |  |  |  |
| 27.41667 | | 322.367 | 6.135053 | 2676.164 | 153.5547 |  |  |  |  |  |
| 27.425 | | 322.4562 | 6.132004 | 2674.891 | 153.2813 |  |  |  |  |  |
| 27.43333 | | 322.5438 | 6.131439 | 2673.641 | 153.0781 |  |  |  |  |  |
| 27.44167 | | 322.633 | 6.131425 | 2672.375 | 152.7188 |  |  |  |  |  |
| 27.45 | | 322.7229 | 6.128235 | 2671.117 | 152.3828 |  |  |  |  |  |
| 27.45833 | | 322.8105 | 6.128012 | 2669.844 | 151.9063 |  |  |  |  |  |
| 27.46667 | | 322.9005 | 6.128398 | 2668.57 | 151.375 |  |  |  |  |  |
| 27.475 | | 322.9881 | 6.125884 | 2667.313 | 150.8828 |  |  |  |  |  |
| 27.48333 | | 323.075 | 6.125824 | 2666.063 | 150.3359 |  |  |  |  |  |
| 27.49167 | | 323.1642 | 6.126902 | 2664.828 | 149.8828 |  |  |  |  |  |
| 27.5 | | 323.2526 | 6.125065 | 2663.594 | 149.4844 |  |  |  |  |  |
| 27.50833 | | 323.3433 | 6.124794 | 2662.391 | 148.7578 |  |  |  |  |  |
| 27.51667 | | 323.4347 | 6.125376 | 2661.188 | 148.2109 |  |  |  |  |  |
| 27.525 | | 323.5253 | 6.123272 | 2660 | 147.8984 |  |  |  |  |  |
| 27.53333 | | 323.613 | 6.123208 | 2658.813 | 147.3438 |  |  |  |  |  |
| 27.54167 | | 323.7044 | 6.123944 | 2657.617 | 146.9844 |  |  |  |  |  |
| 27.55 | | 323.7913 | 6.121998 | 2656.422 | 146.625 |  |  |  |  |  |
| 27.55833 | | 323.8819 | 6.121657 | 2655.273 | 146.5 |  |  |  |  |  |
| 27.56667 | | 323.9726 | 6.122486 | 2654.102 | 146.1016 |  |  |  |  |  |
| 27.575 | | 324.061 | 6.120317 | 2652.906 | 145.6953 |  |  |  |  |  |
| 27.58333 | | 324.1494 | 6.120122 | 2651.742 | 145.2109 |  |  |  |  |  |
| 27.59167 | | 324.2386 | 6.120942 | 2650.563 | 144.7344 |  |  |  |  |  |
| 27.6 | | 324.3262 | 6.11898 | 2649.375 | 144.3438 |  |  |  |  |  |
| 27.60833 | | 324.4161 | 6.118409 | 2648.164 | 143.6875 |  |  |  |  |  |
| 27.61667 | | 324.5031 | 6.118911 | 2646.992 | 143.1563 |  |  |  |  |  |
| 27.625 | | 324.5907 | 6.117313 | 2645.82 | 142.6328 |  |  |  |  |  |
| 27.63333 | | 324.6799 | 6.116964 | 2644.664 | 142.3516 |  |  |  |  |  |
| 27.64167 | | 324.7698 | 6.118081 | 2643.516 | 141.6406 |  |  |  |  |  |
| 27.65 | | 324.8597 | 6.116542 | 2642.359 | 141.1953 |  |  |  |  |  |
| 27.65833 | | 324.9481 | 6.115968 | 2641.234 | 140.7188 |  |  |  |  |  |
| 27.66667 | | 325.0373 | 6.11664 | 2640.102 | 140.5547 |  |  |  |  |  |
| 27.675 | | 325.1272 | 6.11464 | 2638.969 | 140.5703 |  |  |  |  |  |
| 27.68333 | | 325.2178 | 6.114042 | 2637.813 | 140.4297 |  |  |  |  |  |
| 27.69167 | | 325.307 | 6.114929 | 2636.719 | 140.0781 |  |  |  |  |  |
| 27.7 | | 325.4006 | 6.113401 | 2635.594 | 139.5156 |  |  |  |  |  |
| 27.70833 | | 325.4913 | 6.112852 | 2634.484 | 139.3203 |  |  |  |  |  |
| 27.71667 | | 325.5827 | 6.113478 | 2633.328 | 139.0859 |  |  |  |  |  |
| 27.725 | | 325.6733 | 6.111637 | 2632.148 | 138.5938 |  |  |  |  |  |
| 27.73333 | | 325.7617 | 6.111089 | 2630.984 | 138.3047 |  |  |  |  |  |
| 27.74167 | | 325.8524 | 6.112016 | 2629.859 | 138.0469 |  |  |  |  |  |
| 27.75 | | 325.9445 | 6.110392 | 2628.766 | 137.6328 |  |  |  |  |  |
| 27.75833 | | 326.0352 | 6.110836 | 2627.617 | 137.375 |  |  |  |  |  |
| 27.76667 | | 326.1274 | 6.111847 | 2626.492 | 136.9922 |  |  |  |  |  |
| 27.775 | | 326.2165 | 6.11043 | 2625.391 | 136.7734 |  |  |  |  |  |
| 27.78333 | | 326.3034 | 6.111397 | 2624.266 | 136.8516 |  |  |  |  |  |
| 27.79167 | | 326.3926 | 6.112755 | 2623.133 | 136.4688 |  |  |  |  |  |
| 27.8 | | 326.4803 | 6.111629 | 2622.039 | 136.1641 |  |  |  |  |  |
| 27.80833 | | 326.5679 | 6.112638 | 2620.914 | 136.0625 |  |  |  |  |  |
| 27.81667 | | 326.6601 | 6.113775 | 2619.805 | 136.1016 |  |  |  |  |  |
| 27.825 | | 326.7515 | 6.112393 | 2618.688 | 136.2266 |  |  |  |  |  |
| 27.83333 | | 326.8414 | 6.113364 | 2617.523 | 136.1172 |  |  |  |  |  |
| 27.84167 | | 326.929 | 6.113876 | 2616.43 | 135.8984 |  |  |  |  |  |
| 27.85 | | 327.0167 | 6.112214 | 2615.328 | 135.4844 |  |  |  |  |  |
| 27.85833 | | 327.1059 | 6.112429 | 2614.195 | 135.6172 |  |  |  |  |  |
| 27.86667 | | 327.1973 | 6.113378 | 2613.047 | 135.5547 |  |  |  |  |  |
| 27.875 | | 327.2894 | 6.111961 | 2611.891 | 135.4063 |  |  |  |  |  |
| 27.88333 | | 327.3816 | 6.112197 | 2610.758 | 135.3281 |  |  |  |  |  |
| 27.89167 | | 327.4722 | 6.113026 | 2609.656 | 135.3516 |  |  |  |  |  |
| 27.9 | | 327.5584 | 6.111261 | 2608.578 | 135.0625 |  |  |  |  |  |
| 27.90833 | | 327.6483 | 6.110884 | 2607.422 | 134.5547 |  |  |  |  |  |
| 27.91667 | | 327.736 | 6.11189 | 2606.297 | 134.3906 |  |  |  |  |  |
| 27.925 | | 327.8259 | 6.110451 | 2605.18 | 134.0469 |  |  |  |  |  |
| 27.93333 | | 327.9158 | 6.111014 | 2604.063 | 133.8984 |  |  |  |  |  |
| 27.94167 | | 328.0049 | 6.112537 | 2602.922 | 133.4922 |  |  |  |  |  |
| 27.95 | | 328.0941 | 6.111151 | 2601.836 | 133.2734 |  |  |  |  |  |
| 27.95833 | | 328.1825 | 6.111834 | 2600.766 | 133.1719 |  |  |  |  |  |
| 27.96667 | | 328.2709 | 6.112683 | 2599.672 | 133.2188 |  |  |  |  |  |
| 27.975 | | 328.3593 | 6.110978 | 2598.594 | 132.9766 |  |  |  |  |  |
| 27.98333 | | 328.4485 | 6.111736 | 2597.5 | 132.7422 |  |  |  |  |  |
| 27.99167 | | 328.5346 | 6.112116 | 2596.438 | 132.3828 |  |  |  |  |  |
| 28 | | 328.6245 | 6.110439 | 2595.352 | 132.0469 |  |  |  |  |  |
| 28.00833 | | 328.7152 | 6.111108 | 2594.25 | 131.9141 |  |  |  |  |  |
| 28.01667 | | 328.8036 | 6.111468 | 2593.141 | 131.5156 |  |  |  |  |  |
| 28.025 | | 328.8928 | 6.109582 | 2592.047 | 130.9453 |  |  |  |  |  |
| 28.03333 | | 328.9827 | 6.109881 | 2590.977 | 130.4219 |  |  |  |  |  |
| 28.04167 | | 329.0718 | 6.110638 | 2589.93 | 129.9609 |  |  |  |  |  |
| 28.05 | | 329.1617 | 6.108993 | 2588.867 | 129.25 |  |  |  |  |  |
| 28.05833 | | 329.2539 | 6.109468 | 2587.789 | 128.625 |  |  |  |  |  |
| 28.06667 | | 329.3468 | 6.111152 | 2586.742 | 128.1953 |  |  |  |  |  |
| 28.075 | | 329.442 | 6.110054 | 2585.719 | 128.0234 |  |  |  |  |  |
| 28.08333 | | 329.5326 | 6.11152 | 2584.703 | 127.7969 |  |  |  |  |  |
| 28.09167 | | 329.621 | 6.112955 | 2583.672 | 127.5781 |  |  |  |  |  |
| 28.1 | | 329.7124 | 6.112227 | 2582.695 | 127.3281 |  |  |  |  |  |
| 28.10833 | | 329.8031 | 6.113382 | 2581.703 | 127.1641 |  |  |  |  |  |
| 28.11667 | | 329.8945 | 6.11411 | 2580.695 | 127.2109 |  |  |  |  |  |
| 28.125 | | 329.9859 | 6.112596 | 2579.641 | 127.1328 |  |  |  |  |  |
| 28.13333 | | 330.078 | 6.113803 | 2578.609 | 127.2188 |  |  |  |  |  |
| 28.14167 | | 330.1672 | 6.114447 | 2577.563 | 127.125 |  |  |  |  |  |
| 28.15 | | 330.2549 | 6.113049 | 2576.539 | 127.2031 |  |  |  |  |  |
| 28.15833 | | 330.3418 | 6.11385 | 2575.484 | 127 |  |  |  |  |  |
| 28.16667 | | 330.4294 | 6.114665 | 2574.422 | 126.8047 |  |  |  |  |  |
| 28.175 | | 330.5178 | 6.113081 | 2573.367 | 126.6016 |  |  |  |  |  |
| 28.18333 | | 330.6085 | 6.113866 | 2572.281 | 126.6094 |  |  |  |  |  |
| 28.19167 | | 330.7014 | 6.115171 | 2571.234 | 126.3125 |  |  |  |  |  |
| 28.2 | | 330.7906 | 6.114103 | 2570.148 | 126.2891 |  |  |  |  |  |
| 28.20833 | | 330.8782 | 6.115153 | 2569.109 | 125.9375 |  |  |  |  |  |
| 28.21667 | | 330.9666 | 6.116334 | 2568.078 | 125.4063 |  |  |  |  |  |
| 28.225 | | 331.055 | 6.114981 | 2567.031 | 125.3359 |  |  |  |  |  |
| 28.23333 | | 331.1457 | 6.116251 | 2565.969 | 124.9609 |  |  |  |  |  |
| 28.24167 | | 331.2341 | 6.117642 | 2564.945 | 124.4375 |  |  |  |  |  |
| 28.25 | | 331.3255 | 6.116209 | 2563.898 | 124.1953 |  |  |  |  |  |
| 28.25833 | | 331.4199 | 6.117313 | 2562.891 | 123.7891 |  |  |  |  |  |
| 28.26667 | | 331.5083 | 6.11831 | 2561.898 | 123.5938 |  |  |  |  |  |
| 28.275 | | 331.5945 | 6.116835 | 2560.867 | 123.3906 |  |  |  |  |  |
| 28.28333 | | 331.6859 | 6.117728 | 2559.867 | 122.9531 |  |  |  |  |  |
| 28.29167 | | 331.778 | 6.119124 | 2558.891 | 122.7578 |  |  |  |  |  |
| 28.3 | | 331.8687 | 6.11763 | 2557.883 | 122.6484 |  |  |  |  |  |
| 28.30833 | | 331.9616 | 6.119171 | 2556.914 | 122.4375 |  |  |  |  |  |
| 28.31667 | | 332.0508 | 6.119897 | 2555.898 | 121.875 |  |  |  |  |  |
| 28.325 | | 332.1407 | 6.118363 | 2554.891 | 121.6484 |  |  |  |  |  |
| 28.33333 | | 332.2313 | 6.11964 | 2553.922 | 121.4063 |  |  |  |  |  |
| 28.34167 | | 332.3205 | 6.120196 | 2552.93 | 121 |  |  |  |  |  |
| 28.35 | | 332.4104 | 6.118453 | 2551.906 | 120.5625 |  |  |  |  |  |
| 28.35833 | | 332.504 | 6.119455 | 2550.906 | 120.3047 |  |  |  |  |  |
| 28.36667 | | 332.5894 | 6.120225 | 2549.961 | 119.9063 |  |  |  |  |  |
| 28.375 | | 332.6779 | 6.118274 | 2548.969 | 119.5 |  |  |  |  |  |
| 28.38333 | | 332.7678 | 6.119421 | 2547.984 | 119.0078 |  |  |  |  |  |
| 28.39167 | | 332.8539 | 6.120397 | 2547.023 | 118.5156 |  |  |  |  |  |
| 28.4 | | 332.9461 | 6.118671 | 2546.078 | 118.3281 |  |  |  |  |  |
| 28.40833 | | 333.0382 | 6.12038 | 2545.102 | 118.2109 |  |  |  |  |  |
| 28.41667 | | 333.1304 | 6.121698 | 2544.156 | 117.9688 |  |  |  |  |  |
| 28.425 | | 333.2203 | 6.120214 | 2543.211 | 117.5156 |  |  |  |  |  |
| 28.43333 | | 333.3109 | 6.121612 | 2542.289 | 117.4531 |  |  |  |  |  |
| 28.44167 | | 333.3971 | 6.122693 | 2541.367 | 117.0156 |  |  |  |  |  |
| 28.45 | | 333.4915 | 6.121048 | 2540.406 | 116.7969 |  |  |  |  |  |
| 28.45833 | | 333.5814 | 6.122608 | 2539.438 | 116.3984 |  |  |  |  |  |
| 28.46667 | | 333.6706 | 6.123945 | 2538.492 | 116.0625 |  |  |  |  |  |
| 28.475 | | 333.762 | 6.122048 | 2537.563 | 115.8594 |  |  |  |  |  |
| 28.48333 | | 333.8526 | 6.123604 | 2536.586 | 115.5469 |  |  |  |  |  |
| 28.49167 | | 333.9441 | 6.124314 | 2535.664 | 115.2266 |  |  |  |  |  |
| 28.5 | | 334.0317 | 6.122107 | 2534.711 | 114.8984 |  |  |  |  |  |
| 28.50833 | | 334.1216 | 6.123009 | 2533.797 | 114.6719 |  |  |  |  |  |
| 28.51667 | | 334.2123 | 6.123873 | 2532.852 | 114.1172 |  |  |  |  |  |
| 28.525 | | 334.3052 | 6.121438 | 2531.914 | 113.9688 |  |  |  |  |  |
| 28.53333 | | 334.3936 | 6.122755 | 2530.977 | 113.6406 |  |  |  |  |  |
| 28.54167 | | 334.4835 | 6.123676 | 2530.055 | 113.6484 |  |  |  |  |  |
| 28.55 | | 334.5757 | 6.121745 | 2529.125 | 113.3047 |  |  |  |  |  |
| 28.55833 | | 334.6678 | 6.123653 | 2528.195 | 113.0625 |  |  |  |  |  |
| 28.56667 | | 334.7577 | 6.123964 | 2527.313 | 113.1094 |  |  |  |  |  |
| 28.575 | | 334.8476 | 6.122821 | 2526.375 | 113.0078 |  |  |  |  |  |
| 28.58333 | | 334.9375 | 6.1248 | 2525.453 | 112.7813 |  |  |  |  |  |
| 28.59167 | | 335.0304 | 6.125339 | 2524.5 | 112.75 |  |  |  |  |  |
| 28.6 | | 335.1226 | 6.12494 | 2523.586 | 113.1328 |  |  |  |  |  |
| 28.60833 | | 335.2117 | 6.127048 | 2522.664 | 112.9375 |  |  |  |  |  |
| 28.61667 | | 335.3024 | 6.126943 | 2521.727 | 112.8906 |  |  |  |  |  |
| 28.625 | | 335.3938 | 6.126247 | 2520.789 | 112.8984 |  |  |  |  |  |
| 28.63333 | | 335.4852 | 6.128109 | 2519.859 | 112.8047 |  |  |  |  |  |
| 28.64167 | | 335.5736 | 6.127412 | 2518.922 | 112.6563 |  |  |  |  |  |
| 28.65 | | 335.662 | 6.126147 | 2517.922 | 112.5938 |  |  |  |  |  |
| 28.65833 | | 335.7497 | 6.127475 | 2517.008 | 112.25 |  |  |  |  |  |
| 28.66667 | | 335.8403 | 6.127293 | 2516.063 | 112.0234 |  |  |  |  |  |
| 28.675 | | 335.9272 | 6.125655 | 2515.109 | 111.9219 |  |  |  |  |  |
| 28.68333 | | 336.0149 | 6.127164 | 2514.18 | 111.5313 |  |  |  |  |  |
| 28.69167 | | 336.1041 | 6.127393 | 2513.258 | 111.5156 |  |  |  |  |  |
| 28.7 | | 336.1932 | 6.125589 | 2512.32 | 111.2344 |  |  |  |  |  |
| 28.70833 | | 336.2824 | 6.127767 | 2511.422 | 110.9375 |  |  |  |  |  |
| 28.71667 | | 336.3708 | 6.128197 | 2510.508 | 110.8594 |  |  |  |  |  |
| 28.725 | | 336.4607 | 6.126046 | 2509.594 | 110.8359 |  |  |  |  |  |
| 28.73333 | | 336.5514 | 6.128379 | 2508.711 | 110.6875 |  |  |  |  |  |
| 28.74167 | | 336.6443 | 6.128954 | 2507.781 | 110.7578 |  |  |  |  |  |
| 28.75 | | 336.7357 | 6.127137 | 2506.891 | 110.8906 |  |  |  |  |  |
| 28.75833 | | 336.8263 | 6.129174 | 2506 | 111.0313 |  |  |  |  |  |
| 28.76667 | | 336.9177 | 6.12956 | 2505.086 | 110.9766 |  |  |  |  |  |
| 28.775 | | 337.0099 | 6.12728 | 2504.164 | 110.9766 |  |  |  |  |  |
| 28.78333 | | 337.1013 | 6.12947 | 2503.266 | 111.0391 |  |  |  |  |  |
| 28.79167 | | 337.1927 | 6.129147 | 2502.328 | 111.125 |  |  |  |  |  |
| 28.8 | | 337.2826 | 6.127173 | 2501.383 | 110.8438 |  |  |  |  |  |
| 28.80833 | | 337.374 | 6.129402 | 2500.43 | 110.9453 |  |  |  |  |  |
| 28.81667 | | 337.4677 | 6.129116 | 2499.508 | 110.9609 |  |  |  |  |  |
| 28.825 | | 337.5583 | 6.127761 | 2498.586 | 110.7266 |  |  |  |  |  |
| 28.83333 | | 337.649 | 6.129938 | 2497.641 | 110.3984 |  |  |  |  |  |
| 28.84167 | | 337.7381 | 6.129824 | 2496.711 | 110.1563 |  |  |  |  |  |
| 28.85 | | 337.8295 | 6.128818 | 2495.813 | 109.7891 |  |  |  |  |  |
| 28.85833 | | 337.9179 | 6.131065 | 2494.883 | 109.5938 |  |  |  |  |  |
| 28.86667 | | 338.0056 | 6.130909 | 2493.953 | 109.2578 |  |  |  |  |  |
| 28.875 | | 338.097 | 6.129623 | 2493.063 | 108.8906 |  |  |  |  |  |
| 28.88333 | | 338.1884 | 6.131948 | 2492.188 | 108.9922 |  |  |  |  |  |
| 28.89167 | | 338.2776 | 6.131348 | 2491.305 | 108.7188 |  |  |  |  |  |
| 28.9 | | 338.3667 | 6.130262 | 2490.445 | 108.7344 |  |  |  |  |  |
| 28.90833 | | 338.4559 | 6.132477 | 2489.555 | 108.6953 |  |  |  |  |  |
| 28.91667 | | 338.545 | 6.132079 | 2488.695 | 108.7969 |  |  |  |  |  |
| 28.925 | | 338.6364 | 6.130564 | 2487.828 | 108.5469 |  |  |  |  |  |
| 28.93333 | | 338.7271 | 6.133059 | 2486.914 | 108.9922 |  |  |  |  |  |
| 28.94167 | | 338.8193 | 6.132786 | 2486.039 | 109.0703 |  |  |  |  |  |
| 28.95 | | 338.9114 | 6.131746 | 2485.125 | 108.9766 |  |  |  |  |  |
| 28.95833 | | 339.0021 | 6.134463 | 2484.219 | 108.875 |  |  |  |  |  |
| 28.96667 | | 339.0927 | 6.133838 | 2483.297 | 108.8906 |  |  |  |  |  |
| 28.975 | | 339.1856 | 6.132722 | 2482.422 | 108.6797 |  |  |  |  |  |
| 28.98333 | | 339.277 | 6.134722 | 2481.453 | 108.0781 |  |  |  |  |  |
| 28.99167 | | 339.3684 | 6.133828 | 2480.531 | 107.7578 |  |  |  |  |  |
| 29 | | 339.4583 | 6.132864 | 2479.617 | 107.3984 |  |  |  |  |  |
| 29.00833 | | 339.5475 | 6.135003 | 2478.734 | 107.2891 |  |  |  |  |  |
| 29.01667 | | 339.6352 | 6.134188 | 2477.813 | 106.6797 |  |  |  |  |  |
| 29.025 | | 339.7228 | 6.133029 | 2476.938 | 106.1563 |  |  |  |  |  |
| 29.03333 | | 339.8135 | 6.135351 | 2476.109 | 105.875 |  |  |  |  |  |
| 29.04167 | | 339.9034 | 6.1343 | 2475.258 | 105.7188 |  |  |  |  |  |
| 29.05 | | 339.9918 | 6.133396 | 2474.406 | 105.2188 |  |  |  |  |  |
| 29.05833 | | 340.0772 | 6.135704 | 2473.539 | 104.8594 |  |  |  |  |  |
| 29.06667 | | 340.1626 | 6.135042 | 2472.719 | 104.5859 |  |  |  |  |  |
| 29.075 | | 340.2533 | 6.133682 | 2471.914 | 104.2656 |  |  |  |  |  |
| 29.08333 | | 340.342 | 6.136409 | 2471.063 | 103.8047 |  |  |  |  |  |
| 29.09167 | | 340.4315 | 6.135451 | 2470.203 | 103.4297 |  |  |  |  |  |
| 29.1 | | 340.5203 | 6.134294 | 2469.398 | 103.2734 |  |  |  |  |  |
| 29.10833 | | 340.6136 | 6.136615 | 2468.578 | 103.0859 |  |  |  |  |  |
| 29.11667 | | 340.7038 | 6.134988 | 2467.727 | 103.0469 |  |  |  |  |  |
| 29.125 | | 340.7926 | 6.133793 | 2466.906 | 103.0469 |  |  |  |  |  |
| 29.13333 | | 340.8829 | 6.135736 | 2466.102 | 103.0156 |  |  |  |  |  |
| 29.14167 | | 340.9769 | 6.134244 | 2465.281 | 103.2891 |  |  |  |  |  |
| 29.15 | | 341.0686 | 6.133638 | 2464.438 | 103.4609 |  |  |  |  |  |
| 29.15833 | | 341.1567 | 6.135878 | 2463.586 | 103.7734 |  |  |  |  |  |
| 29.16667 | | 341.2477 | 6.134774 | 2462.727 | 104.3047 |  |  |  |  |  |
| 29.175 | | 341.3394 | 6.134137 | 2461.867 | 104.7188 |  |  |  |  |  |
| 29.18333 | | 341.4297 | 6.136318 | 2460.992 | 104.6875 |  |  |  |  |  |
| 29.19167 | | 341.5171 | 6.134927 | 2460.086 | 104.7969 |  |  |  |  |  |
| 29.2 | | 341.6044 | 6.134367 | 2459.195 | 104.7188 |  |  |  |  |  |
| 29.20833 | | 341.6939 | 6.136887 | 2458.281 | 104.7891 |  |  |  |  |  |
| 29.21667 | | 341.782 | 6.135561 | 2457.32 | 104.7578 |  |  |  |  |  |
| 29.225 | | 341.8722 | 6.135309 | 2456.391 | 104.7344 |  |  |  |  |  |
| 29.23333 | | 341.9625 | 6.137931 | 2455.523 | 104.6016 |  |  |  |  |  |
| 29.24167 | | 342.0528 | 6.136898 | 2454.633 | 104.3047 |  |  |  |  |  |
| 29.25 | | 342.1423 | 6.136661 | 2453.773 | 103.8516 |  |  |  |  |  |
| 29.25833 | | 342.2311 | 6.139074 | 2452.883 | 103.5781 |  |  |  |  |  |
| 29.26667 | | 342.3214 | 6.137304 | 2452.016 | 103.5078 |  |  |  |  |  |
| 29.275 | | 342.4109 | 6.136967 | 2451.148 | 103.3203 |  |  |  |  |  |
| 29.28333 | | 342.5012 | 6.139394 | 2450.297 | 103.0078 |  |  |  |  |  |
| 29.29167 | | 342.5893 | 6.137822 | 2449.461 | 102.6328 |  |  |  |  |  |
| 29.3 | | 342.6825 | 6.137789 | 2448.656 | 102.8594 |  |  |  |  |  |
| 29.30833 | | 342.7713 | 6.140219 | 2447.828 | 102.8594 |  |  |  |  |  |
| 29.31667 | | 342.8593 | 6.138741 | 2446.969 | 102.5703 |  |  |  |  |  |
| 29.325 | | 342.9481 | 6.138122 | 2446.133 | 102.4297 |  |  |  |  |  |
| 29.33333 | | 343.0392 | 6.140313 | 2445.305 | 102.2813 |  |  |  |  |  |
| 29.34167 | | 343.1272 | 6.138194 | 2444.492 | 102.2422 |  |  |  |  |  |
| 29.35 | | 343.216 | 6.137565 | 2443.602 | 102.1328 |  |  |  |  |  |
| 29.35833 | | 343.3055 | 6.13976 | 2442.75 | 102.0859 |  |  |  |  |  |
| 29.36667 | | 343.3958 | 6.137237 | 2441.922 | 102.1172 |  |  |  |  |  |
| 29.375 | | 343.4861 | 6.136863 | 2441.078 | 102.1016 |  |  |  |  |  |
| 29.38333 | | 343.5704 | 6.139331 | 2440.258 | 101.5938 |  |  |  |  |  |
| 29.39167 | | 343.66 | 6.137412 | 2439.414 | 101.3281 |  |  |  |  |  |
| 29.4 | | 343.7502 | 6.136645 | 2438.57 | 101.1797 |  |  |  |  |  |
| 29.40833 | | 343.8412 | 6.139323 | 2437.727 | 101.1563 |  |  |  |  |  |
| 29.41667 | | 343.9278 | 6.136907 | 2436.875 | 101.1172 |  |  |  |  |  |
| 29.425 | | 344.0189 | 6.136301 | 2436.031 | 100.7422 |  |  |  |  |  |
| 29.43333 | | 344.1084 | 6.13944 | 2435.258 | 100.5078 |  |  |  |  |  |
| 29.44167 | | 344.1994 | 6.137121 | 2434.445 | 100.0313 |  |  |  |  |  |
| 29.45 | | 344.2867 | 6.137429 | 2433.625 | 100.0156 |  |  |  |  |  |
| 29.45833 | | 344.3755 | 6.140394 | 2432.789 | 99.75781 |  |  |  |  |  |
| 29.46667 | | 344.4673 | 6.138267 | 2431.961 | 99.59375 |  |  |  |  |  |
| 29.475 | | 344.5553 | 6.137953 | 2431.164 | 99.21875 |  |  |  |  |  |
| 29.48333 | | 344.6463 | 6.140218 | 2430.375 | 99.09375 |  |  |  |  |  |
| 29.49167 | | 344.7322 | 6.137818 | 2429.594 | 99.0625 |  |  |  |  |  |
| 29.5 | | 344.8224 | 6.13777 | 2428.766 | 99.0625 |  |  |  |  |  |
| 29.50833 | | 344.912 | 6.139809 | 2427.977 | 99.15625 |  |  |  |  |  |
| 29.51667 | | 345.0045 | 6.137358 | 2427.172 | 99.21875 |  |  |  |  |  |
| 29.525 | | 345.094 | 6.137846 | 2426.391 | 99.23438 |  |  |  |  |  |
| 29.53333 | | 345.1843 | 6.139424 | 2425.57 | 99.25781 |  |  |  |  |  |
| 29.54167 | | 345.2723 | 6.136872 | 2424.75 | 99.28125 |  |  |  |  |  |
| 29.55 | | 345.3633 | 6.137102 | 2423.906 | 99.375 |  |  |  |  |  |
| 29.55833 | | 345.4536 | 6.138495 | 2423.055 | 99.41406 |  |  |  |  |  |
| 29.56667 | | 345.5424 | 6.135814 | 2422.203 | 99.39844 |  |  |  |  |  |
| 29.575 | | 345.6357 | 6.136185 | 2421.359 | 99.40625 |  |  |  |  |  |
| 29.58333 | | 345.7267 | 6.137542 | 2420.523 | 99.21875 |  |  |  |  |  |
| 29.59167 | | 345.817 | 6.135373 | 2419.688 | 99.39844 |  |  |  |  |  |
| 29.6 | | 345.9028 | 6.136396 | 2418.844 | 99.32813 |  |  |  |  |  |
| 29.60833 | | 345.9901 | 6.138365 | 2418.008 | 99.25 |  |  |  |  |  |
| 29.61667 | | 346.0797 | 6.136164 | 2417.164 | 99.14063 |  |  |  |  |  |
| 29.625 | | 346.1707 | 6.137163 | 2416.336 | 99.10938 |  |  |  |  |  |
| 29.63333 | | 346.2595 | 6.138721 | 2415.531 | 99.42188 |  |  |  |  |  |
| 29.64167 | | 346.349 | 6.136372 | 2414.68 | 99.79688 |  |  |  |  |  |
| 29.65 | | 346.4385 | 6.137351 | 2413.859 | 99.82813 |  |  |  |  |  |
| 29.65833 | | 346.5266 | 6.139056 | 2413.047 | 99.96094 |  |  |  |  |  |
| 29.66667 | | 346.6161 | 6.136618 | 2412.227 | 100.1328 |  |  |  |  |  |
| 29.675 | | 346.7049 | 6.137514 | 2411.414 | 100.1875 |  |  |  |  |  |
| 29.68333 | | 346.7944 | 6.138942 | 2410.531 | 100.1172 |  |  |  |  |  |
| 29.69167 | | 346.8847 | 6.135956 | 2409.648 | 100.4141 |  |  |  |  |  |
| 29.7 | | 346.9757 | 6.136612 | 2408.797 | 100.3984 |  |  |  |  |  |
| 29.70833 | | 347.0653 | 6.137811 | 2407.953 | 100.6484 |  |  |  |  |  |
| 29.71667 | | 347.1541 | 6.134694 | 2407.094 | 100.3828 |  |  |  |  |  |
| 29.725 | | 347.2421 | 6.135133 | 2406.25 | 99.95313 |  |  |  |  |  |
| 29.73333 | | 347.3309 | 6.136904 | 2405.422 | 99.57813 |  |  |  |  |  |
| 29.74167 | | 347.4219 | 6.133601 | 2404.547 | 99.25 |  |  |  |  |  |
| 29.75 | | 347.5122 | 6.134799 | 2403.711 | 98.98438 |  |  |  |  |  |
| 29.75833 | | 347.6003 | 6.137111 | 2402.844 | 98.04688 |  |  |  |  |  |
| 29.76667 | | 347.6898 | 6.134605 | 2402.039 | 97.60938 |  |  |  |  |  |
| 29.775 | | 347.7816 | 6.135719 | 2401.266 | 97.05469 |  |  |  |  |  |
| 29.78333 | | 347.8718 | 6.13759 | 2400.5 | 96.85156 |  |  |  |  |  |
| 29.79167 | | 347.9636 | 6.134491 | 2399.711 | 96.5 |  |  |  |  |  |
| 29.8 | | 348.0538 | 6.135187 | 2398.938 | 96.01563 |  |  |  |  |  |
| 29.80833 | | 348.1449 | 6.137141 | 2398.242 | 95.50781 |  |  |  |  |  |
| 29.81667 | | 348.2351 | 6.133795 | 2397.492 | 95.40625 |  |  |  |  |  |
| 29.825 | | 348.3224 | 6.135321 | 2396.758 | 95.27344 |  |  |  |  |  |
| 29.83333 | | 348.4113 | 6.136721 | 2395.984 | 94.90625 |  |  |  |  |  |
| 29.84167 | | 348.5012 | 6.133281 | 2395.234 | 94.64063 |  |  |  |  |  |
| 29.85 | | 348.5925 | 6.134636 | 2394.5 | 94.5625 |  |  |  |  |  |
| 29.85833 | | 348.6787 | 6.135105 | 2393.773 | 94.41406 |  |  |  |  |  |
| 29.86667 | | 348.7657 | 6.131966 | 2392.992 | 94.16406 |  |  |  |  |  |
| 29.875 | | 348.8534 | 6.133327 | 2392.227 | 93.82031 |  |  |  |  |  |
| 29.88333 | | 348.9411 | 6.134587 | 2391.484 | 93.64844 |  |  |  |  |  |
| 29.89167 | | 349.028 | 6.131146 | 2390.727 | 93.55469 |  |  |  |  |  |
| 29.9 | | 349.1157 | 6.132825 | 2389.945 | 93.39063 |  |  |  |  |  |
| 29.90833 | | 349.2049 | 6.1337 | 2389.172 | 93.25 |  |  |  |  |  |
| 29.91667 | | 349.2911 | 6.130244 | 2388.406 | 93.20313 |  |  |  |  |  |
| 29.925 | | 349.3802 | 6.131912 | 2387.672 | 93.16406 |  |  |  |  |  |
| 29.93333 | | 349.4664 | 6.132617 | 2386.906 | 93.0625 |  |  |  |  |  |
| 29.94167 | | 349.557 | 6.129346 | 2386.133 | 92.85156 |  |  |  |  |  |
| 29.95 | | 349.6454 | 6.131217 | 2385.375 | 92.54688 |  |  |  |  |  |
| 29.95833 | | 349.7346 | 6.132151 | 2384.609 | 92.625 |  |  |  |  |  |
| 29.96667 | | 349.8238 | 6.129092 | 2383.828 | 92.48438 |  |  |  |  |  |
| 29.975 | | 349.9143 | 6.131494 | 2383.055 | 92.375 |  |  |  |  |  |
| 29.98333 | | 350.0028 | 6.132504 | 2382.281 | 92.25781 |  |  |  |  |  |
| 29.99167 | | 350.0919 | 6.12951 | 2381.539 | 92.17188 |  |  |  |  |  |
| 30 | | 350.1818 | 6.131798 | 2380.797 | 91.99219 |  |  |  |  |  |
| 30.00833 | | 350.2687 | 6.132468 | 2380.016 | 91.92969 |  |  |  |  |  |
| 30.01667 | | 350.3586 | 6.129191 | 2379.25 | 91.875 |  |  |  |  |  |
| 30.025 | | 350.4456 | 6.13118 | 2378.5 | 91.72656 |  |  |  |  |  |
| 30.03333 | | 350.534 | 6.131649 | 2377.727 | 91.44531 |  |  |  |  |  |
| 30.04167 | | 350.6217 | 6.128359 | 2376.961 | 91.14063 |  |  |  |  |  |
| 30.05 | | 350.7101 | 6.130258 | 2376.203 | 91.00781 |  |  |  |  |  |
| 30.05833 | | 350.7985 | 6.130784 | 2375.438 | 90.82813 |  |  |  |  |  |
| 30.06667 | | 350.8869 | 6.127411 | 2374.672 | 90.6875 |  |  |  |  |  |
| 30.075 | | 350.9768 | 6.129129 | 2373.914 | 90.72656 |  |  |  |  |  |
| 30.08333 | | 351.0667 | 6.129443 | 2373.188 | 90.67969 |  |  |  |  |  |
| 30.09167 | | 351.1573 | 6.126285 | 2372.461 | 90.57813 |  |  |  |  |  |
| 30.1 | | 351.2442 | 6.128302 | 2371.711 | 90.49219 |  |  |  |  |  |
| 30.10833 | | 351.3348 | 6.128572 | 2370.961 | 90.60156 |  |  |  |  |  |
| 30.11667 | | 351.4261 | 6.125673 | 2370.219 | 90.79688 |  |  |  |  |  |
| 30.125 | | 351.516 | 6.127814 | 2369.453 | 90.78906 |  |  |  |  |  |
| 30.13333 | | 351.6037 | 6.128066 | 2368.711 | 90.71875 |  |  |  |  |  |
| 30.14167 | | 351.6907 | 6.124825 | 2367.961 | 91.23438 |  |  |  |  |  |
| 30.15 | | 351.7798 | 6.127022 | 2367.227 | 91.39063 |  |  |  |  |  |
| 30.15833 | | 351.8675 | 6.127311 | 2366.453 | 91.39844 |  |  |  |  |  |
| 30.16667 | | 351.9537 | 6.124792 | 2365.672 | 91.77344 |  |  |  |  |  |
| 30.175 | | 352.0421 | 6.12685 | 2364.914 | 92.10156 |  |  |  |  |  |
| 30.18333 | | 352.1327 | 6.126835 | 2364.164 | 92.32813 |  |  |  |  |  |
| 30.19167 | | 352.219 | 6.124512 | 2363.328 | 92.46875 |  |  |  |  |  |
| 30.2 | | 352.3066 | 6.126641 | 2362.555 | 92.52344 |  |  |  |  |  |
| 30.20833 | | 352.3958 | 6.127128 | 2361.789 | 92.59375 |  |  |  |  |  |
| 30.21667 | | 352.4864 | 6.124649 | 2360.977 | 92.69531 |  |  |  |  |  |
| 30.225 | | 352.5792 | 6.127244 | 2360.156 | 92.39844 |  |  |  |  |  |
| 30.23333 | | 352.6698 | 6.126657 | 2359.359 | 92.17969 |  |  |  |  |  |
| 30.24167 | | 352.7575 | 6.123725 | 2358.57 | 92.05469 |  |  |  |  |  |
| 30.25 | | 352.8495 | 6.12612 | 2357.789 | 91.72656 |  |  |  |  |  |
| 30.25833 | | 352.9387 | 6.125072 | 2357.008 | 91.36719 |  |  |  |  |  |
| 30.26667 | | 353.0286 | 6.122394 | 2356.227 | 91.21875 |  |  |  |  |  |
| 30.275 | | 353.1177 | 6.124759 | 2355.508 | 91.03125 |  |  |  |  |  |
| 30.28333 | | 353.2047 | 6.124627 | 2354.766 | 90.95313 |  |  |  |  |  |
| 30.29167 | | 353.2938 | 6.122049 | 2354.023 | 90.82031 |  |  |  |  |  |
| 30.3 | | 353.3829 | 6.123907 | 2353.297 | 90.95313 |  |  |  |  |  |
| 30.30833 | | 353.4714 | 6.123802 | 2352.586 | 90.75781 |  |  |  |  |  |
| 30.31667 | | 353.5576 | 6.12169 | 2351.844 | 90.60156 |  |  |  |  |  |
| 30.325 | | 353.6482 | 6.122766 | 2351.109 | 90.75 |  |  |  |  |  |
| 30.33333 | | 353.7373 | 6.122079 | 2350.359 | 90.59375 |  |  |  |  |  |
| 30.34167 | | 353.825 | 6.120517 | 2349.617 | 90.67188 |  |  |  |  |  |
| 30.35 | | 353.9127 | 6.122973 | 2348.852 | 90.67969 |  |  |  |  |  |
| 30.35833 | | 354.0018 | 6.122814 | 2348.117 | 90.54688 |  |  |  |  |  |
| 30.36667 | | 354.0917 | 6.120157 | 2347.375 | 90.49219 |  |  |  |  |  |
| 30.375 | | 354.1794 | 6.122475 | 2346.617 | 90.57031 |  |  |  |  |  |
| 30.38333 | | 354.2678 | 6.121564 | 2345.867 | 90.49219 |  |  |  |  |  |
| 30.39167 | | 354.3526 | 6.120113 | 2345.102 | 90.48438 |  |  |  |  |  |
| 30.4 | | 354.4424 | 6.121901 | 2344.352 | 90.5625 |  |  |  |  |  |
| 30.40833 | | 354.5323 | 6.121879 | 2343.609 | 90.32031 |  |  |  |  |  |
| 30.41667 | | 354.62 | 6.120174 | 2342.867 | 90.32031 |  |  |  |  |  |
| 30.425 | | 354.7077 | 6.121029 | 2342.094 | 90.23438 |  |  |  |  |  |
| 30.43333 | | 354.7954 | 6.121114 | 2341.359 | 90.14844 |  |  |  |  |  |
| 30.44167 | | 354.8823 | 6.119152 | 2340.602 | 90.07031 |  |  |  |  |  |
| 30.45 | | 354.9715 | 6.12103 | 2339.844 | 90.00781 |  |  |  |  |  |
| 30.45833 | | 355.0599 | 6.120566 | 2339.125 | 89.70313 |  |  |  |  |  |
| 30.46667 | | 355.1476 | 6.119143 | 2338.375 | 89.66406 |  |  |  |  |  |
| 30.475 | | 355.2382 | 6.120351 | 2337.633 | 89.52344 |  |  |  |  |  |
| 30.48333 | | 355.3295 | 6.119945 | 2336.891 | 89.39063 |  |  |  |  |  |
| 30.49167 | | 355.415 | 6.1183 | 2336.156 | 89.17188 |  |  |  |  |  |
| 30.5 | | 355.5056 | 6.120055 | 2335.414 | 88.74219 |  |  |  |  |  |
| 30.50833 | | 355.5948 | 6.119182 | 2334.711 | 88.53125 |  |  |  |  |  |
| 30.51667 | | 355.6854 | 6.117389 | 2333.961 | 88.04688 |  |  |  |  |  |
| 30.525 | | 355.776 | 6.119757 | 2333.234 | 87.85156 |  |  |  |  |  |
| 30.53333 | | 355.8651 | 6.118809 | 2332.508 | 87.85938 |  |  |  |  |  |
| 30.54167 | | 355.9535 | 6.117342 | 2331.789 | 87.6875 |  |  |  |  |  |
| 30.55 | | 356.0427 | 6.119772 | 2331.094 | 87.47656 |  |  |  |  |  |
| 30.55833 | | 356.1318 | 6.119273 | 2330.391 | 87.45313 |  |  |  |  |  |
| 30.56667 | | 356.218 | 6.118004 | 2329.703 | 87.50781 |  |  |  |  |  |
| 30.575 | | 356.3086 | 6.119838 | 2329 | 87.61719 |  |  |  |  |  |
| 30.58333 | | 356.3949 | 6.11989 | 2328.258 | 87.58594 |  |  |  |  |  |
| 30.59167 | | 356.4876 | 6.117928 | 2327.539 | 87.5625 |  |  |  |  |  |
| 30.6 | | 356.5784 | 6.119054 | 2326.836 | 87.64844 |  |  |  |  |  |
| 30.60833 | | 356.67 | 6.118353 | 2326.109 | 87.66406 |  |  |  |  |  |
| 30.61667 | | 356.7615 | 6.115996 | 2325.359 | 87.46094 |  |  |  |  |  |
| 30.625 | | 356.8516 | 6.117349 | 2324.617 | 87.38281 |  |  |  |  |  |
| 30.63333 | | 356.9432 | 6.115957 | 2323.891 | 87.1875 |  |  |  |  |  |
| 30.64167 | | 357.0325 | 6.114362 | 2323.164 | 86.90625 |  |  |  |  |  |
| 30.65 | | 357.1204 | 6.115362 | 2322.422 | 86.57813 |  |  |  |  |  |
| 30.65833 | | 357.2097 | 6.114036 | 2321.688 | 86.375 |  |  |  |  |  |
| 30.66667 | | 357.2998 | 6.112118 | 2320.992 | 86.45313 |  |  |  |  |  |
| 30.675 | | 357.3877 | 6.113422 | 2320.273 | 86.34375 |  |  |  |  |  |
| 30.68333 | | 357.4748 | 6.113821 | 2319.57 | 86.375 |  |  |  |  |  |
| 30.69167 | | 357.5641 | 6.111872 | 2318.883 | 86.49219 |  |  |  |  |  |
| 30.7 | | 357.652 | 6.113729 | 2318.203 | 86.28906 |  |  |  |  |  |
| 30.70833 | | 357.7443 | 6.113668 | 2317.508 | 86.30469 |  |  |  |  |  |
| 30.71667 | | 357.8344 | 6.112031 | 2316.773 | 86.57813 |  |  |  |  |  |
| 30.725 | | 357.923 | 6.114379 | 2316.063 | 86.74219 |  |  |  |  |  |
| 30.73333 | | 358.0145 | 6.114078 | 2315.344 | 87.01563 |  |  |  |  |  |
| 30.74167 | | 358.1053 | 6.113445 | 2314.602 | 86.96094 |  |  |  |  |  |
| 30.75 | | 358.1947 | 6.114877 | 2313.898 | 86.89063 |  |  |  |  |  |
| 30.75833 | | 358.2848 | 6.114899 | 2313.172 | 86.98438 |  |  |  |  |  |
| 30.76667 | | 358.3734 | 6.113138 | 2312.414 | 86.98438 |  |  |  |  |  |
| 30.775 | | 358.462 | 6.11466 | 2311.656 | 86.96094 |  |  |  |  |  |
| 30.78333 | | 358.5528 | 6.113554 | 2310.898 | 87.17188 |  |  |  |  |  |
| 30.79167 | | 358.6399 | 6.11199 | 2310.172 | 87.24219 |  |  |  |  |  |
| 30.8 | | 358.7263 | 6.1132 | 2309.453 | 87.14844 |  |  |  |  |  |
| 30.80833 | | 358.8164 | 6.112869 | 2308.719 | 87.14063 |  |  |  |  |  |
| 30.81667 | | 358.9072 | 6.111762 | 2307.992 | 86.99219 |  |  |  |  |  |
| 30.825 | | 358.9936 | 6.113331 | 2307.273 | 87.10156 |  |  |  |  |  |
| 30.83333 | | 359.0822 | 6.113629 | 2306.508 | 87.04688 |  |  |  |  |  |
| 30.84167 | | 359.1715 | 6.112196 | 2305.766 | 86.89844 |  |  |  |  |  |
| 30.85 | | 359.2638 | 6.114468 | 2305.063 | 86.96875 |  |  |  |  |  |
| 30.85833 | | 359.3524 | 6.114787 | 2304.336 | 86.98438 |  |  |  |  |  |
| 30.86667 | | 359.4403 | 6.113844 | 2303.625 | 86.76563 |  |  |  |  |  |
| 30.875 | | 359.5304 | 6.115345 | 2302.883 | 86.46875 |  |  |  |  |  |
| 30.88333 | | 359.6212 | 6.116188 | 2302.172 | 86.32031 |  |  |  |  |  |
| 30.89167 | | 359.7127 | 6.114574 | 2301.461 | 85.96094 |  |  |  |  |  |
| 30.9 | | 359.8013 | 6.116838 | 2300.734 | 85.84375 |  |  |  |  |  |
| 30.90833 | | 359.8914 | 6.1161 | 2300.008 | 85.69531 |  |  |  |  |  |
| 30.91667 | | 359.983 | 6.115105 | 2299.328 | 85.71875 |  |  |  |  |  |
| 30.925 | | 360.0731 | 6.116546 | 2298.641 | 85.875 |  |  |  |  |  |
| 30.93333 | | 360.1617 | 6.115801 | 2297.945 | 85.875 |  |  |  |  |  |
| 30.94167 | | 360.2525 | 6.113903 | 2297.281 | 85.84375 |  |  |  |  |  |
| 30.95 | | 360.3433 | 6.115564 | 2296.578 | 85.89844 |  |  |  |  |  |
| 30.95833 | | 360.4341 | 6.115361 | 2295.883 | 85.92188 |  |  |  |  |  |
| 30.96667 | | 360.5264 | 6.113563 | 2295.164 | 86.07813 |  |  |  |  |  |
| 30.975 | | 360.6157 | 6.115579 | 2294.43 | 86.33594 |  |  |  |  |  |
| 30.98333 | | 360.7051 | 6.115362 | 2293.703 | 86.44531 |  |  |  |  |  |
| 30.99167 | | 360.7974 | 6.113752 | 2292.992 | 86.60938 |  |  |  |  |  |
| 31 | | 360.8882 | 6.115826 | 2292.266 | 86.55469 |  |  |  |  |  |
| 31.00833 | | 360.9805 | 6.11594 | 2291.547 | 86.38281 |  |  |  |  |  |
| 31.01667 | | 361.0713 | 6.11444 | 2290.805 | 86.10156 |  |  |  |  |  |
| 31.025 | | 361.1621 | 6.115799 | 2290.047 | 85.89063 |  |  |  |  |  |
| 31.03333 | | 361.2559 | 6.115837 | 2289.32 | 85.69531 |  |  |  |  |  |
| 31.04167 | | 361.346 | 6.114478 | 2288.578 | 85.625 |  |  |  |  |  |
| 31.05 | | 361.4368 | 6.11675 | 2287.867 | 85.47656 |  |  |  |  |  |
| 31.05833 | | 361.5291 | 6.11602 | 2287.172 | 85.09375 |  |  |  |  |  |
| 31.06667 | | 361.6221 | 6.115079 | 2286.492 | 84.78125 |  |  |  |  |  |
| 31.075 | | 361.7122 | 6.11669 | 2285.813 | 84.60938 |  |  |  |  |  |
| 31.08333 | | 361.7993 | 6.1151 | 2285.125 | 84.51563 |  |  |  |  |  |
| 31.09167 | | 361.8842 | 6.114171 | 2284.414 | 84.52344 |  |  |  |  |  |
| 31.1 | | 361.9713 | 6.115707 | 2283.734 | 84.78125 |  |  |  |  |  |
| 31.10833 | | 362.0629 | 6.1145 | 2283.063 | 84.78906 |  |  |  |  |  |
| 31.11667 | | 362.1486 | 6.113254 | 2282.398 | 84.70313 |  |  |  |  |  |
| 31.125 | | 362.2364 | 6.115228 | 2281.703 | 84.4375 |  |  |  |  |  |
| 31.13333 | | 362.3221 | 6.115131 | 2281.016 | 84.32813 |  |  |  |  |  |
| 31.14167 | | 362.4107 | 6.112588 | 2280.305 | 84.32031 |  |  |  |  |  |
| 31.15 | | 362.4985 | 6.11499 | 2279.563 | 84.28125 |  |  |  |  |  |
| 31.15833 | | 362.5879 | 6.115505 | 2278.852 | 84.17188 |  |  |  |  |  |
| 31.16667 | | 362.6794 | 6.113349 | 2278.156 | 84.39844 |  |  |  |  |  |
| 31.175 | | 362.7717 | 6.115031 | 2277.484 | 84.23438 |  |  |  |  |  |
| 31.18333 | | 362.8655 | 6.115349 | 2276.789 | 84.02344 |  |  |  |  |  |
| 31.19167 | | 362.9548 | 6.113881 | 2276.078 | 84.125 |  |  |  |  |  |
| 31.2 | | 363.0442 | 6.116413 | 2275.383 | 84.05469 |  |  |  |  |  |
| 31.20833 | | 363.135 | 6.116089 | 2274.695 | 84.21094 |  |  |  |  |  |
| 31.21667 | | 363.2273 | 6.114067 | 2273.969 | 84.10938 |  |  |  |  |  |
| 31.225 | | 363.3174 | 6.115704 | 2273.281 | 84.11719 |  |  |  |  |  |
| 31.23333 | | 363.4074 | 6.11457 | 2272.609 | 84.17188 |  |  |  |  |  |
| 31.24167 | | 363.4968 | 6.112278 | 2271.891 | 84.03125 |  |  |  |  |  |
| 31.25 | | 363.5861 | 6.113869 | 2271.203 | 83.90625 |  |  |  |  |  |
| 31.25833 | | 363.6747 | 6.112477 | 2270.469 | 83.625 |  |  |  |  |  |
| 31.26667 | | 363.7641 | 6.110376 | 2269.789 | 83.625 |  |  |  |  |  |
| 31.275 | | 363.8527 | 6.112486 | 2269.078 | 83.38281 |  |  |  |  |  |
| 31.28333 | | 363.9442 | 6.111328 | 2268.383 | 83.32813 |  |  |  |  |  |
| 31.29167 | | 364.0358 | 6.108837 | 2267.695 | 83.22656 |  |  |  |  |  |
| 31.3 | | 364.1273 | 6.110808 | 2267.023 | 83.26563 |  |  |  |  |  |
| 31.30833 | | 364.2196 | 6.109984 | 2266.352 | 83.03125 |  |  |  |  |  |
| 31.31667 | | 364.3105 | 6.108526 | 2265.664 | 82.96094 |  |  |  |  |  |
| 31.325 | | 364.4013 | 6.110898 | 2265 | 82.76563 |  |  |  |  |  |
| 31.33333 | | 364.4936 | 6.110608 | 2264.305 | 82.57813 |  |  |  |  |  |
| 31.34167 | | 364.5865 | 6.109898 | 2263.625 | 82.52344 |  |  |  |  |  |
| 31.35 | | 364.6747 | 6.11258 | 2262.922 | 82.27344 |  |  |  |  |  |
| 31.35833 | | 364.7615 | 6.111745 | 2262.258 | 81.94531 |  |  |  |  |  |
| 31.36667 | | 364.8483 | 6.111306 | 2261.57 | 81.875 |  |  |  |  |  |
| 31.375 | | 364.9358 | 6.114094 | 2260.906 | 81.70313 |  |  |  |  |  |
| 31.38333 | | 365.0233 | 6.113038 | 2260.242 | 81.54688 |  |  |  |  |  |
| 31.39167 | | 365.1079 | 6.111605 | 2259.547 | 81.3125 |  |  |  |  |  |
| 31.4 | | 365.1962 | 6.114217 | 2258.898 | 81.23438 |  |  |  |  |  |
| 31.40833 | | 365.2873 | 6.113423 | 2258.258 | 81.53125 |  |  |  |  |  |
| 31.41667 | | 365.3748 | 6.112283 | 2257.586 | 81.63281 |  |  |  |  |  |
| 31.425 | | 365.4602 | 6.115392 | 2256.93 | 81.49219 |  |  |  |  |  |
| 31.43333 | | 365.5477 | 6.114341 | 2256.281 | 81.44531 |  |  |  |  |  |
| 31.44167 | | 365.6388 | 6.112731 | 2255.617 | 81.36719 |  |  |  |  |  |
| 31.45 | | 365.7292 | 6.114986 | 2254.953 | 81.28125 |  |  |  |  |  |
| 31.45833 | | 365.8218 | 6.113581 | 2254.227 | 81.13281 |  |  |  |  |  |
| 31.46667 | | 365.9093 | 6.112422 | 2253.539 | 81.27344 |  |  |  |  |  |
| 31.475 | | 365.9975 | 6.114819 | 2252.883 | 81.28125 |  |  |  |  |  |
| 31.48333 | | 366.0851 | 6.11303 | 2252.211 | 81.45313 |  |  |  |  |  |
| 31.49167 | | 366.1725 | 6.112234 | 2251.531 | 81.25781 |  |  |  |  |  |
| 31.5 | | 366.2608 | 6.11441 | 2250.867 | 81.10938 |  |  |  |  |  |
| 31.50833 | | 366.3512 | 6.11356 | 2250.203 | 81.14063 |  |  |  |  |  |
| 31.51667 | | 366.4416 | 6.112467 | 2249.508 | 81.375 |  |  |  |  |  |
| 31.525 | | 366.5284 | 6.114747 | 2248.828 | 81.54688 |  |  |  |  |  |
| 31.53333 | | 366.6159 | 6.113449 | 2248.125 | 81.74219 |  |  |  |  |  |
| 31.54167 | | 366.6991 | 6.112651 | 2247.461 | 82.17188 |  |  |  |  |  |
| 31.55 | | 366.7873 | 6.1165 | 2246.805 | 82.25 |  |  |  |  |  |
| 31.55833 | | 366.8763 | 6.11547 | 2246.125 | 82.58594 |  |  |  |  |  |
| 31.56667 | | 366.9638 | 6.114945 | 2245.398 | 82.78906 |  |  |  |  |  |
| 31.575 | | 367.0506 | 6.118684 | 2244.703 | 82.73438 |  |  |  |  |  |
| 31.58333 | | 367.1381 | 6.117453 | 2243.992 | 82.86719 |  |  |  |  |  |
| 31.59167 | | 367.2263 | 6.116613 | 2243.25 | 83.32031 |  |  |  |  |  |
| 31.6 | | 367.3124 | 6.120175 | 2242.547 | 83.39063 |  |  |  |  |  |
| 31.60833 | | 367.4013 | 6.118664 | 2241.805 | 83.48438 |  |  |  |  |  |
| 31.61667 | | 367.4903 | 6.118018 | 2241.094 | 83.50781 |  |  |  |  |  |
| 31.625 | | 367.58 | 6.12169 | 2240.406 | 83.36719 |  |  |  |  |  |
| 31.63333 | | 367.6675 | 6.119973 | 2239.695 | 83.39063 |  |  |  |  |  |
| 31.64167 | | 367.7557 | 6.118811 | 2238.938 | 83 |  |  |  |  |  |
| 31.65 | | 367.8454 | 6.121723 | 2238.234 | 82.72656 |  |  |  |  |  |
| 31.65833 | | 367.9336 | 6.120027 | 2237.523 | 82.71875 |  |  |  |  |  |
| 31.66667 | | 368.0219 | 6.119213 | 2236.82 | 82.73438 |  |  |  |  |  |
| 31.675 | | 368.1072 | 6.122454 | 2236.148 | 82.42969 |  |  |  |  |  |
| 31.68333 | | 368.1947 | 6.120982 | 2235.461 | 82.35938 |  |  |  |  |  |
| 31.69167 | | 368.2837 | 6.119854 | 2234.828 | 82.20313 |  |  |  |  |  |
| 31.7 | | 368.3705 | 6.123433 | 2234.172 | 82.00781 |  |  |  |  |  |
| 31.70833 | | 368.4565 | 6.122044 | 2233.484 | 81.84375 |  |  |  |  |  |
| 31.71667 | | 368.5455 | 6.122041 | 2232.797 | 81.78906 |  |  |  |  |  |
| 31.725 | | 368.633 | 6.125383 | 2232.156 | 81.70313 |  |  |  |  |  |
| 31.73333 | | 368.722 | 6.123994 | 2231.477 | 81.61719 |  |  |  |  |  |
| 31.74167 | | 368.8116 | 6.124761 | 2230.813 | 81.39063 |  |  |  |  |  |
| 31.75 | | 368.9028 | 6.128277 | 2230.164 | 81.27344 |  |  |  |  |  |
| 31.75833 | | 368.991 | 6.127338 | 2229.508 | 80.99219 |  |  |  |  |  |
| 31.76667 | | 369.0785 | 6.127121 | 2228.836 | 80.54688 |  |  |  |  |  |
| 31.775 | | 369.1682 | 6.130365 | 2228.172 | 80.49219 |  |  |  |  |  |
| 31.78333 | | 369.2572 | 6.128509 | 2227.508 | 80.34375 |  |  |  |  |  |
| 31.79167 | | 369.3483 | 6.128134 | 2226.859 | 80.32031 |  |  |  |  |  |
| 31.8 | | 369.4358 | 6.131159 | 2226.211 | 79.9375 |  |  |  |  |  |
| 31.80833 | | 369.5255 | 6.128609 | 2225.563 | 80.01563 |  |  |  |  |  |
| 31.81667 | | 369.613 | 6.128392 | 2224.953 | 80.01563 |  |  |  |  |  |
| 31.825 | | 369.6998 | 6.131482 | 2224.289 | 79.83594 |  |  |  |  |  |
| 31.83333 | | 369.7844 | 6.129508 | 2223.641 | 79.78906 |  |  |  |  |  |
| 31.84167 | | 369.8741 | 6.129024 | 2222.969 | 79.71094 |  |  |  |  |  |
| 31.85 | | 369.963 | 6.132433 | 2222.359 | 79.75781 |  |  |  |  |  |
| 31.85833 | | 370.0513 | 6.129784 | 2221.68 | 79.8125 |  |  |  |  |  |
| 31.86667 | | 370.1402 | 6.130087 | 2221.023 | 79.71094 |  |  |  |  |  |
| 31.875 | | 370.2278 | 6.132821 | 2220.375 | 79.46094 |  |  |  |  |  |
| 31.88333 | | 370.3181 | 6.130826 | 2219.711 | 79.78906 |  |  |  |  |  |
| 31.89167 | | 370.4064 | 6.131539 | 2219.063 | 79.67188 |  |  |  |  |  |
| 31.9 | | 370.4975 | 6.134486 | 2218.391 | 79.77344 |  |  |  |  |  |
| 31.90833 | | 370.5865 | 6.133049 | 2217.719 | 79.64844 |  |  |  |  |  |
| 31.91667 | | 370.6783 | 6.13387 | 2217.055 | 79.5625 |  |  |  |  |  |
| 31.925 | | 370.768 | 6.13667 | 2216.43 | 79.57813 |  |  |  |  |  |
| 31.93333 | | 370.8584 | 6.134469 | 2215.703 | 79.5 |  |  |  |  |  |
| 31.94167 | | 370.9474 | 6.1361 | 2215.047 | 79.32031 |  |  |  |  |  |
| 31.95 | | 371.0385 | 6.13948 | 2214.367 | 79.39844 |  |  |  |  |  |
| 31.95833 | | 371.1289 | 6.136876 | 2213.711 | 79.33594 |  |  |  |  |  |
| 31.96667 | | 371.2171 | 6.138774 | 2213.055 | 79.08594 |  |  |  |  |  |
| 31.975 | | 371.309 | 6.141458 | 2212.391 | 78.88281 |  |  |  |  |  |
| 31.98333 | | 371.3951 | 6.138433 | 2211.727 | 78.53906 |  |  |  |  |  |
| 31.99167 | | 371.484 | 6.139245 | 2211.078 | 78.41406 |  |  |  |  |  |
| 32 | | 371.5722 | 6.141421 | 2210.398 | 78.32813 |  |  |  |  |  |
| 32.00833 | | 371.6612 | 6.138455 | 2209.742 | 78.25 |  |  |  |  |  |
| 32.01667 | | 371.7502 | 6.139673 | 2209.117 | 78.0625 |  |  |  |  |  |
| 32.025 | | 371.8377 | 6.141945 | 2208.484 | 77.97656 |  |  |  |  |  |
| 32.03333 | | 371.9252 | 6.13925 | 2207.875 | 77.65625 |  |  |  |  |  |
| 32.04167 | | 372.0149 | 6.141065 | 2207.242 | 77.61719 |  |  |  |  |  |
| 32.05 | | 372.1017 | 6.143335 | 2206.594 | 77.60156 |  |  |  |  |  |
| 32.05833 | | 372.1877 | 6.140919 | 2205.953 | 77.59375 |  |  |  |  |  |
| 32.06667 | | 372.2759 | 6.142484 | 2205.328 | 77.79688 |  |  |  |  |  |
| 32.075 | | 372.3642 | 6.144949 | 2204.703 | 77.97656 |  |  |  |  |  |
| 32.08333 | | 372.4533 | 6.142528 | 2204.086 | 78.11719 |  |  |  |  |  |
| 32.09167 | | 372.5424 | 6.144641 | 2203.438 | 78.32813 |  |  |  |  |  |
| 32.1 | | 372.6331 | 6.147567 | 2202.789 | 78.36719 |  |  |  |  |  |
| 32.10833 | | 372.7222 | 6.145109 | 2202.148 | 78.47656 |  |  |  |  |  |
| 32.11667 | | 372.8105 | 6.147302 | 2201.469 | 78.67969 |  |  |  |  |  |
| 32.125 | | 372.8968 | 6.149571 | 2200.789 | 78.6875 |  |  |  |  |  |
| 32.13333 | | 372.9866 | 6.147222 | 2200.117 | 78.72656 |  |  |  |  |  |
| 32.14167 | | 373.0764 | 6.149242 | 2199.438 | 78.78906 |  |  |  |  |  |
| 32.15 | | 373.1663 | 6.151335 | 2198.773 | 78.98438 |  |  |  |  |  |
| 32.15833 | | 373.2569 | 6.148997 | 2198.102 | 78.9375 |  |  |  |  |  |
| 32.16667 | | 373.3445 | 6.15123 | 2197.43 | 79.03125 |  |  |  |  |  |
| 32.175 | | 373.4337 | 6.15328 | 2196.766 | 79.17969 |  |  |  |  |  |
| 32.18333 | | 373.5228 | 6.150389 | 2196.109 | 79.52344 |  |  |  |  |  |
| 32.19167 | | 373.6133 | 6.152925 | 2195.445 | 79.5625 |  |  |  |  |  |
| 32.2 | | 373.7018 | 6.154635 | 2194.766 | 79.875 |  |  |  |  |  |
| 32.20833 | | 373.7916 | 6.151801 | 2194.109 | 80.13281 |  |  |  |  |  |
| 32.21667 | | 373.8851 | 6.154389 | 2193.438 | 80.39063 |  |  |  |  |  |
| 32.225 | | 373.9757 | 6.156022 | 2192.758 | 80.58594 |  |  |  |  |  |
| 32.23333 | | 374.0677 | 6.153007 | 2192.047 | 80.39844 |  |  |  |  |  |
| 32.24167 | | 374.1568 | 6.15596 | 2191.375 | 80.24219 |  |  |  |  |  |
| 32.25 | | 374.2481 | 6.15725 | 2190.664 | 80.05469 |  |  |  |  |  |
| 32.25833 | | 374.3394 | 6.154523 | 2189.961 | 79.80469 |  |  |  |  |  |
| 32.26667 | | 374.4308 | 6.157807 | 2189.25 | 79.40625 |  |  |  |  |  |
| 32.275 | | 374.5206 | 6.158526 | 2188.563 | 79.29688 |  |  |  |  |  |
| 32.28333 | | 374.6134 | 6.156065 | 2187.906 | 78.74219 |  |  |  |  |  |
| 32.29167 | | 374.7076 | 6.159256 | 2187.266 | 78.39844 |  |  |  |  |  |
| 32.3 | | 374.796 | 6.159911 | 2186.625 | 77.92188 |  |  |  |  |  |
| 32.30833 | | 374.8859 | 6.157943 | 2186 | 77.52344 |  |  |  |  |  |
| 32.31667 | | 374.9742 | 6.161137 | 2185.391 | 77.35938 |  |  |  |  |  |
| 32.325 | | 375.0648 | 6.162092 | 2184.75 | 77.53125 |  |  |  |  |  |
| 32.33333 | | 375.1554 | 6.159715 | 2184.164 | 77.67188 |  |  |  |  |  |
| 32.34167 | | 375.2452 | 6.163224 | 2183.57 | 77.625 |  |  |  |  |  |
| 32.35 | | 375.3336 | 6.163519 | 2182.977 | 77.60938 |  |  |  |  |  |
| 32.35833 | | 375.4228 | 6.161339 | 2182.383 | 77.61719 |  |  |  |  |  |
| 32.36667 | | 375.5104 | 6.164153 | 2181.773 | 77.83594 |  |  |  |  |  |
| 32.375 | | 375.5966 | 6.163956 | 2181.094 | 78.11719 |  |  |  |  |  |
| 32.38333 | | 375.6879 | 6.161028 | 2180.438 | 78.60938 |  |  |  |  |  |
| 32.39167 | | 375.777 | 6.163955 | 2179.797 | 78.95313 |  |  |  |  |  |
| 32.4 | | 375.8669 | 6.164138 | 2179.148 | 79.17188 |  |  |  |  |  |
| 32.40833 | | 375.9597 | 6.161258 | 2178.5 | 79.17188 |  |  |  |  |  |
| 32.41667 | | 376.0481 | 6.16432 | 2177.836 | 79.59375 |  |  |  |  |  |
| 32.425 | | 376.1364 | 6.164108 | 2177.141 | 79.63281 |  |  |  |  |  |
| 32.43333 | | 376.2256 | 6.161463 | 2176.422 | 79.90625 |  |  |  |  |  |
| 32.44167 | | 376.3154 | 6.165194 | 2175.703 | 79.97656 |  |  |  |  |  |
| 32.45 | | 376.4074 | 6.16493 | 2175.023 | 80.07813 |  |  |  |  |  |
| 32.45833 | | 376.4958 | 6.162401 | 2174.359 | 80.07813 |  |  |  |  |  |
| 32.46667 | | 376.5828 | 6.165546 | 2173.641 | 79.76563 |  |  |  |  |  |
| 32.475 | | 376.6733 | 6.165737 | 2172.969 | 79.64063 |  |  |  |  |  |
| 32.48333 | | 376.7639 | 6.163126 | 2172.266 | 79.83594 |  |  |  |  |  |
| 32.49167 | | 376.8501 | 6.166297 | 2171.578 | 79.77344 |  |  |  |  |  |
| 32.5 | | 376.94 | 6.166613 | 2170.898 | 79.50781 |  |  |  |  |  |
| 32.50833 | | 377.0313 | 6.164688 | 2170.227 | 79.60938 |  |  |  |  |  |
| 32.51667 | | 377.1219 | 6.167735 | 2169.609 | 79.57813 |  |  |  |  |  |
| 32.525 | | 377.2095 | 6.167211 | 2168.961 | 79.39844 |  |  |  |  |  |
| 32.53333 | | 377.2964 | 6.165308 | 2168.273 | 79.32813 |  |  |  |  |  |
| 32.54167 | | 377.3856 | 6.168662 | 2167.617 | 79.42969 |  |  |  |  |  |
| 32.55 | | 377.4762 | 6.168729 | 2166.992 | 79.46875 |  |  |  |  |  |
| 32.55833 | | 377.5653 | 6.167095 | 2166.313 | 79.71875 |  |  |  |  |  |
| 32.56667 | | 377.6537 | 6.170845 | 2165.656 | 79.64063 |  |  |  |  |  |
| 32.575 | | 377.742 | 6.170968 | 2165.016 | 79.75 |  |  |  |  |  |
| 32.58333 | | 377.8312 | 6.169027 | 2164.359 | 79.875 |  |  |  |  |  |
| 32.59167 | | 377.921 | 6.172805 | 2163.68 | 79.8125 |  |  |  |  |  |
| 32.6 | | 378.0094 | 6.17173 | 2163.008 | 79.85938 |  |  |  |  |  |
| 32.60833 | | 378.1 | 6.170333 | 2162.305 | 80.28125 |  |  |  |  |  |
| 32.61667 | | 378.192 | 6.174528 | 2161.648 | 80.38281 |  |  |  |  |  |
| 32.625 | | 378.2833 | 6.173599 | 2160.961 | 80.13281 |  |  |  |  |  |
| 32.63333 | | 378.3739 | 6.17257 | 2160.289 | 80.23438 |  |  |  |  |  |
| 32.64167 | | 378.4623 | 6.176266 | 2159.625 | 79.88281 |  |  |  |  |  |
| 32.65 | | 378.5507 | 6.175291 | 2158.953 | 79.625 |  |  |  |  |  |
| 32.65833 | | 378.6413 | 6.173501 | 2158.234 | 79.28125 |  |  |  |  |  |
| 32.66667 | | 378.7333 | 6.177695 | 2157.563 | 79.04688 |  |  |  |  |  |
| 32.675 | | 378.8203 | 6.176381 | 2156.922 | 78.89844 |  |  |  |  |  |
| 32.68333 | | 378.9108 | 6.175002 | 2156.258 | 78.61719 |  |  |  |  |  |
| 32.69167 | | 379.0029 | 6.178812 | 2155.641 | 78.15625 |  |  |  |  |  |
| 32.7 | | 379.0942 | 6.177247 | 2155.031 | 77.97656 |  |  |  |  |  |
| 32.70833 | | 379.1855 | 6.176021 | 2154.43 | 77.95313 |  |  |  |  |  |
| 32.71667 | | 379.2775 | 6.17999 | 2153.805 | 77.99219 |  |  |  |  |  |
| 32.725 | | 379.3674 | 6.178705 | 2153.164 | 78.08594 |  |  |  |  |  |
| 32.73333 | | 379.4616 | 6.177945 | 2152.563 | 78.32031 |  |  |  |  |  |
| 32.74167 | | 379.5522 | 6.181831 | 2151.977 | 78.53906 |  |  |  |  |  |
| 32.75 | | 379.6435 | 6.180309 | 2151.344 | 78.57031 |  |  |  |  |  |
| 32.75833 | | 379.7326 | 6.180127 | 2150.695 | 78.49219 |  |  |  |  |  |
| 32.76667 | | 379.8225 | 6.183947 | 2150.039 | 78.60938 |  |  |  |  |  |
| 32.775 | | 379.9131 | 6.182859 | 2149.367 | 78.5625 |  |  |  |  |  |
| 32.78333 | | 380.0029 | 6.183357 | 2148.68 | 78.47656 |  |  |  |  |  |
| 32.79167 | | 380.0927 | 6.187673 | 2147.984 | 78.32031 |  |  |  |  |  |
| 32.8 | | 380.1819 | 6.186676 | 2147.313 | 78.22656 |  |  |  |  |  |
| 32.80833 | | 380.2746 | 6.186668 | 2146.672 | 78.14063 |  |  |  |  |  |
| 32.81667 | | 380.3652 | 6.190674 | 2146 | 78.03906 |  |  |  |  |  |
| 32.825 | | 380.457 | 6.188714 | 2145.344 | 78 |  |  |  |  |  |
| 32.83333 | | 380.5421 | 6.18833 | 2144.695 | 77.83594 |  |  |  |  |  |
| 32.84167 | | 380.6338 | 6.191976 | 2144.063 | 77.9375 |  |  |  |  |  |
| 32.85 | | 380.7254 | 6.189778 | 2143.414 | 78.24219 |  |  |  |  |  |
| 32.85833 | | 380.8112 | 6.189552 | 2142.781 | 78.49219 |  |  |  |  |  |
| 32.86667 | | 380.8978 | 6.19236 | 2142.141 | 78.5 |  |  |  |  |  |
| 32.875 | | 380.9858 | 6.190382 | 2141.5 | 78.60156 |  |  |  |  |  |
| 32.88333 | | 381.0724 | 6.190203 | 2140.867 | 78.54688 |  |  |  |  |  |
| 32.89167 | | 381.1619 | 6.193452 | 2140.211 | 78.72656 |  |  |  |  |  |
| 32.9 | | 381.2484 | 6.191216 | 2139.516 | 78.86719 |  |  |  |  |  |
| 32.90833 | | 381.3364 | 6.190677 | 2138.82 | 78.80469 |  |  |  |  |  |
| 32.91667 | | 381.4281 | 6.193915 | 2138.164 | 78.90625 |  |  |  |  |  |
| 32.925 | | 381.5175 | 6.191326 | 2137.5 | 78.78125 |  |  |  |  |  |
| 32.93333 | | 381.6026 | 6.19189 | 2136.844 | 78.64844 |  |  |  |  |  |
| 32.94167 | | 381.6928 | 6.196276 | 2136.164 | 78.54688 |  |  |  |  |  |
| 32.95 | | 381.783 | 6.194583 | 2135.492 | 78.59375 |  |  |  |  |  |
| 32.95833 | | 381.8739 | 6.195299 | 2134.828 | 78.50781 |  |  |  |  |  |
| 32.96667 | | 381.9633 | 6.198698 | 2134.164 | 78.4375 |  |  |  |  |  |
| 32.975 | | 382.0514 | 6.196421 | 2133.516 | 78.53906 |  |  |  |  |  |
| 32.98333 | | 382.1401 | 6.197003 | 2132.875 | 78.53906 |  |  |  |  |  |
| 32.99167 | | 382.2281 | 6.201181 | 2132.234 | 78.47656 |  |  |  |  |  |
| 33 | | 382.3183 | 6.199571 | 2131.578 | 78.52344 |  |  |  |  |  |
| 33.00833 | | 382.407 | 6.201683 | 2130.922 | 78.625 |  |  |  |  |  |
| 33.01667 | | 382.4965 | 6.204957 | 2130.273 | 78.5 |  |  |  |  |  |
| 33.025 | | 382.5867 | 6.203308 | 2129.609 | 78.47656 |  |  |  |  |  |
| 33.03333 | | 382.6754 | 6.204839 | 2128.945 | 78.44531 |  |  |  |  |  |
| 33.04167 | | 382.7634 | 6.207418 | 2128.313 | 78.39063 |  |  |  |  |  |
| 33.05 | | 382.8529 | 6.205515 | 2127.664 | 78.41406 |  |  |  |  |  |
| 33.05833 | | 382.9402 | 6.206829 | 2127 | 78.08594 |  |  |  |  |  |
| 33.06667 | | 383.0303 | 6.209217 | 2126.367 | 77.74219 |  |  |  |  |  |
| 33.075 | | 383.1205 | 6.207676 | 2125.711 | 77.53125 |  |  |  |  |  |
| 33.08333 | | 383.2085 | 6.209682 | 2125.07 | 77.4375 |  |  |  |  |  |
| 33.09167 | | 383.2987 | 6.211985 | 2124.43 | 77.39844 |  |  |  |  |  |
| 33.1 | | 383.3903 | 6.210335 | 2123.781 | 77.29688 |  |  |  |  |  |
| 33.10833 | | 383.4798 | 6.212284 | 2123.18 | 77.22656 |  |  |  |  |  |
| 33.11667 | | 383.5692 | 6.213826 | 2122.578 | 77.125 |  |  |  |  |  |
| 33.125 | | 383.6594 | 6.21224 | 2121.953 | 77.17969 |  |  |  |  |  |
| 33.13333 | | 383.751 | 6.214364 | 2121.32 | 77.36719 |  |  |  |  |  |
| 33.14167 | | 383.8412 | 6.215908 | 2120.672 | 77.39844 |  |  |  |  |  |
| 33.15 | | 383.9314 | 6.215304 | 2120.047 | 77.59375 |  |  |  |  |  |
| 33.15833 | | 384.0208 | 6.218161 | 2119.406 | 77.79688 |  |  |  |  |  |
| 33.16667 | | 384.111 | 6.219205 | 2118.773 | 77.94531 |  |  |  |  |  |
| 33.175 | | 384.199 | 6.218332 | 2118.117 | 77.92969 |  |  |  |  |  |
| 33.18333 | | 384.2885 | 6.22062 | 2117.445 | 78.4375 |  |  |  |  |  |
| 33.19167 | | 384.3758 | 6.221473 | 2116.805 | 78.72656 |  |  |  |  |  |
| 33.2 | | 384.4653 | 6.220114 | 2116.125 | 78.90625 |  |  |  |  |  |
| 33.20833 | | 384.5526 | 6.222057 | 2115.453 | 79.00781 |  |  |  |  |  |
| 33.21667 | | 384.6398 | 6.223624 | 2114.781 | 78.92969 |  |  |  |  |  |
| 33.225 | | 384.73 | 6.221395 | 2114.133 | 79.15625 |  |  |  |  |  |
| 33.23333 | | 384.8166 | 6.22287 | 2113.406 | 79.45313 |  |  |  |  |  |
| 33.24167 | | 384.906 | 6.223954 | 2112.719 | 79.49219 |  |  |  |  |  |
| 33.25 | | 384.994 | 6.221387 | 2112.023 | 79.59375 |  |  |  |  |  |
| 33.25833 | | 385.0828 | 6.223348 | 2111.352 | 79.71875 |  |  |  |  |  |
| 33.26667 | | 385.1701 | 6.224875 | 2110.703 | 79.375 |  |  |  |  |  |
| 33.275 | | 385.2603 | 6.222061 | 2110.008 | 79.20313 |  |  |  |  |  |
| 33.28333 | | 385.3483 | 6.223845 | 2109.313 | 79.14063 |  |  |  |  |  |
| 33.29167 | | 385.4406 | 6.225623 | 2108.633 | 79.14844 |  |  |  |  |  |
| 33.3 | | 385.5315 | 6.223418 | 2107.953 | 79.47656 |  |  |  |  |  |
| 33.30833 | | 385.6224 | 6.226302 | 2107.281 | 79.48438 |  |  |  |  |  |
| 33.31667 | | 385.7141 | 6.227599 | 2106.664 | 79.42969 |  |  |  |  |  |
| 33.325 | | 385.8035 | 6.226549 | 2106.031 | 79.52344 |  |  |  |  |  |
| 33.33333 | | 385.8944 | 6.229348 | 2105.383 | 79.67188 |  |  |  |  |  |
| 33.34167 | | 385.9853 | 6.229506 | 2104.727 | 79.92969 |  |  |  |  |  |
| 33.35 | | 386.0762 | 6.22907 | 2104.016 | 79.74219 |  |  |  |  |  |
| 33.35833 | | 386.1678 | 6.231496 | 2103.352 | 79.6875 |  |  |  |  |  |
| 33.36667 | | 386.2609 | 6.230913 | 2102.703 | 79.5625 |  |  |  |  |  |
| 33.375 | | 386.3496 | 6.231486 | 2102.023 | 79.53125 |  |  |  |  |  |
| 33.38333 | | 386.4369 | 6.234188 | 2101.328 | 79.07031 |  |  |  |  |  |
| 33.39167 | | 386.5249 | 6.233818 | 2100.641 | 78.83594 |  |  |  |  |  |
| 33.4 | | 386.6129 | 6.23473 | 2099.992 | 78.53906 |  |  |  |  |  |
| 33.40833 | | 386.7002 | 6.237768 | 2099.344 | 78.3125 |  |  |  |  |  |
| 33.41667 | | 386.7868 | 6.237162 | 2098.703 | 77.95313 |  |  |  |  |  |
| 33.425 | | 386.8741 | 6.237393 | 2098.055 | 77.60156 |  |  |  |  |  |
| 33.43333 | | 386.9621 | 6.240126 | 2097.461 | 77.57813 |  |  |  |  |  |
| 33.44167 | | 387.0501 | 6.24018 | 2096.828 | 77.46094 |  |  |  |  |  |
| 33.45 | | 387.136 | 6.240546 | 2096.219 | 77.59375 |  |  |  |  |  |
| 33.45833 | | 387.2225 | 6.243355 | 2095.602 | 77.60156 |  |  |  |  |  |
| 33.46667 | | 387.312 | 6.243637 | 2095.016 | 77.64844 |  |  |  |  |  |
| 33.475 | | 387.4 | 6.2437 | 2094.406 | 77.60156 |  |  |  |  |  |
| 33.48333 | | 387.4895 | 6.246655 | 2093.781 | 77.70313 |  |  |  |  |  |
| 33.49167 | | 387.5789 | 6.246455 | 2093.148 | 77.69531 |  |  |  |  |  |
| 33.5 | | 387.6691 | 6.246016 | 2092.492 | 77.75781 |  |  |  |  |  |
| 33.50833 | | 387.76 | 6.248521 | 2091.836 | 77.53906 |  |  |  |  |  |
| 33.51667 | | 387.8509 | 6.24804 | 2091.188 | 77.78125 |  |  |  |  |  |
| 33.525 | | 387.9389 | 6.247448 | 2090.539 | 77.82031 |  |  |  |  |  |
| 33.53333 | | 388.0284 | 6.250355 | 2089.883 | 77.65625 |  |  |  |  |  |
| 33.54167 | | 388.1207 | 6.250329 | 2089.234 | 77.41406 |  |  |  |  |  |
| 33.55 | | 388.2102 | 6.250484 | 2088.578 | 77.42188 |  |  |  |  |  |
| 33.55833 | | 388.3004 | 6.252846 | 2087.953 | 77.34375 |  |  |  |  |  |
| 33.56667 | | 388.3884 | 6.251778 | 2087.266 | 77.26563 |  |  |  |  |  |
| 33.575 | | 388.48 | 6.251952 | 2086.617 | 77.17969 |  |  |  |  |  |
| 33.58333 | | 388.5702 | 6.25463 | 2085.977 | 77.09375 |  |  |  |  |  |
| 33.59167 | | 388.6596 | 6.25428 | 2085.359 | 77.17969 |  |  |  |  |  |
| 33.6 | | 388.7476 | 6.255222 | 2084.719 | 77.10938 |  |  |  |  |  |
| 33.60833 | | 388.8393 | 6.258412 | 2084.078 | 77.10938 |  |  |  |  |  |
| 33.61667 | | 388.9294 | 6.258007 | 2083.438 | 77.17188 |  |  |  |  |  |
| 33.625 | | 389.0175 | 6.258417 | 2082.805 | 77.3125 |  |  |  |  |  |
| 33.63333 | | 389.1076 | 6.260698 | 2082.164 | 77.53125 |  |  |  |  |  |
| 33.64167 | | 389.1971 | 6.259691 | 2081.516 | 77.92188 |  |  |  |  |  |
| 33.65 | | 389.288 | 6.260174 | 2080.867 | 78.02344 |  |  |  |  |  |
| 33.65833 | | 389.3781 | 6.262193 | 2080.227 | 78.24219 |  |  |  |  |  |
| 33.66667 | | 389.4683 | 6.261201 | 2079.57 | 78.66406 |  |  |  |  |  |
| 33.675 | | 389.5571 | 6.262671 | 2078.914 | 79.02344 |  |  |  |  |  |
| 33.68333 | | 389.6465 | 6.265488 | 2078.234 | 79.19531 |  |  |  |  |  |
| 33.69167 | | 389.7324 | 6.264807 | 2077.531 | 79.36719 |  |  |  |  |  |
| 33.7 | | 389.8233 | 6.265947 | 2076.852 | 79.71094 |  |  |  |  |  |
| 33.70833 | | 389.9164 | 6.268692 | 2076.164 | 79.82813 |  |  |  |  |  |
| 33.71667 | | 390.0051 | 6.268047 | 2075.461 | 80.03906 |  |  |  |  |  |
| 33.725 | | 390.0938 | 6.2697 | 2074.742 | 79.9375 |  |  |  |  |  |
| 33.73333 | | 390.1847 | 6.271805 | 2074.055 | 80.08594 |  |  |  |  |  |
| 33.74167 | | 390.2727 | 6.270933 | 2073.359 | 80.24219 |  |  |  |  |  |
| 33.75 | | 390.3615 | 6.272413 | 2072.656 | 80.3125 |  |  |  |  |  |
| 33.75833 | | 390.4509 | 6.274268 | 2071.969 | 80.29688 |  |  |  |  |  |
| 33.76667 | | 390.5432 | 6.272973 | 2071.281 | 80.39844 |  |  |  |  |  |
| 33.775 | | 390.6392 | 6.274572 | 2070.625 | 80.82031 |  |  |  |  |  |
| 33.78333 | | 390.7272 | 6.275829 | 2069.953 | 80.76563 |  |  |  |  |  |
| 33.79167 | | 390.8138 | 6.273908 | 2069.266 | 80.89063 |  |  |  |  |  |
| 33.8 | | 390.904 | 6.274778 | 2068.586 | 80.71875 |  |  |  |  |  |
| 33.80833 | | 390.9977 | 6.276208 | 2067.93 | 80.85156 |  |  |  |  |  |
| 33.81667 | | 391.0887 | 6.275076 | 2067.25 | 80.92969 |  |  |  |  |  |
| 33.825 | | 391.1774 | 6.277292 | 2066.539 | 80.8125 |  |  |  |  |  |
| 33.83333 | | 391.2661 | 6.279184 | 2065.875 | 80.69531 |  |  |  |  |  |
| 33.84167 | | 391.357 | 6.278452 | 2065.188 | 80.57813 |  |  |  |  |  |
| 33.85 | | 391.4436 | 6.281345 | 2064.531 | 80.64063 |  |  |  |  |  |
| 33.85833 | | 391.5475 | 6.283084 | 2063.695 | 80.35156 |  |  |  |  |  |
| 33.86667 | | 391.6182 | 6.282521 | 2063.164 | 80.29688 |  |  |  |  |  |
| 33.875 | | 391.7069 | 6.286074 | 2062.508 | 80.17188 |  |  |  |  |  |
| 33.88333 | | 391.7985 | 6.287961 | 2061.852 | 80.17188 |  |  |  |  |  |
| 33.89167 | | 391.8865 | 6.287688 | 2061.203 | 80.30469 |  |  |  |  |  |
| 33.9 | | 391.9746 | 6.291283 | 2060.531 | 80.21094 |  |  |  |  |  |
| 33.90833 | | 392.0647 | 6.292769 | 2059.898 | 80.36719 |  |  |  |  |  |
| 33.91667 | | 392.1729 | 6.292057 | 2059.109 | 80.375 |  |  |  |  |  |
| 33.925 | | 392.2617 | 6.296231 | 2058.438 | 80.42188 |  |  |  |  |  |
| 33.93333 | | 392.3519 | 6.296277 | 2057.789 | 80.32813 |  |  |  |  |  |
| 33.94167 | | 392.4226 | 6.295447 | 2057.25 | 80.38281 |  |  |  |  |  |
| 33.95 | | 392.5315 | 6.299643 | 2056.461 | 80.58594 |  |  |  |  |  |
| 33.95833 | | 392.6231 | 6.299752 | 2055.773 | 80.60938 |  |  |  |  |  |
| 33.96667 | | 392.7133 | 6.299618 | 2055.109 | 80.73438 |  |  |  |  |  |
| 33.975 | | 392.784 | 6.303688 | 2054.57 | 80.67188 |  |  |  |  |  |
| 33.98333 | | 392.8886 | 6.305085 | 2053.773 | 80.98438 |  |  |  |  |  |
| 33.99167 | | 392.9781 | 6.304935 | 2053.094 | 80.85156 |  |  |  |  |  |
| 34 | | 393.0697 | 6.309449 | 2052.406 | 81.02344 |  |  |  |  |  |
| 34.00833 | | 393.1606 | 6.309111 | 2051.734 | 81.15625 |  |  |  |  |  |
| 34.01667 | | 393.2493 | 6.308844 | 2051.047 | 81.22656 |  |  |  |  |  |
| 34.025 | | 393.3236 | 6.313097 | 2050.516 | 81.09375 |  |  |  |  |  |
| 34.03333 | | 393.4167 | 6.313351 | 2049.797 | 81.01563 |  |  |  |  |  |
| 34.04167 | | 393.522 | 6.313298 | 2049 | 80.89063 |  |  |  |  |  |
| 34.05 | | 393.5934 | 6.317361 | 2048.43 | 80.67969 |  |  |  |  |  |
| 34.05833 | | 393.6988 | 6.317163 | 2047.602 | 80.42969 |  |  |  |  |  |
| 34.06667 | | 393.7925 | 6.31715 | 2046.914 | 80.00781 |  |  |  |  |  |
| 34.075 | | 393.8654 | 6.321257 | 2046.383 | 79.77344 |  |  |  |  |  |
| 34.08333 | | 393.9527 | 6.321333 | 2045.727 | 79.58594 |  |  |  |  |  |
| 34.09167 | | 394.0407 | 6.321236 | 2045.055 | 79.21875 |  |  |  |  |  |
| 34.1 | | 394.1309 | 6.325137 | 2044.414 | 78.82813 |  |  |  |  |  |
| 34.10833 | | 394.2369 | 6.32414 | 2043.641 | 78.4375 |  |  |  |  |  |
| 34.11667 | | 394.3084 | 6.324458 | 2043.18 | 78.48438 |  |  |  |  |  |
| 34.125 | | 394.3986 | 6.328305 | 2042.531 | 78.46875 |  |  |  |  |  |
| 34.13333 | | 394.5031 | 6.327712 | 2041.789 | 78.25781 |  |  |  |  |  |
| 34.14167 | | 394.5768 | 6.328109 | 2041.297 | 78.29688 |  |  |  |  |  |
| 34.15 | | 394.6676 | 6.331969 | 2040.68 | 78.29688 |  |  |  |  |  |
| 34.15833 | | 394.7571 | 6.331964 | 2040.063 | 78.25781 |  |  |  |  |  |
| 34.16667 | | 394.8466 | 6.332098 | 2039.422 | 78.17188 |  |  |  |  |  |
| 34.175 | | 394.9367 | 6.336333 | 2038.773 | 78.23438 |  |  |  |  |  |
| 34.18333 | | 395.0471 | 6.336118 | 2038.008 | 78.70313 |  |  |  |  |  |
| 34.19167 | | 395.1185 | 6.337048 | 2037.477 | 78.9375 |  |  |  |  |  |
| 34.2 | | 395.2058 | 6.341065 | 2036.828 | 79.10156 |  |  |  |  |  |
| 34.20833 | | 395.296 | 6.340322 | 2036.18 | 78.97656 |  |  |  |  |  |
| 34.21667 | | 395.3912 | 6.341029 | 2035.531 | 79.17969 |  |  |  |  |  |
| 34.225 | | 395.4821 | 6.34517 | 2034.859 | 79.53906 |  |  |  |  |  |
| 34.23333 | | 395.5889 | 6.343809 | 2034.016 | 80.11719 |  |  |  |  |  |
| 34.24167 | | 395.6589 | 6.344503 | 2033.461 | 80.17969 |  |  |  |  |  |
| 34.25 | | 395.7512 | 6.348655 | 2032.773 | 80.59375 |  |  |  |  |  |
| 34.25833 | | 395.8609 | 6.346706 | 2032 | 80.76563 |  |  |  |  |  |
| 34.26667 | | 395.9482 | 6.348268 | 2031.313 | 81.03906 |  |  |  |  |  |
| 34.275 | | 396.0196 | 6.352098 | 2030.727 | 81.10938 |  |  |  |  |  |
| 34.28333 | | 396.1292 | 6.350675 | 2029.859 | 81.19531 |  |  |  |  |  |
| 34.29167 | | 396.2194 | 6.352987 | 2029.164 | 81.52344 |  |  |  |  |  |
| 34.3 | | 396.3067 | 6.356286 | 2028.438 | 81.58594 |  |  |  |  |  |
| 34.30833 | | 396.3922 | 6.354903 | 2027.742 | 81.38281 |  |  |  |  |  |
| 34.31667 | | 396.479 | 6.356986 | 2027.031 | 81.39063 |  |  |  |  |  |
| 34.325 | | 396.5702 | 6.36019 | 2026.336 | 81.30469 |  |  |  |  |  |
| 34.33333 | | 396.64 | 6.358485 | 2025.797 | 81.04688 |  |  |  |  |  |
| 34.34167 | | 396.7276 | 6.360489 | 2025.078 | 81.03906 |  |  |  |  |  |
| 34.35 | | 396.8159 | 6.36524 | 2024.375 | 80.79688 |  |  |  |  |  |
| 34.35833 | | 396.9063 | 6.362957 | 2023.734 | 80.82031 |  |  |  |  |  |
| 34.36667 | | 396.9932 | 6.365569 | 2023.047 | 81.07031 |  |  |  |  |  |
| 34.375 | | 397.0808 | 6.369964 | 2022.375 | 81.17188 |  |  |  |  |  |
| 34.38333 | | 397.1862 | 6.368758 | 2021.602 | 81.21094 |  |  |  |  |  |
| 34.39167 | | 397.2596 | 6.370831 | 2021.07 | 81.22656 |  |  |  |  |  |
| 34.4 | | 397.3685 | 6.374511 | 2020.281 | 81.10938 |  |  |  |  |  |
| 34.40833 | | 397.4397 | 6.373067 | 2019.742 | 81.38281 |  |  |  |  |  |
| 34.41667 | | 397.5287 | 6.3758 | 2019.031 | 81.72656 |  |  |  |  |  |
| 34.425 | | 397.637 | 6.379019 | 2018.227 | 81.86719 |  |  |  |  |  |
| 34.43333 | | 397.7111 | 6.377066 | 2017.672 | 81.80469 |  |  |  |  |  |
| 34.44167 | | 397.82 | 6.381271 | 2016.867 | 81.95313 |  |  |  |  |  |
| 34.45 | | 397.8927 | 6.383971 | 2016.336 | 81.89063 |  |  |  |  |  |
| 34.45833 | | 397.9824 | 6.382774 | 2015.617 | 81.92188 |  |  |  |  |  |
| 34.46667 | | 398.0885 | 6.386536 | 2014.766 | 81.95313 |  |  |  |  |  |
| 34.475 | | 398.1604 | 6.388397 | 2014.211 | 81.99219 |  |  |  |  |  |
| 34.48333 | | 398.248 | 6.386946 | 2013.531 | 82.09375 |  |  |  |  |  |
| 34.49167 | | 398.352 | 6.390485 | 2012.703 | 81.76563 |  |  |  |  |  |
| 34.5 | | 398.4211 | 6.393029 | 2012.172 | 81.67188 |  |  |  |  |  |
| 34.50833 | | 398.5258 | 6.392008 | 2011.352 | 81.40625 |  |  |  |  |  |
| 34.51667 | | 398.6127 | 6.395776 | 2010.664 | 81.28125 |  |  |  |  |  |
| 34.525 | | 398.7024 | 6.398175 | 2009.984 | 81.09375 |  |  |  |  |  |
| 34.53333 | | 398.7921 | 6.397262 | 2009.305 | 81 |  |  |  |  |  |
| 34.54167 | | 398.8633 | 6.399737 | 2008.805 | 80.75 |  |  |  |  |  |
| 34.55 | | 398.9545 | 6.402364 | 2008.141 | 80.53125 |  |  |  |  |  |
| 34.55833 | | 399.0613 | 6.400471 | 2007.367 | 80.44531 |  |  |  |  |  |
| 34.56667 | | 399.134 | 6.402796 | 2006.836 | 80.26563 |  |  |  |  |  |
| 34.575 | | 399.2436 | 6.405337 | 2006.055 | 80.17969 |  |  |  |  |  |
| 34.58333 | | 399.317 | 6.403398 | 2005.5 | 79.97656 |  |  |  |  |  |
| 34.59167 | | 399.4274 | 6.407447 | 2004.766 | 79.86719 |  |  |  |  |  |
| 34.6 | | 399.4972 | 6.409411 | 2004.25 | 79.79688 |  |  |  |  |  |
| 34.60833 | | 399.6011 | 6.408304 | 2003.461 | 79.64063 |  |  |  |  |  |
| 34.61667 | | 399.6724 | 6.411423 | 2002.945 | 79.30469 |  |  |  |  |  |
| 34.625 | | 399.7806 | 6.414002 | 2002.148 | 79.5 |  |  |  |  |  |
| 34.63333 | | 399.871 | 6.414477 | 2001.508 | 79.53125 |  |  |  |  |  |
| 34.64167 | | 399.9416 | 6.418227 | 2000.984 | 79.64063 |  |  |  |  |  |
| 34.65 | | 400.0306 | 6.421781 | 2000.32 | 79.75 |  |  |  |  |  |
| 34.65833 | | 400.1224 | 6.421916 | 1999.68 | 80.08594 |  |  |  |  |  |
| 34.66667 | | 400.2122 | 6.4272 | 1999.063 | 80.49219 |  |  |  |  |  |
| 34.675 | | 400.2998 | 6.430024 | 1998.359 | 80.97656 |  |  |  |  |  |
| 34.68333 | | 400.3888 | 6.430191 | 1997.695 | 81.4375 |  |  |  |  |  |
| 34.69167 | | 400.4807 | 6.434794 | 1997.008 | 81.84375 |  |  |  |  |  |
| 34.7 | | 400.5704 | 6.435539 | 1996.32 | 82.22656 |  |  |  |  |  |
| 34.70833 | | 400.6594 | 6.435774 | 1995.617 | 82.71094 |  |  |  |  |  |
| 34.71667 | | 400.7648 | 6.439396 | 1994.742 | 83.14063 |  |  |  |  |  |
| 34.725 | | 400.836 | 6.439283 | 1994.133 | 83.55469 |  |  |  |  |  |
| 34.73333 | | 400.9272 | 6.440094 | 1993.391 | 84.09375 |  |  |  |  |  |
| 34.74167 | | 401.0333 | 6.444517 | 1992.5 | 84.61719 |  |  |  |  |  |
| 34.75 | | 401.1209 | 6.444225 | 1991.758 | 84.96875 |  |  |  |  |  |
| 34.75833 | | 401.19 | 6.445973 | 1991.164 | 84.97656 |  |  |  |  |  |
| 34.76667 | | 401.2804 | 6.450075 | 1990.43 | 84.90625 |  |  |  |  |  |
| 34.775 | | 401.3701 | 6.450461 | 1989.656 | 85.09375 |  |  |  |  |  |
| 34.78333 | | 401.4584 | 6.452214 | 1988.883 | 85.17969 |  |  |  |  |  |
| 34.79167 | | 401.5645 | 6.456651 | 1987.969 | 85.17969 |  |  |  |  |  |
| 34.8 | | 401.6536 | 6.457117 | 1987.211 | 84.85938 |  |  |  |  |  |
| 34.80833 | | 401.7255 | 6.459435 | 1986.641 | 84.51563 |  |  |  |  |  |
| 34.81667 | | 401.8302 | 6.464336 | 1985.789 | 84.21875 |  |  |  |  |  |
| 34.825 | | 401.9028 | 6.464328 | 1985.211 | 83.85156 |  |  |  |  |  |
| 34.83333 | | 401.9926 | 6.465744 | 1984.492 | 83.15625 |  |  |  |  |  |
| 34.84167 | | 402.0866 | 6.46926 | 1983.781 | 83.03125 |  |  |  |  |  |
| 34.85 | | 402.1763 | 6.469255 | 1983.094 | 83.0625 |  |  |  |  |  |
| 34.85833 | | 402.2632 | 6.471178 | 1982.469 | 82.77344 |  |  |  |  |  |
| 34.86667 | | 402.3536 | 6.474724 | 1981.797 | 82.74219 |  |  |  |  |  |
| 34.875 | | 402.4441 | 6.475265 | 1981.164 | 82.6875 |  |  |  |  |  |
| 34.88333 | | 402.553 | 6.478843 | 1980.445 | 82.82813 |  |  |  |  |  |
| 34.89167 | | 402.6235 | 6.481795 | 1979.906 | 83.14063 |  |  |  |  |  |
| 34.9 | | 402.7318 | 6.482353 | 1979.094 | 82.92188 |  |  |  |  |  |
| 34.90833 | | 402.8016 | 6.485023 | 1978.57 | 83.32813 |  |  |  |  |  |
| 34.91667 | | 402.9105 | 6.489297 | 1977.758 | 83.51563 |  |  |  |  |  |
| 34.925 | | 402.981 | 6.489277 | 1977.219 | 83.33594 |  |  |  |  |  |
| 34.93333 | | 403.0686 | 6.491456 | 1976.531 | 83.28125 |  |  |  |  |  |
| 34.94167 | | 403.1747 | 6.496142 | 1975.648 | 83.125 |  |  |  |  |  |
| 34.95 | | 403.2645 | 6.496916 | 1974.984 | 83.03906 |  |  |  |  |  |
| 34.95833 | | 403.3535 | 6.501354 | 1974.234 | 82.98438 |  |  |  |  |  |
| 34.96667 | | 403.4261 | 6.50413 | 1973.664 | 83.14844 |  |  |  |  |  |
| 34.975 | | 403.5166 | 6.504493 | 1972.992 | 82.625 |  |  |  |  |  |
| 34.98333 | | 403.6049 | 6.507388 | 1972.32 | 82.60938 |  |  |  |  |  |
| 34.99167 | | 403.696 | 6.510941 | 1971.641 | 82.41406 |  |  |  |  |  |
| 35 | | 403.8015 | 6.511015 | 1970.836 | 82.52344 |  |  |  |  |  |
| 35.00833 | | 403.8741 | 6.51475 | 1970.281 | 82.53125 |  |  |  |  |  |
| 35.01667 | | 403.9802 | 6.517929 | 1969.477 | 82.64844 |  |  |  |  |  |
| 35.025 | | 404.0735 | 6.517631 | 1968.82 | 82.79688 |  |  |  |  |  |
| 35.03333 | | 404.1456 | 6.521697 | 1968.289 | 82.91406 |  |  |  |  |  |
| 35.04167 | | 404.2341 | 6.524459 | 1967.625 | 82.92188 |  |  |  |  |  |
| 35.05 | | 404.3219 | 6.524779 | 1966.93 | 82.71094 |  |  |  |  |  |
| 35.05833 | | 404.4111 | 6.52946 | 1966.234 | 82.77344 |  |  |  |  |  |
| 35.06667 | | 404.519 | 6.531825 | 1965.383 | 82.85938 |  |  |  |  |  |
| 35.075 | | 404.606 | 6.531466 | 1964.664 | 82.77344 |  |  |  |  |  |
| 35.08333 | | 404.6788 | 6.536328 | 1964.086 | 82.61719 |  |  |  |  |  |
| 35.09167 | | 404.7681 | 6.538333 | 1963.406 | 82.5625 |  |  |  |  |  |
| 35.1 | | 404.8587 | 6.538818 | 1962.734 | 82.49219 |  |  |  |  |  |
| 35.10833 | | 404.9637 | 6.545293 | 1961.898 | 82.25 |  |  |  |  |  |
| 35.11667 | | 405.0536 | 6.546166 | 1961.172 | 82.29688 |  |  |  |  |  |
| 35.125 | | 405.1429 | 6.545978 | 1960.484 | 82.47656 |  |  |  |  |  |
| 35.13333 | | 405.2171 | 6.550556 | 1959.945 | 82.46094 |  |  |  |  |  |
| 35.14167 | | 405.3063 | 6.550962 | 1959.258 | 82.73438 |  |  |  |  |  |
| 35.15 | | 405.3963 | 6.550749 | 1958.578 | 82.82031 |  |  |  |  |  |
| 35.15833 | | 405.4855 | 6.556009 | 1957.914 | 83 |  |  |  |  |  |
| 35.16667 | | 405.5919 | 6.55615 | 1957.086 | 83.25 |  |  |  |  |  |
| 35.175 | | 405.6647 | 6.556335 | 1956.516 | 83.39453 |  |  |  |  |  |
| 35.18333 | | 405.7546 | 6.561965 | 1955.82 | 83.58984 |  |  |  |  |  |
| 35.19167 | | 405.846 | 6.562588 | 1955.086 | 83.66406 |  |  |  |  |  |
| 35.2 | | 405.9345 | 6.562841 | 1954.383 | 83.64063 |  |  |  |  |  |
| 35.20833 | | 406.043 | 6.569028 | 1953.523 | 83.33984 |  |  |  |  |  |
| 35.21667 | | 406.1308 | 6.569564 | 1952.813 | 83.50391 |  |  |  |  |  |
| 35.225 | | 406.22 | 6.571223 | 1952.102 | 83.42969 |  |  |  |  |  |
| 35.23333 | | 406.3114 | 6.576685 | 1951.391 | 83.22266 |  |  |  |  |  |
| 35.24167 | | 406.4014 | 6.577236 | 1950.695 | 83.04297 |  |  |  |  |  |
| 35.25 | | 406.4913 | 6.579665 | 1950.008 | 83.16406 |  |  |  |  |  |
| 35.25833 | | 406.5612 | 6.584699 | 1949.477 | 83.07422 |  |  |  |  |  |
| 35.26667 | | 406.6526 | 6.58477 | 1948.773 | 83.11719 |  |  |  |  |  |
| 35.275 | | 406.7419 | 6.585442 | 1948.086 | 83.44922 |  |  |  |  |  |
| 35.28333 | | 406.8504 | 6.591529 | 1947.281 | 83.67969 |  |  |  |  |  |
| 35.29167 | | 406.9218 | 6.591358 | 1946.742 | 83.78516 |  |  |  |  |  |
| 35.3 | | 407.0124 | 6.592065 | 1946.047 | 84.01953 |  |  |  |  |  |
| 35.30833 | | 407.1031 | 6.598058 | 1945.352 | 84.03906 |  |  |  |  |  |
| 35.31667 | | 407.193 | 6.597412 | 1944.656 | 84.10938 |  |  |  |  |  |
| 35.325 | | 407.3029 | 6.599809 | 1943.773 | 84.20703 |  |  |  |  |  |
| 35.33333 | | 407.3929 | 6.603707 | 1943.047 | 84.05469 |  |  |  |  |  |
| 35.34167 | | 407.4636 | 6.6032 | 1942.469 | 84.06641 |  |  |  |  |  |
| 35.35 | | 407.5685 | 6.605514 | 1941.609 | 83.79297 |  |  |  |  |  |
| 35.35833 | | 407.6427 | 6.60991 | 1941.039 | 83.70703 |  |  |  |  |  |
| 35.36667 | | 407.7334 | 6.609234 | 1940.336 | 83.57813 |  |  |  |  |  |
| 35.375 | | 407.8248 | 6.611083 | 1939.641 | 83.46094 |  |  |  |  |  |
| 35.38333 | | 407.9304 | 6.616233 | 1938.805 | 83.21484 |  |  |  |  |  |
| 35.39167 | | 408.0025 | 6.615631 | 1938.25 | 83.30859 |  |  |  |  |  |
| 35.4 | | 408.1117 | 6.619222 | 1937.453 | 83.56641 |  |  |  |  |  |
| 35.40833 | | 408.1996 | 6.622606 | 1936.781 | 83.70703 |  |  |  |  |  |
| 35.41667 | | 408.2716 | 6.622478 | 1936.227 | 83.61719 |  |  |  |  |  |
| 35.425 | | 408.3809 | 6.626519 | 1935.422 | 83.83984 |  |  |  |  |  |
| 35.43333 | | 408.4537 | 6.629548 | 1934.875 | 83.875 |  |  |  |  |  |
| 35.44167 | | 408.5622 | 6.629553 | 1934.039 | 84.08984 |  |  |  |  |  |
| 35.45 | | 408.6336 | 6.632044 | 1933.453 | 84.125 |  |  |  |  |  |
| 35.45833 | | 408.7242 | 6.635428 | 1932.719 | 84.17578 |  |  |  |  |  |
| 35.46667 | | 408.8363 | 6.635274 | 1931.898 | 84.57422 |  |  |  |  |  |
| 35.475 | | 408.9277 | 6.639619 | 1931.172 | 84.70313 |  |  |  |  |  |
| 35.48333 | | 409.0176 | 6.641261 | 1930.461 | 84.81641 |  |  |  |  |  |
| 35.49167 | | 409.1083 | 6.642141 | 1929.742 | 85.17188 |  |  |  |  |  |
| 35.5 | | 409.1982 | 6.646286 | 1929.039 | 85.57422 |  |  |  |  |  |
| 35.50833 | | 409.2874 | 6.647426 | 1928.32 | 85.88281 |  |  |  |  |  |
| 35.51667 | | 409.3774 | 6.648133 | 1927.57 | 86.38672 |  |  |  |  |  |
| 35.525 | | 409.4638 | 6.652183 | 1926.852 | 86.80859 |  |  |  |  |  |
| 35.53333 | | 409.5494 | 6.653665 | 1926.125 | 87.11328 |  |  |  |  |  |
| 35.54167 | | 409.6379 | 6.654759 | 1925.367 | 87.58984 |  |  |  |  |  |
| 35.55 | | 409.725 | 6.659097 | 1924.586 | 87.89844 |  |  |  |  |  |
| 35.55833 | | 409.8121 | 6.660303 | 1923.836 | 88.125 |  |  |  |  |  |
| 35.56667 | | 409.9006 | 6.66123 | 1923.047 | 88.27344 |  |  |  |  |  |
| 35.575 | | 409.9884 | 6.665058 | 1922.258 | 88.40625 |  |  |  |  |  |
| 35.58333 | | 410.0763 | 6.666188 | 1921.484 | 88.62109 |  |  |  |  |  |
| 35.59167 | | 410.1655 | 6.666587 | 1920.695 | 88.82422 |  |  |  |  |  |
| 35.6 | | 410.2554 | 6.670519 | 1919.93 | 88.91406 |  |  |  |  |  |
| 35.60833 | | 410.3447 | 6.671407 | 1919.164 | 88.83984 |  |  |  |  |  |
| 35.61667 | | 410.4382 | 6.672055 | 1918.414 | 88.94531 |  |  |  |  |  |
| 35.625 | | 410.5274 | 6.675997 | 1917.664 | 89.03906 |  |  |  |  |  |
| 35.63333 | | 410.6159 | 6.676837 | 1916.914 | 89.01172 |  |  |  |  |  |
| 35.64167 | | 410.7059 | 6.678057 | 1916.141 | 88.87891 |  |  |  |  |  |
| 35.65 | | 410.7958 | 6.682068 | 1915.406 | 88.96094 |  |  |  |  |  |
| 35.65833 | | 410.8879 | 6.682287 | 1914.664 | 88.73828 |  |  |  |  |  |
| 35.66667 | | 410.9785 | 6.684141 | 1913.922 | 88.97266 |  |  |  |  |  |
| 35.675 | | 411.0699 | 6.688438 | 1913.164 | 89.11328 |  |  |  |  |  |
| 35.68333 | | 411.1591 | 6.687835 | 1912.438 | 89.06641 |  |  |  |  |  |
| 35.69167 | | 411.2527 | 6.690085 | 1911.703 | 88.91797 |  |  |  |  |  |
| 35.7 | | 411.3426 | 6.694514 | 1910.969 | 89.08203 |  |  |  |  |  |
| 35.70833 | | 411.4319 | 6.69299 | 1910.25 | 89.00391 |  |  |  |  |  |
| 35.71667 | | 411.5232 | 6.695713 | 1909.477 | 89.03516 |  |  |  |  |  |
| 35.725 | | 411.6153 | 6.699945 | 1908.727 | 89.14844 |  |  |  |  |  |
| 35.73333 | | 411.706 | 6.69815 | 1908 | 89.24609 |  |  |  |  |  |
| 35.74167 | | 411.7938 | 6.701214 | 1907.289 | 89.36719 |  |  |  |  |  |
| 35.75 | | 411.8858 | 6.705331 | 1906.523 | 89.24609 |  |  |  |  |  |
| 35.75833 | | 411.9746 | 6.703476 | 1905.805 | 89.30469 |  |  |  |  |  |
| 35.76667 | | 412.0648 | 6.707065 | 1905.063 | 89.49609 |  |  |  |  |  |
| 35.775 | | 412.1515 | 6.711049 | 1904.32 | 89.875 |  |  |  |  |  |
| 35.78333 | | 412.2425 | 6.7087 | 1903.555 | 89.76953 |  |  |  |  |  |
| 35.79167 | | 412.332 | 6.713097 | 1902.805 | 90.00781 |  |  |  |  |  |
| 35.8 | | 412.4215 | 6.716634 | 1902.078 | 90.10547 |  |  |  |  |  |
| 35.80833 | | 412.511 | 6.714577 | 1901.328 | 90.02344 |  |  |  |  |  |
| 35.81667 | | 412.6013 | 6.718498 | 1900.563 | 89.84375 |  |  |  |  |  |
| 35.825 | | 412.6929 | 6.721557 | 1899.766 | 89.67188 |  |  |  |  |  |
| 35.83333 | | 412.7796 | 6.719004 | 1899.039 | 89.45313 |  |  |  |  |  |
| 35.84167 | | 412.8698 | 6.722736 | 1898.258 | 88.9375 |  |  |  |  |  |
| 35.85 | | 412.96 | 6.726101 | 1897.5 | 88.80078 |  |  |  |  |  |
| 35.85833 | | 413.0531 | 6.724195 | 1896.758 | 88.51953 |  |  |  |  |  |
| 35.86667 | | 413.1405 | 6.729268 | 1896.039 | 88.32031 |  |  |  |  |  |
| 35.875 | | 413.2314 | 6.732086 | 1895.32 | 87.99219 |  |  |  |  |  |
| 35.88333 | | 413.3195 | 6.730428 | 1894.609 | 87.84766 |  |  |  |  |  |
| 35.89167 | | 413.4076 | 6.734741 | 1893.93 | 87.73047 |  |  |  |  |  |
| 35.9 | | 413.4971 | 6.737816 | 1893.211 | 87.84375 |  |  |  |  |  |
| 35.90833 | | 413.586 | 6.736298 | 1892.516 | 88.09375 |  |  |  |  |  |
| 35.91667 | | 413.6797 | 6.741288 | 1891.797 | 88.27344 |  |  |  |  |  |
| 35.925 | | 413.7714 | 6.744093 | 1891.109 | 88.57031 |  |  |  |  |  |
| 35.93333 | | 413.8581 | 6.742058 | 1890.383 | 88.82031 |  |  |  |  |  |
| 35.94167 | | 413.9433 | 6.74657 | 1889.664 | 88.91797 |  |  |  |  |  |
| 35.95 | | 414.0314 | 6.748507 | 1888.906 | 89.26172 |  |  |  |  |  |
| 35.95833 | | 414.1181 | 6.746173 | 1888.141 | 89.60547 |  |  |  |  |  |
| 35.96667 | | 414.2062 | 6.75053 | 1887.375 | 90.03125 |  |  |  |  |  |
| 35.975 | | 414.2943 | 6.752546 | 1886.594 | 90.28906 |  |  |  |  |  |
| 35.98333 | | 414.3817 | 6.750233 | 1885.82 | 90.39453 |  |  |  |  |  |
| 35.99167 | | 414.4698 | 6.754413 | 1885.063 | 90.63281 |  |  |  |  |  |
| 36 | | 414.5557 | 6.756939 | 1884.266 | 90.60156 |  |  |  |  |  |
| 36.00833 | | 414.641 | 6.755241 | 1883.477 | 90.85938 |  |  |  |  |  |
| 36.01667 | | 414.7312 | 6.75997 | 1882.664 | 91.16797 |  |  |  |  |  |
| 36.025 | | 414.8214 | 6.762404 | 1881.875 | 91.33594 |  |  |  |  |  |
| 36.03333 | | 414.911 | 6.760564 | 1881.102 | 91.3125 |  |  |  |  |  |
| 36.04167 | | 414.9998 | 6.764928 | 1880.32 | 91.38672 |  |  |  |  |  |
| 36.05 | | 415.0914 | 6.766829 | 1879.57 | 91.12109 |  |  |  |  |  |
| 36.05833 | | 415.1802 | 6.765262 | 1878.781 | 91.21484 |  |  |  |  |  |
| 36.06667 | | 415.2719 | 6.770469 | 1877.969 | 91.16797 |  |  |  |  |  |
| 36.075 | | 415.3614 | 6.772441 | 1877.195 | 91.38672 |  |  |  |  |  |
| 36.08333 | | 415.4495 | 6.770635 | 1876.438 | 91.42969 |  |  |  |  |  |
| 36.09167 | | 415.5404 | 6.776221 | 1875.672 | 91.28906 |  |  |  |  |  |
| 36.1 | | 415.6335 | 6.777601 | 1874.945 | 91.11719 |  |  |  |  |  |
| 36.10833 | | 415.7216 | 6.776128 | 1874.18 | 91.39453 |  |  |  |  |  |
| 36.11667 | | 415.8104 | 6.782031 | 1873.422 | 91.42969 |  |  |  |  |  |
| 36.125 | | 415.8978 | 6.783515 | 1872.641 | 91.30469 |  |  |  |  |  |
| 36.13333 | | 415.9824 | 6.782244 | 1871.859 | 91.29688 |  |  |  |  |  |
| 36.14167 | | 416.0719 | 6.787615 | 1871.133 | 91.51563 |  |  |  |  |  |
| 36.15 | | 416.1593 | 6.788979 | 1870.391 | 91.72266 |  |  |  |  |  |
| 36.15833 | | 416.2502 | 6.787383 | 1869.602 | 91.71875 |  |  |  |  |  |
| 36.16667 | | 416.3369 | 6.79255 | 1868.836 | 91.94922 |  |  |  |  |  |
| 36.175 | | 416.4271 | 6.793686 | 1868.094 | 92.17578 |  |  |  |  |  |
| 36.18333 | | 416.5131 | 6.791878 | 1867.328 | 92.16406 |  |  |  |  |  |
| 36.19167 | | 416.6026 | 6.79752 | 1866.531 | 92.25 |  |  |  |  |  |
| 36.2 | | 416.69 | 6.797481 | 1865.734 | 92.34375 |  |  |  |  |  |
| 36.20833 | | 416.7852 | 6.795598 | 1864.977 | 92.34375 |  |  |  |  |  |
| 36.21667 | | 416.8797 | 6.800843 | 1864.188 | 92.39453 |  |  |  |  |  |
| 36.225 | | 416.9663 | 6.801467 | 1863.391 | 92.46875 |  |  |  |  |  |
| 36.23333 | | 417.0544 | 6.801036 | 1862.625 | 92.24219 |  |  |  |  |  |
| 36.24167 | | 417.1454 | 6.806485 | 1861.859 | 92.35156 |  |  |  |  |  |
| 36.25 | | 417.2385 | 6.806904 | 1861.086 | 92.33594 |  |  |  |  |  |
| 36.25833 | | 417.3266 | 6.806718 | 1860.32 | 92.30859 |  |  |  |  |  |
| 36.26667 | | 417.4175 | 6.812001 | 1859.547 | 92.23828 |  |  |  |  |  |
| 36.275 | | 417.5063 | 6.81209 | 1858.781 | 92.22656 |  |  |  |  |  |
| 36.28333 | | 417.5958 | 6.8128 | 1858.047 | 92.25391 |  |  |  |  |  |
| 36.29167 | | 417.6825 | 6.8178 | 1857.266 | 92.44922 |  |  |  |  |  |
| 36.3 | | 417.7727 | 6.817979 | 1856.508 | 92.59375 |  |  |  |  |  |
| 36.30833 | | 417.8665 | 6.818582 | 1855.734 | 92.70313 |  |  |  |  |  |
| 36.31667 | | 417.9582 | 6.823302 | 1854.977 | 92.66406 |  |  |  |  |  |
| 36.325 | | 418.047 | 6.823407 | 1854.211 | 92.69922 |  |  |  |  |  |
| 36.33333 | | 418.1379 | 6.824643 | 1853.445 | 92.875 |  |  |  |  |  |
| 36.34167 | | 418.2274 | 6.829046 | 1852.641 | 92.87891 |  |  |  |  |  |
| 36.35 | | 418.3148 | 6.828825 | 1851.844 | 92.89453 |  |  |  |  |  |
| 36.35833 | | 418.4043 | 6.829736 | 1851.055 | 92.875 |  |  |  |  |  |
| 36.36667 | | 418.4924 | 6.834482 | 1850.289 | 92.96484 |  |  |  |  |  |
| 36.375 | | 418.5805 | 6.834561 | 1849.5 | 92.81641 |  |  |  |  |  |
| 36.38333 | | 418.6622 | 6.835677 | 1848.703 | 92.68359 |  |  |  |  |  |
| 36.39167 | | 418.7496 | 6.841014 | 1847.922 | 92.57031 |  |  |  |  |  |
| 36.4 | | 418.8363 | 6.841279 | 1847.156 | 92.63281 |  |  |  |  |  |
| 36.40833 | | 418.9237 | 6.841893 | 1846.383 | 92.43359 |  |  |  |  |  |
| 36.41667 | | 419.0075 | 6.847239 | 1845.586 | 92.28125 |  |  |  |  |  |
| 36.425 | | 419.0942 | 6.846762 | 1844.836 | 92.11328 |  |  |  |  |  |
| 36.43333 | | 419.1823 | 6.8474 | 1844.086 | 92.02734 |  |  |  |  |  |
| 36.44167 | | 419.2718 | 6.852521 | 1843.328 | 92.25781 |  |  |  |  |  |
| 36.45 | | 419.3656 | 6.851662 | 1842.555 | 92.22266 |  |  |  |  |  |
| 36.45833 | | 419.4587 | 6.853258 | 1841.813 | 92.42969 |  |  |  |  |  |
| 36.46667 | | 419.5532 | 6.857319 | 1841.07 | 92.74219 |  |  |  |  |  |
| 36.475 | | 419.6441 | 6.856854 | 1840.328 | 92.60156 |  |  |  |  |  |
| 36.48333 | | 419.7372 | 6.858666 | 1839.563 | 92.85547 |  |  |  |  |  |
| 36.49167 | | 419.8275 | 6.86172 | 1838.766 | 93 |  |  |  |  |  |
| 36.5 | | 419.922 | 6.86098 | 1838.016 | 93.12109 |  |  |  |  |  |
| 36.50833 | | 420.0179 | 6.864 | 1837.211 | 93.47266 |  |  |  |  |  |
| 36.51667 | | 420.1082 | 6.866498 | 1836.398 | 93.61328 |  |  |  |  |  |
| 36.525 | | 420.1971 | 6.866781 | 1835.648 | 93.66406 |  |  |  |  |  |
| 36.53333 | | 420.2866 | 6.870328 | 1834.836 | 93.94531 |  |  |  |  |  |
| 36.54167 | | 420.3733 | 6.872598 | 1834.039 | 93.96484 |  |  |  |  |  |
| 36.55 | | 420.4657 | 6.872423 | 1833.234 | 93.95703 |  |  |  |  |  |
| 36.55833 | | 420.5567 | 6.875455 | 1832.414 | 94.38672 |  |  |  |  |  |
| 36.56667 | | 420.6469 | 6.877993 | 1831.609 | 94.29688 |  |  |  |  |  |
| 36.575 | | 420.7336 | 6.878156 | 1830.82 | 94.24219 |  |  |  |  |  |
| 36.58333 | | 420.8218 | 6.881552 | 1830 | 94.35938 |  |  |  |  |  |
| 36.59167 | | 420.9092 | 6.884224 | 1829.227 | 94.5 |  |  |  |  |  |
| 36.6 | | 420.9987 | 6.884135 | 1828.438 | 94.74609 |  |  |  |  |  |
| 36.60833 | | 421.0897 | 6.88767 | 1827.594 | 94.44531 |  |  |  |  |  |
| 36.61667 | | 421.1814 | 6.890354 | 1826.813 | 94.37891 |  |  |  |  |  |
| 36.625 | | 421.2766 | 6.89068 | 1826.039 | 94.50391 |  |  |  |  |  |
| 36.63333 | | 421.3662 | 6.894323 | 1825.242 | 94.41406 |  |  |  |  |  |
| 36.64167 | | 421.4543 | 6.896045 | 1824.438 | 94.20313 |  |  |  |  |  |
| 36.65 | | 421.541 | 6.895815 | 1823.617 | 94.28125 |  |  |  |  |  |
| 36.65833 | | 421.6327 | 6.899077 | 1822.875 | 94.39063 |  |  |  |  |  |
| 36.66667 | | 421.7244 | 6.90006 | 1822.102 | 94.41406 |  |  |  |  |  |
| 36.675 | | 421.8147 | 6.899846 | 1821.305 | 94.15234 |  |  |  |  |  |
| 36.68333 | | 421.9049 | 6.903196 | 1820.523 | 94.03125 |  |  |  |  |  |
| 36.69167 | | 421.9966 | 6.904186 | 1819.781 | 94.05469 |  |  |  |  |  |
| 36.7 | | 422.0826 | 6.904164 | 1818.977 | 94.07813 |  |  |  |  |  |
| 36.70833 | | 422.1658 | 6.907209 | 1818.164 | 94.05469 |  |  |  |  |  |
| 36.71667 | | 422.2532 | 6.908515 | 1817.383 | 93.88281 |  |  |  |  |  |
| 36.725 | | 422.3435 | 6.907839 | 1816.633 | 93.80859 |  |  |  |  |  |
| 36.73333 | | 422.4359 | 6.91082 | 1815.867 | 93.61719 |  |  |  |  |  |
| 36.74167 | | 422.5268 | 6.91172 | 1815.07 | 93.55078 |  |  |  |  |  |
| 36.75 | | 422.6128 | 6.91185 | 1814.297 | 93.36719 |  |  |  |  |  |
| 36.75833 | | 422.6996 | 6.915282 | 1813.516 | 93.50781 |  |  |  |  |  |
| 36.76667 | | 422.7919 | 6.916599 | 1812.75 | 93.51953 |  |  |  |  |  |
| 36.775 | | 422.8822 | 6.916842 | 1811.984 | 93.86328 |  |  |  |  |  |
| 36.78333 | | 422.971 | 6.920425 | 1811.234 | 94.08594 |  |  |  |  |  |
| 36.79167 | | 423.0613 | 6.920464 | 1810.477 | 94.11719 |  |  |  |  |  |
| 36.8 | | 423.1473 | 6.920866 | 1809.711 | 94.3125 |  |  |  |  |  |
| 36.80833 | | 423.2383 | 6.924449 | 1808.922 | 94.70313 |  |  |  |  |  |
| 36.81667 | | 423.3286 | 6.92499 | 1808.141 | 95.07031 |  |  |  |  |  |
| 36.825 | | 423.4146 | 6.925916 | 1807.32 | 95.36719 |  |  |  |  |  |
| 36.83333 | | 423.4991 | 6.930059 | 1806.516 | 95.51563 |  |  |  |  |  |
| 36.84167 | | 423.5915 | 6.931387 | 1805.711 | 95.76953 |  |  |  |  |  |
| 36.85 | | 423.6832 | 6.93267 | 1804.914 | 95.87109 |  |  |  |  |  |
| 36.85833 | | 423.7692 | 6.936685 | 1804.055 | 95.97266 |  |  |  |  |  |
| 36.86667 | | 423.863 | 6.937128 | 1803.211 | 96.05078 |  |  |  |  |  |
| 36.875 | | 423.9561 | 6.938476 | 1802.383 | 96.00781 |  |  |  |  |  |
| 36.88333 | | 424.0485 | 6.942879 | 1801.563 | 96.47656 |  |  |  |  |  |
| 36.89167 | | 424.1381 | 6.942384 | 1800.727 | 96.30859 |  |  |  |  |  |
| 36.9 | | 424.2255 | 6.944982 | 1799.914 | 96.22266 |  |  |  |  |  |
| 36.90833 | | 424.3193 | 6.949612 | 1799.094 | 96.15625 |  |  |  |  |  |
| 36.91667 | | 424.4181 | 6.947683 | 1798.289 | 96.28906 |  |  |  |  |  |
| 36.925 | | 424.5041 | 6.950709 | 1797.484 | 96.33984 |  |  |  |  |  |
| 36.93333 | | 424.5922 | 6.954115 | 1796.625 | 96.54297 |  |  |  |  |  |
| 36.94167 | | 424.6853 | 6.951576 | 1795.836 | 96.625 |  |  |  |  |  |
| 36.95 | | 424.7678 | 6.954182 | 1795.055 | 96.70703 |  |  |  |  |  |
| 36.95833 | | 424.8552 | 6.957813 | 1794.258 | 97.14844 |  |  |  |  |  |
| 36.96667 | | 424.9462 | 6.955991 | 1793.43 | 97.32031 |  |  |  |  |  |
| 36.975 | | 425.0315 | 6.95907 | 1792.633 | 97.46094 |  |  |  |  |  |
| 36.98333 | | 425.1196 | 6.96299 | 1791.797 | 97.65234 |  |  |  |  |  |
| 36.99167 | | 425.2077 | 6.962189 | 1790.984 | 97.98047 |  |  |  |  |  |
| 37 | | 425.2944 | 6.964512 | 1790.164 | 98.14453 |  |  |  |  |  |
| 37.00833 | | 425.3854 | 6.967984 | 1789.289 | 98.42969 |  |  |  |  |  |
| 37.01667 | | 425.4707 | 6.966956 | 1788.477 | 98.34766 |  |  |  |  |  |
| 37.025 | | 425.5588 | 6.968387 | 1787.633 | 98.53516 |  |  |  |  |  |
| 37.03333 | | 425.6526 | 6.97216 | 1786.789 | 98.69922 |  |  |  |  |  |
| 37.04167 | | 425.7408 | 6.971374 | 1785.945 | 98.64453 |  |  |  |  |  |
| 37.05 | | 425.8303 | 6.974002 | 1785.102 | 98.72656 |  |  |  |  |  |
| 37.05833 | | 425.9199 | 6.977876 | 1784.25 | 98.63281 |  |  |  |  |  |
| 37.06667 | | 426.0102 | 6.977235 | 1783.438 | 98.86719 |  |  |  |  |  |
| 37.075 | | 426.1054 | 6.979743 | 1782.602 | 98.94922 |  |  |  |  |  |
| 37.08333 | | 426.1956 | 6.982984 | 1781.758 | 98.94141 |  |  |  |  |  |
| 37.09167 | | 426.2816 | 6.982142 | 1780.945 | 99.13672 |  |  |  |  |  |
| 37.1 | | 426.3733 | 6.9856 | 1780.125 | 99.23828 |  |  |  |  |  |
| 37.10833 | | 426.4622 | 6.989484 | 1779.32 | 99.32031 |  |  |  |  |  |
| 37.11667 | | 426.5539 | 6.988858 | 1778.477 | 99.41016 |  |  |  |  |  |
| 37.125 | | 426.6434 | 6.99256 | 1777.641 | 99.33203 |  |  |  |  |  |
| 37.13333 | | 426.7323 | 6.996208 | 1776.82 | 99.39453 |  |  |  |  |  |
| 37.14167 | | 426.8232 | 6.995088 | 1775.977 | 99.78125 |  |  |  |  |  |
| 37.15 | | 426.9121 | 6.999243 | 1775.148 | 99.74609 |  |  |  |  |  |
| 37.15833 | | 426.9966 | 7.002317 | 1774.313 | 99.78125 |  |  |  |  |  |
| 37.16667 | | 427.0876 | 7.001122 | 1773.477 | 99.92969 |  |  |  |  |  |
| 37.175 | | 427.1807 | 7.004569 | 1772.656 | 99.95703 |  |  |  |  |  |
| 37.18333 | | 427.2682 | 7.007584 | 1771.82 | 100.1914 |  |  |  |  |  |
| 37.19167 | | 427.357 | 7.006096 | 1770.938 | 100.0547 |  |  |  |  |  |
| 37.2 | | 427.4437 | 7.009968 | 1770.117 | 100.2734 |  |  |  |  |  |
| 37.20833 | | 427.5332 | 7.013249 | 1769.273 | 100.3281 |  |  |  |  |  |
| 37.21667 | | 427.6219 | 7.012067 | 1768.422 | 100.5742 |  |  |  |  |  |
| 37.225 | | 427.71 | 7.016598 | 1767.586 | 100.6289 |  |  |  |  |  |
| 37.23333 | | 427.7973 | 7.018959 | 1766.719 | 100.707 |  |  |  |  |  |
| 37.24167 | | 427.8825 | 7.017186 | 1765.898 | 100.75 |  |  |  |  |  |
| 37.25 | | 427.9691 | 7.021506 | 1765.039 | 101.0781 |  |  |  |  |  |
| 37.25833 | | 428.0572 | 7.024241 | 1764.195 | 100.9844 |  |  |  |  |  |
| 37.26667 | | 428.1445 | 7.023146 | 1763.328 | 100.8906 |  |  |  |  |  |
| 37.275 | | 428.2332 | 7.028798 | 1762.484 | 101.2266 |  |  |  |  |  |
| 37.28333 | | 428.322 | 7.032034 | 1761.641 | 101.0117 |  |  |  |  |  |
| 37.29167 | | 428.4121 | 7.030525 | 1760.797 | 101.1758 |  |  |  |  |  |
| 37.3 | | 428.5002 | 7.036139 | 1759.906 | 101.2773 |  |  |  |  |  |
| 37.30833 | | 428.5896 | 7.038496 | 1759.102 | 101.3242 |  |  |  |  |  |
| 37.31667 | | 428.6805 | 7.037701 | 1758.258 | 101.3047 |  |  |  |  |  |
| 37.325 | | 428.7721 | 7.044576 | 1757.375 | 101.2109 |  |  |  |  |  |
| 37.33333 | | 428.8608 | 7.047836 | 1756.563 | 100.9922 |  |  |  |  |  |
| 37.34167 | | 428.9503 | 7.049038 | 1755.703 | 101.1797 |  |  |  |  |  |
| 37.35 | | 429.0411 | 7.056009 | 1754.852 | 101.1367 |  |  |  |  |  |
| 37.35833 | | 429.1306 | 7.059054 | 1753.992 | 100.875 |  |  |  |  |  |
| 37.36667 | | 429.2207 | 7.059933 | 1753.156 | 101.1133 |  |  |  |  |  |
| 37.375 | | 429.3087 | 7.066712 | 1752.328 | 100.8789 |  |  |  |  |  |
| 37.38333 | | 429.394 | 7.069256 | 1751.516 | 101.0781 |  |  |  |  |  |
| 37.39167 | | 429.4841 | 7.069973 | 1750.648 | 100.9141 |  |  |  |  |  |
| 37.4 | | 429.5778 | 7.076451 | 1749.813 | 100.9961 |  |  |  |  |  |
| 37.40833 | | 429.6651 | 7.078098 | 1749 | 101.1133 |  |  |  |  |  |
| 37.41667 | | 429.7567 | 7.077869 | 1748.125 | 100.8125 |  |  |  |  |  |
| 37.425 | | 429.8419 | 7.083575 | 1747.328 | 100.9141 |  |  |  |  |  |
| 37.43333 | | 429.9314 | 7.085058 | 1746.461 | 100.9023 |  |  |  |  |  |
| 37.44167 | | 430.0201 | 7.084217 | 1745.641 | 101.1719 |  |  |  |  |  |
| 37.45 | | 430.1088 | 7.090337 | 1744.789 | 101.0938 |  |  |  |  |  |
| 37.45833 | | 430.1997 | 7.091222 | 1743.93 | 101.0547 |  |  |  |  |  |
| 37.46667 | | 430.2948 | 7.091072 | 1743.133 | 100.625 |  |  |  |  |  |
| 37.475 | | 430.3821 | 7.096036 | 1742.281 | 100.6172 |  |  |  |  |  |
| 37.48333 | | 430.4666 | 7.096506 | 1741.445 | 100.457 |  |  |  |  |  |
| 37.49167 | | 430.5582 | 7.097209 | 1740.563 | 100.2344 |  |  |  |  |  |
| 37.5 | | 430.6469 | 7.10223 | 1739.734 | 100.2461 |  |  |  |  |  |
| 37.50833 | | 430.7392 | 7.1023 | 1738.898 | 100.0391 |  |  |  |  |  |
| 37.51667 | | 430.8307 | 7.103291 | 1738.117 | 99.88672 |  |  |  |  |  |
| 37.525 | | 430.9223 | 7.107215 | 1737.273 | 99.41406 |  |  |  |  |  |
| 37.53333 | | 431.0118 | 7.106717 | 1736.469 | 99.16406 |  |  |  |  |  |
| 37.54167 | | 431.1005 | 7.108124 | 1735.664 | 99.16797 |  |  |  |  |  |
| 37.55 | | 431.19 | 7.11226 | 1734.828 | 99.07422 |  |  |  |  |  |
| 37.55833 | | 431.2801 | 7.112177 | 1734.023 | 98.91016 |  |  |  |  |  |
| 37.56667 | | 431.3696 | 7.113781 | 1733.211 | 98.89453 |  |  |  |  |  |
| 37.575 | | 431.459 | 7.117781 | 1732.445 | 98.86328 |  |  |  |  |  |
| 37.58333 | | 431.5471 | 7.117969 | 1731.648 | 99.07031 |  |  |  |  |  |
| 37.59167 | | 431.6386 | 7.120177 | 1730.828 | 98.88672 |  |  |  |  |  |
| 37.6 | | 431.7267 | 7.12434 | 1730.008 | 98.92188 |  |  |  |  |  |
| 37.60833 | | 431.8154 | 7.124515 | 1729.203 | 99.16797 |  |  |  |  |  |
| 37.61667 | | 431.9048 | 7.127494 | 1728.375 | 99.44922 |  |  |  |  |  |
| 37.625 | | 431.9929 | 7.131409 | 1727.555 | 99.63281 |  |  |  |  |  |
| 37.63333 | | 432.0795 | 7.131669 | 1726.695 | 99.87109 |  |  |  |  |  |
| 37.64167 | | 432.1675 | 7.13506 | 1725.891 | 99.98438 |  |  |  |  |  |
| 37.65 | | 432.2549 | 7.139165 | 1725.055 | 100.1602 |  |  |  |  |  |
| 37.65833 | | 432.3415 | 7.139958 | 1724.195 | 100.4375 |  |  |  |  |  |
| 37.66667 | | 432.4288 | 7.143183 | 1723.328 | 100.6797 |  |  |  |  |  |
| 37.675 | | 432.5162 | 7.147279 | 1722.461 | 101.3789 |  |  |  |  |  |
| 37.68333 | | 432.6035 | 7.147719 | 1721.594 | 101.6367 |  |  |  |  |  |
| 37.69167 | | 432.693 | 7.150785 | 1720.75 | 101.7539 |  |  |  |  |  |
| 37.7 | | 432.781 | 7.155057 | 1719.891 | 102.0195 |  |  |  |  |  |
| 37.70833 | | 432.8676 | 7.155305 | 1719.008 | 102.3711 |  |  |  |  |  |
| 37.71667 | | 432.955 | 7.158268 | 1718.125 | 102.2344 |  |  |  |  |  |
| 37.725 | | 433.0451 | 7.16235 | 1717.195 | 102.3984 |  |  |  |  |  |
| 37.73333 | | 433.136 | 7.162202 | 1716.305 | 102.4961 |  |  |  |  |  |
| 37.74167 | | 433.2254 | 7.165123 | 1715.445 | 102.625 |  |  |  |  |  |
| 37.75 | | 433.3163 | 7.168367 | 1714.555 | 102.6055 |  |  |  |  |  |
| 37.75833 | | 433.4029 | 7.168051 | 1713.664 | 102.3398 |  |  |  |  |  |
| 37.76667 | | 433.493 | 7.170991 | 1712.82 | 102.375 |  |  |  |  |  |
| 37.775 | | 433.5804 | 7.173703 | 1711.953 | 102.4883 |  |  |  |  |  |
| 37.78333 | | 433.6698 | 7.173383 | 1711.086 | 102.625 |  |  |  |  |  |
| 37.79167 | | 433.7628 | 7.177142 | 1710.211 | 102.5703 |  |  |  |  |  |
| 37.8 | | 433.8551 | 7.178807 | 1709.359 | 102.7695 |  |  |  |  |  |
| 37.80833 | | 433.9438 | 7.179306 | 1708.539 | 103.0391 |  |  |  |  |  |
| 37.81667 | | 434.0326 | 7.183926 | 1707.695 | 103.2773 |  |  |  |  |  |
| 37.825 | | 434.1227 | 7.18596 | 1706.82 | 103.3203 |  |  |  |  |  |
| 37.83333 | | 434.2101 | 7.18681 | 1705.961 | 103.4375 |  |  |  |  |  |
| 37.84167 | | 434.3044 | 7.192002 | 1705.102 | 103.7344 |  |  |  |  |  |
| 37.85 | | 434.396 | 7.193248 | 1704.219 | 103.9727 |  |  |  |  |  |
| 37.85833 | | 434.4875 | 7.194591 | 1703.328 | 104.0273 |  |  |  |  |  |
| 37.86667 | | 434.5784 | 7.200428 | 1702.438 | 104.1211 |  |  |  |  |  |
| 37.875 | | 434.6685 | 7.200898 | 1701.578 | 104.2109 |  |  |  |  |  |
| 37.88333 | | 434.7587 | 7.203308 | 1700.703 | 104.5 |  |  |  |  |  |
| 37.89167 | | 434.8495 | 7.209727 | 1699.805 | 104.6406 |  |  |  |  |  |
| 37.9 | | 434.9397 | 7.209205 | 1698.906 | 104.8867 |  |  |  |  |  |
| 37.90833 | | 435.0292 | 7.211224 | 1698.039 | 105.2148 |  |  |  |  |  |
| 37.91667 | | 435.1221 | 7.217983 | 1697.164 | 105.3555 |  |  |  |  |  |
| 37.925 | | 435.2112 | 7.216987 | 1696.297 | 105.6445 |  |  |  |  |  |
| 37.93333 | | 435.3015 | 7.219834 | 1695.391 | 105.8711 |  |  |  |  |  |
| 37.94167 | | 435.3891 | 7.226325 | 1694.508 | 106.0469 |  |  |  |  |  |
| 37.95 | | 435.4794 | 7.224906 | 1693.602 | 106.2813 |  |  |  |  |  |
| 37.95833 | | 435.569 | 7.228538 | 1692.688 | 106.4961 |  |  |  |  |  |
| 37.96667 | | 435.6586 | 7.234091 | 1691.797 | 106.5859 |  |  |  |  |  |
| 37.975 | | 435.7475 | 7.233238 | 1690.883 | 106.6055 |  |  |  |  |  |
| 37.98333 | | 435.8364 | 7.236573 | 1689.969 | 106.6602 |  |  |  |  |  |
| 37.99167 | | 435.9281 | 7.241574 | 1689.07 | 106.875 |  |  |  |  |  |
| 38 | | 436.0149 | 7.240235 | 1688.156 | 107.0117 |  |  |  |  |  |
| 38.00833 | | 436.1017 | 7.243214 | 1687.242 | 107.0938 |  |  |  |  |  |
| 38.01667 | | 436.1871 | 7.247923 | 1686.344 | 107.0781 |  |  |  |  |  |
| 38.025 | | 436.276 | 7.245923 | 1685.453 | 107.2695 |  |  |  |  |  |
| 38.03333 | | 436.3642 | 7.249473 | 1684.555 | 107.3906 |  |  |  |  |  |
| 38.04167 | | 436.4496 | 7.254331 | 1683.648 | 107.4336 |  |  |  |  |  |
| 38.05 | | 436.5378 | 7.252683 | 1682.734 | 107.6055 |  |  |  |  |  |
| 38.05833 | | 436.6288 | 7.256149 | 1681.828 | 107.9688 |  |  |  |  |  |
| 38.06667 | | 436.7177 | 7.260838 | 1680.945 | 108.3867 |  |  |  |  |  |
| 38.075 | | 436.8038 | 7.259674 | 1680.016 | 108.4844 |  |  |  |  |  |
| 38.08333 | | 436.8927 | 7.263329 | 1679.109 | 108.6055 |  |  |  |  |  |
| 38.09167 | | 436.9824 | 7.26784 | 1678.211 | 108.5313 |  |  |  |  |  |
| 38.1 | | 437.0741 | 7.267125 | 1677.289 | 108.8359 |  |  |  |  |  |
| 38.10833 | | 437.1637 | 7.272318 | 1676.336 | 109.2891 |  |  |  |  |  |
| 38.11667 | | 437.2484 | 7.276304 | 1675.375 | 109.5 |  |  |  |  |  |
| 38.125 | | 437.3387 | 7.276389 | 1674.469 | 109.8984 |  |  |  |  |  |
| 38.13333 | | 437.4276 | 7.280825 | 1673.547 | 109.7852 |  |  |  |  |  |
| 38.14167 | | 437.5158 | 7.283855 | 1672.664 | 109.8555 |  |  |  |  |  |
| 38.15 | | 437.6054 | 7.283823 | 1671.719 | 109.7461 |  |  |  |  |  |
| 38.15833 | | 437.695 | 7.288249 | 1670.75 | 109.875 |  |  |  |  |  |
| 38.16667 | | 437.7839 | 7.290714 | 1669.82 | 110.1094 |  |  |  |  |  |
| 38.175 | | 437.8721 | 7.291484 | 1668.852 | 110.2852 |  |  |  |  |  |
| 38.18333 | | 437.9582 | 7.296518 | 1667.961 | 110.3125 |  |  |  |  |  |
| 38.19167 | | 438.0471 | 7.299043 | 1667.039 | 110.25 |  |  |  |  |  |
| 38.2 | | 438.1395 | 7.299838 | 1666.141 | 110.2031 |  |  |  |  |  |
| 38.20833 | | 438.2284 | 7.304703 | 1665.211 | 110.2891 |  |  |  |  |  |
| 38.21667 | | 438.3187 | 7.306741 | 1664.266 | 110.7227 |  |  |  |  |  |
| 38.225 | | 438.4069 | 7.307825 | 1663.328 | 110.7227 |  |  |  |  |  |
| 38.23333 | | 438.4966 | 7.312642 | 1662.414 | 110.6836 |  |  |  |  |  |
| 38.24167 | | 438.5869 | 7.315034 | 1661.508 | 110.7422 |  |  |  |  |  |
| 38.25 | | 438.6779 | 7.316892 | 1660.594 | 110.6133 |  |  |  |  |  |
| 38.25833 | | 438.7703 | 7.32247 | 1659.672 | 110.7539 |  |  |  |  |  |
| 38.26667 | | 438.8592 | 7.324099 | 1658.695 | 110.8008 |  |  |  |  |  |
| 38.275 | | 438.9474 | 7.326336 | 1657.773 | 110.7148 |  |  |  |  |  |
| 38.28333 | | 439.0384 | 7.3313 | 1656.859 | 110.5781 |  |  |  |  |  |
| 38.29167 | | 439.1301 | 7.332269 | 1655.93 | 110.5859 |  |  |  |  |  |
| 38.3 | | 439.219 | 7.334944 | 1655.031 | 110.2383 |  |  |  |  |  |
| 38.30833 | | 439.3072 | 7.339826 | 1654.086 | 110.2695 |  |  |  |  |  |
| 38.31667 | | 439.3968 | 7.340514 | 1653.164 | 110.6055 |  |  |  |  |  |
| 38.325 | | 439.485 | 7.343668 | 1652.25 | 110.707 |  |  |  |  |  |
| 38.33333 | | 439.5746 | 7.348495 | 1651.352 | 110.8477 |  |  |  |  |  |
| 38.34167 | | 439.6621 | 7.348939 | 1650.43 | 110.8047 |  |  |  |  |  |
| 38.35 | | 439.7524 | 7.352146 | 1649.563 | 110.9609 |  |  |  |  |  |
| 38.35833 | | 439.8427 | 7.356398 | 1648.641 | 111.0391 |  |  |  |  |  |
| 38.36667 | | 439.9302 | 7.356548 | 1647.68 | 111.3867 |  |  |  |  |  |
| 38.375 | | 440.0198 | 7.359668 | 1646.742 | 111.582 |  |  |  |  |  |
| 38.38333 | | 440.1108 | 7.364146 | 1645.805 | 111.8047 |  |  |  |  |  |
| 38.39167 | | 440.2018 | 7.36462 | 1644.883 | 111.9492 |  |  |  |  |  |
| 38.4 | | 440.2907 | 7.368334 | 1643.945 | 112.0664 |  |  |  |  |  |
| 38.40833 | | 440.3824 | 7.373118 | 1643 | 112.2734 |  |  |  |  |  |
| 38.41667 | | 440.4713 | 7.373295 | 1642.023 | 112.2383 |  |  |  |  |  |
| 38.425 | | 440.5603 | 7.377722 | 1641.078 | 112.4063 |  |  |  |  |  |
| 38.43333 | | 440.6506 | 7.381653 | 1640.109 | 112.5234 |  |  |  |  |  |
| 38.44167 | | 440.7395 | 7.381423 | 1639.164 | 112.8906 |  |  |  |  |  |
| 38.45 | | 440.8298 | 7.385743 | 1638.211 | 112.8711 |  |  |  |  |  |
| 38.45833 | | 440.9152 | 7.389275 | 1637.258 | 112.8203 |  |  |  |  |  |
| 38.46667 | | 441.0027 | 7.388848 | 1636.313 | 112.8086 |  |  |  |  |  |
| 38.475 | | 441.0902 | 7.394117 | 1635.352 | 112.9883 |  |  |  |  |  |
| 38.48333 | | 441.1805 | 7.397844 | 1634.406 | 112.9023 |  |  |  |  |  |
| 38.49167 | | 441.268 | 7.397359 | 1633.414 | 113.0508 |  |  |  |  |  |
| 38.5 | | 441.3562 | 7.402453 | 1632.477 | 113.2031 |  |  |  |  |  |
| 38.50833 | | 441.4458 | 7.405956 | 1631.547 | 113.2969 |  |  |  |  |  |
| 38.51667 | | 441.5361 | 7.405211 | 1630.609 | 113.6367 |  |  |  |  |  |
| 38.525 | | 441.625 | 7.411105 | 1629.633 | 113.5664 |  |  |  |  |  |
| 38.53333 | | 441.7118 | 7.41427 | 1628.711 | 113.8242 |  |  |  |  |  |
| 38.54167 | | 441.8021 | 7.41393 | 1627.758 | 114.125 |  |  |  |  |  |
| 38.55 | | 441.8903 | 7.420419 | 1626.789 | 114.7305 |  |  |  |  |  |
| 38.55833 | | 441.9807 | 7.423549 | 1625.836 | 114.8906 |  |  |  |  |  |
| 38.56667 | | 442.0674 | 7.423068 | 1624.844 | 115.0469 |  |  |  |  |  |
| 38.575 | | 442.1563 | 7.429559 | 1623.906 | 115.4336 |  |  |  |  |  |
| 38.58333 | | 442.248 | 7.431427 | 1622.922 | 115.9258 |  |  |  |  |  |
| 38.59167 | | 442.3398 | 7.430452 | 1621.93 | 116.3555 |  |  |  |  |  |
| 38.6 | | 442.428 | 7.436954 | 1620.898 | 116.6367 |  |  |  |  |  |
| 38.60833 | | 442.5183 | 7.438834 | 1619.922 | 116.8008 |  |  |  |  |  |
| 38.61667 | | 442.6114 | 7.439397 | 1618.938 | 116.9805 |  |  |  |  |  |
| 38.625 | | 442.7024 | 7.445512 | 1617.922 | 117.2695 |  |  |  |  |  |
| 38.63333 | | 442.7927 | 7.446819 | 1616.898 | 117.2969 |  |  |  |  |  |
| 38.64167 | | 442.8828 | 7.447862 | 1615.867 | 117.5469 |  |  |  |  |  |
| 38.65 | | 442.9736 | 7.452945 | 1614.859 | 117.7813 |  |  |  |  |  |
| 38.65833 | | 443.0629 | 7.453638 | 1613.875 | 117.625 |  |  |  |  |  |
| 38.66667 | | 443.1495 | 7.455246 | 1612.883 | 117.2578 |  |  |  |  |  |
| 38.675 | | 443.236 | 7.460465 | 1611.867 | 117.0938 |  |  |  |  |  |
| 38.68333 | | 443.3253 | 7.461254 | 1610.898 | 116.7188 |  |  |  |  |  |
| 38.69167 | | 443.4154 | 7.463288 | 1609.891 | 116.7578 |  |  |  |  |  |
| 38.7 | | 443.5054 | 7.468039 | 1608.891 | 116.7773 |  |  |  |  |  |
| 38.70833 | | 443.5934 | 7.468753 | 1607.945 | 116.6367 |  |  |  |  |  |
| 38.71667 | | 443.6806 | 7.470767 | 1607.016 | 116.5234 |  |  |  |  |  |
| 38.725 | | 443.7686 | 7.475654 | 1606.07 | 116.293 |  |  |  |  |  |
| 38.73333 | | 443.8593 | 7.476192 | 1605.148 | 116.3047 |  |  |  |  |  |
| 38.74167 | | 443.9493 | 7.478778 | 1604.172 | 116.5195 |  |  |  |  |  |
| 38.75 | | 444.0387 | 7.48326 | 1603.211 | 116.8086 |  |  |  |  |  |
| 38.75833 | | 444.1266 | 7.484032 | 1602.258 | 116.9531 |  |  |  |  |  |
| 38.76667 | | 444.218 | 7.48794 | 1601.297 | 117.4141 |  |  |  |  |  |
| 38.775 | | 444.3053 | 7.492348 | 1600.367 | 117.6406 |  |  |  |  |  |
| 38.78333 | | 444.3904 | 7.493119 | 1599.398 | 117.8945 |  |  |  |  |  |
| 38.79167 | | 444.4798 | 7.49643 | 1598.398 | 118.1484 |  |  |  |  |  |
| 38.8 | | 444.5726 | 7.500419 | 1597.383 | 118.6289 |  |  |  |  |  |
| 38.80833 | | 444.662 | 7.50081 | 1596.398 | 119.207 |  |  |  |  |  |
| 38.81667 | | 444.7499 | 7.50493 | 1595.352 | 119.4883 |  |  |  |  |  |
| 38.825 | | 444.8392 | 7.508399 | 1594.344 | 119.7383 |  |  |  |  |  |
| 38.83333 | | 444.9314 | 7.50927 | 1593.328 | 119.8516 |  |  |  |  |  |
| 38.84167 | | 445.0228 | 7.514398 | 1592.305 | 120.1719 |  |  |  |  |  |
| 38.85 | | 445.1115 | 7.517605 | 1591.258 | 120.3008 |  |  |  |  |  |
| 38.85833 | | 445.2001 | 7.518501 | 1590.188 | 120.5586 |  |  |  |  |  |
| 38.86667 | | 445.2908 | 7.523807 | 1589.156 | 120.6211 |  |  |  |  |  |
| 38.875 | | 445.3829 | 7.526629 | 1588.125 | 120.707 |  |  |  |  |  |
| 38.88333 | | 445.4702 | 7.527384 | 1587.117 | 120.7852 |  |  |  |  |  |
| 38.89167 | | 445.5602 | 7.533244 | 1586.078 | 120.8555 |  |  |  |  |  |
| 38.9 | | 445.6475 | 7.534894 | 1585.063 | 120.6914 |  |  |  |  |  |
| 38.90833 | | 445.7354 | 7.535773 | 1584.031 | 120.6484 |  |  |  |  |  |
| 38.91667 | | 445.8234 | 7.54137 | 1583.016 | 121 |  |  |  |  |  |
| 38.925 | | 445.9127 | 7.54268 | 1582 | 120.8242 |  |  |  |  |  |
| 38.93333 | | 445.9985 | 7.543518 | 1580.992 | 120.7891 |  |  |  |  |  |
| 38.94167 | | 446.0858 | 7.549321 | 1579.977 | 120.3398 |  |  |  |  |  |
| 38.95 | | 446.1751 | 7.550575 | 1578.992 | 120.3906 |  |  |  |  |  |
| 38.95833 | | 446.261 | 7.551338 | 1577.984 | 120.3672 |  |  |  |  |  |
| 38.96667 | | 446.3496 | 7.557218 | 1576.93 | 120.0898 |  |  |  |  |  |
| 38.975 | | 446.4376 | 7.558725 | 1575.953 | 119.8594 |  |  |  |  |  |
| 38.98333 | | 446.5276 | 7.560103 | 1574.953 | 119.7422 |  |  |  |  |  |
| 38.99167 | | 446.6176 | 7.566355 | 1574.008 | 119.6289 |  |  |  |  |  |
| 39 | | 446.7056 | 7.567795 | 1572.992 | 119.3477 |  |  |  |  |  |
| 39.00833 | | 446.7928 | 7.569203 | 1572 | 119.5664 |  |  |  |  |  |
| 39.01667 | | 446.8836 | 7.574911 | 1571.039 | 119.6445 |  |  |  |  |  |
| 39.025 | | 446.9771 | 7.575679 | 1570.078 | 120.0547 |  |  |  |  |  |
| 39.03333 | | 447.0671 | 7.576918 | 1569.094 | 120.125 |  |  |  |  |  |
| 39.04167 | | 447.1579 | 7.583697 | 1568.117 | 120.2695 |  |  |  |  |  |
| 39.05 | | 447.2465 | 7.583778 | 1567.156 | 120.2461 |  |  |  |  |  |
| 39.05833 | | 447.3372 | 7.585916 | 1566.133 | 120.4375 |  |  |  |  |  |
| 39.06667 | | 447.4294 | 7.593178 | 1565.125 | 120.7266 |  |  |  |  |  |
| 39.075 | | 447.5194 | 7.592829 | 1564.078 | 120.9688 |  |  |  |  |  |
| 39.08333 | | 447.6101 | 7.595965 | 1563.078 | 120.9453 |  |  |  |  |  |
| 39.09167 | | 447.7002 | 7.602182 | 1562.063 | 121.0234 |  |  |  |  |  |
| 39.1 | | 447.7909 | 7.601917 | 1561.063 | 121.3281 |  |  |  |  |  |
| 39.10833 | | 447.8802 | 7.604939 | 1560.047 | 121.6953 |  |  |  |  |  |
| 39.11667 | | 447.9682 | 7.611198 | 1559 | 121.6875 |  |  |  |  |  |
| 39.125 | | 448.054 | 7.610859 | 1557.969 | 121.8789 |  |  |  |  |  |
| 39.13333 | | 448.1455 | 7.614599 | 1556.969 | 122.2461 |  |  |  |  |  |
| 39.14167 | | 448.2348 | 7.620547 | 1555.953 | 122.543 |  |  |  |  |  |
| 39.15 | | 448.3207 | 7.619883 | 1554.898 | 122.75 |  |  |  |  |  |
| 39.15833 | | 448.4093 | 7.623134 | 1553.828 | 122.8711 |  |  |  |  |  |
| 39.16667 | | 448.4987 | 7.628521 | 1552.813 | 123.3477 |  |  |  |  |  |
| 39.175 | | 448.5887 | 7.627859 | 1551.766 | 123.7695 |  |  |  |  |  |
| 39.18333 | | 448.6773 | 7.631429 | 1550.703 | 123.9063 |  |  |  |  |  |
| 39.19167 | | 448.7667 | 7.636942 | 1549.625 | 123.7813 |  |  |  |  |  |
| 39.2 | | 448.8567 | 7.636464 | 1548.578 | 124.1914 |  |  |  |  |  |
| 39.20833 | | 448.9502 | 7.640832 | 1547.531 | 124.2344 |  |  |  |  |  |
| 39.21667 | | 449.0389 | 7.645058 | 1546.445 | 124.4805 |  |  |  |  |  |
| 39.225 | | 449.1282 | 7.645011 | 1545.344 | 124.6289 |  |  |  |  |  |
| 39.23333 | | 449.219 | 7.649795 | 1544.297 | 124.8359 |  |  |  |  |  |
| 39.24167 | | 449.309 | 7.653829 | 1543.281 | 124.957 |  |  |  |  |  |
| 39.25 | | 449.3976 | 7.654406 | 1542.188 | 125.0703 |  |  |  |  |  |
| 39.25833 | | 449.487 | 7.659224 | 1541.141 | 125.0586 |  |  |  |  |  |
| 39.26667 | | 449.577 | 7.663135 | 1540.07 | 124.9492 |  |  |  |  |  |
| 39.275 | | 449.6649 | 7.663999 | 1539.023 | 125.1367 |  |  |  |  |  |
| 39.28333 | | 449.7543 | 7.669461 | 1537.945 | 125.0547 |  |  |  |  |  |
| 39.29167 | | 449.8436 | 7.672948 | 1536.906 | 125.4375 |  |  |  |  |  |
| 39.3 | | 449.9323 | 7.674328 | 1535.844 | 125.3281 |  |  |  |  |  |
| 39.30833 | | 450.0209 | 7.680075 | 1534.813 | 125.3867 |  |  |  |  |  |
| 39.31667 | | 450.111 | 7.682995 | 1533.781 | 125.3984 |  |  |  |  |  |
| 39.325 | | 450.1996 | 7.684837 | 1532.727 | 125.6094 |  |  |  |  |  |
| 39.33333 | | 450.2896 | 7.69028 | 1531.695 | 125.9805 |  |  |  |  |  |
| 39.34167 | | 450.3797 | 7.692444 | 1530.609 | 126.0586 |  |  |  |  |  |
| 39.35 | | 450.4676 | 7.694831 | 1529.586 | 126.4766 |  |  |  |  |  |
| 39.35833 | | 450.5583 | 7.70058 | 1528.539 | 126.8555 |  |  |  |  |  |
| 39.36667 | | 450.6484 | 7.702041 | 1527.5 | 127.0977 |  |  |  |  |  |
| 39.375 | | 450.7363 | 7.704618 | 1526.43 | 127.3906 |  |  |  |  |  |
| 39.38333 | | 450.8257 | 7.710777 | 1525.344 | 128.0156 |  |  |  |  |  |
| 39.39167 | | 450.9143 | 7.712516 | 1524.281 | 128.3555 |  |  |  |  |  |
| 39.4 | | 451.003 | 7.715843 | 1523.18 | 128.6875 |  |  |  |  |  |
| 39.40833 | | 451.093 | 7.721751 | 1522.078 | 129.25 |  |  |  |  |  |
| 39.41667 | | 451.183 | 7.723621 | 1520.984 | 129.375 |  |  |  |  |  |
| 39.425 | | 451.271 | 7.726926 | 1519.883 | 129.5195 |  |  |  |  |  |
| 39.43333 | | 451.3603 | 7.733196 | 1518.742 | 129.5352 |  |  |  |  |  |
| 39.44167 | | 451.4475 | 7.734755 | 1517.625 | 129.6602 |  |  |  |  |  |
| 39.45 | | 451.5355 | 7.738335 | 1516.516 | 129.6992 |  |  |  |  |  |
| 39.45833 | | 451.6255 | 7.744342 | 1515.367 | 129.5586 |  |  |  |  |  |
| 39.46667 | | 451.7184 | 7.745115 | 1514.273 | 129.5352 |  |  |  |  |  |
| 39.475 | | 451.8112 | 7.749369 | 1513.172 | 129.5664 |  |  |  |  |  |
| 39.48333 | | 451.9019 | 7.75481 | 1512.109 | 129.8047 |  |  |  |  |  |
| 39.49167 | | 451.9913 | 7.754789 | 1511.016 | 129.7383 |  |  |  |  |  |
| 39.5 | | 452.0792 | 7.760599 | 1509.938 | 129.8398 |  |  |  |  |  |
| 39.50833 | | 452.172 | 7.766548 | 1508.875 | 130.293 |  |  |  |  |  |
| 39.51667 | | 452.2614 | 7.766172 | 1507.805 | 130.5547 |  |  |  |  |  |
| 39.525 | | 452.3528 | 7.773074 | 1506.719 | 130.707 |  |  |  |  |  |
| 39.53333 | | 452.4449 | 7.778501 | 1505.609 | 130.6836 |  |  |  |  |  |
| 39.54167 | | 452.5357 | 7.77853 | 1504.531 | 130.8398 |  |  |  |  |  |
| 39.55 | | 452.625 | 7.785859 | 1503.453 | 130.9688 |  |  |  |  |  |
| 39.55833 | | 452.7137 | 7.790746 | 1502.305 | 131.1836 |  |  |  |  |  |
| 39.56667 | | 452.8023 | 7.790951 | 1501.18 | 131.2188 |  |  |  |  |  |
| 39.575 | | 452.8937 | 7.798271 | 1500.063 | 131.2813 |  |  |  |  |  |
| 39.58333 | | 452.9859 | 7.803516 | 1498.992 | 131.5703 |  |  |  |  |  |
| 39.59167 | | 453.0724 | 7.804415 | 1497.883 | 131.6094 |  |  |  |  |  |
| 39.6 | | 453.1624 | 7.81206 | 1496.773 | 131.6211 |  |  |  |  |  |
| 39.60833 | | 453.2532 | 7.816749 | 1495.648 | 131.5117 |  |  |  |  |  |
| 39.61667 | | 453.3446 | 7.817988 | 1494.555 | 131.8984 |  |  |  |  |  |
| 39.625 | | 453.434 | 7.825585 | 1493.453 | 132.1641 |  |  |  |  |  |
| 39.63333 | | 453.5219 | 7.829083 | 1492.32 | 132.3125 |  |  |  |  |  |
| 39.64167 | | 453.6098 | 7.830438 | 1491.227 | 132.3984 |  |  |  |  |  |
| 39.65 | | 453.7006 | 7.837121 | 1490.133 | 132.6602 |  |  |  |  |  |
| 39.65833 | | 453.7878 | 7.84038 | 1489.063 | 132.8633 |  |  |  |  |  |
| 39.66667 | | 453.8765 | 7.841272 | 1487.914 | 132.9141 |  |  |  |  |  |
| 39.675 | | 453.9679 | 7.847882 | 1486.781 | 133.043 |  |  |  |  |  |
| 39.68333 | | 454.0607 | 7.850545 | 1485.664 | 133.2969 |  |  |  |  |  |
| 39.69167 | | 454.1494 | 7.852546 | 1484.547 | 133.6563 |  |  |  |  |  |
| 39.7 | | 454.2352 | 7.858667 | 1483.414 | 134.0859 |  |  |  |  |  |
| 39.70833 | | 454.3246 | 7.860848 | 1482.281 | 134.1484 |  |  |  |  |  |
| 39.71667 | | 454.4153 | 7.862657 | 1481.188 | 134.5625 |  |  |  |  |  |
| 39.725 | | 454.5067 | 7.869134 | 1480.055 | 134.7266 |  |  |  |  |  |
| 39.73333 | | 454.5968 | 7.871467 | 1478.914 | 134.9063 |  |  |  |  |  |
| 39.74167 | | 454.6882 | 7.874463 | 1477.75 | 135.2227 |  |  |  |  |  |
| 39.75 | | 454.7769 | 7.880909 | 1476.586 | 135.6914 |  |  |  |  |  |
| 39.75833 | | 454.8662 | 7.882968 | 1475.445 | 135.7109 |  |  |  |  |  |
| 39.76667 | | 454.9562 | 7.886777 | 1474.281 | 135.8203 |  |  |  |  |  |
| 39.775 | | 455.0483 | 7.892429 | 1473.133 | 136.0859 |  |  |  |  |  |
| 39.78333 | | 455.1377 | 7.893429 | 1471.984 | 136.168 |  |  |  |  |  |
| 39.79167 | | 455.2277 | 7.898595 | 1470.813 | 136.3086 |  |  |  |  |  |
| 39.8 | | 455.3164 | 7.903173 | 1469.617 | 136.1992 |  |  |  |  |  |
| 39.80833 | | 455.4043 | 7.904418 | 1468.477 | 136.4336 |  |  |  |  |  |
| 39.81667 | | 455.493 | 7.910646 | 1467.336 | 136.5469 |  |  |  |  |  |
| 39.825 | | 455.5837 | 7.914955 | 1466.172 | 136.5664 |  |  |  |  |  |
| 39.83333 | | 455.6744 | 7.915678 | 1465.023 | 136.6094 |  |  |  |  |  |
| 39.84167 | | 455.7645 | 7.922379 | 1463.883 | 136.8555 |  |  |  |  |  |
| 39.85 | | 455.8524 | 7.925555 | 1462.766 | 137.1953 |  |  |  |  |  |
| 39.85833 | | 455.939 | 7.926075 | 1461.602 | 137.3828 |  |  |  |  |  |
| 39.86667 | | 456.0283 | 7.932433 | 1460.461 | 137.4688 |  |  |  |  |  |
| 39.875 | | 456.119 | 7.935967 | 1459.328 | 138.0156 |  |  |  |  |  |
| 39.88333 | | 456.2091 | 7.937 | 1458.188 | 138.8359 |  |  |  |  |  |
| 39.89167 | | 456.2998 | 7.944565 | 1457.008 | 139.1172 |  |  |  |  |  |
| 39.9 | | 456.3898 | 7.950111 | 1455.836 | 139.4805 |  |  |  |  |  |
| 39.90833 | | 456.4806 | 7.954882 | 1454.664 | 139.8281 |  |  |  |  |  |
| 39.91667 | | 456.5734 | 7.969006 | 1453.5 | 140.25 |  |  |  |  |  |
| 39.925 | | 456.6669 | 7.980326 | 1452.273 | 140.5469 |  |  |  |  |  |
| 39.93333 | | 456.7591 | 7.992088 | 1451.016 | 140.7773 |  |  |  |  |  |
| 39.94167 | | 456.8547 | 8.013687 | 1449.82 | 141 |  |  |  |  |  |
| 39.95 | | 456.9531 | 8.031236 | 1448.609 | 141.2031 |  |  |  |  |  |
| 39.95833 | | 457.0487 | 8.051942 | 1447.398 | 141.3906 |  |  |  |  |  |
| 39.96667 | | 457.1443 | 8.077382 | 1446.18 | 141.3555 |  |  |  |  |  |
| 39.975 | | 457.2414 | 8.098369 | 1444.984 | 141.293 |  |  |  |  |  |
| 39.98333 | | 457.3391 | 8.12237 | 1443.773 | 141.1289 |  |  |  |  |  |
| 39.99167 | | 457.434 | 8.145651 | 1442.578 | 141.1719 |  |  |  |  |  |
| 40 | | 457.5303 | 8.163749 | 1441.383 | 141.0898 |  |  |  |  |  |
| 40.00833 | | 457.6231 | 8.1856 | 1440.188 | 140.9727 |  |  |  |  |  |
| 40.01667 | | 457.7195 | 8.202373 | 1439.023 | 140.8398 |  |  |  |  |  |
| 40.025 | | 457.8137 | 8.216131 | 1437.859 | 140.6016 |  |  |  |  |  |
| 40.03333 | | 457.9044 | 8.234507 | 1436.711 | 140.4492 |  |  |  |  |  |
| 40.04167 | | 457.9966 | 8.246141 | 1435.531 | 139.9531 |  |  |  |  |  |
| 40.05 | | 458.0901 | 8.256161 | 1434.383 | 139.6797 |  |  |  |  |  |
| 40.05833 | | 458.1836 | 8.272085 | 1433.227 | 139.3359 |  |  |  |  |  |
| 40.06667 | | 458.2764 | 8.280508 | 1432.086 | 139.0703 |  |  |  |  |  |
| 40.075 | | 458.3665 | 8.289778 | 1430.945 | 138.7148 |  |  |  |  |  |
| 40.08333 | | 458.4558 | 8.302728 | 1429.805 | 138.3633 |  |  |  |  |  |
| 40.09167 | | 458.5479 | 8.309863 | 1428.703 | 138.0859 |  |  |  |  |  |
| 40.1 | | 458.6394 | 8.31956 | 1427.586 | 137.8516 |  |  |  |  |  |
| 40.10833 | | 458.7301 | 8.331258 | 1426.477 | 137.8984 |  |  |  |  |  |
| 40.11667 | | 458.8208 | 8.337767 | 1425.352 | 137.9531 |  |  |  |  |  |
| 40.125 | | 458.9088 | 8.346693 | 1424.258 | 137.9258 |  |  |  |  |  |
| 40.13333 | | 459.0009 | 8.357356 | 1423.141 | 137.7813 |  |  |  |  |  |
| 40.14167 | | 459.0903 | 8.363425 | 1422.031 | 137.9336 |  |  |  |  |  |
| 40.15 | | 459.1803 | 8.372565 | 1420.906 | 138.0078 |  |  |  |  |  |
| 40.15833 | | 459.2717 | 8.382335 | 1419.75 | 138.0313 |  |  |  |  |  |
| 40.16667 | | 459.3639 | 8.38812 | 1418.594 | 138.1289 |  |  |  |  |  |
| 40.175 | | 459.456 | 8.39786 | 1417.453 | 138.3242 |  |  |  |  |  |
| 40.18333 | | 459.5467 | 8.406543 | 1416.32 | 138.5352 |  |  |  |  |  |
| 40.19167 | | 459.6389 | 8.412473 | 1415.148 | 138.4102 |  |  |  |  |  |
| 40.2 | | 459.7289 | 8.422583 | 1414 | 138.3164 |  |  |  |  |  |
| 40.20833 | | 459.8238 | 8.429649 | 1412.844 | 138.4688 |  |  |  |  |  |
| 40.21667 | | 459.9146 | 8.435275 | 1411.672 | 138.4453 |  |  |  |  |  |
| 40.225 | | 460.0046 | 8.445402 | 1410.492 | 138.3242 |  |  |  |  |  |
| 40.23333 | | 460.0932 | 8.451203 | 1409.305 | 138.3477 |  |  |  |  |  |
| 40.24167 | | 460.1833 | 8.457123 | 1408.18 | 138.6055 |  |  |  |  |  |
| 40.25 | | 460.2719 | 8.467864 | 1407.039 | 138.8398 |  |  |  |  |  |
| 40.25833 | | 460.3599 | 8.473901 | 1405.859 | 138.8086 |  |  |  |  |  |
| 40.26667 | | 460.4478 | 8.480021 | 1404.703 | 138.6719 |  |  |  |  |  |
| 40.275 | | 460.5371 | 8.48992 | 1403.563 | 138.9063 |  |  |  |  |  |
| 40.28333 | | 460.63 | 8.494588 | 1402.391 | 139.1836 |  |  |  |  |  |
| 40.29167 | | 460.7214 | 8.500362 | 1401.188 | 139.2578 |  |  |  |  |  |
| 40.3 | | 460.8107 | 8.511168 | 1400 | 139.4922 |  |  |  |  |  |
| 40.30833 | | 460.9008 | 8.51504 | 1398.836 | 139.75 |  |  |  |  |  |
| 40.31667 | | 460.9922 | 8.521972 | 1397.688 | 140.0195 |  |  |  |  |  |
| 40.325 | | 461.0844 | 8.532684 | 1396.492 | 140.0742 |  |  |  |  |  |
| 40.33333 | | 461.1744 | 8.535454 | 1395.289 | 140.1055 |  |  |  |  |  |
| 40.34167 | | 461.2644 | 8.541915 | 1394.109 | 140.3516 |  |  |  |  |  |
| 40.35 | | 461.3538 | 8.551128 | 1392.914 | 140.6094 |  |  |  |  |  |
| 40.35833 | | 461.4431 | 8.553618 | 1391.711 | 140.7031 |  |  |  |  |  |
| 40.36667 | | 461.5359 | 8.560439 | 1390.508 | 140.7188 |  |  |  |  |  |
| 40.375 | | 461.6246 | 8.569644 | 1389.336 | 140.8828 |  |  |  |  |  |
| 40.38333 | | 461.7139 | 8.571849 | 1388.156 | 140.8398 |  |  |  |  |  |
| 40.39167 | | 461.8067 | 8.578888 | 1386.953 | 141.043 |  |  |  |  |  |
| 40.4 | | 461.8989 | 8.586982 | 1385.742 | 141.4219 |  |  |  |  |  |
| 40.40833 | | 461.9896 | 8.589879 | 1384.563 | 141.5234 |  |  |  |  |  |
| 40.41667 | | 462.081 | 8.59727 | 1383.391 | 141.75 |  |  |  |  |  |
| 40.425 | | 462.1725 | 8.604754 | 1382.203 | 141.8945 |  |  |  |  |  |
| 40.43333 | | 462.2667 | 8.607903 | 1381.039 | 142.3008 |  |  |  |  |  |
| 40.44167 | | 462.3588 | 8.615629 | 1379.844 | 142.8477 |  |  |  |  |  |
| 40.45 | | 462.4475 | 8.621799 | 1378.617 | 143.2813 |  |  |  |  |  |
| 40.45833 | | 462.5361 | 8.626049 | 1377.43 | 143.5195 |  |  |  |  |  |
| 40.46667 | | 462.6296 | 8.634857 | 1376.219 | 143.9727 |  |  |  |  |  |
| 40.475 | | 462.719 | 8.640897 | 1375.039 | 144.3984 |  |  |  |  |  |
| 40.48333 | | 462.8104 | 8.645559 | 1373.789 | 144.293 |  |  |  |  |  |
| 40.49167 | | 462.9018 | 8.653953 | 1372.539 | 144.3789 |  |  |  |  |  |
| 40.5 | | 462.9947 | 8.65836 | 1371.289 | 144.3789 |  |  |  |  |  |
| 40.50833 | | 463.0854 | 8.663488 | 1370.063 | 144.793 |  |  |  |  |  |
| 40.51667 | | 463.174 | 8.672035 | 1368.797 | 144.6445 |  |  |  |  |  |
| 40.525 | | 463.2648 | 8.675989 | 1367.555 | 144.8047 |  |  |  |  |  |
| 40.53333 | | 463.3569 | 8.681599 | 1366.367 | 144.8242 |  |  |  |  |  |
| 40.54167 | | 463.4483 | 8.690317 | 1365.156 | 144.9414 |  |  |  |  |  |
| 40.55 | | 463.5377 | 8.693372 | 1363.961 | 144.9531 |  |  |  |  |  |
| 40.55833 | | 463.6284 | 8.699252 | 1362.711 | 144.9492 |  |  |  |  |  |
| 40.56667 | | 463.7136 | 8.706783 | 1361.523 | 145.25 |  |  |  |  |  |
| 40.575 | | 463.8036 | 8.708851 | 1360.297 | 145.2695 |  |  |  |  |  |
| 40.58333 | | 463.8929 | 8.714725 | 1359.086 | 145.4414 |  |  |  |  |  |
| 40.59167 | | 463.9837 | 8.722613 | 1357.867 | 145.3438 |  |  |  |  |  |
| 40.6 | | 464.0751 | 8.724354 | 1356.672 | 145.332 |  |  |  |  |  |
| 40.60833 | | 464.1659 | 8.731074 | 1355.469 | 145.3555 |  |  |  |  |  |
| 40.61667 | | 464.2545 | 8.73919 | 1354.219 | 145.3945 |  |  |  |  |  |
| 40.625 | | 464.3452 | 8.740332 | 1353.016 | 145.6484 |  |  |  |  |  |
| 40.63333 | | 464.4353 | 8.747865 | 1351.789 | 145.7344 |  |  |  |  |  |
| 40.64167 | | 464.5267 | 8.755214 | 1350.586 | 145.7813 |  |  |  |  |  |
| 40.65 | | 464.6209 | 8.75638 | 1349.391 | 145.8125 |  |  |  |  |  |
| 40.65833 | | 464.7103 | 8.763917 | 1348.172 | 146.168 |  |  |  |  |  |
| 40.66667 | | 464.7996 | 8.770665 | 1346.961 | 146.4688 |  |  |  |  |  |
| 40.675 | | 464.8896 | 8.771906 | 1345.711 | 146.4727 |  |  |  |  |  |
| 40.68333 | | 464.979 | 8.779854 | 1344.492 | 146.8945 |  |  |  |  |  |
| 40.69167 | | 465.069 | 8.786039 | 1343.266 | 146.9492 |  |  |  |  |  |
| 40.7 | | 465.1591 | 8.787015 | 1342.055 | 146.9297 |  |  |  |  |  |
| 40.70833 | | 465.2505 | 8.794297 | 1340.789 | 147.0938 |  |  |  |  |  |
| 40.71667 | | 465.3412 | 8.799251 | 1339.523 | 147.2578 |  |  |  |  |  |
| 40.725 | | 465.4313 | 8.800467 | 1338.305 | 147.2695 |  |  |  |  |  |
| 40.73333 | | 465.5227 | 8.807672 | 1337.031 | 147.4805 |  |  |  |  |  |
| 40.74167 | | 465.6141 | 8.812589 | 1335.805 | 147.5234 |  |  |  |  |  |
| 40.75 | | 465.7049 | 8.81427 | 1334.578 | 147.3516 |  |  |  |  |  |
| 40.75833 | | 465.7952 | 8.821555 | 1333.328 | 147.582 |  |  |  |  |  |
| 40.76667 | | 465.8869 | 8.825863 | 1332.094 | 147.6523 |  |  |  |  |  |
| 40.775 | | 465.9759 | 8.828634 | 1330.867 | 147.8164 |  |  |  |  |  |
| 40.78333 | | 466.0662 | 8.836029 | 1329.617 | 148.0977 |  |  |  |  |  |
| 40.79167 | | 466.1566 | 8.839503 | 1328.383 | 148.0469 |  |  |  |  |  |
| 40.8 | | 466.2476 | 8.842621 | 1327.18 | 148.3984 |  |  |  |  |  |
| 40.80833 | | 466.3379 | 8.849237 | 1325.922 | 148.7539 |  |  |  |  |  |
| 40.81667 | | 466.4248 | 8.852179 | 1324.68 | 148.9648 |  |  |  |  |  |
| 40.825 | | 466.5131 | 8.855676 | 1323.43 | 149.0859 |  |  |  |  |  |
| 40.83333 | | 466.6048 | 8.862855 | 1322.164 | 149.4805 |  |  |  |  |  |
| 40.84167 | | 466.6958 | 8.865562 | 1320.93 | 149.7109 |  |  |  |  |  |
| 40.85 | | 466.7834 | 8.869003 | 1319.648 | 150.1328 |  |  |  |  |  |
| 40.85833 | | 466.873 | 8.875176 | 1318.359 | 150.2344 |  |  |  |  |  |
| 40.86667 | | 466.9606 | 8.876797 | 1317.086 | 150.582 |  |  |  |  |  |
| 40.875 | | 467.0475 | 8.880389 | 1315.82 | 150.8242 |  |  |  |  |  |
| 40.88333 | | 467.135 | 8.886363 | 1314.531 | 151.1875 |  |  |  |  |  |
| 40.89167 | | 467.2233 | 8.888325 | 1313.25 | 151.4492 |  |  |  |  |  |
| 40.9 | | 467.3164 | 8.893148 | 1311.953 | 151.7578 |  |  |  |  |  |
| 40.90833 | | 467.4095 | 8.898952 | 1310.68 | 151.9766 |  |  |  |  |  |
| 40.91667 | | 467.4991 | 8.900135 | 1309.383 | 152.0586 |  |  |  |  |  |
| 40.925 | | 467.588 | 8.907119 | 1308.094 | 152.3789 |  |  |  |  |  |
| 40.93333 | | 467.677 | 8.914033 | 1306.789 | 152.6875 |  |  |  |  |  |
| 40.94167 | | 467.7646 | 8.917686 | 1305.492 | 152.4766 |  |  |  |  |  |
| 40.95 | | 467.8576 | 8.928562 | 1304.195 | 152.4063 |  |  |  |  |  |
| 40.95833 | | 467.9487 | 8.938088 | 1302.906 | 152.3438 |  |  |  |  |  |
| 40.96667 | | 468.0397 | 8.944563 | 1301.625 | 152.3438 |  |  |  |  |  |
| 40.975 | | 468.13 | 8.95931 | 1300.32 | 152.2773 |  |  |  |  |  |
| 40.98333 | | 468.2197 | 8.971354 | 1299.016 | 152 |  |  |  |  |  |
| 40.99167 | | 468.3086 | 8.979632 | 1297.773 | 152.2617 |  |  |  |  |  |
| 41 | | 468.3976 | 8.996654 | 1296.523 | 152.4922 |  |  |  |  |  |
| 41.00833 | | 468.4886 | 9.00801 | 1295.266 | 152.4453 |  |  |  |  |  |
| 41.01667 | | 468.583 | 9.017145 | 1294.016 | 152.2617 |  |  |  |  |  |
| 41.025 | | 468.6768 | 9.031913 | 1292.758 | 152.5586 |  |  |  |  |  |
| 41.03333 | | 468.7651 | 9.041078 | 1291.523 | 152.8164 |  |  |  |  |  |
| 41.04167 | | 468.8575 | 9.049196 | 1290.227 | 152.9063 |  |  |  |  |  |
| 41.05 | | 468.9451 | 9.061505 | 1288.93 | 153.168 |  |  |  |  |  |
| 41.05833 | | 469.0347 | 9.068528 | 1287.672 | 153.3516 |  |  |  |  |  |
| 41.06667 | | 469.1257 | 9.074975 | 1286.43 | 153.6797 |  |  |  |  |  |
| 41.075 | | 469.214 | 9.085278 | 1285.117 | 153.8047 |  |  |  |  |  |
| 41.08333 | | 469.305 | 9.090065 | 1283.813 | 153.9297 |  |  |  |  |  |
| 41.09167 | | 469.3974 | 9.096017 | 1282.523 | 153.8516 |  |  |  |  |  |
| 41.1 | | 469.4863 | 9.105098 | 1281.219 | 154.0977 |  |  |  |  |  |
| 41.10833 | | 469.5753 | 9.109368 | 1279.914 | 154.2969 |  |  |  |  |  |
| 41.11667 | | 469.6649 | 9.115604 | 1278.594 | 154.2656 |  |  |  |  |  |
| 41.125 | | 469.7525 | 9.12364 | 1277.305 | 154.3789 |  |  |  |  |  |
| 41.13333 | | 469.8463 | 9.12729 | 1276.008 | 154.2617 |  |  |  |  |  |
| 41.14167 | | 469.9373 | 9.134606 | 1274.742 | 154.25 |  |  |  |  |  |
| 41.15 | | 470.0256 | 9.141217 | 1273.43 | 154.168 |  |  |  |  |  |
| 41.15833 | | 470.1173 | 9.144278 | 1272.125 | 154.0508 |  |  |  |  |  |
| 41.16667 | | 470.2069 | 9.15256 | 1270.844 | 153.7969 |  |  |  |  |  |
| 41.175 | | 470.2965 | 9.158764 | 1269.555 | 153.8242 |  |  |  |  |  |
| 41.18333 | | 470.3855 | 9.162111 | 1268.273 | 153.8555 |  |  |  |  |  |
| 41.19167 | | 470.4745 | 9.170617 | 1267 | 153.6133 |  |  |  |  |  |
| 41.2 | | 470.5662 | 9.176542 | 1265.727 | 153.543 |  |  |  |  |  |
| 41.20833 | | 470.6586 | 9.179897 | 1264.469 | 153.7813 |  |  |  |  |  |
| 41.21667 | | 470.7489 | 9.189001 | 1263.219 | 153.8867 |  |  |  |  |  |
| 41.225 | | 470.8378 | 9.193791 | 1261.93 | 154.0469 |  |  |  |  |  |
| 41.23333 | | 470.9268 | 9.19682 | 1260.648 | 154.0898 |  |  |  |  |  |
| 41.24167 | | 471.0164 | 9.206842 | 1259.391 | 154.2969 |  |  |  |  |  |
| 41.25 | | 471.1068 | 9.210529 | 1258.125 | 154.5469 |  |  |  |  |  |
| 41.25833 | | 471.1964 | 9.213792 | 1256.805 | 154.6406 |  |  |  |  |  |
| 41.26667 | | 471.2847 | 9.223709 | 1255.516 | 155.0156 |  |  |  |  |  |
| 41.275 | | 471.3757 | 9.226867 | 1254.203 | 155.25 |  |  |  |  |  |
| 41.28333 | | 471.466 | 9.229879 | 1252.914 | 155.5039 |  |  |  |  |  |
| 41.29167 | | 471.5543 | 9.239911 | 1251.594 | 155.6289 |  |  |  |  |  |
| 41.3 | | 471.6425 | 9.242423 | 1250.273 | 155.7227 |  |  |  |  |  |
| 41.30833 | | 471.7315 | 9.245193 | 1248.969 | 155.9375 |  |  |  |  |  |
| 41.31667 | | 471.8225 | 9.255263 | 1247.625 | 156.1758 |  |  |  |  |  |
| 41.325 | | 471.9142 | 9.257114 | 1246.289 | 156.4688 |  |  |  |  |  |
| 41.33333 | | 472.0025 | 9.260885 | 1244.961 | 156.7422 |  |  |  |  |  |
| 41.34167 | | 472.0894 | 9.269791 | 1243.656 | 156.8203 |  |  |  |  |  |
| 41.35 | | 472.1811 | 9.271521 | 1242.352 | 156.5664 |  |  |  |  |  |
| 41.35833 | | 472.2714 | 9.276039 | 1241.023 | 156.5273 |  |  |  |  |  |
| 41.36667 | | 472.3617 | 9.28343 | 1239.695 | 156.875 |  |  |  |  |  |
| 41.375 | | 472.4507 | 9.285431 | 1238.352 | 156.9141 |  |  |  |  |  |
| 41.38333 | | 472.5376 | 9.290656 | 1237.023 | 157.0352 |  |  |  |  |  |
| 41.39167 | | 472.6279 | 9.297471 | 1235.695 | 157.1602 |  |  |  |  |  |
| 41.4 | | 472.7162 | 9.299398 | 1234.43 | 157.4453 |  |  |  |  |  |
| 41.40833 | | 472.8037 | 9.305332 | 1233.133 | 157.2227 |  |  |  |  |  |
| 41.41667 | | 472.8933 | 9.310859 | 1231.789 | 157.3711 |  |  |  |  |  |
| 41.425 | | 472.983 | 9.313066 | 1230.477 | 157.4063 |  |  |  |  |  |
| 41.43333 | | 473.0706 | 9.319535 | 1229.148 | 157.7148 |  |  |  |  |  |
| 41.44167 | | 473.1581 | 9.324927 | 1227.82 | 157.9766 |  |  |  |  |  |
| 41.45 | | 473.245 | 9.327301 | 1226.477 | 157.8516 |  |  |  |  |  |
| 41.45833 | | 473.334 | 9.333371 | 1225.188 | 158.0586 |  |  |  |  |  |
| 41.46667 | | 473.4257 | 9.338336 | 1223.859 | 158.3672 |  |  |  |  |  |
| 41.475 | | 473.5139 | 9.340688 | 1222.547 | 158.3047 |  |  |  |  |  |
| 41.48333 | | 473.6036 | 9.347506 | 1221.188 | 158.4492 |  |  |  |  |  |
| 41.49167 | | 473.6918 | 9.351877 | 1219.844 | 158.7656 |  |  |  |  |  |
| 41.5 | | 473.7801 | 9.354629 | 1218.539 | 158.8789 |  |  |  |  |  |
| 41.50833 | | 473.869 | 9.361446 | 1217.195 | 159.3086 |  |  |  |  |  |
| 41.51667 | | 473.9594 | 9.365599 | 1215.844 | 159.6602 |  |  |  |  |  |
| 41.525 | | 474.0497 | 9.368153 | 1214.531 | 159.9063 |  |  |  |  |  |
| 41.53333 | | 474.1387 | 9.375143 | 1213.195 | 160.207 |  |  |  |  |  |
| 41.54167 | | 474.2283 | 9.378223 | 1211.836 | 160.293 |  |  |  |  |  |
| 41.55 | | 474.3166 | 9.380806 | 1210.5 | 160.5508 |  |  |  |  |  |
| 41.55833 | | 474.4062 | 9.387769 | 1209.125 | 160.5313 |  |  |  |  |  |
| 41.56667 | | 474.4958 | 9.390449 | 1207.75 | 160.5586 |  |  |  |  |  |
| 41.575 | | 474.5862 | 9.393639 | 1206.391 | 160.4219 |  |  |  |  |  |
| 41.58333 | | 474.6772 | 9.401279 | 1205.016 | 160.6328 |  |  |  |  |  |
| 41.59167 | | 474.7675 | 9.403078 | 1203.68 | 160.7773 |  |  |  |  |  |
| 41.6 | | 474.8565 | 9.407238 | 1202.313 | 160.4258 |  |  |  |  |  |
| 41.60833 | | 474.9454 | 9.414449 | 1200.977 | 160.3516 |  |  |  |  |  |
| 41.61667 | | 475.0378 | 9.415452 | 1199.648 | 160.1914 |  |  |  |  |  |
| 41.625 | | 475.1295 | 9.41975 | 1198.336 | 160.4023 |  |  |  |  |  |
| 41.63333 | | 475.2213 | 9.428073 | 1196.961 | 160.3242 |  |  |  |  |  |
| 41.64167 | | 475.3102 | 9.427508 | 1195.609 | 160.4375 |  |  |  |  |  |
| 41.65 | | 475.4019 | 9.433162 | 1194.313 | 160.6875 |  |  |  |  |  |
| 41.65833 | | 475.4915 | 9.440273 | 1193 | 160.6914 |  |  |  |  |  |
| 41.66667 | | 475.5805 | 9.440386 | 1191.68 | 160.5156 |  |  |  |  |  |
| 41.675 | | 475.6694 | 9.446284 | 1190.32 | 160.3438 |  |  |  |  |  |
| 41.68333 | | 475.7598 | 9.45363 | 1188.992 | 160.4414 |  |  |  |  |  |
| 41.69167 | | 475.8515 | 9.455934 | 1187.641 | 160.5703 |  |  |  |  |  |
| 41.7 | | 475.9404 | 9.464362 | 1186.281 | 160.7148 |  |  |  |  |  |
| 41.70833 | | 476.028 | 9.473593 | 1184.938 | 160.6094 |  |  |  |  |  |
| 41.71667 | | 476.1163 | 9.477858 | 1183.617 | 160.3633 |  |  |  |  |  |
| 41.725 | | 476.2094 | 9.488931 | 1182.313 | 160.4531 |  |  |  |  |  |
| 41.73333 | | 476.2976 | 9.499777 | 1180.969 | 160.5625 |  |  |  |  |  |
| 41.74167 | | 476.3872 | 9.50619 | 1179.609 | 160.9297 |  |  |  |  |  |
| 41.75 | | 476.4803 | 9.518396 | 1178.258 | 161.0391 |  |  |  |  |  |
| 41.75833 | | 476.5714 | 9.528729 | 1176.938 | 161.1797 |  |  |  |  |  |
| 41.76667 | | 476.6631 | 9.535634 | 1175.625 | 161.543 |  |  |  |  |  |
| 41.775 | | 476.7513 | 9.546954 | 1174.281 | 161.6367 |  |  |  |  |  |
| 41.78333 | | 476.841 | 9.555717 | 1172.93 | 161.8594 |  |  |  |  |  |
| 41.79167 | | 476.9313 | 9.561829 | 1171.539 | 162.1133 |  |  |  |  |  |
| 41.8 | | 477.0216 | 9.572573 | 1170.18 | 162.4805 |  |  |  |  |  |
| 41.80833 | | 477.1092 | 9.579317 | 1168.828 | 162.8867 |  |  |  |  |  |
| 41.81667 | | 477.1974 | 9.584865 | 1167.422 | 163.0313 |  |  |  |  |  |
| 41.825 | | 477.2864 | 9.594057 | 1166.078 | 163.0469 |  |  |  |  |  |
| 41.83333 | | 477.3747 | 9.599567 | 1164.695 | 163.3242 |  |  |  |  |  |
| 41.84167 | | 477.465 | 9.604947 | 1163.305 | 164.0508 |  |  |  |  |  |
| 41.85 | | 477.5519 | 9.613772 | 1161.906 | 164.2227 |  |  |  |  |  |
| 41.85833 | | 477.6401 | 9.619021 | 1160.492 | 164.6523 |  |  |  |  |  |
| 41.86667 | | 477.7284 | 9.624023 | 1159.117 | 164.9688 |  |  |  |  |  |
| 41.875 | | 477.8194 | 9.632752 | 1157.758 | 165.4297 |  |  |  |  |  |
| 41.88333 | | 477.9098 | 9.636676 | 1156.352 | 165.7305 |  |  |  |  |  |
| 41.89167 | | 478.0015 | 9.642275 | 1154.883 | 165.7656 |  |  |  |  |  |
| 41.9 | | 478.0904 | 9.650492 | 1153.5 | 165.9883 |  |  |  |  |  |
| 41.90833 | | 478.1821 | 9.65362 | 1152.063 | 166.082 |  |  |  |  |  |
| 41.91667 | | 478.2718 | 9.659553 | 1150.648 | 166.4141 |  |  |  |  |  |
| 41.925 | | 478.3621 | 9.66715 | 1149.203 | 166.3477 |  |  |  |  |  |
| 41.93333 | | 478.4538 | 9.669386 | 1147.773 | 166.5195 |  |  |  |  |  |
| 41.94167 | | 478.5455 | 9.677011 | 1146.391 | 166.3984 |  |  |  |  |  |
| 41.95 | | 478.6386 | 9.684865 | 1144.984 | 166.7461 |  |  |  |  |  |
| 41.95833 | | 478.7289 | 9.686334 | 1143.594 | 166.6914 |  |  |  |  |  |
| 41.96667 | | 478.8165 | 9.695307 | 1142.164 | 166.8242 |  |  |  |  |  |
| 41.975 | | 478.9034 | 9.701517 | 1140.805 | 166.9961 |  |  |  |  |  |
| 41.98333 | | 478.9951 | 9.702233 | 1139.391 | 167.2773 |  |  |  |  |  |
| 41.99167 | | 479.0854 | 9.710792 | 1138.023 | 167.6602 |  |  |  |  |  |
| 42 | | 479.1737 | 9.717129 | 1136.594 | 167.6602 |  |  |  |  |  |
| 42.00833 | | 479.2613 | 9.718122 | 1135.227 | 167.6289 |  |  |  |  |  |
| 42.01667 | | 479.3502 | 9.72705 | 1133.82 | 167.7656 |  |  |  |  |  |
| 42.025 | | 479.4419 | 9.732735 | 1132.414 | 167.9336 |  |  |  |  |  |
| 42.03333 | | 479.5316 | 9.734107 | 1130.984 | 167.8711 |  |  |  |  |  |
| 42.04167 | | 479.6212 | 9.742413 | 1129.539 | 167.9336 |  |  |  |  |  |
| 42.05 | | 479.7136 | 9.746578 | 1128.148 | 167.9688 |  |  |  |  |  |
| 42.05833 | | 479.8067 | 9.749181 | 1126.758 | 167.875 |  |  |  |  |  |
| 42.06667 | | 479.8956 | 9.757011 | 1125.352 | 167.7813 |  |  |  |  |  |
| 42.075 | | 479.9832 | 9.760871 | 1123.938 | 167.6094 |  |  |  |  |  |
| 42.08333 | | 480.0728 | 9.764328 | 1122.555 | 167.5313 |  |  |  |  |  |
| 42.09167 | | 480.1618 | 9.771935 | 1121.148 | 167.5742 |  |  |  |  |  |
| 42.1 | | 480.2514 | 9.775506 | 1119.758 | 167.6289 |  |  |  |  |  |
| 42.10833 | | 480.3397 | 9.778735 | 1118.375 | 167.4648 |  |  |  |  |  |
| 42.11667 | | 480.4272 | 9.786021 | 1117 | 167.3242 |  |  |  |  |  |
| 42.125 | | 480.5148 | 9.788943 | 1115.633 | 167.1445 |  |  |  |  |  |
| 42.13333 | | 480.6045 | 9.791753 | 1114.258 | 166.9297 |  |  |  |  |  |
| 42.14167 | | 480.6941 | 9.799204 | 1112.852 | 166.9922 |  |  |  |  |  |
| 42.15 | | 480.7845 | 9.802248 | 1111.453 | 166.8477 |  |  |  |  |  |
| 42.15833 | | 480.8734 | 9.805363 | 1110.086 | 166.9336 |  |  |  |  |  |
| 42.16667 | | 480.9623 | 9.813048 | 1108.711 | 166.8672 |  |  |  |  |  |
| 42.175 | | 481.054 | 9.816211 | 1107.352 | 166.875 |  |  |  |  |  |
| 42.18333 | | 481.1451 | 9.820593 | 1105.984 | 166.625 |  |  |  |  |  |
| 42.19167 | | 481.234 | 9.827826 | 1104.594 | 166.5195 |  |  |  |  |  |
| 42.2 | | 481.3223 | 9.830625 | 1103.219 | 166.6367 |  |  |  |  |  |
| 42.20833 | | 481.4126 | 9.83664 | 1101.813 | 166.8867 |  |  |  |  |  |
| 42.21667 | | 481.5016 | 9.843901 | 1100.438 | 167.1914 |  |  |  |  |  |
| 42.225 | | 481.5905 | 9.846999 | 1099.047 | 167.4297 |  |  |  |  |  |
| 42.23333 | | 481.6788 | 9.854024 | 1097.695 | 167.9414 |  |  |  |  |  |
| 42.24167 | | 481.7684 | 9.861198 | 1096.305 | 168.125 |  |  |  |  |  |
| 42.25 | | 481.8601 | 9.864095 | 1094.898 | 168.7617 |  |  |  |  |  |
| 42.25833 | | 481.9477 | 9.871976 | 1093.477 | 169.2148 |  |  |  |  |  |
| 42.26667 | | 482.0346 | 9.877931 | 1092.031 | 169.7383 |  |  |  |  |  |
| 42.275 | | 482.1249 | 9.880411 | 1090.594 | 170.207 |  |  |  |  |  |
| 42.28333 | | 482.2166 | 9.888866 | 1089.117 | 170.6797 |  |  |  |  |  |
| 42.29167 | | 482.3042 | 9.894721 | 1087.695 | 171.0313 |  |  |  |  |  |
| 42.3 | | 482.3925 | 9.897926 | 1086.195 | 171.2539 |  |  |  |  |  |
| 42.30833 | | 482.4807 | 9.907665 | 1084.719 | 171.4453 |  |  |  |  |  |
| 42.31667 | | 482.5745 | 9.914257 | 1083.234 | 171.4727 |  |  |  |  |  |
| 42.325 | | 482.6648 | 9.917919 | 1081.75 | 171.75 |  |  |  |  |  |
| 42.33333 | | 482.7565 | 9.929707 | 1080.266 | 171.7852 |  |  |  |  |  |
| 42.34167 | | 482.8475 | 9.935186 | 1078.797 | 171.7891 |  |  |  |  |  |
| 42.35 | | 482.9392 | 9.93962 | 1077.336 | 171.7813 |  |  |  |  |  |
| 42.35833 | | 483.0303 | 9.951672 | 1075.891 | 171.9531 |  |  |  |  |  |
| 42.36667 | | 483.1192 | 9.95692 | 1074.461 | 172.125 |  |  |  |  |  |
| 42.375 | | 483.2116 | 9.962364 | 1072.992 | 172.2656 |  |  |  |  |  |
| 42.38333 | | 483.3006 | 9.973311 | 1071.563 | 172.4063 |  |  |  |  |  |
| 42.39167 | | 483.3923 | 9.978007 | 1070.125 | 172.6172 |  |  |  |  |  |
| 42.4 | | 483.4785 | 9.983074 | 1068.695 | 172.9297 |  |  |  |  |  |
| 42.40833 | | 483.5688 | 9.992878 | 1067.242 | 173.1289 |  |  |  |  |  |
| 42.41667 | | 483.6557 | 9.996151 | 1065.789 | 173.293 |  |  |  |  |  |
| 42.425 | | 483.744 | 10.00088 | 1064.336 | 173.5273 |  |  |  |  |  |
| 42.43333 | | 483.8329 | 10.01014 | 1062.883 | 173.9453 |  |  |  |  |  |
| 42.44167 | | 483.9211 | 10.01337 | 1061.422 | 174.0742 |  |  |  |  |  |
| 42.45 | | 484.0101 | 10.01886 | 1059.938 | 174.2148 |  |  |  |  |  |
| 42.45833 | | 484.0991 | 10.02726 | 1058.477 | 174.2227 |  |  |  |  |  |
| 42.46667 | | 484.1908 | 10.03026 | 1057.016 | 174.2422 |  |  |  |  |  |
| 42.475 | | 484.2811 | 10.03594 | 1055.547 | 174.4688 |  |  |  |  |  |
| 42.48333 | | 484.3707 | 10.04281 | 1054.055 | 174.8359 |  |  |  |  |  |
| 42.49167 | | 484.4597 | 10.04535 | 1052.578 | 175.0625 |  |  |  |  |  |
| 42.5 | | 484.5479 | 10.05142 | 1051.117 | 175.0898 |  |  |  |  |  |
| 42.50833 | | 484.6396 | 10.0579 | 1049.672 | 175.082 |  |  |  |  |  |
| 42.51667 | | 484.73 | 10.06012 | 1048.227 | 175.1211 |  |  |  |  |  |
| 42.525 | | 484.8189 | 10.06643 | 1046.758 | 175.2422 |  |  |  |  |  |
| 42.53333 | | 484.9093 | 10.07236 | 1045.25 | 175.2305 |  |  |  |  |  |
| 42.54167 | | 485.0003 | 10.07501 | 1043.781 | 175.293 |  |  |  |  |  |
| 42.55 | | 485.0892 | 10.08191 | 1042.32 | 175.4063 |  |  |  |  |  |
| 42.55833 | | 485.1754 | 10.08689 | 1040.867 | 175.4141 |  |  |  |  |  |
| 42.56667 | | 485.2651 | 10.08933 | 1039.406 | 174.9883 |  |  |  |  |  |
| 42.575 | | 485.3561 | 10.09604 | 1037.945 | 174.7852 |  |  |  |  |  |
| 42.58333 | | 485.4471 | 10.10016 | 1036.5 | 174.5938 |  |  |  |  |  |
| 42.59167 | | 485.534 | 10.10225 | 1035.039 | 174.5391 |  |  |  |  |  |
| 42.6 | | 485.6216 | 10.10899 | 1033.57 | 174.5898 |  |  |  |  |  |
| 42.60833 | | 485.7091 | 10.1136 | 1032.109 | 174.4883 |  |  |  |  |  |
| 42.61667 | | 485.796 | 10.1159 | 1030.711 | 174.6953 |  |  |  |  |  |
| 42.625 | | 485.8815 | 10.12199 | 1029.289 | 174.7734 |  |  |  |  |  |
| 42.63333 | | 485.9698 | 10.12637 | 1027.859 | 174.8789 |  |  |  |  |  |
| 42.64167 | | 486.0594 | 10.12803 | 1026.414 | 174.7969 |  |  |  |  |  |
| 42.65 | | 486.1511 | 10.1346 | 1024.953 | 174.9766 |  |  |  |  |  |
| 42.65833 | | 486.2401 | 10.13879 | 1023.508 | 175.207 |  |  |  |  |  |
| 42.66667 | | 486.329 | 10.14151 | 1022.016 | 175.5195 |  |  |  |  |  |
| 42.675 | | 486.4187 | 10.14911 | 1020.547 | 175.6719 |  |  |  |  |  |
| 42.68333 | | 486.5097 | 10.15208 | 1019.07 | 175.6484 |  |  |  |  |  |
| 42.69167 | | 486.6028 | 10.1547 | 1017.625 | 175.7969 |  |  |  |  |  |
| 42.7 | | 486.6945 | 10.16216 | 1016.141 | 175.7891 |  |  |  |  |  |
| 42.70833 | | 486.7855 | 10.16256 | 1014.648 | 175.8086 |  |  |  |  |  |
| 42.71667 | | 486.8752 | 10.16504 | 1013.141 | 175.8867 |  |  |  |  |  |
| 42.725 | | 486.9669 | 10.17315 | 1011.664 | 176.1367 |  |  |  |  |  |
| 42.73333 | | 487.0523 | 10.17221 | 1010.203 | 176.2969 |  |  |  |  |  |
| 42.74167 | | 487.1392 | 10.17568 | 1008.719 | 176.3906 |  |  |  |  |  |
| 42.75 | | 487.2275 | 10.18355 | 1007.266 | 176.2734 |  |  |  |  |  |
| 42.75833 | | 487.3192 | 10.1831 | 1005.789 | 176.1914 |  |  |  |  |  |
| 42.76667 | | 487.4075 | 10.18722 | 1004.313 | 176.3047 |  |  |  |  |  |
| 42.775 | | 487.493 | 10.19423 | 1002.813 | 176.2617 |  |  |  |  |  |
| 42.78333 | | 487.5812 | 10.1939 | 1001.32 | 176.4648 |  |  |  |  |  |
| 42.79167 | | 487.6722 | 10.19801 | 999.8438 | 176.3164 |  |  |  |  |  |
| 42.8 | | 487.7633 | 10.20597 | 998.3984 | 176.4219 |  |  |  |  |  |
| 42.80833 | | 487.8502 | 10.20539 | 996.9297 | 176.5977 |  |  |  |  |  |
| 42.81667 | | 487.9384 | 10.21025 | 995.4531 | 176.3906 |  |  |  |  |  |
| 42.825 | | 488.028 | 10.21752 | 993.9844 | 176.3594 |  |  |  |  |  |
| 42.83333 | | 488.117 | 10.21689 | 992.4766 | 176.332 |  |  |  |  |  |
| 42.84167 | | 488.2046 | 10.22157 | 991.0313 | 176.3281 |  |  |  |  |  |
| 42.85 | | 488.2935 | 10.228 | 989.5469 | 176.6484 |  |  |  |  |  |
| 42.85833 | | 488.3818 | 10.22684 | 988.0625 | 177.0039 |  |  |  |  |  |
| 42.86667 | | 488.4699 | 10.23081 | 986.6172 | 176.8047 |  |  |  |  |  |
| 42.875 | | 488.5564 | 10.23649 | 985.1563 | 177.1484 |  |  |  |  |  |
| 42.88333 | | 488.6443 | 10.23528 | 983.6797 | 177.1172 |  |  |  |  |  |
| 42.89167 | | 488.7321 | 10.23987 | 982.2109 | 177.2031 |  |  |  |  |  |
| 42.9 | | 488.8221 | 10.2442 | 980.6875 | 177.2617 |  |  |  |  |  |
| 42.90833 | | 488.9092 | 10.24333 | 979.1719 | 177.3594 |  |  |  |  |  |
| 42.91667 | | 488.9971 | 10.24752 | 977.7266 | 177.7227 |  |  |  |  |  |
| 42.925 | | 489.0843 | 10.25077 | 976.2031 | 177.8203 |  |  |  |  |  |
| 42.93333 | | 489.1721 | 10.24933 | 974.7344 | 177.793 |  |  |  |  |  |
| 42.94167 | | 489.2621 | 10.25324 | 973.25 | 177.5352 |  |  |  |  |  |
| 42.95 | | 489.3527 | 10.25639 | 971.7656 | 177.9023 |  |  |  |  |  |
| 42.95833 | | 489.4405 | 10.25546 | 970.2656 | 177.7344 |  |  |  |  |  |
| 42.96667 | | 489.527 | 10.26005 | 968.7422 | 177.9258 |  |  |  |  |  |
| 42.975 | | 489.6169 | 10.26348 | 967.2578 | 177.9844 |  |  |  |  |  |
| 42.98333 | | 489.7048 | 10.26311 | 965.7969 | 177.9961 |  |  |  |  |  |
| 42.99167 | | 489.7968 | 10.26828 | 964.3594 | 178.1641 |  |  |  |  |  |
| 43 | | 489.8853 | 10.27062 | 962.8203 | 178.0195 |  |  |  |  |  |
| 43.00833 | | 489.9739 | 10.27075 | 961.3594 | 178.0313 |  |  |  |  |  |
| 43.01667 | | 490.0638 | 10.27572 | 959.8516 | 178.0625 |  |  |  |  |  |
| 43.025 | | 490.153 | 10.27745 | 958.3672 | 178.4492 |  |  |  |  |  |
| 43.03333 | | 490.2388 | 10.27779 | 956.875 | 178.2813 |  |  |  |  |  |
| 43.04167 | | 490.3267 | 10.28268 | 955.3828 | 178.4453 |  |  |  |  |  |
| 43.05 | | 490.4146 | 10.28361 | 953.9141 | 178.3438 |  |  |  |  |  |
| 43.05833 | | 490.5024 | 10.2832 | 952.4219 | 178.5508 |  |  |  |  |  |
| 43.06667 | | 490.5923 | 10.28737 | 950.9297 | 178.7305 |  |  |  |  |  |
| 43.075 | | 490.6795 | 10.28735 | 949.3906 | 178.7422 |  |  |  |  |  |
| 43.08333 | | 490.7674 | 10.28775 | 947.9375 | 178.8945 |  |  |  |  |  |
| 43.09167 | | 490.8573 | 10.2917 | 946.4219 | 179.2266 |  |  |  |  |  |
| 43.1 | | 490.9445 | 10.291 | 944.9531 | 179.2383 |  |  |  |  |  |
| 43.10833 | | 491.0331 | 10.29208 | 943.4375 | 179.2461 |  |  |  |  |  |
| 43.11667 | | 491.1223 | 10.29589 | 941.9297 | 179.3281 |  |  |  |  |  |
| 43.125 | | 491.2095 | 10.29403 | 940.4297 | 179.293 |  |  |  |  |  |
| 43.13333 | | 491.2987 | 10.29529 | 938.9219 | 179.4766 |  |  |  |  |  |
| 43.14167 | | 491.3866 | 10.29856 | 937.3828 | 179.5586 |  |  |  |  |  |
| 43.15 | | 491.4737 | 10.29615 | 935.8906 | 179.6445 |  |  |  |  |  |
| 43.15833 | | 491.5616 | 10.29705 | 934.3984 | 179.6055 |  |  |  |  |  |
| 43.16667 | | 491.6515 | 10.29943 | 932.8984 | 179.7422 |  |  |  |  |  |
| 43.175 | | 491.7394 | 10.29665 | 931.4141 | 179.7031 |  |  |  |  |  |
| 43.18333 | | 491.8279 | 10.29718 | 929.8906 | 180.0039 |  |  |  |  |  |
| 43.19167 | | 491.9131 | 10.29963 | 928.3906 | 180.0781 |  |  |  |  |  |
| 43.2 | | 491.9989 | 10.2973 | 926.875 | 180.2109 |  |  |  |  |  |
| 43.20833 | | 492.0895 | 10.29775 | 925.3906 | 180.4727 |  |  |  |  |  |
| 43.21667 | | 492.1766 | 10.30001 | 923.875 | 180.4141 |  |  |  |  |  |
| 43.225 | | 492.2638 | 10.29735 | 922.3906 | 180.5508 |  |  |  |  |  |
| 43.23333 | | 492.3524 | 10.29824 | 920.8438 | 180.4766 |  |  |  |  |  |
| 43.24167 | | 492.4389 | 10.30045 | 919.3359 | 180.6602 |  |  |  |  |  |
| 43.25 | | 492.526 | 10.29841 | 917.8203 | 180.8438 |  |  |  |  |  |
| 43.25833 | | 492.6139 | 10.29954 | 916.2656 | 180.8906 |  |  |  |  |  |
| 43.26667 | | 492.7004 | 10.30211 | 914.7813 | 180.6992 |  |  |  |  |  |
| 43.275 | | 492.791 | 10.29959 | 913.2578 | 180.5781 |  |  |  |  |  |
| 43.28333 | | 492.8809 | 10.30154 | 911.7734 | 180.7578 |  |  |  |  |  |
| 43.29167 | | 492.9681 | 10.30405 | 910.2422 | 180.6797 |  |  |  |  |  |
| 43.3 | | 493.0567 | 10.30126 | 908.7109 | 180.8164 |  |  |  |  |  |
| 43.30833 | | 493.1438 | 10.3032 | 907.1953 | 180.7266 |  |  |  |  |  |
| 43.31667 | | 493.2317 | 10.30449 | 905.7188 | 181.0391 |  |  |  |  |  |
| 43.325 | | 493.3182 | 10.30099 | 904.2344 | 180.9258 |  |  |  |  |  |
| 43.33333 | | 493.4026 | 10.30218 | 902.7031 | 180.7305 |  |  |  |  |  |
| 43.34167 | | 493.4871 | 10.30338 | 901.2188 | 180.6641 |  |  |  |  |  |
| 43.35 | | 493.5749 | 10.30053 | 899.6875 | 180.9492 |  |  |  |  |  |
| 43.35833 | | 493.6621 | 10.30102 | 898.1953 | 180.9883 |  |  |  |  |  |
| 43.36667 | | 493.7479 | 10.30262 | 896.6484 | 180.8398 |  |  |  |  |  |
| 43.375 | | 493.833 | 10.29963 | 895.1563 | 180.832 |  |  |  |  |  |
| 43.38333 | | 493.9202 | 10.29942 | 893.6797 | 180.4102 |  |  |  |  |  |
| 43.39167 | | 494.0094 | 10.30121 | 892.1875 | 180.5195 |  |  |  |  |  |
| 43.4 | | 494.0939 | 10.29837 | 890.6406 | 180.4219 |  |  |  |  |  |
| 43.40833 | | 494.1797 | 10.29826 | 889.125 | 180.3359 |  |  |  |  |  |
| 43.41667 | | 494.271 | 10.2998 | 887.6484 | 180.4336 |  |  |  |  |  |
| 43.425 | | 494.3602 | 10.29675 | 886.1406 | 180.5781 |  |  |  |  |  |
| 43.43333 | | 494.446 | 10.29686 | 884.6953 | 180.6289 |  |  |  |  |  |
| 43.44167 | | 494.5318 | 10.29813 | 883.1797 | 180.5781 |  |  |  |  |  |
| 43.45 | | 494.6197 | 10.29472 | 881.6953 | 180.5508 |  |  |  |  |  |
| 43.45833 | | 494.7082 | 10.29512 | 880.2031 | 180.5469 |  |  |  |  |  |
| 43.46667 | | 494.794 | 10.29562 | 878.6797 | 181 |  |  |  |  |  |
| 43.475 | | 494.8792 | 10.29193 | 877.1563 | 181.043 |  |  |  |  |  |
| 43.48333 | | 494.967 | 10.29187 | 875.6484 | 181.2578 |  |  |  |  |  |
| 43.49167 | | 495.0556 | 10.2925 | 874.1484 | 181.3516 |  |  |  |  |  |
| 43.5 | | 495.1421 | 10.28919 | 872.6484 | 181.2773 |  |  |  |  |  |
| 43.50833 | | 495.2272 | 10.28943 | 871.1406 | 181.5586 |  |  |  |  |  |
| 43.51667 | | 495.3137 | 10.29014 | 869.5703 | 181.4141 |  |  |  |  |  |
| 43.525 | | 495.3995 | 10.28637 | 868.0547 | 181.4297 |  |  |  |  |  |
| 43.53333 | | 495.4832 | 10.2855 | 866.5156 | 181.582 |  |  |  |  |  |
| 43.54167 | | 495.5697 | 10.28572 | 864.9922 | 181.8477 |  |  |  |  |  |
| 43.55 | | 495.6576 | 10.28142 | 863.5 | 181.8672 |  |  |  |  |  |
| 43.55833 | | 495.7461 | 10.2806 | 861.9453 | 181.8945 |  |  |  |  |  |
| 43.56667 | | 495.8333 | 10.28053 | 860.4531 | 181.9531 |  |  |  |  |  |
| 43.575 | | 495.9211 | 10.27612 | 858.9375 | 182.1797 |  |  |  |  |  |
| 43.58333 | | 496.0068 | 10.27458 | 857.3984 | 182.4531 |  |  |  |  |  |
| 43.59167 | | 496.093 | 10.27466 | 855.8516 | 182.3984 |  |  |  |  |  |
| 43.6 | | 496.1792 | 10.27038 | 854.3281 | 182.5625 |  |  |  |  |  |
| 43.60833 | | 496.2688 | 10.26853 | 852.7969 | 182.7969 |  |  |  |  |  |
| 43.61667 | | 496.3578 | 10.26917 | 851.2813 | 183.0234 |  |  |  |  |  |
| 43.625 | | 496.444 | 10.26472 | 849.7266 | 183.1016 |  |  |  |  |  |
| 43.63333 | | 496.5309 | 10.26288 | 848.1719 | 183.1758 |  |  |  |  |  |
| 43.64167 | | 496.6158 | 10.26379 | 846.6641 | 183.2656 |  |  |  |  |  |
| 43.65 | | 496.704 | 10.2593 | 845.1172 | 183.3984 |  |  |  |  |  |
| 43.65833 | | 496.7895 | 10.25802 | 843.5625 | 183.3555 |  |  |  |  |  |
| 43.66667 | | 496.8757 | 10.25926 | 842.0078 | 183.418 |  |  |  |  |  |
| 43.675 | | 496.9647 | 10.255 | 840.4766 | 183.4297 |  |  |  |  |  |
| 43.68333 | | 497.0543 | 10.25403 | 838.9297 | 183.2656 |  |  |  |  |  |
| 43.69167 | | 497.1399 | 10.25429 | 837.3984 | 183.2227 |  |  |  |  |  |
| 43.7 | | 497.2281 | 10.24986 | 835.8516 | 183.0859 |  |  |  |  |  |
| 43.70833 | | 497.3157 | 10.24917 | 834.3359 | 182.7656 |  |  |  |  |  |
| 43.71667 | | 497.4019 | 10.24926 | 832.7891 | 182.3555 |  |  |  |  |  |
| 43.725 | | 497.4874 | 10.24482 | 831.2734 | 182.3945 |  |  |  |  |  |
| 43.73333 | | 497.5709 | 10.24379 | 829.7656 | 182.5313 |  |  |  |  |  |
| 43.74167 | | 497.6571 | 10.24458 | 828.25 | 182.3359 |  |  |  |  |  |
| 43.75 | | 497.7426 | 10.24015 | 826.75 | 182.0313 |  |  |  |  |  |
| 43.75833 | | 497.8316 | 10.23852 | 825.2656 | 181.9141 |  |  |  |  |  |
| 43.76667 | | 497.9171 | 10.23926 | 823.8125 | 181.918 |  |  |  |  |  |
| 43.775 | | 498.0033 | 10.23391 | 822.2891 | 182.1875 |  |  |  |  |  |
| 43.78333 | | 498.0895 | 10.23169 | 820.7578 | 182.2031 |  |  |  |  |  |
| 43.79167 | | 498.1771 | 10.23262 | 819.2656 | 182.4648 |  |  |  |  |  |
| 43.8 | | 498.2647 | 10.22717 | 817.7891 | 182.9219 |  |  |  |  |  |
| 43.80833 | | 498.3537 | 10.22552 | 816.2969 | 183 |  |  |  |  |  |
| 43.81667 | | 498.4433 | 10.2258 | 814.7813 | 182.832 |  |  |  |  |  |
| 43.825 | | 498.5322 | 10.22026 | 813.2266 | 183.0586 |  |  |  |  |  |
| 43.83333 | | 498.6212 | 10.21833 | 811.7031 | 183.4258 |  |  |  |  |  |
| 43.84167 | | 498.706 | 10.21802 | 810.1484 | 183.6602 |  |  |  |  |  |
| 43.85 | | 498.7895 | 10.21202 | 808.5625 | 183.9102 |  |  |  |  |  |
| 43.85833 | | 498.875 | 10.20943 | 807.0313 | 183.8906 |  |  |  |  |  |
| 43.86667 | | 498.9633 | 10.20973 | 805.5234 | 183.9531 |  |  |  |  |  |
| 43.875 | | 499.0495 | 10.20327 | 803.9688 | 184.168 |  |  |  |  |  |
| 43.88333 | | 499.1337 | 10.20108 | 802.3828 | 184.1406 |  |  |  |  |  |
| 43.89167 | | 499.2185 | 10.20173 | 800.8125 | 184.1211 |  |  |  |  |  |
| 43.9 | | 499.3027 | 10.19559 | 799.25 | 184.1406 |  |  |  |  |  |
| 43.90833 | | 499.3882 | 10.19272 | 797.7266 | 184.3047 |  |  |  |  |  |
| 43.91667 | | 499.473 | 10.19347 | 796.1875 | 184.2852 |  |  |  |  |  |
| 43.925 | | 499.5592 | 10.18642 | 794.625 | 184.2695 |  |  |  |  |  |
| 43.93333 | | 499.6468 | 10.18287 | 793.0938 | 184.1094 |  |  |  |  |  |
| 43.94167 | | 499.7358 | 10.18359 | 791.5625 | 184.1523 |  |  |  |  |  |
| 43.95 | | 499.8213 | 10.17605 | 790.0234 | 184.2266 |  |  |  |  |  |
| 43.95833 | | 499.9055 | 10.17288 | 788.4609 | 184.043 |  |  |  |  |  |
| 43.96667 | | 499.9903 | 10.17299 | 786.9375 | 184.0078 |  |  |  |  |  |
| 43.975 | | 500.0738 | 10.1653 | 785.3984 | 183.9844 |  |  |  |  |  |
| 43.98333 | | 500.1607 | 10.16184 | 783.8906 | 184.1914 |  |  |  |  |  |
| 43.99167 | | 500.2455 | 10.16219 | 782.3438 | 184.125 |  |  |  |  |  |
| 44 | | 500.3324 | 10.15429 | 780.8047 | 184.0156 |  |  |  |  |  |
| 44.00833 | | 500.4186 | 10.15049 | 779.2891 | 183.957 |  |  |  |  |  |
| 44.01667 | | 500.5055 | 10.1515 | 777.7578 | 184.0742 |  |  |  |  |  |
| 44.025 | | 500.5883 | 10.14428 | 776.2266 | 184.293 |  |  |  |  |  |
| 44.03333 | | 500.6718 | 10.14092 | 774.6563 | 184.3789 |  |  |  |  |  |
| 44.04167 | | 500.7573 | 10.1415 | 773.1406 | 184.2578 |  |  |  |  |  |
| 44.05 | | 500.8456 | 10.13611 | 771.625 | 184.2578 |  |  |  |  |  |
| 44.05833 | | 500.9345 | 10.13288 | 770.0938 | 184.4453 |  |  |  |  |  |
| 44.06667 | | 501.0194 | 10.13321 | 768.5469 | 184.2539 |  |  |  |  |  |
| 44.075 | | 501.1049 | 10.12777 | 766.9766 | 184.2305 |  |  |  |  |  |
| 44.08333 | | 501.1904 | 10.12447 | 765.4375 | 184.3203 |  |  |  |  |  |
| 44.09167 | | 501.2753 | 10.12455 | 763.9141 | 184.1133 |  |  |  |  |  |
| 44.1 | | 501.3601 | 10.11916 | 762.3828 | 184.1719 |  |  |  |  |  |
| 44.10833 | | 501.4443 | 10.11532 | 760.8281 | 183.8164 |  |  |  |  |  |
| 44.11667 | | 501.5277 | 10.11506 | 759.3281 | 183.5508 |  |  |  |  |  |
| 44.125 | | 501.6153 | 10.10949 | 757.7969 | 183.4688 |  |  |  |  |  |
| 44.13333 | | 501.7022 | 10.10481 | 756.25 | 183.3047 |  |  |  |  |  |
| 44.14167 | | 501.7884 | 10.10353 | 754.75 | 183.1797 |  |  |  |  |  |
| 44.15 | | 501.8746 | 10.0977 | 753.2109 | 182.8359 |  |  |  |  |  |
| 44.15833 | | 501.9602 | 10.09222 | 751.7422 | 182.5547 |  |  |  |  |  |
| 44.16667 | | 502.0464 | 10.09042 | 750.25 | 182.3555 |  |  |  |  |  |
| 44.175 | | 502.134 | 10.08482 | 748.7344 | 182.3789 |  |  |  |  |  |
| 44.18333 | | 502.2209 | 10.07958 | 747.2266 | 182.1719 |  |  |  |  |  |
| 44.19167 | | 502.3084 | 10.07865 | 745.7109 | 182.1641 |  |  |  |  |  |
| 44.2 | | 502.3987 | 10.07441 | 744.2266 | 181.9688 |  |  |  |  |  |
| 44.20833 | | 502.485 | 10.07029 | 742.75 | 182.0781 |  |  |  |  |  |
| 44.21667 | | 502.5698 | 10.06947 | 741.2578 | 182.1406 |  |  |  |  |  |
| 44.225 | | 502.6567 | 10.06458 | 739.7344 | 181.9961 |  |  |  |  |  |
| 44.23333 | | 502.7443 | 10.05909 | 738.2344 | 182.0938 |  |  |  |  |  |
| 44.24167 | | 502.8326 | 10.05775 | 736.7109 | 182.2891 |  |  |  |  |  |
| 44.25 | | 502.9174 | 10.05324 | 735.2188 | 182.4141 |  |  |  |  |  |
| 44.25833 | | 503.0002 | 10.04797 | 733.6797 | 182.3438 |  |  |  |  |  |
| 44.26667 | | 503.0844 | 10.04674 | 732.1563 | 182.4883 |  |  |  |  |  |
| 44.275 | | 503.1699 | 10.04263 | 730.6484 | 182.375 |  |  |  |  |  |
| 44.28333 | | 503.254 | 10.03567 | 729.1172 | 182.5664 |  |  |  |  |  |
| 44.29167 | | 503.3402 | 10.03375 | 727.5703 | 182.4531 |  |  |  |  |  |
| 44.3 | | 503.4265 | 10.029 | 726.0313 | 182.5117 |  |  |  |  |  |
| 44.30833 | | 503.5103 | 10.02205 | 724.5078 | 182.2852 |  |  |  |  |  |
| 44.31667 | | 503.5941 | 10.02009 | 722.9766 | 182.3281 |  |  |  |  |  |
| 44.325 | | 503.6779 | 10.01648 | 721.4688 | 182.2148 |  |  |  |  |  |
| 44.33333 | | 503.763 | 10.0082 | 719.9141 | 182.1289 |  |  |  |  |  |
| 44.34167 | | 503.8488 | 10.00684 | 718.4063 | 182.0977 |  |  |  |  |  |
| 44.35 | | 503.9353 | 10.00422 | 716.8828 | 181.8477 |  |  |  |  |  |
| 44.35833 | | 504.0219 | 9.995666 | 715.3906 | 181.9023 |  |  |  |  |  |
| 44.36667 | | 504.107 | 9.994197 | 713.8594 | 181.7578 |  |  |  |  |  |
| 44.375 | | 504.1894 | 9.990717 | 712.3594 | 181.5781 |  |  |  |  |  |
| 44.38333 | | 504.2739 | 9.980968 | 710.8438 | 181.3125 |  |  |  |  |  |
| 44.39167 | | 504.3597 | 9.979268 | 709.3359 | 181.2617 |  |  |  |  |  |
| 44.4 | | 504.4455 | 9.97673 | 707.8516 | 181.3086 |  |  |  |  |  |
| 44.40833 | | 504.5313 | 9.967788 | 706.3359 | 181.3242 |  |  |  |  |  |
| 44.41667 | | 504.6165 | 9.966714 | 704.8281 | 181.0859 |  |  |  |  |  |
| 44.425 | | 504.7009 | 9.964257 | 703.3438 | 180.9805 |  |  |  |  |  |
| 44.43333 | | 504.7854 | 9.9551 | 701.8594 | 181.0938 |  |  |  |  |  |
| 44.44167 | | 504.8678 | 9.953024 | 700.3594 | 180.9258 |  |  |  |  |  |
| 44.45 | | 504.9536 | 9.950353 | 698.8359 | 180.8555 |  |  |  |  |  |
| 44.45833 | | 505.0394 | 9.94258 | 697.3203 | 180.75 |  |  |  |  |  |
| 44.46667 | | 505.1273 | 9.941108 | 695.8438 | 180.957 |  |  |  |  |  |
| 44.475 | | 505.2131 | 9.939455 | 694.3438 | 181.25 |  |  |  |  |  |
| 44.48333 | | 505.2962 | 9.932609 | 692.8125 | 181.2148 |  |  |  |  |  |
| 44.49167 | | 505.38 | 9.930794 | 691.3359 | 181.2891 |  |  |  |  |  |
| 44.5 | | 505.4658 | 9.929436 | 689.8359 | 181.4258 |  |  |  |  |  |
| 44.50833 | | 505.5537 | 9.92286 | 688.3516 | 181.4648 |  |  |  |  |  |
| 44.51667 | | 505.6409 | 9.921914 | 686.8047 | 181.3711 |  |  |  |  |  |
| 44.525 | | 505.7287 | 9.920663 | 685.2578 | 181.6875 |  |  |  |  |  |
| 44.53333 | | 505.8145 | 9.914234 | 683.75 | 181.7344 |  |  |  |  |  |
| 44.54167 | | 505.9024 | 9.914122 | 682.2344 | 182.0156 |  |  |  |  |  |
| 44.55 | | 505.9848 | 9.912275 | 680.6953 | 181.8438 |  |  |  |  |  |
| 44.55833 | | 506.0706 | 9.905847 | 679.1875 | 181.8789 |  |  |  |  |  |
| 44.56667 | | 506.1592 | 9.905645 | 677.6875 | 181.5625 |  |  |  |  |  |
| 44.575 | | 506.247 | 9.904687 | 676.1328 | 181.5977 |  |  |  |  |  |
| 44.58333 | | 506.3342 | 9.898153 | 674.6172 | 181.5 |  |  |  |  |  |
| 44.59167 | | 506.4194 | 9.8971 | 673.0547 | 181.5781 |  |  |  |  |  |
| 44.6 | | 506.5052 | 9.895235 | 671.5703 | 181.6289 |  |  |  |  |  |
| 44.60833 | | 506.5924 | 9.888683 | 670.0469 | 181.6055 |  |  |  |  |  |
| 44.61667 | | 506.6802 | 9.88639 | 668.5781 | 181.4336 |  |  |  |  |  |
| 44.625 | | 506.7654 | 9.884135 | 667.0625 | 181.3984 |  |  |  |  |  |
| 44.63333 | | 506.8526 | 9.877665 | 665.5547 | 181.2891 |  |  |  |  |  |
| 44.64167 | | 506.937 | 9.874365 | 664.0234 | 181.2422 |  |  |  |  |  |
| 44.65 | | 507.0249 | 9.872415 | 662.5 | 181.4258 |  |  |  |  |  |
| 44.65833 | | 507.1107 | 9.865462 | 660.9922 | 181.4141 |  |  |  |  |  |
| 44.66667 | | 507.1958 | 9.861856 | 659.5 | 181.543 |  |  |  |  |  |
| 44.675 | | 507.2803 | 9.859896 | 658 | 181.2813 |  |  |  |  |  |
| 44.68333 | | 507.3675 | 9.853103 | 656.4922 | 180.9531 |  |  |  |  |  |
| 44.69167 | | 507.4526 | 9.849033 | 654.9844 | 180.8242 |  |  |  |  |  |
| 44.7 | | 507.5364 | 9.84716 | 653.4531 | 180.793 |  |  |  |  |  |
| 44.70833 | | 507.6222 | 9.840852 | 651.9375 | 180.4219 |  |  |  |  |  |
| 44.71667 | | 507.7074 | 9.836933 | 650.4141 | 180.0703 |  |  |  |  |  |
| 44.725 | | 507.7925 | 9.835373 | 648.9375 | 179.8047 |  |  |  |  |  |
| 44.73333 | | 507.8756 | 9.828838 | 647.4766 | 179.7188 |  |  |  |  |  |
| 44.74167 | | 507.9601 | 9.823989 | 645.9922 | 179.5 |  |  |  |  |  |
| 44.75 | | 508.0459 | 9.822522 | 644.4922 | 179.1133 |  |  |  |  |  |
| 44.75833 | | 508.1331 | 9.815376 | 643.0313 | 178.9766 |  |  |  |  |  |
| 44.76667 | | 508.2182 | 9.810154 | 641.5859 | 178.9805 |  |  |  |  |  |
| 44.775 | | 508.302 | 9.807988 | 640.125 | 178.7969 |  |  |  |  |  |
| 44.78333 | | 508.3857 | 9.800709 | 638.6406 | 178.4922 |  |  |  |  |  |
| 44.79167 | | 508.4675 | 9.794935 | 637.1719 | 178.5195 |  |  |  |  |  |
| 44.8 | | 508.5519 | 9.79328 | 635.7344 | 178.6484 |  |  |  |  |  |
| 44.80833 | | 508.6391 | 9.786359 | 634.2656 | 178.5039 |  |  |  |  |  |
| 44.81667 | | 508.7229 | 9.779919 | 632.7734 | 178.25 |  |  |  |  |  |
| 44.825 | | 508.8073 | 9.778788 | 631.3047 | 178.1289 |  |  |  |  |  |
| 44.83333 | | 508.8925 | 9.771341 | 629.8516 | 177.875 |  |  |  |  |  |
| 44.84167 | | 508.979 | 9.76335 | 628.3594 | 177.8828 |  |  |  |  |  |
| 44.85 | | 509.0628 | 9.762739 | 626.8594 | 177.6367 |  |  |  |  |  |
| 44.85833 | | 509.1486 | 9.754592 | 625.3906 | 177.3281 |  |  |  |  |  |
| 44.86667 | | 509.2351 | 9.746192 | 623.9297 | 177.3477 |  |  |  |  |  |
| 44.875 | | 509.3243 | 9.745484 | 622.4531 | 177.2227 |  |  |  |  |  |
| 44.88333 | | 509.4095 | 9.737379 | 621.0078 | 177.1172 |  |  |  |  |  |
| 44.89167 | | 509.4919 | 9.728538 | 619.5234 | 177.0156 |  |  |  |  |  |
| 44.9 | | 509.5777 | 9.727755 | 618.0703 | 177.0234 |  |  |  |  |  |
| 44.90833 | | 509.6656 | 9.719347 | 616.625 | 176.8398 |  |  |  |  |  |
| 44.91667 | | 509.7487 | 9.710387 | 615.1484 | 177.0703 |  |  |  |  |  |
| 44.925 | | 509.8311 | 9.710132 | 613.6719 | 177.0234 |  |  |  |  |  |
| 44.93333 | | 509.9155 | 9.701829 | 612.2109 | 176.9375 |  |  |  |  |  |
| 44.94167 | | 510.0013 | 9.6936 | 610.7422 | 176.8125 |  |  |  |  |  |
| 44.95 | | 510.0865 | 9.69205 | 609.2578 | 176.8516 |  |  |  |  |  |
| 44.95833 | | 510.1696 | 9.683995 | 607.8125 | 176.6289 |  |  |  |  |  |
| 44.96667 | | 510.2527 | 9.675456 | 606.3047 | 176.5195 |  |  |  |  |  |
| 44.975 | | 510.3392 | 9.672783 | 604.8281 | 176.4023 |  |  |  |  |  |
| 44.98333 | | 510.4264 | 9.665494 | 603.3594 | 176.2539 |  |  |  |  |  |
| 44.99167 | | 510.5081 | 9.656709 | 601.8984 | 176.0898 |  |  |  |  |  |
| 45 | | 510.5939 | 9.653081 | 600.4141 | 175.8516 |  |  |  |  |  |
| 45.00833 | | 510.6784 | 9.646862 | 598.9688 | 175.4727 |  |  |  |  |  |
| 45.01667 | | 510.7656 | 9.637614 | 597.5078 | 175.2539 |  |  |  |  |  |
| 45.025 | | 510.8487 | 9.632985 | 596.0547 | 175.5508 |  |  |  |  |  |
| 45.03333 | | 510.9315 | 9.627646 | 594.6016 | 175.4531 |  |  |  |  |  |
| 45.04167 | | 511.015 | 9.618926 | 593.1484 | 175.5664 |  |  |  |  |  |
| 45.05 | | 511.1004 | 9.614213 | 591.7109 | 175.4844 |  |  |  |  |  |
| 45.05833 | | 511.1859 | 9.610374 | 590.2969 | 175.6367 |  |  |  |  |  |
| 45.06667 | | 511.2679 | 9.602367 | 588.8594 | 175.6172 |  |  |  |  |  |
| 45.075 | | 511.3533 | 9.598639 | 587.3516 | 175.7227 |  |  |  |  |  |
| 45.08333 | | 511.4354 | 9.597635 | 585.8984 | 175.7227 |  |  |  |  |  |
| 45.09167 | | 511.5208 | 9.591642 | 584.4219 | 176.0039 |  |  |  |  |  |
| 45.1 | | 511.6029 | 9.58998 | 582.9688 | 176.3516 |  |  |  |  |  |
| 45.10833 | | 511.6904 | 9.591923 | 581.4766 | 176.2813 |  |  |  |  |  |
| 45.11667 | | 511.7771 | 9.587656 | 580.0234 | 176.2578 |  |  |  |  |  |
| 45.125 | | 511.8633 | 9.588037 | 578.5469 | 176.1328 |  |  |  |  |  |
| 45.13333 | | 511.9493 | 9.590936 | 577.0781 | 176.2305 |  |  |  |  |  |
| 45.14167 | | 512.0321 | 9.587441 | 575.5703 | 176.1367 |  |  |  |  |  |
| 45.15 | | 512.1155 | 9.586917 | 574.0625 | 175.957 |  |  |  |  |  |
| 45.15833 | | 512.2003 | 9.590972 | 572.5938 | 175.9258 |  |  |  |  |  |
| 45.16667 | | 512.287 | 9.586297 | 571.1328 | 176.0156 |  |  |  |  |  |
| 45.175 | | 512.3718 | 9.585562 | 569.6797 | 175.7148 |  |  |  |  |  |
| 45.18333 | | 512.4565 | 9.588313 | 568.1953 | 175.4727 |  |  |  |  |  |
| 45.19167 | | 512.5393 | 9.582437 | 566.7422 | 175.1016 |  |  |  |  |  |
| 45.2 | | 512.6227 | 9.58095 | 565.2891 | 174.8203 |  |  |  |  |  |
| 45.20833 | | 512.7075 | 9.583208 | 563.8281 | 174.6992 |  |  |  |  |  |
| 45.21667 | | 512.7922 | 9.576515 | 562.3594 | 174.3008 |  |  |  |  |  |
| 45.225 | | 512.8736 | 9.573771 | 560.9297 | 173.9102 |  |  |  |  |  |
| 45.23333 | | 512.9564 | 9.576209 | 559.5 | 173.6445 |  |  |  |  |  |
| 45.24167 | | 513.0398 | 9.568613 | 558.0859 | 173.1875 |  |  |  |  |  |
| 45.25 | | 513.1252 | 9.565128 | 556.6797 | 172.9883 |  |  |  |  |  |
| 45.25833 | | 513.21 | 9.566271 | 555.2422 | 172.9219 |  |  |  |  |  |
| 45.26667 | | 513.2947 | 9.558628 | 553.8438 | 172.7031 |  |  |  |  |  |
| 45.275 | | 513.3795 | 9.555736 | 552.4453 | 172.4414 |  |  |  |  |  |
| 45.28333 | | 513.4642 | 9.556409 | 551.0391 | 172.4297 |  |  |  |  |  |
| 45.29167 | | 513.5497 | 9.549184 | 549.6563 | 172.1719 |  |  |  |  |  |
| 45.3 | | 513.6344 | 9.545114 | 548.2344 | 172.0977 |  |  |  |  |  |
| 45.30833 | | 513.7225 | 9.545093 | 546.8047 | 172.1055 |  |  |  |  |  |
| 45.31667 | | 513.8093 | 9.537098 | 545.3828 | 171.9531 |  |  |  |  |  |
| 45.325 | | 513.8921 | 9.532496 | 543.9844 | 172.082 |  |  |  |  |  |
| 45.33333 | | 513.9768 | 9.531695 | 542.5391 | 171.9492 |  |  |  |  |  |
| 45.34167 | | 514.0602 | 9.523378 | 541.1328 | 171.8594 |  |  |  |  |  |
| 45.35 | | 514.1464 | 9.51834 | 539.7109 | 171.5625 |  |  |  |  |  |
| 45.35833 | | 514.2284 | 9.516242 | 538.2813 | 171.6641 |  |  |  |  |  |
| 45.36667 | | 514.3125 | 9.508074 | 536.8594 | 171.4414 |  |  |  |  |  |
| 45.375 | | 514.3959 | 9.502235 | 535.3984 | 171.2188 |  |  |  |  |  |
| 45.38333 | | 514.4793 | 9.499689 | 533.9922 | 171.293 |  |  |  |  |  |
| 45.39167 | | 514.5627 | 9.491678 | 532.5625 | 171.4063 |  |  |  |  |  |
| 45.4 | | 514.6469 | 9.484978 | 531.1797 | 171.4844 |  |  |  |  |  |
| 45.40833 | | 514.7343 | 9.482516 | 529.7344 | 171.2227 |  |  |  |  |  |
| 45.41667 | | 514.815 | 9.474666 | 528.3359 | 171 |  |  |  |  |  |
| 45.425 | | 514.9011 | 9.467777 | 526.9297 | 170.6055 |  |  |  |  |  |
| 45.43333 | | 514.9852 | 9.464767 | 525.4844 | 170.8242 |  |  |  |  |  |
| 45.44167 | | 515.0726 | 9.457206 | 524.0313 | 170.6563 |  |  |  |  |  |
| 45.45 | | 515.1561 | 9.450012 | 522.6016 | 170.5195 |  |  |  |  |  |
| 45.45833 | | 515.2394 | 9.446353 | 521.2031 | 170.5 |  |  |  |  |  |
| 45.46667 | | 515.3242 | 9.438652 | 519.7969 | 170.2188 |  |  |  |  |  |
| 45.475 | | 515.4097 | 9.430482 | 518.4297 | 170.0273 |  |  |  |  |  |
| 45.48333 | | 515.4944 | 9.426394 | 516.9688 | 169.6523 |  |  |  |  |  |
| 45.49167 | | 515.5785 | 9.41907 | 515.5625 | 169.3516 |  |  |  |  |  |
| 45.5 | | 515.6653 | 9.410147 | 514.1484 | 169.375 |  |  |  |  |  |
| 45.50833 | | 515.7467 | 9.406077 | 512.7188 | 169.7188 |  |  |  |  |  |
| 45.51667 | | 515.8287 | 9.39981 | 511.3359 | 169.5508 |  |  |  |  |  |
| 45.525 | | 515.9095 | 9.389595 | 509.9531 | 169.3672 |  |  |  |  |  |
| 45.53333 | | 515.9929 | 9.385956 | 508.5781 | 169.4531 |  |  |  |  |  |
| 45.54167 | | 516.0763 | 9.380524 | 507.1953 | 169.3789 |  |  |  |  |  |
| 45.55 | | 516.157 | 9.369405 | 505.7734 | 169.3945 |  |  |  |  |  |
| 45.55833 | | 516.2384 | 9.365572 | 504.3047 | 169.2344 |  |  |  |  |  |
| 45.56667 | | 516.3198 | 9.36154 | 502.9219 | 169.2734 |  |  |  |  |  |
| 45.575 | | 516.4019 | 9.350002 | 501.5234 | 169.4414 |  |  |  |  |  |
| 45.58333 | | 516.4833 | 9.347075 | 500.1016 | 169.1563 |  |  |  |  |  |
| 45.59167 | | 516.5687 | 9.344013 | 498.6953 | 169.0039 |  |  |  |  |  |
| 45.6 | | 516.6514 | 9.333507 | 497.2891 | 168.8555 |  |  |  |  |  |
| 45.60833 | | 516.7362 | 9.329929 | 495.8984 | 168.6719 |  |  |  |  |  |
| 45.61667 | | 516.8203 | 9.325452 | 494.4766 | 168.6094 |  |  |  |  |  |
| 45.625 | | 516.9037 | 9.31485 | 493.0469 | 168.4844 |  |  |  |  |  |
| 45.63333 | | 516.9891 | 9.31046 | 491.6719 | 168.2891 |  |  |  |  |  |
| 45.64167 | | 517.0732 | 9.306333 | 490.2969 | 168.043 |  |  |  |  |  |
| 45.65 | | 517.1586 | 9.295723 | 488.9063 | 167.9297 |  |  |  |  |  |
| 45.65833 | | 517.2421 | 9.291302 | 487.5234 | 167.8359 |  |  |  |  |  |
| 45.66667 | | 517.3275 | 9.286584 | 486.125 | 167.9258 |  |  |  |  |  |
| 45.675 | | 517.4089 | 9.275575 | 484.7344 | 167.4766 |  |  |  |  |  |
| 45.68333 | | 517.493 | 9.270006 | 483.3594 | 167.4297 |  |  |  |  |  |
| 45.69167 | | 517.5764 | 9.264551 | 481.9844 | 167.4023 |  |  |  |  |  |
| 45.7 | | 517.6584 | 9.254206 | 480.6016 | 167.1758 |  |  |  |  |  |
| 45.70833 | | 517.7405 | 9.247537 | 479.2109 | 166.8633 |  |  |  |  |  |
| 45.71667 | | 517.8239 | 9.242502 | 477.8047 | 166.7344 |  |  |  |  |  |
| 45.725 | | 517.9087 | 9.232735 | 476.4688 | 166.6641 |  |  |  |  |  |
| 45.73333 | | 517.9901 | 9.225353 | 475.0703 | 166.3867 |  |  |  |  |  |
| 45.74167 | | 518.0728 | 9.220844 | 473.6797 | 165.9883 |  |  |  |  |  |
| 45.75 | | 518.1536 | 9.212068 | 472.3203 | 165.8047 |  |  |  |  |  |
| 45.75833 | | 518.2377 | 9.202919 | 470.9609 | 165.707 |  |  |  |  |  |
| 45.76667 | | 518.3222 | 9.198799 | 469.5859 | 165.207 |  |  |  |  |  |
| 45.775 | | 518.4059 | 9.190073 | 468.2109 | 164.9648 |  |  |  |  |  |
| 45.78333 | | 518.4918 | 9.179487 | 466.8594 | 164.7813 |  |  |  |  |  |
| 45.79167 | | 518.5776 | 9.174918 | 465.5156 | 164.6211 |  |  |  |  |  |
| 45.8 | | 518.6607 | 9.166421 | 464.1641 | 164.5977 |  |  |  |  |  |
| 45.80833 | | 518.7424 | 9.155269 | 462.7891 | 164.582 |  |  |  |  |  |
| 45.81667 | | 518.8276 | 9.152051 | 461.4766 | 164.4453 |  |  |  |  |  |
| 45.825 | | 518.9113 | 9.144674 | 460.1406 | 164.5469 |  |  |  |  |  |
| 45.83333 | | 518.9979 | 9.132097 | 458.7891 | 164.5039 |  |  |  |  |  |
| 45.84167 | | 519.0803 | 9.128608 | 457.4297 | 164.5781 |  |  |  |  |  |
| 45.85 | | 519.162 | 9.120355 | 456.0547 | 164.5859 |  |  |  |  |  |
| 45.85833 | | 519.2458 | 9.108031 | 454.6797 | 164.5547 |  |  |  |  |  |
| 45.86667 | | 519.3316 | 9.103264 | 453.3281 | 164.7852 |  |  |  |  |  |
| 45.875 | | 519.4167 | 9.096 | 451.9375 | 164.7109 |  |  |  |  |  |
| 45.88333 | | 519.5005 | 9.08468 | 450.5703 | 164.7109 |  |  |  |  |  |
| 45.89167 | | 519.5856 | 9.078805 | 449.1875 | 164.8438 |  |  |  |  |  |
| 45.9 | | 519.6708 | 9.071851 | 447.8125 | 164.9844 |  |  |  |  |  |
| 45.90833 | | 519.758 | 9.060639 | 446.4297 | 164.7734 |  |  |  |  |  |
| 45.91667 | | 519.8417 | 9.054172 | 445.0313 | 164.9492 |  |  |  |  |  |
| 45.925 | | 519.9282 | 9.047829 | 443.6563 | 165.0352 |  |  |  |  |  |
| 45.93333 | | 520.0161 | 9.036839 | 442.2813 | 164.9766 |  |  |  |  |  |
[truncated: 122,105 more chars]
