# Supplementary material for: Characterization dataset for pre- and post-irradiated shrimp waste chitosan
Source: Data Brief. 2020 Jul 25;32:106081. doi: 10.1016/j.dib.2020.106081 (PMC7397402; doi:10.1016/j.dib.2020.106081)
Supplement: Supplementary file 1 [file mmc1.zip › XRD-CH20.docx]

| [Measurement conditions] | | |
| --- | --- | --- |
| Sample identification | |  |
| Comment - 1 | Configuration=flat sample stage, Owner=User-1, Creation date=7/16/2007 2:29:55 PM | |
| Comment - 2 | Goniometer=PW3050/60 (Theta/Theta); Minimum step size 2Theta:0.001; Minimum step size Omega:0.001 | |
| Comment - 3 | Sample stage=PW3071/xx Bracket | |
| Comment - 4 | Diffractometer system=XPERT-PRO | |
| Comment - 5 | Measurement program=NORMAL PHASE PRS, Owner=User-1, Creation date=7/16/2007 3:30:03 PM | |
| Anode material | Cu |  |
| K-Alpha1 wavelength | 1.540598 |  |
| K-Alpha2 wavelength | 1.544426 |  |
| Ratio K-Alpha2/K-Alpha1 | 0.5 |  |
| Divergence slit | Fixed | 1 |
| Receiving slit | 0.3 |  |
| Monochromator used | NO |  |
| Generator voltage | 40 |  |
| Tube current | 30 |  |
| File date and time | 03-10-16 10:48 |  |
| Unit cell |  |  |
| h k l | 0 0 0 |  |
| Scan axis | Gonio |  |
| Scan range | 5.01 | 45 |
| Scan step size | 0.03 |  |
| No. of points | 1333 |  |
| Scan type | CONTINUOUS |  |
| Time per step | 0.5 |  |
| [Scan points] | |  |
| Angle | Intensity |  |
| 5.025 | 138 |  |
| 5.055 | 151 |  |
| 5.085 | 130 |  |
| 5.115 | 141 |  |
| 5.145 | 134 |  |
| 5.175 | 116 |  |
| 5.205 | 138 |  |
| 5.235 | 143 |  |
| 5.265 | 114 |  |
| 5.295 | 131 |  |
| 5.325 | 124 |  |
| 5.355 | 133 |  |
| 5.385 | 139 |  |
| 5.415 | 147 |  |
| 5.445 | 130 |  |
| 5.475 | 139 |  |
| 5.505 | 136 |  |
| 5.535 | 140 |  |
| 5.565 | 127 |  |
| 5.595 | 143 |  |
| 5.625 | 130 |  |
| 5.655 | 145 |  |
| 5.685 | 128 |  |
| 5.715 | 151 |  |
| 5.745 | 142 |  |
| 5.775 | 139 |  |
| 5.805 | 125 |  |
| 5.835 | 144 |  |
| 5.865 | 129 |  |
| 5.895 | 150 |  |
| 5.925 | 129 |  |
| 5.955 | 154 |  |
| 5.985 | 152 |  |
| 6.015 | 124 |  |
| 6.045 | 135 |  |
| 6.075 | 134 |  |
| 6.105 | 143 |  |
| 6.135 | 125 |  |
| 6.165 | 133 |  |
| 6.195 | 140 |  |
| 6.225 | 149 |  |
| 6.255 | 122 |  |
| 6.285 | 153 |  |
| 6.315 | 112 |  |
| 6.345 | 118 |  |
| 6.375 | 124 |  |
| 6.405 | 126 |  |
| 6.435 | 127 |  |
| 6.465 | 141 |  |
| 6.495 | 142 |  |
| 6.525 | 131 |  |
| 6.555 | 123 |  |
| 6.585 | 140 |  |
| 6.615 | 138 |  |
| 6.645 | 168 |  |
| 6.675 | 139 |  |
| 6.705 | 147 |  |
| 6.735 | 137 |  |
| 6.765 | 156 |  |
| 6.795 | 149 |  |
| 6.825 | 139 |  |
| 6.855 | 148 |  |
| 6.885 | 143 |  |
| 6.915 | 148 |  |
| 6.945 | 156 |  |
| 6.975 | 160 |  |
| 7.005 | 160 |  |
| 7.035 | 151 |  |
| 7.065 | 161 |  |
| 7.095 | 162 |  |
| 7.125 | 151 |  |
| 7.155 | 155 |  |
| 7.185 | 173 |  |
| 7.215 | 175 |  |
| 7.245 | 169 |  |
| 7.275 | 185 |  |
| 7.305 | 164 |  |
| 7.335 | 183 |  |
| 7.365 | 181 |  |
| 7.395 | 162 |  |
| 7.425 | 176 |  |
| 7.455 | 186 |  |
| 7.485 | 196 |  |
| 7.515 | 188 |  |
| 7.545 | 207 |  |
| 7.575 | 194 |  |
| 7.605 | 180 |  |
| 7.635 | 214 |  |
| 7.665 | 212 |  |
| 7.695 | 233 |  |
| 7.725 | 252 |  |
| 7.755 | 198 |  |
| 7.785 | 192 |  |
| 7.815 | 234 |  |
| 7.845 | 233 |  |
| 7.875 | 225 |  |
| 7.905 | 221 |  |
| 7.935 | 237 |  |
| 7.965 | 263 |  |
| 7.995 | 267 |  |
| 8.025 | 243 |  |
| 8.055 | 264 |  |
| 8.085 | 263 |  |
| 8.115 | 245 |  |
| 8.145 | 261 |  |
| 8.175 | 302 |  |
| 8.205 | 290 |  |
| 8.235 | 274 |  |
| 8.265 | 325 |  |
| 8.295 | 297 |  |
| 8.325 | 332 |  |
| 8.355 | 325 |  |
| 8.385 | 311 |  |
| 8.415 | 306 |  |
| 8.445 | 296 |  |
| 8.475 | 272 |  |
| 8.505 | 324 |  |
| 8.535 | 357 |  |
| 8.565 | 291 |  |
| 8.595 | 355 |  |
| 8.625 | 320 |  |
| 8.655 | 342 |  |
| 8.685 | 319 |  |
| 8.715 | 352 |  |
| 8.745 | 317 |  |
| 8.775 | 354 |  |
| 8.805 | 362 |  |
| 8.835 | 376 |  |
| 8.865 | 351 |  |
| 8.895 | 376 |  |
| 8.925 | 346 |  |
| 8.955 | 343 |  |
| 8.985 | 326 |  |
| 9.015 | 327 |  |
| 9.045 | 344 |  |
| 9.075 | 352 |  |
| 9.105 | 317 |  |
| 9.135 | 376 |  |
| 9.165 | 336 |  |
| 9.195 | 345 |  |
| 9.225 | 326 |  |
| 9.255 | 354 |  |
| 9.285 | 321 |  |
| 9.315 | 321 |  |
| 9.345 | 337 |  |
| 9.375 | 343 |  |
| 9.405 | 370 |  |
| 9.435 | 329 |  |
| 9.465 | 333 |  |
| 9.495 | 311 |  |
| 9.525 | 315 |  |
| 9.555 | 333 |  |
| 9.585 | 307 |  |
| 9.615 | 305 |  |
| 9.645 | 269 |  |
| 9.675 | 286 |  |
| 9.705 | 277 |  |
| 9.735 | 278 |  |
| 9.765 | 227 |  |
| 9.795 | 276 |  |
| 9.825 | 221 |  |
| 9.855 | 261 |  |
| 9.885 | 228 |  |
| 9.915 | 229 |  |
| 9.945 | 222 |  |
| 9.975 | 233 |  |
| 10.005 | 232 |  |
| 10.035 | 221 |  |
| 10.065 | 253 |  |
| 10.095 | 211 |  |
| 10.125 | 213 |  |
| 10.155 | 209 |  |
| 10.185 | 213 |  |
| 10.215 | 207 |  |
| 10.245 | 193 |  |
| 10.275 | 199 |  |
| 10.305 | 199 |  |
| 10.335 | 181 |  |
| 10.365 | 229 |  |
| 10.395 | 187 |  |
| 10.425 | 203 |  |
| 10.455 | 188 |  |
| 10.485 | 199 |  |
| 10.515 | 206 |  |
| 10.545 | 183 |  |
| 10.575 | 181 |  |
| 10.605 | 197 |  |
| 10.635 | 178 |  |
| 10.665 | 181 |  |
| 10.695 | 174 |  |
| 10.725 | 191 |  |
| 10.755 | 185 |  |
| 10.785 | 187 |  |
| 10.815 | 171 |  |
| 10.845 | 173 |  |
| 10.875 | 178 |  |
| 10.905 | 191 |  |
| 10.935 | 160 |  |
| 10.965 | 174 |  |
| 10.995 | 190 |  |
| 11.025 | 159 |  |
| 11.055 | 196 |  |
| 11.085 | 154 |  |
| 11.115 | 177 |  |
| 11.145 | 185 |  |
| 11.175 | 170 |  |
| 11.205 | 161 |  |
| 11.235 | 156 |  |
| 11.265 | 148 |  |
| 11.295 | 149 |  |
| 11.325 | 201 |  |
| 11.355 | 170 |  |
| 11.385 | 181 |  |
| 11.415 | 157 |  |
| 11.445 | 147 |  |
| 11.475 | 173 |  |
| 11.505 | 158 |  |
| 11.535 | 158 |  |
| 11.565 | 159 |  |
| 11.595 | 161 |  |
| 11.625 | 150 |  |
| 11.655 | 153 |  |
| 11.685 | 143 |  |
| 11.715 | 179 |  |
| 11.745 | 165 |  |
| 11.775 | 164 |  |
| 11.805 | 158 |  |
| 11.835 | 158 |  |
| 11.865 | 154 |  |
| 11.895 | 155 |  |
| 11.925 | 141 |  |
| 11.955 | 149 |  |
| 11.985 | 173 |  |
| 12.015 | 154 |  |
| 12.045 | 151 |  |
| 12.075 | 158 |  |
| 12.105 | 142 |  |
| 12.135 | 156 |  |
| 12.165 | 146 |  |
| 12.195 | 152 |  |
| 12.225 | 148 |  |
| 12.255 | 144 |  |
| 12.285 | 177 |  |
| 12.315 | 171 |  |
| 12.345 | 144 |  |
| 12.375 | 133 |  |
| 12.405 | 148 |  |
| 12.435 | 152 |  |
| 12.465 | 163 |  |
| 12.495 | 150 |  |
| 12.525 | 158 |  |
| 12.555 | 159 |  |
| 12.585 | 137 |  |
| 12.615 | 143 |  |
| 12.645 | 150 |  |
| 12.675 | 157 |  |
| 12.705 | 148 |  |
| 12.735 | 154 |  |
| 12.765 | 159 |  |
| 12.795 | 158 |  |
| 12.825 | 148 |  |
| 12.855 | 143 |  |
| 12.885 | 152 |  |
| 12.915 | 186 |  |
| 12.945 | 139 |  |
| 12.975 | 158 |  |
| 13.005 | 166 |  |
| 13.035 | 138 |  |
| 13.065 | 162 |  |
| 13.095 | 171 |  |
| 13.125 | 176 |  |
| 13.155 | 143 |  |
| 13.185 | 182 |  |
| 13.215 | 154 |  |
| 13.245 | 147 |  |
| 13.275 | 152 |  |
| 13.305 | 150 |  |
| 13.335 | 154 |  |
| 13.365 | 162 |  |
| 13.395 | 164 |  |
| 13.425 | 167 |  |
| 13.455 | 169 |  |
| 13.485 | 156 |  |
| 13.515 | 143 |  |
| 13.545 | 168 |  |
| 13.575 | 174 |  |
| 13.605 | 149 |  |
| 13.635 | 147 |  |
| 13.665 | 175 |  |
| 13.695 | 159 |  |
| 13.725 | 149 |  |
| 13.755 | 158 |  |
| 13.785 | 158 |  |
| 13.815 | 156 |  |
| 13.845 | 153 |  |
| 13.875 | 150 |  |
| 13.905 | 124 |  |
| 13.935 | 120 |  |
| 13.965 | 143 |  |
| 13.995 | 159 |  |
| 14.025 | 151 |  |
| 14.055 | 151 |  |
| 14.085 | 172 |  |
| 14.115 | 169 |  |
| 14.145 | 150 |  |
| 14.175 | 148 |  |
| 14.205 | 163 |  |
| 14.235 | 171 |  |
| 14.265 | 147 |  |
| 14.295 | 160 |  |
| 14.325 | 156 |  |
| 14.355 | 171 |  |
| 14.385 | 161 |  |
| 14.415 | 143 |  |
| 14.445 | 135 |  |
| 14.475 | 139 |  |
| 14.505 | 140 |  |
| 14.535 | 164 |  |
| 14.565 | 145 |  |
| 14.595 | 135 |  |
| 14.625 | 156 |  |
| 14.655 | 154 |  |
| 14.685 | 146 |  |
| 14.715 | 134 |  |
| 14.745 | 155 |  |
| 14.775 | 183 |  |
| 14.805 | 150 |  |
| 14.835 | 158 |  |
| 14.865 | 175 |  |
| 14.895 | 156 |  |
| 14.925 | 164 |  |
| 14.955 | 138 |  |
| 14.985 | 162 |  |
| 15.015 | 165 |  |
| 15.045 | 170 |  |
| 15.075 | 152 |  |
| 15.105 | 152 |  |
| 15.135 | 167 |  |
| 15.165 | 169 |  |
| 15.195 | 166 |  |
| 15.225 | 168 |  |
| 15.255 | 149 |  |
| 15.285 | 169 |  |
| 15.315 | 152 |  |
| 15.345 | 136 |  |
| 15.375 | 167 |  |
| 15.405 | 160 |  |
| 15.435 | 156 |  |
| 15.465 | 148 |  |
| 15.495 | 145 |  |
| 15.525 | 159 |  |
| 15.555 | 169 |  |
| 15.585 | 131 |  |
| 15.615 | 163 |  |
| 15.645 | 164 |  |
| 15.675 | 163 |  |
| 15.705 | 141 |  |
| 15.735 | 166 |  |
| 15.765 | 161 |  |
| 15.795 | 173 |  |
| 15.825 | 173 |  |
| 15.855 | 132 |  |
| 15.885 | 167 |  |
| 15.915 | 149 |  |
| 15.945 | 145 |  |
| 15.975 | 155 |  |
| 16.005 | 167 |  |
| 16.035 | 188 |  |
| 16.065 | 150 |  |
| 16.095 | 184 |  |
| 16.125 | 159 |  |
| 16.155 | 174 |  |
| 16.185 | 162 |  |
| 16.215 | 135 |  |
| 16.245 | 155 |  |
| 16.275 | 184 |  |
| 16.305 | 156 |  |
| 16.335 | 189 |  |
| 16.365 | 155 |  |
| 16.395 | 156 |  |
| 16.425 | 168 |  |
| 16.455 | 171 |  |
| 16.485 | 153 |  |
| 16.515 | 160 |  |
| 16.545 | 155 |  |
| 16.575 | 155 |  |
| 16.605 | 189 |  |
| 16.635 | 170 |  |
| 16.665 | 159 |  |
| 16.695 | 168 |  |
| 16.725 | 152 |  |
| 16.755 | 156 |  |
| 16.785 | 176 |  |
| 16.815 | 204 |  |
| 16.845 | 176 |  |
| 16.875 | 197 |  |
| 16.905 | 189 |  |
| 16.935 | 182 |  |
| 16.965 | 162 |  |
| 16.995 | 182 |  |
| 17.025 | 179 |  |
| 17.055 | 170 |  |
| 17.085 | 164 |  |
| 17.115 | 183 |  |
| 17.145 | 187 |  |
| 17.175 | 189 |  |
| 17.205 | 185 |  |
| 17.235 | 176 |  |
| 17.265 | 201 |  |
| 17.295 | 204 |  |
| 17.325 | 172 |  |
| 17.355 | 184 |  |
| 17.385 | 199 |  |
| 17.415 | 200 |  |
| 17.445 | 194 |  |
| 17.475 | 210 |  |
| 17.505 | 184 |  |
| 17.535 | 191 |  |
| 17.565 | 204 |  |
| 17.595 | 212 |  |
| 17.625 | 197 |  |
| 17.655 | 216 |  |
| 17.685 | 207 |  |
| 17.715 | 224 |  |
| 17.745 | 201 |  |
| 17.775 | 218 |  |
| 17.805 | 184 |  |
| 17.835 | 230 |  |
| 17.865 | 202 |  |
| 17.895 | 237 |  |
| 17.925 | 207 |  |
| 17.955 | 226 |  |
| 17.985 | 217 |  |
| 18.015 | 243 |  |
| 18.045 | 236 |  |
| 18.075 | 238 |  |
| 18.105 | 247 |  |
| 18.135 | 253 |  |
| 18.165 | 230 |  |
| 18.195 | 254 |  |
| 18.225 | 249 |  |
| 18.255 | 249 |  |
| 18.285 | 258 |  |
| 18.315 | 242 |  |
| 18.345 | 244 |  |
| 18.375 | 251 |  |
| 18.405 | 244 |  |
| 18.435 | 239 |  |
| 18.465 | 253 |  |
| 18.495 | 251 |  |
| 18.525 | 276 |  |
| 18.555 | 289 |  |
| 18.585 | 272 |  |
| 18.615 | 253 |  |
| 18.645 | 295 |  |
| 18.675 | 325 |  |
| 18.705 | 297 |  |
| 18.735 | 292 |  |
| 18.765 | 317 |  |
| 18.795 | 303 |  |
| 18.825 | 300 |  |
| 18.855 | 337 |  |
| 18.885 | 324 |  |
| 18.915 | 339 |  |
| 18.945 | 309 |  |
| 18.975 | 338 |  |
| 19.005 | 317 |  |
| 19.035 | 382 |  |
| 19.065 | 434 |  |
| 19.095 | 384 |  |
| 19.125 | 407 |  |
| 19.155 | 373 |  |
| 19.185 | 432 |  |
| 19.215 | 456 |  |
| 19.245 | 444 |  |
| 19.275 | 434 |  |
| 19.305 | 421 |  |
| 19.335 | 442 |  |
| 19.365 | 424 |  |
| 19.395 | 503 |  |
| 19.425 | 526 |  |
| 19.455 | 491 |  |
| 19.485 | 512 |  |
| 19.515 | 532 |  |
| 19.545 | 522 |  |
| 19.575 | 542 |  |
| 19.605 | 548 |  |
| 19.635 | 567 |  |
| 19.665 | 589 |  |
| 19.695 | 583 |  |
| 19.725 | 544 |  |
| 19.755 | 563 |  |
| 19.785 | 575 |  |
| 19.815 | 609 |  |
| 19.845 | 559 |  |
| 19.875 | 585 |  |
| 19.905 | 603 |  |
| 19.935 | 642 |  |
| 19.965 | 605 |  |
| 19.995 | 614 |  |
| 20.025 | 637 |  |
| 20.055 | 605 |  |
| 20.085 | 659 |  |
| 20.115 | 632 |  |
| 20.145 | 619 |  |
| 20.175 | 558 |  |
| 20.205 | 570 |  |
| 20.235 | 563 |  |
| 20.265 | 590 |  |
| 20.295 | 629 |  |
| 20.325 | 549 |  |
| 20.355 | 559 |  |
| 20.385 | 565 |  |
| 20.415 | 602 |  |
| 20.445 | 522 |  |
| 20.475 | 563 |  |
| 20.505 | 533 |  |
| 20.535 | 509 |  |
| 20.565 | 526 |  |
| 20.595 | 474 |  |
| 20.625 | 506 |  |
| 20.655 | 491 |  |
| 20.685 | 508 |  |
| 20.715 | 485 |  |
| 20.745 | 481 |  |
| 20.775 | 483 |  |
| 20.805 | 469 |  |
| 20.835 | 500 |  |
| 20.865 | 490 |  |
| 20.895 | 440 |  |
| 20.925 | 453 |  |
| 20.955 | 473 |  |
| 20.985 | 483 |  |
| 21.015 | 488 |  |
| 21.045 | 474 |  |
| 21.075 | 481 |  |
| 21.105 | 460 |  |
| 21.135 | 443 |  |
| 21.165 | 420 |  |
| 21.195 | 449 |  |
| 21.225 | 418 |  |
| 21.255 | 456 |  |
| 21.285 | 400 |  |
| 21.315 | 415 |  |
| 21.345 | 406 |  |
| 21.375 | 409 |  |
| 21.405 | 442 |  |
| 21.435 | 427 |  |
| 21.465 | 408 |  |
| 21.495 | 390 |  |
| 21.525 | 406 |  |
| 21.555 | 412 |  |
| 21.585 | 425 |  |
| 21.615 | 415 |  |
| 21.645 | 380 |  |
| 21.675 | 399 |  |
| 21.705 | 380 |  |
| 21.735 | 398 |  |
| 21.765 | 360 |  |
| 21.795 | 388 |  |
| 21.825 | 363 |  |
| 21.855 | 420 |  |
| 21.885 | 366 |  |
| 21.915 | 366 |  |
| 21.945 | 387 |  |
| 21.975 | 361 |  |
| 22.005 | 379 |  |
| 22.035 | 361 |  |
| 22.065 | 361 |  |
| 22.095 | 366 |  |
| 22.125 | 348 |  |
| 22.155 | 365 |  |
| 22.185 | 334 |  |
| 22.215 | 374 |  |
| 22.245 | 316 |  |
| 22.275 | 349 |  |
| 22.305 | 373 |  |
| 22.335 | 363 |  |
| 22.365 | 341 |  |
| 22.395 | 327 |  |
| 22.425 | 296 |  |
| 22.455 | 325 |  |
| 22.485 | 329 |  |
| 22.515 | 339 |  |
| 22.545 | 308 |  |
| 22.575 | 345 |  |
| 22.605 | 295 |  |
| 22.635 | 319 |  |
| 22.665 | 332 |  |
| 22.695 | 311 |  |
| 22.725 | 300 |  |
| 22.755 | 293 |  |
| 22.785 | 280 |  |
| 22.815 | 304 |  |
| 22.845 | 298 |  |
| 22.875 | 286 |  |
| 22.905 | 288 |  |
| 22.935 | 288 |  |
| 22.965 | 280 |  |
| 22.995 | 304 |  |
| 23.025 | 268 |  |
| 23.055 | 273 |  |
| 23.085 | 307 |  |
| 23.115 | 286 |  |
| 23.145 | 300 |  |
| 23.175 | 257 |  |
| 23.205 | 302 |  |
| 23.235 | 281 |  |
| 23.265 | 291 |  |
| 23.295 | 256 |  |
| 23.325 | 266 |  |
| 23.355 | 276 |  |
| 23.385 | 243 |  |
| 23.415 | 241 |  |
| 23.445 | 256 |  |
| 23.475 | 268 |  |
| 23.505 | 274 |  |
| 23.535 | 250 |  |
| 23.565 | 229 |  |
| 23.595 | 271 |  |
| 23.625 | 254 |  |
| 23.655 | 254 |  |
| 23.685 | 287 |  |
| 23.715 | 282 |  |
| 23.745 | 260 |  |
| 23.775 | 234 |  |
| 23.805 | 250 |  |
| 23.835 | 254 |  |
| 23.865 | 251 |  |
| 23.895 | 257 |  |
| 23.925 | 261 |  |
| 23.955 | 241 |  |
| 23.985 | 226 |  |
| 24.015 | 260 |  |
| 24.045 | 235 |  |
| 24.075 | 236 |  |
| 24.105 | 214 |  |
| 24.135 | 247 |  |
| 24.165 | 242 |  |
| 24.195 | 266 |  |
| 24.225 | 252 |  |
| 24.255 | 228 |  |
| 24.285 | 238 |  |
| 24.315 | 262 |  |
| 24.345 | 248 |  |
| 24.375 | 258 |  |
| 24.405 | 265 |  |
| 24.435 | 239 |  |
| 24.465 | 250 |  |
| 24.495 | 223 |  |
| 24.525 | 239 |  |
| 24.555 | 206 |  |
| 24.585 | 247 |  |
| 24.615 | 213 |  |
| 24.645 | 213 |  |
| 24.675 | 232 |  |
| 24.705 | 239 |  |
| 24.735 | 276 |  |
| 24.765 | 229 |  |
| 24.795 | 269 |  |
| 24.825 | 249 |  |
| 24.855 | 220 |  |
| 24.885 | 208 |  |
| 24.915 | 252 |  |
| 24.945 | 227 |  |
| 24.975 | 233 |  |
| 25.005 | 243 |  |
| 25.035 | 235 |  |
| 25.065 | 244 |  |
| 25.095 | 255 |  |
| 25.125 | 241 |  |
| 25.155 | 221 |  |
| 25.185 | 253 |  |
| 25.215 | 244 |  |
| 25.245 | 268 |  |
| 25.275 | 273 |  |
| 25.305 | 189 |  |
| 25.335 | 245 |  |
| 25.365 | 239 |  |
| 25.395 | 242 |  |
| 25.425 | 246 |  |
| 25.455 | 219 |  |
| 25.485 | 231 |  |
| 25.515 | 265 |  |
| 25.545 | 251 |  |
| 25.575 | 229 |  |
| 25.605 | 224 |  |
| 25.635 | 225 |  |
| 25.665 | 236 |  |
| 25.695 | 256 |  |
| 25.725 | 219 |  |
| 25.755 | 243 |  |
| 25.785 | 232 |  |
| 25.815 | 241 |  |
| 25.845 | 213 |  |
| 25.875 | 209 |  |
| 25.905 | 228 |  |
| 25.935 | 241 |  |
| 25.965 | 225 |  |
| 25.995 | 240 |  |
| 26.025 | 274 |  |
| 26.055 | 256 |  |
| 26.085 | 249 |  |
| 26.115 | 255 |  |
| 26.145 | 256 |  |
| 26.175 | 243 |  |
| 26.205 | 244 |  |
| 26.235 | 248 |  |
| 26.265 | 259 |  |
| 26.295 | 283 |  |
| 26.325 | 237 |  |
| 26.355 | 250 |  |
| 26.385 | 240 |  |
| 26.415 | 216 |  |
| 26.445 | 206 |  |
| 26.475 | 244 |  |
| 26.505 | 219 |  |
| 26.535 | 261 |  |
| 26.565 | 230 |  |
| 26.595 | 239 |  |
| 26.625 | 224 |  |
| 26.655 | 250 |  |
| 26.685 | 229 |  |
| 26.715 | 221 |  |
| 26.745 | 246 |  |
| 26.775 | 242 |  |
| 26.805 | 234 |  |
| 26.835 | 256 |  |
| 26.865 | 214 |  |
| 26.895 | 238 |  |
| 26.925 | 230 |  |
| 26.955 | 224 |  |
| 26.985 | 229 |  |
| 27.015 | 209 |  |
| 27.045 | 212 |  |
| 27.075 | 216 |  |
| 27.105 | 230 |  |
| 27.135 | 218 |  |
| 27.165 | 204 |  |
| 27.195 | 238 |  |
| 27.225 | 244 |  |
| 27.255 | 227 |  |
| 27.285 | 230 |  |
| 27.315 | 200 |  |
| 27.345 | 247 |  |
| 27.375 | 227 |  |
| 27.405 | 221 |  |
| 27.435 | 227 |  |
| 27.465 | 228 |  |
| 27.495 | 205 |  |
| 27.525 | 177 |  |
| 27.555 | 236 |  |
| 27.585 | 247 |  |
| 27.615 | 238 |  |
| 27.645 | 240 |  |
| 27.675 | 230 |  |
| 27.705 | 255 |  |
| 27.735 | 209 |  |
| 27.765 | 234 |  |
| 27.795 | 242 |  |
| 27.825 | 246 |  |
| 27.855 | 224 |  |
| 27.885 | 224 |  |
| 27.915 | 243 |  |
| 27.945 | 243 |  |
| 27.975 | 213 |  |
| 28.005 | 240 |  |
| 28.035 | 246 |  |
| 28.065 | 208 |  |
| 28.095 | 224 |  |
| 28.125 | 233 |  |
| 28.155 | 237 |  |
| 28.185 | 229 |  |
| 28.215 | 232 |  |
| 28.245 | 232 |  |
| 28.275 | 212 |  |
| 28.305 | 204 |  |
| 28.335 | 226 |  |
| 28.365 | 226 |  |
| 28.395 | 218 |  |
| 28.425 | 239 |  |
| 28.455 | 241 |  |
| 28.485 | 223 |  |
| 28.515 | 226 |  |
| 28.545 | 217 |  |
| 28.575 | 217 |  |
| 28.605 | 219 |  |
| 28.635 | 219 |  |
| 28.665 | 236 |  |
| 28.695 | 230 |  |
| 28.725 | 222 |  |
| 28.755 | 227 |  |
| 28.785 | 210 |  |
| 28.815 | 241 |  |
| 28.845 | 222 |  |
| 28.875 | 223 |  |
| 28.905 | 210 |  |
| 28.935 | 206 |  |
| 28.965 | 237 |  |
| 28.995 | 275 |  |
| 29.025 | 215 |  |
| 29.055 | 224 |  |
| 29.085 | 236 |  |
| 29.115 | 221 |  |
| 29.145 | 210 |  |
| 29.175 | 235 |  |
| 29.205 | 227 |  |
| 29.235 | 244 |  |
| 29.265 | 209 |  |
| 29.295 | 195 |  |
| 29.325 | 206 |  |
| 29.355 | 230 |  |
| 29.385 | 195 |  |
| 29.415 | 215 |  |
| 29.445 | 215 |  |
| 29.475 | 213 |  |
| 29.505 | 198 |  |
| 29.535 | 213 |  |
| 29.565 | 219 |  |
| 29.595 | 212 |  |
| 29.625 | 214 |  |
| 29.655 | 214 |  |
| 29.685 | 209 |  |
| 29.715 | 218 |  |
| 29.745 | 222 |  |
| 29.775 | 209 |  |
| 29.805 | 193 |  |
| 29.835 | 213 |  |
| 29.865 | 194 |  |
| 29.895 | 209 |  |
| 29.925 | 202 |  |
| 29.955 | 226 |  |
| 29.985 | 196 |  |
| 30.015 | 206 |  |
| 30.045 | 207 |  |
| 30.075 | 194 |  |
| 30.105 | 203 |  |
| 30.135 | 213 |  |
| 30.165 | 213 |  |
| 30.195 | 200 |  |
| 30.225 | 195 |  |
| 30.255 | 193 |  |
| 30.285 | 182 |  |
| 30.315 | 191 |  |
| 30.345 | 197 |  |
| 30.375 | 210 |  |
| 30.405 | 214 |  |
| 30.435 | 215 |  |
| 30.465 | 197 |  |
| 30.495 | 210 |  |
| 30.525 | 213 |  |
| 30.555 | 173 |  |
| 30.585 | 208 |  |
| 30.615 | 195 |  |
| 30.645 | 185 |  |
| 30.675 | 197 |  |
| 30.705 | 205 |  |
| 30.735 | 177 |  |
| 30.765 | 201 |  |
| 30.795 | 190 |  |
| 30.825 | 210 |  |
| 30.855 | 178 |  |
| 30.885 | 219 |  |
| 30.915 | 210 |  |
| 30.945 | 197 |  |
| 30.975 | 219 |  |
| 31.005 | 198 |  |
| 31.035 | 205 |  |
| 31.065 | 182 |  |
| 31.095 | 182 |  |
| 31.125 | 207 |  |
| 31.155 | 172 |  |
| 31.185 | 177 |  |
| 31.215 | 197 |  |
| 31.245 | 166 |  |
| 31.275 | 200 |  |
| 31.305 | 192 |  |
| 31.335 | 185 |  |
| 31.365 | 198 |  |
| 31.395 | 181 |  |
| 31.425 | 215 |  |
| 31.455 | 191 |  |
| 31.485 | 200 |  |
| 31.515 | 195 |  |
| 31.545 | 181 |  |
| 31.575 | 200 |  |
| 31.605 | 204 |  |
| 31.635 | 199 |  |
| 31.665 | 210 |  |
| 31.695 | 176 |  |
| 31.725 | 202 |  |
| 31.755 | 200 |  |
| 31.785 | 212 |  |
| 31.815 | 191 |  |
| 31.845 | 179 |  |
| 31.875 | 195 |  |
| 31.905 | 196 |  |
| 31.935 | 182 |  |
| 31.965 | 205 |  |
| 31.995 | 194 |  |
| 32.025 | 167 |  |
| 32.055 | 193 |  |
| 32.085 | 192 |  |
| 32.115 | 188 |  |
| 32.145 | 204 |  |
| 32.175 | 182 |  |
| 32.205 | 194 |  |
| 32.235 | 209 |  |
| 32.265 | 196 |  |
| 32.295 | 192 |  |
| 32.325 | 211 |  |
| 32.355 | 185 |  |
| 32.385 | 203 |  |
| 32.415 | 197 |  |
| 32.445 | 195 |  |
| 32.475 | 195 |  |
| 32.505 | 219 |  |
| 32.535 | 181 |  |
| 32.565 | 201 |  |
| 32.595 | 179 |  |
| 32.625 | 230 |  |
| 32.655 | 193 |  |
| 32.685 | 186 |  |
| 32.715 | 196 |  |
| 32.745 | 207 |  |
| 32.775 | 172 |  |
| 32.805 | 188 |  |
| 32.835 | 179 |  |
| 32.865 | 183 |  |
| 32.895 | 178 |  |
| 32.925 | 188 |  |
| 32.955 | 197 |  |
| 32.985 | 185 |  |
| 33.015 | 205 |  |
| 33.045 | 191 |  |
| 33.075 | 183 |  |
| 33.105 | 209 |  |
| 33.135 | 198 |  |
| 33.165 | 183 |  |
| 33.195 | 169 |  |
| 33.225 | 201 |  |
| 33.255 | 205 |  |
| 33.285 | 168 |  |
| 33.315 | 234 |  |
| 33.345 | 189 |  |
| 33.375 | 217 |  |
| 33.405 | 198 |  |
| 33.435 | 210 |  |
| 33.465 | 197 |  |
| 33.495 | 202 |  |
| 33.525 | 211 |  |
| 33.555 | 191 |  |
| 33.585 | 185 |  |
| 33.615 | 240 |  |
| 33.645 | 205 |  |
| 33.675 | 194 |  |
| 33.705 | 190 |  |
| 33.735 | 231 |  |
| 33.765 | 208 |  |
| 33.795 | 188 |  |
| 33.825 | 188 |  |
| 33.855 | 181 |  |
| 33.885 | 200 |  |
| 33.915 | 187 |  |
| 33.945 | 174 |  |
| 33.975 | 200 |  |
| 34.005 | 190 |  |
| 34.035 | 181 |  |
| 34.065 | 184 |  |
| 34.095 | 214 |  |
| 34.125 | 207 |  |
| 34.155 | 206 |  |
| 34.185 | 214 |  |
| 34.215 | 219 |  |
| 34.245 | 201 |  |
| 34.275 | 198 |  |
| 34.305 | 211 |  |
| 34.335 | 214 |  |
| 34.365 | 207 |  |
| 34.395 | 198 |  |
| 34.425 | 222 |  |
| 34.455 | 201 |  |
| 34.485 | 214 |  |
| 34.515 | 233 |  |
| 34.545 | 186 |  |
| 34.575 | 221 |  |
| 34.605 | 209 |  |
| 34.635 | 226 |  |
| 34.665 | 200 |  |
| 34.695 | 219 |  |
| 34.725 | 193 |  |
| 34.755 | 196 |  |
| 34.785 | 200 |  |
| 34.815 | 218 |  |
| 34.845 | 209 |  |
| 34.875 | 207 |  |
| 34.905 | 221 |  |
| 34.935 | 220 |  |
| 34.965 | 223 |  |
| 34.995 | 196 |  |
| 35.025 | 199 |  |
| 35.055 | 204 |  |
| 35.085 | 202 |  |
| 35.115 | 231 |  |
| 35.145 | 193 |  |
| 35.175 | 206 |  |
| 35.205 | 194 |  |
| 35.235 | 216 |  |
| 35.265 | 209 |  |
| 35.295 | 229 |  |
| 35.325 | 200 |  |
| 35.355 | 209 |  |
| 35.385 | 187 |  |
| 35.415 | 198 |  |
| 35.445 | 243 |  |
| 35.475 | 200 |  |
| 35.505 | 218 |  |
| 35.535 | 195 |  |
| 35.565 | 196 |  |
| 35.595 | 203 |  |
| 35.625 | 205 |  |
| 35.655 | 179 |  |
| 35.685 | 184 |  |
| 35.715 | 199 |  |
| 35.745 | 228 |  |
| 35.775 | 202 |  |
| 35.805 | 206 |  |
| 35.835 | 189 |  |
| 35.865 | 213 |  |
| 35.895 | 200 |  |
| 35.925 | 226 |  |
| 35.955 | 208 |  |
| 35.985 | 217 |  |
| 36.015 | 199 |  |
| 36.045 | 191 |  |
| 36.075 | 200 |  |
| 36.105 | 194 |  |
| 36.135 | 193 |  |
| 36.165 | 199 |  |
| 36.195 | 210 |  |
| 36.225 | 187 |  |
| 36.255 | 186 |  |
| 36.285 | 203 |  |
| 36.315 | 202 |  |
| 36.345 | 213 |  |
| 36.375 | 191 |  |
| 36.405 | 192 |  |
| 36.435 | 204 |  |
| 36.465 | 199 |  |
| 36.495 | 199 |  |
| 36.525 | 210 |  |
| 36.555 | 207 |  |
| 36.585 | 176 |  |
| 36.615 | 191 |  |
| 36.645 | 216 |  |
| 36.675 | 199 |  |
| 36.705 | 214 |  |
| 36.735 | 171 |  |
| 36.765 | 198 |  |
| 36.795 | 183 |  |
| 36.825 | 210 |  |
| 36.855 | 208 |  |
| 36.885 | 194 |  |
| 36.915 | 200 |  |
| 36.945 | 230 |  |
| 36.975 | 181 |  |
| 37.005 | 190 |  |
| 37.035 | 191 |  |
| 37.065 | 206 |  |
| 37.095 | 220 |  |
| 37.125 | 180 |  |
| 37.155 | 219 |  |
| 37.185 | 183 |  |
| 37.215 | 202 |  |
| 37.245 | 203 |  |
| 37.275 | 206 |  |
| 37.305 | 196 |  |
| 37.335 | 211 |  |
| 37.365 | 202 |  |
| 37.395 | 194 |  |
| 37.425 | 213 |  |
| 37.455 | 189 |  |
| 37.485 | 211 |  |
| 37.515 | 189 |  |
| 37.545 | 207 |  |
| 37.575 | 206 |  |
| 37.605 | 189 |  |
| 37.635 | 219 |  |
| 37.665 | 176 |  |
| 37.695 | 201 |  |
| 37.725 | 216 |  |
| 37.755 | 217 |  |
| 37.785 | 204 |  |
| 37.815 | 192 |  |
| 37.845 | 191 |  |
| 37.875 | 195 |  |
| 37.905 | 179 |  |
| 37.935 | 207 |  |
| 37.965 | 197 |  |
| 37.995 | 200 |  |
| 38.025 | 201 |  |
| 38.055 | 202 |  |
| 38.085 | 214 |  |
| 38.115 | 188 |  |
| 38.145 | 195 |  |
| 38.175 | 201 |  |
| 38.205 | 210 |  |
| 38.235 | 181 |  |
| 38.265 | 203 |  |
| 38.295 | 195 |  |
| 38.325 | 188 |  |
| 38.355 | 195 |  |
| 38.385 | 196 |  |
| 38.415 | 199 |  |
| 38.445 | 209 |  |
| 38.475 | 200 |  |
| 38.505 | 216 |  |
| 38.535 | 201 |  |
| 38.565 | 219 |  |
| 38.595 | 204 |  |
| 38.625 | 183 |  |
| 38.655 | 198 |  |
| 38.685 | 197 |  |
| 38.715 | 207 |  |
| 38.745 | 180 |  |
| 38.775 | 194 |  |
| 38.805 | 195 |  |
| 38.835 | 198 |  |
| 38.865 | 202 |  |
| 38.895 | 231 |  |
| 38.925 | 212 |  |
| 38.955 | 180 |  |
| 38.985 | 185 |  |
| 39.015 | 217 |  |
| 39.045 | 222 |  |
| 39.075 | 185 |  |
| 39.105 | 196 |  |
| 39.135 | 193 |  |
| 39.165 | 212 |  |
| 39.195 | 208 |  |
| 39.225 | 202 |  |
| 39.255 | 216 |  |
| 39.285 | 205 |  |
| 39.315 | 168 |  |
| 39.345 | 197 |  |
| 39.375 | 216 |  |
| 39.405 | 165 |  |
| 39.435 | 204 |  |
| 39.465 | 182 |  |
| 39.495 | 222 |  |
| 39.525 | 206 |  |
| 39.555 | 223 |  |
| 39.585 | 191 |  |
| 39.615 | 215 |  |
| 39.645 | 234 |  |
| 39.675 | 221 |  |
| 39.705 | 190 |  |
| 39.735 | 199 |  |
| 39.765 | 229 |  |
| 39.795 | 207 |  |
| 39.825 | 186 |  |
| 39.855 | 189 |  |
| 39.885 | 192 |  |
| 39.915 | 202 |  |
| 39.945 | 184 |  |
| 39.975 | 190 |  |
| 40.005 | 192 |  |
| 40.035 | 207 |  |
| 40.065 | 197 |  |
| 40.095 | 179 |  |
| 40.125 | 187 |  |
| 40.155 | 188 |  |
| 40.185 | 174 |  |
| 40.215 | 196 |  |
| 40.245 | 224 |  |
| 40.275 | 190 |  |
| 40.305 | 193 |  |
| 40.335 | 192 |  |
| 40.365 | 204 |  |
| 40.395 | 202 |  |
| 40.425 | 169 |  |
| 40.455 | 207 |  |
| 40.485 | 214 |  |
| 40.515 | 198 |  |
| 40.545 | 227 |  |
| 40.575 | 174 |  |
| 40.605 | 178 |  |
| 40.635 | 207 |  |
| 40.665 | 195 |  |
| 40.695 | 187 |  |
| 40.725 | 191 |  |
| 40.755 | 200 |  |
| 40.785 | 217 |  |
| 40.815 | 174 |  |
| 40.845 | 198 |  |
| 40.875 | 180 |  |
| 40.905 | 196 |  |
| 40.935 | 192 |  |
| 40.965 | 197 |  |
| 40.995 | 210 |  |
| 41.025 | 200 |  |
| 41.055 | 182 |  |
| 41.085 | 193 |  |
| 41.115 | 197 |  |
| 41.145 | 175 |  |
| 41.175 | 192 |  |
| 41.205 | 219 |  |
| 41.235 | 185 |  |
| 41.265 | 188 |  |
| 41.295 | 188 |  |
| 41.325 | 205 |  |
| 41.355 | 182 |  |
| 41.385 | 202 |  |
| 41.415 | 210 |  |
| 41.445 | 200 |  |
| 41.475 | 192 |  |
| 41.505 | 193 |  |
| 41.535 | 174 |  |
| 41.565 | 194 |  |
| 41.595 | 163 |  |
| 41.625 | 197 |  |
| 41.655 | 193 |  |
| 41.685 | 187 |  |
| 41.715 | 179 |  |
| 41.745 | 188 |  |
| 41.775 | 224 |  |
| 41.805 | 223 |  |
| 41.835 | 188 |  |
| 41.865 | 200 |  |
| 41.895 | 209 |  |
| 41.925 | 238 |  |
| 41.955 | 236 |  |
| 41.985 | 226 |  |
| 42.015 | 251 |  |
| 42.045 | 295 |  |
| 42.075 | 251 |  |
| 42.105 | 260 |  |
| 42.135 | 210 |  |
| 42.165 | 234 |  |
| 42.195 | 236 |  |
| 42.225 | 205 |  |
| 42.255 | 231 |  |
| 42.285 | 236 |  |
| 42.315 | 221 |  |
| 42.345 | 185 |  |
| 42.375 | 189 |  |
| 42.405 | 199 |  |
| 42.435 | 193 |  |
| 42.465 | 203 |  |
| 42.495 | 198 |  |
| 42.525 | 188 |  |
| 42.555 | 197 |  |
| 42.585 | 212 |  |
| 42.615 | 209 |  |
| 42.645 | 170 |  |
| 42.675 | 200 |  |
| 42.705 | 212 |  |
| 42.735 | 192 |  |
| 42.765 | 222 |  |
| 42.795 | 211 |  |
| 42.825 | 194 |  |
| 42.855 | 230 |  |
| 42.885 | 214 |  |
| 42.915 | 228 |  |
| 42.945 | 201 |  |
| 42.975 | 234 |  |
| 43.005 | 229 |  |
| 43.035 | 238 |  |
| 43.065 | 252 |  |
| 43.095 | 224 |  |
| 43.125 | 227 |  |
| 43.155 | 247 |  |
| 43.185 | 277 |  |
| 43.215 | 291 |  |
| 43.245 | 265 |  |
| 43.275 | 304 |  |
| 43.305 | 327 |  |
| 43.335 | 315 |  |
| 43.365 | 347 |  |
| 43.395 | 333 |  |
| 43.425 | 383 |  |
| 43.455 | 385 |  |
| 43.485 | 375 |  |
| 43.515 | 417 |  |
| 43.545 | 417 |  |
| 43.575 | 416 |  |
| 43.605 | 478 |  |
| 43.635 | 420 |  |
| 43.665 | 451 |  |
| 43.695 | 434 |  |
| 43.725 | 417 |  |
| 43.755 | 416 |  |
| 43.785 | 400 |  |
| 43.815 | 428 |  |
| 43.845 | 389 |  |
| 43.875 | 367 |  |
| 43.905 | 321 |  |
| 43.935 | 321 |  |
| 43.965 | 315 |  |
| 43.995 | 307 |  |
| 44.025 | 310 |  |
| 44.055 | 252 |  |
| 44.085 | 266 |  |
| 44.115 | 225 |  |
| 44.145 | 226 |  |
| 44.175 | 225 |  |
| 44.205 | 257 |  |
| 44.235 | 200 |  |
| 44.265 | 208 |  |
| 44.295 | 207 |  |
| 44.325 | 192 |  |
| 44.355 | 210 |  |
| 44.385 | 216 |  |
| 44.415 | 209 |  |
| 44.445 | 177 |  |
| 44.475 | 193 |  |
| 44.505 | 212 |  |
| 44.535 | 207 |  |
| 44.565 | 218 |  |
| 44.595 | 198 |  |
| 44.625 | 240 |  |
| 44.655 | 217 |  |
| 44.685 | 197 |  |
| 44.715 | 173 |  |
| 44.745 | 160 |  |
| 44.775 | 196 |  |
| 44.805 | 170 |  |
| 44.835 | 206 |  |
| 44.865 | 166 |  |
| 44.895 | 159 |  |
| 44.925 | 171 |  |
| 44.955 | 195 |  |
| 44.985 | 184 |  |
